# Cigarette smoke and chewing tobacco alter expression of different sets of miRNAs in oral keratinocytes

**DOI:** 10.1038/s41598-018-25498-2

**Published:** 2018-05-04

**Authors:** Mohd Younis Bhat, Jayshree Advani, Pavithra Rajagopalan, Krishna Patel, Vishalakshi Nanjappa, Hitendra S. Solanki, Arun H. Patil, Firdous A. Bhat, Premendu P. Mathur, Bipin Nair, T. S. Keshava Prasad, Joseph A. Califano, David Sidransky, Harsha Gowda, Aditi Chatterjee

**Affiliations:** 1 Institute of Bioinformatics, International Technology Park, Bangalore, 560066 India; 2 School of Biotechnology, Amrita Vishwa Vidyapeetham, Kollam, 690525 India; 3 Manipal Academy of Higher Education, Manipal, 576104 India; 4 School of Biotechnology, Kalinga Institute of Industrial Technology, Bhubaneswar, 751024 India; 5 Center for Systems Biology and Molecular Medicine, Yenepoya Research Centre, Yenepoya (Deemed to be University), Mangalore, 575018 India; 6 Department of Surgery, UC San Diego, Moores Cancer Center, La Jolla, CA 92093 USA; 7 Department of Otolaryngology-Head and Neck Surgery, Johns Hopkins University School of Medicine, Baltimore, MD 21231 USA

## Abstract

Carcinogenic effect of tobacco in oral cancer is through chewing and/or smoking. Significant differences exist in development of oral cancer between tobacco users and non-users. However, molecular alterations induced by different forms of tobacco are yet to be fully elucidated. We developed cellular models of chronic exposure to chewing tobacco and cigarette smoke using immortalized oral keratinocytes. Chronic exposure to tobacco resulted in increased cell scattering and invasiveness in immortalized oral keratinocytes. miRNA sequencing using Illumina HiSeq 2500 resulted in the identification of 10 significantly dysregulated miRNAs (4 fold; p ≤ 0.05) in chewing tobacco treated cells and 6 in cigarette smoke exposed cells. We integrated this data with global proteomic data and identified 36 protein targets that showed inverse expression pattern in chewing tobacco treated cells and 16 protein targets that showed inverse expression in smoke exposed cells. In addition, we identified 6 novel miRNAs in chewing tobacco treated cells and 18 novel miRNAs in smoke exposed cells. Integrative analysis of dysregulated miRNAs and their targets indicates that signaling mechanisms leading to oncogenic transformation are distinct between both forms of tobacco. Our study demonstrates alterations in miRNA expression in oral cells in response to two frequently used forms of tobacco.

## Introduction

Oral squamous cell carcinoma (OSCC) is one of the most common cancers worldwide and remains the most common malignancy of the head and neck cancers. Tobacco use, alcohol consumption and human papilloma virus (HPV) 16/18 have been identified as the main risk factors for the initiation and progression of OSCC^1^. Tobacco is mainly consumed worldwide in the form of manufactured cigarettes. Tobacco is also consumed in the form of smokeless tobacco, especially chewing tobacco in South-East Asian countries^2^. Despite being one of the most common cancers in India, molecular alterations in oral cancer development in tobacco chewers and smokers is not well understood.

MicroRNAs have been established as key regulators of oncogenic potential in cells. Alterations at the genetic and epigenetic levels in the complex enzymatic machinery involved in miRNA biogenesis can result in aberrant miRNA expression^3^. Post-transcriptional regulation of gene expression by miRNAs has an influence on multiple pathways, including those involved in cellular transformation and proliferation^4^. miRNAs function as either oncogenes or tumor suppressors, playing crucial roles in tumorigenesis, tumor invasion and metastasis^5^. In recent years multiple studies have revealed the altered expression of miRNAs which play a role in the development and progression of diverse cancers including oral squamous cell carcinoma^6–8^. Increased expression of microRNAs including miR-155 and miR-23a, have been observed in oral cancer patients that are tobacco chewers compared to non-chewers^9,10^. Fanconi anemia complementation group G protein (FANCG) is a miR-23a target with a role in DNA double strand break repair pathway. Decreased expression of FANCG in normal oral fibroblasts contributes to the development of carcinogenesis on treatment with areca nut^10^. In contrast, microRNAs such as miR-145 are found to be significantly downregulated upon cigarette smoke condensate treatment in oral fibroblasts while its target protein MMP-2 is overexpressed which plays a key role in perturbation of stromal-epithelial communication and promotes pro-tumorogenic interactions^11^. Similarly, a decrease in miR-101-3p and a corresponding increase in expression levels of its target protein COX2 was observed in an esophageal non-tumorigenic cell line upon treatment with cigarette smoke condensate thereby facilitating cell transformation and cancer development^12^. Downregulation of miR-200c levels in human bronchial epithelial cells with increased expression of IL-6 and activation of nuclear factor-κB (NF-κB) pathway by cigarette smoke extract is also shown to regulate epithelial-mesenchymal transition and carcinogenesis^13^.

Taken together, these studies indicate that molecular mechanisms for cellular transformation may vary depending upon the form of tobacco used. Till date no study has systematically investigated the differences in molecular alterations induced in oral cells upon exposure to different forms of tobacco. To achieve this, we developed two cellular models where immortalized, oral keratinocytes (OKF6/TERT1) were chronically treated with either chewing tobacco or exposed to cigarette smoke for a period of six months. To understand specific molecular alterations brought about by each form of tobacco, we performed miRNA sequencing of oral keratinocytes chronically treated with chewing tobacco/cigarette smoke. miRNA dysregulation is in turn known to affect expression of their target proteins leading to diverse functional consequences. Hence, in addition to studying miRNA dysregulation, we have investigated proteomic alterations associated with exposure to these two forms of tobacco.

We observed that chronic treatment of, immortalized oral keratinocytes with either chewing tobacco extract or cigarette smoke condensate affected expression of distinct set of miRNAs and their corresponding protein targets.

## Results

### Chronic exposure to chewing tobacco and cigarette smoke results in phenotypic changes in oral cells

**Figure 1:**
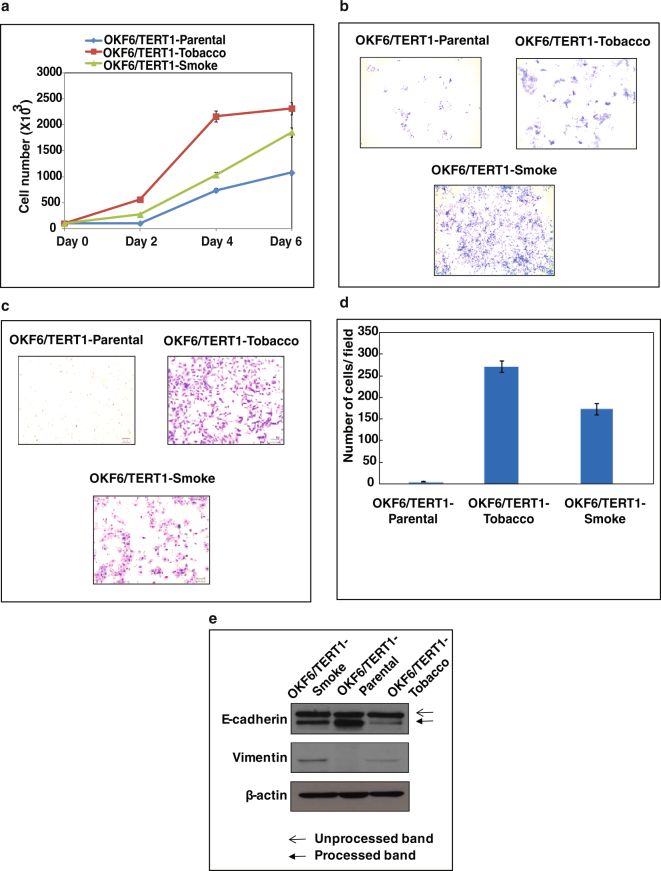
Chronic treatment of oral keratinocytes with chewing tobacco and cigarette smoke. OKF6/TERT1-Parental, OKF6/TERT1-Tobacco and OKF6/TERT1-Smoke cells were assessed for (**a**) cellular proliferation (**b**) colony formation (**c**,**d**) invasive ability and (**e**) epithelial–mesenchymal transition (EMT) using Western blotting. β-actin was used as a loading control.

In this study, we chronically treated immortalized oral keratinocytes (OKF6/TERT1) with chewing tobacco extract and cigarette smoke condensate to model the effects of tobacco chewing and smoking in oral cancer. We observed an increase in proliferative capability of both OKF6/TERT1-Tobacco and OKF6/TERT1-Smoke cells compared to OKF6/TERT1-Parental cells (Fig. 1a). Both tobacco treated and smoke exposed cells displayed increased cell scattering phenomena (Fig. 1b**)** which is one of the features of cancer cell phenotype. We also observed an increase in the invasive ability of oral keratinocytes upon chronic treatment with chewing tobacco/cigarette smoke (Fig. 1c,d). We also observed change in expression levels of markers associated with EMT in both chewing tobacco treated and cigarette smoke exposed cells compared to parental cells.There was a decrease in expression of E-Cadherin (processed band, lower) and a increase in vimentin expression in OKF6/TERT1-Tobacco and OKF6/TERT1-Smoke cells compared to OKF6/TERT1-Parental cells (Fig. 1e**)**. To further elucidate the molecular alterations imparted by each type of insult, we studied miRNA and proteomic expression pattern in these cells compared to untreated parental cells.

### Chronic exposure to chewing tobacco/cigarette smoke results in altered miRNA expression in oral keratinocytes

**Figure 2:**
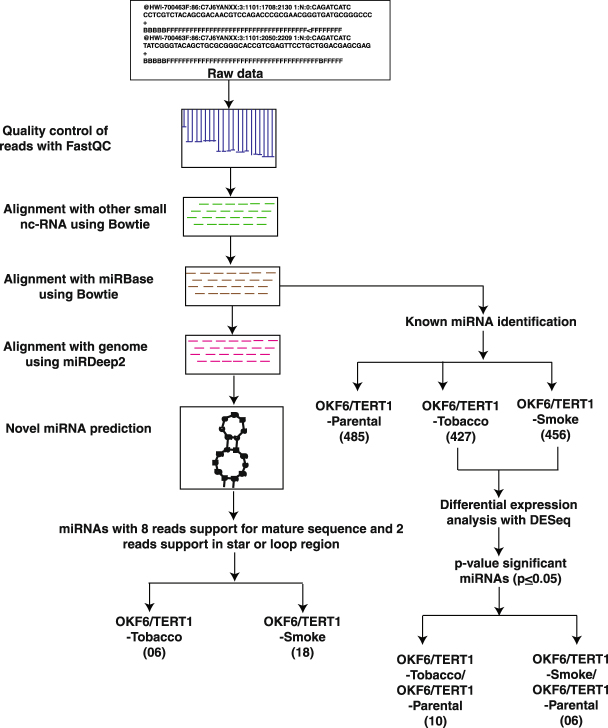
Bioinformatics workflow. Pipeline depicting the analysis of dysregulated miRNA in OKF6/TERT1-Tobacco and OKF6/TERT1-Smoke cells and novel miRNA identification.

**Figure 3:**
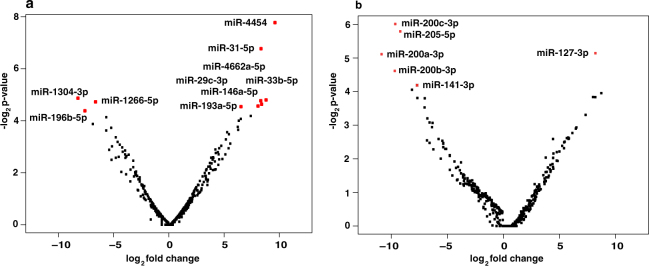
A volcano plot showing distribution of miRNAs in (**a**) OKF6/TERT1- Tobacco cells (**b**) OKF6/TERT1-Smoke cells. The red dots represent significantly dysregulated miRNAs (p-value ≤ 0.05).

We carried out deep small RNA sequencing on Illumina HiSeq 2500 platform. We acquired 31 million reads for untreated control cells, 56 million reads for tobacco treated cells and 47 million reads for smoke exposed cells. A schematic representation of the workflow is provided in Supplementary Fig. S1. miRNA expression analysis resulted in identification of 427 and 456 miRNAs in chewing tobacco treated and cigarette smoke exposed oral keratinocytes, respectively **(**Fig. 2**)**. Amongst these 10 miRNAs were significantly dysregulated (≥4 fold, p ≤ 0.05) in chewing tobacco treated cells and 6 miRNAs were significantly dysregulated in cigarette smoke exposed cells (Fig. 3a,b). A complete list of miRNAs along with corresponding fold-changes in OKF6/TERT1-Tobacco and OKF6/TERT1-Smoke cells are provided in Supplementary Tables S1A,B.

### Altered protein expression pattern associated with chronic exposure to chewing tobacco/cigarette smoke in oral keratinocytes

We employed a TMT-based proteomic approach to investigate global proteomic alterations in response to chewing tobacco/cigarette smoke treatment. LC-MS - based quantitative proteomics led to quantification of 2,821 and 5,342 proteins in OKF6/TERT1-Tobacco and OKF6/TERT1-Smoke cells, respectively. The proteomics workflow employed to carry out this investigation is outlined in Supplementary Fig. S1. Amongst the 311 significantly dysregulated proteins in OKF6/TERT1-Tobacco, 197 were overexpressed (≥1.5-fold, p-value ≤ 0.05) and 114 proteins were downregulated (≤0.6-fold, p-value ≤ 0.05). In case of OKF6/TERT1-Smoke cells, 229 proteins were overexpressed (≥1.5-fold, p-value ≤ 0.05) and 159 were downregulated (≤0.6-fold, p-value ≤ 0.05). The complete list of identified proteins and peptides is provided in Supplementary Tables S2A,B.

### Correlation of miRNAs with protein expression

**Figure 4:**
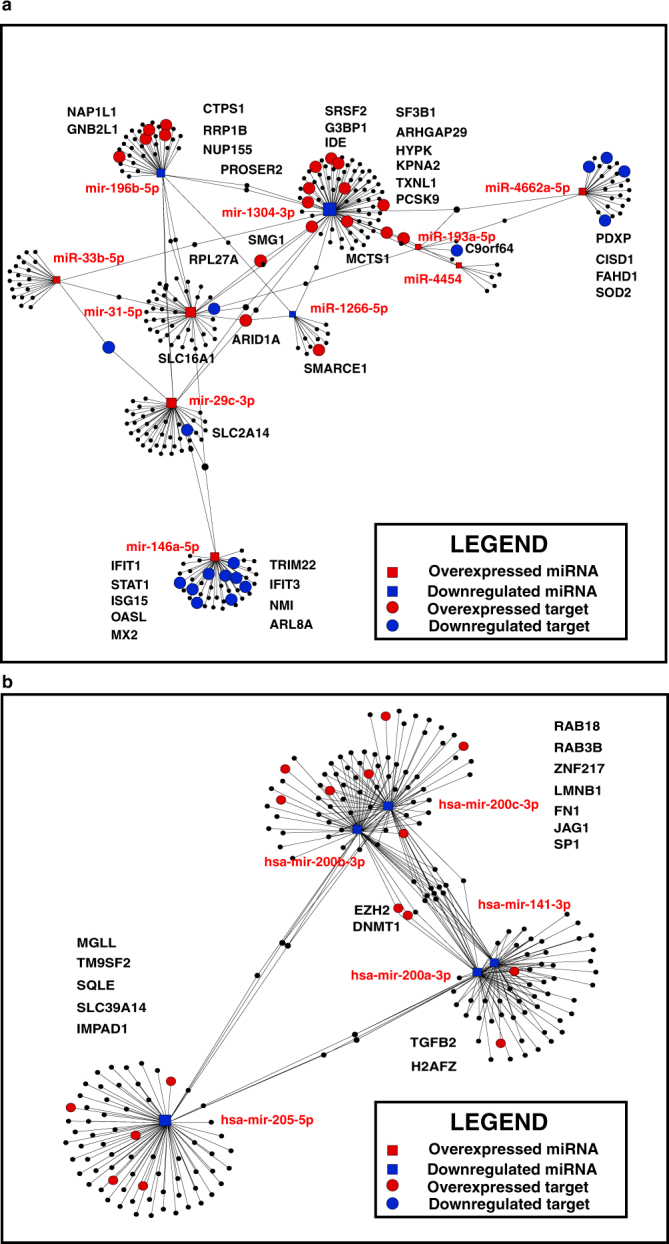
miRNA-mRNA interaction network of significantly dysregulated miRNAs and their targets in (**a**) OKF6/TERT1-Tobacco cells (**b**) OKF6/TERT1-Smoke cells. Square blocks represent dysregulated miRNAs and circles represent their target proteins showing inverse correlation. Overexpressed miRNAs and proteins are highlighted in red while downregulated miRNAs and proteins are highlighted in blue color.

Since we observed altered expression of both miRNAs and proteins in response to two forms of tobacco, we compared expression pattern using experimentally proven targets of miRNAs from miRTarBase^14^. We fetched targets of 10 significantly dysregulated miRNAs identified in OKF6/TERT1-Tobacco cells from miRTarBase. There were 293 proteins that were identified in our proteomics data (Supplementary Table S3A). In case of OKF6/TERT1-Smoke cells, we identified 218 protein targets in our proteomic data for the 6 significantly dysregulated miRNAs (Supplementary Table S3B). To understand the correlation between significantly dysregulated miRNAs and their target proteins, miRNA-gene interaction network analysis was generated using miRNet tool^15^. The 10 miRNAs significantly dysregulated in OKF6/TERT1-Tobacco cells showed directional relationship with 36 target proteins identified in proteomics data **(**Fig. 4a**)**. Reverse transcriptase PCR-based validation of some of the targets is provided in Supplementary Fig. S2A. Our proteomics data revealed downregulation of superoxidase dismutase, SOD2, in tobacco treated OKF6/TERT1 cells and in conjunction with this we observed a significant decrease in SOD2 expression in OKF6/TERT1-Tobacco cells compared to OKF6/TERT1-Parental cells (Supplementary Fig. S2A, see Western panel). The 6 miRNAs which were significantly dysregulated in OKF6/TERT1-Smoke cells showed directional relationship with 16 target proteins (Fig. 4b). Expression of some of these targets was validated using reverse transcriptase PCR and is provided in Supplementary Fig. S2B. We observed a 1.8 fold increase in TGFB2 expression in smoke exposed OKF6/TERT1 cells in our proteomics data and this increase in expression was confirmed by Western blot analysis of OKF6/TERT1-Smoke cells compared to OKF6/TERT1-Parental cells as depicted in Supplementary Fig. S2B (see Western panel). Significantly dysregulated miRNAs and their corresponding targets which showed a directional relationship are highlighted (miRNAs and protein targets which are upregulated are highlighted in red and miRNAs and protein targets which are downregulated are in blue color) in Supplementary Table S3A,B.

### Bioinformatics analysis

**Figure 5:**
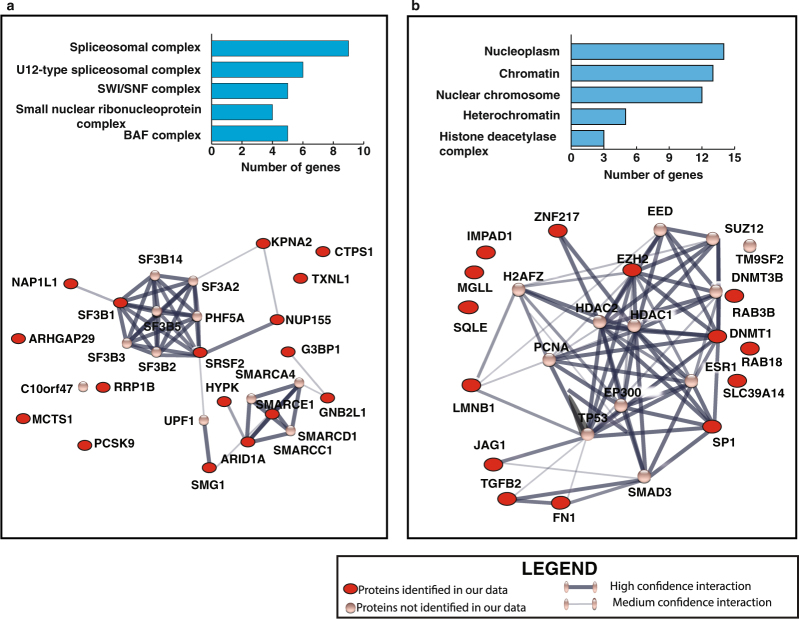
STITCH generated protein-protein interaction network of overexpressed protein targets of significantly downregulated miRNAs in **(a**) OKF6/TERT1-Tobacco cells and (**b**) OKF6/TERT1-Smoke cells. Red circles represent proteins identified in our proteomic data set.

We used STITCH tool (version 5.0)^16^ to analyse the protein-protein interaction of targets which showed inverse relationship with their corresponding miRNAs in tobacco and smoke exposed cells. Functional enrichment analysis was performed for proteins which were overexpressed and targeted by significantly downregulated miRNAs in tobacco treated cells. This revealed enrichment of spliceosome-associated proteins that have a major role in processing of pre-mRNA/splicing into its mature forms (Fig. 5a). Similarly proteins which were overexpressed and are targeted by the significantly downregulated miRNAs in smoke exposed cells, showed enrichment of proteins which play an essential role in chromatin remodelling and transcriptional regulation (Fig. 5b).

### Novel miRNA analysis

**Figure 6:**
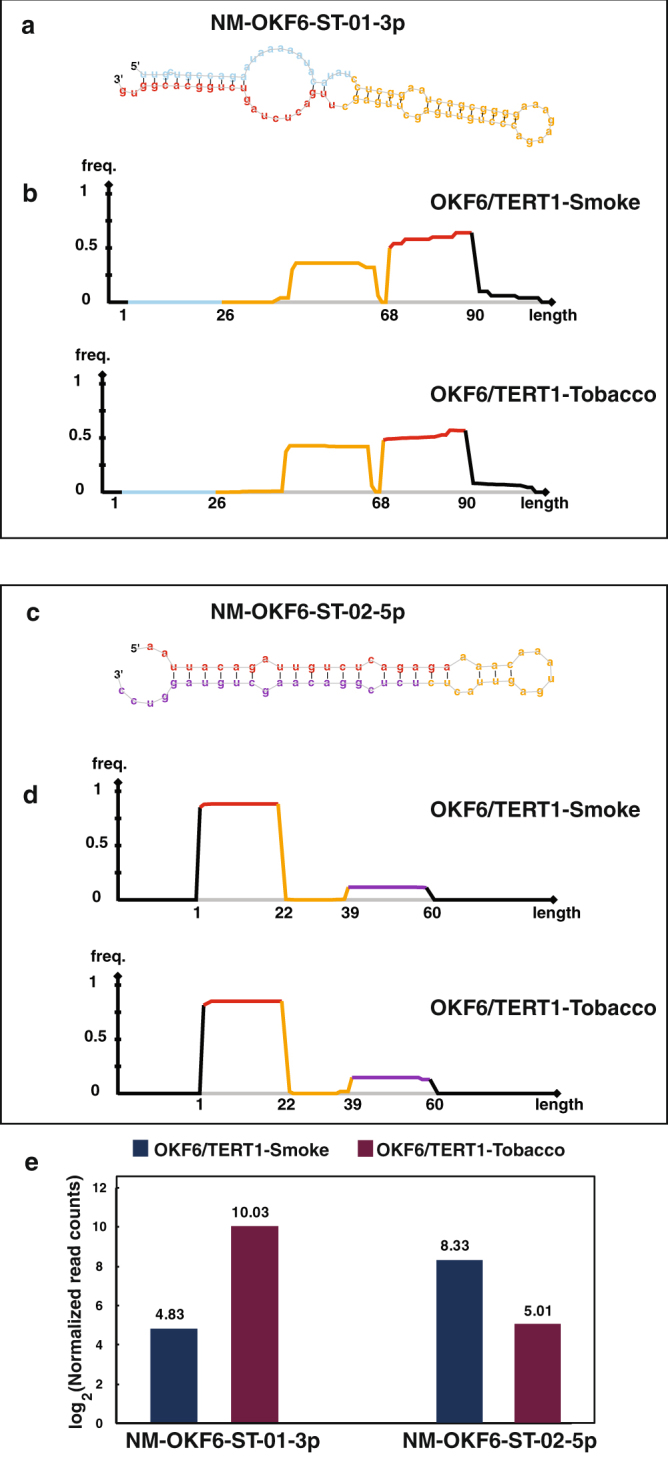
Expression pattern of novel miRNA common to OKF6/TERT1-Tobacco and OKF6/TERT1-Smoke cells: (**a**) Secondary structure of novel miRNA NM-OKF6-ST-01-3p identified in both OKF6/TERT1-Smoke and OKF6/TERT1-Tobacco cells (**b**) Read distribution frequency plot of NM-OKF6-ST-01-3p in OKF6/TERT1-Smoke and OKF6/TERT1-Tobacco cells (**c**) Secondary structure of novel miRNA NM-OKF6-ST-02-5p identified in OKF6/TERT1-Smoke and OKF6/TERT1-Tobacco cells (**d**) Read distribution frequency plot of NM-OKF6-ST-02-5p precursor in OKF6/TERT1-Smoke and OKF6/TERT1-Tobacco cells (**e**) Expression pattern of NM-OKF6-ST-01-3p and NM-OKF6-ST-02-5p in OKF6/TERT1-Tobacco and OKF6/TERT1-Smoke cells using log transformed normalized read counts.

Small RNA-Seq datasets for OKF6/TERT1-Parental, OKF6/TERT1-Tobacco and OKF6/TERT1-Smoke cells were analyzed for novel miRNAs using miRDeep2^17^. Novel miRNA with miRDeep2 score ≥1, ≥8 reads supporting mature miRNA, and ≥2 reads supporting loop/star sequence were filtered to acquire high confidence list. This led us to identify 3 novel miRNAs in OKF6/TERT1-Parental, 6 novel miRNAs in OKF6/TERT1-Tobacco and 18 novel miRNAs in OKF6/TERT1-Smoke cells. The genomic locations of the novel miRNAs are provided in Supplementary Tables S4A–C. The secondary structures of these novel miRNAs are depicted in Supplementary Fig. S3A–C, Supplementary Fig. S4A–F and Supplementary Fig. S5A–R, respectively. Amongst the novel miRNAs, two miRNAs were common between OKF6/TERT1-Tobacco and OKF6/TERT1-Smoke cells, but these were not identified in OKF6/TERT1-Parental cells (Fig. 6). To compare their expression pattern, we normalized read support of each novel miRNA with number of unique reads (length ≥18) from their respective sequence library. Novel miRNA NM-OKF6-ST-01-3p in tobacco treated cells showed a relatively higher expression with 1,726 reads compared to smoke exposed cells with 47 reads. Conversely, miRNA NM-OKF6-ST-02-5p showed higher expression in smoke exposed cells with 532 reads compared to tobacco treated cells with 53 reads.

## Discussion

Chewing tobacco is one of the known risk factors associated with oral cancer^18^. This is a common practice in India compared to the the western world^2^. Therefore, limited molecular level investigations have been carried out on the effect of chewing tobacco compared to tobacco smoking. In this study, using cellular models, we have systematically studied the alterations in miRNA expression and their targets in response to both chewing tobacco and cigarette smoke.

During the last few decades, miRNAs have emerged as one of the key regulators of cellular processes ranging from cell cycle regulation, differentiation, cell migration and apoptosis^19^. Their discovery has initiated a revolutionary change in cancer research, making them one of the important parameters to be elucidated in cancer. We identified several miRNAs previously reported to be dysregulated in oral cancer substantiating our data in both chewing tobacco treated and cigarette smoke exposed oral cells. We observed significant upregulation of miR-31-5p in OKF6/TERT1-Tobacco cells. Association of miR-31-5p dysregulation has been shown to result in initiation and progression of oral leukoplakia to full blown OSCC^20,21^. We also observed overexpression of miR-29c-3p and miR-146a-5p in chewing tobacco treated cells. High expression of miR-29c-3p has been reported in aggressive oral tongue tumours and in OSCC^22,23^. Also, miR-29c-3p was found to be upregulated with decreased expression level of its target protein PTX3 in meningiomas^24^. The role of miR-146a-5p remains ambiguous in oral cancer related studies. Loss or decreased expression of miR-146a-5p was found to be associated with aggressive behaviour in oral squamous cell carcinoma^25^. Contrary to this, Hung *et al*., reported that miR-146a expression contributes to oral carcinogenesis by targeting the IRAK1, TRAF6 and NUMB genes. The plasma levels of miR-146a in OSCC patients were also significantly higher as compared to control groups^26^. miR-1266-5p has been reported as an oncomiR and is reported to be over expressed in HNSCC patients who are users of alcohol^27^. Similarly, miR-196b-5p is reported to be overexpressed in oesophageal adenocarcinoma, gastric and oral cancer in smokers^28–30^. In contrary to this data we observed downregulation of miR-1266-5p and miR-196b-5p in oral keratinocytes chronically treated with tobacco, indicating that expression profile of miRNAs possibly depends on the type of insult which induces carcinoma. Interestingly, downregulation of miR-196b has been associated with increase in EMT^31^. This is in concordance with our cellular assays where we observed an increase in protein markers associated with EMT in tobacco treated cells. Similarly we observed increased expression of miR-193a-5p in the tobacco treated cells, where as previous studies have shown miR-193a-5p is silenced by DNA hypermethylation in HNSCC and OSCC in smokers, indicating that the expression profile of miRNAs in oral cells may depend on the form of tobacco used^32^.We identified several miRNAs in chewing tobacco treated oral keratinocytes which are reported to be dysregulated in other cancer types but not in OSCC. miR-4454 and miR-33b-5p were seen to be significantly overexpressed in chewing tobacco treated cells and have been previously reported to be highly expressed in bladder cancer patients^33,34^. Decreased expression of miR-1304-3p is reported in non-small cell lung carcinoma^35^.

Regulation of gene expression at the post-transcriptional level by miRNAs influences a wide variety of pathways, including oncogenic pathways. Since miRNAs are known to be involved in tumorigenesis by inhibiting target proteins, we also studied the expression profile of proteins targeted by significantly dysregulated miRNAs. miR-1304-3p identified as downregulated and its target protein ras GTPase-activating protein-binding protein 1 (G3BP1) was found to be upregulated (1.52 fold; p-value ≤ 0.05) in tobacco treated cells. Overexpression of G3BP1 has been reported in multiple cancers including oral squamous cell carcinoma^36–38^. Knockdown of G3BP1 induced programmed cell death and decreased cell viability in OSCC cell lines^39^. Chromatin remodelling complex protein, SMARCE1 (a target of miR-1266-5p) which was overexpressed (1.64 fold; p-value ≤ 0.05) in tobacco treated cells has been associated with both breast cancer invasion and metastasis in ovarian cell carcinoma^40–42^. We observed downregulation of signal transducer and activator of transcription 1, (STAT1) (0.35 fold; p-value ≤ 0.05) in OKF6/TERT1-Tobacco cells. STAT1 is a target protein of miR-146a-5p (significantly upregulated in tobacco treated cells) and is reported to be downregulated due to promoter methylation in HNSCC. Its exogenous expression in HNSCC cell lines led to growth inhibition and increased chemotherapy-induced apoptosis^43^. GNB2L1 (RACK1 – target of miR-196b-5p) has been reported to promote progression of oral squamous cell carcinoma via the AKT/mTOR pathway and induces OSCC cell migration; invasion and metastasis^44^. We observed a 1.55 fold (p-value ≤ 0.05) overexpression of GNB2L1 and downregulation of miR-196b-5p in the cells chronically treated with chewing tobacco.

We identified significant downregulation of members of miR-200 family in OKF6/TERT1 cells chronically exposed to cigarette smoke. Multiple miR-200 members including miR-200a, miR-200b and miR-200c have been previously reported to be downregulated in head and neck cancers^45–48^. In addition, we also observed decrease in expression of miR-205 in smoke exposed cells. Loss of miR-205 expression has been associated with poor prognosis of head and neck cancer patients^49,50^. miR-200 family target protein, fibronectin 1, FN1 which was seen to be 1.6 fold overexpressed in OKF6/TERT1-Smoke cells is reported to be overexpressed in salivary gland carcinoma and in OSCC patients with lymph node metastasis^51,52^. Transforming growth factor-β 2, TGFB2 which is known to be overexpressed in multiple cancers including OSCC and induces malignancy was 1.8 fold overexpressed (p-value ≤ 0.05) in our data. It is targeted by miR-141-3p which was downregulated in our data. It is suggested that the ability of OSCC cells to secrete TGF-β2 could contribute to clinical progression by maintaining a microenvironment conducive for tumor growth and proliferation^53,54^. In addition to the proteins discussed above we have identified a number of proteins which were overexpressed in smoke exposed cells and inversely correlated to their respective miRNAs, but have not been reported in literature in the context of oral cancer. H2A histone family member Z, H2AFZ (1.7 fold overexpressed; p-value ≤ 0.05) which showed inverse relationship with miR-200a-3p and miR-141-3p has been reported to be highly expressed in breast, prostate, bladder and hepatocellular cancers and enhances tumorigenesis by accelerating cell cycle and epithelial to mesenchymal transition^55–57^. We also observed overexpression of a number of protein targets of miR-205-5p in smoke exposed cells. Reports indicate high expression of squalene monooxygenase (SQLE) in breast and hepatocellular carcinoma and plays a key role in induction of epithelial-mesenchymal transition in esophageal carcinoma^58,59^. Hypomethylation of inositol monophosphatase domain containing 1 (IMPAD1) has been associated with its overexpression in breast carcinoma^60^. Sharieha *et al*., reported upregulation of transmembrane 9 superfamily member 2 (TM9SF2) in breast carcinoma cells when compared to normal cells which in turn promoted growth and survival of carcinoma cells. Laminin B1 (LMNB1) (1.6 fold overexpressed; p-value ≤ 0.05) which is targeted by both miR-200b-3p and miR-200c-3p (downregulated in smoke exposed cells) is reported to be overexpressed in colorectal and hepatocellular carcinoma. It is also observed that its elevation in plasma could be used for early detection of hepatocellular carcinoma^61,62^. Loss of expression of miR-200 family plays a crucial role in the repression of E-Cadherin and promotes EMT^13,63^. We observed change in expression of markers associated with EMT progression in smoke exposed oral cells.

Our data indicates dysregulation of proteins which play a key role in mRNA processing and splicing in tobacco treated cells. We and others have shown in the past that aberrant activation of spliceosome complex-associated proteins has been shown to influence cellular transformation leading to development of cancer, including HNSCC^64,65^.Our proteomic data shows significant overexpression of splicing proteins serine and arginine rich splicing factor 2 (SRSF2) and splicing factor 3b (SF3B1) (1.9 and 1.5 fold overexpressed respectively; p-value ≤ 0.05) in tobacco treated cells. Increased expression of SRSF2 is associated with progression and poor prognosis in human hepatocellular carcinoma^66^. mRNA processing/splicing and chromosome remodelling were thought to traverse different paths but studies are revealing that there is a strong cross talk between the two in executing their vital processes^67^. Mutations in several components of chromatin remodelling complex, SWI/SNF have been linked to malignant transformation and progression of cancer. SWI/SNF-related matrix-associated actin-dependent regulator of chromatin subfamily E member 1 (SMARCE1) is known to play an essential role in breast cancer metastasis by protecting cells against anoikis through the HIF1A/PTK2 pathway^41^. We observed a significant increased expression of SMARCE1 in tobacco treated oral cells.

In contrast, we have observed increased expression of proteins responsible for transcriptional regulation and nucleosome assembly in smoke exposed oral keratinocytes. Acrolein which is a major component of cigarette smoke, forms adducts with histone proteins and specifically inhibits appropriate covalent modifications of cytosolic histone H3 and H4 by inhibiting acetylation and results in aberrant nucleosome assembly. Electrophiles, such as metabolically activated polycyclic aromatic hydrocarbons and alkylating agents are assumed to have the same effect on nucleosome assembly^68^. DNA methyltransferase 1 (DNMT1) which is reported to be overexpressed in response to cigarette smoke in non-small cell lung carcinoma and was found significantly overexpressed (1.9 fold overexpressed; p-value ≤ 0.05) in smoke exposed cells^69^. Overexpression of DNMT1 in lung cancer is associated with increased expression of histone deacetylases (HDACs) which increases the stability of DNMT1 and in turn stimulates cancer progression^70^. Sp1 transcription factor (Sp1) which plays a role in multiple cellular processes including chromatin remodelling is significantly upregulated in the smoke exposed cells. Nicotine promotes lung cancer proliferation via α7-nicotinic acetylcholine receptor (α7-nAChR). It has been unveiled that nicotine induces binding of Sp1 on α7-nAChR promoter, leading to transcriptional activation of α7-nAChR in lung cancer, which in turn facilitates tumor growth and progression^71^.

To the best of our knowledge, this is the first global study employing an integrative analysis approach on high-throughput proteomic and miRNA profiling data to investigate the molecular alterations brought about in immortal oral keratinocytes in response to chewing tobacco and cigarette smoke. Our results indicate that immortal oral keratinocytes treated with chewing tobacco or exposed to cigarette smoke show distinct alterations in both miRNA and their protein targets. In addition, we have also identified a set of high confidence novel miRNAs in both tobacco treated and smoke exposed oral keratinocytes. Taken together, these findings imply that altered expression of miRNAs and their target proteins may be prominently involved in the initiation and progression of oral squamous cell carcinoma. Functional and clinical validation of this data is essential before these findings can be taken forward as potential diagnostic biomarkers in OSCC patients segregated based on the history of tobacco usage.

## Materials and Methods

### Cell culture

Human oral keratinocytes, OKF6/TERT1 cells were a generous gift from Dr. James Rheinwald (Brigham and Women’s Hospital, Boston, MA). OKF6/TERT1 cells are normal oral mucosal epithelial cells immortalized by hTERT^72^. OKF6/TERT1 were cultured and maintained in keratinocytes serum free medium (KSFM) supplemented with 1% penicillin/streptomycin, CaCl_2_ (0.4 mM), bovine pituitary extract (25 µg/ml) and epidermal growth factor (EGF) (0.2 ng/ml). Cells were grown at 37 °C in a humidified 5% CO_2_ incubator.

### Treatment of OKF6/TERT1 cells with chewing tobacco extract and cigarette smoke condensate

To study the chronic exposure effect of chewing tobacco, OKF6/TERT1 cells were treated with 1% chewing tobacco extract for a period of 6 months^73^. To study the chronic effect of cigarette smoke condensate (CSC Murty Pharmaceuticals, Inc, KY) OKF6/TERT1 cells were subjected to chronic exposure with 0.1% CSC in a smoke dedicated incubator for 6 months^74^. All cells were cultured for equal duration of time. Throughout the study, OKF6/TERT1 cells treated with chewing tobacco extract and cigarette smoke condensate have been referred to as “OKF6/TERT1-Tobacco” and “OKF6/TERT1-Smoke” cells, respectively. The parental control cells have been referred to as “OKF6/TERT1-Parental”.

### Cell proliferation assays

OKF6/TERT1-Parental, OKF6/TERT1-Tobacco and OKF6/TERT1-Smoke cells were seeded at a density of 25 × 10^3^ cells. Cellular proliferation was monitored for 6 days where the cells were counted every 48 h using trypan blue exclusion method. All assays were performed in replicates.

### Western blotting

Cells were grown to 80% confluence and the proteins were harvested in RIPA lysis buffer (10 mM Tris pH 7.4, 150 mM NaCl, 5 mM EDTA, 1% Triton-X-100, 0.1% SDS containing protease and phosphatase inhibitor cocktails) and sonicated. Western blot analysis was carried out as described previously^74^ VIM antibody was obtained from Santa Cruz (Santa Cruz Biotechnology, Dallas, TX). Antibodies for E-cadherin were purchased from Cell Signaling (Cell Signaling Technology, Danvers, MA). SOD2 and TGFB2 antibodies were purchased from Cusabio (Baltimore Avenue, MD). β-actin antibody was procured from Sigma (Sigma Aldrich, USA) and was used as loading control.

### Colony formation assays

Colony formation assays were carried out as described previously^75^.OKF6/TERT1-Parental, OKF6/TERT1-Tobacco and OKF6/TERT1-Smoke cells were seeded at a cell density of 8.0 × 10^3^ cells per well in 6-well dishes. The colonies formed were fixed with methanol and stained with 4% methylene blue. Colonies formed was counted for ten randomly selected viewing fields and representative images were photographed at 3x magnification. All experiments were carried out in replicates.

### Cell invasion assays

Invasion assays were performed in a transwell system (BD Biosciences) with Matrigel-coated filters, and cellular invasion was evaluated after 48 h as described previously^75^. Briefly, invasiveness of the cells was assayed in the membrane invasion culture system using polyethylene terephthalate (PET) membrane (8-μm pore size) in the upper compartment of a transwell coated with Matrigel (BD BioCoat Matrigel Invasion Chamber; BD Biosciences). The cells were seeded at 2.0 × 10^4^ cells per 500 μl of serum free media on the Matrigel-coated PET membrane in the upper compartment. The lower compartment was filled with complete growth media and the plates were maintained at 37 °C for 48 h. At the end of the incubation time, the upper surface of the membrane was wiped with a cotton-tip applicator to remove non migratory cells. Cells that migrated to bottom side of membrane were fixed and stained using 4% methylene blue. The number of cells that penetrated was counted for ten randomly selected viewing fields at 10x magnification. All experiments were performed in triplicates.

### RNA isolation and miRNA enrichment

Total RNA, including small RNA, was isolated using the Qiagen RNAeasy isolation kit from OKF6-TERT1-Parental, OKF6/TERT1-Tobacco and OKF6/TERT1-Smoke cells. The quantity and quality of RNA was analyzed on denaturing agarose gel as well as on Bioanalyzer RNA 6000 Pico chip. RNA isolated from each condition was used to construct sequencing libraries with the Illumina TruSeq Small RNA Sample Prep Kit (Illumina, USA) as per manufacturer’s instructions. Briefly, 3′ and 5′ adapters were sequentially ligated to small RNA molecules and ligation products were subjected to reverse transcription to create single stranded cDNA. To selectively enrich fragments with adapter molecules on both ends, the cDNA was amplified with 50 PCR cycles using a common primer and a primer containing an index tag to allow sample multiplexing. The amplified cDNA constructs were gel purified, and validated by checking the size, purity, and concentration of the amplicons on the Agilent Bioanalyzer High Sensitivity DNA chip (#5067-4626, Genomics Agilent, Santa Clara, CA). The libraries were pooled in equimolar amounts, and sequenced on an Illumina HiSeq 2500 instrument to generate 50-base pair reads.

### miRNA sequencing and data analysis

The quality of the raw reads was checked using FASTQC (http://www.bioinformatics.babraham.ac.uk/projects/fastqc) for OKF6/TERT1-Parental, OKF6/TERT1-Tobacco and OKF6/TERT1-Smoke samples. Cutadapt (http://cutadapt.readthedocs.io/en/stable/index.html) was used to remove low quality bases with Phred score ≤30 and adaptor sequences from 5′ and 3′ end of the read. The adaptor trimmed reads were aligned against nc-RNA sequence databases (GtRNA, piRNA, Rfam& snoRNA)^76–79^ using Bowtie (version 1.1.2)^80^. Remaining reads were used for known miRNA identification. These reads were first aligned to mature and precursor sequence from miRBase 21 miRNA database (Human)^81^ using Bowtie. Unaligned reads were further aligned against human genome hg19 using miRDeep2 (version 2.0.0.6). Known miRNA that are quantified with at least 10 reads in both samples of a pairs i.e. OKF6/TERT1-Tobacco vs. OKF6/TERT1-Parental and OKF6/TERT1-Smoke vs. OKF6/TERT1-Parental were further investigated for differential expression using DESeq^82^.

Novel miRNAs were assessed for the genomic location using UCSC BLAT^83^. They were also queried against database YM500V3^84^ using ‘genomic location search’ option. YM500V3 is a repository of novel miRNA built using >8,000 smRNA data-sets pertaining to various cancers along with their CLIP-Seq results from different cell lines.

### Sample preparation, in-solution digestion and TMT labelling

OKF6/TERT1-Parental, OKF6/TERT1-Tobacco and OKF6/TERT1-Smoke cells were cultured and grown till 80% confluence. The cells were serum starved for 12 hours in ice-cold 1X PBS, lysed in 0.5% of SDS buffer followed by sonication (Branson Sonifier, Danbury, CT) and centrifugation. Supernatant was collected and protein estimation was carried out by using bicinchoninic acid assay (BCA)^85^. In-solution trypsin digestion of all three conditions was carried out as previously described^86^. Equal amounts of protein was taken from each condition, reduced using 5 mM dithiothreitol (DTT) and kept for incubation for 20 min at 60 °C. Alkylation was carried out using iodoacetamide (IAA) (20 mM) and incubated for 10 min in the dark at room temperature. The reduced and alkylated proteins were then precipitated using chilled acetone (1:5 v/v of sample) and incubated at −80 °C for 4–6 h to ensure complete removal of SDS from sample. Trypsin digestion was carried out at 37 °C for 12–16 h with an enzyme to substrate ratio of 1:50 (catalogue no. V5111; Promega). After trypsin digestion, the peptides were labelled with TMT reagents as per the manufacturer’s instructions. Briefly, peptide samples were dissolved in 50 mM TEABC (pH 8.0) and added to TMT reagents dissolved in anhydrous acetonitrile. Peptides from OKF6/TERT1-Parental cells and OKF6/TERT1-Tobacco were labelled with 126 and 129 C, respectively. In a separate experiment, OKF6/TERT1-Parental cells and OKF6/TERT1-Smoke cells were labelled with 128 N and 129 C, respectively. After incubation at room temperature for 1 h, the reaction was quenched with 5% hydroxylamine. The labelled samples from all conditions were pooled and subjected to fractionation.

### Basic pH reverse-phase liquid chromatography

TMT labelled peptides were fractionated using a basic pH reverse HPLC system (Agilent,1290 series) by injecting 900 µL of the sample reconstituted in solvent A (10 mM TEABC in water; pH 8.5) on Xbridge (4.6 × 250 mm, 5 µm; Waters) column. The peptides were resolved by using a gradient of organic solvent; 2% solvent A (10 mM TEABC in water; pH 8.5) to 45% solvent B (10 mM TEABC in 90% acetonitrile, pH 8.5) over 100 min and further taken to 100% solvent B before coming back to 2% solvent A for re-equilibration^87^. A total of 96 fractions were collected from 96-well plate, concatenated to 12 fractions, and dried under vacuum for LC-MS^2^/MS^3^ analysis.

### LC-MS^2^/MS^3^ analysis

Briefly all the 12 fractions were reconstituted in 0.1% formic acid and analyzed in triplicate on an Orbitrap Fusion Tribrid mass spectrometer (Thermo Scientific) that was interfaced with an Easy-nLC II nanoflow liquid chromatography system (Thermo Scientific). Peptides reconstituted in 0.1% formic acid were loaded onto a trap column that was packed (75 µm × 2 cm) with Magic C18 AQ (Michrom Bioresources, Inc.) at a flow rate of 300 nL/min. Peptides were separated on an analytical column (75 µm × 20 cm) at a flow rate of 300 nL/min by using a linear gradient of 7–25% solvent B (0.1% formic acid in 95% acetonitrile) over 75 min which was increased to 35% for next 25 minutes. The total run time was set to 120 min and acquisition was carried out.

### Data analysis

Proteome Discoverer (version 2.1.0.81) software suite (Thermo Fisher Scientific) was used for MS/MS searches using SEQUEST and Mascot (version 2.4.1; Matrix 617 Science) algorithms againt NCBI RefSeq human protein database (version 75 containing 36,709 protein sequences and known contaminants). The search parameters included trypsin as the protease with a maximum of 2 missed cleavages allowed; oxidation of methionine was set as a dynamic modification, carbamidomethylation of cysteine and TMT modification at peptide N-terminus and lysine were set as static modifications. Precursor ion mass tolerance and fragment ion mass tolerance were allowed with 10 ppm and 0.05 Da, respectively and all the PSMs were identified with 1% FDR^88^. Proteins were identified at MS^2^ level and quantification was done at MS^3^ level.

### Data availability

Raw sequencing data is available in SRA (Sequencing Read Archive) database with accession number PRJNA398169.

## Electronic supplementary material

Supplementary information
