# Supplementary material for: Cigarette smoke and chewing tobacco alter expression of different sets of miRNAs in oral keratinocytes
Source: Sci Rep. 2018 May 4;8:7040. doi: 10.1038/s41598-018-25498-2 (PMC5935709; doi:10.1038/s41598-018-25498-2)
Supplement: Supplementary file 1 — Supplementary information [file 41598_2018_25498_MOESM1_ESM.pdf]

Cigarette smoke and chewing tobacco alter expression of different sets of miRNAs in oral keratinocytes

Mohd Younis Bhat[1,2#], Jayshree Advani[1,3#], Pavithra Rajagopalan[1,4], Krishna Patel[1,2], Vishalakshi Nanjappa[1], Hitendra S. Solanki[1,4], Arun H. Patil[1,4,5], Firdous A. Bhat[1,2], Premendu P. Mathur[4], Bipin Nair[2], T.S. Keshava Prasad[1,5], Joseph A. Califano[6], David Sidransky[7], Harsha Gowda[1,*] and Aditi Chatterjee[1,*]

[1] Institute of Bioinformatics, International Technology Park, Bangalore 560066, India
[2] School of Biotechnology, Amrita Vishwa Vidyapeetham, Kollam, Kerala 690525, India
[3] Manipal Academy of Higher Education, Manipal, Karnataka 576104, India
[4] School of Biotechnology, Kalinga Institute of Industrial Technology, Bhubaneswar 751024, India
[5] Center for Systems Biology and Molecular Medicine, Yenepoya, Mangalore 575020, India
[6] Department of Surgery, UC San Diego, Moores Cancer Center, La Jolla, CA 92093, USA
[7] Department of Otolaryngology-Head and Neck Surgery, Johns Hopkins University School of Medicine, Baltimore, MD 21231, USA

[#] These authors contributed equally to the manuscript

***Corresponding Authors:**
Aditi Chatterjee, Ph.D.
Faculty Scientist
Institute of Bioinformatics
7th floor, Discoverer building,
International Tech Park,
Bangalore 560 066
India
Tel.: 91-80-28416140
Email: aditi@ibioinformatics.org

and

Harsha Gowda, Ph.D.
Faculty Scientist
Institute of Bioinformatics
7th floor, Discoverer building,
International Tech Park,
Bangalore 560 066
India
Tel.: 91-80-28416140
E-mail: harsha@ibioinformatics.org

Supplementary Methods

Reverse transcriptase PCR

Total RNA was isolated from OKF6/TERT1-Parental, OKF6/TERT1-Tobacco and OKF6/TERT1-Smoke cells using the Qiazol Lysis Reagent (Catalog No. 79306, Qiagen, Valencia, CA) according to the manufacturer's instructions. cDNA synthesis was carried out with 1μg of total RNA using Protoscript II First Strand cDNA synthesis Kit (Catalog No. E6560S, New England Biolabs, USA) PCR was carried out using 3 μL of cDNA, 10 μM of each forward and reverse primers, 50 mM $MgCl_{2,}$ 10 mM dNTP mix, 0.5 Units of Taq polymerase and PCR buffer in 25 μl reaction volume. Thermal cycling conditions performed to amplify the target sequence are as follows: initial denaturation cycle of 94°C for 2 min, 30 cycles of amplification with 94°C for 45 sec, primer-specific annealing temperature was 57°C for all genes except SP1 and SQLE for which was 53°C for 30 sec and 72°C for 45 sec. RT–PCR was performed using real-time PCR cycler, the Rotor-Gene Q (QIAGEN'S). GAPDH gene was used as a loading control. The list of forward and reverse primers used for validation is provided in table below. A non-template and non-specific control was for all reactions. We could not detect any amplification in either of the control reactions. All reactions were carried out in triplicate and p-value was determined. Amplicon sizes were confirmed by running equal volumes of reaction on 2% agarose gel along with 100 bp DNA ladder.

**Gene Symbol**	**GenBank Accession No.**	**Forward Primer (5' – 3')**	**Reverse Primer (5' – 3')**
MGLL	NM_007283.6	GCGTGCTCTCTCGGAATAAG	AGTCTTGTCCTGGCTCTTGG
SP1	NM_138473.2	TCACCCAATGAGAACAGCAA	CACAACATACTGCCCACCAG
SQLE	NM_003129.3	TGGTGGAGGAATGACTGTTG	CATAAAGAGCCTGAGCAAGGA
LMNB1	NM_005573.3	AGGCGAAGAAGAGAGGTTGA	GGCGGAATGAGAGATGCTAA
SRSF2	NM_003016.4	TCCAAGTCCAAGTCCTCGTC	GACACCGCTCCTTCCTCTTC
STAT1	NM_007315.3	CCTTCTTCCTGACTCCACCA	TCCTTACAAAACCTCGTCCA
KPNA2	NM_001320611.1	TTTGAATCTGCTTGGGCACT	GGAACACTGAGCCATCACCT
CTPS1	NM_001905.3	CGGGAACTTAGAGGACTTGG	GGGACTCGGTAGATGGATGA
GAPDH	NM_002046.6	CCACCCAGAAGACTGTGGAT	TTCTAGACGGCAGGTCAGGT

Supplementary Figure Legends

Supplementary Figure S1: Experimental workflow employed to profile miRNA expression in chewing tobacco treated and cigarette smoke exposed OKF6/TERT1 cells.
RNA was isolated from OKF6/TERT1 cells chronically treated with chewing tobacco and cigarette smoke. Samples were processed further depending on the integrity of the isolated RNA and miRNA enrichment was carried out. Illumina HiSeq 2500 platform was employed to carry out miRNA sequencing. Data was processed further to identify dysregulated miRNAs.
Experimental workflow employed to analyze the proteome of chewing tobacco and cigarette smoke treated OKF6/TERT1 cells.
OKF6/TERT1 cells were chronically treated with chewing tobacco and cigarette smoke. Equal amount of protein from each sample were digested and labelled with TMT reagents. The peptides were fractionated by basic pH reverse-phase liquid chromatography (bRPLC). The fractionated samples were subjected to LC-MS^2/MS^3 analysis on a Orbitrap Fusion Tribrid mass spectrometer. Proteins were identified at the MS^2 level and quantified at the MS^3 level. The data was searched and quantitated using the Sequest and Mascot search algorithms.

Supplementary Figure S2A: Verification of a subset of identified targets by reverse transcriptase-PCR analysis. Relative expression levels of a subset of targets in OKF6/TERT1-Parental and OKF6/TERT1-Tobacco cells normalized to GAPDH. SOD2 expression in parental and tobacco treated cells was validated using Western blot analysis, β -actin was used as loading control (*p≤0.05; **p≤0.01; ***p≤0.001).

Supplementary Figure S2B: Verification of a subset of identified targets by reverse transcriptase-PCR analysis. Relative expression levels of a subset of targets in OKF6/TERT1-Parental and OKF6/TERT1-Smoke cells normalized to GAPDH**.** TGFB2 expression in parental and smoke exposed cells was validated using Western blot analysis, β-actin was used as loading control (*p≤0.05; **p≤0.01; ***p≤0.001).

Supplementary Figure S3A-C: Secondary structures of novel miRNAs identified in OKF6/TERT1-Parental cells.

Supplementary Figure S4A-F: Secondary structures of novel miRNAs identified in OKF6/TERT1-Tobacco cells**.**

Supplementary Figure S5A-R: Secondary structures of novel miRNAs identified in OKF6/TERT1-Smoke cells**.**

Supplementary Tables

Supplementary Table S1(A): miRNA expression of OKF6/TERT1-Tobacco cells

Supplementary Table S1(B): miRNA expression of OKF6/TERT1-Smoke cells

Supplementary Table S2(A): List of all proteins and peptides identified in OKF6/TERT1 cells chronically treated with chewing tobacco using Sequest and Mascot search algorithms

Supplementary Table S2(B): List of all proteins and peptides identified in OKF6/TERT1 cells chronically treated with cigarette smoke using Sequest and Mascot search algorithms

Supplementary Table S3(A): A list of significantly dysregulated miRNAs and their targets identified in the proteomic data set in OKF6/TERT1-Tobacco cells. Significantly dysregulated miRNAs and their corresponding targets which showed a directional relationship are highlighted (miRNAs and protein targets which are upregulated are highlighted in red and miRNAs and protein targets which are downregulated are in blue color).

Supplementary Table S3(B): A list of significantly dysregulated miRNAs and their targets identified in the proteomic data set in OKF6/TERT1-Smoke cells. Significantly dysregulated miRNAs and their corresponding targets which showed a directional relationship are highlighted (miRNAs and protein targets which are upregulated are highlighted in red and miRNAs and protein targets which are downregulated are in blue color).

Supplementary Table S4(A): Novel miRNAs identified in OKF6/TERT1-Parental cells

Supplementary Table S4(B): Novel miRNAs identified in OKF6/TERT1-Tobacco cells

Supplementary Table S4(C): Novel miRNAs identified in OKF6/TERT1-Smoke cells

NM-OKF6-P: Novel miRNAs identified in OKF6/TERT1-Parental cells
NM-OKF6-T: Novel miRNAs identified in OKF6/TERT1-Tobacco cells
NM-OKF6-S: Novel miRNAs identified in OKF6/TERT1-Smoke cells
NM-OKF6-ST: Novel miRNAs identified in both OKF6/TERT1-Tobacco and OKF6/TERT1-Smoke cells
TR: Total number of reads
MR: Reads supporting mature miRNA sequences
SR: Reads supporting star sequence of precursor
LR: Reads supporting loop region of precursor
MPC: PhastCon100way score of novel mature miRNA
PPC: PhastCon100way score of novel precursor

Supplementary Figure S1

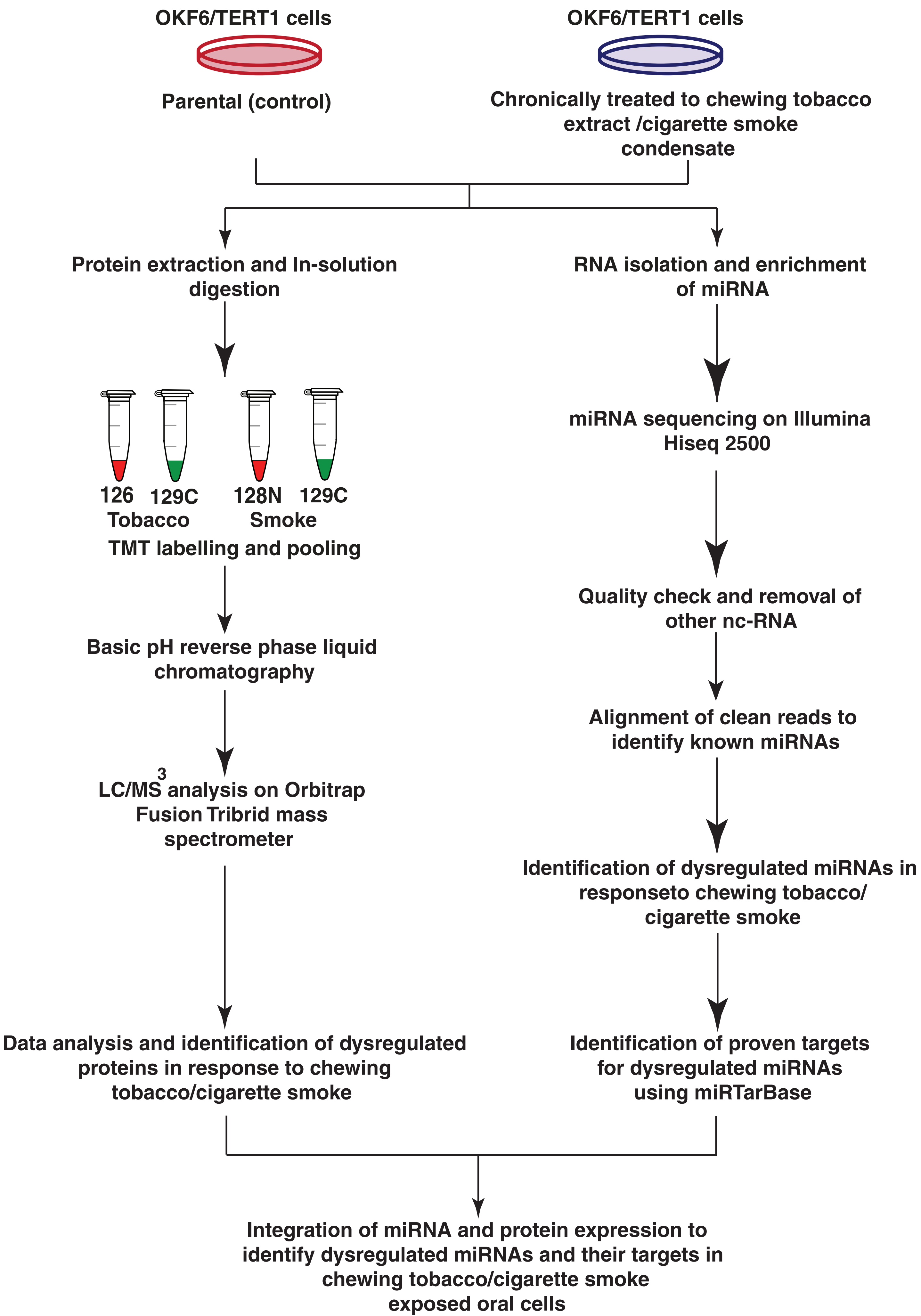

Supplementary figure S2

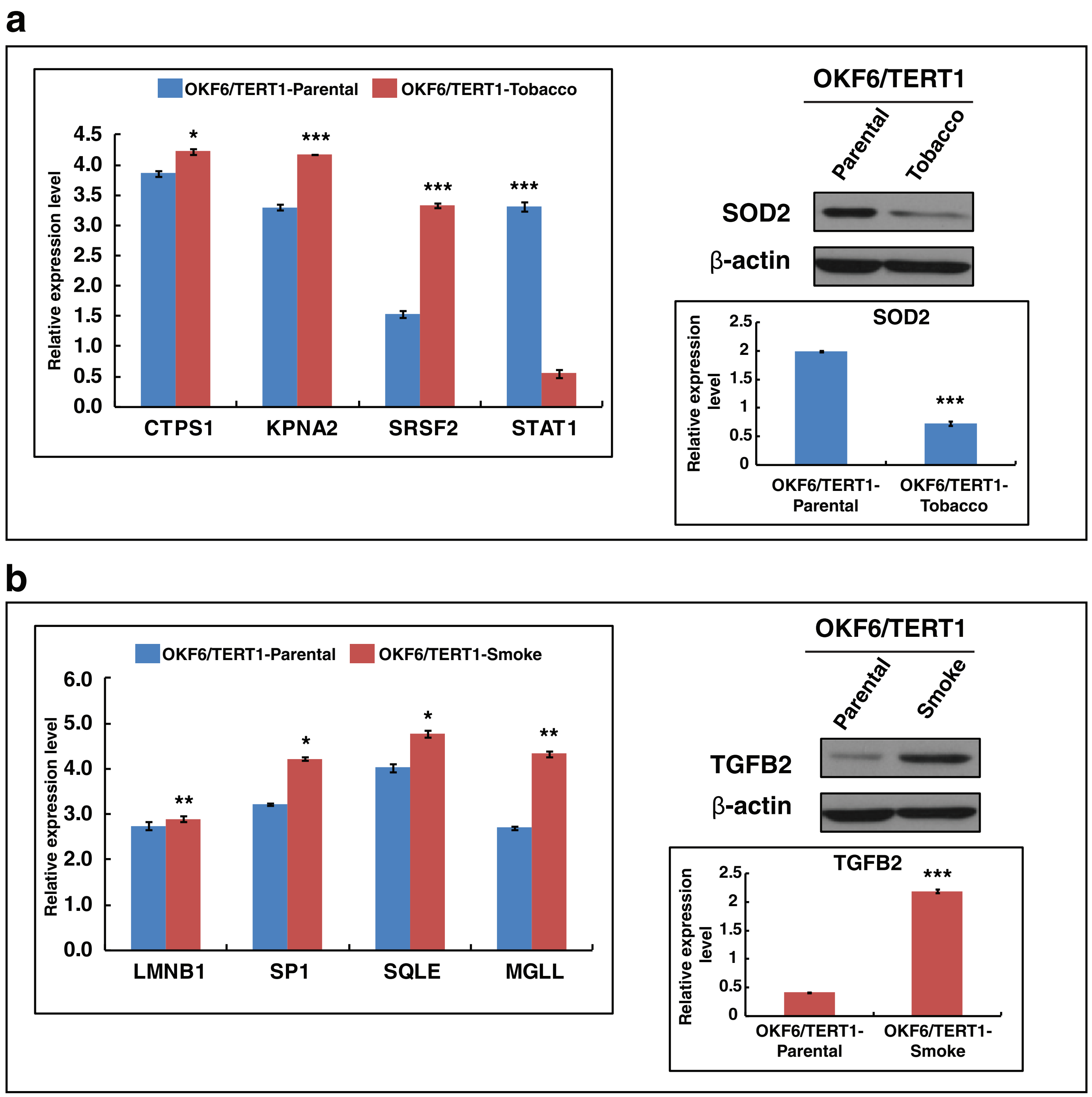

Supplementary Figure S3

a

Novel miRNA ID : NM-OKF6-P-01-5p
Score total : 209
Score for star read(s): 3.9
Score for read counts 206.6
Score for mfe : 0.7
Score for randfold : -2.2
Total read count : 417
Mature read count : 320
Loop read count : 0
Star read count : 97

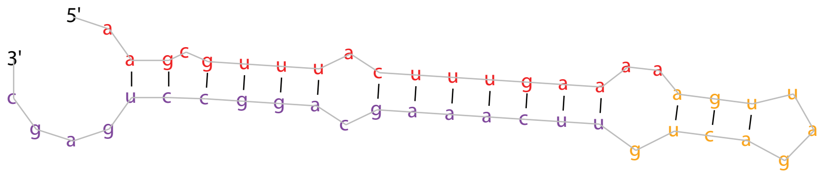

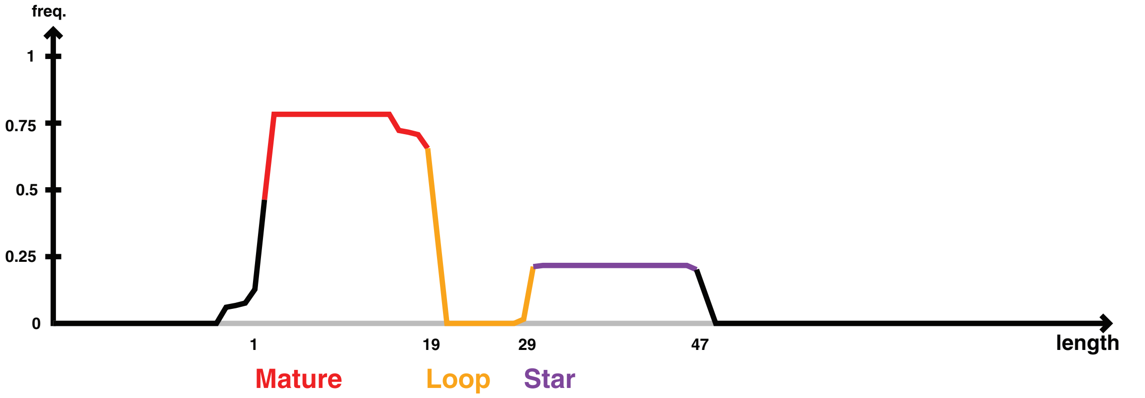

5'- gcugagugucccguggcccaaagcguuuacuuugaaaaaguuagacuguucaaagcaggccugagccaccuagauaucucagcuaggaaugauggaauagaaccgcca -3'

b

Novel miRNA ID : NM-OKF6-P-02-3p
Score total : 230.8
Score for star read(s): 3.9
Score for read counts 224.4
Score for mfe : 0.8
Score for randfold : 1.6
Total read count : 452
Mature read count : 450
Loop read count : 0
Star read count : 2

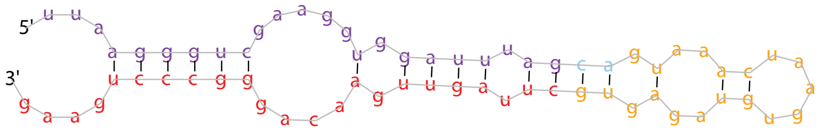

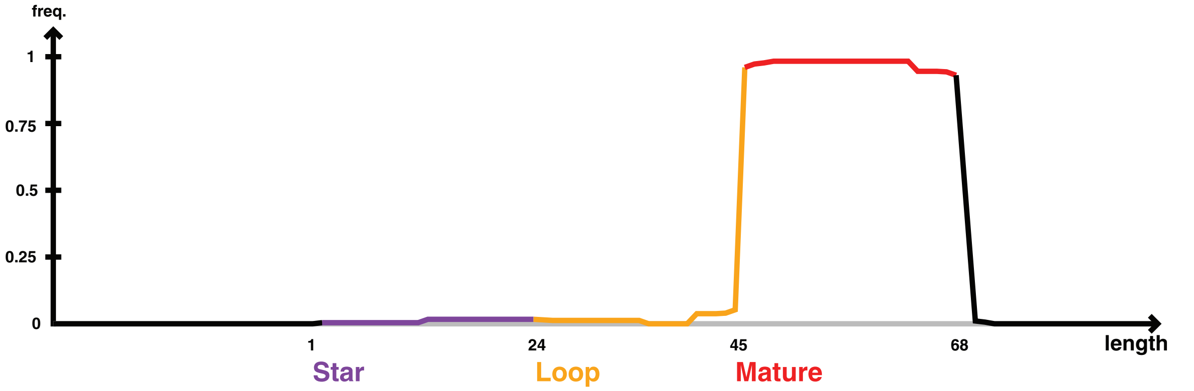

5'- gcuccccaugagggagcuuaugaaauuuaagggucgaagguggauuuagcaguaaacuaaguguagagugcuuaguugaacagggcccugaagcgcauacacaccgcccguca -3'

c

Novel miRNA ID	:	NM-OKF6-P-03-3p
Score total	:	1.4
Score for star read(s):		-1.3
Score for read counts		0
Score for mfe	:	1.1
Score for randfold	:	1.6
Total read count	:	1333
Mature read count	:	857
Loop read count	:	476
Star read count	:	0

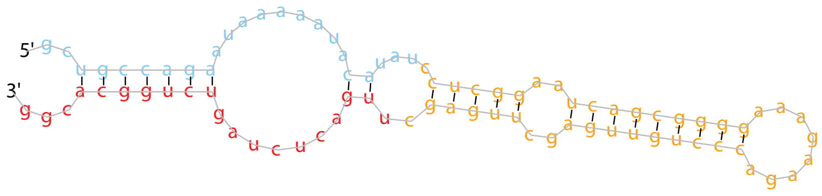

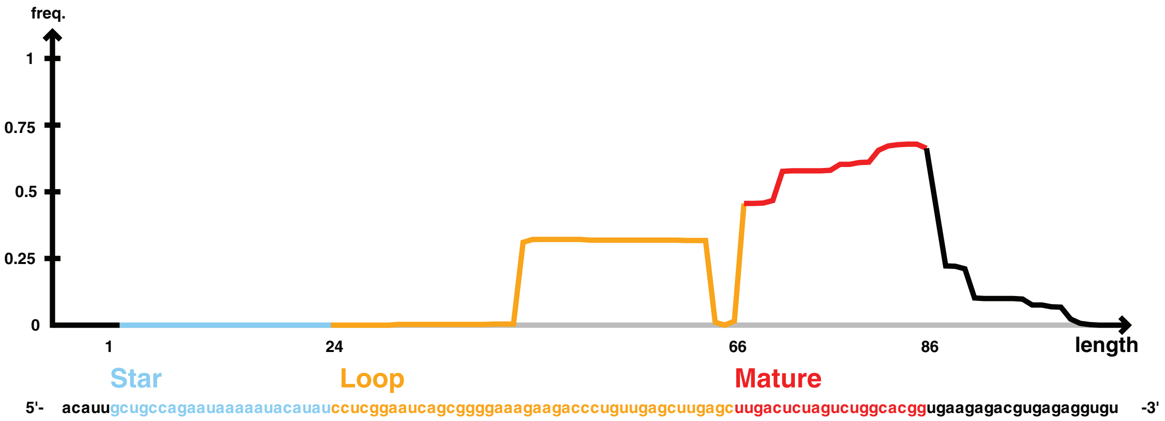

Supplementary Figure S4

a

Novel miRNA ID	:	NM-OKF6-T-01-5p
Score total	:	0.7
Score for star read(s)	:	-1.3
Score for read counts		0
Score for mfe	:	0.4
Score for randfold	:	1.6
Total read count	:	672
Mature read count	:	670
Loop read count	:	2
Star read count	:	0

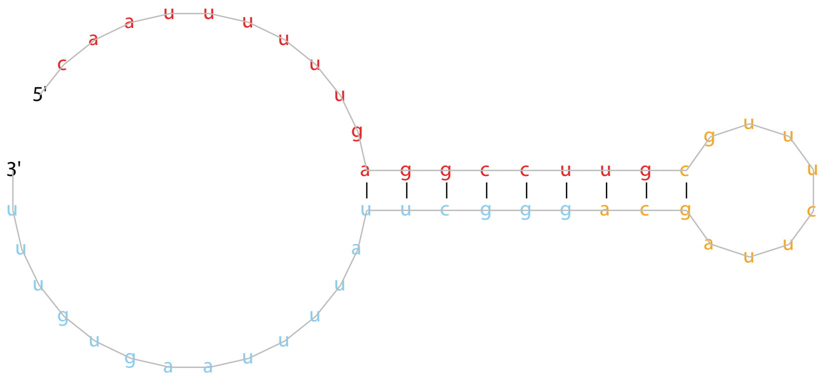

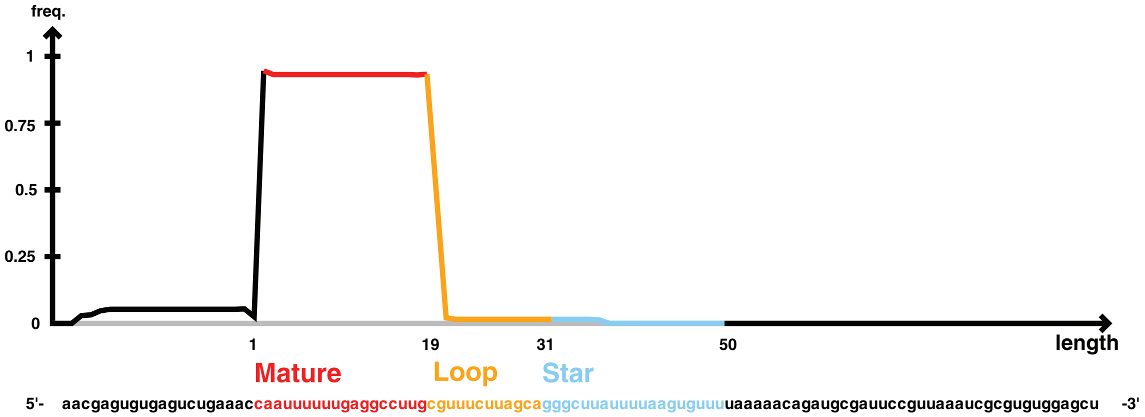

b

Novel miRNA ID	:	NM-OKF6-T-02-3p
Score total	:	0.8
Score for star read(s)	:	-1.3
Score for read counts		0
Score for mfe	:	0.5
Score for randfold	:	1.6
Total read count	:	67
Mature read count	:	62
Loop read count	:	1
Star read count	:	4

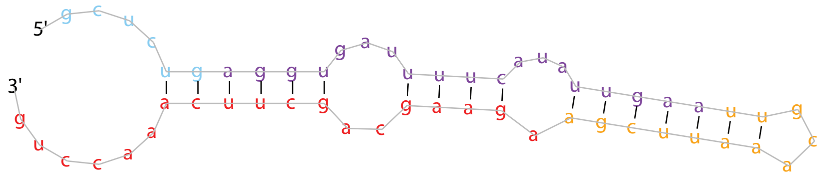

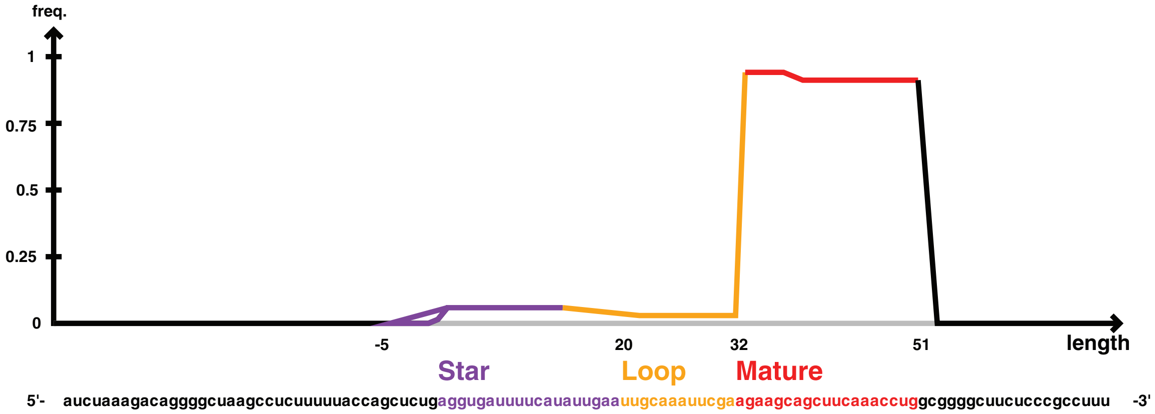

c

Novel miRNA ID : NM-OKF6-T-03-5p
Score total : 63.1
Score for star read(s): 3.9
Score for read counts 70.5
Score for mfe : -9
Score for randfold : -2.2
Total read count : 150
Mature read count : 148
Loop read count : 0
Star read count : 2

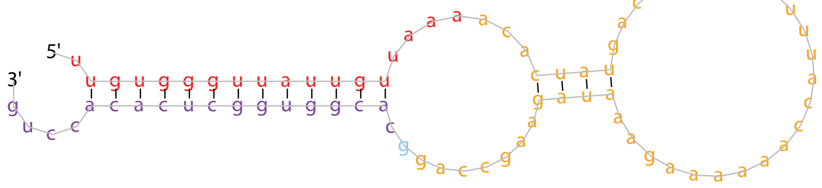

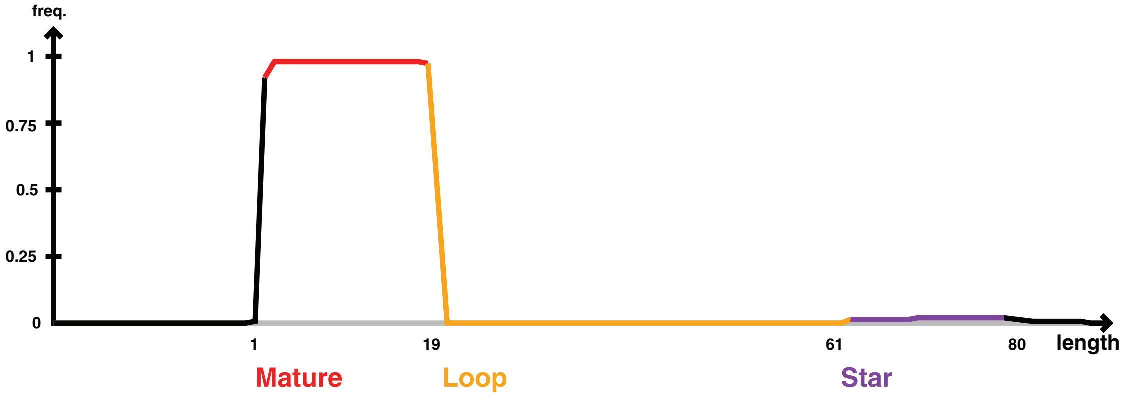

5'- uuuucugagaugaguuuacauuguggguuauuguuaaaacacuaugacaaaauauuuaccaaaaaagaaauagaagccaggcacgguggcucacaccuguaaucccag -3'

d

Novel miRNA ID : NM-OKF6-T-04-5p
Score total : 9855.8
Score for star read(s): 3.9
Score for read counts 9855.6
Score for mfe : -1.3
Score for randfold : -2.2
Total read count : 19343
Mature read count : 19326
Loop read count : 0
Star read count : 17

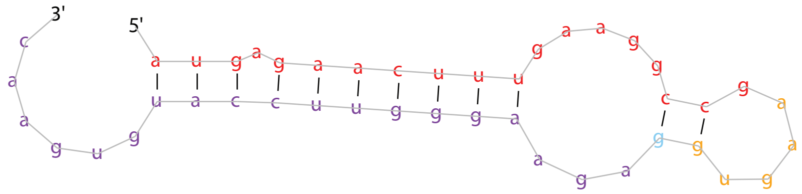

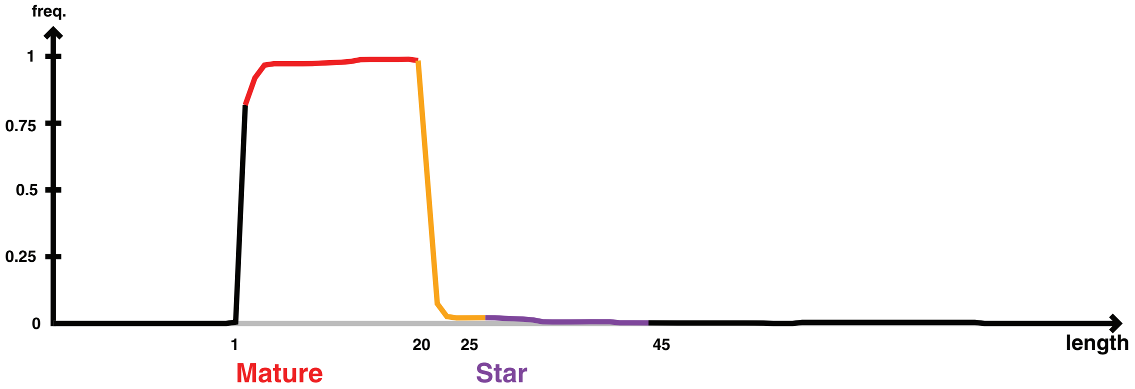

5'- cauuguucauacuuuuuaaugagaacuuugaaggccgaaguggagaaggguuccaugugaacagcaguugaacgugugucagucaguccugagagaagggccagcgcca -3'

e

Novel miRNA ID : NM-OKF6-ST-01-3p
Score total : 1.4
Score for star read(s): -1.3
Score for read counts 0
Score for mfe : 1.1
Score for randfold : 1.6
Total read count : 1726
Mature read count : 926
Loop read count : 800
Star read count : 0

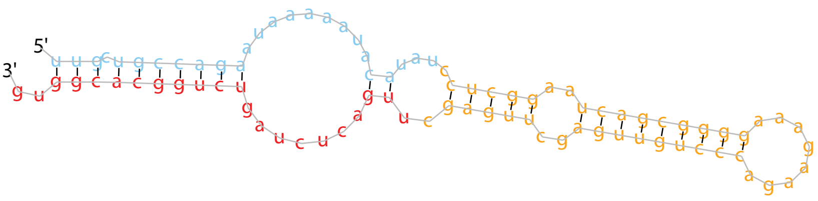

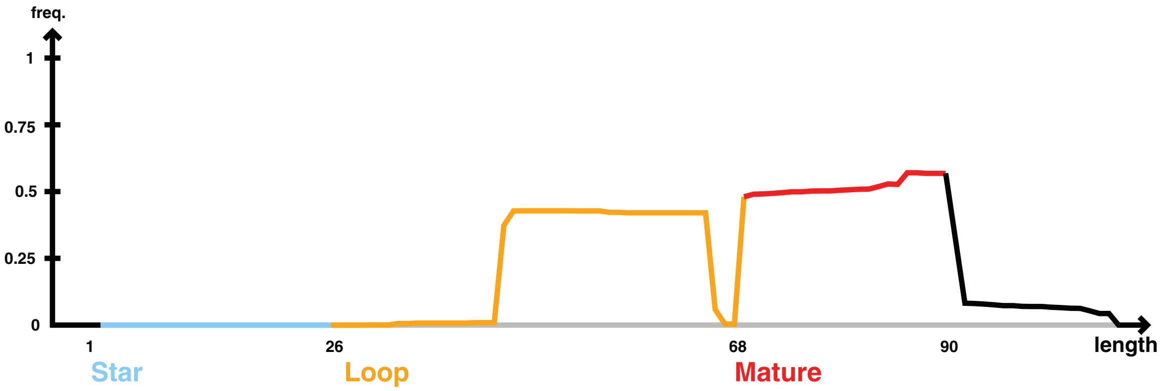

5'- acauugcugccagaauaaaaauacauauccucggaaucagcggggaaagaagacccuguugagcuugagcuugacucuagucuggcacggugaagagacgugagagguguag -3'

f

Novel miRNA ID : NM-OKF6-ST-02-5p
Score total : 27.9
Score for star read(s): 3.9
Score for read counts 21
Score for mfe : 1.4
Score for randfold : 1.6
Total read count : 53
Mature read count : 46
Loop read count : 0
Star read count : 7

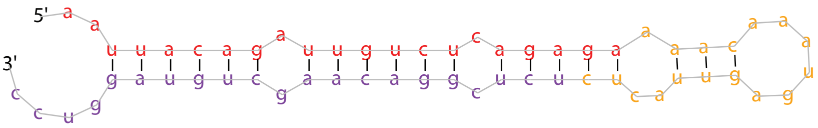

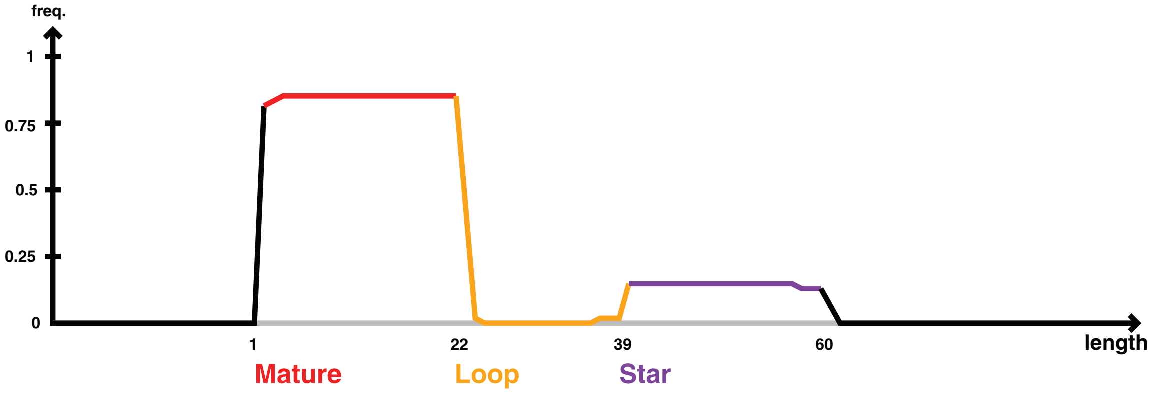

5'- aaggauaauaggacaagacaaauuacagauugucucagagaaaacaaaugaguuacucucucggacaagcuguagguccuaccuaaauguccagcaggacauuagacaguc -3'

Supplementary Figure S5

a

Novel miRNA ID : NM-OKF6-S-01-3p
Score total : 13.9
Score for star read(s): 3.9
Score for read counts 6.2
Score for mfe : 2.2
Score for randfold : 1.6
Total read count : 24
Mature read count : 22
Loop read count : 0
Star read count : 2

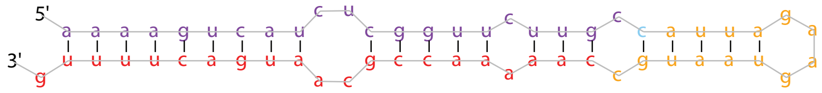

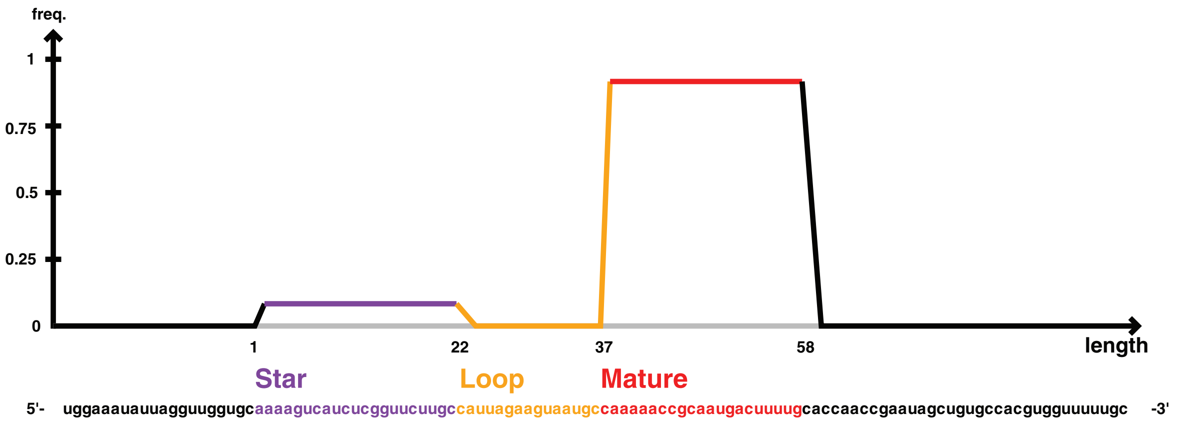

b

Novel miRNA ID : NM-OKF6-S-02-5p
Score total : 11.4
Score for star read(s): 3.9
Score for read counts 3.7
Score for mfe : 2.2
Score for randfold : 1.6
Total read count : 19
Mature read count : 17
Loop read count : 0
Star read count : 2

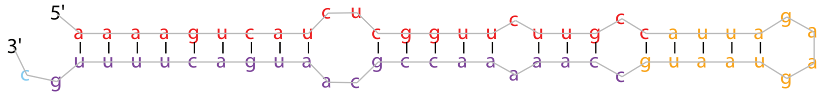

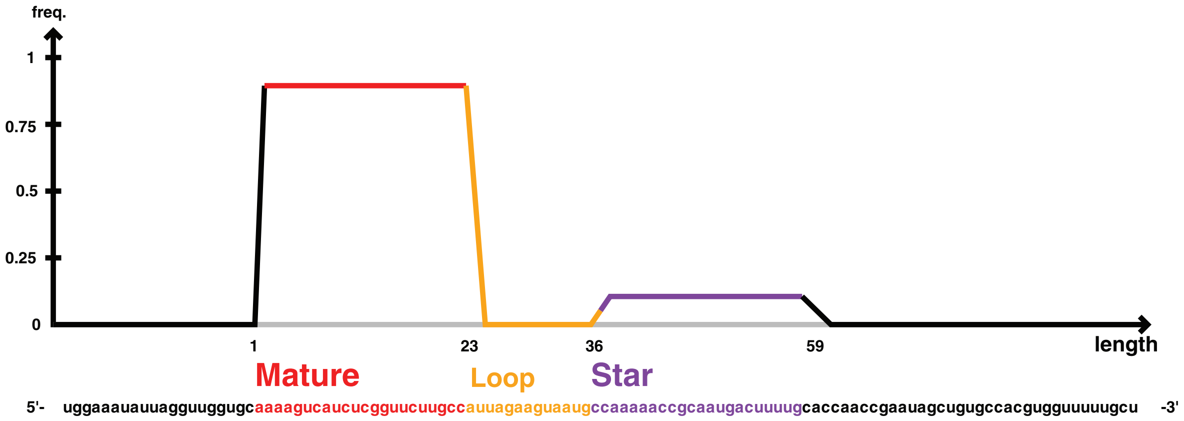

c

Novel miRNA ID	: NM-OKF6-S-03-5p
Score total	: 8.2
Score for star read(s):	3.9
Score for read counts	5.2
Score for mfe	: 1.3
Score for randfold	: -2.2
Total read count	: 22
Mature read count	: 19
Loop read count	: 0
Star read count	: 3

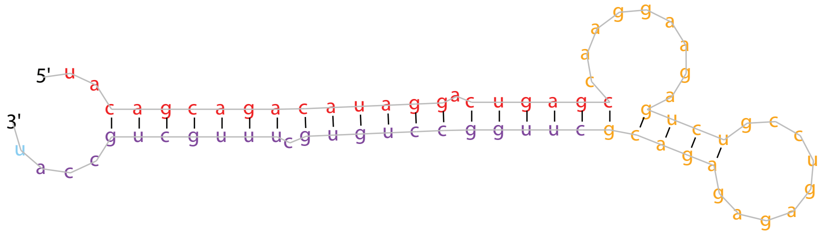

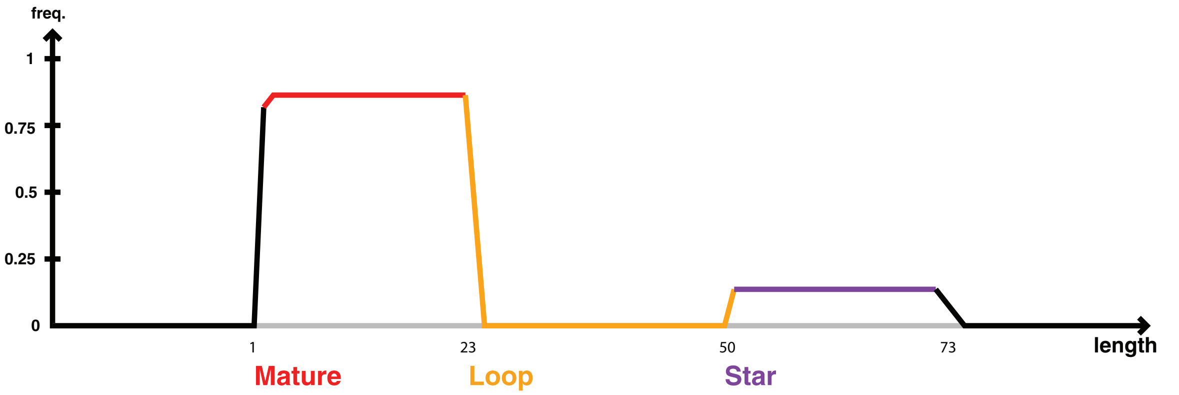

5'- uugagagaaggagaggaaga**uacagcagacauaggacugagc**caaggaagagucugccugagagagacgcuuggccugugcuuugcugccauccgugcggccuuggccacau -3'

d

Novel miRNA ID	: NM-OKF6-S-04-3p
Score total	: 40.7
Score for star read(s):	3.9
Score for read counts	37.3
Score for mfe	: 1.6
Score for randfold	: -2.2
Total read count	: 85
Mature read count	: 49
Loop read count	: 0
Star read count	: 36

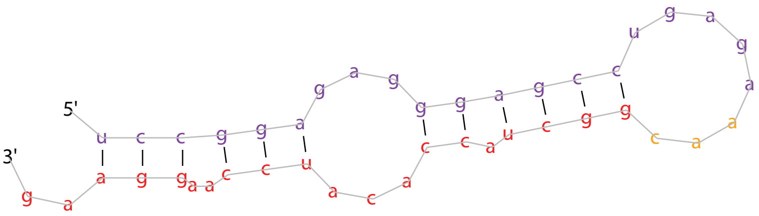

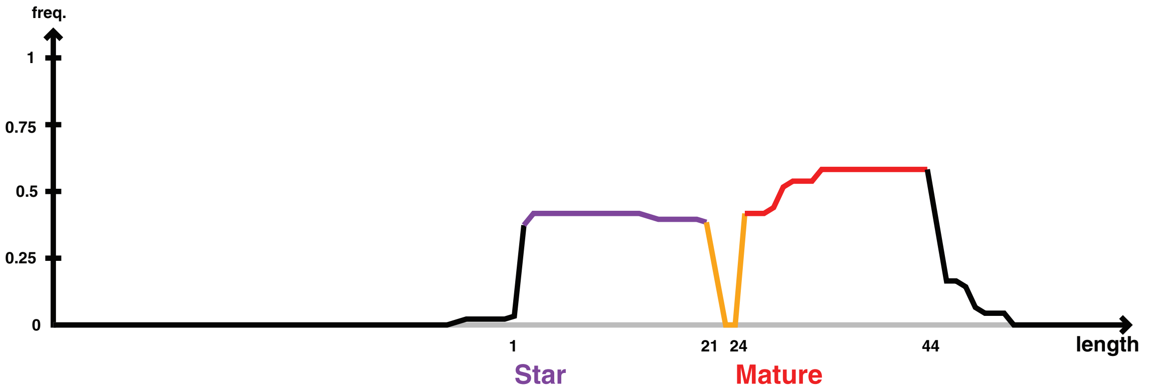

5'- ccgugccuaccauggugaccacgggugacggggaaucaggguucgauuccggagagggagccugagaaacggcuaccacauccaaggaaggcagcaggcgcgcaaauuac -3'

e

Novel miRNA ID	:	NM-OKF6-S-05-5p
Score total	:	7.3
Score for star read(s):		3.9
Score for read counts		3.2
Score for mfe	:	2.4
Score for randfold	:	-2.2
Total read count	:	18
Mature read count	:	13
Loop read count	:	0
Star read count	:	5

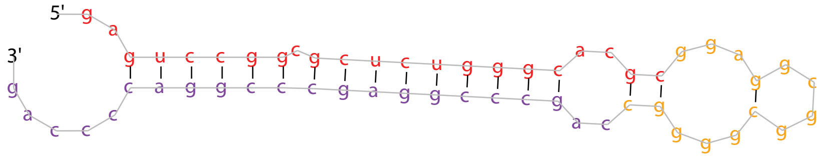

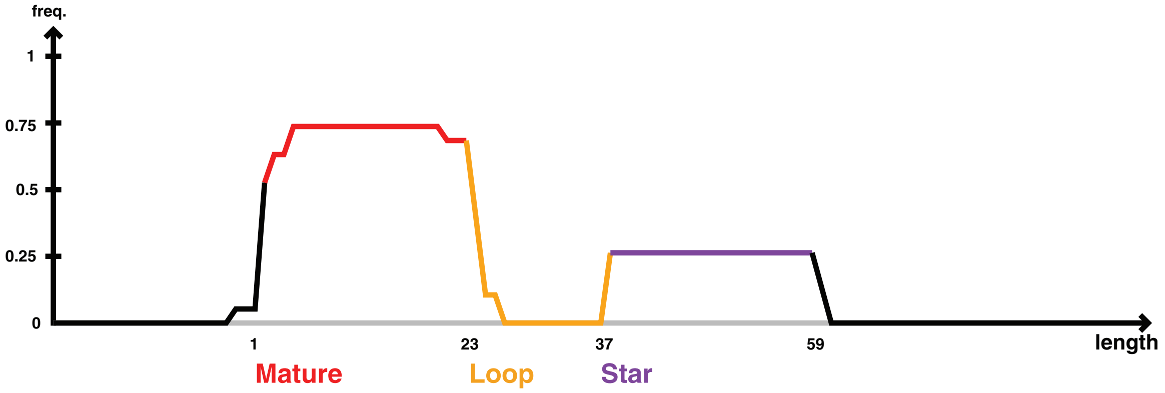

5'- agucacugcagcugcugagcgaguccggcgcucugggcacgcggaggcggcggggccagcccggagcccggaccccagcgcccgccccugccgguucccgcgcggcggccca -3'

f

Novel miRNA ID	:	NM-OKF6-S-06-5p
Score total	:	17.3
Score for star read(s):		3.9
Score for read counts		9.3
Score for mfe	:	2.5
Score for randfold	:	1.6
Total read count	:	30
Mature read count	:	28
Loop read count	:	0
Star read count	:	2

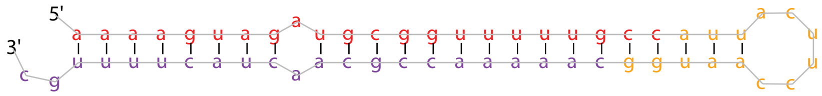

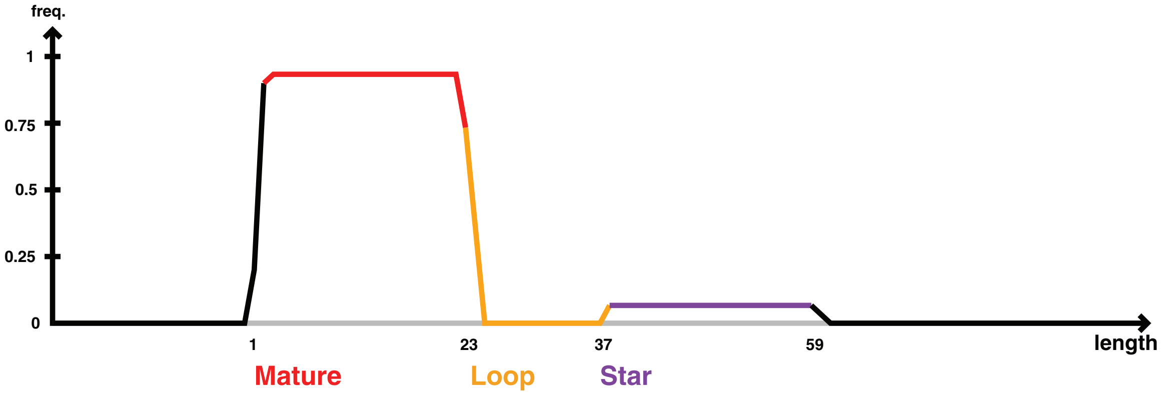

5'- ggauuuauauagguuggugcaaaaguagaugcgguuuuugccauuacuuccaauggcaaaaaccgcaacuacuuuugcaccaaccuaguaacagaacuaacacuggaaaaug -3'

g

Novel miRNA ID : NM-OKF6-S-07-5p
Score total : 18
Score for star read(s): 3.9
Score for read counts 9.8
Score for mfe : 2.7
Score for randfold : 1.6
Total read count : 31
Mature read count : 13
Loop read count : 0
Star read count : 18

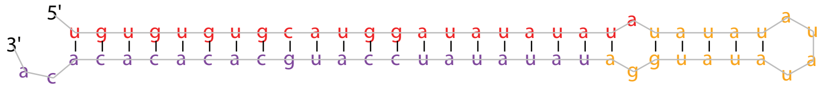

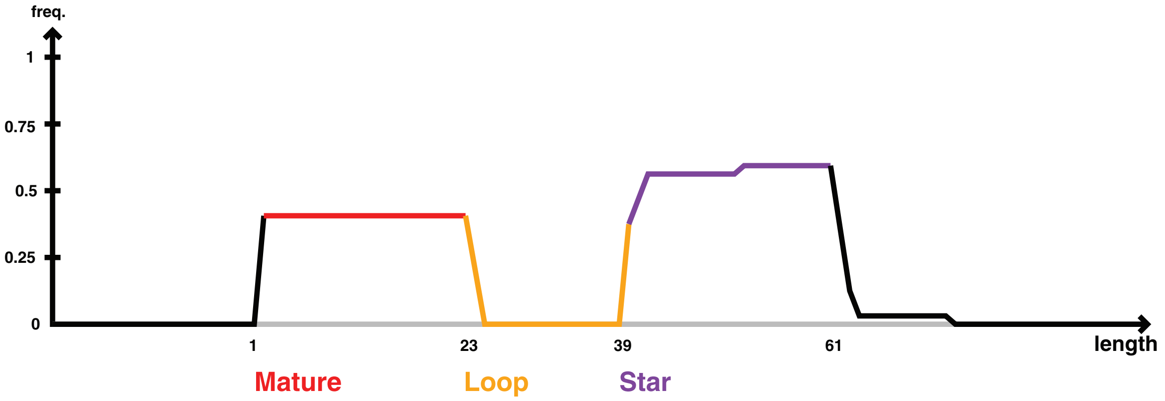

5'- ugugugugugugugugugugugugugugugcauggauauauauauauauauauauauauggauauauauccaugcacacacacacacacacacacaauaucccca uggaagcacuu -3'

h

Novel miRNA ID : NM-OKF6-S-08-5p
Score total : 104
Score for star read(s): 3.9
Score for read counts 109.2
Score for mfe : -6.9
Score for randfold : -2.2
Total read count : 226
Mature read count : 223
Loop read count : 0
Star read count : 3

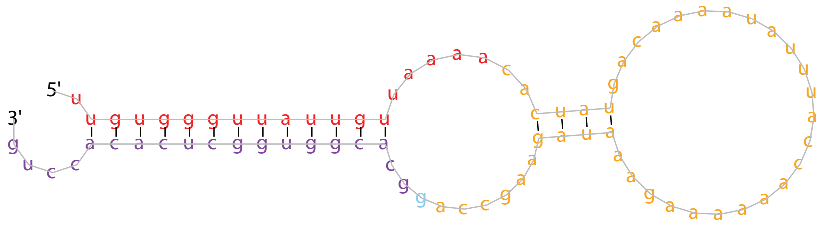

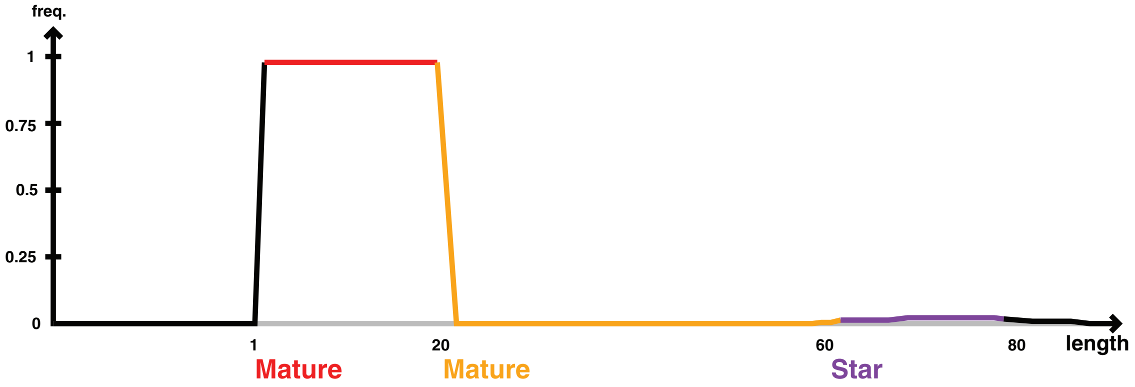

5'- uuuucugagaugaguuuacauuguggguuauuguuaaaacacuaugacaaaauauuuaccaaaaaagaaauagaagccagg cacgguggcucacaccuguaaucccagc -3'

i

Novel miRNA ID	: NM-OKF6-S-09-5p
Score total	: 2
Score for star read(s):	-1.3
Score for read counts	0
Score for mfe	: 1.7
Score for randfold	: 1.6
Total read count	: 42
Mature read count	: 29
Loop read count	: 0
Star read count	: 13

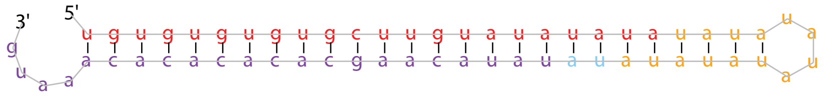

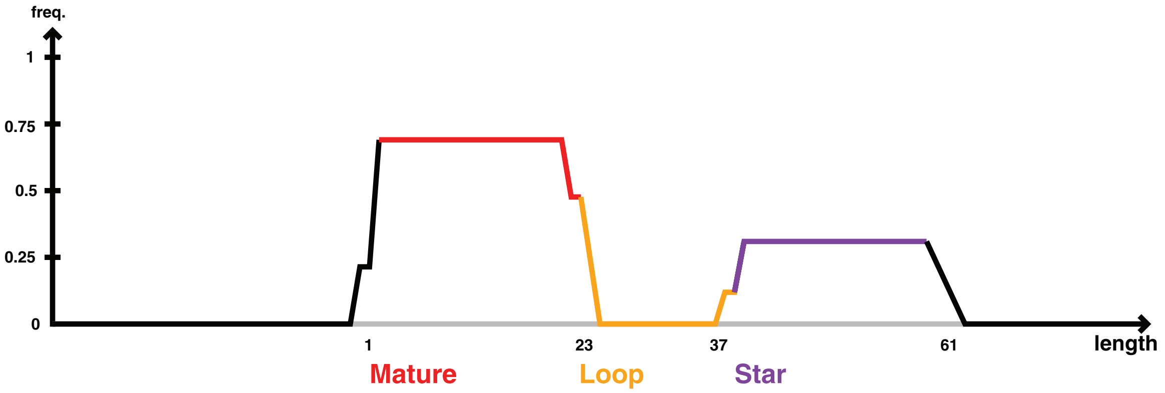

5'- cugaguauuuuagaaucccagacuacuauauaugugugugugcuuguauauauauauauauauauauauauauacaagcacacacacaaaugcaagauauauauauauauac -3'

j

Novel miRNA ID	: NM-OKF6-S-10-3p
Score total	: 1.2
Score for star read(s):	-1.3
Score for read counts	0
Score for mfe	: 0.9
Score for randfold	: 1.6
Total read count	: 633
Mature read count	: 631
Loop read count	: 0
Star read count	: 2

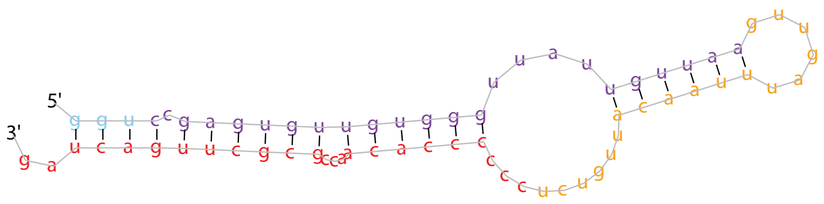

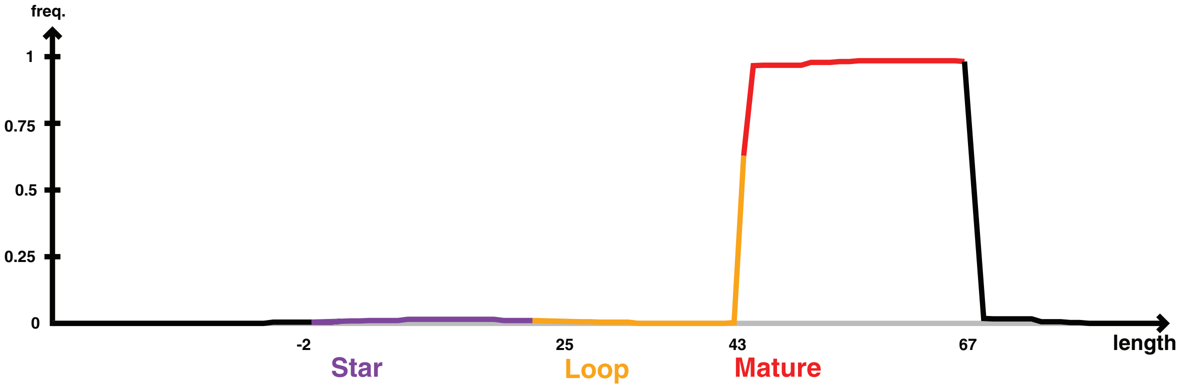

5'- aagagacucacaggauaacacaguugguccgaguguuguggguuauuguuaaguugauuuaacauugucucccccacaaccgcgcuugacuagcuugcuguuuugcacuaaua -3'

k

Novel miRNA ID : NM-OKF6-S-11-3p
Score total : 16.4
Score for star read(s): 3.9
Score for read counts 13.4
Score for mfe : 1.3
Score for randfold : -2.2
Total read count : 38
Mature read count : 35
Loop read count : 1
Star read count : 2

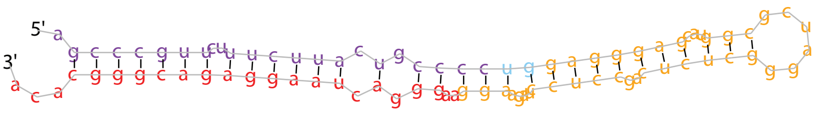

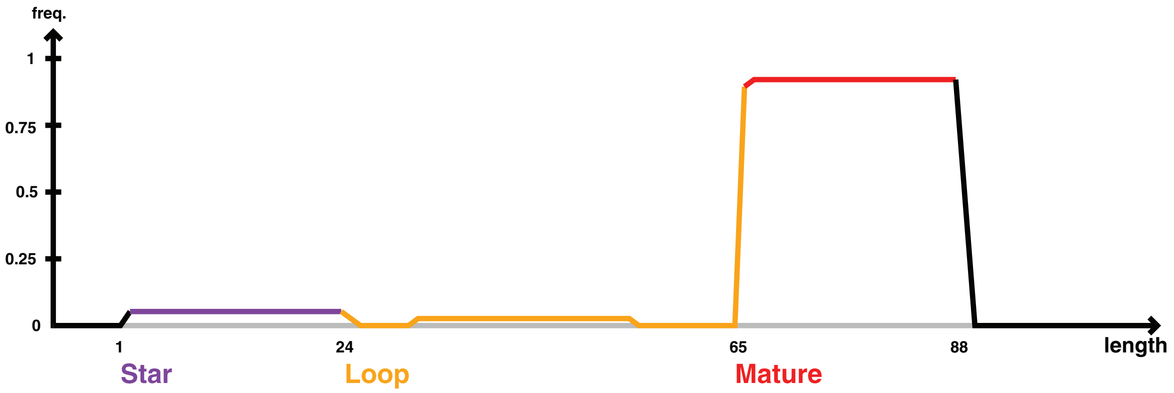

5'- aauagaagcccguucuuucuuacugccccuggagggagcauggcgcuagggcucucagccuccuagaaggaagggacuaaggagacgggcacagacuugcgucaucucguuuc -3'

l

Novel miRNA ID : NM-OKF6-S-12-5p
Score total : 9.2
Score for star read(s): 3.9
Score for read counts 1.1
Score for mfe : 2.6
Score for randfold : 1.6
Total read count : 14
Mature read count : 8
Loop read count : 0
Star read count : 6

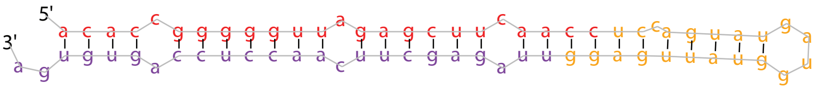

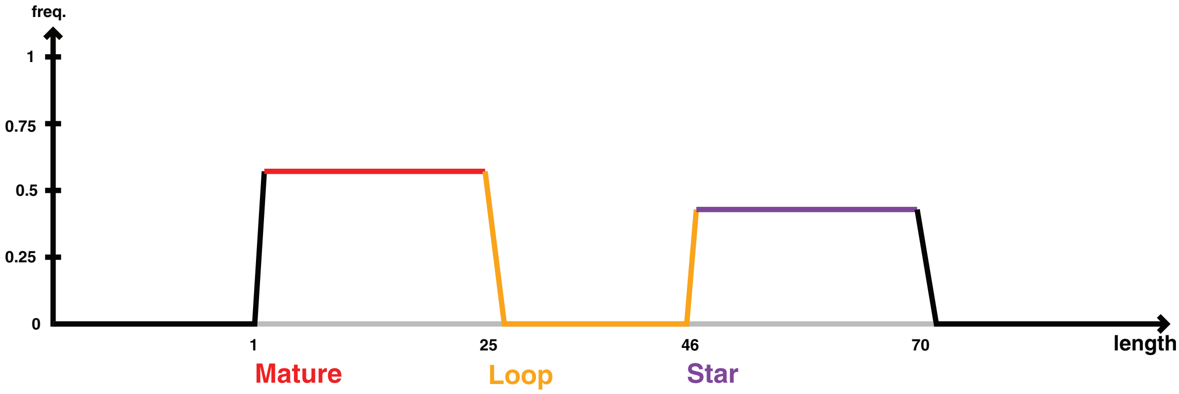

5'- guugcuccccaaauaccaucacaccggggguuagagcuucaaccuccaguaugaugguauugagguuagagcuucaaccuccagugugaugguaucgggguuagagcuucaauc -3'

m

Novel miRNA ID : NM-OKF6-S-13-3p
Score total : 19.6
Score for star read(s): 3.9
Score for read counts 12.4
Score for mfe : 1.8
Score for randfold : 1.6
Total read count : 36
Mature read count : 30
Loop read count : 0
Star read count : 6

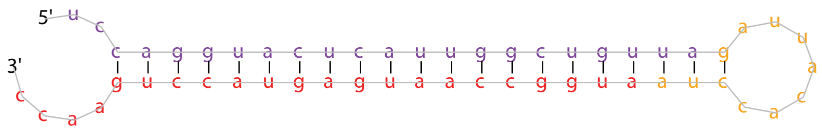

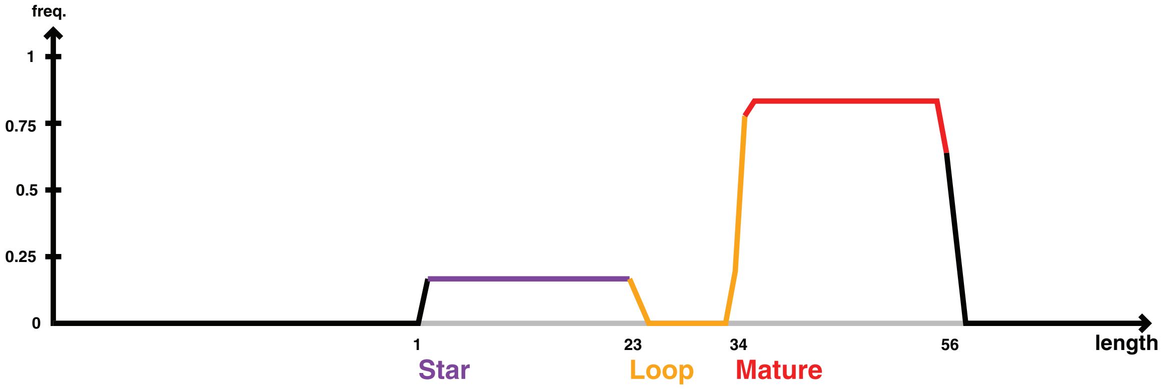

5'- auaauuuucuucagaaaauacagguuauuccaauggcuccagguacucauuggcuguuagauuacaccuaauggccaaugaguaccugaacccaaaugugaacacuaauaau -3'

n

Novel miRNA ID : NM-OKF6-S-14-5p
Score total : 321.8
Score for star read(s): 3.9
Score for read counts 338.7
Score for mfe : -18.5
Score for randfold : -2.2
Total read count : 676
Mature read count : 199
Loop read count : 476
Star read count : 1

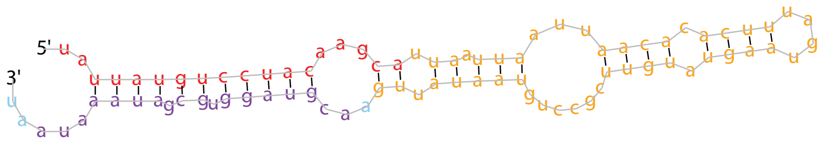

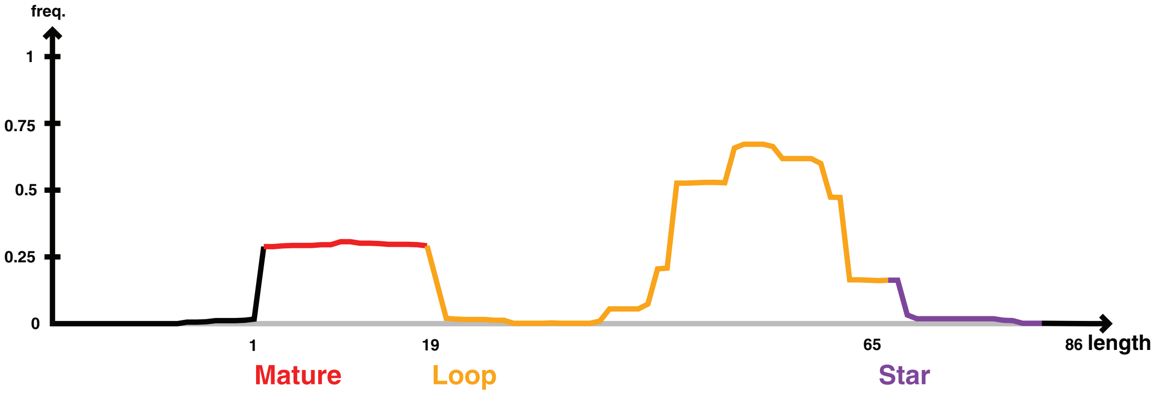

5'- ugcagacauucaauuguuauuauuauguccuacaagcauuaauuaauuaacacacuuuaguaaguauguucgccuguaauauugaacguaggugcgauaaauaauagg -3'

o

Novel miRNA ID	: NM-OKF6-S-15-3p
Score total	: 24.2
Score for star read(s):	3.9
Score for read counts	21
Score for mfe	: 1.5
Score for randfold	: -2.2
Total read count	: 53
Mature read count	: 51
Loop read count	: 0
Star read count	: 2

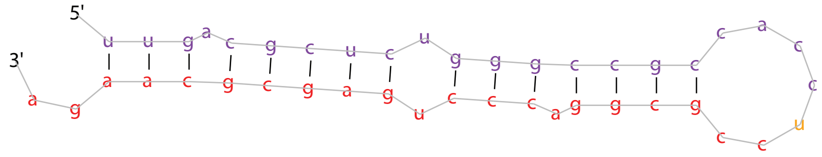

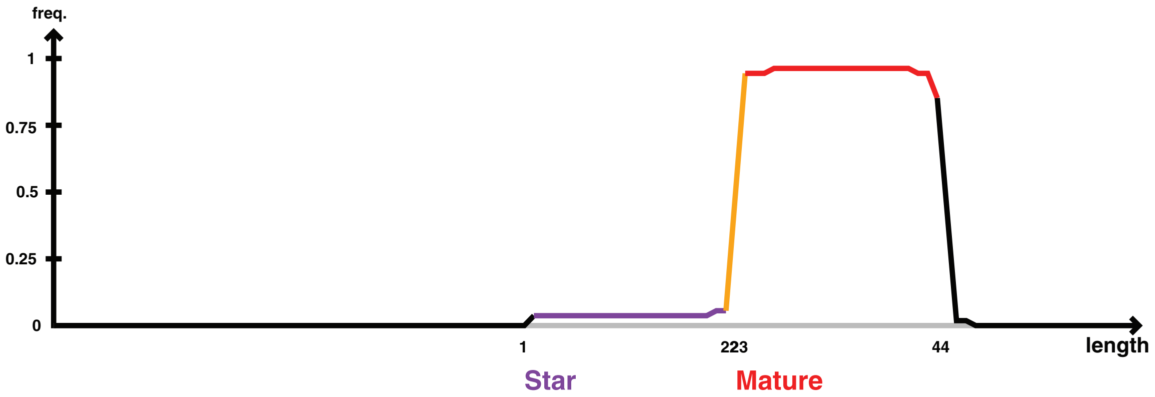

5'- cucccgccgcuccuccacgcucgugccgcucccccccccgcgcucccaguugacgcucugggccgccaccuccgcggacccugagcgcaagagccaagccgccagcgcugcg -3'

p

Novel miRNA ID	: NM-OKF6-S-16-3p
Score total	: 25.6
Score for star read(s):	3.9
Score for read counts	18.5
Score for mfe	: 1.6
Score for randfold	: 1.6
Total read count	: 48
Mature read count	: 46
Loop read count	: 0
Star read count	: 2

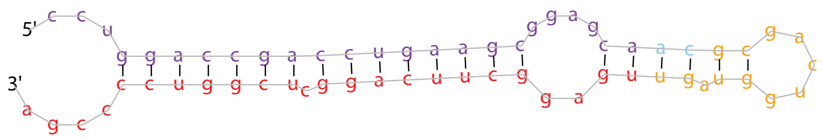

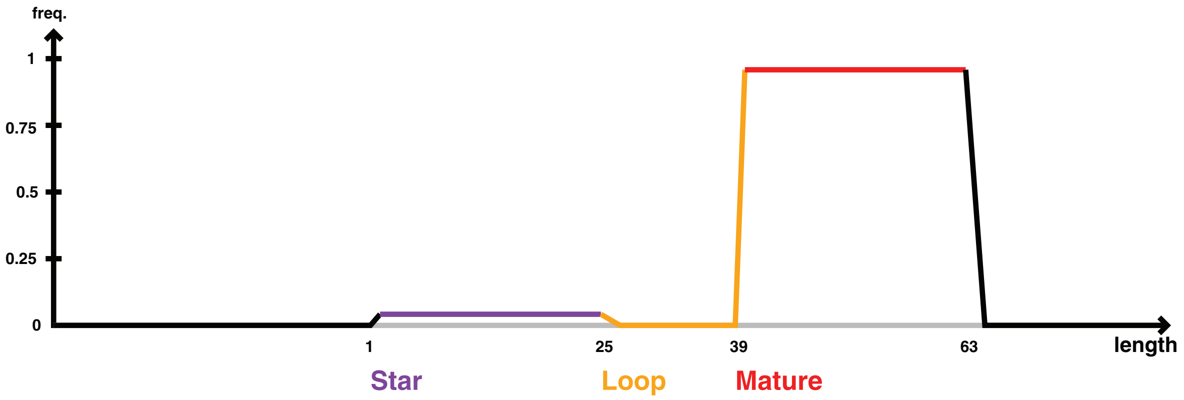

5'- agcauccuugucccuuguagcugaggcacagaccuggaccgaccugaagcggagcaacgcgacugguaguugaggcuucaggcucgguccccgauuuucuagcucuauauucuc -3'

q

Novel miRNA ID	: NM-OKF6-ST-01-3p
Score total	: 1.4
Score for star read(s):	-1.3
Score for read counts	0
Score for mfe	: 1.1
Score for randfold	: 1.6
Total read count	: 47
Mature read count	: 29
Loop read count	: 18
Star read count	: 0

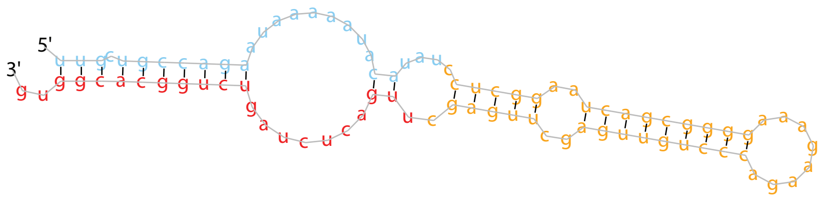

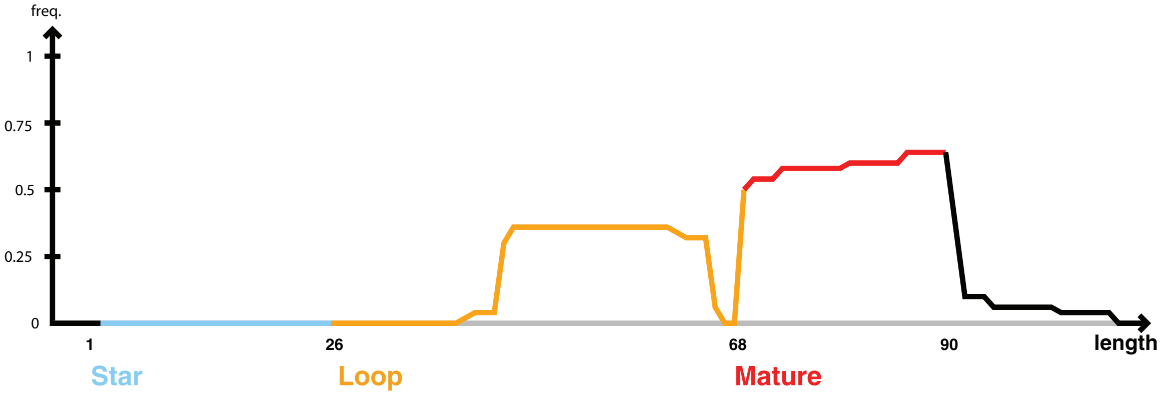

5'- acauugcugccagaauaaaaauacauauccucggaaucagcggggaaagaagacccuguugagcuugagcuugacucuagucuggcacggugaagagacgugagagguguag -3'

r

Novel miRNA ID	: NM-OKF6-ST-02-5p
Score total	: 272.1
Score for star read(s):	3.9
Score for read counts	265.2
Score for mfe	: 1.4
Score for randfold	: 1.6
Total read count	: 532
Mature read count	: 471
Loop read count	: 0
Star read count	: 61

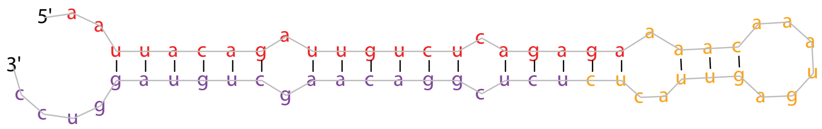

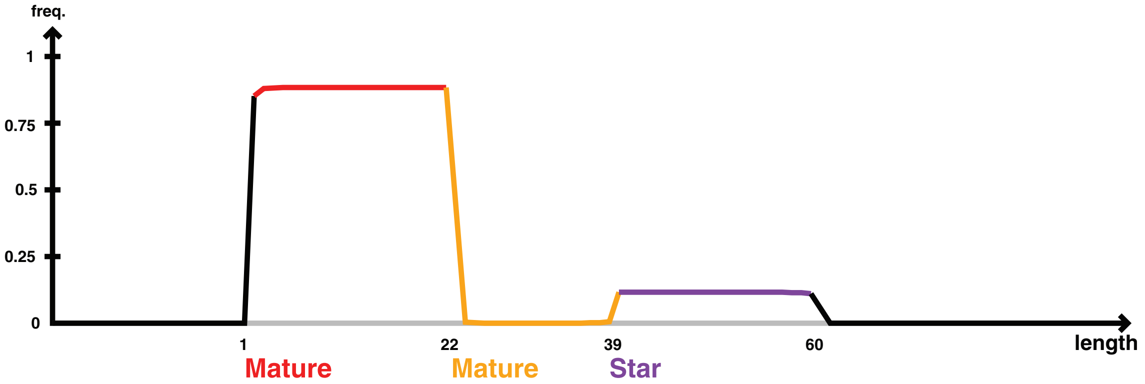

5'- aggauaauaggacaagacaaauuacagauugucucagagaaaacaaaugaguuacucucucggacaagcuguagguccuaccuaaauguccagcaggacauuagacaguc -3'

Bhat et al,2017. Cigarette smoke and chewing tobacco alter expression of different sets of miRNAs in oral keratinocytes		
**Supplementary Table S1(A). miRNA expression of OKF6/TERT1-Tobacco cells**		
**miRNA ID**	**Log2 Fold-change**	**p-value**
miR-1304-3p	-8.19	0.034273
miR-196b-5p	-7.56	0.048036
miR-615-3p	-6.84	0.068225
miR-1266-5p	-6.60	0.037814
miR-501-3p	-5.63	0.081109
miR-184	-5.63	0.056952
miR-143-3p	-5.36	0.135816
miR-675-5p	-5.18	0.075565
miR-99b-5p	-5.15	0.177155
miR-7706	-5.11	0.123868
miR-4745-5p	-4.77	0.097265
miR-196a-5p	-4.76	0.167739
miR-4787-3p	-4.60	0.109997
miR-5581-3p	-4.60	0.109997
miR-99a-5p	-4.50	0.163814
miR-548p	-4.40	0.118447
miR-598-5p	-4.40	0.118447
miR-100-5p	-4.26	0.244715
miR-501-5p	-4.18	0.127384
miR-4746-5p	-4.14	0.142947
miR-628-3p	-3.92	0.153417
miR-1273d	-3.92	0.138344
miR-570-3p	-3.92	0.138344
miR-6751-5p	-3.92	0.138344
miR-183-5p	-3.89	0.168311
miR-365b-3p	-3.83	0.260853
miR-95-3p	-3.82	0.162225
miR-1254	-3.80	0.160549
miR-203b-5p	-3.60	0.154579
miR-216b-5p	-3.60	0.154579
miR-4720-5p	-3.60	0.154579
miR-7976	-3.60	0.154579
miR-27b-5p	-3.44	0.315085
miR-148b-3p	-3.43	0.230119
miR-4798-5p	-3.18	0.181392
miR-3939	-3.18	0.215669
miR-146b-5p	-3.12	0.275922
miR-23b-5p	-3.01	0.246558
miR-320d	-3.00	0.254930
miR-148b-5p	-2.97	0.258111
miR-1303	-2.96	0.259784
miR-548n	-2.92	0.242308
miR-409-3p	-2.92	0.264342
miR-654-3p	-2.86	0.273207
miR-503-3p	-2.82	0.271864
miR-500a-3p	-2.78	0.395171
miR-30c-1-3p	-2.73	0.400871
miR-4758-3p	-2.71	0.298194
miR-1262	-2.60	0.309220
miR-3918	-2.60	0.309220
miR-4446-3p	-2.60	0.309220
miR-4743-5p	-2.60	0.309220
miR-24-1-5p	-2.60	0.283326
miR-1226-3p	-2.60	0.316723
miR-155-5p	-2.60	0.303711
miR-191-5p	-2.51	0.468659
miR-3200-3p	-2.44	0.341672
miR-1287-5p	-2.40	0.351936
miR-219b-5p	-2.33	0.346487
miR-4652-5p	-2.33	0.346487
miR-23b-3p	-2.31	0.450703
miR-4435	-2.30	0.369460
miR-1276	-2.27	0.372787
miR-942-5p	-2.25	0.381899
miR-181d-5p	-2.22	0.486605
miR-1272	-2.18	0.356800
miR-301a-5p	-2.18	0.356800
miR-3661	-2.18	0.356800
miR-15b-3p	-2.15	0.503770
miR-550a-3p	-2.14	0.414987
miR-365a-5p	-2.14	0.403687
miR-128-1-5p	-2.08	0.441418
miR-335-3p	-2.07	0.417675
miR-1180-3p	-2.05	0.517795
miR-4516	-1.92	0.443874
miR-4768-5p	-1.86	0.463167
miR-98-5p	-1.79	0.500393
miR-4286	-1.78	0.485965
miR-625-3p	-1.67	0.505875
miR-1246	-1.66	0.534999
miR-629-5p	-1.64	0.513753
miR-1284	-1.60	0.870614
miR-2277-3p	-1.60	0.518513
miR-411-3p	-1.60	0.870614
miR-4521	-1.60	0.870614
miR-4664-3p	-1.60	0.870614
miR-4717-3p	-1.60	0.518513
miR-539-3p	-1.60	0.870614
miR-548aa	-1.60	0.518513
miR-584-3p	-1.60	0.518513
miR-7845-5p	-1.60	0.518513
miR-7975	-1.60	0.870614
miR-548u	-1.60	0.528980
miR-3614-5p	-1.60	0.532116
miR-4687-5p	-1.60	0.530197
miR-4714-3p	-1.60	0.530197
miR-5000-3p	-1.60	0.532690
miR-27b-3p	-1.59	0.591426
miR-361-5p	-1.57	0.610828
miR-342-3p	-1.51	0.629263
miR-320b	-1.51	0.618532
miR-4485-3p	-1.50	0.545679

Bhat et al,2017. Cigarette smoke and chewing tobacco alter expression of different sets of miRNAs in oral keratinocytes		
**Supplementary Table S1(A). miRNA expression of OKF6/TERT1-Tobacco cells**		
**miRNA ID**	**Log2 Fold-change**	**p-value**
miR-7704	-1.47	0.560538
miR-421	-1.46	0.627878
miR-1294	-1.46	0.566225
miR-185-3p	-1.43	0.577466
miR-542-3p	-1.40	0.569596
miR-3935	-1.37	0.585878
miR-3177-3p	-1.37	0.595836
miR-183-3p	-1.36	0.580887
miR-30e-3p	-1.33	0.649346
miR-3605-5p	-1.30	0.612047
miR-181b-5p	-1.28	0.612995
miR-3130-5p	-1.27	0.634106
miR-340-3p	-1.27	0.634106
miR-6515-5p	-1.27	0.634106
miR-3677-3p	-1.25	0.621096
miR-26a-1-3p	-1.21	0.619329
miR-125b-1-3p	-1.18	0.689191
miR-3691-5p	-1.18	0.678277
miR-769-3p	-1.14	0.659365
miR-28-3p	-1.12	0.651186
miR-10b-5p	-1.11	0.712122
miR-550a-5p	-1.08	0.654034
miR-193b-3p	-1.07	0.691922
let-7d-3p	-1.04	0.721070
miR-3065-5p	-1.01	0.772487
miR-548i	-1.01	0.772487
miR-889-3p	-1.01	0.772487
miR-500a-5p	-1.01	0.696265
miR-6720-3p	-1.01	0.724269
miR-6837-3p	-1.01	0.724269
miR-30d-5p	-0.98	0.710233
miR-937-3p	-0.97	0.693718
miR-940	-0.97	0.699768
let-7i-5p	-0.96	0.689244
miR-1286	-0.94	0.720772
miR-450b-5p	-0.94	0.706281
miR-4742-3p	-0.92	0.743972
miR-877-5p	-0.92	0.699212
miR-324-5p	-0.88	0.744312
miR-24-3p	-0.87	0.713227
miR-192-3p	-0.86	0.802012
miR-106b-3p	-0.85	0.766888
miR-598-3p	-0.83	0.753642
miR-92b-3p	-0.82	0.724749
miR-152-5p	-0.78	0.744505
miR-222-5p	-0.75	0.749903
miR-3940-3p	-0.75	0.800085
miR-6775-3p	-0.75	0.800085
miR-766-3p	-0.75	0.747061
miR-941	-0.74	0.750575
miR-1301-3p	-0.71	0.757887
miR-25-3p	-0.71	0.755209
miR-542-5p	-0.70	0.795549
miR-548d-5p	-0.70	0.795549
miR-181c-5p	-0.70	0.792963
miR-16-2-3p	-0.69	0.764026
miR-96-5p	-0.66	0.804072
miR-362-5p	-0.65	0.793722
miR-181a-5p	-0.65	0.797746
miR-628-5p	-0.65	0.785901
miR-135b-3p	-0.60	1.000000
miR-548l	-0.60	1.000000
miR-652-5p	-0.60	1.000000
miR-3615	-0.60	0.850633
miR-219a-1-3p	-0.60	0.870135
miR-324-3p	-0.60	0.870135
miR-6735-5p	-0.60	0.966077
miR-629-3p	-0.60	0.903581
miR-149-5p	-0.59	0.820147
miR-330-5p	-0.58	0.797006
miR-30c-2-3p	-0.57	0.820099
miR-652-3p	-0.57	0.798890
miR-576-5p	-0.57	0.800027
miR-30c-5p	-0.57	0.823389
miR-1910-5p	-0.54	0.827513
miR-151a-3p	-0.53	0.807585
miR-3909	-0.52	0.815156
miR-186-5p	-0.49	0.817472
miR-548k	-0.46	0.843060
miR-99b-3p	-0.46	0.843174
miR-345-5p	-0.45	0.844348
miR-3184-5p	-0.39	0.863923
miR-3074-5p	-0.35	0.863158
miR-125b-5p	-0.34	0.857570
miR-484	-0.28	0.889469
miR-3157-5p	-0.27	1.000000
miR-4638-3p	-0.27	1.000000
miR-641	-0.25	0.889866
miR-4775	-0.20	0.905858
miR-1910-3p	-0.18	1.000000
miR-151b	-0.17	0.923725
miR-450a-5p	-0.14	0.928880
miR-320a	-0.14	0.918981
miR-374b-5p	-0.12	0.927357
miR-181a-2-3p	-0.10	0.925483
miR-3065-3p	-0.09	1.000000
miR-148a-5p	-0.07	0.938091
miR-7705	-0.06	0.979519
miR-7-1-3p	-0.06	0.946299
miR-28-5p	-0.03	0.945757
miR-1322	-0.01	1.000000
miR-190b	-0.01	1.000000

Bhat et al,2017. Cigarette smoke and chewing tobacco alter expression of different sets of miRNAs in oral keratinocytes		
**Supplementary Table S1(A). miRNA expression of OKF6/TERT1-Tobacco cells**		
**miRNA ID**	**Log2 Fold-change**	**p-value**
miR-3928-3p	-0.01	1.000000
miR-548am-5p	-0.01	1.000000
miR-548c-5p	-0.01	1.000000
miR-5690	-0.01	1.000000
let-7a-2-3p	-0.01	1.000000
miR-769-5p	0.00	0.953227
miR-425-3p	0.01	1.000000
miR-671-3p	0.01	1.000000
miR-15b-5p	0.04	1.000000
miR-1908-5p	0.05	1.000000
miR-30a-3p	0.07	1.000000
miR-200a-3p	0.08	1.000000
miR-103a-2-5p	0.08	1.000000
miR-454-5p	0.10	1.000000
miR-330-3p	0.13	1.000000
miR-891a-5p	0.14	1.000000
miR-584-5p	0.17	1.000000
miR-1268a	0.19	1.000000
miR-503-5p	0.21	1.000000
miR-212-5p	0.22	0.995323
miR-203a-3p	0.23	0.994123
miR-151a-5p	0.24	0.975706
miR-25-5p	0.25	1.000000
miR-195-5p	0.30	0.972613
miR-197-3p	0.33	0.970450
miR-582-3p	0.35	0.961027
miR-30a-5p	0.35	0.945138
miR-221-5p	0.36	0.960457
miR-125a-3p	0.40	0.953881
miR-1307-3p	0.40	0.952030
miR-195-3p	0.40	1.000000
miR-146b-3p	0.40	1.000000
miR-331-5p	0.40	0.935666
miR-192-5p	0.42	0.924434
miR-125a-5p	0.42	0.924632
miR-589-5p	0.42	0.948638
miR-23a-3p	0.43	0.921546
miR-147b	0.43	0.926353
miR-328-3p	0.45	0.924214
miR-93-5p	0.47	0.914627
miR-6510-3p	0.50	0.927334
miR-127-3p	0.51	0.907459
let-7d-5p	0.52	0.906068
miR-200c-3p	0.54	0.887730
miR-3158-3p	0.57	0.892895
miR-125b-2-3p	0.57	0.929549
miR-181c-3p	0.63	0.885706
miR-4677-3p	0.65	0.863074
miR-375	0.70	0.845920
miR-92a-3p	0.71	0.851015
miR-411-5p	0.73	0.859310
miR-449c-5p	0.73	0.866633
miR-26a-2-3p	0.74	0.832441
miR-30d-3p	0.75	0.857689
miR-18a-3p	0.78	0.822191
miR-7974	0.83	0.823562
miR-21-3p	0.85	0.797183
miR-191-3p	0.87	0.792156
miR-374a-5p	0.90	0.819995
miR-140-3p	0.91	0.800497
miR-181a-3p	0.91	0.816010
miR-339-5p	0.93	0.819588
miR-2467-5p	0.94	0.796291
miR-331-3p	0.94	0.813413
miR-423-5p	0.94	0.795438
miR-185-5p	0.96	0.771035
miR-361-3p	0.97	0.805202
let-7b-3p	0.98	0.804007
miR-128-3p	0.98	0.815814
miR-1343-3p	0.99	0.921903
miR-6511b-3p	0.99	0.827887
miR-100-3p	0.99	0.813853
miR-200b-3p	1.01	0.804485
miR-16-5p	1.01	0.759290
miR-193b-5p	1.04	0.745519
miR-26a-5p	1.04	0.760988
miR-454-3p	1.05	0.781667
miR-135b-5p	1.07	0.745162
miR-130a-3p	1.08	0.747634
miR-423-3p	1.10	0.749496
miR-30b-3p	1.10	0.722255
let-7e-3p	1.12	0.726349
miR-181b-2-3p	1.14	0.752630
miR-548o-3p	1.17	0.735519
let-7b-5p	1.18	0.699336
miR-301a-3p	1.20	0.760163
miR-26b-3p	1.21	0.728574
miR-4640-3p	1.21	0.815933
miR-4640-5p	1.21	0.815933
miR-548h-3p	1.21	0.815933
miR-5683	1.21	0.815933
miR-93-3p	1.21	0.704750
miR-582-5p	1.22	0.712787
miR-27a-5p	1.26	0.743535
miR-152-3p	1.27	0.680988
miR-381-3p	1.31	0.718668
miR-10a-5p	1.32	0.663814
miR-24-2-5p	1.34	0.655078
miR-425-5p	1.36	0.710003
miR-576-3p	1.40	0.738107
miR-625-5p	1.40	0.660796
let-7g-5p	1.42	0.633302

Bhat et al,2017. Cigarette smoke and chewing tobacco alter expression of different sets of miRNAs in oral keratinocytes		
**Supplementary Table S1(A). miRNA expression of OKF6/TERT1-Tobacco cells**		
**miRNA ID**	**Log2 Fold-change**	**p-value**
miR-107	1.44	0.685515
miR-642a-5p	1.47	0.632292
miR-30b-5p	1.50	0.645728
let-7i-3p	1.53	0.623028
miR-744-3p	1.54	0.612986
miR-589-3p	1.56	0.628701
miR-130b-3p	1.56	0.682878
miR-1296-5p	1.56	0.600592
miR-2116-3p	1.57	0.678114
miR-1285-3p	1.63	0.581691
miR-26b-5p	1.67	0.656430
let-7a-3p	1.67	0.596112
miR-874-3p	1.67	0.599259
miR-561-5p	1.68	0.572163
miR-30e-5p	1.69	0.619097
let-7f-1-3p	1.70	0.569542
miR-1255a	1.73	0.596246
miR-548e-5p	1.73	0.596246
miR-15a-5p	1.74	0.632151
miR-200c-5p	1.78	0.555574
miR-194-5p	1.79	0.633326
miR-148a-3p	1.79	0.538040
miR-1307-5p	1.80	0.615422
miR-320c	1.83	0.541494
miR-6842-3p	1.84	0.531868
miR-616-5p	1.86	0.590502
miR-34c-3p	1.88	0.535674
miR-449a	1.90	0.524335
miR-1248	1.93	0.543237
let-7e-5p	1.93	0.499828
miR-1260b	1.94	0.502635
miR-92a-1-5p	1.96	0.517515
miR-502-3p	1.98	0.494427
miR-410-3p	1.99	0.557208
miR-3929	2.03	0.490491
miR-21-5p	2.03	0.540837
miR-20a-3p	2.10	0.500149
miR-6859-5p	2.10	0.528785
miR-374a-3p	2.13	0.541390
miR-205-5p	2.14	0.511525
miR-455-3p	2.17	0.503710
miR-33b-3p	2.17	0.441911
miR-2355-3p	2.21	0.475372
miR-34b-3p	2.21	0.475372
miR-141-3p	2.26	0.430402
miR-19b-1-5p	2.31	0.453054
miR-374b-3p	2.31	0.412802
miR-2277-5p	2.33	0.418257
miR-873-3p	2.37	0.400899
miR-188-3p	2.40	0.463182
miR-32-3p	2.44	0.385309
miR-103a-3p	2.47	0.379857
miR-34a-3p	2.49	0.380784
miR-659-5p	2.53	0.367396
miR-27a-3p	2.60	0.398375
miR-429	2.63	0.456570
miR-944	2.65	0.402691
miR-200a-5p	2.68	0.345084
miR-301b-3p	2.70	0.435551
miR-1271-5p	2.73	0.347996
miR-129-5p	2.80	0.426628
miR-590-3p	2.85	0.311342
miR-339-3p	2.89	0.415598
miR-132-3p	2.98	0.399047
miR-340-5p	2.99	0.397826
miR-660-5p	3.01	0.379331
miR-574-3p	3.08	0.344864
miR-221-3p	3.10	0.276000
miR-424-3p	3.21	0.319864
miR-505-3p	3.29	0.251536
miR-1275	3.33	0.271847
miR-6087	3.39	0.272322
let-7g-3p	3.40	0.291363
miR-23a-5p	3.42	0.298627
miR-200b-5p	3.42	0.233851
miR-212-3p	3.47	0.227583
miR-22-3p	3.53	0.346724
miR-574-5p	3.54	0.315264
miR-19a-3p	3.59	0.301389
miR-497-5p	3.61	0.212791
miR-19b-3p	3.69	0.254128
miR-874-5p	3.76	0.192965
miR-126-5p	3.76	0.211068
miR-141-5p	3.83	0.224687
miR-29a-3p	3.88	0.167453
miR-363-3p	3.92	0.195039
miR-130b-5p	4.00	0.176206
miR-33a-3p	4.00	0.176206
miR-22-5p	4.03	0.232982
miR-210-3p	4.05	0.165641
miR-29b-1-5p	4.08	0.168810
miR-17-3p	4.19	0.230836
miR-222-3p	4.21	0.185097
miR-378a-3p	4.26	0.134527
miR-101-3p	4.28	0.228405
miR-3605-3p	4.29	0.147891
miR-378a-5p	4.49	0.144579
miR-210-5p	4.53	0.202678
miR-4645-3p	4.55	0.153255
miR-935	4.60	0.175068
miR-2355-5p	4.61	0.130234
miR-873-5p	4.79	0.121914

Bhat et al,2017. Cigarette smoke and chewing tobacco alter expression of different sets of miRNAs in oral keratinocytes		
**Supplementary Table S1(A). miRNA expression of OKF6/TERT1-Tobacco cells**		
**miRNA ID**	**Log2 Fold-change**	**p-value**
miR-424-5p	4.89	0.173563
miR-126-3p	5.16	0.153526
miR-34a-5p	5.21	0.102884
miR-20a-5p	5.32	0.084988
miR-29b-3p	5.45	0.130243
miR-132-5p	5.49	0.089554
miR-455-5p	5.61	0.115020
miR-32-5p	6.28	0.073599
miR-9-5p	6.33	0.079637
miR-199b-5p	6.38	0.062483
miR-205-3p	6.56	0.059538
miR-146a-5p	6.58	0.042983
miR-31-3p	7.46	0.055035
miR-193a-5p	8.12	0.042290
miR-29c-3p	8.37	0.036712
miR-31-5p	8.39	0.009173
miR-33b-5p	8.45	0.040271
miR-4662a-5p	8.85	0.035949
miR-4454	9.66	0.004575

Bhat et al,2017. Cigarette smoke and chewing tobacco alter expression of different sets of miRNAs in oral keratinocytes		
**Supplementary Table S1(B) . miRNA expression of OKF6/TERT1-Smoke cells**		
**miRNA ID**	**Log2 Fold-change**	**p-value**
miR-200a-3p	-10.82	0.028930
miR-200b-3p	-9.64	0.040834
miR-200c-3p	-9.60	0.015514
miR-205-5p	-9.16	0.018067
miR-429	-8.11	0.060195
miR-141-3p	-7.65	0.054884
miR-501-3p	-7.63	0.071376
miR-151b	-6.99	0.086732
miR-183-5p	-6.97	0.071767
miR-99b-5p	-6.77	0.171195
miR-129-1-3p	-6.74	0.108741
miR-548p	-6.54	0.118262
miR-365b-3p	-6.54	0.180150
miR-1260a	-6.32	0.130167
miR-6871-3p	-6.32	0.130167
miR-4485-3p	-6.27	0.119311
miR-203a-3p	-6.20	0.205832
miR-216b-5p	-5.74	0.166215
miR-141-5p	-5.54	0.249379
miR-200c-5p	-5.40	0.172471
miR-409-5p	-5.32	0.183260
miR-600	-5.32	0.196789
miR-675-5p	-5.32	0.180646
miR-320d	-5.14	0.203806
miR-6751-5p	-5.06	0.204016
miR-1248	-4.74	0.235456
miR-1291	-4.74	0.235456
miR-4658	-4.74	0.235456
miR-4743-5p	-4.74	0.235456
miR-7845-5p	-4.74	0.235456
miR-7976	-4.74	0.232033
miR-181d-5p	-4.74	0.314122
miR-143-3p	-4.51	0.352995
miR-6892-5p	-4.32	0.264261
miR-184	-4.18	0.301708
miR-96-5p	-4.10	0.388667
miR-320b	-4.05	0.383697
miR-6840-5p	-4.00	0.295368
miR-24-3p	-3.84	0.316946
miR-100-5p	-3.80	0.416217
miR-190b	-3.74	0.269250
miR-4446-3p	-3.74	0.338344
miR-4738-3p	-3.74	0.269250
miR-3144-5p	-3.74	0.337089
miR-3146	-3.74	0.337089
miR-7704	-3.56	0.354302
miR-181a-5p	-3.55	0.367201
miR-181c-5p	-3.53	0.423177
miR-501-5p	-3.52	0.345051
miR-584-5p	-3.48	0.434903
miR-7706	-3.47	0.394638
miR-148b-3p	-3.44	0.351767
miR-129-5p	-3.43	0.377292
miR-146a-5p	-3.39	0.374476
miR-598-3p	-3.38	0.417613
miR-23b-3p	-3.16	0.419797
miR-1266-5p	-3.15	0.377804
miR-147b	-3.15	0.373365
miR-3685	-3.15	0.396205
miR-4479	-3.15	0.396205
miR-4642	-3.15	0.396205
miR-4720-5p	-3.15	0.396205
miR-6515-5p	-3.15	0.396205
miR-3138	-3.15	0.422621
miR-451a	-3.15	0.422621
miR-323a-5p	-3.00	0.430220
miR-4741	-3.00	0.399730
miR-3074-5p	-2.99	0.411152
miR-4758-3p	-2.91	0.393596
miR-183-3p	-2.87	0.398993
miR-1246	-2.86	0.429575
miR-193b-3p	-2.79	0.441646
miR-361-5p	-2.76	0.467478
miR-4519	-2.74	0.389960
miR-766-5p	-2.74	0.389960
miR-6847-5p	-2.74	0.466238
miR-500a-3p	-2.71	0.480895
miR-23a-3p	-2.68	0.415323
miR-3605-5p	-2.65	0.436547
miR-34c-3p	-2.63	0.417499
miR-191-5p	-2.62	0.482834
miR-221-5p	-2.60	0.464476
miR-5581-3p	-2.57	0.437232
miR-365a-5p	-2.57	0.423941
miR-4745-5p	-2.55	0.425079
miR-3936	-2.52	0.497571
miR-181b-5p	-2.51	0.421609
miR-338-5p	-2.50	0.435953

Bhat et al,2017. Cigarette smoke and chewing tobacco alter expression of different sets of miRNAs in oral keratinocytes		
**Supplementary Table S1(B) . miRNA expression of OKF6/TERT1-Smoke cells**		
**miRNA ID**	**Log2 Fold-change**	**p-value**
miR-4515	-2.42	0.487815
miR-1273d	-2.36	0.480413
miR-937-3p	-2.35	0.447049
miR-338-3p	-2.35	0.460687
miR-342-3p	-2.32	0.502571
miR-4787-3p	-2.28	0.467953
miR-548n	-2.25	0.493477
miR-135b-5p	-2.24	0.453770
miR-330-5p	-2.21	0.458817
miR-30c-1-3p	-2.19	0.507549
miR-1255a	-2.15	0.597095
miR-586	-2.15	0.597095
miR-1910-3p	-2.15	0.550403
miR-6800-3p	-2.15	0.523024
miR-181c-3p	-2.10	0.484806
miR-30d-5p	-2.10	0.482005
miR-24-1-5p	-2.04	0.538266
miR-6720-3p	-2.04	0.538266
miR-4523	-2.00	0.573293
miR-570-3p	-1.97	0.529044
miR-642a-5p	-1.89	0.539965
miR-24-2-5p	-1.86	0.510634
miR-3163	-1.82	0.515859
miR-103a-2-5p	-1.81	0.550516
miR-219b-5p	-1.81	0.550516
miR-6775-3p	-1.81	0.550516
miR-500a-5p	-1.78	0.525667
miR-5094	-1.74	0.538409
miR-1284	-1.74	0.690618
miR-584-3p	-1.74	0.674933
miR-27b-3p	-1.72	0.514546
miR-23b-5p	-1.69	0.537221
miR-5001-5p	-1.62	0.632748
miR-628-3p	-1.60	0.539464
miR-34b-3p	-1.57	0.707200
miR-125a-5p	-1.53	0.509353
miR-181a-2-3p	-1.53	0.526014
miR-421	-1.52	0.549563
miR-3929	-1.52	0.538333
miR-1910-5p	-1.50	0.549628
miR-1226-3p	-1.50	0.537057
miR-4742-3p	-1.47	0.598977
miR-4775	-1.46	0.537887
miR-3935	-1.42	0.557937
miR-1322	-1.42	0.819588
miR-450a-2-3p	-1.42	0.819588
miR-330-3p	-1.41	0.544438
miR-148b-5p	-1.37	0.561620
miR-1276	-1.31	0.590871
miR-30c-5p	-1.31	0.565093
miR-362-5p	-1.20	0.576567
miR-27a-5p	-1.18	0.581565
miR-30a-5p	-1.18	0.562487
miR-27a-3p	-1.17	0.557501
miR-615-3p	-1.15	0.581373
miR-135b-3p	-1.15	0.916199
miR-199b-5p	-1.15	0.916199
miR-4482-3p	-1.15	0.916199
miR-6735-5p	-1.15	0.787103
miR-30e-3p	-1.09	0.583638
miR-503-3p	-1.02	0.645997
miR-4768-5p	-1.00	0.660896
miR-98-5p	-1.00	0.585882
miR-212-5p	-0.99	0.612699
miR-1294	-0.97	0.634391
miR-3130-5p	-0.93	0.713799
miR-6837-3p	-0.93	0.713799
miR-6782-3p	-0.93	0.989062
miR-320c	-0.88	0.723259
miR-3691-5p	-0.86	0.766917
miR-652-3p	-0.84	0.629199
miR-193b-5p	-0.83	0.659515
miR-34a-3p	-0.76	0.678429
miR-3614-5p	-0.74	0.725824
miR-1262	-0.74	0.869524
miR-550a-3p	-0.69	0.647057
miR-125a-3p	-0.67	0.643346
miR-1254	-0.64	0.668182
let-7i-5p	-0.64	0.648926
miR-374b-5p	-0.62	0.654056
miR-196b-5p	-0.60	0.651220
miR-6842-3p	-0.60	0.753205
miR-34a-5p	-0.60	0.656506
miR-4286	-0.58	0.667939
miR-192-3p	-0.57	0.826006
miR-3140-3p	-0.57	1.000000
miR-4762-5p	-0.57	1.000000
miR-548am-5p	-0.57	1.000000

Bhat et al,2017. Cigarette smoke and chewing tobacco alter expression of different sets of miRNAs in oral keratinocytes		
**Supplementary Table S1(B) . miRNA expression of OKF6/TERT1-Smoke cells**		
**miRNA ID**	**Log2 Fold-change**	**p-value**
miR-340-3p	-0.53	0.793063
miR-4714-3p	-0.53	0.793063
miR-576-5p	-0.50	0.692001
miR-3918	-0.49	0.920620
miR-1301-3p	-0.48	0.688649
miR-3174	-0.46	0.847623
miR-363-3p	-0.44	0.770021
miR-26a-5p	-0.43	0.689055
miR-106b-3p	-0.43	0.677702
miR-548aa	-0.42	0.936215
miR-149-5p	-0.41	0.682745
miR-27b-5p	-0.38	0.687667
miR-30c-2-3p	-0.38	0.687897
miR-324-5p	-0.37	0.690046
miR-3157-5p	-0.35	0.951208
miR-582-3p	-0.34	0.698722
miR-186-5p	-0.31	0.714303
miR-361-3p	-0.31	0.706614
miR-425-3p	-0.31	0.733374
miR-769-3p	-0.29	0.781132
miR-455-3p	-0.29	0.731216
miR-28-3p	-0.28	0.723232
miR-891a-5p	-0.28	0.888035
miR-6768-5p	-0.28	1.000000
miR-30a-3p	-0.23	0.722729
miR-2277-3p	-0.21	0.979576
miR-2355-3p	-0.21	0.979576
miR-5699-3p	-0.21	0.979576
miR-877-5p	-0.21	0.752174
let-7e-3p	-0.21	0.754769
miR-320a	-0.19	0.728146
miR-4746-5p	-0.17	0.736754
miR-4435	-0.16	0.757480
miR-181a-3p	-0.14	0.734541
miR-629-5p	-0.10	0.767300
miR-26b-3p	-0.06	0.765857
miR-26b-5p	-0.06	0.740651
miR-99b-3p	-0.04	0.750949
miR-4717-3p	-0.04	1.000000
miR-449c-5p	-0.02	0.883341
miR-449a	0.04	1.000000
miR-3177-3p	0.12	1.000000
miR-185-3p	0.15	1.000000
miR-425-5p	0.16	1.000000
miR-3928-3p	0.17	1.000000
miR-548h-3p	0.17	1.000000
miR-766-3p	0.17	1.000000
miR-125b-5p	0.18	1.000000
miR-542-3p	0.19	1.000000
miR-4687-5p	0.19	1.000000
miR-582-5p	0.21	1.000000
miR-148a-5p	0.22	1.000000
miR-375	0.24	1.000000
miR-28-5p	0.25	1.000000
miR-3909	0.27	1.000000
miR-15b-3p	0.29	1.000000
miR-548u	0.29	1.000000
miR-3615	0.30	1.000000
miR-7-1-3p	0.31	1.000000
miR-574-3p	0.32	1.000000
miR-30e-5p	0.32	1.000000
let-7b-3p	0.33	1.000000
miR-25-3p	0.34	1.000000
miR-181b-2-3p	0.35	1.000000
miR-92a-3p	0.37	1.000000
miR-331-5p	0.38	1.000000
let-7e-5p	0.38	1.000000
miR-151a-5p	0.39	1.000000
miR-671-3p	0.39	1.000000
miR-942-5p	0.40	1.000000
miR-99a-5p	0.43	1.000000
miR-26a-2-3p	0.44	1.000000
miR-4512	0.46	1.000000
miR-151a-3p	0.48	1.000000
miR-196a-5p	0.49	1.000000
miR-7705	0.50	1.000000
miR-374a-5p	0.50	1.000000
miR-941	0.51	1.000000
miR-1287-5p	0.55	1.000000
miR-192-5p	0.58	1.000000
let-7d-3p	0.58	1.000000
miR-93-5p	0.62	1.000000
miR-4638-3p	0.65	1.000000
miR-222-5p	0.66	1.000000
miR-26a-1-3p	0.68	1.000000
miR-548d-5p	0.71	1.000000
miR-589-5p	0.71	1.000000
miR-542-5p	0.72	1.000000

Bhat et al,2017. Cigarette smoke and chewing tobacco alter expression of different sets of miRNAs in oral keratinocytes		
**Supplementary Table S1(B) . miRNA expression of OKF6/TERT1-Smoke cells**		
**miRNA ID**	**Log2 Fold-change**	**p-value**
miR-548k	0.73	1.000000
let-7b-5p	0.75	1.000000
miR-550a-5p	0.76	1.000000
miR-125b-1-3p	0.77	1.000000
miR-152-5p	0.80	1.000000
miR-148a-3p	0.82	0.983195
miR-3939	0.84	1.000000
miR-21-5p	0.84	1.000000
miR-128-1-5p	0.84	1.000000
miR-769-5p	0.89	1.000000
miR-21-3p	0.89	0.964369
miR-345-5p	0.90	0.998390
miR-20a-3p	0.91	1.000000
miR-3661	0.91	1.000000
miR-221-3p	0.92	0.953988
miR-152-3p	0.93	0.993117
miR-23a-5p	0.95	0.984227
miR-130a-3p	0.96	0.966373
miR-2116-3p	0.96	1.000000
miR-195-5p	0.97	1.000000
miR-874-3p	0.98	0.991203
miR-641	0.99	1.000000
miR-197-3p	1.00	1.000000
miR-4521	1.02	1.000000
miR-194-5p	1.03	1.000000
miR-210-3p	1.04	1.000000
miR-4677-3p	1.06	0.968734
miR-331-3p	1.06	1.000000
miR-107	1.07	0.980793
miR-99a-3p	1.07	0.956708
miR-92b-3p	1.07	0.915987
miR-940	1.09	0.936807
miR-450b-5p	1.10	0.989660
miR-625-3p	1.13	0.942081
miR-1307-3p	1.14	0.966243
miR-548c-5p	1.15	0.999161
let-7i-3p	1.15	0.943290
miR-502-3p	1.18	0.913220
miR-628-5p	1.21	0.907901
miR-576-3p	1.22	1.000000
miR-4454	1.23	0.898525
miR-15b-5p	1.24	0.887769
miR-503-5p	1.24	0.899931
miR-16-2-3p	1.28	0.923453
miR-423-5p	1.30	0.877923
miR-5000-3p	1.30	0.882248
miR-1268a	1.37	0.921588
miR-2355-5p	1.38	0.892912
miR-1260b	1.38	0.858396
miR-1303	1.40	0.866581
miR-30b-5p	1.42	0.844626
miR-222-3p	1.47	0.912890
miR-132-3p	1.49	0.896559
miR-16-5p	1.49	0.804294
miR-454-3p	1.49	0.899713
miR-30b-3p	1.54	0.812728
miR-126-5p	1.55	0.866448
miR-146b-5p	1.59	0.879689
miR-339-5p	1.60	0.879335
miR-450a-5p	1.61	0.826474
miR-3184-5p	1.62	0.872043
let-7g-5p	1.62	0.741362
miR-95-3p	1.62	0.799080
miR-1286	1.63	0.783560
miR-191-3p	1.64	0.802208
miR-30d-3p	1.65	0.862656
miR-423-3p	1.65	0.764413
miR-3940-3p	1.65	0.783277
miR-548l	1.65	0.939143
miR-212-3p	1.67	0.779331
let-7a-3p	1.67	0.819797
miR-22-3p	1.68	0.786232
miR-6511b-3p	1.69	0.834075
miR-15a-5p	1.71	0.826200
miR-1272	1.75	0.779417
miR-328-3p	1.79	0.763774
miR-1180-3p	1.80	0.813410
miR-103a-3p	1.83	0.690345
miR-324-3p	1.83	0.740285
miR-625-5p	1.83	0.740285
miR-744-3p	1.85	0.728452
miR-1343-3p	1.85	0.879807
miR-93-3p	1.87	0.719200
miR-6859-5p	1.88	0.870728
miR-659-5p	1.89	0.712474
miR-660-5p	1.90	0.780752
miR-873-3p	1.92	0.703791
miR-219a-1-3p	1.99	0.695841

Bhat et al,2017. Cigarette smoke and chewing tobacco alter expression of different sets of miRNAs in oral keratinocytes		
**Supplementary Table S1(B) . miRNA expression of OKF6/TERT1-Smoke cells**		
**miRNA ID**	**Log2 Fold-change**	**p-value**
miR-3677-3p	2.00	0.695449
miR-188-3p	2.02	0.828276
miR-10b-5p	2.03	0.721410
miR-5683	2.04	0.820325
miR-374b-3p	2.05	0.669262
miR-185-5p	2.07	0.720789
miR-484	2.07	0.707168
miR-128-3p	2.09	0.717730
miR-92a-1-5p	2.10	0.704031
miR-548e-5p	2.11	0.710643
miR-629-3p	2.11	0.676360
miR-195-3p	2.12	0.707059
miR-19b-1-5p	2.16	0.696533
let-7d-5p	2.19	0.590310
miR-210-5p	2.21	0.687945
miR-574-5p	2.22	0.715447
miR-548i	2.31	0.654502
miR-3187-3p	2.33	0.736300
miR-301a-3p	2.34	0.637533
miR-454-5p	2.36	0.619637
miR-140-3p	2.37	0.590008
miR-18a-3p	2.37	0.653268
miR-3200-3p	2.38	0.599752
miR-874-5p	2.39	0.601471
miR-652-5p	2.41	0.712266
miR-5690	2.43	0.620246
miR-548o-3p	2.44	0.664974
miR-615-5p	2.45	0.700911
miR-3065-5p	2.52	0.597006
let-7a-2-3p	2.58	0.542873
miR-590-3p	2.59	0.539523
miR-374a-3p	2.60	0.638516
miR-4664-3p	2.62	0.654491
miR-301a-5p	2.67	0.532457
miR-126-3p	2.71	0.580450
miR-4645-3p	2.72	0.627158
miR-1307-5p	2.80	0.515688
miR-10a-5p	2.83	0.421856
miR-25-5p	2.84	0.487471
miR-589-3p	2.84	0.570837
miR-616-5p	2.85	0.594372
miR-100-3p	2.85	0.550441
let-7f-1-3p	2.86	0.482744
miR-1271-5p	2.86	0.489092
miR-22-5p	2.89	0.537376
miR-339-3p	2.89	0.576723
miR-3605-3p	2.90	0.481604
miR-455-5p	2.96	0.547321
miR-32-3p	2.97	0.473246
miR-130b-3p	3.01	0.497449
miR-1908-5p	3.03	0.511692
miR-2277-5p	3.05	0.444936
miR-2467-5p	3.08	0.442097
miR-3158-3p	3.15	0.466461
miR-1296-5p	3.22	0.482632
miR-505-3p	3.23	0.424804
miR-19b-3p	3.27	0.443336
miR-424-3p	3.28	0.473424
miR-4662a-5p	3.37	0.468304
miR-873-5p	3.61	0.350674
miR-539-3p	3.69	0.401297
miR-33b-3p	3.71	0.375107
miR-101-3p	3.72	0.420775
miR-340-5p	3.79	0.397521
miR-1285-3p	3.79	0.393106
miR-125b-2-3p	3.81	0.329164
miR-29a-3p	3.82	0.243835
miR-17-3p	3.83	0.395912
let-7g-3p	3.87	0.366912
miR-497-5p	3.91	0.327610
miR-132-5p	4.01	0.299253
miR-424-5p	4.05	0.366526
miR-561-5p	4.09	0.354079
miR-3065-3p	4.18	0.309196
miR-378a-5p	4.24	0.284232
miR-301b-3p	4.24	0.327178
miR-1275	4.27	0.318145
miR-411-3p	4.43	0.278577
miR-378a-3p	4.47	0.166218
miR-409-3p	4.47	0.295889
miR-33a-3p	4.48	0.245843
miR-31-3p	4.48	0.245148
miR-654-3p	4.49	0.306655
miR-20a-5p	4.51	0.224252
miR-19a-3p	4.80	0.259545
miR-29b-1-5p	4.81	0.217917
miR-335-3p	4.88	0.261313
miR-7974	5.04	0.215001

Bhat et al,2017. Cigarette smoke and chewing tobacco alter expression of different sets of miRNAs in oral keratinocytes		
**Supplementary Table S1(B) . miRNA expression of OKF6/TERT1-Smoke cells**		
**miRNA ID**	**Log2 Fold-change**	**p-value**
miR-31-5p	5.29	0.191418
miR-155-5p	5.45	0.201060
miR-130b-5p	5.46	0.178166
miR-889-3p	5.51	0.175718
miR-29b-3p	5.70	0.163255
miR-32-5p	5.83	0.161366
miR-146b-3p	5.85	0.165931
miR-29c-3p	5.89	0.164171
miR-935	6.20	0.146984
miR-193a-5p	6.50	0.134117
miR-411-5p	6.56	0.130961
miR-410-3p	7.38	0.101094
miR-9-5p	8.04	0.069978
miR-381-3p	8.25	0.070126
miR-127-3p	8.25	0.028338
miR-33b-5p	8.78	0.064567

Bhat et al,2017. Cigarette smoke and chewing tobacco alter expression of different sets of miRNAs in oral keratinocytes

Supplementary Table S2(A). List of all proteins and peptides identified in OKF6/TERT1 cells chronically treated with chewing tobacco using Sequest and Mascot search algorithms

Accession	Gene_Symbol	Description	Gene_ID	Refseq_Accession	Coverage	Proteins	#Unique_Peptides	#Peptides	#PSMS	#AAs	MW [kDa]	calc. pI	OKF6-STE-6M/OKF6P_R1	OKF6-STE-6M/OKF6P_R2	OKF6-STE-6M/OKF6P_R3	Median fold change	Status	P-value (6M)	Sequence	#PSMs	Protein Accessions	Modifications	OKF6-STE-6M/OKF6P_R1	OKF6-STE-6M/OKF6P_R2	OKF6-STE-6M/OKF6P_R3	Xcorr	Ionscore	# Missed Cleavages	Theo. MH+ [Da]	Charge	m_z	DeltaM [ppm]	Deltam/z [Da]	RT [min]
16554616	MRPS6	28S ribosomal protein S6, mitochondria	64968	NP_115865.1	32.8	1	4	4	10	125	14.218	9.26	0.85	0.83	0.74	0.81	Significant	0.0018																
																			AMQRPETAATLK	2	16554616	2xTMT6plex [N-Term; K12]			0.736	3.008258343	30.76	0	1775.02496	3	592.3470459	0.91513942	0.000541465	39.52565387
																			YELALILK	2	16554616	2xTMT6plex [N-Term; K8]	0.801			2.034118414	19.22	0	1420.917959	2	710.9624634	-0.217515857	-0.000154536	96.67855135
																			DLENLGER	4	16554616	1xTMT6plex [N-Term]	0.854	0.832		2.234650373	23.37	0	1174.626532	2	587.8164673	-0.744244799	-0.000437105	48.15355026
																			ECEGIVPVPLAEK	2	16554616	2xTMT6plex [N-Term; K13]; 1xCarbamidomethyl [C2				1.822557211	3.55	0	1899.066156	2	950.0360107	-0.743278806	-0.000705768	68.82298214
10863877	PLSCR1	phospholipid scramblase 1	5359	NP_066928.1	7.232704403	1	2	2	8	318	35.026	4.94	0.543	0.54	0.54	0.54	Significant	1																
																			SLDEQCVVGK	6	10863877	2xTMT6plex [N-Term; K10]; 1xCarbamidomethyl [C6	0.543	0.559	0.54	4.622082233	66.77	0	1592.871814	2	796.9400024	0.57408437	0.000457221	50.59636596
																			VYFAAEDTDCCTR	2	10863877	1xTMT6plex [N-Term]; 2xCarbamidomethyl [C10; C11		0.476		3.386893034	26.92	0	1836.809401	2	918.9065552	-1.941882721	-0.001783434	45.92047397
119395750	KRT1	keratin, type II cytoskeletal 1	3848	NP_006112.3	22.51552795	1	6	12	53	644	65.999	8.12	0.59	0.57	0.89	0.69	Significant	0.022																
																			GSYGSGGSSYGSGGGSYGSGGGGGGHGSYGSGSSSGGYI	2	119395750	1xTMT6plex [N-Term]				5.817292213	54.21	0	3541.471052	3	1181.159546	-1.967536819	-0.002322658	31.75493446
																			GGGGGGYGSGGSSYGSGGGSYGSGGGGGGGI	8	119395750	1xTMT6plex [N-Term]	0.675	0.55		7.7242136	101.75	0	2613.114777	3	871.7098999	0.14128619	0.000123066	34.76447699
																			SLVNLGGSK	2	119395750	2xTMT6plex [N-Term; K9]		0.532		4.083090305	56.42	0	1332.825121	2	666.9172974	1.648120618	0.001098328	49.50623398
																			AQYEDIAQK	4	119395750	2xTMT6plex [N-Term; K9]	0.56	0.695		3.852577925	55.76	0	1523.846979	2	762.4266968	-0.565716456	-0.000431033	50.73536588
																			YEELQITAGR	6	119395750	1xTMT6plex [N-Term]	0.21	0.186	0.202	3.774420261	44.97	0	1408.76336	2	704.8848267	-0.697916851	-0.0004916	47.08808655
																			LALDIEIATYR	8	119395750	1xTMT6plex [N-Term]	3.505			2.008917332	16.81	0	1506.87291	2	753.9402466	0.203218241	0.000153112	85.16699517
																			NKYEDEINK	12	119395750	3xTMT6plex [N-Term; K2; K9]	0.918	0.83	0.912	3.587960243	28.5	1	1840.041939	2	920.526123	1.646768851	0.001515062	36.94344344
																			SISISVAR	2	119395750	1xTMT6plex [N-Term]		0.378		3.025356531	22.4	0	1061.651625	2	531.3290405	-0.772716109	-0.000410178	41.33976191
																			AEAESLYQSK	1	119395750	2xTMT6plex [N-Term; K10]		1.44		1.949577808		0	1583.868108	2	792.4356689	-2.55516817	-0.002023525	50.2020458
																			YEDEINKR	6	119395750	2xTMT6plex [N-Term; K7]	1.059	1.134	1.172	3.56978035	27.49	1	1524.842228	3	508.9517822	-0.940779502	-0.00047818	33.28155655
																			YEDEINK	4	119395750	2xTMT6plex [N-Term; K7]	1.202		0.999	3.350680113	32.52	0	1368.741117	2	684.8745117	0.46013632	0.000314904	42.23766616
																			IEISELNR	2	119395750	1xTMT6plex [N-Term]			0.894	2.538354635	17.82	0	1202.694218	2	601.8471069	-6.053536057	-0.003640276	47.95686965
47132620	KRT2	keratin, type II cytoskeletal 2 epidermal	3849	NP_000414.2	14.55399061	1	2	11	324	639	65.393	8	0.86	1.1	0.71	0.89	Significant	0.016																
																			NLDLDSIIAEVK	45	47132620	2xTMT6plex [N-Term; K12]	0.705	0.96	0.55	4.597734928	71.71	0	1788.051886	2	894.5292969	-0.318284947	-0.000284555	97.09807644
																			LALDVEIATYR	151	47132620	1xTMT6plex [N-Term]	0.753	0.754	0.751	4.44096899	56.01	0	1492.85726	2	746.9325562	0.385451914	0.000287712	110.5595349
																			GFSSGSAVVSGGSR	6	47132620	1xTMT6plex [N-Term]	0.964	1.1	0.755	5.464034081	68.65	0	1483.770236	2	742.3886108	-0.196169397	-0.000145535	34.46005222
																			GGSISGGGYGSGGGK	4	47132620	2xTMT6plex [N-Term; K15]	0.69		0.604	5.336164951	90.81	0	1655.875319	2	828.4423218	1.236816966	0.001024007	39.4479817
																			AQYEEIAQR	12	47132620	1xTMT6plex [N-Term]	0.495	0.423	0.431	3.645548105	42.75	0	1336.705845	2	668.8570557	0.740363431	0.000494824	43.93391917
																			LLEGEECR	29	47132620	1xTMT6plex [N-Term]; 1xCarbamidomethyl [C7	0.83	0.873	0.841	3.377986431	36.73	0	1234.629903	2	617.817688	-1.461112705	-0.000901967	39.11707538
																			FASFIDK	41	47132620	2xTMT6plex [N-Term; K7]	0.677	0.84	0.69	3.288649321	38.52	0	1285.755645	2	643.3812866	-0.27093625	-0.000174179	75.39418814
																			KLLEGEECR	24	47132620	2xTMT6plex [N-Term; K1]; 1xCarbamidomethyl [C8	0.579	0.555	0.552	3.393291712	31.56	1	1591.887798	2	796.4476318	0.118602501	9.44E-05	39.43144923
																			YEDEINKR	6	47132620	2xTMT6plex [N-Term; K7]	1.059	1.134	1.172	3.56978035	27.49	1	1524.842228	3	508.9517822	-0.940779502	-0.00047818	33.28155655
																			YEDEINK	4	47132620	2xTMT6plex [N-Term; K7]	1.202		0.999	3.350680113	32.52	0	1368.741117	2	684.8745117	0.46013632	0.000314904	42.23766616
																			IEISELNR	2	47132620	1xTMT6plex [N-Term]			0.894	2.538354635	17.82	0	1202.694218	2	601.8471069	-6.053536057	-0.003640276	47.95686965
20149568	NDUFV1	NADH dehydrogenase [ubiquinone] flavoprotein 1, mitochondrial isoform 1 precursor	4723	NP_009034.2	4.74137931	1	2	2	7	464	50.785	8.21	1.42	1.71	1.42	1.52	Significant	0.004																
																			AAYIYIR	6	20149568	1xTMT6plex [N-Term]	1.39	1.714	1.424	2.77845335	31.4	0	1098.650897	2	549.8292847	0.360584002	0.000198078	53.00000381
																			YLVVNADEGEPGTCK	1	20149568	2xTMT6plex [N-Term; K15]; 1xCarbamidomethyl [C14	100			1.337235212		0	2110.089077	2	1055.54541	-2.622096651	-0.002766429	50.30345257
28827793	DHFRL1	dihydrofolate reductase, mitochondria	200895	NP_789785.1	3.743315508	1	1	1	6	187	21.606	7.97	1.63	2.22	1.99	1.95	Significant	0.0077																
																			SLDDALK	6	28827793	2xTMT6plex [N-Term; K7]	1.629	2.223	1.992	2.963023186	38.12	0	1219.729824	2	610.3689575	0.667606093	0.00040715	46.7788719
33636742	PLOD2	procollagen-lysine,2-oxoglutarate 5-dioxygenase 2 isoform 1 precursor	5352	NP_891988.1	12.79683377	1	7	7	30	758	87.043	6.71	1.46	1.73	1.45	1.54	Significant	0.0035																
																			IFTENIVEQPCPDVFWFPIFSEK	2	33636742	2xTMT6plex [N-Term; K23]; 1xCarbamidomethyl [C11	100			3.002804279	16.85	0	3300.70553	3	1100.906738	0.039986527	4.40E-05	116.2541264
																			ACDELVEEMEHYGK	8	33636742	2xTMT6plex [N-Term; K14]; 1xCarbamidomethyl [C2	1.115	1.345	100	2.981454372	27.29	0	2168.040412	3	723.350769	-1.2259509	-0.00088597	77.46059706
																			IVQQWNLQDNDDDQLFYTK	2	33636742	2xTMT6plex [N-Term; K19]				1.911482453	8.85	0	2841.445944	3	947.8188477	-1.391605305	-0.001318057	83.19428789
																			DKLDPDMALCR	6	33636742	2xTMT6plex [N-Term; K2]; 1xCarbamidomethyl [C10	1.25	1.733	1.467	3.256323814	17.76	1	1791.949747	3	597.987854	-0.411701434	-0.000245916	46.69603599
																			LADKYPVVHIGK	6	33636742	3xTMT6plex [N-Term; K4; K12]	1.496	1.729	1.146	3.118700027	10.32	1	2027.262043	4	507.570343	-1.233737738	-0.000625277	54.45141131
																			EFIAPVTLK	4	33636742	2xTMT6plex [N-Term; K9]	100	100		2.410449743	16.42	0	1475.923773	2	738.4658203	0.400538978	0.000295583	72.80781514
																			LLVITVATK	2	33636742	2xTMT6plex [N-Term; K9]	100			1.507034779	6.95	0	1415.960158	2	708.484314	0.842502298	0.000596475	76.091752
7706254	NOP58	nucleolar protein 58	51602	NP_057018.1	29.30056711	1	12	12	44	529	59.541	8.92	0.886	0.905	2.456	1.42	Non-Significant	0.11																
																			EAHEPLAVADAK	2	7706254	2xTMT6plex [N-Term; K12]	0.821			5.428192616	43.65	0	1708.963406	3	570.3268433	1.504353544	0.000856962	38.86962472
																			KIEQVDKEDEITEK	4	7706254	4xTMT6plex [N-Term; K1; K7; K14]	0.715	0.881		4.918598652	25.15	2	2620.521514	3	874.1793823	0.793743782	0.000693341	42.86883973
																			LNLSCIHSPVVNELMR	6	7706254	1xTMT6plex [N-Term]; 1xCarbamidomethyl [C5	100	0.01		3.540265083	22.85	0	2111.130281	3	704.3811035	-0.721667032	-0.000507844	82.8144305
																			VAEEEETSVK	4	7706254	2xTMT6plex [N-Term; K10]	1.271	1.563		4.125188351	65.86	0	1578.862689	2	789.9354248	0.56017498	0.00044222	44.44831906
																			TYDPSGDSTLPTCSK	2	7706254	2xTMT6plex [N-Term; K15]; 1xCarbamidomethyl [C13			1.948	2.660474539	36.41	0	2087.036707	2	1044.021729	-0.252122422	-0.000263094	49.10114173
																			SQMDGLIPGVEPR	6	7706254	1xTMT6plex [N-Term]	0.01	100		2.977535963	33.5	0	1627.867507	2	814.4385986	1.482513543	0.001206668	63.05179194
																			LIAHAGSLLNLAK	4	7706254	2xTMT6plex [N-Term; K13]	1.269	0.978		2.87630868	16.95	0	1779.12566	3	593.7123413	-1.792854934	-0.001063238	70.98669544
																			VKVEEEEEEK	2	7706254	3xTMT6plex [N-Term; K2; K10]	0.846			2.915243626	24.38	1	1935.088949	3	645.7015381	0.574644289	0.000370663	44.4456809
																			IISDNLTYCK	6	7706254	2xTMT6plex [N-Term; K10]; 1xCarbamidomethyl [C9	100	0.786	100	2.201221943	30.3	0	1684.934414	2	842.9702759	-0.675944522	-0.000569461	54.13001591
																			MMAIAPNVTVMVGELVGAR	2	7706254	1xTMT6plex [N-Term]				1.201095104	2.49	0	2188.185353	2	1094.596436	0.110559076	0.000120962	104.2055657
																			LQEVDSLWK	3	7706254	2xTMT6plex [N-Term; K9]	100			1.916269779	15.41	0	1575.914665	2	788.4604492	-0.661725234	-0.000521411	70.89648505
																			LSELLPEEVEAEVK	3	7706254	2xTMT6plex [N-Term; K14]				1.371527672	5.35	0	2043.162559	2	1022.084717	-0.19664429	-0.000200888	96.14550452
193083197	UBLCP1	ubiquitin-like domain-containi CTD phosphatase 1	134510	NP_659486.2	18.23899371	1	5	5	21	318	36.781	6.46	2.14	1.34	1.18	1.55	Significant	0.035																
																			ELGVSTNANYK	6	193083197	2xTMT6plex [N-Term; K11]	2.17	1.633	1.165	3.886206388	28.56	0	1653.921206	3	551.9816284	5.51749616	0.003041835	42.72873032
																			LLVLDVDYTLFDHR	6	193083197	1xTMT6plex [N-Term]	2.632	1.029	1.538	3.601837635	45.13	0	1948.074129	3	650.0287476	-1.252367239	-0.000813235	102.9009542
																			LDDFLDLNHK	2	193083197	2xTMT6plex [N-Term; K10]	1.828			2.070535421	22.37	0	1687.941942	3	563.3192749	0.787643815	0.000443166	75.44100002
																			TLTGVLPER	2	193083197	1xTMT6plex [N-Term]		1.105		2.655232668	27.44	0	1214.730603	2	607.8690796	0.229877873	0.00013962	56.8453455
																			GLIDVKPLGVIWGK	5	193083197	3xTMT6plex [N-Term; K6; K14]				1.561081171	5.6	0	2182.393057	3	728.1367798	1.250322649	0.000909565	84.10822181
385648268	SLC4A7	sodium bicarbonate cotransporter 3 isoform b	9497	NP_001245308.1	5.835543767	1	5	5	13	1131	127.278	6.76	1.93	2.09	1.35	1.79	Significant	0.015																
																			ALSMNTENAK	2	385648268	2xTMT6plex [N-Term; K10]			1.353	4.426970959	46.88	0	1536.845599	2	768.9266357	0.257719042	0.000198037	43.66407684
																			SIATLMTDEIFHDVAYK	2	385648268	2xTMT6plex [N-Term; K17]	0.661			2.238305569	17.8	0	2412.288504	3	804.7679443	0.321494635	0.000258713	105.1068615
																			FEEDVEDGGDR	4	385648268	1xTMT6plex [N-Term]	2.181	1.929		3.808953762	36.28	0	1496.670247	2	748.8380127	-1.00121545	-0.000749245	47.45538223
																			GPDEEAVVDLGK	4	385648268	2xTMT6plex [N-Term; K12]		100		1.912386775	28.59	0	1686.931437	2	843.9683838	-1.153474191	-0.000972916	49.46843433
																			YSVDPSIVNISDEMAK	1	385648268	2xTMT6plex [N-Term; K16]				1.098311186		0	2226.172806	2	1113.589722	-0.287066944	-0.00031953	71.71973519
4504437	HMOX1	heme oxygenase 1	3162	NP_002124.1	4.513888889	1	1	1	12	288	32.798	8.25	3.03	3.05	3.23	3.1	Significant	0.00042																
																			EVHTQAENAEFMR	6	4504437	1xTMT6plex [N-Term]	3.67	3.482	3.271	5.309963703	39.84	0	1790.869298	3	597.6279297	-0.034802931	-2.08E-05	36.18635158
																			EVHTQAENAEFMR	6	4504437	1xTMT6plex [N-Term]; 1xOxidation [M12]	1.037	1.481	2.893	3.176583767	16.51	0	1806.864213	3	597.6279297	-0.034802931	-2.08E-05	36.18635158
124028529	SYMPK	symplekin	8189	NP_004810.2	1.491365777	1	2	2	8	1274	141.059	6.13	2.54	2.45	2.81	2.6	Significant	0.0017																
																			AVACSGAAQVR	6	124028529	1xTMT6plex [N-Term]; 1xCarbamidomethyl [C4	2.548	2.445	2.814	3.890422821	60	0	1318.709885	2	659.8582764	-0.461675181	-0.000304408	33.86869417
																			AALEQLLK	2	124028529	2xTMT6plex [N-Term; K8]	2.386			2.344355106	39.03	0	1343.866258	2	672.4370117	0.363739693	0.000244409	72.25159165
4757818	ATP6V1G1	V-type proton ATPase subunit G 1	9550	NP_004879.1	11.86440678	1	2	2	16	118	13.749	8.79	1.508	3.033	8.718	4.42	Non-Significant	0.18																
																			GSCSTEVEK	10	4757818	2xTMT6plex [N-Term; K9]; 1xCarbamidomethyl [C3	0.956	1.419	0.876	3.47955513	55.38	0	1454.756115	2	727.8822021	0.695839574	0.000506138	27.38250934
																			GSCSTEVEKETQEK	6	4757818	3xTMT6plex [N-Term; K9; K14]; 1xCarbamidomethyl [C3	4.595	16.546	28.824	2.937583208	9	1	2299.205452	3	767.0727539	-0.758419065	-0.000581254	32.21555718
28302131	HBG1	hemoglobin subunit gamma-1	3047	NP_000550.2	12.92517007	1	2	2	26	147	16.118	7.2	0.31	0.36	0.33	0.33	Significant	0.0019																
																			LHVDPENFK	16	28302131	2xTMT6plex [N-Term; K9]	0.31	0.366	0.324	3.527098656	43.43	0	1556.883699	3	519.6323242	-0.82182359	-0.000426495	53.00821479
																			LLVVYPWTQR	10	28302131	1xTMT6plex [N-Term]	0.37	0.233	0.392	3.267573833	29.01	0	1503.888501	2	752.4465942	-1.721559435	-0.001294517	82.95177065
188528675	SLIT1	slit homolog 1 protein precursor	6585	NP_003052.2	0.586701434	1	1	1	6	1534	167.816	6.57	0.24	0.5	0.54	0.43	Significant	0.045																
																			GLNVAEVQK	6	188528675	2xTMT6plex [N-Term; K9]	0.242	0.502	0.539	2.440283298	16.91	0	1415.862235	2	708.4356079	1.203507142	0.000852	46.20658747
226371706	PARP9	poly [ADP-ribose] polymerase 9 isoform a	83666	NP_113646.2	8.899297424	1	7	7	21	854	96.281	7.91	0.35	0.35	0.32	0.34	Significant	0.00086																
																			VSAGEIAVTGAGR	2	226371706	1xTMT6plex [N-Term]			0.371	4.345033646	67.32	0	1416.800808	2	708.9049072	1.220928952	0.000864907	46.97368463
																			KEHDILSQLQK	4	226371706	3xTMT6plex [N-Term; K1; K11]		0.308	0.321	3.962651968	24.06	1	2026.226386	3	676.0801392	-0.257512948	-0.000173927	46.52373256
																			EIHLVSNEDPTVAAFK	4	226371706	2xTMT6plex [N-Term; K16]		100	0.01	2.186769485	14.09	0	2228.232704	3	743.4164429	0.929617126	0.000690468	63.914948
																			AFSSEMAK	2	226371706	2xTMT6plex [N-Term; K8]	0.366			3.809786797	38.69	0	1328.728444	2	664.8681641	0.457275527	0.000303798	45.93465691
																			FVIFPTDLEIYK	6	226371706	2xTMT6plex [N-Term; K12]	0.49	0.01	0.01	1.715453744	9.82	0	1943.129408	2	972.0679932	-0.359524112	-0.000349301	96.57468536
																			ENIIFLK	2	226371706	2xTMT6plex [N-Term; K7]	0.01			2.09940815	22.01	0	1334.844794	2	667.9258423	-0.289441656	-0.00019318	73.17423542
																			TELEIEGAR	1	226371706	1xTMT6plex [N-Term]		0.373		2.33196187	9.63	0	1246.684047	2	623.8455811	-0.129552171	-8.08E-05	40.29750873
153082755	IFIT2	interferon-induced protein with tetratricopeptide repeats 2	3433	NP_001538.4	22.24576271	1	10	10	27	472	54.597	6.76	0.57	0.36	0.33	0.42	Significant	0.031																
																			KAEELIQQEHADQAEIR	4	153082755	2xTMT6plex [N-Term; K1]	0.537		0.388	5.909845829	39.03	1	2466.335271	4	617.3393555	0.129996519	8.02E-05	54.03890247
																			GQNEAALECLR	2	153082755	1xTMT6plex [N-Term]; 1xCarbamidomethyl [C9		0.383		3.948349953	51.45	0	1489.763043	2	745.3848267	-0.446983626	-0.00033295	47.67030504
																			MREEGEEEGEGEK	2	153082755	2xTMT6plex [N-Term; K13]	0.368			3.426510096	11.45	1	1966.942806	3	656.3199463	1.26068088	0.000826562	18.79193262
																			EEGEEEGEGEK	2	153082755	2xTMT6plex [N-Term; K11]		100		2.623484135	23.94	0	1679.80121	2	840.4051514	1.080910267	0.000907857	27.71309835
																			LVEEALEK	4	153082755	2xTMT6plex [N-Term; K8]	0.427			3.157428741	43.2	0	1388.840103	2	694.9245605	1.254070751	0.000870852	63.56786654
																			APGVTDVLR	2	153082755	1xTMT6plex [N-Term]			0.29	2.764544249	35.82	0	1156.688739	2	578.8475342	-0.818543998	-0.0004734	42.99962849
																			LNPDNQYLK	3	153082755	2xTMT6plex [N-Term; K9]	0.803		0.571	3.218377829	17.72	0	1562.894264	2	781.9498901	-1.126049665	-0.000879948	45.85838298
																			VFQVMNLR	4	153082755	1xTMT6plex [N-Term]		0.01		1.810385108	28.24	0	1235.713179	2	618.3602905	0.101354173	6.26E-05	78.60618436
																			LSDVQIYVDK	2	153082755	2xTMT6plex [N-Term; K10]	0.803			1.53896904	10.58	0	1637.951444	2	819.4794312	0.086488942	7.08E-05	69.05356885
																			ALEYIPNNAYLHCQIGCCYR	2	153082755	1xTMT6plex [N-Term]; 3xCarbamidomethyl [C13; C17; C18				1.051918626	1.09	0	2744.294463	3	915.4373779	1.136094944	0.00103926	69.72230794
240255483	SULF2	extracellular sulfatase Sulf-2 isoform a precursor	55959	NP_001155313.1	1.034482759	1	1	1	6	870	100.39	9.17	1.89	1.67	1.99	1.85	Significant	0.0026																
																			SVAIEVDGR	6	240255483	1xTMT6plex [N-Term]	1.885	1.667	1.991	3.744044065	55.34	0	1174.662918	2	587.835083	-0.024078716	-1.41E-05	39.37351012
4557871	TF	serotransferrin precursor	7018	NP_001054.1	27.93696275	1	16	16	73	698	77	7.12	5.55	4.37	6.37	5.43	Significant	0.011																
																			KPVDEYKDCHLAQVPSHTVVAR	12	4557871	3xTMT6plex [N-Term; K1; K7]; 1xCarbamidomethyl [C9	5.281	7.054	6.311	6.88876915	23.46	1	3236.781712	5	648.1625977	0.670237119	0.000433882	44.09848962
																			KPVEEYANCHLAR	7	4557871	2xTMT6plex [N-Term; K1]; 1xCarbamidomethyl [C9	8.56	8.355		4.539106846	60.81	0	2045.10025	4	512.0299683	-1.079308031	-0.000551823	45.31084323
																			EDPQTFYYAVAVVK	2	4557871	2xTMT6plex [N-Term; K14]		100		2.889977694	36.82	0	2088.141764	2	1044.57251	-1.925582266	-0.002010444	85.85537151
																			DSGFQMNQLR	2	4557871	1xTMT6plex [N-Term]		2.145		3.26022768	39.13	0	1424.715364	2	712.861084	-0.331667126	-0.000236266	52.96420632
																			EDLIWELLNQAQEHFGK	3	4557871	2xTMT6plex [N-Term; K17]	100			2.393669367	19.02	0	2528.354944	3	843.4554443	-1.251597287	-0.001054827	111.6795079
																			ADRDQYELLCLDNTR	6	4557871	1xTMT6plex [N-Term]; 1xCarbamidomethyl [C10	0.617		100	4.774323463	24.81	1	2111.038883	3	704.3516846	0.766300872	0.00053923	62.44342323
																			SVIPSDGPSVACVK	4	4557871	2xTMT6plex [N-Term; K14]; 1xCarbamidomethyl [C12	4.455	6.82		3.471910715	36.07	0	1874.045755	2	937.5272827	0.818282949	0.00076675	61.39980633
																			EFQLFSSPHGK	6	4557871	2xTMT6plex [N-Term; K11]	100	100	100	2.839578629	28.6	0	1734.957926	3	578.9909058	0.136974593	7.92E-05	54.49739947
																			FDEFFSEGCAPGSK	4	4557871	2xTMT6plex [N-Term; K14]; 1xCarbamidomethyl [C9	100	100		2.460909367	21.31	0	2035.983549	2	1018.493713	-1.669361321	-0.001699396	82.42527597
																			SASDLTWDNLK	2	4557871	2xTMT6plex [N-Term; K11]		100		2.020701885	27.82	0	1707.931771	2	854.4664307	-3.622189118	-0.003093226	63.14938807
																			ASYLDCIR	6	4557871	1xTMT6plex [N-Term]; 1xCarbamidomethyl [C6	100	2.828	5.57	2.982764482	32.94	0	1226.640074	2	613.8226929	-1.601910661	-0.000982484	46.71395103
																			DSAHGFLK	4	4557871	2xTMT6plex [N-Term; K8]	6.722	5.445		3.062274456	21.62	0	1332.767607	3	444.9269409	-1.003255246	-0.000445702	44.71915337
																			YLGEEYVK	2	4557871	2xTMT6plex [N-Term; K8]			100	2.96676898	32.24	0	1458.824453	2	729.9154663	-0.546133437	-0.000398356	50.47105972
																			DYELLCLDGTR	2	4557871	1xTMT6plex [N-Term]; 1xCarbamidomethyl [C6				2.017580032	8.5	0	1583.793674	2	792.401001	0.6638195	0.000525677	76.73274184
																			GDVAFVK	10	4557871	2xTMT6plex [N-Term; K7]		1.75		3.030616999	40.57	0	1193.72943	2	597.3685303	0.29627055	0.000176833	45.47780087
																			NLNEKDYELLCLDGTR	1	4557871	2xTMT6plex [N-Term; K5]; 1xCarbamidomethyl [C11			100	1.866053104		1	2411.264081	3	804.4258423	-0.459109618	-0.000369012	69.84379877
4758412	GALNT2	polypeptide N-acetylgalactosaminyltransferase 2 precursor	2590	NP_004472.1	27.49562172	1	9	9	42	571	64.691	8.35	2.31	1.76	1.7	1.92	Significant	0.01																
																			EIILVDDYSNDPEDGALLGK	8	4758412	2xTMT6plex [N-Term; K20]	1.846	100	1.751	4.092479706	50.18	0	2634.391449	3	878.8029175	1.044055699	0.000916817	86.01337728
																			VLTFLDSHCECNEHWLEPLLER	6	4758412	1xTMT6plex [N-Term]; 2xCarbamidomethyl [C9; C11	1.947			3.828424215	24.19	0	3026.470178	4	757.3726196	-0.505332709	-0.000382344	93.96764051
																			VPDHQDIAFGALQQGTNCLDTLGHFADGVVGVYECHNAGGNQEWALTK	2	4758412	2xTMT6plex [N-Term; K48]; 2xCarbamidomethyl [C18; C35]	100			2.507444382	1.5	0	5668.737808	5	1134.553833	0.396977094	0.000450072	102.7686245
																			HVGSNLCLDSR	6	4758412	1xTMT6plex [N-Term]; 1xCarbamidomethyl [C7		1.713	1.695	4.62042141	55.92	0	1486.763377	3	496.258728	-1.174455865	-0.000582046	34.50410264
																			WYLENVYPELR	4	4758412	1xTMT6plex [N-Term]			1.685	3.564915419	38.86	0	1710.905273	2	855.9552002	-1.256217634	-0.001074635	88.68612208
																			GGFDWNLVFK	2	4758412	2xTMT6plex [N-Term; K10]			1.592	2.788553715	44.97	0	1640.920084	2	820.9623413	-1.632262801	-0.001339206	96.34816473
																			VDLPATSVVITFHNEAR	2	4758412	1xTMT6plex [N-Term]	100			2.724119663	27.77	0	2098.149419	3	700.0561523	2.13738412	0.00149485	70.60094628
																			AAEVWMDEYK	6	4758412	2xTMT6plex [N-Term; K10]	100	1.401	1.966	2.495323896	33.44	0	1699.876565	2	850.4436035	1.980068036	0.001682936	68.73745466
																			GADAAQAK	6	4758412	2xTMT6plex [N-Term; K8]	0.992		1.76	2.591285944	38.91	0	1189.694107	2	595.350769	0.129550896	7.71E-05	29.11666508
17402893	PSAT1	phosphoserine aminotransferase isoform 1	29968	NP_478059.1	44.05405405	1	11	11	57	370	40.397	7.66	1.52	1.89	2.74	2.05	Significant	0.03																
																			GAVLVCDMSSNFLSKPVDVSK	6	17402893	3xTMT6plex [N-Term; K15; K21]; 1xCarbamidomethyl [C6	100	100	100	2.945443153	32.89	0	2940.614161	3	980.8691406	-7.240921056	-0.007097585	76.77292397
																			ASLYNAVTIEDVQK	4	17402893	2xTMT6plex [N-Term; K14]	0.427	0.567		3.288229942	38.4	0	2009.131927	2	1005.06958	-0.021817258	-2.19E-05	72.82341682
																			GVGISVLEMSHR	6	17402893	1xTMT6plex [N-Term]	1.702	2.189	2.757	4.182737827	60.31	0	1513.835813	3	505.2811584	-4.552170151	-0.002297079	49.76886122
																			ALELNMLSLK	6	17402893	2xTMT6plex [N-Term; K10]	1.015	100		2.739343882	41.47	0	1589.970071	2	795.4889526	0.350706877	0.000278807	95.07442882
																			ECPSVLEYK	6	17402893	2xTMT6plex [N-Term; K9]; 1xCarbamidomethyl [C2	1.95	2.533	2.441	3.858337402	48.18	0	1582.855101	2	791.930542	-0.817380962	-0.000646898	54.15282073
																			DDLLGFALR	8	17402893	1xTMT6plex [N-Term]	1.147	100	100	2.715025425	57.01	0	1248.714953	2	624.8595581	-2.49349866	-0.001556835	75.31890147
																			LPHSVLLEIQK	4	17402893	2xTMT6plex [N-Term; K11]	1.306	0.403		2.77683568	15.64	0	1735.088212	2	868.0464478	-1.494668783	-0.001296691	59.72875015
																			SQTIYEIIDNSQGFYVCPVEPQNR	4	17402893	1xTMT6plex [N-Term]; 1xCarbamidomethyl [C17				2.221243143	6.76	0	3086.509066	3	1029.508667	0.771812592	0.000794069	95.84064691
																			ELLAVPDNYK	6	17402893	2xTMT6plex [N-Term; K10]	1.548	1.711		3.75050807	42.91	0	1619.940879	2	810.4735107	-0.700331499	-0.000567248	69.85877222
																			IPDPSTWNLNPDASYVVYYCANETVHGVEFDFIPDVK	5	17402893	2xTMT6plex [N-Term; K36]; 1xCarbamidomethyl [C19			100	2.493449926	14.95	0	4631.248102	4	1158.569702	1.916754092	0.002219241	108.5291454
																			FLEMHQL	2	17402893	1xTMT6plex [N-Term]			1.034	1.375665784	26	0	1146.617882	2	573.812439	-0.24467638	-0.000140275	63.03306271
23308577	PHGDH	D-3-phosphoglycerate dehydrogenase	26227	NP_006614.2	18.94934334	1	10	10	44	533	56.614	6.71	1.75	2.11	1.82	1.89	Significant	0.0034																
																			GTIQVITQGTSLK	6	23308577	2xTMT6plex [N-Term; K13]	1.47	100	1.336	3.4560256	47.89	0	1804.09442	2	902.5507813	-0.074208976	-6.69E-05	54.31619689
																			KILQDGGLQVVEK	2	23308577	3xTMT6plex [N-Term; K1; K13]	1.038			4.156117439	32.74	1	2114.315201	3	705.4436035	0.499607095	0.000352109	87.86533437
																			AGTGVDNVDLEAATR	4	23308577	1xTMT6plex [N-Term]	6.009	100		4.658825397	84.2	0	1717.891808	2	859.4494629	-0.092269344	-7.93E-05	53.4000299
																			GGIVDEGALLR	4	23308577	1xTMT6plex [N-Term]	1.785		100	4.481899738	75.69	0	1328.773531	2	664.8900146	-0.585519735	-0.000389012	64.91616822
																			ALVDHENVISCPHLGASTK	4	23308577	2xTMT6plex [N-Term; K19]; 1xCarbamidomethyl [C11		1.98	1.734	5.100189209	54.31	0	2506.348813	4	627.3414917	-1.865818955	-0.001169098	44.78076247
																			ILQDGGLQVVEK	6	23308577	2xTMT6plex [N-Term; K12]	100	100	100	3.003665209	50.05	0	1757.057306	2	879.0335083	1.385254513	0.001216986	68.35152668
																			VLISDSLDPCCR	4	23308577	1xTMT6plex [N-Term]; 2xCarbamidomethyl [C10; C11	3.094	1.894		2.883440256	43.04	0	1663.834493	2	832.4215088	0.749911202	0.000623864	65.14779004
																			KVLISDSLDPCCR	4	23308577	2xTMT6plex [N-Term; K1]; 2xCarbamidomethyl [C11; C12	1.128	1.28		3.643768549	14.63	1	2021.092388	3	674.3686523	-0.487067665	-0.000328136	62.92467865
																			TLGILGLGR	4	23308577	1xTMT6plex [N-Term]	100	5.958		2.965748787	37.35	0	1128.730209	2	564.8683472	-0.701446684	-0.000395872	78.23960613
																			DLPLLLFR	6	23308577	1xTMT6plex [N-Term]	100	1.45	5.15	2.59215951	32.68	0	1215.766261	2	608.3855591	-1.989745904	-0.001209533	88.81259397
33356547	MCM2	DNA replication licensing factor MCM2	4171	NP_004517.2	18.14159292	1	12	12	50	904	101.832	5.52	1.47	3.463	3.877	2.94	Non-Significant	0.058																
																			AIPELDAYEAEGLALDDEDVEELTASQR	10	33356547	1xTMT6plex [N-Term]	1.956	100	100	4.289848328	50.41	0	3291.595357	3	1097.870483	0.467975296	0.000513462	96.28209692
																			ATEDGEEDEEMIESIENLEDLK	7	33356547	2xTMT6plex [N-Term; K22]	100	100	100	2.859899998	30.14	0	2996.418193	3	999.4789429	1.362394372	0.001360768	103.1901084
																			QLVAEQVTYQR	4	33356547	1xTMT6plex [N-Term]		4.262	3.371	4.231797695	38.15	0	1563.869222	2	782.4318848	-8.139413815	-0.006364489	43.27078208
																			VMLESFIDTQK	2	33356547	2xTMT6plex [N-Term; K11]		2.678		3.105551243	40.72	0	1768.991929	2	884.9974365	-2.44894454	-0.002166082	88.37972084
																			DTVDPVQDEMLAR	6	33356547	1xTMT6plex [N-Term]	3.884		100	3.008696556	31.46	0	1717.862816	2	859.4347534	-0.340861946	-0.000292777	49.08957496
																			GVCLIDEFDK	3	33356547	2xTMT6plex [N-Term; K10]; 1xCarbamidomethyl [C3	100	100		1.804781079	24.25	0	1653.892215	2	827.449707	-0.04683951	-3.87E-05	66.9234206
																			EWVSMAGPR	2	33356547	1xTMT6plex [N-Term]		100		2.55426383	30.72	0	1261.656058	2	631.3317261	0.092987654	5.87E-05	55.98892905
																			LNQMDQDK	6	33356547	2xTMT6plex [N-Term; K8]		3.158	3.897	3.487321138	29.83	0	1449.777185	2	725.3914185	-1.120603879	-0.000812313	35.39845168
																			AGIVTSLQAR	3	33356547	1xTMT6plex [N-Term]		2.211		2.437154293	18.14	0	1244.752401	2	622.8793335	-0.812215997	-0.000505504	43.81473902
																			QLHLNQLIR	2	33356547	1xTMT6plex [N-Term]	1.274			2.574602842	17.78	0	1363.837134	3	455.2839355	0.087503575	3.98E-05	50.07762901
																			ESLVVNYEDLAAR	3	33356547	1xTMT6plex [N-Term]				1.369993567	13.11	0	1707.911481	2	854.458374	-1.176350852	-0.001004552	60.65839656
																			YDPSLTFSENVDLTEPIISR	2	33356547	1xTMT6plex [N-Term]				0.962380767	3.52	0	2525.297265	2	1263.150391	-1.489072218	-0.001880175	90.38547873
67089147	FDFT1	squalene synthase isoform 1	2222	NP_004453.3	32.13429257	1	11	11	56	417	48.084	6.54	4.93	4.91	5.01	4.95	Significant	0.000038																
																			MDQDSLSSSLK	6	67089147	2xTMT6plex [N-Term; K11]	0.338	2.333	2.046	3.567463636	44.13	0	1668.887857	2	834.9473877	-0.214909212	-0.00017933	47.95553187
																			TQNLPNCQLISR	2	67089147	1xTMT6plex [N-Term]; 1xCarbamidomethyl [C7	2.609			2.707253218	20.16	0	1672.900205	2	836.953125	-0.736045101	-0.000615665	53.03842415
																			SFAAVIQALDGEMR	8	67089147	1xTMT6plex [N-Term]	100	2.691		3.156714916	44.71	0	1736.920271	2	868.9637451	-0.033107809	-2.88E-05	104.3485296
																			HVTSEQEWDK	8	67089147	2xTMT6plex [N-Term; K10]	5.538	5.04	5.212	3.85239315	37.85	0	1716.89507	2	858.9516602	0.188527757	0.000161841	38.6793845
																			LFSASEFEDPLVGEDTER	6	67089147	1xTMT6plex [N-Term]	100	100		2.283334494	18.62	0	2270.102588	2	1135.553955	-0.860971553	-0.000977247	96.21187832
																			QVLEDFPTISLEFR	7	67089147	1xTMT6plex [N-Term]		100		2.146636009	21.13	0	1923.042495	2	962.0224609	-2.521725346	-0.002424692	95.49283166
																			YQTVIADICR	6	67089147	1xTMT6plex [N-Term]; 1xCarbamidomethyl [C9	100	100		2.275898695	29.47	0	1467.782715	2	734.3951416	0.198342113	0.000145562	63.00508131
																			DYLEDQQGGR	2	67089147	1xTMT6plex [N-Term]	2.733			2.905443668	42.98	0	1409.685838	2	705.3457642	-1.12516537	-0.000793065	37.26481911
																			IPDSDPSSSK	4	67089147	2xTMT6plex [N-Term; K10]	4.515		4.271	3.407083035	43.65	0	1490.810259	2	745.9041748	-6.161830836	-0.00459306	36.94713226
																			ALDTLEDDMTISVEK	2	67089147	2xTMT6plex [N-Term; K15]				1.699306011	9.97	0	2138.130272	2	1069.567505	-1.187548957	-0.001269567	80.37550351
																			EFWPQEVWSR	5	67089147	1xTMT6plex [N-Term]	100			1.686380744	5.34	0	1592.805893	2	796.9067383	0.192441842	0.000153261	87.77971718
4505415	NQO1	NAD(P)H dehydrogenase [quinone] 1 isoform a	1728	NP_000894.1	33.94160584	1	8	8	46	274	30.848	8.88	2.88	2.95	3.31	3.04	Significant	0.0019																
																			LKDPANFQYPAESVLAYK	12	4505415	3xTMT6plex [N-Term; K2; K18]	3.562	100	8.124	3.590634584	26.15	1	2741.548114	3	914.5216675	0.85186592	0.000778477	78.38761278
																			EGHLSPDIVAEQK	6	4505415	2xTMT6plex [N-Term; K13]	4.269	3.805	2.578	4.975418568	40.33	0	1881.048198	3	627.6873779	-0.328152643	-0.000205757	42.41279786
																			ALIVLAHSER	8	4505415	1xTMT6plex [N-Term]	2.665	2.678		3.665201664	54.64	0	1337.810251	2	669.4092407	0.713281508	0.000477118	44.74316099
																			EAAAAALK	6	4505415	2xTMT6plex [N-Term; K8]	3.535	3.479	3.689	3.488672972	50.59	0	1202.750894	2	601.878479	-1.008157378	-0.000606281	38.01781543
																			IQILEGWK	6	4505415	2xTMT6plex [N-Term; K8]	4.139	5.213	100	3.278888464	37.27	0	1444.892807	2	722.9491577	-1.223814185	-0.00088414	82.14887433
																			TSFNYAMK	4	4505415	2xTMT6plex [N-Term; K8]	3.324	2.935		3.800091505	44.52	0	1419.770643	2	710.3890381	0.110223349	7.82E-05	59.91778589
																			KEVQDEEK	2	4505415	3xTMT6plex [N-Term; K1; K8]			2.962	2.938932657	20.15	1	1691.978277	3	564.6639404	-0.596030665	-0.000336157	32.9572726
																			TSFNYAMK	1	4505415	2xTMT6plex [N-Term; K8]; 1xOxidation [M7]	0.564			1.28427577	12.27	0	1435.765558	2	710.3890381	0.110223349	7.82E-05	59.91778589
																			GWEVVESDLYAMNFNPIISR	1	4505415	1xTMT6plex [N-Term]				0.830404818		0	2569.295825	2	1285.150146	-1.093268911	-0.001404466	115.2086051
38679960	ACACA	acetyl-CoA carboxylase 1 isoform 1	31	NP_942131.1	3.902643726	1	9	9	35	2383	269.827	6.52	2.42	2.84	2.05	2.44	Significant	0.0087																
																			VEVGTEVTDYR	2	38679960	1xTMT6plex [N-Term]		2.724		4.022089005	44.81	0	1496.779404	2	748.8927612	-0.773647112	-0.00057899	59.15416711
																			VSALNSVHCEHVEDEGESR	6	38679960	1xTMT6plex [N-Term]; 1xCarbamidomethyl [C9	0.731	2.905	2.121	5.606604576	59.27	0	2383.114567	4	596.5336304	-0.786838221	-0.000468781	34.42911917
																			GVISDILDWK	4	38679960	2xTMT6plex [N-Term; K10]	4.877	3.694		2.985850573	41.45	0	1603.945965	2	802.475708	-1.138046053	-0.000912682	102.213515
																			IYVSANSGAR	6	38679960	1xTMT6plex [N-Term]	2.695	2.903	2	3.340305805	51.3	0	1266.700366	2	633.854126	0.48119756	0.000304767	34.96293827
																			LLLEDLVK	4	38679960	2xTMT6plex [N-Term; K8]	1.839	1.88		3.464728117	30.3	0	1400.912874	2	700.9589233	-1.64446366	-0.001151875	96.54519388
																			AVVMDLLR	2	38679960	1xTMT6plex [N-Term]	100			2.170154333	16.5	0	1145.691381	2	573.3494263	0.17001879	9.74E-05	83.4966459
																			DLAEWLEK	4	38679960	2xTMT6plex [N-Term; K8]	100			1.827567577	10.29	0	1461.835352	2	731.4182129	-4.243007766	-0.003101289	78.03267545
																			IIEFVPTK	6	38679960	2xTMT6plex [N-Term; K8]	100	100		1.447306871	6.2	0	1404.886659	2	702.9460449	-1.313889799	-0.000922933	68.33862485
																			ILNVPQELYEK	1	38679960	2xTMT6plex [N-Term; K11]			100	1.323879242		0	1804.062057	2	902.5336914	-1.081363855	-0.000975424	72.71784749

Bhat et al,2017. Cigarette smoke and chewing tobacco alter expression of different sets of miRNAs in oral keratinocytes

Supplementary Table S2(A). List of all proteins and peptides identified in OKF6/TERT1 cells chronically treated with chewing tobacco using Sequest and Mascot search algorithms

Accession	Gene_Symbol	Description	Gene_ID	Refseq_Accession	Coverage	Proteins	#Unique_Peptides	#Peptides	#PSMS	#AAs	MW [kDa]	calc. pI	OKF6-STE-6M/OKF6P_R1	OKF6-STE-6M/OKF6P_R2	OKF6-STE-6M/OKF6P_R3	Median fold change	Status	P-value (6M)
21361337	EIF5	eukaryotic translation initiation factor 5	1983	NP_001960.2	23.20185615	1	8	8	30	431	49.192	5.58	1.75	1.8	1.6	1.71	Significant	0.0012
23510448	MCM5	DNA replication licensing factor MCM5	4174	NP_006730.2	12.94277929	1	6	6	25	734	82.233	8.37	1.38	2.26	2.48	2.04	Significant	0.026
13129100	DCTPP1	dCTP pyrophosphatase 1	79077	NP_077001.1	43.52941176	1	6	6	26	170	18.669	5.03	0.736	2.953	1.881	1.86	Non-Significant	0.1
40018633	IDI1	isopentenyl-diphosphate Delta-isomerase 1 isoform [illegible]	3422	NP_004499.2	26.05633803	1	5	5	27	284	32.465	7.88	1.38	1.86	1.92	1.72	Significant	0.0097
4507813	UGDH	UDP-glucose 6-dehydrogenase isoform 1	7358	NP_003350.1	13.76518219	1	5	5	13	494	54.989	7.12	1.88	2.43	2.52	2.27	Significant	0.0076
19923206	GLUL	glutamine synthetase	2752	NP_002056.2	14.20911528	1	4	4	20	373	42.037	6.89	1.77	1.36	1.55	1.56	Significant	0.0057
22907049	ALDH3A1	aldehyde dehydrogenase, dimeric NADP-preferring	218	NP_000682.3	4.635761589	1	2	2	10	453	50.363	6.54	1.963	4.875	10.674	5.84	Non-Significant	0.15
297374823	INS	insulin preproprotein	3630	NP_001172027.1	25.45454545	1	2	2	83	110	11.973	5.3	7.56	8.68	10.24	8.83	Significant	0.0077
34222121	NUP37	nucleoporin Nup37	79023	NP_076962.2	4.601226994	1	1	1	6	326	36.684	5.92	2.49	3.09	2.17	2.58	Significant	0.011
333440449	CLASP2	CLIP-associating protein 2 isoform 1	23122	NP_055912.2	1.188118812	1	1	1	6	1515	165.752	8.31	2.31	2.45	2.63	2.46	Significant	0.0014
157266317	ATR	serine/threonine-protein kinase ATR	545	NP_001175.2	0.378214826	1	1	1	6	2644	301.172	7.43	2.22	2.47	2.21	2.3	Significant	0.0014
21614544	S100A8	protein S100-A8 isoform d	6279	NP_002955.2	63.44086022	1	6	6	53	93	10.828	7.03	0.072	0.039	0.033	0.05	Non-Significant	0.06
4506773	S100A9	protein S100-A9	6280	NP_002956.1	70.1754386	1	7	7	64	114	13.234	6.13	0.035	0.036	0.037	0.04	Non-Significant	1
295849266	DBI	acyl-CoA-binding protein isoform 4	1622	NP_001171488.1	18.91891892	1	2	2	16	148	16.484	9.38	1.644	6.984	5.73	4.79	Non-Significant	0.097
333033787	NACA	nascent polypeptide-associated complex subunit alpha isoform a	4666	NP_001106674.2	5.945945946	1	4	4	20	925	94.622	5.14	2.876	3.953	0.784	2.54	Non-Significant	0.11
164607124	FANCI	Fanconi anemia group I protein isoform 1	55215	NP_001106849.1	1.430722892	1	2	2	8	1328	149.229	6.74	3.14	2.75	2.66	2.85	Significant	0.0027
148298677	HMGCS1	hydroxymethylglutaryl-CoA synthase, cytoplasmic	3157	NP_001091742.1	31.34615385	1	13	13	64	520	57.257	5.41	2.8	3.28	3.13	3.07	Significant	0.0021
5453790	NNMT	nicotinamide N-methyltransferase	4837	NP_006160.1	30.68181818	1	5	5	21	264	29.555	5.74	2.2	3.06	3.26	2.84	Significant	0.013
7427519	MCM6	DNA replication licensing factor MCM6	4175	NP_005906.2	15.71254568	1	13	13	47	821	92.831	5.41	2.1	1.65	2.33	2.03	Significant	0.0096
50659095	DDX21	nucleolar RNA helicase 2 isoform 1	9188	NP_004719.2	15.70881226	1	11	12	42	783	87.29	9.28	1.08	1.89	2.36	1.78	Significant	0.042
33946297	ZNF326	DBIRD complex subunit ZNF326 isoform 1	284695	NP_892021.1	3.780068729	1	2	2	8	582	65.613	5.15	2.06	2.41	1.81	2.09	Significant	0.0068
338968930	CSDE1	cold shock domain-containing protein E1 isoform 4	7812	NP_001229820.1	16.46919431	1	14	14	47	844	93.683	6.52	1.61	1.89	1.73	1.74	Significant	0.0022
33469968	MCM7	DNA replication licensing factor MCM7 isoform 1	4176	NP_005907.3	21.418637	1	10	10	38	719	81.257	6.46	2.36	3.51	2.43	2.77	Significant	0.018
33469919	MCM4	DNA replication licensing factor MCM4	4173	NP_005905.2	10.77636153	1	8	8	32	863	96.498	6.74	2.11	2.61	2.82	2.52	Significant	0.0069
4506693	RPS17	40S ribosomal protein S17	6218	NP_001012.1	56.2962963	1	6	6	41	135	15.54	9.85	2.05	1.67	3.44	2.38	Significant	0.047
42558250	CAPRIN1	caprin-1 isoform 1	4076	NP_005889.3	18.47672779	1	9	9	36	709	78.318	5.25	2.04	2.05	2.13	2.07	Significant	0.00019
71979932	SLC7A5	large neutral amino acids transporter small subunit [illegible]	8140	NP_003477.4	9.467455621	1	4	4	26	507	54.974	7.72	1.55	1.44	1.61	1.53	Significant	0.0011
21359945	NCAPG	condensin complex subunit 3	64151	NP_071741.2	4.039408867	1	3	3	15	1015	114.262	5.59	2.49	2.61	2.55	2.55	Significant	0.00018
18104959	TXNDC9	thioredoxin domain-containing protein 9	10190	NP_005774.2	25.22123894	1	4	4	14	226	26.517	5.88	1.77	1.79	1.59	1.72	Significant	0.0014
119393891	GAA	lysosomal alpha-glucosidase preproprotein	2548	NP_000143.2	5.462184874	1	4	4	14	952	105.257	6	0.43	0.29	0.31	0.34	Significant	0.016

Sequence	#PSMs	Protein Accessions	Modifications	OKF6-STE-6M/OKF6P_R1	OKF6-STE-6M/OKF6P_R2	OKF6-STE-6M/OKF6P_R3	Xcorr	Ionscore	# Missed Cleavages	Theo. MH+ [Da]	Charge	m_z	DeltaM [ppm]	Deltam/z [Da]	RT [min]
EMYDADLLEEEVIISWSEK	2	21361337	2xTMT6plex [N-Term; K19]			1.628	3.690410376	30.78	0	2757.394485	3	919.802002	-1.099799336	-0.00101086	107.3832351
TVIVNMVDVAK	4	21361337	2xTMT6plex [N-Term; K11]	1.572			3.295612335	37.08	0	1646.991535	2	823.9989014	-0.612398791	-0.000504308	72.35849601
FVLCPECENPETDLHVNPK	4	21361337	2xTMT6plex [N-Term; K19]; 2xCarbamidomethyl [C4; C7				1.828712106	10.02	0	2756.378793	3	919.4644775	0.031326314	2.88E-05	62.442939
LQDMLDGFIK	6	21361337	2xTMT6plex [N-Term; K10]	100		100	1.844712853	22.19	0	1637.933686	2	819.4710693	0.718302509	0.000588266	98.74941642
EIVAEAER	4	21361337	1xTMT6plex [N-Term]	1.723	1.786		3.296902418	46.97	0	1145.636369	2	573.3223267	0.879956625	0.000504055	34.85197055
SVSDQFYR	2	21361337	1xTMT6plex [N-Term]			1.542	3.703877926	38.27	0	1230.631618	2	615.819458	0.017743429	1.09E-05	43.55282495
VLTLSDDLER	6	21361337	1xTMT6plex [N-Term]	100	100	100	2.655123949	19.57	0	1389.778676	2	695.3917847	-1.714570891	-0.001191437	56.89273664
AMGPLVLTEVLFNEK	2	21361337	2xTMT6plex [N-Term; K15]				1.812752247	6.14	0	2119.223719	2	1060.114868	-0.594360031	-0.000629791	110.9104932
GSSAAGLTASVMR	8	23510448	1xTMT6plex [N-Term]	3.455	1.807	3.161	5.394622326	86.29	0	1436.772879	2	718.8909302	1.186695258	0.000852506	44.4303951
LFQVSTLDAALSGTLSGVEGFTSQEDQEMLSR	2	23510448	1xTMT6plex [N-Term]			100	4.805411816	25.59	0	3645.815537	3	1215.947021	3.010147665	0.003658148	111.7408563
SFAGAVSPQEEEEFRR	5	23510448	1xTMT6plex [N-Term]	0.884	1.496	1.473	4.299563885	17.39	1	2068.029698	3	690.0143433	-0.590646665	-0.000407158	44.93183625
LSAEAAEK	4	23510448	2xTMT6plex [N-Term; K8]	2.427	2.836		3.41925168	48.53	0	1276.751288	2	638.8792114	-0.110889598	-7.08E-05	40.76003258
LAALPNVYEVISK	5	23510448	2xTMT6plex [N-Term; K13]			100	1.40307796	5.51	0	1875.135556	2	938.0713501	-0.070786716	-6.64E-05	85.42853176
SIAPSIFGGTDMK	1	23510448	2xTMT6plex [N-Term; K13]				0.806063235		0	1781.987178	2	891.4971924	-0.038953353	-3.47E-05	79.37419552
AALQEELSDVLIYLVALAAR	7	13129100	1xTMT6plex [N-Term]		100	0.932	3.729952812	30.5	0	2387.374728	3	796.4630737	-0.024972449	-1.99E-05	113.5569512
LHAEFAAER	4	13129100	1xTMT6plex [N-Term]		1.989	2.093	3.349271774	52.3	0	1272.689801	2	636.8477783	-1.195200412	-0.00076056	35.78674084
VDLPLAVLSK	6	13129100	2xTMT6plex [N-Term; K10]	2.803	3.337	1.999	3.355568409	55.06	0	1512.976537	2	756.9909668	-1.242369729	-0.000939838	83.14749785
TDGEPGPQGWSPR	3	13129100	1xTMT6plex [N-Term]				3.102749825	35.05	0	1612.7917	2	806.8991699	-0.394716968	-0.000318298	44.98758505
DWEQFHQPR	4	13129100	1xTMT6plex [N-Term]	0.726			3.644916773	23.09	0	1471.727977	3	491.2479248	0.845043433	0.000414558	42.56259565
GDTGGEDTAAPGR	2	13129100	1xTMT6plex [N-Term]				1.815618277	20.33	0	1432.686566	2	716.8469238	0.00347337	2.49E-06	18.27791666
KNVTLNPDPNEIK	6	40018633	3xTMT6plex [N-Term; K1; K13]	2.051	1.992	1.933	5.703281403	35.58	1	2169.284629	3	723.765625	-1.063636357	-0.00076911	40.53211443
AQSDGIWGEHEIDYILLVR	10	40018633	1xTMT6plex [N-Term]	100			3.226115704	14.23	0	2443.28189	3	815.0985718	-0.297844457	-0.000242573	86.91187087
NCHLNENIEK	6	40018633	2xTMT6plex [N-Term; K10]; 1xCarbamidomethyl [C2	1.111	1.463	1.899	4.330788136	24.95	0	1728.910325	3	576.9694214	-9.609306861	-0.005537877	35.52255996
AFSVFLFNTENK	2	40018633	2xTMT6plex [N-Term; K12]	100			2.206989527	28.15	0	1875.041656	2	938.0239868	-0.511421805	-0.000479469	99.93534804
AELGIPLEEVPPEEINYLTR	3	40018633	1xTMT6plex [N-Term]	100			1.347972274	3.94	0	2511.354386	2	1256.178345	-1.980256909	-0.002486563	89.37143075
EQIVVDLSHPGVSEDDQVSR	2	4507813	1xTMT6plex [N-Term]		100		2.336812258	27.14	0	2438.236062	3	813.4172974	0.523792533	0.00042571	51.84340901
VLIGGDETPEGQR	2	4507813	1xTMT6plex [N-Term]	1.707			3.674731255	39.24	0	1599.853966	2	800.4324951	2.342666553	0.001873962	45.26694225
ESSSIYISK	6	4507813	2xTMT6plex [N-Term; K9]	2.169	2.412	2.517	3.671332598	46.02	0	1471.840831	2	736.4238892	-0.223733219	-0.00016465	41.98469046
DVLNLVYLCEALNLPEVAR	2	4507813	1xTMT6plex [N-Term]; 1xCarbamidomethyl [C9				3.435233355	40.43	0	2430.326397	3	810.7870483	8.309291111	0.00673143	110.8525077
YIEACAR	1	4507813	1xTMT6plex [N-Term]; 1xCarbamidomethyl [C5	1.878			2.483547449	28.67	0	1111.576746	2	556.2923584	0.624839336	0.000347278	41.88965224
LTGFHETSNINDFSAGVANR	6	19923206	1xTMT6plex [N-Term]	2.19	1.387		5.186774731	48.24	0	2379.189052	3	793.7349243	0.490780343	0.00038922	65.3934669
RPSANCDPFSVTEALIR	4	19923206	1xTMT6plex [N-Term]; 1xCarbamidomethyl [C6			100	3.601286411	15.49	0	2162.122553	3	721.3783569	-0.941338508	-0.00067843	63.7230371
ACLYAGVK	8	19923206	2xTMT6plex [N-Term; K8]; 1xCarbamidomethyl [C2	1.491	1.35	1.527	3.736909389	39.16	0	1339.780814	2	670.3939209	-0.185846164	-0.000124497	48.71019208
LVLCEVFK	2	19923206	2xTMT6plex [N-Term; K8]; 1xCarbamidomethyl [C4			100	1.827521682	19.72	0	1465.885279	2	733.4455566	-0.984066605	-0.000721264	77.22174434
VAYGGTGDAATR	6	22907049	1xTMT6plex [N-Term]	2.551	9.931	10.674	4.007722378	70.85	0	1367.711659	2	684.3577271	-2.545279493	-0.001740604	29.97990584
IQQLEALQR	4	22907049	1xTMT6plex [N-Term]	1.16	1.338		3.24824667	32.15	0	1327.789515	2	664.397644	-1.132456647	-0.000751832	63.56740114
GIVEQCCTSICSLYQLENYCN	61	297374823	1xTMT6plex [N-Term]; 4xCarbamidomethyl [C6; C7; C11; C20]	100	100	100	2.485904217	30.66	0	2840.256088	3	947.4226074	-0.992542323	-0.000939691	85.22397177
GFFYTPK	22	297374823	2xTMT6plex [N-Term; K7]	6.697	6.968	7.185	2.665607691	25.02	0	1317.76073	2	659.3840942	0.137715868	9.07E-05	62.26549753
EGQEIASVSDDHTCR	6	34222121	1xTMT6plex [N-Term]; 1xCarbamidomethyl [C14	2.49	3.092	2.165	5.270107269	66.31	0	1932.891884	3	644.9686279	-0.286457273	-0.000184564	30.87211564
AQTGSGGADPTTDVSGQS	6	333440449	1xTMT6plex [N-Term]	2.314	2.447	2.632	3.834070683	68.52	0	1864.872194	2	932.9400024	0.28632676	0.000266981	27.94616533
EAYTHAQISR	6	157266317	1xTMT6plex [N-Term]	2.22	2.469	2.211	4.331077576	49.98	0	1404.743293	3	468.9198608	1.235876718	0.000578697	26.12158835
KLLETECPQYIR	6	21614544	2xTMT6plex [N-Term; K1]; 1xCarbamidomethyl [C7	0.042	0.04	0.035	5.050275326	39.57	1	2008.130154	3	670.0481567	-0.117843535	-7.89E-05	47.33119909
ALNSIIDVYHK	14	21614544	2xTMT6plex [N-Term; K11]	0.101	0.037	0.041	4.064512253	62.22	0	1731.020527	3	577.6782227	-0.237958617	-0.000137304	72.69919898
ELDINTDGAVNFQEFLILVIK	9	21614544	2xTMT6plex [N-Term; K21]		0.13		4.564929962	23.35	0	2849.606467	3	950.5397339	-0.63829159	-0.000606293	114.2084779
LLETECPQYIR	12	21614544	1xTMT6plex [N-Term]; 1xCarbamidomethyl [C6	0.042	0.027	0.025	3.621240377	38.69	0	1650.872259	2	825.9403687	0.728120956	0.000601017	59.1562186
GNFHAVYR	6	21614544	1xTMT6plex [N-Term]	0.037	0.035	0.034	3.065960646	37.1	0	1192.642457	2	596.8251343	0.448445118	0.000267417	34.56883561
MLTELEK	6	21614544	2xTMT6plex [N-Term; K7]	0.034	0.01	0.01	3.454520464	36.5	0	1321.780145	2	661.3938599	0.225443364	0.000148993	54.45927186
LGHPDTLNQGEFK	12	4506773	2xTMT6plex [N-Term; K13]	0.032	0.032	0.032	4.525735855	43.35	0	1914.048532	3	638.6876221	-0.114531611	-7.31E-05	46.6744597
NIETIINTFHQYSVK	12	4506773	2xTMT6plex [N-Term; K15]	0.289	0.01	0.01	3.689754248	32.37	0	2265.264338	3	755.7599487	0.421359834	0.000318164	92.50457593
VIEHIMEDLDTNADK	13	4506773	2xTMT6plex [N-Term; K15]	0.029	0.046	0.01	4.786539078	50	0	2201.152405	3	734.3885498	-0.594482206	-0.000436182	91.64900579
MHEGDEGPGHHHKPGLGEGTP	8	4506773	2xTMT6plex [N-Term; K13]	0.01	0.034	0.03	4.690697193	13.49	0	2634.288338	4	659.3274536	-0.134148897	-8.83E-05	29.41047213
LTWASHEK	4	4506773	2xTMT6plex [N-Term; K8]			0.04	3.690747023	42.25	0	1429.82037	2	715.4152832	2.041771337	0.001459683	45.73767463
VIEHIMEDLDTNADK	1	4506773	2xTMT6plex [N-Term; K15]; 1xOxidation [M6				1.231276035		0	2217.14732	3	734.3885498	-0.594482206	-0.000436182	91.64900579
KDLQNFLK	6	4506773	3xTMT6plex [N-Term; K1; K8]	0.022	0.01	0.044	3.655459642	18.58	1	1693.061553	3	565.0250244	-0.609976531	-0.000344243	53.96267557
MHEGDEGPGHHHKPGLGEGTP	2	4506773	2xTMT6plex [N-Term; K13]; 1xOxidation [M1		0.121		3.12444663	7.64	0	2650.283253	4	659.3274536	-0.134148897	-8.83E-05	29.41047213
DLQNFLK	6	4506773	2xTMT6plex [N-Term; K7]	0.01	0.029	0.138	3.291375875	33.47	0	1335.803658	2	668.4053345	-0.198707861	-0.000132717	74.53605522
QATVGDINTERPGMLDFTGK	6	295849266	2xTMT6plex [N-Term; K20]	100	100	100	2.607696295	19.07	0	2608.380507	3	870.1310425	-0.74099181	-0.000644263	57.79770531
WDAWNELK	10	295849266	2xTMT6plex [N-Term; K8]	1.437	5.107	4.382	3.250067711	33.5	0	1519.830935	2	760.4181519	-1.255395596	-0.000953995	70.30497919
IEDLSQQAQLAAAEK	4	333033787	2xTMT6plex [N-Term; K15]	100	2.25		3.366477489	45.1	0	2073.159205	2	1037.084106	0.835237651	0.00086579	76.71832176
DIELVMSQANVSR	6	333033787	1xTMT6plex [N-Term]	0.948	100	0.808	3.027848959	41.76	0	1690.899536	2	845.9523926	-1.198843401	-0.001013562	64.30122746
NILFVITKPDVYK	4	333033787	3xTMT6plex [N-Term; K8; K13]			100	2.588125467	26.67	0	2237.387638	3	746.4671021	-0.395433335	-0.000294913	91.81836484
SPASDTYIVFGEAK	6	333033787	2xTMT6plex [N-Term; K14]	100	100	0.01	2.953557253	26.12	0	1943.052614	2	972.0272217	-2.803696917	-0.002723865	60.53146425
EDADVSVTQR	6	164607124	1xTMT6plex [N-Term]	3.142	5.107	2.655	4.05390501	55.66	0	1348.690589	2	674.8481445	-1.168850374	-0.000788209	27.14945715
CLSQQADVR	2	164607124	1xTMT6plex [N-Term]; 1xCarbamidomethyl [C1		1.11		1.869978189	6.45	0	1305.67825	2	653.3421631	-0.919742766	-0.000600444	29.51467029
EDTHHLVNYIPQGSIDSLFEGTWYLVR	2	148298677	1xTMT6plex [N-Term]				4.589837074	24.3	0	3418.72692	4	855.4356689	-1.776839417	-0.001518632	103.8314749
VTQDATPGSALDK	8	148298677	2xTMT6plex [N-Term; K13]	2.764	3.333	4.012	4.700745106	63.29	0	1760.97945	2	880.9918823	-1.681758162	-0.001480771	40.84567574
DVGIVALEIYFPSQYVDQAELEK	6	148298677	2xTMT6plex [N-Term; K23]			100	3.990357161	39.99	0	3084.65454	3	1028.889648	-0.047816538	-4.92E-05	108.5767406
LEVGTETIIDK	10	148298677	2xTMT6plex [N-Term; K11]	2.274	5.151	1.097	3.314928532	45.08	0	1675.988223	2	838.4960938	-1.976457802	-0.00165626	60.32927955
ASSELFSQK	4	148298677	2xTMT6plex [N-Term; K9]	1.384	3.341		4.092757702	44.24	0	1454.825515	2	727.9163818	-0.019423721	-1.41E-05	53.14391985
YTIGLGQAK	8	148298677	2xTMT6plex [N-Term; K9]	3.203	2.376	2.53	4.232117653	57.9	0	1408.856422	2	704.9320679	0.31057259	0.000218776	55.41893321
ITASLCDLK	4	148298677	2xTMT6plex [N-Term; K9]; 1xCarbamidomethyl [C6	100	6.931		3.127009153	47.21	0	1478.865272	2	739.9354858	-1.066290718	-0.00078845	63.35790172
LEDTYFDRDVEK	2	148298677	2xTMT6plex [N-Term; K12]	3.748			3.431806326	22.39	1	1988.037693	3	663.3494873	-1.903372331	-0.001261325	64.48165593
MLLNDFLNDQNR	6	148298677	1xTMT6plex [N-Term]	3.784	100	100	2.963811874	21.34	0	1721.88422	2	861.4434814	-2.632987353	-0.00226685	79.06423476
DKNSIYSGLEAFGDVK	6	148298677	3xTMT6plex [N-Term; K2; K16]		100	100	2.77644062	18.85	1	2430.348351	3	810.7878418	0.255408088	0.00020691	75.79781531
NNLSYDCIGR	2	148298677	1xTMT6plex [N-Term]; 1xCarbamidomethyl [C7	4.392			3.314603567	20.28	0	1440.710279	2	720.8588257	0.066604083	4.80E-05	46.68474931
LSIQCYLSALDR	2	148298677	1xTMT6plex [N-Term]; 1xCarbamidomethyl [C5				0.909682691	6.73	0	1667.898808	2	834.4527588	-0.339817903	-0.000283391	90.02803871
LEDTYFDR	4	148298677	1xTMT6plex [N-Term]	4.004	3.14		2.529051304	20.69	0	1287.641848	2	644.3255615	1.552121717	0.000999288	55.18945524
GDLLIDIGSGPTIYQLLSACESFK	3	5453790	2xTMT6plex [N-Term; K24]; 1xCarbamidomethyl [C20		2.543		4.595474243	55.86	0	3055.642595	3	1019.218201	-0.833277826	-0.000848733	116.5930583
EIVVTDYSDQNLQELEK	6	5453790	2xTMT6plex [N-Term; K17]	100	100	100	2.886013746	22.1	0	2481.31247	3	827.7744751	-1.450106375	-0.001199389	73.97938094
SSYYMIGEQK	4	5453790	2xTMT6plex [N-Term; K10]	1.294	2.73		3.421806812	42.44	0	1663.876565	2	832.4412231	-0.838325971	-0.000697435	58.23538353
KEPEAFDWSPVVTYVCDLEGNR	2	5453790	2xTMT6plex [N-Term; K1]; 1xCarbamidomethyl [C16		1.307		4.130133629	17.48	1	3069.539193	3	1023.850647	-0.588007488	-0.000601637	101.8074242
EAVEAAVK	6	5453790	2xTMT6plex [N-Term; K8]	3.286	3.27	3.145	3.790809155	49.73	0	1274.772023	2	637.8897095	0.093393415	5.95E-05	36.90849475
KVEEEEDESALK	2	7427519	3xTMT6plex [N-Term; K1; K12]	1.976			4.475092411	22.38	1	2093.158091	3	698.3909912	0.157304321	0.000109754	41.76156817
AEAVESAQAGDK	6	7427519	2xTMT6plex [N-Term; K12]	2.752	2.974	2.313	4.931783199	58.14	0	1633.879736	2	817.4420776	-1.748553765	-0.001428463	32.91524683
ELRDEEQTAESIK	4	7427519	2xTMT6plex [N-Term; K13]	2.371	0.893		3.475808859	27.45	1	2006.08062	3	669.3651733	0.17284925	0.000115583	52.32615326
ASSAAGLTAAVVR	2	7427519	1xTMT6plex [N-Term]	1.212			3.691698313	29.19	0	1402.821543	2	701.9137573	-0.930589902	-0.000652726	51.01574905
VSGVDGYETEGIR	6	7427519	1xTMT6plex [N-Term]	1.491	0.828	2.419	3.631181002	28.93	0	1610.822331	2	805.914978	0.216184418	0.000174117	50.17971459
GDINVCIVGDPSTAK	5	7427519	2xTMT6plex [N-Term; K15]; 1xCarbamidomethyl [C6		100	100	2.473674297	29.17	0	2004.083597	2	1002.545837	0.399631377	0.000400447	57.35775885
KVEEEEDESALKR	3	7427519	3xTMT6plex [N-Term; K1; K12]	3.089	1.338		2.452818632	18.59	2	2249.259202	4	563.0701904	-0.12014678	-6.76E-05	43.76139928
DFYVAFQDLPTR	6	7427519	1xTMT6plex [N-Term]	100	1.459		3.243695021	39.29	0	1700.884538	2	850.9453125	-0.699165625	-0.0005946	86.41401953
DEEQTAESIK	2	7427519	2xTMT6plex [N-Term; K10]	0.661			1.710562825	15.77	0	1607.852852	2	804.4291992	-1.076157248	-0.000865151	42.59868013
VFEMSQDK	4	7427519	2xTMT6plex [N-Term; K8]	1.851		1.873	2.938107729	33.98	0	1441.776122	2	721.3914185	-0.389814985	-0.000281013	53.81019773
MHCCDEVQPK	4	7427519	2xTMT6plex [N-Term; K10]; 2xCarbamidomethyl [C3; C4	2.717	2.644		3.250257969	16.32	0	1761.848653	3	587.9539185	-0.823282354	-0.0004835	32.65261734
YLQLAEELIRPER	1	7427519	1xTMT6plex [N-Term]	100			1.653445005		0	1859.058813	3	620.357605	-0.296789208	-0.000183916	97.9803046
AVYTSGK	2	7427519	2xTMT6plex [N-Term; K7]		1.174		2.864680767	29.67	0	1183.708695	2	592.3587646	1.316098194	0.000778938	32.06470147
NEEPSEEEIDAPKPK	4	50659095	3xTMT6plex [N-Term; K13; K15]		2.412	2.159	4.848133564	35.63	0	2399.290896	3	800.4353027	0.191261979	0.000152964	47.43806731
EQLGEEIDSK	4	50659095	2xTMT6plex [N-Term; K10]	3.211	2.849		3.555958509	71.23	0	1605.873588	2	803.4415283	1.365263519	0.00109622	51.94643046
KKEEPSQNDISPK	2	50659095	4xTMT6plex [N-Term; K1; K2; K13]	1.344			3.612213373	7.36	2	2416.42174	4	604.8607788	-0.188239336	-0.000113716	37.21655493
ELKEQLGEEIDSK	2	50659095	3xTMT6plex [N-Term; K3; K13]	1.168			2.931504965	15.13	1	2205.258139	3	735.7567139	-1.156838917	-0.000850376	62.75828566
TAITVEHLAIK	4	50659095	2xTMT6plex [N-Term; K11]	1.483	100		2.853404284	24.97	0	1654.030363	3	552.0142822	-1.251265006	-0.000689877	48.75465904
GVTFLFPIQAK	6	50659095	2xTMT6plex [N-Term; K11]	100		100	2.438080788	30.65	0	1679.029635	2	840.0183105	-0.172930986	-0.000145178	82.15025229
AAVIGDVIR	2	50659095	1xTMT6plex [N-Term]		2.123		2.824432373	38.86	0	1142.709474	2	571.8581543	-0.38682295	-0.000221013	61.464406
LHGELQDR	6	50659095	1xTMT6plex [N-Term]	6.405	1.986	6.38	3.080016613	33.6	0	1196.658501	2	598.8329468	0.096865302	5.80E-05	27.91383077
STYEQVDLIGK	2	50659095	2xTMT6plex [N-Term; K11]				2.003777266	18.22	0	1710.967822	2	855.9863281	-1.427659812	-0.00122134	53.36974006
TFHHVYSGK	3	50659095	2xTMT6plex [N-Term; K9]	0.616	1.062		2.338843107	21.83	0	1533.857819	3	511.9570007	-0.892907229	-0.000456531	32.63679306
WQLSVATEQPELEGPR	5	50659095	1xTMT6plex [N-Term]				1.573842525	1.59	0	2069.086485	2	1035.045288	-1.539359591	-0.001592534	65.86689485
DLIAQAR	4	50659095	1xTMT6plex [N-Term]		1.684		2.697048903	27.47	0	1015.60976	2	508.3086243	0.208648208	0.000105953	36.72302685
STNVTVAAAR	6	33946297	1xTMT6plex [N-Term]	2.057	2.405	9.4	3.628771067	56.32	0	1218.700366	2	609.8539429	0.199657105	0.000121661	27.78290891
DYGPGSYGGMDR	2	33946297	1xTMT6plex [N-Term]			1.222	4.076613903	37.16	0	1503.673559	2	752.3408813	0.616812943	0.000463743	42.99789316
GDLETLQPGDDVEFTIK	6	338968930	2xTMT6plex [N-Term; K17]			100	2.207124472	23.04	0	2335.243328	2	1168.124146	-0.990780852	-0.001156857	71.08693164
EMGVIAAMR	6	338968930	1xTMT6plex [N-Term]	2.33	2.412	1.71	3.302084446	47.02	0	1206.653615	2	603.8306274	0.300718291	0.000181431	49.5886384
GPDNSMGFGAER	2	338968930	1xTMT6plex [N-Term]	1.286			3.748589039	20.04	0	1466.689543	2	733.8491211	0.969903622	0.000711274	43.53833833
IKQEILPEER	2	338968930	2xTMT6plex [N-Term; K2]			1.841	4.328694344	16.81	1	1713.031091	3	571.6817627	-0.208083825	-0.000118818	52.16050314
IKVDFVIPK	2	338968930	3xTMT6plex [N-Term; K2; K9]	1.02			2.485191584	2.28	1	1746.149639	3	582.7213745	-0.039609918	-2.31E-05	71.10880296
ELPFGDKDTK	4	338968930	3xTMT6plex [N-Term; K7; K10]		1.726	1.442	2.693139076	10.81	1	1837.067426	3	613.0274658	0.227738477	0.000139457	54.82151859
EATFSNPK	4	338968930	2xTMT6plex [N-Term; K8]		1.923	1.843	3.467378378	35.37	0	1351.762187	2	676.3847046	-0.040155223	-2.71E-05	32.7370539
LLGYVATLK	4	338968930	2xTMT6plex [N-Term; K9]		100		1.9931463	25.74	0	1435.928858	2	718.4675293	-0.749526165	-0.000538133	83.09337487
GTVSFHSHSDHR	2	338968930	1xTMT6plex [N-Term]			100	2.796033144	17.77	0	1595.787618	3	532.600769	0.085443016	4.54E-05	18.24705188
EVATDVR	6	338968930	1xTMT6plex [N-Term]	1.876	1.858	4.719	2.84692049	43.98	0	1018.57304	2	509.7899475	-0.414030661	-0.00021086	26.65025914
VDFVIPK	2	338968930	2xTMT6plex [N-Term; K7]	2.567			2.19379878	26.83	0	1275.807681	2	638.4069824	-0.777810218	-0.000496168	69.19569166
GEVYPFGIVGMANK	3	338968930	2xTMT6plex [N-Term; K14]	100			1.506099105	3.12	0	1940.071576	2	970.538174	-1.318783657	-0.001279267	82.49926373
DQFGFINYEVGDSK	2	338968930	2xTMT6plex [N-Term; K14]				1.390564919		0	2077.064242	2	1039.033447	-2.226131796	-0.002311909	67.73398125
EAFGFIER	2	338968930	1xTMT6plex [N-Term]	100			1.521680951	13.32	0	1197.646539	2	599.3268433	-0.108117511	-6.47E-05	54.71807981
SEDDESGAGELTR	8	33469968	1xTMT6plex [N-Term]	3.075	1.98	3.107	3.607594013	61.55	0	1594.739389	2	797.8729248	-0.511914756	-0.000408185	31.33691153
EQVALYVDLDDVAEDDPELVDSICENAR	2	33469968	1xTMT6plex [N-Term]; 1xCarbamidomethyl [C24			0.941	3.262412786	25.13	0	3421.61544	3	1141.206055	-3.457296036	-0.003943179	96.85255564
GSSGVGLTAAVLR	2	33469968	1xTMT6plex [N-Term]	1.237			2.981769562	56.33	0	1416.837194	2	708.921814	-0.59444396	-0.000421115	58.12130972
MQEHSDQVPVGNIPR	2	33469968	1xTMT6plex [N-Term]	2.488			5.50033617	42.4	0	1935.99081	3	646.0015869	-0.311312331	-0.000200899	45.10564919
GFTPAQFQAALDEYEELNVWQVNASR	4	33469968	1xTMT6plex [N-Term]			100	3.822478056	20.07	0	3212.585007	3	1071.53125	-1.808680544	-0.001936847	107.0540886
GNINICLMGDPGVAK	6	33469968	2xTMT6plex [N-Term; K15]; 1xCarbamidomethyl [C6			100	2.103937626	18.6	0	2017.097473	2	1009.050659	-1.701226971	-0.00171577	63.20529113
SLEQNIQLPAALLSR	4	33469968	1xTMT6plex [N-Term]				1.759968042	22.4	0	1882.095927	2	941.5504761	-1.196358554	-0.001125831	81.7431661
DVLDVYIEHR	6	33469968	1xTMT6plex [N-Term]	1.948	100	1.667	2.269869566	14.71	0	1487.805559	3	496.6068726	0.339678649	0.000168459	53.06281065
SQLLSYIDR	2	33469968	1xTMT6plex [N-Term]				1.150379658	13.7	0	1323.746982	2	662.3776855	0.840692191	0.000556432	52.15943326
QIAEEDFYEK	2	33469968	2xTMT6plex [N-Term; K10]		100		1.348615646	2.71	0	1729.904888	2	865.4556885	-0.455150385	-0.000393683	51.83944384
VEAIDVEEAK	2	33469919	2xTMT6plex [N-Term; K10]	3.077			4.587156773	64.16	0	1560.888509	2	780.9470215	-1.116710924	-0.000871531	59.5698519
GLQVDLQSDGAAAEDIVASEQSLGQK	12	33469919	2xTMT6plex [N-Term; K26]	2.488	2.472	100	4.916814804	64.47	0	3087.621007	3	1029.87793	-0.573793706	-0.000590553	88.20407557
ALADDDFLTVTGK	4	33469919	2xTMT6plex [N-Term; K13]	0.62	100		2.729050636	41.67	0	1824.015501	2	912.5109863	-0.441160587	-0.000402342	67.52252966
KEELAEALK	4	33469919	3xTMT6plex [N-Term; K1; K9]	1.987		2.47	3.895147562	32.22	1	1718.066698	3	573.3610229	1.058158952	0.000605996	54.52560054
GQSDIAITK	2	33469919	2xTMT6plex [N-Term; K9]	2.457			3.587803125	48.98	0	1390.830601	2	695.9185791	-0.517048499	-0.000359563	33.15352411
IAEPSVCGR	2	33469919	1xTMT6plex [N-Term]; 1xCarbamidomethyl [C7	0.955			2.325607538	18.97	0	1217.650973	2	609.3292236	0.162218591	9.88E-05	39.72584527
YQQLFEDIR	4	33469919	1xTMT6plex [N-Term]		100		1.427900314	13.98	0	1440.768445	2	720.8878174	-0.060491591	-4.36E-05	65.72147228
DMFEEALR	2	33469919	1xTMT6plex [N-Term]				1.367875099	14.42	0	1239.624089	2	620.3148804	-1.294931122	-0.000802614	55.67974672
LLDFGSLSNLQVTQPTVGMNFK	11	4506693	2xTMT6plex [N-Term; K22]	100	3.48	100	4.023074627	46.65	0	2867.574121	3	956.5281982	-1.422754309	-0.001359951	111.9426221
DNYVPEVSALDQEIIEVDPDTK	8	4506693	2xTMT6plex [N-Term; K22]	1.263	0.768		3.123928547	25.92	0	2947.518834	3	983.1779175	0.123928438	0.00012176	96.75027921
RDNYVPEVSALDQEIIEVDPDTK	2	4506693	2xTMT6plex [N-Term; K23]		1.013		3.025490284	17.94	1	3103.619945	3	1035.210693	-0.77910373	-0.000806014	86.94863292
IAGYVTHLMK	6	4506693	2xTMT6plex [N-Term; K10]		1.908	0.843	2.841341972	29.8	0	1590.944191	3	530.9855347	-1.345073011	-0.000713312	77.09932743
IAGYVTHLMK	4	4506693	2xTMT6plex [N-Term; K10]; 1xOxidation [M9			100	1.879015803	16.42	0	1606.939106	3	530.9855347	-1.345073011	-0.000713312	77.09932743
LLDFGSLSNLQVTQPTVGMNFK	4	4506693	2xTMT6plex [N-Term; K22]; 1xOxidation [M19				1.429592013	9.08	0	2883.569036	3	956.5281982	-1.422754309	-0.001359951	111.9426221
VCEEIAIIPSKK	4	4506693	3xTMT6plex [N-Term; K11; K12]; 1xCarbamidomethyl [C2			100	1.963754058	9.97	1	2074.254909	3	692.0889893	-1.202714551	-0.000831579	53.79973358
LGNDFHTNK	2	4506693	2xTMT6plex [N-Term; K9]	2.031			2.834270239	20.82	0	1503.831998	3	501.9496155	1.526437495	0.000765169	38.98135218
LNQDQLDAVSK	6	42558250	2xTMT6plex [N-Term; K11]	100	2.121	2.024	4.20672226	49.96	0	1688.95832	2	844.9821167	-0.8072855	-0.000681736	47.89729031
SSGPPPPSGSSGSEAAAGAGAAAPASQHPATGTGAVQTEAMK	2	42558250	2xTMT6plex [N-Term; K42]			2.546	5.897470474	25.28	0	4162.051133	4	1041.266846	-1.340708541	-0.001395024	38.38227537
LNEQYEHASIHLWDLLEGK	2	42558250	2xTMT6plex [N-Term; K19]	100			3.304224968	21.11	0	2753.466285	4	689.1217651	-0.383089174	-0.000263706	92.64375544
TVLELQYVLDK	6	42558250	2xTMT6plex [N-Term; K11]	100	100	100	2.465959787	29.22	0	1779.066808	2	890.0358887	-1.296941879	-0.001153673	83.89396043
EKPVCGTTYK	2	42558250	3xTMT6plex [N-Term; K2; K10]; 1xCarbamidomethyl [C5	1.79			2.804636478	10.79	0	1870.071131	3	624.0274048	-1.855669805	-0.001156745	39.14747651
QILGVIDK	6	42558250	2xTMT6plex [N-Term; K8]	1.844	100	1.706	3.569917917	37.58	0	1343.866258	2	672.4367065	-0.090436129	-6.08E-05	65.19839086
YQEVTNNLEFAK	6	42558250	2xTMT6plex [N-Term; K12]	100	100		2.005394697	16.63	0	1914.037299	2	957.5211792	-1.158285454	-0.001108501	63.30074166
SFMALSQDIQK	2	42558250	2xTMT6plex [N-Term; K11]				1.365861416	1.04	0	1725.960963	2	863.4835815	-0.623637548	-0.000538187	73.76373011
LDDYQER	4	42558250	1xTMT6plex [N-Term]	1.963	1.91		2.767605782	31.92	0	1167.584333	2	584.295105	-1.198773419	-0.000699835	40.08851628
ALAAPAAEEK	6	71979932	2xTMT6plex [N-Term; K10]	1.241	1.268	1.298	4.101687908	70.38	0	1428.846251	2	714.9260254	-1.033427319	-0.000738304	34.4844309
SADGSAPAGEGEGVTLQR	12	71979932	1xTMT6plex [N-Term]	2.534	1.943	2.282	5.05381918	81.8	0	1930.966763	2	965.9862671	-0.77983751	-0.00075292	28.82648887
ALAAPAAEEKEEAR	6	71979932	2xTMT6plex [N-Term; K10]	1.633	1.502	1.56	4.044431686	39.46	1	1914.069661	3	638.694397	-0.534897999	-0.000341277	41.10145182
GDVSNLDPNFSFEGTK	2	71979932	2xTMT6plex [N-Term; K16]				1.604696751	12.93	0	2185.117734	2	1093.053345	-8.38441636	-0.009160468	64.25915768
EFGDQAEAAQDATLTTTTFQNEDEK	3	21359945	2xTMT6plex [N-Term; K25]	100			3.058248281	28.9	0	3218.537731	3	1073.518311	0.822463754	0.000882377	68.22160188
EVEETATAK	8	21359945	2xTMT6plex [N-Term; K9]	2.457	2.521	2.549	4.253465176	58.71	0	1435.804445	2	718.3988037	-9.830501758	-0.007057339	35.91918369
VIEFAAK	4	21359945	2xTMT6plex [N-Term; K7]	2.498	100		2.773762941	32.71	0	1235.77638	2	618.3916016	-0.367327784	-0.000226967	62.26864344
TQDYVVGFTDLGNTDDFTTETLEWR	4	18104959	1xTMT6plex [N-Term]	100		1.556	4.169666767	25.71	0	3152.489769	3	1051.502075	0.603613676	0.000634295	96.34478996
VLEHQLLQTTK	2	18104959	2xTMT6plex [N-Term; K11]	1.773			3.626568794	22.35	0	1768.07329	3	590.0296021	0.544356588	0.000320821	47.63459398
LVEEHLDSEIQK	6	18104959	2xTMT6plex [N-Term; K12]	1.725	1.792	1.591	4.334706783	41.2	0	1898.063513	3	633.3590698	-0.451658934	-0.000285759	50.61233442
VIPTLALLK	2	18104959	2xTMT6plex [N-Term; K9]				1.315467	3.51	0	1425.980894	2	713.4942627	0.248916817	0.000177475	91.68990084
DFPAMVQELHQGGR	4	119393891	1xTMT6plex [N-Term]	0.459	0.268		3.435040712	41.13	0	1813.921668	3	605.3112793	-1.31397591	-0.000794483	58.60260108
AITQEQCEAR	6	119393891	1xTMT6plex [N-Term]; 1xCarbamidomethyl [C7	0.354	0.399	0.31	3.579988718	39.42	0	1434.720843	2	717.8632813	-1.085625826	-0.000778785	25.24827675
AHFPLDVQWNDLDYMDSR	2	119393891	1xTMT6plex [N-Term]		0.01		1.470610142	1.66	0	2451.16006	3	817.7253418	0.576054028	0.000470667	79.11477903
LDVMMETENR	2	119393891	1xTMT6plex [N-Term]	0.617			1.149990559	1.32	0	1466.718066	2	733.8635254	1.164641856	0.000854101	59.82753455

Bhat et al,2017. Cigarette smoke and chewing tobacco alter expression of different sets of miRNAs in oral keratinocytes

Supplementary Table S2(A). List of all proteins and peptides identified in OKF6/TERT1 cells chronically treated with chewing tobacco using Sequest and Mascot search algorithms

Accession	Gene_Symbol	Description	Gene_ID	Refseq_Accession	Coverage	Proteins	#Unique_Peptides	#Peptides	#PSMS	#AAs	MW [kDa]	calc. pI	OKF6-STE-6M/OKF6P_R1	OKF6-STE-6M/OKF6P_R2	OKF6-STE-6M/OKF6P_R3	Median fold change	Status	P-value (6M)	Sequence	#PSMSs	Protein Accessions	Modifications	OKF6-STE-6M/OKF6P_R1	OKF6-STE-6M/OKF6P_R2	OKF6-STE-6M/OKF6P_R3	Xcorr	Ionscore	# Missed Cleavages	Theo. MH+ [Da]	Charge	m_z	DeltaM [ppm]	Deltam/z [Da]	RT [min]
4505595	SERPINB2	plasminogen activator inhibitor 2	5055	NP_002566.1	27.46987952	1	12	12	49	415	46.566	5.63	0.23	0.21	0.24	0.23	Significant	0.0015																
																			GSYPDAILQAQAADK	10	4505595	2xTMT6plex [N-Term; K15]	0.052	0.01		3.270899057	54.03	0	2006.095876	2	1003.552185	0.606789138	0.000608639	69.00903439
																			SMGMEDAFNK	4	4505595	2xTMT6plex [N-Term; K10]	0.198	0.232		4.059146404	50.18	0	1587.79112	2	794.3994141	0.271512414	0.000215553	64.12538333
																			GSTEDQMAK	4	4505595	2xTMT6plex [N-Term; K9]	0.229	0.22		3.522076845	51.56	0	1424.74555	2	712.8764038	-0.013639497	-9.72E-06	28.7327361
																			MAEDEVEVYIPQFK	2	4505595	2xTMT6plex [N-Term; K14]	0.01			2.461602449	11.36	0	2156.134964	2	1078.569824	-1.202152251	-0.001296001	96.39490432
																			LNIGYIEDLK	2	4505595	2xTMT6plex [N-Term; K10]		0.01		2.168211699	25.29	0	1635.972179	2	818.4890747	-0.798721368	-0.000653343	76.74571114
																			TPVQMMYLR	4	4505595	1xTMT6plex [N-Term]	0.136	0.131		3.21236062	29.56	0	1367.737679	2	684.3729248	0.653385067	0.00044683	70.84387754
																			EKLNIGYIEDLK	4	4505595	3xTMT6plex [N-Term; K2; K12]		0.01	0.242	2.919597387	12.59	1	2122.272667	3	708.0939331	-2.554517936	-0.001807128	72.15083488
																			ITNCILFFGR	2	4505595	1xTMT6plex [N-Term]; 1xCarbamidomethyl [C4	0.01			2.029357433	34.44	0	1469.813622	2	735.4101563	-0.398581147	-0.00029292	84.87528225
																			LEEHYELR	8	4505595	1xTMT6plex [N-Term]	0.234	0.215		3.777849913	53.62	0	1317.700031	3	439.9052429	0.868239917	0.00038136	39.04191969
																			MVLVNAVYFK	4	4505595	2xTMT6plex [N-Term; K10]		0.01		1.564735055	7.78	0	1641.980242	2	821.4937134	-0.055848533	-4.59E-05	92.74232625
																			IPNLLPEGSVDGDTR	3	4505595	1xTMT6plex [N-Term]	0.311	0.01		2.694878817	19.35	0	1811.970058	2	906.4873657	-1.436637805	-0.001301572	72.31656172
																			SMGMEDAFNK	1	4505595	2xTMT6plex [N-Term; K10]; 1xOxidation [M2		0.199		1.338958025	20.43	0	1603.786035	2	794.3994141	0.271512414	0.000215553	64.12538333
																			GKIPNLLPEGSVDGDTR	1	4505595	2xTMT6plex [N-Term; K2]	0.184			2.251841545	7.97	1	2226.249417	3	742.7547607	0.140306185	0.000104119	53.66394134
7657532	S100A6	protein S100-A6	6277	NP_055439.1	35.55555556	1	4	4	34	90	10.173	5.48	0.41	0.41	0.44	0.42	Significant	0.00057																
																			LQDAEIAR	8	7657532	1xTMT6plex [N-Term]	0.409	0.417	1.124	3.237752438	41.39	0	1144.652353	2	572.8299561	0.24677307	0.000141235	37.82482256
																			ELKELIQK	2	7657532	3xTMT6plex [N-Term; K3; K8]		0.104		2.851980209	9.8	1	1688.092518	3	563.3691406	0.207426427	0.000116718	66.6514082
																			EGDKHTLSK	3	7657532	3xTMT6plex [N-Term; K4; K9]	0.62	0.453		2.13111949	8.86	1	1702.010245	3	568.0077515	-0.907295049	-0.000514742	26.97195183
																			ELTIGSK	21	7657532	2xTMT6plex [N-Term; K7]	0.4	0.407	0.442	3.091781616	33.83	0	1205.75056	2	603.3793945	0.790265028	0.000476431	50.72972255
13489087	SERPINB1	leukocyte elastase inhibitor	1992	NP_109591.1	31.13456464	1	11	11	59	379	42.715	6.28	0.27	0.3	0.24	0.27	Significant	0.0041																
																			TFHFNTVEEVHSR	14	13489087	1xTMT6plex [N-Term]	0.213	0.228	0.232	4.887193203	51.46	0	1831.928862	4	458.7373047	-0.804103276	-0.000368265	44.05672865
																			TYGADLASVDFQHASEDAR	4	13489087	1xTMT6plex [N-Term]	0.327			5.063435555	58.4	0	2282.088669	3	761.3674316	-0.406486451	-0.000309213	65.87546689
																			KIEEQLTLEK	13	13489087	3xTMT6plex [N-Term; K1; K10]	0.276	0.61	0.25	4.307276726	22.57	1	1918.18279	3	640.0647583	-1.599554366	-0.001022746	16.88316745
																			KFAYGYIEDLK	2	13489087	3xTMT6plex [N-Term; K1; K11]	0.181			3.648303747	21.6	1	2034.187875	3	678.7333374	-1.187861258	-0.000805444	81.83843889
																			ADLSGMSGAR	6	13489087	1xTMT6plex [N-Term]; 1xOxidation [M6]	0.303	0.475		1.877802968	40.55	0	1209.609502	2	597.3115234	0.991077868	0.000591482	35.82148411
																			KTINQWVK	2	13489087	3xTMT6plex [N-Term; K1; K8]	0.324			3.425412178	25.37	1	1704.077537	3	568.6966553	-1.246762334	-0.000708193	51.60604267
																			FQSLNADINKR	4	13489087	2xTMT6plex [N-Term; K10]	0.417	0.297		3.21404171	17.46	1	1764.016838	3	588.677063	-0.114786408	-6.75E-05	56.42722047
																			FAYGYIEDLK	4	13489087	2xTMT6plex [N-Term; K10]	0.297	0.229		2.071294785	22.43	0	1676.92998	2	838.96875	0.144937477	0.000121525	74.97820415
																			ADLSGMSGAR	2	13489087	1xTMT6plex [N-Term]		0.424		2.921236038	43.63	0	1193.614587	2	597.3115234	0.991077868	0.000591482	35.82148411
																			VLELPYQGEELSMVILLPDDIEDESTGLK	2	13489087	2xTMT6plex [N-Term; K29]	0.489			1.65687263	0.22	0	3703.964382	3	1235.324829	-1.200841815	-0.001482625	112.9981356
																			IEEQLTLEK	2	13489087	2xTMT6plex [N-Term; K9]				2.287117958	7.89	0	1560.924895	2	780.9659424	-0.183727209	-0.000143392	54.25374076
																			GASYILK	4	13489087	2xTMT6plex [N-Term; K7]	0.262	0.262		2.737127066	36.4	0	1209.76073	2	605.3840332	0.049105785	2.97E-05	58.69477571
4504937	KYNU	kynureninase isoform a	8942	NP_003928.1	5.376344086	1	2	2	8	465	52.318	7.03	0.205	0.095	0.112	0.14	Non-Significan	0.058																
																			KPVVNIITPSHVEER	6	4504937	2xTMT6plex [N-Term; K1]	0.157	0.095	0.112	4.774837494	35.34	0	2176.285408	4	544.8269043	0.174162589	9.48E-05	50.9697424
																			IAAYGHEVGK	2	4504937	2xTMT6plex [N-Term; K10]	0.334			3.106774569	15.25	0	1502.873134	3	501.6300354	1.609398103	0.00080624	42.42076614
20149498	FTL	ferritin light chain	2512	NP_000137.2	17.14285714	1	3	3	14	175	20.007	5.78	0.45	0.3	0.29	0.35	Significant	0.022																
																			KLNQALLDLHALGSAR	2	20149498	2xTMT6plex [N-Term; K1]	0.394			4.034005165	22.98	1	2178.312292	4	545.3335571	0.049072681	2.67E-05	70.80494474
																			ALFQDIK	5	20149498	2xTMT6plex [N-Term; K7]	0.437			3.047640562	33.09	0	1292.797844	2	646.9038696	2.025458059	0.001309254	64.53625318
																			AAMALEK	7	20149498	2xTMT6plex [N-Term; K7]	0.462	0.298	0.294	2.611181021	29.13	0	1191.717151	2	596.3563232	-9.885865631	-0.005890578	44.15823424
555943884	SLC2A14	solute carrier family 2, facilitated glucose transporter member 14 isoform c	144195	NP_001273166.1	2.056074766	1	1	1	8	535	58.211	7.23	0.29	0.26	0.26	0.27	Significant	0.0014																
																			AFEGQAHGADR	8	555943884	1xTMT6plex [N-Term]	0.285	0.26	0.259	4.144838333	40.86	0	1387.691592	3	463.235199	-0.395113853	-0.000182765	15.74335562
732170489	LAMA3	laminin subunit alpha-3 isoform 1 precursor	3909	NP_937762.2	13.29132913	1	32	32	148	3333	366.384	7.24	0.26	0.35	0.36	0.32	Significant	0.0095																
																			AASASESALQTVIK	6	732170489	2xTMT6plex [N-Term; K14]	0.01	0.01	0.23	3.463768721	69.88	0	1834.068599	2	917.5375977	-0.370873532	-0.000340104	56.9132442
																			GATSSKPETPGVYDMDGR	8	732170489	2xTMT6plex [N-Term; K6]	0.238	0.237	0.18	5.604372501	41.07	0	2326.174931	3	776.0628052	-0.459433485	-0.000356241	48.040779
																			GIYFSEEGGHVVLAHSVLLGPEFK	8	732170489	2xTMT6plex [N-Term; K24]	1.175	2.039	1.477	5.382157326	24.14	0	3043.665713	4	761.6724243	0.707671547	0.000538479	85.52225204
																			ANDITDEVLDGLNPIQTDVER	2	732170489	1xTMT6plex [N-Term]	0.18			2.979683876	32.18	0	2556.299056	3	852.7706299	-0.672617532	-0.000573137	90.60274425
																			ALTDADNSVNK	4	732170489	2xTMT6plex [N-Term; K11]	0.157	0.133		4.817403316	65.12	0	1605.884821	2	803.4459229	-0.156858619	-0.000125948	46.56616103
																			NLQVTLEDGYIELSTSDSGGPIFK	2	732170489	2xTMT6plex [N-Term; K24]	0.363			4.959383488	51.76	0	3041.608318	3	1014.54303	2.044476086	0.002072832	101.2676233
																			DYIGMAVVDGQLTCVYNLGDR	6	732170489	1xTMT6plex [N-Term]; 1xCarbamidomethyl [C14	0.223	0.01	0.01	4.413581848	44.06	0	2588.268625	3	863.4298096	2.41510517	0.002083647	92.01630019
																			VPTQPHAPIPTFGQTIQTTVDR	8	732170489	1xTMT6plex [N-Term]	0.227	0.252	0.185	5.082307339	25.41	0	2633.424866	3	878.4796753	-0.149201558	-0.00013097	65.04877868
																			YLTTADSSLLQTNIALQLMEK	2	732170489	2xTMT6plex [N-Term; K21]			0.169	4.337684631	27.47	0	2811.557803	3	937.8581543	0.749445956	0.00070237	105.755683
																			SQFAVDMQTTSSR	2	732170489	1xTMT6plex [N-Term]		1.012		2.992800236	40.85	0	1686.83185	2	843.9195557	-0.009077298	-7.66E-06	41.88434807
																			GCIELDDLNENVLSLYNFK	10	732170489	2xTMT6plex [N-Term; K19]; 1xCarbamidomethyl [C2	0.01	0.219	0.01	3.379558563	30.73	0	2714.411139	3	905.4741211	-1.226283927	-0.001109546	111.3099609
																			EAELQVDQILTK	6	732170489	2xTMT6plex [N-Term; K12]	0.215	0.01	0.01	3.402783632	50.08	0	1845.07335	2	923.0418091	1.621406576	0.001495807	73.2715486
																			TSLVEEAEK	2	732170489	2xTMT6plex [N-Term; K9]	0.2			4.267947674	47.57	0	1463.835746	2	732.4216919	0.247001116	0.000180785	52.72722184
																			KEESDKNYFEGTGYAR	8	732170489	3xTMT6plex [N-Term; K1; K6]	0.299	0.34	0.192	4.868306637	14.46	2	2581.350142	3	861.1218262	0.303339563	0.000261009	39.48968677
																			GATSSKPETPGVYDMDGR	6	732170489	2xTMT6plex [N-Term; K6]; 1xOxidation [M15]	0.338	0.472	0.221	3.773169994	32.19	0	2342.169846	3	776.0628052	-0.459433485	-0.000356241	48.040779
																			FISLNIEDGK	7	732170489	2xTMT6plex [N-Term; K10]	0.183	0.303		2.610595703	38.07	0	1593.925229	2	797.4656372	-0.77262466	-0.000615753	81.08535021
																			LAASLNEAR	6	732170489	1xTMT6plex [N-Term]	0.184	0.176	0.17	3.893328905	54.33	0	1173.678902	2	587.3428955	-0.330341096	-0.000193857	40.56477071
																			GDIDAMISSAK	8	732170489	2xTMT6plex [N-Term; K11]; 1xOxidation [M6	0.18	0.162	0.199	2.747499943	38.97	0	1581.855829	2	791.4315796	0.033555326	2.65E-05	43.18698314
																			GDIDAMISSAK	6	732170489	2xTMT6plex [N-Term; K11]	0.287	0.01	0.01	2.529931545	37.83	0	1565.860914	2	791.4315796	0.033555326	2.65E-05	43.18698314
																			LPQELLKPR	3	732170489	2xTMT6plex [N-Term; K7]	0.836		0.936	3.087763548	8.67	0	1551.998669	3	518.0040894	-0.61486109	-0.000318088	56.15016601
																			GLVFHTGTK	4	732170489	2xTMT6plex [N-Term; K9]		1.011	0.97	3.241921186	29.9	0	1417.856756	3	473.2904968	0.127860953	6.04E-05	41.60116762
																			LLIDDQLLR	5	732170489	1xTMT6plex [N-Term]	0.821	0.464	0.01	3.163733006	31.97	0	1327.814667	2	664.4108276	-0.217347021	-0.000144298	76.85577358
																			LPNDLEDLK	4	732170489	2xTMT6plex [N-Term; K9]	0.239	0.254	0.19	2.642232418	49.67	0	1514.88303	2	757.9452515	0.129488339	9.81E-05	66.90359107
																			EINSLQSDFTK	5	732170489	2xTMT6plex [N-Term; K11]	0.01	0.01		1.707135081	16	0	1739.957986	2	870.4814453	-1.363179467	-0.001185937	54.10914302
																			LSLSPEVLDLTSNSLK	2	732170489	2xTMT6plex [N-Term; K16]				1.310752153		0	2174.268421	2	1087.637573	-0.25336137	-0.000275438	91.29704206
																			AASASESALQTVIKEDLPR	2	732170489	2xTMT6plex [N-Term; K14]				2.501561403	4.08	1	2444.376074	3	815.4632568	-0.350315239	-0.000285434	71.91472313
																			KANDITDEVLDGLNPIQTDVER	4	732170489	2xTMT6plex [N-Term; K1]	0.188			1.987914562	8.62	1	2913.556951	3	971.855896	-1.309750172	-0.001272011	84.21081954
																			SPQTYMDGLLHYVSVISDNSGLR	2	732170489	1xTMT6plex [N-Term]				2.482913256	19.81	0	2781.407895	3	927.8054199	-2.224860434	-0.002062748	102.5278764
																			SAISNHGSK	2	732170489	2xTMT6plex [N-Term; K9]	0.529			2.01964879	14.41	0	1358.779234	3	453.5986023	1.486455426	0.000673255	28.3266782
																			LSLSPEVLDLTSNSLKR	2	732170489	2xTMT6plex [N-Term; K16]			0.01	1.423145652	1.94	1	2330.369532	3	777.4606323	-0.938897164	-0.000729326	78.86998589
																			SLQELAK	2	732170489	2xTMT6plex [N-Term; K7]	0.189			2.907670498	36.82	0	1246.777109	2	623.8931274	1.499540535	0.000934796	48.90476439
																			GGQLSFTDLGLPPTDHLQASFGFQTFQPSGILLDHQTWTR	1	732170489	1xTMT6plex [N-Term]				1.444711804		0	4643.352041	4	1161.592651	-0.703147472	-0.00081624	100.7669551
																			SLPTNSFVGCLK	2	732170489	2xTMT6plex [N-Term; K12]; 1xCarbamidomethyl [C10	100	100		1.578542233		0	1781.003162	2	891.0059814	0.855681033	0.000761985	69.03781417
																			LNSELPK	1	732170489	2xTMT6plex [N-Term; K7]	0.488			2.782742739		0	1258.777109	2	629.8916626	-0.84216235	-0.000530047	47.7085756
91199540	DLD	dihydrolipoyl dehydrogenase, mitochondrial isoform 1 precursor	1738	NP_000099.2	34.7740668	1	11	11	50	509	54.143	7.85	0.62	0.59	0.45	0.55	Significant	0.0088																
																			IPNIYAIGDVVAGPMLAHK	4	91199540	2xTMT6plex [N-Term; K19]	0.583	0.855		4.050297737	30.49	0	2437.404143	3	813.1393433	-0.273493768	-0.000222205	101.5508554
																			VLGAHILGPGAGEMVNEAALALEYGASCEDIAR	6	91199540	1xTMT6plex [N-Term]; 1xCarbamidomethyl [C28	0.63			6.485852718	47.87	0	3583.808618	4	896.7076416	0.033042013	2.96E-05	108.0885952
																			NILIATGSEVTPFPGITIDEDTIVSSTGALSLK	6	91199540	2xTMT6plex [N-Term; K33]			0.702	5.08065033	38.28	0	3818.109072	4	955.28302	0.308516101	0.000294487	114.4765632
																			ALLNNSHYYHMAHGK	2	91199540	2xTMT6plex [N-Term; K15]		0.67		4.952521801	25.61	0	2214.164247	4	554.2958374	-1.231900763	-0.000681908	39.64070841
																			VCHAHPTLSEAFR	3	91199540	1xTMT6plex [N-Term]; 1xCarbamidomethyl [C2	0.768		0.68	4.012357712	31.85	0	1753.900539	3	585.3051758	0.248026465	0.000145005	39.17258309
																			IDVSIEAASGGK	8	91199540	2xTMT6plex [N-Term; K12]		1.419		2.803821325	44.59	0	1604.925958	2	802.9661255	-0.612597381	-0.000491587	62.04990948
																			EANLAASFGK	2	91199540	2xTMT6plex [N-Term; K10]		0.594		3.155176163	40.52	0	1465.8415	2	733.4246826	0.401731275	0.000294437	51.29659021
																			NLGLEELGIELDPR	5	91199540	1xTMT6plex [N-Term]				2.091647863	9.84	0	1796.995544	2	899.0009155	-0.550933649	-0.000495013	97.15495899
																			SEEQLKEEGIEYK	5	91199540	3xTMT6plex [N-Term; K6; K13]	0.72	1.005		3.7698493	26.89	1	2269.253054	3	757.0901489	1.251269592	0.000946482	46.84630519
																			GIEMSEVR	6	91199540	1xTMT6plex [N-Term]	0.438	0.449	0.402	3.142418385	31.66	0	1149.613525	2	575.3105469	0.254381141	0.00014622	46.28844305
																			GIEMSEVR	1	91199540	1xTMT6plex [N-Term]; 1xOxidation [M4]		1.409		1.491668463	22.13	0	1165.60844	2	575.3105469	0.254381141	0.00014622	46.28844305
																			AAQLGFK	2	91199540	2xTMT6plex [N-Term; K7]	0.734			2.311263084	20.14	0	1192.745415	2	596.8772583	1.530327881	0.000912646	48.65662923
218931251	DAGLB	sn1-specific diacylglycerol lipase beta isoform 1	221955	NP_631918.3	2.976190476	1	2	2	8	672	73.684	6.55	0.027	0.25	1.109	0.46	Non-Significan	0.29																
																			IIHLQEEGASGR	4	218931251	1xTMT6plex [N-Term]	0.027	0.196		3.186491728	13.52	0	1538.848821	3	513.6209106	-0.417270623	-0.000214039	38.25673604
																			ESVVVAVR	4	218931251	1xTMT6plex [N-Term]		1.491	1.109	3.088670969	28.14	0	1087.667275	2	544.3319702	-9.755777854	-0.00530552	39.11615448
4507475	TGM1	protein-glutamine gamma-glutamyltransferase K	7051	NP_000350.1	2.203182375	1	1	1	6	817	89.73	6.04	0.03	0.02	0.03	0.03	Significant	0.015																
																			AVETAAAHGSKPNVYANR	6	4507475	2xTMT6plex [N-Term; K11]	0.026	0.022	0.033	5.460584164	28.86	0	2314.266798	4	579.3218384	-0.550664416	-0.000318596	33.44199218
669033275	TPM2	tropomyosin beta chain isoform Tpm2.3	7169	NP_001288155.1	46.12676056	1	1	14	114	284	32.795	4.68	2.43	2.06	1.9	2.13	Significant	0.0054																
																			KATDAEADVASLNR	12	669033275	2xTMT6plex [N-Term; K1]	1.851	1.897	1.721	4.062987804	55.99	1	1919.059825	3	640.3546753	-5.39453797	-0.003450814	40.68041543
																			ATDAEADVASLNR	8	669033275	1xTMT6plex [N-Term]	3.743	2.361	2.468	4.139335155	51.47	0	1561.80193	2	781.4029541	-2.112000767	-0.001649263	41.60521378
																			QLEEEQQALQK	2	669033275	2xTMT6plex [N-Term; K11]	2.76			4.280395985	38.33	0	1802.005999	2	901.505188	-1.608931069	-0.001449652	52.60908437
																			TIDDLEETLASAK	6	669033275	2xTMT6plex [N-Term; K13]		100		2.269931555	28.7	0	1864.031545	2	932.5187378	-0.721915928	-0.000672837	78.34970863
																			IQLVEEELDR	18	669033275	1xTMT6plex [N-Term]	1.297	2.38	1.828	3.785353422	49.84	0	1472.815789	2	736.911499	-0.046111079	-3.40E-05	68.55083794
																			KLVILEGELER	15	669033275	2xTMT6plex [N-Term; K1]	0.853	0.956	0.684	4.428694725	31.82	1	1757.093692	3	586.3692017	-0.364123743	-0.000213267	59.71334727
																			MEIQEMQLK	20	669033275	2xTMT6plex [N-Term; K9]	1.324	0.962		3.55449605	46.32	0	1607.890106	2	804.4488525	0.200447856	0.000161149	72.61134951
																			AEQAEADK	6	669033275	2xTMT6plex [N-Term; K8]	1.855	1.353	2.024	3.345167875	46.74	0	1319.720716	2	660.3638306	-0.251119183	-0.000165704	26.71381538
																			EENVEIHQTLDQTLLELNNL	4	669033275	1xTMT6plex [N-Term]				2.305280685	6.62	0	2594.351091	3	865.4542847	-1.075658612	-0.000930212	99.08640446
																			SLEAQADK	4	669033275	2xTMT6plex [N-Term; K8]	2.592	2.328		3.287427664	47.33	0	1319.757101	2	660.3823242	0.204869138	0.000135189	39.69798513
																			AEQAEADKK	10	669033275	3xTMT6plex [N-Term; K8; K9]	1.354	1.532	1.56	2.406741858	16.76	1	1676.978611	3	559.6641235	-0.473187032	-0.000264508	34.59182199
																			GTEDEVEK	4	669033275	2xTMT6plex [N-Term; K8]	3.027		7.464	3.056016922	36.73	0	1364.730946	2	682.8652954	-5.592318185	-0.003816005	30.32165682
																			CGDLEEELK	2	669033275	2xTMT6plex [N-Term; K9]; 1xCarbamidomethyl [C1	1.337			2.410672665	15.73	0	1550.81363	2	775.9111938	0.954876385	0.000740418	56.19358147
																			EDKYEEEIK	13	669033275	3xTMT6plex [N-Term; K3; K9]	1.54	1.287	1.151	2.958414555	24.3	1	1870.041271	2	935.5215454	-2.917801748	-0.002728205	47.94373045
4885375	HIST1H1C	histone H1.2	3006	NP_005310.1	39.90610329	1	4	13	125	213	21.352	10.93	2.13	1.67	1.96	1.92	Significant	0.0049																
																			ASGPPVSELITK	11	4885375	2xTMT6plex [N-Term; K12]	2.468	2.746	2.739	4.860414505	78.21	0	1656.993643	2	829.0006104	0.181601858	0.000150457	66.19361867
																			KASGPPVSELITK	16	4885375	3xTMT6plex [N-Term; K1; K13]	1.721	1.428	1.877	5.893885612	42.56	1	2014.251538	3	672.0892334	0.798716189	0.000536272	58.09582745
																			KALAAAGYDVEK	12	4885375	3xTMT6plex [N-Term; K1; K12]	1.152	1.152	1.007	6.476112366	60.9	1	1923.151824	3	641.7223511	0.351419294	0.000225278	52.13334856
																			ALAAAGYDVEK	5	4885375	2xTMT6plex [N-Term; K11]	2.065	2.053		4.63003397	59.06	0	1565.893929	2	783.4494629	-1.456049294	-0.001140009	51.35155329
																			SGVSLAALK	10	4885375	2xTMT6plex [N-Term; K9]	2.54	100	100	3.625626802	59.93	0	1303.834958	2	652.4209595	-0.242020423	-0.000157777	61.01925825
																			KPAAATVTK	12	4885375	3xTMT6plex [N-Term; K1; K9]	2.024	1.285	100	4.255025864	37.17	0	1574.024439	3	525.3461304	-0.381891605	-0.000200369	36.52909542
																			KAAGGATPK	6	4885375	3xTMT6plex [N-Term; K1; K9]	1.384	1.538	1.553	4.330204487	24.6	1	1487.951274	3	496.6552124	-0.127768276	-6.34E-05	24.41122666
																			GTGASGSFK	20	4885375	2xTMT6plex [N-Term; K9]	1.768	1.906	1.992	3.84473753	55.74	0	1269.720322	2	635.364624	1.299000139	0.000824683	32.06205911
																			AASGEAKPK	15	4885375	3xTMT6plex [N-Term; K7; K9]	1.259	1.961	2.248	2.505070686	12.35	0	1545.956753	2	773.4815674	-0.579048782	-0.000447592	34.75106002
																			AAGGATPK	7	4885375	2xTMT6plex [N-Term; K8]	2.538	1.799	100	2.660259485	26.78	0	1130.693379	2	565.8505249	0.348524803	0.000197037	22.28976633
																			KPVGAAK	4	4885375	3xTMT6plex [N-Term; K1; K7]	3.019	2.728		3.018432856	12.01	0	1357.913432	3	453.3092957	-0.072447261	-3.28E-05	28.44253988
																			KAASGEAKPK	2	4885375	4xTMT6plex [N-Term; K1; K8; K10]	1.906	2.287		1.973932862		1	1903.214648	4	476.559845	1.524637704	0.000725428	26.06840674
																			GTLVQTK	5	4885375	2xTMT6plex [N-Term; K7]	2.149	1.798	2.043	3.357786417	40.19	0	1204.766544	2	602.8865967	-0.520657407	-0.000313635	34.71154277
4505641	PCNA	proliferating cell nuclear antigen	5111	NP_002583.1	22.22222222	1	5	5	27	261	28.75	4.69	2.74	5.512	2.084	3.45	Non-Significan	0.082																
																			LMDLDVEQLGIPEQEYSCVVK	9	4505641	2xTMT6plex [N-Term; K21]; 1xCarbamidomethyl [C18	100	2.918		2.773319244	26.03	0	2923.519703	3	975.1762085	-1.925932537	-0.001876834	94.13158958
																			DLSHIGDAVVISCAK	5	4505641	2xTMT6plex [N-Term; K15]; 1xCarbamidomethyl [C13			5.698	3.360664368	24.86	0	2043.130882	3	681.7137451	-2.05554058	-0.001399913	56.77489134
																			YLNFFTK	6	4505641	2xTMT6plex [N-Term; K7]	3.774	100	3.892	2.515175819	25.78	0	1390.813494	2	695.9093018	-1.558292599	-0.001083647	73.63218063
																			LVQGSILK	4	4505641	2xTMT6plex [N-Term; K8]	100		100	2.276948214	33.12	0	1315.871343	2	658.4386597	-0.988458385	-0.000650342	52.92470347
																			VSDYEMK	3	4505641	2xTMT6plex [N-Term; K7]	2.575		1.533	2.64865756	30.85	0	1329.712459	2	665.3605347	1.002649804	0.000666618	44.93107392
4502709	CDK1	cyclin-dependent kinase 1 isoform 1	983	NP_001777.1	25.58922559	1	5	6	27	297	34.074	8.4	2.252	1.855	8.015	4.04	Non-Significan	0.18																
																			KPLFHGDSEIDQLFR	4	4502709	2xTMT6plex [N-Term; K1]	2.036	1.778		3.285978079	23.86	0	2260.249023	4	565.8171387	-1.016681116	-0.000574488	70.88667826
																			WKPGSLASHVK	4	4502709	3xTMT6plex [N-Term; K2; K11]	2.662	2.518		4.155053139	15.97	0	1897.162664	4	475.0462646	0.297830946	0.000141258	51.9727208
																			ALGTPNNEVWPEVESLQDYK	2	4502709	2xTMT6plex [N-Term; K20]			100	1.76789093	7.72	0	2747.429231	3	916.4824219	1.267055384	0.001160382	83.15639899
																			DLKPQNLLIDDK	6	4502709	3xTMT6plex [N-Term; K3; K12]	0.713		100	2.38833189	10.52	0	2099.267916	3	700.4265137	-1.395083663	-0.000976218	57.91728246
																			IADFGLAR	18	4502709	1xTMT6plex [N-Term]	1.152	0.755	4.681	2.275216579	33.17	0	1091.64106	2	546.3240356	-0.243176028	-0.00013273	61.80413661
																			SPEVLLGSAR	2	4502709	1xTMT6plex [N-Term]		0.836		2.622908592	5.32	0	1257.736417	2	629.3712158	-1.003333733	-0.000630965	54.37543747
145309326	LAMC1	laminin subunit gamma-1 precursor	3915	NP_002284.3	3.107520199	1	5	5	18	1609	177.489	5.12	2.29	2.29	1.11	1.9	Significant	0.04																
																			GKTEQQTADQLLAR	2	145309326	2xTMT6plex [N-Term; K2]			1.082	4.089167118	23.74	1	2017.144223	3	673.0529175	-0.011986547	-8.06E-06	40.75343476
																			AFDITYVR	4	145309326	1xTMT6plex [N-Term]	1.932			3.273728132	28.55	0	1213.67784	2	607.3424072	-0.248564211	-0.000150838	60.14331805
																			EVMDLLR	8	145309326	1xTMT6plex [N-Term]	100	2.286		2.156861305	23.58	0	1104.628447	2	552.8186035	1.343339704	0.000741946	74.72445595
																			NTIEETGNLAEQAR	2	145309326	1xTMT6plex [N-Term]			1.536	1.727675557	1.74	0	1774.913271	2	887.9607544	0.54132733	0.000480405	41.0059653
																			DIEEIMK	2	145309326	2xTMT6plex [N-Term; K7]				1.338421702	15.82	0	1335.75941	2	668.3833008	-0.063422724	-4.24E-05	68.63759568
195927037	DNMT1	DNA (cytosine-5)-methyltransferase 1 isoform a	1786	NP_001124295.1	1.164215686	1	2	2	8	1632	184.703	7.85	2.59	2.37	2.49	2.48	Significant	0.00066																
																			GVCSCVEAGK	7	195927037	2xTMT6plex [N-Term; K10]; 2xCarbamidomethyl [C3; C5	2.587	3.457	2.492	4.04426527	62.54	0	1524.791455	2	762.8996582	0.383263067	0.000292198	34.20744021
																			DMSALVAAR	1	195927037	1xTMT6plex [N-Term]		1.196		2.419368029		0	1162.645159	2	581.8253784	-1.444089841	-0.000839482	52.48492775
24430190	KRT15	keratin, type I cytoskeletal 15	3866	NP_002266.2	31.14035088	1	8	13	391	456	49.167	4.77	0.39	0.36	0.37	0.37	Significant	0.00056																
																			FVSSGSGGGYGGGMR	6	24430190	1xTMT6plex [N-Term]	0.4	0.366	0.399	4.874829769	59.85	0	1604.768856	2	802.8887329	0.830823896	0.00066664	40.82740997
																			ALEEANADLEVK	217	24430190	2xTMT6plex [N-Term; K12]	0.606	0.627	0.555	5.218453407	90.06	0	1759.984201	2	880.4959717	0.264854295	0.00023307	51.10169088
																			EVASNTEMIQTSK	6	24430190	2xTMT6plex [N-Term; K13]	0.364	0.351		5.01432085	67.32	0	1896.014849	2	948.5097046	-1.432573334	-0.00135809	49.63799574
																			EVASNTEMIQTSK	4	24430190	2xTMT6plex [N-Term; K13]; 1xOxidation [M8	0.497			3.079568624	47.43	0	1912.009764	2	948.5097046	-1.432573334	-0.00135809	49.63799574
																			LEQEIATYR	30	24430190	1xTMT6plex [N-Term]	0.873	0.965	0.895	3.989738941	51.78	0	1351.741896	2	676.3740845	-0.742660038	-0.000501942	59.56020918
																			VLDELTLAR	31	24430190	1xTMT6plex [N-Term]	0.836	0.802	0.88	3.858995199	41.24	0	1258.756818	2	629.8816528	-0.626805691	-0.000394498	67.98248171
																			EQYEAMAEK	6	24430190	2xTMT6plex [N-Term; K9]	0.347	0.436	0.37	4.329832554	48.31	0	1556.803065	2	778.9063721	1.543066479	0.001201125	44.70261666
																			IMATTIDNSR	3	24430190	1xTMT6plex [N-Term]	0.461		0.357	2.781294346	20.55	0	1350.724866	2	675.866394	0.477910753	0.000322763	47.19757519
																			ITMQNLNDR	28	24430190	1xTMT6plex [N-Term]	0.566	0.576	0.616	3.402525187	42.38	0	1333.70955	2	667.3587646	0.526671549	0.000351213	48.81082799
																			LTMQNLNDR	16	24430190	1xTMT6plex [N-Term]; 1xOxidation [M3]	0.656	0.624	0.59	1.89945972	33.06	0	1349.704465	2	667.3587646	0.526671549	0.000351213	48.81082799
																			LKYENELALR	6	24430190	2xTMT6plex [N-Term; K2]	0.366	0.332	0.344	3.500450134	18.66	1	1707.020527	3	569.6781006	-0.455836456	-0.000259374	47.23233886
																			LASYLDK	51	24430190	2xTMT6plex [N-Term; K7]	0.689	0.687	0.676	3.160550594	38.22	0	1267.76621	2	634.3873291	0.924415808	0.000585972	69.69252583
																			GGSLLAGGGGFGGGSLSGGGGSR	2	24430190	1xTMT6plex [N-Term]				1.682545662	4.43	0	2051.046745	2	1026.022339	-4.555686333	-0.004671963	52.52682339
																			EQYEAMAEK	2	24430190	2xTMT6plex [N-Term; K9]; 1xOxidation [M6]	0.371			2.382699728	42.97	0	1572.79798	2	778.9063721	1.543066479	0.001201125	44.70261666
																			IHDWYQK	2	24430190	2xTMT6plex [N-Term; K7]	0.338			3.023718357	26.81	0	1447.809806	3	483.2747192	-0.139034254	-6.71E-05	51.56962103
																			VILEIDNAR	3	24430190	1xTMT6plex [N-Term]	0.693		0.527	2.38424468	26.44	0	1271.752067	2	636.3796387	-0.052121991	-3.31E-05	61.45169608
40317626	THBS1	thrombospondin-1 precursor	7057	NP_003237.2	22.99145299	1	19	19	74	1170	129.3	4.94	1.23	1.53	1.29	1.35	Significant	0.0046																
																			DCVGDVTENQICNK	10	40317626	2xTMT6plex [N-Term; K14]; 2xCarbamidomethyl [C2; C12	100	1.669	1.548	4.266801357	51.41	0	2110.03091	2	1055.511475	-7.221430349	-0.007618721	43.16870651
																			GDACDHDDDNDGIPDDKDNCR	3	40317626	2xTMT6plex [N-Term; K17]; 2xCarbamidomethyl [C4; C20		1.518	1.375	6.830832958	32.12	1	2877.205198	4	720.0568848	0.177628103	0.000127768	30.15975225
																			AQGYSGLSVK	6	40317626	2xTMT6plex [N-Term; K10]	1.062	1.524	1.151	4.599987984	58.54	0	1467.85715	2	734.4250488	-9.761688153	-0.007164382	44.14808554
																			DDFDHDSVPDIDDICPENVDISETDFR	4	40317626	1xTMT6plex [N-Term]; 1xCarbamidomethyl [C15	100			3.897334337	23.44	0	3409.485154	3	1137.165039	-1.346400495	-0.001530177	83.81537748
																			DLQAICGISCDELSSMVLELR	4	40317626	1xTMT6plex [N-Term]; 2xCarbamidomethyl [C6; C10			1.224	5.799779415	76.64	0	2638.308775	3	880.1074829	-0.333376267	-0.000293183	102.3868379
																			FVFGTTPEDILR	6	40317626	1xTMT6plex [N-Term]	1.631	1.587	100	3.828286171	36.27	0	1623.894374	2	812.4502563	-0.700744276	-0.000568967	92.17983897
																			DNCPFHYNPAQYDYDRDDVGDR	2	40317626	1xTMT6plex [N-Term]; 1xCarbamidomethyl [C3		0.929		4.098110676	10.49	1	2961.269564	4	741.0708618	-2.683377586	-0.001986551	49.08234614
																			AQLYIDCEK	4	40317626	2xTMT6plex [N-Term; K9]; 1xCarbamidomethyl [C7		1.636	1.236	4.172516346	42.96	0	1597.866	2	799.4371948	0.696452917	0.000556419	47.03617292
																			NPCTDGTHDCNK	2	40317626	2xTMT6plex [N-Term; K12]; 2xCarbamidomethyl [C3; C10	1.493			3.657343388	20.61	0	1876.868202	3	626.2947388	0.778482257	0.000487036	27.74137235
																			GGVNDNFQGVLQNVR	2	40317626	1xTMT6plex [N-Term]	1.191			2.400145531	31.1	0	1845.976875	2	923.491394	-0.738494659	-0.000681622	71.83693939
																			TCHIQECDKR	6	40317626	2xTMT6plex [N-Term; K9]; 2xCarbamidomethyl [C2; C7	1.185			3.946121454	16.03	1	1804.919844	3	602.3112183	-0.411173297	-0.000247378	26.0527956
																			IEDANLIPPVPDDKFQDLVDAVR	4	40317626	2xTMT6plex [N-Term; K14]				1.850450635	7.72	1	3037.661022	3	1013.224731	-0.45457477	-0.000460281	92.34771782
																			GTLLALER	4	40317626	1xTMT6plex [N-Term]	1.283		0.77	2.534425497	37.79	0	1101.682925	2	551.3463745	2.312365364	0.001273747	54.55521355
																			FTGSQPFGQGVEHATANK	2	40317626	2xTMT6plex [N-Term; K18]				1.691069484	11.58	0	2334.224264	3	778.7473145	1.339035594	0.00104187	36.55389727
																			IMADSGPIYDK	2	40317626	2xTMT6plex [N-Term; K11]		1.403		2.591054201	15.8	0	1667.907865	2	834.4579468	0.451028923	0.000376137	64.30681263
																			FQMIPLDPK	6	40317626	2xTMT6plex [N-Term; K9]	0.01	1.419	0.492	1.642273307	11.22	0	1546.906743	2	773.9567261	-0.366564801	-0.000283521	76.82199865
																			FQDLVDAVR	2	40317626	1xTMT6plex [N-Term]				2.061237812	21.61	0	1291.720767	2	646.3636475	-0.579527824	-0.000374294	72.09340226
																			TIVTTLQDSIR	3	40317626	1xTMT6plex [N-Term]				1.971007586	12.25	0	1475.863074	2	738.4344482	-0.985203608	-0.000727013	61.92085069
																			MENAELDVPIQSVFTR	2	40317626	1xTMT6plex [N-Term]				1.477702856	10.38	0	2078.078956	2	1039.542114	-0.964599718	-0.001002257	76.7946447
669033286	HMGB3	high mobility group protein B3 isoform b	3149	NP_001288160.1	43.18181818	1	7	8	36	220	25.485	9.06	1.67	1.25	1.57	1.5	Significant	0.0071																
																			KVEEEDEEEEEEEEEEEEDE	6	669033286	2xTMT6plex [N-Term; K1]	1.216	2.837	100	4.346535206	27.98	1	3257.327642	3	1086.445923	-1.359060493	-0.001475635	41.08386842
																			KLGEMWNNLNDSEK	4	669033286	3xTMT6plex [N-Term; K1; K14]	2.006	1.902		4.090102673	34.93	1	2365.278892	3	789.097229	-0.743226639	-0.000585979	73.24396718
																			STNPGISIGDVAK	2	669033286	2xTMT6plex [N-Term; K13]			100	3.208517075	54.33	0	1716.98962	2	858.9990845	0.740805476	0.000635978	49.9333386
																			KNPEVPVNFAEFSK	4	669033286	3xTMT6plex [N-Term; K1; K14]		2.171	2.858	4.008979797	34	1	2293.315929	3	765.1083984	-2.305455359	-0.001762379	59.52124965
																			KKVEEEDEEEEEEEEEEEEDE	6	669033286	3xTMT6plex [N-Term; K1; K2]		100		2.617940187	10.81	2	3614.585537	3	1205.533936	0.474804264	0.000572074	39.87044382
																			SKFDEMAK	9	669033286	3xTMT6plex [N-Term; K2; K8]	1.626	1.117	1.513	3.088344097	16.84	1	1642.94414	2	821.9751587	-0.668895041	-0.000549479	39.66208968
																			RPPSGFFLFCSEFRPK	2	669033286	2xTMT6plex [N-Term; K16]; 1xCarbamidomethyl [C10	100			2.566329956	10.64	0	2430.315663	4	608.3348389	0.766451362	0.00046568	75.83340741
																			KDPNAPK	3	669033286	3xTMT6plex [N-Term; K1; K7]	2.203	1.723		2.46927166	12.45	1	1456.909075	3	486.3074646	-0.847253403	-0.000411457	21.70500052
4507157	SORL1	sortilin-related receptor preproprotein	6653	NP_003096.1	0.677506775	1	1	1	6	2214	248.282	5.53	1.56	1.65	1.66	1.62	Significant	0.00038																
																			KISGDTCSGGDVEAR	6	4507157	2xTMT6plex [N-Term; K1]; 1xCarbamidomethyl [C7	1.559	1.651	1.66	3.989685535	21.16	1	2010.032624	3	670.6828613	0.699702262	0.000468808	36.91484617
189083772	GSN	gelsolin isoform b	2934	NP_001121134.1	35.29411765	1	23	23	110	731	80.591	5.85	0.51	0.58	0.41	0.5	Significant	0.0096																
																			AQPVQVAEGSEPDGFWEALGGK	10	189083772	2xTMT6plex [N-Term; K22]	0.544	0.851		4.443356037	37.42	0	2730.413916	3	910.8092041	-0.313683324	-0.000285495	96.39109704
																			DPDQTDGLGLSYLSSHIANVER	6	189083772	1xTMT6plex [N-Term]	0.599		0.01	4.297252655	30.9	0	2616.310289	3	872.7748413	-0.121749404	-0.000106178	80.79299182
																			EVQGFESATFLGYFK	16	189083772	2xTMT6plex [N-Term; K15]	0.709	1.261		4.605374336	59.57	0	2181.163227	2	1091.085938	0.628513255	0.000685445	96.00096324
																			VPFDAATLHTSTAMAAQHGMDDDGTGQK	4	189083772	2xTMT6plex [N-Term; K28]; 1xOxidation [M	100			3.077495575	14.94	0	3347.60366	4	837.6568604	0.582965144	0.000487884	58.60101412
																			SEDCFILDHGK	6	189083772	2xTMT6plex [N-Term; K11]; 1xCarbamidomethyl [C4	0.693	0.662	0.01	3.55607748	38.92	0	1778.914741	3	593.6419067	-2.009222293	-0.001191412	49.37220013
																			DSQEEEKTEALTSAK	6	189083772	3xTMT6plex [N-Term; K7; K15]	0.748	1.044		4.448760509	34.99	1	2353.270161	3	785.0951538	0.31767529	0.000249192	52.65826348
																			TPSAAYLWVGTGASEAEK	6	189083772	2xTMT6plex [N-Term; K18]				2.428411245	20.99	0	2296.222533	2	1148.613647	-1.095215332	-0.001257429	67.18505638
																			QTQVSVLPEGGETPLFK	6	189083772	2xTMT6plex [N-Term; K17]			100	2.709791422	23.11	0	2288.290219	2	1144.647705	-0.911275909	-0.001042632	70.17045468
																			MVVEHPEFLK	6	189083772	2xTMT6plex [N-Term; K10]		0.491	0.774	2.744548321	29.1	0	1686.96532	3	562.9929199	-0.660057656	-0.000371165	65.55957545
																			YIETDPANR	6	189083772	1xTMT6plex [N-Term]	0.59			2.700572729	45.1	0	1307.679296	2	654.3435669	0.429156494	0.0002806	38.43660066
																			VVEHPEFLK	4	189083772	2xTMT6plex [N-Term; K9]	0.352	0.563	0.237		41.63	0	1555.924835	3	519.3130493	-0.1547252	-8.02E-05	46.03208748
																			TEALTSAK	4	189083772	2xTMT6plex [N-Term; K8]	0.694	0.831		3.402081966	46.27	0	1278.766938	2	639.8869019	-0.321230594	-0.00020539	40.58573736
																			AGALNSNDAFVLK	2	189083772	2xTMT6plex [N-Term; K13]			1.573	2.513380289	17.59	0	1778.021255	2	889.513916	-0.393386045	-0.000349724	59.55551429
																			AGKEPGLQIWR	4	189083772	2xTMT6plex [N-Term; K3]	0.645	0.583		3.19527626	4.94	1	1713.021195	3	571.678894	0.544948836	0.00031117	56.38538377
																			HVVPNEVVVQR	2	189083772	1xTMT6plex [N-Term]		0.631		2.946653605	31.81	0	1504.879727	2	752.9429932	-0.676035338	-0.000508676	38.90550944
																			TASDFITK	4	189083772	2xTMT6plex [N-Term; K8]	0.688		0.583	3.465964317	36.1	0	1340.782588	2	670.895813	1.313730189	0.000880713	50.93357894
																			DSQEEEK	2	189083772	2xTMT6plex [N-Term; K7]			100	2.958912611	25.58	0	1322.683996	2	661.8458862	0.377876302	0.000249905	24.93398828
																			RTPITVVK	2	189083772	2xTMT6plex [N-Term; K8]		0.475		3.932851791	28.62	1	1371.908792	3	457.9741516	-0.648684534	-0.000296645	43.98356731
																			AVEVLPK	7	189083772	2xTMT6plex [N-Term; K7]	0.502	0.541	0.525	2.718402386	33.34	0	1213.79203	2	607.3995361	-0.19348815	-0.000117427	39.66624663

Bhat et al,2017. Cigarette smoke and chewing tobacco alter expression of different sets of miRNAs in oral keratinocytes

Supplementary Table S2(A). List of all proteins and peptides identified in OKF6/TERT1 cells chronically treated with chewing tobacco using Sequest and Mascot search algorithms

Accession	Gene_Symbol	Description	Gene_ID	Refseq_Accession	Coverage	Proteins	#Unique_Peptides	#Peptides	#PSMS	#AAs	MW [kDa]	calc. pI	OKF6-STE-6M/OKF6P_R1	OKF6-STE-6M/OKF6P_R2	OKF6-STE-6M/OKF6P_R3	Median fold change	Status	P-value (6M)	Sequence	#PSMSs	Protein Accessions	Modifications	OKF6-STE-6M/OKF6P_R1	OKF6-STE-6M/OKF6P_R2	OKF6-STE-6M/OKF6P_R3	Xcorr	Ionscore	# Missed Cleavages	Theo. MH+ [Da]	Charge	m_z	DeltaM [ppm]	Deltam/z [Da]	RT [min]
																			TGAQELLR	2	189083772	1xTMT6plex [N-Term]			1.089	2.56367135	23.84	0	1116.657438	2	558.8325806	0.399489402	0.000223046	42.01672119
																			EPGLQIWR	2	189083772	1xTMT6plex [N-Term]			100	2.135707378	21.5	0	1227.704723	2	614.3557129	-0.467383353	-0.000286904	53.75186019
																			TPITVVK	2	189083772	2xTMT6plex [N-Term; K7]			0.551	2.944895744	36.37	0	1215.807681	2	608.4078979	0.689844661	0.000419359	50.37292295
																			VSNGAGTMSVSLVADENPFAQGALK	1	189083772	2xTMT6plex [N-Term; K25]				1.645085454	0.25	0	2921.544278	3	974.5106812	-9.168939582	-0.008929154	89.43743243
4506141	HTRA1	serine protease HTRA1 precursor	5654	NP_002766.1	16.66666667	1	6	6	23	480	51.255	7.83	0.6	0.76	0.72	0.69	Significant	0.0048																
																			GACGQGQEDPNSLR	6	4506141	1xTMT6plex [N-Term]; 1xCarbamidomethyl [C3	0.604		0.241	4.070591927	56.16	0	1717.812512	3	573.2775269	3.210755754	0.001838492	31.20086427
																			EVPVASGSGFIVSEDGLIVTNAHVVTNK	4	4506141	2xTMT6plex [N-Term; K28]			0.711	5.484000206	52.53	0	3297.809477	4	825.2080688	0.29370424	0.000242145	82.47691127
																			ESTLNMVVR	2	4506141	1xTMT6plex [N-Term]		0.762		3.104501009	33.33	0	1277.708488	2	639.3584595	0.903707946	0.000577338	44.58310406
																			IAPAVVHIELFR	4	4506141	1xTMT6plex [N-Term]	0.515			3.171844959	27.41	0	1593.967814	3	531.9946899	1.06826763	0.000567595	74.16091409
																			ADIALIK	3	4506141	2xTMT6plex [N-Term; K7]			0.75	2.693772078	38.91	0	1201.79203	2	601.399292	-0.601714445	-0.000361568	55.52287789
																			YNFIADVVEK	4	4506141	2xTMT6plex [N-Term; K10]			100	1.643605947	15.74	0	1655.940879	2	828.4702759	-4.592085552	-0.003802111	81.83266414
4505571	SQSTM1	sequestosome-1 isoform 1	8878	NP_003891.1	26.81818182	1	5	5	20	440	47.657	5.22	0.98	1.02	1.09	1.03	Significant	0.00097																
																			DEDGDLVAFSSDEELTMAMSYVK	6	4505571	2xTMT6plex [N-Term; K23]			1.542	3.474927664	26.09	0	3010.431341	3	1004.146667	-1.957071239	-0.001963876	107.0548518
																			KIALESEGRPEEQMESDNCSGGDDDWTHLSSK	2	4505571	3xTMT6plex [N-Term; K1; K32]; 1xCarbamidomethyl [C19	0.876			4.573376656	22.48	1	4295.043554	5	859.8134766	-1.228825141	-0.001055571	65.68978361
																			EAALYPHLPPEADPR	6	4505571	1xTMT6plex [N-Term]	1.141	1.023	1.042	3.306419849	32.37	0	1905.006778	3	635.6742554	0.753279882	0.000478334	46.84014966
																			LTPVSPESSSTEEK	2	4505571	2xTMT6plex [N-Term; K14]	0.933			3.04297924	43.26	0	1949.047923	2	975.0286255	1.052527513	0.001025713	45.0089963
																			DEDGDLVAFSSDEELTMAMSYVK	3	4505571	2xTMT6plex [N-Term; K23]; 1xOxidation [M			0.949	3.136191607	11.12	0	3026.426256	3	1004.146667	-1.957071239	-0.001963876	107.0548518
																			EVDPSTGELQSLQMPESEGPSSLDPSQEGPTGLK	1	4505571	2xTMT6plex [N-Term; K34]				1.070875883	1.43	0	3984.963606	4	996.9940186	-2.349351757	-0.00234052	71.63880027
148612842	IFITM3	interferon-induced transmembrane protein [illegible]	10410	NP_066362.2	31.57894737	1	3	3	14	133	14.622	7.01	0.82	1.02	0.66	0.84	Significant	0.015																
																			EEHEVAVLGAPHNPAPPTSTVIHIR	6	148612842	1xTMT6plex [N-Term]	0.795	0.723	0.782	7.879145145	53.35	0	2900.558005	5	580.916626	-1.372659047	-0.000796295	43.7618419
																			MVGDVTGAQAYASTAK	6	148612842	2xTMT6plex [N-Term; K16]	0.829	100	100	4.424339771	54.54	0	2028.083597	2	1014.544983	-0.447604669	-0.00045389	50.38084129
																			KMVGDVTGAQAYASTAK	2	148612842	3xTMT6plex [N-Term; K1; K17]			0.247	4.877254009	32	1	2385.341492	3	795.7843628	-1.239575593	-0.000985604	45.28647676
4504431	HMGA2	high mobility group protein HMGI-C isoform a	8091	NP_003474.1	53.21100917	1	5	5	28	109	11.825	10.62	0.347	0.262	0.01	0.21	Non-Significant	0.18																
																			GEGAGQPSTSAQGQPAAPAPQK	6	4504431	2xTMT6plex [N-Term; K22]	0.378			4.706462383	56.81	0	2493.309785	3	831.7736206	-1.394260599	-0.001158775	36.5750388
																			KPAQEETEETSSQESAEED	4	4504431	2xTMT6plex [N-Term; K1]		0.073	0.01	4.539194584	37.11	0	2582.19935	3	861.4036255	-1.172304979	-0.001009042	29.9595313
																			KWPQQVVQK	6	4504431	3xTMT6plex [N-Term; K1; K9]	0.318	0.293		3.889223337	32.28	1	1828.1412	3	610.0513916	-0.863409956	-0.000526145	53.44360867
																			AEATGEK	4	4504431	2xTMT6plex [N-Term; K7]	0.317	0.354		2.909908056	37.07	0	1163.667224	2	582.3372192	-0.053299978	-3.10E-05	21.91918611
																			KAEATGEK	8	4504431	3xTMT6plex [N-Term; K1; K8]	0.342	0.358		2.591320515	32.87	1	1520.925119	2	760.9654541	-0.977863313	-0.000743628	27.95328961
41322923	PLEC	plectin isoform 1a	5339	NP_958786.1	57.29052122	1	2	235	1787	4547	515.884	5.8	1.01	0.99	0.96	0.99	Significant	0.00022																
																			GDECQLVGPAQPSHWK	6	41322923	2xTMT6plex [N-Term; K16]; 1xCarbamidomethyl [C4	1.266	1.125	1.408	5.225980282	51.9	0	2267.164307	3	756.3917236	-1.627368405	-0.001229837	45.7760475
																			RQELEAELAK	14	41322923	2xTMT6plex [N-Term; K10]	0.974	1.047	0.983	5.4715662	40.11	1	1644.968491	3	548.9942017	-0.26706866	-0.00014644	42.89150603
																			SLAAEEEAAR	6	41322923	1xTMT6plex [N-Term]	1.021	0.908	1.005	4.060409069	67.26	0	1275.674211	2	638.340332	-0.645248993	-0.000411564	33.90928655
																			DALDGPAAEAEPEHSFDGLR	12	41322923	1xTMT6plex [N-Term]	1.316	1.556	2.729	3.873622656	49.37	0	2326.114884	3	776.0438232	0.873751583	0.000677482	58.59209881
																			AQAEAQQPTFDALRDELR	6	41322923	1xTMT6plex [N-Term]	0.653	0.866	100	5.450631618	20.8	1	2288.183238	3	763.4005737	1.717315004	0.001309844	61.37110094
																			SSSVGSSSSYPISPAVSR	2	41322923	1xTMT6plex [N-Term]	1.168			3.643047094	42.75	0	1984.018465	2	992.5133057	0.438553441	0.000435049	48.21533703
																			GYYSPYSVSGSGSTAGSR	6	41322923	1xTMT6plex [N-Term]	0.661	0.555	0.599	4.647460461	42.02	0	2011.955864	2	1006.47229	-9.225307674	-0.009280456	42.71505522
																			EQLQLLEEQHR	8	41322923	1xTMT6plex [N-Term]	1.414	1.255	1.042	4.13542366	65.92	0	1651.896499	2	826.4512939	-0.719136686	-0.00059397	52.28282662
																			DTHDQLSEPSEVR	8	41322923	1xTMT6plex [N-Term]	1.038	0.992	100	5.132863522	52.97	0	1741.855422	3	581.2897949	-0.339083467	-0.000196878	32.98123797
																			LLEAAAQSTK	6	41322923	2xTMT6plex [N-Term; K10]	1.226	1.089	1.104	4.677412033	65.04	0	1489.899015	2	745.453125	-0.027652881	-2.06E-05	47.394452
																			QSSEAEIQAK	8	41322923	2xTMT6plex [N-Term; K10]	1.123	1.166	1.202	4.571704865	62.38	0	1548.863357	2	774.9362793	1.242642704	0.000962342	22.89428824
																			AQAEAQQPTFDALR	8	41322923	1xTMT6plex [N-Term]	1.269		0.98	4.243383884	38.94	0	1774.928528	2	887.9675293	-0.420065506	-0.000372793	54.24746812
																			AALAHSEEVTASQVAATK	6	41322923	2xTMT6plex [N-Term; K18]	0.905	0.938	0.945	6.170174599	63.35	0	2242.244331	3	748.0864868	0.256876207	0.000191993	44.04468484
																			GLFDEEMNEILTDPSDDTK	13	41322923	2xTMT6plex [N-Term; K19]		0.783	0.927	3.96105361	51.74	0	2627.279849	3	876.430481	-1.126544973	-0.000986583	88.62165042
																			EQELQQTLQQEQSVLDQLR	15	41322923	1xTMT6plex [N-Term]	1.111	4.583	1.352	3.556969881	40.25	0	2542.331025	3	848.1137085	-1.751318642	-0.001484144	87.78895674
																			GFFDPNTEENLTYLQLMER	24	41322923	1xTMT6plex [N-Term]	0.959	1.692	0.785	3.645915985	31.19	0	2546.243455	3	849.4177246	-1.898778322	-0.001611584	92.26274236
																			QVQVALETAQR	6	41322923	1xTMT6plex [N-Term]	0.853	0.838	0.796	3.750156879	53.58	0	1471.843007	2	736.4255981	0.619970376	0.00045625	55.55691539
																			ATTAALLEAQAATGFLVDPVR	3	41322923	1xTMT6plex [N-Term]		1.459		3.701076508	44.92	0	2457.39144	2	1229.201294	1.575268214	0.001935525	117.024896
																			LQEAGILSAEELQR	6	41322923	1xTMT6plex [N-Term]	0.966		1.685	5.295166969	58.58	0	1785.990793	2	893.4992676	0.260419176	0.000232553	93.47670802
																			VLSSSGSEAAVPSVCFLVPPPNQEAQEAVTR	4	41322923	1xTMT6plex [N-Term]; 1xCarbamidomethyl [C15				4.185986519	19.62	0	3455.767799	3	1152.59314	-0.848863367	-0.000977825	86.17113175
																			ESADPLGAWLQDAR	10	41322923	1xTMT6plex [N-Term]	1.701	1.694	100	3.758331537	49.26	0	1757.901978	2	879.4535522	-1.223388925	-0.001075299	87.72849509
																			AKLEQLFQDEVAK	6	41322923	3xTMT6plex [N-Term; K2; K13]	0.734	0.743	0.48	4.553472996	34.52	1	2206.30503	3	736.105835	-0.942089674	-0.000692846	84.95097515
																			LLEAQIATGGVIDPVHSHR	3	41322923	1xTMT6plex [N-Term]	0.683	1.02		5.290122032	51.1	0	2242.25053	3	748.0886841	0.432068621	0.000322935	66.98597845
																			DPYSGSTISLFQAMQK	8	41322923	2xTMT6plex [N-Term; K16]	0.983		1.394	4.19281292	32.8	0	2231.178226	3	744.397644	0.068712084	5.11E-05	90.28572194
																			DLLPSDMAVALLEAQAGTGHIIDPATSAR	13	41322923	1xTMT6plex [N-Term]	0.964	1.354		4.12898159	43.87	0	3162.666628	3	1054.891724	-1.90053593	-0.002003587	103.9443819
																			QEELYSELQAR	6	41322923	1xTMT6plex [N-Term]	1.158	1.28		3.45531559	63.77	0	1594.827417	2	797.9169922	-0.44446502	-0.000354423	57.87025896
																			LLEAQIATGGIIDPVHSHR	6	41322923	1xTMT6plex [N-Term]	1.15		100	3.080124617	34.91	0	2256.26618	3	752.7589111	-1.772885485	-0.001333367	65.62193604
																			QLAEAHAQAK	6	41322923	2xTMT6plex [N-Term; K10]	0.875	1.007	1.124	4.588809013	52.66	0	1524.889847	3	508.9674072	-1.428601756	-0.000726153	26.00986848
																			TLLQGSGCLAGIYLEDTK	5	41322923	2xTMT6plex [N-Term; K18]; 1xCarbamidomethyl [C8	1.329			5.749361038	76.02	0	2397.309968	3	799.774353	-0.61001205	-0.000487463	88.1483848
																			LQMEAGLCEEQLNQADALLQSDVR	26	41322923	1xTMT6plex [N-Term]; 1xCarbamidomethyl [C8]; 1xOxidation [M3]	1.149	1.091		5.257513046	65.89	0	2976.460401	4	740.871582	-0.33370259	-0.000246979	90.29856489
																			LQMEAGLCEEQLNQADALLQSDVR	92	41322923	1xTMT6plex [N-Term]; 1xCarbamidomethyl [C8	1.163	2.302	1.355	5.793747902	73.29	0	2960.465486	4	740.871582	-0.33370259	-0.000246979	90.29856489
																			AFEETQIDIPGGGSHGGSTMSLWEVMQSDLIPEEQR	4	41322923	1xTMT6plex [N-Term]				2.896802664	13.29	0	4160.974239	4	1040.995117	-3.749107566	-0.003899985	95.98800473
																			FLEGTSCIAGVFVDATK	2	41322923	2xTMT6plex [N-Term; K17]; 1xCarbamidomethyl [C7			1.228	3.74762702	43.43	0	2273.225176	3	758.4161987	3.900573291	0.002955627	101.7545257
																			EALEAESAWCYLYGTGSVAGVYLPGSR	4	41322923	1xTMT6plex [N-Term]; 1xCarbamidomethyl [C10				3.689950943	23.71	0	3135.529467	3	1045.847778	-0.21843171	-0.0002283	101.9949974
																			SQVMDEATALQLR	4	41322923	1xTMT6plex [N-Term]	0.608	4.199		3.880594015	40.12	0	1690.899536	2	845.953186	-0.260340534	-0.000220105	59.43915964
																			AYSDPSTGEPATYGELQQR	8	41322923	1xTMT6plex [N-Term]	0.96	1.056		4.562547684	60.99	0	2299.103985	3	767.0387573	-0.985721985	-0.000755426	50.03753883
																			KQEELQQLEQQR	8	41322923	2xTMT6plex [N-Term; K1]		1.113	1.226	4.145999908	26.15	1	2015.128573	2	1008.068665	0.734033343	0.000739586	48.68426315
																			TVTVWELISSEYFTAEQR	2	41322923	1xTMT6plex [N-Term]			1.255	4.052988052	45.25	0	2388.228457	3	796.7470093	-0.830183546	-0.000660889	109.9996557
																			QVEVTVHKGDECQLVGPAQPSHWK	2	41322923	3xTMT6plex [N-Term; K8; K24]; 1xCarbamidomethyl [C12		0.948		6.154533386	16.5	1	3416.835204	5	684.1716919	-1.712531332	-0.001170287	50.88942634
																			GGAEGELQALR	5	41322923	1xTMT6plex [N-Term]	1.112	1.278		4.758570671	64.63	0	1329.732394	2	665.3690186	-1.228563455	-0.00081683	50.4820435
																			QEQIQAMPLADSQAVR	4	41322923	1xTMT6plex [N-Term]		0.848	1.223	3.953153372	46.41	0	2014.05889	2	1007.532227	-0.850613161	-0.000856592	47.90060383
																			GGELVYTDSEAR	8	41322923	1xTMT6plex [N-Term]	1.054	1.175		4.681869507	65.55	0	1525.769567	2	763.388916	0.647556008	0.000494011	42.0606006
																			GFFDPNTHENLTYR	12	41322923	1xTMT6plex [N-Term]	0.848	0.817		4.955785275	49.26	0	1939.949991	3	647.3212891	-0.349139154	-0.000225771	61.25660307
																			REEAAVDAQQQK	38	41322923	2xTMT6plex [N-Term; K12]	1.072	0.72	1.017	5.348618984	51.26	1	1831.007396	3	611.0071411	-0.28702241	-0.00017518	27.74690146
																			QLQLAQEAAQK	4	41322923	2xTMT6plex [N-Term; K11]	1.14	0.99		4.746269703	68.02	0	1685.99504	2	843.5014038	0.291137978	0.000245429	53.8519135
																			AEVVETTQVYTEEETRR	6	41322923	1xTMT6plex [N-Term]	0.932	0.828	0.929	5.174630165	30.79	1	2269.150935	3	757.0544434	-0.951171578	-0.000719451	44.66228578
																			DSQDAGGFGPEDR	2	41322923	1xTMT6plex [N-Term]		0.916		3.821832418	62.13	0	1579.718594	2	790.3632202	0.360443746	0.0002847	32.76730248
																			EYGSCSHHYQQLLQSLEQGAQEESR	5	41322923	1xTMT6plex [N-Term]; 1xCarbamidomethyl [C5			100	5.411483288	29.53	0	3193.480619	4	799.1256104	-0.002287081	-1.83E-06	62.18901229
																			EEAAVDAQQQK	8	41322923	2xTMT6plex [N-Term; K11]	1.336	1.384		4.615660667	65.57	0	1674.906285	2	837.956665	-0.138044664	-0.000115606	37.46344313
																			LKTEAEIALK	6	41322923	3xTMT6plex [N-Term; K2; K10]	1.067	0.915	0.859	4.26043272	24.75	1	1803.155847	3	601.7191772	-7.136627563	-0.004289484	56.67017184
																			AQAEVEGLGK	10	41322923	2xTMT6plex [N-Term; K10]	0.888	0.865		4.591239929	60.57	0	1459.852064	2	730.428833	-1.14737953	-0.000837502	51.59479026
																			LQEDKEQMAQQLAEETQGFQR	3	41322923	2xTMT6plex [N-Term; K5]	100			3.546393871	20.66	1	2965.508955	3	989.1749268	0.429023637	0.000424091	79.1465508
																			GLHQSIEEFR	14	41322923	1xTMT6plex [N-Term]	0.869	0.857	0.805	4.439191341	52.23	0	1444.774593	2	722.8920898	1.5987044	0.001154884	49.81199374
																			HISDLYEDLR	6	41322923	1xTMT6plex [N-Term]	1.356	1.208		3.787079334	53.68	0	1489.784824	2	745.3961182	0.091367641	6.81E-05	64.7218241
																			LLEAQIATGGIIDPEESHR	4	41322923	1xTMT6plex [N-Term]	0.965			3.511247396	29.81	0	2278.224041	3	760.0790405	-0.646230547	-0.000490753	80.46124137
																			QEQALLEEIER	6	41322923	1xTMT6plex [N-Term]	1.528	0.823	100	3.536032915	52.18	0	1586.858717	2	793.9342651	1.598714121	0.001268467	67.86525627
																			GIIRPGTAFELLEAQAATGYVIDPIK	6	41322923	2xTMT6plex [N-Term; K26]	100		0.01	2.482069731	19.31	0	3201.828756	3	1067.944824	-2.759836463	-0.002945508	104.4542238
																			DDGTGQLLLPLSDAR	3	41322923	1xTMT6plex [N-Term]	100	0.01		2.541205406	40.79	0	1799.970058	2	900.4888916	0.24923366	0.000224307	79.25737652
																			SMVEEGTGLR	6	41322923	1xTMT6plex [N-Term]	1.055	0.948	0.925	4.386152267	65.61	0	1307.682667	2	654.3392334	-8.776298269	-0.005738307	39.23365068
																			AEMEVLLASK	28	41322923	2xTMT6plex [N-Term; K10]	0.954	1.347	0.01	4.172862053	61.18	0	1548.907136	2	774.9577026	0.64059582	0.000496112	79.32594043
																			QTNLENLDQAFSVAER	4	41322923	1xTMT6plex [N-Term]				2.766792536	33.33	0	2064.055913	2	1032.530762	-0.807106285	-0.000832956	83.91862726
																			AQVEQELTTLR	8	41322923	1xTMT6plex [N-Term]	0.779	0.903	0.7	3.767987251	47.66	0	1516.853237	2	758.9295654	-0.911901422	-0.00069161	64.77813178
																			LAQGHTTVDELAR	8	41322923	1xTMT6plex [N-Term]	0.941	0.986		4.045354366	46.44	0	1639.896499	3	547.3031006	-1.067568708	-0.000583567	42.37472743
																			SDEGQLSPATR	4	41322923	1xTMT6plex [N-Term]	1.022	0.959		3.676899433	54.95	0	1389.717138	2	695.3631592	1.369911418	0.000951895	31.8352175
																			AEAELLQQQK	4	41322923	2xTMT6plex [N-Term; K10]	1.39	1.33		4.361347675	51.46	0	1615.941942	2	808.4743652	-0.302059893	-0.000244056	53.21157228
																			SLESLHSFVAAATK	12	41322923	2xTMT6plex [N-Term; K14]	0.988	0.661	0.823	4.213026047	30.26	0	1919.100233	3	640.3703613	-1.929370629	-0.001234219	67.79985453
																			LQLEETDHQK	8	41322923	2xTMT6plex [N-Term; K10]	0.882	1.088	0.972	4.340321064	39.54	0	1698.94267	3	566.9822998	-6.076735913	-0.003441342	38.35778371
																			QLEMSAEAER	14	41322923	1xTMT6plex [N-Term]	1.223	0.996	1.081	4.402211666	49.7	0	1392.699045	2	696.8528442	-0.454673563	-0.000316612	79.17021003
																			DPYTGQSVSLFQALK	8	41322923	2xTMT6plex [N-Term; K15]	100	1.482		3.482981205	30.6	0	2112.174127	2	1056.589722	-0.927849935	-0.00097989	88.97945631
																			YLQDLLAWVEENQHR	8	41322923	1xTMT6plex [N-Term]		3.315	100	3.159788132	27.68	0	2143.113368	3	715.0427856	0.203257373	0.000145201	107.9631089
																			AQLMADFQAGR	11	41322923	1xTMT6plex [N-Term]	0.955	0.925	100	3.039898634	38.08	0	1436.751749	2	718.8804932	1.364402811	0.000980154	60.26671536
																			ALQALEELR	6	41322923	1xTMT6plex [N-Term]	0.925	1.553	100	3.203480482	48.88	0	1271.752067	2	636.3795776	-0.148107927	-9.42E-05	63.1581268
																			SQVEEELFSVR	10	41322923	1xTMT6plex [N-Term]	100	2.724	100	3.195386171	47.22	0	1551.821603	2	776.4141235	-0.407598197	-0.00031626	59.06948836
																			LLDAQLSTGGIVDPSK	9	41322923	2xTMT6plex [N-Term; K16]	100	100		1.947249174	23.55	0	2072.200341	2	1036.602905	-0.872171039	-0.000903657	67.54050242
																			GLVGPELHDR	2	41322923	1xTMT6plex [N-Term]	0.926			3.857473612	48.49	0	1321.742565	3	441.2520752	-0.675331253	-0.000297538	36.79975889
																			IIITVVEEQEQK	12	41322923	2xTMT6plex [N-Term; K12]	2.295	1.624	1.088	2.863016844	25.74	0	1887.1203	2	944.0632324	-0.589197334	-0.000555943	80.32810508
																			ELAEQELEK	8	41322923	2xTMT6plex [N-Term; K9]		0.877	0.964	4.668473721	67.21	0	1546.872859	2	773.9387817	-1.663028362	-0.001286247	44.6269313
																			LLDAQLATGGIVDPR	6	41322923	1xTMT6plex [N-Term]	1.071	0.749	0.999	3.192177057	56.62	0	1768.016614	2	884.5115356	-0.463582147	-0.00040981	73.83687008
																			GLLSAEVAR	26	41322923	1xTMT6plex [N-Term]	0.894	0.993	0.693	3.353006124	54.12	0	1144.688739	2	572.8466187	-2.426734202	-0.001388928	43.02139018
																			SLAQAEAEK	10	41322923	2xTMT6plex [N-Term; K9]	0.982	0.901	1.083	4.452575207	55.02	0	1404.809865	2	702.9095459	1.388036149	0.000974963	37.75338535
																			VLADPSDDTK	12	41322923	2xTMT6plex [N-Term; K10]	0.905	0.921	0.968	3.946383953	52.5	0	1518.841559	2	759.9228516	-2.062575244	-0.001566363	43.77595946
																			LQAEEVAQQK	6	41322923	2xTMT6plex [N-Term; K10]	0.889	0.93	1.001	4.941749573	40.47	0	1601.926292	2	801.465271	-1.889305287	-0.001513264	39.72930285
																			LSVAAQEAAR	20	41322923	1xTMT6plex [N-Term]	0.862	1.03	0.822	3.763278484	51.21	0	1244.716016	2	622.8621826	0.861846687	0.000536377	35.17815816
																			KQITMEELVR	10	41322923	2xTMT6plex [N-Term; K1]	0.678	0.383	0.647	3.687356472	21.46	1	1705.008247	3	569.0085449	1.662235728	0.000944709	48.56988074
																			LVASMEEAR	12	41322923	1xTMT6plex [N-Term]	0.974	0.946	1.069	3.795512438	61.31	0	1234.666288	2	617.8307495	-9.772759388	-0.006033048	46.17539441
																			NLVDNITGQR	4	41322923	1xTMT6plex [N-Term]	0.955	0.971		3.795066357	47.01	0	1358.758943	2	679.8829346	-0.258117436	-0.00017536	58.14416078
																			GCLDEETSR	10	41322923	1xTMT6plex [N-Term]; 1xCarbamidomethyl [C2	0.927	0.993	1.217	3.443276405	54.14	0	1295.609896	2	648.3093262	1.142059623	0.000739832	26.93657944
																			QELEAELAK	6	41322923	2xTMT6plex [N-Term; K9]	1.301	0.885		3.821063519	55.2	0	1488.86738	2	744.9376221	0.394548657	0.000293715	47.78783408
																			VTLVQTLEIQR	4	41322923	1xTMT6plex [N-Term]	1.281			3.632424831	37.05	0	1528.926008	2	764.9672241	0.760744589	0.000581561	73.20705923
																			QYDIDDAIAK	7	41322923	2xTMT6plex [N-Term; K10]	1.097	1.027	0.971	4.382020473	51.17	0	1609.883758	2	805.4459229	0.503578672	0.000405352	51.4915855
																			SAEAELQSK	10	41322923	2xTMT6plex [N-Term; K9]	0.957	0.962	1.046	4.534872055	53.14	0	1420.80478	2	710.9058228	-0.289246062	-0.000205481	36.14162419
																			HKPLLIDMNK	6	41322923	3xTMT6plex [N-Term; K2; K10]	1.006	0.88	0.98	4.492433548	34.99	0	1896.170786	3	632.7280273	-0.662782466	-0.000418916	45.07606799
																			QLEMSAEAER	12	41322923	1xTMT6plex [N-Term]; 1xOxidation [M4]	1.036	0.919	1.052	2.359773159	43.15	0	1408.69396	2	696.8528442	-0.454673563	-0.000316612	79.17021003
																			LAEVEAALEK	6	41322923	2xTMT6plex [N-Term; K10]	1.047	1.257	2.227	3.718738794	42.44	0	1530.91433	2	765.9598389	-1.260132972	-0.000964578	71.89396598
																			AQAQAAAEK	30	41322923	2xTMT6plex [N-Term; K9]	0.902	0.836	0.933	4.136806488	57.24	0	1345.783985	2	673.3950195	-0.908360862	-0.000611229	33.52428866
																			SLSAIYLEK	6	41322923	2xTMT6plex [N-Term; K9]	1.16	1.265		4.144323826	57.61	0	1481.897952	2	741.4529419	0.442128327	0.000327595	73.92988277
																			SKEQAELEAAR	8	41322923	2xTMT6plex [N-Term; K2]	0.874	0.955		3.297512293	43.43	1	1689.953569	2	845.4830933	3.160254539	0.002670342	37.04551416
																			VVIVDPETGK	10	41322923	2xTMT6plex [N-Term; K10]	1.397	1.381	100	3.910618544	53.11	0	1514.919416	2	757.9634399	0.123830225	9.38E-05	62.65555887
																			EQAELEAAR	12	41322923	1xTMT6plex [N-Term]	1.549	1.528	1.628	3.920178413	50.03	0	1245.663646	2	623.3345337	-1.489284201	-0.000927574	30.69799617
																			ELIPTEEALR	6	41322923	1xTMT6plex [N-Term]	0.871	0.972	0.891	3.143421888	30.5	0	1399.799411	2	700.4069214	5.111520537	0.003577552	57.73123923
																			FPVTDAVNK	9	41322923	2xTMT6plex [N-Term; K9]	0.946	1.012	0.99	4.137485504	42.82	0	1448.851336	2	724.9294434	0.189066154	0.000136964	49.87106611
																			DLSELGSVR	8	41322923	1xTMT6plex [N-Term]	1.057	1.016	1.059	3.615808487	54.71	0	1204.673482	2	602.8395996	-1.294741914	-0.000779871	45.09511859
																			SLLAWQSLR	6	41322923	1xTMT6plex [N-Term]	1.518	1.272	100	3.830304623	46.12	0	1302.773137	2	651.8902588	0.079920381	5.21E-05	80.21785157
																			AQAEQAALR	6	41322923	1xTMT6plex [N-Term]	1.185	1.242	1.296	3.220926285	48.51	0	1186.674151	2	593.8405151	-0.334907912	-0.000198713	35.97708257
																			AAEEAEEAR	12	41322923	1xTMT6plex [N-Term]	1.065	0.732	0.973	3.579045773	44.8	0	1204.600711	2	602.8041992	0.340791348	0.000205259	21.58932271
																			LEQLFQDEVAK	6	41322923	2xTMT6plex [N-Term; K11]	1.623	100	100	2.496484995	32.68	0	1778.010021	2	889.508667	0.020193573	1.80E-05	82.78409461
																			LLLEAQAATGFLLDPVKGER	6	41322923	2xTMT6plex [N-Term; K17]	100	100		2.292008638	18.16	1	2599.522344	3	867.1773071	-1.914095216	-0.001658578	98.58884648
																			QLAEGTAQQR	2	41322923	1xTMT6plex [N-Term]	0.523			2.655335665	36.15	0	1330.727643	2	665.8672485	-0.317622986	-0.000211335	16.31965993
																			EAQAVPATLPELEATK	10	41322923	2xTMT6plex [N-Term; K16]	0.983			3.26303339	31.22	0	2126.210906	2	1063.615356	5.893286781	0.006265185	75.02315866
																			VSITEAMHR	12	41322923	1xTMT6plex [N-Term]	0.93	0.849	0.889	3.539974213	48.54	0	1272.693172	3	424.9022217	-0.83301377	-0.00035339	40.24969425
																			DYELQLVTYK	6	41322923	2xTMT6plex [N-Term; K10]	2.491			2.595542669	27.19	0	1729.977659	2	865.4910889	-1.594023837	-0.001378813	66.24837843
																			QAEVELASR	14	41322923	1xTMT6plex [N-Term]	1.096	1.023	1.02	3.304299116	50.54	0	1231.684381	2	616.3460083	0.291155403	0.000179306	34.25804713
																			SLVPAAELLESR	4	41322923	1xTMT6plex [N-Term]	1.001	1.208		3.522369623	38.87	0	1513.878724	2	757.4423218	-0.896383106	-0.000678508	75.33529009
																			QLAEEDAAR	2	41322923	1xTMT6plex [N-Term]		1.162		3.744080782	41.59	0	1231.647996	2	616.3272095	-0.692994013	-0.000426762	26.87640877
																			GYFSEEMNR	11	41322923	1xTMT6plex [N-Term]	0.731	0.906	1.2	3.749310255	44.62	0	1361.635717	2	681.3208008	-1.022055667	-0.000695834	51.76264784
																			QAADAEMEK	2	41322923	2xTMT6plex [N-Term; K9]			0.974	4.437349796	45.22	0	1450.7612	2	725.8843994	0.221758153	0.000160859	36.69540017
																			LAAEQELIR	6	41322923	1xTMT6plex [N-Term]	1.02	0.882	100	3.503214121	52.7	0	1271.752067	2	636.3798828	0.331821753	0.000210998	56.50507914
																			GIYQSLEGAVQAGQLK	10	41322923	2xTMT6plex [N-Term; K16]	100	100		2.636237383	27.95	0	2120.211575	2	1060.60791	-1.429549549	-0.001515474	73.32570445
																			QSAEEQAQAR	6	41322923	1xTMT6plex [N-Term]		100		3.001390696	31.33	0	1346.686172	2	673.8473511	0.930675954	0.000626664	16.75911343
																			SSIAGLLLK	10	41322923	2xTMT6plex [N-Term; K9]	1.466	100		4.127904892	43.41	0	1359.897558	2	680.4523315	-0.12622573	-8.58E-05	88.126762
																			QLAAEEER	12	41322923	1xTMT6plex [N-Term]	1.031	1.045	0.957	3.709287643	68.46	0	1174.626532	2	587.8168335	-0.120708845	-7.09E-05	25.54223234
																			EMSVYEAYR	8	41322923	1xTMT6plex [N-Term]	1.438	1.512	1.448	3.885821104	54.61	0	1376.671768	2	688.8393555	-0.242209151	-0.000166721	44.60077687
																			LSVYAALQR	4	41322923	1xTMT6plex [N-Term]	1.055			3.381951809	29.05	0	1249.746588	2	625.37677	-0.259517362	-0.000162165	68.25252544
																			MVEGYQGLR	6	41322923	1xTMT6plex [N-Term]	1.833	3.405	1.178	3.438977003	46.46	0	1281.682273	2	641.3449097	0.210493617	0.000134893	42.72012092
																			QLAEEDLAQQR	2	41322923	1xTMT6plex [N-Term]	1.585			3.507365942	14.05	0	1529.812101	2	765.4104004	0.930343831	0.000711626	45.67716606
																			LYVHEAVK	17	41322923	2xTMT6plex [N-Term; K8]	0.86	0.984	0.906	4.046372414	49.71	0	1416.861507	2	708.9345093	0.165834619	0.000117482	46.65196145
																			GYFDEEMNR	12	41322923	1xTMT6plex [N-Term]	0.83	0.921	0.869	4.375277519	46.28	0	1389.630631	2	695.3197021	1.076881037	0.000748233	43.40984957
																			QVEEEILALK	8	41322923	2xTMT6plex [N-Term; K10]	1.067	100		2.769417048	36.2	0	1629.982744	2	815.4962158	1.479083526	0.00120544	96.97744521
																			LPLLAVCDYK	7	41322923	2xTMT6plex [N-Term; K10]; 1xCarbamidomethyl [C7	1.501			2.516533136	35.33	0	1649.970071	2	825.4886475	-0.032150962	-2.65E-05	82.56724702
																			IISLETYNLLR	4	41322923	1xTMT6plex [N-Term]	100	100		2.758282661	29.53	0	1563.93076	2	782.4688721	-0.186715028	-0.000146005	97.55707242
																			SEEMQTVQQEQLLQETQALQQSFLSEK	4	41322923	2xTMT6plex [N-Term; K27]				2.235877037	5.32	0	3638.862376	3	1213.62207	-2.945643828	-0.003572931	97.37944823
																			LEDLLQDAQDEK	6	41322923	2xTMT6plex [N-Term; K12]	100		100	1.818243027	23.31	0	1875.011144	2	938.0088501	-0.383984218	-0.000359987	81.37706968
																			KAALEEVER	6	41322923	2xTMT6plex [N-Term; K1]	0.768	0.922	0.788	4.369659424	26.92	1	1502.894264	3	501.6361694	-0.205270075	-0.000102833	37.16321371
																			DVAEVDTVR	6	41322923	1xTMT6plex [N-Term]	0.758	0.97	0.935	3.902173758	54.09	0	1232.668397	2	616.8372192	-1.001959197	-0.000617542	31.91050836
																			AEEAEAQK	12	41322923	2xTMT6plex [N-Term; K8]	1.173	0.958	1.199	3.785338879	53.62	0	1333.736366	2	667.3717651	-0.084219464	-5.62E-05	27.74865458
																			LKAEATEAAR	12	41322923	2xTMT6plex [N-Term; K2]	0.846	0.859	0.724	3.077266693	35.3	1	1517.905163	2	759.4567261	0.667333153	0.000506474	34.09220477
																			LTAEDLFEAR	5	41322923	1xTMT6plex [N-Term]	0.808		100	2.888223648	21	0	1393.752461	2	697.3814697	2.297368589	0.001600982	80.41375529
																			FLQEEAEK	6	41322923	2xTMT6plex [N-Term; K8]	0.991	0.981	1.134	4.004667282	42.31	0	1451.814616	2	726.4117432	1.097542418	0.000796714	50.20568124
																			LTVNEAVK	14	41322923	2xTMT6plex [N-Term; K8]	1.142	1.019	0.945	3.842901468	51.3	0	1331.829872	2	666.4194946	1.381676332	0.000920079	46.70501575
																			LQLEACETR	11	41322923	1xTMT6plex [N-Term]; 1xCarbamidomethyl [C6	1.237	1.102	1.024	3.889783144	47.37	0	1348.709216	2	674.8546143	-5.38610865	-0.003632147	40.83173072
																			AQLMADFQAGR	4	41322923	1xTMT6plex [N-Term]; 1xOxidation [M4]		0.97	1.011	2.042158842	37.4	0	1452.746664	2	718.8804932	1.364402811	0.000980154	60.26671536
																			TEAEIALK	6	41322923	2xTMT6plex [N-Term; K8]	1.412	1.45	1.536	3.716859102	49.96	0	1332.813888	2	666.9094238	-1.73843758	-0.001158507	43.92449486
																			LSVYQAMK	6	41322923	2xTMT6plex [N-Term; K8]	0.863	1.032	0.947	3.180441618	39.52	0	1397.822679	2	699.4152832	0.437213002	0.000305573	58.53508899
																			AGTLSITEFADMLSGNAGGFR	2	41322923	1xTMT6plex [N-Term]				1.320676804	8.34	0	2344.180461	2	1172.593018	-0.726349263	-0.000851347	100.0088601
																			GWLYYEAGQR	8	41322923	1xTMT6plex [N-Term]	0.998	0.743	100	3.409739017	32.71	0	1471.75313	2	736.3804321	0.311212393	0.000229014	57.67929268
																			SELELTLGK	8	41322923	2xTMT6plex [N-Term; K9]	0.87	0.939	1.597	2.794185638	35.14	0	1447.877217	2	724.44104	-1.666620517	-0.001206531	59.6510126
																			QVEVTVHK	22	41322923	2xTMT6plex [N-Term; K8]	1.269	1.117	1.219	3.655986786	51.87	0	1397.851671	2	699.4286499	-1.178490785	-0.000823678	35.65984339
																			QEELQQLEQQR	2	41322923	1xTMT6plex [N-Term]	1.284			2.993887424	17.38	0	1657.870678	2	829.4398193	1.015580948	0.000841851	54.09878275
																			LAEDEAFQR	8	41322923	1xTMT6plex [N-Term]	1.026	1.063	1.22	3.967163086	41.31	0	1307.679296	2	654.3430176	-0.410982839	-0.000268717	48.49082043
																			VPVDVAYQR	7	41322923	1xTMT6plex [N-Term]	0.912	0.892	0.903	3.633316278	30.09	0	1275.725852	2	638.3661499	-0.649908686	-0.000414553	37.43007233
																			FKEMELPAK	4	41322923	3xTMT6plex [N-Term; K2; K9]	0.592			3.55743885	19.86	1	1780.064589	3	594.0257568	-1.051597902	-0.000623971	59.09379399
																			RLEEQAAQHK	3	41322923	2xTMT6plex [N-Term; K10]			0.972	4.303077698	19.36	1	1667.959323	4	417.7453003	0.028736837	1.20E-05	30.85692878
																			VPLDEALQR	12	41322923	1xTMT6plex [N-Term]	1.072	0.79	1.127	3.298638582	26.75	0	1269.736417	2	635.3713989	-0.705436519	-0.000447859	53.31280248
																			RDDGTGQLLLPLSDAR	2	41322923	1xTMT6plex [N-Term]			1.324	3.027326107	20.14	1	1956.071169	3	652.6954346	0.297295388	0.000193844	61.86356932
																			LAAIGEATR	2	41322923	1xTMT6plex [N-Term]			0.959	3.680299997	46.74	0	1130.673088	2	565.8408203	1.128111222	0.000637762	47.77911374
																			GFFDPNTHENLTYLQLLER	5	41322923	1xTMT6plex [N-Term]		0.816		2.562074661	13.38	0	2536.303353	3	846.1073608	1.646403821	0.001391927	88.78179237
																			EAAAATQK	6	41322923	2xTMT6plex [N-Term; K8]	1.023		0.763	3.367313147	38.54	0	1247.735972	2	624.3712769	-0.557032159	-0.000347515	30.2176176
																			LSIYNALK	8	41322923	2xTMT6plex [N-Term; K8]	0.897	1.621	1.723	3.162568331	31.89	0	1379.866258	2	690.4349976	-2.565105706	-0.001769751	64.36839527
																			VKAEAEAAR	6	41322923	2xTMT6plex [N-Term; K2]	0.7	0.558	0.644	2.732027531	16.55	1	1402.841834	3	468.2853394	-0.263246779	-0.000123098	29.02341259
																			AALEEVER	8	41322923	1xTMT6plex [N-Term]	0.861	0.999	0.937	3.484559774	38.36	0	1145.636369	2	573.3215942	-0.398672258	-0.000228367	37.15343815
																			LLFNDVQTLK	12	41322923	2xTMT6plex [N-Term; K10]	1.128	100	1.573	2.152041674	20.08	0	1649.003814	2	825.0046387	-1.099601004	-0.000906623	71.61371522
																			ELAQEQAR	5	41322923	1xTMT6plex [N-Term]		1.043	1.358	2.820463896	30.97	0	1173.642517	2	587.3257446	1.445114495	0.000848024	23.37045911
																			MQAVQEATR	4	41322923	1xTMT6plex [N-Term]	1.037	0.863		3.234050512	37.37	0	1262.672436	2	631.8405151	1.043147375	0.000658577	35.88958917
																			EAEGQLQK	12	41322923	2xTMT6plex [N-Term; K8]	1.242	1.246	1.392	3.917584181	42.06	0	1360.783651	2	680.8972168	2.576782686	0.001753222	32.38060356
																			AQFEQLKDGK	2	41322923	3xTMT6plex [N-Term; K7; K10]			0.45	2.312387705	2.43	1	1851.094309	3	617.7024536	-0.811255347	-0.00050057	45.40686636
																			APVPASELLASGVLSR	18	41322923	1xTMT6plex [N-Term]	0.874	0.888	0.976	2.637322903	26.59	0	1796.047914	2	898.5269165	-0.756116903	-0.000679011	69.54071015
																			KESYSALMR	6	41322923	2xTMT6plex [N-Term; K1]	0.927	0.924	1.051	3.535586834	15.88	1	1542.87142	3	514.961731	-0.505569613	-0.00026001	41.07028164
																			LLEAQACTGGIIDPSTGER	4	41322923	1xTMT6plex [N-Term]; 1xCarbamidomethyl [C7				1.167092443	1.12	0	2217.138262	2	1109.071533	-1.115223075	-0.001236302	56.77850381
																			ELAEEAAR	4	41322923	1xTMT6plex [N-Term]		0.999	1.022	3.348232985	50.14	0	1117.605068	2	559.3066406	0.837648313	0.00046808	30.25603955
																			VPPGYHPLDVEK	10	41322923	2xTMT6plex [N-Term; K12]	1.087	0.994		3.70762825	44.92	0	1809.031091	2	905.0185547	-0.695695616	-0.000629267	53.78940467
																			AELELELGR	12	41322923	1xTMT6plex [N-Term]	0.797	100		3.491680145	31.17	0	1258.720433	2	629.8630371	-1.298875594	-0.000817461	53.59386809
																			VQMEELSK	2	41322923	2xTMT6plex [N-Term; K8]	0.937			3.364141226	33.12	0	1421.807422	2	711.4074097	0.084593691	6.01E-05	52.46498938
																			AFCGFEDPR	6	41322923	1xTMT6plex [N-Term]; 1xCarbamidomethyl [C3	1.038	0.897		3.038888931	33.17	0	1327.630238	2	664.3184814	-0.415318482	-0.000275695	43.78761611
																			QITMEELVR	6	41322923	1xTMT6plex [N-Term]	0.919		100	3.512044191	26.42	0	1347.750352	2	674.3781738	-0.950764916	-0.000640697	54.93325495
																			LQAEEAER	4	41322923	1xTMT6plex [N-Term]	0.984	1.028		2.688658714	45.37	0	1174.626532	2	587.815979	-1.575626071	-0.000925386	31.46887835
																			TELATQEK	4	41322923	2xTMT6plex [N-Term; K8]	1.023	1.202		3.769670963	45.15	0	1377.798966	2	689.4025269	-0.863064273	-0.000594565	39.93451646
																			LHVAILER	6	41322923	1xTMT6plex [N-Term]	0.975	1.299	1.032	2.854303122	40.39	0	1179.741108	2	590.3739624	-0.390174852	-0.000230153	46.33245324
																			ELYQQLQR	8	41322923	1xTMT6plex [N-Term]	1.305	1.223	1.197	3.076324463	35.42	0	1306.731666	2	653.8704224	1.45568261	0.000951093	42.02497103
																			QTLSIYQALK	4	41322923	2xTMT6plex [N-Term; K10]		100	100	2.209670067	18.73	0	1622.988164	2	811.9981689	0.552906451	0.00044868	60.72427437
																			DLLPSDMAVALLEAQAGTGHIIDPATSAR	3	41322923	1xTMT6plex [N-Term]; 1xOxidation [M7]				1.007540703	4.91	0	3178.661543	3	1054.891724	-1.90053593	-0.002003587	103.9443819
																			LKQSAEEQAQAR	2	41322923	2xTMT6plex [N-Term; K2]	0.182			2.920006752	19.15	1	1817.028131	3	606.3480835	0.861928473	0.000522049	35.71819165
																			VLALPEPSPAAPTLR	10	41322923	1xTMT6plex [N-Term]	0.832	0.831	1.076	2.675418615	21.42	0	1761.047186	2	881.0273438	0.127594537	0.00011235	61.21173038
																			LQLEATER	10	41322923	1xTMT6plex [N-Term]	0.893	0.946	0.969	2.981302261	51.6	0	1188.678568	2	594.8434448	0.879370139	0.000522644	40.01518746
																			VSIYEAMR	9	41322923	1xTMT6plex [N-Term]	0.388	1.137	1.003	3.190891743	34.93	0	1197.64991	2	599.3284912	-0.170674354	-0.000102204	56.77018704
																			EAIAELER	8	41322923	1xTMT6plex [N-Term]	0.965	1.024	1.033	3.187807083	47.44	0	1159.652019	2	580.3296509	0.00559462	3.24E-06	43.25754889
																			QLLEEELAR	10	41322923	1xTMT6plex [N-Term]	100	1.367	100	2.870892763	39.16	0	1329.757546	2	665.3823242	-0.131191209	-8.72E-05	52.99897488
																			LLLEAQAATGFLLDPVK	5	41322923	2xTMT6plex [N-Term; K17]			100	1.442302346	4.4	0	2257.357176	2	1129.178955	-2.898377722	-0.003271337	106.0311782
																			AEEAEAQKR	2	41322923	2xTMT6plex [N-Term; K8]			0.692	3.067323923	27.98	1	1489.837477	3	497.283844	-0.334403977	-0.000166069	26.58498407
																			QVAEEAAR	6	41322923	1xTMT6plex [N-Term]	1.805	0.81	0.602	3.214732885	42.99	0	1102.605403	2	551.8066406	0.54578909	0.000300895	20.38344301
																			LTVDEAVR	14	41322923	1xTMT6plex [N-Term]	0.951	0.98		3.011199713	44.85	0	1131.657104	2	566.3321533	-0.06541679	-3.70E-05	48.40331377
																			SYVDPSTDER	2	41322923	1xTMT6plex [N-Term]		1.194		3.274896622	45.62	0	1397.674604	2	699.3395996	-1.918780839	-0.001340916	31.03649944
																			GLVEDTLR	5	41322923	1xTMT6plex [N-Term]	0.764	0.685		3.156651735	32.79	0	1131.657104	2	566.3328247	1.121138248	0.000634372	54.42803355
																			LTVEEAVR	8	41322923	1xTMT6plex [N-Term]	1.049	1.08		3.084803581	32.96	0	1145.672754	2	573.3399048	-0.193039144	-0.00011058	50.68133318
																			LSYTQLLR	8	41322923	1xTMT6plex [N-Term]	0.913	1.069	100	2.887237549	43.38	0	1222.735689	2	611.8709106	-0.935648602	-0.000572025	66.26841838
																			ILITIVEEVETLR	6	41322923	1xTMT6plex [N-Term]			0.866	1.946662188	18.49	0	1757.062167	2	879.0332031	-1.7289485	-0.001518935	117.5441691
																			KELIPTEEALR	4	41322923	2xTMT6plex [N-Term; K1]	0.846	0.799		4.170377254	31.29	1	1757.057306	3	586.3569946	-0.498204173	-0.000291791	61.25879252
																			GKAEEQAVR	5	41322923	2xTMT6plex [N-Term; K2]	0.743	0.921		2.499674559	16.45	1	1445.847648	3	482.6206665	-0.139349593	-6.72E-05	14.97951406
																			GYFDEEMNR	6	41322923	1xTMT6plex [N-Term]; 1xOxidation [M7]	0.745		0.514	2.21927762	38.41	0	1405.625546	2	695.3197021	1.076881037	0.000748233	43.40984957
																			LVASMEEAR	7	41322923	1xTMT6plex [N-Term]; 1xOxidation [M5]	0.865	1.054	0.907	2.412864208	31.14	0	1250.661203	2	617.8307495	-9.772759388	-0.006033048	46.17539441
																			HISDLYEDLRDGHNLISLLEVLSGDSLPR	2	41322923	1xTMT6plex [N-Term]				2.885494471		1	3505.848826	4	877.2164307	-1.407238017	-0.001233391	105.9994727
																			SIQEELQQLR	3	41322923	1xTMT6plex [N-Term]	100			1.601855159	14.11	0	1472.827023	2	736.9170532	-0.130982583	-9.65E-05	60.52609112
																			LKAEAELLQQQK	11	41322923	3xTMT6plex [N-Term; K2; K12]	1.199	0.643	0.843	2.388824463	10.88	1	2086.283901	3	696.0991211	-0.522852498	-0.000363606	53.11044745
																			MGIVGPEFK	4	41322923	2xTMT6plex [N-Term; K9]	1.395	0.672		3.084182978	29.27	0	1435.838329	2	718.4232178	0.578217518	0.000415113	72.81683707
																			KDLLPSDMAVALLEAQAGTGHIIDPATSAR	4	41322923	2xTMT6plex [N-Term; K1]				1.945028901	18.2	1	3519.924523	4	880.7366333	0.051124143	4.50E-05	90.23505429
																			LDLQYAK	6	41322923	2xTMT6plex [N-Term; K7]	1.044	0.974	1.043	3.231675386	43.57	0	1308.792759	2	654.899353	-1.015665241	-0.000664648	56.82899146
																			TPVEVPVGGFK	6	41322923	2xTMT6plex [N-Term; K11]	1.452	0.846	0.824	3.13785696	35.21	0	1587.95105	2	794.4796143	0.567861097	0.000450868	54.86652699
																			ESYSALMR	2	41322923	1xTMT6plex [N-Term]			1.044	3.09669137	27.5	0	1185.613525	2	593.3108521	0.761455182	0.000451396	48.00726605

Bhat et al,2017. Cigarette smoke and chewing tobacco alter expression of different sets of miRNAs in oral keratinocytes

Supplementary Table S2(A). List of all proteins and peptides identified in OKF6/TERT1 cells chronically treated with chewing tobacco using Sequest and Mascot search algorithms

Accession	Gene_Symbol	Description	Gene_ID	Refseq_Accession	Coverage	Proteins	#Unique_Peptides	#Peptides	#PSMS	#AAs	MW [kDa]	calc. pI	OKF6-STE-6M/OKF6P_R1	OKF6-STE-6M/OKF6P_R2	OKF6-STE-6M/OKF6P_R3	Median fold change	Status	P-value (6M)	Sequence	#PSMSs	Protein Accessions	Modifications	OKF6-STE-6M/OKF6P_R1	OKF6-STE-6M/OKF6P_R2	OKF6-STE-6M/OKF6P_R3	Xcorr	Ionscore	# Missed Cleavages	Theo. MH+ [Da]	Charge	m_z	DeltaM [ppm]	Deltam/z [Da]	RT [min]
																			CVEDPETGLCLLPLTDK	2	41322923	2xTMT6plex [N-Term; K17]; 2xCarbamidomethyl [C1; C10				1.609672308	3	0	2418.266055	2	1209.634521	-1.773357915	-0.002144226	78.41218894
																			ATVSAPFGK	2	41322923	2xTMT6plex [N-Term; K9]	0.692			3.127102137	19.11	0	1335.803658	2	668.4060059	0.806509807	0.000538669	53.57685593
																			GANVIAGVWLEEAGQK	2	41322923	2xTMT6plex [N-Term; K16]				1.807765245	10.36	0	2100.18536	2	1050.594849	-1.399530932	-0.001469637	84.27136315
																			VQSGSESVIQEYVDLR	4	41322923	1xTMT6plex [N-Term]			100	1.515907288	6.65	0	2038.065415	2	1019.534851	-1.46671522	-0.001494631	68.77373229
																			SWSLATFR	3	41322923	1xTMT6plex [N-Term]	100			1.963997483	27.65	0	1196.662524	2	598.8344727	-0.714593699	-0.000427564	52.29172401
																			GYFSEEMNR	4	41322923	1xTMT6plex [N-Term]; 1xOxidation [M7]		0.898		1.980423927	26.29	0	1377.630632	2	681.3208008	-1.022055667	-0.000695834	51.76264784
																			AQFEQLK	4	41322923	2xTMT6plex [N-Term; K7]		0.974	0.957	3.387481689	43.67	0	1321.788008	2	661.3978271	0.279906364	0.000184988	52.19253207
																			IKELQNAGDR	4	41322923	2xTMT6plex [N-Term; K2]		0.665	0.653	2.807488441	12.96	1	1601.937525	3	534.6502686	-0.794591498	-0.000424295	32.63782798
																			FLEVQYLTGGLIEPDTPGR	3	41322923	1xTMT6plex [N-Term]				1.590105534	4.9	0	2334.254278	2	1167.630615	-0.138914279	-0.000162131	93.45110976
																			KEAEQEAAR	2	41322923	2xTMT6plex [N-Term; K1]	1.443			2.392676353	15.89	1	1489.837477	3	497.2839966	-0.027146522	-1.35E-05	26.85089296
																			FISETLR	5	41322923	1xTMT6plex [N-Term]	1.093	1.168	1.507	2.797239304	32.12	0	1094.640726	2	547.8239136	-0.160081347	-8.76E-05	58.33265825
																			TQLASWSDPTEETGPVAGILDTETLEK	3	41322923	2xTMT6plex [N-Term; K27]				1.503997564		0	3346.730617	3	1116.242676	-5.122398009	-0.005714429	92.3597754
																			AGVAAPATQVAQVTLQSVQR	3	41322923	1xTMT6plex [N-Term]	100			1.077830195		0	2224.261095	2	1112.632935	-1.125016025	-0.001251165	56.37001485
																			DVGAYSK	4	41322923	2xTMT6plex [N-Term; K7]		0.959	0.897	3.115622997	28.29	0	1197.687959	2	599.3477173	0.165828095	9.93E-05	34.98184302
																			EGLTSIEEVTK	3	41322923	2xTMT6plex [N-Term; K11]				1.814509988	6.71	0	1663.951838	2	832.4712524	-9.982030014	-0.008304809	55.41794682
																			DPYTGQQISLFQAMQK	3	41322923	2xTMT6plex [N-Term; K16]		0.01	0.01	1.512784719	4.8	0	2313.231324	2	1157.118164	-0.982281788	-0.001136122	73.30884446
																			LLDPEDVDVPQPDEK	4	41322923	2xTMT6plex [N-Term; K15]	100	0.274		1.571609735	10.19	0	2167.153451	2	1084.079468	-0.826717243	-0.000895812	62.47923783
																			LCFEGLR	6	41322923	1xTMT6plex [N-Term]; 1xCarbamidomethyl [C2	1.244	1.211	100	2.685109854	24.75	0	1123.613131	2	562.3103638	0.284598901	0.00015989	59.83962279
																			ASDSELER	2	41322923	1xTMT6plex [N-Term]	1.286			2.580566406	28.97	0	1135.579248	2	568.2936401	0.665777786	0.000378022	30.54205926
																			RFPSSFEEIEILWSQFLK	2	41322923	2xTMT6plex [N-Term; K18]				2.17956233	5.28	1	2714.495795	3	905.5004883	-3.272499544	-0.002961062	111.9941712
																			SMVEEGTGLR	2	41322923	1xTMT6plex [N-Term]; 1xOxidation [M2]	0.799			2.397660494	29	0	1323.677582	2	654.3392334	-8.776298269	-0.005738307	39.23365068
																			EQLNEYK	4	41322923	2xTMT6plex [N-Term; K7]	1.138	1.09		3.092662573	37.82	0	1381.772751	2	691.3901978	0.265881501	0.000183694	47.22682005
																			MQEEVVR	4	41322923	1xTMT6plex [N-Term]	1.081	1.169		2.646664143	24.98	0	1119.60296	2	560.3049316	-0.333483175	-0.000186684	38.91645323
																			KFAEQTLR	2	41322923	2xTMT6plex [N-Term; K1]	0.992			2.452953815	14.85	1	1450.87822	3	484.2969666	-1.291095125	-0.000624407	45.40517589
																			LSAEAEK	6	41322923	2xTMT6plex [N-Term; K7]	1.04	1.01	0.976	3.238425255	31.02	0	1205.714174	2	603.3550415	-9.42816501	-0.005683836	36.45747315
																			EMELPAK	2	41322923	2xTMT6plex [N-Term; K7]	1.148			2.754300833	23.64	0	1275.73828	2	638.3726196	-0.249033985	-0.000158851	54.89764034
																			LLLWSQR	8	41322923	1xTMT6plex [N-Term]	1.179	1.055	0.405	2.313713074	22.33	0	1144.703995	2	572.8561401	0.88137496	0.000504457	95.11755843
																			LEAEAGR	2	41322923	1xTMT6plex [N-Term]			1.178	2.441844463	29.59	0	974.5468254	2	487.7767944	-0.526555317	-0.000256576	31.82923437
																			ELEQLGR	2	41322923	1xTMT6plex [N-Term]	0.905			2.513141155	41.91	0	1073.615239	2	537.3109131	-0.642425796	-0.000344859	41.95144552
																			TSSEDNLYLAVLR	2	41322923	1xTMT6plex [N-Term]				1.642982244	6.27	0	1709.927131	2	855.4664917	-0.832673824	-0.000711906	83.18320015
																			ANELQLR	1	41322923	1xTMT6plex [N-Term]	1.583			2.611222982	37.65	0	1072.631224	2	536.8193359	0.159938473	8.58E-05	41.21452817
																			VPQPEGLGR	2	41322923	1xTMT6plex [N-Term]	1.021			2.550184488	25.88	0	1181.683988	2	591.3455811	-0.086334947	-5.10E-05	41.28361026
																			MQAVQEATR	2	41322923	1xTMT6plex [N-Term]; 1xOxidation [M1]	0.824			2.330215216	22.76	0	1278.667351	2	631.8405151	1.043147375	0.000658577	35.88958917
																			LISLFQAMK	2	41322923	2xTMT6plex [N-Term; K9]				1.374297142	2.12	0	1508.927478	2	754.9661255	-1.659240403	-0.001251837	98.38169778
																			SIITYVSSLYDAMPR	1	41322923	1xTMT6plex [N-Term]				1.080153227		0	1945.030215	2	973.0171509	-1.640212148	-0.001595131	100.5784243
																			ALAEEAK	1	41322923	2xTMT6plex [N-Term; K7]			0.871	3.142791271	34.07	0	1189.719259	2	595.3632202	-0.080397381	-4.78E-05	38.12928192
																			VAQLLER	2	41322923	1xTMT6plex [N-Term]	1.092			2.705996037	28.33	0	1057.65671	2	529.3325195	0.994890393	0.000526126	53.2346346
61743954	AHNAK	neuroblast differentiation-associated protein AHNAK isoform 1	79026	NP_001611.1	63.7860781	1	219	219	1497	5890	628.699	6.15	1.01	1.02	1.03	1.02	Significant	0.000032																
																			AVEVQGPSLESGDHGK	10	61743954	2xTMT6plex [N-Term; K16]	0.849	0.927	0.842	5.226090431	68.27	0	2068.107503	3	690.0407104	0.036080163	2.49E-05	40.43314088
																			GPHVDVSGPDIDIEGPEGK	8	61743954	2xTMT6plex [N-Term; K19]	0.807	100		6.006889343	47.53	0	2376.244725	3	792.7544556	1.720516905	0.00136279	57.99071605
																			IEGEMQVPDVDIR	6	61743954	1xTMT6plex [N-Term]	1.128	100	1.296	3.500826359	45.02	0	1729.899201	2	865.4541016	0.997292212	0.000862608	67.67232353
																			VKGDVDVSVPK	16	61743954	3xTMT6plex [N-Term; K2; K11]	0.948	0.931	0.933	3.984330654	34.52	1	1830.13036	2	915.5687256	-0.101576439	-9.29E-05	43.22063294
																			LPSGSGAASPTGSAVDIR	8	61743954	1xTMT6plex [N-Term]	1.382	1.287	1.32	4.879358292	73.38	0	1872.002421	2	936.5062866	1.536286575	0.001437966	46.7501102
																			GEGPEVDMNLPK	8	61743954	2xTMT6plex [N-Term; K12]	1.271	0.723	0.796	3.22611022	51.92	0	1743.935142	2	872.4714966	0.329464123	0.000287282	53.48360953
																			GPAFNMASPESDFGINLK	17	61743954	2xTMT6plex [N-Term; K18]	0.889	0.694	0.527	3.710375786	38.21	0	2353.226238	3	785.0805054	0.307880844	0.000241504	96.05415569
																			GKGGVTGSPEASISGSK	4	61743954	3xTMT6plex [N-Term; K2; K17]	0.628	0.589		5.760925293	34.24	1	2206.264622	3	736.0922241	-1.134377397	-0.000834246	37.76113777
																			VKGDVDVTLPK	8	61743954	3xTMT6plex [N-Term; K2; K11]	0.968	1.06	0.969	4.111862659	26	1	1858.161661	3	620.059021	0.456993759	0.000283056	47.86037744
																			VTAYTVDVTGR	8	61743954	1xTMT6plex [N-Term]	1.181	1.055	1.306	4.052669048	48.08	0	1410.77901	2	705.8928223	-0.455102284	-0.000321024	46.11487508
																			GSEVGFHGAAPDISVK	6	61743954	2xTMT6plex [N-Term; K16]	0.977	0.96	1.003	4.612439156	50	0	2029.111861	3	677.0358276	-9.329692567	-0.00631033	47.50869088
																			GEGPEVDVNLPK	8	61743954	2xTMT6plex [N-Term; K12]	1.01	1.254	0.996	3.725242138	60.54	0	1711.963071	2	856.4853516	0.207495714	0.000177613	50.53863016
																			GEGPDVDVNLPK	10	61743954	2xTMT6plex [N-Term; K12]	1.137	1.231	1.049	3.3883605	53.41	0	1697.947421	2	849.4779663	0.727217564	0.000617389	55.21905283
																			VDISAPDVDVHGPDWHLK	4	61743954	2xTMT6plex [N-Term; K18]	1.295	1.067		4.443796158	31.91	0	2458.313079	4	615.3335571	-0.27694779	-0.000170206	76.30000295
																			VDINAPDVDVR	8	61743954	1xTMT6plex [N-Term]	1.395	1.407	1.194	4.552298069	79.99	0	1441.784824	2	721.3892822	-9.388194863	-0.006767878	48.78299284
																			VDIEGPDVNIEGPEGK	7	61743954	2xTMT6plex [N-Term; K16]	1.269	100	100	3.663486242	55.95	0	2126.138135	2	1063.572021	-0.643660554	-0.000684256	73.44888628
																			GGVTGSPEASISGSK	8	61743954	2xTMT6plex [N-Term; K15]	1.215	1.422	1.532	4.939518452	66.63	0	1791.985263	2	896.49646	0.212112166	0.000190051	29.34377008
																			SPSLDVTVPEAELNLETPEISVGGK	4	61743954	2xTMT6plex [N-Term; K25]	0.594			3.361674547	19.87	0	3039.650182	3	1013.887634	-0.602966742	-0.000610936	90.658077
																			ELLLPNWQGSGSHGLTIAQR	6	61743954	1xTMT6plex [N-Term]	0.788	0.845	0.562	4.903656006	64.29	0	2406.309108	3	802.7744141	-0.173997804	-0.000139564	78.79391913
																			VKGDLDIAGPNLEGDFK	2	61743954	3xTMT6plex [N-Term; K2; K17]	1.364			3.638436556	17.96	1	2475.406201	3	825.8076172	0.847413447	0.000699231	76.85071746
																			VDIDAPDVDVHGPDWHLK	11	61743954	2xTMT6plex [N-Term; K18]	1.247	1.23	1.012	5.435830116	28.58	0	2486.307994	4	622.3324585	0.004039876	2.51E-06	72.03917783
																			GPQVSSALNLDTSK	12	61743954	2xTMT6plex [N-Term; K14]	0.945	1.108	1.426	4.065683842	64.01	0	1875.058762	2	938.0321045	-0.976025745	-0.000915053	60.32347174
																			VDINAPDVEVQGK	4	61743954	2xTMT6plex [N-Term; K13]	1.345	1.45		3.803865671	56.25	0	1842.037299	2	921.5218506	-0.474598492	-0.000437114	61.00496247
																			GGQIGLQAPGLSVSGPQGHLESGSGK	5	61743954	2xTMT6plex [N-Term; K26]	1.106	1.316		7.180103302	99.59	0	2876.563039	3	959.5266113	0.779212673	0.000747151	61.34171372
																			LNVGAPDVTLR	10	61743954	1xTMT6plex [N-Term]	1.157	1.016		3.639765501	55.68	0	1383.81573	2	692.411499	-0.006086883	-4.21E-06	65.18542377
																			LPQFGISTPGSDLHVNAK	12	61743954	2xTMT6plex [N-Term; K18]	1.141	100	100	3.564603806	31.95	0	2339.312351	3	780.4436646	1.748023235	0.001363057	69.17144677
																			AGAISASGPELQGAGHSK	6	61743954	2xTMT6plex [N-Term; K18]	1.423	1.373		6.249421597	54.69	0	2096.150037	3	699.3884888	0.417936969	0.00029202	38.12372188
																			LKSEDGVEGDLGETQSR	9	61743954	2xTMT6plex [N-Term; K2]	0.893	0.837	0.621	4.815243244	35.86	1	2278.192689	3	760.0682373	-1.110896347	-0.000843612	45.98099235
																			GHYEVTGSDDETGK	8	61743954	2xTMT6plex [N-Term; K14]	1.025	1.043	1.111	4.521501541	70.08	0	1952.960171	3	651.657959	-0.433632435	-0.000282289	34.49497652
																			VDVSAPDVEAHGPEWNLK	10	61743954	2xTMT6plex [N-Term; K18]	1.149	0.742	1.647	4.988375664	50.08	0	2421.281445	3	807.7650146	-0.394119688	-0.000318092	65.30130943
																			VDVEGPDVNIEGPEGK	10	61743954	2xTMT6plex [N-Term; K16]	1.929	1.556	100	3.409388304	49.07	0	2112.122485	2	1056.562988	-1.791968755	-0.001892429	60.50060921
																			GGADVSGGVSAPDISLGEGHLSVK	4	61743954	2xTMT6plex [N-Term; K24]	1.449		100	5.82490921	70.67	0	2667.435379	3	889.8165894	-0.061637329	-5.48E-05	63.35520874
																			GEETGIDVTLPTGEVTVPGVSGDVSLPEIATGGLEGK	5	61743954	2xTMT6plex [N-Term; K37]			100	4.590123177	31.13	0	4039.137472	4	1010.539063	-0.755492979	-0.000762885	101.0984977
																			GEGPEFDVNLSK	4	61743954	2xTMT6plex [N-Term; K12]	0.968	1.073		3.740505695	40.08	0	1749.942336	2	875.4751587	0.402837738	0.000352471	62.79025311
																			HELTEISNVDVETQSGK	6	61743954	2xTMT6plex [N-Term; K17]	1.138	1.1		6.220626354	61.21	0	2344.23964	3	782.0853882	0.841049161	0.000657207	46.01175325
																			VHAPGLNLSGVGGK	4	61743954	2xTMT6plex [N-Term; K14]			1.014	4.316561699	24.24	0	1764.053224	3	588.68927	0.01875714	1.10E-05	56.44606065
																			IKGDVDVSAPK	14	61743954	3xTMT6plex [N-Term; K2; K11]	1.012	1.011	0.988	4.678144932	31.17	1	1816.11471	3	606.0429688	-0.19678823	-0.00011913	42.03066538
																			SEDGVEGDLGETQSR	4	61743954	1xTMT6plex [N-Term]		1.362	1.167	4.264303207	49.78	0	1807.850731	2	904.4281006	-0.999008433	-0.000903029	47.40124792
																			GDVPSVGLEGPDVDLQGPEAK	4	61743954	2xTMT6plex [N-Term; K21]			1.21	2.957235336	45.5	0	2537.349918	3	846.4473267	-8.864208822	-0.0074972	63.47987473
																			AKGEVDVDVPK	12	61743954	3xTMT6plex [N-Term; K2; K11]	0.925	0.948		3.792345047	25.06	1	1844.109625	3	615.3740234	-1.143059757	-0.000702642	40.28522985
																			GGKPGLTIQAPQLEVSVPSANIEGLEGK	5	61743954	3xTMT6plex [N-Term; K3; K28]			1.136	3.934575081	40.8	0	3476.993144	4	870.0024414	-1.498031426	-0.001302161	79.50061206
																			DDGVFVQEVTQNSPAAR	2	61743954	1xTMT6plex [N-Term]		1.225		2.926821709	27.09	0	2062.040263	2	1031.523193	-0.558947015	-0.000576286	64.42962328
																			FKAEAPLPSPK	8	61743954	3xTMT6plex [N-Term; K2; K11]	0.702	0.741	0.742	4.982645035	32.05	1	1872.156181	3	624.7224121	-1.868557714	-0.001166077	48.15249608
																			LKTDVDVSLPK	8	61743954	3xTMT6plex [N-Term; K2; K11]	0.963	0.978	0.966	3.782135248	16.16	1	1902.187875	3	634.7332153	-1.462812345	-0.000927515	66.040805
																			VDIDVPDVDVQGPDWHLK	16	61743954	2xTMT6plex [N-Term; K18]	1.476	1.029	1.346	3.278748035	37.53	0	2505.33896	3	835.7854004	1.072953368	0.000896037	80.44289521
																			LEGDLTGPSVDVEVPDVELECPDAK	12	61743954	2xTMT6plex [N-Term; K25]; 1xCarbamidomethyl [C21	0.343	100	100	2.360610247	13.58	0	3141.591347	3	1047.867554	-1.031088016	-0.001079752	82.92098444
																			KQGFDLNVPGGEIDASLK	2	61743954	3xTMT6plex [N-Term; K1; K18]	0.837			2.781425953	25.03	1	2575.469863	3	859.1604614	-1.177428903	-0.001010811	81.30252615
																			GPSLDIDTPDVNIEGPEGK	2	61743954	2xTMT6plex [N-Term; K19]			100	3.316150665	26.02	0	2411.270605	3	804.4276123	-0.962880704	-0.000773922	70.61250731
																			VKGDADVSVPK	6	61743954	3xTMT6plex [N-Term; K2; K11]	0.968	1.094	1.004	3.747444868	27.11	1	1802.09906	3	601.3717041	0.831654996	0.000499575	30.46515906
																			VDINTPDVDVHGPDWHLK	8	61743954	2xTMT6plex [N-Term; K18]	0.848	0.217	1.471	3.054155827	21.95	0	2515.334543	4	629.59021	1.775832866	0.001116703	50.63527
																			VDINAPDVEVHGPDWHLK	6	61743954	2xTMT6plex [N-Term; K18]	1.013	2.007	1.713	2.933770657	20.64	0	2499.339629	4	625.5900269	-0.540546034	-0.000337752	57.39601847
																			ISMPDFDLHLK	16	61743954	2xTMT6plex [N-Term; K11]	1.217	1.149	1.314	3.318145037	34.88	0	1773.997348	3	592.0040283	0.103354685	6.11E-05	87.91292301
																			VQANLGAPDINIEGLDAK	4	61743954	2xTMT6plex [N-Term; K18]			0.01	2.34761405	30.96	0	2296.291281	2	1148.647705	-1.370846885	-0.001573932	70.8748188
																			VKGDVDISLPK	7	61743954	3xTMT6plex [N-Term; K2; K11]	0.887	0.929	0.937	4.40493536	21.34	1	1858.161661	3	620.059082	0.555534953	0.000344091	53.68595384
																			GEGPEVDVTLPK	6	61743954	2xTMT6plex [N-Term; K12]	0.455	0.846	0.994	2.786386251	46.93	0	1698.967822	2	849.9888306	1.508093784	0.001281101	54.59764817
																			KPDIDITGPK	10	61743954	3xTMT6plex [N-Term; K1; K10]	1.212	1.092	1.174	3.88612318	43.16	0	1771.093247	3	591.0360718	0.234534253	0.000138461	46.04951421
																			VSVGAPDLSLEASEGSIK	6	61743954	2xTMT6plex [N-Term; K18]		2.648	0.507	2.470193386	33.11	0	2217.237849	2	1109.122437	-0.113845759	-0.000126212	68.34055208
																			VNVEAPDVNLEGLGGK	6	61743954	2xTMT6plex [N-Term; K16]	1.146	0.915	1.525	3.255768299	42.83	0	2069.16429	2	1035.085205	-0.558945279	-0.000578277	70.08773037
																			VEGDLKGPEADIK	6	61743954	3xTMT6plex [N-Term; K6; K13]	0.872	0.984	0.895	3.327507257	20.68	1	2058.204982	3	686.7401123	0.38958902	0.000267285	46.4180511
																			GEGPDVHMTLPK	8	61743954	2xTMT6plex [N-Term; K12]	0.994	1.018	1.01	3.243128538	33.82	0	1738.956212	2	869.982666	1.060176925	0.000921801	49.63439474
																			GSLGATGEIK	8	61743954	2xTMT6plex [N-Term; K10]	0.995	1.187	1.157	4.280141354	72.2	0	1390.830601	2	695.9182129	-1.043656035	-0.000725774	40.41141141
																			VKGDVDVSLPK	18	61743954	3xTMT6plex [N-Term; K2; K11]	1.032	0.996	1.054	4.243915081	32.15	1	1844.146011	2	922.5768433	0.216573653	0.000199697	47.81776932
																			GPEVDIEGPEGK	2	61743954	2xTMT6plex [N-Term; K12]			0.795	3.66969943	60.02	0	1684.915787	2	842.9620972	0.671241819	0.000565493	51.98116046
																			LEGELQAPDLELSLPAIHVEGLDIK	4	61743954	2xTMT6plex [N-Term; K25]	100			2.742802382	17.27	0	3157.776052	3	1053.263062	-0.449775305	-0.00047343	94.31260347
																			APEVNLNAPDVDVHGPDWNLK	8	61743954	2xTMT6plex [N-Term; K21]	3.142	1.135	0.886	4.037738323	43.89	0	2758.456449	3	920.1502075	-7.388099752	-0.00679325	66.21277531
																			VDIDTPDINIEGSEGK	4	61743954	2xTMT6plex [N-Term; K16]	1.115	100		2.480401039	28.77	0	2160.143614	2	1080.574463	-0.909642645	-0.000982479	60.03157433
																			GPEVDVSGPK	6	61743954	2xTMT6plex [N-Term; K10]	0.861	0.913	0.905	4.164059162	55.56	0	1442.825515	2	721.9164429	0.065019773	4.69E-05	44.74880905
																			VDIDAPDVSIEGPDAK	6	61743954	2xTMT6plex [N-Term; K16]	1.406	1.227	2.072	2.714724541	40.25	0	2099.127236	2	1050.067139	-0.112001905	-0.000117553	75.13767071
																			VDINAPDVDVQGPDWHLK	10	61743954	2xTMT6plex [N-Term; K18]	0.784	100	1.124	2.878123283	19.22	0	2476.323644	3	826.1140747	1.626100491	0.00134225	60.50652269
																			ADIDISGPNVDVDVPDVNIEGPDAK	6	61743954	2xTMT6plex [N-Term; K25]				2.336512327	14.94	0	3022.562096	3	1008.191895	-0.319370196	-0.000321772	76.734704
																			VDVSAPDVEMQGPDWNLK	9	61743954	2xTMT6plex [N-Term; K18]	1.795	2.66	1.147	3.649957657	45.63	0	2458.268831	3	820.0958862	1.738655005	0.001424694	67.74461696
																			VDINAPDVDVHGPDWHLK	6	61743954	2xTMT6plex [N-Term; K18]	100	1.831	0.884	2.933079243	26.15	0	2485.323978	4	622.0854492	-1.614073269	-0.001002874	59.74152187
																			GDLDASVPSMK	8	61743954	2xTMT6plex [N-Term; K11]	1.479	0.547	0.967	2.878717969	33.7	0	1577.860914	2	797.4317017	0.186478794	0.00014861	40.3504828
																			VDVNAPDVQAPDWHLK	6	61743954	2xTMT6plex [N-Term; K16]	0.994	2.348	0.884	3.545465469	35.29	0	2262.228287	3	754.7460938	-2.015402259	-0.001519767	60.38748836
																			MDIDAPDVDVHGPDWHLK	5	61743954	2xTMT6plex [N-Term; K18]		2.022	0.753	2.750156403	20.07	0	2518.280065	4	630.3255615	0.139561026	8.79E-05	73.35677879
																			LPTGQISGPEIK	6	61743954	2xTMT6plex [N-Term; K12]	1.15	0.97	1.321	4.082183838	39.12	0	1698.020192	2	849.5122681	-1.727156839	-0.001466374	55.58652023
																			MKGDVDVSLPK	2	61743954	3xTMT6plex [N-Term; K2; K11]				4.012805462		1	1876.118081	3	626.0437622	-0.718419016	-0.00044928	48.36855544
																			VDIDVPDVNVQGPDWHLK	8	61743954	2xTMT6plex [N-Term; K18]	100	100	100	2.194995165	21.7	0	2504.354944	3	835.4545898	-2.28719925	-0.00190932	77.06374706
																			VKGDMDVSVPK	14	61743954	3xTMT6plex [N-Term; K2; K11]	0.901	0.947	0.968	4.40498209	26.65	1	1862.102431	2	931.5549316	0.0835138	7.78E-05	47.53628699
																			VKGEYDVTVPK	6	61743954	3xTMT6plex [N-Term; K2; K11]	0.922	1.023	0.99	3.682578325	21.44	1	1922.156575	3	641.3901978	-0.278415551	-0.000178386	44.80196396
																			VKGDMDVSLPK	10	61743954	3xTMT6plex [N-Term; K2; K11]	0.963	0.961	1	4.460400105	19.15	1	1876.118081	3	626.0443115	0.159963446	0.000100037	54.0912211
																			VDIDVPDVNIEGPDAK	6	61743954	2xTMT6plex [N-Term; K16]	1.222	0.96	100	2.385206223	29.22	0	2154.169435	2	1077.586426	-1.791891314	-0.001930019	68.840473
																			FAGGLHFSGPK	12	61743954	2xTMT6plex [N-Term; K11]	1.011	1.04	1.103	3.276579618	38.5	0	1575.904769	3	525.9764404	6.345129008	0.003333106	55.16147351
																			IKGDMDISVPK	4	61743954	3xTMT6plex [N-Term; K2; K11]	1.058	0.929		3.325686455	15.99	1	1890.133731	3	630.715271	-1.307596218	-0.000823844	55.58809856
																			SSGCDVNLPGVNVK	6	61743954	2xTMT6plex [N-Term; K14]; 1xCarbamidomethyl [C4	100	100	100	2.839604139	20.7	0	1904.031168	2	952.5198364	0.645147823	0.000614191	55.46358561
																			MKGDYDVTVPK	4	61743954	3xTMT6plex [N-Term; K2; K11]	0.937	0.882		3.393996716	18.34	1	1940.112996	3	647.3759766	0.196188315	0.000126876	45.87390804
																			MPDMHVNMPK	6	61743954	2xTMT6plex [N-Term; K10]	0.979	0.937	0.938	2.552508831	38.58	0	1657.862846	3	553.2920532	-0.747457508	-0.000413061	50.61616629
																			VDINAPDVGVQGPDWHLK	6	61743954	2xTMT6plex [N-Term; K18]	0.883	1.154	1.138	3.482935905	27.88	0	2418.318165	3	806.7763672	-1.495480434	-0.001205516	63.31910766
																			VSGPDLDLNLK	4	61743954	2xTMT6plex [N-Term; K11]	1.15	1.017		3.733888149	55.97	0	1628.962343	2	814.9842529	-0.68375468	-0.000556905	73.08007633
																			VEGDLKGPEVDIR	4	61743954	2xTMT6plex [N-Term; K6]	0.475	0.932		3.677785635	20.62	1	1885.079498	3	629.0308838	-0.742574949	-0.000466604	50.73499212
																			VDIDTPDIDIHGPEGK	6	61743954	2xTMT6plex [N-Term; K16]	2.491	1.319	1.527	3.128494501	33.03	0	2179.164684	3	727.0588379	-1.249803718	-0.000907843	56.0645773
																			LDFEGPDAK	2	61743954	2xTMT6plex [N-Term; K9]	1.151			3.348012447	44.7	0	1449.798966	2	725.4031372	0.021778235	1.58E-05	63.1276824
																			LEGPDVSLK	8	61743954	2xTMT6plex [N-Term; K9]	1.456	1.425	1.141	3.747000694	44.65	0	1415.851002	2	708.4299927	1.205587001	0.000853466	50.98660059
																			LKGDVDVSGPK	15	61743954	3xTMT6plex [N-Term; K2; K11]	1.027	1.099	1.001	3.78134346	45.81	1	1802.09906	2	901.5528564	-0.346295822	-0.00031203	44.80476206
																			GNVDISAPK	10	61743954	2xTMT6plex [N-Term; K9]	1.011	1.064	1.04	3.751635313	50.48	0	1358.804386	2	679.9052734	-0.821115248	-0.000557867	39.23510847
																			ADIEISGPK	12	61743954	2xTMT6plex [N-Term; K9]	1.08	0.997	1.114	3.931933165	60.37	0	1387.819702	2	694.4134521	-0.053323299	-3.70E-05	44.15020513
																			GPQVSGELK	8	61743954	2xTMT6plex [N-Term; K9]		0.845	0.862	3.390076399	42.3	0	1372.820036	2	686.9128418	-1.186664098	-0.000814538	34.32724383
																			LEGGEVDLK	8	61743954	2xTMT6plex [N-Term; K9]	0.932	1.228	1.479	3.254258156	46.33	0	1417.830266	2	709.418396	-0.529659883	-0.000375484	58.88863519
																			VEGDMQVPDLDIK	6	61743954	2xTMT6plex [N-Term; K13]		100		2.209839106	31.16	0	1917.040335	2	959.0222168	-1.657897433	-0.001589128	70.29364403
																			ANVDISAPK	6	61743954	2xTMT6plex [N-Term; K9]	1.002	0.989	0.912	3.950672388	54.08	0	1372.820036	2	686.9136963	0.058207283	4.00E-05	43.4539921
																			ADIDVSGPSVDTDAPDLDIEGPEGK	7	61743954	2xTMT6plex [N-Term; K25]		100		2.571328163	13.71	0	2970.483177	3	990.8311768	-1.413775575	-0.001399866	68.10721076
																			GEYDVTMPK	4	61743954	2xTMT6plex [N-Term; K9]	0.687		0.909	3.139604807	41.44	0	1497.802337	2	749.4053955	0.786055407	0.000588678	53.07400131
																			GDFDVSVPK	8	61743954	2xTMT6plex [N-Term; K9]	1.206	1.657	1.43	3.478405237	42.15	0	1421.804052	2	711.40448	-1.665685971	-0.00118414	63.17911853
																			LKGPQITGPSLEGDLGLK	4	61743954	3xTMT6plex [N-Term; K2; K18]	1.041			3.057883263	16.27	1	2510.516085	3	837.5103149	0.121952702	0.000102055	74.10205351
																			GPTVGGGLPGIGVQGLEGNLQMPGIK	4	61743954	2xTMT6plex [N-Term; K26]				1.841276169	10.42	0	2904.638119	3	968.8840942	-0.133949614	-0.000129692	85.17556027
																			GDVDVSGPK	6	61743954	2xTMT6plex [N-Term; K9]	1.396	1.245	1.515	3.954593897	61.06	0	1331.757101	2	666.3828735	1.027972977	0.000684505	35.18805515
																			MPTFSTPGAK	2	61743954	2xTMT6plex [N-Term; K10]	0.93			3.630608559	56.26	0	1494.839057	2	747.9230347	-0.176750842	-0.000132107	61.14756075
																			APDVDVNIAGPDAALK	4	61743954	2xTMT6plex [N-Term; K16]		0.883	2.074	3.001861095	42.39	0	2024.142826	2	1012.574768	-0.280062839	-0.000283444	67.29151009
																			MDIDAPDVEVQGPDWHLK	2	61743954	2xTMT6plex [N-Term; K18]		0.801		2.404133081	18.53	0	2523.29538	3	841.7686768	-1.546955855	-0.001301142	80.87928536
																			IKGDVDVTGPK	5	61743954	3xTMT6plex [N-Term; K2; K11]		0.959		3.943537235	40.94	1	1816.11471	2	908.5617065	0.785234506	0.000713038	40.9813266
																			ADVDVSGPK	4	61743954	2xTMT6plex [N-Term; K9]	0.965	1.025		3.760979414	57.48	0	1345.772751	2	673.3901978	0.272993945	0.000183694	44.7896363
																			GEGPEVDVK	2	61743954	2xTMT6plex [N-Term; K9]			0.945	3.066952705	45.89	0	1387.783316	2	694.3953247	0.04080901	2.83E-05	41.05730956
																			ADLGVSGPK	4	61743954	2xTMT6plex [N-Term; K9]	0.989	1.102		3.297575951	50.55	0	1301.782922	2	651.395813	1.096232358	0.000713528	47.96955159
																			GDMDVSLPK	4	61743954	2xTMT6plex [N-Term; K9]	1.212	1.534		3.361474514	43.36	0	1419.791772	2	710.3995361	0.016386646	1.16E-05	60.48580014
																			GDVDVTGPK	6	61743954	2xTMT6plex [N-Term; K9]	1.384	1.303	1.32	3.613803387	61.51	0	1345.772751	2	673.3894653	-0.815483845	-0.000548728	37.15016212
																			TDVDVSLPK	2	61743954	2xTMT6plex [N-Term; K9]	1.331			3.423400879	39.18	0	1431.845916	2	716.4259644	-0.8829924	-0.000632155	59.66113242
																			GEVDVDVPK	10	61743954	2xTMT6plex [N-Term; K9]	1.421	1.236	1.388	2.9085567	45.26	0	1415.814616	2	708.4042358	-9.479503996	-0.00671061	44.94008619
																			GDLDASVPSMK	2	61743954	2xTMT6plex [N-Term; K11]; 1xOxidation [M10			0.881	2.722769022	41.02	0	1593.855829	2	797.4317017	0.186478794	0.00014861	40.3504828
																			GDMDISVPK	6	61743954	2xTMT6plex [N-Term; K9]	1.47		1.543	2.615273714	42.02	0	1419.791772	2	710.3997803	0.360297112	0.000255773	47.91273328
																			MKGDVVVSLPK	2	61743954	3xTMT6plex [N-Term; K2; K11]	0.809			3.862905741	13.33	1	1860.159552	3	620.7244873	-0.345919755	-0.000214489	63.89788538
																			LEGDLTGPSVGVEVPDVELECPDAK	7	61743954	2xTMT6plex [N-Term; K25]; 1xCarbamidomethyl [C21		100	100	2.86589694	24.66	0	3083.585868	3	1028.523926	-9.289115814	-0.009547929	77.81889107
																			VDIDVPDVNLEAPEGK	4	61743954	2xTMT6plex [N-Term; K16]	0.332	1.443		2.146986246	21.58	0	2168.185085	2	1084.594238	-1.791866168	-0.001942549	69.31659368
																			GDMDVSVPK	8	61743954	2xTMT6plex [N-Term; K9]	1.217	1.224	1.325	3.428225994	53.28	0	1405.776122	2	703.3920288	0.468550559	0.000329339	51.01946751
																			MQVGGDGVK	4	61743954	2xTMT6plex [N-Term; K9]	1.274	1.092		3.218151808	41.76	0	1348.765892	2	674.8869629	0.561351124	0.000378566	46.54021263
																			ADVDISGPK	13	61743954	2xTMT6plex [N-Term; K9]	1.033	0.992	0.933	4.151010036	45.48	0	1359.788402	2	680.3981323	0.431293896	0.000293234	45.21237596
																			ADLDVSGPK	64	61743954	2xTMT6plex [N-Term; K9]	1.123	1.032	1.085	3.995265722	59.53	0	1359.788402	2	680.3969727	-1.274365554	-0.000866434	34.63890182
																			DIDISSPEFK	6	61743954	2xTMT6plex [N-Term; K10]	1.067	1.031	0.859	3.451611519	41.7	0	1608.888509	2	804.9467773	-1.386884478	-0.001115671	64.9994539
																			GDVVVSLPK	12	61743954	2xTMT6plex [N-Term; K9]	1.516	3.445	1.685	3.042677402	43.11	0	1371.861173	2	686.4327393	-2.165455539	-0.001485352	51.00683631
																			MPDVDISVPK	2	61743954	2xTMT6plex [N-Term; K10]	1.184			3.500643492	40.24	0	1558.891486	2	779.9492798	-0.13048996	-0.00010171	73.23307279
																			GKKPDIDITGPK	6	61743954	4xTMT6plex [N-Term; K2; K3; K12]	0.792	0.803	0.828	4.576816082	18.35	1	2185.372605	3	729.1286621	-0.536417393	-0.000390757	50.34718099
																			EMGVDVHFPK	8	61743954	2xTMT6plex [N-Term; K10]	0.919	0.882	0.822	2.848648787	24.57	0	1616.88707	3	539.6341553	0.521285827	0.000280953	53.71325517
																			VKGDFDVSVPK	4	61743954	3xTMT6plex [N-Term; K2; K11]	0.63		0.876	3.67559576	9.41	1	1878.13036	3	626.7153931	0.673807977	0.000421833	52.62448646
																			GGVDVTLPR	4	61743954	1xTMT6plex [N-Term]	1.058	1.054		3.12466836	37.55	0	1142.673088	2	571.8400879	-0.165680589	-9.47E-05	50.87124415
																			GEYDMTVPK	6	61743954	2xTMT6plex [N-Term; K9]	1.174	1.253	1.242	2.84700489	35.04	0	1497.802337	2	749.4054565	0.867555021	0.000649713	53.29599332
																			VEGDLKGPEVDIK	16	61743954	3xTMT6plex [N-Term; K6; K13]	0.336	0.613	0.729	3.384250402	16.06	1	2086.236282	3	696.0842896	0.974708551	0.000677824	39.78165684
																			VPGIDATTK	2	61743954	2xTMT6plex [N-Term; K9]	1.115			3.21041894	47.78	0	1359.824787	2	680.4161987	0.245444075	0.00016688	52.94327796
																			GEYDVTVPK	2	61743954	2xTMT6plex [N-Term; K9]		1.495		3.367541313	40.15	0	1465.830266	2	733.4177246	-1.42836541	-0.001046871	53.22106094
																			MEGGGAEVHAQLPSLEGDLR	2	61743954	1xTMT6plex [N-Term]				2.272249937	16.96	0	2295.16006	3	765.7229614	-2.496171294	-0.001909704	54.90910385
																			GPGVDVNLK	4	61743954	2xTMT6plex [N-Term; K9]	0.76	0.827		3.59163475	39.3	0	1356.825121	2	678.9151001	-1.619865856	-0.001098937	48.81709745
																			MPSMNIQTHK	6	61743954	2xTMT6plex [N-Term; K10]	0.929	0.9	0.938	2.832520008	12.95	0	1644.896589	3	548.9699707	-0.747567132	-0.00040989	47.51936045
																			IDVDAPDIDIHGPDAK	2	61743954	2xTMT6plex [N-Term; K16]			1.124	2.203732252	17.97	0	2149.154119	3	717.0547485	-2.059849729	-0.001475645	56.20822387
																			EGDQIVGATIYFDNLQSGEVTQLLNTMGHHTVGLK	2	61743954	2xTMT6plex [N-Term; K35]				1.277762294	3.18	0	4244.206177	4	1061.802002	-4.712126759	-0.004999809	99.57143345
																			MDAEVPDVNIEGPDAK	4	61743954	2xTMT6plex [N-Term; K16]		1.095	0.519	2.420570374	19.49	0	2158.110206	2	1079.558228	-0.475926518	-0.000513551	57.22372867
																			GDVDVSLPK	17	61743954	2xTMT6plex [N-Term; K9]	1.413	1.152	1.356	2.953605652	41.57	0	1387.819702	2	694.409729	-5.418781833	-0.003760146	47.06841434
																			GDVDISLPK	6	61743954	2xTMT6plex [N-Term; K9]	1.483	100	1.507	2.932806492	39.23	0	1401.835352	2	701.4203491	-1.376850577	-0.000965059	50.22898686
																			VDVECPDVNIEGPEGK	4	61743954	2xTMT6plex [N-Term; K16]; 1xCarbamidomethyl [C5		100	100	2.15459156	24.18	0	2215.13167	2	1108.068237	-1.115766913	-0.001235785	62.2523783
																			IKGDVDVSVPEVEGK	4	61743954	3xTMT6plex [N-Term; K2; K15]	1.567	0.825		3.215534925	13.18	1	2258.321074	3	753.4454956	0.380582024	0.000286492	61.52584835
																			GDYDVTVPK	2	61743954	2xTMT6plex [N-Term; K9]			1.382	2.696362972	38.36	0	1451.814616	2	726.4116211	0.929380022	0.000674644	51.29044699
																			VDIEAPDVSLEGPEGK	7	61743954	2xTMT6plex [N-Term; K16]	100	1.261		2.226641178	29.57	0	2113.142886	2	1057.074341	-0.700789987	-0.000740435	66.91832684
																			VDVDVPDVNIEGPDAK	4	61743954	2xTMT6plex [N-Term; K16]	1.304	0.965		2.296224356	40.47	0	2140.153785	2	1070.579346	-1.107459551	-0.001185067	61.46288865
																			VESEIKVPDVELK	6	61743954	3xTMT6plex [N-Term; K6; K13]	100	0.01		2.526367426	10.41	1	2172.309447	3	724.7746582	-0.011725137	-8.49E-06	66.61450544
																			VEGDLKGPEVDIRDPK	1	61743954	3xTMT6plex [N-Term; K6; K16]	0.88			2.656410694		2	2454.4171	4	614.3607178	1.605906246	0.000985391	53.86365822
																			FKIPEMHLK	2	61743954	3xTMT6plex [N-Term; K2; K9]	0.731			2.416950703	6.28	1	1830.127858	4	458.2879028	1.050909157	0.000480825	63.40936773
																			GDVDVSVPK	5	61743954	2xTMT6plex [N-Term; K9]	1.41	1.213	1.264	2.893097162	34.7	0	1373.804052	2	687.4051514	-0.746471539	-0.000512753	52.30841359
																			VSAPEVSVGHK	6	61743954	2xTMT6plex [N-Term; K11]	1.09	1.111	1.084	4.366130352	27.68	0	1567.920813	3	523.3114624	-0.62419158	-0.000326228	41.48973306
																			VDIETPNLEGTLTGPR	5	61743954	1xTMT6plex [N-Term]	0.615	100		2.364274263	32.26	0	1941.049036	2	971.027771	-0.397273741	-0.000385564	72.02072927
																			ISMPDVDLHLK	22	61743954	2xTMT6plex [N-Term; K11]	1.189	1.432	0.704	2.659386396	22.9	0	1725.997348	3	576.0031738	-1.378985679	-0.000793375	65.60011929
																			ISIPDVGLHLK	6	61743954	2xTMT6plex [N-Term; K11]	1.125	1.237	2.608	3.234929323	29.46	0	1650.035448	3	550.6836548	0.583408962	0.000320882	85.07506653
																			GPQITGPSLEGDLGLK	6	61743954	2xTMT6plex [N-Term; K16]	0.501		100	2.050178528	15.07	0	2040.174127	2	1020.589539	-1.140094628	-0.001162996	68.53606477
																			TPEMIIQKPK	4	61743954	3xTMT6plex [N-Term; K8; K10]	0.739		0.705	2.781658411	14.38	0	1872.159552	3	624.7249756	0.438732806	0.000273793	45.45380757
																			EVDVNLPK	8	61743954	2xTMT6plex [N-Term; K8]	0.937	1.034	1.108	3.194786787	35.37	0	1371.824787	2	686.4115601	-6.519478943	-0.004471791	47.85087222
																			GDVDVSAPK	2	61743954	2xTMT6plex [N-Term; K9]				3.274595737	44.61	0	1345.772751	2	673.3898315	-0.27124495	-0.000182517	40.75695414
																			GDVDVTLPK	6	61743954	2xTMT6plex [N-Term; K9]	0.864	0.968	100	2.113588095	24.75	0	1401.835352	2	701.421936	0.887201419	0.000621855	37.96712537
																			MSLPDVDLDLK	2	61743954	2xTMT6plex [N-Term; K11]	1.101			3.495131016	43	0	1703.965379	2	852.4867554	0.501549033	0.000427311	88.47993729
																			GDMDVSVPK	5	61743954	2xTMT6plex [N-Term; K9]; 1xOxidation [M3]	1.137	1.276	1.127	2.313531876	24.37	0	1421.771037	2	703.3920288	0.468550559	0.000329339	51.01946751
																			ISAPNVDFNLEGPK	6	61743954	2xTMT6plex [N-Term; K14]	100	100	100	1.792519808	8.65	0	1959.095148	2	980.0504761	-0.751602884	-0.000736231	74.22236798
																			APDVEGQGLDWSLK	8	61743954	2xTMT6plex [N-Term; K14]	0.564	0.972		1.778829575	16.46	0	1973.074413	2	987.0413818	0.544592676	0.000537261	71.32006836
																			ISMPDVNLNLK	5	61743954	2xTMT6plex [N-Term; K11]	1.273	100	100	2.875165224	30.52	0	1701.997348	2	851.5011597	-1.354669597	-0.001152822	77.98067191
																			APNISMPDVDLNLK	4	61743954	2xTMT6plex [N-Term; K14]	100	1.266		2.425267935	20.52	0	1985.114169	2	993.0595703	-1.161074278	-0.001152432	77.9319813
																			MEGDLKGPEVDIK	2	61743954	3xTMT6plex [N-Term; K6; K13]	1.271			2.573180437	1.86	1	2118.208353	3	706.7400513	-1.299244903	-0.000917357	64.21112208
																			GGVQVPAVDISSSLGGR	2	61743954	1xTMT6plex [N-Term]	100			1.346620679	7.67	0	1828.012591	2	914.5084229	-1.653384057	-0.001511203	60.41603236
																			GEVDVSLANVEGDLK	6	61743954	2xTMT6plex [N-Term; K15]	0.01		1.648	1.620357156	13.13	0	2003.106107	2	1002.055786	-0.904028183	-0.000905432	73.08362729
																			ISMPDVGLNLK	8	61743954	2xTMT6plex [N-Term; K11]	1.706	100		2.74799943	39.68	0	1644.975885	2	822.9915771	-0.004251202	-3.50E-06	78.68013021
																			VSMPDVDLNLK	7	61743954	2xTMT6plex [N-Term; K11]		0.01	100	2.518639565	26.7	0	1688.965714	2	844.980896	-6.630387888	-0.005599249	69.52881567
																			ISIPDVDLDLK	7	61743954	2xTMT6plex [N-Term; K11]	1.055	3.972	3.371	2.582414389	20.35	0	1686.008959	2	843.5077515	-0.434487794	-0.000366275	85.06397552
																			ISMPDVDLHVK	9	61743954	2xTMT6plex [N-Term; K11]	1.244	1.051	1.429	2.670517683	23.39	0	1711.981698	3	571.3324585	0.656513024	0.000374646	60.26582186
																			ISMPDIDLNLTGPK	4	61743954	2xTMT6plex [N-Term; K14]		3.14		2.042906046	16.17	0	1972.11892	2	986.5623779	-0.730514073	-0.00072033	79.9043597
																			ISMPDVDLHLK	9	61743954	2xTMT6plex [N-Term; K11]; 1xOxidation [M3	1.891	0.982	1.004	1.853723645	12.19	0	1741.992263	3	576.0031738	-1.378985679	-0.000793375	65.60011929
																			SPQISMSDIDLNLK	4	61743954	2xTMT6plex [N-Term; K14]	0.651			2.475030422	28.61	0	2019.119648	2	1010.064941	1.465025861	0.001479031	83.23649233
																			ISMPDFDLNLK	5	61743954	2xTMT6plex [N-Term; K11]	100	1.104		2.400102139	23.57	0	1750.981364	2	875.9926147	-1.948083448	-0.001705529	88.62556228
																			MYFPDVEFDIK	3	61743954	2xTMT6plex [N-Term; K11]	0.779			2.82147336	27.2	0	1861.98103	2	931.4937744	-0.406745215	-0.000378676	100.4917426
																			ISMPDVDLHMK	4	61743954	2xTMT6plex [N-Term; K11]	1.125	0.983		2.838966608	32.63	0	1743.953769	3	581.9894409	0.000288945	1.68E-07	76.75111698
																			GDVDVSVPEVEGK	4	61743954	2xTMT6plex [N-Term; K13]				2.087913513	24.76	0	1787.979115	2	894.4943237	1.261558884	0.00112782	50.24189167
																			VSMPDVELNLK	7	61743954	2xTMT6plex [N-Term; K11]	1.372	1.066	1.109	3.028838158	33.62	0	1702.981364	2	851.9954224	1.29430457	0.001102088	75.7694311
																			IPMPDFDLHLK	9	61743954	2xTMT6plex [N-Term; K11]	1.071	0.908	100	2.287509918	14.14	0	1784.018084	3	595.3436279	-0.982770832	-0.000584427	83.7692165
																			VPDVEIK	26	61743954	2xTMT6plex [N-Term; K7]	0.988	0.948	1.007	2.836465597	42.19	0	1257.78186	2	629.3946533	0.135413485	8.52E-05	48.14683669
																			ISMPDVDFNLK	11	61743954	2xTMT6plex [N-Term; K11]	100	0.567	1.522	2.459109545	17.16	0	1736.965714	2	868.9855347	-1.10604029	-0.000960577	77.23262762
																			ISMPDLDLNLK	22	61743954	2xTMT6plex [N-Term; K11]	1.714	1.094	100	2.340597391	12.24	0	1716.997014	2	859.0007935	-1.574665486	-0.001351848	80.13244248
																			ISMPEVDLNLK	6	61743954	2xTMT6plex [N-Term; K11]				1.466918111	10.64	0	1716.997014	2	859.0013428	-0.934808338	-0.000802532	78.55449993
																			ISMPEIDLNLK	6	61743954	2xTMT6plex [N-Term; K11]	1.222	1.783	100	1.853166699	18.32	0	1731.012664	2	866.0084839	-1.717432012	-0.001486448	79.06384376
																			GEYDVTMPK	4	61743954	2xTMT6plex [N-Term; K9]; 1xOxidation [M7]	1.001	1.127		2.422406912	27.48	0	1513.797252	2	749.4053955	0.786055407	0.000588678	53.07400131
																			MPEMSIK	2	61743954	2xTMT6plex [N-Term; K7]			1.478	2.602142811	34.37	0	1293.731086	2	647.3687744	-0.629413549	-0.000407146	51.31543577
																			GPEVDIK	14	61743954	2xTMT6plex [N-Term; K7]	0.971	0.97	0.952	2.483335733	33.46	0	1215.73491	2	608.3709717	-0.199698654	-0.00012139	49.7329223
																			MPEMNIK	4	61743954	2xTMT6plex [N-Term; K7]		1.006	1.22	2.79838872	29.83	0	1320.741986	2	660.8746948	0.096535462	6.37E-05	50.66846763
																			VTAPDVDLHLK	2	61743954	2xTMT6plex [N-Term; K11]		0.928		2.12659812	21.73	0	1665.993978	3	556.0018311	-1.823311475	-0.001012542	56.73970988
																			ISMPDFDLHLK	4	61743954	2xTMT6plex [N-Term; K11]; 1xOxidation [M3		100	1.535	2.254517555	13.2	0	1789.992263	3	592.0040283	0.103354685	6.11E-05	87.91292301
																			APEVDIK	8	61743954	2xTMT6plex [N-Term; K7]	0.974	0.899	1.028	2.712181091	42.61	0	1229.75056	2	615.3787842	-0.217800775	-0.00013392	50.00469717
																			MPDMNIK	2	61743954	2xTMT6plex [N-Term; K7]			1.341	2.574495316	28.63	0	1306.726335	2	653.8670654	0.39699925	0.000259385	50.83784654
																			GPALDIK	10	61743954	2xTMT6plex [N-Term; K7]	0.805	0.917	0.864	2.724312544	35.83	0	1171.74508	2	586.3763428	0.280442291	0.000164303	43.62251771
																			VESDLKGPEVDIEGPEGK	5	61743954	3xTMT6plex [N-Term; K6; K18]			100	2.169825077	11.16	1	2585.427724	3	862.4799805	-0.903387756	-0.000778548	53.22282213
																			GPGVDLPSVNLSMPK	6	61743954	2xTMT6plex [N-Term; K15]			0.01	1.399926901	5.93	0	1969.119254	2	985.0612183	-2.079288267	-0.002047183	70.42322348
																			MDIDVPDVEVQGPDWHLK	4	61743954	2xTMT6plex [N-Term; K18]			100	1.862979412	13.4	0	2551.326681	3	851.112793	-1.118991845	-0.000951638	74.71560881
																			MKGEVDVSLANVEGDLK	2	61743954	3xTMT6plex [N-Term; K2; K17]		100		1.867832065	0.91	1	2491.404486	3	831.137146	-3.051010689	-0.002533767	72.97723005
																			GEIDASVPELEGDLR	5	61743954	1xTMT6plex [N-Term]				1.503990531	8.61	0	1828.948988	2	914.9769897	-1.249497841	-0.001142634	61.28865964
																			APDVDIK	10	61743954	2xTMT6plex [N-Term; K7]	0.877	0.899	0.807	2.810521364	35.89	0	1215.73491	2	608.3713379	0.402753303	0.000244821	41.89324124
																			GPDWHLK	4	61743954	2xTMT6plex [N-Term; K7]	0.82			2.707974195	25.78	0	1310.762127	2	655.8846436	-0.089154716	-5.84E-05	50.75970886
																			VQTPEVDVK	6	61743954	2xTMT6plex [N-Term; K9]	1.178	1.126	1.236	3.665341139	45.3	0	1472.872465	2	736.9395142	-0.484624232	-0.000356895	51.3746906
																			ISMSEVDLNVAAPK	4	61743954	2xTMT6plex [N-Term; K14]			100	1.57583952	10.21	0	1932.08762	2	966.5466919	-0.782889411	-0.000756305	78.5286429
																			VDTNAPDLSLEGPEGK	4	61743954	2xTMT6plex [N-Term; K16]			100	2.230504751	18.65	0	2100.122485	2	1050.564575	-0.290949399	-0.000305515	54.31412711
																			VGGSGVNVNAK	2	61743954	2xTMT6plex [N-Term; K11]				1.815688133	8.36	0	1459.863298	2	730.4342651	-1.400231491	-0.001022073	39.46687473
																			ISMPDIDFNLK	3	61743954	2xTMT6plex [N-Term; K11]				1.577090859	4.96	0	1750.981364	2	875.9937134	-0.6932068	-0.000606896	87.39585858

Bhat et al,2017. Cigarette smoke and chewing tobacco alter expression of different sets of miRNAs in oral keratinocytes

Supplementary Table S2(A). List of all proteins and peptides identified in OKF6/TERT1 cells chronically treated with chewing tobacco using Sequest and Mascot search algorithms

Sequence	#PSMs	Protein Accessions	Modifications	OKF6-STE-6M/OKF6P_R1	OKF6-STE-6M/OKF6P_R2	OKF6-STE-6M/OKF6P_R3	Xcorr	Ionscore	# Missed Cleavages	Theo. MH+ [Da]	Charge	m_z	DeltaM [ppm]	Deltam/z [Da]	RT [min]
FSMPSLK	2	61743954	2xTMT6plex [N-Term; K7]	0.915			2.73694849	24.84	0	1267.748451	2	634.3773804	-0.762783667	-0.000483509	67.96186755
FSVSGAK	6	61743954	2xTMT6plex [N-Term; K7]	0.896	1.198		2.480217695	24.43	0	1153.69813	2	577.3526001	-0.179045698	-0.000103282	44.44498778
MPFLSISSPK	6	61743954	2xTMT6plex [N-Term; K10]	100	100	100	2.104370832	21.95	0	1564.917307	2	782.9620972	-0.248903926	-0.000194757	69.37259627
FSMPGFK	7	61743954	2xTMT6plex [N-Term; K7]	1.412	0.986	0.848	2.849398851	24.55	0	1271.722236	2	636.3652954	0.847496632	0.00053889	56.05187467
LKGEIDASVPELEGDLR	3	61743954	2xTMT6plex [N-Term; K2]	100			1.660045743	0.02	1	2299.290947	3	767.1015625	-0.353491584	-0.000270927	67.05426916
ISMQDVDLSLGSPK	4	61743954	2xTMT6plex [N-Term; K14]				1.319783449	6.19	0	1948.082534	2	974.5432739	-1.675056565	-0.001631574	71.95935059
IDVTAPDVSIEEPEGK	3	61743954	2xTMT6plex [N-Term; K16]				1.439643145	6.23	0	2157.169101	2	1079.085327	-2.65298308	-0.002861467	64.92523499
ISMPDVDLNLK	1	61743954	2xTMT6plex [N-Term; K11]				1.4323107	6.05	0	1702.981364	2	851.9940186	-0.354343646	-0.00030172	72.39649618
FSMPGFK	2	61743954	2xTMT6plex [N-Term; K7]; 1xOxidation [M3]		2.799	0.01	1.445465446	24.33	0	1287.717151	2	636.3652954	0.847496632	0.00053889	56.05187467
VDIDVPDVNIEGPEGK	1	61743954	2xTMT6plex [N-Term; K16]		100		1.637587309		0	2154.169435	2	1077.58728	-0.998553359	-0.001075527	66.6096849
VPDVDIK	6	61743954	2xTMT6plex [N-Term; K7]	1.07	0.996	1.089	2.841577768	34.63	0	1243.76621	2	622.3862305	-0.824369156	-0.000512661	48.57540066
LEVPDMNIR	6	61743954	1xTMT6plex [N-Term]	100	1.021	100	2.531183958	14.01	0	1315.724138	2	658.3648071	-1.368122797	-0.000900036	57.02364384
MPEMSIKPQK	3	61743954	3xTMT6plex [N-Term; K7; K10]	0.316	100		1.353689551	6.22	0	1876.100323	3	626.0394287	1.817692141	0.001136724	37.13873051
VKGDMDVSLPK	2	61743954	3xTMT6plex [N-Term; K2; K11]; 1xOxidation [M5			0.897	2.304536819	18.46	1	1892.112996	3	626.0443115	0.159963446	0.000100037	54.0912211
MPDMHVNMPK	1	61743954	2xTMT6plex [N-Term; K10]; 1xOxidation [M1			0.946	2.877375364	21.18	0	1673.857761	3	553.2920532	-0.747457508	-0.000413061	50.61616629
GPEIDVK	10	61743954	2xTMT6plex [N-Term; K7]	0.999		0.931	2.63498354	36.03	0	1215.73491	2	608.3716431	0.9047966	0.000549996	46.18897567

Accession	Gene_Symbol	Description	Gene_ID	Refseq_Accession	Coverage	Proteins	#Unique_Peptides	#Peptides	#PSMS	#AAs	MW [kDa]	calc. pI	OKF6-STE-6M/OKF6P_R1	OKF6-STE-6M/OKF6P_R2	OKF6-STE-6M/OKF6P_R3	Median fold change	Status	P-value (6M)
12667788	MYH9	myosin-9	4627	NP_002464.1	59.84693878	1	96	115	1225	1960	226.392	5.6	1.02	1.05	1.08	1.05	Significant	0.00027

Sequence	#PSMs	Protein Accessions	Modifications	OKF6-STE-6M/OKF6P_R1	OKF6-STE-6M/OKF6P_R2	OKF6-STE-6M/OKF6P_R3	Xcorr	Ionscore	# Missed Cleavages	Theo. MH+ [Da]	Charge	m_z	DeltaM [ppm]	Deltam/z [Da]	RT [min]
KEEELQAALAR	18	12667788	2xTMT6plex [N-Term; K1]	0.939	0.973	0.938	4.138899803	53.33	1	1716.005605	2	858.5010376	-6.297313166	-0.005403112	44.61299936
MEDSVGCLETAEEVKR	6	12667788	2xTMT6plex [N-Term; K15]; 1xCarbamidomethyl [C7	1.865	100	1.041	4.97214222	37.78	1	2311.167403	3	771.0617676	1.44791951	0.001115461	57.1267798
VIQYLAYVASSHK	6	12667788	2xTMT6plex [N-Term; K13]	0.902	100	1.138	3.429250956	38.68	0	1937.126054	3	646.3798218	-0.589619847	-0.000380723	88.34844983
DLEAHIDSANK	29	12667788	2xTMT6plex [N-Term; K11]	1.035	1.013	0.988	4.861137867	69.69	0	1670.91137	2	835.9588623	-0.551842929	-0.00046104	38.81304191
KANLQIDQINTDLNLER	7	12667788	2xTMT6plex [N-Term; K1]	1.141	0.94	0.839	4.123247147	31.76	1	2456.387307	3	819.4680176	0.892580893	0.000730841	58.93803709
VSHLLGINVTDFTR	14	12667788	1xTMT6plex [N-Term]	1.017	0.86	1.175	3.953921318	40.81	0	1801.016949	3	601.0108032	0.504025223	0.000302586	80.15194181
KVEAQLQELQVK	12	12667788	3xTMT6plex [N-Term; K1; K12]	1.386	1.61	1.521	5.037176609	28.41	1	2100.299551	3	700.770813	-0.792836981	-0.000555065	54.32501322
ALEQQVEEMK	6	12667788	2xTMT6plex [N-Term; K10]	1.052	0.817	0.921	3.977100611	64.91	0	1662.913678	2	831.960144	-0.400997401	-0.000333412	54.7076908
QLEEAEEEAQR	6	12667788	1xTMT6plex [N-Term]	1.076	1.32	1.181	4.714777946	62.98	0	1560.770296	2	780.8880615	-0.928511472	-0.000724597	37.25282075
DELADEIANSSGK	6	12667788	2xTMT6plex [N-Term; K13]	0.934	0.971	1.173	4.969425678	58.44	0	1806.948543	2	903.9779053	-0.005192801	-4.69E-06	66.12471956
QIATLHAQVADMK	10	12667788	2xTMT6plex [N-Term; K13]	1.149	1.073	1.099	4.899812698	61.87	0	1884.077724	2	942.5435791	1.145241036	0.001078862	57.39266253
QIATLHAQVADMK	6	12667788	2xTMT6plex [N-Term; K13]; 1xOxidation [M12		1.167	1.11	4.392225742	45.91	0	1900.072639	2	942.5435791	1.145241036	0.001078862	57.39266253
ELESQISELQEDLESER	18	12667788	1xTMT6plex [N-Term]	0.991	1.103	1.29	4.533257008	50.72	0	2263.113881	3	755.0432129	0.53223211	0.000401501	91.53338142
LQQELDDLLVDLDHQR	12	12667788	1xTMT6plex [N-Term]	1.085	0.917	100	4.158302307	46.37	0	2179.155627	3	727.0562744	-0.622593355	-0.000452243	97.96809994
LQLQEQLQAETELCAEAEELR	14	12667788	1xTMT6plex [N-Term]; 1xCarbamidomethyl [C14	100	1.015	3.895	4.11145401	34.39	0	2730.38174	3	910.7987061	-0.064066477	-5.83E-05	117.5417041
VISGVLQLGNIVFK	6	12667788	2xTMT6plex [N-Term; K14]	0.01	1.222	1.543	3.503041506	45.42	0	1945.22504	2	973.1159668	-0.196911022	-0.000191518	100.7724247
SMEAEMIQLQEELAAAER	10	12667788	1xTMT6plex [N-Term]	100		0.813	4.078587055	66.63	0	2278.125648	3	760.0455933	-1.502083455	-0.001140645	97.6760944
IAQLEEELEEEQGNTELINDR	14	12667788	1xTMT6plex [N-Term]	1.931	1.17	1.282	5.213269711	62.32	0	2701.336563	3	901.114624	-2.681857487	-0.002414867	89.36893354
TQLEELEDELQATEDAK	12	12667788	2xTMT6plex [N-Term; K17]	1.159	1.124	1.418	3.995464325	43.98	0	2420.24445	3	807.4188843	-0.971231633	-0.000783539	87.48657366
KMEDSVGCLETAEEVKR	8	12667788	3xTMT6plex [N-Term; K1; K16]; 1xCarbamidomethyl [C8	100	1.036	1	4.346344471	23.21	2	2668.425298	4	667.8620605	0.417567432	0.000278562	46.16850311
ANLQIDQINTDLNLER	7	12667788	1xTMT6plex [N-Term]	1.433	1.844	100	3.83530736	43.89	0	2099.129412	2	1050.067871	-0.450869058	-0.000473216	95.49103021
LEEEQIILEDQNCK	8	12667788	2xTMT6plex [N-Term; K14]; 1xCarbamidomethyl [C13	1.536	1.435	100	4.224692822	37.02	0	2219.16297	3	740.3922729	-0.317273834	-0.000234694	69.17125754
NMDPLNDNIATLLHQSSDK	4	12667788	2xTMT6plex [N-Term; K19]	0.982	1.726		5.326061249	45.77	0	2584.344121	3	862.1190186	-0.626482334	-0.000539682	85.16972195
MEDSVGCLETAEEVK	4	12667788	2xTMT6plex [N-Term; K15]; 1xCarbamidomethyl [C7	1.4	1.443		3.617064714	44.5	0	2155.066292	2	1078.036865	0.075036555	8.09E-05	68.24865189
EDQSILCTGESGAGK	10	12667788	2xTMT6plex [N-Term; K15]; 1xCarbamidomethyl [C7	1.753	1.309	1.499	4.520535469	77.09	0	2010.021391	2	1005.514282	-0.051281482	-5.15E-05	42.92864444
EQADFAIEALAK	32	12667788	2xTMT6plex [N-Term; K12]	1.529	100	0.51	3.484187841	54.67	0	1763.994371	2	882.5020752	1.418581978	0.001251185	76.39598563
NFINNPLAQADWAAK	6	12667788	2xTMT6plex [N-Term; K15]	0.775	100	1.11	3.742403507	42.81	0	2131.170044	2	1066.088379	-0.26419173	-0.000281519	80.50698959
IIGLDQVAGMSETALPGAFK	9	12667788	2xTMT6plex [N-Term; K20]	1.219		1.187	4.88001442	62.11	0	2476.388553	3	826.1339722	-0.480266351	-0.000396442	98.66422412
KLEGDSTDLSDQIAELQAQIAELK	28	12667788	3xTMT6plex [N-Term; K1; K24]	0.936	1.021	0.947	4.907475471	56.65	1	3302.829827	4	826.463562	0.784514193	0.000647779	106.3930276
DFSALESQLQDTQELLQEENR	16	12667788	1xTMT6plex [N-Term]	0.907	1.5	1.037	5.196689606	55.23	0	2722.336898	3	908.1173096	0.175463565	0.000159224	94.97041141
LEGDSTDLSDQIAELQAQIAELK	11	12667788	2xTMT6plex [N-Term; K23]	1.408	0.778	100	4.846818924	61.38	0	2945.571932	3	982.5285034	-0.331034555	-0.000325029	105.764618
KMEDSVGCLETAEEVK	10	12667788	3xTMT6plex [N-Term; K1; K16]; 1xCarbamidomethyl [C8	1.173	1.145	1.33	6.366542339	38.3	1	2512.324187	3	838.1109619	-2.330355232	-0.001951536	58.99038517
KQELEEICHDLEAR	16	12667788	2xTMT6plex [N-Term; K1]; 1xCarbamidomethyl [C8	1.137	1.233	1.26	3.971699953	33.5	1	2228.174537	4	557.7981567	-1.678673197	-0.000935094	62.97967391
ELEDATETADAMNR	20	12667788	1xTMT6plex [N-Term]	1.069	1.218	1.49	4.801186085	57.05	0	1794.837723	2	905.9181519	-1.994137385	-0.001805525	39.53686825
LQQLFNHTMFILEQEEYQR	4	12667788	1xTMT6plex [N-Term]			1.802	4.64874506	37.09	0	2696.370387	3	899.4614868	-0.177998833	-0.000159984	103.4314357
LQVELDNVTGLLSQSDSK	34	12667788	2xTMT6plex [N-Term; K18]	1.398	1.269	1.186	4.554018497	55.04	0	2404.33354	3	802.1167603	0.90980377	0.000729157	94.41939654
DLGEELEALKTELEDTLDSTAAQQELR	12	12667788	2xTMT6plex [N-Term; K10]			0.81	5.113897324	50.22	1	3475.805573	4	869.7038574	-3.444756114	-0.002993326	112.3674334
EMEAELEDER	30	12667788	1xTMT6plex [N-Term]	1.126	1.196	1.13	4.615645409	77.91	0	1479.683455	2	740.3470459	2.271206632	0.001680333	41.37008736
IAQLEEQLDNETK	13	12667788	2xTMT6plex [N-Term; K13]	1.305	1.126	100	3.618124962	45.26	0	1989.090456	2	995.0491943	0.329598824	0.000327801	85.13797427
NTDQASMPDNTAAQK	2	12667788	2xTMT6plex [N-Term; K15]; 1xOxidation [M7			1.137	4.371851921	35.54	0	2066.022454	3	689.3435059	-3.140949346	-0.002163091	29.31300229
QAQQERDELADEIANSSGK	2	12667788	2xTMT6plex [N-Term; K19]		2.543		4.95339632	32.82	1	2547.305093	3	849.7721558	-1.248109169	-0.001059772	51.80347346
KLEEEQIILEDQNCK	4	12667788	3xTMT6plex [N-Term; K1; K15]; 1xCarbamidomethyl [C14		1.291	1.215	4.556018829	32.18	1	2576.420865	3	859.4793701	1.045039496	0.000897487	60.98776906
EQLEEEEEAK	16	12667788	2xTMT6plex [N-Term; K10]	1.316	1.38	1.628	4.411446571	82.13	0	1691.873981	2	846.4408569	0.269409656	0.000227904	38.02424208
HSQAVEELAEQLEQTK	7	12667788	2xTMT6plex [N-Term; K16]	1.135	0.903		5.971982002	45.2	0	2298.23416	3	766.74823	-1.750791396	-0.001341243	80.62720146
HEMPPHIYAITDTAYR	6	12667788	1xTMT6plex [N-Term]		0.994	0.985	4.753227234	41.84	0	2144.079625	3	715.3644409	-0.399073842	-0.000285215	43.64412618
EQLEEEEEAKHNLEK	14	12667788	3xTMT6plex [N-Term; K10; K15]	0.807	0.822	1.038	5.821277618	39.19	1	2542.360373	4	636.3449707	-0.912379506	-0.000579899	52.02026805
NKHEAMITDLEER	18	12667788	2xTMT6plex [N-Term; K2]	0.779	0.736	0.753	5.191141129	49.71	1	2044.089745	4	511.7775879	-0.598455867	-0.000305824	42.04813944
EMEAELEDERK	14	12667788	2xTMT6plex [N-Term; K11]	0.879	0.833	0.925	4.213075161	44.85	1	1836.941349	3	612.9862061	1.478242113	0.000905148	43.98168113
TFHIFYYLLSGAGEHLK	14	12667788	2xTMT6plex [N-Term; K17]	0.759	2.876	100	3.024085999	18.25	0	2454.358573	3	818.7903442	-0.853060013	-0.000697905	101.5479844
AGVLAHLEEER	12	12667788	1xTMT6plex [N-Term]	1.676	1.489	1.709	4.231396198	57.68	0	1452.800808	3	484.9386597	0.425202067	0.000205911	53.69342542
LDPHLVLDQLR	10	12667788	1xTMT6plex [N-Term]	0.934	1.269		3.6594944	46.08	0	1547.910693	2	774.4589233	-0.079300642	-6.14E-05	84.30943887
VMQEQGTHPK	4	12667788	2xTMT6plex [N-Term; K10]	1.064	1.133		3.616249084	44.8	0	1612.888132	3	538.3010864	0.355708082	0.000191239	28.12437486
DVLLQVDDER	16	12667788	1xTMT6plex [N-Term]	1.221	0.933	4.003	4.493911266	66.82	0	1430.768839	2	715.8873291	-1.018736804	-0.000728788	49.52640145
ALELDSNLYR	14	12667788	1xTMT6plex [N-Term]	1.07	1.185	1.491	4.279184341	49.77	0	1422.77901	2	711.894043	1.264678132	0.000899679	44.31512085
DKADFCIIHYAGK	6	12667788	3xTMT6plex [N-Term; K2; K13]; 1xCarbamidomethyl [C6	1.159	1.103	0.985	4.06019783	19.44	1	2225.235571	4	557.0643921	0.075367021	4.19E-05	51.25403601
DLGEELEALK	16	12667788	2xTMT6plex [N-Term; K10]	1.16	2.043	0.01	3.433280468	50.44	0	1574.904159	2	787.9544678	-1.587743045	-0.001250272	70.94488482
QLLQANPILEAFGNAK	6	12667788	2xTMT6plex [N-Term; K16]	1.01	0.757		3.391020775	32.11	0	2185.274509	3	729.0963745	0.027930201	2.03E-05	98.94921035
KKVEAQLQELQVK	12	12667788	4xTMT6plex [N-Term; K1; K2; K13]	0.603	0.534	0.604	5.545407295	16.62	2	2457.557446	4	615.1395874	-8.515015859	-0.005231535	49.67335697
ELEDATETADAMNR	12	12667788	1xTMT6plex [N-Term]; 1xOxidation [M12]	1.006	1.03	0.91	3.385944843	57.79	0	1810.832638	2	905.9181519	-1.994137385	-0.001805525	39.53686825
LTEMETLQSQLMAEK	4	12667788	2xTMT6plex [N-Term; K15]	100			2.753127098	30.45	0	2210.181262	2	1105.592407	-1.685014229	-0.001862093	103.3820044
QTLENERGELANEVK	6	12667788	2xTMT6plex [N-Term; K15]	0.972	0.747	0.904	4.472751141	35.64	1	2188.197381	3	730.0673218	-4.555785532	-0.003322986	43.44950373
ALEEAMEQK	6	12667788	2xTMT6plex [N-Term; K9]	0.925	1.056	0.966	4.312901497	61.93	0	1506.823801	2	753.9080811	-9.898463654	-0.00745762	49.51705221
MQQNIQELEEQLEEEESAR	8	12667788	1xTMT6plex [N-Term]	3.228	2.074		3.815178156	32.53	0	2562.219091	3	854.7427979	-2.049249157	-0.001750208	83.73641431
TEMEDLMSSK	13	12667788	2xTMT6plex [N-Term; K10]	1.285	100	100	3.755452871	57.5	0	1628.827565	2	814.9163818	-1.275984131	-0.001039179	62.9752963
RGDLPFVVPR	7	12667788	1xTMT6plex [N-Term]	1.123	1.528	100	4.451939106	26.97	1	1384.826235	3	462.2798767	-0.836309291	-0.000386048	55.26785662
ASITALEAK	24	12667788	2xTMT6plex [N-Term; K9]	0.944	0.897	0.978	4.587734699	62.75	0	1361.840437	2	681.4248047	1.391950884	0.000947807	54.44673596
ADFCIIHYAGK	16	12667788	2xTMT6plex [N-Term; K11]; 1xCarbamidomethyl [C4	1.263	1.18	1.194	3.130453587	32.98	0	1752.950733	2	876.9789429	-0.0705883	-6.19E-05	54.99212348
LQEMEGTVK	16	12667788	2xTMT6plex [N-Term; K9]	0.993	1.18	0.994	4.407355309	50.75	0	1492.844536	2	746.9266968	1.058880981	0.000790372	47.34007542
EMEAELEDER	10	12667788	1xTMT6plex [N-Term]; 1xOxidation [M2]	1.179	1.209	1.311	3.194990396	50.76	0	1495.67837	2	740.3470459	2.271206632	0.001680333	41.37008736
VEAQLQELQVK	6	12667788	2xTMT6plex [N-Term; K11]	100	1.31	1.156	2.859863043	35.08	0	1743.041656	2	872.0236206	-0.970349192	-0.00084568	57.63140534
THEAQIQEMR	12	12667788	1xTMT6plex [N-Term]	1.191	1.223	1.158	4.855857849	43.5	0	1471.752478	3	491.2557068	0.060799169	2.98E-05	27.65125317
VEDMAELTCLNEASVLHNLK	10	12667788	2xTMT6plex [N-Term; K20]; 1xCarbamidomethyl [C9	1.037	0.01	100	2.921220064	25.48	0	2744.436308	3	915.4824829	-1.243267888	-0.001137357	83.16239164
THEAQIQEMR	10	12667788	1xTMT6plex [N-Term]; 1xOxidation [M9]	1.252	1.092	100	3.09626174	31.26	0	1487.747393	3	491.2557068	0.060799169	2.98E-05	27.65125317
KFDQLLAEEK	12	12667788	3xTMT6plex [N-Term; K1; K10]	1.479	1.049	1.208	3.74681735	21.57	1	1908.140925	3	636.7192383	1.172074725	0.000745495	67.86442023
FDQLLAEEK	4	12667788	2xTMT6plex [N-Term; K9]	1.112	2.208		3.206862211	53.23	0	1550.88303	2	775.9446411	-0.660619413	-0.000512272	76.14735231
VEDMAELTCLNEASVLHNLK	2	12667788	2xTMT6plex [N-Term; K20]; 1xCarbamidomethyl [C9]; 1xOxidation [M4]	100			2.271770716	14.6	0	2760.431223	3	915.4824829	-1.243267888	-0.001137357	83.16239164
CQHLQAEK	6	12667788	2xTMT6plex [N-Term; K8]; 1xCarbamidomethyl [C1	0.919	0.873	0.837	4.014299393	35.92	0	1471.809154	3	491.274292	-0.564858178	-0.000277121	25.81115511
QELEEICHDLEAR	4	12667788	1xTMT6plex [N-Term]; 1xCarbamidomethyl [C7	0.801			3.0504632	32.81	0	1870.916642	3	624.3115234	1.803700081	0.001124858	69.45870155
DLQGRDEQSEEK	6	12667788	2xTMT6plex [N-Term; K12]	0.283	0.293	0.297	2.971063137	27.45	1	1891.976155	3	631.3299561	-0.444025648	-0.000280029	29.33345778
TELEDTLDSTAAQQELR	4	12667788	1xTMT6plex [N-Term]				2.774915218	24.15	0	2149.082187	2	1075.044556	-0.163898746	-0.000176116	56.91845861
IAEFTTNLTEEEEK	11	12667788	2xTMT6plex [N-Term; K14]	1.445	100	100	3.002529144	27.88	0	2112.111251	2	1056.559204	-0.056728487	-5.99E-05	96.64384908
VMQEQGTHPK	6	12667788	2xTMT6plex [N-Term; K10]; 1xOxidation [M2	1.026	1.111	1.261	3.360104322	27.12	0	1628.883047	3	538.3010864	0.355708082	0.000191239	28.12437486
GDLPFVVPR	8	12667788	1xTMT6plex [N-Term]	1.08	0.977	0.919	3.379627466	38.56	0	1228.725124	2	614.8655396	-1.075568824	-0.000660789	65.65294845
NKHEAMITDLEER	2	12667788	2xTMT6plex [N-Term; K2]; 1xOxidation [M6]		0.737		4.218317509	25.33	1	2060.08466	4	511.7775879	-0.598455867	-0.000305824	42.04813944
KMEDSVGCLETAEEVK	2	12667788	3xTMT6plex [N-Term; K1; K16]; 1xCarbamidomethyl [C8]; 1xOxidation [M2]	1.102			3.713214159	29.11	1	2528.319102	3	838.1109619	-2.330355232	-0.001951536	58.99038517
IMGIPEEEQMGLLR	16	12667788	1xTMT6plex [N-Term]	1.003	1.788	0.915	2.80235219	18.86	0	1844.981157	2	922.9929199	-1.405735902	-0.001296778	73.26113808
TDLLLEPYNK	14	12667788	2xTMT6plex [N-Term; K10]	1.854	100	1.866	3.248836517	32.93	0	1663.967094	2	832.4885254	1.610657602	0.001340041	69.16759895
DMFQETMEAMR	12	12667788	1xTMT6plex [N-Term]	100		2.434	2.366911888	31.68	0	1617.72725	2	809.3618164	-6.734273344	-0.005447109	69.67636136
QACVLMIK	5	12667788	2xTMT6plex [N-Term; K8]; 1xCarbamidomethyl [C3		1.651	1.253	3.355790377	43.78	0	1420.842034	2	710.9245605	-0.133530854	-9.49E-05	66.74766306
GALALEEK	18	12667788	2xTMT6plex [N-Term; K8]	0.895	1.009	0.94	3.824853897	54.12	0	1288.787673	2	644.8971558	-0.495369855	-0.000319213	44.18873252
LTEMETLQSQLMAEK	2	12667788	2xTMT6plex [N-Term; K15]; 1xOxidation [M12	0.682			2.270932436	17.29	0	2226.176177	2	1105.592407	-1.685014229	-0.001862093	103.3820044
LKDVLLQVDDER	9	12667788	2xTMT6plex [N-Term; K2]	0.666	0.966	1.181	2.860114813	16.44	1	1901.110798	3	634.3746948	-0.666361658	-0.000422276	67.36287138
VKPLLQVSR	15	12667788	2xTMT6plex [N-Term; K2]	1.098	1.186	1.086	3.294209957	26.25	0	1497.988104	2	749.4981079	0.557267651	0.00041739	45.36681944
ADGAEAKPAE	5	12667788	2xTMT6plex [N-Term; K7]	1.563	1.349		2.042784214	31.7	0	1416.77348	2	708.8900146	-0.513175278	-0.000363527	31.41134278
YEILTPNSIPK	6	12667788	2xTMT6plex [N-Term; K11]	0.641	1.114	0.974	2.18566823	20.69	0	1733.024943	2	867.0157471	-0.418787611	-0.000362885	60.43249124
NAEQYKDQADK	10	12667788	3xTMT6plex [N-Term; K6; K11]	0.843	1.036	0.985	2.953680754	23.35	1	1997.09068	2	999.0466309	-2.351050604	-0.002347631	38.01056565
VEEEAAQK	8	12667788	2xTMT6plex [N-Term; K8]	1.103	0.832	1.109	3.443967819	45.96	0	1361.767666	2	681.387085	-0.56749631	-0.000386399	35.3442166
EQEVNILK	26	12667788	2xTMT6plex [N-Term; K8]	1.625	1.069	1.096	3.123903513	45.51	0	1430.861901	2	715.9344482	-0.196361106	-0.000140483	55.35462879
EEILAQAK	8	12667788	2xTMT6plex [N-Term; K8]	1.267	1.393	1.344	3.706701279	52.37	0	1359.824787	2	680.4160156	-0.023863369	-1.62E-05	45.75717177
LQEMEGTVK	9	12667788	2xTMT6plex [N-Term; K9]; 1xOxidation [M4]	1.163	1.066	1.141	2.46706295	34.55	0	1508.839451	2	746.9266968	1.058880981	0.000790372	47.34007542
QEEEMMAK	4	12667788	2xTMT6plex [N-Term; K8]	1.167		1.291	3.613760948	47.74	0	1453.743108	2	727.3748169	-0.516219773	-0.000375225	41.98587944
QQQLTAMK	4	12667788	2xTMT6plex [N-Term; K8]	1.618	1.601		4.283803463	50.61	0	1405.823741	2	703.4151001	-0.581626746	-0.000408832	41.41378418
VAAYDKLEK	11	12667788	3xTMT6plex [N-Term; K6; K9]	0.756	0.774	0.866	4.033283234	34.02	1	1724.056133	2	862.5306396	-1.235617034	-0.001065137	43.93594648
LKDVLLQVDDERR	5	12667788	2xTMT6plex [N-Term; K2]	1.046	0.942		4.697195053	13.38	2	2057.211909	4	515.0570679	-2.657677413	-0.001366851	60.46360249
KLVWVPSDK	11	12667788	3xTMT6plex [N-Term; K1; K9]	0.663	0.706	0.602	4.047985077	24.32	1	1759.108503	3	587.0413208	0.515177058	0.000302084	59.10632432
TEMEDLMSSK	4	12667788	2xTMT6plex [N-Term; K10]; 1xOxidation [M	1.089	0.843		2.315953732	27.35	0	1644.82248	2	814.9163818	-1.275984131	-0.001039179	62.9752963
SGFEPASLKEEVGEEAIVELVENGKK	2	12667788	4xTMT6plex [N-Term; K9; K25; K26]	100			2.611670256	5.23	2	3705.076823	4	927.0200195	-5.013319801	-0.004643684	94.42320387
ALEEAMEQK	7	12667788	2xTMT6plex [N-Term; K9]; 1xOxidation [M6]	0.969	1.022	0.94	2.873091936	34.44	0	1522.818716	2	753.9080811	-9.898463654	-0.00745762	49.51705221
VEEEEER	6	12667788	1xTMT6plex [N-Term]		100	1.383	3.147355795	38.91	0	1148.563263	2	574.7855835	0.546066732	0.000313596	25.81106331
SVHELEK	4	12667788	2xTMT6plex [N-Term; K7]	0.81			3.355924368	33.06	0	1299.767272	2	650.387207	-0.103708951	-6.74E-05	34.32314516
LVWVPSDK	9	12667788	2xTMT6plex [N-Term; K8]	1.223	1.011	2.149	2.67508173	26.93	0	1401.850608	2	701.4281616	-1.113754778	-0.000780659	61.45573892
QTLENER	2	12667788	1xTMT6plex [N-Term]	1.313			2.646174431	33.94	0	1118.600317	2	559.803772	-0.044801246	-2.51E-05	28.73549051
NMDPLNDNIATLLHQSSDK	2	12667788	2xTMT6plex [N-Term; K19]; 1xOxidation [M2				2.024447441	13.39	0	2600.339036	3	862.1190186	-0.626482334	-0.000539682	85.16972195
VVFQEFR	12	12667788	1xTMT6plex [N-Term]	1.599	1.135	1.16	2.68443346	27.72	0	1153.65671	2	577.3317261	-0.463449445	-0.000267331	54.44124223
QLAAENR	4	12667788	1xTMT6plex [N-Term]	0.779	1.084		2.802093744	38.57	0	1030.584274	2	515.7961426	0.713203441	0.000367508	21.30296196
FVSELWK	10	12667788	2xTMT6plex [N-Term; K7]	100	3.878	1.534	2.518531561	27.51	0	1366.813494	2	683.9100952	-0.424622902	-0.00029019	70.45559314
ITDVIIGFQACCR	2	12667788	1xTMT6plex [N-Term]; 2xCarbamidomethyl [C11; C12	0.01			1.291034937	7.29	0	1781.923977	2	891.4648438	-0.87885343	-0.000783025	93.34206874
DEQSEEK	4	12667788	2xTMT6plex [N-Term; K7]	1.513			2.94849968	29.61	0	1322.683996	2	661.8453979	-0.360442527	-0.000238376	23.23821774
TVGQLYK	4	12667788	2xTMT6plex [N-Term; K7]	1.228	1.028		2.741590977	31	0	1266.782194	2	633.8947754	0.063224168	4.00E-05	53.36307664
ADEWLMK	8	12667788	2xTMT6plex [N-Term; K7]	0.874		100	3.158114672	36.3	0	1350.749179	2	675.8774414	-1.164670336	-0.000786589	65.2501774
QEEEMMAK	2	12667788	2xTMT6plex [N-Term; K8]; 1xOxidation [M6]			1.305	2.322684288	27.63	0	1469.738023	2	727.3748169	-0.516219773	-0.000375225	41.98587944
SMAVAAR	6	12667788	1xTMT6plex [N-Term]	1.02	1.164	1.023	2.397806168	31.92	0	934.5341523	2	467.7705078	-0.44224708	-0.000206647	31.70238335
VNKDDIQK	7	12667788	3xTMT6plex [N-Term; K3; K8]	1.093		1.152	2.694631338	24.85	1	1647.004432	2	824.0057983	-0.067808143	-5.58E-05	41.93964281
IIGLDQVAGMSETALPGAFK	1	12667788	2xTMT6plex [N-Term; K20]; 1xOxidation [M10				1.081138372		0	2492.383468	3	826.1339722	-0.480266351	-0.000396442	98.66422412
TLEEEAK	10	12667788	2xTMT6plex [N-Term; K7]	1.161	0.992	0.983	3.211398363	38.12	0	1277.735303	2	639.3717651	0.743716977	0.000475137	25.33300192
NLPIYSEEIVEMYK	2	12667788	2xTMT6plex [N-Term; K14]				1.530916929	4.06	0	2186.181914	2	1093.592896	-1.555041853	-0.001699802	92.83685742
ADEWLMK	4	12667788	2xTMT6plex [N-Term; K7]; 1xOxidation [M6]	0.948	1.153	100	1.522166729	31.69	0	1366.744094	2	675.8774414	-1.164670336	-0.000786589	65.2501774
MEDSVGCLETAEEVKR	1	12667788	2xTMT6plex [N-Term; K15]; 1xCarbamidomethyl [C7]; 1xOxidation [M1]		1.223		2.19893527		1	2327.162318	3	771.0617676	1.44791951	0.001115461	57.1267798
KTLEEEAK	2	12667788	3xTMT6plex [N-Term; K1; K8]	0.421			2.565201044	10.4	1	1634.993198	3	545.6692505	-7.65E-05	-4.17E-08	38.40184675
IMGIPEEEQMGLLR	2	12667788	1xTMT6plex [N-Term]; 1xOxidation [M]	0.604			1.260435104		0	1860.976072	2	922.9929199	-1.405735902	-0.001296778	73.26113808
NCAAYLK	2	12667788	2xTMT6plex [N-Term; K7]; 1xCarbamidomethyl [C2	1.621			3.065582752	35.36	0	1297.733864	2	649.3717041	1.747348349	0.001133797	39.79516013
QEEEMMAK	2	12667788	2xTMT6plex [N-Term; K8]; 2xOxidation [M5; M6]			0.478	1.532766223	5.55	0	1485.732938	2	727.3748169	-0.516219773	-0.000375225	41.98587944
KDQGELER	4	12667788	2xTMT6plex [N-Term; K1]	1.403	1.511		2.454710484	22.19	1	1432.816013	3	478.2766418	-0.447344868	-0.000213654	29.40682694
KLEMDLK	4	12667788	3xTMT6plex [N-Term; K1; K7]	1.275	1.416		2.961013317	22.5	1	1563.974712	2	782.4904175	-0.737396234	-0.000576635	51.99219288

Accession	Gene_Symbol	Description	Gene_ID	Refseq_Accession	Coverage	Proteins	#Unique_Peptides	#Peptides	#PSMS	#AAs	MW [kDa]	calc. pI	OKF6-STE-6M/OKF6P_R1	OKF6-STE-6M/OKF6P_R2	OKF6-STE-6M/OKF6P_R3	Median fold change	Status	P-value (6M)
256222411	FLNB	filamin-B isoform 1	2317	NP_001157789.1	58.26053931	1	2	114	831	2633	281.459	5.71	0.97	0.92	0.98	0.96	Significant	0.00038

Sequence	#PSMs	Protein Accessions	Modifications	OKF6-STE-6M/OKF6P_R1	OKF6-STE-6M/OKF6P_R2	OKF6-STE-6M/OKF6P_R3	Xcorr	Ionscore	# Missed Cleavages	Theo. MH+ [Da]	Charge	m_z	DeltaM [ppm]	Deltam/z [Da]	RT [min]
YAPTEVGLHEMHIK	12	256222411	2xTMT6plex [N-Term; K14]	0.828	0.361	0.819	5.652737141	48.86	0	2083.141052	3	695.0512695	-0.862633017	-0.000598995	47.98849625
KGEITGEVHMPSGK	10	256222411	3xTMT6plex [N-Term; K1; K14]	1.538	1.406	1.407	4.331250668	30.59	1	2157.230485	4	540.0638428	1.416702467	0.000764038	41.35664579
SPFVVQVGEACNPNACR	6	256222411	1xTMT6plex [N-Term]; 2xCarbamidomethyl [C11; C16	0.906	100	1.113	3.279423952	44.32	0	2134.037109	2	1067.522949	0.708894655	0.000756404	58.28093942
AAGSGELGVTMK	12	256222411	2xTMT6plex [N-Term; K12]; 1xOxidation [M11	0.92	0.847	0.892	3.256871939	75.92	0	1594.887464	2	789.9500732	0.203240161	0.000160447	47.272934
AAGSGELGVTMK	8	256222411	2xTMT6plex [N-Term; K12]	1.116	0.953	0.921	5.584784508	77.13	0	1578.892549	2	789.9500732	0.203240161	0.000160447	47.272934
IPEINSSDMSAHVTSPSGR	8	256222411	1xTMT6plex [N-Term]; 1xOxidation [M9]		1.061	1.21	4.890679359	70.33	0	2230.097126	3	744.0361328	-1.471120904	-0.001093581	39.7974528
IPEINSSDMSAHVTSPSGR	8	256222411	1xTMT6plex [N-Term]	1.062	1.493	1.049	5.581353188	51.92	0	2214.102211	3	744.0361328	-1.471120904	-0.001093581	39.7974528
DAGYGGISLAVEGPSK	6	256222411	2xTMT6plex [N-Term; K16]	0.63		100	3.166222095	52.38	0	1979.084977	2	990.0446777	-1.464485499	-0.001449171	60.12892007
GQHVTGSPFQFTVGPLGEGGAHK	16	256222411	2xTMT6plex [N-Term; K23]	1.034	0.701	0.908	4.885681629	47.45	0	2766.472768	4	692.3719482	-2.459728261	-0.001701193	54.74531345
APSVATVGSICDLNLK	10	256222411	2xTMT6plex [N-Term; K16]; 1xCarbamidomethyl [C11	100	0.854	0.948	4.047980785	38.34	0	2103.188397	3	701.7335205	-1.135612276	-0.000796136	71.76268194
VNIGQGSHPQK	8	256222411	2xTMT6plex [N-Term; K11]	1.052	1.228	0.996	3.962500095	44.98	0	1622.93786	3	541.6505127	-0.539043474	-0.000291611	33.15460707
GAGTGGLGLTVEGPCEAK	6	256222411	2xTMT6plex [N-Term; K18]; 1xCarbamidomethyl [C15	100	100		3.945218325	56.32	0	2132.142175	2	1066.575439	0.669540834	0.000713778	40.52619059
GLHVVEVTYDDVPIPNSPFK	10	256222411	2xTMT6plex [N-Term; K20]	0.847		0.55	4.863924026	65.78	0	2684.469974	3	895.4942017	-0.716040615	-0.00064073	72.3974891
YGGPNHIVGSPFK	4	256222411	2xTMT6plex [N-Term; K13]	1.189	1.356		3.32463932	41.67	0	1831.026675	3	611.013916	0.284084815	0.000173389	59.10989065
VHSPSGAVEECHVSELEPDK	4	256222411	2xTMT6plex [N-Term; K20]; 1xCarbamidomethyl [C11			1.22	6.783832073	42.9	0	2664.333951	4	666.8392334	0.432657381	0.000288186	45.96374503
AHIANPSGASTECFVTDNADGTYQVEYTPFEK	8	256222411	2xTMT6plex [N-Term; K32]; 1xCarbamidomethyl [C13	100	1.673	1.206	4.671991348	50.33	0	3977.890382	3	1326.633545	-1.081182225	-0.001433608	74.48851408
FNDEHIPESPYLVPVIAPSDDAR	6	256222411	1xTMT6plex [N-Term]	2.444	1.603	100	4.606320381	28.61	0	2810.41984	3	937.4779053	-0.240910514	-0.000225687	80.92339284
VLQSFTVDSSK	6	256222411	2xTMT6plex [N-Term; K11]	0.934	1.054	100	4.341757774	68.48	0	1668.957258	2	834.9827881	0.624283139	0.000520951	71.18644401
IEYNDQNDGSCDVK	8	256222411	2xTMT6plex [N-Term; K14]; 1xCarbamidomethyl [C11	1.6	1.282	1.605	4.113658905	46.8	0	2115.006469	2	1058.006592	-0.265770464	-0.000281053	48.52294726
EPGEYAVHIMCDDEDIKDSPYMAFIHPATGGYNPDLVR	5	256222411	2xTMT6plex [N-Term; K17]; 1xCarbamidomethyl [C11	0.526			4.914373398	22.52	1	4781.272619	5	957.0606079	0.274903961	0.000262878	89.0243016
QMQLENVSVALEFLDR	10	256222411	1xTMT6plex [N-Term]	1.974	100		3.62029624	31.81	0	2121.121156	2	1061.063232	-0.927498292	-0.000983668	99.44496327
HVGNQQYNVTYVVK	14	256222411	2xTMT6plex [N-Term; K14]	1.179	1.042	1.06	5.962059021	74.83	0	2107.170044	3	703.0605469	-1.403235115	-0.000985618	42.93298114
VVASGPGLEHGK	6	256222411	2xTMT6plex [N-Term; K12]	0.987	1.032	0.942	4.407282829	53.43	0	1608.947362	3	536.9868164	-0.911006342	-0.000488587	40.07377641
SADFVVESIGSEVGSLGFAIEGPSQAK	80	256222411	2xTMT6plex [N-Term; K27]	1.229	1.074	1.142	4.819457531	48.64	0	3139.65633	3	1047.223999	0.354634455	0.000371143	106.0190807
AHGPGLEGGLVGKPAEFTIDTK	7	256222411	3xTMT6plex [N-Term; K13; K22]	0.745	0.854	0.963	7.213775635	44.61	0	2881.639054	4	721.164978	-0.337200672	-0.000242923	63.77402481
FADEHVPGSPFTVK	16	256222411	2xTMT6plex [N-Term; K14]	1.051	1.143	0.856	3.436097145	41.88	0	1989.084583	3	663.6994629	-0.376006195	-0.000249303	62.69930325
IPYLPITNFNQNWQDGK	11	256222411	2xTMT6plex [N-Term; K17]	1.81	1.026	1.11	3.718952656	50.11	0	2506.349465	3	836.1204834	-1.024611581	-0.000856012	98.17977633
ALGALVDSCAPGLCPDWESWDPQKPVDNAR	10	256222411	2xTMT6plex [N-Term; K24]; 2xCarbamidomethyl [C9; C14		1.261	100	4.666683197	37.22	0	3782.867085	4	946.4699097	-2.452269116	-0.002319152	92.44361519
DLDIIDNYDYSHTVK	8	256222411	2xTMT6plex [N-Term; K15]	0.975	0.932		4.250972271	39.16	0	2269.175249	3	757.0632324	-0.046093564	-3.49E-05	68.36148938
AWGPGLHGGIVGR	10	256222411	1xTMT6plex [N-Term]	0.894	0.839		4.61315012	65.91	0	1505.853847	3	502.6217041	-2.183249935	-0.001095885	49.36873218
EATTDFTVDSRPLTQVGGDHIK	4	256222411	2xTMT6plex [N-Term; K22]	0.518	0.649		5.581129551	22.7	0	2845.509607	4	712.1333008	0.620807955	0.000441629	58.3489046
DAGEGLLAVQITDQEGKPK	2	256222411	3xTMT6plex [N-Term; K17; K19]		0.838		3.052948236	33.69	0	2656.512456	3	886.1740723	-1.804216319	-0.001597641	57.22891677
GEITGEVHMPSGK	12	256222411	2xTMT6plex [N-Term; K13]	1.338	1.286	1.283	3.485159159	35.4	0	1799.97259	3	600.6622925	-0.147729246	-8.86E-05	42.92937216
MDGTYACSYTPVK	4	256222411	2xTMT6plex [N-Term; K13]; 1xCarbamidomethyl [C7	1.626	1.371		3.800438881	52.53	0	1950.970541	2	975.9874268	-1.519533131	-0.001482282	52.01922242
EAGAGGLSIAVEGPSK	4	256222411	2xTMT6plex [N-Term; K16]		100		3.608000517	50.07	0	1901.074413	2	951.0420532	1.271541659	0.001208648	50.56860423
VTASGPGLSSYGVPASLPVDFAIDAR	9	256222411	1xTMT6plex [N-Term]			1.525	3.115795135	35.35	0	2776.471875	3	926.1606445	-1.618912941	-0.001498289	92.89919437
KVVPCLVTPVTGR	16	256222411	2xTMT6plex [N-Term; K1]; 1xCarbamidomethyl [C5	0.646	0.636	0.647	5.352870941	37.02	1	1884.150495	3	628.7218018	0.189376294	0.000118938	56.02156042
ADDTDSQSWR	4	256222411	1xTMT6plex [N-Term]	1.487	1.435		3.874990463	46.08	0	1409.649452	2	705.3279419	-0.599539791	-0.00042257	41.06611751
TATPEIVDNKDGTVTVR	4	256222411	2xTMT6plex [N-Term; K10]	0.978	0.669		5.079841137	23.78	1	2274.270546	3	758.762207	0.669183248	0.000507301	51.76118472
LGSAADFLLDISETDLSSLTASIK	6	256222411	2xTMT6plex [N-Term; K24]		100		2.702002764	23.37	0	2925.607255	3	975.8735352	-0.411125331	-0.00040093	117.5235969
SPFTVGVAAPLDLSK	6	256222411	2xTMT6plex [N-Term; K15]	1.354	0.934	0.96	3.919673681	49.15	0	1960.151935	2	980.5734253	-6.305957127	-0.006180317	75.66201144
VSYFPTVPGVYIVSTK	4	256222411	2xTMT6plex [N-Term; K16]	0.925	1.087		3.379010439	38.18	0	2215.277863	2	1108.141113	-1.31512509	-0.001456684	95.13078265
LTVMSLQESGLK	6	256222411	2xTMT6plex [N-Term; K12]	1.569	100	100	3.736111403	60.35	0	1764.034128	2	882.5202637	-0.497165126	-0.000438508	83.16469289
FVPQEMGVHTVSVK	6	256222411	2xTMT6plex [N-Term; K14]; 1xOxidation [M6	1.389	1.381	1.496	4.306391716	41.66	0	2032.130154	3	678.0419922	-9.217120009	-0.006243462	46.31261003
VHSPSGAVEECHVSELEPDKYAVR	6	256222411	2xTMT6plex [N-Term; K20]; 1xCarbamidomethyl [C11	1.08	0.975	1.023	7.456816673	15.36	1	3153.603918	5	631.526123	-0.763981682	-0.000481859	43.59234937
EVGEHLVSIK	12	256222411	2xTMT6plex [N-Term; K10]	0.833	0.863	0.88	4.668834209	56.34	0	1568.941214	2	784.9741211	-0.158171955	-0.000124081	44.90588207
VEVGKDQEFTVDTR	8	256222411	2xTMT6plex [N-Term; K5]	1.118	1.171	0.968	3.649640799	29	1	2081.127905	2	1041.068481	0.856122596	0.00089085	52.81096978
CVYKPMQPGPHVVK	6	256222411	3xTMT6plex [N-Term; K4; K14]; 1xCarbamidomethyl [C1	0.93	0.912	0.976	4.418566227	18.79	0	2327.333511	4	582.5894165	0.99918882	0.000581361	40.92964104
VDIQTEDLEDGTCK	8	256222411	2xTMT6plex [N-Term; K14]; 1xCarbamidomethyl [C13	1.086	0.73	0.914	3.574944258	41.01	0	2081.047271	2	1041.021118	-5.916036625	-0.006155776	50.88020646
IAGPGLGSGVR	2	256222411	1xTMT6plex [N-Term]	0.886			3.530774832	46.91	0	1212.726187	2	606.8677979	1.758371468	0.001066212	42.80250335
YTPTQQGNMQVLVTYGGDPIPK	6	256222411	2xTMT6plex [N-Term; K22]	1.604	0.898	0.562	3.654643536	30.13	0	2865.522086	3	955.8452759	-0.283146755	-0.000270454	79.71169026
VKVEPAVDTSR	14	256222411	2xTMT6plex [N-Term; K2]	0.711	0.762	0.702	3.949471235	27.43	1	1658.984141	2	829.9963989	0.831907629	0.000690061	35.93795281
VTEAEIVPMGK	4	256222411	2xTMT6plex [N-Term; K11]	1.75	1.618		2.869454622	35.89	0	1631.94425	2	816.4752197	-0.666289228	-0.000543673	53.59100449

Bhat et al,2017. Cigarette smoke and chewing tobacco alter expression of different sets of miRNAs in oral keratinocytes

Supplementary Table S2(A). List of all proteins and peptides identified in OKF6/TERT1 cells chronically treated with chewing tobacco using Sequest and Mascot search algorithms

Accession	Gene_Symbol	Description	Gene_ID	Refseq_Accession	Coverage	Proteins	#Unique_Peptides	#Peptides	#PSMS	#AAs	MW [kDa]	calc. pI	OKF6-STE-6M/OKF6P_R1	OKF6-STE-6M/OKF6P_R2	OKF6-STE-6M/OKF6P_R3	Median fold change	Status	P-value (6M)	Sequence	#PSMSs	Protein Accessions	Modifications	OKF6-STE-6M/OKF6P_R1	OKF6-STE-6M/OKF6P_R2	OKF6-STE-6M/OKF6P_R3	Xcorr	Ionscore	# Missed Cleavages	Theo. MH+ [Da]	Charge	m_z	DeltaM [ppm]	Deltam/z [Da]	RT [min]
																			YAPTEVGLHEMHIK	10	256222411	2xTMT6plex [N-Term; K14]; 1xOxidation [M11	0.796	0.933	0.86	4.314597607	27.16	0	2099.135967	3	695.0512695	-0.862633017	-0.000598995	47.98849625
																			VFGPGIEGK	4	256222411	2xTMT6plex [N-Term; K9]	1.219	1.163		3.479481936	55.94	0	1361.819308	2	681.4134521	0.234874681	0.000159928	59.73578834
																			AEITFDDHK	12	256222411	2xTMT6plex [N-Term; K9]	1.089	1.054	0.866	3.985968351	45.27	0	1533.831329	3	511.9486389	0.022530539	1.15E-05	47.85191011
																			SPFEVSVDK	8	256222411	2xTMT6plex [N-Term; K9]	0.908	1.058	0.87	4.007147312	54.61	0	1465.830266	2	733.4182129	-0.762147416	-0.000558589	65.92866816
																			VAVTEGCQPSR	8	256222411	1xTMT6plex [N-Term]; 1xCarbamidomethyl [C7	1.147	1.152	1.202	4.05578661	39.84	0	1432.741579	2	716.8742676	-0.223608887	-0.000160187	29.10263738
																			AEVSIQNNK	8	256222411	2xTMT6plex [N-Term; K9]	1.055	1.069	1.023	4.298034668	43.25	0	1460.847313	2	730.9273682	0.100173457	7.32E-05	39.64225426
																			DGTYAVTYIPDK	2	256222411	2xTMT6plex [N-Term; K12]		100		2.240540981	19.32	0	1800.978387	2	900.9918213	-1.122174419	-0.001010506	53.33594425
																			DGTCTVTYLPTLPGDYSILVK	4	256222411	2xTMT6plex [N-Term; K21]; 1xCarbamidomethyl [C4				2.424680233	13.56	0	2771.49414	3	924.5040894	1.289851692	0.001191605	100.5399732
																			ATQTGDASK	15	256222411	2xTMT6plex [N-Term; K9]	1.029	0.964	1.078	3.819610834	45.07	0	1336.747265	2	668.8773193	0.072595529	4.85E-05	23.4065598
																			GEAGVPAEFSIWTR	17	256222411	1xTMT6plex [N-Term]	1.179	1.067	0.809	3.030471087	33.1	0	1748.9169	2	874.961731	-0.408827851	-0.000357503	80.95334595
																			SSFLVDCSK	7	256222411	2xTMT6plex [N-Term; K9]; 1xCarbamidomethyl [C7	1.106	1.148	1.35	4.374504566	56.29	0	1500.813236	2	750.9110718	1.086447433	0.000815277	51.79069762
																			TYSVEYLPK	4	256222411	2xTMT6plex [N-Term; K9]		1.135	1.404	2.667188406	32.69	0	1557.892867	2	779.449646	-0.546384049	-0.000425604	61.20546414
																			VQAQGPGLK	2	256222411	2xTMT6plex [N-Term; K9]	0.984			3.683722258	38.04	0	1355.841106	2	678.4237671	-0.625678266	-0.00042416	41.00645763
																			IFAQDGEGQR	4	256222411	1xTMT6plex [N-Term]		1.074	1.983	3.472350121	36.97	0	1349.701094	2	675.3543091	0.183384353	0.000123757	38.36583252
																			TATFTIVTEDAGEGGLDLAIEGPSK	4	256222411	2xTMT6plex [N-Term; K25]				2.137927055	18.31	0	2950.566118	3	984.1935425	-0.015003424	-1.48E-05	118.7690749
																			LPNNHIGISFIPR	2	256222411	1xTMT6plex [N-Term]	1.01			3.282739401	24.21	0	1706.99034	3	569.6681519	-0.256453469	-0.000145921	73.40587044
																			ADIEMPFDPSK	13	256222411	2xTMT6plex [N-Term; K11]	1.151	0.881	1.047	2.489080191	27.07	0	1707.902779	2	854.4559326	1.059401271	0.000904677	56.69289442
																			EAFTNKPNVFTVVTR	5	256222411	2xTMT6plex [N-Term; K6]	0.683	0.721	100	2.540704966	11.18	0	2181.243209	3	727.7521973	-0.536658691	-0.000390194	57.93816636
																			YMIGVTYGGDDIPLSPYR	3	256222411	1xTMT6plex [N-Term]	0.942	100		3.046576262	19.96	0	2246.136471	2	1123.571167	-0.629474497	-0.000706943	93.76641083
																			MDCQETPEGYK	6	256222411	2xTMT6plex [N-Term; K11]; 1xCarbamidomethyl [C3	1.397	0.638	1.085	3.076840401	36.63	0	1815.865742	2	908.4369507	0.486091657	0.000441339	46.26692013
																			DLAEDAPWK	17	256222411	2xTMT6plex [N-Term; K9]	1.295	1.401	1.716	4.300972462	53.74	0	1502.825515	2	751.9154663	-1.237211369	-0.000929656	63.03048465
																			LKPGAPLKPK	10	256222411	4xTMT6plex [N-Term; K2; K8; K10]	0.894	1.112	1.096	2.579379797	15.27	0	1965.339455	3	655.784668	-0.002164384	-1.42E-06	49.97639713
																			NTVELLVEDK	6	256222411	2xTMT6plex [N-Term; K10]	1.05	2.444	100	3.054084539	33.29	0	1617.946359	2	809.4765015	-0.39081043	-0.000316155	69.13555924
																			AEISCIDNK	6	256222411	2xTMT6plex [N-Term; K9]; 1xCarbamidomethyl [C5	0.947	0.998	0.935	3.898831367	49.18	0	1507.81905	2	754.4133911	0.302155993	0.000227798	47.97996648
																			VLFASQEIPASPFR	8	256222411	1xTMT6plex [N-Term]	0.923	0.763	1.239	2.431885481	31.02	0	1791.000236	2	896.0018921	-2.081764278	-0.00186422	80.47361689
																			VMYTPMAPGNYLISVK	4	256222411	2xTMT6plex [N-Term; K16]	0.564		1.209	2.51602602	22.35	0	2242.237989	2	1121.621826	-0.719556201	-0.000806708	103.5025837
																			GDYVLAVK	8	256222411	2xTMT6plex [N-Term; K8]	1.182	1.295	1.23	3.182701588	50.03	0	1322.808409	2	661.9076538	-0.285599041	-0.000188896	49.36617506
																			VEPAVDTSR	4	256222411	1xTMT6plex [N-Term]	1.375	1.263		3.245295286	44.22	0	1202.657832	2	601.8325806	0.04343115	2.61E-05	34.16138754
																			YMGSHIPESPLQFYVNYPNSGSVSAYGPGLVYGVANK	4	256222411	2xTMT6plex [N-Term; K37]				1.947907448	3.94	0	4421.231664	4	1106.060059	-2.998975401	-0.003314791	94.4076181
																			SPFEVQVGPEAGMQK	4	256222411	2xTMT6plex [N-Term; K15]	100	100		2.881113052	29.26	0	2062.104332	2	1031.557251	1.402883976	0.001446447	66.60216077
																			VKAEGPGLSK	4	256222411	3xTMT6plex [N-Term; K2; K10]	0.61	0.576		3.450109959	19.02	1	1673.056467	3	558.3566895	-0.569144344	-0.000317404	42.63813313
																			DLAEDAPWKK	2	256222411	3xTMT6plex [N-Term; K9; K10]		0.573		2.762537003	9.63	1	1860.08341	3	620.6989136	-0.657388447	-0.000407599	62.45597215
																			YGGELVPHFPAR	4	256222411	1xTMT6plex [N-Term]	0.774	0.829		2.396565676	14.95	0	1571.853178	3	524.6226838	0.547181254	0.000286696	59.06740296
																			VTVLFAGQHISK	4	256222411	2xTMT6plex [N-Term; K12]	1.692			2.080852032	16.2	0	1758.067811	3	586.6940918	-0.050646155	-2.97E-05	54.61209265
																			LLGWIQNK	6	256222411	2xTMT6plex [N-Term; K8]	1.004	1.182	1.43	3.481220961	36.33	0	1429.893141	2	715.4491577	-1.470494718	-0.001051325	88.43098523
																			AEGPGLSK	4	256222411	2xTMT6plex [N-Term; K8]	0.911	1.128		3.255458832	52.68	0	1216.730158	2	608.8693237	0.996400828	0.000606175	33.32106065
																			TATPEIVDNK	2	256222411	2xTMT6plex [N-Term; K10]	0.982			4.153255939	50.94	0	1545.888844	2	773.4481201	0.077518106	5.99E-05	46.74742397
																			VPVKDVVDPSK	10	256222411	3xTMT6plex [N-Term; K4; K11]	0.74	0.679	0.738	2.667834759	14.52	1	1870.161661	3	624.05896	0.356152533	0.000222021	45.70789811
																			VVPCLVTPVTGR	6	256222411	1xTMT6plex [N-Term]; 1xCarbamidomethyl [C4	1.182	1.102	1.225	3.535137892	19.13	0	1526.8926	2	763.9492798	-0.86273238	-0.00065865	62.93304465
																			LDVTILSPSR	3	256222411	1xTMT6plex [N-Term]	100	100		3.056127787	25.32	0	1329.793932	2	665.3995361	-1.606372479	-0.001068072	64.45087635
																			HIPGSPFTAK	4	256222411	2xTMT6plex [N-Term; K10]	0.917			3.513520002	39.81	0	1512.89387	3	504.9702148	1.468438266	0.00074053	46.93274358
																			APLNVQFNSPLPGDAVK	9	256222411	2xTMT6plex [N-Term; K17]		100	0.712	2.628546	37.16	0	2225.269424	3	742.4333496	7.222374043	0.005357243	68.31201968
																			GIEPTGNMVK	2	256222411	2xTMT6plex [N-Term; K10]; 1xOxidation [M8	0.82			2.043748856	17.48	0	1519.855436	2	760.4306641	-0.910688588	-0.000692057	40.57478893
																			VKVDPSHDASK	2	256222411	3xTMT6plex [N-Term; K2; K11]	0.673			3.128416061	14.79	1	1870.100123	4	468.2796631	-1.76486072	-0.000825117	34.85368514
																			ETTDFKVDTK	4	256222411	3xTMT6plex [N-Term; K6; K10]	0.745	0.291	0.423	2.363007784	6.41	1	1871.072905	3	624.3621216	-0.58447958	-0.000364535	41.12385567
																			GLEELVK	10	256222411	2xTMT6plex [N-Term; K7]	0.88	0.983	0.599	2.899133682	40.07	0	1245.78186	2	623.3949585	0.626652396	0.000390336	62.41930882
																			GPGLEAVGNIANKPTYFDIYTAGAGVGDIGVEVEDPQGK	2	256222411	3xTMT6plex [N-Term; K13; K39]				1.571613193	5.7	0	4606.428398	4	1152.359253	-2.869086439	-0.00330406	119.8170648
																			FVPQEMGVHTVSVK	2	256222411	2xTMT6plex [N-Term; K14]		100		1.885838747	8.35	0	2016.135239	3	678.0419922	-9.217120009	-0.006243462	46.31261003
																			YADEEIPR	4	256222411	1xTMT6plex [N-Term]	1.155		1.143	3.507359505	47.13	0	1221.631283	2	611.3154297	-6.303403577	-0.003850217	37.99974782
																			AYGPGLEK	4	256222411	2xTMT6plex [N-Term; K8]	0.921	0.871		3.006130934	39.18	0	1292.761459	2	646.8839722	-0.611786542	-0.000395447	48.0884883
																			DGTVTVR	4	256222411	1xTMT6plex [N-Term]	1.237	1.093		2.73395133	41.99	0	976.5624755	2	488.7849121	0.073870082	3.61E-05	26.84352864
																			AIVHDNK	2	256222411	2xTMT6plex [N-Term; K7]			1.139	2.878873587	22.03	0	1254.757042	3	418.9237061	-0.380181917	-0.000159012	30.35395101
																			GAGGQGK	4	256222411	2xTMT6plex [N-Term; K7]	100	100		2.43762517	41.35	0	1032.620214	2	516.8138428	0.188565818	9.74E-05	13.39554974
																			IGNLQTDLSDGLR	6	256222411	1xTMT6plex [N-Term]	100			1.765077353	15.29	0	1630.896165	2	815.951416	-0.373677223	-0.000304714	63.11345632
																			AQGDASK	4	256222411	2xTMT6plex [N-Term; K7]	0.765	1.188		2.645100594	33.16	0	1134.651908	2	567.8294067	-0.327266393	-0.000185667	21.37539074
																			TFEMSDFIVDTR	4	256222411	1xTMT6plex [N-Term]				1.871363044	8.12	0	1689.835538	2	845.4198608	-1.830586612	-0.001546695	74.43097766
																			FTVDTISAGQGDVMVFVEDPEGNKEEAQVTPDSDK	2	256222411	3xTMT6plex [N-Term; K24; K35]				1.635497689	1.19	1	4442.216416	4	1111.309937	0.337721896	0.000375058	93.49408189
																			ALSEFFK	2	256222411	2xTMT6plex [N-Term; K7]	100			2.171847582	22.91	0	1299.771295	2	650.3885498	-1.13254588	-0.000736025	86.40483138
																			WCNEHLK	4	256222411	2xTMT6plex [N-Term; K7]; 1xCarbamidomethyl [C2	1.264			2.917763948	31.53	0	1444.777126	2	722.8922729	0.099349883	7.18E-05	44.31099071
																			VGEAGLLSVDCSEAGPGALGLEAVSDSGTK	2	256222411	2xTMT6plex [N-Term; K30]; 1xCarbamidomethyl [C11				1.327359557		0	3304.698272	3	1102.235962	-1.494610826	-0.001646413	86.22991462
																			RLPNNHIGISFIPR	2	256222411	1xTMT6plex [N-Term]	0.66			2.918626308	8.14	1	1863.091451	4	466.5279541	-0.786066486	-0.000366128	54.63997907
																			DVVDPSK	2	256222411	2xTMT6plex [N-Term; K7]	1.196			2.642720699	19.39	0	1217.714174	2	609.3609619	0.388554338	0.000236754	38.47013423
																			DEPCLLK	6	256222411	2xTMT6plex [N-Term; K7]; 1xCarbamidomethyl [C4	1.076	1.175	1.04	2.859192848	27.41	0	1332.759744	2	666.8837891	0.418053593	0.000278583	52.41215599
																			VLPTYDASK	2	256222411	2xTMT6plex [N-Term; K9]			1.252	2.181076527	23.71	0	1451.851002	2	726.4296265	0.6712188	0.000487255	55.38485949
																			VFGPGVER	2	256222411	1xTMT6plex [N-Term]			0.86	1.976571679	30.42	0	1089.62541	2	545.3164063	0.115461698	6.29E-05	45.11794617
160420317	FLNA	filamin-A isoform 2	2316	NP_001104026.1	51.41669815	1	91	98	803	2647	280.564	6.06	1.35	1.31	1.32	1.33	Significant	0.000082																
																			VTAQGPGLEPSGNIANK	8	160420317	2xTMT6plex [N-Term; K17]	1.737	1.787	1.648	4.610630035	65.82	0	2111.186088	2	1056.09668	-0.002555436	-2.70E-06	45.30445602
																			VANPSGNLTETYVQDR	6	160420317	1xTMT6plex [N-Term]	1.653	1.495	1.767	4.169687271	52.16	0	1993.018799	2	997.0136108	0.575047104	0.00057304	45.2651036
																			LQVEPAVDTSGVQCYGPGIEGQGVFR	15	160420317	1xTMT6plex [N-Term]; 1xCarbamidomethyl [C14	1.602	0.976	100	3.16502738	25.83	0	2992.503586	3	998.1680908	-4.63392897	-0.00462235	70.74067466
																			ALGALVDSCAPGLCPDWDSWDASKPVTNAR	8	160420317	2xTMT6plex [N-Term; K24]; 2xCarbamidomethyl [C9; C14	1.93	1.615	3.757	3.330980539	30.19	0	3687.829972	4	922.7055054	-8.075221974	-0.007445011	82.39875866
																			EAGAGGLAIAVEGPSK	8	160420317	2xTMT6plex [N-Term; K16]	1.378	100	1.621	4.558171272	76.1	0	1885.079498	2	943.045166	1.887178368	0.001778741	58.43311575
																			DAGEGLLAVQITDPEGKPK	8	160420317	3xTMT6plex [N-Term; K17; K19]	100	1.005	100	3.248421907	26.77	0	2625.506643	3	875.840271	-0.145915349	-0.000127701	66.73892892
																			SPYTVTVGQACNPSACR	16	160420317	1xTMT6plex [N-Term]; 2xCarbamidomethyl [C11; C16	1.778	1.358	1.508	5.245191574	61.91	0	2097.005475	2	1049.006836	0.439071338	0.000460368	38.98003727
																			AGNNMLLVGVHGPR	8	160420317	1xTMT6plex [N-Term]	1.945	2.075	1.431	4.215191841	58.28	0	1663.92636	3	555.3135986	-0.070304531	-3.90E-05	45.41977478
																			FVPAEMGTHTVSVK	6	160420317	2xTMT6plex [N-Term; K14]	1.507	1.29	1.402	5.118516445	45.58	0	1961.09304	3	654.3686523	-0.834110732	-0.000545256	50.29222062
																			VEPGLGADNSVVR	2	160420317	1xTMT6plex [N-Term]	1.426			3.863450289	54.3	0	1541.848486	2	771.4271851	-0.90341744	-0.000696466	49.94826796
																			AWGPGLEGGVVGK	7	160420317	2xTMT6plex [N-Term; K13]	1.103	1.636	0.895	3.982056141	69.43	0	1684.978662	2	842.9919434	-1.217671948	-0.001025876	58.1112047
																			ATCAPQHGAPGPGPADASK	6	160420317	2xTMT6plex [N-Term; K19]; 1xCarbamidomethyl [C3	1.655	1.933	1.512	6.630395412	65.82	0	2248.154471	3	750.0559082	-0.577972129	-0.000433124	32.72865673
																			VDINTEDLEDGTCR	8	160420317	1xTMT6plex [N-Term]; 1xCarbamidomethyl [C13	1.543	1.465	1.383	3.025089502	40.52	0	1865.874837	2	933.4425049	1.552057816	0.001447973	47.7734489
																			GAGTGGLGLAVEGPSEAK	6	160420317	2xTMT6plex [N-Term; K18]	100	100	100	4.174788475	59.83	0	2029.13299	2	1015.070679	0.537585205	0.000545416	47.6964701
																			VHSPSGALEECYVTEIDQDK	6	160420317	2xTMT6plex [N-Term; K20]; 1xCarbamidomethyl [C11	0.889	100	2.369	4.421214104	34.26	0	2735.359831	3	912.4573975	-0.801425524	-0.000730729	55.15037089
																			THEAEIVEGENHTYCIR	11	160420317	1xTMT6plex [N-Term]; 1xCarbamidomethyl [C15	1.105	1.625	1.8	7.052743435	52.5	0	2287.09746	4	572.5298462	0.041395536	2.37E-05	38.77923734
																			APSVANVGSHCDLSLK	4	160420317	2xTMT6plex [N-Term; K16]; 1xCarbamidomethyl [C11	1.279	1.299		6.189416409	49.39	0	2113.147594	3	705.0540161	-0.047043641	-3.31E-05	57.58176667
																			VNVGAGSHPNK	8	160420317	2xTMT6plex [N-Term; K11]	1.66	1.527	1.616	4.039983273	41.18	0	1537.885096	3	513.2995605	-0.629090806	-0.00032249	39.20492526
																			YAPSEAGLHEMDIR	12	160420317	1xTMT6plex [N-Term]	1.229	1.274	1.23	4.728778839	68.74	0	1817.905349	2	909.4476318	-9.550683225	-0.008681119	46.81510631
																			YAPSEAGLHEMDIR	6	160420317	1xTMT6plex [N-Term]; 1xOxidation [M11]	0.819	1.334		4.159446716	47.77	0	1833.900264	2	909.4476318	-9.550683225	-0.008681119	46.81510631
																			SPFSVAVSPSLDLSK	12	160420317	2xTMT6plex [N-Term; K15]	1.402		0.691	3.505364418	51.12	0	1992.141764	2	996.5762329	1.719456102	0.0017127	95.0129736
																			RAPSVANVGSHCDLSLK	4	160420317	2xTMT6plex [N-Term; K17]; 1xCarbamidomethyl [C12	1.293	1.138		7.128590584	45.94	1	2269.248705	4	568.0673828	-0.442476842	-0.000251022	48.64320594
																			DAGEGGLSLAIEGPSK	4	160420317	2xTMT6plex [N-Term; K16]	1.181	1.791		4.192687988	79.18	0	1959.079892	2	980.0438843	0.306340078	0.000300072	67.04056274
																			SAGQGEVLVYVEDPAGHQEEAK	8	160420317	2xTMT6plex [N-Term; K22]	1.456	1.379		7.621052742	87.9	0	2771.425208	3	924.4793701	-0.595840881	-0.000550443	62.17176421
																			FNEEHIPDSPFVVPVASPSGDAR	7	160420317	1xTMT6plex [N-Term]	1.868		1.083	4.051708221	24.1	0	2696.35176	3	899.4547119	-0.807623784	-0.000725879	73.5569594
																			AHEPTYFTVDCAEAGQGDVSIGIK	10	160420317	2xTMT6plex [N-Term; K24]; 1xCarbamidomethyl [C11	2.15	1.44	100	5.248930931	40.84	0	3023.518457	3	1008.51001	-0.98596161	-0.000993691	71.32347818
																			ALTQTGGPHVK	8	160420317	2xTMT6plex [N-Term; K11]	1.328	1.386		4.654204369	56.39	0	1566.936797	3	522.9833984	-0.737111734	-0.000385002	28.78293923
																			QMQLENVSVALEFLDR	10	160420317	1xTMT6plex [N-Term]	1.974	100		3.62029624	31.81	0	2121.121156	2	1061.063232	-0.927498292	-0.000983668	99.44496327
																			YNEQHVPGSPFTAR	12	160420317	1xTMT6plex [N-Term]	1.667	1.516	1.761	3.890192747	39.59	0	1831.928862	3	611.3143921	-0.130414709	-7.96E-05	47.83694378
																			AFGPGLQGGSAGSPAR	14	160420317	1xTMT6plex [N-Term]	1.332	1.38	1.328	5.404365063	92.79	0	1658.881184	2	829.9438477	-0.461050199	-0.000382414	37.30435088
																			VTCTVCTPDGSEVDVDVVENEDGTFDIFYTAPQPGK	69	160420317	2xTMT6plex [N-Term; K36]; 2xCarbamidomethyl [C3; C6	1.45	1.749	1.233	3.526330233	25.93	0	4420.088884	4	1105.778076	0.359955997	0.000397759	99.99349694
																			ANLPQSFQVDTSK	9	160420317	2xTMT6plex [N-Term; K13]	1.46	1.167	1.222	4.094137669	52.74	0	1893.048198	2	947.0272217	-0.544661581	-0.000515535	60.30580172
																			GQHVPGSPFQFTVGPLGEGGAHK	8	160420317	2xTMT6plex [N-Term; K23]	1.686	1.577		4.068607807	35.53	0	2762.477853	4	691.3753052	0.556588399	0.000384391	55.62334836
																			GLVEPVDVVDNADGTQTVNYVPSR	9	160420317	1xTMT6plex [N-Term]	100	1.051	1.055	3.808677197	48.61	0	2773.420568	3	925.1442261	-0.880821096	-0.000814296	63.09927829
																			LVSNHSLHETSSVFVDSLTK	6	160420317	2xTMT6plex [N-Term; K20]		100	100	4.057463169	31.98	0	2658.450301	3	886.8208618	-0.853452397	-0.000756287	72.38817827
																			EEGPYEVEVTYDGVPVPGSPFPLEAVAPTKPSK	2	160420317	3xTMT6plex [N-Term; K30; K33]				3.506798506	16.7	0	4173.225054	4	1044.060303	-1.359383313	-0.001418253	88.52073962
																			AEISCTDNQDGTCSVSYLPVLPGDYSILVK	13	160420317	2xTMT6plex [N-Term; K30]; 2xCarbamidomethyl [C5; C13	1.2	2.956	1.311	4.115255356	32.03	0	3759.886149	4	940.723999	-3.187010397	-0.002995699	96.5228649
																			LTVSSLQESGLK	5	160420317	2xTMT6plex [N-Term; K12]	2.24	1.92		4.328017235	65.13	0	1720.025672	2	860.5175171	1.21279567	0.00104302	65.54034258
																			VAQPTITDNKDGTVTVR	6	160420317	2xTMT6plex [N-Term; K10]	1.383	1.015		4.875253201	26.56	1	2273.28653	3	758.4332275	-0.616289585	-0.000467001	42.75031518
																			DVDIIDHHDNTYTVK	12	160420317	2xTMT6plex [N-Term; K15]	1.349	1.459	100	4.785354614	46.91	0	2243.170832	3	748.3951416	0.017655672	1.32E-05	44.61743575
																			ASGPGLNTTGVPASLPVEFTIDAK	6	160420317	2xTMT6plex [N-Term; K24]	100	1.021	100	3.406463385	27.51	0	2800.549681	3	934.1885376	0.492354404	0.000459621	91.69752838
																			DGSCSVEYIPYEAGTYSLNVTYGGHQVPGSPFK	2	160420317	2xTMT6plex [N-Term; K33]; 1xCarbamidomethyl [C4			100	3.267163038	11.11	0	4037.963153	4	1010.245728	-0.513403335	-0.000518276	85.6049436
																			EATTEFSVDAR	32	160420317	1xTMT6plex [N-Term]	1.447	1.517	1.538	5.033298492	72.61	0	1454.732454	2	727.8701782	0.430447063	0.000313093	46.78676511
																			VPVHDVTDASK	14	160420317	2xTMT6plex [N-Term; K11]	1.177	1.217	1.079	3.883363485	61.15	0	1625.926292	2	813.4656982	-1.335875824	-0.001086018	30.16231976
																			YGGPYHIGGSPFK	12	160420317	2xTMT6plex [N-Term; K13]		1.306	2.483	3.010603666	33.71	0	1838.000126	3	613.338501	0.448389353	0.000274713	59.3878648
																			GAGSYTIMVLFADQATPTSPIR	4	160420317	1xTMT6plex [N-Term]	100			3.68240118	20.46	0	2525.327125	3	842.4459229	-1.548352003	-0.001303365	97.83730403
																			SADFVVEAIGDDVGTLGFSVEGPSQAK	9	160420317	2xTMT6plex [N-Term; K27]	100			3.217819452	29.74	0	3153.635595	3	1051.887573	3.986366269	0.004190516	116.2983304
																			VTVLFAGQHIAK	8	160420317	2xTMT6plex [N-Term; K12]	1.311	1.259	2.157	4.016952515	40.24	0	1742.072897	3	581.3616943	-1.358624081	-0.000788941	69.22519405
																			TFSVWYVPEVTGTHK	4	160420317	2xTMT6plex [N-Term; K15]	1.434	100		3.283524036	24.12	0	2209.205761	3	737.0734253	-0.017373254	-1.28E-05	87.38616027
																			LDVQFSGLTK	6	160420317	2xTMT6plex [N-Term; K10]	1.791	2.433	100	3.936377048	58.69	0	1565.930315	2	783.468689	-0.136270631	-0.000106695	76.85762368
																			GTVEPQLEAR	10	160420317	1xTMT6plex [N-Term]	1.432	1.336	1.423	4.003969193	63.66	0	1328.737145	2	664.8726807	0.70708351	0.000469764	43.93526819
																			IECDDKGDGSCDVR	8	160420317	2xTMT6plex [N-Term; K6]; 2xCarbamidomethyl [C3; C11	2.376	2.156		4.119438648	30.93	1	2083.978874	2	1042.494507	1.373618471	0.001431296	34.46456981
																			IPEISIQDMTAQVTSPSGK	2	160420317	2xTMT6plex [N-Term; K19]		2.169		3.293464184	21.34	0	2460.341996	3	820.7860107	0.602646528	0.000494239	85.33099708
																			DKGEYTLVVK	18	160420317	3xTMT6plex [N-Term; K2; K10]	1.47	1.63	1.597	4.491065023	42.2	1	1839.119461	2	920.0544434	-9.706450084	-0.008925661	47.11300229
																			VDVGKDQEFTVK	8	160420317	3xTMT6plex [N-Term; K5; K12]	1.092	1.398	1.049	3.022209883	22.89	1	2052.194417	3	684.7359619	-0.528535602	-0.000361553	58.01368721
																			YGGDEIPFSPYR	5	160420317	1xTMT6plex [N-Term]	100			3.405335426	23.12	0	1629.811038	2	815.4084473	-0.871511308	-0.000710199	65.4672113
																			WGDEHIPGSPYR	6	160420317	1xTMT6plex [N-Term]	2.193	1.79	1.902	3.383687496	29.5	0	1642.817521	3	548.2785034	2.091725869	0.001145441	51.71800379
																			CSGPGLSPGMVR	2	160420317	1xTMT6plex [N-Term]; 1xCarbamidomethyl [C1	1.331			4.052734852	58.21	0	1446.73947	2	723.8723755	-1.37973248	-0.000998057	42.03953273
																			YGGQPVPNFPSK	6	160420317	2xTMT6plex [N-Term; K12]	100	100		2.549159527	34.89	0	1748.973576	2	874.9897461	-0.778103522	-0.000680441	52.504031
																			FADQHVPGSPFSVK	6	160420317	2xTMT6plex [N-Term; K14]	0.597	100	100	2.558298826	28.5	0	1974.084918	3	658.7005615	1.121339965	0.000737873	52.76542693
																			AVPTGDASK	8	160420317	2xTMT6plex [N-Term; K9]	1.126	1.167	1.14	2.986764908	48.01	0	1303.762187	2	652.3847656	0.051995679	3.39E-05	30.93933126
																			TPCEEILVK	6	160420317	2xTMT6plex [N-Term; K9]; 1xCarbamidomethyl [C3	1.488	1.628	1.606	3.505600691	50.76	0	1546.891487	2	773.9451294	-5.497807061	-0.004252255	49.39252522
																			DAPQDFHPDR	8	160420317	1xTMT6plex [N-Term]	1.334	1.298	1.259	4.298020363	50.32	0	1426.691258	3	476.2357178	0.940967645	0.00044749	25.1089875
																			MDCQECPEGYR	2	160420317	1xTMT6plex [N-Term]; 2xCarbamidomethyl [C3; C6	1.621			3.769823313	44.47	0	1673.691928	2	837.3493042	-0.356297089	-0.000298166	37.87765633
																			AHVVPCFDASK	7	160420317	2xTMT6plex [N-Term; K11]; 1xCarbamidomethyl [C6	1.226	0.793	1.11	3.111355305	46.65	0	1688.919433	3	563.6444702	-0.340658919	-0.000191782	43.73927815
																			DAEMPATEK	6	160420317	2xTMT6plex [N-Term; K9]	1.444	1.268		3.727119684	46.61	0	1449.765952	2	725.387146	0.73380961	0.000531926	44.85653755
																			DLAEDAPWK	17	160420317	2xTMT6plex [N-Term; K9]	1.295	1.401	1.716	4.300972462	53.74	0	1502.825515	2	751.9154663	-1.237211369	-0.000929656	63.03048465
																			NDNDTFTVK	4	160420317	2xTMT6plex [N-Term; K9]	2.014	1.961		4.098739624	51.68	0	1511.810593	2	756.4092407	0.404379567	0.000305673	48.35447147
																			VLPTHDASK	2	160420317	2xTMT6plex [N-Term; K9]	1.279			3.856505156	41.66	0	1425.846585	3	475.9542542	1.139015367	0.000541354	34.99596307
																			SPFEVYVDK	6	160420317	2xTMT6plex [N-Term; K9]	1.456	1.538	1.818	3.116574526	29.06	0	1541.861566	2	771.4356689	1.618050984	0.001247405	67.92977242
																			LSPFMADIR	8	160420317	1xTMT6plex [N-Term]	0.635	0.545		3.181017876	37.88	0	1278.707759	2	639.8569946	-0.818625037	-0.000523391	79.24159272
																			GAGSGELK	4	160420317	2xTMT6plex [N-Term; K8]		1.326	1.171	2.688839912	45.17	0	1176.698858	2	588.852356	-1.209379891	-0.000711538	30.98296744
																			GKLDVQFSGLTK	6	160420317	3xTMT6plex [N-Term; K2; K12]	1.047	1.087	1.104	4.025177002	19.8	1	1980.209673	3	660.7406616	-1.132070381	-0.000747246	55.53641379
																			GEYTLVVK	2	160420317	2xTMT6plex [N-Term; K8]	1.031			2.851415634	44.98	0	1366.834624	2	683.9213257	0.549618202	0.000375619	54.32431621
																			DLAEDAPWKK	2	160420317	3xTMT6plex [N-Term; K9; K10]		0.573		2.762537003	9.63	1	1860.08341	3	620.6989136	-0.657388447	-0.000407599	62.45597215
																			AEAGVPAEFSIWTR	12	160420317	1xTMT6plex [N-Term]	1.478	1.813	1.185	2.606739759	30.57	0	1762.93255	2	881.9684448	-1.666162192	-0.001468666	71.23410032
																			VKAEGPGLSR	4	160420317	2xTMT6plex [N-Term; K2]	0.965	0.798		2.847208261	11.69	1	1471.899683	3	491.3048401	0.192739815	9.46E-05	31.50695988
																			LLGWIQNK	6	160420317	2xTMT6plex [N-Term; K8]	1.004	1.182	1.43	3.481220961	36.33	0	1429.893141	2	715.4491577	-1.470494718	-0.001051325	88.43098523
																			KDGSCGVAYVVQEPGDYEVSVK	3	160420317	3xTMT6plex [N-Term; K1; K22]; 1xCarbamidomethyl [C5				1.564265847	0.37	1	3073.611913	3	1025.208496	-0.318068376	-0.000325873	54.65433467
																			MDCQECPEGYR	2	160420317	1xTMT6plex [N-Term]; 2xCarbamidomethyl [C3; C6]; 1xOxidation [M1	1.451			2.693662167	21.2	0	1689.686843	2	837.3493042	-0.356297089	-0.000298166	37.87765633
																			FVPAEMGTHTVSVK	10	160420317	2xTMT6plex [N-Term; K14]; 1xOxidation [M6	2.096	1.927	0.951	4.154501915	33.07	0	1977.087955	3	654.3686523	-0.834110732	-0.000545256	50.29222062
																			AEISFEDR	16	160420317	1xTMT6plex [N-Term]	1.09	1.269	1.168	3.513823986	53.23	0	1195.615633	2	598.3079224	-5.909109284	-0.003532512	41.58363539
																			VQVQDNEGCPVEALVK	3	160420317	2xTMT6plex [N-Term; K16]; 1xCarbamidomethyl [C9			100	1.799162149	8.4	0	2243.210589	2	1122.106689	-1.99995211	-0.002243157	66.37515016
																			GEITGEVR	20	160420317	1xTMT6plex [N-Term]	1.364	1.388	1.474	2.878591299	46.79	0	1089.610154	2	545.3087158	0.001055997	5.75E-07	34.13957044
																			YWPQAEGEYAVHVLCNSEDIR	5	160420317	1xTMT6plex [N-Term]; 1xCarbamidomethyl [C15				1.360714793	1.25	0	2765.31908	3	922.4442749	-0.292426641	-0.000269551	92.01085505
																			IANLQTDLSDGLR	6	160420317	1xTMT6plex [N-Term]	100	100		2.082431555	20.28	0	1644.911815	2	822.9591064	-0.534149835	-0.000439315	63.94389864
																			AEGPGLSR	2	160420317	1xTMT6plex [N-Term]			1.71	2.540458202	34.48	0	1015.573374	2	508.290741	0.818083277	0.000415412	29.65909585
																			FGGEHVPNSPFQVTALAGDQPSVQPPLR	5	160420317	1xTMT6plex [N-Term]	100			2.422567606	3.21	0	3174.653362	3	1058.88562	-3.482134009	-0.003684856	70.79174009
																			LYSVSYLLK	6	160420317	2xTMT6plex [N-Term; K9]	100		100	2.10893774	25.76	0	1543.949988	2	772.4778442	-1.020566372	-0.000787852	81.36790493
																			AEFTVETR	14	160420317	1xTMT6plex [N-Term]	1.333	1.355	1.107	3.383237123	44.12	0	1181.636369	2	591.3215942	-0.386526219	-0.000228367	39.01050375
																			VTYTPMAPGSYLISIK	3	160420317	2xTMT6plex [N-Term; K16]				1.458453298	0.09	0	2199.249934	2	1100.127319	-1.169470593	-0.001285979	79.53150219
																			KGEITGEVR	6	160420317	2xTMT6plex [N-Term; K1]	1.834	1.81	1.753	3.402941465	15.11	1	1446.868049	3	482.9606934	-0.360780567	-0.000174001	35.48496985
																			VKETADFK	12	160420317	3xTMT6plex [N-Term; K2; K8]	1.017	1.04	1.001	3.29244709	22.67	1	1624.987719	2	812.9970093	-0.601324737	-0.000488573	36.54842829
																			LKPGAPLRPK	8	160420317	3xTMT6plex [N-Term; K2; K10]	1.454	1.514	1.548	4.266438484	13.18	1	1764.182671	4	441.8014832	0.811609371	0.000357957	37.5532207
																			DGTVTVR	4	160420317	1xTMT6plex [N-Term]	1.237	1.093		2.73395133	41.99	0	976.5624755	2	488.7849121	0.073870082	3.61E-05	26.84352864
																			GAGGQGK	4	160420317	2xTMT6plex [N-Term; K7]	100	100		2.43762517	41.35	0	1032.620214	2	516.8138428	0.188565818	9.74E-05	13.39554974
																			EEPCLLK	6	160420317	2xTMT6plex [N-Term; K7]; 1xCarbamidomethyl [C4	1.363	1.566	1.534	2.606098413	27.64	0	1346.775394	2	673.8916626	0.48573453	0.000327088	52.66616706
																			WCNEHLK	4	160420317	2xTMT6plex [N-Term; K7]; 1xCarbamidomethyl [C2	1.264			2.917763948	31.53	0	1444.777126	2	722.8922729	0.099349883	7.18E-05	44.31099071
																			VTANNDK	4	160420317	2xTMT6plex [N-Term; K7]		1.989	0.412	3.057397604	27.94	0	1219.704672	2	610.3565063	0.872404064	0.000532038	25.44476861
																			VTGDDSMR	2	160420317	1xTMT6plex [N-Term]; 1xOxidation [M7]	2.273			1.55492723	25.74	0	1125.540754	2	563.2740479	0.057779449	3.25E-05	22.52771959
																			DQEFTVK	4	160420317	2xTMT6plex [N-Term; K7]	1.928	1.818		3.22877717	34.89	0	1324.751288	2	662.8771362	-3.239830074	-0.002145985	52.15020963
194595509	SPTAN1	spectrin alpha chain, non-erythrocytic 1 isoform	6709	NP_001123910.1	47.51715785	1	90	90	479	2477	284.919	5.35	1.08	1.07	1.12	1.09	Significant	0.0002																
																			SSEEIESAFR	6	194595509	1xTMT6plex [N-Term]	0.991	1.086	1.134	4.479233265	61.17	0	1383.69534	2	692.3514404	0.191045939	0.000132175	46.06145522
																			QFQDAGHFDAENIKK	4	194595509	3xTMT6plex [N-Term; K14; K15]	0.955	0.784		5.712639809	20.72	1	2435.328619	4	609.5881958	0.958500264	0.000583566	47.83769396
																			LQQSHPLSATQIQVK	10	194595509	2xTMT6plex [N-Term; K15]	1.215	1.181	1.131	5.659464359	48.66	0	2136.254108	3	712.7561646	-0.078528886	-5.59E-05	44.8812134
																			SQLLGSAHEVQR	6	194595509	1xTMT6plex [N-Term]	1.008	0.905	1	4.853790283	63.57	0	1553.85972	3	518.6242065	-1.064092899	-0.00055115	37.45976042
																			HALLEADVAAHQDR	10	194595509	1xTMT6plex [N-Term]	1.227	1.154	1.142	5.595077991	71.71	0	1774.939761	3	592.317749	-0.601225862	-0.000355713	38.02680678
																			KFEEFQTDMAAHEER	10	194595509	2xTMT6plex [N-Term; K1]	1.193	1.25	0.916	5.685240269	34.23	1	2326.153802	4	582.2928467	-1.824832583	-0.00106121	46.80869736
																			GNAMVEEGHFAAEDVK	6	194595509	2xTMT6plex [N-Term; K16]	1.633	0.9	1.174	4.96509552	56.04	0	2162.095224	3	721.3702393	0.434894554	0.000313428	44.42573063
																			GNAMVEEGHFAAEDVK	2	194595509	2xTMT6plex [N-Term; K16]; 1xOxidation [M4		1.209		4.662463665	53.55	0	2178.090139	3	721.3702393	0.434894554	0.000313428	44.42573063
																			MQHNLEQQIQAR	8	194595509	1xTMT6plex [N-Term]	0.847	0.841	0.948	4.472182751	35.28	0	1724.906352	3	575.6395874	-1.242643095	-0.000714481	43.0061338
																			LDETGNLMISEGHFASETIR	6	194595509	1xTMT6plex [N-Term]	0.01	1.355	0.01	4.109241486	33.33	0	2449.223054	3	817.0785522	-0.796477771	-0.000650251	77.22819235
																			VLETAEDIQER	4	194595509	1xTMT6plex [N-Term]	1.209	1.26		3.922664881	50.92	0	1531.816518	2	766.4118042	-0.121288392	-9.29E-05	57.79116576
																			SADESGQALLAAGHYASDEVR	6	194595509	1xTMT6plex [N-Term]	1.019	1.185		7.348431587	97.7	0	2376.162897	3	792.7259521	0.171025864	0.000135462	64.4028254
																			AQLADSFHLQQFFR	8	194595509	1xTMT6plex [N-Term]	0.766	100	100	3.662740707	41.85	0	1937.023097	3	646.3456421	-0.37358381	-0.000241213	82.35037961
																			KHEAFETDFTVHK	4	194595509	3xTMT6plex [N-Term; K1; K13]	1.235		1.247	3.771628857	20.33	1	2276.264228	4	569.8214722	-0.074331496	-4.23E-05	45.7647817
																			GEIDAHEDSFK	18	194595509	2xTMT6plex [N-Term; K11]	1.048	1.046	1.108	3.944281101	67.87	0	1705.879736	2	853.4432983	-0.243581247	-0.00020776	47.0950675
																			EKEPIVGSTDYGKDEDSAEALLK	6	194595509	4xTMT6plex [N-Term; K2; K13; K23]	0.695	0.957	1.086	4.425505161	18.02	2	3410.871247	4	853.4718018	-1.720923578	-0.001467462	64.77846227
																			DCEQAENWMAAR	4	194595509	1xTMT6plex [N-Term]; 1xCarbamidomethyl [C2	1.186	2.228		4.195970535	65.48	0	1709.757305	2	855.3832397	1.109895645	0.000948826	52.87316189
																			ALINADELASDVAGAEALLDR	5	194595509	1xTMT6plex [N-Term]	100	1.118		4.664208889	60.16	0	2356.255735	3	786.0895996	-0.631931353	-0.000496331	108.5862706
																			KHALLEADVAAHQDR	4	194595509	2xTMT6plex [N-Term; K1]		0.904	1.011	5.844764233	51.08	1	2132.197656	4	533.8048706	-0.001584339	-8.45E-07	44.54021924
																			HQLLEADISAHEDR	4	194595509	1xTMT6plex [N-Term]	1.032	1.186		3.267295837	40.97	0	1862.955805	3	621.6571045	0.512812253	0.000318449	37.93979518
																			EQADYCVSHMKPYVDGK	2	194595509	3xTMT6plex [N-Term; K11; K17]; 1xCarbamidomethyl [C6		0.729		5.259498119	40.18	0	2714.388519	4	679.3521729	-0.610507206	-0.000414288	46.92300505
																			LIQNNHYAMEDVATR	4	194595509	1xTMT6plex [N-Term]	1.258	1.178		4.593864441	40.97	0	2004.017025	3	668.6768799	-0.468315164	-0.000312837	44.58632459
																			QEAFLLNEDLGDSLDSVEALLK	8	194595509	2xTMT6plex [N-Term; K22]		100	100	2.775276899	20.74	0	2877.54974	3	959.8538208	-0.983829268	-0.000943673	108.8838515
																			QQVAPTDDETGK	2	194595509	2xTMT6plex [N-Term; K12]			1.697	4.026583672	49.51	0	1746.927414	2	873.9676514	0.350400578	0.000306062	34.39504696
																			QFQDAGHFDAENIK	3	194595509	2xTMT6plex [N-Term; K14]		1.017		4.352909088	35.2	0	2078.070724	3	693.3614502	-0.445973302	-0.000308921	56.51645937
																			KIEDLGAAMEEALILDNK	4	194595509	3xTMT6plex [N-Term; K1; K18]		100	0.465	3.14509201	17.27	1	2660.514765	3	887.5090942	-0.764974201	-0.000678408	104.098804
																			LSDDNTIGKEEIQQR	2	194595509	2xTMT6plex [N-Term; K9]		1.292		4.15318203	16.03	1	2204.192296	3	735.4014282	-1.163338623	-0.000854741	45.55308617
																			HQAFEAELSANQSR	16	194595509	1xTMT6plex [N-Term]	1.151	1.113	1.151	5.643149376	62.3	0	1816.91394	2	908.960022	-0.64547619	-0.000586387	43.50615672
																			EAIVTSEELGQDLEHVEVLQK	3	194595509	2xTMT6plex [N-Term; K21]	1.493			3.549453974	39.9	0	2824.534424	3	942.1813965	-1.695244651	-0.001596092	97.39550416
																			NVEDIELWLYEVEGHLASDDYGK	2	194595509	2xTMT6plex [N-Term; K23]				2.656141758	14.36	0	3152.582831	3	1051.532471	0.008798936	9.25E-06	111.7970208
																			HQAFEAELHANADR	8	194595509	1xTMT6plex [N-Term]		1.248	1.185	7.211834431	71.99	0	1837.914274	3	613.3059692	-5.9415204	-0.003640002	35.26717412
																			HQALQAEIAGHEPR	8	194595509	1xTMT6plex [N-Term]	1.256	1.017	1.297	6.307799339	57.37	0	1785.955745	3	595.989502	-1.003821416	-0.000597594	34.49851861
																			EKEQLMASDDFGR	6	194595509	2xTMT6plex [N-Term; K2]	0.978	1.175		4.22820425	36.34	1	1984.020997	3	662.0111084	-1.121356423	-0.000741598	53.29862176
																			GACAGSEDAVK	10	194595509	2xTMT6plex [N-Term; K11]; 1xCarbamidomethyl [C3	1.136	1.181	1.223	4.455861092	75.7	0	1522.793564	2	761.9005737	0.201813919	0.00015366	28.44634953
																			REELITNWEQIR	2	194595509	1xTMT6plex [N-Term]	1.07			4.435927868	22.88	1	1815.991462	3	606.0021973	0.317389639	0.000192126	61.87228724
																			DLTNVQNLQK	6	194595509	2xTMT6plex [N-Term; K10]	1.376		1.187	4.592870235	62.15	0	1630.952841	2	815.9743652	-6.981894855	-0.005693571	46.1471219
																			ALSSEGKPYVTK	4	194595509	3xTMT6plex [N-Term; K7; K12]	1.195	1.191		2.664470434	17.56	0	1967.178039	3	656.3973999	-0.199475066	-0.000130801	52.16106603
																			LIQEQHPEEELIK	4	194595509	2xTMT6plex [N-Term; K13]	1.081	1.24		4.401173592	35.62	0	2064.174127	3	688.7294922	-0.098444941	-6.77E-05	62.89852098
																			LEESLEYQQFVANVEEEEAWINEK	2	194595509	2xTMT6plex [N-Term; K24]				2.225087404	16.32	0	3384.688753	3	1128.900757	-0.305889925	-0.000345114	108.1798763
																			DLSSVQTLLTK	12	194595509	2xTMT6plex [N-Term; K11]	1.808		100	3.528986931	47.26	0	1663.004208	2	832.0054321	-0.372934828	-0.000310096	88.08089456
																			LIQSHPESAEDLQEK	4	194595509	2xTMT6plex [N-Term; K15]			1.153	5.524181843	53.9	0	2182.175583	3	728.0621338	-1.711531758	-0.001244954	41.36859357
																			GLVSSDELAK	8	194595509	2xTMT6plex [N-Term; K10]	0.8	0.887	0.982	4.493393421	43.6	0	1476.86738	2	738.9379883	0.893683832	0.000659926	46.15738203
																			LDENSAFLQFNWK	4	194595509	2xTMT6plex [N-Term; K13]		0.01		2.511069059	29.69	0	2070.106047	2	1035.55481	-1.789521547	-0.00185225	95.61887326
																			LIQNNHYAMEDVATR	4	194595509	1xTMT6plex [N-Term]; 1xOxidation [M9]	1.649	1.151		3.767712593	32.65	0	2020.01194	3	668.6768799	-0.468315164	-0.000312837	44.58632459
																			LQIASDENYKDPTNLQGK	4	194595509	3xTMT6plex [N-Term; K10; K18]	0.808			3.551170826	27.76	1	2721.50262	3	907.8414307	2.615724907	0.002372901	59.37811591
																			DLIGVQNLLK	3	194595509	2xTMT6plex [N-Term; K10]	1.235			3.197809219	44.63	0	1570.993249	2	786.0003052	0.053737699	4.22E-05	91.87103833

Bhat et al,2017. Cigarette smoke and chewing tobacco alter expression of different sets of miRNAs in oral keratinocytes

Supplementary Table S2(A). List of all proteins and peptides identified in OKF6/TERT1 cells chronically treated with chewing tobacco using Sequest and Mascot search algorithms

Accession	Gene_Symbol	Description	Gene_ID	Refseq_Accession	Coverage	Proteins	#Unique_Peptides	#Peptides	#PSMS	#AAs	MW [kDa]	calc. pI	OKF6-STE-6M/OKF6P_R1	OKF6-STE-6M/OKF6P_R2	OKF6-STE-6M/OKF6P_R3	Median fold change	Status	P-value (6M)	Sequence	#PSMSs	Protein Accessions	Modifications	OKF6-STE-6M/OKF6P_R1	OKF6-STE-6M/OKF6P_R2	OKF6-STE-6M/OKF6P_R3	Xcorr	Ionscore	# Missed Cleavages	Theo. MH+ [Da]	Charge	m_z	DeltaM [ppm]	Deltam/z [Da]	RT [min]
																			ITALDEFATK	6	194595509	2xTMT6plex [N-Term; K10]	1.054			3.362785816	37.67	0	1566.91433	2	783.9603882	-0.530037154	-0.000415261	84.3768477
																			DVEDEETWIR	6	194595509	1xTMT6plex [N-Term]	1.316	0.01	100	4.047188759	45.1	0	1520.743018	2	760.8737793	-1.799335073	-0.001368163	51.28201146
																			DVDEIEAWISEK	8	194595509	2xTMT6plex [N-Term; K12]	0.952	100		3.329702139	29.19	0	1892.00533	2	946.5044556	-1.953169544	-0.001847704	94.57493235
																			ADVVESWIGEK	6	194595509	2xTMT6plex [N-Term; K11]	100	100		2.831310987	33.67	0	1690.941608	2	845.973999	-0.524064888	-0.000443082	81.22061769
																			MMLDQCLELQLFHR	2	194595509	1xTMT6plex [N-Term]; 1xCarbamidomethyl [C6	1.196			4.592073917	43.38	0	2063.043774	3	688.3530273	0.365814463	0.000251564	89.70018228
																			DVTGAEALLER	6	194595509	1xTMT6plex [N-Term]	100	0.932		3.470284224	40.52	0	1402.773925	2	701.890686	0.121823132	8.54E-05	51.76248425
																			EQADYCVSHMKPYVDGK	2	194595509	3xTMT6plex [N-Term; K11; K17]; 1xCarbamidomethyl [C6]; 1xOxidation [M10]	0.802			2.130244493	1.99	0	2730.383434	4	679.3521729	-0.610507206	-0.000414288	46.92300505
																			EANELQQWINEK	6	194595509	2xTMT6plex [N-Term; K12]	100		100	2.644863367	38.9	0	1960.054011	2	980.5313721	0.742877807	0.00072804	76.92461398
																			QVEELYHSLLELGEK	9	194595509	2xTMT6plex [N-Term; K15]	100			3.208680391	11.56	0	2245.24802	3	749.0874634	-0.081400041	-6.09E-05	95.41793031
																			LSDDNTIGK	2	194595509	2xTMT6plex [N-Term; K9]	1.239			3.669698	34.72	0	1420.80478	2	710.9053345	-0.97657659	-0.000693762	47.0501351
																			EANQQQQFNR	5	194595509	1xTMT6plex [N-Term]	2.406	1.211		3.114337206	46.82	0	1491.750169	2	746.378479	-0.327147399	-0.000244011	15.03166615
																			KLLVGSEDYGR	2	194595509	2xTMT6plex [N-Term; K1]	0.703			3.684657812	17.21	1	1694.984141	3	565.6661377	-0.165944952	-9.38E-05	45.84019231
																			DLTGVQNLR	6	194595509	1xTMT6plex [N-Term]	1.167	1.095	1.175	3.450423479	46.12	0	1244.716016	2	622.8616943	0.077280178	4.81E-05	39.7576211
																			DCEQAENWMAAR	2	194595509	1xTMT6plex [N-Term]; 1xCarbamidomethyl [C2]; 1xOxidation [M9]	100			2.519875526	28.83	0	1725.75222	2	855.3832397	1.109895645	0.000948826	52.87316189
																			EELYQNLTR	4	194595509	1xTMT6plex [N-Term]		1.392	1.211	3.602936506	49.64	0	1394.74771	2	697.8783569	1.238508711	0.000863704	46.5541443
																			LFGAAEVQR	6	194595509	1xTMT6plex [N-Term]	0.962	1.234	1.569	3.320988655	46.57	0	1219.699638	2	610.3533325	-0.204272371	-0.000124575	47.20853681
																			TKQDEVNAAWQR	4	194595509	2xTMT6plex [N-Term; K2]	1.104	1.074		3.38333869	9.04	1	1904.03903	3	635.3508911	-0.477947217	-0.000303343	37.00658522
																			SLQQLAEER	4	194595509	1xTMT6plex [N-Term]	1.14	0.91		3.685990334	57.7	0	1302.721495	2	651.8638916	-0.758824414	-0.000494268	56.19518536
																			DMDDEESWIK	6	194595509	2xTMT6plex [N-Term; K10]	1.045	1.324	100	2.967088699	48.12	0	1725.840573	2	863.425354	1.656298883	0.001429254	64.05274852
																			DLAALEDK	6	194595509	2xTMT6plex [N-Term; K8]	1.214	1.166	0.414	3.764495134	48.42	0	1332.777503	2	666.892395	0.008170203	5.44E-06	59.20874726
																			LLEATELK	6	194595509	2xTMT6plex [N-Term; K8]	1.287	100	1.433	3.767199993	38.64	0	1374.860838	2	687.9336548	-0.585717234	-0.00040264	64.36331095
																			DTEQVDNWMSK	2	194595509	2xTMT6plex [N-Term; K11]		100		2.2058599	19.28	0	1810.90457	2	905.9563599	0.482088663	0.000436508	66.51130991
																			IDGITIQAR	4	194595509	1xTMT6plex [N-Term]	1.223	1.276		3.02075243	35.01	0	1215.725852	2	608.3665161	-0.079527335	-4.83E-05	60.13275889
																			SLSAQEEK	4	194595509	2xTMT6plex [N-Term; K8]		1.001	0.912	3.691731215	49.04	0	1349.767666	2	675.3872681	-0.301227535	-0.000203294	35.6714413
																			EAALTSEEVGADLEQVEVLQK	4	194595509	2xTMT6plex [N-Term; K21]				2.088361502	5.96	0	2716.465676	3	906.1628418	3.053931694	0.0027653	86.22067806
																			ALCAEADR	10	194595509	1xTMT6plex [N-Term]; 1xCarbamidomethyl [C3	1.139	0.991	1.097	3.384783745	46.51	0	1134.577474	2	567.7921753	-0.352451967	-0.000199942	28.18972399
																			AGTFQAFEQFGQQLLAHGHYASPEIK	2	194595509	2xTMT6plex [N-Term; K26]				2.700659037	4.05	0	3333.742066	4	834.190918	-0.067244255	-5.60E-05	100.7876494
																			LAQFVEHWK	10	194595509	2xTMT6plex [N-Term; K9]	1.043	0.753		2.513463259	21.25	0	1615.936069	3	539.3169556	0.151373079	8.15E-05	70.63874376
																			EAGSVSLR	4	194595509	1xTMT6plex [N-Term]		1.272	1.137	3.082070112	43.6	0	1047.599589	2	524.3036499	0.41425626	0.000216987	30.88994097
																			DADETKEWIEEK	2	194595509	3xTMT6plex [N-Term; K6; K12]	0.666			3.248402119	0.25	1	2180.16899	3	727.3944092	-0.144879107	-0.000105287	70.43220868
																			AINVQEEK	6	194595509	2xTMT6plex [N-Term; K8]	0.965	0.93	0.97	4.04638052	49.51	0	1388.814951	2	694.9065552	-6.564530741	-0.004558459	38.39428809
																			LLEAQSHFR	4	194595509	1xTMT6plex [N-Term]	0.881	1.308		3.892683744	54.64	0	1329.74765	3	443.9209595	0.507478219	0.000224939	40.98503129
																			ECEDVMDWINDK	2	194595509	2xTMT6plex [N-Term; K12]; 1xCarbamidomethyl [C2				1.665743351	8.47	0	2011.950534	2	1006.477966	-0.933538266	-0.000939116	75.98953446
																			QETFDAGLQAFQQEGIANITALK	2	194595509	2xTMT6plex [N-Term; K23]				2.211752892	12.87	0	2951.587857	3	984.5357056	1.594538075	0.001568806	95.78940104
																			ASAFNSWFENAEEDLTDPVR	3	194595509	1xTMT6plex [N-Term]				1.646654129	2.45	0	2527.193863	2	1264.099854	-0.566695247	-0.000716074	97.71023114
																			MTLVASEDYGDTLAAIQGLLK	2	194595509	2xTMT6plex [N-Term; K21]				1.661695957	8.62	0	2667.467925	3	889.8275146	0.024639589	2.19E-05	115.4476552
																			LDILDQER	11	194595509	1xTMT6plex [N-Term]	1.906	100	1.461	2.924754858	24.57	0	1230.689132	2	615.8485107	0.497659692	0.000306232	52.78428491
																			DLASVQALLR	4	194595509	1xTMT6plex [N-Term]	0.169	1.202		1.772123456	20.32	0	1314.794266	2	657.9005127	-0.393513562	-0.000258695	79.06936169
																			DLASVNNLLK	4	194595509	2xTMT6plex [N-Term; K10]	100	100		2.22147584	13.81	0	1544.941214	2	772.9732056	-1.34582285	-0.001039609	72.33556183
																			DVDETISWIK	6	194595509	2xTMT6plex [N-Term; K10]				2.018847704	16.45	0	1663.930709	2	832.4667358	-2.712553046	-0.00225675	70.32220034
																			AALLELWELR	4	194595509	1xTMT6plex [N-Term]				1.495507121	9.49	0	1442.856866	2	721.9321899	0.164162377	0.000118431	94.38647698
																			QWELLLEK	2	194595509	2xTMT6plex [N-Term; K8]				1.942871332	18.85	0	1516.913936	2	758.9598389	-1.012117819	-0.000767648	80.16194695
																			KLDPAQSASR	2	194595509	2xTMT6plex [N-Term; K1]		0.717		2.815136194	2	1	1530.900412	3	510.9721985	1.065163333	0.000543553	29.5132725
																			EFSMMFK	2	194595509	2xTMT6plex [N-Term; K7]				2.394260645	24.66	0	1377.731086	2	689.3702393	1.535419826	0.001057698	75.32551083
																			VAEDLESEGLMAEEVQAVQQQEVYGMMPR	1	194595509	1xTMT6plex [N-Term]				1.310765505		0	3495.664321	3	1165.890503	-2.107124787	-0.002455267	100.2972582
																			ELPTAFDYVEFTR	9	194595509	1xTMT6plex [N-Term]	100	0.976	1.049	2.357008934	19.27	0	1816.931882	2	908.9675293	-2.256356604	-0.002049823	79.08450196
																			EAFLNTEDKGDSLDSVEALIK	2	194595509	3xTMT6plex [N-Term; K9; K21]		0.01		1.529174328		1	2981.628608	3	994.5464478	-1.280584802	-0.001272743	81.99723421
																			VASNPYTWFTMEALEETWR	1	194595509	1xTMT6plex [N-Term]				0.97406584		0	2560.237976	2	1280.619141	-2.722965625	-0.00348572	104.1570257
41872631	FASN	fatty acid synthase	2194	NP_004095.4	47.82954998	1	83	84	567	2511	273.254	6.44	2.28	2.27	2.54	2.36	Significant	0.0014																
																			GTHTGVWVGVSGSETSEALSR	10	41872631	1xTMT6plex [N-Term]	2.743	2.057	2.508	7.378953934	57.31	0	2346.188718	3	782.7357178	1.654768981	0.001294133	45.37426865
																			GYDYGPHFQGILEASLEGDSGR	13	41872631	1xTMT6plex [N-Term]	10.942	100	100	3.448961258	30.7	0	2597.246961	3	866.4217529	1.441834034	0.001248266	78.96846336
																			GVDLVLNSLAEEK	12	41872631	2xTMT6plex [N-Term; K13]	10.759	1.886	100	3.253736973	55.54	0	1845.07335	2	923.0410156	0.761324746	0.00070235	75.40522858
																			CTVFHGAQVEDAFR	8	41872631	1xTMT6plex [N-Term]; 1xCarbamidomethyl [C1	1.975	2.394	2.218	5.10954237	46.86	0	1865.916583	3	622.6430054	-1.136244105	-0.000706712	58.89324606
																			GILADEDSSRPVWLK	8	41872631	2xTMT6plex [N-Term; K15]	100	1.678	1.742	4.06563139	35.81	0	2144.211575	3	715.4088135	0.145759724	0.00010418	68.28841952
																			LPEDPLLSGLLDSPALK	9	41872631	2xTMT6plex [N-Term; K17]	2.187	3.216		3.768598795	65.92	0	2236.320456	3	746.1109619	-0.94969744	-0.000707943	106.4132451
																			VLLSLEHGLWAPNLHFHSPNPEIPALLDGR	7	41872631	1xTMT6plex [N-Term]	100	100		3.700218916	25.29	0	3571.937522	5	715.1937866	0.6451052	0.000460855	92.20235517
																			ACLDTAVENMPSLK	2	41872631	2xTMT6plex [N-Term; K14]; 1xCarbamidomethyl [C2	3.811			3.710643053	57.16	0	2007.065504	2	1004.036194	-0.195979995	-0.000196672	76.84690145
																			VTVAGGVHISGLHTESAPR	10	41872631	1xTMT6plex [N-Term]	100	2.156	2.266	6.581652641	47.42	0	2117.166466	4	530.0439453	-5.911150681	-0.003128722	41.70213102
																			EDGLAQQQTQLNLR	6	41872631	1xTMT6plex [N-Term]	2.491	3.837	3.068	4.336976528	54.18	0	1842.987105	2	921.9975586	0.399117009	0.000367784	50.60821908
																			LPESENLQEFWDNLIGGVDMVTDDDRR	4	41872631	1xTMT6plex [N-Term]	100	2.597		5.429121494	27.4	1	3392.626614	3	1131.5448	-1.994851396	-0.002255929	116.600665
																			ALGLGVEQLPVVFEDVVLHQATILPK	10	41872631	2xTMT6plex [N-Term; K26]	2.594	2.344		5.081834793	70.31	0	3243.912092	3	1081.976929	1.276623008	0.001380418	116.1951962
																			FDLSQNHPLGMAIFLK	11	41872631	2xTMT6plex [N-Term; K16]	2.648	100		3.895944834	38.44	0	2289.282965	3	763.7658691	0.038746575	2.96E-05	92.45314599
																			SNMGHPEPASGLAALAK	2	41872631	2xTMT6plex [N-Term; K17]; 1xOxidation [M3		1.584		5.080831528	50.68	0	2125.147595	3	703.7224121	0.001653804	1.16E-06	59.13570293
																			SNMGHPEPASGLAALAK	4	41872631	2xTMT6plex [N-Term; K17]	2.02	2.135		5.458007813	51.94	0	2109.15268	3	703.7224121	0.001653804	1.16E-06	59.13570293
																			ADEASELACPTPK	6	41872631	2xTMT6plex [N-Term; K13]; 1xCarbamidomethyl [C9	2.096	1.341	2.363	3.356096983	64.63	0	1846.962085	2	923.9850464	0.395738193	0.000365457	41.17275609
																			AAPLDSIHSLAAYYIDCIR	15	41872631	1xTMT6plex [N-Term]; 1xCarbamidomethyl [C17	2.14	2.373	4.726	4.615446091	36.9	0	2378.237583	3	793.4169922	-0.487422917	-0.000386403	102.9180892
																			LFDHPESPTPNPTEPLFLAQAEVYK	8	41872631	2xTMT6plex [N-Term; K25]	3.692	100		4.174006462	27.93	0	3298.74	3	1100.249756	-1.602469996	-0.001762044	88.81694271
																			LHLSGIDANPNALFPPVEFPAPR	6	41872631	1xTMT6plex [N-Term]	2.337	100		5.608689308	55.43	0	2701.466336	3	901.1588135	-1.646987139	-0.001483093	91.61282349
																			EAHLPPGAMAAVGLSWEECK	17	41872631	2xTMT6plex [N-Term; K20]; 1xCarbamidomethyl [C19	2.061	3.405	1.024	4.059102535	26	0	2611.341285	3	876.4490356	-1.387875829	-0.001215472	66.67643723
																			HFLLEEDKPEEPTAHAFVSTLTR	4	41872631	2xTMT6plex [N-Term; K8]		3.345		3.099200249	7.74	0	3125.66717	4	782.1709595	-1.651461622	-0.00129048	62.96142462
																			VVEVLAGHGHLYSR	6	41872631	1xTMT6plex [N-Term]	2.402	2.403		5.644134521	74.89	0	1765.991068	2	883.4986572	-0.58351194	-0.000515238	49.54881124
																			TLLEGSGLESIISIIHSSLAEPR	4	41872631	1xTMT6plex [N-Term]			100	3.535446167	26.88	0	2651.481712	3	884.49823	-0.593988104	-0.000524983	108.9129556
																			SLYQSAGVAPESFEYIEAHGTGTK	4	41872631	2xTMT6plex [N-Term; K24]	5.264			5.078361511	61.77	0	3000.535487	3	1000.848999	-1.014238859	-0.00101442	82.01238497
																			GNAGQSNYGFANSAMER	6	41872631	1xTMT6plex [N-Term]	2.988	1.443	2.749	3.689843416	57.95	0	2002.923853	2	1001.96405	-1.512196312	-0.001514407	39.92181868
																			LQVVDQPLPVR	8	41872631	1xTMT6plex [N-Term]	1.881	2.701		4.093883038	49.33	0	1492.904879	2	746.9558716	-0.27639801	-0.000206318	66.89484185
																			SDEAVKPFGLK	6	41872631	3xTMT6plex [N-Term; K6; K11]	2.374	1.875	100	2.98916626	30.86	0	1878.13036	3	626.7148438	-0.203633362	-0.000127483	48.63896314
																			QEPLLIGSTK	4	41872631	2xTMT6plex [N-Term; K10]	2.432	2.611		3.472066164	51.11	0	1543.945965	2	772.4767456	0.161813265	0.000124915	63.19677308
																			AALQEELQLCK	10	41872631	2xTMT6plex [N-Term; K11]; 1xCarbamidomethyl [C10	3.26	3.315	2.021	3.62536335	47.9	0	1760.998077	2	881.0018921	-0.891165262	-0.00078467	57.46052534
																			VSVHVIEGDHR	8	41872631	1xTMT6plex [N-Term]	1.579	2.357	2.055	3.099245548	40.14	0	1476.812041	2	738.9098511	0.260092975	0.000192054	36.41262295
																			AQVADVVVSR	12	41872631	1xTMT6plex [N-Term]	2.133	2.661	2.319	3.563922644	59.06	0	1272.747316	2	636.8776245	0.51575315	0.000328212	43.81991863
																			EAHLPPGAMAAVGLSWEECK	4	41872631	2xTMT6plex [N-Term; K20]; 1xCarbamidomethyl [C19]; 1xOxidation [M9]	100			3.560189962	26.96	0	2627.3362	3	876.4490356	-1.387875829	-0.001215472	66.67643723
																			VFTTVGSAEK	2	41872631	2xTMT6plex [N-Term; K10]		3.319		4.617109776	54.55	0	1496.872465	2	748.9388428	-1.373906711	-0.001028282	51.70542974
																			NVTFHGVLLDAFFNESSADWR	4	41872631	1xTMT6plex [N-Term]	100	1.821		3.770738602	6.95	0	2654.320066	3	885.4421997	-3.021594497	-0.002673426	102.8327581
																			VVVQVLAEEPEAVLK	16	41872631	2xTMT6plex [N-Term; K15]	3.083	6.198	2.713	2.613292933	32	0	2081.262213	2	1041.134644	-0.097421951	-0.00010138	89.60663464
																			VAAAVDLIIK	10	41872631	2xTMT6plex [N-Term; K10]	2.966		100	3.621710777	50.01	0	1470.965972	2	735.9874268	1.091055576	0.000802453	84.78962934
																			VLQGDLVMNVYR	2	41872631	1xTMT6plex [N-Term]			2.11	3.274011374	34.07	0	1635.908978	2	818.4578247	-0.370091457	-0.000302718	76.42861539
																			RPTPQDSPIFLPVDDTSFR	12	41872631	1xTMT6plex [N-Term]	100	2.968	100	4.092456818	21.26	0	2417.26624	3	806.4279175	1.224296835	0.000986484	56.98018211
																			YHGNVMLLR	6	41872631	1xTMT6plex [N-Term]	2.283	2.529		3.672129393	47.92	0	1331.745542	2	666.3766479	0.35845319	0.000238684	48.47186713
																			WLSTSIPEAQWHSSLAR	4	41872631	1xTMT6plex [N-Term]		100		3.032499313	24.59	0	2198.155567	3	733.3901978	0.215081101	0.000157594	78.95706173
																			GLKPSCTIIPLMK	12	41872631	3xTMT6plex [N-Term; K3; K13]; 1xCarbamidomethyl [C6	2.178	7.333	1.575	3.037122011	19.94	0	2145.31065	3	715.7739258	-1.596941799	-0.001141979	61.67089512
																			DTVTISGPQAPVFEFVEQLR	4	41872631	1xTMT6plex [N-Term]				2.456636906	17.73	0	2462.312856	3	821.4428711	0.489166617	0.000401494	97.87245776
																			DNLEFFLAGIGR	6	41872631	1xTMT6plex [N-Term]	100	100	0.832	3.346801043	37.07	0	1580.863408	2	790.9345093	-1.054060271	-0.000833163	90.19178441
																			GLVQALQTK	10	41872631	2xTMT6plex [N-Term; K9]	2.637	2.373	100	3.709764004	54.91	0	1415.898621	2	708.4525146	-0.613068699	-0.000434022	64.7141764
																			QQEQQVPILEK	2	41872631	2xTMT6plex [N-Term; K11]			100	2.831495762	28.54	0	1798.04747	2	899.5276489	0.306805895	0.000275826	56.35352781
																			SFYGSTLFLCR	6	41872631	1xTMT6plex [N-Term]; 1xCarbamidomethyl [C10	100	100	2.06	3.792754173	30.39	0	1579.814016	2	790.4102783	-0.465598714	-0.00036778	80.90110234
																			SEGVVAVLLTK	8	41872631	2xTMT6plex [N-Term; K11]	2.055		100	2.964411259	34.27	0	1573.992915	2	787.5010376	1.196724137	0.000941818	80.76131021
																			YSGTLNLDR	8	41872631	1xTMT6plex [N-Term]	1.659	2.558	2.987	3.840139627	65.43	0	1267.684381	2	634.3421021	-5.879924488	-0.003726944	42.05101922
																			GTPLISPLIK	14	41872631	2xTMT6plex [N-Term; K10]	1.349	1.519	2.628	3.204901218	28.41	0	1496.981622	2	748.9937744	-0.901709049	-0.000674921	62.56897054
																			GYAVLGGER	6	41872631	1xTMT6plex [N-Term]	0.266	2.062	1.956	3.419159651	49.24	0	1150.641788	2	575.8250732	0.939914043	0.000540752	38.36470454
																			TGTVSLEVR	4	41872631	1xTMT6plex [N-Term]		0.599	3.406	3.577936411	55.31	0	1190.694218	2	595.8500366	-1.193570768	-0.000710589	38.7101083
																			HSQDLAFLSMLNDIAAVPATAMPFR	2	41872631	1xTMT6plex [N-Term]			100	2.541079521	9.51	0	2945.521485	3	982.5060425	-6.080603617	-0.005970183	105.84257
																			CVLLSNLSSTSHVPEVDPGSAELQK	2	41872631	2xTMT6plex [N-Term; K25]; 1xCarbamidomethyl [C1				2.030425072	4.98	0	3125.655285	3	1042.556152	-0.441852547	-0.00046036	65.50825784
																			FDASFFGVHPK	11	41872631	2xTMT6plex [N-Term; K11]	1.927	2.834	3.443	3.146132708	30.66	0	1709.941548	3	570.6517944	-0.419926178	-0.00023935	63.7746256
																			LSPDAIPGK	8	41872631	2xTMT6plex [N-Term; K9]	2.645	2.644	2.794	3.403247118	37.21	0	1355.829872	2	678.4177246	-1.253757042	-0.000849941	44.23621357
																			EQGVTFPSGDIQEQLIR	5	41872631	1xTMT6plex [N-Term]		100		2.0213871	19.44	0	2146.134163	2	1073.56958	-1.062139445	-0.001139747	62.72936596
																			WTSQDSLLGMEFSGR	4	41872631	1xTMT6plex [N-Term]	100			2.914969683	23.05	0	1942.953028	2	971.9786377	-1.558930829	-0.001514465	81.68904958
																			LPESENLQEFWDNLIGGVDMVTDDDRR	6	41872631	1xTMT6plex [N-Term]; 1xOxidation [M20]	100	100		3.2235291	9.55	1	3408.621529	3	1131.5448	-1.994851396	-0.002255929	116.600665
																			DIMLATGK	10	41872631	2xTMT6plex [N-Term; K8]	2.902	2.865	3.786	3.731718302	38.55	0	1306.780479	2	653.8938599	-0.027842042	-1.82E-05	48.98874837
																			VLEALLPLK	8	41872631	2xTMT6plex [N-Term; K9]	100	100	100	2.536859989	34.84	0	1453.975808	2	727.491394	-0.204235903	-0.000148477	102.7676427
																			DPSQQELPR	4	41872631	1xTMT6plex [N-Term]		2.659	1.415	3.258650303	31.72	0	1298.690195	2	649.8484497	-0.440602339	-0.000286103	29.57782346
																			LGMLSPEGTCK	4	41872631	2xTMT6plex [N-Term; K11]; 1xCarbamidomethyl [C10	1.543		100	2.29329443	26.49	0	1650.89592	2	825.9515381	-0.073019821	-6.03E-05	59.69365493
																			DGLLENQTPEFFQDVCKPK	3	41872631	3xTMT6plex [N-Term; K17; K19]; 1xCarbamidomethyl [C16				1.521707416	10.36	0	2952.574405	3	984.8599854	-3.048944439	-0.003000745	70.58174665
																			VYQWDDPDPR	6	41872631	1xTMT6plex [N-Term]	2.628	2.754		2.92827487	52.52	0	1519.737873	2	760.3709717	-2.110015603	-0.001603335	51.7801198
																			GMGLSLMR	4	41872631	1xTMT6plex [N-Term]		1.795	2.206	2.573654413	37.91	0	1093.605937	2	547.3071289	0.954825193	0.000522101	60.4517128
																			EACPELDYFVVFSSVSCGR	2	41872631	1xTMT6plex [N-Term]; 2xCarbamidomethyl [C3; C17			100	2.689502954	12.34	0	2451.152198	2	1226.075195	-3.706083615	-0.004542088	96.88050755
																			AGLYGLPR	6	41872631	1xTMT6plex [N-Term]	3.771	2.909	2.904	2.745118856	45.13	0	1075.646146	2	538.326416	-0.548617919	-0.000295059	40.90169676
																			SLLVNPEGPTLMR	12	41872631	1xTMT6plex [N-Term]	100	100	100	2.409017324	25.24	0	1655.935193	2	828.4697876	-1.747879204	-0.001447187	59.35932287
																			LTPGCEAEAETEAICFFVQQFTDMEHNR	4	41872631	1xTMT6plex [N-Term]; 2xCarbamidomethyl [C5; C15	100	100		2.119988918	12.55	0	3559.612955	3	1187.205933	-2.727970305	-0.003236839	110.2925895
																			EGGFLLLHTLLR	2	41872631	1xTMT6plex [N-Term]		0.738		2.240480185	6.92	0	1597.962728	3	533.3253784	-0.717404776	-0.000382129	95.71349776
																			VYATILNAGTNTDGFK	3	41872631	2xTMT6plex [N-Term; K16]				1.327311993	10.75	0	2143.17994	2	1072.093506	-0.095676171	-0.000102526	70.71773227
																			KLQELSSK	4	41872631	3xTMT6plex [N-Term; K1; K8]	2.017			3.417904139	27.62	1	1620.029918	2	810.5200195	1.755654305	0.001422106	42.93403196
																			LSFFFDFR	6	41872631	1xTMT6plex [N-Term]	100	0.844	100	2.67866683	31.45	0	1307.698575	2	654.3529053	-0.031385769	-2.05E-05	107.2501362
																			QELSFAAR	6	41872631	1xTMT6plex [N-Term]	1.083	1.705	3.122	2.838688135	36.66	0	1150.641788	2	575.8237305	-1.394041583	-0.000802021	40.2319946
																			IPGLLSPHPLLQLSYTATDR	6	41872631	1xTMT6plex [N-Term]	100	100		2.013717651	10.82	0	2421.370311	3	807.7927246	-2.763031266	-0.002230107	87.01224217
																			MVVPGLDGAQIPR	8	41872631	1xTMT6plex [N-Term]	100	1.211	100	1.857677341	10.26	0	1581.898414	2	791.4522705	-0.726452701	-0.000574587	61.17557174
																			LQELSSK	8	41872631	2xTMT6plex [N-Term; K7]	2.834	3.649	3.455	3.084961176	34.79	0	1262.772023	2	631.8892822	-0.582398771	-0.000367718	38.35046981
																			DGAWGAFR	2	41872631	1xTMT6plex [N-Term]	100			2.424227715	27.85	0	1108.573709	2	554.7908936	0.723117794	0.000400815	58.38894755
																			SLLVNPEGPTLMR	2	41872631	1xTMT6plex [N-Term]; 1xOxidation [M12]				1.136391521	4.09	0	1671.930108	2	828.4697876	-1.747879204	-0.001447187	59.35932287
																			VMGLVPAK	6	41872631	2xTMT6plex [N-Term; K8]		0.804	2.715	2.642870426	31.31	0	1272.811386	2	636.909668	0.529196633	0.000336784	63.00339546
																			AAEQYTPK	2	41872631	2xTMT6plex [N-Term; K8]	0.98			2.831784964	23.54	0	1365.777837	2	683.3931885	0.92506489	0.000631717	34.974136
																			GMGLSLMR	2	41872631	1xTMT6plex [N-Term]; 1xOxidation [M7]	1.517			1.735890627	23.83	0	1109.600852	2	547.3071289	0.954825193	0.000522101	60.4517128
																			LMSAISK	4	41872631	2xTMT6plex [N-Term; K7]	2.479	2.656		3.126069546	39.47	0	1207.748451	2	604.37677	-1.811404465	-0.00109386	62.92296451
																			LYTLQDK	6	41872631	2xTMT6plex [N-Term; K7]	2.993	3.085	100	3.412623167	38.67	0	1338.803323	2	669.9047852	-0.769117825	-0.000514849	59.83758132
																			TPEAVQK	2	41872631	2xTMT6plex [N-Term; K7]	2.189			2.761752605	30.95	0	1230.745809	2	615.8764648	-0.126331935	-7.77E-05	34.27544059
																			LSIPTYGLQCTR	5	41872631	1xTMT6plex [N-Term]; 1xCarbamidomethyl [C10		1.806	2.966	1.2761904	9.58	0	1637.888243	2	819.4453735	-2.913892146	-0.002386315	59.99728949
																			VLFPATGYLSIVWK	3	41872631	2xTMT6plex [N-Term; K14]			100	1.113925219		0	2052.229791	2	1026.61792	-0.598284001	-0.000613908	102.2142304
																			GGPEVQQVPAGERPLWFICSGMGTQWR	2	41872631	1xTMT6plex [N-Term]; 1xCarbamidomethyl [C19]; 1xOxidation [M22]				1.487273455	3.75	0	3288.624387	3	1096.878662	-0.898297138	-0.000984721	83.68468428
																			VLQGDLVMNVYR	1	41872631	1xTMT6plex [N-Term]; 1xOxidation [M8]			100	0.954559565		0	1651.903893	2	818.4578247	-0.370091457	-0.000302718	76.42861539
																			LLEQGLR	12	41872631	1xTMT6plex [N-Term]	2.125	1.137	100	2.596036911	22.8	0	1057.65671	2	529.331604	-0.73634685	-0.000389401	56.30719544
33350932	DYNC1H1	cytoplasmic dynein 1 heavy chain 1	1778	NP_001367.2	23.87171761	1	101	101	426	4646	532.072	6.4	1.29	1.21	1.37	1.29	Significant	0.0013																
																			GKEDEGEEAASPMLQIQR	6	33350932	2xTMT6plex [N-Term; K2]	1.243	1.259	1.277	5.138544559	39.6	1	2446.264809	3	816.0932007	0.098207185	8.01E-05	47.07257019
																			FTQDTQPHYIYSPR	6	33350932	1xTMT6plex [N-Term]	1.232	0.756	1.434	4.460734367	31.03	0	1981.996941	3	661.3375244	0.544179542	0.000359521	46.6232928
																			DYIPVDQEELR	8	33350932	1xTMT6plex [N-Term]	1.287	1.249	1.359	4.140300751	67.41	0	1605.832168	2	803.4199829	0.324797524	0.000260785	53.04375861
																			VMSQEIQEQLHK	4	33350932	2xTMT6plex [N-Term; K12]	1.591	0.976		3.856166601	48.99	0	1928.067553	3	643.3609619	0.404255643	0.000259811	52.51039503
																			FYFVGDEDLLEIIGNSK	4	33350932	2xTMT6plex [N-Term; K17]	1.717			4.206250668	59.76	0	2417.300449	3	806.4381104	-0.277700404	-0.000223762	107.1326312
																			AVDDLNLHSYSNLPIWVNK	6	33350932	2xTMT6plex [N-Term; K19]	100	100	1.396	3.376012564	30.27	0	2656.449907	3	886.1534424	-1.555949371	-0.001377767	80.21532146
																			LCDEQLSSQSHYDFGLR	4	33350932	1xTMT6plex [N-Term]; 1xCarbamidomethyl [C2	1.958			4.497989178	41.27	0	2284.086561	3	762.0339355	0.303057965	0.000230737	88.2554693
																			GNEIVLSAGSTPR	4	33350932	1xTMT6plex [N-Term]	1.786	1.396		4.428119659	62.29	0	1529.848486	2	765.427002	-1.149881028	-0.000879572	48.43721958
																			VAEVLFDAADANAIEEVNLAYENVK	8	33350932	2xTMT6plex [N-Term; K25]		100	100	2.948996067	12.06	0	3165.67198	3	1055.894531	-0.928696978	-0.000979983	117.1931734
																			LVPLLLEDGGEAPAALEAALEEK	4	33350932	2xTMT6plex [N-Term; K23]	1.54	1.2		3.366679192	35.74	0	2806.585397	3	936.1990356	-1.013251337	-0.000947925	112.4636439
																			RSELEEQQMHLNVGLR	4	33350932	1xTMT6plex [N-Term]	1.108	1.188		6.078719616	48.96	1	2168.144351	4	542.7915039	-0.076030454	-4.12E-05	43.79973209
																			SVLVSAGNVK	6	33350932	2xTMT6plex [N-Term; K10]	1.303	1.132		4.429641724	55.08	0	1431.893535	2	716.4498901	-0.72049111	-0.000515833	48.75752041
																			GTVGEPTYDAEFQHFLR	5	33350932	1xTMT6plex [N-Term]	1.635	0.567	2.506	3.418450117	37.59	0	2196.092298	3	732.7051392	3.900555763	0.002855327	78.82512779
																			SIPLDEGEDEAQR	6	33350932	1xTMT6plex [N-Term]	1.387	1.623	1.357	4.278588295	60.53	0	1687.833624	2	844.4205322	0.097013783	8.19E-05	47.30063657
																			KLEHLITELVHQR	4	33350932	2xTMT6plex [N-Term; K1]	100	2.608		4.276586056	27.95	1	2074.253714	4	519.3178101	-2.074936926	-0.001075986	71.68411288
																			EVWNTYELDLVNYQNK	5	33350932	2xTMT6plex [N-Term; K16]	2.378			4.577821255	29.43	0	2486.296761	3	829.4371948	0.108821546	9.02E-05	87.30491313
																			YQVGVHYELTEEEK	4	33350932	2xTMT6plex [N-Term; K14]	0.01			3.485502005	27.63	0	2182.14322	3	728.0525513	-0.054854972	-3.99E-05	57.13048126
																			SLETCMYDHK	4	33350932	2xTMT6plex [N-Term; K10]; 1xCarbamidomethyl [C5	1.601	0.736		4.194777012	48.43	0	1741.865348	3	581.2930908	-0.361106594	-0.000209666	49.06747638
																			VPQIEVETHK	6	33350932	2xTMT6plex [N-Term; K10]	1.337	1.24	1.185	4.183855534	54.08	0	1637.962677	3	546.6591797	0.1882781	0.000102798	46.56853352
																			EFGPVVIDYGK	5	33350932	2xTMT6plex [N-Term; K11]	3.071	2.225		3.792466879	51.28	0	1681.956529	2	841.4815674	-0.399103284	-0.000335637	77.08906633
																			QWIVFDGDVDPEWVENLNSVLDDNK	2	33350932	2xTMT6plex [N-Term; K25]			1.164	2.59894824	17.06	0	3404.705071	3	1135.57251	-0.615428673	-0.000698451	107.1731798
																			IQFVGACNPPTDPGR	2	33350932	1xTMT6plex [N-Term]; 1xCarbamidomethyl [C7	1.733			3.35246563	24.9	0	1857.947883	2	929.4783325	0.81019445	0.00075265	60.5737303
																			TEYLSNADER	2	33350932	1xTMT6plex [N-Term]	0.896			3.798339844	33	0	1426.701154	2	713.8543091	0.131789381	9.40E-05	36.95789479
																			YPLIIDPSGQATEFIMNEYK	2	33350932	2xTMT6plex [N-Term; K20]			100	2.572660685	12.12	0	2787.467925	3	929.8255615	-2.078463266	-0.001931217	98.18413616
																			FLSDPQVHTVLVER	2	33350932	1xTMT6plex [N-Term]	0.943			2.033789158	0.48	0	1869.043163	3	623.6856079	-0.477730824	-0.000297633	61.59752444
																			EQPWVSVQPR	6	33350932	1xTMT6plex [N-Term]	1.3	1.31	1.327	3.369035721	34.59	0	1454.795329	2	727.9002686	-1.421691824	-0.001034135	44.52639709
																			VQVALEELQDLK	3	33350932	2xTMT6plex [N-Term; K12]	2.331	0.307		2.359211922	33.89	0	1843.094085	2	922.0501709	-0.553532852	-0.000510107	96.84555333
																			ALPDMEVVGLNFSSATTPELLLK	7	33350932	2xTMT6plex [N-Term; K23]	1.145			2.898036003	17.12	0	2903.620403	3	968.5443115	-0.696182492	-0.000673817	101.560411
																			GTFDNAETK	6	33350932	2xTMT6plex [N-Term; K9]	1.421	1.399	1.495	3.597705126	57.32	0	1440.77348	2	720.8901367	-0.335175867	-0.000241456	41.87788156
																			SACDTVDTWLDDTAK	6	33350932	2xTMT6plex [N-Term; K15]; 1xCarbamidomethyl [C3		100	100	2.207837105	19.85	0	2156.05817	2	1078.531372	-1.253569784	-0.001351385	68.63614214
																			VQYPQSQACK	4	33350932	2xTMT6plex [N-Term; K10]; 1xCarbamidomethyl [C9	1.669	1.743		3.558648348	47.56	0	1666.898697	2	833.9525146	-0.566682982	-0.000472302	42.15442073
																			TPVIDADKPVSSQLR	5	33350932	2xTMT6plex [N-Term; K8]	1.285	0.962		3.828254223	22.68	0	2084.211575	3	695.4085693	-0.201458524	-0.000139961	52.52446094
																			SLLQALNEVK	4	33350932	2xTMT6plex [N-Term; K10]	0.811	0.531		2.834892511	36.84	0	1572.972514	2	786.9893188	-0.732863847	-0.000576387	83.764417
																			GWENHVEGQK	8	33350932	2xTMT6plex [N-Term; K10]	1.319	1.29	1.179	3.267412663	49.98	0	1641.874925	2	821.4420166	1.115507093	0.000915762	36.38419259
																			GIFEALRPLETLPVEGLIR	7	33350932	1xTMT6plex [N-Term]				2.875897169	12.13	0	2352.385233	3	784.8001099	0.231088785	0.000181203	99.4689696
																			LEGVEGVAHIIDPK	6	33350932	2xTMT6plex [N-Term; K14]	1.974	1.844		2.171493053	20.55	0	1935.131534	3	645.7147217	-0.99306993	-0.000640574	66.33484344
																			ILDDDTIITTLENLK	4	33350932	2xTMT6plex [N-Term; K15]				2.043450832	20.72	0	2175.252436	2	1088.12793	-1.771543815	-0.001926777	105.2496685
																			ASVVTLPVYLNFTR	8	33350932	1xTMT6plex [N-Term]	100	100		2.295548439	23.45	0	1809.047186	2	905.026001	-1.360299993	-0.001230423	94.91160938
																			IFVFEPPPGVK	4	33350932	2xTMT6plex [N-Term; K11]	1.419	100		2.579950094	23.15	0	1688.018736	2	844.5110474	-2.320882674	-0.001958847	84.92588782
																			ADLAAVEAK	2	33350932	2xTMT6plex [N-Term; K9]			1.312	3.900214195	55.81	0	1345.809137	2	673.4042969	-5.810549048	-0.003909945	45.15647343
																			YTGEDFDEDLR	4	33350932	1xTMT6plex [N-Term]			2.336	3.562909603	39.04	0	1588.732847	2	794.8658447	-5.30905299	-0.004217333	49.60588579
																			IQGLTVEQAEAVVR	4	33350932	1xTMT6plex [N-Term]		100	100	1.995668173	35.32	0	1742.000964	2	871.5042114	0.104478451	9.10E-05	70.86999722
																			VKDDIESLHDK	4	33350932	3xTMT6plex [N-Term; K2; K11]	1.711	1.836		3.925059795	13.16	1	1986.147467	4	497.2922058	-0.238455513	-0.000118402	43.83917736
																			LALESICLLLGESTTDWK	8	33350932	2xTMT6plex [N-Term; K18]; 1xCarbamidomethyl [C7	100			2.757678509	15.39	0	2507.383133	3	836.4650879	-0.966225741	-0.000807566	116.6757563
																			ANEVEQMIR	4	33350932	1xTMT6plex [N-Term]	1.483	1.587		3.181819916	41.5	0	1318.698651	2	659.8532104	0.373897734	0.000246529	47.68225656
																			NVHLAPGWLMQLEK	2	33350932	2xTMT6plex [N-Term; K14]				2.557869673	15.08	0	2094.193422	3	698.7362061	0.306864713	0.000214211	92.44296709
																			YATLATVSR	2	33350932	1xTMT6plex [N-Term]	1.575			3.459776402	38.16	0	1210.699303	2	605.8531494	-0.232090556	-0.000140496	44.35533515
																			GTFDNAETKK	2	33350932	3xTMT6plex [N-Term; K9; K10]	1.433			2.580370188	15.31	1	1798.031375	3	600.0147095	-1.000829049	-0.000599841	42.28717148
																			ALGEYLER	6	33350932	1xTMT6plex [N-Term]	1.643	1.876	1.789	3.137235641	49.4	0	1179.657104	2	590.3324585	0.454642443	0.000268161	55.51876605
																			TLINELVK	12	33350932	2xTMT6plex [N-Term; K8]	1.44	1.432		3.303239107	42.82	0	1387.892473	2	694.4492188	-0.945202907	-0.00065592	73.54508912
																			TSFLDDAFR	4	33350932	1xTMT6plex [N-Term]	1.286	100		3.118310213	50.26	0	1300.673482	2	650.8397217	-1.011476472	-0.0006578	72.92773961
																			ENFIPTIVNFSAEEISDAIR	4	33350932	1xTMT6plex [N-Term]		100	100	1.790026307	18.89	0	2494.302685	2	1247.650391	-3.68043544	-0.00459006	114.1724509
																			KLVPLLLEDGGEAPAALEAALEEK	5	33350932	3xTMT6plex [N-Term; K1; K24]		100		3.13972044	7.95	1	3163.843292	3	1055.283569	-2.256010026	-0.002379221	98.62177747
																			WAIAQLNYADMLK	4	33350932	2xTMT6plex [N-Term; K13]	1.45			1.992862344	17.22	0	1995.113775	2	998.0598755	-0.651919431	-0.000650327	97.99608877
																			KYTGEDFDEDLR	2	33350932	2xTMT6plex [N-Term; K1]			1.107	4.132327557	14.47	1	1945.990742	3	649.3354492	0.540571046	0.000350649	52.62246863
																			QMVEHGGFYR	10	33350932	1xTMT6plex [N-Term]	0.937	1.064	1.602	3.191494465	20.36	0	1452.725535	3	484.9104614	-5.991257432	-0.002901218	36.63825504
																			LVQTPLTDR	2	33350932	1xTMT6plex [N-Term]			1.613	3.322702885	32.18	0	1271.752067	2	636.3800049	0.523793625	0.000333068	53.15766745
																			EALELTDTGLLSGSEER	5	33350932	1xTMT6plex [N-Term]		0.01	100	2.069284916	20.39	0	2049.05491	2	1025.029541	-1.514946591	-0.001552104	66.97612427
																			VKEHINSVSAMK	2	33350932	3xTMT6plex [N-Term; K2; K12]	1.145			4.236344337	22.16	1	2030.203542	3	677.4078979	2.757599196	0.001866163	30.33736195
																			EYQTQLIQR	2	33350932	1xTMT6plex [N-Term]		1.236		2.781258106	21.06	0	1407.779344	2	704.3932495	-0.086609143	-6.10E-05	38.11888741
																			VWEQIDQMK	6	33350932	2xTMT6plex [N-Term; K9]	1.905	100	0.01	3.143623829	39.39	0	1634.897634	2	817.9520264	-0.524959855	-0.000429128	71.52337685
																			SMANPPAAVK	2	33350932	2xTMT6plex [N-Term; K10]	1.472			4.142406464	45.73	0	1443.839391	2	722.4232788	-0.076395486	-5.52E-05	40.68362269
																			GWDDLFNK	6	33350932	2xTMT6plex [N-Term; K8]	2.08	1.165	1.226	3.039109468	31.62	0	1452.788736	2	726.897522	-0.666720951	-0.000484302	75.50857719
																			IMFEVQDLK	4	33350932	2xTMT6plex [N-Term; K9]	0.728	0.963		2.047068119	30.67	0	1580.912222	2	790.958252	-1.894187235	-0.001497272	83.86429499
																			GVWSELSK	10	33350932	2xTMT6plex [N-Term; K8]	0.756	0.922	0.957	2.711050987	34.18	0	1363.798572	2	682.4022217	-1.030665857	-0.00070281	58.03808985
																			KYEFGESDLR	8	33350932	2xTMT6plex [N-Term; K1]	1.607	1.378	1.498	4.196495056	30.04	1	1701.921206	3	567.9780273	-0.985779332	-0.00055924	43.57419252
																			AELGEYIR	4	33350932	1xTMT6plex [N-Term]	1.524	1.037		2.814254045	43.8	0	1179.657104	2	590.3314819	-1.201029356	-0.000708401	51.71634224
																			VPLAIVNK	8	33350932	2xTMT6plex [N-Term; K8]	1.356	100	0.843	3.315285444	38.72	0	1311.876429	2	656.4412231	-0.959793857	-0.000629565	62.98908363
																			RVEDVLGK	4	33350932	2xTMT6plex [N-Term; K8]	1.153	1.248		4.117147923	22.72	1	1373.851671	3	458.6224365	0.790318443	0.000361927	42.1236826
																			DLFQVAFNR	5	33350932	1xTMT6plex [N-Term]	0.998			2.860225677	32.85	0	1338.736751	2	669.87146	-0.827659451	-0.000554009	68.52221187
																			DLSSQLLK	6	33350932	2xTMT6plex [N-Term; K8]	100	0.01	100	2.690280437	27.01	0	1361.840437	2	681.4230957	-1.117864992	-0.000761177	54.29185406
																			ILDDDTIITTLENLKR	4	33350932	2xTMT6plex [N-Term; K15]		100		2.182885885	10.42	1	2331.353547	3	777.7893066	-0.077464924	-6.02E-05	105.05797
																			MVVLSLPR	2	33350932	1xTMT6plex [N-Term]	1.119			2.871735096	23.55	0	1143.712117	2	572.359314	-0.669119704	-0.00038264	67.47913485
																			VAAPDVVVPTLDTVR	6	33350932	1xTMT6plex [N-Term]	2.301	1.135	1.697	2.77099824	31.39	0	1781.037015	2	891.0209961	-1.291277205	-0.001149906	68.49081805
																			DLEASIAR	6	33350932	1xTMT6plex [N-Term]	1.317	1.276	1.094	3.305097342	36.08	0	1103.625804	2	552.3161621	-0.685314939	-0.000378166	44.56476667
																			LYQEMFAWK	2	33350932	2xTMT6plex [N-Term; K9]				1.804437041	16.44	0	1673.912556	2	837.4584351	-1.769926871	-0.001481351	83.78449471
																			QNLDALLNQLK	3	33350932	2xTMT6plex [N-Term; K11]	100			1.739287615	19.96	0	1728.04199	2	864.5244141	-0.253937695	-0.000219407	87.1937324
																			DIQMPDGIR	2	33350932	1xTMT6plex [N-Term]	1.044			3.021669388	33.99	0	1273.677188	2	637.3421631	-0.10833053	-6.90E-05	60.0271372
																			LAETVFNFQEK	4	33350932	2xTMT6plex [N-Term; K11]		100	100	1.8875705	9.26	0	1783.999457	2	892.5019531	-1.58473703	-0.001413585	71.03505071
																			TVENIKDPLFR	4	33350932	2xTMT6plex [N-Term; K6]	100		100	1.535580397	6.79	1	1790.05764	3	597.3581543	1.267702544	0.00075642	58.55326939
																			DQQEAEK	2	33350932	2xTMT6plex [N-Term; K7]	0.872			2.953952789	31.59	0	1305.705066	2	653.3566284	0.700277545	0.000457178	27.00855869

Bhat et al,2017. Cigarette smoke and chewing tobacco alter expression of different sets of miRNAs in oral keratinocytes

Supplementary Table S2(A). List of all proteins and peptides identified in OKF6/TERT1 cells chronically treated with chewing tobacco using Sequest and Mascot search algorithms

Accession	Gene_Symbol	Description	Gene_ID	Refseq_Accession	Coverage	Proteins	#Unique_Peptides	#Peptides	#PSMS	#AAs	MW [kDa]	calc. pI	OKF6-STE-6M/OKF6P_R1	OKF6-STE-6M/OKF6P_R2	OKF6-STE-6M/OKF6P_R3	Median fold change	Status	P-value (6M)	Sequence	#PSMs	Protein Accessions	Modifications	OKF6-STE-6M/OKF6P_R1	OKF6-STE-6M/OKF6P_R2	OKF6-STE-6M/OKF6P_R3	Xcorr	Ionscore	# Missed Cleavages	Theo. MH+ [Da]	Charge	m_z	DeltaM [ppm]	Deltam/z [Da]	RT [min]
																			LSCLPAFK	2	33350932	2xTMT6plex [N-Term; K8]; 1xCarbamidomethyl [C3		100		1.851400852	11.18	0	1393.827764	2	697.416748	-1.108369549	-0.000772438	78.10698844
																			SWSHYTVPAGMTVIQWVSDFSER	1	33350932	1xTMT6plex [N-Term]				1.383899331		0	2912.423879	3	971.4787598	-0.739295932	-0.000717714	99.08715605
																			ESPEVLLTLDILK	3	33350932	2xTMT6plex [N-Term; K13]				1.663705349	6.67	0	1928.172001	2	964.588562	-1.117076983	-0.001076958	108.230017
																			QLTAYMK	2	33350932	2xTMT6plex [N-Term; K7]	0.12			2.883892059	23.57	0	1312.769915	2	656.8882446	-0.534893571	-0.000351096	52.71644999
																			SQELEVK	2	33350932	2xTMT6plex [N-Term; K7]	1.481			2.731903076	32.76	0	1290.766938	2	645.8860474	-1.642251111	-0.001059882	42.21903731
																			VEDVLGK	4	33350932	2xTMT6plex [N-Term; K7]	1.136	1.728		3.051529408	38.75	0	1217.75056	2	609.37854	-0.620916877	-0.000378061	60.83326875
																			MAPSVEK	4	33350932	2xTMT6plex [N-Term; K7]	1.429	1.438		2.25663209	33.2	0	1219.712066	2	610.3591309	-0.885882234	-0.000540261	44.94089721
																			LDMEIER	6	33350932	1xTMT6plex [N-Term]	1.779	1.679	1.715	3.06155467	33.12	0	1134.602626	2	567.8056641	1.256691081	0.000712923	52.75215241
																			WQALLVQIR	1	33350932	1xTMT6plex [N-Term]	2.75			1.550302863		0	1355.836071	2	678.4217529	0.116377915	7.89E-05	96.72639431
																			MPDGPVALEESYSAVMGIVSEVEQYVK	2	33350932	2xTMT6plex [N-Term; K27]				1.600943208		0	3385.731152	3	1129.247192	-1.219125612	-0.001375877	109.9058539
																			EVDGLDVSK	2	33350932	2xTMT6plex [N-Term; K9]				1.531399131	2.18	0	1419.809531	2	710.40802	-0.540537953	-0.00038373	52.73721441
																			MQMLEDEDDLAYAETEK	1	33350932	2xTMT6plex [N-Term; K17]				1.016159773		0	2489.182778	2	1245.094116	-0.732034683	-0.000911084	66.25845417
																			VPVNLLR	2	33350932	1xTMT6plex [N-Term]	1.353			2.137464046	15.82	0	1039.682531	2	520.345459	1.067920944	0.000555149	58.30844857
																			VDNEFDQR	2	33350932	1xTMT6plex [N-Term]	1.211			2.712258816	31.23	0	1251.616696	2	626.3120728	0.138347318	8.66E-05	39.39566556
																			LVEAISR	4	33350932	1xTMT6plex [N-Term]	0.658	0.64	1.536	2.589752197	25.12	0	1016.630161	2	508.8187561	0.073268563	3.72E-05	48.05601655
																			AHQANQLYPFAISLIESVR	1	33350932	1xTMT6plex [N-Term]				1.50446105		0	2386.308045	3	796.1072388	-0.369596628	-0.00029399	100.9976245
																			LNTQEIFDDWAR	4	33350932	1xTMT6plex [N-Term]				1.400369525	3.65	0	1736.880515	2	868.9432373	-0.758135412	-0.000658395	78.72997413
																			REEFVQWVELLPDTQTPSWLGLPNNAER	1	33350932	1xTMT6plex [N-Term]	100			1.381994009	0.88	1	3553.827697	3	1185.278442	-1.948074626	-0.002307707	107.5439716
																			TMAITPR	2	33350932	1xTMT6plex [N-Term]	0.94			2.349688768	18.94	0	1018.591667	2	509.7991943	-0.544956476	-0.000277544	41.32476263
																			WTDENIDTVALK	1	33350932	2xTMT6plex [N-Term; K12]				0.953518987	8.68	0	1863.0264	2	932.0164185	-0.450587247	-0.000419728	62.96775199
112382250	SPTBN1	spectrin beta chain, non-erythrocytic 1 isoform	6711	NP_003119.2	36.4213198	1	70	70	387	2364	274.439	5.57	1.09	1.08	1.1	1.09	Significant	0.000028																
																			LESEHPDQAQAILSR	6	112382250	1xTMT6plex [N-Term]	1.105	0.792	1.328	5.124026775	44.91	0	1923.01332	3	641.6722412	-5.797847094	-0.003716446	41.58803776
																			IVSSSDVGHDEYSTQSLVK	5	112382250	2xTMT6plex [N-Term; K19]	100	1.315		4.408159733	31.68	0	2509.318618	3	837.1113281	0.323958462	0.000270972	53.53528553
																			TALPAQSAATLPAR	6	112382250	1xTMT6plex [N-Term]	1.164	1.131	1.597	4.46340847	65.68	0	1596.927071	2	798.9680176	1.05667709	0.000843718	41.49774892
																			DALLSALSIQNYHLECNETK	8	112382250	2xTMT6plex [N-Term; K20]; 1xCarbamidomethyl [C16	0.823	100		4.436986446	17.3	0	2777.4544	3	926.489563	-0.095304951	-8.82E-05	74.59180561
																			KHEAIETDIAAYEER	6	112382250	2xTMT6plex [N-Term; K1]	1.107	1.151	1.17	5.577035427	53.66	1	2233.186482	3	745.0600586	-9.340679302	-0.00695316	45.14403977
																			FATDGEGYKPCDPQVIR	5	112382250	2xTMT6plex [N-Term; K9]; 1xCarbamidomethyl [C11	0.645	0.921	0.844	3.394827127	17.56	0	2411.242951	3	804.4186401	-0.656988896	-0.000528053	47.02236335
																			HEVSASTQSTPASSR	6	112382250	1xTMT6plex [N-Term]	100	2.364	1.311	3.679306269	27.81	0	1773.89287	3	591.9691772	0.061006097	3.61E-05	17.34256841
																			EVVAGSHELGQDYEHVTMLQER	10	112382250	1xTMT6plex [N-Term]	0.681	0.785		5.885603428	46.44	0	2756.351108	3	919.4546509	-0.619936726	-0.000569588	59.90924073
																			DVSSVELLMNNHQGIK	6	112382250	2xTMT6plex [N-Term; K16]	1.796		0.01	3.777421236	35.55	0	2242.226573	3	748.079834	-0.724287587	-0.000541339	66.45214995
																			QALQDTLALYK	6	112382250	2xTMT6plex [N-Term; K11]	0.842		100	3.654411077	51.56	0	1722.020192	2	861.5137939	0.069111051	5.95E-05	73.54576963
																			LVSQDNFGFDLPAVEAATK	4	112382250	2xTMT6plex [N-Term; K19]	1.68			4.349299908	51.19	0	2480.343711	3	827.4519653	-0.954744254	-0.000789365	92.99000506
																			AQQYYFDAAEAEAWMSEQELYMMSEEK	43	112382250	2xTMT6plex [N-Term; K27]; 1xOxidation [M	1.026	100	1.167	4.098066807	32.34	0	3752.684672	4	934.9273071	-0.631099858	-0.000589556	106.3962402
																			AQQYYFDAAEAEAWMSEQELYMMSEEK	12	112382250	2xTMT6plex [N-Term; K27]	1.178	1.173	0.902	5.427389622	47	0	3736.689757	4	934.9273071	-0.631099858	-0.000589556	106.3962402
																			EAEKLESEHPDQAQAILSR	8	112382250	2xTMT6plex [N-Term; K4]	1.037		0.943	7.085224628	52.01	1	2609.393514	4	653.1031494	-1.052610016	-0.000686668	48.06588961
																			EIGQSVDEVEK	6	112382250	2xTMT6plex [N-Term; K11]	1.255	1.154	1.173	4.996728897	60.97	0	1690.926351	2	845.9666138	-0.236835234	-0.000200235	48.4309541
																			KQQMLENQMEVR	6	112382250	2xTMT6plex [N-Term; K1]	1.176	1.063	1.107	5.388741016	42.15	1	1992.077072	3	664.6952515	-2.947042337	-0.001956912	46.15567935
																			LVSDGNINSDR	4	112382250	1xTMT6plex [N-Term]	1.231	1.484		3.596393585	73.59	0	1418.743687	2	709.8748779	-0.851317004	-0.0006039	40.92296076
																			ELEAENYHDIK	10	112382250	2xTMT6plex [N-Term; K11]	1.39	1.248	1.198	4.207282543	37.17	0	1818.9638	3	606.9919434	-1.386878549	-0.000840894	40.44670021
																			VIESTQDLGNDLAGVMALQR	2	112382250	1xTMT6plex [N-Term]			1.445	5.158049107	55.21	0	2359.248875	3	787.0869141	-1.138566825	-0.000895387	93.54451107
																			VAHMEFCYQELCQLAAER	2	112382250	1xTMT6plex [N-Term]; 2xCarbamidomethyl [C7; C12			1.063	5.298651218	36	0	2484.167137	3	828.7270508	-0.216441254	-0.000179225	82.63704459
																			ALVADSHPESER	4	112382250	1xTMT6plex [N-Term]	0.949	0.873		4.635794163	55.99	0	1539.796451	3	513.9368896	-0.217661683	-0.000111718	27.86834777
																			VSEEAESQQQWDTSK	6	112382250	2xTMT6plex [N-Term; K15]		1.367	1.76	4.172775745	59.94	0	2210.097727	2	1105.554077	1.425790741	0.001575568	42.50073105
																			TQILAASYELHK	6	112382250	2xTMT6plex [N-Term; K12]	1.348	1.069	1.32	4.60140419	39.91	0	1832.068205	3	611.3574829	-5.627279325	-0.00343652	57.22332197
																			EVVAGSHELGQDYEHVTMLQER	2	112382250	1xTMT6plex [N-Term]; 1xOxidation [M18]				2.504973173	9.35	0	2772.346023	3	919.4546509	-0.619936726	-0.000569588	59.90924073
																			EIEELQSQAQALSQEGK	6	112382250	2xTMT6plex [N-Term; K17]	1.974			3.898685694	34.26	0	2346.25529	3	782.7543945	-2.838308465	-0.002219799	83.29074524
																			KQEDFMTTMDANEEK	4	112382250	3xTMT6plex [N-Term; K1; K15]	1.138	1.12		4.760604858	32.84	1	2504.261587	3	835.4263306	1.138818418	0.000950633	55.32736543
																			MLTAQDMSYDEAR	4	112382250	1xTMT6plex [N-Term]	1.392		100	3.799684763	48.04	0	1759.819236	2	880.4136353	0.430429329	0.000378739	55.66905517
																			DLMLWMEDVIR	2	112382250	1xTMT6plex [N-Term]			1.604	3.862531424	45.61	0	1649.859251	2	825.4336548	0.474028504	0.00039104	108.1212414
																			WLHGLESQIQSDDYGK	5	112382250	2xTMT6plex [N-Term; K16]	100		0.01	3.194268465	11.34	0	2334.213031	3	778.7434692	1.209557485	0.000941122	74.50847115
																			KHQILEQAVEDYAETVHQLSK	2	112382250	3xTMT6plex [N-Term; K1; K21]				2.722865582	24.16	1	3153.751123	4	789.1930542	-0.233526076	-0.000184121	95.75660495
																			DLTSVNILLK	4	112382250	2xTMT6plex [N-Term; K10]	100			2.589254141	42.93	0	1573.992915	2	787.4987183	-1.75034877	-0.001377518	75.1473321
																			SGHFEQAIK	4	112382250	2xTMT6plex [N-Term; K9]	1.035	1.245		3.83498621	40.42	0	1474.841834	3	492.2852783	-0.374548002	-0.000184133	38.45509402
																			EAVCEVALDYK	8	112382250	2xTMT6plex [N-Term; K11]; 1xCarbamidomethyl [C4	1.542	100	0.346	2.379932642	34.14	0	1754.939893	2	877.9733276	-0.293267345	-0.000257333	55.08639591
																			AQQYYFDAAEAEAWMSEQELYMMSEEK	3	112382250	2xTMT6plex [N-Term; K27]; 2xOxidation [M22; M		100		2.069262028	19.51	0	3768.679587	4	934.9273071	-0.631099858	-0.000589556	106.3962402
																			LAEISDVWEEMK	6	112382250	2xTMT6plex [N-Term; K12]	100	1.419	0.623	2.455657244	31.77	0	1908.018872	2	954.5111084	-2.060442474	-0.001965682	91.45653471
																			EASLGEASK	6	112382250	2xTMT6plex [N-Term; K9]	1.21	0.87	0.774	3.724244118	37.63	0	1349.767666	2	675.3876343	0.241400573	0.000162917	37.34161622
																			EQWANLEQLSAIR	4	112382250	1xTMT6plex [N-Term]		1.778	100	2.534021139	32.86	0	1786.964913	2	893.9846802	-1.58332624	-0.001414674	71.11402384
																			LLDPEDISVDHPDEK	6	112382250	2xTMT6plex [N-Term; K15]	100	100	0.01	2.094882011	19.13	0	2180.1487	3	727.3873901	-0.496461477	-0.000360787	61.96166549
																			ILSSDDYGK	6	112382250	2xTMT6plex [N-Term; K9]		1.137	1.409	3.918862343	48.69	0	1455.809531	2	728.4081421	-0.359470316	-0.00026166	58.21976085
																			LEMNLGLQK	4	112382250	2xTMT6plex [N-Term; K9]	1.441	1.579		3.062952995	47.33	0	1503.896906	2	752.4521484	0.075879536	5.71E-05	75.60425005
																			EVDDLEQWIAER	8	112382250	1xTMT6plex [N-Term]	0.519	100		2.658827782	21.68	0	1731.875095	2	866.4407349	-0.520767023	-0.000450952	83.64345484
																			LQALDTGWNELHK	6	112382250	2xTMT6plex [N-Term; K13]	100		1.29	2.462620258	26.13	0	1983.106381	3	661.7061157	-1.304757049	-0.000862491	68.96383951
																			VAVVNQIAR	4	112382250	1xTMT6plex [N-Term]	1.085	1.031		3.048299551	38.19	0	1198.746922	2	599.8760376	-1.771470398	-0.001061772	35.90313836
																			QQMLENQMEVR	2	112382250	1xTMT6plex [N-Term]			1.375	2.672250032	19.06	0	1634.819177	2	817.9138184	0.72373677	0.000591589	44.04266025
																			EGEDMIAEEHFGSEK	4	112382250	2xTMT6plex [N-Term; K15]	100		0.77	2.701561451	18.14	0	2166.04252	3	722.6864014	0.983790273	0.000710311	36.51694819
																			AKDEQSAVSMLK	4	112382250	3xTMT6plex [N-Term; K2; K12]	0.892		0.58	3.889499664	22.64	1	1994.155923	3	665.3922729	3.180497364	0.002114136	35.35057191
																			GNLEVLLFTIQSK	4	112382250	2xTMT6plex [N-Term; K13]		100		2.354687214	26.68	0	1920.15702	2	960.5795288	-2.728424164	-0.002619501	101.4231214
																			INAVVETGR	6	112382250	1xTMT6plex [N-Term]	1.38	1.421	1.651	3.276720285	44.82	0	1187.694552	2	594.3508301	-0.141984107	-8.43E-05	39.71028428
																			GVNASAQK	6	112382250	2xTMT6plex [N-Term; K8]	1.296	1.011	1.184	3.521588326	42.42	0	1232.736306	2	616.871582	-0.339932796	-0.000209524	27.32177198
																			LQAAYAGDK	4	112382250	2xTMT6plex [N-Term; K9]	1.122	0.898		3.763440132	34.96	0	1394.804386	2	697.9071655	1.913131844	0.001334222	43.76991767
																			DVEDEILWVGER	3	112382250	1xTMT6plex [N-Term]	1.391			2.645986557	17.93	0	1688.869281	2	844.9376831	-0.705672769	-0.000595895	74.22790584
																			QIEAQEKPR	2	112382250	2xTMT6plex [N-Term; K7]			2.316	2.822591782	5.85	0	1556.916062	3	519.6433105	-0.438828651	-0.00022774	28.40484118
																			DLVAIEAK	4	112382250	2xTMT6plex [N-Term; K8]	1.045		0.848	3.693351269	49.77	0	1316.818973	2	658.9136353	0.77492642	0.000510219	55.78608233
																			VQAVVAVAR	10	112382250	1xTMT6plex [N-Term]	1.14	0.8	1.188	3.398807049	40.73	0	1141.725458	2	571.3658447	-0.915804117	-0.000522798	35.7045599
																			LILEVHQFSR	8	112382250	1xTMT6plex [N-Term]	0.723	0.599	0.918	3.679234505	24.59	0	1470.863014	3	490.9598389	1.325039479	0.000649651	61.16000657
																			DLDDFQSWLSR	6	112382250	1xTMT6plex [N-Term]	0.01	1.854	0.67	2.790369034	23.92	0	1610.801202	2	805.9059448	2.117671897	0.001705574	93.23253228
																			MWEVLESTTQTK	4	112382250	2xTMT6plex [N-Term; K12]				2.07010746	9.37	0	1911.029771	2	956.0166016	-2.011514974	-0.001922032	73.5141501
																			HQILEQAVEDYAETVHQLSK	2	112382250	2xTMT6plex [N-Term; K20]				1.545331001	0.84	0	2796.493228	3	932.8355103	-0.447320333	-0.000416976	89.77761222
																			VLVLSQDYGK	4	112382250	2xTMT6plex [N-Term; K10]			0.966	2.20069623	30.69	0	1579.945965	2	790.468811	-9.885977138	-0.007809655	58.94491444
																			ENEVLEAWK	4	112382250	2xTMT6plex [N-Term; K9]				1.964211106	20.73	0	1575.878279	2	788.4415894	-1.50838367	-0.001188515	74.29463248
																			ITDLYTDLR	6	112382250	1xTMT6plex [N-Term]	100			2.206028461	41.66	0	1338.746647	2	669.8765869	-0.560249302	-0.000375016	63.57248195
																			EMIDKWEDRWEWLR	2	112382250	2xTMT6plex [N-Term; K5]				2.314935207	4.02	2	2450.269106	4	613.3237305	1.626660104	0.000996439	84.55653412
																			LLQLTEK	8	112382250	2xTMT6plex [N-Term; K7]	1.182	1.986	0.915	3.287641287	39.85	0	1302.839709	2	651.9225464	-1.452793118	-0.000946378	56.75995049
																			DALLLWCQMK	6	112382250	2xTMT6plex [N-Term; K10]; 1xCarbamidomethyl [C7		100	100	2.222469807	13.72	0	1735.96394	2	868.4841919	-1.631912318	-0.00141647	81.81786608
																			LNDGNEYLFQAK	2	112382250	2xTMT6plex [N-Term; K12]				1.411642194	16.78	0	1870.011084	2	935.5075684	-1.72403323	-0.001611981	72.56983029
																			YKEVAELTR	5	112382250	2xTMT6plex [N-Term; K2]	0.773	0.882	0.648	2.546206236	11.47	1	1566.925564	3	522.979248	-1.514289785	-0.000790926	46.53649263
																			IDDIFER	2	112382250	1xTMT6plex [N-Term]	1.604			2.810217857	22.8	0	1136.614905	2	568.8102417	-1.494016624	-0.000849061	65.2687297
																			DDEEMNTWIQAISSAISSDKHEVSASTQSTPASSR	1	112382250	2xTMT6plex [N-Term; K20]				1.522373319	1.21	1	4224.040293	4	1056.762939	-2.453943803	-0.002591389	97.82958725
																			SWHNVYCVINNQEMGFYK	1	112382250	2xTMT6plex [N-Term; K18]; 1xCarbamidomethyl [C7				1.342628717		0	2747.347433	3	916.4536743	-0.350665798	-0.000321134	80.0853616
																			KLPEELGR	2	112382250	2xTMT6plex [N-Term; K1]	1.113			2.709349155	8.5	1	1399.867321	3	467.2937317	-0.484889982	-0.000226261	41.51663239
																			TAASGIPYHSEVPVSLK	1	112382250	2xTMT6plex [N-Term; K17]			0.281	1.747842193		0	2214.253439	3	738.756897	1.21858136	0.000899416	59.65316506
																			DLTSVMR	2	112382250	1xTMT6plex [N-Term]			1.179	2.464381218	28.95	0	1050.581496	2	525.7943115	-0.142695379	-7.50E-05	49.33739171
																			ALAVEGK	1	112382250	2xTMT6plex [N-Term; K7]	1.372			2.924006224	35.83	0	1145.72943	2	573.3685913	0.41522647	0.000237869	40.04461042
15431310	KRT14	keratin, type I cytoskeletal 14	3861	NP_000517.2	73.72881356	1	18	39	1697	472	51.589	5.16	0.73	0.71	0.73	0.72	Significant	0.000085																
																			LLEGEDAHLSSSQFSSGSQSSR	59	15431310	1xTMT6plex [N-Term]	0.924	0.98	0.971	7.097453594	77.57	0	2538.226954	2	1269.616699	-0.32766672	-0.000415846	46.21967046
																			ASLENSLEETK	70	15431310	2xTMT6plex [N-Term; K11]	0.749	0.681	0.749	5.134794235	76.83	0	1678.926351	2	839.9658203	-1.183723752	-0.000993693	47.08874863
																			ADLEMQIESLKEELAYLK	16	15431310	3xTMT6plex [N-Term; K11; K18]	0.99	1.045	0.697	5.534199238	55.35	1	2810.582844	3	937.5316162	-0.906860722	-0.000849602	106.2077722
																			ALEEANADLEVK	217	15431310	2xTMT6plex [N-Term; K12]	0.606	0.627	0.555	5.218453407	90.06	0	1759.984201	2	880.4959717	0.264854295	0.00023307	51.10169088
																			ISSVLAGGSCR	12	15431310	1xTMT6plex [N-Term]; 1xCarbamidomethyl [C10	0.724	0.759	0.837	4.074535847	52.1	0	1335.725201	2	668.3656616	-0.863948522	-0.000576999	54.83568128
																			MSVEADINGLR	6	15431310	1xTMT6plex [N-Term]	1.436	1.688		3.755783319	62.24	0	1433.76198	2	717.3839111	-1.000196961	-0.000717022	64.76776505
																			KNHEEEMNALR	11	15431310	2xTMT6plex [N-Term; K1]	0.773	0.864	0.797	3.628271818	36.26	1	1828.973987	3	610.3292847	-0.375246503	-0.000228772	35.80008094
																			ASLENSLEETKGR	10	15431310	2xTMT6plex [N-Term; K11]	0.24	0.184	0.187	5.08894968	44.04	1	1892.048926	3	631.3532104	-2.033743573	-0.001282647	42.91752663
																			ILTATVDNANVLLQIDNAR	22	15431310	1xTMT6plex [N-Term]	0.944	1.211	1.273	5.63916111	65.48	0	2283.286975	3	761.7672729	0.127184038	9.68E-05	95.96319519
																			GSCGIGGGIGGGSSR	158	15431310	1xTMT6plex [N-Term]; 1xCarbamidomethyl [C3	0.79	0.779	0.729	5.403667927	114.21	0	1507.748455	2	754.3773193	-0.724980431	-0.000546544	34.52531152
																			YCMQLAQIQEMIGSVEEQLAQLR	10	15431310	1xTMT6plex [N-Term]; 1xCarbamidomethyl [C2	0.564	0.241		4.682034492	40.77	0	2967.49395	3	995.1660767	-1.73886338	-0.001729293	117.0940986
																			YCMQLAQIQEMIGSVEEQLAQLR	22	15431310	1xTMT6plex [N-Term]; 1xCarbamidomethyl [C2]; 1xOxidation [M11]	0.874	1.102	0.801	6.575696945	64.21	0	2983.488865	3	995.1660767	-1.73886338	-0.001729293	117.0940986
																			YCMQLAQIQEMIGSVEEQLAQLR	9	15431310	1xTMT6plex [N-Term]; 1xCarbamidomethyl [C2]; 2xOxidation [M3; M11]	100		1.212	5.655329704	62.51	0	2999.48378	3	995.1660767	-1.73886338	-0.001729293	117.0940986
																			APSTYGGGLSVSSSR	181	15431310	1xTMT6plex [N-Term]	0.656	0.631	0.626	5.400057316	76.28	0	1654.859779	2	827.9257813	-9.362388399	-0.00774672	39.14057374
																			EVATNSELVQSGK	82	15431310	2xTMT6plex [N-Term; K13]	0.696	0.701	0.693	5.450167656	73.38	0	1820.016563	2	910.5032349	-9.54398646	-0.008685107	43.74892866
																			TMQNLEIELQSQLSMK	76	15431310	2xTMT6plex [N-Term; K16]	0.916	1.066	100	4.230701447	53.2	0	2351.271474	3	784.4284668	-0.266583158	-0.000208936	95.83338731
																			TMQNLEIELQSQLSMK	24	15431310	2xTMT6plex [N-Term; K16]; 1xOxidation [M15	0.863	0.954	1.001	3.193665028	48.84	0	2367.266389	3	784.4284668	-0.266583158	-0.000208936	95.83338731
																			GQVGGDVNVEMDAAPGVDLSR	10	15431310	1xTMT6plex [N-Term]; 1xOxidation [M11]	0.853	0.714	0.585	4.618817329	72.51	0	2331.144804	2	1166.07605	0.008000093	9.32E-06	55.83773387
																			GQVGGDVNVEMDAAPGVDLSR	155	15431310	1xTMT6plex [N-Term]	0.839	0.724	0.609	5.678504944	66.99	0	2315.149889	2	1166.07605	0.008000093	9.32E-06	55.83773387
																			QRPAEIKDYSPYFK	4	15431310	3xTMT6plex [N-Term; K7; K14]	1.245	1.419		5.293942451	18.92	1	2429.379592	4	608.1005249	0.278955737	0.000169422	48.07340145
																			QRPAEIKDYSPYFKTIEDLR	2	15431310	3xTMT6plex [N-Term; K7; K14]	0.541			6.562573433		2	3156.766045	5	632.1588745	-0.246829	-0.000155836	78.91012884
																			QFTSSSSMK	25	15431310	2xTMT6plex [N-Term; K9]	0.795	0.786	0.734	4.360666752	55.43	0	1460.781936	2	730.8937378	-1.189078274	-0.000868492	32.92057792
																			ADLEMQIESLK	8	15431310	2xTMT6plex [N-Term; K11]	1.281	0.551		2.498028994	35.84	0	1734.971193	2	867.987793	-1.662167367	-0.001441906	71.02939572
																			NHEEEMNALR	8	15431310	1xTMT6plex [N-Term]		0.962	1.027	4.156452179	41.93	0	1471.716092	2	736.3625488	1.17476887	0.000864463	28.88820698
																			LEQEIATYR	30	15431310	1xTMT6plex [N-Term]	0.873	0.965	0.895	3.989738941	51.78	0	1351.741896	2	676.3740845	-0.742660038	-0.000501942	59.56020918
																			VVSTHEQVLR	74	15431310	1xTMT6plex [N-Term]	0.712	0.722	0.753	4.458293438	64.6	0	1396.810979	3	466.2755127	0.72022339	0.000335339	33.70728172
																			MSVEADINGLRR	4	15431310	1xTMT6plex [N-Term]		3.428		3.775520086	34.04	1	1589.863091	3	530.6262207	0.640438401	0.000339403	47.35430851
																			ADLEMQIESLKEELAYLK	10	15431310	3xTMT6plex [N-Term; K11; K18]; 1xOxidation [M5	100	1.285	1.461	2.494909048	26.58	1	2826.577759	3	937.5316162	-0.906860722	-0.000849602	106.2077722
																			EVATNSELVQSGKSEISELR	2	15431310	2xTMT6plex [N-Term; K13]	0.01			3.065496922	15.76	1	2634.435045	3	878.815918	-0.700037662	-0.000614735	60.33944928
																			DAEEWFFTK	79	15431310	2xTMT6plex [N-Term; K9]	0.765	0.778	0.679	3.898383379	50.42	0	1630.85173	2	815.9307861	1.573175279	0.001282808	73.73351799
																			VLDELTLAR	31	15431310	1xTMT6plex [N-Term]	0.836	0.802	0.88	3.858995199	41.24	0	1258.756818	2	629.8816528	-0.626805691	-0.000394498	67.98248171
																			KDAEEWFFTK	16	15431310	3xTMT6plex [N-Term; K1; K10]	0.711	0.489	0.439	4.102272034	23.34	1	1988.109625	3	663.3737183	-1.520768677	-0.001007818	65.04503686
																			NHEEEMNALR	12	15431310	1xTMT6plex [N-Term]; 1xOxidation [M6]	100	1.113	1.026	3.034799576	35.28	0	1487.711007	2	736.3625488	1.17476887	0.000864463	28.88820698
																			CEMEQQNQEYK	10	15431310	2xTMT6plex [N-Term; K11]; 1xCarbamidomethyl [C1]; 1xOxidation [M3]	1.819	0.875	0.895	3.481099367	43.3	0	1960.914483	3	654.3094482	-0.352898325	-0.000230668	30.65856668
																			CEMEQQNQEYK	14	15431310	2xTMT6plex [N-Term; K11]; 1xCarbamidomethyl [C1	0.885	0.803	0.891	3.460950851	27.46	0	1944.919568	3	654.3094482	-0.352898325	-0.000230668	30.65856668
																			TMQNLEIELQSQLSMK	2	15431310	2xTMT6plex [N-Term; K16]; 2xOxidation [M2; M15			100	2.116810083	19.73	0	2383.261304	3	784.4284668	-0.266583158	-0.000208936	95.83338731
																			TKYETELNLR	12	15431310	2xTMT6plex [N-Term; K2]	0.883	0.88		3.41303134	25.69	1	1724.994706	2	862.9996948	-1.5030432	-0.001296371	47.08014913
																			VTMQNLNDR	14	15431310	1xTMT6plex [N-Term]	0.936	0.83	0.827	3.519829035	42.79	0	1319.6939	2	660.3503418	-0.373735341	-0.000246608	33.03499222
																			KNHEEEMNALR	13	15431310	2xTMT6plex [N-Term; K1]; 1xOxidation [M7]	0.891	0.645	0.944	3.066138506	19.28	1	1844.968902	3	610.3292847	-0.375246503	-0.000228772	35.80008094
																			VMDVHDGK	20	15431310	2xTMT6plex [N-Term; K8]	0.786	0.516	0.78	3.494419336	45.36	0	1358.750242	3	453.5879211	-0.759368598	-0.000343931	31.09849038
																			YETELNLR	12	15431310	1xTMT6plex [N-Term]	1.389	1.264	1.178	3.169751167	43.48	0	1266.689132	2	633.8482666	0.09803757	6.21E-05	45.1476665
																			ADLEMQIESLK	2	15431310	2xTMT6plex [N-Term; K11]; 1xOxidation [M5	100			1.664554358	15.95	0	1750.966108	2	867.987793	-1.662167367	-0.001441906	71.02939572
																			VMDVHDGK	19	15431310	2xTMT6plex [N-Term; K8]; 1xOxidation [M2]	0.76	0.742	0.805	2.501206875	42.96	0	1374.745157	3	453.5879211	-0.759368598	-0.000343931	31.09849038
																			EELAYLK	16	15431310	2xTMT6plex [N-Term; K7]	0.771	1.132	1.244	3.250023842	39.59	0	1323.792424	2	662.3997803	-0.106083947	-7.02E-05	54.24797047
																			LASYLDK	51	15431310	2xTMT6plex [N-Term; K7]	0.689	0.687	0.676	3.160550594	38.22	0	1267.76621	2	634.3873291	0.924415808	0.000585972	69.69252583
																			MSVEADINGLR	4	15431310	1xTMT6plex [N-Term]; 1xOxidation [M1]	1.373	1.227		2.568712234	27.3	0	1449.756895	2	717.3839111	-1.000196961	-0.000717022	64.76776505
																			QFTSSSSMK	6	15431310	2xTMT6plex [N-Term; K9]; 1xOxidation [M8]		0.776	0.751	2.715188742	36.34	0	1476.776851	2	730.8937378	-1.189078274	-0.000868492	32.92057792
																			SEISELRR	2	15431310	1xTMT6plex [N-Term]		0.339		3.65511775	23.66	1	1218.700366	3	406.9051514	0.439034547	0.000178351	31.29536983
																			VTMQNLNDR	12	15431310	1xTMT6plex [N-Term]; 1xOxidation [M3]	0.775	0.76	0.858	2.103149652	31.21	0	1335.688815	2	660.3503418	-0.373735341	-0.000246608	33.03499222
																			DYSPYFK	18	15431310	2xTMT6plex [N-Term; K7]	0.722	0.787	0.802	3.307164431	35.13	0	1377.745474	2	689.37677	0.572848235	0.00039462	55.89878755
																			SEISELR	9	15431310	1xTMT6plex [N-Term]	0.814	0.792	0.706	2.968187571	40.38	0	1062.599255	2	531.8031006	-0.310830375	-0.000165144	43.56074208
																			ADLEMQIESLKEELAYLKK	2	15431310	4xTMT6plex [N-Term; K11; K18; K19]; 1xOxidation [M5	100			1.502521396	3.11	2	3183.835654	4	796.7140503	-0.4029285	-0.000320715	106.8927788
																			DVTSSSR	4	15431310	1xTMT6plex [N-Term]		1.627		2.553364992	30.27	0	980.5210045	2	490.7636108	-1.080527911	-0.00052974	27.03209141
223029410	TLN1	talin-1	7094	NP_006280.3	37.97717434	1	69	69	317	2541	269.599	6.07	1.26	1.38	1.49	1.38	Significant	0.0023																
																			VLVQNAAGSQEK	6	223029410	2xTMT6plex [N-Term; K12]	1.361	1.436	1.606	4.742009163	70.75	0	1701.989955	2	851.4997559	1.339819159	0.001140179	38.62901006
																			AATAPLLEAVDNLSAFASNPEFSSIPAQISPEGR	17	223029410	1xTMT6plex [N-Term]	100	1.568	1.132	4.656891346	41.36	0	3699.906735	3	1233.973267	-0.402303468	-0.000496162	111.0040937
																			AAMEPIVISAK	4	223029410	2xTMT6plex [N-Term; K11]	1.187	100		4.496215343	66.73	0	1587.954421	2	794.4810791	0.290060671	0.000230302	69.31288732
																			KFFYSDQNVDSR	4	223029410	2xTMT6plex [N-Term; K1]	1.298	1.25		5.050081253	48.94	1	1964.027797	3	655.3470459	-0.617239068	-0.000404092	45.71366668
																			ALSTDPAAPNLK	4	223029410	2xTMT6plex [N-Term; K12]	1.422	1.384		3.773159266	63.03	0	1655.973242	2	828.4892578	-1.209605898	-0.001001538	57.08842539
																			EGISQEALHTQMLTAVQEISHLIEPLANAAR	2	223029410	1xTMT6plex [N-Term]	1.219			2.703062534	9.39	0	3599.905295	4	900.7311401	-0.712427943	-0.000641168	115.5097996
																			DHFGLEGDEESTMLEDSVSPK	4	223029410	2xTMT6plex [N-Term; K21]	1.645	2.067		5.217783451	29.56	0	2780.333676	3	927.4493408	-0.074296501	-6.89E-05	71.8329336
																			EADESLNFEEQILEAAK	6	223029410	2xTMT6plex [N-Term; K17]	1.244	1.382		4.440696716	39.32	0	2394.244056	2	1197.624878	-0.658705039	-0.00078855	93.96398454
																			GVAALTSDPAVQAIVLDTASDVLDK	6	223029410	2xTMT6plex [N-Term; K25]	100	100		3.697403193	45.83	0	2927.634138	3	976.5483398	-1.254319555	-0.001224063	105.2150758
																			VQELGHGCAALVTK	6	223029410	2xTMT6plex [N-Term; K14]; 1xCarbamidomethyl [C8	1.451	1.491	1.497	5.334026337	47.47	0	1941.099188	3	647.7044678	-0.174019797	-0.000112597	53.21809579
																			ADAEGESDLENSR	6	223029410	1xTMT6plex [N-Term]	1.416	1.392	1.266	3.614718676	72.96	0	1621.750288	2	811.3777466	-1.277536963	-0.001035923	34.67900798
																			QEDVIATANLSR	6	223029410	1xTMT6plex [N-Term]	1.559	1.595	1.465	4.414402485	67.48	0	1545.843401	2	773.4250488	-0.37519567	-0.000289997	42.20394476
																			AVSSAIAQLLGEVAQGNENYAGIAAR	4	223029410	1xTMT6plex [N-Term]	100	1.06		3.601283312	48.37	0	2802.494736	3	934.8358154	-0.657568028	-0.000614277	109.4099031
																			EVANSTANLVK	4	223029410	2xTMT6plex [N-Term; K11]	1.046	1.175		4.356801987	45.18	0	1603.941942	2	802.4753418	0.913383279	0.000732507	42.42334998
																			EQGVEEHETLLLR	4	223029410	1xTMT6plex [N-Term]	1.35	1.332		4.474657536	60.37	0	1781.959493	2	891.4840698	0.768658571	0.000684859	43.3168639
																			GVGAAATAVTQALNELLQHVK	4	223029410	2xTMT6plex [N-Term; K21]		1.411	1.447	3.696480513	34.54	0	2549.481542	3	850.4971313	-1.843863921	-0.001566966	111.6999982
																			ALDGAFTEENR	6	223029410	1xTMT6plex [N-Term]	1.22	1.272	100	4.585151672	60.87	0	1451.732788	2	726.3703003	0.369183601	0.000267978	43.82112342
																			AVTDSINQLITMCTQQAPGQK	4	223029410	2xTMT6plex [N-Term; K21]; 1xCarbamidomethyl [C13	0.752	100		3.622307301	44.42	0	2762.458106	3	921.4899902	-0.973103219	-0.000896052	95.89643064
																			DPPSWSVLAGHSR	4	223029410	1xTMT6plex [N-Term]	1.039	1.047		4.361824512	47.45	0	1637.85972	3	546.6252441	0.891005412	0.000486447	54.49107524
																			LKPLPGETMEK	10	223029410	3xTMT6plex [N-Term; K2; K11]	0.924	1.475	1.486	3.992934942	31.15	0	1930.165031	3	644.0591431	-1.116713206	-0.00071848	52.2777419
																			TLAESALQLLYTAK	2	223029410	2xTMT6plex [N-Term; K14]				3.133969307	30.96	0	1980.178149	2	990.5914307	-1.295141993	-0.001282306	106.8251225
																			TLSHPQQMALLDQTK	2	223029410	2xTMT6plex [N-Term; K15]	0.769			4.323082924	43.57	0	2169.210194	3	723.7415161	-0.09189527	-6.64E-05	54.60280295
																			MATNAAAQNAIK	2	223029410	2xTMT6plex [N-Term; K12]	1.173			3.865390539	46.38	0	1661.940896	2	831.4744873	0.482489706	0.000400935	41.71424946
																			LHTDDELNWLDHGR	2	223029410	1xTMT6plex [N-Term]	1.725			5.378237724	38.39	0	1949.966704	3	650.6604004	-0.028712349	-1.87E-05	63.2870981
																			ASSLIEEAK	2	223029410	2xTMT6plex [N-Term; K9]			1.345	3.961882114	52.08	0	1405.830266	2	703.4190063	0.334133731	0.000234868	45.19114239
																			LQAAGNAVK	2	223029410	2xTMT6plex [N-Term; K9]			1.341	4.120853901	51.84	0	1329.825456	2	665.4157104	-0.986250907	-0.000655771	38.27875527
																			ELLENPVQPINDMSYFGCLDSVMENSK	3	223029410	2xTMT6plex [N-Term; K27]; 1xCarbamidomethyl [C18				2.213647366	1.53	0	3587.747213	3	1196.586304	-0.795662854	-0.000951546	97.9410663
																			EAAYHPEVAPDVR	4	223029410	1xTMT6plex [N-Term]	1.165			3.846283436	41.78	0	1682.86995	2	841.9382324	-0.452736261	-0.000380948	39.1965056
																			NLGTALAELR	2	223029410	1xTMT6plex [N-Term]	2.072			3.482999563	53.51	0	1286.762966	2	643.885376	0.395794049	0.000254647	67.63444033
																			GLAGAVSELLR	12	223029410	1xTMT6plex [N-Term]	0.571		100	3.147360563	51.47	0	1314.794266	2	657.9003296	-0.672044544	-0.0004418	84.11881998
																			AQEACGPLEMDSALSVVQNLEK	11	223029410	2xTMT6plex [N-Term; K22]; 1xCarbamidomethyl [C5	100			3.209315062	23.43	0	2847.46325	3	949.8267212	0.828746154	0.000786608	96.28570827
																			ACEFAGFQCQIQFGPHNEQK	3	223029410	2xTMT6plex [N-Term; K20]; 2xCarbamidomethyl [C2; C9				2.325842857	13.23	0	2854.380524	3	952.1288452	-2.992581191	-0.002847322	63.13278186
																			MVAAATNNLCEAANAAVQGHASQEK	3	223029410	2xTMT6plex [N-Term; K25]; 1xCarbamidomethyl [C10			100	2.240478754	0.64	0	3014.518808	3	1005.501648	-9.426928194	-0.009472551	63.63890006
																			EVIQEWNLTNIK	6	223029410	2xTMT6plex [N-Term; K12]	100	1.211	100	2.655072451	28.75	0	1945.115883	2	973.0598145	-1.815400193	-0.001765582	71.77590994
																			ALGDLISATK	6	223029410	2xTMT6plex [N-Term; K10]	1.623	1.152	1.229	3.114301205	43.11	0	1446.893201	2	723.9451904	-6.978200339	-0.005048355	65.07567377
																			KSTVLQQQYNR	2	223029410	2xTMT6plex [N-Term; K1]			1.106	3.845822096	15.79	1	1823.053952	3	608.3553467	-1.352215021	-0.00082172	37.88804462
																			VMVTNVTSLLK	6	223029410	2xTMT6plex [N-Term; K11]	1.11	1.18	1.914	2.368317842	37.75	0	1663.022835	2	832.0148315	-0.269619907	-0.000224192	76.06970637
																			AIAVTVQEMVTK	7	223029410	2xTMT6plex [N-Term; K12]	100	100	100	2.235929489	20.99	0	1748.039213	2	874.5230103	-0.268444886	-0.000234626	72.6302774
																			ADQDSEAMK	4	223029410	2xTMT6plex [N-Term; K9]	1.822	1.968		3.545555115	46.57	0	1452.740465	2	726.8737793	-0.126007538	-9.15E-05	34.64917707
																			ISIGNVVK	6	223029410	2xTMT6plex [N-Term; K8]	1.047	3.405	2.161	3.258071423	47.56	0	1287.840043	2	644.4227295	-1.444989721	-0.000930458	55.24953131
																			GTEWVDPEDPTVIAENELLGAAAAIEAAAK	2	223029410	2xTMT6plex [N-Term; K30]				1.881766915	1.71	0	3509.841565	3	1170.617798	-0.776235982	-0.000908155	112.6648484
																			AVAEQIPLLVQGVR	6	223029410	1xTMT6plex [N-Term]	100	100		1.77899158	20.97	0	1722.047521	2	861.5265503	-0.985213266	-0.000848292	71.09276201
																			GIWLEAGK	8	223029410	2xTMT6plex [N-Term; K8]	1.338	2.059	0.587	3.417806387	41.63	0	1331.808743	2	666.40802	0.015211691	1.01E-05	63.73179976
																			SIAAATSALVK	6	223029410	2xTMT6plex [N-Term; K11]	0.856		0.796	2.398469448	30.78	0	1489.9354	2	745.4699707	-1.835860635	-0.001367657	58.08873816
																			LAQAAQSSVATITR	6	223029410	1xTMT6plex [N-Term]		100		1.438318372	19.14	0	1645.943449	2	823.475708	0.419215876	0.000345003	50.02499569
																			GISMSSSK	6	223029410	2xTMT6plex [N-Term; K8]	1.428	1.289	1.444	3.37569499	51.19	0	1254.712794	2	627.859375	-1.052408174	-0.000660235	36.89695035
																			STVLQQQYNR	2	223029410	1xTMT6plex [N-Term]		1.436		3.401236773	23.35	0	1465.796057	2	733.4029541	1.756447026	0.001287297	33.37472466
																			AVEDEATK	4	223029410	2xTMT6plex [N-Term; K8]	1.337	1.172		2.828705549	52.7	0	1320.741117	2	660.8739014	-0.447397009	-0.000295448	32.84078969
																			GITMATAK	6	223029410	2xTMT6plex [N-Term; K8]	1.462	1.358	1.295	3.326780081	48.53	0	1250.754265	2	625.8805542	-0.346184358	-0.000216496	50.77261863
																			ADQDSEAMK	2	223029410	2xTMT6plex [N-Term; K9]; 1xOxidation [M8]		1.156		2.113275528	26.62	0	1468.73538	2	726.8737793	-0.126007538	-9.15E-05	34.64917707
																			KEEITGTLR	12	223029410	2xTMT6plex [N-Term; K1]	1.53	1.627	1.6	2.975605011	32.86	1	1504.909914	2	752.9581909	-0.537171066	-0.000404197	46.46963328
																			GSQAQPDSPSAQLALIAASQSFLQPGGK	4	223029410	2xTMT6plex [N-Term; K28]	100			1.962290645	9.31	0	3212.731561	3	1071.578247	-3.539990456	-0.003791013	90.08646648
																			QAAASATQTIAAAQHAASTPK	2	223029410	2xTMT6plex [N-Term; K21]				1.799798846	4.29	0	2453.351256	3	818.4572754	2.452609206	0.002005704	47.52595257
																			TMQFEPSTMVYDACR	3	223029410	1xTMT6plex [N-Term]; 1xCarbamidomethyl [C14				1.256722569	7.53	0	2064.939034	2	1032.970581	-2.493391107	-0.00257435	66.64789499
																			IGITNHDEYSLVR	4	223029410	1xTMT6plex [N-Term]	1.003	1.192		3.474494219	13.74	0	1745.938364	3	582.6508789	-0.160670764	-9.35E-05	46.03898023
																			KLEQLKPR	3	223029410	3xTMT6plex [N-Term; K1; K6]			1.599	3.249557257	5.44	1	1699.119736	4	425.5357666	0.882943219	0.000375057	42.38215133
																			TMLESAGGLIQTAR	4	223029410	1xTMT6plex [N-Term]				1.582463145	19.19	0	1676.920271	2	838.9628296	-1.126207575	-0.00094428	59.67834259
																			DKAPGQLECETAIAALNSCLR	3	223029410	2xTMT6plex [N-Term; K2]; 2xCarbamidomethyl [C9; C19	100			2.795638561	8.49	1	2775.453355	3	925.8213501	-1.389988063	-0.001285949	65.89337552
																			ALDYYMLR	4	223029410	1xTMT6plex [N-Term]	1.109		1.536	2.372262001	29.22	0	1273.68121	2	637.343689	-0.870720478	-0.00055451	61.06453926
																			AVGDASK	2	223029410	2xTMT6plex [N-Term; K7]		1.19		2.059243679	25.71	0	1105.661745	2	553.3342285	-0.510290559	-0.000282104	27.00284136
																			VSHVLAALQAGNR	2	223029410	1xTMT6plex [N-Term]				1.664830208	6.43	0	1564.91209	3	522.3091431	0.502372769	0.000262056	40.41252142
																			AGFLDLK	2	223029410	2xTMT6plex [N-Term; K7]	100			1.762420058	15.69	0	1221.76073	2	611.3844604	0.748017525	0.000456949	68.25145491
																			LLAALLEDEGGSGRPLLQAAK	2	223029410	2xTMT6plex [N-Term; K21]		100		1.065018773		0	2580.512507	3	860.8424683	0.133343727	0.000114698	89.23668355
																			AIADMLR	6	223029410	1xTMT6plex [N-Term]	1.617		1.404	1.671979308	20.1	0	1018.591667	2	509.799469	-0.005666371	-2.89E-06	51.48442657
																			SAQPASAEPR	4	223029410	1xTMT6plex [N-Term]	0.01			2.181855202	23.12	0	1242.66398	2	621.8358765	0.399166384	0.000248015	17.09134167
																			DPVQLNLLYVQAR	2	223029410	1xTMT6plex [N-Term]				1.298229218	7.56	0	1758.011135	2	879.5095215	0.359109643	0.000315659	87.22248152
																			GISMSSSK	2	223029410	2xTMT6plex [N-Term; K8]; 1xOxidation [M4]	1.436			1.868888021	19.33	0	1270.707709	2	627.859375	-1.052408174	-0.000660235	36.89695035

Bhat et al,2017. Cigarette smoke and chewing tobacco alter expression of different sets of miRNAs in oral keratinocytes

Supplementary Table S2(A). List of all proteins and peptides identified in OKF6/TERT1 cells chronically treated with chewing tobacco using Sequest and Mascot search algorithms

Accession	Gene_Symbol	Description	Gene_ID	Refseq_Accession	Coverage	Proteins	#Unique_Peptides	#Peptides	#PSMS	#AAs	MW [kDa]	calc. pI	OKF6-STE-6M/OKF6P_R1	OKF6-STE-6M/OKF6P_R2	OKF6-STE-6M/OKF6P_R3	Median fold change	Status	P-value (6M)	Sequence	#PSMs	Protein Accessions	Modifications	OKF6-STE-6M/OKF6P_R1	OKF6-STE-6M/OKF6P_R2	OKF6-STE-6M/OKF6P_R3	Xcorr	Ionscore	# Missed Cleavages	Theo. MH+ [Da]	Charge	m_z	DeltaM [ppm]	Deltam/z [Da]	RT [min]
																			MLDGTVK	2	223029410	2xTMT6plex [N-Term; K7]	1.361			2.362642288	35.93	0	1221.727716	2	611.3672485	-0.40535193	-0.000247615	52.05849037
																			KELIECAR	2	223029410	2xTMT6plex [N-Term; K1]; 1xCarbamidomethyl [C6	0.895			2.399632454	11.24	1	1476.860855	3	492.9581604	-0.627918889	-0.000309116	40.86944481
																			DVDNALR	4	223029410	1xTMT6plex [N-Term]	0.922	0.986		2.399350405	15.39	0	1031.568289	2	516.2872314	-1.069070644	-0.00055141	37.21469251
																			AVASAAAALVLK	2	223029410	2xTMT6plex [N-Term; K12]				1.104005098	9.87	0	1542.998335	2	772.00177	-1.34238054	-0.001035645	70.18676045
58530840	DSP	desmoplakin isoform I	1832	NP_004406.2	27.06374086	1	70	70	284	2871	331.569	6.81	0.64	0.63	0.74	0.67	Significant	0.0027															
																			LSLQDAVSQGVIDQDMATR	5	58530840	1xTMT6plex [N-Term]	0.291		100	3.562901258	36.94	0	2276.175376	3	759.3951416	-1.978878851	-0.001501425	73.49198791
																			YEVTSGGGGTSR	6	58530840	1xTMT6plex [N-Term]	0.435	0.765	0.665	3.864345789	60.65	0	1399.701488	2	700.3549194	0.767561653	0.000537179	28.07636079
																			RVEEDIQQQK	9	58530840	2xTMT6plex [N-Term; K10]	0.745	0.663	0.602	4.748207569	32.65	1	1730.980118	3	577.6595459	-9.262882062	-0.005344622	33.2331738
																			SVQNDSQAIAEVLNQLK	2	58530840	2xTMT6plex [N-Term; K17]	1.045			3.862733841	30.03	0	2315.297095	3	772.4373169	0.13061114	0.000100801	99.00753212
																			ELDECFAQANDQMEILDSLIR	4	58530840	1xTMT6plex [N-Term]; 1xCarbamidomethyl [C5	0.495			3.885095119	32.5	0	2739.316697	3	913.7781982	1.220993019	0.001114896	106.7387663
																			AVTGYNDPETGNIISLFQAMNK	2	58530840	2xTMT6plex [N-Term; K22]	0.188			2.913026571	26.49	0	2841.4857	3	947.8312378	-2.301648428	-0.002180034	103.612118
																			FLEFQYLTGGLVDPEVHGR	4	58530840	1xTMT6plex [N-Term]		0.878		4.234998226	36.01	0	2406.265512	3	802.7592773	-0.927881291	-0.000744243	99.74053728
																			GYFNEELSEILSDPSDDTK	8	58530840	2xTMT6plex [N-Term; K19]		0.516		3.393676996	41.86	0	2617.292129	3	873.1011353	-1.251712885	-0.001092033	89.3189436
																			EIMWINDCEEEELLYDWSDK	2	58530840	2xTMT6plex [N-Term; K20]; 1xCarbamidomethyl [C8]; 1xOxidation [M3]	0.758			3.918836117	37.55	0	3091.431676	3	1031.14917	0.414246783	0.000426872	100.6926852
																			EIMWINDCEEEELLYDWSDK	6	58530840	2xTMT6plex [N-Term; K20]; 1xCarbamidomethyl [C8	0.861	1.111	0.872	4.398246765	31.71	0	3075.436761	3	1031.14917	0.414246783	0.000426872	100.6926852
																			GAGSIAGASASPK	6	58530840	2xTMT6plex [N-Term; K13]	0.628	0.549	0.426	4.47387743	76.55	0	1531.884427	2	766.4395752	-8.194710484	-0.006276675	37.89113653
																			IKDFLQGSSCIAGIYNETTK	2	58530840	3xTMT6plex [N-Term; K2; K20]; 1xCarbamidomethyl [C10		0.763		4.175075054	27.48	1	2932.605705	3	978.2062988	-0.464408025	-0.000453975	89.74421404
																			GVITDQNSDGYCQTGTMSR	6	58530840	1xTMT6plex [N-Term]; 1xCarbamidomethyl [C12		0.943	0.638	3.726730824	41.34	0	2319.054275	2	1160.030029	-0.643683189	-0.000746368	38.82265535
																			GVITDQNSDGYCQTGTMSR	2	58530840	1xTMT6plex [N-Term]; 1xCarbamidomethyl [C12]; 1xOxidation [M17]	0.72			3.214697599	29.43	0	2335.04919	2	1160.030029	-0.643683189	-0.000746368	38.82265535
																			GFFDPNTEENLTYLQLK	7	58530840	2xTMT6plex [N-Term; K17]		0.86	0.414	2.832281113	32.4	0	2487.317162	3	829.7756958	-1.860477517	-0.001542533	86.55124131
																			GIVDSITGQR	2	58530840	1xTMT6plex [N-Term]	0.518			4.093389511	72.6	0	1274.726581	2	637.8676758	1.172347485	0.000747211	50.23866948
																			AITGFDDPFSGK	6	58530840	2xTMT6plex [N-Term; K12]	100	0.347	0.01	3.031881332	38.72	0	1712.925958	2	856.9678955	1.492688936	0.001278433	74.24149366
																			LISPESTVMLLEAQAATGGIIDPHR	3	58530840	1xTMT6plex [N-Term]			0.438	3.871632814	14.37	0	2848.543994	3	950.1855469	-0.669427962	-0.000635632	102.0138432
																			LKAEFQEEAK	6	58530840	3xTMT6plex [N-Term; K2; K10]	0.01	0.344	0.513	3.696209192	23.3	1	1880.109625	3	627.3746948	-0.049873338	-3.13E-05	44.45357973
																			AFIGFEGVK	9	58530840	2xTMT6plex [N-Term; K9]	0.578			3.585253954	51.03	0	1425.850608	2	713.4296265	0.959686576	0.000684185	80.39937102
																			SSSFSDTLEESSPIAAIFDTENLEK	2	58530840	2xTMT6plex [N-Term; K25]				2.193152905	13.87	0	3175.593455	3	1059.202393	-0.261615864	-0.000276929	107.0847284
																			LTYEIEDEKR	6	58530840	2xTMT6plex [N-Term; K9]		0.617	0.706	4.510401249	40.81	1	1753.973636	3	585.3258667	-6.037201544	-0.003529697	44.28251099
																			LTYEIEDEK	4	58530840	2xTMT6plex [N-Term; K9]	0.8	0.606		3.920749505	52.99	0	1597.872525	2	799.4401855	0.356407499	0.000284747	64.2900567
																			ETQSQLETER	8	58530840	1xTMT6plex [N-Term]	0.636	0.831	0.687	4.218556881	47	0	1449.738267	2	725.3726807	-0.125927472	-9.13E-05	25.24056946
																			ALLQAILQTEDMLK	4	58530840	2xTMT6plex [N-Term; K14]	100	0.01		2.022802353	16.72	0	2045.208069	2	1023.107483	-0.185814682	-0.000190015	108.9761017
																			LTVDSAIAR	6	58530840	1xTMT6plex [N-Term]	0.651	0.717	0.648	3.237674713	50.78	0	1174.699303	2	587.8528442	-0.758784342	-0.000445672	43.67948549
																			NKYETEINITK	2	58530840	3xTMT6plex [N-Term; K2; K11]		0.558		3.724973202	9.09	1	2040.194417	3	680.7359619	-0.531644339	-0.000361553	45.95691933
																			TVSVSEAIK	2	58530840	2xTMT6plex [N-Term; K9]	0.748			3.601284027	57.12	0	1391.851002	2	696.4296265	0.700153742	0.000487255	52.64491577
																			LSSEVEALR	4	58530840	1xTMT6plex [N-Term]	0.719	0.94		3.421760559	41.64	0	1232.704782	2	616.8554077	-1.008879279	-0.000621825	52.38260331
																			GLIDYETFK	4	58530840	2xTMT6plex [N-Term; K9]	0.777	0.01		3.059035063	41.16	0	1543.877217	2	772.4421387	-0.139775526	-0.000107898	79.88661781
																			EMSVQEAYK	4	58530840	2xTMT6plex [N-Term; K9]	0.88	0.606		3.93080616	41.03	0	1542.823801	2	771.9152832	-0.331174402	-0.000255472	49.61981471
																			ETQTECEWTVDTSK	2	58530840	2xTMT6plex [N-Term; K14]; 1xCarbamidomethyl [C6	100			2.897373676	25.44	0	2172.053085	2	1086.530029	-0.139460795	-0.000151458	84.18901646
																			IEVLEEELR	6	58530840	1xTMT6plex [N-Term]	0.322	0.362	100	2.989876747	34.39	0	1358.772862	2	679.890686	0.907797283	0.000616745	87.48658185
																			VVIVDPETNK	4	58530840	2xTMT6plex [N-Term; K10]	0.666	0.687		3.73093915	42.55	0	1571.940879	2	786.4724121	-2.119520711	-0.001665881	52.87873509
																			KQQLEVELR	2	58530840	2xTMT6plex [N-Term; K1]			0.633	3.329999447	16.8	1	1600.978662	3	534.3311768	0.196862985	0.000105058	48.39998979
																			AELIVQPELK	4	58530840	2xTMT6plex [N-Term; K10]	0.702	0.647		3.238352537	36.81	0	1597.992915	2	799.4993286	-0.960162854	-0.000767167	71.69814083
																			EYENELAK	2	58530840	2xTMT6plex [N-Term; K8]	0.584			3.805859327	44.89	0	1453.793881	2	727.4003906	-0.25876433	-0.000188095	44.21234295
																			ISITEGIER	8	58530840	1xTMT6plex [N-Term]	0.476	1.504	0.01	3.188753128	44.79	0	1246.720433	2	623.8647461	1.430190325	0.000891524	37.26386685
																			KQHLEIELK	4	58530840	3xTMT6plex [N-Term; K1; K9]		1.103	1.43	2.961714745	10.98	1	1825.15143	3	609.0554199	0.151349428	9.21E-05	42.09997112
																			GLVGIEFK	10	58530840	2xTMT6plex [N-Term; K8]	0.527	0.322	1.685	3.223669609	42.31	0	1320.829144	2	660.9189453	1.112751794	0.000734878	69.2977197
																			DLIDFDDR	6	58530840	1xTMT6plex [N-Term]	0.566	0.892	0.522	3.842499256	40.61	0	1237.626198	2	619.3171387	0.648769193	0.000401467	61.64915023
																			VLLQEEGTR	4	58530840	1xTMT6plex [N-Term]	0.604	0.673		3.590001345	41.98	0	1273.731332	2	637.3692627	-0.064989667	-4.14E-05	41.78819541
																			STLEAETR	6	58530840	1xTMT6plex [N-Term]	0.683	0.686	0.594	3.277604818	39.26	0	1135.615633	2	568.3114014	-0.09423578	-5.35E-05	27.55088303
																			LTEEETVCLDLDKVEAYR	2	58530840	2xTMT6plex [N-Term; K13]; 1xCarbamidomethyl [C8				2.817853689	7.7	1	2641.379504	3	881.1309204	-0.490728246	-0.000432067	92.63198263
																			LLEAASVSSK	2	58530840	2xTMT6plex [N-Term; K10]				2.047235727	24.54	0	1462.888116	2	731.9478149	0.162495552	0.000118856	48.40014326
																			IKNDLNLK	5	58530840	3xTMT6plex [N-Term; K2; K8]	0.668	0.647	0.789	3.145822525	19.05	1	1645.061553	3	549.0245361	-1.518223776	-0.000832524	47.60163058
																			QLQNIIQATSR	2	58530840	1xTMT6plex [N-Term]			0.562	1.850987673	22.19	0	1500.869556	2	750.9384155	-0.00121617	-9.13E-07	51.35917802
																			AQIDNLTR	4	58530840	1xTMT6plex [N-Term]	0.622	0.707		2.879713535	34.57	0	1159.663252	2	580.3347168	-0.944305382	-0.000547538	42.50639528
																			KQVQTSQK	4	58530840	3xTMT6plex [N-Term; K1; K8]	0.681	0.475		2.898154259	25.65	1	1634.020416	3	545.345459	0.861392619	0.000469178	27.05756838
																			LPVEEAYK	2	58530840	2xTMT6plex [N-Term; K8]	0.664			2.96903491	43.38	0	1406.829538	2	703.918396	-0.01616245	-1.14E-05	51.68727537
																			LGIYEAMK	2	58530840	2xTMT6plex [N-Term; K8]	0.636			2.763319016	27.91	0	1382.81178	2	691.909729	0.290551338	0.000200889	65.81748777
																			AEFQEEAK	2	58530840	2xTMT6plex [N-Term; K8]	0.811			3.407014847	29.93	0	1409.767666	2	705.3883057	1.183605047	0.000834304	26.64732201
																			EHLMLEEELR	4	58530840	1xTMT6plex [N-Term]	0.383	0.4		1.807705283	5.08	0	1527.803844	3	509.9399719	0.993616746	0.000506017	54.65537643
																			QKVEEELNR	2	58530840	2xTMT6plex [N-Term; K2]			0.591	2.563827991	10.71	1	1602.921541	3	534.9786987	0.001292269	6.90E-07	38.08166778
																			ISTEEAIR	4	58530840	1xTMT6plex [N-Term]	0.728	0.768		2.712381124	33.32	0	1147.652019	2	574.3296509	0.005653118	3.24E-06	36.88148717
																			YIELLTR	6	58530840	1xTMT6plex [N-Term]	0.539	100	0.797	2.271661758	31.55	0	1136.687676	2	568.8468018	-1.186820622	-0.000674522	59.45110798
																			QMGQPCDAYQK	2	58530840	2xTMT6plex [N-Term; K11]; 1xCarbamidomethyl [C6	0.623			2.97919631	20.46	0	1783.887146	2	892.4451904	-2.265855569	-0.002021015	43.40280615
																			LASLEELKR	2	58530840	2xTMT6plex [N-Term; K8]	0.298			2.561458588	21.44	1	1516.946299	3	506.3201294	-0.306007145	-0.000154732	60.45419194
																			WLPYEAGQR	2	58530840	1xTMT6plex [N-Term]		0.65		2.319362402	5.84	0	1348.721101	2	674.864502	0.464162865	0.000313013	57.71877743
																			MSQLEVK	4	58530840	2xTMT6plex [N-Term; K7]	0.577			2.950068712	27.18	0	1292.764829	2	646.8850098	-1.61399715	-0.001043259	49.11090761
																			YSLVEAK	2	58530840	2xTMT6plex [N-Term; K7]	0.853			3.228952169	34.49	0	1267.76621	2	634.3861084	-1.001338508	-0.000634732	55.19038845
																			LVFDGLR	4	58530840	1xTMT6plex [N-Term]	0.753	0.504		2.190494776	27.1	0	1048.635246	2	524.8214722	0.401680126	0.000210608	73.60940529
																			ENLGWQK	2	58530840	2xTMT6plex [N-Term; K7]	0.78			3.147212267	32.03	0	1332.767607	2	666.8878174	0.563890975	0.000375768	48.9061425
																			DKSEEVQK	2	58530840	3xTMT6plex [N-Term; K2; K8]	0.844			2.310736179	18.54	1	1649.967712	3	550.661377	1.130785386	0.00062192	32.68187092
																			EKEYEIER	2	58530840	2xTMT6plex [N-Term; K2]	0.592			2.35048008	7.54	1	1553.857544	3	518.6239014	-0.252795656	-0.000130936	36.9543732
																			LKQESDQLVLNQHPASDK	1	58530840	3xTMT6plex [N-Term; K2; K18]			0.941	3.293009043		1	2737.545154	4	685.1399536	-2.618764796	-0.001792247	43.35767022
																			SLLATMK	2	58530840	2xTMT6plex [N-Term; K7]	0.747			2.842253923	20.27	0	1221.764101	2	611.3860474	0.586779855	0.000358453	57.78284535
																			SMVEDITGLR	3	58530840	1xTMT6plex [N-Term]				1.583398223	0.7	0	1349.729617	2	675.3681641	-0.418946871	-0.000282732	63.88707161
																			VQYDLQK	2	58530840	2xTMT6plex [N-Term; K7]			0.712	3.076655865	26.5	0	1351.798572	2	676.40271	-0.317397972	-0.000214529	50.20529186
																			DMLANFR	4	58530840	1xTMT6plex [N-Term]	100			2.136819363	21.02	0	1095.581831	2	548.293457	-2.001920293	-0.001096634	48.24270999
																			VTAMQLYECQLIDK	1	58530840	2xTMT6plex [N-Term; K14]; 1xCarbamidomethyl [C9				1.109265685		0	2170.165218	2	1085.584229	-1.860687249	-0.002018999	74.65143949
																			DLKDEIVR	7	58530840	2xTMT6plex [N-Term; K3]	0.641	0.649	0.646	2.965539694	7.24	1	1445.8728	2	723.4408569	1.132455903	0.000818694	44.88289097
4501891	ACTN1	alpha-actinin-1 isoform b	87	NP_001093.1	60.53811659	1	28	45	415	892	102.993	5.41	0.88	0.86	0.8	0.85	Significant	0.00081															
																			ASIHEAWTDGK	29	4501891	2xTMT6plex [N-Term; K11]	0.819	0.739	0.777	4.337274551	52.02	0	1672.905891	2	836.956543	-0.048713141	-4.07E-05	41.97986562
																			HEAFESDLAAHQDR	4	4501891	1xTMT6plex [N-Term]	1.38	1.58		5.814505577	79.39	0	1854.893205	3	618.9694214	0.272937631	0.000168757	35.43437257
																			ETADTDTADQVMASFK	4	4501891	2xTMT6plex [N-Term; K16]; 1xOxidation [M12	0.963	0.641		3.566560984	48.17	0	2204.0793	3	730.032959	-0.027876839	-2.03E-05	74.3777998
																			ETADTDTADQVMASFK	11	4501891	2xTMT6plex [N-Term; K16]	1.117		1.003	4.968540668	57.8	0	2188.084385	3	730.032959	-0.027876839	-2.03E-05	74.3777998
																			IDQLEGDHQLIQEALIFDNK	6	4501891	2xTMT6plex [N-Term; K20]	2.402	100	100	4.15430069	19.68	0	2797.51363	3	933.1762695	0.223703557	0.000208605	92.50841277
																			VEQIAAIAQELNELDYYDSPSVNAR	7	4501891	1xTMT6plex [N-Term]	100		100	4.013683796	33.24	0	3037.531575	3	1013.179382	-2.627468109	-0.002660339	101.45071
																			KHEAFESDLAAHQDR	22	4501891	2xTMT6plex [N-Term; K1]	1.221	1.179	1.081	6.276325703	49.49	1	2212.1511	3	738.0541992	-1.381096323	-0.001018398	32.08635561
																			VLAVNQENEQLMEDYEK	10	4501891	2xTMT6plex [N-Term; K17]	1.031	0.95	0.8	3.999629021	58.15	0	2510.284875	3	837.4332886	0.174160575	0.000145731	75.60132926
																			LAILGIHNEVSK	8	4501891	2xTMT6plex [N-Term; K12]	0.84	0.903	0.82	4.003884792	49.95	0	1752.078376	3	584.6976318	-0.019167058	-1.12E-05	70.60197429
																			KQFGAQANVIGPWIQTK	6	4501891	3xTMT6plex [N-Term; K1; K17]	0.519		100	4.900903225	38.12	1	2573.517089	3	858.5097656	-0.911163563	-0.000781632	70.8907687
																			KTFTAWCNSHLR	14	4501891	2xTMT6plex [N-Term; K1]; 1xCarbamidomethyl [C7	0.781	0.745	0.66	5.023783207	36.72	1	1979.068556	3	660.3609009	-0.205699433	-0.000135698	47.56763229
																			KDDPLTNLNTAFDVAEK	4	4501891	3xTMT6plex [N-Term; K1; K17]	1.59	1.135		3.915622711	27.4	1	2578.433144	3	860.1489868	-0.285588472	-0.000245457	78.06096791
																			MVSDINNAWGCLEQVEK	4	4501891	2xTMT6plex [N-Term; K17]; 1xCarbamidomethyl [C11	0.954	0.988		3.94170928	38.13	0	2451.241237	3	817.7511597	-0.942781991	-0.000770329	88.07544328
																			ACLISLGYDIGNDPQGEAEFAR	16	4501891	1xTMT6plex [N-Term]; 1xCarbamidomethyl [C2	0.901	0.925	0.408	4.680227757	58.2	0	2625.281632	3	875.7651367	-0.295238324	-0.000258361	87.13511188
																			GISQEQMNEFR	6	4501891	1xTMT6plex [N-Term]	0.864	0.799	0.954	4.96427536	71.26	0	1567.773607	2	784.3902588	-0.233446891	-0.000182996	44.29899097
																			DGLGFCALIHR	4	4501891	1xTMT6plex [N-Term]; 1xCarbamidomethyl [C6	1.384			3.766245127	43.85	0	1487.799034	3	496.6047363	0.417713926	0.000207158	69.98472184
																			LSNRPAFMPSEGR	6	4501891	1xTMT6plex [N-Term]	0.809	1.414		4.672259331	31.18	0	1690.88964	3	564.3008423	-0.985359714	-0.000555378	34.99616168
																			LMLLLEVISGER	6	4501891	1xTMT6plex [N-Term]	1.529	0.977		4.07251215	44.34	0	1601.94978	2	801.4775391	-1.23531026	-0.000989452	112.6907183
																			HRPELIDYGK	6	4501891	2xTMT6plex [N-Term; K10]	0.949	0.904	0.963	4.155093193	30.33	0	1685.973911	3	562.6628418	0.03637104	2.04E-05	38.04295289
																			KAGTQIENIEEDFR	8	4501891	2xTMT6plex [N-Term; K1]	0.932	1.54		4.128901005	19.88	1	2108.138804	3	703.3851318	0.967041548	0.000679553	44.82282783
																			ICDQWDNLGALTQK	4	4501891	2xTMT6plex [N-Term; K14]; 1xCarbamidomethyl [C2	1.231			3.575600863	28.08	0	2120.121045	3	707.3781738	-0.508072701	-0.000359059	90.85272194
																			VPENTMHAMQQK	12	4501891	2xTMT6plex [N-Term; K12]	0.928	0.932	0.583	4.320982933	48.66	0	1871.987194	3	624.6676636	0.664081816	0.000414384	47.95685695
																			VLAVNQENEQLMEDYEK	2	4501891	2xTMT6plex [N-Term; K17]; 1xOxidation [M12				2.73925519	22.6	0	2526.27979	3	837.4332886	0.174160575	0.000145731	75.60132926
																			TINEVENQILTR	4	4501891	1xTMT6plex [N-Term]	1.053	0.357		3.503479242	38.67	0	1658.927465	2	829.9678345	0.559014985	0.000463683	75.81927579
																			MLDAEDIVGTARPDEK	6	4501891	2xTMT6plex [N-Term; K16]	0.979	1.362	100	3.662569761	9.42	0	2218.178954	3	740.0638428	-0.892051421	-0.000659577	65.9516523
																			DLLLDPAWEK	12	4501891	2xTMT6plex [N-Term; K10]	0.813	0.901	0.572	4.328368187	53.46	0	1657.956529	2	829.480835	-1.288404181	-0.001068059	87.29609035
																			DHSGTLGPEEFK	4	4501891	2xTMT6plex [N-Term; K12]	0.914		1.72	5.018087387	37.54	0	1774.937585	3	592.3175659	0.315342867	0.000186571	42.07077655
																			DYETATLSEIK	6	4501891	2xTMT6plex [N-Term; K11]	1.431	4.048	0.87	3.132280111	35.47	0	1727.946752	2	864.4762573	-0.876445736	-0.000757226	56.81717678
																			ELPPDQAEYCIAR	52	4501891	1xTMT6plex [N-Term]; 1xCarbamidomethyl [C10	0.858	0.866	0.824	4.162083626	66.41	0	1790.894451	2	895.951416	0.616921477	0.000552421	45.27772466
																			LASDLLEWIR	6	4501891	1xTMT6plex [N-Term]	1.309	1.315	1.315	3.386930704	34.01	0	1444.836131	2	722.9197998	-2.635558831	-0.001903975	99.6950994
																			TFTAWCNSHLR	6	4501891	1xTMT6plex [N-Term]; 1xCarbamidomethyl [C6	1.145	1.212	100	4.04608202	42.53	0	1621.810662	3	541.2745972	-0.877578454	-0.000474422	56.54119384
																			VPENTMHAMQQK	4	4501891	2xTMT6plex [N-Term; K12]; 1xOxidation [M	1.179	0.478		3.476126909	41.05	0	1887.982109	3	624.6676636	0.664081816	0.000414384	47.95685695
																			NYITMDELR	6	4501891	1xTMT6plex [N-Term]	0.999	0.422	1.357	4.054458618	67.4	0	1383.713967	2	692.3583984	-3.213565163	-0.002223328	58.52151635
																			AGTQIENIEEDFR	3	4501891	1xTMT6plex [N-Term]		100		2.657090187	19.39	0	1750.880909	2	875.9437256	-0.419267879	-0.000367044	61.54894165
																			QKDYETATLSEIK	7	4501891	3xTMT6plex [N-Term; K2; K13]	1.285	1.057	0.932	3.141475201	8.33	1	2213.263225	3	738.4255981	-0.444455226	-0.000327899	55.11367824
																			GYEEWLLNEIR	5	4501891	1xTMT6plex [N-Term]	0.919			2.728914261	28.04	0	1650.868888	2	825.9386597	0.699750263	0.000577598	98.48431158
																			NVNIQNFHISWK	2	4501891	2xTMT6plex [N-Term; K12]				2.037489176	26.42	0	1958.101236	3	653.3706055	-2.029181983	-0.001324448	65.45105583
																			GISQEQMNEFR	11	4501891	1xTMT6plex [N-Term]; 1xOxidation [M7]	0.832	0.898	0.808	2.943035603	53.09	0	1583.768522	2	784.3902588	-0.233446891	-0.000182996	44.29899097
																			VGWEQLLTTIAR	8	4501891	1xTMT6plex [N-Term]	0.83		1.103	2.625579834	24.77	0	1615.936908	2	808.4710693	-1.265815587	-0.001022739	94.62438909
																			ALDFIASK	18	4501891	2xTMT6plex [N-Term; K8]	0.918	1.079	1.387	3.579351664	49.04	0	1322.808409	2	661.9074097	-0.654723735	-0.000433037	72.28877043
																			LLETIDQLYLEYAK	6	4501891	2xTMT6plex [N-Term; K14]	1.445	0.567		2.30548811	15.47	0	2170.241143	2	1085.621948	-2.084356219	-0.002261778	109.9806516
																			SIVNYKPK	10	4501891	3xTMT6plex [N-Term; K6; K8]	0.739	0.777	100	2.771903276	12.55	0	1636.040089	3	546.0180664	-0.270813199	-0.000147687	48.996196
																			MAPYTGPDSVPGALDYMSFSTALYGESDL	6	4501891	1xTMT6plex [N-Term]	0.525	1.302		2.021089077	1.5	0	3284.521663	3	1095.512451	0.346186063	0.000379019	108.0955964
																			ATLPDADKER	5	4501891	2xTMT6plex [N-Term; K8]	1.034	0.907	0.883	3.816573858	18.77	1	1573.894992	3	525.3024902	-1.317957606	-0.000691442	33.7695688
																			IMSIVDPNR	8	4501891	1xTMT6plex [N-Term]	1.155	1.179		3.049948215	34.42	0	1273.713573	2	637.3606567	0.364137254	0.000231903	65.52208235
																			ILAGDKNYITMDELR	5	4501891	2xTMT6plex [N-Term; K6]	0.01	0.01	0.609	2.540530443	7.39	1	2210.22551	3	737.4121704	-1.607138962	-0.001184047	63.08025524
																			VPENTMHAMQQK	2	4501891	2xTMT6plex [N-Term; K12]; 2xOxidation [M6; M9	0.84			2.379861593	12.28	0	1903.977024	3	624.6676636	0.664081816	0.000414384	47.95685695
																			TIPWLENR	4	4501891	1xTMT6plex [N-Term]	0.669	0.915		2.966814518	17.42	0	1257.715288	2	629.3609009	-0.606251824	-0.000381246	70.11456491
																			CQLEINFNTLQTK	2	4501891	2xTMT6plex [N-Term; K13]; 1xCarbamidomethyl [C1				1.278594732	6.91	0	2067.130882	2	1034.06897	-0.10594727	-0.000109503	72.41597984
																			LDHLAEK	8	4501891	2xTMT6plex [N-Term; K7]	0.738	0.644		2.917098284	35.57	0	1283.772358	2	642.3897705	-0.072633107	-4.66E-05	48.3032588
5031839	KRT6A	keratin, type II cytoskeletal 6C	3853	NP_005545.1	60.63829787	1	4	41	1216	564	60.008	8	0.51	0.55	0.52	0.52	Significant	0.00052															
																			TAAENEFVTLK	11	5031839	2xTMT6plex [N-Term; K11]	0.643	0.824	0.939	5.086859226	59.78	0	1680.957258	2	840.9821167	-0.178988229	-0.000150436	68.33065447
																			QNLEPLFEQYINNLR	23	5031839	1xTMT6plex [N-Term]	0.683	0.724	0.849	3.930259228	42.93	0	2120.133769	3	707.3828125	0.054279594	3.84E-05	97.60946724
																			ISIGGGSCAISGGYGSR	65	5031839	1xTMT6plex [N-Term]; 1xCarbamidomethyl [C8	0.559	0.576	0.508	5.743015766	92.64	0	1827.922062	2	914.4562378	-9.225390081	-0.008431647	48.67605713
																			QCANLQAAIADAEQR	10	5031839	1xTMT6plex [N-Term]; 1xCarbamidomethyl [C2	0.653	0.443		5.274423599	79.59	0	1887.954425	2	944.480957	0.11253582	0.000106231	69.19360706
																			NLDLDSIIAEVK	45	5031839	2xTMT6plex [N-Term; K12]	0.705	0.96	0.55	4.597734928	71.71	0	1788.051886	2	894.5292969	-0.318284947	-0.000284555	97.09807644
																			ADTLTDEINFLR	46	5031839	1xTMT6plex [N-Term]	0.993	0.873	0.62	4.62463522	49.13	0	1636.874367	2	818.9413452	0.639651832	0.000523515	81.36577885
																			KQCANLQAAIADAEQR	10	5031839	2xTMT6plex [N-Term; K1]; 1xCarbamidomethyl [C3	0.452	0.459	0.573	5.826293468	34.6	1	2245.21232	3	749.0750732	-0.736439678	-0.000551154	60.32043363
																			ALYDAELSQMQTHISDTSVVLSMDNNR	12	5031839	1xTMT6plex [N-Term]; 1xOxidation [M]	0.01		0.608	5.389070034	42.91	0	3283.577221	3	1089.863525	-1.922962464	-0.002094479	88.89941277
																			ALYDAELSQMQTHISDTSVVLSMDNNR	22	5031839	1xTMT6plex [N-Term]	100		0.357	6.996612549	55.4	0	3267.582306	3	1089.863525	-1.922962464	-0.002094479	88.89941277
																			LALDVEIATYR	151	5031839	1xTMT6plex [N-Term]	0.753	0.754	0.751	4.44096899	56.01	0	1492.85726	2	746.9325562	0.385451914	0.000287712	110.5595349
																			AIGGGLSSVGGGSSTIK	68	5031839	2xTMT6plex [N-Term; K17]	0.348	0.529	0.509	5.736055374	121.69	0	1906.100962	2	953.5540161	-0.108081073	-0.000103007	47.03564509
																			GSGGLGGACGGAGFGSR	78	5031839	1xTMT6plex [N-Term]; 1xCarbamidomethyl [C9	0.559	0.55	0.585	6.163519859	124.59	0	1653.796468	2	827.4003906	-1.791810575	-0.001481645	35.93234501
																			WTLLQEQGTK	38	5031839	2xTMT6plex [N-Term; K10]	0.646	0.541	0.642	4.431287766	67.05	0	1661.962677	2	831.4860229	1.258667518	0.001045929	58.76221027
																			AEAESWYQTK	55	5031839	2xTMT6plex [N-Term; K10]	0.495	0.506	0.465	4.686650276	61.21	0	1670.879007	2	835.9353027	-9.383385141	-0.007839251	47.77075624
																			GMQDLVEDFK	38	5031839	2xTMT6plex [N-Term; K10]	0.507	0.611	0.517	3.552022934	60.89	0	1639.876565	2	820.4421387	0.265985721	0.000218092	90.125009
																			NKLEGLEDALQK	23	5031839	3xTMT6plex [N-Term; K2; K12]	0.557	0.564	0.429	4.601424217	31.03	1	2045.220966	3	682.4105835	-1.842833503	-0.001256334	67.44463759
																			TAAENEFVTLKK	13	5031839	3xTMT6plex [N-Term; K11; K12]	0.817	0.842	0.639	4.115298748	28.47	1	2038.215153	3	680.0773926	1.212764203	0.000823958	39.63700693
																			SGFSSVSVSR	21	5031839	1xTMT6plex [N-Term]	0.792	0.656	0.687	3.524495363	48.9	0	1241.668731	2	621.3381348	0.21068522	0.000130801	43.83221283
																			SLYGLGGSK	18	5031839	2xTMT6plex [N-Term; K9]	0.459	0.464	0.37	3.995541811	55.67	0	1339.798572	2	670.4026489	-0.411351713	-0.000275564	45.49490491
																			YEELQVTAGR	69	5031839	1xTMT6plex [N-Term]	0.595	0.586	0.605	3.652048111	59.59	0	1394.74771	2	697.8771973	-0.424398438	-0.000295964	40.86582826
																			LEGLEDALQK	14	5031839	2xTMT6plex [N-Term; K10]	1.078	1.133	0.832	3.599620104	50.22	0	1573.920144	2	787.4643555	0.819874823	0.000645209	80.44883768
																			KDVDAAYMNK	8	5031839	3xTMT6plex [N-Term; K1; K10]	0.673	0.672	0.658	4.060960293	58.88	1	1842.039831	2	921.5233154	-0.258773245	-0.000238335	40.85936967
																			ALYDAELSQMQTHISDTSVVLSMDNNR	3	5031839	1xTMT6plex [N-Term]; 2xOxidation [M10; M23]				2.384183168	10.46	0	3299.572136	3	1089.863525	-1.922962464	-0.002094479	88.89941277
																			EYQELMNVK	41	5031839	2xTMT6plex [N-Term; K9]	0.687	0.716	0.615	4.131864071	49.21	0	1611.88165	2	806.4436646	-0.991051941	-0.000798729	52.61948067
																			NTKQEIAEINR	10	5031839	2xTMT6plex [N-Term; K3]	0.169	0.126	0.124	3.367176533	31.79	1	1774.022317	2	887.5140991	-0.786820886	-0.000697919	32.68673791
																			DVDAAYMNK	31	5031839	2xTMT6plex [N-Term; K9]	0.741	0.76	0.707	4.151451111	53.84	0	1484.781936	2	742.8936768	-1.252072328	-0.000929527	41.62478759
																			AQYEEIAQR	12	5031839	1xTMT6plex [N-Term]	0.495	0.423	0.431	3.645548105	42.75	0	1336.705845	2	668.8570557	0.740363431	0.000494824	43.93391917
																			GMQDLVEDFK	11	5031839	2xTMT6plex [N-Term; K10]; 1xOxidation [M2	0.539	0.474	0.612	2.773050308	46.42	0	1655.87148	2	820.4421387	0.265985721	0.000218092	90.125009
																			LLKEYQELMNVK	8	5031839	3xTMT6plex [N-Term; K3; K12]	0.155	0.01	0.127	4.227711678	15.41	1	2195.307673	3	732.4400024	-1.010649126	-0.000739562	76.7615935
																			YTTTSSSSR	4	5031839	1xTMT6plex [N-Term]				2.833668947	22.41	0	1218.616361	2	609.8117676	-0.084377457	-5.14E-05	17.0386438
																			QLDSIVGER	24	5031839	1xTMT6plex [N-Term]	0.883	0.805	0.856	3.483621359	48.9	0	1245.700031	2	623.3537598	0.169769001	0.000105741	54.97838979
																			EYQELMNVK	24	5031839	2xTMT6plex [N-Term; K9]; 1xOxidation [M6]	0.61	0.666	0.646	2.821333647	41.8	0	1627.876565	2	806.4436646	-0.991051941	-0.000798729	52.61948067
																			DVDAAYMNK	24	5031839	2xTMT6plex [N-Term; K9]; 1xOxidation [M7]	0.68	0.723	0.752	3.092167854	44.75	0	1500.776851	2	742.8936768	-1.252072328	-0.000929527	41.62478759
																			KDVDAAYMNK	11	5031839	3xTMT6plex [N-Term; K1; K10]; 1xOxidation [M8	0.53	0.54	0.663	3.171750546	30.81	1	1858.034746	2	921.5233154	-0.258773245	-0.000238335	40.85936967
																			LLEGEECR	29	5031839	1xTMT6plex [N-Term]; 1xCarbamidomethyl [C7	0.83	0.873	0.841	3.377986431	36.73	0	1234.629903	2	617.817688	-1.461112705	-0.000901967	39.11707538
																			QNLEPLFEQYINNLRR	7	5031839	1xTMT6plex [N-Term]			0.01	2.52909255	26.92	1	2276.23488	3	759.4154663	-1.333102416	-0.001011485	89.70715979
																			KDVDAAYMNKVELQAK	2	5031839	4xTMT6plex [N-Term; K1; K10; K16]			0.553	3.064446211	10.06	2	2739.588488	4	685.6531982	0.903500201	0.000618805	62.72366777
																			NKYEDEINK	12	5031839	3xTMT6plex [N-Term; K2; K9]	0.918	0.83	0.912	3.587960243	28.5	1	1840.041939	2	920.526123	1.646768851	0.001515062	36.94344344
																			FASFIDK	41	5031839	2xTMT6plex [N-Term; K7]	0.677	0.84	0.69	3.288649321	38.52	0	1285.755645	2	643.3812866	-0.27093625	-0.000174179	75.39418814
																			QEIAEINR	10	5031839	1xTMT6plex [N-Term]	0.62	0.622	0.615	3.046356916	39.66	0	1201.673817	2	601.3400269	-0.865142477	-0.00051981	40.54304946
																			KLLEGEECR	24	5031839	2xTMT6plex [N-Term; K1]; 1xCarbamidomethyl [C8	0.579	0.555	0.552	3.393291712	31.56	1	1591.887798	2	796.4476318	0.118602501	9.44E-05	39.43144923
																			SEIDHVKK	11	5031839	3xTMT6plex [N-Term; K7; K8]	0.591	0.384	0.403	3.117077827	10.66	1	1643.009517	4	411.5080872	0.609620822	0.000250403	32.42606733
																			SEIDHVK	8	5031839	2xTMT6plex [N-Term; K7]	0.548	0.685	0.498	2.960561991	34.59	0	1285.751622	2	643.3790283	-0.654993827	-0.00042108	31.18781299
																			FLEQQNK	19	5031839	2xTMT6plex [N-Term; K7]	0.833	0.763	0.782	2.620012999	32.3	0	1364.793821	2	682.9000854	-0.679261254	-0.000463526	39.10866059
																			YEDEINKR	6	5031839	2xTMT6plex [N-Term; K7]	1.059	1.134	1.172	3.56978035	27.49	1	1524.842228	3	508.9517822	-0.940779502	-0.00047818	33.28155655
																			YEDEINK	4	5031839	2xTMT6plex [N-Term; K7]	1.202		0.999	3.350680113	32.52	0	1368.741117	2	684.8745117	0.46013632	0.000314904	42.23766616
																			GEMALKDAK	2	5031839	3xTMT6plex [N-Term; K6; K9]	0.295		0.417	2.261339903		1	1649.986339	3	550.6671753	0.384099487	0.000211253	42.54348205
568815719	CLTC	clathrin heavy chain 1 isoform 2	1213	NP_001275582.1	42.94222752	1	55	55	310	1679	191.936	5.69	0.95	1.09	1.14	1.06	Significant	0.0029															
																			GQCDLELINVCNENSLFK	9	568815719	2xTMT6plex [N-Term; K18]; 2xCarbamidomethyl [C3; C11	0.893	0.787	0.993	2.958172083	35.76	0	2611.326029	3	871.1134644	-0.072527747	-6.31E-05	83.14032048
																			VSQPIEGHAASFAQFK	6	568815719	2xTMT6plex [N-Term; K16]	1.146	100	100	4.85609293	49.53	0	2175.196259	3	725.7369385	0.00148478	1.08E-06	65.91981799
																			AFMTADLPNELIELLEK	12	568815719	2xTMT6plex [N-Term; K17]	1.906	1.723		3.479284286	36.09	0	2405.340205	2	1203.173462	-0.232076059	-0.000279111	112.3106787
																			AHTMTDDVTFWK	4	568815719	2xTMT6plex [N-Term; K12]	1.489	1.635		3.430215359	33.5	0	1909.98824	3	637.3339844	-0.439942499	-0.000280095	69.20000231
																			ALEHFTDLYDIK	10	568815719	2xTMT6plex [N-Term; K12]	0.867	0.851	0.715	4.171984196	57.32	0	1923.062785	2	962.0350952	0.066841129	6.43E-05	80.68549436
																			LAELEEFINGPNNAHIQQVGDR	2	568815719	1xTMT6plex [N-Term]				2.747021437	26.4	0	2693.384457	3	898.4654541	-0.983211032	-0.000882722	86.49787056
																			IHEGCEEPATHNALAK	4	568815719	2xTMT6plex [N-Term; K16]; 1xCarbamidomethyl [C5	1.065			6.930800915	59.23	0	2235.159222	3	745.7238159	-1.041203719	-0.000775752	37.41793627
																			SVDPTLALSVYLR	8	568815719	1xTMT6plex [N-Term]	1.009	100		3.38739562	32.27	0	1662.962788	2	831.9859619	1.118081618	0.000929664	88.93278891
																			RPISADSAIMNPASK	8	568815719	2xTMT6plex [N-Term; K15]	1.066	1.068	1.027	5.07395792	47.08	0	2016.131216	3	672.7142944	-1.431379662	-0.00096195	41.7803951
																			KVSQPIEGHAASFAQFK	4	568815719	3xTMT6plex [N-Term; K1; K17]	0.749	0.677		5.9925704	39.36	1	2532.454154	4	633.8692627	0.421326976	0.000266748	54.83280953
																			AVDVFFPPEAQNDFPVAMQISEK	5	568815719	2xTMT6plex [N-Term; K23]	1.261	2.112		3.38254714	39.99	0	3037.574515	3	1013.194946	-1.392417796	-0.001409858	101.5731129
																			KFDVNTSAVQVLIEHIGNLDR	4	568815719	2xTMT6plex [N-Term; K1]	100	1.115		3.317946911	11.83	1	2826.587798	4	707.4022827	-0.175674934	-0.00012414	93.4295086
																			FQSVPAQPGQTSPLLQYFGILLDQGQLNK	2	568815719	2xTMT6plex [N-Term; K29]		1.304		4.821471691	63.55	0	3646.004488	3	1216.006226	-0.100038875	-0.000121581	118.5890228
																			SVNESLNNLFITEEDYQALR	2	568815719	1xTMT6plex [N-Term]			100	3.749982357	39.4	0	2584.309227	3	862.1069946	-1.081903532	-0.000931991	87.02016561
																			HSSLAGCQIINYR	12	568815719	1xTMT6plex [N-Term]; 1xCarbamidomethyl [C7	1.215	1.288	1.208	5.832695961	63.63	0	1747.911104	3	583.3078613	-1.185990306	-0.000691002	40.60737937
																			RPISADSAIMNPASK	4	568815719	2xTMT6plex [N-Term; K15]; 1xOxidation [M10		0.996	1.205	4.798737526	22.28	0	2032.126131	3	672.7142944	-1.431379662	-0.00096195	41.7803951
																			DAMQYASESK	6	568815719	2xTMT6plex [N-Term; K10]	1.274	1.334	1.193	4.064490795	62.22	0	1587.808879	2	794.4083862	0.388548613	0.00030847	48.71930367
																			LHIIEVGTPPTGNQPFPK	6	568815719	2xTMT6plex [N-Term; K18]		1.207	0.961	2.583208799	24.39	0	2403.380037	3	801.7971802	-1.268876586	-0.001016531	73.6261309
																			AFMTADLPNELIELLEK	2	568815719	2xTMT6plex [N-Term; K17]; 1xOxidation [M3		0.753		2.917035341	35.63	0	2421.33512	2	1203.173462	-0.232076059	-0.000279111	112.3106787
																			GQFSTDELVAEVEKR	4	568815719	2xTMT6plex [N-Term; K14]		0.447		3.873944283	26.01	1	2166.180668	3	722.7320557	0.436418901	0.000315121	75.7750118
																			KFNALFAQGNYSEAAK	4	568815719	3xTMT6plex [N-Term; K1; K16]		0.776		3.65437746	14.81	1	2446.369756	3	816.126709	-1.709433689	-0.001393969	71.38872209
																			GQFSTDELVAEVEK	6	568815719	2xTMT6plex [N-Term; K14]	100	100		2.731832743	55.39	0	2010.079557	2	1005.543457	0.039810613	4.00E-05	77.08323799
																			IYIDSNNNPER	4	568815719	1xTMT6plex [N-Term]	1.044	1.012		4.031416893	43.11	0	1563.796451	2	782.4006958	-1.493716421	-0.001167934	41.00881092
																			LECSEELGDLVK	4	568815719	2xTMT6plex [N-Term; K12]; 1xCarbamidomethyl [C3		0.791		2.00481534	16.59	0	1849.998136	2	925.5022583	-0.48454559	-0.000448204	80.29787862
																			RDPHLACVAYER	6	568815719	1xTMT6plex [N-Term]; 1xCarbamidomethyl [C7	0.665	0.82	1.208	4.855773926	24.73	1	1715.884889	3	572.633606	0.801686117	0.000458534	32.18612165
																			AHIAQLCEK	4	568815719	2xTMT6plex [N-Term; K9]; 1xCarbamidomethyl [C7	0.676			3.894343615	53.56	0	1527.871754	3	509.9631042	1.966895541	0.001001721	37.76019766
																			VVGAMQLYSVDR	10	568815719	1xTMT6plex [N-Term]	100	100		2.352637529	37.57	0	1566.851129	2	783.9286499	-0.705769228	-0.000552918	63.16095384
																			IVLDNSVFSEHR	6	568815719	1xTMT6plex [N-Term]	1.179	0.99	1.053	3.601362705	38.9	0	1644.890686	3	548.9680176	-0.721078692	-0.000395365	51.1673052
																			NNLAGAEELFAR	9	568815719	1xTMT6plex [N-Term]	3.183			3.353827238	48.17	0	1533.822272	2	767.4089355	-7.613161228	-0.005838618	61.78839158
																			FDFVHDLVLYLYR	6	568815719	1xTMT6plex [N-Term]	100	2.544	1.966	3.11808157	14.37	0	1929.047186	3	643.6889648	2.672356222	0.001718367	110.5445132
																			EVCFACVDGK	7	568815719	2xTMT6plex [N-Term; K10]; 2xCarbamidomethyl [C3; C6	0.55	0.965	0.91	2.465162039	22.49	0	1642.83332	2	821.9204712	0.210363893	0.000172796	52.45193864
																			LASTLVHLGEYQAAVDGAR	4	568815719	1xTMT6plex [N-Term]	100	0.34		2.969526052	20.08	0	2200.192347	3	734.0687256	-0.328654078	-0.000241034	74.19849156
																			FQEHLQLQNLGINPANIGFSTLTMESDK	2	568815719	2xTMT6plex [N-Term; K28]			100	2.153808355	2.61	0	3603.888138	3	1201.96521	-1.959333064	-0.002353739	90.28685337
																			TLQIFNIEMK	6	568815719	2xTMT6plex [N-Term; K10]	100	100	100	2.093868971	31.28	0	1694.991535	2	847.9981079	-1.531293599	-0.001297765	79.06245256
																			HNIMDFAMPYFIQVMK	2	568815719	2xTMT6plex [N-Term; K16]				1.98149693	6.27	0	2443.274057	3	815.0959473	-0.314538241	-0.000256168	107.7099912
																			LLYNNVSNFGR	6	568815719	1xTMT6plex [N-Term]	0.946	0.914	100	2.961693764	32.68	0	1525.832443	2	763.418457	-1.838385016	-0.001402534	56.58475653
																			ADDPSSYMEVVQAANTSGNWEELVK	5	568815719	2xTMT6plex [N-Term; K25]			1.198	1.700500607	3.99	0	3198.566529	3	1066.859253	-1.039072005	-0.001107847	100.4514911
																			EAIDSYIK	12	568815719	2xTMT6plex [N-Term; K8]	1.009	0.943	0.859	3.916736603	45.04	0	1396.808803	2	698.9078979	-0.202870688	-0.000141686	56.96686735

Bhat et al,2017. Cigarette smoke and chewing tobacco alter expression of different sets of miRNAs in oral keratinocytes

Supplementary Table S2(A). List of all proteins and peptides identified in OKF6/TERT1 cells chronically treated with chewing tobacco using Sequest and Mascot search algorithms

Accession	Gene_Symbol	Description	Gene_ID	Refseq_Accession	Coverage	Proteins	#Unique_Peptides	#Peptides	#PSMS	#AAs	MW [kDa]	calc. pI	OKF6-STE-6M/OKF6P_R1	OKF6-STE-6M/OKF6P_R2	OKF6-STE-6M/OKF6P_R3	Median fold change	Status	P-value (6M)	Sequence	#PSMs	Protein Accessions	Modifications	OKF6-STE-6M/OKF6P_R1	OKF6-STE-6M/OKF6P_R2	OKF6-STE-6M/OKF6P_R3	Xcorr	Ionscore	# Missed Cleavages	Theo. MH+ [Da]	Charge	m_z	DeltaM [ppm]	Deltam/z [Da]	RT [min]
																			ENPYYDSR	4	568815719	1xTMT6plex [N-Term]		0.929	0.883	3.271442652	46.5	0	1272.605797	2	636.8059692	-0.891747814	-0.000567422	29.27803637
																			ESNCYDPER	2	568815719	1xTMT6plex [N-Term]; 1xCarbamidomethyl [C4	0.794			2.728656292	38.14	0	1398.61571	2	699.8101807	-1.876842836	-0.001312491	31.47494867
																			VIQCFAETGQVQK	6	568815719	2xTMT6plex [N-Term; K13]; 1xCarbamidomethyl [C4	100	0.798	0.655	1.835151911	19.75	0	1966.083203	2	983.5444336	-0.820342953	-0.000806431	55.14069714
																			ISGETIFVTAPHEATAGIIGVNR	4	568815719	1xTMT6plex [N-Term]		100		2.748379707	12.29	0	2582.413967	3	861.4755859	-0.682287009	-0.000587316	67.55849162
																			EHLELFWSR	10	568815719	1xTMT6plex [N-Term]	1.192	0.927	0.744	2.980496407	27.52	0	1445.773865	3	482.5970459	1.880913315	0.000906458	64.40635868
																			YIEIYVQK	6	568815719	2xTMT6plex [N-Term; K8]	100	100		2.439537048	25.1	0	1513.903037	2	757.4550171	-0.184833708	-0.00013991	69.90465821
																			AMLSANIR	4	568815719	1xTMT6plex [N-Term]	1.272	1.248		2.880108118	53.11	0	1104.63968	2	552.8227539	-1.311493264	-0.000724364	46.57086312
																			LEKHELIEFR	3	568815719	2xTMT6plex [N-Term; K3]	0.498		0.691	3.251455784	9.88	1	1772.047076	3	591.3545532	1.145961638	0.000676899	52.59503575
																			ESYVETELIFALAK	2	568815719	2xTMT6plex [N-Term; K14]				1.744463682	4.99	0	2071.17273	2	1036.089111	-0.861112994	-0.000891757	103.6915038
																			YHEQLSTQSLIELFESFK	2	568815719	2xTMT6plex [N-Term; K18]				1.713835478	1.49	0	2657.422689	3	886.4782104	-0.982633419	-0.000870424	106.3835731
																			ADDPSSYMEVVQAANTSGNWEELVK	2	568815719	2xTMT6plex [N-Term; K25]; 1xOxidation [M8				1.615242243	1.78	0	3214.561444	3	1066.859253	-1.039072005	-0.001107847	100.4514911
																			LDASESLR	6	568815719	1xTMT6plex [N-Term]	1.413	1.373	1.501	2.710187912	42.3	0	1119.620719	2	560.3132935	-1.257779455	-0.000704118	37.25731183
																			QLPLVKPYLR	3	568815719	2xTMT6plex [N-Term; K6]	0.64			2.72202158	12.58	0	1685.087818	3	562.3674316	-0.04553954	-2.56E-05	70.59545036
																			LLLPWLEAR	6	568815719	1xTMT6plex [N-Term]	1.107	0.513	0.948	2.581824303	30.28	0	1339.829923	2	670.4174194	-1.762315329	-0.001180601	94.02413426
																			IAAYLFK	10	568815719	2xTMT6plex [N-Term; K7]	1.252	1.151	0.781	3.339648485	33.53	0	1283.812766	2	642.4088135	-1.881603719	-0.001207813	88.30512667
																			QSVELCK	4	568815719	2xTMT6plex [N-Term; K7]; 1xCarbamidomethyl [C6	1.095	1.105		3.134954214	30.74	0	1321.754993	2	661.3813477	0.321831582	0.000212691	38.3904183
																			NLILVVR	8	568815719	1xTMT6plex [N-Term]	0.372	100	100	1.9125067	33.06	0	1055.713831	2	528.3609619	0.772972846	0.000408019	59.49247125
																			AYEFAER	4	568815719	1xTMT6plex [N-Term]	0.937	0.802		2.853046894	31.76	0	1114.57304	2	557.7900391	-0.214086463	-0.000119308	43.12361587
																			VMEYINR	3	568815719	1xTMT6plex [N-Term]	1.171	1.184		2.276413679	31.73	0	1153.623695	2	577.3154297	-0.097722507	-5.64E-05	59.35714191
																			NLQNLLILTAIK	2	568815719	2xTMT6plex [N-Term; K12]				1.825882554	6.27	0	1812.172276	2	906.5881958	-1.744435934	-0.001580609	103.9639666
																			AMLSANIR	2	568815719	1xTMT6plex [N-Term]; 1xOxidation [M2]	1.127	0.979		1.783747554	16.55	0	1120.634595	2	552.8227539	-1.311493264	-0.000724364	46.57086312
119703753	KRT6B	keratin, type II cytoskeletal 6B	3854	NP_0055462	59.04255319	1	3	40	1009	564	60.03	8	0.21	0.19	0.19	0.2	Significant	0.0011																
																			TAAENEFVTLK	11	119703753	2xTMT6plex [N-Term; K11]	0.643	0.824	0.939	5.086859226	59.78	0	1680.957258	2	840.9821167	-0.178988229	-0.000150436	68.33065447
																			QNLEPLFEQYINNLR	23	119703753	1xTMT6plex [N-Term]	0.683	0.724	0.849	3.930259228	42.93	0	2120.133769	3	707.3828125	0.054279594	3.84E-05	97.60946724
																			ISIGGGSCAISGGYGSR	65	119703753	1xTMT6plex [N-Term]; 1xCarbamidomethyl [C8	0.559	0.576	0.508	5.743015766	92.64	0	1827.922062	2	914.4562378	-9.225390081	-0.008431647	48.67605713
																			QCANLQAAIADAEQR	10	119703753	1xTMT6plex [N-Term]; 1xCarbamidomethyl [C2	0.653	0.443		5.274423599	79.59	0	1887.954425	2	944.480957	0.11253582	0.000106231	69.19360706
																			ATGGGLSSVGGGSSTIK	8	119703753	2xTMT6plex [N-Term; K17]	0.171	0.17	0.181	5.424235344	97.86	0	1894.064576	2	947.5355835	-0.362040355	-0.000342864	47.55115455
																			NLDLDSIIAEVK	45	119703753	2xTMT6plex [N-Term; K12]	0.705	0.96	0.55	4.597734928	71.71	0	1788.051886	2	894.5292969	-0.318284947	-0.000284555	97.09807644
																			ADTLTDEINFLR	46	119703753	1xTMT6plex [N-Term]	0.993	0.873	0.62	4.62463522	49.13	0	1636.874367	2	818.9413452	0.639651832	0.000523515	81.36577885
																			KQCANLQAAIADAEQR	10	119703753	2xTMT6plex [N-Term; K1]; 1xCarbamidomethyl [C3	0.452	0.459	0.573	5.826293468	34.6	1	2245.21232	3	749.0750732	-0.736439678	-0.000551154	60.32043363
																			ALYDAELSQMQTHISDTSVVLSMDNNR	12	119703753	1xTMT6plex [N-Term]; 1xOxidation [M	0.01		0.608	5.389070034	42.91	0	3283.577221	3	1089.863525	-1.922962464	-0.002094479	88.89941277
																			ALYDAELSQMQTHISDTSVVLSMDNNR	22	119703753	1xTMT6plex [N-Term]	100		0.357	6.996612549	55.4	0	3267.582306	3	1089.863525	-1.922962464	-0.002094479	88.89941277
																			LALDVEIATYR	151	119703753	1xTMT6plex [N-Term]	0.753	0.754	0.751	4.44096899	56.01	0	1492.85726	2	746.9325562	0.385451914	0.000287712	110.5595349
																			GSGGLGGACGGAGFGSR	78	119703753	1xTMT6plex [N-Term]; 1xCarbamidomethyl [C9	0.559	0.55	0.585	6.163519859	124.59	0	1653.796468	2	827.4003906	-1.791810575	-0.001481645	35.93234501
																			WTLLQEQGTK	38	119703753	2xTMT6plex [N-Term; K10]	0.646	0.541	0.642	4.431287766	67.05	0	1661.962677	2	831.4860229	1.258667518	0.001045929	58.76221027
																			AEAESWYQTK	55	119703753	2xTMT6plex [N-Term; K10]	0.495	0.506	0.465	4.686650276	61.21	0	1670.879007	2	835.9353027	-9.383385141	-0.007839251	47.77075624
																			NKLEGLEDALQK	23	119703753	3xTMT6plex [N-Term; K2; K12]	0.557	0.564	0.429	4.601424217	31.03	1	2045.220966	3	682.4105835	-1.842833503	-0.001256334	67.44463759
																			TAAENEFVTLKK	13	119703753	3xTMT6plex [N-Term; K11; K12]	0.817	0.842	0.639	4.115298748	28.47	1	2038.215153	3	680.0773926	1.212764203	0.000823958	39.63700693
																			SLYGLGGSK	18	119703753	2xTMT6plex [N-Term; K9]	0.459	0.464	0.37	3.995541811	55.67	0	1339.798572	2	670.4026489	-0.411351713	-0.000275564	45.49490491
																			LEGLEDALQK	14	119703753	2xTMT6plex [N-Term; K10]	1.078	1.133	0.832	3.599620104	50.22	0	1573.920144	2	787.4643555	0.819874823	0.000645209	80.44883768
																			SGFSSISVSR	6	119703753	1xTMT6plex [N-Term]	0.217	0.25		3.528537512	46.38	0	1255.684381	2	628.3452759	-0.880979491	-0.000553116	48.36295836
																			KDVDAAYMNK	8	119703753	3xTMT6plex [N-Term; K1; K10]	0.673	0.672	0.658	4.060960293	58.88	1	1842.039831	2	921.5233154	-0.258773245	-0.000238335	40.85936967
																			ALYDAELSQMQTHISDTSVVLSMDNNR	3	119703753	1xTMT6plex [N-Term]; 2xOxidation [M10; M23]				2.384183168	10.46	0	3299.572136	3	1089.863525	-1.922962464	-0.002094479	88.89941277
																			EYQELMNVK	41	119703753	2xTMT6plex [N-Term; K9]	0.687	0.716	0.615	4.131864071	49.21	0	1611.88165	2	806.4436646	-0.991051941	-0.000798729	52.61948067
																			NTKQEIAEINR	10	119703753	2xTMT6plex [N-Term; K3]	0.169	0.126	0.124	3.367176533	31.79	1	1774.022317	2	887.5140991	-0.786820886	-0.000697919	32.6863791
																			DVDAAYMNK	31	119703753	2xTMT6plex [N-Term; K9]	0.741	0.76	0.707	4.151451111	53.84	0	1484.781936	2	742.8936768	-1.252072328	-0.000929527	41.62478759
																			AQYEEIAQR	12	119703753	1xTMT6plex [N-Term]	0.495	0.423	0.431	3.645548105	42.75	0	1336.705845	2	668.8570557	0.740363431	0.000494824	43.93391917
																			YEELQITAGR	6	119703753	1xTMT6plex [N-Term]	0.21	0.186	0.202	3.774420261	44.97	0	1408.76336	2	704.8848267	-0.697916851	-0.0004916	47.08808655
																			LLKEYQELMNVK	8	119703753	3xTMT6plex [N-Term; K3; K12]	0.155	0.01	0.127	4.227711678	15.41	1	2195.307673	3	732.4400024	-1.010649126	-0.000739562	76.7615935
																			YTTTSSSSR	4	119703753	1xTMT6plex [N-Term]				2.833668947	22.41	0	1218.616361	2	609.8117676	-0.084377457	-5.14E-05	17.0386438
																			EYQELMNVK	24	119703753	2xTMT6plex [N-Term; K9]; 1xOxidation [M6]	0.61	0.666	0.646	2.821333647	41.8	0	1627.876565	2	806.4436646	-0.991051941	-0.000798729	52.61948067
																			DVDAAYMNK	24	119703753	2xTMT6plex [N-Term; K9]; 1xOxidation [M7]	0.68	0.723	0.752	3.092167854	44.75	0	1500.776851	2	742.8936768	-1.252072328	-0.000929527	41.62478759
																			NMQDLVEDLK	4	119703753	2xTMT6plex [N-Term; K10]	0.01		0.01	2.339784384	25.87	0	1662.913678	2	831.9593506	-1.355294718	-0.001126869	74.2662738
																			KDVDAAYMNK	11	119703753	3xTMT6plex [N-Term; K1; K10]; 1xOxidation [M8	0.53	0.54	0.663	3.171750546	30.81	1	1858.034746	2	921.5233154	-0.258773245	-0.000238335	40.85936967
																			LLEGEECR	29	119703753	1xTMT6plex [N-Term]; 1xCarbamidomethyl [C7	0.83	0.873	0.841	3.377986431	36.73	0	1234.629903	2	617.817688	-1.461112705	-0.000901967	39.11707538
																			QNLEPLFEQYINNLRR	7	119703753	1xTMT6plex [N-Term]			0.01	2.52909255	26.92	1	2276.23488	3	759.4154663	-1.333102416	-0.001011485	89.70715979
																			KDVDAAYMNKVELQAK	2	119703753	4xTMT6plex [N-Term; K1; K10; K16]			0.553	3.064446211	10.06	2	2739.588488	4	685.6531982	0.903500201	0.000618805	62.72366777
																			NKYEDEINK	12	119703753	3xTMT6plex [N-Term; K2; K9]	0.918	0.83	0.912	3.587960243	28.5	1	1840.041939	2	920.526123	1.646768851	0.001515062	36.94344344
																			FASFIDK	41	119703753	2xTMT6plex [N-Term; K7]	0.677	0.84	0.69	3.288649321	38.52	0	1285.755645	2	643.3812866	-0.27093625	-0.000174179	75.39418814
																			QEIAEINR	10	119703753	1xTMT6plex [N-Term]	0.62	0.622	0.615	3.046356916	39.66	0	1201.673817	2	601.3400269	-0.865142477	-0.00051981	40.54304946
																			KLLEGEECR	24	119703753	2xTMT6plex [N-Term; K1]; 1xCarbamidomethyl [C8	0.579	0.555	0.552	3.393291712	31.56	1	1591.887798	2	796.4476318	0.118602501	9.44E-05	39.43144923
																			SEIDHVKK	11	119703753	3xTMT6plex [N-Term; K7; K8]	0.591	0.384	0.403	3.117077827	10.66	1	1643.009517	4	411.5080872	0.609620822	0.000250403	32.42606733
																			SEIDHVK	8	119703753	2xTMT6plex [N-Term; K7]	0.548	0.685	0.498	2.960561991	34.59	0	1285.751622	2	643.3790283	-0.654993827	-0.00042108	31.18781299
																			FLEQQNK	19	119703753	2xTMT6plex [N-Term; K7]	0.833	0.763	0.782	2.620012999	32.3	0	1364.793821	2	682.9000854	-0.679261254	-0.000463526	39.10866059
																			YEDEINKR	6	119703753	2xTMT6plex [N-Term; K7]	1.059	1.134	1.172	3.56978035	27.49	1	1524.842228	3	508.9517822	-0.940779502	-0.00047818	33.28155655
																			YEDEINK	4	119703753	2xTMT6plex [N-Term; K7]	1.202		0.999	3.350680113	32.52	0	1368.741117	2	684.8745117	0.46013632	0.000314904	42.23766616
																			GEMALKDAK	2	119703753	3xTMT6plex [N-Term; K6; K9]	0.295		0.417	2.261339903		1	1649.986339	3	550.6671753	0.384099487	0.000211253	42.54348205
119395754	KRT5	keratin, type II cytoskeletal 5	3852	NP_0004152	58.13559322	1	26	41	1208	590	62.34	7.74	0.78	0.81	0.8	0.8	Significant	0.00012																
																			TEAESWYQTK	27	119395754	2xTMT6plex [N-Term; K10]	0.938	1.3	0.89	4.612578392	66.51	0	1700.889572	2	850.942749	-6.673321603	-0.005675292	44.16413663
																			QCANLQNAIADAEQR	20	119395754	1xTMT6plex [N-Term]; 1xCarbamidomethyl [C2	1.047	1.066	1.371	3.990529299	49.87	0	1930.960239	2	965.9850464	1.334850602	0.001288772	58.51220839
																			QNLEPLFEQYINNLR	23	119395754	1xTMT6plex [N-Term]	0.683	0.724	0.849	3.930259228	42.93	0	2120.133769	3	707.3828125	0.054279594	3.84E-05	97.60946724
																			MFFDAELSQMQTHVSDTSVVLSMDNNR	48	119395754	1xTMT6plex [N-Term]	3.183	1.492		4.69660759	40.7	0	3331.559462	3	1116.522827	-0.134206838	-0.000149755	91.90781316
																			MFFDAELSQMQTHVSDTSVVLSMDNNR	4	119395754	1xTMT6plex [N-Term]; 2xOxidation [M			0.681	3.533763647	33.13	0	3363.549292	3	1116.522827	-0.134206838	-0.000149755	91.90781316
																			MFFDAELSQMQTHVSDTSVVLSMDNNR	13	119395754	1xTMT6plex [N-Term]; 1xOxidation [M			0.562	6.148090839	43.73	0	3347.554377	3	1116.522827	-0.134206838	-0.000149755	91.90781316
																			NLDLDSIIAEVK	45	119395754	2xTMT6plex [N-Term; K12]	0.705	0.96	0.55	4.597734928	71.71	0	1788.051886	2	894.5292969	-0.318284947	-0.000284555	97.09807644
																			LALDVEIATYR	151	119395754	1xTMT6plex [N-Term]	0.753	0.754	0.751	4.44096899	56.01	0	1492.85726	2	746.9325562	0.385451914	0.000287712	110.5595349
																			SGGGGGGGFGR	4	119395754	1xTMT6plex [N-Term]	1.462	2.61		3.260510206	69.42	0	1094.554036	2	547.7811279	0.861756792	0.00047162	20.64774743
																			ISISTSGGSFR	106	119395754	1xTMT6plex [N-Term]	0.785	0.837	0.852	4.332402229	61.01	0	1340.737145	2	670.8684082	-5.672546462	-0.003802697	45.97770092
																			VSLAGACGVGGYGSR	72	119395754	1xTMT6plex [N-Term]; 1xCarbamidomethyl [C7	0.816	0.858	0.838	5.349248409	70.06	0	1639.842355	2	820.425354	0.656092221	0.000537944	45.63866007
																			KQCANLQNAIADAEQR	12	119395754	2xTMT6plex [N-Term; K1]; 1xCarbamidomethyl [C3	0.717	0.682		5.892766953	48.74	1	2288.218134	2	1144.613281	0.503583982	0.000576155	54.76146205
																			SFSTASAITPSVSR	45	119395754	1xTMT6plex [N-Term]	0.836	0.807	0.84	5.209585667	73.09	0	1639.885266	2	820.4468994	0.766150018	0.000628199	52.46069158
																			GLGVGFGSGGGSSSSVK	66	119395754	2xTMT6plex [N-Term; K17]	0.81	0.808	0.819	5.626618862	118.67	0	1898.038361	2	949.5222168	-0.634553165	-0.000602203	58.69295824
																			LAELEEALQK	33	119395754	2xTMT6plex [N-Term; K10]	1.1	1.039	1.006	3.848426104	51.36	0	1601.951444	2	801.4783325	-1.283185545	-0.0010278	86.0671235
																			WTLLQEQGTK	38	119395754	2xTMT6plex [N-Term; K10]	0.646	0.541	0.642	4.431287766	67.05	0	1661.962677	2	831.4860229	1.258667518	0.001045929	58.76221027
																			VDALMDEINFMK	21	119395754	2xTMT6plex [N-Term; K12]	1.247	1.022	1.547	3.121954203	39.26	0	1884.001113	2	942.5032959	-0.954279227	-0.000898932	103.3961699
																			DVDAAYMNKVELEAK	10	119395754	3xTMT6plex [N-Term; K9; K15]	0.447	0.01		3.869705677	22.52	1	2383.314608	3	795.1090088	-0.895963464	-0.000711788	73.73636773
																			SLYNLGGSK	22	119395754	2xTMT6plex [N-Term; K9]	0.7	0.612	0.673	4.326107979	62.88	0	1396.820036	2	698.9089355	-6.759336211	-0.004720788	48.18772199
																			KDVDAAYMNK	8	119395754	3xTMT6plex [N-Term; K1; K10]	0.673	0.672	0.658	4.060960293	58.88	1	1842.039831	2	921.5233154	-0.258773245	-0.000238335	40.85936967
																			NMQDLVEDFK	18	119395754	2xTMT6plex [N-Term; K10]	1.322	1.049	1.014	4.042321682	41.96	0	1696.898028	2	848.9515991	-1.24144632	-0.001053304	84.18821364
																			TTAENEFVMLK	9	119395754	2xTMT6plex [N-Term; K11]	2.407	1.089	0.767	3.58686161	31.15	0	1740.960628	2	870.9838867	-0.075620607	-6.58E-05	65.02124409
																			YEELQQTAGR	27	119395754	1xTMT6plex [N-Term]	0.947	1.113	0.87	3.611407518	48.2	0	1423.737873	2	712.3717651	-1.137678917	-0.000809878	37.85580375
																			FVSTTSSSR	18	119395754	1xTMT6plex [N-Term]	0.673	0.932	0.882	3.37026906	48.55	0	1200.642182	2	600.8253784	1.081084737	0.000648998	33.51292516
																			EYQELMNTK	28	119395754	2xTMT6plex [N-Term; K9]	0.935	1.255	0.951	4.530275822	44.13	0	1613.860914	2	807.4336548	-0.546224076	-0.000440765	48.14980384
																			TTAENEFVMLKK	7	119395754	3xTMT6plex [N-Term; K11; K12]	1.85			3.496758938	19.99	1	2098.218523	3	700.0782471	0.793306759	0.000554844	61.00889692
																			DVDAAYMNK	31	119395754	2xTMT6plex [N-Term; K9]	0.741	0.76	0.707	4.151451111	53.84	0	1484.781936	2	742.8936768	-1.252072328	-0.000929527	41.62478759
																			AQYEEIANR	14	119395754	1xTMT6plex [N-Term]	0.761	0.791	0.669	3.545786142	50.72	0	1322.690195	2	661.843811	-7.446603691	-0.004924775	40.07906626
																			VDALMDEINFMK	21	119395754	2xTMT6plex [N-Term; K12]; 1xOxidation [M	1.099	100	100	1.950728893	35.74	0	1899.996028	2	942.5032959	-0.954279227	-0.000898932	103.3961699
																			QLDSIVGER	24	119395754	1xTMT6plex [N-Term]	0.883	0.805	0.856	3.483621359	48.9	0	1245.700031	2	623.3537598	0.169769001	0.000105741	54.97838979
																			NTKHEISEMNR	3	119395754	2xTMT6plex [N-Term; K3]		0.157	0.417	3.683115005	13.55	1	1816.973987	3	606.3300781	0.932349617	0.000564685	28.50058688
																			DVDAAYMNK	24	119395754	2xTMT6plex [N-Term; K9]; 1xOxidation [M7]	0.68	0.723	0.752	3.092167854	44.75	0	1500.776851	2	742.8936768	-1.252072328	-0.000929527	41.62478759
																			EYQELMNTK	17	119395754	2xTMT6plex [N-Term; K9]; 1xOxidation [M6]	0.817	0.983	0.946	2.847823381	40.28	0	1629.855829	2	807.4336548	-0.546224076	-0.000440765	48.14980384
																			KDVDAAYMNK	11	119395754	3xTMT6plex [N-Term; K1; K10]; 1xOxidation [M8	0.53	0.54	0.663	3.171750546	30.81	1	1858.034746	2	921.5233154	-0.258773245	-0.000238335	40.85936967
																			LLEGEECR	29	119395754	1xTMT6plex [N-Term]; 1xCarbamidomethyl [C7	0.83	0.873	0.841	3.377986431	36.73	0	1234.629903	2	617.817688	-1.461112705	-0.000901967	39.11707538
																			QNLEPLFEQYINNLRR	7	119395754	1xTMT6plex [N-Term]			0.01	2.52909255	26.92	1	2276.23488	3	759.4154663	-1.333102416	-0.001011485	89.70715979
																			HEISEMNR	6	119395754	1xTMT6plex [N-Term]	1.29	1.38		3.080938101	30.94	0	1244.625486	2	622.8166504	0.432130995	0.000268921	27.0723246
																			DVDAAYMNKVELEAKVDALMDEINFMK	2	119395754	4xTMT6plex [N-Term; K9; K15; K27]		0.451		3.22262454	1.68	2	4019.134948	4	1005.537048	-2.13596725	-0.002146185	106.4775329
																			NKYEDEINK	12	119395754	3xTMT6plex [N-Term; K2; K9]	0.918	0.83	0.912	3.587960243	28.5	1	1840.041939	2	920.526123	1.646768851	0.001515062	36.94344344
																			FASFIDK	41	119395754	2xTMT6plex [N-Term; K7]	0.677	0.84	0.69	3.288649321	38.52	0	1285.755645	2	643.3812866	-0.27093625	-0.000174179	75.39418814
																			KLLEGEECR	24	119395754	2xTMT6plex [N-Term; K1]; 1xCarbamidomethyl [C8	0.579	0.555	0.552	3.393291712	31.56	1	1591.887798	2	796.4476318	0.118602501	9.44E-05	39.43144923
																			TSFTSVSR	6	119395754	1xTMT6plex [N-Term]	0.816	0.8	0.758	3.207475901	34.91	0	1113.610154	2	557.3086548	-0.108583499	-6.05E-05	34.46596221
																			AEIDNVK	19	119395754	2xTMT6plex [N-Term; K7]	0.896	0.895	0.858	3.386349678	46.49	0	1246.740723	2	623.8738403	-0.255952125	-0.000159553	41.61348958
																			FLEQQNK	19	119395754	2xTMT6plex [N-Term; K7]	0.833	0.763	0.782	2.620012999	32.3	0	1364.793821	2	682.9000854	-0.679261254	-0.000463526	39.10866059
																			YEDEINKR	6	119395754	2xTMT6plex [N-Term; K7]	1.059	1.134	1.172	3.56978035	27.49	1	1524.842228	3	508.9517822	-0.940779502	-0.00047818	33.28155655
																			AEIDNVKK	6	119395754	3xTMT6plex [N-Term; K7; K8]	0.618	0.587		2.020307302	6.71	1	1603.998618	3	535.336853	-1.628603628	-0.000870759	44.36566315
																			YEDEINK	4	119395754	2xTMT6plex [N-Term; K7]	1.202		0.999	3.350680113	32.52	0	1368.741117	2	684.8745117	0.46013632	0.000314904	42.23766616
																			NMQDLVEDFK	3	119395754	2xTMT6plex [N-Term; K10]; 1xOxidation [M2				1.261620879	8.85	0	1712.892943	2	848.9515991	-1.24144632	-0.001053304	84.18821364
																			TTAENEFVMLK	4	119395754	2xTMT6plex [N-Term; K11]; 1xOxidation [M9		100		1.47871995	6.29	0	1756.955543	2	870.9838867	-0.075620607	-6.58E-05	65.02124409
153792590	HSP90AA1	heat shock protein HSP 90-alpha isoform 1	3320	NP_001017963.2	49.29742389	1	24	41	463	854	98.099	5.16	1.09	1.16	1.27	1.18	Significant	0.002																
																			ADLINNLGTIAK	22	153792590	2xTMT6plex [N-Term; K12]	1.397	1.655	1.929	4.478831291	79.53	0	1701.031091	2	851.0209351	2.058873118	0.001751104	74.74577045
																			HLEINPDHSIIETLR	12	153792590	1xTMT6plex [N-Term]	1.902	2.75	2.238	5.235696793	41.33	0	2016.107554	4	504.7833557	2.003128034	0.00100963	58.5764621
																			ELHINLIPNKQDR	13	153792590	2xTMT6plex [N-Term; K10]	0.567	0.625	0.692	5.782775879	18.52	1	2048.201679	3	683.4053955	-0.022188519	-1.51E-05	48.2501972
																			VFIMDNCEELIPEYLNFIR	8	153792590	1xTMT6plex [N-Term]; 1xCarbamidomethyl [C7	100		1.841	4.278920174	27.31	0	2644.335248	3	882.1164551	-0.164720273	-0.000145192	107.7336055
																			KHLEINPDHSIIETLR	14	153792590	2xTMT6plex [N-Term; K1]	1.396	1.275	1.34	5.359332085	44.33	1	2373.365449	3	791.7935181	0.232386976	0.000183846	53.79342934
																			DQVANSAFVER	14	153792590	1xTMT6plex [N-Term]	1.821	1.734	1.596	4.723406792	58.39	0	1464.764422	2	732.8825073	-4.563513053	-0.003342236	38.50791032
																			ELISNSSDALDK	22	153792590	2xTMT6plex [N-Term; K12]	1.778	1.628	1.844	4.555279732	69.22	0	1749.963465	2	875.4871216	2.000844093	0.001750702	55.03166437
																			NPDDITNEEYGEFYK	11	153792590	2xTMT6plex [N-Term; K15]	1.668	100	1.215	4.148765564	46	0	2292.107229	3	764.7062378	-1.338711844	-0.001022824	65.34435738
																			KHGLEVIYMIEPIDEYCVQQLK	7	153792590	3xTMT6plex [N-Term; K1; K22]; 1xCarbamidomethyl [C17	100	1.444	1.881	4.913609028	31.78	1	3392.856517	4	848.9691772	-0.482607642	-0.000409409	91.535291
																			LGLGIDEDDPTADDTSAAVTEEMPPLEGDDDTSR	12	153792590	1xTMT6plex [N-Term]	100	100		5.32486248	56.28	0	3776.701748	3	1259.572021	-0.062739102	-7.90E-05	83.72937565
																			TLTIVDTGIGMTK	112	153792590	2xTMT6plex [N-Term; K13]	1.38	1.918	1.148	5.43728447	77.13	0	1808.060342	2	904.5341797	0.409441534	0.000370147	82.52676804
																			LVTSPCCIVTSTYGWTANMER	18	153792590	1xTMT6plex [N-Term]; 2xCarbamidomethyl [C6; C7	2.289	1.814		4.286273956	32.02	0	2675.282895	3	892.4314575	-1.149549983	-0.001025124	92.09872164
																			HNDDEQYAWESSAGGSFTVR	5	153792590	1xTMT6plex [N-Term]		3.514		5.683339596	20.51	0	2485.12176	3	829.0448608	-0.696541512	-0.000576997	53.1127403
																			GVVDSEDIPLNLSR	72	153792590	1xTMT6plex [N-Term]	1.313	1.533	1.788	4.148833275	65.84	0	1742.948594	2	871.9779053	-0.034627025	-3.02E-05	73.03318811
																			DNSTMGYMAAK	6	153792590	2xTMT6plex [N-Term; K11]	1.914	2.007	2.266	4.966165343	76.58	0	1646.828234	2	823.9179077	0.184997853	0.00015233	43.6862046
																			LGIHEDSQNR	6	153792590	1xTMT6plex [N-Term]		2.13	1.808	3.477885008	31.67	0	1397.733457	3	466.5824585	-0.453957595	-0.000211504	32.28137538
																			SLTNDWEDHLAVK	31	153792590	2xTMT6plex [N-Term; K13]	1.66	2.373	1.777	3.927908897	35.62	0	1986.069661	3	662.6934814	-1.898429917	-0.001256805	59.87165218
																			HIYYITGETK	10	153792590	2xTMT6plex [N-Term; K10]	1.876	1.827	1.588	4.277496815	54.1	0	1682.951778	2	841.9798584	0.39322985	0.000330893	43.72346902
																			EDQTEYLEER	18	153792590	1xTMT6plex [N-Term]	1.944	2.024	1.802	4.666929245	57.19	0	1540.732847	2	770.8695679	-0.641498501	-0.000494189	32.67161311
																			ELHINLIPNK	8	153792590	2xTMT6plex [N-Term; K10]	1.933	1.497		3.856056452	37.85	0	1649.015047	2	825.012146	1.193441012	0.000984001	64.51090772
																			HGLEVIYMIEPIDEYCVQQLK	6	153792590	2xTMT6plex [N-Term; K21]; 1xCarbamidomethyl [C16		100		2.263687611	13.8	0	3035.598622	3	1012.536255	-1.45288693	-0.001470127	93.45311333
																			YIDQEELNK	19	153792590	2xTMT6plex [N-Term; K9]	1.275	1.351	1.4	3.791282892	54.22	0	1609.883758	2	805.4441528	-1.695362117	-0.001364668	54.42197424
																			EKYIDQEELNK	4	153792590	3xTMT6plex [N-Term; K2; K11]	0.801	0.782		4.17748785	29.16	1	2096.184246	3	699.4006348	1.481118313	0.001034899	48.24794731
																			DLVILLYETALLSSGFSLEDPQTHANR	2	153792590	1xTMT6plex [N-Term]		100		2.679760218	10.81	0	3231.709873	3	1077.904419	-3.456317089	-0.003723271	116.2661402
																			VILHLKEDQTEYLEER	9	153792590	2xTMT6plex [N-Term; K6]	0.523		0.218	3.634666681	13.42	1	2473.37026	4	619.0985107	0.789675036	0.00048829	71.13611857
																			YESLTDPSK	6	153792590	2xTMT6plex [N-Term; K9]	1.905		1.746	3.183604002	31.2	0	1497.820096	2	749.4136353	-0.067866754	-5.08E-05	42.36499642
																			EGLELPEDEEEKKK	2	153792590	4xTMT6plex [N-Term; K12; K13; K14]	0.963			3.975937128	8.37	2	2589.479315	4	648.1246948	-0.913362432	-0.000591283	53.67155292
																			EGLELPEDEEEK	6	153792590	2xTMT6plex [N-Term; K12]	1.024	2.617	1.255	2.718668461	30.02	0	1874.963525	2	937.986145	0.79403628	0.000744395	38.82471014
																			EGLELPEDEEEKK	2	153792590	3xTMT6plex [N-Term; K12; K13]	1.25			3.364272594	13.71	1	2232.22142	3	744.744751	-0.770492702	-0.000573303	51.48909641
																			QKAEADKNDK	3	153792590	4xTMT6plex [N-Term; K2; K7; K10]	0.867	0.839		2.46920228	4.58	2	2063.226669	3	688.413147	-0.863570667	-0.000593914	29.2147933
																			KFYEQFSK	6	153792590	3xTMT6plex [N-Term; K1; K8]	1.114	1.254	1.15	3.9442451	25.23	1	1764.029918	3	588.6821289	1.085717839	0.000638413	50.91288029
																			LGLGIDEDDPTADDTSAAVTEEMPPLEGDDDTSR	2	153792590	1xTMT6plex [N-Term]; 1xOxidation [M23]				1.438482285	1.65	0	3792.696663	3	1259.572021	-0.062739102	-7.90E-05	83.72937565
																			DNSTMGYMAAK	3	153792590	2xTMT6plex [N-Term; K11]; 1xOxidation [M	1.414	1.489		2.204852819	20.25	0	1662.823149	2	823.9179077	0.184997853	0.00015233	43.6862046
																			TLTIVDTGIGMTK	8	153792590	2xTMT6plex [N-Term; K13]; 1xOxidation [M11				1.712855339	13.32	0	1824.055257	2	904.5341797	0.409441534	0.000370147	82.52676804
																			APFDLFENR	8	153792590	1xTMT6plex [N-Term]	2.777	1.969	3.189	3.023033142	28.89	0	1337.705117	2	669.3568115	0.919183802	0.000614798	64.24405005
																			YYTSASGDEMVSLKDYCTR	2	153792590	2xTMT6plex [N-Term; K14]; 1xCarbamidomethyl [C17				1.973593354	6.45	1	2704.299874	3	902.1056519	0.935046602	0.000842882	65.02131463
																			CLELFTELAEDKENYK	4	153792590	3xTMT6plex [N-Term; K12; K16]; 1xCarbamidomethyl [C1			100	1.691680551	8.76	1	2689.43618	3	897.1495361	-0.790195944	-0.000708394	91.39285291
																			YYTSASGDEMVSLK	2	153792590	2xTMT6plex [N-Term; K14]	100			2.518295765	14.17	0	2009.030164	2	1005.021118	2.386862183	0.002397639	64.61034967
																			RAPFDLFENR	2	153792590	1xTMT6plex [N-Term]	0.823			4.398855209	34.77	1	1493.806228	3	498.6064148	-1.028684448	-0.000512218	67.96780058
																			FYEQFSK	10	153792590	2xTMT6plex [N-Term; K7]	1.154	2.228	100	2.583279133	30.42	0	1406.772023	2	703.8895874	-0.088916548	-6.25E-05	53.68403994
																			AILFVPR	28	153792590	1xTMT6plex [N-Term]	1.117	1.536	1.596	2.721352577	27.27	0	1044.676717	2	522.8417358	-0.500021015	-0.00026118	58.53335234
																			EMLQQSK	10	153792590	2xTMT6plex [N-Term; K7]	1.537	1.462	1.364	3.228847504	37.07	0	1321.754993	2	661.3809814	-0.232062203	-0.000153365	41.1996223
																			KCLELFTELAEDKENYK	2	153792590	4xTMT6plex [N-Term; K1; K13; K17]; 1xCarbamidomethyl [C2	0.739			1.502447248	0.47	2	3046.694075	4	762.4269409	-2.672240114	-0.002035375	80.12375779
																			LVTSPCCIVTSTYGWTANMER	2	153792590	1xTMT6plex [N-Term]; 2xCarbamidomethyl [C6; C7]; 1xOxidation [M19			100	1.751575708	11.19	0	2691.27781	3	892.4314575	-1.149549983	-0.001025124	92.09872164
																			AEADKNDK	4	153792590	3xTMT6plex [N-Term; K5; K8]		2.457	1.619	1.319380522	10.64	1	1577.910197	2	789.4589844	0.313782116	0.00024756	28.65007209
12025678	ACTN4	alpha-actinin-4	81	NP_0049152	54.99451153	1	29	44	390	911	104.788	5.44	0.73	0.87	0.81	0.81	Significant	0.0025																
																			ASIHEAWTDGK	29	12025678	2xTMT6plex [N-Term; K11]	0.819	0.739	0.777	4.337274551	52.02	0	1672.905891	2	836.956543	-0.048713141	-4.07E-05	41.97986562
																			HEAFESDLAAHQDR	4	12025678	1xTMT6plex [N-Term]	1.38	1.58		5.814505577	79.39	0	1854.893205	3	618.9694214	0.272937631	0.000168757	35.43437257
																			KDDPVTNLNNAFEVAEK	10	12025678	3xTMT6plex [N-Term; K1; K17]	1.282	0.902	1.03	3.346189499	23.74	1	2591.428393	3	864.4806519	-0.382114975	-0.000330075	63.64663891
																			QLEAIDQLHLEYAK	10	12025678	2xTMT6plex [N-Term; K14]	1.066	100	0.853	4.354112148	34.03	0	2129.200676	3	710.4042358	-1.184172303	-0.000840447	73.50001098
																			VEQIAAIAQELNELDYYDSHNVNTR	8	12025678	1xTMT6plex [N-Term]	100	100		2.61275959	24.25	0	3134.559186	3	1045.524902	0.308608386	0.00032245	94.72660392
																			VLAVNQENEHLMEDYEK	8	12025678	2xTMT6plex [N-Term; K17]; 1xOxidation [M12	1.216	1.16	100	4.589642048	52.05	0	2535.280125	3	840.4332275	-0.031867298	-2.68E-05	67.9693681
																			VLAVNQENEHLMEDYEK	6	12025678	2xTMT6plex [N-Term; K17]	1.055	0.975	100	5.39163208	52.31	0	2519.28521	3	840.4332275	-0.031867298	-2.68E-05	67.9693681
																			HRPELIEYDK	6	12025678	2xTMT6plex [N-Term; K10]	0.744	0.981	0.818	4.269965649	24.97	0	1757.99504	3	586.6697998	-0.110339297	-6.47E-05	40.39192969
																			KHEAFESDLAAHQDR	22	12025678	2xTMT6plex [N-Term; K1]	1.221	1.179	1.081	6.276325703	49.49	1	2212.1511	3	738.0541992	-1.381096323	-0.001018398	32.08635561
																			ICDQWDALGSLTHSR	6	12025678	1xTMT6plex [N-Term]; 1xCarbamidomethyl [C2	1.058	1.432		4.497675896	41.18	0	1987.985725	3	663.3334961	0.105599979	7.00E-05	94.62981853
																			ACLISLGYDVENDR	6	12025678	1xTMT6plex [N-Term]; 1xCarbamidomethyl [C2	0.899	0.905	0.01	3.675924778	40.71	0	1853.926479	2	927.4663086	-0.614022461	-0.000569176	78.19615096
																			KTFTAWCNSHLR	14	12025678	2xTMT6plex [N-Term; K1]; 1xCarbamidomethyl [C7	0.781	0.745	0.66	5.023783207	36.72	1	1979.068556	3	660.3609009	-0.205699433	-0.000135698	47.56763229
																			ETTDTDTADQVIASFK	8	12025678	2xTMT6plex [N-Term; K16]	1.254		100	4.499399662	52.29	0	2200.138529	3	734.0511475	0.163813691	0.000120138	87.35305695
																			GISQEQMQEFR	6	12025678	1xTMT6plex [N-Term]	0.935	0.94	1.02	4.917563438	65.4	0	1581.789257	2	791.3977051	-0.710255013	-0.000561737	43.45491355
																			ASFNHFDKDHGGALGPEEFK	6	12025678	3xTMT6plex [N-Term; K8; K20]	0.751	1.038		6.948112965	52.64	1	2890.509102	3	964.1723022	-2.334855722	-0.002249641	52.97417616
																			RDHALLEEQSK	6	12025678	2xTMT6plex [N-Term; K11]	0.778	0.756	0.802	6.429866314	47.57	1	1784.006667	3	595.3399658	-0.741695133	-0.000441063	29.73338751
																			LMLLLEVISGER	6	12025678	1xTMT6plex [N-Term]	1.529	0.977		4.07251215	44.34	0	1601.94978	2	801.4775391	-1.23531026	-0.000989452	112.6907183
																			DYETATLSDIK	10	12025678	2xTMT6plex [N-Term; K11]	1.229	1.921	1.463	4.230872631	41.97	0	1713.931102	2	857.46875	-0.512879426	-0.00043952	53.11820511
																			TINEVENQILTR	4	12025678	1xTMT6plex [N-Term]	1.053	0.357		3.503479242	38.67	0	1658.927465	2	829.9678345	0.559014985	0.000463683	75.81927579
																			ISNRPAFMPSEGK	12	12025678	2xTMT6plex [N-Term; K13]	1.022	1.14	1.258	3.736617565	34.99	0	1892.046424	3	631.3536377	-0.033775103	-2.13E-05	44.6700003
																			MLDAEDIVNTARPDEK	8	12025678	2xTMT6plex [N-Term; K16]	1.07	100	0.66	2.822241545	17.57	0	2275.200417	3	759.071167	-0.645992953	-0.000489921	71.3360394
																			DLLLDPAWEK	12	12025678	2xTMT6plex [N-Term; K10]	0.813	0.901	0.572	4.328368187	53.46	0	1657.956529	2	829.480835	-1.288404181	-0.001068059	87.29609035
																			NVNVQNFHISWK	4	12025678	2xTMT6plex [N-Term; K12]	0.866	0.617		3.157196283	27.95	0	1944.085586	3	648.6993408	-1.089061653	-0.000705743	61.07071108
																			KAGTQIENIDEDFR	6	12025678	2xTMT6plex [N-Term; K1]	0.76	1.277	1.078	3.882331848	18.09	1	2094.123154	3	698.7122192	-0.500961538	-0.000349692	47.77145689
																			NFITAEELR	6	12025678	1xTMT6plex [N-Term]	0.72	1.057	1.183	3.712983608	52.63	0	1321.731332	2	661.3692017	-0.154985875	-0.000102425	53.84458478
																			ELPPDQAEYCIAR	52	12025678	1xTMT6plex [N-Term]; 1xCarbamidomethyl [C10	0.858	0.866	0.824	4.162083626	66.41	0	1790.894451	2	895.951416	0.616921477	0.000552421	45.27772466
																			LASDLLEWIR	6	12025678	1xTMT6plex [N-Term]	1.309	1.315	1.315	3.386930704	34.01	0	1444.836131	2	722.9197998	-2.635558831	-0.001903975	99.6950994
																			AGTQIENIDEDFR	6	12025678	1xTMT6plex [N-Term]	100			2.718459129	25.47	0	1736.865259	2	868.9364014	0.15403289	0.000133767	59.54379461
																			TFTAWCNSHLR	6	12025678	1xTMT6plex [N-Term]; 1xCarbamidomethyl [C6	1.145	1.212	100	4.04608202	42.53	0	1621.810662	3	541.2745972	-0.87757845	-0.000474422	56.54119384
																			GYEEWLLNEIR	5	12025678	1xTMT6plex [N-Term]	0.919			2.728914261	28.04	0	1650.868888	2	825.9386597	0.699750263	0.000577598	98.48431158
																			GISQEQMQEFR	10	12025678	1xTMT6plex [N-Term]; 1xOxidation [M7]	0.988	0.974	0.77	3.018685818	44.59	0	1597.784172	2	791.3977051	-0.710255013	-0.000561737	43.45491355
																			VGWEQLLTTIAR	8	12025678	1xTMT6plex [N-Term]	0.83		1.103	2.625579834	24.77	0	1615.936908	2	808.4710693	-1.265815587	-0.001022739	94.62438909
																			VLAGDKNFITAEELR	4	12025678	2xTMT6plex [N-Term; K6]			100	2.734941483	1.76	1	2134.227225	3	712.0805664	-0.036889816	-2.62E-05	59.66340507
																			ALDFIASK	18	12025678	2xTMT6plex [N-Term; K8]	0.918	1.079	1.387	3.579351664	49.04	0	1322.808409	2	661.9074097	-0.654723735	-0.000433037	72.28877043
																			SIVDYKPNLDLLEQHQLIQEALIFDNK	2	12025678	3xTMT6plex [N-Term; K6; K28]				2.065212965	0.67	0	4012.236228	4	1003.813721	-0.791341117	-0.000793762	100.1592511
																			QGEAEFNR	4	12025678	1xTMT6plex [N-Term]		1.067	0.856	2.800581217	37.56	0	1179.595566	2	590.3010864	-0.568142554	-0.000335089	24.7762091
																			EAILAIHK	14	12025678	2xTMT6plex [N-Term; K8]	0.476	0.796	0.772	3.953683376	45.7	0	1352.866592	3	451.6268921	-0.346930341	-0.00015645	48.09285912
																			MAPYQGPDAVPGALDYK	6	12025678	2xTMT6plex [N-Term; K17]	0.956	0.567	1.264	1.954459429	9.14	0	2251.183311	2	1126.093262	-1.805340543	-0.002032076	61.0435249
																			DDPVTNLNNAFEVAEK	2	12025678	2xTMT6plex [N-Term; K16]				1.004043818	7.41	0	2234.170498	2	1117.586914	-1.766237181	-0.001973038	69.79888092
																			CQLEINFNTLQTK	2	12025678	2xTMT6plex [N-Term; K13]; 1xCarbamidomethyl [C1				1.278594732	6.91	0	2067.130882	2	1034.06897	-0.10594727	-0.000109503	72.41597984
																			STLPDADR	2	12025678	1xTMT6plex [N-Term]		0.812		3.116557837	25.58	0	1103.589418	2	552.2984619	0.207321782	0.000114399	30.74443716
																			KAGTQIENIDEDFRDGLK	4	12025678	3xTMT6plex [N-Term; K1; K18]		0.762		2.562581062	6.26	2	2736.513519	4	684.8829346	-1.319441225	-0.000902667	55.25884336
																			QFASQANVVGPWIQTK	2	12025678	2xTMT6plex [N-Term; K16]				1.858385921	12.04	0	2232.254108	2	1116.629272	-1.272193033	-0.001419929	67.27401615
																			LDHLAEK	8	12025678	2xTMT6plex [N-Term; K7]	0.738	0.644		2.917098284	35.57	0	1283.772358	2	642.3897705	-0.072633107	-4.66E-05	48.3032588
																			VQQLVPK	7	12025678	2xTMT6plex [N-Term; K7]	0.956	0.858	0.855	2.648086309	31.67	0	1269.829479	2	635.4180908	-0.451713702	-0.0002868	49.62512511
																			TIPWLEDR	5	12025678	1xTMT6plex [N-Term]	1.016		0.942	2.391861916	24.72	0	1258.699303	2	629.8526001	-1.096071702	-0.000689812	65.16942341
431822403	HSP90AB1	heat shock protein HSP 90-beta isoform a	3326	NP_001258899.1	62.43093923	1	25	42	526	724	83.212	5.03	1.3	1.38	1.24	1.31	Significant	0.00096																
																			ADLINNLGTIAK	22	431822403	2xTMT6plex [N-Term; K12]	1.397	1.655	1.929	4.478831291	79.53	0	1701.031091	2	851.0209351	2.058873118	0.001751104	74.74577045
																			HSQFIGYPITLYLEK	8	431822403	2xTMT6plex [N-Term; K15]	0.392	100	100	3.647228718	31.28	0	2267.284011	3	756.4317017	-1.525869504	-0.001153193	85.06038388
																			ELISNASDALDK	18	431822403	2xTMT6plex [N-Term; K12]	1.358	1.032	1.87	5.155765057	86.93	0	1733.968551	2	867.487793	-0.139115845	-0.000120611	70.01023764
																			EQVANSAFVER	8	431822403	1xTMT6plex [N-Term]	1.163	1.256	1.432	4.707737446	70.22	0	1478.780073	2	739.8942871	0.828411725	0.000612519	38.13169554

Bhat et al,2017. Cigarette smoke and chewing tobacco alter expression of different sets of miRNAs in oral keratinocytes

Supplementary Table S2(A). List of all proteins and peptides identified in OKF6/TERT1 cells chronically treated with chewing tobacco using Sequest and Mascot search algorithms

Accession	Gene_Symbol	Description	Gene_ID	Refseq_Accession	Coverage	Proteins	#Unique_Peptides	#Peptides	#PSMS	#AAs	MW [kDa]	calc. pI	OKF6-STE-6M/OKF6P_R1	OKF6-STE-6M/OKF6P_R2	OKF6-STE-6M/OKF6P_R3	Median fold change	Status	P-value (6M)	Sequence	#PSMSs	Protein Accessions	Modifications	OKF6-STE-6M/OKF6P_R1	OKF6-STE-6M/OKF6P_R2	OKF6-STE-6M/OKF6P_R3	Xcorr	Ionscore	# Missed Cleavages	Theo. MH+ [Da]	Charge	m_z	DeltaM [ppm]	Deltam/z [Da]	RT [min]
																			VFIMDSCDELIPEYLNFIR	12	431822403	1xTMT6plex [N-Term]; 1xCarbamidomethyl [C7	1.593	1.694	1.42	3.377512217	26.85	0	2603.308698	3	868.44104	-0.050552135	-4.39E-05	105.8355786
																			GFEVVYMTEPIDEYCVQQLK	37	431822403	2xTMT6plex [N-Term; K20]; 1xCarbamidomethyl [C15	2.076	1.268	1.477	3.879631758	35.78	0	2906.472024	3	969.494812	-0.736819355	-0.000713848	87.81991199
																			NPDDITQEEYGEFYK	15	431822403	2xTMT6plex [N-Term; K15]	1.251	0.98	100	3.863103867	41.95	0	2306.122879	2	1153.564331	-0.647480947	-0.000746585	66.08016516
																			DNSTMGYMMAK	8	431822403	2xTMT6plex [N-Term; K11]	1.579	1.359	1.792	5.077943325	75.95	0	1706.831605	2	853.9188843	-0.652106107	-0.000556518	60.72323735
																			DLVVLLFETALLSSGFSLEDPQTHSNR	25	431822403	1xTMT6plex [N-Term]	100	1.601	1.292	3.169044971	21.56	0	3217.694223	3	1073.234497	-1.642598301	-0.001761793	117.864499
																			YHTSQSGDEMTSLSEYVSR	8	431822403	1xTMT6plex [N-Term]	1.64	1.296	1.542	5.015532494	25.11	0	2406.108084	3	808.0385132	-0.830992414	-0.000670916	41.60629096
																			YHTSQSGDEMTSLSEYVSR	16	431822403	1xTMT6plex [N-Term]; 1xOxidation [M10]	1.475	1.163	1.383	6.617705822	60.89	0	2422.102999	3	808.0385132	-0.830992414	-0.000670916	41.60629096
																			HLEINPDHPIVETLR	6	431822403	1xTMT6plex [N-Term]			1.362	4.883922577	39.07	0	2012.11264	3	671.3760376	0.457078272	0.000306564	56.40877316
																			TLTIVDTGIGMTK	112	431822403	2xTMT6plex [N-Term; K13]	1.38	1.918	1.148	5.43728447	77.13	0	1808.060342	2	904.5341797	0.409441534	0.000370147	82.52676804
																			HNDDEQYAWESSAGGSFTVR	5	431822403	1xTMT6plex [N-Term]		3.514		5.683339596	20.51	0	2485.12176	3	829.0448608	-0.696541512	-0.000576997	53.1127403
																			GVVDSEDIPLNLSR	72	431822403	1xTMT6plex [N-Term]	1.313	1.533	1.788	4.148833275	65.84	0	1742.948594	2	871.9779053	-0.034627025	-3.02E-05	73.03318811
																			LGLGIDEDEVAAEEPNAAVPDEIPPLEGDEDASR	8	431822403	1xTMT6plex [N-Term]		1.47		5.636335373	43.39	0	3761.807868	3	1254.605469	-1.59903435	-0.002005087	88.42082222
																			KHLEINPDHPIVETLR	6	431822403	2xTMT6plex [N-Term; K1]		0.67	1.001	5.732170105	38.75	1	2369.370535	4	593.0982056	0.193121176	0.000114394	51.46825506
																			IEDVGSDEEDDSGKDK	2	431822403	3xTMT6plex [N-Term; K14; K16]			0.585	3.457731485	22.03	1	2425.218519	3	809.0784302	0.914720602	0.000739466	44.93924866
																			SLTNDWEDHLAVK	31	431822403	2xTMT6plex [N-Term; K13]	1.66	2.373	1.777	3.927908897	35.62	0	1986.069661	3	662.6934814	-1.898429917	-0.001256805	59.87165218
																			LGIHEDSTNR	10	431822403	1xTMT6plex [N-Term]	1.616	1.482	1.33	3.473070383	45.56	0	1370.722558	3	457.5788879	-0.326216008	-0.000149051	28.94535622
																			EDQTEYLEER	18	431822403	1xTMT6plex [N-Term]	1.944	2.024	1.802	4.666929245	57.19	0	1540.732847	2	770.8695679	-0.641498501	-0.000494189	32.67161311
																			SIYYITGESK	14	431822403	2xTMT6plex [N-Term; K10]	1.116	1.213	1.196	3.991729736	52.65	0	1618.909245	2	809.9580078	-0.312472735	-0.000252933	54.61589588
																			GFEVVYMTEPIDEYCVQQLK	13	431822403	2xTMT6plex [N-Term; K20]; 1xCarbamidomethyl [C15]; 1xOxidation [M7]	100	100	100	2.141558886	17.04	0	2922.466939	3	969.494812	-0.736819355	-0.000713848	87.81991199
																			YIDQEELNK	19	431822403	2xTMT6plex [N-Term; K9]	1.275	1.351	1.4	3.791282892	54.22	0	1609.883758	2	805.4441528	-1.695362117	-0.001364668	54.42197424
																			EKYIDQEELNK	4	431822403	3xTMT6plex [N-Term; K2; K11]	0.801	0.782		4.17748785	29.16	1	2096.184246	3	699.4006348	1.481118313	0.001034899	48.24794731
																			VILHLKEDQTEYLEER	9	431822403	2xTMT6plex [N-Term; K6]	0.523		0.218	3.634666681	13.42	1	2473.37026	4	619.0985107	0.789675036	0.00048829	71.13611857
																			YESLTDPSK	6	431822403	2xTMT6plex [N-Term; K9]	1.905		1.746	3.183604002	31.2	0	1497.820096	2	749.4136353	-0.067866754	-5.08E-05	42.36499642
																			EGLELPEDEEEKKK	2	431822403	4xTMT6plex [N-Term; K12; K13; K14]	0.963			3.975937128	8.37	2	2589.479315	4	648.1246948	-0.913362432	-0.000591283	53.67155292
																			EGLELPEDEEEK	6	431822403	2xTMT6plex [N-Term; K12]	1.024	2.617	1.255	2.718668461	30.02	0	1874.963525	2	937.986145	0.79403628	0.000744395	38.82471014
																			EGLELPEDEEEKK	2	431822403	3xTMT6plex [N-Term; K12; K13]	1.25			3.364272594	13.71	1	2232.22142	3	744.744751	-0.770492702	-0.000573303	51.48909641
																			KHSQFIGYPITLYLEK	6	431822403	3xTMT6plex [N-Term; K1; K16]		0.749		3.641346455	29.28	1	2624.541906	3	875.5187378	-0.093784402	-8.20E-05	85.34902045
																			QKAEADKNDK	3	431822403	4xTMT6plex [N-Term; K2; K7; K10]	0.867	0.839		2.46920228	4.58	2	2063.226669	3	688.413147	-0.863570667	-0.000593914	29.2147933
																			TLTIVDTGIGMTK	8	431822403	2xTMT6plex [N-Term; K13]; 1xOxidation [M11				1.712855339	13.32	0	1824.055257	2	904.5341797	0.409441534	0.000370147	82.52676804
																			CLELFSELAEDKENYK	4	431822403	3xTMT6plex [N-Term; K12; K16]; 1xCarbamidomethyl [C1	100	100		2.009843111	4	1	2675.42053	3	892.4783325	-0.032130054	-2.87E-05	103.2017906
																			IDIIPNPQER	21	431822403	1xTMT6plex [N-Term]	1.81	100	1.34	2.960270643	29.95	0	1423.810644	2	712.4093018	0.479309259	0.000341223	63.04375129
																			AKFENLCK	4	431822403	3xTMT6plex [N-Term; K2; K8]; 1xCarbamidomethyl [C7		0.452	0.619	3.212839365	13.67	1	1697.002324	3	566.3391724	0.377271047	0.00021341	42.63429799
																			FYEAFSK	11	431822403	2xTMT6plex [N-Term; K7]	1.375	1.458	1.484	3.498731375	36.92	0	1349.75056	2	675.3790894	0.253758693	0.000171255	57.58021864
																			EEEDKDDEEKPK	1	431822403	4xTMT6plex [N-Term; K5; K10; K12]		1.258		2.301478386		1	2407.301016	4	602.5806274	-0.139577216	-8.40E-05	32.06117966
																			SLVSVTK	10	431822403	2xTMT6plex [N-Term; K7]	0.869	1.071	0.57	3.324399948	41.58	0	1191.771295	2	596.3894653	0.301235702	0.000179502	42.2477619
																			EMLQQSK	10	431822403	2xTMT6plex [N-Term; K7]	1.537	1.462	1.364	3.228847504	37.07	0	1321.754993	2	661.3809814	-0.232062203	-0.000153365	41.1996223
																			ADHGEPIGR	2	431822403	1xTMT6plex [N-Term]	0.619			2.841734886	29.29	0	1180.627201	2	590.8171387	-0.169550769	-0.000100088	24.48694369
																			KFYEAFSK	3	431822403	3xTMT6plex [N-Term; K1; K8]	0.582		0.674	1.883521676	12.43	1	1707.008455	3	569.6746216	0.502022173	0.000285652	60.90053747
																			ALLFIPR	12	431822403	1xTMT6plex [N-Term]	0.984	1.187	0.854	2.524053335	27.85	0	1058.692367	2	529.8496704	-0.286466302	-0.00015164	80.57592081
																			AEADKNDK	4	431822403	3xTMT6plex [N-Term; K5; K8]		2.457	1.619	1.319380522	10.64	1	1577.910197	2	789.4589844	0.313782116	0.00024756	28.65007209
																			IEDVGSDEEDDSGK	1	431822403	2xTMT6plex [N-Term; K14]			0.661	1.227139354		0	1952.933681	2	976.97052	0.042218055	4.12E-05	40.5395537
6005942	VCP	transitional endoplasmic reticulum ATPase	7415	NP_009057.1	66.00496278	1	41	41	340	806	89.266	5.26	1.08	1.17	1.21	1.15	Significant	0.0011																
																			AIANECQANFISIK	11	6005942	2xTMT6plex [N-Term; K14]; 1xCarbamidomethyl [C6	1.054	1.158	1.512	3.723666906	64.24	0	2037.120317	2	1019.063904	0.10496051	0.000106909	74.48506044
																			KYEMFAQTLQQSR	6	6005942	2xTMT6plex [N-Term; K1]	1.647	100	1.141	3.601224184	27.04	1	2088.131216	3	696.7145996	-0.943581447	-0.000656774	52.31508718
																			EAVCIVLSDDTCSDEK	6	6005942	2xTMT6plex [N-Term; K16]; 2xCarbamidomethyl [C4; C12			1.374	3.343739748	33.89	0	2299.119784	3	767.045166	0.50467439	0.000386769	66.79892409
																			LIVDEAINEDNSVVSLSQPK	24	6005942	2xTMT6plex [N-Term; K20]	1.45	1.892	1.199	3.608157873	44.66	0	2628.449632	3	876.8198853	-1.723376482	-0.001509936	81.16668872
																			VVETDPSPYCIVAPDTVIHCEGEPIKR	6	6005942	2xTMT6plex [N-Term; K26]; 2xCarbamidomethyl [C10; C2(	1.119	1.023	100	6.177518845	26.48	1	3539.827846	4	885.7116699	-0.846550349	-0.000749161	71.02446463
																			EDEEESLNEVGYDDIGGCR	14	6005942	1xTMT6plex [N-Term]; 1xCarbamidomethyl [C18	1.376	1.216		5.014746189	56.62	0	2415.045544	3	805.6870728	0.464339781	0.000373801	62.05420828
																			WALSQSNPSALR	8	6005942	1xTMT6plex [N-Term]	1.119	1.165	0.976	3.667323589	55.53	0	1558.853906	2	779.9301758	-0.533249137	-0.000415629	62.34637509
																			LADDVDLEQVANETHGHVGADLAALCSEAALQAIR	16	6005942	1xTMT6plex [N-Term]; 1xCarbamidomethyl [C26	1.397	1.297	100	7.438237667	78.77	0	3901.955159	4	976.244751	0.516478577	0.000503819	93.20819524
																			MDLIDLEDETIDAEVMNSLAVTMDDFR	4	6005942	1xTMT6plex [N-Term]; 1xOxidation [M23]	100	0.567		3.622459888	34.79	0	3346.557791	3	1116.190063	-0.643595732	-0.000717943	112.3784164
																			GDDLSTAILK	12	6005942	2xTMT6plex [N-Term; K10]	1.084	0.834	1.517	3.526488781	70.72	0	1490.88303	2	745.9451904	0.049694962	3.70E-05	64.18372055
																			ETVVEVPQVTWEDIGGLEDVK	13	6005942	2xTMT6plex [N-Term; K21]	1.333	100		3.535611391	27.38	0	2800.502062	3	934.1699219	-2.445770383	-0.002283128	96.0143198
																			GGNIGDGGGAADR	21	6005942	1xTMT6plex [N-Term]	0.987	1.08	1.289	3.987061262	82.46	0	1345.665771	2	673.3359985	-0.780773139	-0.00052533	20.47132912
																			AHVIVMAATNRPNSIDPALR	10	6005942	1xTMT6plex [N-Term]	0.916	1.011	0.976	3.766069651	24.27	0	2375.317898	3	792.4440918	-0.074128762	-5.87E-05	48.41144258
																			REDEEESLNEVGYDDIGGCR	4	6005942	1xTMT6plex [N-Term]; 1xCarbamidomethyl [C19		0.492	0.55	5.347448349	54.62	1	2571.146655	3	857.7212524	0.991582575	0.000849835	45.21141082
																			KMDLIDLEDETIDAEVMNSLAVTMDDFR	2	6005942	2xTMT6plex [N-Term; K1]; 1xOxidation [M2]				3.032847404	19.97	1	3703.815686	3	1235.279663	2.362438781	0.002916679	115.6266988
																			GVLFYGPPGCGK	6	6005942	2xTMT6plex [N-Term; K12]; 1xCarbamidomethyl [C10	1.098	0.898	100	3.632905483	50.05	0	1709.944919	2	855.4766235	0.614768523	0.00052561	65.69510358
																			ELQELVQYPVEHPDK	4	6005942	2xTMT6plex [N-Term; K15]			0.889	2.983733654	23.84	0	2282.243269	3	761.4192505	-0.030849102	-2.35E-05	67.71919202
																			LAGESESNLR	11	6005942	1xTMT6plex [N-Term]	1.115	1.344	1.198	3.853788853	54.67	0	1304.70076	2	652.8543701	0.539552361	0.000351977	34.47553592
																			GPELLTMWFGESEANVR	4	6005942	1xTMT6plex [N-Term]		0.789		2.6002841	32.06	0	2165.089855	2	1083.047119	-1.336562888	-0.001446889	105.0954144
																			VINQILTEMDGMSTK	6	6005942	2xTMT6plex [N-Term; K15]	0.99	3.303		2.989359856	41.14	0	2138.160133	2	1069.581543	-2.022010622	-0.002161691	105.7781334
																			GILLYGPPGTGK	20	6005942	2xTMT6plex [N-Term; K12]	0.921	0.986	100	4.076074123	49.11	0	1630.993249	2	816.0006104	0.425981607	0.000347387	66.10245537
																			EVDIGIPDATGR	6	6005942	1xTMT6plex [N-Term]	1.121	1.403	1.126	3.766639948	45.86	0	1471.795388	2	736.4006958	-0.865112398	-0.000636634	51.05740037
																			NAPAIIFIDELDAIAPK	4	6005942	2xTMT6plex [N-Term; K17]		0.731		2.222305536	18.11	0	2269.320791	2	1135.164185	0.13300483	0.000150915	112.7304032
																			IHVLPIDDTVEGITGNLFEVYLKPYFLEAYRPIR	2	6005942	2xTMT6plex [N-Term; K23]			0.947	3.688635588	5.23	0	4449.46265	5	890.6911011	-8.147280191	-0.007250204	114.9518179
																			KMDLIDLEDETIDAEVMNSLAVTMDDFR	4	6005942	2xTMT6plex [N-Term; K1]		100	0.529	2.641771793	9.33	1	3687.820771	3	1235.279663	2.362438781	0.002916679	115.6266988
																			AHVIVMAATNRPNSIDPALR	2	6005942	1xTMT6plex [N-Term]; 1xOxidation [M6]	1.008			2.705992937	8.85	0	2391.312813	3	792.4440918	-0.074128762	-5.87E-05	48.41144258
																			LDQLIYIPLPDEK	6	6005942	2xTMT6plex [N-Term; K13]	0.88	0.888		3.052363634	32.45	0	2015.1829	2	1008.093994	-1.086099306	-0.001094344	97.08454067
																			DVDLEFLAK	22	6005942	2xTMT6plex [N-Term; K9]	1.118	0.992	1.231	3.207843304	36.89	0	1507.877217	2	754.4416504	-0.790753211	-0.000596179	70.83014091
																			RDHFEEAMR	2	6005942	1xTMT6plex [N-Term]		0.984		4.215380192	33.25	1	1419.700048	3	473.9049072	0.084693727	4.01E-05	27.77317259
																			ESIESEIR	8	6005942	1xTMT6plex [N-Term]		1.088	1.164	3.415489674	55.67	0	1191.641848	2	596.3233643	-2.010632959	-0.001197977	37.96270405
																			EMVELPLR	18	6005942	1xTMT6plex [N-Term]	1.017	1.681	1.319	2.98930645	41.52	0	1215.69686	2	608.3527832	1.175783451	0.000714698	61.54692651
																			LEILQIHTK	18	6005942	2xTMT6plex [N-Term; K9]	1.06	1.213	1.32	2.911663055	30.81	0	1552.982685	3	518.3319092	-0.972554911	-0.000503454	55.78494788
																			MDELQLFR	8	6005942	1xTMT6plex [N-Term]	1.087	1.532	100	2.977061749	43.03	0	1280.687024	2	640.8480835	1.457352306	0.000933206	96.58384214
																			QAAPCVLFFDELDSIAK	3	6005942	2xTMT6plex [N-Term; K17]; 1xCarbamidomethyl [C5		1.085		2.009511471	9.87	0	2382.27794	2	1191.639893	-2.27989088	-0.002715667	108.039765
																			KAFEEAEK	6	6005942	3xTMT6plex [N-Term; K1; K8]	0.977	0.872		3.739186287	33.08	1	1638.966984	2	819.9865112	-0.755219034	-0.00061889	43.13840435
																			GDTVLLK	5	6005942	2xTMT6plex [N-Term; K7]	1.212	1.021	1.143	2.922332525	43.96	0	1203.771295	2	602.3890381	-0.411613175	-0.000247744	49.09497258
																			ETVVEVPQVTWEDIGGLEDVKR	2	6005942	2xTMT6plex [N-Term; K21]				1.478204131	2.86	1	2956.603173	3	986.204834	-1.090446261	-0.001074672	94.76303197
																			LGDVISIQPCPDVK	3	6005942	2xTMT6plex [N-Term; K14]; 1xCarbamidomethyl [C10			100	2.009958267	16.03	0	1999.129819	2	1000.067017	-1.531994994	-0.001531328	67.2788022
																			KQLAQIK	2	6005942	3xTMT6plex [N-Term; K1; K7]	0.763			3.05654335	13.22	1	1516.01896	3	506.011261	0.178097367	9.00E-05	47.94953546
																			FGMTPSK	5	6005942	2xTMT6plex [N-Term; K7]	1.466	1.53	1.558	2.671622515	36.1	0	1225.701501	2	613.3544312	0.069123426	4.24E-05	45.11255673
																			EMVELPLR	2	6005942	1xTMT6plex [N-Term]; 1xOxidation [M2]		1.094		1.627843499	23.38	0	1231.691775	2	608.3527832	1.175783451	0.000714698	61.54692651
																			AFEEAEK	3	6005942	2xTMT6plex [N-Term; K7]	0.611	1.073		3.081395864	31.86	0	1281.709089	2	641.357666	-0.806149195	-0.000516624	39.04083745
																			GDIFLVR	8	6005942	1xTMT6plex [N-Term]	1.47	1.472	1.416	2.432400942	24.22	0	1048.635246	2	524.8206787	-1.111633553	-0.000582849	52.1721393
																			RDHFEEAMR	2	6005942	1xTMT6plex [N-Term]; 1xOxidation [M8]	0.635			1.531326056	13.79	1	1435.694963	3	473.9049072	0.084693727	4.01E-05	27.77317259
																			AIGVKPPR	1	6005942	2xTMT6plex [N-Term; K5]	0.781			2.581217527	4.25	0	1295.856362	3	432.6235352	-0.239116972	-0.000103287	36.88207604
13654237	PRKDC	DNA-dependent protein kinase catalytic subunit isoform 1	5591	NP_008835.5	16.98158915	1	63	63	281	4128	468.788	7.12	1.5	1.54	1.42	1.48	Significant	0.00056																
																			AQEPESGLSEETQVK	6	13654237	2xTMT6plex [N-Term; K15]	1.209	1.301	1.026	3.875542641	47.05	0	2090.101749	2	1045.554443	-0.066619364	-6.96E-05	43.76816193
																			STVLTPMFVETQASQGTLQTR	9	13654237	1xTMT6plex [N-Term]	100	100	100	3.249326468	34.99	0	2524.327854	3	842.1123657	-2.103416172	-0.001769904	72.46634457
																			QMFLTQTDTGDDR	4	13654237	1xTMT6plex [N-Term]	3.061		2.376	3.368677855	42.92	0	1756.837329	2	878.9219971	-0.348216289	-0.00030588	52.89105444
																			DVLIQGLIDENPGLQLIIR	5	13654237	1xTMT6plex [N-Term]			2.164	4.715911388	74.28	0	2348.375062	3	783.4616089	-2.039077966	-0.001596173	106.0535199
																			ATQQQHDFTLTQTADGR	12	13654237	1xTMT6plex [N-Term]	1.312	1.784	1.783	6.265936852	54.06	0	2147.067874	3	716.3619995	1.66316361	0.001190308	25.65602124
																			AVAFFLESIAMHDIIAAEK	4	13654237	2xTMT6plex [N-Term; K19]	1.525	1.766		6.284480572	56.72	0	2534.409288	3	845.4733276	-1.522398084	-0.001286127	116.7075504
																			KNILEESLCELVAK	4	13654237	3xTMT6plex [N-Term; K1; K14]; 1xCarbamidomethyl [C9	2.454		100	4.876824379	29.78	1	2333.37173	3	778.4619751	-0.153309062	-0.000119242	94.95173034
																			ATQMPEGGQGAPPMYQLYK	2	13654237	2xTMT6plex [N-Term; K19]	1.858			4.755376816	44.52	0	2525.293272	3	842.4348145	-1.339211038	-0.0011273	71.90745471
																			LQETLSAADR	6	13654237	1xTMT6plex [N-Term]	0.98	1.601	1.922	4.240533352	63.69	0	1332.73206	2	666.8679199	-2.623600314	-0.001748278	42.46773679
																			LVINTEEVFRPYAK	2	13654237	2xTMT6plex [N-Term; K14]	1.331			2.753125191	16.85	0	2137.242147	3	713.0853882	-0.250429844	-0.00017841	85.28534226
																			GIFTSEIGTK	6	13654237	2xTMT6plex [N-Term; K10]	1.646	1.375		3.433878183	54.74	0	1510.888116	2	755.9477539	0.07653942	5.78E-05	69.42826242
																			VTELALTASDR	8	13654237	1xTMT6plex [N-Term]		0.918	1.861	3.648876429	38.93	0	1404.789575	2	702.897583	-1.199627621	-0.000842612	50.15600602
																			LLALNSLYSPK	4	13654237	2xTMT6plex [N-Term; K11]	100	1.36		2.801337004	42.1	0	1677.035114	2	839.0205688	-0.747160675	-0.000626507	87.94091328
																			EMKPVIFLDVFLPR	2	13654237	2xTMT6plex [N-Term; K3]	2.005			3.038131714	11.49	0	2162.281175	3	721.432373	0.643417998	0.00046375	108.1213656
																			DPTVHDDVLELEMDELNR	7	13654237	1xTMT6plex [N-Term]	1.044	1.493	0.88	2.964663029	15.93	0	2369.149221	3	790.3876343	-0.367650109	-0.000290339	68.42700474
																			NELEIPGQYDGR	2	13654237	1xTMT6plex [N-Term]	1.375			3.267594099	22.63	0	1619.822666	2	810.414917	-0.066800908	-5.41E-05	58.80642971
																			SIGEYDVLR	6	13654237	1xTMT6plex [N-Term]	1.327	1.713		3.676093578	72.16	0	1280.704782	2	640.8555298	-0.780437226	-0.000499755	61.64505365
																			TQEGSLSAR	6	13654237	1xTMT6plex [N-Term]	2.837	2.459		3.238774776	55.55	0	1177.637431	2	589.3223267	-0.046270343	-2.72E-05	25.35289371
																			AMHGELQK	10	13654237	2xTMT6plex [N-Term; K8]	1.601	1.605	1.637	3.728497744	52.27	0	1371.781876	3	457.9320374	-0.231559739	-0.000105883	32.68168912
																			KIPDTVLEK	5	13654237	3xTMT6plex [N-Term; K1; K9]	1.724	1.674	1.411	3.854431868	31.25	1	1730.103083	3	577.3724976	-0.083061073	-4.79E-05	50.65005859
																			AIRPQIDLK	5	13654237	2xTMT6plex [N-Term; K9]	0.941	1.11		3.072915554	17.86	0	1511.967369	3	504.660675	0.068081155	3.43E-05	44.51602791
																			MEVQEQEDISSLIR	4	13654237	1xTMT6plex [N-Term]	100	100		1.731572628	27.51	0	2035.021501	2	1018.012756	-1.604398128	-0.001632492	81.03588504
																			SMEEDPQTSR	2	13654237	1xTMT6plex [N-Term]			1.179	2.608394623	28.63	0	1408.657574	2	704.8326416	0.306975331	0.000216212	28.35875395
																			TSALSDETK	4	13654237	2xTMT6plex [N-Term; K9]	1.78	1.14		3.218200207	34.62	0	1409.788795	2	705.397644	-0.556079084	-0.000391977	38.45944307
																			YPEETLSLMTK	2	13654237	2xTMT6plex [N-Term; K11]	1.312			3.250041246	34.65	0	1769.975944	2	885.489563	-2.313479712	-0.002047402	72.59404495
																			LACDVDQVTR	4	13654237	1xTMT6plex [N-Term]; 1xCarbamidomethyl [C3	1.818			3.523651361	33.94	0	1405.73068	2	703.3695679	0.838882017	0.000589621	45.25727038
																			LLEEALLR	8	13654237	1xTMT6plex [N-Term]	100	1.744		2.735780478	49.67	0	1185.74044	2	593.3738403	-0.030112777	-1.79E-05	83.18637027
																			ICSKPVVLPK	3	13654237	3xTMT6plex [N-Term; K4; K10]; 1xCarbamidomethyl [C2	1.094		1.986	3.205836773	9.15	0	1828.169723	3	610.0576782	-6.1492223	-0.003747274	47.5054141
																			GYGLFAGPCK	4	13654237	2xTMT6plex [N-Term; K10]; 1xCarbamidomethyl [C9	100	100		2.473051786	28.57	0	1527.839392	2	764.421814	-1.989934498	-0.00152015	53.09278424
																			QVSNMVAK	2	13654237	2xTMT6plex [N-Term; K8]	2.384			3.760056257	42.65	0	1334.786627	2	667.8968506	-0.152037877	-0.000101469	43.47697836
																			SLEYCSTASIDSENPPDLNK	2	13654237	2xTMT6plex [N-Term; K20]; 1xCarbamidomethyl [C5				2.020323515	8.27	0	2698.328197	3	900.1130981	-1.280662008	-0.001151882	61.41054653
																			MYAALGDPK	2	13654237	2xTMT6plex [N-Term; K9]		0.894		2.266586781	22.51	0	1423.801943	2	712.4058228	1.703683454	0.001212854	47.84769082
																			AALSALESFLK	6	13654237	2xTMT6plex [N-Term; K11]				2.162820578	7.74	0	1607.977265	2	804.4918823	-0.483123474	-0.000388426	102.2268512
																			ILELSGSSSEDSEK	2	13654237	2xTMT6plex [N-Term; K14]	100			1.42149353	5.19	0	1939.027187	2	970.0158081	-1.468715385	-0.00142394	50.28053344
																			DLLLNTMSQEEK	4	13654237	2xTMT6plex [N-Term; K12]			100	1.90104866	19.52	0	1879.024685	2	940.0149536	-1.093420142	-0.001027282	76.46306503
																			VTVMASLR	4	13654237	1xTMT6plex [N-Term]		1.33	0.957	2.79916954	35.91	0	1105.660081	2	553.3308716	-5.077931304	-0.002807233	47.08777676
																			EIFNFVLK	6	13654237	2xTMT6plex [N-Term; K8]	1.399	100	2.135	2.680489302	26.26	0	1467.897558	2	734.4523315	-0.116938721	-8.58E-05	93.91086783
																			APGLGAFR	6	13654237	1xTMT6plex [N-Term]	1.204	1.35	1.098	2.524226665	27.16	0	1017.604281	2	509.3053894	-0.765092503	-0.000389281	42.80491253
																			SVGPDFGK	6	13654237	2xTMT6plex [N-Term; K8]	1.283	1.186		3.137294292	32.26	0	1264.730158	2	632.8687134	-0.006603928	-4.18E-06	53.61328767
																			LQYFMEQFYGIIR	2	13654237	1xTMT6plex [N-Term]		2.483		2.161936998	8.43	0	1937.019257	2	969.0144653	1.237553036	0.001198582	118.0616198
																			NLLIFENLIDLK	4	13654237	2xTMT6plex [N-Term; K12]	100	100		1.685168862	9.99	0	1903.166856	2	952.0855713	-1.571313553	-0.001495236	104.2783398
																			SIELFYK	6	13654237	2xTMT6plex [N-Term; K7]	1.56		100	2.878638983	38.09	0	1357.81316	2	679.4103394	0.178427301	0.000121135	70.97655108
																			AYVPALQMAFK	5	13654237	2xTMT6plex [N-Term; K11]	100	100	100	1.586369038	5.2	0	1696.986055	2	848.99646	-0.242882447	-0.000206084	83.74038911
																			SDPGLLTNTMDVFVK	3	13654237	2xTMT6plex [N-Term; K15]				1.518728375	6.56	0	2095.150948	2	1048.078491	-0.59301127	-0.000621224	87.43667662
																			ILDVMYSR	8	13654237	1xTMT6plex [N-Term]	2.496	1.74		2.600038767	32.98	0	1225.68121	2	613.3441162	-0.207662582	-0.000127264	89.1338615
																			IWSEPFYQETYLPYMIR	4	13654237	1xTMT6plex [N-Term]	0.01	0.01		1.609095573	1.99	0	2465.241271	2	1233.121704	-2.084606053	-0.002569528	105.6204816
																			WPVAGQIR	6	13654237	1xTMT6plex [N-Term]	0.579	1.593	1.528	2.676081657	23.81	0	1155.683594	2	578.3449097	-0.90936141	-0.000525467	58.16652864
																			AFLGELK	2	13654237	2xTMT6plex [N-Term; K7]	2.064			3.134442568	22.12	0	1235.77638	2	618.3914795	-0.564888305	-0.000349038	74.58245272
																			FIQTFGK	3	13654237	2xTMT6plex [N-Term; K7]	1.87			2.850896597	33.86	0	1298.787279	2	649.897583	0.469611641	0.000304963	64.31094415
																			GLSSLLCNFTK	3	13654237	2xTMT6plex [N-Term; K11]; 1xCarbamidomethyl [C7				1.798205853	13.53	0	1697.966049	2	849.4853516	-1.54422699	-0.001311023	88.40902017
																			DILPCLDGYLK	5	13654237	2xTMT6plex [N-Term; K11]; 1xCarbamidomethyl [C5		100	100	1.514979959	7.52	0	1764.997014	2	883.00177	-0.425429013	-0.00037544	89.41694784
																			DCLSIPYR	4	13654237	1xTMT6plex [N-Term]; 1xCarbamidomethyl [C2	0.979		0.692	2.563941956	13.86	0	1252.655724	2	626.8273926	-6.558556826	-0.004107807	46.53138693
																			ECSPWMSDFK	5	13654237	2xTMT6plex [N-Term; K10]; 1xCarbamidomethyl [C2	100			1.728547096	10.96	0	1744.843885	2	872.9249878	-0.679461396	-0.000592777	64.10127007
																			YNFPVEVEVPMER	6	13654237	1xTMT6plex [N-Term]	100			1.81927681	12.19	0	1837.935587	2	919.4726563	1.332511334	0.001224535	77.12906538
																			QCLPSLDLSCK	3	13654237	2xTMT6plex [N-Term; K11]; 2xCarbamidomethyl [C2; C10	1.303	100		2.577871084	12.73	0	1778.954498	2	889.9822388	1.519470602	0.001351535	72.60506899
																			LLQIIER	5	13654237	1xTMT6plex [N-Term]	1.349	0.521		2.346637964	25.96	0	1113.71931	2	557.3632813	-0.022043256	-1.23E-05	94.03175159
																			KFIQTFGK	2	13654237	3xTMT6plex [N-Term; K1; K8]			1.772	1.854374647	5.55	1	1656.045174	3	552.6863403	-0.426729848	-0.000235561	59.84760015
																			DYVDLFR	2	13654237	1xTMT6plex [N-Term]	4.211			2.494345427	16.85	0	1156.61999	2	578.812561	-1.854411739	-0.001072425	64.37493685
																			LSDFNDITNMLLLK	4	13654237	2xTMT6plex [N-Term; K14]	100			1.456367254	4.75	0	2095.187334	2	1048.094971	-2.228432596	-0.002334492	102.6809496
																			LSFAVPFR	4	13654237	1xTMT6plex [N-Term]	4.174			2.319535494	17.31	0	1165.693096	2	583.3497925	-0.675451425	-0.000393685	76.46245541
																			LKPVELR	4	13654237	2xTMT6plex [N-Term; K2]		2.071	2.075	2.546921968	11.67	0	1312.871678	3	438.2952576	-0.349093464	-0.000152772	38.11221402
																			LPVLAGCLK	2	13654237	2xTMT6plex [N-Term; K9]; 1xCarbamidomethyl [C7	100			2.264346123	24.83	0	1428.901264	2	714.9539795	-0.406785017	-0.000290628	69.42939665
																			LLPAELPAK	3	13654237	2xTMT6plex [N-Term; K9]		1.466	100	2.043883085	13.25	0	1409.913208	2	705.4594116	-1.178482337	-0.000830779	74.5420769
316659409	ACTG1	actin, cytoplasmic 2	71	NP_001186883.1	75.73333333	1	10	20	1226	375	41.766	5.48	1.06	1.06	1.02	1.04	Significant	0.00016																
																			TTGIVMDSGDGVTHTVPIYEGYALPHAILR	14	316659409	1xTMT6plex [N-Term]	1.542		100	6.90781641	47.02	0	3412.777241	4	853.9509888	1.431024582	0.001220942	80.24707993
																			SYELPDGQVITIGNER	64	316659409	1xTMT6plex [N-Term]	1.248	1.218	1.377	3.803804874	38.48	0	2020.05485	2	1010.528687	-2.35325448	-0.002376852	85.21825869
																			AVFPSIVGRPR	34	316659409	1xTMT6plex [N-Term]	0.352	0.364	0.444	4.536440372	51.94	0	1427.868434	3	476.627655	-0.015640173	-7.44E-06	49.14656194
																			EITALAPSTMK	42	316659409	2xTMT6plex [N-Term; K11]	1.222	1.44	1.159	4.486789703	61.33	0	1619.94425	2	810.4758301	0.082321506	6.67E-05	67.20350059
																			EKLCYVALDFEQEMATAASSSSLEK	8	316659409	3xTMT6plex [N-Term; K2; K25]; 1xCarbamidomethyl [C4			0.211	4.246613026	40.14	1	3494.800183	4	874.4539795	-1.744131547	-0.001523848	101.7506571
																			KDLYANTVLSGGTTMYPGIADR	10	316659409	2xTMT6plex [N-Term; K1]; 1xOxidation [M15]	1.173	1.084	0.86	5.290822506	37.84	1	2817.485701	3	934.5020142	0.251152162	0.000234533	93.25141133
																			KDLYANTVLSGGTTMYPGIADR	39	316659409	2xTMT6plex [N-Term; K1]	1.275	1.103	1.564	7.366149902	64.22	1	2801.490786	3	934.5020142	0.251152162	0.000234533	93.25141133
																			VAPEEHPVLLTEAPLNPK	97	316659409	2xTMT6plex [N-Term; K18]	1.121	1.154	1.082	4.732988358	47.43	0	2412.390267	3	804.8043213	3.375679839	0.002714486	45.9233339
																			LCYVALDFEQEMATAASSSSLEK	55	316659409	2xTMT6plex [N-Term; K23]; 1xCarbamidomethyl [C2]; 1xOxidation [M12]	1.952	1.486	1.727	5.247089386	44.55	0	3024.494611	3	1003.505005	0.254574876	0.000255296	110.2411838
																			LCYVALDFEQEMATAASSSSLEK	208	316659409	2xTMT6plex [N-Term; K23]; 1xCarbamidomethyl [C2	1.277	1.353	1.284	6.348411083	76.62	0	3008.499696	3	1003.505005	0.254574876	0.000255296	110.2411838
																			KYSVWIGGSILASLSTFQQMWISKQEYDESGPSIVHR	6	316659409	3xTMT6plex [N-Term; K1; K24]	2.162		1.523	4.725753784	14.28	2	4915.605985	5	983.9237671	-3.307085806	-0.003251266	112.4623301
																			DLYANTVLSGGTTMYPGIADR	73	316659409	1xTMT6plex [N-Term]	1.248	1.165	1.05	5.963079929	69.35	0	2444.232891	3	815.4163208	0.621251089	0.000506161	81.3192129
																			DLYANTVLSGGTTMYPGIADR	49	316659409	1xTMT6plex [N-Term]; 1xOxidation [M14]	1.182	1.145	1.532	5.278543949	65.06	0	2460.227806	3	815.4163208	0.621251089	0.000506161	81.3192129
																			DSYVGDEAQSK	44	316659409	2xTMT6plex [N-Term; K11]	1.107	1.161	1.095	4.72834444	60.64	0	1656.848101	2	828.9279175	0.275976378	0.000228625	44.20964203
																			HQGVMVGMGQK	24	316659409	2xTMT6plex [N-Term; K11]	1.133	1.188	1.182	5.080441952	68.53	0	1629.896923	3	549.3018799	-0.456592314	-0.000250501	32.73665634
																			HQGVMVGMGQK	52	316659409	2xTMT6plex [N-Term; K11]; 2xOxidation [M5; M8	1.128	1.098	1.01	5.129117489	55.53	0	1661.886753	3	549.3018799	-0.456592314	-0.000250501	32.73665634
																			HQGVMVGMGQK	41	316659409	2xTMT6plex [N-Term; K11]; 1xOxidation [M	1.106	1.108	1.093	5.131017685	75.89	0	1645.891838	3	549.3018799	-0.456592314	-0.000250501	32.73665634
																			GYSFTTTAER	143	316659409	1xTMT6plex [N-Term]	1.183	1.218	1.149	4.540827751	67.95	0	1361.689861	2	681.3491821	0.90109198	0.000613504	37.07707612
																			QEYDESGPSIVHR	6	316659409	1xTMT6plex [N-Term]	0.923	2.302	100	3.69130969	42.15	0	1745.865593	3	582.6269531	0.408493645	0.000237725	33.11011339
																			YPIEHGIVTNWDDMEK	14	316659409	2xTMT6plex [N-Term; K16]	3.59	2.183	2.957	3.76259613	23.93	0	2405.221153	3	802.4120483	0.182444567	0.000146273	81.58947618
																			EITALAPSTMK	30	316659409	2xTMT6plex [N-Term; K11]; 1xOxidation [M10	1.059	1.146	1.148	3.327458143	52.93	0	1635.939165	2	810.4758301	0.082321506	6.67E-05	67.20350059
																			TTGIVMDSGDGVTHTVPIYEGYALPHAILR	8	316659409	1xTMT6plex [N-Term]; 1xOxidation [M6]		100		3.075448751	13.68	0	3428.772156	4	853.9509888	1.431024582	0.001220942	80.24707993
																			IWHHTFYNELR	48	316659409	1xTMT6plex [N-Term]	1.078	1.014	1.089	3.88049674	43.62	0	1744.91209	3	582.3091431	0.450549471	0.000262056	45.4901325
																			AGFAGDDAPR	7	316659409	1xTMT6plex [N-Term]	1.487	1.259		3.371614456	57.95	0	1205.611216	2	603.3093262	0.132093786	7.96E-05	32.14685543
																			CPEALFQPSFLGMESCGIHETTFNSIMK	13	316659409	2xTMT6plex [N-Term; K28]; 2xCarbamidomethyl [C1; C16]; 1xOxidation [M				2.55368185	17.31	0	3705.782553	4	927.2000732	-1.10363228	-0.001022455	85.43236938
																			CPEALFQPSFLGMESCGIHETTFNSIMK	10	316659409	2xTMT6plex [N-Term; K28]; 2xCarbamidomethyl [C1; C16	0.302			2.922416449	9.33	0	3689.787638	4	927.2000732	-1.10363228	-0.001022455	85.43236938
																			DLTDYLMK	55	316659409	2xTMT6plex [N-Term; K8]	1.087	1.087	1.065	3.984642267	41.49	0	1456.812173	2	736.9068604	-0.437522932	-0.000322193	64.73083726
																			DLTDYLMK	31	316659409	2xTMT6plex [N-Term; K8]; 1xOxidation [M7]	1.04	0.908	0.72	2.499874353	44.45	0	1472.807088	2	736.9068604	-0.437522932	-0.000322193	64.73083726
																			IKIIAPPER	1	316659409	2xTMT6plex [N-Term; K2]		1.029		2.575391769	0.47	1	1494.977205	3	498.9970093	-0.488842215	-0.000243603	55.327609
4503483	EEF2	elongation factor 2	1938	NP_001952.1	55.12820513	1	38	39	376	858	95.277	6.83	1.8	1.85	1.58	1.74	Significant	0.0023																
																			GHVFEESQVAGTPMFVVK	31	4503483	2xTMT6plex [N-Term; K18]	1.99	1.869	1.436	4.596632004	51.88	0	2420.304823	3	807.4416504	2.303388326	0.001858301	68.89350341
																			AYLPVNESFGFTADLR	24	4503483	1xTMT6plex [N-Term]	1.49	1.891	100	4.10464716	34.84	0	2029.059207	3	677.0244751	-0.165227821	-0.000111752	96.4533151
																			DLEEDHACIPIKK	16	4503483	3xTMT6plex [N-Term; K12; K13]; 1xCarbamidomethyl [C8	1.607	1.904	1.064	5.364313602	20.22	1	2255.267265	3	752.4278564	0.776553611	0.000583779	43.49911877
																			ETVSEESNVLCLSK	8	4503483	2xTMT6plex [N-Term; K14]; 1xCarbamidomethyl [C11	2.066	2.132		4.760384083	73.32	0	2053.088742	2	1027.046875	-1.1050667	-0.0011344	67.06497708
																			ALLELQLEPEELYQTFQR	22	4503483	1xTMT6plex [N-Term]	1.616	1.546	1.758	4.645280361	54.77	0	2449.317607	3	817.1102295	-0.600719741	-0.000490451	105.9539948
																			YVEPIEDVPCGNIVGLVGVDQFLVK	16	4503483	2xTMT6plex [N-Term; K25]; 1xCarbamidomethyl [C10	2.009	1.662	2.592	3.686154366	49.57	0	3217.758293	3	1073.252197	-5.051614088	-0.005418291	100.4332425
																			KEDLYLKPIQR	24	4503483	3xTMT6plex [N-Term; K1; K7]	1.862	1.833	1.67	3.824422836	14.65	1	2090.294072	3	697.4373169	1.591060148	0.001108595	49.56558113
																			NMSVIAHVDHGK	10	4503483	2xTMT6plex [N-Term; K12]	1.823	1.884	1.713	5.548277378	50.78	0	1765.978344	3	589.3302002	-1.300461126	-0.000765635	37.7630032
																			SDPMVQCIIEESGEHIIAGAGELHLEICLK	4	4503483	2xTMT6plex [N-Term; K30]; 2xCarbamidomethyl [C7; C28			1.459	4.528357506	31.66	0	3806.953123	4	952.491272	-2.591355621	-0.002466292	105.5474538
																			WLPAGDALLQMITIHLPSPVTAQK	12	4503483	2xTMT6plex [N-Term; K24]		1.831	2.042	3.309034348	36.23	0	3058.752754	4	765.444519	1.141619528	0.000872983	110.3254228
																			DLEEDHACIPIK	8	4503483	2xTMT6plex [N-Term; K12]; 1xCarbamidomethyl [C8	1.991	2.248	2.099	3.417535305	31.6	0	1898.00937	3	633.3354492	-9.259903576	-0.005858461	47.45817143
																			GEGQLGPAER	4	4503483	1xTMT6plex [N-Term]	1.796	1.552		3.628946304	47.53	0	1242.66398	2	621.8352661	-0.583161216	-0.000362337	31.96866666
																			STLTDSLVCK	8	4503483	2xTMT6plex [N-Term; K10]; 1xCarbamidomethyl [C9	1.732	100	2.112	3.809707403	63.04	0	1581.892215	2	791.4487305	-1.283647824	-0.001015296	62.388341
																			DSVVAGFQWATK	16	4503483	2xTMT6plex [N-Term; K12]	100	100	0.737	3.382055283	50.26	0	1766.984141	2	883.9954224	-0.324283294	-0.000286502	82.74831441
																			GVQYLNEIK	10	4503483	2xTMT6plex [N-Term; K9]	2.047	2.558	3.038	4.182044983	47.76	0	1521.9041	2	761.4567261	1.363783985	0.001037774	60.28117237
																			IWCFGPDGTGPNILTDITK	5	4503483	2xTMT6plex [N-Term; K19]; 1xCarbamidomethyl [C3			1.086	2.839053869	27.77	0	2563.363066	3	855.123291	-3.022046789	-0.002582201	100.6850409
																			IMGPNYTPGK	6	4503483	2xTMT6plex [N-Term; K10]	2.247	2.201	2.278	3.748728991	56.28	0	1535.865606	2	768.4362183	-0.290465885	-0.000223058	53.89183119
																			VNFTVDQIR	3	4503483	1xTMT6plex [N-Term]	2.418	2.913	100		45.48	0	1320.747316	2	660.8775024	0.312158736	0.000206141	60.04360974
																			EDLYLKPIQR	15	4503483	2xTMT6plex [N-Term; K6]	2.634	2.246	2.759	3.10496974	24.93	0	1733.036177	2	867.0216064	-0.138727268	-0.00012021	52.377898
																			EGALCEENMR	4	4503483	1xTMT6plex [N-Term]; 1xCarbamidomethyl [C5	2.976		0.997	3.894786835	55.4	0	1437.666365	2	719.3375854	1.063681028	0.000764609	36.03284562
																			IKPVLMMNK	8	4503483	3xTMT6plex [N-Term; K2; K9]	3.015	1.819	1.68	3.755853415	22.44	1	1761.109765	3	587.7073364	-1.311248567	-0.000769751	52.79139124
																			TFCQLILDPIFK	6	4503483	2xTMT6plex [N-Term; K12]; 1xCarbamidomethyl [C3	100	100	100	2.566299677	29.18	0	1953.128363	2	977.0661621	-1.697369878	-0.001657591	98.00071036
																			GPLMMYISK	20	4503483	2xTMT6plex [N-Term; K9]	1.252	100	1.601	2.576771021	45.77	0	1497.85735	2	749.4318237	-0.653426084	-0.00048937	73.56272615

Bhat et al,2017. Cigarette smoke and chewing tobacco alter expression of different sets of miRNAs in oral keratinocytes

Supplementary Table S2(A). List of all proteins and peptides identified in OKF6/TERT1 cells chronically treated with chewing tobacco using Sequest and Mascot search algorithms

Accession	Gene_Symbol	Description	Gene_ID	Refseq_Accession	Coverage	Proteins	#Unique_Peptides	#Peptides	#PSMS	#AAs	MW [kDa]	calc. pI	OKF6-STE-6M/OKF6P_R1	OKF6-STE-6M/OKF6P_R2	OKF6-STE-6M/OKF6P_R3	Median fold change	Status	P-value (6M)	Sequence	#PSMs	Protein Accessions	Modifications	OKF6-STE-6M/OKF6P_R1	OKF6-STE-6M/OKF6P_R2	OKF6-STE-6M/OKF6P_R3	Xcorr	Ionscore	# Missed Cleavages	Theo. MH+ [Da]	Charge	m_z	DeltaM [ppm]	Deltam/z [Da]	RT [min]
																			CELLYEGPPDDEAAMGIK	6	4503483	2xTMT6plex [N-Term; K18]; 1xCarbamidomethyl [C1				1.780209303	7.55	0	2466.229669	2	1233.617188	-1.042315739	-0.001285295	68.77051206
																			VFSGLVSTGLK	8	4503483	2xTMT6plex [N-Term; K11]		0.401	0.727	2.62675786	31.47	0	1565.9667	2	783.4853516	-2.090539345	-0.001636858	75.10440452
																			YEWDVAEAR	10	4503483	1xTMT6plex [N-Term]	1.98	1.598	1.978	3.918358088	48.91	0	1367.679296	2	684.3428345	-0.660713875	-0.000451822	48.9780839
																			QFAEMYVAK	6	4503483	2xTMT6plex [N-Term; K9]	1.737	3.205	100	3.266884804	37.11	0	1544.854707	2	772.930603	-0.503319379	-0.000388778	54.20740083
																			LDSEDKDKEGKPLLK	6	4503483	5xTMT6plex [N-Term; K6; K8; K11; K15]	1.033	0.967		3.737111807	7.63	2	2860.736816	3	954.2504883	0.033538125	3.20E-05	43.90671861
																			LMEPIYLVEIQCPEQVVGGIYGVLNR	4	4503483	1xTMT6plex [N-Term]; 1xCarbamidomethyl [C12				2.049598694	13.13	0	3218.715493	3	1073.575317	-1.272048297	-0.001364787	113.8290851
																			EGALCEENMR	4	4503483	1xTMT6plex [N-Term]; 1xCarbamidomethyl [C5]; 1xOxidation [M9]	2.822	0.947		2.531513691	45.84	0	1453.66128	2	719.3375854	1.063681028	0.000764609	36.03284562
																			IKPVLMMNK	10	4503483	3xTMT6plex [N-Term; K2; K9]; 1xOxidation [M	2.933	1.476	1.551	2.811733007	19.87	0	1777.10468	3	587.7073364	-1.311248567	-0.000769751	52.79139124
																			GGGQIIPTAR	2	4503483	1xTMT6plex [N-Term]	2.065			2.908091545	32.06	0	1198.710537	2	599.8590088	0.170481629	0.000102179	39.39309849
																			YFDPANGK	2	4503483	2xTMT6plex [N-Term; K8]	2.886			3.290526152	31.84	0	1369.751622	2	685.3804321	1.434900883	0.000982729	49.57856213
																			DGAGFLINLIDSPGHVDFSSEVTAALR	5	4503483	1xTMT6plex [N-Term]	100			1.881557941	7.21	0	3030.57338	3	1010.863403	0.751237687	0.000758894	114.5421684
																			AGIIASAR	4	4503483	1xTMT6plex [N-Term]		2.127	2.25	3.141519308	47.25	0	987.6148454	2	494.3078918	-6.41782434	-0.003169169	36.26531654
																			VFDAIMNFK	4	4503483	2xTMT6plex [N-Term; K9]			1.124	1.655241847	16.29	0	1542.875442	2	771.9406738	-0.88886874	-0.000685707	94.43032269
																			KIWCFGPDGTGPNILTDITK	2	4503483	3xTMT6plex [N-Term; K1; K20]; 1xCarbamidomethyl [C4				2.146843672	8.51	1	2920.620961	3	974.2106323	-1.238653489	-0.001205879	93.66638776
																			GHVFEESQVAGTPMFVVK	1	4503483	2xTMT6plex [N-Term; K18]; 1xOxidation [M14				1.686156154		0	2436.299738	3	807.4416504	2.303388326	0.001858301	68.89350341
																			NMSVIAHVDHGK	2	4503483	2xTMT6plex [N-Term; K12]; 1xOxidation [M2		1.013		2.968224287	1.14	0	1781.973259	3	589.3302002	-1.300641126	-0.000765635	37.7630032
																			IMGPNYTPGK	2	4503483	2xTMT6plex [N-Term; K10]; 1xOxidation [M2		2.543		2.44681716	22.11	0	1551.860521	2	768.4362183	-0.290465885	-0.000223058	53.89183119
																			TILMMGR	2	4503483	1xTMT6plex [N-Term]	3.284			2.325512886	19.51	0	1050.600123	2	525.8035889	-0.211541594	-0.000111123	58.69806163
																			SATSPEGK	4	4503483	2xTMT6plex [N-Term; K8]	1.901			2.782444715	34.21	0	1234.704338	2	617.8568115	1.626945669	0.001004398	27.77541021
																			IMGPNYTPGKK	3	4503483	3xTMT6plex [N-Term; K10; K11]	1.105	1.184		3.518986702	7.5	1	1893.123501	3	631.7111816	-2.38194081	-0.001503103	53.96316585
																			KVEDMMK	9	4503483	3xTMT6plex [N-Term; K1; K7]	1.463	1.663	1.575	2.9134202	27.76	1	1567.915482	2	784.4606934	-0.875105365	-0.000686046	38.29002904
32189394	ATP5B	ATP synthase subunit beta, mitochondrial precurso	506	NP_001677.2	79.58412098	1	26	26	281	529	56.525	5.4	0.55	0.52	0.75	0.61	Significant	0.014																
																			AHGGYSVFAGVGER	18	32189394	1xTMT6plex [N-Term]	0.666	0.679	0.642	5.606964111	65.07	0	1635.84407	3	545.9528198	-0.099977343	-5.45E-05	42.30240686
																			VALTGLTVAEYFR	15	32189394	1xTMT6plex [N-Term]	0.924	0.993	0.752	5.138283253	40.51	0	1668.952223	2	834.9782104	-1.844835053	-0.001539471	85.82689164
																			SLQDIIAILGMDELSEEDKLTVSR	8	32189394	2xTMT6plex [N-Term; K19]	0.482	0.527	0.839	5.019659042	52.71	1	3133.706651	4	788.1799316	-1.16509689	-0.000917427	110.3242943
																			SLQDIIAILGMDELSEEDKLTVSR	8	32189394	2xTMT6plex [N-Term; K19]; 1xOxidation [M11	1.727	0.746	0.697	4.401648045	54.24	1	3149.701566	4	788.1799316	-1.16509689	-0.000917427	110.3242943
																			DQEGQDVLLFIDNIFR	6	32189394	1xTMT6plex [N-Term]	100	0.546		2.647993803	49.33	0	2151.128349	2	1076.068481	0.621474133	0.000668435	116.8876484
																			IPSAVGYQPTLATDMGTMQER	12	32189394	1xTMT6plex [N-Term]	0.736	0.733	0.429	4.960816383	57.44	0	2495.247161	3	832.4193726	-1.441177562	-0.001198698	76.86200151
																			IPSAVGYQPTLATDMGTMQER	10	32189394	1xTMT6plex [N-Term]; 1xOxidation [M	0.675	0.722	0.01	3.893739223	42.51	0	2511.242076	3	832.4193726	-1.441177562	-0.001198698	76.86200151
																			GFQQILAGEYDHLPEQAFYMVGPIEEAVAK	22	32189394	2xTMT6plex [N-Term; K30]	0.7	0.654	0.787	7.057177067	69.62	0	3808.966054	3	1270.325195	-1.318229784	-0.001673697	109.7026773
																			GFQQILAGEYDHLPEQAFYMVGPIEEAVAK	10	32189394	2xTMT6plex [N-Term; K30]; 1xOxidation [M20	0.01		0.873	6.462920666	40.3	0	3824.960969	3	1270.325195	-1.318229784	-0.001673697	109.7026773
																			TIAMDGTEGLVR	19	32189394	1xTMT6plex [N-Term]	0.752	0.748	0.985	4.257246494	75.83	0	1491.803844	2	746.4063721	1.087978578	0.000811525	53.07650461
																			IMDPNIVGSEHYDVAR	10	32189394	1xTMT6plex [N-Term]; 1xOxidation [M2]	0.747	0.687	0.749	4.25225687	53.46	0	2061.027256	3	682.3494263	0.677230656	0.000461653	57.67555969
																			IMDPNIVGSEHYDVAR	16	32189394	1xTMT6plex [N-Term]	0.7	0.84	0.916	4.05000639	57.57	0	2045.032341	3	682.3494263	0.677230656	0.000461653	57.67555969
																			GSITSVQAIYVPADDLTDPAPATTFAHLDATTVLSR	4	32189394	1xTMT6plex [N-Term]			0.547	5.857236385	37.7	0	3944.049042	4	986.7714233	3.757924716	0.00370536	92.32998231
																			QFAPIHAEAPEFMEMSVEQEILVTGIK	4	32189394	2xTMT6plex [N-Term; K27]			0.748	6.223758221	49.35	0	3502.83662	4	884.4588623	-3.629689161	-0.00320758	88.31831464
																			QFAPIHAEAPEFMEMSVEQEILVTGIK	4	32189394	2xTMT6plex [N-Term; K27]; 2xOxidation [M13; M15			0.887	5.903302193	53.41	0	3534.82645	4	884.4588623	-3.629689161	-0.00320758	88.31831464
																			TIAMDGTEGLVR	6	32189394	1xTMT6plex [N-Term]; 1xOxidation [M4]	0.655	0.777	0.735	3.062193155	40.2	0	1507.798759	2	746.4063721	1.087978578	0.000811525	53.07650461
																			FTQAGSEVSALLGR	7	32189394	1xTMT6plex [N-Term]	0.538		100	3.121110439	44.63	0	1664.9169	2	832.9614258	-0.796050241	-0.000662679	74.72307884
																			LVLEVAQHLGESTVR	18	32189394	1xTMT6plex [N-Term]	0.925	0.849	0.519	3.747294188	37.95	0	1880.080277	3	627.3640137	-1.483641102	-0.000929788	74.98649487
																			FLSQPFQVAEVFTGHMGK	6	32189394	2xTMT6plex [N-Term; K18]	100	0.478	0.859	3.294074774	19.82	0	2481.336458	3	827.7833252	-0.417184078	-0.000345058	98.29371755
																			TVLIMELINNVAK	6	32189394	2xTMT6plex [N-Term; K13]	0.756		0.404	2.488372087	37.04	0	1916.165476	2	958.5847778	-1.668528098	-0.001598588	119.1384255
																			IGLFGGAGVGK	6	32189394	2xTMT6plex [N-Term; K11]	1.356	2.229	1.645	3.317286015	52.59	0	1433.888056	2	717.4467163	-1.325112379	-0.000950031	63.74904494
																			IVAVIGAVVDVQFDEGLPPILNALEVQGR	2	32189394	1xTMT6plex [N-Term]				1.908960462	19.21	0	3260.845579	3	1087.619873	-0.157370033	-0.000171053	118.7648418
																			AIAELGIYPAVDPLDSTSR	10	32189394	1xTMT6plex [N-Term]	0.752	0.387	0.527	2.634233713	22.83	0	2217.196429	2	1109.100952	-0.812387708	-0.000900612	79.73586011
																			EGNDLYHEMIESGVINLK	4	32189394	2xTMT6plex [N-Term; K18]	0.467			3.202148438	20.18	0	2519.321595	3	840.4470825	2.024012577	0.001699713	95.48700583
																			VLDSGAPIK	4	32189394	2xTMT6plex [N-Term; K9]	0.81		0.973	3.735787392	61.63	0	1357.845523	2	679.427124	1.067048387	0.000724443	50.27067203
																			IMNVIGEPIDER	10	32189394	1xTMT6plex [N-Term]	0.762	0.93		4.120880127	46.37	0	1614.872258	2	807.9391479	-0.767281471	-0.000619531	68.12795473
																			VVDLLAPYAK	11	32189394	2xTMT6plex [N-Term; K10]	0.564	0.664	0.795	3.603881121	37.51	0	1546.960887	2	773.9830322	-1.356696794	-0.001049378	85.85596988
																			VALVYGQMNEPPGAR	6	32189394	1xTMT6plex [N-Term]	1.285	100	0.468	2.493558884	27.62	0	1830.973369	2	915.9880371	-2.49686933	-0.002285851	55.28059484
																			IPVGPETLGR	4	32189394	1xTMT6plex [N-Term]	0.596	0.701		2.857839108	30.26	0	1267.757152	2	634.3822632	0.076779048	4.87E-05	63.89442533
																			VLDSGAPIKIPVGPETLGR	4	32189394	2xTMT6plex [N-Term; K9]	0.01		0.691	2.403657913	2.66	1	2377.421902	3	793.1452026	-0.356264003	-0.00028233	65.95700932
																			LVPLKETIK	3	32189394	3xTMT6plex [N-Term; K5; K9]	0.206	0.271		1.911246061	0.27	1	1728.160204	3	576.7259521	1.793285892	0.001033028	60.25234214
																			ADKLAEEHSS	8	32189394	2xTMT6plex [N-Term; K3]	0.278	0.29		2.718723297	33.82	1	1544.832057	2	772.9193115	-0.460077924	-0.000355372	32.07182931
4557701	KRT17	keratin, type I cytoskeletal 17	3872	NP_000413.1	79.86111111	1	21	35	795	432	48.076	5.02	0.53	0.57	0.54	0.55	Significant	0.00048																
																			GQVGGEINVEMDAAPGVDLSR	99	4557701	1xTMT6plex [N-Term]	1.258	100	100	3.173252821	36.46	0	2343.181189	2	1172.093628	-0.516486147	-0.00060511	73.01806462
																			LLEGEDAHLTQYK	32	4557701	2xTMT6plex [N-Term; K13]	0.58	0.6	0.596	4.980884075	60.9	0	1975.090063	2	988.048645	-0.024895542	-2.46E-05	63.50793901
																			LSGGLGAGSCR	15	4557701	1xTMT6plex [N-Term]; 1xCarbamidomethyl [C10	0.712	0.628	0.282	4.20837307	69.35	0	1263.667686	2	632.3372192	-0.414605393	-0.000261962	48.04282829
																			KNHEEEMNALR	11	4557701	2xTMT6plex [N-Term; K1]	0.773	0.864	0.797	3.628271818	36.26	1	1828.973987	3	610.3292847	-0.375246503	-0.000228772	35.80008094
																			ILTATVDNANILLQIDNAR	6	4557701	1xTMT6plex [N-Term]	0.902	100		4.402533054	55.2	0	2297.302625	3	766.4372559	-2.355341353	-0.001803644	100.5787228
																			ALEEANTELEVK	70	4557701	2xTMT6plex [N-Term; K12]	0.447	0.381	0.378	4.865480423	82.28	0	1804.010415	2	902.5084229	-0.469086469	-0.000423118	64.4672149
																			ASLEGNLAETENR	71	4557701	1xTMT6plex [N-Term]	0.56	0.561	0.596	5.075448513	66.82	0	1632.839044	2	816.9234009	0.294749084	0.000240639	54.30994029
																			YCVQLSQIQGLIGSVEEQLAQLR	34	4557701	1xTMT6plex [N-Term]; 1xCarbamidomethyl [C2	0.825	0.672		5.34431982	49.69	0	2861.539244	3	954.5184326	0.524560886	0.000500351	114.0442126
																			EVATNSELVQSGK	82	4557701	2xTMT6plex [N-Term; K13]	0.696	0.701	0.693	5.450167656	73.38	0	1820.016563	2	910.5032349	-9.54398646	-0.008685107	43.74892866
																			LGSAGGLGSTLGGSSYSSCYSFGSGGGYGSSFGGVDGLLAGGEK	6	4557701	2xTMT6plex [N-Term; K44]; 1xCarbamidomethyl [C19]			1.025	5.426093102	40.31	0	4483.140008	4	1121.539673	-0.701799128	-0.000786566	94.55615977
																			GSSGLGGGSSR	16	4557701	1xTMT6plex [N-Term]	0.618	0.355		3.998183489	70.61	0	1150.60138	2	575.8041382	-0.330516562	-0.000190146	17.14726948
																			TIVEEVQDGK	36	4557701	2xTMT6plex [N-Term; K10]	0.476	0.55	0.589	4.154781818	68.64	0	1575.899408	2	788.4528809	-0.585913825	-0.000461671	46.671743
																			ADLEMQIENLKEELAYLKK	2	4557701	4xTMT6plex [N-Term; K11; K18; K19]	100			2.940661907	17.84	2	3194.851638	4	799.4646606	-4.640428901	-0.00370637	105.5080342
																			ADLEMQIENLK	8	4557701	2xTMT6plex [N-Term; K11]	100	2.31	0.209	2.574895859	42.79	0	1761.982092	2	881.4951782	0.560542197	0.000493833	86.66240089
																			NHEEEMNALR	8	4557701	1xTMT6plex [N-Term]		0.962	1.027	4.156452179	41.93	0	1471.716092	2	736.3625488	1.17476887	0.000864463	28.88820698
																			TMQALEIELQSQLSMK	10	4557701	2xTMT6plex [N-Term; K16]	0.401			2.73297143	27.14	0	2308.26566	3	770.0934448	0.052378138	4.03E-05	100.8391276
																			QFTSSSSIK	18	4557701	2xTMT6plex [N-Term; K9]	0.533	0.523	0.533	4.347120762	58.63	0	1442.825515	2	721.916687	0.403439939	0.000291047	39.65630323
																			LEQEIATYR	30	4557701	1xTMT6plex [N-Term]	0.873	0.965	0.895	3.989738941	51.78	0	1351.741896	2	676.3740845	-0.742660038	-0.000501942	59.56020918
																			ATMQNLNDR	8	4557701	1xTMT6plex [N-Term]	0.559	0.52	0.613	3.764918089	44.3	0	1291.6626	2	646.3349609	0.034982046	2.26E-05	31.80804879
																			ADLEMQIENLKEELAYLK	6	4557701	3xTMT6plex [N-Term; K11; K18]	100	0.01	0.602	2.279923201	16.18	1	2837.593743	3	946.5266724	-9.965972128	-0.00942646	103.5103069
																			EVATNSELVQSGKSEISELR	2	4557701	2xTMT6plex [N-Term; K13]	0.01			3.065496922	15.76	1	2634.435045	3	878.815918	-0.700037662	-0.000614735	60.33944928
																			VLDELTLAR	31	4557701	1xTMT6plex [N-Term]	0.836	0.802	0.88	3.858995199	41.24	0	1258.756818	2	629.8816528	-0.626805691	-0.000394498	67.98248171
																			NHEEEMNALR	12	4557701	1xTMT6plex [N-Term]; 1xOxidation [M6]	100	1.113	1.026	3.034799576	35.28	0	1487.711007	2	736.3625488	1.17476887	0.000864463	28.88820698
																			CEMEQQNQEYK	10	4557701	2xTMT6plex [N-Term; K11]; 1xCarbamidomethyl [C1]; 1xOxidation [M3]	1.819	0.875	0.895	3.481099367	43.3	0	1960.914483	3	654.3094482	-0.352898325	-0.000230668	30.65856668
																			CEMEQQNQEYK	14	4557701	2xTMT6plex [N-Term; K11]; 1xCarbamidomethyl [C1	0.885	0.803	0.891	3.460950851	27.46	0	1944.919568	3	654.3094482	-0.352898325	-0.000230668	30.65856668
																			TKFETEQALR	22	4557701	2xTMT6plex [N-Term; K2]	0.708	0.654	0.591	3.654014587	25.22	1	1680.968491	2	840.9873657	-0.616445039	-0.000518112	41.51534922
																			KNHEEEMNALR	13	4557701	2xTMT6plex [N-Term; K1]; 1xOxidation [M7]	0.891	0.645	0.944	3.066138506	19.28	1	1844.968902	3	610.3292847	-0.375246503	-0.000228772	35.80008094
																			EQVHQTTR	3	4557701	1xTMT6plex [N-Term]	0.386			3.034813166	42.69	0	1227.664315	3	409.8927917	-0.401295251	-0.000164219	16.5946865
																			TIEELQNK	6	4557701	2xTMT6plex [N-Term; K8]	0.602	0.733		3.585800648	44.38	0	1432.841165	2	716.9244995	0.38876147	0.000278517	43.670601
																			LSVEADINGLRR	2	4557701	1xTMT6plex [N-Term]	1.88			2.779716253	19.64	1	1571.90667	3	524.6399536	-0.866730947	-0.00045414	54.94249371
																			KDAEDWFFSK	14	4557701	3xTMT6plex [N-Term; K1; K10]	0.678	0.483	0.485	3.026944876	19.82	1	1960.078325	2	980.5419922	-0.825025704	-0.000808557	65.71699562
																			TMQALEIELQSQLSMK	6	4557701	2xTMT6plex [N-Term; K16]; 1xOxidation [M15				1.726732373	18.31	0	2324.260575	3	770.0934448	0.052378138	4.03E-05	100.8391276
																			EELAYLK	16	4557701	2xTMT6plex [N-Term; K7]	0.771	1.132	1.244	3.250023842	39.59	0	1323.792424	2	662.3997803	-0.106083947	-7.02E-05	54.24797047
																			FETEQALR	6	4557701	1xTMT6plex [N-Term]	1.009	1.115	0.896	3.28198266	44.28	0	1222.662918	2	611.8347168	-0.622171687	-0.000380353	43.64425593
																			DAEDWFFSK	2	4557701	2xTMT6plex [N-Term; K9]	100			1.879194617	14.86	0	1602.82043	2	801.9136963	-0.19587464	-0.000156976	84.39377391
																			LSVEADINGLR	4	4557701	1xTMT6plex [N-Term]				2.269045353	25.66	0	1415.805559	2	708.4070435	0.88376829	0.000625622	69.32299519
																			LASYLDK	51	4557701	2xTMT6plex [N-Term; K7]	0.689	0.687	0.676	3.160550594	38.22	0	1267.76621	2	634.3873291	0.924415808	0.000585972	69.69252583
																			SEISELRR	2	4557701	1xTMT6plex [N-Term]		0.339		3.65511775	23.66	1	1218.700366	3	406.9051514	0.439034547	0.000178351	31.29536983
																			ADLEMQIENLKEELAYLK	2	4557701	3xTMT6plex [N-Term; K11; K18]; 1xOxidation [M5				1.476866007	6.19	1	2853.588658	3	946.5266724	-9.965972128	-0.00942646	103.5103069
																			SEISELR	9	4557701	1xTMT6plex [N-Term]	0.814	0.792	0.706	2.968187571	40.38	0	1062.599255	2	531.8031006	-0.310830375	-0.000165144	43.56074208
384475521	TKT	transketolase isoform 2	7086	NP_001244957.1	61.8066561	1	27	27	307	631	68.77	7.52	1.4	1.56	1.56	1.51	Significant	0.0013																
																			GITGVEDKESWHGKPLPK	18	384475521	4xTMT6plex [N-Term; K8; K14; K18]	0.77	1.092	0.99	6.008454323	21.08	1	2894.690979	3	965.5690308	0.538915956	0.000519998	45.93012659
																			ISSDLDGHPVPK	14	384475521	2xTMT6plex [N-Term; K12]	2.032	2.014	2.038	3.733355761	55.94	0	1722.979056	2	861.9923706	-0.923469764	-0.00079556	39.91217785
																			QAFTDVATGSLGQGLGAACGMAYTGK	6	384475521	2xTMT6plex [N-Term; K26]; 1xCarbamidomethyl [C19]; 1xOxidation [M21]	100			3.112524033	35.07	0	3006.506513	3	997.5084229	-0.295048283	-0.000294115	81.57352411
																			QAFTDVATGSLGQGLGAACGMAYTGK	12	384475521	2xTMT6plex [N-Term; K26]; 1xCarbamidomethyl [C19	2.556	100		5.433049679	50.45	0	2990.511598	3	997.5084229	-0.295048283	-0.000294115	81.57352411
																			KISSDLDGHPVPK	21	384475521	3xTMT6plex [N-Term; K1; K13]	1.446	1.557	1.49	4.324873924	28.21	1	2080.236951	3	694.0834961	-0.488232242	-0.000338546	36.54188036
																			LGQSDPAPLQHQMDIYQK	2	384475521	2xTMT6plex [N-Term; K18]; 1xOxidation [M13		3.01		4.303319931	40.71	0	2543.332829	3	848.4488525	-0.324221353	-0.000274868	48.54559155
																			LGQSDPAPLQHQMDIYQK	6	384475521	2xTMT6plex [N-Term; K18]	7.375	100	100	3.839419603	26.65	0	2527.337914	3	848.4488525	-0.324221353	-0.000274868	48.54559155
																			ILTVEDHYYEGGIGEAVSSAVVGEPGITVTHLAVNR	21	384475521	1xTMT6plex [N-Term]	100	100	1.598	8.397192955	53	0	3982.075925	4	996.2747192	0.281655384	0.000280393	91.62917935
																			TSRPENAIIYNNNEDFQVGQAK	8	384475521	2xTMT6plex [N-Term; K22]	2.636	2.916		5.879769325	45.43	0	2966.537218	3	989.5171509	-0.107544001	-0.000106344	57.53758109
																			LDNLVAILDINR	8	384475521	1xTMT6plex [N-Term]	2.878	1.204	100	4.178573132	40.08	0	1597.947472	2	799.4771729	-0.25226541	-0.000201553	100.3874303
																			MAAISESNINLCGSHCGVSIGEDGPSQMALEDLAMFR	6	384475521	1xTMT6plex [N-Term]; 2xCarbamidomethyl [C12; C16	0.873		1.089	6.633712769	44.51	0	4196.93807	4	1053.989136	0.410079808	0.00043191	93.41599876
																			MAAISESNINLCGSHCGVSIGEDGPSQMALEDLAMFR	10	384475521	1xTMT6plex [N-Term]; 2xCarbamidomethyl [C12; C16]; 1xOxidation [M			1.531	5.802956104	45.27	0	4212.932985	4	1053.989136	0.410079808	0.00043191	93.41599876
																			MAAISESNINLCGSHCGVSIGEDGPSQMALEDLAMFR	7	384475521	1xTMT6plex [N-Term]; 2xCarbamidomethyl [C12; C16]; 2xOxidation [M			1.199	5.151881218	34.38	0	4228.9279	4	1053.989136	0.410079808	0.00043191	93.41599876
																			MAAISESNINLCGSHCGVSIGEDGPSQMALEDLAMFR	6	384475521	1xTMT6plex [N-Term]; 2xCarbamidomethyl [C12; C16]; 3xOxidation [M1; M28; M3			3.296	4.851617813	26.78	0	4244.922815	4	1053.989136	0.410079808	0.00043191	93.41599876
																			SKDDQVTVIGAGVTLHEALAAAELLK	8	384475521	3xTMT6plex [N-Term; K2; K26]	100	2.642	1.607	8.471651077	53.94	1	3336.934567	4	834.9888306	-0.32202141	-0.000268641	101.1318175
																			NMAEQIIQEIYSQIQSK	9	384475521	2xTMT6plex [N-Term; K17]	1.996	2.067		3.841269493	48.68	0	2481.342331	3	827.7838135	-2.193752246	-0.001814483	115.8746206
																			VYCLLGDGELSEGSVWEAMAFASIYK	4	384475521	2xTMT6plex [N-Term; K26]; 1xCarbamidomethyl [C3		3.486	2.689	4.05480957	45	0	3353.683808	3	1118.561646	-4.002919575	-0.004474842	116.6045372
																			FIECYIAEQNMVSIAVGCATR	4	384475521	1xTMT6plex [N-Term]; 2xCarbamidomethyl [C4; C18			3.944	5.806216717	59.65	0	2661.30363	3	887.7703857	-2.640120939	-0.002342054	100.0724357
																			KAYGQALAK	10	384475521	3xTMT6plex [N-Term; K1; K9]	1.133	1.212	1.282	4.774761677	38.84	1	1637.035338	3	546.3497925	-0.313865304	-0.00017127	38.18462718
																			VLDPFTIKPLDR	24	384475521	2xTMT6plex [N-Term; K8]	1.657	1.27	1.898	3.571093798	23.77	0	1872.135891	3	624.7183228	2.416668438	0.001508111	4.747422229
																			AVELAANTK	12	384475521	2xTMT6plex [N-Term; K9]	1.51	1.497		3.69697547	58.93	0	1374.835686	2	687.9215698	0.128683332	8.85E-05	49.20221959
																			ILATPPQEDAPSVDIANIR	18	384475521	1xTMT6plex [N-Term]	2.23	2.194	100	2.983245611	23.28	0	2249.233877	3	750.4151001	-1.391574733	-0.001043326	66.43101824
																			IIALDGDTK	12	384475521	2xTMT6plex [N-Term; K9]	1.563	1.229	2.038	3.699113131	46.19	0	1403.851002	2	702.4281616	-1.392724591	-0.000977589	52.36914032
																			NSTFSEIFK	4	384475521	2xTMT6plex [N-Term; K9]	100	100		3.002596378	27.01	0	1530.856815	2	765.9310303	-1.327036666	-0.001015752	61.59891588
																			SVPTSTVFYPSDGVATEK	14	384475521	2xTMT6plex [N-Term; K18]	2.652	2.659	1.838	2.874649763	22.31	0	2343.248414	2	1172.126343	-1.282229877	-0.001502292	56.86255746
																			GITGVEDK	8	384475521	2xTMT6plex [N-Term; K8]	1.673	1.843	1.78	3.493388176	43.69	0	1276.751288	2	638.8731689	-9.576289048	-0.00611327	39.63599755
																			SGKPAELLK	2	384475521	3xTMT6plex [N-Term; K3; K9]	1.46	0.793		2.079692125		0	1630.050654	3	544.0217896	0.099206944	5.39E-05	50.70919366
																			AYGQALAK	5	384475521	2xTMT6plex [N-Term; K8]		2.362	2.377	3.540707588	40.32	0	1279.777443	2	640.392334	-0.040390812	-2.58E-05	36.20743513
																			MPSLPSYK	10	384475521	2xTMT6plex [N-Term; K8]	1.831	1.727	1.553	3.212301254	36.25	0	1380.79613	2	690.9017944	0.132312934	9.13E-05	56.4818516
																			KLILDSAR	10	384475521	2xTMT6plex [N-Term; K1]	1.16	1.127	1.314	3.046549082	30.95	1	1373.888056	2	687.4473267	-0.494479652	-0.00033968	44.36570981
																			DAIAQAVR	2	384475521	1xTMT6plex [N-Term]			1.722	2.615951061	41.82	0	1072.631224	2	536.819458	0.387547571	0.000207848	37.5275548
																			LILDSAR	6	384475521	1xTMT6plex [N-Term]	1.761	1.739	1.159	2.683711767	31.19	0	1016.630161	2	508.8189392	0.433488977	0.000220349	45.17717876
24234699	KRT19	keratin, type I cytoskeletal 19	3880	NP_002267.2	76.5	1	21	32	605	400	44.079	5.14	0.55	0.57	0.52	0.55	Significant	0.00071																
																			QSSATSSFGGLGGGSVR	14	24234699	1xTMT6plex [N-Term]	0.621	0.684	0.66	5.488426685	108.42	0	1783.913606	2	892.454895	-6.217964201	-0.005546155	41.35669145
																			EVAGHTEQLQMSR	50	24234699	1xTMT6plex [N-Term]	0.452	0.485	0.461	4.785831451	52.13	0	1714.874384	3	577.6282959	0.598488467	0.000345302	29.93780884
																			EVAGHTEQLQMSR	48	24234699	1xTMT6plex [N-Term]; 1xOxidation [M11]	0.519	0.493	0.447	3.920547724	56.85	0	1730.869299	3	577.6282959	0.598488467	0.000345302	29.93780884
																			SLLEGQEDHYNNLSASK	28	24234699	2xTMT6plex [N-Term; K17]	0.58	0.596	0.599	6.303720474	80.93	0	2363.224324	2	1182.114014	-1.512030074	-0.001786633	46.86231051
																			DKILGATIENSR	12	24234699	2xTMT6plex [N-Term; K2]	0.858	0.609	0.45	4.764064312	46.2	1	1775.042719	2	888.0255127	0.580392018	0.00051511	44.77092583
																			TLQGLEIELQSQLSMK	6	24234699	2xTMT6plex [N-Term; K16]	0.593			3.152331829	38.79	0	2276.29359	3	759.4346313	-1.866554934	-0.001416276	99.28319653
																			FGAQLAHIQALISGIEAQLGDVR	3	24234699	1xTMT6plex [N-Term]		100		3.474964857	25.23	0	2636.47215	3	879.4956055	0.042885433	3.77E-05	112.3814183
																			MSVEADINGLR	6	24234699	1xTMT6plex [N-Term]	1.436	1.688		3.755783319	62.24	0	1433.76198	2	717.3839111	-1.000196961	-0.000717022	64.76776505
																			KNHEEEISTLR	12	24234699	2xTMT6plex [N-Term; K1]	0.629	0.71	0.608	4.582782269	36.79	1	1814.017232	3	605.3427734	-1.910123806	-0.001154999	30.54243413
																			GQVGGQVSVEVDSAPGTDLAK	6	24234699	2xTMT6plex [N-Term; K21]	0.649			4.652263641	53.66	0	2472.334603	3	824.7820435	-1.223749772	-0.001008506	59.01409333
																			DYSHYYTTIQDLR	19	24234699	1xTMT6plex [N-Term]	0.665	0.56	0.566	4.14150095	38.76	0	1903.938758	3	635.3170166	-1.187693303	-0.000753765	61.10785278
																			ALEAANGELEVK	6	24234699	2xTMT6plex [N-Term; K12]	0.69	0.719		5.177536964	69.02	0	1701.978721	2	851.4931641	0.193988912	0.000165083	54.35657224
																			AALEDTLAETEAR	20	24234699	1xTMT6plex [N-Term]	0.484	0.485	0.487	5.146541595	78.95	0	1618.848546	2	809.9290161	1.364974239	0.001104843	59.9151859
																			ILGATIENSR	9	24234699	1xTMT6plex [N-Term]	0.873	0.888	0.854	3.714605331	49.28	0	1302.757881	2	651.8825684	-0.015767512	-1.03E-05	45.65701127
																			SQYEVMAEQNR	8	24234699	1xTMT6plex [N-Term]	0.547	0.556		4.148043633	61.33	0	1583.768522	2	792.3866577	-1.567615645	-0.00124137	41.71599068
																			TDLEMQIEGLK	4	24234699	2xTMT6plex [N-Term; K11]	0.859	0.946		3.609122753	47.74	0	1734.971193	2	867.9890747	-0.18463473	-0.000160168	84.89662329
																			TLQGLEIELQSQLSMK	6	24234699	2xTMT6plex [N-Term; K16]; 1xOxidation [M15			100	1.997135758	22.42	0	2292.288505	3	759.4346313	-1.866554934	-0.001416276	99.28319653
																			LEQEIATYR	30	24234699	1xTMT6plex [N-Term]	0.873	0.965	0.895	3.989738941	51.78	0	1351.741896	2	676.3740845	-0.742660038	-0.000501942	59.56020918
																			DYSHYYTTIQDLRDKILGATIENSR	13	24234699	2xTMT6plex [N-Term; K15]	0.636	0.863	0.424	3.007154226	14.5	2	3430.800703	5	686.9645386	-2.074366751	-0.001423348	91.54526027
																			KDAEAWFTSR	24	24234699	2xTMT6plex [N-Term; K1]	0.56	0.527	0.549	4.115560532	25.8	1	1668.910976	2	834.9575806	-1.85252373	-0.001545849	54.55995725
																			MSVEADINGLRR	4	24234699	1xTMT6plex [N-Term]		3.428		3.775520086	34.04	1	1589.863091	3	530.6262207	0.640438401	0.000339834	47.35430851
																			IVLQIDNAR	27	24234699	1xTMT6plex [N-Term]	0.822	0.805	0.786	3.429313183	48.49	0	1270.768051	2	635.8876343	-0.046826258	-2.98E-05	71.78171565
																			DAEAWFTSR	29	24234699	1xTMT6plex [N-Term]	0.559	0.593	0.424	3.542104959	51.86	0	1311.653081	2	656.3303833	0.311615601	0.000204366	67.15413911
																			VLDELTLAR	31	24234699	1xTMT6plex [N-Term]	0.836	0.802	0.88	3.858995199	41.24	0	1258.756818	2	629.8816528	-0.626805691	-0.000394498	67.98248171
																			ITMQNLNDR	28	24234699	1xTMT6plex [N-Term]	0.566	0.576	0.616	3.402525187	42.38	0	1333.70955	2	667.3587646	0.526671549	0.000351213	48.81082799
																			TKFETEQALR	22	24234699	2xTMT6plex [N-Term; K2]	0.708	0.654	0.591	3.654014587	25.22	1	1680.968491	2	840.9873657	-0.616445039	-0.000518112	41.51534922
																			GVSVSSAR	26	24234699	1xTMT6plex [N-Term]	0.569	0.595	0.639	2.983020306	43.88	0	991.5733745	2	496.2902222	-0.208531278	-0.000103387	31.52132297
																			LTMQNLNDR	16	24234699	1xTMT6plex [N-Term]; 1xOxidation [M3]	0.656	0.624	0.59	1.89945972	33.06	0	1349.704465	2	667.3587646	0.526671549	0.000351213	48.81082799
																			FGPGVAFR	6	24234699	1xTMT6plex [N-Term]		0.716	0.927	2.564065933	37.46	0	1079.619931	2	540.3148804	2.365010236	0.001276656	57.59080944
																			APSIHGGSGGR	6	24234699	1xTMT6plex [N-Term]			0.471	3.195515394	40.14	0	1224.664649	3	408.8927612	-0.750065411	-0.000306193	23.35403307
																			SQYEVMAEQNR	19	24234699	1xTMT6plex [N-Term]; 1xOxidation [M6]	0.532	0.842	0.472	2.993422747	41.21	0	1599.763437	2	792.3866577	-1.567615645	-0.00124137	41.71599068
																			EELAYLK	16	24234699	2xTMT6plex [N-Term; K7]	0.771	1.132	1.244	3.250023842	39.59	0	1323.792424	2	662.3997803	-0.106083947	-7.02E-05	54.24797047
																			FETEQALR	6	24234699	1xTMT6plex [N-Term]	1.009	1.115	0.896	3.28198266	44.28	0	1222.662918	2	611.8347168	-0.622171687	-0.000380353	43.64425593
																			LASYLDK	51	24234699	2xTMT6plex [N-Term; K7]	0.689	0.687	0.676	3.160550594	38.22	0	1267.76621	2	634.3873291	0.924415808	0.000585972	69.69252583
																			MSVEADINGLR	4	24234699	1xTMT6plex [N-Term]; 1xOxidation [M1]	1.373	1.227		2.568712234	27.3	0	1449.756895	2	717.3839111	-1.000196961	-0.000717022	64.76776505
																			IRDWYQK	10	24234699	2xTMT6plex [N-Term; K7]	0.425	0.4	0.392	3.265621901	11.89	1	1466.852005	3	489.6218567	-0.673613723	-0.000329364	52.65410183
																			SQYEVMAEQNRK	2	24234699	2xTMT6plex [N-Term; K12]		1.288		1.790570974	0	1	1941.026417	3	647.6796875	-0.982603835	-0.000635753	41.88122149
27436946	LMNA	lamin isoform A	4000	NP_733821.1	54.6686747	1	3	38	469	664	74.095	7.02	0.72	0.77	0.71	0.74	Significant	0.00064																
																			SNEDQSMGNWQIK	6	27436946	2xTMT6plex [N-Term; K13]	0.886	100	1.025	4.09638834	38.99	0	1995.000596	2	997.9941406	-9.820057218	-0.00979551	51.57611777
																			NSNLVGAAHEELQQSR	10	27436946	1xTMT6plex [N-Term]	0.849	0.788		5.859630108	61.98	0	1982.025281	3	661.3460693	-0.820671766	-0.000542197	42.07806646
																			EAALSTALSEK	12	27436946	2xTMT6plex [N-Term; K11]	0.878	0.809	0.795	4.230268002	50.09	0	1577.915058	2	789.4609375	-0.29159998	-0.00023006	52.00948651
																			MQQQLDEYQELLDIK	8	27436946	2xTMT6plex [N-Term; K15]	0.799	100	0.67	3.99401021	44.09	0	2352.252119	3	784.7553711	-0.237503771	-0.000186223	92.75514387
																			TALINSTGEEVAMR	10	27436946	1xTMT6plex [N-Term]	0.81	1.414	1.237	4.020965576	45.1	0	1720.9101	2	860.9584351	-0.294508593	-0.000253411	47.8012571
																			SVGGSGGGSFGDNLVTR	6	27436946	1xTMT6plex [N-Term]	0.963	0.791		5.705610275	84.65	0	1795.913606	2	898.4598999	-0.602782511	-0.000541273	52.78746216
																			TVLCGTCGQPADK	4	27436946	2xTMT6plex [N-Term; K13]; 2xCarbamidomethyl [C4; C7	0.581	0.573		3.851686954	58.85	0	1864.966125	2	932.986084	-0.661546198	-0.000616881	44.83962391
																			AQHEDQVEQYK	14	27436946	2xTMT6plex [N-Term; K11]	0.778	0.755	0.706	5.622924805	54.41	0	1832.954297	2	916.9805298	-0.280694226	-0.00025725	34.8693129
																			VAVEEVDEEGK	10	27436946	2xTMT6plex [N-Term; K11]	0.77	0.804		4.93041563	70.02	0	1661.899802	2	831.4525757	-1.159848993	-0.000963776	46.56701304
																			ASASGSGAQVGGPISSGSSASSVTVTR	26	27436946	1xTMT6plex [N-Term]	0.772	0.729	0.999	9.399476051	110.66	0	2594.321916	3	865.4447021	-0.910941188	-0.000787758	41.66389073
																			AAYEAELGDAR	22	27436946	1xTMT6plex [N-Term]	0.627	0.681	0.708	4.936688423	74.17	0	1394.711324	2	697.8590698	-0.330743398	-0.000230646	38.95790587
																			ASSHSSQTQGGGSVTK	142	27436946	2xTMT6plex [N-Term; K16]	0.845	0.828	0.806	5.802528381	80.05	0	1977.040152	3	659.6849976	0.145275644	9.57E-05	28.66776877
																			LALDMEIHAYR	26	27436946	1xTMT6plex [N-Term]	0.679	0.988	0.371	4.030868053	63.49	0	1560.840564	3	520.9517822	0.146760465	7.64E-05	90.92299012
																			EGDLIAAQAR	6	27436946	1xTMT6plex [N-Term]	0.973	1.048	1.184	3.909815788	64.41	0	1272.71093	2	636.8584595	-1.012118822	-0.000644067	45.70550085
																			ITESEEVVSR	4	27436946	1xTMT6plex [N-Term]	0.831	0.932		3.479677439	53.75	0	1377.74229	2	689.3745728	-0.305704623	-0.000210591	41.87675256
																			TLEGELHDLR	16	27436946	1xTMT6plex [N-Term]	0.789	0.703	0.693	4.239264011	65.89	0	1411.774259	2	706.3909912	0.316532103	0.000223436	45.62075709
																			AQHEDQVEQYKK	4	27436946	3xTMT6plex [N-Term; K11; K12]	0.373		0.462	4.501769543	17.47	1	2190.212192	4	548.3087158	0.383977065	0.000210248	35.25731436
																			LADALQELR	13	27436946	1xTMT6plex [N-Term]	0.791	0.879	0.859	3.563678503	52.78	0	1257.736417	2	629.371582	-0.421000372	-0.000264754	62.90467401
																			SLETENAGLR	6	27436946	1xTMT6plex [N-Term]	0.722	0.876	0.871	4.305250168	51.86	0	1318.71641	2	659.8581543	-5.594642029	-0.003688873	36.56471692
																			IDSLSAQLSQLQK	5	27436946	2xTMT6plex [N-Term; K13]			1.725	2.83401227	31.95	0	1889.110798	2	945.0578003	-1.309655349	-0.001237042	73.58335544
																			SNEDQSMGNWQIK	2	27436946	2xTMT6plex [N-Term; K13]; 1xOxidation [M7	0.682			2.781531811	30.87	0	2010.995511	2	997.9941406	-9.820057218	-0.00979551	51.57611777
																			LKDLEALLNSK	4	27436946	3xTMT6plex [N-Term; K2; K11]	0.721			4.434978485	18.67	1	1931.214424	3	644.4091187	-0.839716682	-0.000540558	88.97032351
																			LEAALGEAK	11	27436946	2xTMT6plex [N-Term; K9]	0.798	0.818	100	4.151691437	51.81	0	1359.824787	2	680.4152222	-1.190862292	-0.000809682	54.89204617
																			KEGDLIAAQAR	6	27436946	2xTMT6plex [N-Term; K1]	0.794	0.987	0.839	3.364347219	24.22	1	1629.968825	3	543.994812	0.648702687	0.000352455	43.95616425
																			LALDMEIHAYR	4	27436946	1xTMT6plex [N-Term]; 1xOxidation [M5]	0.669		1.926	3.412200451	39.63	0	1576.835479	3	520.9517822	0.146760465	7.64E-05	90.92299012
																			VAVEEVDEEGKFVR	3	27436946	2xTMT6plex [N-Term; K11]	0.234	0.729		3.011364937	6.33	1	2064.137741	3	688.7176514	0.319674191	0.000219951	59.41330684
																			DLEALLNSK	4	27436946	2xTMT6plex [N-Term; K9]	1.611	100		2.399184465	24.68	0	1460.872465	2	730.9393921	-0.655724805	-0.000478965	74.16171792
																			LQEKEDLQELNDR	5	27436946	2xTMT6plex [N-Term; K4]	0.796	0.912		3.529002666	13.28	1	2088.133718	3	696.7158813	-0.300463052	-0.000209136	56.70557386
																			LLEGEEER	8	27436946	1xTMT6plex [N-Term]	0.891	0.901	0.91	3.30015111	57.52	0	1203.641848	2	602.324585	0.03776196	2.27E-05	47.24056336
																			LQTMKEELDFQK	8	27436946	3xTMT6plex [N-Term; K5; K12]	0.995	0.466	0.792	2.616175413	12.25	1	2197.250552	3	733.0877075	-0.902250954	-0.000660824	63.75229531
																			KQLQDEMLR	13	27436946	2xTMT6plex [N-Term; K1]	0.643	0.616	0.638	3.526650667	28.59	1	1618.935082	2	809.9707642	-0.513170476	-0.000415395	43.47374931
																			KTLDSVAK	4	27436946	3xTMT6plex [N-Term; K1; K8]			0.61	2.813788414	34.02	1	1548.992804	2	775.0001221	0.105255896	8.15E-05	41.71366296
																			LQLELSK	12	27436946	2xTMT6plex [N-Term; K7]	0.811	0.768	0.93	3.28993082	40.42	0	1288.824059	2	644.9160156	0.539856465	0.00034789	64.43397998
																			KLLEGEEER	2	27436946	2xTMT6plex [N-Term; K1]		0.786		2.727909088	12.36	1	1560.899743	3	520.9711914	-0.462452027	-0.000240614	36.54987775
																			LAVYIDR	13	27436946	1xTMT6plex [N-Term]	0.6	0.643	0.742	3.004096746	33.6	0	1078.645811	2	539.8256836	-1.595141317	-0.000860296	49.1983752
																			AGQVVTIWAAGAGATHSPPTDLVWK	2	27436946	2xTMT6plex [N-Term; K25]				1.77304697		0	2991.645647	3	997.8860474	-0.687885665	-0.00068597	78.83866997
																			EELDFQK	8	27436946	2xTMT6plex [N-Term; K7]	0.959	1.128		3.204521894	30.03	0	1366.761852	2	683.8839722	-0.866832843	-0.000592377	58.23378286
																			QLQDEMLR	2	27436946	1xTMT6plex [N-Term]			100	2.273235559	25.84	0	1261.677188	2	631.3423462	0.180896361	0.000114116	50.57383575
																			TLDSVAK	6	27436946	2xTMT6plex [N-Term; K7]	0.652	0.863	0.981	2.952090263	41.2	0	1191.73491	2	596.3705444	-0.920735647	-0.000548636	44.53974523
																			EDLQELNDR	2	27436946	1xTMT6plex [N-Term]	1.271			2.780449152	27.57	0	1360.690589	2	680.8494263	0.725410369	0.00049353	46.94406012

Bhat et al,2017. Cigarette smoke and chewing tobacco alter expression of different sets of miRNAs in oral keratinocytes

Supplementary Table S2(A). List of all proteins and peptides identified in OKF6/TERT1 cells chronically treated with chewing tobacco using Sequest and Mascot search algorithms

Accession	Gene_Symbol	Description	Gene_ID	Refseq_Accession	Coverage	Proteins	#Unique_Peptides	#Peptides	#PSMS	#AAs	MW [kDa]	calc. pI	OKF6-STE-6M/OKF6P_R1	OKF6-STE-6M/OKF6P_R2	OKF6-STE-6M/OKF6P_R3	Median fold change	Status	P-value (6M)	Sequence	#PSMs	Protein Accessions	Modifications	OKF6-STE-6M/OKF6P_R1	OKF6-STE-6M/OKF6P_R2	OKF6-STE-6M/OKF6P_R3	Xcorr	Ionscore	# Missed Cleavages	Theo. MH+ [Da]	Charge	m_z	DeltaM [ppm]	Deltam/z [Da]	RT [min]
365192532	MYH10	myosin-10 isoform 1	4628	NP_001242941.1	24.0159442	1	27	46	245	2007	232.385	5.55	0.82	1.03	0.84	0.9	Significant	0.0055																
																			LQQELDDLTVDLDHQR	3	365192532	1xTMT6plex [N-Term]	1.209		0.01	3.920865536	62.07	0	2167.119241	3	723.0454712	1.208574115	0.000873041	86.51573916
																			DLEAQIEAANK	6	365192532	2xTMT6plex [N-Term; K11]	1.362	1.099	1.185	3.650596142	52.86	0	1659.931771	2	830.4692383	-0.344121072	-0.000285609	52.93704733
																			ELDDATEANEGLSR	4	365192532	1xTMT6plex [N-Term]	0.973	1.093		4.474626064	67.38	0	1748.850002	2	874.9282227	-0.47670612	-0.000416844	39.00427889
																			EQADFAVEALAK	5	365192532	2xTMT6plex [N-Term; K12]	100	100		3.016440868	53.01	0	1749.978721	2	875.4926147	-0.439129804	-0.000384234	74.76483741
																			ELEAELEDER	6	365192532	1xTMT6plex [N-Term]		1.263	1.566	3.851930618	71.52	0	1461.727034	2	731.3668213	-0.45693338	-0.000333956	45.08435822
																			TQLEELEDELQATEDAK	12	365192532	2xTMT6plex [N-Term; K17]	1.159	1.124	1.418	3.995464325	43.98	0	2420.24445	3	807.4188843	-0.971231633	-0.000783539	87.48657366
																			EDQSILCTGESGAGK	10	365192532	2xTMT6plex [N-Term; K15]; 1xCarbamidomethyl [C7	1.753	1.309	1.499	4.520535469	77.09	0	2010.021391	2	1005.514282	-0.051281482	-5.15E-05	42.92864444
																			KLDGETTDLQDQIAELQAQIDELK	7	365192532	3xTMT6plex [N-Term; K1; K24]		100		2.706674814	23.36	1	3401.861855	4	851.2197876	-1.333061002	-0.001133722	97.44705
																			IAQLEEELEEEQSNMELLNDR	5	365192532	1xTMT6plex [N-Term]	1.064			2.870167255	28.3	0	2761.339934	3	921.1185913	0.465873747	0.000428812	94.11546266
																			LQQLFNHTMFILEQEEYQR	4	365192532	1xTMT6plex [N-Term]			1.802	4.64874506	37.09	0	2696.370387	3	899.4614868	-0.177998833	-0.000159984	103.4314357
																			ALEQQVEEMR	4	365192532	1xTMT6plex [N-Term]	0.93	0.887		4.200110435	51.18	0	1461.756894	2	731.3795776	-3.43123852	-0.002507818	49.83083116
																			AGVLAHLEEER	12	365192532	1xTMT6plex [N-Term]	1.676	1.489	1.709	4.231396198	57.68	0	1452.800808	3	484.9386597	0.425202067	0.000205911	53.69342542
																			QLEEAEEEATR	2	365192532	1xTMT6plex [N-Term]	1.472			4.044686317	38.86	0	1533.759397	2	767.383606	0.351231141	0.000269352	45.36743562
																			LDPHLVLDQLR	10	365192532	1xTMT6plex [N-Term]	0.934	1.269		3.6594944	46.08	0	1547.910693	2	774.4589233	-0.079300642	-6.14E-05	84.30943887
																			DKADFCIIHYAGK	6	365192532	3xTMT6plex [N-Term; K2; K13]; 1xCarbamidomethyl [C6	1.159	1.103	0.985	4.06019783	19.44	1	2225.235571	4	557.0643921	0.075367021	4.19E-05	51.25403601
																			KEEELQGALAR	4	365192532	2xTMT6plex [N-Term; K1]			1.112	3.324520111	31.48	1	1701.989955	3	568.0006104	-1.57282674	-0.000892312	45.88597725
																			EIFMQVEDER	2	365192532	1xTMT6plex [N-Term]	1.23			3.303752184	52.78	0	1524.75656	2	762.8810425	-1.148759814	-0.00087579	69.70673413
																			NSLQEQQEEEEEAR	2	365192532	1xTMT6plex [N-Term]	1.153			3.295411825	28.51	0	1947.909308	2	974.456665	-1.670812836	-0.001627296	33.98579175
																			ALEEALEAK	4	365192532	2xTMT6plex [N-Term; K9]		1.277		3.301868916	50.5	0	1431.845916	2	716.4268799	0.395814674	0.000283373	71.56601783
																			ADFCIIHYAGK	16	365192532	2xTMT6plex [N-Term; K11]; 1xCarbamidomethyl [C4	1.263	1.18	1.194	3.130453587	32.98	0	1752.950733	2	876.9789429	-0.0705883	-6.19E-05	54.99212348
																			VEDMAELTCLNEASVLHNLK	10	365192532	2xTMT6plex [N-Term; K20]; 1xCarbamidomethyl [C9	1.037	0.01	100	2.921220064	25.48	0	2744.436308	3	915.4824829	-1.243267888	-0.001137357	83.16239164
																			KFDQLLAEEK	12	365192532	3xTMT6plex [N-Term; K1; K10]	1.479	1.049	1.208	3.74681735	21.57	1	1908.140925	3	636.7192383	1.172074725	0.000745495	67.86442023
																			FDQLLAEEK	4	365192532	2xTMT6plex [N-Term; K9]	1.112	2.208		3.206862211	53.23	0	1550.88303	2	775.9446411	-0.660619413	-0.000512272	76.14735231
																			VEDMAELTCLNEASVLHNLK	2	365192532	2xTMT6plex [N-Term; K20]; 1xCarbamidomethyl [C9]; 1xOxidation [M4]	100			2.271770716	14.6	0	2760.431223	3	915.4824829	-1.243267888	-0.001137357	83.16239164
																			ALELDPNLYR	6	365192532	1xTMT6plex [N-Term]	1.147	0.01	100	2.99339056	26.09	0	1432.799745	2	716.902832	-0.947778993	-0.000678989	74.84478204
																			ADMEDLMSSK	6	365192532	2xTMT6plex [N-Term; K10]	0.992		0.291	2.56010437	37.68	0	1584.801351	2	792.9050293	0.903131329	0.000715642	62.94348621
																			DLSEELEALK	2	365192532	2xTMT6plex [N-Term; K10]				2.108099937	41.45	0	1604.914724	2	802.9597778	-1.523498975	-0.001222543	85.03111992
																			AVIYNPATQADWTAK	6	365192532	2xTMT6plex [N-Term; K15]		100	1.161	1.462546468	13.85	0	2107.158811	2	1054.081543	-1.424435825	-0.001500756	60.09600425
																			KQQQLSALK	2	365192532	3xTMT6plex [N-Term; K1; K9]	0.879			3.141822815	20.99	1	1731.109565	3	577.7080078	-0.055058618	-3.18E-05	44.51660249
																			SDLLLEGFNNYR	2	365192532	1xTMT6plex [N-Term]	1.782			2.432257891	21.65	0	1669.874701	2	835.4414673	0.572977309	0.0004784	77.83492175
																			EQEVAELK	4	365192532	2xTMT6plex [N-Term; K8]	1.025	1.059		3.592775345	47.46	0	1403.814616	2	702.4118652	1.308982489	0.000918784	45.34066689
																			NMDPLNDNVATLLHQSSDR	2	365192532	1xTMT6plex [N-Term]				1.317866802	5.66	0	2369.171687	3	790.3952637	-0.189785475	-0.000149878	58.33041754
																			EQEVAELKK	3	365192532	3xTMT6plex [N-Term; K8; K9]	0.988	1.036		2.399578333	7.72	1	1761.072511	3	587.6954956	-0.328023741	-0.000192558	44.74185205
																			GDDETLHK	2	365192532	2xTMT6plex [N-Term; K8]			0.636	2.147005558	22.36	0	1372.747265	3	458.25354	-0.872806556	-0.000399381	32.9803117
																			VIQYLAHVASSHK	2	365192532	2xTMT6plex [N-Term; K13]	0.902			1.551387072	7.27	0	1911.121638	4	478.5353088	-1.167962517	-0.00055803	69.67033938
																			ELEAELEDERK	5	365192532	2xTMT6plex [N-Term; K11]	0.402	0.839	0.86	3.814759493	25.41	1	1818.984929	3	606.9940186	-9.580297044	-0.005808805	46.39617185
																			ALAYDKLEK	2	365192532	3xTMT6plex [N-Term; K6; K9]	0.72	0.538		2.248070002		1	1738.071783	3	580.0291138	0.57825494	0.000335016	54.9941135
																			VEEEEER	6	365192532	1xTMT6plex [N-Term]		100	1.383	3.147355795	38.91	0	1148.563263	2	574.7855835	0.546066732	0.000313596	25.81106331
																			IVFQEFR	7	365192532	1xTMT6plex [N-Term]	1.014	1.634		2.653630972	33.23	0	1167.67236	2	584.3395996	-0.374806551	-0.000218826	74.00495659
																			LQELEGAVK	5	365192532	2xTMT6plex [N-Term; K9]			100	2.279907227	16.64	0	1444.877551	2	722.9417725	-0.887679461	-0.000641294	56.46702797
																			ELACEVK	3	365192532	2xTMT6plex [N-Term; K7]; 1xCarbamidomethyl [C4	1.122	1.079		3.174056768	37.7	0	1306.744094	2	653.8757324	0.071891467	4.70E-05	45.79056675
																			TVGQLYK	4	365192532	2xTMT6plex [N-Term; K7]	1.228	1.028		2.741590977	31	0	1266.782194	2	633.8947754	0.063224168	4.00E-05	53.36307664
																			ADEWLMK	8	365192532	2xTMT6plex [N-Term; K7]	0.874		100	3.158114672	36.3	0	1350.749179	2	675.8774414	-1.164670336	-0.000786589	65.2501774
																			DEIFAQSK	4	365192532	2xTMT6plex [N-Term; K8]		1.642	1.16	3.271160126	23.85	0	1395.788402	2	698.4019775	5.929908943	0.004138449	44.39357387
																			FVAELWK	5	365192532	2xTMT6plex [N-Term; K7]	1.128	100	100	2.408267021	17.53	0	1350.81858	2	675.9127808	-0.218154064	-0.000147343	73.16816474
																			ADEWLMK	4	365192532	2xTMT6plex [N-Term; K7]; 1xOxidation [M6]	0.948	1.153	100	1.522166729	31.69	0	1366.744094	2	675.8774414	-1.164670336	-0.000786589	65.2501774
																			NCAAYLK	2	365192532	2xTMT6plex [N-Term; K7]; 1xCarbamidomethyl [C2	1.621			3.065582752	35.36	0	1297.733864	2	649.3717041	1.747348349	0.001133797	39.79516013
																			LKEIFMQVEDER	1	365192532	2xTMT6plex [N-Term; K2]		1.913		2.320290089	10.78	1	1995.098519	3	665.7041626	-0.292851218	-0.000194756	87.27995298
33286418	PKM	pyruvate kinase PKM isoform a	5315	NP_002645.3	65.53672316	1	29	30	826	531	57.9	7.84	1.1	1.11	1.12	1.11	Significant	0.000027																
																			GDLGIEIPAEK	37	33286418	2xTMT6plex [N-Term; K11]	0.927	1.064	1.101	3.874292612	54.22	0	1599.935794	2	800.4719238	0.485692147	0.000388538	64.32086212
																			KGVNLPGAAVDLPAVSEK	10	33286418	3xTMT6plex [N-Term; K1; K18]	2.11	2.624	1.084	4.025949091	36.36	1	2452.474221	3	818.1623566	-0.474629543	-0.000388006	57.93346125
																			RFDEILEASDGIMVAR	61	33286418	1xTMT6plex [N-Term]	1.285	1.664	1.103	6.160237312	49.58	1	2051.079291	3	684.364502	-0.164874477	-0.000112724	81.50352958
																			DPVQEAWAEDVDLR	36	33286418	1xTMT6plex [N-Term]	1.567	1.608	1.231	4.890119076	68.37	0	1871.933672	2	936.4694824	-1.059987477	-0.000992113	83.22440992
																			CCSGAIIVLTK	4	33286418	2xTMT6plex [N-Term; K11]; 2xCarbamidomethyl [C1; C2	1.61	1.697		3.759759903	53.71	0	1679.958855	2	840.4823608	-0.839282646	-0.00070498	68.98158289
																			TATESFASDPILYRPVAVALDTK	6	33286418	2xTMT6plex [N-Term; K23]	1.136	1.181		4.045522213	35.84	0	2923.618095	3	975.2098999	-1.008371433	-0.000982698	89.99091059
																			GIFPVLCKDPVQEAWAEDVDLR	14	33286418	2xTMT6plex [N-Term; K8]; 1xCarbamidomethyl [C7	0.683	0.646	0.553	4.815615177	39.49	1	3015.601399	3	1005.87146	-0.521397552	-0.000524109	97.82489827
																			EAEAAIYHLQLFEELR	133	33286418	1xTMT6plex [N-Term]	1.115	1.437	2.265	4.401194096	48.77	0	2161.149085	3	721.0536499	-1.243969233	-0.000896134	85.10956402
																			AGKPVICATQMLESMIK	13	33286418	3xTMT6plex [N-Term; K3; K17]; 1xCarbamidomethyl [C7	2.128	100	2.582	5.94026041	46.54	0	2564.458119	3	855.4884644	-2.838363216	-0.002426288	93.24391706
																			AEGSDVANAVLDGADCIMLSGETAK	22	33286418	2xTMT6plex [N-Term; K25]; 1xCarbamidomethyl [C16]; 1xOxidation [M18]	1.045	100	0.92	5.251690865	68.78	0	2968.464373	3	990.1582031	-1.454241809	-0.001438955	82.36811585
																			AEGSDVANAVLDGADCIMLSGETAK	53	33286418	2xTMT6plex [N-Term; K25]; 1xCarbamidomethyl [C16	1.717	1.105	1.498	5.171498775	58.23	0	2952.469458	3	990.1582031	-1.454241809	-0.001438955	82.36811585
																			LNFSHGTHEYHAETIK	30	33286418	2xTMT6plex [N-Term; K16]	1.158	1.338	1.111	6.992025375	68.04	0	2342.22935	4	586.3129272	0.225940459	0.000132301	38.94495747
																			GSGTAEVELK	75	33286418	2xTMT6plex [N-Term; K10]	1.086	1.207	1.261	4.303888798	65.06	0	1448.83608	2	724.9220581	0.524297364	0.00037981	39.56917958
																			EAEAAIYHLQLFEELRR	6	33286418	1xTMT6plex [N-Term]			0.382	5.795260906	27.79	1	2317.250196	4	580.0683594	0.609256611	0.00035295	83.44702445
																			LAPITSDPTEATAVGAVEASFK	37	33286418	2xTMT6plex [N-Term; K22]	1.46	1.383	1.168	5.675824642	69.72	0	2633.443819	3	878.4852905	-0.94946319	-0.000833453	93.83767215
																			IYVDDGLISLQVK	55	33286418	2xTMT6plex [N-Term; K13]	1.379	1.589	1.298	3.939882517	59.46	0	1921.141036	2	961.0738525	-0.316016296	-0.000303556	81.55620435
																			NTGIICTIGPASR	6	33286418	1xTMT6plex [N-Term]; 1xCarbamidomethyl [C6	1.034	1.335		4.633892536	44.8	0	1588.867842	2	794.9376221	0.079006335	6.28E-05	56.76330823
																			GVNLPGAAVDLPAVSEK	13	33286418	2xTMT6plex [N-Term; K17]	1.061	0.803		3.336400032	64.03	0	2095.216326	2	1048.109619	-2.082843999	-0.002182004	70.92428011
																			ITLDNAYMEK	18	33286418	2xTMT6plex [N-Term; K10]	1.178	1.257	1.403	4.470714092	54.39	0	1655.907865	2	828.458313	0.89660578	0.000742348	64.26568992
																			LDIDSPPITAR	20	33286418	1xTMT6plex [N-Term]	1.021	0.978	1.385	4.173104763	43.4	0	1426.81031	2	713.9102173	1.995969816	0.001423935	57.2144631
																			GSGTAEVELKK	28	33286418	3xTMT6plex [N-Term; K10; K11]	0.785	0.753	0.883	3.549044132	69.06	1	1806.093975	2	903.5509644	0.374931176	0.00033858	42.28521841
																			VNFAMNVGK	12	33286418	2xTMT6plex [N-Term; K9]	1.188	1.536	1.259	4.871505737	51.99	0	1437.828827	2	719.4180298	-0.030385875	-2.18E-05	59.79163378
																			RFDEILEASDGIMVAR	14	33286418	1xTMT6plex [N-Term]; 1xOxidation [M13]	1.179		100	4.448275089	41.27	1	2067.074206	3	684.364502	-0.164874477	-0.000112724	81.50352958
																			ITLDNAYMEK	12	33286418	2xTMT6plex [N-Term; K10]; 1xOxidation [M8	1.168	1.155	1.356	2.907870293	44.65	0	1671.90278	2	828.458313	0.89660578	0.000742348	64.26568992
																			GDYPLEAVR	12	33286418	1xTMT6plex [N-Term]	1.069		1.046	3.472897291	47.64	0	1248.678568	2	624.8391724	-6.006056027	-0.003749817	43.90196791
																			KASDVHEVR	8	33286418	2xTMT6plex [N-Term; K1]	1.005	0.855		3.281455278	27.21	1	1498.874197	3	500.2960815	-0.337220492	-0.000168484	19.70814981
																			GIFPVLCK	50	33286418	2xTMT6plex [N-Term; K8]; 1xCarbamidomethyl [C7	1.237	1.09	1.203	3.305967808	39.91	0	1391.8485	2	696.4273071	-0.83498469	-0.000581086	75.05021177
																			GDVVIVLTGWRPGSGFTNTMR	8	33286418	1xTMT6plex [N-Term]	1.329	100		1.960686684	5.04	0	2492.328129	3	831.4475708	0.012294667	1.02E-05	86.76134447
																			VNFAMNVGK	8	33286418	2xTMT6plex [N-Term; K9]; 1xOxidation [M5]	1.253	1.265	1.167	2.824705124	32.8	0	1453.823742	2	719.4180298	-0.030385875	-2.18E-05	59.79163378
																			CDENILWLDYK	6	33286418	2xTMT6plex [N-Term; K11]; 1xCarbamidomethyl [C1		100	1.369	2.431798935	14.83	0	1927.003556	2	964.005127	-0.300400976	-0.000289437	75.66399044
																			GADFLVTEVENGGSLGSK	2	33286418	2xTMT6plex [N-Term; K18]		100		1.123611689	9.29	0	2238.201798	2	1119.601685	-2.549001337	-0.00285259	74.59322677
																			APIIAVTR	10	33286418	1xTMT6plex [N-Term]	0.826	0.849	0.837	2.709722519	43.26	0	1069.693096	2	535.3498535	-0.621952925	-0.000332649	45.65280144
																			ASDVHEVR	3	33286418	1xTMT6plex [N-Term]		100		2.200623274	21.95	0	1141.616302	2	571.3120117	0.389752231	0.000222474	17.97566283
																			VVEVGSK	4	33286418	2xTMT6plex [N-Term; K7]		0.761		2.705645084	27	0	1175.739995	2	588.3731079	-0.897919346	-0.00052786	51.86133037
71773329	ANXA6	annexin A6 isoform 1	309	NP_001146.2	65.37890045	1	38	38	275	673	75.826	5.6	1.27	1.43	1.53	1.41	Significant	0.0029																
																			KAMEGAGTDEK	2	71773329	3xTMT6plex [N-Term; K1; K11]	1.13			3.568588495	36.31	1	1824.01401	3	608.6776123	2.34297217	0.001424538	34.1744159
																			LILGLMMPPAHYDAK	6	71773329	2xTMT6plex [N-Term; K15]	1.513			3.688644886	39.97	0	2128.206295	3	710.0766602	4.290695012	0.00304402	87.17514962
																			GTVRPANDFNPDADAK	10	71773329	2xTMT6plex [N-Term; K16]	1.447	1.626	1.529	4.433211327	25.26	0	2146.129302	3	716.0474854	-0.651747922	-0.000466245	38.64666515
																			DAISGIGTDEK	2	71773329	2xTMT6plex [N-Term; K11]	1.751			4.448802948	58.62	0	1563.863023	2	782.4355469	0.507851384	0.000397105	51.44485448
																			SLHQAIEGDTSGDFLK	12	71773329	2xTMT6plex [N-Term; K16]	1.129	0.862	1.108	5.43034935	57.57	0	2176.165018	3	726.0597534	-0.143057209	-0.000103772	66.69638029
																			TNEQMHQLVAAYK	10	71773329	2xTMT6plex [N-Term; K13]	1.387	1.673	1.428	4.588112354	30.54	0	1991.078452	3	664.3579102	-9.680618677	-0.006424957	48.96767823
																			GSIHDFPGFDPNQDAEALYTAMK	2	71773329	2xTMT6plex [N-Term; K23]		100		3.050937414	21.57	0	2982.470778	3	994.8286133	0.170393539	0.000169398	84.74085397
																			GLGTDEDTIIDIITHR	10	71773329	1xTMT6plex [N-Term]	2.231	1	1.536	4.575245857	49.48	0	1998.0705	3	666.6938477	-1.756930624	-0.001170157	99.24758286
																			SLEDALSSDTSGHFR	12	71773329	1xTMT6plex [N-Term]	0.965	1.345	1.176	4.604727745	74.39	0	1850.908186	3	617.6400757	-1.357257609	-0.000837386	54.2741066
																			EDAQVAAEILEIADTPSGDK	6	71773329	2xTMT6plex [N-Term; K20]	100	2.427		4.902284145	53.9	0	2530.328848	3	844.1142578	-0.248312586	-0.000209437	97.56302665
																			EEDDVVSEDLVQQDVQDLYEAGELK	10	71773329	2xTMT6plex [N-Term; K25]	100	100	100	2.997541428	16.86	0	3323.641862	3	1108.550415	-1.555535472	-0.001723348	102.3812041
																			MLVVLLQGTREEDDVVSEDLVQQDVQDLYEAGELK	4	71773329	2xTMT6plex [N-Term; K35]; 1xOxidation [M1	0.871	0.662		4.032173634	13.14	1	4450.295112	4	1109.328979	-1.377538953	-0.001527105	109.4920024
																			MLVVLLQGTREEDDVVSEDLVQQDVQDLYEAGELK	9	71773329	2xTMT6plex [N-Term; K35]	2.105	1.233	1.098	4.838788033	30.27	1	4434.300197	4	1109.328979	-1.377538953	-0.001527105	109.4920024
																			EEGGENLDQAR	2	71773329	1xTMT6plex [N-Term]	2.141			3.908207178	65.01	0	1446.702216	2	723.8547363	-0.013882435	-1.00E-05	30.6068161
																			DLEADIIGDTSGHFQK	7	71773329	2xTMT6plex [N-Term; K16]	1.844	1.518	1.697	4.535861969	50.61	0	2204.159933	3	735.3913574	-0.187783277	-0.000137968	75.34995448
																			LSGGDDDAAGQFFPEAAQVAYQMWELSAVAR	4	71773329	1xTMT6plex [N-Term]				3.578741074	33.6	0	3529.689549	3	1177.234863	0.138265347	0.000162678	112.3331292
																			GFGSDKEAILDIITSR	6	71773329	2xTMT6plex [N-Term; K6]	1.88	0.95	1.011	3.304495811	21.82	1	2180.232704	3	727.416626	1.202036683	0.000873573	98.98877156
																			EIKDAISGIGTDEK	6	71773329	3xTMT6plex [N-Term; K3; K14]		0.537	100	3.953277826	16.77	1	2163.247575	3	721.753479	-0.781722028	-0.000563686	64.92224193
																			MTNYDVEHTIK	10	71773329	2xTMT6plex [N-Term; K11]	1.873	1.782	1.724	3.508602381	41.14	0	1808.961691	2	904.9840698	-0.457744112	-0.000414021	43.42086128
																			DAFVAIVQSVK	6	71773329	2xTMT6plex [N-Term; K11]	4.431	1.304	1.768	3.394672155	42.68	0	1634.988164	2	817.996521	-1.467006224	-0.001199269	78.00528017
																			AMEGAGTDEK	4	71773329	2xTMT6plex [N-Term; K10]	1.067			3.726732731	30.59	0	1466.756115	2	733.8812866	-0.558012204	-0.000409234	34.5335891
																			GELSGDFEK	12	71773329	2xTMT6plex [N-Term; K9]	1.263	1.634	1.501	3.570112944	51.44	0	1439.778231	2	720.3857422	-9.739697893	-0.007011503	46.68779716
																			SEIDLLNIR	8	71773329	1xTMT6plex [N-Term]	2.091	1.513	0.547	3.549966335	53.26	0	1301.762632	2	651.3839722	-1.508688308	-0.000981977	67.30302078
																			MTNYDVEHTIK	6	71773329	2xTMT6plex [N-Term; K11]; 1xOxidation [M1	1.671	1.742	1.899	2.851438761	22.28	0	1824.956606	2	904.9840698	-0.457744112	-0.000414021	43.42086128
																			SELDMLDIR	6	71773329	1xTMT6plex [N-Term]	2.256	2.419	1.816	3.299249411	67.9	0	1320.703068	2	660.8551636	-0.013138125	-8.68E-06	66.92213814
																			AINEAYKEDYHK	12	71773329	3xTMT6plex [N-Term; K7; K12]	1.138	1.2	1.224	4.623951435	18.89	1	2168.19548	4	542.8044434	0.213914061	0.000115952	39.36213702
																			LIVGLMRPPAYCDAK	2	71773329	2xTMT6plex [N-Term; K15]; 1xCarbamidomethyl [C12	1	1.021		2.059868097		0	2162.223008	3	721.4118652	-0.909099971	-0.000655226	79.72593242
																			CLIEILASR	4	71773329	1xTMT6plex [N-Term]; 1xCarbamidomethyl [C1	0.987	100		2.723452091	54.25	0	1303.760524	2	652.3839722	0.110584678	7.21E-05	88.10315956
																			LVFDEYLK	9	71773329	2xTMT6plex [N-Term; K8]	1.47	2.089	1.822	3.859214544	45.62	0	1484.876488	2	742.9415894	-0.394779678	-0.0002931	73.40416605
																			SEISGDLAR	2	71773329	1xTMT6plex [N-Term]			1.835	3.224111557	42.27	0	1176.642182	2	588.824707	-0.038055324	-2.24E-05	38.57158181
																			SELDMLDIR	7	71773329	1xTMT6plex [N-Term]; 1xOxidation [M5]		0.727	1.373	2.165833235	44.61	0	1336.697983	2	660.8551636	-0.013138125	-8.68E-06	66.92213814
																			EAILDIITSR	6	71773329	1xTMT6plex [N-Term]	100	100	100	1.818113208	28.1	0	1359.804496	2	680.4051514	-1.081284573	-0.000735168	75.26799472
																			QEVCQSYK	2	71773329	2xTMT6plex [N-Term; K8]; 1xCarbamidomethyl [C4			1.723	3.72238946	36.58	0	1499.792835	2	750.399353	-0.937366335	-0.000702928	36.1413291
																			TTGKPIEASIR	1	71773329	2xTMT6plex [N-Term; K4]	1.304			2.337275743		0	1630.989227	3	544.335144	1.013108413	0.00055079	37.13758923
																			DLIADLK	4	71773329	2xTMT6plex [N-Term; K7]		1.353	1.314	3.007342577	37.52	0	1245.78186	2	623.3933716	-1.921007212	-0.001196578	61.23651024
																			ALIEILATR	8	71773329	1xTMT6plex [N-Term]	100	100	100	2.24669075	27.56	0	1228.782639	2	614.8952637	0.497910477	0.000305912	71.22012846
																			VFQEFIK	8	71773329	2xTMT6plex [N-Term; K7]	1.457	100		3.086522818	35.28	0	1368.829144	2	684.9190063	1.162910155	0.000795913	75.87894717
																			LMLAVVK	8	71773329	2xTMT6plex [N-Term; K7]	1.086	1.479	0.811	3.083244801	41.37	0	1231.821222	2	616.414856	0.984813413	0.000606557	80.63480358
																			DLMTDLK	10	71773329	2xTMT6plex [N-Term; K7]	1.244	1.328	1.436	2.838707209	38.51	0	1293.748845	2	647.3760986	-3.033320099	-0.001962177	60.56057019
																			GAGTDEK	4	71773329	2xTMT6plex [N-Term; K7]	1.509	1.677		2.683608055	30.71	0	1135.635924	2	568.3214111	-0.332953869	-0.000189057	19.53714151
																			EMSGDVR	10	71773329	1xTMT6plex [N-Term]	1.794	1.643	2.168	2.769628048	40.59	0	1022.513811	2	511.7605896	0.089856213	4.59E-05	24.56733719
																			AMEGAGTDEK	6	71773329	2xTMT6plex [N-Term; K10]; 1xOxidation [M2	1.144	1.3		1.936986089	23.67	0	1482.75103	2	733.8812866	-0.558012204	-0.000409234	34.5335891
5729877	HSPA8	heat shock cognate 71 kDa protein isoform 1	3312	NP_006588.1	73.83900929	1	31	38	497	646	70.854	5.52	1.12	1.15	1.12	1.13	Significant	0.000078																
																			SINPDEAVAYGAAVQAAILSGDK	17	5729877	2xTMT6plex [N-Term; K23]	1.142	1.531	2.498	4.021234512	39.16	0	2718.47143	3	906.8199463	-9.61744991	-0.008714921	97.40003198
																			TLSSSTQASIEIDSLYEGIDFYTSITR	75	5729877	1xTMT6plex [N-Term]		3.405	0.615	6.246430397	62.37	0	3226.620449	3	1076.210571	-1.019200388	-0.001096191	112.064118
																			GPAVGIDLGTTYSCVGVFQHGK	42	5729877	2xTMT6plex [N-Term; K22]; 1xCarbamidomethyl [C14	1.281	1.516	1.198	4.755177975	48.02	0	2721.443442	3	907.819397	0.071935343	6.53E-05	76.36659792
																			IINEPTAAAIAYGLDK	31	5729877	2xTMT6plex [N-Term; K16]	1.572	1.737	2.328	4.13507843	46.05	0	2118.221077	2	1059.61499	0.768167576	0.000813574	75.35766658
																			SQIHDIVLVGGSTR	27	5729877	1xTMT6plex [N-Term]	0.854	0.88	0.507	4.44071579	57.55	0	1710.969998	2	855.987854	-0.91590863	-0.000783546	53.6498382
																			STAGDTHLGGEDFDNR	25	5729877	1xTMT6plex [N-Term]	1.337	1.225	1.224	4.989221573	69.98	0	1920.888513	2	960.9464722	-1.481285375	-0.001422692	30.39884457
																			TVTNAVVTVPAYFNDSQR	4	5729877	1xTMT6plex [N-Term]	100	1.639		4.261797428	42.29	0	2211.160712	2	1106.082397	-1.444407956	-0.001596909	73.06998019
																			RFDDAVVQSDMK	18	5729877	2xTMT6plex [N-Term; K12]	1.284	1.263	1.26	5.758730412	46.57	1	1868.994054	3	629.0007935	-0.605663442	-0.000380556	39.43358259
																			RFDDAVVQSDMK	16	5729877	2xTMT6plex [N-Term; K12]; 1xOxidation [M11	1.231	1.22	1.17	4.610640049	48.87	1	1884.988969	3	629.0007935	-0.605663442	-0.000380556	39.43358259
																			NVLIFDLGGGTFDVSILTIEDGIFEVK	11	5729877	2xTMT6plex [N-Term; K27]		100	100	3.024727106	21.9	0	3369.859781	3	1123.954956	-2.809094904	-0.003155419	115.9792002
																			VEILANDQGNR	48	5729877	1xTMT6plex [N-Term]	1.325	1.311	1.289	4.071812153	54.4	0	1457.790972	2	729.3981323	-1.360662537	-0.000991781	36.49181231
																			TTPSYVAFTDTER	12	5729877	1xTMT6plex [N-Term]	1.291	1.404		4.477664471	71.19	0	1716.864196	2	858.9349365	-0.931671316	-0.000799777	51.60226222
																			LDKSQIHDIVLVGGSTR	2	5729877	2xTMT6plex [N-Term; K3]	0.201			5.119594574	13.91	1	2296.3389	4	574.8391724	-1.759647443	-0.001010187	60.86487838
																			NSLESYAFNMK	4	5729877	2xTMT6plex [N-Term; K11]	1.554	1.487		3.928229809	53.61	0	1761.924257	2	881.4658813	-0.051786943	-4.56E-05	79.67368307
																			FEELNADLFR	13	5729877	1xTMT6plex [N-Term]	100	2.908	2.228	3.312972784	45.32	0	1482.77901	2	741.8928833	-0.35067831	-0.000259989	73.42433322
																			HWPFMVVNDAGRPK	2	5729877	2xTMT6plex [N-Term; K14]; 1xOxidation [M5	1.277			4.490510464	6.01	0	2128.15262	4	532.7942505	1.198961532	0.000637893	60.00648627
																			DAGTIAGLNVLR	4	5729877	1xTMT6plex [N-Term]			100	3.229917049	56.94	0	1428.837194	2	714.9226685	0.606614992	0.000433377	72.49668107
																			NQVAMNPTNTVFDAK	4	5729877	2xTMT6plex [N-Term; K15]	1.044	100		2.555155277	26.88	0	2108.121045	2	1054.5625	-1.575678023	-0.00166086	57.9579872
																			EIAEAYLGK	4	5729877	2xTMT6plex [N-Term; K9]	1.06	1.182		3.895078421	44.34	0	1451.851002	2	726.4281616	-1.346679384	-0.000977589	58.64433833
																			GVPQIEVTFDIDANGILNVSAVDK	7	5729877	2xTMT6plex [N-Term; K24]	100	0.618		3.692738295	42.82	0	2972.634473	3	991.5429077	-6.829950219	-0.006767648	117.3845648
																			MVNHFIAEFK	14	5729877	2xTMT6plex [N-Term; K10]	1.263	1.688		3.849700689	40.28	0	1693.950004	2	847.4785156	-0.147401045	-0.000124845	82.99665545
																			FDDAVVQSDMK	8	5729877	2xTMT6plex [N-Term; K11]		1.19	4.702	2.99061799	32.7	0	1712.892943	2	856.9483032	-2.109299768	-0.001806502	52.9249257
																			LLQDFFNGK	2	5729877	2xTMT6plex [N-Term; K9]		1.486		3.42045927	44.27	0	1539.893535	2	770.4494019	-1.304135004	-0.001004115	97.18023949
																			CNEIINWLDK	4	5729877	2xTMT6plex [N-Term; K10]; 1xCarbamidomethyl [C1	2.538	100		2.959228992	32.72	0	1762.956212	2	881.9818726	0.145424592	0.000128189	94.55322572
																			QTQTFTTYSDNQPGVLIQVYEGER	4	5729877	1xTMT6plex [N-Term]				2.952844143	11.67	0	3003.48971	3	1001.830505	-4.244095352	-0.004249032	89.6290735
																			LYQSAGGMPGGMPGGFPGGGAPPSGGASSGPTIEEVD	6	5729877	1xTMT6plex [N-Term]				1.557358861	6.69	0	3575.662013	3	1192.556885	-1.65342057	-0.001970691	77.14545513
																			VCNPIITK	19	5729877	2xTMT6plex [N-Term; K8]; 1xCarbamidomethyl [C2	1.555	1.457	1.295	3.791148663	48.52	0	1402.849228	2	701.9276733	-0.825448872	-0.00057899	50.88975585
																			SFYPEEVSSMVLTK	10	5729877	2xTMT6plex [N-Term; K14]	1.627	0.434		2.746345997	29.18	0	2075.1135	2	1038.058838	-1.494361031	-0.001550484	94.64857627
																			MVNHFIAEFK	6	5729877	2xTMT6plex [N-Term; K10]; 1xOxidation [M1	1.069	100	100	2.999364138	27.65	0	1709.944919	2	847.4785156	-0.147401045	-0.000124845	82.99665545
																			EEFEHQQK	8	5729877	2xTMT6plex [N-Term; K8]	1.267	1.462	1.445	3.319740534	32.47	0	1532.810928	3	511.6083069	-0.365632638	-0.000186815	31.662042
																			GTLDPVEK	10	5729877	2xTMT6plex [N-Term; K8]	1.276	1.29	1.236	3.339602709	44.69	0	1316.782588	2	658.8961182	1.801191895	0.001185889	44.13479988
																			IINEPTAAAIAYGLDKK	6	5729877	3xTMT6plex [N-Term; K16; K17]	0.722	100	1.253	2.591082573	15.6	1	2475.478972	3	825.8303833	-0.959413383	-0.000791669	77.50180372
																			VQVEYKGETK	10	5729877	3xTMT6plex [N-Term; K6; K10]	0.332	0.305	0.326	2.194934607	13.15	1	1868.109625	3	623.3751831	0.733937874	0.000457025	39.04477283
																			MKEIAEAYLGK	7	5729877	3xTMT6plex [N-Term; K2; K11]	0.439		0.507	2.990371943	8.28	1	1940.149381	3	647.3876343	-0.531787902	-0.000343916	81.77043035
																			LIGDAAK	8	5729877	2xTMT6plex [N-Term; K7]	1.088	1.128	0.991	2.640163898	25.21	0	1145.72943	2	573.368042	-0.543667299	-0.000311448	38.40743522
																			ITITNDK	9	5729877	2xTMT6plex [N-Term; K7]	1.307	1.284	1.082	3.007526398	39.15	0	1262.772023	2	631.8894043	-0.389061716	-0.000245648	34.74030574
																			MVQEAEK	5	5729877	2xTMT6plex [N-Term; K7]	1.286	1.351	1.238	3.254307032	41.37	0	1292.728444	2	646.868042	0.281152919	0.000181727	35.4466632
																			ATVEDEK	10	5729877	2xTMT6plex [N-Term; K7]	1.7	1.71	1.527	3.137670517	33.96	0	1249.704003	2	625.3555298	-0.176289495	-0.000110155	35.59531556
																			ILDKCNEIINWLDK	2	5729877	3xTMT6plex [N-Term; K4; K14]; 1xCarbamidomethyl [C5	0.251			2.050107718	6.82	1	2461.409178	3	821.1409302	-0.382143962	-0.000313538	99.88360325
																			DNNLLGK	7	5729877	2xTMT6plex [N-Term; K7]	0.943	0.985	100	2.7672925	26.74	0	1231.741058	2	616.3742676	0.163196841	0.000100508	49.63860726
5174735	TUBB4B	tubulin beta-4B chain	10383	NP_006079.1	81.12359551	1	3	23	487	445	49.799	4.89	1.22	1.21	1.17	1.2	Significant	0.00016																
																			EVDEQMLNVQNK	32	5174735	2xTMT6plex [N-Term; K12]	1.281	1.248	1.252	4.451275349	65.29	0	1905.015183	2	953.0117798	0.577339395	0.00054992	49.32032946
																			FWEVISDEHGIDPTGTYHGDSDLQLER	24	5174735	1xTMT6plex [N-Term]	1.559	100	2.844	5.5370121	35.43	0	3345.586129	4	837.1473389	-5.560697149	-0.004650948	75.43246504
																			INVYYNEATGGK	10	5174735	2xTMT6plex [N-Term; K12]	1.242	1.429	0.998	4.174167633	48.84	0	1786.97397	2	893.9906616	0.0427047	3.82E-05	68.49879915
																			AVLVDLEPGTMDSVR	27	5174735	1xTMT6plex [N-Term]	1.203	0.928	1.186	3.73712945	37.34	0	1830.983265	2	923.9922485	-0.519643219	-0.000479885	64.18555723
																			GHYTEGAELVDSVLDVVR	33	5174735	1xTMT6plex [N-Term]	1.17	1.17	1.141	5.213302612	59.43	0	2188.144728	3	730.0530396	-0.074166783	-5.41E-05	91.62180174
																			KEAESCDCLQGFQLTHSLGGGTGSGMGTLLISK	8	5174735	3xTMT6plex [N-Term; K1; K33]; 2xCarbamidomethyl [C6; C8]; 1xOxidation [M26			1.41	4.637863159	18.13	1	4143.112761	5	826.2288208	-0.690194998	-0.000569703	81.6597482
																			KEAESCDCLQGFQLTHSLGGGTGSGMGTLLISK	24	5174735	3xTMT6plex [N-Term; K1; K33]; 2xCarbamidomethyl [C6; C8	1.222	4.053	1.055	6.228266716	38.42	1	4127.117846	5	826.2288208	-0.690194998	-0.000569703	81.6597482
																			EAESCDCLQGFQLTHSLGGGTGSGMGTLLISK	24	5174735	2xTMT6plex [N-Term; K32]; 2xCarbamidomethyl [C5; C7]; 1xOxidation [M25	1.755		1.061	3.532485008	21.69	0	3785.854866	3	1257.289917	-1.260875337	-0.001584441	87.29228064
																			EAESCDCLQGFQLTHSLGGGTGSGMGTLLISK	40	5174735	2xTMT6plex [N-Term; K32]; 2xCarbamidomethyl [C5; C7	1.363	1.338	1.632	5.571903706	46.39	0	3769.859951	3	1257.289917	-1.260875337	-0.001584441	87.29228064
																			VSDTVVEPYNATLSVHQLVENTDETYCIDNEALYDICFR	32	5174735	1xTMT6plex [N-Term]; 2xCarbamidomethyl [C27; C37	3.933	2.117		4.833796501	31.32	0	4822.270146	4	1206.324219	1.015870088	0.0012247	101.9591467
																			EIVHLQAGQCGNQIGAK	66	5174735	2xTMT6plex [N-Term; K17]; 1xCarbamidomethyl [C10	1.015	1.041	1.03	5.308478355	74.46	0	2281.248705	3	761.0859375	-2.387381081	-0.001815403	47.31921529
																			MSATFIGNSTAIQELFK	6	5174735	2xTMT6plex [N-Term; K17]				3.548536539	27.12	0	2316.267375	3	772.7601318	-0.661686213	-0.000510881	103.8703781
																			AVLVDLEPGTMDSVR	16	5174735	1xTMT6plex [N-Term]; 1xOxidation [M11]	1.116	0.944	2.116	2.708437204	44.29	0	1846.97818	2	923.9922485	-0.519643219	-0.000479885	64.18555723
																			IMNTFSVVPSPK	54	5174735	2xTMT6plex [N-Term; K12]	1.243	100	3.414	3.53404808	40.35	0	1778.028648	2	889.5175781	-0.432417104	-0.000384425	77.97312852
																			EVDEQMLNVQNK	31	5174735	2xTMT6plex [N-Term; K12]; 1xOxidation [M6	1.194	1.216	1.331	3.290565252	45.22	0	1921.010098	2	953.0117798	0.577339395	0.00054992	49.32032946
																			FPGQLNADLR	24	5174735	1xTMT6plex [N-Term]	1.108	1.147	1.255	3.543502092	43.73	0	1359.758215	2	680.3824463	-0.440557643	-0.000299526	52.32976605
																			ISEQFTAMFR	23	5174735	1xTMT6plex [N-Term]	1.376	2.039	100	4.103599548	44.54	0	1458.761251	2	729.8840942	-0.232802617	-0.000169802	80.40800158
																			LTTPTYGDLNHLVSATMSGVTTCLR	11	5174735	1xTMT6plex [N-Term]; 1xCarbamidomethyl [C23		100	1.854	5.839260101	41.43	0	2937.501143	3	979.8379517	-0.626968107	-0.000613907	98.35790161
																			YLTVAAVFR	24	5174735	1xTMT6plex [N-Term]	1.536	100	100	3.138554335	48.41	0	1268.756424	2	634.8815308	-0.503860749	-0.000319638	77.4217302
																			ALTVPELTQQMFDAK	22	5174735	2xTMT6plex [N-Term; K15]	1.226	1.258	1.163	2.925591469	30.01	0	2150.193147	2	1075.599365	-0.787627499	-0.000846776	86.5537756
																			ALTVPELTQQMFDAK	9	5174735	2xTMT6plex [N-Term; K15]; 1xOxidation [M11	2.039	100	100	2.555007935	25.04	0	2166.188062	2	1075.599365	-0.787627499	-0.000846776	86.5537756
																			LAVNMVPFPR	4	5174735	1xTMT6plex [N-Term]	1.652	1.913		3.099212646	32.08	0	1372.797243	2	686.9009399	-1.922976755	-0.001319929	73.50072
																			LHFFMPGFAPLTSR	4	5174735	1xTMT6plex [N-Term]	1.204		100	3.054291487	30.56	0	1849.998462	3	617.3383789	1.146778656	0.00070718	88.15653368
																			LAVNMVPFPR	4	5174735	1xTMT6plex [N-Term]; 1xOxidation [M5]	1.084	100		1.945205092	20.38	0	1388.792158	2	686.9009399	-1.922976755	-0.001319929	73.50072
																			ISEQFTAMFR	2	5174735	1xTMT6plex [N-Term]; 1xOxidation [M8]	100			1.61943233	17.77	0	1474.756166	2	729.8840942	-0.232802617	-0.000169802	80.40800158
																			NMMAACDPR	2	5174735	1xTMT6plex [N-Term]; 1xCarbamidomethyl [C6	1.158			2.256533384	24.59	0	1294.590363	2	647.7979736	-1.307629365	-0.000846422	36.95700301
																			NSSYFVEWIPNNVK	3	5174735	2xTMT6plex [N-Term; K14]				2.179470062	13.24	0	2155.158811	2	1078.081421	-1.505992556	-0.001622827	78.89611646
																			SGPFGQIFRPDNFVFGQSGAGNNWAK	1	5174735	2xTMT6plex [N-Term; K26]				1.948169589		0	3256.669236	3	1086.226318	-1.484280879	-0.001611271	96.06523866
																			IREEYPDR	4	5174735	1xTMT6plex [N-Term]	1.255	1.279		4.01290226	23.39	1	1306.69528	3	436.2364197	-0.439748123	-0.000191539	32.87033097
																			TAVCDIPPR	2	5174735	1xTMT6plex [N-Term]; 1xCarbamidomethyl [C4	0.793			2.451532364	8.06	0	1257.682273	2	629.3438721	-1.435751631	-0.00090286	42.35901416
																			LTTPTYGDLNHLVSATMSGVTTCLR	2	5174735	1xTMT6plex [N-Term]; 1xCarbamidomethyl [C23]; 1xOxidation [M17]				1.185863256		0	2953.496058	3	979.8379517	-0.626968107	-0.000613907	98.35790161
62414289	VIM	vimentin	7431	NP_003371.2	71.03004292	1	29	33	632	466	53.619	5.12	1.46	1.41	1.51	1.46	Significant	0.00039																
																			TYSLGSALRPSTSR	12	62414289	1xTMT6plex [N-Term]	1.357	1.315	1.048	3.913672686	40.88	0	1724.949263	3	575.6542969	-0.53662158	-0.000308548	48.96441504

Bhat et al,2017. Cigarette smoke and chewing tobacco alter expression of different sets of miRNAs in oral keratinocytes

Supplementary Table S2(A). List of all proteins and peptides identified in OKF6/TERT1 cells chronically treated with chewing tobacco using Sequest and Mascot search algorithms

Accession	Gene_Symbol	Description	Gene_ID	Refseq_Accession	Coverage	Proteins	#Unique_Peptides	#Peptides	#PSMS	#AAs	MW [kDa]	calc. pI	OKF6-STE-6M/OKF6P_R1	OKF6-STE-6M/OKF6P_R2	OKF6-STE-6M/OKF6P_R3	Median fold change	Status	P-value (6M)	Sequence	#PSMs	Protein Accessions	Modifications	OKF6-STE-6M/OKF6P_R1	OKF6-STE-6M/OKF6P_R2	OKF6-STE-6M/OKF6P_R3	Xcorr	Ionscore	# Missed Cleavages	Theo. MH+ [Da]	Charge	m_z	DeltaM [ppm]	Deltam/z [Da]	RT [min]
																			EMEENFAVEAANYQDTIGR	13	62414289	1xTMT6plex [N-Term]; 1xOxidation [M2]	1.249	100	1.508	4.51372385	41.86	0	2432.123735	3	806.0484619	0.833143569	0.000670994	75.83185097
																			EMEENFAVEAANYQDTIGR	51	62414289	1xTMT6plex [N-Term]	1.686	1.695	2.446	5.636449337	51.45	0	2416.12882	3	806.0484619	0.833143569	0.000670994	75.83185097
																			LLQDSVDFSLADAINTEFK	113	62414289	2xTMT6plex [N-Term; K19]	1.858	1.601	4.456	4.858642578	56.78	0	2584.391055	3	862.1342773	-1.074179059	-0.000925366	113.6241365
																			ETNLDSLPLVDTHSK	22	62414289	2xTMT6plex [N-Term; K15]	1.941	1.404	3.778	5.288379192	44.43	0	2127.169769	3	709.7286987	0.833779904	0.000591197	61.55618479
																			SLYASSPGGVYATR	12	62414289	1xTMT6plex [N-Term]	1.261	1.602		4.400829315	57.53	0	1657.874701	2	829.4414063	0.503494021	0.000417365	50.87825743
																			KVESLQEEIAFLK	30	62414289	3xTMT6plex [N-Term; K1; K13]	1.484	1.462	1.516	6.260183811	34.06	1	2221.341081	3	741.1178589	-0.92642677	-0.00068597	73.3895496
																			EEAENTLQSFR	20	62414289	1xTMT6plex [N-Term]	1.742	1.775	100	4.392612457	69.79	0	1552.780466	2	776.8928223	-1.351452311	-0.001049254	54.17515441
																			DGQVINETSQHHDDLE	31	62414289	1xTMT6plex [N-Term]	1.358	1.311	1.674	3.668993235	65.09	0	2065.962406	2	1033.484375	-0.451532901	-0.000466425	41.88373187
																			FADLSEAANR	25	62414289	1xTMT6plex [N-Term]	1.323	1.613	1.619	3.654096842	59.85	0	1322.690195	2	661.8482056	-0.801765365	-0.000530244	41.46193909
																			MALDIEIATYR	28	62414289	1xTMT6plex [N-Term]	1.464	1.826	1.053	3.592049837	47.4	0	1524.829331	2	762.9169922	-1.720326955	-0.001311602	76.72727952
																			QDVDNASLAR	8	62414289	1xTMT6plex [N-Term]	1.683	1.166		3.812219143	48.28	0	1317.696009	2	659.3510742	-0.862727437	-0.000568406	32.88120589
																			QVQSLTCEVDALK	11	62414289	2xTMT6plex [N-Term; K13]; 1xCarbamidomethyl [C7	1.487	2.52		3.498388529	43.66	0	1949.077784	2	975.0431519	0.637958602	0.000621715	80.53224231
																			TNEKVELQELNDR	4	62414289	2xTMT6plex [N-Term; K4]	0.921	1.246		4.095453739	23.97	1	2046.123154	3	682.7125244	-0.065268707	-4.45E-05	45.54973126
																			QQYESVAAK	8	62414289	2xTMT6plex [N-Term; K9]	1.321	1.387	1.451	4.408474445	41.43	0	1481.836414	2	741.4207153	-1.525334319	-0.001130148	38.91426739
																			DNLAEDIMR	18	62414289	1xTMT6plex [N-Term]	1.569	1.717	1.572	3.640087605	50.13	0	1305.667017	2	653.3363647	-1.19774628	-0.000781929	58.92958856
																			NLQEAEEWYK	13	62414289	2xTMT6plex [N-Term; K10]	1.446	2.282	1.59	3.905136108	41.36	0	1767.931771	2	884.4692993	-0.254052331	-0.000224574	65.52317148
																			ILLAELEQLK	13	62414289	2xTMT6plex [N-Term; K10]	2.998	1.622	1.752	3.592982292	46.87	0	1628.039865	2	814.5229492	-0.763680624	-0.000621651	100.8121365
																			LGDLYEEEMR	6	62414289	1xTMT6plex [N-Term]	100		100	3.234773397	34.9	0	1483.730011	2	750.3650513	-1.400197397	-0.001049955	48.9763126
																			ISLPLPNFSSLNLR	13	62414289	1xTMT6plex [N-Term]	1.821	1.357	1.373	3.099948168	37.91	0	1800.058085	2	900.5325317	-0.165746561	-0.000149177	96.82765352
																			EYQDLLNVK	8	62414289	2xTMT6plex [N-Term; K9]	1.577	1.391		3.344995975	42.56	0	1579.909579	2	790.4584961	0.086288166	6.82E-05	69.52971198
																			VELQELNDR	12	62414289	1xTMT6plex [N-Term]	1.35	0.567	1.603	3.589381695	50.87	0	1344.73206	2	672.869812	0.213889031	0.000143812	44.05252355
																			LQEEMLQR	12	62414289	1xTMT6plex [N-Term]	1.513	0.789		3.088406324	51.05	0	1275.69238	2	638.3494873	-0.89331898	-0.0005698	44.9204893
																			EKLQEEMLQR	18	62414289	2xTMT6plex [N-Term; K2]	1.242	1.179	1.082	3.169742346	31.95	1	1761.993326	2	881.4959717	-4.914218743	-0.00432941	42.48884168
																			LGDLYEEEMR	6	62414289	1xTMT6plex [N-Term]; 1xOxidation [M9]	1.357	4.387		2.526463747	35.64	0	1499.724926	2	750.3650513	-1.400197397	-0.001049955	48.9763126
																			QVDQLTNDK	11	62414289	2xTMT6plex [N-Term; K9]	1.501	1.631	1.552	4.106999874	46.96	0	1518.852793	2	759.9295654	-0.617828554	-0.000469195	43.7137048
																			LQDEIQNMK	7	62414289	2xTMT6plex [N-Term; K9]	1.843	2.09	2.609	3.082299948	45.1	0	1576.876899	2	788.9414063	-0.8643858	-0.000681515	54.58344498
																			LQDEIQNMK	12	62414289	2xTMT6plex [N-Term; K9]; 1xOxidation [M8]	1.961	2.141	2.159	2.923891068	43	0	1592.871814	2	788.9414063	-0.8643858	-0.000681515	54.58344498
																			GTNESLER	14	62414289	1xTMT6plex [N-Term]	2.03	1.492	1.481	3.166389942	39.53	0	1134.595232	2	567.8010864	-0.295972015	-0.000167904	22.11019192
																			SVSSSSYR	10	62414289	1xTMT6plex [N-Term]	1.591	1.685	1.611	3.449117899	33.86	0	1101.573768	2	551.2905884	0.119635939	6.59E-05	27.49933202
																			SYVTTSTR	6	62414289	1xTMT6plex [N-Term]		0.967	1.597	3.348783255	30.55	0	1143.620719	2	572.3135986	-0.697682686	-0.000398942	31.14447929
																			LLEGEESR	6	62414289	1xTMT6plex [N-Term]	1.498	1.484	1.512	3.487635136	41	0	1161.631283	2	581.3189697	-0.534039401	-0.000310178	40.15125271
																			DNLAEDIMR	8	62414289	1xTMT6plex [N-Term]; 1xOxidation [M8]		0.652	1.558	1.986774683	26.09	0	1321.661932	2	653.3363647	-1.19774628	-0.000781929	58.92958856
																			KLLEGEESR	14	62414289	2xTMT6plex [N-Term; K1]	0.985	0.987	0.995	3.586319447	34.85	1	1518.889178	2	759.9478149	-0.543085828	-0.000412444	38.7728649
																			FANYIDK	6	62414289	2xTMT6plex [N-Term; K7]	1.677	1.712	1.086	3.258991003	39.76	0	1328.761459	2	664.8847656	0.599069152	0.00039801	61.70992036
																			FLEQQNK	19	62414289	2xTMT6plex [N-Term; K7]	0.833	0.763	0.782	2.620012999	32.3	0	1364.793821	2	682.9000854	-0.679261254	-0.000463526	39.10866059
																			LQEEMLQR	6	62414289	1xTMT6plex [N-Term]; 1xOxidation [M5]	1.459	1.487	1.131	1.953011036	25.18	0	1291.687753	2	638.3494873	-0.89331898	-0.0005698	44.9204893
																			QESTEYR	6	62414289	1xTMT6plex [N-Term]		100		2.272958279	29.3	0	1141.568683	2	571.288147	0.292908624	0.000167188	21.81209095
16507237	HSPA5	78 kDa glucose-regulated protein precursor	3309	NP_005338.1	52.75229358	1	31	34	344	654	72.288	5.16	0.9	0.98	1.09	0.99	Significant	0.0031																
																			DAGTIAGLNVMR	8	16507237	1xTMT6plex [N-Term]	1.068	1.251	1.766	4.395112038	62.3	0	1446.793614	2	723.9013672	1.274248782	0.000921788	52.72079704
																			IEWLESHQDADIEDFK	9	16507237	2xTMT6plex [N-Term; K16]	1.175	1.17	0.921	4.511322498	31.22	0	2433.233826	3	811.7510986	2.020602535	0.001638866	79.77475539
																			SQIFSTASDNQPTVTIK	12	16507237	2xTMT6plex [N-Term; K17]	1.239	0.929	1.285	4.124456882	54.17	0	2295.259647	3	765.756897	-1.52890852	-0.001169747	55.33133695
																			KVTHAVVTVPAYFNDAQR	2	16507237	2xTMT6plex [N-Term; K1]	0.779			4.641946793	36.47	1	2474.391998	3	825.4716797	3.430059791	0.002829104	58.99393238
																			VTHAVVTVPAYFNDAQR	12	16507237	1xTMT6plex [N-Term]	0.904	1.123	0.829	4.447392464	33.15	0	2117.134104	3	706.3842773	1.972119406	0.001391747	41.80735945
																			IINEPTAAAIAYGLDK	31	16507237	2xTMT6plex [N-Term; K16]	1.572	1.737	2.328	4.13507843	46.05	0	2118.221077	2	1059.61499	0.768167576	0.000813574	75.35766658
																			IEIESFYEGEDFSETLTR	30	16507237	1xTMT6plex [N-Term]	0.87	0.862	0.905	4.393001556	55.2	0	2394.155017	3	798.7234497	0.325125603	0.000259467	91.73649665
																			DNHLLGTFDLTGIPPAPR	19	16507237	1xTMT6plex [N-Term]	0.802	0.769	1.14	5.198044777	43.94	0	2163.175968	3	721.7296753	-0.691432928	-0.000498564	80.23255315
																			EDVGTVVGIDLGTTYSCVGVFK	2	16507237	2xTMT6plex [N-Term; K22]; 1xCarbamidomethyl [C17			100	3.672683001	36.9	0	2774.468654	3	925.494812	0.443066875	0.000409758	97.53600296
																			KEDVGTVVGIDLGTTYSCVGVFK	4	16507237	3xTMT6plex [N-Term; K1; K23]; 1xCarbamidomethyl [C18	0.828			5.679690361	35.59	1	3131.726548	3	1044.579712	-0.627761646	-0.000655326	87.88675324
																			TKPYIQVDIGGGQTK	8	16507237	3xTMT6plex [N-Term; K2; K15]	1.025		1.099	5.738684654	25.39	0	2292.353043	3	764.7890015	-0.258165063	-0.000197268	55.71849878
																			KTKPYIQVDIGGGQTK	4	16507237	4xTMT6plex [N-Term; K1; K3; K16]	0.498			5.341244221	22.57	1	2649.610938	4	663.1586304	0.661868635	0.000438424	53.98080521
																			VEILANDQGNR	48	16507237	1xTMT6plex [N-Term]	1.325	1.311	1.289	4.071812153	54.4	0	1457.790972	2	729.3981323	-1.360662537	-0.000991781	36.49181231
																			AKFEELNMDLFR	12	16507237	2xTMT6plex [N-Term; K2]	0.691	0.654	0.897	3.669103146	22.3	1	1971.077389	3	657.6965332	-1.18875628	-0.000781044	72.78589507
																			KSQIFSTASDNQPTVTIK	6	16507237	3xTMT6plex [N-Term; K1; K18]	0.01	100	100	3.260824919	20.2	1	2652.517542	3	884.8421631	-2.113411919	-0.001868621	50.691226
																			DAGTIAGLNVMR	9	16507237	1xTMT6plex [N-Term]; 1xOxidation [M11]	1.027	1.159	0.993	3.05665803	60.91	0	1462.788529	2	723.9013672	1.274248782	0.000921788	52.72079704
																			NQLTSNPENTVFDAK	6	16507237	2xTMT6plex [N-Term; K15]	1.037	100	0.714	2.609163046	38.04	0	2136.133718	2	1068.571289	0.741201258	0.000791652	52.13826646
																			ETAEAYLGK	14	16507237	2xTMT6plex [N-Term; K9]	1.403	1.613	1.787	3.929911375	56.62	0	1439.814616	2	720.4109497	0.004524237	3.26E-06	50.92426819
																			NELESYAYSLK	6	16507237	2xTMT6plex [N-Term; K11]	0.521	1.743	1.044	3.569284201	34.21	0	1774.962737	2	887.9842529	-0.849409733	-0.000753835	67.98629183
																			TWNDPSVQQDIK	8	16507237	2xTMT6plex [N-Term; K12]	1.121	1.06	1.065	3.905380964	53.45	0	1889.016898	2	945.0116577	-0.454670529	-0.00042944	50.41576348
																			LYGSAGPPPTGEEDTAEKDEL	10	16507237	2xTMT6plex [N-Term; K18]			100	2.12102294	16.31	1	2634.318678	2	1317.662842	-0.102768982	-0.000135363	60.08216626
																			VYEGERPLTK	5	16507237	2xTMT6plex [N-Term; K10]	0.602	1.092	0.772	2.726590157	16.86	0	1649.962677	3	550.6594238	0.63081086	0.000346938	44.53907929
																			ITITNDQNR	10	16507237	1xTMT6plex [N-Term]	0.895	1.023	1.119	3.688944817	48.41	0	1303.716744	2	652.3623047	0.451528296	0.000294332	42.12350679
																			TFAPEEISAMVLTK	8	16507237	2xTMT6plex [N-Term; K14]	3.263	100	0.853	2.621210814	23.79	0	1995.123671	2	998.0639038	-1.573803598	-0.001569966	86.25898481
																			VLEDSDLK	19	16507237	2xTMT6plex [N-Term; K8]	1.111	1.044	1.033	3.05724287	49.91	0	1376.803717	2	688.9064331	1.359918567	0.00093617	60.37534841
																			ELEEIVQPIISK	2	16507237	2xTMT6plex [N-Term; K12]				2.13461566	16.62	0	1856.114486	2	928.5609741	0.099747181	9.26E-05	87.00134716
																			ALSSQHQAR	2	16507237	1xTMT6plex [N-Term]	0.876			3.337975025	32.28	0	1226.680299	3	409.5645142	-1.067800251	-0.000436617	21.94540544
																			VLEDSDLKK	8	16507237	3xTMT6plex [N-Term; K8; K9]	0.747	0.787		2.836889982	37.08	1	1734.061612	2	867.5341187	-0.375722124	-0.000325763	52.9057559
																			LSSEDKETMEK	6	16507237	3xTMT6plex [N-Term; K6; K11]	1.883	1.402		2.857285023	11.86	1	1984.087569	3	662.0344849	0.671608383	0.000444177	39.84818011
																			FEELNMDLFR	8	16507237	1xTMT6plex [N-Term]	100	0.703	100	2.23770237	27.24	0	1542.782381	2	771.8943481	-0.622972462	-0.000480555	97.8133628
																			IINEPTAAAIAYGLDKR	5	16507237	2xTMT6plex [N-Term; K16]	100	1.049		2.210192442	8.84	1	2274.322188	3	758.7784424	-0.621618858	-0.000471254	67.31464135
																			VMEHFIK	13	16507237	2xTMT6plex [N-Term; K7]	0.93	0.84		3.247190475	36.79	0	1361.801549	2	681.4048462	0.636247486	0.000433221	55.50246406
																			STMKPVQK	6	16507237	3xTMT6plex [N-Term; K4; K8]	0.94	1.111		2.682941437	13.12	0	1605.99651	2	803.5018311	-0.077323098	-6.21E-05	36.95571872
																			LIGDAAK	8	16507237	2xTMT6plex [N-Term; K7]	1.088	1.128	0.991	2.640163898	25.21	0	1145.72943	2	573.368042	-0.543667299	-0.000311448	38.40743522
																			MKETAEAYLGK	2	16507237	3xTMT6plex [N-Term; K2; K11]; 1xOxidation [M1			0.808	2.623819351	26.82	1	1944.107911	3	648.7068481	-0.987407332	-0.000639875	43.15017881
																			VMEHFIK	2	16507237	2xTMT6plex [N-Term; K7]; 1xOxidation [M2]		0.926	0.676	2.552256823	23.29	0	1377.796464	2	681.4048462	0.636247486	0.000433221	55.50246406
																			LSSEDKETMEK	3	16507237	3xTMT6plex [N-Term; K6; K11]; 1xOxidation [M9	1.572	1.721		1.925785303	5.57	1	2000.082484	3	662.0344849	0.671608383	0.000444177	39.84818011
																			TFAPEEISAMVLTK	1	16507237	2xTMT6plex [N-Term; K14]; 1xOxidation [M10				0.884798229		0	2011.118586	2	998.0639038	-1.573803598	-0.001569966	86.25898481
29788785	TUBB	tubulin beta chain isoform b	203068	NP_821133.1	81.30630631	1	4	23	488	444	49.639	4.89	1.07	1.12	1.15	1.11	Significant	0.00044																
																			EVDEQMLNVQNK	32	29788785	2xTMT6plex [N-Term; K12]	1.281	1.248	1.252	4.451275349	65.29	0	1905.015183	2	953.0117798	0.577339395	0.00054992	49.32032946
																			FWEVISDEHGIDPTGTYHGDSDLQLDR	21	29788785	1xTMT6plex [N-Term]	1.31	1.438	1.028	5.795618534	38.68	0	3331.570479	4	833.649231	1.385120967	0.001153657	82.40293913
																			AILVDLEPGTMDSVR	24	29788785	1xTMT6plex [N-Term]	0.923	0.984	1.029	3.743171215	38.6	0	1844.998915	2	923.0027466	-0.378718888	-0.000349368	75.34357767
																			GHYTEGAELVDSVLDVVR	33	29788785	1xTMT6plex [N-Term]	1.17	1.17	1.141	5.213302612	59.43	0	2188.144728	3	730.0530396	-0.074166783	-5.41E-05	91.62180174
																			KEAESCDCLQGFQLTHSLGGGTGSGMGTLLISK	8	29788785	3xTMT6plex [N-Term; K1; K33]; 2xCarbamidomethyl [C6; C8]; 1xOxidation [M26			1.41	4.637863159	18.13	1	4143.112761	5	826.2288208	-0.690194998	-0.000569703	81.6597482
																			KEAESCDCLQGFQLTHSLGGGTGSGMGTLLISK	24	29788785	3xTMT6plex [N-Term; K1; K33]; 2xCarbamidomethyl [C6; C8	1.222	4.053	1.055	6.228266716	38.42	1	4127.117846	5	826.2288208	-0.690194998	-0.000569703	81.6597482
																			EAESCDCLQGFQLTHSLGGGTGSGMGTLLISK	24	29788785	2xTMT6plex [N-Term; K32]; 2xCarbamidomethyl [C5; C7]; 1xOxidation [M25	1.755		1.061	3.532485008	21.69	0	3785.854866	3	1257.289917	-1.260875337	-0.001584441	87.29228064
																			EAESCDCLQGFQLTHSLGGGTGSGMGTLLLSK	40	29788785	2xTMT6plex [N-Term; K32]; 2xCarbamidomethyl [C5; C7	1.363	1.338	1.632	5.571903706	46.39	0	3769.859951	3	1257.289917	-1.260875337	-0.001584441	87.29228064
																			MAVTFIGNSTAIQELFK	10	29788785	2xTMT6plex [N-Term; K17]	1.146		3.025	3.853271008	31.8	0	2328.30376	3	776.7723389	-0.557087292	-0.000432356	107.8157115
																			VSDTVVEPYNATLSVHQLVENTDETYCIDNEALYDICFR	32	29788785	1xTMT6plex [N-Term]; 2xCarbamidomethyl [C27; C37	3.933	2.117		4.833796501	31.32	0	4822.270146	4	1206.324219	1.015870088	0.0012247	101.9591467
																			EIVHLQAGQCGNQIGAK	66	29788785	2xTMT6plex [N-Term; K17]; 1xCarbamidomethyl [C10	1.015	1.041	1.03	5.308478355	74.46	0	2281.248705	3	761.0859375	-2.387381081	-0.001815403	47.31921529
																			ISVYYNEATGGK	16	29788785	2xTMT6plex [N-Term; K12]	1.334	1.885	1.533	3.660461664	41.25	0	1759.963071	2	880.4840698	-1.254714715	-0.001104126	53.93269396
																			IMNTFSVVPSPK	54	29788785	2xTMT6plex [N-Term; K12]	1.243	100	3.414	3.53404808	40.35	0	1778.028648	2	889.5175781	-0.432417104	-0.000384425	77.97312852
																			EVDEQMLNVQNK	31	29788785	2xTMT6plex [N-Term; K12]; 1xOxidation [M6	1.194	1.216	1.331	3.290565252	45.22	0	1921.010098	2	953.0117798	0.577339395	0.00054992	49.32032946
																			FPGQLNADLR	24	29788785	1xTMT6plex [N-Term]	1.108	1.147	1.255	3.543502092	43.73	0	1359.758215	2	680.3824463	-0.440557643	-0.000299526	52.32976605
																			ISEQFTAMFR	23	29788785	1xTMT6plex [N-Term]	1.376	2.039	100	4.103599548	44.54	0	1458.761251	2	729.8840942	-0.232802617	-0.000169802	80.40800158
																			AILVDLEPGTMDSVR	14	29788785	1xTMT6plex [N-Term]; 1xOxidation [M11]	0.902	1.387	1.301	2.488433123	28.93	0	1860.99383	2	923.0027466	-0.378718888	-0.000349368	75.34357767
																			LTTPTYGDLNHLVSATMSGVTTCLR	11	29788785	1xTMT6plex [N-Term]; 1xCarbamidomethyl [C23		100	1.854	5.839260101	41.43	0	2937.501143	3	979.8379517	-0.626968107	-0.000613907	98.35790161
																			YLTVAAVFR	24	29788785	1xTMT6plex [N-Term]	1.536	100	100	3.138554335	48.41	0	1268.756424	2	634.8815308	-0.503860749	-0.000319638	77.4217302
																			ALTVPELTQQVFDAK	30	29788785	2xTMT6plex [N-Term; K15]	1.229	1.141	1.143	2.99148345	29.74	0	2118.221077	2	1059.613159	-0.960693219	-0.00101748	85.02429208
																			LAVNMVPFPR	4	29788785	1xTMT6plex [N-Term]	1.652	1.913		3.099212646	32.08	0	1372.797243	2	686.9009399	-1.922976755	-0.001319929	73.50072
																			LHFFMPGFAPLTSR	4	29788785	1xTMT6plex [N-Term]	1.204		100	3.054291487	30.56	0	1849.998462	3	617.3383789	1.146778656	0.00070718	88.15653368
																			LAVNMVPFPR	4	29788785	1xTMT6plex [N-Term]; 1xOxidation [M5]	1.084	100		1.945205092	20.38	0	1388.792158	2	686.9009399	-1.922976755	-0.001319929	73.50072
																			ISEQFTAMFR	2	29788785	1xTMT6plex [N-Term]; 1xOxidation [M8]	100			1.61943233	17.77	0	1474.756166	2	729.8840942	-0.232802617	-0.000169802	80.40800158
																			NMMAACDPR	2	29788785	1xTMT6plex [N-Term]; 1xCarbamidomethyl [C6	1.158			2.256533384	24.59	0	1294.590363	2	647.7979736	-1.307629365	-0.000846422	36.95700301
																			NSSYFVEWIPNNVK	3	29788785	2xTMT6plex [N-Term; K14]				2.179470062	13.24	0	2155.158811	2	1078.081421	-1.505992556	-0.001622827	78.89611646
																			SGPFGQIFRPDNFVFGQSGAGNNWAK	1	29788785	2xTMT6plex [N-Term; K26]				1.948169589		0	3256.669236	3	1086.226318	-1.484280879	-0.001611271	96.06523866
																			IREEYPDR	4	29788785	1xTMT6plex [N-Term]	1.255	1.279		4.01290226	23.39	1	1306.69528	3	436.2364197	-0.439748123	-0.000191539	32.87033097
																			TAVCDIPPR	2	29788785	1xTMT6plex [N-Term]; 1xCarbamidomethyl [C4	0.793			2.451532364	8.06	0	1257.682273	2	629.3438721	-1.435751631	-0.00090286	42.35901416
																			LTTPTYGDLNHLVSATMSGVTTCLR	2	29788785	1xTMT6plex [N-Term]; 1xCarbamidomethyl [C23]; 1xOxidation [M17]				1.185863256		0	2953.496058	3	979.8379517	-0.626968107	-0.000613907	98.35790161
5031875	LMNA	lamin isoform C	4000	NP_005563.1	58.04195804	1	1	36	450	572	65.096	6.84	0.9	0.91	0.67	0.83	Significant	0.0089																
																			SNEDQSMGNWQIK	6	5031875	2xTMT6plex [N-Term; K13]	0.886	100	1.025	4.09638834	38.99	0	1995.000596	2	997.9941406	-9.820057218	-0.00979551	51.57611777
																			NSNLVGAAHEELQQSR	10	5031875	1xTMT6plex [N-Term]	0.849	0.788		5.859630108	61.98	0	1982.025281	3	661.3460693	-0.820671766	-0.000542197	42.07806646
																			EAALSTALSEK	12	5031875	2xTMT6plex [N-Term; K11]	0.878	0.809	0.795	4.230268002	50.09	0	1577.915058	2	789.4609375	-0.29159998	-0.00023006	52.00948651
																			MQQQLDEYQELLDIK	8	5031875	2xTMT6plex [N-Term; K15]	0.799	100	0.67	3.99401021	44.09	0	2352.252119	3	784.7553711	-0.237503771	-0.000186223	92.75514387
																			TALINSTGEEVAMR	10	5031875	1xTMT6plex [N-Term]	0.81	1.414	1.237	4.020965576	45.1	0	1720.9101	2	860.9584351	-0.294508593	-0.000253411	47.8012571
																			AQHEDQVEQYK	14	5031875	2xTMT6plex [N-Term; K11]	0.778	0.755	0.706	5.622924805	54.41	0	1832.954297	2	916.9805298	-0.280694226	-0.00025725	34.8693129
																			VAVEEVDEEGK	10	5031875	2xTMT6plex [N-Term; K11]	0.77	0.804		4.93041563	70.02	0	1661.899802	2	831.4525757	-1.159848993	-0.000963776	46.56701304
																			AAYEAELGDAR	22	5031875	1xTMT6plex [N-Term]	0.627	0.681	0.708	4.936688423	74.17	0	1394.711324	2	697.8590698	-0.330743398	-0.000230646	38.95790587
																			SVTVVEDDEDEDGDDLLHHHHVSGSR	17	5031875	1xTMT6plex [N-Term]	0.898	0.914	0.672	5.734977245	45.94	0	3128.435442	3	1043.484497	1.117347953	0.001165184	39.60202537
																			ASSHSSQTQGGGSVTK	142	5031875	2xTMT6plex [N-Term; K16]	0.845	0.828	0.806	5.802528381	80.05	0	1977.040152	3	659.6849976	0.145275644	9.57E-05	28.66776877
																			LALDMEIHAYR	26	5031875	1xTMT6plex [N-Term]	0.679	0.988	0.371	4.030868053	63.49	0	1560.840564	3	520.9517822	0.146760465	7.64E-05	90.92299012
																			EGDLIAAQAR	6	5031875	1xTMT6plex [N-Term]	0.973	1.048	1.184	3.909815788	64.41	0	1272.71093	2	636.8584595	-1.012118822	-0.000644067	45.70550085
																			ITESEEVVSR	4	5031875	1xTMT6plex [N-Term]	0.831	0.932		3.479677439	53.75	0	1377.74229	2	689.3745728	-0.305704623	-0.000210591	41.87675256
																			TLEGELHDLR	16	5031875	1xTMT6plex [N-Term]	0.789	0.703	0.693	4.239264011	65.89	0	1411.774259	2	706.3909912	0.316532103	0.000223436	45.62075709
																			AQHEDQVEQYKK	4	5031875	3xTMT6plex [N-Term; K11; K12]	0.373		0.462	4.501769543	17.47	1	2190.212192	4	548.3087158	0.383977065	0.000210248	35.25731436
																			LADALQELR	13	5031875	1xTMT6plex [N-Term]	0.791	0.879	0.859	3.563678503	52.78	0	1257.736417	2	629.371582	-0.421000372	-0.000264754	62.90467401
																			SLETENAGLR	6	5031875	1xTMT6plex [N-Term]	0.722	0.876	0.871	4.305250168	51.86	0	1318.71641	2	659.8581543	-5.594642029	-0.003688873	36.56471692
																			IDSLSAQLSQLQK	5	5031875	2xTMT6plex [N-Term; K13]			1.725	2.83401227	31.95	0	1889.110798	2	945.0578003	-1.309655349	-0.001237042	73.58335544
																			SNEDQSMGNWQIK	2	5031875	2xTMT6plex [N-Term; K13]; 1xOxidation [M7	0.682			2.781531811	30.87	0	2010.995511	2	997.9941406	-9.820057218	-0.00979551	51.57611777
																			LKDLEALLNSK	4	5031875	3xTMT6plex [N-Term; K2; K11]	0.721			4.434978485	18.67	1	1931.214424	3	644.4091187	-0.839716682	-0.000540558	88.97032351
																			LEAALGEAK	11	5031875	2xTMT6plex [N-Term; K9]	0.798	0.818	100	4.151691437	51.81	0	1359.824787	2	680.4152222	-1.190862292	-0.000809682	54.89204617
																			KEGDLIAAQAR	6	5031875	2xTMT6plex [N-Term; K1]	0.794	0.987	0.839	3.364347219	24.22	1	1629.968825	3	543.994812	0.648702687	0.000352455	43.95616425
																			LALDMEIHAYR	4	5031875	1xTMT6plex [N-Term]; 1xOxidation [M5]	0.669		1.926	3.412200451	39.63	0	1576.835479	3	520.9517822	0.146760465	7.64E-05	90.92299012
																			VAVEEVDEEGKFVR	3	5031875	2xTMT6plex [N-Term; K11]	0.234	0.729		3.011364937	6.33	1	2064.137741	3	688.7176514	0.319674191	0.000219951	59.41330684
																			DLEALLNSK	4	5031875	2xTMT6plex [N-Term; K9]	1.611	100		2.399184465	24.68	0	1460.872465	2	730.9393921	-0.655724805	-0.000478965	74.16171792
																			LQEKEDLQELNDR	5	5031875	2xTMT6plex [N-Term; K4]	0.796	0.912		3.529002666	13.28	1	2088.133718	3	696.7158813	-0.300463052	-0.000209136	56.70557386
																			LLEGEEER	8	5031875	1xTMT6plex [N-Term]	0.891	0.901	0.91	3.30015111	57.52	0	1203.641848	2	602.324585	0.03776196	2.27E-05	47.24056336
																			LQTMKEELDFQK	8	5031875	3xTMT6plex [N-Term; K5; K12]	0.995	0.466	0.792	2.616175413	12.25	1	2197.250552	3	733.0877075	-0.902250954	-0.000660824	63.75229531
																			KQLQDEMLR	13	5031875	2xTMT6plex [N-Term; K1]	0.643	0.616	0.638	3.526650667	28.59	1	1618.935082	2	809.9707642	-0.513170476	-0.000415395	43.47374931
																			KTLDSVAK	4	5031875	3xTMT6plex [N-Term; K1; K8]			0.61	2.813788414	34.02	1	1548.992804	2	775.0001221	0.105255896	8.15E-05	41.71366296
																			LQLELSK	12	5031875	2xTMT6plex [N-Term; K7]	0.811	0.768	0.93	3.28993082	40.42	0	1288.824059	2	644.9160156	0.539856465	0.00034789	64.43397998
																			KLLEGEEER	2	5031875	2xTMT6plex [N-Term; K1]		0.786		2.727909088	12.36	1	1560.899743	3	520.9711914	-0.462452027	-0.000240614	36.54987775
																			LAVYIDR	13	5031875	1xTMT6plex [N-Term]	0.6	0.643	0.742	3.004096746	33.6	0	1078.645811	2	539.8256836	-1.595141317	-0.000860296	49.1983752
																			AGQVVTIWAAGAGATHSPPTDLVWK	2	5031875	2xTMT6plex [N-Term; K25]				1.77304697		0	2991.645647	3	997.8860474	-0.687885665	-0.00068597	78.83866997
																			EELDFQK	8	5031875	2xTMT6plex [N-Term; K7]	0.959	1.128		3.204521894	30.03	0	1366.761852	2	683.8839722	-0.866832843	-0.000592377	58.23378286
																			QLQDEMLR	2	5031875	1xTMT6plex [N-Term]			100	2.273235559	25.84	0	1261.677188	2	631.3423462	0.180896361	0.000114116	50.57383575
																			TLDSVAK	6	5031875	2xTMT6plex [N-Term; K7]	0.652	0.863	0.981	2.952090263	41.2	0	1191.73491	2	596.3705444	-0.920735647	-0.000548636	44.53974523
																			EDLQELNDR	2	5031875	1xTMT6plex [N-Term]	1.271			2.780449152	27.57	0	1360.690589	2	680.8494263	0.725410369	0.00049353	46.94406012
7669492	GAPDH	glyceraldehyde-3-phosphate dehydrogenase isoform 1	2597	NP_002037.2	77.6119403	1	18	18	711	335	36.03	8.46	1.16	1.15	1.01	1.1	Significant	0.0019																
																			GILGYTEHQVVSSDFNSDTHSSTFDAGAGIALNDHFVK	111	7669492	2xTMT6plex [N-Term; K38]	0.968	1.012	1.146	6.483342648	38.57	0	4495.220641	5	899.8504028	0.504213327	0.00045331	82.90931271
																			GALQNIIPASTGAAK	54	7669492	2xTMT6plex [N-Term; K15]	1.04	0.897	1.118	4.565085888	74.39	0	1870.116218	2	935.5614014	-0.369873069	-0.000345853	63.13658919
																			VPTANVSVVDLTCR	32	7669492	1xTMT6plex [N-Term]; 1xCarbamidomethyl [C13	1.059	1.08	1.009	4.459086895	81	0	1759.957385	2	880.4827271	0.450182243	0.000396151	90.15525787
																			IISNASCTTNCLAPLAK	20	7669492	2xTMT6plex [N-Term; K17]; 2xCarbamidomethyl [C7; C11	1.031	1.26	1.294	4.063583851	47.31	0	2292.245594	3	764.7529907	-0.512729541	-0.000391767	66.14530106
																			IKWGDAGAEYVVESTGVFTTMEK	4	7669492	3xTMT6plex [N-Term; K2; K23]; 1xOxidation [M21	0.194	0.374		3.669798851	19.11	1	3221.700728	3	1069.23999	-0.123294806	-0.000131749	95.02597258
																			IKWGDAGAEYVVESTGVFTTMEK	7	7669492	3xTMT6plex [N-Term; K2; K23]	0.215	0.255		5.198877811	42.97	1	3205.705813	3	1069.23999	-0.123294806	-0.000131749	95.02597258
																			WGDAGAEYVVESTGVFTTMEK	71	7669492	2xTMT6plex [N-Term; K21]; 1xOxidation [M19	0.951	0.963	1.138	4.481196404	41.29	0	2751.358769	3	912.4604492	1.075029286	0.000980199	96.92284526
																			WGDAGAEYVVESTGVFTTMEK	163	7669492	2xTMT6plex [N-Term; K21]	1.431	1.498	1.262	5.273025513	67.17	0	2735.363854	3	912.4604492	1.075029286	0.000980199	96.92284526
																			AGAHLQGGAK	22	7669492	2xTMT6plex [N-Term; K10]	1.477	1.318	1.802	4.208855152	63.45	0	1367.815954	2	684.411438	-0.259101699	-0.000177202	28.86242142
																			VIHDNFGIVEGLMTTVHAITATQK	11	7669492	2xTMT6plex [N-Term; K24]	1.315	1.09	0.835	5.704543591	43.52	0	3053.685797	4	764.175293	-2.113824221	-0.001613739	105.2418086
																			VIHDNFGIVEGLMTTVHAITATQK	4	7669492	2xTMT6plex [N-Term; K24]; 1xOxidation [M13		0.01	1.281	5.407066822	39.13	0	3069.680712	4	764.175293	-2.113824221	-0.001613739	105.2418086
																			VDIVAINDPFIDLNYMVYMFQYDSTHGK	4	7669492	2xTMT6plex [N-Term; K28]; 2xOxidation [M16; M19			1.15	4.34694767	28.7	0	3798.879942	4	950.4760132	0.600480776	0.000570289	108.1676756
																			VIISAPSADAPMFVMGVNHEK	33	7669492	2xTMT6plex [N-Term; K21]; 1xOxidation [M	1.759	1.149	1.463	4.456591606	39.65	0	2687.4301	3	891.1491089	-0.902788081	-0.000803913	75.66697494
																			VIISAPSADAPMFVMGVNHEK	22	7669492	2xTMT6plex [N-Term; K21]	1.218	1.308	1.512	5.269669056	57.54	0	2671.435185	3	891.1491089	-0.902788081	-0.000803913	75.66697494
																			VVDLMAHMASK	35	7669492	2xTMT6plex [N-Term; K11]	1.183	1.259	1.555	4.064733982	59.52	0	1659.93264	3	553.9822998	-0.176824005	-9.78E-05	67.60917871
																			VIISAPSADAPMFVMGVNHEK	11	7669492	2xTMT6plex [N-Term; K21]; 2xOxidation [M12; M15			1.501	3.699855328	35.01	0	2703.425015	3	891.1491089	-0.902788081	-0.000803913	75.66697494
																			VVDLMAHMASK	30	7669492	2xTMT6plex [N-Term; K11]; 1xOxidation [M	1.169	1.193	1.25	3.435029507	55.61	0	1675.927555	3	553.9822998	-0.176824005	-9.78E-05	67.60917871
																			VVDLMAHMASKE	19	7669492	2xTMT6plex [N-Term; K11]	0.566	0.474	0.583	3.435506344	37.92	1	1788.975233	3	596.9969482	0.591839699	0.000352929	82.0679301
																			VVDLMAHMASK	8	7669492	2xTMT6plex [N-Term; K11]; 2xOxidation [M5; M8	0.846		0.785	3.236431837	26.57	0	1691.92247	3	553.9822998	-0.176824005	-9.78E-05	67.60917871
																			VVDLMAHMASKE	13	7669492	2xTMT6plex [N-Term; K11]; 1xOxidation [M	0.328	0.656	0.649	4.174422741	35.62	1	1804.970148	3	596.9969482	0.591839699	0.000352929	82.0679301
																			LVINGNPITIFQER	8	7669492	1xTMT6plex [N-Term]	6.063	1.653	0.978	2.234746695	27.98	0	1843.063899	2	922.0348511	-0.799381705	-0.000736656	97.20220245
																			VVDLMAHMASKE	2	7669492	2xTMT6plex [N-Term; K11]; 2xOxidation [M5; M8		0.584		2.581460476	28.15	1	1820.965063	3	596.9969482	0.591839699	0.000352929	82.0679301
																			TVDGPSGK	6	7669492	2xTMT6plex [N-Term; K8]	1.244	1.152	0.936	3.211820841	26.62	0	1218.709423	2	609.8587646	0.680758563	0.000414823	28.92506524
																			AAFNSGK	8	7669492	2xTMT6plex [N-Term; K7]	1.28	1.162	1.186	3.116848946	36.04	0	1152.677729	2	576.8423462	-0.271790787	-0.000156644	33.90631412
																			QASEGPLK	2	7669492	2xTMT6plex [N-Term; K8]	0.974			2.806685686	26.57	0	1287.767272	2	644.3878174	0.843246795	0.000542953	34.69895908
																			LISWYDNEFGYSNR	8	7669492	1xTMT6plex [N-Term]	0.768	0.772	100	1.615677238	8.03	0	1992.965307	2	996.9851074	-1.188538627	-0.001184358	82.36352133
																			LTGMAFR	3	7669492	1xTMT6plex [N-Term]	1.162		1.023	2.671463728	28.55	0	1024.581102	2	512.7949829	1.548652721	0.00079336	54.03152065
50845388	ANXA2	annexin A2 isoform 1	302	NP_001002858.1	73.38935574	1	29	29	681	357	40.386	8.37	0.69	0.75	0.65	0.7	Significant	0.0017																
																			RAEDGSVIDYELIDQDAR	38	50845388	1xTMT6plex [N-Term]	0.718	0.869	1.139	6.702888012	61.78	1	2294.146184	3	765.3880615	1.502594009	0.001149057	59.21459763
																			SYSPYDMLESIR	28	50845388	1xTMT6plex [N-Term]	0.726	0.715	0.727	4.104779243	46.55	0	1689.835538	2	845.4226685	1.49236065	0.001260922	74.86768324
																			DALNIETAIK	39	50845388	2xTMT6plex [N-Term; K10]	0.857	0.98	0.927	4.232298851	60.72	0	1545.925229	2	773.465332	-1.191427286	-0.000920929	57.3851186
																			DIISDTSGDFR	59	50845388	1xTMT6plex [N-Term]	0.731	0.764	0.696	4.683923721	70.64	0	1454.732454	2	727.8695068	-0.492591008	-0.000358294	49.69982446
																			TPAQYDASELK	23	50845388	2xTMT6plex [N-Term; K11]	0.758	0.719	0.737	4.608807564	59.18	0	1680.920872	2	840.9633789	-0.827485412	-0.000695469	42.15944575
																			GVDEVTIVNILTNR	84	50845388	1xTMT6plex [N-Term]	0.733	0.683	0.668	4.300327778	76.92	0	1772.011529	2	886.5099487	0.616221125	0.000545975	90.22120025
																			GLGTDEDSLIEIICSR	46	50845388	1xTMT6plex [N-Term]; 1xCarbamidomethyl [C14	0.685	0.76	0.616	5.389318943	72.9	0	2007.026587	2	1004.01709	0.157595072	0.000158149	95.98924365
																			AEDGSVIDYELIDQDAR	46	50845388	1xTMT6plex [N-Term]	0.707	0.832	0.877	4.99407196	69.56	0	2138.045073	3	713.3515625	-2.310026136	-0.001646313	68.85797991
																			GDLENAFLNLVQCIQNKPLYFADR	73	50845388	2xTMT6plex [N-Term; K17]; 1xCarbamidomethyl [C13	0.781	0.653	0.765	4.163011074	42.39	0	3296.750188	4	824.9422607	-0.902477009	-0.00074381	108.9314947
																			LSLEGDHSTPPSAYGSVK	16	50845388	2xTMT6plex [N-Term; K18]	0.787	0.735		5.293860912	71.71	0	2303.228347	2	1152.118164	0.30596836	0.000352357	54.64261757
																			TDLEKDIISDTSGDFR	8	50845388	2xTMT6plex [N-Term; K5]	0.745	1.124	0.658	4.189838886	21.15	1	2270.191627	3	757.4017334	-0.43166166	-0.000326652	83.1090766
																			SALSGHLETVILGLLK	14	50845388	2xTMT6plex [N-Term; K16]	0.658	0.674	1.114	4.256260395	40.83	0	2109.304747	3	703.7723389	-1.082545536	-0.000761139	107.5656431
																			EVKGDLENAFLNLVQCIQNKPLYFADR	2	50845388	3xTMT6plex [N-Term; K3; K20]; 1xCarbamidomethyl [C16	0.228			3.999427557	15.98	1	3882.11909	4	971.2819214	-3.409092513	-0.003308626	112.215488
																			TNQELQEINR	6	50845388	1xTMT6plex [N-Term]	0.961	0.914	1.029	4.258672714	54.99	0	1473.785886	2	737.3966675	0.116808649	8.61E-05	35.85574761
																			SYSPYDMLESIR	15	50845388	1xTMT6plex [N-Term]; 1xOxidation [M7]	0.959	0.869	0.623	2.496645212	30.72	0	1705.830453	2	845.4226685	1.49236065	0.001260922	74.86768324
																			QDIAFAYQR	15	50845388	1xTMT6plex [N-Term]	0.838	0.788	0.862	3.7742908	45.81	0	1340.716016	2	670.8617554	0.162795242	0.000109131	43.80429496
																			KELASALK	26	50845388	3xTMT6plex [N-Term; K1; K8]	0.92	0.746	0.776	4.544442177	38.11	1	1547.01354	2	774.0100098	-0.515204767	-0.000398514	41.64389592
																			KLMVALAK	6	50845388	3xTMT6plex [N-Term; K1; K8]	0.488	0.43	0.461	5.037604809	33.83	1	1561.047817	3	521.0210571	0.513191659	0.000267039	48.81777113
																			AYTNFDAER	24	50845388	1xTMT6plex [N-Term]	0.793	0.868	0.852	4.152356625	51.75	0	1315.647996	2	658.3277588	0.186302207	0.000122554	41.37094837
																			ALLYLCGGDD	4	50845388	1xTMT6plex [N-Term]; 1xCarbamidomethyl [C6			0.76	2.232932806	48.2	0	1325.660869	2	663.333313	-1.146359128	-0.000759842	77.78148283
																			DLYDAGVK	26	50845388	2xTMT6plex [N-Term; K8]	0.706	0.678	0.676	3.708744526	45.47	0	1338.766938	2	669.8870239	-0.124471581	-8.33E-05	43.61455018
																			WISIMTER	16	50845388	1xTMT6plex [N-Term]	0.915	1.079	0.918	3.182281733	31.98	0	1264.692109	2	632.8497925	0.157335478	9.95E-05	84.08927356
																			SLYYYIQQDTK	2	50845388	2xTMT6plex [N-Term; K11]		100		1.824229598	8.47	0	1880.020586	2	940.5112915	-2.808337433	-0.002639866	68.93964876
																			SVPHLQK	7	50845388	2xTMT6plex [N-Term; K7]	0.289	0.519	0.267	2.964157581	32.98	0	1266.793427	3	422.9360657	0.170747242	7.21E-05	43.64334894
																			ELASALK	17	50845388	2xTMT6plex [N-Term; K7]	0.857	0.981	0.825	3.242271423	39.22	0	1189.755645	2	595.3758545	-9.424301261	-0.005606308	46.48046109
																			LMVALAK	8	50845388	2xTMT6plex [N-Term; K7]	0.804	0.809		3.464666128	40.03	0	1203.789922	2	602.397583	-1.688554072	-0.001016332	64.59763181
																			DIISDTSGDFRK	2	50845388	2xTMT6plex [N-Term; K12]			0.47	3.15312171	14.92	1	1811.990349	3	604.6670532	-2.065232872	-0.001247394	48.61830342
																			KLMVALAK	4	50845388	3xTMT6plex [N-Term; K1; K8]; 1xOxidation [M3		0.558	0.592	2.511595249	22.1	1	1577.042732	3	521.0210571	0.513191659	0.000267039	48.81777113
																			SEVDMLK	17	50845388	2xTMT6plex [N-Term; K7]	0.937	1.008	0.98	3.591629505	42.37	0	1279.733195	2	640.3704834	0.386984472	0.000247618	47.80410041
																			KGTDVPK	2	50845388	3xTMT6plex [N-Term; K1; K7]	1.027			2.228706598	7.74	1	1431.913826	3	477.975769	-0.748698053	-0.000357357	29.83497189
																			TKGVDEVTIVNILTNR	1	50845388	2xTMT6plex [N-Term; K2]		0.01		1.381607294		1	2230.317102	3	744.1103516	-0.269388823	-0.000200274	77.82678105
																			WISIMTER	7	50845388	1xTMT6plex [N-Term]; 1xOxidation [M5]	0.872	0.626		1.656299353	23.5	0	1280.687024	2	632.8497925	0.157335478	9.95E-05	84.08927356
17986283	TUBA1A	tubulin alpha-1A chain isoform 1	7846	NP_006000.2	78.93569845	1	1	22	675	451	50.104	5.06	1.512	1.443	3.032	2	Non-Significan	0.062																
																			DVNAAIATIK	25	17986283	2xTMT6plex [N-Term; K10]	1.152	1.222	1.158	5.292680264	59.55	0	1473.9041	2	737.4550781	-0.827971101	-0.000610175	68.06345176
																			AYHEQLSVAEITNACFEPANQMVK	43	17986283	2xTMT6plex [N-Term; K24]; 1xCarbamidomethyl [C15	1.482	1.325	1.887	4.971693516	40.84	0	3208.617125	3	1070.209961	-0.559651535	-0.000598569	72.51123941
																			TIGGGDDSFNTFFSETGAGK	31	17986283	2xTMT6plex [N-Term; K20]	1.197	1.314	1.134	5.582830429	57.48	0	2466.218904	3	822.7440186	-0.568402884	-0.000467269	74.49892955

Bhat et al,2017. Cigarette smoke and chewing tobacco alter expression of different sets of miRNAs in oral keratinocytes

Supplementary Table S2(A). List of all proteins and peptides identified in OKF6/TERT1 cells chronically treated with chewing tobacco using Sequest and Mascot search algorithms

Accession	Gene_Symbol	Description	Gene_ID	Refseq_Accession	Coverage	Proteins	#Unique_Peptides	#Peptides	#PSMS	#AAs	MW [kDa]	calc. pI	OKF6-STE-6M/OKF6P_R1	OKF6-STE-6M/OKF6P_R2	OKF6-STE-6M/OKF6P_R3	Median fold change	Status	P-value (6M)	Sequence	#PSMSs	Protein Accessions	Modifications	OKF6-STE-6M/OKF6P_R1	OKF6-STE-6M/OKF6P_R2	OKF6-STE-6M/OKF6P_R3	Xcorr	Ionscore	# Missed Cleavages	Theo. MH+ [Da]	Charge	m_z	DeltaM [ppm]	Deltam/z [Da]	RT [min]
																			AVFVDLEPTVIDEVR	56	17986283	1xTMT6plex [N-Term]	0.919	1.154	1.122	3.982324839	52.47	0	1931.068709	2	966.0397339	1.803039644	0.001740897	80.82953655
																			ECISIHVGQAGVQIGNACWELYCLEHGIQPDGQMPSDK	7	17986283	2xTMT6plex [N-Term; K38]; 3xCarbamidomethyl [C2; C18; C23]; 1xOxidation [M34	100		1.394	5.606026649	30.81	0	4771.277722	4	1193.57251	-1.99373167	-0.002378162	90.33142578
																			ECISIHVGQAGVQIGNACWELYCLEHGIQPDGQMPSDK	28	17986283	2xTMT6plex [N-Term; K38]; 3xCarbamidomethyl [C2; C18; C23	3.149	100	1.189	6.228083134	25.12	0	4755.282807	4	1193.57251	-1.99373167	-0.002378162	90.33142578
																			NLDIERPTYTNLNR	12	17986283	1xTMT6plex [N-Term]	1.097	1.333	1.458	4.746809006	27.62	0	1948.044954	3	650.0187988	-1.596973221	-0.001036992	44.0613833
																			DYEEVGVDSVEGEGEEEGEEY	26	17986283	1xTMT6plex [N-Term]	1.544	1.564	100	3.681097746	50.02	0	2578.067778	3	860.02771	0.309798997	0.000266228	67.03670887
																			VGINYQPPTVVPGGDLAK	5	17986283	2xTMT6plex [N-Term; K18]	100	0.709		3.04886651	24.84	0	2283.311289	2	1142.158691	-0.517860839	-0.000591219	80.08766306
																			IHFPLATYAPVISAEK	4	17986283	2xTMT6plex [N-Term; K16]	1.276	1.48		3.60306406	31.69	0	2215.289097	3	739.1016235	0.551009559	0.000406882	81.69282156
																			AFVHWYVGEGMEEGEFSEAR	60	17986283	1xTMT6plex [N-Term]; 1xOxidation [M11]	1.014	1.094	1.093	4.193455219	50.07	0	2575.176104	3	859.0631714	-0.444066657	-0.000381183	77.35264765
																			AFVHWYVGEGMEEGEFSEAR	159	17986283	1xTMT6plex [N-Term]	1.068	1.065	1.124	4.800458431	48.34	0	2559.181189	3	859.0631714	-0.444066657	-0.000381183	77.35264765
																			FDGALNVDLTEFQTNLVPYPR	10	17986283	1xTMT6plex [N-Term]	1.646	1.619	1.724	4.742277622	74.42	0	2638.371433	3	880.1283569	-0.34697132	-0.000305146	104.4415982
																			QLFHPEQLITGK	37	17986283	2xTMT6plex [N-Term; K12]	1.192	1.224	1.303	4.304189682	46.16	0	1869.09984	2	935.0534058	-0.162980359	-0.000152313	66.83108121
																			LEFSIYPAPQVSTAVVEPYNSILTTHTTLEHSDCAFMVDNEAIYDICR	17	17986283	1xTMT6plex [N-Term]; 2xCarbamidomethyl [C34; C47]				2.73541522	5.3	0	5746.75929	4	1437.44397	-0.912067739	-0.001310358	106.1922846
																			AVCMLSNTTAIAEAWAR	39	17986283	1xTMT6plex [N-Term]; 1xCarbamidomethyl [C3	2.175	1.012	100	2.735594511	25.77	0	2094.067346	2	1047.537109	-0.192968961	-0.000202045	93.62566765
																			EDAANNYAR	14	17986283	1xTMT6plex [N-Term]	1.783	2.081	1.508	3.349599123	46.31	0	1252.611945	2	626.8093872	-0.35677924	-0.000223453	27.54228179
																			EIIDLVLDR	18	17986283	1xTMT6plex [N-Term]	0.86	0.711	0.717	3.620397091	51.22	0	1314.783033	2	657.8947144	-0.66982083	-0.000440335	84.04237698
																			AYHEQLSVAEITNACFEPANQMVK	7	17986283	2xTMT6plex [N-Term; K24]; 1xCarbamidomethyl [C15]; 1xOxidation [M22]	0.99		100	2.569916964	7.08	0	3224.61204	3	1070.209961	-0.559651535	-0.000598569	72.51123941
																			GHYTIGKEIIDLVLDR	6	17986283	2xTMT6plex [N-Term; K7]		5.781	4.657	3.340520859	13.1	1	2300.337838	4	575.838623	-2.249848876	-0.001293853	88.53045888
																			YMACCLLYR	16	17986283	1xTMT6plex [N-Term]; 2xCarbamidomethyl [C4; C5	0.875	0.696	0.789	3.333981514	29.42	0	1478.715564	2	747.8575439	-1.784967331	-0.001334005	48.25769338
																			TIQFVDWCPTGFK	8	17986283	2xTMT6plex [N-Term; K13]; 1xCarbamidomethyl [C8				2.098872662	13.82	0	2057.09304	2	1029.049072	-1.055833989	-0.001085974	97.68642525
																			YMACCLLYR	8	17986283	1xTMT6plex [N-Term]; 2xCarbamidomethyl [C4; C5]; 1xOxidation [M2	1.192	1.123	0.892	1.688249826	34.9	0	1494.710479	2	747.8575439	-1.784967331	-0.001334005	48.25769338
																			FDLMYAK	16	17986283	2xTMT6plex [N-Term; K7]	1.655	2.363	1.156	3.340296984	32.15	0	1345.759016	2	673.3817749	-2.037969117	-0.001371308	81.28086024
																			LSVDYGK	14	17986283	2xTMT6plex [N-Term; K7]	1.364	1.45	0.981	3.336693764	38.06	0	1239.73491	2	620.3706055	-0.786621795	-0.000487601	39.27987182
																			LSVDYGKK	2	17986283	3xTMT6plex [N-Term; K7; K8]		0.439		2.610091925	6.99	1	1596.992804	3	533.0023193	-0.250296799	-0.000133241	41.4246211
																			AVCMLSNTTAIAEAWAR	5	17986283	1xTMT6plex [N-Term]; 1xCarbamidomethyl [C3]; 1xOxidation [M4]		100	100	0.970247626	2.39	0	2110.062261	2	1047.537109	-0.192968961	-0.000202045	93.62566765
																			FDLMYAK	2	17986283	2xTMT6plex [N-Term; K7]; 1xOxidation [M4]	1.858			2.091995478	14.82	0	1361.753931	2	673.3817749	-2.037969117	-0.001371308	81.28086024
7669550	VCL	vinculin isoform meta-VCL	7414	NP_054706.1	46.64902998	1	45	45	218	1134	123.722	5.66	0.95	0.96	0.97	0.96	Significant	0.000036																
																			AVAGNISDPGLQK	6	7669550	2xTMT6plex [N-Term; K13]	1.08	1.024	0.989	4.304399967	74.86	0	1728.005605	2	864.5060425	-0.460912314	-0.00039823	44.50033334
																			AQQVSQGLDVLTAK	6	7669550	2xTMT6plex [N-Term; K14]	1.034	0.974	1.254	3.345640898	49.77	0	1916.121697	2	958.564209	-0.290029204	-0.000277866	60.06128872
																			DPSASPGDAGEQAIR	12	7669550	1xTMT6plex [N-Term]	1.091	1.116	1.169	4.705168724	67.73	0	1699.844857	2	850.425293	-0.910772823	-0.000774086	29.59748638
																			GILEYLTVAEVVETMEDLVTYTK	4	7669550	2xTMT6plex [N-Term; K23]		0.53	0.714	3.648426056	13.22	0	3074.662327	3	1025.557983	-0.95298097	-0.000976698	113.4669249
																			TIESILEPVAQQISHLVIMHEEGEVDGK	2	7669550	2xTMT6plex [N-Term; K28]	2.039			3.609074116	20.55	0	3559.908205	4	890.7314453	-1.194749908	-0.0010633	113.8044365
																			NPGNQAAYEHFETMK	2	7669550	2xTMT6plex [N-Term; K15]			0.686	3.711486101	19.12	0	2195.095559	3	732.3707886	1.026772	0.000751288	53.02053975
																			STVEGIQASVK	10	7669550	2xTMT6plex [N-Term; K11]	1.235	1.185	1.181	5.015704155	69.63	0	1576.931043	2	788.9689941	-0.210071805	-0.000165634	44.07387755
																			WIDNPTVDDR	10	7669550	1xTMT6plex [N-Term]	0.773	1.171	1.163	4.418270588	57.66	0	1459.737873	2	730.3736572	1.482747803	0.001082212	56.68206514
																			ALASQLQDSLK	12	7669550	2xTMT6plex [N-Term; K11]	0.913	0.88	0.834	3.413090467	43.37	0	1631.973242	2	816.4904785	0.268589728	0.000219166	55.9728639
																			SLGEISALTSK	4	7669550	2xTMT6plex [N-Term; K11]		0.567	100	3.01546526	46.42	0	1563.935794	2	782.4707031	-1.064193304	-0.000832165	60.65210296
																			ELTPQVVSAAR	2	7669550	1xTMT6plex [N-Term]			1.088	3.061346769	33.1	0	1399.810644	2	700.4091797	0.31311735	0.000219153	48.26500853
																			MALLMAEMSR	4	7669550	1xTMT6plex [N-Term]	100	0.953		3.495278358	52.42	0	1381.720315	2	691.3634644	-0.479546443	-0.0003313	91.07240291
																			SLLDASEEAIKK	6	7669550	3xTMT6plex [N-Term; K11; K12]	1.235	100	100	3.275039911	14.89	1	1991.199168	3	664.4047852	0.318420558	0.000211346	65.04328959
																			KIAELCDDPK	6	7669550	3xTMT6plex [N-Term; K1; K10]; 1xCarbamidomethyl [C6	0.961	0.962	0.945	3.289450407	25.7	1	1876.081696	3	626.0318604	-0.35616536	-0.000222732	49.24444076
																			AAAVGTANK	4	7669550	2xTMT6plex [N-Term; K9]		1.184	1.021	3.414218187	59.86	0	1260.767607	2	630.887146	-0.468950669	-0.000295619	32.59140803
																			MQEAMTQEVSDVFSDTTTPIK	3	7669550	2xTMT6plex [N-Term; K21]	0.945			2.668581009	22.33	0	2816.409818	3	939.4738159	-1.037894441	-0.000974379	93.92001213
																			QVATALQNLQTK	4	7669550	2xTMT6plex [N-Term; K12]	1.964			3.405950308	24.41	0	1773.063454	2	887.0363159	1.072271798	0.000950603	60.983207
																			AIPDLTAPVAAVQAAVSNLVR	3	7669550	1xTMT6plex [N-Term]			0.793	3.126219511	24.58	0	2305.344096	3	769.1177979	-2.279865865	-0.001751958	109.9348024
																			SLLDASEEAIK	6	7669550	2xTMT6plex [N-Term; K11]	100	100	1.34	2.856167793	36.88	0	1633.941273	2	817.4750977	1.007057308	0.000822736	63.44346343
																			ETVQTTEDQILK	6	7669550	2xTMT6plex [N-Term; K12]	1.015	0.842	0.599	2.859520197	33.99	0	1863.047529	2	932.0288696	1.574607071	0.001466784	39.68873288
																			TNLLQVCER	5	7669550	1xTMT6plex [N-Term]; 1xCarbamidomethyl [C7	1.582	1.011		3.431817532	30.6	0	1361.740851	2	681.3741455	0.120225243	8.19E-05	57.12787191
																			AASDELSK	6	7669550	2xTMT6plex [N-Term; K8]	0.968	1.054	1.079	3.52111578	44.26	0	1278.730552	2	639.8685913	-0.505464432	-0.000323176	34.31464433
																			MSAEINEIIR	4	7669550	1xTMT6plex [N-Term]		100		2.435802221	37.29	0	1404.771816	2	702.8892212	-0.462962867	-0.000325179	60.39535685
																			AANFENHSGK	4	7669550	2xTMT6plex [N-Term; K10]		1.032	0.541	3.533569336	45	0	1532.822161	3	511.612854	1.205300762	0.000615837	29.28973608
																			LLAVAATAPPDAPNREEVFDER	4	7669550	1xTMT6plex [N-Term]			1.573	1.709239006	9.48	1	2610.372496	3	870.7953491	-0.383652801	-0.000333826	68.43323383
																			GILSGTSDLLLTFDEAEVR	4	7669550	1xTMT6plex [N-Term]	100			1.93858254	7.38	0	2265.217558	2	1133.112183	-0.207311489	-0.000234803	114.1157326
																			MTGLVDEAIDTK	9	7669550	2xTMT6plex [N-Term; K12]	1.222		100	2.089921951	12.14	0	1750.966108	2	875.9854736	-1.391851255	-0.001218542	68.24817483
																			EAEAASIK	6	7669550	2xTMT6plex [N-Term; K8]	0.714	1.044	0.769	3.423427105	36.53	0	1276.751288	2	638.8780518	-1.927481412	-0.001230457	33.51804593
																			QILDEAGK	8	7669550	2xTMT6plex [N-Term; K8]	0.653	1.091	1.019	3.618619919	38.53	0	1331.793487	2	666.4007568	0.56321936	0.000375046	44.7848175
																			DEEFPEQK	2	7669550	2xTMT6plex [N-Term; K8]	0.747			3.655533791	37.91	0	1479.773145	2	740.3901978	-0.017889355	-1.32E-05	45.23578458
																			VAMANIQPQMLVAGATSIAR	6	7669550	1xTMT6plex [N-Term]		0.01		1.362744689	2.97	0	2271.251458	2	1136.126465	-2.555918007	-0.002902566	86.80448882
																			SFLDSGYR	4	7669550	1xTMT6plex [N-Term]	1.03	1.072		2.844666481	44.72	0	1173.610154	2	587.3087158	0.000980415	5.75E-07	53.72704868
																			IAELCDDPK	2	7669550	2xTMT6plex [N-Term; K9]; 1xCarbamidomethyl [C5			1.728	2.727039099	19.19	0	1518.823801	2	759.9157104	0.225989636	0.000171619	54.51940145
																			VMLVNSMNTVK	3	7669550	2xTMT6plex [N-Term; K11]		100	100	1.618401051	9.68	0	1693.974504	2	847.4909058	0.017971603	1.52E-05	55.77543031
																			ELLPVLISAMK	6	7669550	2xTMT6plex [N-Term; K11]		1.521	2.458	2.53421855	22.01	0	1672.048321	2	836.5284424	0.769598347	0.000643403	100.9113795
																			AAVHLEGK	6	7669550	2xTMT6plex [N-Term; K8]	0.916	0.978		2.252496004	29.11	0	1282.788342	3	428.2671509	-1.124646392	-0.000480894	38.1152763
																			TISPMVMDAK	6	7669550	2xTMT6plex [N-Term; K10]	100	1.419		2.760307312	25.93	0	1550.868642	2	775.9385986	0.82416756	0.000639088	56.94910818
																			ILGAVAK	3	7669550	2xTMT6plex [N-Term; K7]		0.374	1.212	2.461194992	28.4	0	1129.770901	2	565.3893433	0.450289025	0.000254362	57.1192155
																			EEVFDER	4	7669550	1xTMT6plex [N-Term]	1.02	1.002		2.905809164	40.45	0	1152.573434	2	576.7902222	-0.231017005	-0.000133132	45.58106185
																			GVGQAAIR	2	7669550	1xTMT6plex [N-Term]			0.72	2.580334902	15.21	0	1000.610094	2	500.8086853	-0.000394291	-1.97E-07	32.47310226
																			DMPPAFIK	2	7669550	2xTMT6plex [N-Term; K8]	0.697			3.26647234	29.76	0	1376.801215	2	688.9046631	0.60618909	0.000417301	68.99527583
																			VLQLTSWDEDAWASK	2	7669550	2xTMT6plex [N-Term; K15]				1.087937117		0	2207.174855	2	1104.089844	-1.107238964	-0.001221935	85.53128834
																			LVQAAQMLQSDPYSVPAR	1	7669550	1xTMT6plex [N-Term]				1.126908064	0.41	0	2203.174254	2	1102.088867	-1.722961765	-0.001897992	77.86411265
																			TDAGFTLR	4	7669550	1xTMT6plex [N-Term]		0.878	0.87	3.049556732	28.36	0	1109.615239	2	555.3078613	-6.122152535	-0.003396617	41.973123
																			VDQLTAQLADLAAR	3	7669550	1xTMT6plex [N-Term]				1.340437412	3.44	0	1713.969664	2	857.4887695	0.349091651	0.000299166	81.06897669
48762932	CCT8	T-complex protein 1 subunit theta isoform 1	10694	NP_006576.2	66.42335766	1	31	31	219	548	59.583	5.6	1.07	1.18	1.23	1.16	Significant	0.0017																
																			HEKEDGAISTIVLR	3	48762932	2xTMT6plex [N-Term; K3]	0.651	1.46		3.761505842	42.38	1	2026.16971	3	676.0604248	-1.47504719	-0.000996232	43.8322495
																			EDGAISTIVLR	6	48762932	1xTMT6plex [N-Term]	1.453	100	100	4.034470081	49.2	0	1402.81031	2	701.9085083	-0.406397382	-0.000285049	65.08950042
																			LVPGGGATEIELAK	13	48762932	2xTMT6plex [N-Term; K14]	1.565	0.994	1.361	3.70187974	40.84	0	1813.083521	2	907.0447388	-0.727937198	-0.000659905	67.38583096
																			GSTDNLMDDIER	10	48762932	1xTMT6plex [N-Term]	1.388		100	4.102160931	51.4	0	1594.758016	2	805.8803101	0.255853756	0.000206059	36.3352943
																			GSTDNLMDDIER	10	48762932	1xTMT6plex [N-Term]; 1xOxidation [M7]	1.469	1.142	1.134	2.87514782	76.1	0	1610.752931	2	805.8803101	0.255853756	0.000206059	36.3352943
																			LTPPVLEEMGHCDSVYLSEVGDTQVVVFK	4	48762932	2xTMT6plex [N-Term; K29]; 1xCarbamidomethyl [C12]; 1xOxidation [M9]		1.011	1.507	4.241734505	11.1	0	3722.906156	3	1236.30896	0.292778203	0.000361768	97.52807127
																			LTPPVLEEMGHCDSVYLSEVGDTQVVVFK	6	48762932	2xTMT6plex [N-Term; K29]; 1xCarbamidomethyl [C12	1.47	100	100	5.632815838	20.95	0	3706.911241	3	1236.30896	0.292778203	0.000361768	97.52807127
																			GEENLMDAQVK	4	48762932	2xTMT6plex [N-Term; K11]	0.989	1.108		4.394708633	60.57	0	1691.903842	2	846.4553223	-0.280127474	-0.000236974	46.21373016
																			VADMALHYANK	11	48762932	2xTMT6plex [N-Term; K11]	1.681	1.253	1.276	3.120463371	43.11	0	1690.935082	3	564.3162231	-0.571462746	-0.000322102	50.94229708
																			AIADTGANVVVTGGK	8	48762932	2xTMT6plex [N-Term; K15]	1.088	1.087		4.828023911	98.13	0	1831.068933	2	916.0361328	-2.154077833	-0.001972133	51.75874331
																			AHEILPNLVCCSAK	2	48762932	2xTMT6plex [N-Term; K14]; 2xCarbamidomethyl [C10; C11	1.163			3.66346097	39.29	0	2070.124023	3	690.7131958	0.488604708	0.000337157	59.69838463
																			KETEGDVTSVK	6	48762932	3xTMT6plex [N-Term; K1; K11]	0.974		1.276	3.344881296	31.12	1	1880.094369	3	627.3691406	-0.797920054	-0.000500055	31.20266403
																			ETEGDVTSVK	8	48762932	2xTMT6plex [N-Term; K10]	1.48	1.801	1.444	4.309683323	59.09	0	1522.836474	2	761.9208984	-1.282852777	-0.000976787	40.98156041
																			DMLEAGILDTYLGK	12	48762932	2xTMT6plex [N-Term; K14]				2.746302605	43.44	0	1997.102935	2	999.0559551	-1.152636506	-0.001150967	97.85591823
																			LATNAAVTVLR	10	48762932	1xTMT6plex [N-Term]		3.492	0.869	3.698898315	39.63	0	1357.836465	2	679.4210815	-1.162764517	-0.000789422	49.45446131
																			ILGSGISSSSVLHGMVFK	2	48762932	2xTMT6plex [N-Term; K18]	1.076			2.650549412	19.55	0	2277.304095	3	759.7721558	-0.957603707	-0.000726918	93.93918337
																			HFSGLEEAVYR	10	48762932	1xTMT6plex [N-Term]	1.282	1.191	1.303	4.46628046	44.3	0	1536.800808	2	768.9055786	1.999339502	0.001536293	49.76781346
																			LIAQACVSIFPDSGHFNVDNIR	4	48762932	1xTMT6plex [N-Term]; 1xCarbamidomethyl [C6	1.328			3.561545372	17.63	0	2702.392186	3	901.468689	-0.248622488	-0.000223958	90.34921344
																			DIDEVSSLLR	10	48762932	1xTMT6plex [N-Term]	1.535	1.045	1.48	3.816474438	44.85	0	1375.763026	2	688.3847656	-0.560343595	-0.00038545	71.67039359
																			VADMALHYANK	4	48762932	2xTMT6plex [N-Term; K11]; 1xOxidation [M4	0.994		1.013	3.218773842	33.6	0	1706.929997	3	564.3162231	-0.571462746	-0.000322102	50.94229708
																			AVDDGVNTFK	6	48762932	2xTMT6plex [N-Term; K10]	0.958		1.049	4.499492168	49.39	0	1523.846979	2	762.4282227	1.436950383	0.001094846	49.61634499
																			LFVTNDAATILR	9	48762932	1xTMT6plex [N-Term]	1.1	0.492	1.919	3.089211464	23.07	0	1562.910358	2	781.9595947	0.994550402	0.000777197	77.36443469
																			LYAVHQEGNK	8	48762932	2xTMT6plex [N-Term; K10]	1.023	1.16	1.161	3.391645908	41.25	0	1616.916062	2	808.9620972	0.529468386	0.000428053	38.53348643
																			FAEAFEAIPR	10	48762932	1xTMT6plex [N-Term]	1.73	0.01		3.184365273	40.01	0	1379.752067	2	690.3782959	-1.994440299	-0.001375917	75.25272851
																			KFAEAFEAIPR	4	48762932	2xTMT6plex [N-Term; K1]	1.229			3.086771011	31.19	1	1737.009962	3	579.675415	0.995905162	0.000576632	68.13414075
																			QYGNEVFLAK	4	48762932	2xTMT6plex [N-Term; K10]		100	100	2.780779123	31.9	0	1626.925564	2	813.9666748	0.313056348	0.00025466	62.44552807
																			IAVYSCPFDGMITETK	5	48762932	2xTMT6plex [N-Term; K16]; 1xCarbamidomethyl [C6	100	100		1.646538854	9.85	0	2290.186348	2	1145.595337	-1.288332663	-0.001475261	75.56577757
																			TAEELMNFSK	2	48762932	2xTMT6plex [N-Term; K10]			0.01	2.631101608	30.79	0	1627.876565	2	814.4414063	-0.631902948	-0.00051433	69.37808284
																			APGFAQMLK	6	48762932	2xTMT6plex [N-Term; K9]	1.123	1.257	0.891	3.660718679	38.89	0	1420.838663	2	710.921936	-1.455210744	-0.00103381	58.74860614
																			ELEVQHPAAK	4	48762932	2xTMT6plex [N-Term; K10]	1.333	1.354		3.194149971	37.87	0	1579.920813	3	527.3113403	-0.851241338	-0.000448298	37.74178442
																			QITSYGETCPGLEQYAIK	3	48762932	2xTMT6plex [N-Term; K18]; 1xCarbamidomethyl [C9				2.045330763	2.93	0	2516.310696	2	1258.657593	-1.107781773	-0.001393762	63.59451291
																			GEENLMDAQVK	2	48762932	2xTMT6plex [N-Term; K11]; 1xOxidation [M6	1.252			2.426491261	47.2	0	1707.898757	2	846.4553223	-0.280127474	-0.000236974	46.21373016
																			TVGATALPR	2	48762932	1xTMT6plex [N-Term]	0.594			3.300578833	24.93	0	1114.678174	2	557.8427734	0.086459933	4.82E-05	39.61771617
																			MVINHLEK	5	48762932	2xTMT6plex [N-Term; K8]	1.019	1.13		3.153907299	42.95	0	1441.860127	3	481.2911682	-0.81517708	-0.00039179	51.15355195
																			NVGLDIEAEVPAVK	4	48762932	2xTMT6plex [N-Term; K14]				1.492422342	0.33	0	1912.115549	2	956.5596924	-1.799543117	-0.001720467	69.978778
																			APGFAQMLK	2	48762932	2xTMT6plex [N-Term; K9]; 1xOxidation [M7]	0.969			1.715729475	14.8	0	1436.833578	2	710.921936	-1.455210744	-0.00103381	58.74860614
4507677	HSP90B1	endoplasmin precursor	7184	NP_003290.1	56.41344956	1	36	37	259	803	92.411	4.84	1.12	0.93	0.82	0.95	Significant	0.0083																
																			ELISNASDALDK	18	4507677	2xTMT6plex [N-Term; K12]	1.358	1.032	1.87	5.155765057	86.93	0	1733.968551	2	867.487793	-0.139115845	-0.000120611	70.01023764
																			NLLHVTDTGVGMTR	10	4507677	1xTMT6plex [N-Term]	1.045	0.996	0.812	3.799406052	44.81	0	1742.942069	3	581.6469727	-9.010317398	-0.00523482	47.72117581
																			EEKEESDDEAAVEEEEEEK	2	4507677	3xTMT6plex [N-Term; K3; K19]	1.867			4.231374264	11.19	1	2940.393642	3	980.8013306	-1.429570763	-0.001401167	51.42127942
																			EESDDEAAVEEEEEEKKPK	6	4507677	4xTMT6plex [N-Term; K16; K17; K19]	0.394	0.828	0.95	5.553197861	30.35	1	3136.619115	4	784.9086914	-1.969941129	-0.001544739	42.15622872
																			FQSSHHPTDITSLDQYVER	4	4507677	1xTMT6plex [N-Term]	0.987		100	4.397217274	35.12	0	2489.225831	3	830.4130859	-0.45269396	-0.000375619	56.93826275
																			VEEEPEEEPEETAEDTTEDTEQDEDEEMDVGTDEEEETAK	2	4507677	2xTMT6plex [N-Term; K40]				2.583204508	10.11	0	5075.090179	4	1269.528442	0.346888664	0.000440123	64.27552346
																			LISLTDENALSGNEELTVK	3	4507677	2xTMT6plex [N-Term; K19]			1.269	3.851982355	29.93	0	2504.385969	3	835.4664917	-0.418319044	-0.000349211	88.12947405
																			DISTNYYASQK	17	4507677	2xTMT6plex [N-Term; K11]	1.152	1.126	1.082	4.57028532	61.8	0	1747.926686	2	874.4668579	-0.141058369	-0.00012328	43.16131602
																			TETVEEPMEEEEAAK	4	4507677	2xTMT6plex [N-Term; K15]	100	1.358		3.587136269	53.22	0	2180.068066	2	1090.535767	-1.747338505	-0.001904658	48.48312938
																			VFITDDFHDMMPK	8	4507677	2xTMT6plex [N-Term; K13]	1.267	100		2.91541481	34.62	0	2054.049126	3	685.3540649	-0.722595075	-0.000494749	91.90012358
																			EFEPLLNWMK	8	4507677	2xTMT6plex [N-Term; K10]	1.196	1.173	1.258	3.852632999	35.01	0	1764.975885	2	882.9901733	-1.594701852	-0.001407305	104.0959677
																			EEASDYLELDTIK	8	4507677	2xTMT6plex [N-Term; K13]	0.824	0.931	100	3.145048618	42.89	0	1984.052674	2	992.5355225	5.591757729	0.005547171	72.29011117
																			VFITDDFHDMMPK	5	4507677	2xTMT6plex [N-Term; K13]; 1xOxidation [M	1.17			2.990813017	33.73	0	2070.044041	3	685.3540649	-0.722595075	-0.000494749	91.90012358
																			GLFDEYGSK	8	4507677	2xTMT6plex [N-Term; K9]	0.926	1.056	1.394	4.201300144	48.44	0	1473.798966	2	737.4035645	0.601212425	0.000443033	61.48373732
																			FAFQAEVNR	14	4507677	1xTMT6plex [N-Term]	0.879	1.147	1.166	3.578900099	47.5	0	1310.705451	2	655.8570557	1.055544649	0.000691754	53.46252031
																			LTESPCALVASQYGWSGNMER	4	4507677	1xTMT6plex [N-Term]; 1xCarbamidomethyl [C6	0.675	0.986		3.362704515	29.33	0	2585.232573	3	862.4155273	-0.210653678	-0.00018153	79.48840707
																			GYEVIYLTEPVDEYCIQALPEFDGK	10	4507677	2xTMT6plex [N-Term; K25]; 1xCarbamidomethyl [C15	100			2.665061474	8.41	0	3406.716882	3	1136.241699	-1.860320059	-0.002112528	103.0943126
																			LSLNIDPDAK	12	4507677	2xTMT6plex [N-Term; K10]	0.845	0.721	2.33	3.731123924	43.43	0	1543.909579	2	772.4580078	-0.544225524	-0.000420118	73.84404125
																			SGTSEFLNK	2	4507677	2xTMT6plex [N-Term; K9]		1.821		3.200783491	43.85	0	1440.809865	2	720.9081421	-0.595283481	-0.000428845	52.9425218
																			IYFMAGSSR	8	4507677	1xTMT6plex [N-Term]	1.117	1.242		3.2102139	34.5	0	1260.660809	2	630.833252	-1.254860735	-0.000790977	65.48881647
																			EAESSPFVER	4	4507677	1xTMT6plex [N-Term]	1.259			3.599528313	56.28	0	1379.700425	2	690.3583374	6.503509402	0.004486447	40.80806169
																			GTTITLVLK	16	4507677	2xTMT6plex [N-Term; K9]		0.01	1.015	2.788772345	36.17	0	1403.923773	2	702.4653931	-0.18756516	-0.000131664	59.65958718
																			TETVEEPMEEEEAAKEEKEESDDEAAVEEEEEK	2	4507677	4xTMT6plex [N-Term; K15; K18; K34]				2.282857895	7.62	2	4872.280935	4	1218.824341	-1.108546248	-0.001350287	58.07052459
																			LIINSLYK	8	4507677	2xTMT6plex [N-Term; K8]	1.293	1.709		3.805126429	49.4	0	1421.913208	2	711.4605103	0.376751415	0.000267854	93.25003378
																			KEAESSPFVER	4	4507677	2xTMT6plex [N-Term; K1]	1.043			4.158955097	33.61	1	1736.95832	2	868.9840698	1.463925995	0.001271389	37.21390497
																			SGYLLPDTK	2	4507677	2xTMT6plex [N-Term; K9]	1.389			3.629618883	30.41	0	1451.851002	2	726.4289551	-0.253651201	-0.000184132	60.31749226
																			LTESPCALVASQYGWSGNMER	2	4507677	1xTMT6plex [N-Term]; 1xCarbamidomethyl [C6]; 1xOxidation [M19]			100	2.527849197	10.67	0	2601.227488	3	862.4155273	-0.210653678	-0.00018153	79.48840707
																			IADDKYNDTFWK	4	4507677	3xTMT6plex [N-Term; K5; K12]	1.381	0.698		3.68222332	10.71	1	2203.200231	3	735.0721436	0.747382848	0.000548878	74.76091732
																			EEEAIQLDGLNASQIR	15	4507677	1xTMT6plex [N-Term]		100		2.11795783	26.41	0	2015.060664	2	1008.033325	-0.640173965	-0.000644995	59.75033171
																			EFEPLLNWMK	6	4507677	2xTMT6plex [N-Term; K10]; 1xOxidation [M9		100	100	1.78599894	27.66	0	1780.9708	2	882.9901733	-1.594701852	-0.001407305	104.0959677
																			EVEEDEYK	12	4507677	2xTMT6plex [N-Term; K8]	1.061	0.954	1.029	3.799975395	43.88	0	1498.767726	2	749.8870239	-0.636762069	-0.000477179	32.01385015
																			TDDEVVQREEEAIQLDGLNASQIR	4	4507677	1xTMT6plex [N-Term]				2.227982521	9.42	1	2957.501337	3	986.5048828	-0.41996008	-0.000414011	71.92792948
																			KYSQFINFPIYVWSSK	4	4507677	3xTMT6plex [N-Term; K1; K16]	100			2.383725643	2.13	1	2694.526256	3	898.8479004	1.073180069	0.000963904	100.7861228
																			KTLDMIK	8	4507677	3xTMT6plex [N-Term; K1; K7]	0.719	0.682	0.643	3.123090982	31.23	1	1535.979797	2	768.4931641	-0.485361202	-0.000372752	45.72014252
																			TVWDWELMNDIKPIWQRPSK	2	4507677	3xTMT6plex [N-Term; K12; K20]				1.538348198		0	3229.779921	4	808.1997681	-0.829417001	-0.000669709	89.41555883
																			EFGTNIK	4	4507677	2xTMT6plex [N-Term; K7]	1.1	1.027	1.016	2.931868315	33.73	0	1266.745809	2	633.8770142	0.744545832	0.000471575	49.80505496
																			IYFMAGSSR	4	4507677	1xTMT6plex [N-Term]; 1xOxidation [M4]	0.784	0.845		1.842231631	25.66	0	1276.655724	2	630.833252	-1.254860735	-0.000790977	65.48881647
																			TETVEEPMEEEEAAKEEK	1	4507677	3xTMT6plex [N-Term; K15; K18]				1.58553493		1	2795.411147	3	932.475647	0.443866002	0.000413596	37.85373809
																			IKEDEDDKTVLDLAVVLFETATLR	1	4507677	3xTMT6plex [N-Term; K2; K8]	0.547			3.121359348		2	3420.944463	4	855.9924927	1.075127984	0.000919488	116.9241005
																			KTFEINPR	2	4507677	2xTMT6plex [N-Term; K1]	1.149	0.946		2.990091085	15.06	1	1462.87822	3	488.2973022	-0.592080535	-0.000288714	46.79048918
																			LGVIEDHSNR	1	4507677	1xTMT6plex [N-Term]	100			1.190725565		0	1368.743293	3	456.919281	-0.002493106	-1.14E-06	40.33661338
4506787	IQGAP1	ras GTPase-activating-like protein IQGAP1	8826	NP_003861.1	34.88231744	1	44	44	240	1657	189.134	6.48	0.97	1.06	1.07	1.03	Significant	0.00095																
																			ALESGDVNTVWK	8	4506787	2xTMT6plex [N-Term; K12]	1.711	1.023	0.884	2.886906385	43.25	0	1776.98962	2	888.9978027	-0.726802923	-0.000645761	54.84397813
																			MFLGDNAHLSIINEYLSQSYQK	8	4506787	2xTMT6plex [N-Term; K22]	1.251			4.645471096	34.07	0	3029.580663	3	1010.532471	0.724743659	0.00073189	94.77630133
																			GVLLEIEDLQVNQFK	10	4506787	2xTMT6plex [N-Term; K15]	1.376			3.455339193	46.54	0	2203.273841	3	735.0974121	1.744026573	0.001280856	100.2104342
																			FDVPGDENAEMDAR	7	4506787	1xTMT6plex [N-Term]	0.893	1.488	1.411	3.170484781	41.47	0	1794.816594	2	897.9122314	0.330089786	0.000296225	57.52396618
																			LPYDVTPEQALAHEEVK	12	4506787	2xTMT6plex [N-Term; K17]	0.859	1.44	0.721	4.099783421	35.59	0	2397.306597	3	799.7741089	0.490684069	0.000392107	67.57422007
																			AQAHAENNEFITWNDIQACVDHVNLVVQEEHER	8	4506787	1xTMT6plex [N-Term]; 1xCarbamidomethyl [C19	1.623	100		8.029288292	32.27	0	4144.973268	5	829.8014526	1.179330548	0.000977659	91.58914295
																			SPDVGLYGVIPECGETYHSDLAEAK	7	4506787	2xTMT6plex [N-Term; K25]; 1xCarbamidomethyl [C13		100		2.640176773	24.68	0	3165.581451	3	1055.864868	-0.442249815	-0.000466659	72.20660916
																			ITLQDVVSHSK	4	4506787	2xTMT6plex [N-Term; K11]	1.048	1.098		3.570026398	41.58	0	1684.999791	3	562.338562	0.796210874	0.000447205	61.93902811
																			FVHLLDQSDQDFQEELDLMK	2	4506787	2xTMT6plex [N-Term; K20]; 1xOxidation [M19		1.243		3.001847029	23.86	0	2924.475195	3	975.4990234	2.503561156	0.002440534	93.2356363
																			FVHLLDQSDQDFQEELDLMK	4	4506787	2xTMT6plex [N-Term; K20]	1.077	100		3.579558372	23.43	0	2908.48028	3	975.4990234	2.503561156	0.002440534	93.2356363
																			EDSNLTLQEK	6	4506787	2xTMT6plex [N-Term; K10]	1.47	1.685	1.492	4.550677299	62.24	0	1634.900137	2	817.9468994	-8.327396622	-0.006807231	42.52307256
																			GLQQQNSDWYLK	6	4506787	2xTMT6plex [N-Term; K12]	1.38	100	0.702	3.072034359	35.7	0	1938.048532	2	969.5279541	0.051290318	4.97E-05	62.95785906
																			SVKEDSNLTLQEK	4	4506787	3xTMT6plex [N-Term; K3; K13]			0.509	4.768830299	17.34	1	2178.258474	3	726.7576904	0.020286418	1.47E-05	42.66613596
																			TCLDNLASK	4	4506787	2xTMT6plex [N-Term; K9]; 1xCarbamidomethyl [C2			1.275	4.041179657	42.96	0	1479.824135	2	740.4160767	0.500931357	0.000370645	53.59712977
																			AQEIQDESAVLWLDEIQGGIWQSNK	2	4506787	2xTMT6plex [N-Term; K25]				1.49487114	3.44	0	3315.726141	3	1105.913086	-0.43328895	-0.000478889	104.0336275
																			LTELGTVDPK	4	4506787	2xTMT6plex [N-Term; K10]	0.901	1.312		2.783489943	37.22	0	1530.91433	2	765.9605713	-0.303290567	-0.000232156	64.57544103
																			TLQALQIPAAK	2	4506787	2xTMT6plex [N-Term; K11]	0.703			2.906360388	21.26	0	1612.019798	2	806.5134277	-0.136196373	-0.000109776	73.19834186
																			EQLSDMMMINK	6	4506787	2xTMT6plex [N-Term; K11]	100			2.360080719	30.79	0	1797.931319	2	899.4703979	1.223805612	0.001100159	72.33423251
																			FPDAGEDELLK	2	4506787	2xTMT6plex [N-Term; K11]		1.011		3.322295904	33.19	0	1691.925623	2	846.4660034	-0.527767917	-0.000446472	74.55496551
																			EEIQSSISGVTAAYNR	6	4506787	1xTMT6plex [N-Term]	1.866	0.466	100	2.008323908	17.5	0	1954.0079	2	977.5075684	-0.020394621	-1.99E-05	61.69567777
																			TVLELMNPEAQLPQVYPFAADLYQK	2	4506787	2xTMT6plex [N-Term; K25]				1.933121324	7.63	0	3336.795407	3	1112.930664	-5.384783995	-0.005989307	105.751903
																			VDFTEEEINNMK	6	4506787	2xTMT6plex [N-Term; K12]	0.871	2.043		2.282871246	20.93	0	1926.9883	2	964.0060425	8.567094538	0.008254345	78.73815915
																			RIPADTFAALK	4	4506787	2xTMT6plex [N-Term; K11]		0.777	0.883	3.263445854	17.39	1	1661.015047	3	554.3430176	-0.329849887	-0.000182629	61.46657252
																			TALQEEIK	4	4506787	2xTMT6plex [N-Term; K8]	1.245	1.237		3.726406097	43.68	0	1389.835352	2	695.4222412	1.334015481	0.000927031	47.10600771
																			LTAEEMDER	6	4506787	1xTMT6plex [N-Term]; 1xOxidation [M6]	1.066	1.28	1.081	2.559819221	47.24	0	1338.640862	2	669.8237305	-0.506171983	-0.000338791	29.36988503
																			TDPVDIYK	8	4506787	2xTMT6plex [N-Term; K8]	1.066	1.303	1.17	3.239174604	42.12	0	1408.808803	2	704.907959	-0.114494777	-8.07E-05	55.15253091
																			TLINAEDPPMVVVR	6	4506787	1xTMT6plex [N-Term]	0.01			1.976730347	18.65	0	1782.998521	2	892.0018311	-1.197943015	-0.001067965	59.50298083
																			LIFQMPQNK	4	4506787	2xTMT6plex [N-Term; K9]		0.825	100	2.616730213	21.77	0	1576.928541	2	788.9660645	-2.338941591	-0.001844172	71.75187282
																			SWVNQMESQTGEASK	4	4506787	2xTMT6plex [N-Term; K15]				1.828712583	7.42	0	2140.074489	2	1070.541138	0.238305081	0.000254995	53.04428791
																			YMNPAIVAPDAFDIIDLSAGGQLTTDQR	2	4506787	1xTMT6plex [N-Term]				1.575582266	10.51	0	3221.634994	3	1074.547974	-1.746402548	-0.001875424	105.6620989
																			ALQSPALGLR	2	4506787	1xTMT6plex [N-Term]	1.111			3.438125372	29.74	0	1254.773137	2	627.8902588	0.082977649	5.21E-05	57.75949169
																			YQELINDIAR	5	4506787	1xTMT6plex [N-Term]	0.591			1.754380822	14.79	0	1463.805559	2	732.4055176	-1.230022484	-0.000900257	67.68845276
																			DTQEAQK	2	4506787	2xTMT6plex [N-Term; K7]			1.377	2.992747307	27.68	0	1277.710151	2	639.3587646	0.079373929	5.07E-05	24.93779815
																			LGLAPQIQDLYGK	4	4506787	2xTMT6plex [N-Term; K13]				1.494375467	3.51	0	1874.115155	2	937.5605469	-0.713984942	-0.000669045	74.29618171
																			YLDELMK	31	4506787	2xTMT6plex [N-Term; K7]	0.974	1.216		3.202519655	35.64	0	1369.780145	2	685.3930054	-1.030090717	-0.000705499	73.13622838
																			ATFYGEQVDYYK	2	4506787	2xTMT6plex [N-Term; K12]				1.448299408	1.21	0	1941.999851	2	971.5025635	-1.030034515	-0.001000163	59.05015088
																			LIVDVIR	16	4506787	1xTMT6plex [N-Term]	0.912	1.177		2.704909086	31.52	0	1056.697847	2	528.8525391	-0.042807885	-2.26E-05	84.28967392
																			ILAIGLINEALDEGDAQK	1	4506787	2xTMT6plex [N-Term; K18]				1.258401871		0	2341.337897	2	1171.170532	-1.75514046	-0.002054688	105.9477608
																			EQLWLANEGLITR	2	4506787	1xTMT6plex [N-Term]				1.06065762	0.28	0	1771.9904	2	886.4979858	-0.96191848	-0.000852255	74.66987803
																			NLGSIAK	4	4506787	2xTMT6plex [N-Term; K7]	0.605	0.603		2.726382732	22.05	0	1160.740329	2	580.873291	-0.882091131	-0.000511939	49.79657892
																			NPNAMLVNLEEPLASTYQDILYQAK	1	4506787	2xTMT6plex [N-Term; K25]				1.216823816		0	3293.749185	3	1098.586426	-1.354327849	-0.001486939	104.3582909
																			IPADTFAALK	2	4506787	2xTMT6plex [N-Term; K10]	0.917			2.251487732	33.88	0	1504.913936	2	752.9610596	0.602101292	0.000453055	72.96632155
																			MVVSFNR	4	4506787	1xTMT6plex [N-Term]		0.832	1.082	2.227573872	26.6	0	1081.602566	2	541.3051147	0.357527062	0.000193351	53.16625306
																			QILAPVVK	4	4506787	2xTMT6plex [N-Term; K8]				1.289120793	15.37	0	1325.892079	2	663.4505615	1.333115193	0.000883783	61.50877728
																			EEVITLIR	4	4506787	1xTMT6plex [N-Term]			100	1.105903983	16.29	0	1201.735354	2	601.3700562	-2.095856882	-0.001259333	58.08489032
7305053	MYOF	myoferlin isoform a	26509	NP_038479.1	27.89907812	1	47	47	190	2061	234.561	6.18	1.07	1.08	1.37	1.17	Significant	0.007																
																			TLHSTFQPNISQGK	4	7305053	2xTMT6plex [N-Term; K14]	0.64	0.658		5.202243805	50.6	0	2016.127845	3	672.713623	-0.758478377	-0.00050973	45.32992232
																			ANVTVLDTQIR	6	7305053	1xTMT6plex [N-Term]	1.072	1.086	1.144	4.085600853	56.43	0	1458.847758	2	729.9271851	-0.45563549	-0.000332351	51.81602134
																			IPAHQVLYSTSGENASGK	4	7305053	2xTMT6plex [N-Term; K18]	1.539	1.289		5.433380127	51.49	0	2317.25523	3	773.0906982	0.997398358	0.000770409	43.77925688
																			VIVESASNIPK	8	7305053	2xTMT6plex [N-Term; K11]	2.355	1.117		3.687942743	57.17	0	1614.983079	2	807.9953003	0.151986693	0.000122728	49.0339424
																			AMEELQDQEGWEYASLIGWK	4	7305053	2xTMT6plex [N-Term; K20]			1.89	3.786021233	30.82	0	2841.416952	3	947.8104248	-0.081236208	-7.69E-05	99.58443417
																			GKSDENEDPSVVGEFK	4	7305053	3xTMT6plex [N-Term; K2; K16]	0.999	0.928		4.290928364	28.65	1	2424.286145	3	808.7667847	-0.142011328	-0.000114759	57.83814829
																			KVDNELNPVWNEILEFDLR	6	7305053	2xTMT6plex [N-Term; K1]	0.918	1.538	100	5.426592827	32.93	1	2801.5238	3	934.5120239	-0.814486265	-0.000760601	104.3317212
																			DQLRPTQLLQNVAR	2	7305053	1xTMT6plex [N-Term]	1.452			3.979619265	18.71	0	1881.086759	3	627.7005615	0.197662501	0.00012394	59.0031556
																			FYASSGEHEK	2	7305053	2xTMT6plex [N-Term; K10]	1.152			4.338489056	39.04	0	1612.837143	3	538.2840576	0.295771687	0.000159011	43.47746202
																			GPVGTVSEAQLAR	6	7305053	1xTMT6plex [N-Term]	0.954	0.803		4.636398315	81.84	0	1513.853572	2	757.4295044	-1.215217226	-0.00091983	45.15310855
																			DLTQTASSTAR	14	7305053	1xTMT6plex [N-Term]	1.281	1.251	1.41	4.194411278	48.64	0	1379.732788	2	690.3688965	-1.646450146	-0.001135831	29.07831345
																			SLLTEADAGHTEFTDEVYQNESR	4	7305053	1xTMT6plex [N-Term]				3.576941967	39.23	0	2841.337626	3	947.7841797	0.126627859	0.000119931	57.63793788
																			DYSLDEFEANK	4	7305053	2xTMT6plex [N-Term; K11]	1.813	100		2.615587473	31.45	0	1788.905616	2	894.9569702	0.585765777	0.00052394	57.71462517
																			KLEPISNDDLLVVEK	4	7305053	3xTMT6plex [N-Term; K1; K15]		100	100	3.336464167	40.07	1	2399.436438	3	800.4885864	6.154731632	0.004922629	77.52953954
																			WLLLNDPEDTSSGSK	2	7305053	2xTMT6plex [N-Term; K15]	100			2.197116613	23.62	0	2120.12757	2	1060.566895	-0.498912195	-0.000528879	81.79180503
																			DHYIPNTLNPVFGR	4	7305053	1xTMT6plex [N-Term]	1.261	1.194		3.743557692	30.13	0	1871.996548	3	624.6704102	0.069256939	4.32E-05	67.52207072
																			ELPDSVPQECTVR	6	7305053	1xTMT6plex [N-Term]; 1xCarbamidomethyl [C10	1.377	1.344	1.492	3.30178833	22.63	0	1758.889365	2	879.9477539	-0.644712238	-0.000566989	43.19366568
																			ADCLMGEFK	6	7305053	2xTMT6plex [N-Term; K9]; 1xCarbamidomethyl [C3	1.521	0.851		3.416774035	38.16	0	1528.790392	2	764.8991089	0.358893829	0.000274337	67.94557658

Bhat et al,2017. Cigarette smoke and chewing tobacco alter expression of different sets of miRNAs in oral keratinocytes

Supplementary Table S2(A). List of all proteins and peptides identified in OKF6/TERT1 cells chronically treated with chewing tobacco using Sequest and Mascot search algorithms

Accession	Gene_Symbol	Description	Gene_ID	Refseq_Accession	Coverage	Proteins	#Unique_Peptides	#Peptides	#PSMS	#AAs	MW [kDa]	calc. pI	OKF6-STE-6M/OKF6P_R1	OKF6-STE-6M/OKF6P_R2	OKF6-STE-6M/OKF6P_R3	Median fold change	Status	P-value (6M)	Sequence	#PSMs	Protein Accessions	Modifications	OKF6-STE-6M/OKF6P_R1	OKF6-STE-6M/OKF6P_R2	OKF6-STE-6M/OKF6P_R3	Xcorr	Ionscore	# Missed Cleavages	Theo. MH+ [Da]	Charge	m_z	DeltaM [ppm]	Deltam/z [Da]	RT [min]
																			EELYMPPLVIK	14	7305053	2xTMT6plex [N-Term; K11]	2.068	100	100	2.202339411	31.92	0	1790.053801	2	895.5305786	0.04469506	4.00E-05	81.66723422
																			GWIPGNEENK	4	7305053	2xTMT6plex [N-Term; K10]	1.004	1.046		2.364681005	40.68	0	1601.868777	2	801.4371948	-1.038806416	-0.000832016	59.20748742
																			LIDEVIEDTR	8	7305053	1xTMT6plex [N-Term]	0.868	0.659	100	2.286573648	39.04	0	1431.78924	2	716.3980103	-0.346672662	-0.000248181	73.96086699
																			LISLLNEK	4	7305053	2xTMT6plex [N-Term; K8]	2.122	1.775		3.421505213	43.17	0	1387.892473	2	694.4492798	-0.857249182	-0.000594885	86.97356369
																			IDVGFVYDEPGHAVMR	3	7305053	1xTMT6plex [N-Term]				2.026507854	18.75	0	2034.031612	3	678.680542	-2.231845075	-0.001513214	60.863389
																			KVCTNIIEK	4	7305053	3xTMT6plex [N-Term; K1; K9]; 1xCarbamidomethyl [C3		0.933	1.205	2.820216417	14.08	1	1792.096952	3	598.0375977	0.718442576	0.000429173	42.18469501
																			AASPSELTCPPGWEWEDDAWSYDINR	4	7305053	1xTMT6plex [N-Term]; 1xCarbamidomethyl [C9				1.995233059	13.3	0	3281.468323	3	1094.491943	-2.147210085	-0.002348667	84.59024774
																			LDAVNTLLAMAER	2	7305053	1xTMT6plex [N-Term]	0.01			1.609660506	17.68	0	1645.914457	2	823.4598389	-1.249381835	-0.001028188	105.3085736
																			VCTNIIEK	2	7305053	2xTMT6plex [N-Term; K8]; 1xCarbamidomethyl [C2	1.064			3.401804686	31.91	0	1434.839057	2	717.9245605	1.942541037	0.001393617	56.9901598
																			LQMWVDVFPK	2	7305053	2xTMT6plex [N-Term; K10]				1.709647775	16.5	0	1720.986055	2	860.9962158	-0.523217124	-0.000450225	99.13334035
																			MAPSETHGAAAIFK	2	7305053	2xTMT6plex [N-Term; K14]				2.472628117	9.73	0	1889.035525	3	630.3503418	0.501541984	0.00031581	44.4360377
																			LQTNIEALK	3	7305053	2xTMT6plex [N-Term; K9]		100		2.39264822	24.23	0	1487.91975	2	744.4634399	-0.098645903	-7.34E-05	59.27651587
																			SLSQIHEAAVR	2	7305053	1xTMT6plex [N-Term]	0.497			3.698027849	12.05	0	1439.816792	3	480.6105347	0.179400537	8.61E-05	36.22271926
																			DIVIEMEDTKPLLASK	3	7305053	3xTMT6plex [N-Term; K10; K16]				1.897917151		0	2489.450374	3	830.4874268	-1.063124853	-0.000882199	99.77431985
																			VESVVIK	2	7305053	2xTMT6plex [N-Term; K7]	1.729			3.14648509	40.19	0	1231.802595	2	616.404541	-0.641132559	-0.000394874	56.48775829
																			SIFSPVVK	2	7305053	2xTMT6plex [N-Term; K8]		1.312		2.806775331	31.2	0	1334.844794	2	667.9260254	-0.015094451	-1.01E-05	70.752701
																			DVILDEK	4	7305053	2xTMT6plex [N-Term; K7]	1.025	1.082		3.108554125	38.51	0	1289.771689	2	645.3893433	-0.21631469	-0.000139498	56.18053906
																			GWWPCYAEK	4	7305053	2xTMT6plex [N-Term; K9]; 1xCarbamidomethyl [C5	100		0.01	1.60782373	20.88	0	1654.845205	2	827.927002	0.919751434	0.000761023	72.23848066
																			LEGALGADTTEDGDEK	2	7305053	2xTMT6plex [N-Term; K16]				1.473425388	0.3	0	2079.049379	2	1040.028076	-0.242257954	-0.000251833	46.39523554
																			FSDVTGK	3	7305053	2xTMT6plex [N-Term; K7]	1.36		1.29	2.547752142	36.52	0	1211.703609	2	606.3544922	-1.569397817	-0.000950822	40.83665674
																			EFFLPPK	6	7305053	2xTMT6plex [N-Term; K7]	0.834		100	1.94163394	18.71	0	1335.807681	2	668.4071655	-0.468724145	-0.000313063	68.57921339
																			LTIYDWDR	2	7305053	1xTMT6plex [N-Term]	0.996			2.235840559	18.79	0	1310.694218	2	655.8517456	1.523460553	0.000998395	71.64885313
																			EDIVPQLK	2	7305053	2xTMT6plex [N-Term; K8]	100			1.793339252	8.43	0	1399.856087	2	700.432373	0.987439897	0.000691137	58.73944148
																			NLVDPFVEVSFAGK	2	7305053	2xTMT6plex [N-Term; K14]		100		1.536323071		0	1980.120634	2	990.5614624	-2.518177542	-0.002493148	86.13212979
																			KEHFWSIDQTEFR	2	7305053	2xTMT6plex [N-Term; K1]			2.677	2.246479273	3.84	1	2181.149309	3	727.7211304	-0.215898433	-0.000156969	66.25074948
																			LDMIPDLK	3	7305053	2xTMT6plex [N-Term; K8]	100		100	1.462151408	7.48	0	1402.837994	2	701.9220581	-0.823144987	-0.00057737	74.55357138
																			MYELSCYLPQEK	1	7305053	2xTMT6plex [N-Term; K12]; 1xCarbamidomethyl [C6				1.220804453		0	2019.033142	2	1010.018738	-1.457496661	-0.001471367	77.34866219
																			LDLPNRPETSFLWFTNPCK	3	7305053	2xTMT6plex [N-Term; K19]; 1xCarbamidomethyl [C18				1.244946957		0	2793.479827	3	931.8297119	-1.877560654	-0.001748309	86.56314133
																			IPANQLAELWLK	5	7305053	2xTMT6plex [N-Term; K12]	100	100	100	1.561021447	3.24	0	1854.125326	2	927.5649414	-1.466905965	-0.001359914	91.67331273
38327039	HSPA4	heat shock 70 kDa protein 4	3308	NP_002145.3	35.47619048	1	36	38	178	840	94.271	5.19	1.27	1.23	1.33	1.27	Significant	0.00052																
																			GCALQCAILSPAFK	8	38327039	2xTMT6plex [N-Term; K14]; 2xCarbamidomethyl [C2; C6	1.368	0.881	0.535	3.439157248	48.29	0	1994.096745	2	997.550354	-1.661891378	-0.001656986	70.88954447
																			NKEDQYDHLDAADMTK	6	38327039	3xTMT6plex [N-Term; K2; K16]	1.149	1.27	1.294	4.907698154	25.25	1	2581.317128	3	861.1101074	-0.526291156	-0.000452841	52.22071058
																			AGGIETIANEYSDR	2	38327039	1xTMT6plex [N-Term]	2.034			3.733341932	47.52	0	1724.865259	2	862.937439	1.358210258	0.001171365	65.58021197
																			ELSTTLNADEAVTR	8	38327039	1xTMT6plex [N-Term]	1.484	1.549	1.048	2.9740417	52.66	0	1748.922773	2	874.9650269	0.002098971	1.84E-06	44.86564447
																			LEDTENWLYEDGEDQPK	8	38327039	2xTMT6plex [N-Term; K17]	1.455	100	1.428	2.995332003	33.47	0	2539.224049	3	847.0773315	-2.602263907	-0.002202577	74.75440344
																			AESEEMETSQAGSK	12	38327039	2xTMT6plex [N-Term; K14]	1.278	1.338	1.556	4.269440651	62.52	0	1941.947557	2	971.4762573	-1.194126769	-0.001159466	32.39037564
																			SNLAYDIVQLPTGLTGIK	8	38327039	2xTMT6plex [N-Term; K18]	1.598			3.52284956	56.74	0	2361.379368	3	787.7982788	0.387504775	0.000305015	97.95042144
																			QDLPALEEKPR	4	38327039	2xTMT6plex [N-Term; K9]		1.004	1.349	4.115469456	37.98	0	1754.021255	3	585.3452759	0.011132544	6.51E-06	49.13981068
																			KPVVDCVVSVPCFYTDAER	8	38327039	2xTMT6plex [N-Term; K1]; 2xCarbamidomethyl [C6; C12	0.931	1.304	1.466	4.546669483	27.75	0	2699.393715	3	900.4690552	-0.408451961	-0.000367524	75.06221664
																			AESEEMETSQAGSK	6	38327039	2xTMT6plex [N-Term; K14]; 1xOxidation [M6	0.874	100		2.959393024	50.46	0	1957.942472	2	971.4762573	-1.194126769	-0.001159466	32.39037564
																			SEENEEPMETDQNAKEEEK	2	38327039	3xTMT6plex [N-Term; K15; K19]		2.061		4.452692986	25.76	1	2953.418751	3	985.1434326	-1.018053548	-0.001002246	39.53093684
																			QSLTMDPVVK	4	38327039	2xTMT6plex [N-Term; K10]	1.188	1.341		4.034706116	54.84	0	1575.918035	2	788.4621582	-0.631805543	-0.000497837	60.07388846
																			EDIYAVEIVGGATR	8	38327039	1xTMT6plex [N-Term]	100	100	1.102	3.098920822	32.43	0	1721.927131	2	861.4675903	0.449179323	0.000386727	71.07993161
																			NAVEEYVYEMR	6	38327039	1xTMT6plex [N-Term]	4.625	100	100	3.09942317	47.86	0	1631.793674	2	816.4024048	2.365054097	0.00192964	69.86227924
																			KFDEVLVNHFCEEFGK	5	38327039	3xTMT6plex [N-Term; K1; K16]; 1xCarbamidomethyl [C11	1.763	1.196		3.881936312	21.96	1	2685.43137	4	672.1135864	0.426751969	0.000286503	90.77942421
																			LKETAESVLK	2	38327039	3xTMT6plex [N-Term; K2; K10]	0.968			2.453806162	16.62	1	1805.135111	3	602.3834229	0.334503874	0.000201275	63.52352996
																			STNEAMEWMNNK	6	38327039	2xTMT6plex [N-Term; K12]				2.516175508	22.98	0	1912.929739	2	956.9671021	-1.469729065	-0.001405744	58.80050129
																			SVMDATQIAGLNCLR	3	38327039	1xTMT6plex [N-Term]; 1xCarbamidomethyl [C13			100	1.890979886	24.11	0	1877.977469	2	939.4915161	-0.912179973	-0.000856527	67.23269702
																			WNSPAEEGSSDCEVFSK	5	38327039	2xTMT6plex [N-Term; K17]; 1xCarbamidomethyl [C12	2.273			3.113887072	29.59	0	2387.122561	2	1194.064575	-0.288041086	-0.000343795	62.89373943
																			NFTTEQVTAMLLSK	4	38327039	2xTMT6plex [N-Term; K14]				1.857868314	13.74	0	2041.140384	2	1021.073303	-0.51626272	-0.000526882	88.55628391
																			KEDIYAVEIVGGATR	2	38327039	2xTMT6plex [N-Term; K1]	1.269			3.170655489	14.25	1	2079.185026	3	693.7336426	0.648780343	0.000449645	66.58961431
																			FQESEERPK	4	38327039	2xTMT6plex [N-Term; K9]			1.309	2.892039776	16.27	0	1607.879342	3	536.6314087	0.205926036	0.000110368	35.36011417
																			VLATAFDTTLGGR	7	38327039	1xTMT6plex [N-Term]	1.243	100	100	2.768115997	19.28	0	1550.873973	2	775.9411621	0.692950406	0.000537339	76.68208385
																			LMNETTAVALAYGIYK	2	38327039	2xTMT6plex [N-Term; K16]				1.830987811	10.84	0	2216.240098	2	1108.621094	-2.340315025	-0.00259335	101.150695
																			LMSANASDLPLSIECFMNDVDVSGTMNR	4	38327039	1xTMT6plex [N-Term]; 1xCarbamidomethyl [C15				2.131260872	4.4	0	3316.551935	3	1106.189819	0.895568009	0.000990066	106.0187137
																			VTYMEEER	4	38327039	1xTMT6plex [N-Term]; 1xOxidation [M4]	1.309	1.208		1.865600586	28.93	0	1301.624484	2	651.3153076	-0.879666631	-0.000572498	34.38448663
																			SQVISNAK	2	38327039	2xTMT6plex [N-Term; K8]	1.119			3.717900753	54.16	0	1304.793821	2	652.9014893	1.441274165	0.000940283	34.99853241
																			LKKEDIYAVEIVGGATR	2	38327039	3xTMT6plex [N-Term; K2; K3]	0.564			2.929366112	7.39	2	2549.526984	4	638.1386719	2.303635159	0.001468295	66.57429612
																			LFEELGK	8	38327039	2xTMT6plex [N-Term; K7]	0.764	1.029	100	2.823832273	27.09	0	1293.78186	2	647.394043	-0.811869862	-0.000525191	70.5889684
																			ETAESVLK	2	38327039	2xTMT6plex [N-Term; K8]	0.725			2.1257658	8.81	0	1334.793153	2	667.9003296	0.172288633	0.000114985	47.2790578
																			AFSDPFVEAEK	5	38327039	2xTMT6plex [N-Term; K11]		100	100	1.639718294	6.28	0	1697.915058	2	849.4596558	-1.780770214	-0.001511798	67.61691781
																			SVLEQTK	6	38327039	2xTMT6plex [N-Term; K7]	1.207	1.314		3.108996153	34.46	0	1262.772023	2	631.8889771	-1.065741411	-0.000672894	42.99824616
																			LSGEYEK	4	38327039	2xTMT6plex [N-Term; K7]	1.816	1.05		2.945763111	27.08	0	1283.724739	2	642.3657227	-0.444041844	-0.000285014	39.05358242
																			NTVQGFK	2	38327039	2xTMT6plex [N-Term; K7]		1.307		2.967567205	39.55	0	1251.746143	2	626.3774414	1.168985028	0.000731636	35.81626276
																			MDQPPQAK	2	38327039	2xTMT6plex [N-Term; K8]	1.531			3.155595064	29.83	0	1372.765892	2	686.8859863	-0.871229215	-0.000597997	36.22875161
																			KEPFTLEAYYSSPQDLPYPDPAIAQFSVQK	1	38327039	3xTMT6plex [N-Term; K1; K30]				1.269161105		1	4117.17771	4	1030.047607	-2.212734825	-0.002277556	88.11589592
																			LSQECEK	1	38327039	2xTMT6plex [N-Term; K7]; 1xCarbamidomethyl [C5	1.554			3.000503063	32.36	0	1351.729172	2	676.3688354	0.903900324	0.000610914	33.95903206
																			EFSITDVVPYPISLR	3	38327039	1xTMT6plex [N-Term]		0.01		1.320003629		0	1965.089445	2	983.0480957	-0.269725	-0.000265017	94.81885499
																			SKEIEAK	2	38327039	3xTMT6plex [N-Term; K2; K7]		0.813		2.360350847	3.3	1	1491.934955	3	497.9827881	-0.766965197	-0.000381421	34.99272711
24234688	HSPA9	stress-70 protein, mitochondrial precursor	3313	NP_004125.3	55.16642121	1	32	32	218	679	73.635	6.16	0.82	0.72	0.79	0.78	Significant	0.0015																
																			EQQIVIQSSGGLSK	6	24234688	2xTMT6plex [N-Term; K14]	100	0.949	0.997	4.235349178	59.94	0	1932.116612	2	966.562561	0.638558928	0.000616885	50.43292606
																			DAGQISGLNVLR	7	24234688	1xTMT6plex [N-Term]	0.846	1.141	0.802	4.382258415	67.42	0	1471.843007	2	736.4249268	-0.29233714	-0.000215137	53.15081474
																			TTPSVVAFTADGER	6	24234688	1xTMT6plex [N-Term]	0.906		0.755	3.894447088	49.4	0	1679.88018	2	840.4433594	-0.439483725	-0.00036914	54.05296892
																			VEAVNMAEGIIHDTETK	8	24234688	2xTMT6plex [N-Term; K17]	0.908	1.06		3.646619797	64.58	0	2315.231718	3	772.4153442	-0.102860182	-7.94E-05	100.9286884
																			AQFEGIVTDLIR	13	24234688	1xTMT6plex [N-Term]	0.715	0.655	0.863	4.390210629	53.33	0	1590.905273	2	795.9559937	-0.35348133	-0.000281178	107.1034645
																			GAVVGIDLGTTNSCVAVMEGK	12	24234688	2xTMT6plex [N-Term; K21]; 1xCarbamidomethyl [C14	1.098			3.759066582	53.88	0	2536.351515	3	846.1206665	-1.604323477	-0.001356376	76.96830532
																			VINEPTAAALAYGLDK	10	24234688	2xTMT6plex [N-Term; K16]	0.985	0.906	2.884	4.581740856	50.62	0	2104.205427	3	702.0726929	-0.90356041	-0.000633759	80.26934694
																			QAVTNPNNTFYATK	6	24234688	2xTMT6plex [N-Term; K14]	0.766	0.837	0.846	4.156654358	45.66	0	2027.096211	2	1014.050842	-0.889271895	-0.00090132	49.01953834
																			QAASSLQQASLK	6	24234688	2xTMT6plex [N-Term; K12]	0.817	0.811	0.723	5.888086319	61.78	0	1689.989955	2	845.4976807	-1.106534314	-0.000935016	44.04119506
																			ETAENYLGHTAK	4	24234688	2xTMT6plex [N-Term; K12]	1.264	1.274		3.979409456	58.41	0	1791.964134	3	597.9923096	-0.981280277	-0.00058614	42.58191939
																			VIAVYDLGGGTFDISILEIQK	6	24234688	2xTMT6plex [N-Term; K21]		0.979	1.044	3.633725405	39.8	0	2709.54789	3	903.8514404	-2.997478276	-0.00270727	109.1462771
																			SDIGEVILVGGMTR	8	24234688	1xTMT6plex [N-Term]	2.095	1.518		3.819586277	40.15	0	1675.925022	2	838.4650269	-1.339593975	-0.00112253	74.17271451
																			MKETAENYLGHTAK	10	24234688	3xTMT6plex [N-Term; K2; K14]	0.579	0.644	0.556	5.364594936	26.57	1	2280.262513	3	760.7596436	0.817451522	0.000621335	47.29316532
																			EQQIVIQSSGGLSKDDIENMVK	6	24234688	3xTMT6plex [N-Term; K14; K22]				2.217667341	20.08	1	3105.706875	3	1035.90625	-0.862460659	-0.00089285	72.17982983
																			DQLPADECNK	4	24234688	2xTMT6plex [N-Term; K10]; 1xCarbamidomethyl [C8	1.13	0.992		3.176215887	48.79	0	1647.841242	2	824.4233398	-1.115922125	-0.000919431	40.55021201
																			VQQTVQDLFGR	6	24234688	1xTMT6plex [N-Term]	0.712	0.91	2.766	3.147859335	43.17	0	1519.843007	2	760.4244385	-0.925646181	-0.000703418	68.66192357
																			AMQDAEVSK	12	24234688	2xTMT6plex [N-Term; K9]	0.771	0.537	0.723	4.001371861	59.08	0	1436.781936	2	718.8988037	5.842815576	0.004197426	40.82649311
																			KSQVFSTAADGQTQVEIK	2	24234688	3xTMT6plex [N-Term; K1; K18]				2.72744751	9.25	1	2624.486242	3	875.4997559	-0.581988905	-0.000509141	57.57009028
																			MEEFKDQLPADECNK	6	24234688	3xTMT6plex [N-Term; K5; K15]; 1xCarbamidomethyl [C13	0.39	100	0.328	3.00362587	14.9	1	2541.293221	3	853.1005859	-0.364365472	-0.000310596	43.13745584
																			VLENAEGAR	6	24234688	1xTMT6plex [N-Term]	0.872	1.03		3.452454329	55.9	0	1187.658167	2	594.3325195	-0.340339932	-0.000202104	36.04516661
																			MKETAENYLGHTAK	2	24234688	3xTMT6plex [N-Term; K2; K14]; 1xOxidation [M1		0.587		2.658175468	21.25	1	2296.257428	3	760.7596436	0.817451522	0.000621335	47.29316532
																			ETGVDLTK	6	24234688	2xTMT6plex [N-Term; K8]		0.801	0.907	3.316833019	47.52	0	1320.777503	2	660.8925171	0.193090575	0.000127515	44.54779686
																			RYDDPEVQK	4	24234688	2xTMT6plex [N-Term; K9]		0.783	0.812	3.690367699	20.13	1	1607.879342	3	536.6308594	-0.818994912	-0.000438948	31.39883568
																			AMQDAEVSK	17	24234688	2xTMT6plex [N-Term; K9]; 1xOxidation [M2]	0.674	0.732	0.652	2.803171873	39.28	0	1452.776851	2	718.8988037	5.842815576	0.004197426	40.82649311
																			LYSPSQIGAFVLMK	4	24234688	2xTMT6plex [N-Term; K14]	0.506	100		1.727220893	14.69	0	2012.165476	2	1006.586304	-0.072269466	-7.27E-05	92.97333733
																			DDIENMVK	6	24234688	2xTMT6plex [N-Term; K8]	0.974	0.976	1.062	3.724521637	47.18	0	1421.771037	2	711.3890381	-0.166952427	-0.000118684	54.23243871
																			LLGQFTLIGIPPAPR	4	24234688	1xTMT6plex [N-Term]		100		1.782860041	18.85	0	1822.115206	2	911.5600586	-1.298283715	-0.001182811	101.5546672
																			VINEPTAAALAYGLDKSEDK	2	24234688	3xTMT6plex [N-Term; K16; K20]		0.01		1.516729355	3.13	1	2792.564886	3	931.5244141	-2.21909705	-0.002065657	82.38761764
																			LFEMAYK	8	24234688	2xTMT6plex [N-Term; K7]	1.04		1.791	3.201892614	33.61	0	1359.774666	2	680.3898315	-1.676302787	-0.001139697	71.42891991
																			YDDPEVQK	2	24234688	2xTMT6plex [N-Term; K8]	1.143			3.440557003	39.71	0	1451.778231	2	726.3939209	1.607970712	0.001167208	39.49854319
																			CELSSSVQTDINLPYLTMDSSGPK	1	24234688	2xTMT6plex [N-Term; K24]; 1xCarbamidomethyl [C1				2.356779099		0	3100.558273	3	1034.189819	-1.086317976	-0.001122731	87.39177945
																			EMAGDNK	4	24234688	2xTMT6plex [N-Term; K7]	0.971		0.719	2.472627401	26.49	0	1222.650194	2	611.8286743	-0.099453783	-6.08E-05	33.34543231
																			NAVITVPAYFNDSQR	1	24234688	1xTMT6plex [N-Term]			100	1.243946075		0	1924.012591	2	962.508606	-1.380550184	-0.001328098	69.87040572
																			DDIENMVK	6	24234688	2xTMT6plex [N-Term; K8]; 1xOxidation [M6]	0.969	0.948	1.084	2.317454576	30.15	0	1437.765952	2	711.3890381	-0.166952427	-0.000118684	54.23243871
																			MEEFKDQLPADECNK	2	24234688	3xTMT6plex [N-Term; K5; K15]; 1xCarbamidomethyl [C13]; 1xOxidation [M1]			0.924	3.446265936	17.08	1	2557.288136	3	853.1005859	-0.364365472	-0.000310596	43.13745584
																			LKEEISK	5	24234688	3xTMT6plex [N-Term; K2; K7]	0.744	0.841	0.867	2.827866554	32.77	1	1533.981905	2	767.4938965	-0.905552566	-0.000694551	41.19824649
23510338	UBA1	ubiquitin-like modifier-activating enzyme 1	7317	NP_003325.2	41.11531191	1	31	31	199	1058	117.774	5.76	1.03	1.12	1.16	1.1	Significant	0.0012																
																			AVTLHDQGTAQWADLSSQFYLR	12	23510338	1xTMT6plex [N-Term]	1.338	0.982	1.052	5.119872093	41.16	0	2736.394294	3	912.8045044	1.705267258	0.001555428	80.72828848
																			QLYVLGHEAMK	8	23510338	2xTMT6plex [N-Term; K11]	1.041	1.211	1.09	4.224679947	39.71	0	1746.997683	3	583.0045776	0.856858696	0.000498977	50.44996591
																			GNVQVVIPFLTESYSSSQDPPEK	10	23510338	2xTMT6plex [N-Term; K23]	0.607	100	0.718	2.87083602	18.49	0	2979.571538	3	993.8605347	-1.506081004	-0.001495825	90.7630482
																			SLVASLAEPDFVVTDFAK	12	23510338	2xTMT6plex [N-Term; K18]	1.25	0.901	1.403	4.485931396	43.21	0	2367.321185	3	789.7784424	-0.173466771	-0.000136884	97.34598322
																			KPLLESGTLGTK	6	23510338	3xTMT6plex [N-Term; K1; K12]	0.864		0.773	5.342720032	50.67	0	1931.214424	3	644.4101563	0.772115194	0.00049704	47.43152588
																			IHVSDQELQSANASVDDSR	12	23510338	1xTMT6plex [N-Term]	1.097	1.231	1.219	6.485594273	72.71	0	2300.131597	3	767.3817749	-0.358776415	-0.000275078	41.95098406
																			LQTSSVLVSGLR	6	23510338	1xTMT6plex [N-Term]	1.395	2.041		3.934159994	40	0	1488.894708	2	744.9501953	-1.070844695	-0.000797187	60.55643584
																			CPHPLTFDVNNPLHLDYVMAAANLFAQTYGLTGSQDR	3	23510338	1xTMT6plex [N-Term]; 1xCarbamidomethyl [C1			100	6.27495575	31.37	0	4376.142976	4	1094.791382	0.164810372	0.000180308	107.5524242
																			ALPAVQQNNLDEDLIR	9	23510338	1xTMT6plex [N-Term]	0.962	0.991		4.014568329	52.96	0	2038.113034	2	1019.560303	0.144809805	0.000147569	72.71321385
																			DNPGVVTCLDEAR	6	23510338	1xTMT6plex [N-Term]; 1xCarbamidomethyl [C8	1.246	1.122	1.149	5.470891476	88.21	0	1674.83185	2	837.9191895	-0.44663812	-0.000374022	42.90244591
																			QPAENVNQYLTDPK	4	23510338	2xTMT6plex [N-Term; K14]	1.056	1.424		3.058799505	37.29	0	2075.11734	2	1038.061523	-0.756417466	-0.000784827	60.45474572
																			YDGQVAVFGSDLQEK	6	23510338	2xTMT6plex [N-Term; K15]	100	100	100	2.95356679	28.91	0	2114.117006	2	1057.561401	-0.699784175	-0.000739713	63.70853469
																			HQYYNQEWTLWDR	2	23510338	1xTMT6plex [N-Term]			1.664	3.822940826	16.1	0	2067.987439	3	689.9996338	-1.49481218	-0.001030418	70.25421601
																			ALVLELCCNDESGEDVEVPYVR	3	23510338	1xTMT6plex [N-Term]; 2xCarbamidomethyl [C7; C8	0.01		1.186	2.380067825	21.82	0	2795.342912	3	932.4510498	-1.543873533	-0.001438552	119.177899
																			AENYDIPSADR	6	23510338	1xTMT6plex [N-Term]	1.071	1.276	1.206	3.771258593	50.72	0	1479.727703	2	740.3682251	0.994078377	0.000735483	44.41404479
																			IYDDDFFQNLDGVANALDNVDAR	12	23510338	1xTMT6plex [N-Term]		1.608	100	3.139589071	39.46	0	2829.352882	3	943.7852173	-4.164773721	-0.003927872	102.3466793
																			YFLVGAGAIGCELLK	9	23510338	2xTMT6plex [N-Term; K15]; 1xCarbamidomethyl [C11	100	100	100	2.421780348	18.74	0	2069.18694	2	1035.096069	-1.004340441	-0.001039084	102.8262754
																			LDQPMTEIVSR	4	23510338	1xTMT6plex [N-Term]		1.259	1.575	3.163196802	37.09	0	1517.819495	2	759.413147	-0.314401475	-0.000238602	52.55707531
																			QLYVLGHEAMK	4	23510338	2xTMT6plex [N-Term; K11]; 1xOxidation [M10			1.807	2.541741371	25.8	0	1762.992598	3	583.0045776	0.856858696	0.000498977	50.44996591
																			SDTAAAAVR	6	23510338	1xTMT6plex [N-Term]	100	0.73	1.206	2.736540794	32.78	0	1090.605403	2	545.8063354	-0.007850284	-4.28E-06	23.37326919
																			AAVATFLQSVQVPEFTPK	5	23510338	2xTMT6plex [N-Term; K18]			0.01	2.041661263	15.59	0	2391.368803	2	1196.184326	-3.10606471	-0.003713873	93.44335789
																			SIPICTLK	4	23510338	2xTMT6plex [N-Term; K8]; 1xCarbamidomethyl [C5	1.251		1.831	3.010159016	42.39	0	1389.853979	2	695.430481	-0.2113718	-0.000146888	64.50426178
																			ATLPSPDKLPGFK	6	23510338	3xTMT6plex [N-Term; K8; K13]	0.759	100		3.036666632	11.13	1	2058.256624	3	686.7560425	-1.481515259	-0.001016446	59.78849845
																			GGIVSQVK	4	23510338	2xTMT6plex [N-Term; K8]		0.495	0.685	2.863330841	44.1	0	1245.793093	2	623.3997803	-0.649524491	-0.000404587	39.87711361
																			LAGTQPLEVLEAVQR	6	23510338	1xTMT6plex [N-Term]	100	100		2.52307415	16.78	0	1853.069378	2	927.0374756	-0.919311573	-0.000851774	101.803793
																			GLGVEIAK	7	23510338	2xTMT6plex [N-Term; K8]	1.092	1.187	1.219	3.299099207	47.1	0	1244.797844	2	622.9025269	-0.053855381	-3.35E-05	49.93446669
																			DEFEGLFK	9	23510338	2xTMT6plex [N-Term; K8]	1.567	100	100	3.219070673	27.89	0	1442.793153	2	721.8994751	-1.025105147	-0.000739507	70.79426205
																			NIILGGVK	4	23510338	2xTMT6plex [N-Term; K8]	1.066	1.154		3.1983459	42.38	0	1271.845129	2	636.4257813	-0.662659298	-0.0004214	65.18746306
																			QFLFRPWDVTK	6	23510338	2xTMT6plex [N-Term; K11]	0.01	100		2.472614765	8.1	0	1895.09436	3	632.3688354	-1.270212394	-0.000802391	75.07032483
																			AEVSQPR	2	23510338	1xTMT6plex [N-Term]	0.856			2.181562901	22.79	0	1015.573374	2	508.2901611	-0.32380169	-0.000164422	26.39195847
																			MYPIDFEK	2	23510338	2xTMT6plex [N-Term; K8]	100	100		1.711523533	4.76	0	1500.817259	2	750.9110718	-1.593755201	-0.001195968	65.15410774
																			LVVADTR	4	23510338	1xTMT6plex [N-Term]	1.027	1.011		2.398713112	44.94	0	1002.614511	2	501.8106995	-0.387720519	-0.000194367	41.67404874
110611220	RRBP1	ribosome-binding protein 1	6238	NP_004578.2	35.00511771	1	33	33	195	977	108.589	5.54	0.99	1.03	1.01	1.01	Significant	0.00013																
																			SVEEEEQVWR	6	110611220	1xTMT6plex [N-Term]	1.354	1.019	1.362	4.083958626	67.32	0	1519.759003	2	760.3830566	-0.109273082	-8.30E-05	44.78778093
																			HMAAASAECQNYAK	8	110611220	2xTMT6plex [N-Term; K14]; 1xCarbamidomethyl [C9	1.073	1.029	1.143	4.750989914	49.92	0	2009.993736	3	670.6635132	-8.830967429	-0.00591673	32.91306978
																			HMAAASAECQNYAK	2	110611220	2xTMT6plex [N-Term; K14]; 1xCarbamidomethyl [C9]; 1xOxidation [M2]		1.131		4.395633698	46.81	0	2025.988651	3	670.6635132	-8.830967429	-0.00591673	32.91306978
																			AQEQQQQMAELHSK	2	110611220	2xTMT6plex [N-Term; K14]; 1xOxidation [M8	0.837			4.443924904	40.68	0	2130.101373	3	710.704895	-0.582522232	-0.00041361	18.27259994
																			EAEETQSTLQAECDQYR	14	110611220	1xTMT6plex [N-Term]; 1xCarbamidomethyl [C13	1.102	1.03	1.149	5.640337467	62.13	0	2287.034585	3	763.0170288	0.851795506	0.000649362	39.78630751
																			DAQDVQASQAEADQQQTR	14	110611220	1xTMT6plex [N-Term]	0.997	0.98	1.072	5.043939114	83.98	0	2218.053346	3	740.0233154	0.922723101	0.000682216	32.25013605
																			DALNQATSQVESK	2	110611220	2xTMT6plex [N-Term; K13]			1.051	4.983327866	54.46	0	1849.006727	2	925.0079346	1.008990718	0.000932815	52.46673663
																			ELCHTQSSHASLR	25	110611220	1xTMT6plex [N-Term]; 1xCarbamidomethyl [C3	1.017	1.064	1.255	5.597132683	55.33	0	1754.880532	4	439.4763184	1.65920554	0.000727927	21.25348299
																			EQEITAVQAR	10	110611220	1xTMT6plex [N-Term]	1.194	0.987	1.157	3.973699331	64.7	0	1373.758609	2	687.3831177	0.254674701	0.000174931	34.08514243
																			GELESSDQVR	4	110611220	1xTMT6plex [N-Term]	1.21	1.369		3.640528679	58.31	0	1348.690589	2	674.8482666	-0.987829889	-0.000666138	31.87769611
																			HPPAPAEPSSDLASK	3	110611220	2xTMT6plex [N-Term; K15]	0.922			4.731770039	40.31	0	1962.069661	3	654.6947021	-0.055199206	-3.61E-05	38.5421802
																			NTDVAQSPEAPK	2	110611220	2xTMT6plex [N-Term; K12]	1.021			3.412726164	36.5	0	1714.937585	2	857.9721069	-0.377589726	-0.000323771	36.65550589
																			LQSSEAEVR	6	110611220	1xTMT6plex [N-Term]	1.064	0.838	0.01	3.638931751	60.4	0	1247.679296	2	624.342041	-1.996152984	-0.001245279	34.35012164
																			AENSQLTER	4	110611220	1xTMT6plex [N-Term]		0.765		3.195619106	56.2	0	1276.66946	2	638.838623	0.39942504	0.000254967	22.81533446
																			AAILETAPK	8	110611220	2xTMT6plex [N-Term; K9]	0.895	0.961	0.883	3.963498592	56.79	0	1371.861173	2	686.434021	-0.296843311	-0.000203614	41.05011566
																			VGAAEEELQK	5	110611220	2xTMT6plex [N-Term; K10]	0.362	1.203	0.427	3.788734436	46.04	0	1531.873194	2	766.4396973	-0.702283162	-0.000537904	43.74752398
																			SIEALLEAGQAR	4	110611220	1xTMT6plex [N-Term]	100		0.01	2.279521704	21.12	0	1486.842673	2	743.9248657	-0.146602389	-0.000108987	72.9482915
																			LLATEQEDAAVAK	6	110611220	2xTMT6plex [N-Term; K13]	100	0.59		2.19023633	24.86	0	1817.04205	2	909.0253906	0.800652907	0.00072741	50.57638403
																			AGIIQDTWHK	4	110611220	2xTMT6plex [N-Term; K10]		0.857	0.942	3.267668486	24.87	0	1626.936797	3	542.9833374	-0.822473848	-0.000446038	47.061362
																			LQQENSILR	2	110611220	1xTMT6plex [N-Term]	0.981			3.515932322	48.92	0	1329.76878	2	665.3881836	0.233798165	0.000155449	49.93332138
																			LTAEFEEAQTSACR	6	110611220	1xTMT6plex [N-Term]; 1xCarbamidomethyl [C13		0.904	0.95	2.841357231	39.81	0	1841.890093	2	921.4490356	0.380733392	0.000350635	49.76499648
																			LKGELESSDQVR	6	110611220	2xTMT6plex [N-Term; K2]	1.01	1.131		2.921901226	16.31	1	1819.032548	3	607.0154419	-0.426224592	-0.000258439	48.64048196
																			GDPVAILK	8	110611220	2xTMT6plex [N-Term; K8]	0.912	1.14	0.593	3.086220741	40.43	0	1270.813494	2	635.9104004	0.023584303	1.50E-05	58.19556566
																			ESEEALQK	4	110611220	2xTMT6plex [N-Term; K8]	1.274	1.079		3.291964054	42.47	0	1391.778231	2	696.3916016	-1.655619372	-0.001152127	37.75975605
																			KLQEQLEK	8	110611220	3xTMT6plex [N-Term; K1; K8]	0.859	0.97		3.526350975	24.23	1	1703.067032	3	568.3613281	1.408720241	0.000799715	39.2032895
																			QSDELALVR	2	110611220	1xTMT6plex [N-Term]	1.038			3.175742149	28.12	0	1259.715681	2	630.3619995	0.826308232	0.000520457	45.83577329
																			AMEALATAEQACK	8	110611220	2xTMT6plex [N-Term; K13]; 1xCarbamidomethyl [C12				1.890148878	14.03	0	1851.970876	2	926.4871216	-2.110878734	-0.001954643	57.77636334
																			IPDHDPAPNVTVLLR	4	110611220	1xTMT6plex [N-Term]		1.023	0.976	3.053193331	19.84	0	1886.069712	3	629.3604126	-1.605416285	-0.001009309	58.31927228
																			EVQQLQGK	2	110611220	2xTMT6plex [N-Term; K8]	0.99			3.38378644	38.92	0	1387.830935	2	694.418396	-1.022968848	-0.000709854	40.65599258
																			HLEEIVEK	2	110611220	2xTMT6plex [N-Term; K8]			0.975	3.389798164	28.39	0	1454.861901	2	727.9353638	1.065454434	0.000775045	48.50783831
																			LIEILSEK	6	110611220	2xTMT6plex [N-Term; K8]	1.031	1.225	100	2.799176455	27.58	0	1402.892138	2	701.9488525	-1.218833457	-0.000854946	92.06728303
																			LQEQLEK	4	110611220	2xTMT6plex [N-Term; K7]	1.355	1.316		3.4210186	35.82	0	1345.809137	2	673.4072266	-1.456755602	-0.000980257	36.38631482
																			AAAGEAK	2	110611220	2xTMT6plex [N-Term; K7]			0.686	2.742493868	25.07	0	1075.65118	2	538.3293457	0.218310782	0.000117413	25.83896577
																			IQEEIEK	8	110611220	2xTMT6plex [N-Term; K7]	1.075			2.955672979	27.6	0	1346.793153	2	673.9011841	1.439682151	0.000969477	54.0188294
4505257	MSN	moesin	4478	NP_002435.1	26.63250232	1	26	39	320	577	67.778	6.4	0.81	0.76	0.76	0.78	Significant	0.00046																
																			AQMVQEDLEK	6	4505257	2xTMT6plex [N-Term; K10]	0.778	0.812	0.761	4.375065804	69.31	0	1648.898028	2	824.9450684	-9.198950445	-0.007584066	48.08222079
																			ILALCMGNHELYMR	8	4505257	1xTMT6plex [N-Term]; 1xCarbamidomethyl [C5	0.981	0.797		4.492577076	47.63	0	1949.996096	3	650.6697998	-0.640778687	-0.000416505	79.80310241
																			ALTSELANAR	14	4505257	1xTMT6plex [N-Term]	0.793	0.865	0.863	4.082233906	56.19	0	1274.726581	2	637.866333	-0.934415578	-0.000595562	35.80294707
																			FYPEDVSEELIQDITQR	12	4505257	1xTMT6plex [N-Term]	1.206	1.462	0.772	3.653496504	28.02	0	2311.165523	3	771.0582886	-2.254346256	-0.001736722	99.74499108
																			ESEAVEWQQK	6	4505257	2xTMT6plex [N-Term; K10]	1.191	0.913		4.249434948	61.37	0	1691.900471	2	846.453125	-0.885193914	-0.00074883	43.95486328
																			KAQQELEEQTR	10	4505257	2xTMT6plex [N-Term; K1]	0.735	0.788	0.825	3.865861177	43.54	1	1818.012147	2	909.5110474	1.469432736	0.001335723	33.41024853
																			KPDTIEVQQMK	9	4505257	3xTMT6plex [N-Term; K1; K11]	0.928	0.765	0.866	4.951767921	43.06	0	2004.176659	3	668.7305908	0.279696371	0.000186854	42.3317132
																			KAPDFVFYAPR	12	4505257	2xTMT6plex [N-Term; K1]	0.737	0.828	0.648	3.476797581	26.85	1	1769.015047	3	590.3424683	-1.24127539	-0.000731945	54.53779583
																			AQQELEEQTR	7	4505257	1xTMT6plex [N-Term]	0.972	0.854	0.931	3.850249052	46.45	0	1460.754252	2	730.8809204	0.213930791	0.00015625	34.4062764
																			IQVWHEEHR	13	4505257	1xTMT6plex [N-Term]	0.771	0.827	0.825	4.184273243	48.24	0	1462.775262	3	488.2633057	0.069526872	3.39E-05	35.99607146
																			KTANDMIHAENMR	2	4505257	2xTMT6plex [N-Term; K1]	0.734			3.16523695	31.72	1	1989.041021	4	498.0157776	0.130586328	6.49E-05	44.39787733
																			APDFVFYAPR	18	4505257	1xTMT6plex [N-Term]	0.79	0.794	0.433	3.595981359	31.71	0	1411.757152	2	706.3810425	-1.660391136	-0.001172035	65.18701339
																			EDAVLEYLK	22	4505257	2xTMT6plex [N-Term; K9]	0.932	2.043	1.354	3.169789076	47.45	0	1537.887781	2	769.4467163	-1.05676294	-0.000812591	76.05811569
																			RKPDTIEVQQMK	18	4505257	3xTMT6plex [N-Term; K2; K12]	0.874	0.775	0.851	4.093585968	16.43	1	2160.27777	4	540.8242188	-1.261175779	-0.000681122	39.6647313
																			VTTMDAELEFAIQPNTTGK	2	4505257	2xTMT6plex [N-Term; K19]	100			2.649563313	29.67	0	2524.336911	3	842.1146851	-2.935002133	-0.002469645	79.69920076
																			KESEAVEWQQK	2	4505257	3xTMT6plex [N-Term; K1; K11]			0.78	4.690810204	15.88	1	2049.158366	3	683.7237549	-0.807454216	-0.000551534	40.51982556
																			KPDTIEVQQMK	8	4505257	3xTMT6plex [N-Term; K1; K11]; 1xOxidation [M10			0.855	4.497064114	24.67	0	2020.171574	3	668.7305908	0.279696371	0.000186854	42.3317132
																			GSELWLGVDALGLNIYEQNDR	7	4505257	1xTMT6plex [N-Term]				2.147302389	8.59	0	2591.330296	3	864.447876	-0.471479963	-0.000407253	106.1621266
																			TQEQLALEMAELTAR	6	4505257	1xTMT6plex [N-Term]				1.73484683	22.25	0	1933.026193	2	967.0157471	-1.021755102	-0.00098754	75.68541803
																			QLFDQVVK	12	4505257	2xTMT6plex [N-Term; K8]	1.369	1.393	1.305	4.03649807	44.99	0	1434.872072	2	717.9403687	0.968068663	0.000694527	67.82446326
																			KFVIKPIDK	4	4505257	4xTMT6plex [N-Term; K1; K5; K9]	0.446	0.888		2.754837513	4.15	1	2004.339121	4	501.8404541	0.432055754	0.000216497	57.66246428
																			EVWFFGLQYQDTK	15	4505257	2xTMT6plex [N-Term; K13]	1.128	100	100	2.418915272	13.58	0	2119.126448	2	1060.063232	-3.425886292	-0.003629943	89.69204692
																			AQMVQEDLEK	8	4505257	2xTMT6plex [N-Term; K10]; 1xOxidation [M3	0.622	0.543	0.759	2.865151405	43.23	0	1664.892943	2	824.9450684	-9.198950445	-0.007584066	48.08222079
																			FVIKPIDKK	2	4505257	4xTMT6plex [N-Term; K4; K8; K9]	0.614	0.411		2.204612494		1	2004.339121	4	501.8398743	-0.725101692	-0.000363337	63.42701961
																			ALELEQER	16	4505257	1xTMT6plex [N-Term]	0.758	0.76	0.71	3.355436802	38.87	0	1216.673482	2	608.8408813	0.824983307	0.000501868	39.59920667
																			SGYLAGDK	4	4505257	2xTMT6plex [N-Term; K8]	1.244	1.284		2.55376029	37.58	0	1268.725073	2	634.8658447	-0.520409732	-0.000330128	46.07099872
																			IGFPWSEIR	4	4505257	1xTMT6plex [N-Term]		0.996	1.093	2.909641981	30.66	0	1333.746588	2	667.3757935	-1.70756271	-0.001138728	82.08487989
																			KTQEQLALEMAELTAR	6	4505257	2xTMT6plex [N-Term; K1]	0.853	0.744	0.728	2.504420757	12.6	1	2290.284088	3	764.0993042	-0.317957212	-0.000242737	64.3368194
																			FVIKPIDK	8	4505257	3xTMT6plex [N-Term; K4; K8]	1.014	0.898	0.786	2.657356024	10.37	1	1647.081226	3	549.6986694	0.139319651	7.65E-05	56.62551159
																			EALLQASR	8	4505257	1xTMT6plex [N-Term]	0.896	0.826	0.385	2.956180096	36.64	0	1116.657438	2	558.8323975	0.071536599	3.99E-05	44.82140575
																			GFSTWLK	7	4505257	2xTMT6plex [N-Term; K7]	1.366	1.161	1.113	3.296529531	36.58	0	1296.771629	2	648.8890991	-0.545807601	-0.000353894	71.15204658
																			LNKDQWEER	8	4505257	2xTMT6plex [N-Term; K3]	0.653	0.656	0.647	3.328296661	15.21	1	1675.91679	3	559.3098145	-1.133547105	-0.000633244	48.72645756
																			KESPLLFK	3	4505257	3xTMT6plex [N-Term; K1; K8]		0.01	1.922	2.586546183	12.5	1	1649.06049	3	550.3583984	0.092138828	5.06E-05	48.9020217
																			ESPLLFK	2	4505257	2xTMT6plex [N-Term; K7]			0.981	2.906086683	27.68	0	1291.802595	2	646.4057617	1.278568031	0.000825829	69.38032606
																			AMLENEK	9	4505257	2xTMT6plex [N-Term; K7]	0.675	0.804	0.724	3.303355694	39.51	0	1292.728444	2	646.8676758	-0.285417639	-0.000184484	33.53856113
																			AQSEAEK	6	4505257	2xTMT6plex [N-Term; K7]		1.102	100	2.633359671	22.22	0	1220.688688	2	610.8481445	0.2661387	0.000162436	20.11847446
																			QEAEEAK	4	4505257	2xTMT6plex [N-Term; K7]	1.375			3.26480031	27.84	0	1262.699252	2	631.8534546	0.301203701	0.000190165	29.05025669
																			QIEEQTKK	4	4505257	3xTMT6plex [N-Term; K7; K8]	0.515	0.557		2.78971982	7.76	1	1691.030646	3	564.3476563	-1.319290106	-0.000743653	29.64244371
																			LFFLQVK	2	4505257	2xTMT6plex [N-Term; K7]	0.846			2.326160192	22.22	0	1352.870615	2	676.9376831	-1.866829713	-0.00126279	89.42239087
																			KVTAQDVR	2	4505257	2xTMT6plex [N-Term; K1]		1.052		2.478715658	9.24	1	1374.84692	3	458.9534302	-0.859901302	-0.000394078	28.18542629
																			EKEELMER	4	4505257	2xTMT6plex [N-Term; K2]	0.823	0.839		2.509242773	8.85	1	1521.8347	3	507.949707	-0.086682377	-4.40E-05	35.52617194
4503571	ENO1	alpha-enolase isoform 1	2023	NP_001419.1	60.13824885	1	21	23	324	434	47.139	7.39	1.06	1.1	1.34	1.17	Significant	0.0056																
																			FTASAGIQVVGDDLTVTNPK	8	4503571	2xTMT6plex [N-Term; K20]	1.464			3.151371241	40.7	0	2491.380825	3	831.1326904	1.081106925	0.000897816	79.60719657
																			GNPTVEVDLFTSK	41	4503571	2xTMT6plex [N-Term; K13]	1.254	1.285	1.12	4.245165348	61.06	0	1865.04205	2	933.024353	-0.332633418	-0.000310188	64.49949788
																			AAVPSGASTGIYEALELR	70	4503571	1xTMT6plex [N-Term]	1.115	1.212	1.115	4.654744148	66.15	0	2034.106886	2	1017.556091	-0.97325899	-0.000989856	90.31027468
																			VNQIGSVTESIQACK	32	4503571	2xTMT6plex [N-Term; K15]; 1xCarbamidomethyl [C14	2.979	2.406	2.243	3.623705626	40.1	0	2092.14726	3	698.0535278	-0.587857236	-0.000409961	55.9674302
																			SGETEDTFIADLVVGLCTGQIK	19	4503571	2xTMT6plex [N-Term; K22]; 1xCarbamidomethyl [C17		1.757	0.602	5.462022305	47.59	0	2811.485032	3	937.8348999	1.819201231	0.001704886	116.8625625

Bhat et al,2017. Cigarette smoke and chewing tobacco alter expression of different sets of miRNAs in oral keratinocytes

Supplementary Table S2(A). List of all proteins and peptides identified in OKF6/TERT1 cells chronically treated with chewing tobacco using Sequest and Mascot search algorithms

Accession	Gene_Symbol	Description	Gene_ID	Refseq_Accession	Coverage	Proteins	#Unique_Peptides	#Peptides	#PSMS	#AAs	MW [kDa]	calc. pI	OKF6-STE-6M/OKF6P_R1	OKF6-STE-6M/OKF6P_R2	OKF6-STE-6M/OKF6P_R3	Median fold change	Status	P-value (6M)	Sequence	#PSMs	Protein Accessions	Modifications	OKF6-STE-6M/OKF6P_R1	OKF6-STE-6M/OKF6P_R2	OKF6-STE-6M/OKF6P_R3	Xcorr	Ionscore	# Missed Cleavages	Theo. MH+ [Da]	Charge	m_z	DeltaM [ppm]	Deltam/z [Da]	RT [min]
																			DYPVVSIEDPFDQDDWGAWQK	62	4503571	2xTMT6plex [N-Term; K21]	1.19	1.336	1.342	4.380342484	34.15	0	2968.440524	3	990.1516724	-0.020307012	-2.01E-05	104.9440258
																			IGAEVYHNLK	18	4503571	2xTMT6plex [N-Term; K10]	1.884	0.988	1.907	4.114402771	49.93	0	1601.941548	2	801.4737549	-0.820850409	-0.000657477	48.47931552
																			LAMQEFMILPVGAANFR	18	4503571	1xTMT6plex [N-Term]	1.172	1.129		4.356115341	48.7	0	2137.149953	3	713.0546875	-0.20781415	-0.000148043	104.6620923
																			VVIGMDVAASEFFR	66	4503571	1xTMT6plex [N-Term]	2.322	1.124	0.773	3.004650116	43.18	0	1769.945758	2	885.4762573	-0.293557902	-0.000259791	101.8048845
																			DATNVGDEGGFAPNILENK	16	4503571	2xTMT6plex [N-Term; K19]	1.455	1.783	1.23	5.197173119	62.42	0	2419.250539	3	807.0866089	-2.176417762	-0.0017551	63.56641072
																			LAQANGWGVMVSHR	2	4503571	1xTMT6plex [N-Term]		1.586		3.945802689	20.3	0	1754.932173	3	585.6485596	-0.597059578	-0.000349266	62.74549482
																			LMIEMDGTENK	14	4503571	2xTMT6plex [N-Term; K11]	1.538	1.454	100	3.711425781	41.85	0	1738.911964	2	869.9594727	-0.169656375	-0.000147509	66.06022497
																			TIAPALVSK	4	4503571	2xTMT6plex [N-Term; K9]	1.121			3.961619854	46.33	0	1357.881908	2	679.4442139	-0.557445843	-0.000378473	58.89196014
																			KLNVTEQEK	40	4503571	3xTMT6plex [N-Term; K1; K9]	1.211	1.271	1.253	4.197585583	42.1	1	1776.08341	2	888.5444336	-1.024559145	-0.000909851	36.19672055
																			SCNCLLLK	8	4503571	2xTMT6plex [N-Term; K8]; 2xCarbamidomethyl [C2; C4	0.957	1.08		2.938744783	34.16	0	1465.827113	2	733.413147	-5.522721348	-0.004047677	53.16716474
																			YISPDQLADLYK	41	4503571	2xTMT6plex [N-Term; K12]	1.226	1.245	1.293	3.927219391	41.56	0	1884.051886	2	942.5299072	0.345846699	0.000325797	93.13165754
																			LAMQEFMILPVGAANFR	4	4503571	1xTMT6plex [N-Term]; 1xOxidation [M7]	100			1.55277586	13.33	0	2153.144868	3	713.0546875	-0.20781415	-0.000148043	104.6620923
																			IEEELGSK	4	4503571	2xTMT6plex [N-Term; K8]	0.786		1.168	3.502087593	43.75	0	1362.788067	2	681.8977661	0.138258154	9.42E-05	45.94455235
																			IDKLMIEMDGTENK	6	4503571	3xTMT6plex [N-Term; K3; K14]	0.549	0.478	0.542	2.706885338	14.91	1	2324.280866	3	775.4313354	-0.607694347	-0.000470817	67.60297816
																			LNVTEQEK	10	4503571	2xTMT6plex [N-Term; K8]	1.894	1.797	1.512	3.520886898	44.31	0	1418.825515	2	709.9165039	0.152155778	0.000107941	39.83655162
																			LMIEMDGTENK	22	4503571	2xTMT6plex [N-Term; K11]; 1xOxidation [M	1.311	1.227	1.174	3.06310463	37.8	0	1754.906879	2	869.9594727	-0.169656375	-0.000147509	66.06022497
																			VVIGMDVAASEFFR	8	4503571	1xTMT6plex [N-Term]; 1xOxidation [M5]	1.247		1.314	1.993044019	24.82	0	1785.940673	2	885.4762573	-0.293557902	-0.000259791	101.8048845
																			AVEHINK	19	4503571	2xTMT6plex [N-Term; K7]	0.7	0.948	0.98	3.325081587	40.85	0	1268.772692	2	634.8893433	-1.010509267	-0.000641053	29.62219938
																			EGLELLK	4	4503571	2xTMT6plex [N-Term; K7]	1.578	1.38		3.000424623	30.06	0	1259.79751	2	630.4014282	-1.531940389	-0.000964967	61.62459348
																			AAVPSGASTGIYEALELRDNDK	2	4503571	2xTMT6plex [N-Term; K22]	100			1.435704827	1.52	1	2735.461594	3	912.4928589	0.888135356	0.00080982	57.89741677
																			DATNVGDEGGFAPNILENKEGLELLK	2	4503571	3xTMT6plex [N-Term; K19; K26]				1.366963744		1	3430.867275	3	1144.292847	-0.958469295	-0.001096127	87.3749397
733605926	TUBA1C	tubulin alpha-1C chain isoform a	84790	NP_001290043.1	69.7495183	1	3	22	573	519	57.693	5.07	1.26	1.16	1.15	1.19	Significant	0.00087																
																			DVNAAIATIK	25	733605926	2xTMT6plex [N-Term; K10]	1.152	1.222	1.158	5.292680264	59.55	0	1473.9041	2	737.4550781	-0.827971101	-0.000610175	68.06345176
																			TIGGGDDSFNTFFSETGAGK	31	733605926	2xTMT6plex [N-Term; K20]	1.197	1.314	1.134	5.582830429	57.48	0	2466.218904	3	822.7440186	-0.568402884	-0.000467269	74.49892955
																			AYHEQLTVAEITNACFEPANQMVK	9	733605926	2xTMT6plex [N-Term; K24]; 1xCarbamidomethyl [C15			0.83	4.622302532	30.48	0	3222.632775	4	806.4118652	-2.216905555	-0.001786068	72.17994116
																			AVFVDLEPTVIDEVR	56	733605926	1xTMT6plex [N-Term]	0.919	1.154	1.122	3.982324839	52.47	0	1931.068709	2	966.0397339	1.803039644	0.001740897	80.82953655
																			ECISIHVGQAGVQIGNACWELYCLEHGIQPDGQMPSDK	7	733605926	2xTMT6plex [N-Term; K38]; 3xCarbamidomethyl [C2; C18; C23]; 1xOxidation [M34	100		1.394	5.606026649	30.81	0	4771.277722	4	1193.57251	-1.99373167	-0.002378162	90.33142578
																			ECISIHVGQAGVQIGNACWELYCLEHGIQPDGQMPSDK	28	733605926	2xTMT6plex [N-Term; K38]; 3xCarbamidomethyl [C2; C18; C23	3.149	100	1.189	6.228083134	25.12	0	4755.282807	4	1193.57251	-1.99373167	-0.002378162	90.33142578
																			NLDIERPTYTNLNR	12	733605926	1xTMT6plex [N-Term]	1.097	1.333	1.458	4.746809006	27.62	0	1948.044954	3	650.0187988	-1.596973221	-0.001036992	44.0613833
																			VGINYQPPTVVPGGDLAK	5	733605926	2xTMT6plex [N-Term; K18]	100	0.709		3.04886651	24.84	0	2283.311289	2	1142.158691	-0.517860839	-0.000591219	80.08766306
																			IHFPLATYAPVISAEK	4	733605926	2xTMT6plex [N-Term; K16]	1.276	1.48		3.60306406	31.69	0	2215.289097	3	739.1016235	0.551009559	0.000406882	81.69282156
																			AFVHWYVGEGMEEGEFSEAR	60	733605926	1xTMT6plex [N-Term]; 1xOxidation [M11]	1.014	1.094	1.093	4.193455219	50.07	0	2575.176104	3	859.0631714	-0.444066657	-0.000381183	77.35264765
																			AFVHWYVGEGMEEGEFSEAR	159	733605926	1xTMT6plex [N-Term]	1.068	1.065	1.124	4.800458431	48.34	0	2559.181189	3	859.0631714	-0.444066657	-0.000381183	77.35264765
																			FDGALNVDLTEFQTNLVPYPR	10	733605926	1xTMT6plex [N-Term]	1.646	1.619	1.724	4.742277622	74.42	0	2638.371433	3	880.1283569	-0.34697132	-0.000305146	104.4415982
																			QLFHPEQLITGK	37	733605926	2xTMT6plex [N-Term; K12]	1.192	1.224	1.303	4.304189682	46.16	0	1869.09984	2	935.0534058	-0.162980359	-0.000152313	66.83108121
																			LEFSIYPAPQVSTAVVEPYNSILTTHTTLEHSDCAFMVDNEAIYDICR	17	733605926	1xTMT6plex [N-Term]; 2xCarbamidomethyl [C34; C47]				2.73541522	5.3	0	5746.75929	4	1437.44397	-0.912067739	-0.001310358	106.1922846
																			EDAANNYAR	14	733605926	1xTMT6plex [N-Term]	1.783	2.081	1.508	3.349599123	46.31	0	1252.611945	2	626.8093872	-0.35677924	-0.000223453	27.54228179
																			EIIDLVLDR	18	733605926	1xTMT6plex [N-Term]	0.86	0.711	0.717	3.620397091	51.22	0	1314.783033	2	657.8947144	-0.66982083	-0.000440335	84.04237698
																			EDMAALEKDYEEVGADSADGEDEGEEY	4	733605926	2xTMT6plex [N-Term; K8]	0.361			2.821202278	7.05	1	3424.478621	3	1142.162109	-1.99912038	-0.002281982	74.89724575
																			AVCMLSNTTAVAEAWAR	5	733605926	1xTMT6plex [N-Term]; 1xCarbamidomethyl [C3				1.549752474	10.39	0	2080.051696	2	1040.528564	-0.886455733	-0.000921937	91.26314566
																			GHYTIGKEIIDLVLDR	6	733605926	2xTMT6plex [N-Term; K7]		5.781	4.657	3.340520859	13.1	1	2300.337838	4	575.838623	-2.249848876	-0.001293853	88.53045888
																			YMACCLLYR	16	733605926	1xTMT6plex [N-Term]; 2xCarbamidomethyl [C4; C5	0.875	0.696	0.789	3.333981514	29.42	0	1478.715564	2	747.8575439	-1.784967331	-0.001334005	48.25769338
																			TIQFVDWCPTGFK	8	733605926	2xTMT6plex [N-Term; K13]; 1xCarbamidomethyl [C8				2.098872662	13.82	0	2057.09304	2	1029.049072	-1.055833989	-0.001085974	97.68642525
																			YMACCLLYR	8	733605926	1xTMT6plex [N-Term]; 2xCarbamidomethyl [C4; C5]; 1xOxidation [M2	1.192	1.123	0.892	1.688249826	34.9	0	1494.710479	2	747.8575439	-1.784967331	-0.001334005	48.25769338
																			FDLMYAK	16	733605926	2xTMT6plex [N-Term; K7]	1.655	2.363	1.156	3.340296984	32.15	0	1345.759016	2	673.3817749	-2.037969117	-0.001371308	81.28086024
																			LSVDYGK	14	733605926	2xTMT6plex [N-Term; K7]	1.364	1.45	0.981	3.336693764	38.06	0	1239.73491	2	620.3706055	-0.786621795	-0.000487601	39.27987182
																			LSVDYGKK	2	733605926	3xTMT6plex [N-Term; K7; K8]		0.439		2.610091925	6.99	1	1596.992804	3	533.0023193	-0.250296799	-0.000133241	41.4246211
																			FDLMYAK	2	733605926	2xTMT6plex [N-Term; K7]; 1xOxidation [M4]	1.858			2.091995478	14.82	0	1361.753931	2	673.3817749	-2.037969117	-0.001371308	81.28086024
295842578	MX1	interferon-induced GTP-binding protein Mx1 isoform a	4599	NP_001171517.1	65.40785498	1	32	35	189	662	75.473	5.83	0.15	0.21	0.12	0.16	Significant	0.026																
																			GQQEIQDQLSLSEALQR	12	295842578	1xTMT6plex [N-Term]			0.124	4.71675539	42.48	0	2172.14579	3	724.7196655	-0.620104771	-0.000448986	67.99886345
																			ALEEPAVDMLHTVTDMVR	9	295842578	1xTMT6plex [N-Term]	0.152	0.01	0.01	3.308746099	38.54	0	2256.156555	3	752.7243042	0.799863668	0.000601539	93.26237093
																			VAVGNQPADIGYK	4	295842578	2xTMT6plex [N-Term; K13]	0.159	0.234		4.235338211	52.02	0	1790.021255	2	895.513916	-0.390748852	-0.000349724	57.33074052
																			ALGVEQDLALPAIAVIGDQSSGK	12	295842578	2xTMT6plex [N-Term; K23]	0.01	0.01		3.810830832	57.39	0	2710.539116	3	904.1845093	-0.052180752	-4.71E-05	108.345893
																			QETISLVVVPSNVDIATTEALSMAQEVDPEGDR	6	295842578	1xTMT6plex [N-Term]				2.997096777	26.76	0	3742.88943	3	1248.300781	-0.438001184	-0.000546463	108.6738318
																			VRPCIDLIDSLR	7	295842578	1xTMT6plex [N-Term]; 1xCarbamidomethyl [C4	0.265	0.01	0.01	4.229460716	29.32	0	1685.956991	3	562.6575317	0.623174167	0.000350215	68.78237565
																			AMLQLLQDKDTYSWLLK	4	295842578	3xTMT6plex [N-Term; K9; K17]	0.133	0.145		4.775332451	47.14	1	2753.58787	3	918.5339966	-0.157468701	-0.000144535	105.6683058
																			SSVLEALSGVALPR	8	295842578	1xTMT6plex [N-Term]	0.01	0.117		4.629473686	55.22	0	1627.958037	2	814.4830322	0.461303736	0.000375492	94.25003457
																			NPGSVAENNLCSQYEEK	2	295842578	2xTMT6plex [N-Term; K17]; 1xCarbamidomethyl [C11		0.186		4.570549488	58.47	0	2397.17566	3	799.7302856	0.268709384	0.000214715	46.2798116
																			TIGILTKPDLVDKGTEDK	2	295842578	4xTMT6plex [N-Term; K7; K13; K18]	0.01			3.106286287	6.11	1	2859.721276	4	715.6854248	-0.491905019	-0.000351678	65.41583498
																			LTSELITHICK	6	295842578	2xTMT6plex [N-Term; K11]; 1xCarbamidomethyl [C10	0.147	0.156	0.01	3.645566463	34.35	0	1773.034462	3	591.6829834	-0.03692804	-2.18E-05	71.42976036
																			VVSEVDIAK	3	295842578	2xTMT6plex [N-Term; K9]	0.155	0.158			59.92	0	1417.866652	2	709.4367065	-0.363499672	-0.000257697	48.76799998
																			LHFQMEQIVYCQDQVYR	2	295842578	1xTMT6plex [N-Term]; 1xCarbamidomethyl [C11	0.01			2.875366926	18.75	0	2486.215801	3	829.4099121	-0.248627603	-0.000206047	82.81651375
																			AMLQLLQDK	6	295842578	2xTMT6plex [N-Term; K9]	0.187	0.01	0.01	3.447919846	46.62	0	1517.912556	2	759.4594727	-0.584689478	-0.000443754	79.68648835
																			ATVPCLAEK	5	295842578	2xTMT6plex [N-Term; K9]; 1xCarbamidomethyl [C5	0.212	0.242	0.115	3.749871731	42.44	0	1446.839057	2	723.9232788	0.154652438	0.000111879	52.3274292
																			YGVDIPEDENEK	2	295842578	2xTMT6plex [N-Term; K12]	0.184			2.941602707	35.36	0	1865.953294	2	933.4800415	-0.261502894	-0.000243976	53.09496749
																			TIGILTKPDLVDK	6	295842578	3xTMT6plex [N-Term; K7; K13]	0.01	0.01		3.103589296	19.22	0	2100.324703	3	700.7787476	-1.434827774	-0.001004535	63.63708594
																			LAFTDVSIK	6	295842578	2xTMT6plex [N-Term; K9]	0.096	0.129	0.121	3.800651312	41.15	0	1451.887387	2	726.4466553	-0.932161225	-0.000676697	64.31386378
																			ELPGFVNYR	10	295842578	1xTMT6plex [N-Term]	0.106	0.01	0.051	3.056459188	38.42	0	1323.725852	2	662.3665771	0.019178348	1.27E-05	68.73274045
																			SLPLLENQIK	7	295842578	2xTMT6plex [N-Term; K10]	0.115	0.147		4.041563988	45.17	0	1613.003814	2	807.0056152	0.086719417	6.99E-05	78.04799427
																			NFEEFFNLHR	4	295842578	1xTMT6plex [N-Term]	0.09	0.107		3.245796442	32.31	0	1581.801142	3	527.9382324	-0.631163013	-0.000332791	75.61675401
																			WSTIIENNFQEGHK	6	295842578	2xTMT6plex [N-Term; K14]				2.321280718	9.74	0	2161.144223	3	721.0513306	-2.214068237	-0.001594974	64.98195713
																			ALEEPAVDMLHTVTDMVR	5	295842578	1xTMT6plex [N-Term]; 1xOxidation [M]		0.01		1.252722383	22.07	0	2272.15147	3	752.7243042	0.799863668	0.000601539	93.26237093
																			AQNAIAGEGMGISHELITLEISSR	2	295842578	1xTMT6plex [N-Term]				1.697118759	4.9	0	2726.434444	3	909.4769897	-6.612248374	-0.006009287	82.08109137
																			DTYSWLLK	4	295842578	2xTMT6plex [N-Term; K8]	0.01			2.120793819	23.42	0	1483.856087	2	742.4310303	-0.878301566	-0.000651637	98.6041243
																			KLVNEDKWR	7	295842578	3xTMT6plex [N-Term; K1; K7]	0.109	0.144	0.104	3.545197964	8.49	2	1875.141928	3	625.7184448	-0.611685615	-0.000382332	43.51263348
																			TFETIVK	4	295842578	2xTMT6plex [N-Term; K7]		0.01	0.185	2.967485428	35.51	0	1295.79751	2	648.4018555	-0.829946417	-0.000537721	52.40401694
																			GTEDKVVDVVR	2	295842578	2xTMT6plex [N-Term; K5]	0.383	0.8		2.567967415		1	1674.979056	3	558.9976807	-0.338492479	-0.000188989	48.77519625
																			KSWDFGAFQSSSATDSSMEEIFQHLMAYHQEASK	2	295842578	3xTMT6plex [N-Term; K1; K34]				1.515381098	2.38	1	4568.21056	5	914.4475098	-0.463486052	-0.00042346	99.02420997
																			IFFENHPYFR	6	295842578	1xTMT6plex [N-Term]	0.11	0.133	0.145	2.771538973	21.36	0	1598.831714	3	533.6150513	-0.69658451	-0.00037124	64.58788058
																			DVPDLTLIDLPGITR	7	295842578	1xTMT6plex [N-Term]	0.01	0.01	0.126	1.621049404	13.03	0	1867.073795	2	934.0394287	-1.18579037	-0.001106979	95.06441176
																			ITEELQK	10	295842578	2xTMT6plex [N-Term; K7]	0.162	0.145	0.138	3.364105463	41.27	0	1318.798238	2	659.9030151	0.391010105	0.000257832	47.54943348
																			NLVFHLK	5	295842578	2xTMT6plex [N-Term; K7]	0.114	0.151	0.01	2.549662828	29.09	0	1328.845463	3	443.6207275	1.63022507	0.000722106	57.88478151
																			MFFLIDK	7	295842578	2xTMT6plex [N-Term; K7]	0.01	0.01		2.530653954	30.11	0	1371.811051	2	686.4083252	-1.222915775	-0.000838805	84.51289675
																			DLLEEGK	2	295842578	2xTMT6plex [N-Term; K7]	0.099			2.995141029	32.14	0	1261.740389	2	631.3737183	-0.181397508	-0.000114438	53.48175396
																			EKELEEEK	2	295842578	3xTMT6plex [N-Term; K2; K8]		0.431		2.030973434	6.61	1	1720.993592	3	574.3361816	0.232117003	0.000133157	35.87459197
20070125	P4HB	protein disulfide-isomerase precursor	5034	NP_000909.2	62.4015748	1	29	29	228	508	57.081	4.87	1.09	1.06	0.89	1.01	Significant	0.0038																
																			LGETYKDHENIVIAK	7	20070125	3xTMT6plex [N-Term; K6; K15]	1.309	1.216	1.231	4.515923977	16.81	1	2417.400721	4	605.1066284	1.639129103	0.000990608	53.89505304
																			VDATEESDLAQQYGVR	11	20070125	1xTMT6plex [N-Term]	1.226	1.194	0.01	5.149090767	68.32	0	2009.997729	3	670.6704712	-0.432252121	-0.000289609	60.18553408
																			FLESGGQDGAGDDDDLEDLEEAEEPDMEEDDDQK	4	20070125	2xTMT6plex [N-Term; K34]; 1xOxidation [M27				2.620291471	18.82	0	4231.766876	4	1054.697998	-0.426745687	-0.000449766	76.85048739
																			FLESGGQDGAGDDDDLEDLEEAEEPDMEEDDDQK	14	20070125	2xTMT6plex [N-Term; K34]	0.889	1.624	0.895	3.316466331	30.34	0	4215.771961	4	1054.697998	-0.426745687	-0.000449766	76.85048739
																			HNQLPLVIEFTEQTAPK	2	20070125	2xTMT6plex [N-Term; K17]	1.409			4.080925465	29.47	0	2423.369866	3	808.4613647	-0.134144492	-0.000108361	92.51592224
																			ILFIFIDSDHTDNQR	7	20070125	1xTMT6plex [N-Term]	1.298	1.384	2.017	3.536820173	32.08	0	2063.07592	3	688.3626099	-1.281397418	-0.000881207	99.88528699
																			YKPESEELTAER	22	20070125	2xTMT6plex [N-Term; K2]	1	0.92	0.954	3.887169123	24.58	0	1910.027128	3	637.3461914	-1.626684357	-0.00103567	32.92186089
																			SNFAEALAAHK	2	20070125	2xTMT6plex [N-Term; K11]		1.341		4.169844151	44.99	0	1616.916062	3	539.6436157	0.143673487	7.74E-05	47.72383219
																			IKPHLMSQELPEDWDKQPVK	2	20070125	4xTMT6plex [N-Term; K2; K16; K20]		0.936		4.725407124	12.76	1	3334.90032	5	667.7865601	1.011749271	0.000674817	67.44949905
																			MDSTANEVEAVK	8	20070125	2xTMT6plex [N-Term; K12]	1.506	1.361	1.404	5.230447769	83.53	0	1751.924971	2	876.4645996	-1.740132312	-0.001524291	44.53301355
																			MDSTANEVEAVK	6	20070125	2xTMT6plex [N-Term; K12]; 1xOxidation [M1	1.528	1.17	1.315	3.428671837	59.76	0	1767.919886	2	876.4645996	-1.740132312	-0.001524291	44.53301355
																			QFLQAAEAIDDIPFGITSNSDVFSK	5	20070125	2xTMT6plex [N-Term; K25]			100	2.779272795	24.74	0	3171.661416	3	1057.884644	-6.948527301	-0.007346125	102.0472308
																			KSNFAEALAAHK	8	20070125	3xTMT6plex [N-Term; K1; K12]	0.962	0.939		5.733545303	29.79	1	1974.173957	4	494.2992859	0.687429131	0.000339276	45.89288749
																			DHENIVIAK	2	20070125	2xTMT6plex [N-Term; K9]	1.725			4.32727766	53.72	0	1496.883699	2	748.9456787	0.255137975	0.000190956	40.62227972
																			LITLEEEMTK	16	20070125	2xTMT6plex [N-Term; K10]	1.173	1.492	0.529	3.447272301	47.87	0	1664.95448	2	832.9803467	-0.638894719	-0.000531865	87.77469275
																			EADDIVNWLK	19	20070125	2xTMT6plex [N-Term; K10]	1.666	100	3.221	2.943321943	28.99	0	1660.931043	2	830.9685669	-0.713913406	-0.00059288	78.17580054
																			NFEDVAFDEK	6	20070125	2xTMT6plex [N-Term; K10]	1.128	1.413		2.894275904	39.38	0	1671.863023	2	836.434021	-1.350318645	-0.001128774	61.42568801
																			NNFEGEVTK	2	20070125	2xTMT6plex [N-Term; K9]	1.435			3.931926489	52.6	0	1495.815679	2	748.4105835	-1.195673931	-0.000894254	46.39144108
																			YQLDKDGVVLFK	6	20070125	3xTMT6plex [N-Term; K5; K12]	1.058	1.018		3.424921989	10.73	1	2112.267188	3	704.7601929	-0.550511188	-0.000387609	77.21311651
																			ILEFFGLK	6	20070125	2xTMT6plex [N-Term; K8]	1.128	1.197	1.124	3.628854275	47.49	0	1424.891744	2	712.949646	0.190107205	0.000135441	99.27721407
																			DVESDSAK	10	20070125	2xTMT6plex [N-Term; K8]	1.248	1.313	0.943	3.501703501	47.57	0	1308.704731	2	654.855896	-0.165138711	-0.000108059	34.29596067
																			ENLLDFIK	21	20070125	2xTMT6plex [N-Term; K8]	1.058	1.038	1.709	3.50371027	37.51	0	1449.871737	2	725.4388428	-0.916172852	-0.000664167	81.39820539
																			THILLFLPK	2	20070125	2xTMT6plex [N-Term; K9]	0.937			3.284542084	22.27	0	1540.002692	2	770.5050049	0.026795814	2.06E-05	85.69366482
																			SVSDYDGK	8	20070125	2xTMT6plex [N-Term; K8]	1.271	1.192	1.1	3.224518776	36.79	0	1328.709817	2	664.859375	1.246690571	0.000828245	39.60193852
																			QLAPIWDK	4	20070125	2xTMT6plex [N-Term; K8]		0.884	1.017	3.034496307	28.37	0	1428.861507	2	714.9338379	-0.775308695	-0.000553904	69.90008823
																			ALAPEYAK	12	20070125	2xTMT6plex [N-Term; K8]	0.877	0.922	0.884	3.173234224	38.38	0	1320.792759	2	660.9006958	1.026846607	0.000678126	47.29798913
																			IKPHLMSQELPEDWDKQPVK	1	20070125	4xTMT6plex [N-Term; K2; K16; K20]; 1xOxidation [M6			0.809	2.401572704		1	3350.895235	5	667.7865601	1.011749271	0.000674817	67.44949905
																			KEECPAVR	2	20070125	2xTMT6plex [N-Term; K1]; 1xCarbamidomethyl [C4	1.22			2.725652695	23.67	1	1446.813905	3	482.9428406	0.043791752	2.11E-05	30.43350238
																			IKPHLMSQELPEDWDK	3	20070125	3xTMT6plex [N-Term; K2; K16]		1.946		2.222138405	3.99	0	2653.46267	3	885.1581421	-1.053943777	-0.0009322	58.8114561
																			IFGGEIK	6	20070125	2xTMT6plex [N-Term; K7]	1.198	1.053	1.251	2.749716043	34.48	0	1221.76073	2	611.3829346	-1.749818374	-0.00106893	60.72323244
																			LKAEGSEIR	2	20070125	2xTMT6plex [N-Term; K2]	0.829			3.196992159	12.35	1	1460.883699	3	487.6330566	0.628237467	0.000305927	24.81980386
																			TLDGFKK	2	20070125	3xTMT6plex [N-Term; K6; K7]	0.496		0.543	1.748498201		1	1495.945126	3	499.3195801	-0.627754059	-0.000313029	37.88233683
156766050	AHNAK2	protein AHNAK2	113146	NP_612429.2	21.20793788	1	41	41	157	5795	616.242	5.36	0.87	0.79	0.81	0.82	Significant	0.00085																
																			SAPIQTQPEARPEAELPK	2	156766050	2xTMT6plex [N-Term; K18]	1.102			3.708645582	1.74	0	2420.354944	3	807.4562988	-0.248313011	-0.000200335	51.52220137
																			LEGDLSLAHEDVAGK	2	156766050	2xTMT6plex [N-Term; K15]	1.486			4.182722569	20.02	0	2012.106441	3	671.3729248	-1.103180176	-0.000739905	68.50969979
																			GEEVEGAGWMPGR	2	156766050	1xTMT6plex [N-Term]	0.914			3.597608805	49.38	0	1603.773607	2	802.3893433	-1.369923132	-0.001098523	52.98341171
																			EPTTHAEAQGDEGDGEEGLQR	18	156766050	1xTMT6plex [N-Term]	0.747	1.025	1.131	5.162457466	29.01	0	2455.117069	3	819.0427856	-1.329890313	-0.001088345	27.66318967
																			TLEGQAQETAVAQR	4	156766050	1xTMT6plex [N-Term]	1.047	1.362		2.579658747	37.19	0	1730.923442	2	865.9650269	-0.38422789	-0.000332535	39.19856775
																			GEEVEGAGWMPGREPTTHAEAQGDEGDGEEGLQR	2	156766050	1xTMT6plex [N-Term]		0.952		6.474700451	34.93	1	3810.709902	4	953.4335327	0.629438985	0.000599652	45.81085299
																			SMEDSVDVSAPK	4	156766050	2xTMT6plex [N-Term; K12]		1.062	0.931	4.244821548	60.66	0	1722.898422	2	861.9520874	-0.884500963	-0.000761953	42.49121966
																			LLEGHVPEEAGLK	4	156766050	2xTMT6plex [N-Term; K13]		0.885	0.704	3.213602543	43.82	0	1850.07877	3	617.3649292	0.791667728	0.000488216	55.83356978
																			LPEGPVPEGAGPK	2	156766050	2xTMT6plex [N-Term; K13]		0.78		3.808567286	54.76	0	1705.988892	2	853.4997559	1.959543093	0.001671479	50.16638585
																			LPEGHVPEGAGLK	12	156766050	2xTMT6plex [N-Term; K13]	0.839	0.784	0.8	3.694530725	39.69	0	1762.02634	3	588.0137329	0.173221286	0.00010174	44.51264608
																			SIEASVDVSAPK	2	156766050	2xTMT6plex [N-Term; K12]	0.877			3.159374714	33.09	0	1660.952172	2	830.980835	1.337216033	0.001110526	53.53012133
																			DAHDVSPTSTDTEAQLTVER	2	156766050	1xTMT6plex [N-Term]		0.623		4.353582382	18.3	0	2401.168042	3	801.0601807	-0.854982982	-0.000684319	41.91776294
																			ALVDVSAPK	6	156766050	2xTMT6plex [N-Term; K9]	0.796	0.762	0.734	3.998682976	61.99	0	1357.845523	2	679.4268188	0.617548387	0.000419268	49.06239262
																			LEGDLSLADK	6	156766050	2xTMT6plex [N-Term; K10]	1.692	1.115	1.187	3.45062995	58.56	0	1518.877945	2	759.9434814	1.146583655	0.00087076	48.4619465
																			VQADQVDVK	4	156766050	2xTMT6plex [N-Term; K9]	1.044	0.951		3.666611671	56.09	0	1459.852064	2	730.4291992	-0.645669875	-0.000471291	47.07734796
																			VDPECSVEDSK	4	156766050	2xTMT6plex [N-Term; K11]; 1xCarbamidomethyl [C5	1.021	1.189		2.759255409	35.47	0	1722.862037	2	861.9346924	0.041364673	3.56E-05	48.23704491
																			LEGDLSLADKDVTAK	6	156766050	3xTMT6plex [N-Term; K10; K15]	1.058			3.150752068	17.01	1	2262.315989	3	754.7758789	-1.284180134	-0.000968407	60.17413606
																			MPSFGVSAPGK	7	156766050	2xTMT6plex [N-Term; K11]	1.343	1.105	1.58	2.768687725	43.4	0	1535.865606	2	768.43573	-0.926304396	-0.00071134	52.63389705
																			VEADVSLPSMQGDLK	7	156766050	2xTMT6plex [N-Term; K15]	100	100	100	2.471214056	25.94	0	2047.114563	2	1024.063599	2.617301309	0.002678958	70.40715523
																			AEVTAPDVK	2	156766050	2xTMT6plex [N-Term; K9]	0.775			3.834777355	43.26	0	1387.819702	2	694.4130859	-0.581073319	-0.000403212	45.26103359
																			STEDGAELEEQK	2	156766050	2xTMT6plex [N-Term; K12]				2.460518599	25.93	0	1793.916909	2	897.4615479	-0.607462291	-0.000544868	40.21372845
																			MPSFGVSAPGR	2	156766050	1xTMT6plex [N-Term]	0.897			3.623501301	41.68	0	1334.708822	2	667.8585205	0.706053342	0.000471188	63.21889544
																			MSLSSMEVDVQAPR	4	156766050	1xTMT6plex [N-Term]				1.810670257	23.54	0	1778.897821	2	889.9524536	-0.107073849	-9.52E-05	59.61196472
																			SMEASVDVSAPK	2	156766050	2xTMT6plex [N-Term; K12]; 1xOxidation [M2			0.629	2.154412508	33.45	0	1694.903508	2	847.9542847	-1.306961755	-0.001107587	37.08190202
																			LLEGPVPEEVGLK	6	156766050	2xTMT6plex [N-Term; K13]		0.01	100	2.032933235	18.87	0	1838.103922	2	919.5540161	-1.722543214	-0.001583107	68.24418624
																			EGEATATADR	4	156766050	1xTMT6plex [N-Term]		0.265	100	2.786885262	30.19	0	1249.622175	2	625.3143311	-0.631791465	-0.00039475	22.0162988
																			AEVTAPDVEVSLPSVEVDVEAPR	2	156766050	1xTMT6plex [N-Term]	100			2.849928141	14.45	0	2637.382057	2	1319.196777	1.600423226	0.002110464	72.89444604
																			MPLFGASAPGK	2	156766050	2xTMT6plex [N-Term; K11]	0.924			2.177960396	25.7	0	1533.886341	2	767.4470825	0.356570789	0.00027347	65.81898938
																			LPEGPLPK	4	156766050	2xTMT6plex [N-Term; K8]		0.813	0.844	2.916707993	35.35	0	1308.829144	2	654.9180298	-0.276048015	-0.00018065	50.88399233
																			VEVTSPNLDVSLPSMEVDIQAPGAK	2	156766050	2xTMT6plex [N-Term; K25]	100			1.254625201	1.77	0	3054.643323	3	1018.885193	-0.752152208	-0.000765852	86.73743062
																			VSAPGESIEALVDVSELK	2	156766050	2xTMT6plex [N-Term; K18]				1.449258327	2.07	0	2301.295364	2	1151.14978	-1.338273728	-0.001539882	93.62955406
																			MPEMDLK	4	156766050	2xTMT6plex [N-Term; K7]	0.773	0.988		2.169203758	31.55	0	1321.726001	2	661.3668213	0.276046718	0.000182429	66.75657482
																			LEGDLSLAEK	6	156766050	2xTMT6plex [N-Term; K10]		100		1.867257953	18.06	0	1532.893595	2	766.9500122	-0.552560165	-0.000423508	55.57018114
																			LPEGPVPEGAGLK	3	156766050	2xTMT6plex [N-Term; K13]	100			1.860034823	17.19	0	1722.020192	2	861.5145874	0.990653127	0.000852962	56.57878273
																			SMEDSVDVSAPK	2	156766050	2xTMT6plex [N-Term; K12]; 1xOxidation [M2		0.904		1.921901345	34.44	0	1738.893337	2	861.9520874	-0.884500963	-0.000761953	42.49121966
																			LPETQVLPGEIDETPLSKPGHDLASMEDK	2	156766050	3xTMT6plex [N-Term; K18; K29]				1.113545656	4.15	0	3834.044981	4	959.2660522	-0.678718074	-0.000650559	70.39926701
																			DVDPSLSSATAGGSFQDTEK	1	156766050	2xTMT6plex [N-Term; K20]				0.968864381		0	2470.234948	2	1235.620972	-0.113916543	-0.0001407	49.21722356
																			VMVTSAAR	2	156766050	1xTMT6plex [N-Term]		0.178		1.925835848	8.94	0	1063.613131	2	532.3093872	-1.535366469	-0.000816518	32.62403966
																			VEADVSLSSMQGDLK	1	156766050	2xTMT6plex [N-Term; K15]				1.427163243		0	2037.093827	2	1019.050415	-0.134412991	-0.000136906	69.52532249
																			KADDESK	2	156766050	3xTMT6plex [N-Term; K1; K7]		0.821		2.270797253	8.51	1	1479.862184	3	493.9589844	0.14571965	7.19E-05	23.27138489
																			IQAPSADLEVQAGQVDLK	2	156766050	2xTMT6plex [N-Term; K18]				1.068332195		0	2340.317496	2	1170.661255	-0.966951868	-0.001131487	65.08897602
																			ESPGLWGASILK	2	156766050	2xTMT6plex [N-Term; K12]				1.855161071	2.56	0	1716.009628	2	858.5080566	-0.460917431	-0.000395469	71.13460356
62241042	EPRS	bifunctional glutamate/proline--tRNA ligase	2058	NP_004437.2	29.89417989	1	37	37	166	1512	170.483	7.33	1.17	1.1	1.14	1.14	Significant	0.00032																
																			DQDLEPGAPSMGAK	4	62241042	2xTMT6plex [N-Term; K14]	1.428		1.455	3.619449139	54.86	0	1873.972984	2	937.4852905	-5.16524272	-0.004839763	42.94311878
																			EYIPGQPPLSQSSDSSPTR	6	62241042	1xTMT6plex [N-Term]	1.206	0.979	0.834	3.644447327	49.66	0	2275.140371	2	1138.073486	-0.296458082	-0.000337242	45.98972004
																			AIQGGTSHHLGQNFSK	10	62241042	2xTMT6plex [N-Term; K16]	1.136	1.076		5.872221947	45.16	0	2140.166356	4	535.7973633	0.592217982	0.000316861	34.51446652
																			ERPTPSLNNNCTTSEDSLVLYNR	3	62241042	1xTMT6plex [N-Term]; 1xCarbamidomethyl [C11		100		4.594260216	21.09	0	2909.426064	3	970.4775391	-2.749769997	-0.002666751	60.55061868
																			SLCIPFKPLCELQPGAK	7	62241042	3xTMT6plex [N-Term; K7; K17]; 2xCarbamidomethyl [C3; C10	100	1.135		3.502349854	20.1	0	2645.512597	3	882.5083618	-0.780635399	-0.000688394	87.94472037
																			GSQFGQSCCLR	2	62241042	1xTMT6plex [N-Term]; 2xCarbamidomethyl [C8; C9			1.334	4.404395103	53.35	0	1528.719798	2	764.8632202	-0.41479826	-0.000317055	38.13191559
																			EAPCVLIYIPDGHTK	10	62241042	2xTMT6plex [N-Term; K15]; 1xCarbamidomethyl [C4	1.657	1.354	100	3.966424942	35.62	0	2171.193482	3	724.4015503	-1.558797556	-0.00112815	70.06689044
																			LTVAENEAETK	4	62241042	2xTMT6plex [N-Term; K11]		1.232	1.137	5.035963535	63.69	0	1662.931437	2	831.9702148	1.032079533	0.000858139	50.63128349
																			AYVDDTPAEQMK	6	62241042	2xTMT6plex [N-Term; K12]	1.062	0.985	1.177	4.51752615	54.27	0	1825.940621	2	913.4729004	-1.148492303	-0.001048539	50.85035394
																			LSSCDSFTSTINELNHCLSLR	4	62241042	1xTMT6plex [N-Term]; 2xCarbamidomethyl [C4; C17	100			3.225340128	20.44	0	2683.301716	3	895.1043091	-1.245399981	-0.001113928	97.4780669
																			DDVSISVEEGKENILHVSENVIFTDVNSILR	2	62241042	2xTMT6plex [N-Term; K11]			100	6.886179924	10.62	1	3929.090796	4	983.0271606	-1.013888984	-0.000995915	111.074416
																			KEENLADWYSQVITK	6	62241042	3xTMT6plex [N-Term; K1; K15]	0.969		1.646	3.579666615	20.89	1	2511.406201	3	837.8070068	0.106170723	8.89E-05	84.73052038
																			NPEVGLKPVWYSPK	2	62241042	3xTMT6plex [N-Term; K7; K14]	0.752			3.513230801	10.64	0	2301.3574	3	767.7904663	-0.240920519	-0.000184815	74.58626262
																			HEELMLGDPCLK	2	62241042	2xTMT6plex [N-Term; K12]; 1xCarbamidomethyl [C10	1.233			3.502443552	19.27	0	1900.007261	3	634.0072021	-0.10955468	-6.94E-05	57.54346273
																			QFIAAQGSSR	4	62241042	1xTMT6plex [N-Term]		1.101	1.22	3.386658192	42.58	0	1293.711265	2	647.359375	0.161202894	0.000104275	35.17961363
																			AALLNQHYQVNFK	6	62241042	2xTMT6plex [N-Term; K13]	1.525	0.643	100	3.229614735	28.42	0	2004.143101	3	668.7183838	-1.248994052	-0.000834388	59.964166
																			AYVDDTPAEQMK	6	62241042	2xTMT6plex [N-Term; K12]; 1xOxidation [M11	1.081	1.006	2.32	2.450695276	38.49	0	1841.935536	2	913.4729004	-1.148492303	-0.001048539	50.85035394
																			SVVNMEWDK	6	62241042	2xTMT6plex [N-Term; K9]	1.174	1.177	1.33	3.934344053	40.71	0	1565.839785	2	783.4227905	-0.945642924	-0.000740363	61.74723348
																			KPYIWEYSR	2	62241042	2xTMT6plex [N-Term; K1]			0.429	2.680330276	6.68	0	1699.957198	3	567.3238525	-0.113998642	-6.46E-05	62.12321108
																			VAVQGDVVR	5	62241042	1xTMT6plex [N-Term]	1.255	1.233		2.923179626	57.47	0	1171.699638	2	586.3530273	-0.733551904	-0.000429751	40.34498397
																			GMTVEGLK	2	62241042	2xTMT6plex [N-Term; K8]			1.079	3.338900089	41.52	0	1292.764829	2	646.8860474	-0.008759085	-5.66E-06	45.16309486
																			IVQIPFCGEIDCEDWIK	6	62241042	2xTMT6plex [N-Term; K17]; 2xCarbamidomethyl [C7; C12		1.179		1.372783542	5.01	0	2580.324238	2	1290.66394	-1.408404638	-0.00181707	92.80263031
																			KAPKEDVDAAVK	1	62241042	4xTMT6plex [N-Term; K1; K4; K12]		0.665		4.043897629		2	2187.35187	4	547.5941772	1.375734017	0.000752304	37.6062083
																			EVIPVNVPEAQEEMK	6	62241042	2xTMT6plex [N-Term; K15]	0.987	1.246	1.822	1.997767448	14.93	0	2170.182977	2	1085.592041	-2.843626006	-0.003085594	62.1547795
																			FVELPGAEMGK	11	62241042	2xTMT6plex [N-Term; K11]	0.93	1.135	3.371	2.423526287	25.4	0	1635.918035	2	818.4619751	-0.832489561	-0.000680942	71.08455685
																			SLYDEVAAQGEVVR	2	62241042	1xTMT6plex [N-Term]				1.441792488	12.92	0	1764.932944	2	882.9679565	-2.440746588	-0.002153877	64.97069842
																			GDIIQLQR	2	62241042	1xTMT6plex [N-Term]	1.334			2.246742725	25.24	0	1171.699638	2	586.3529053	-0.941916417	-0.000551822	38.37965907
																			EDVDAAVK	4	62241042	2xTMT6plex [N-Term; K8]	1.495	1.357		3.056947231	50.89	0	1304.746202	2	652.8757935	-1.450179302	-0.000946058	40.94404843
																			VASQGEVVR	4	62241042	1xTMT6plex [N-Term]		0.674	0.618	2.776321411	18.6	0	1173.678902	2	587.3433228	0.397704868	0.000233389	28.56320915
																			NETSAPFK	2	62241042	2xTMT6plex [N-Term; K8]		1.176		3.25224638	18.33	0	1351.762187	2	676.3856812	1.404718009	0.000949422	35.78424388
																			DQVDIAVQELLQLK	5	62241042	2xTMT6plex [N-Term; K14]		100		1.725949883	3.08	0	2070.221077	2	1035.613647	-0.51124884	-0.000529199	110.954763
																			FNHWELK	6	62241042	2xTMT6plex [N-Term; K7]	1.108	1.121	100	2.864031553	28.99	0	1431.814891	3	477.9428711	-0.580472207	-0.000277043	63.75934859
																			IISLDAK	4	62241042	2xTMT6plex [N-Term; K7]	1.22		1.347	2.886656046	35.44	0	1217.786945	2	609.3965454	-0.928651512	-0.00056545	59.41308117
																			WDVSTTK	6	62241042	2xTMT6plex [N-Term; K7]	1.117	1.311	1.235	2.644314528	32.57	0	1294.740723	2	647.8737183	-0.435026529	-0.000281623	44.54724117
																			VIDPVAPR	2	62241042	1xTMT6plex [N-Term]	1.318			2.836269379	37.96	0	1095.67236	2	548.3399658	0.269031725	0.000147385	46.53155253
																			NLQMWEEMK	2	62241042	2xTMT6plex [N-Term; K9]				1.338200569	4.49	0	1666.869705	2	833.9390869	0.715195748	0.000596069	59.38156151
																			YYTLFGR	2	62241042	1xTMT6plex [N-Term]	0.801			2.546047211	11.56	0	1148.630161	2	574.8184814	-0.413387521	-0.000237415	73.11017034
																			LNLNNTVLSK	2	62241042	2xTMT6plex [N-Term; K10]				1.179154038	0.85	0	1573.967763	2	787.4866333	-1.126349903	-0.000886419	56.79838597
54607035	ITGB4	integrin beta-4 isoform 1 precursor	3691	NP_000204.3	24.25905598	1	33	33	152	1822	202.039	6.09	0.98	1.02	0.84	0.95	Significant	0.0033																
																			ALEHVDGTHVCQLPEDQK	10	54607035	2xTMT6plex [N-Term; K18]; 1xCarbamidomethyl [C11	0.895	0.867	100	5.802583218	40.25	0	2534.307342	4	634.3326416	0.550105438	0.000348534	40.67375572
																			VAPGYYTLTADQDAR	6	54607035	1xTMT6plex [N-Term]	1.003	1.342	0.867	4.027351856	57.38	0	1869.954408	2	935.4805298	-0.33421119	-0.00031248	61.37085238
																			GNIHLKPSFSDGLK	8	54607035	3xTMT6plex [N-Term; K6; K14]	0.926	0.941	1.023	5.733614445	30.19	0	2200.305699	4	550.8270264	-8.827555762	-0.00485583	48.75667204
																			RPMSIPIIPDIPIVDAQSGEDYDSFLMYSDDVLR	2	54607035	1xTMT6plex [N-Term]	0.586			4.92018795	21.41	0	4097.044885	4	1025.016724	0.04393441	4.50E-05	105.2840621
																			EAIINLATQPK	6	54607035	2xTMT6plex [N-Term; K11]	1.188	1.254	1.204	4.027685165	49.42	0	1656.009628	2	828.5080566	-0.477617241	-0.000395469	63.55494792
																			DYSTLTSVSSHDSR	2	54607035	1xTMT6plex [N-Term]	1.552			4.526274204	57.57	0	1783.865987	3	595.2938232	0.521152693	0.000309889	44.77540351
																			ISGNLDAPEGGFDAILQTAVCTR	2	54607035	1xTMT6plex [N-Term]; 1xCarbamidomethyl [C21		100		3.044923306	13.29	0	2634.339481	3	878.7850952	0.474955692	0.000417065	95.26761373
																			GECQCGHCVCYGEGR	2	54607035	1xTMT6plex [N-Term]; 4xCarbamidomethyl [C3; C5; C8; C10]		1.114		5.6650033	41.06	0	2057.82475	3	686.6134644	0.529387357	0.000363129	24.41201044
																			SCVQCQAWGTGEK	2	54607035	2xTMT6plex [N-Term; K13]; 2xCarbamidomethyl [C2; C5		100		3.570192814	38.4	0	1968.967188	2	984.9876099	0.383651169	0.000377698	41.63580726

Bhat et al,2017. Cigarette smoke and chewing tobacco alter expression of different sets of miRNAs in oral keratinocytes

Supplementary Table S2(A). List of all proteins and peptides identified in OKF6/TERT1 cells chronically treated with chewing tobacco using Sequest and Mascot search algorithms

Accession	Gene_Symbol	Description	Gene_ID	Refseq_Accession	Coverage	Proteins	#Unique_Peptides	#Peptides	#PSMS	#AAs	MW [kDa]	calc. pI	OKF6-STE-6M/OKF6P_R1	OKF6-STE-6M/OKF6P_R2	OKF6-STE-6M/OKF6P_R3	Median fold change	Status	P-value (6M)	Sequence	#PSMSs	Protein Accessions	Modifications	OKF6-STE-6M/OKF6P_R1	OKF6-STE-6M/OKF6P_R2	OKF6-STE-6M/OKF6P_R3	Xcorr	Ionscore	# Missed Cleavages	Theo. MH+ [Da]	Charge	m_z	DeltaM [ppm]	Deltam/z [Da]	RT [min]
																			DVVSFEQPEFSVSR	12	54607035	1xTMT6plex [N-Term]	0.949	1.304	1.431	3.375471354	31.26	0	1854.943509	2	927.9750977	-0.318170067	-0.000295094	63.26128382
																			DYIPVEGELLFQPGEAWK	8	54607035	2xTMT6plex [N-Term; K18]	100	100		2.542590857	12.41	0	2549.369197	3	850.4608765	-0.439789368	-0.000373728	93.02556053
																			QEVEENLNEVYR	6	54607035	1xTMT6plex [N-Term]	100	100	1.924	2.876338959	34.65	0	1750.880909	2	875.9446411	0.626522659	0.000548483	45.26684128
																			VPSVELTNLYPYCDYEMK	7	54607035	2xTMT6plex [N-Term; K18]; 1xCarbamidomethyl [C13	1.164	1.208	100	3.458784342	23.34	0	2679.345033	3	893.7866821	0.171727311	0.000153372	87.10402399
																			ENLMASDHLDTPMLR	6	54607035	1xTMT6plex [N-Term]		0.62		2.727230072	14.3	0	1971.982948	3	657.9995728	0.617349009	0.000405801	61.74165504
																			LVFSALGPTSLR	6	54607035	1xTMT6plex [N-Term]	1.605	1.025	100	3.033428907	44.79	0	1489.89398	2	745.449707	-1.236804447	-0.000921354	72.13781883
																			LTAGVPDTPTR	6	54607035	1xTMT6plex [N-Term]	1.413	1.45	1.482	3.083515644	30.29	0	1356.768445	2	678.8868408	-1.503778616	-0.00102014	33.6208412
																			VLSTSSTLTR	2	54607035	1xTMT6plex [N-Term]	0.576			3.128142834	22.72	0	1293.757546	2	647.3825073	0.148218218	9.59E-05	46.07069627
																			YWIQGDSESEAHLLDSK	6	54607035	2xTMT6plex [N-Term; K17]	100		100	2.697555542	24.03	0	2436.244725	3	812.7521973	-1.102735327	-0.000895511	61.47428161
																			NVISLTEDVDEFR	4	54607035	1xTMT6plex [N-Term]	0.588			2.6584692	25.87	0	1765.91696	2	883.4627075	0.667431759	0.000589315	90.91888547
																			HVTQEFVSR	4	54607035	1xTMT6plex [N-Term]	1.043	0.563		3.530586958	30.89	0	1331.726915	2	666.3667603	-0.503858696	-0.000335501	39.79120961
																			FHVQLSNPK	2	54607035	2xTMT6plex [N-Term; K9]		1.084		2.853810072	25.66	0	1527.904769	3	509.9725037	-1.185272609	-0.000603661	45.59689406
																			VPLFIRPEDDDEKQLLVEAIDVPAGTATLGR	2	54607035	2xTMT6plex [N-Term; K13]	100			3.303365946	6.89	1	3836.120974	4	959.7836304	-2.159156523	-0.002070696	98.1512018
																			VSVPQTDMRPEK	3	54607035	2xTMT6plex [N-Term; K12]	0.763		0.204	3.390877485	14.98	0	1845.030439	3	615.6815796	-0.13755354	-8.46E-05	43.85170126
																			QLLVEAIDVPAGTATLGR	6	54607035	1xTMT6plex [N-Term]		100		1.840528131	19.53	0	2053.18547	2	1027.093018	-3.268990477	-0.003355922	77.07683238
																			TCEECNFK	2	54607035	2xTMT6plex [N-Term; K8]; 2xCarbamidomethyl [C2; C5	1.356			3.383579254	36.95	0	1545.744171	2	773.3754272	-0.383613164	-0.000296484	40.16735049
																			LLELQEVDSLLR	5	54607035	1xTMT6plex [N-Term]	1.128	100		1.668421507	14.73	0	1656.973353	2	828.9899902	-0.391491661	-0.000324346	104.7834087
																			VDKDCAYCTDEMFR	2	54607035	2xTMT6plex [N-Term; K3]; 2xCarbamidomethyl [C5; C8]; 1xOxidation [M12			0.913	2.794120073	20.77	1	2284.044846	3	762.0199585	0.208541563	0.000158773	41.13296479
																			VDKDCAYCTDEMFR	2	54607035	2xTMT6plex [N-Term; K3]; 2xCarbamidomethyl [C5; C8	1.42			2.446503639	14.45	1	2268.049931	3	762.0199585	0.208541563	0.000158773	41.13296479
																			MLLIENLR	4	54607035	1xTMT6plex [N-Term]	0.764	100		2.348928928	41.11	0	1230.744145	2	615.8757324	0.035168764	2.16E-05	67.55920329
																			AEEVVVR	6	54607035	1xTMT6plex [N-Term]	0.793	0.883	0.851	3.107688665	39.75	0	1030.609426	2	515.8082275	-0.239840495	-0.000123591	34.67629646
																			VPLFIRPEDDDEK	2	54607035	2xTMT6plex [N-Term; K13]			100	1.307963252	6.01	0	2031.116277	3	677.710083	-0.286313772	-0.000193846	57.12805214
																			VSVPQTDMRPEK	4	54607035	2xTMT6plex [N-Term; K12]; 1xOxidation [M8		0.835	0.833	3.136384964	8.01	0	1861.025354	3	615.6815796	-0.13755354	-8.46E-05	43.85170126
																			LTVPGLSENVPYK	1	54607035	2xTMT6plex [N-Term; K13]			100	1.093720555	2.6	0	1875.099171	2	938.0529175	-0.326622225	-0.000306225	75.38847998
																			YEGQFCEYDNFQCPR	2	54607035	1xTMT6plex [N-Term]; 2xCarbamidomethyl [C6; C13				1.553435802		0	2241.953105	2	1121.479004	-1.058620493	-0.001186689	54.62090677
																			VLVDNPK	2	54607035	2xTMT6plex [N-Term; K7]	1.119			2.979705811	32.02	0	1242.782194	2	621.8948364	0.162668538	0.000101081	46.87885076
4758304	PDIA4	protein disulfide-isomerase A4 precursor	9601	NP_004902.1	45.73643411	1	27	27	199	645	72.887	5.07	0.982	0.976	0.977	0.98	Non-Significant	1																
																			IDATSASVLASR	8	4758304	1xTMT6plex [N-Term]	1.036	0.975	0.913	4.421196461	62.02	0	1419.800474	2	710.4039307	0.078220938	5.55E-05	45.98369001
																			MDATANDVPSDR	4	4758304	1xTMT6plex [N-Term]	1.175		1.077	3.698279142	39.17	0	1520.721237	2	760.8641968	-0.078952874	-6.00E-05	33.50292533
																			GESDPAYQQYQDAANNLR	12	4758304	1xTMT6plex [N-Term]	1.246	1.178	1.087	4.941523552	59.07	0	2269.068268	2	1135.037354	-0.369230297	-0.000418904	48.90688898
																			DLGLSESGEDVNAAILDESGK	7	4758304	2xTMT6plex [N-Term; K21]	100	100	100	4.032012939	47.52	0	2577.329577	3	859.7810059	-0.431610255	-0.000370801	70.32416404
																			SHMMDVQGSTQDSAIK	10	4758304	2xTMT6plex [N-Term; K16]	1.146	1.284	1.185	6.018318176	49.26	0	2193.104409	3	737.0387573	1.084167032	0.000798344	33.5306797
																			SHMMDVQGSTQDSAIK	12	4758304	2xTMT6plex [N-Term; K16]; 1xOxidation [M	1.182	1.226	1.41	5.852294445	56.65	0	2209.099324	3	737.0387573	1.084167032	0.000798344	33.5306797
																			SHMMDVQGSTQDSAIK	6	4758304	2xTMT6plex [N-Term; K16]; 2xOxidation [M3; M4		0.986	1.342	3.855423689	33.44	0	2225.094239	3	737.0387573	1.084167032	0.000798344	33.5306797
																			DFPEYTFAIADEEDYAGEVK	10	4758304	2xTMT6plex [N-Term; K20]			1.031	4.551457882	47.28	0	2767.339079	3	923.1173096	-0.615511513	-0.000567776	92.56767748
																			VDATAETDLAK	6	4758304	2xTMT6plex [N-Term; K11]	0.852	0.762	0.881	4.143276691	73.87	0	1591.894323	2	796.4506226	-0.222717556	-0.000177271	43.15385538
																			RFDVSGYPTLK	5	4758304	2xTMT6plex [N-Term; K11]		0.646	0.507	3.69617486	16.66	1	1741.004877	3	581.0067139	0.408833756	0.000237261	62.47994349
																			VSQGQLVVMQPEK	4	4758304	2xTMT6plex [N-Term; K13]		0.868		3.052822113	35.8	0	1901.09304	2	951.0477295	-2.554943669	-0.002428593	62.84983735
																			GQAVDYEGSR	4	4758304	1xTMT6plex [N-Term]	1.038	0.897		3.832004547	65.81	0	1310.653809	2	655.8307495	0.315051492	0.000206462	26.52469095
																			DLGLSESGEDVNAAILDESGKK	4	4758304	3xTMT6plex [N-Term; K21; K22]	1.166			2.889901638	14.83	1	2934.587472	3	978.8676147	0.279186867	0.000273099	76.02224368
																			QLEPVYNSLAK	6	4758304	2xTMT6plex [N-Term; K11]	1.081	0.01	1.014	3.171114922	35.91	0	1720.004542	2	860.506897	1.148325638	0.000987563	60.52070428
																			TFDSIVMDPK	7	4758304	2xTMT6plex [N-Term; K10]	1.032	100	1.061	2.839670181	39.8	0	1610.886401	2	805.9462891	-0.682521747	-0.000549732	66.98913641
																			VEGFPTIYFAPSGDK	6	4758304	2xTMT6plex [N-Term; K15]	3.267	100	100	2.414637566	26.73	0	2086.126114	2	1043.566406	-0.27700147	-0.00028893	77.27926031
																			DKDPPIPVAK	6	4758304	3xTMT6plex [N-Term; K2; K10]	0.873	0.934	0.898	2.66905427	30.63	1	1767.098332	3	589.7042847	-0.017738738	-1.04E-05	49.76713069
																			MDATANDVPSDR	2	4758304	1xTMT6plex [N-Term]; 1xOxidation [M1]	1.318			2.413214207	30.49	0	1536.716152	2	760.8641968	-0.078952874	-6.00E-05	33.50292533
																			FDVSGYPTIK	16	4758304	2xTMT6plex [N-Term; K10]	0.731	0.911		2.745783806	28.84	0	1584.903766	2	792.9556885	0.211194605	0.000167362	74.96107223
																			EVSQPDWTPPPEVTLVLTK	6	4758304	2xTMT6plex [N-Term; K19]	100			2.434645653	9.23	0	2594.448176	2	1297.72644	-0.991201385	-0.00128581	86.70702967
																			FIEEHATK	12	4758304	2xTMT6plex [N-Term; K8]	0.886	0.915	0.886	3.93857193	46.37	0	1432.820036	2	716.913208	-0.625796927	-0.000448327	38.33631759
																			TQEEIVAK	6	4758304	2xTMT6plex [N-Term; K8]	0.963	0.949	1.022	3.481130123	43.74	0	1375.819702	2	688.413269	-0.319964936	-0.000220107	39.53892403
																			QFAPEYEK	6	4758304	2xTMT6plex [N-Term; K8]	0.785	0.914	0.929	3.070252895	32.35	0	1469.804052	2	735.4059448	0.381961417	0.000280704	45.09105069
																			SPPIPLAK	4	4758304	2xTMT6plex [N-Term; K8]			0.908	2.984713793	44.99	0	1280.83423	2	640.9212646	0.798719187	0.000511513	55.164747
																			FAMEPEEFDSDTLR	5	4758304	1xTMT6plex [N-Term]		100	100	1.733335018	2.1	0	1915.89451	2	958.4494019	-1.556797124	-0.00149133	66.11034805
																			VEGFPTIYFAPSGDKK	2	4758304	3xTMT6plex [N-Term; K15; K16]	0.344			2.395638943	10.23	1	2443.384009	3	815.1334229	0.698459465	0.000568868	75.98031994
																			DLEHLSK	8	4758304	2xTMT6plex [N-Term; K7]		0.868	0.833	3.199472666	40.45	0	1299.767272	2	650.3873901	0.17804221	0.000115707	33.48467131
																			EFVTAFK	8	4758304	2xTMT6plex [N-Term; K7]	0.828	0.99	0.524	3.03629899	38.55	0	1299.771295	2	650.3895264	0.370122326	0.000240537	72.15009665
																			QVQEFLK	4	4758304	2xTMT6plex [N-Term; K7]	1.03		1.015	3.463392019	32.9	0	1349.819308	2	675.4140625	1.141308315	0.00077028	55.87094181
																			AATQFWR	3	4758304	1xTMT6plex [N-Term]	1.298		1.331	2.279090881	23.44	0	1108.610094	2	554.8082886	-0.716078237	-0.000396926	45.30552653
41359283	HSPD1	60 kDa heat shock protein, mitochondria	3329	NP_955472.1	51.48342059	1	26	26	326	573	61.016	5.87	0.73	0.93	0.85	0.84	Significant	0.0048																
																			ALMLQGVDLLADAVAVTMGPK	18	41399285	2xTMT6plex [N-Term; K21]	0.686		100	3.863496065	46.74	0	2571.465423	3	863.1584473	0.174254604	0.000150292	112.0912294
																			ALMLQGVDLLADAVAVTMGPK	13	41399285	2xTMT6plex [N-Term; K21]; 1xOxidation [M	0.971		1.195	4.077415466	56.98	0	2587.460338	3	863.1584473	0.174254604	0.000150292	112.0912294
																			ALMLQGVDLLADAVAVTMGPK	2	41399285	2xTMT6plex [N-Term; K21]; 2xOxidation [M3; M18			0.93	4.611672401	38.27	0	2603.455253	3	863.1584473	0.174254604	0.000150292	112.0912294
																			IMQSSSEVGYDAMAGDFVNMVEK	12	41399285	2xTMT6plex [N-Term; K23]	1.088	1.537	1.095	4.756359577	48.45	0	2966.434987	3	989.4818115	-1.383883249	-0.0013684	97.35254469
																			TLNDELEIIEGMK	4	41399285	2xTMT6plex [N-Term; K13]	0.468		1.821	3.118083	57.31	0	1963.0822	2	982.0435791	-1.18101365	-0.001159213	107.2819969
																			KPLVIIAEDVDGEALSTLVLNR	101	41399285	2xTMT6plex [N-Term; K1]	0.827	0.83	0.779	7.153730869	84.81	0	2823.659565	4	706.6704102	0.086864225	6.13E-05	105.2102835
																			IQEIIEQLDVTTSEYEK	9	41399285	2xTMT6plex [N-Term; K17]	1.053	1.017	1.23	4.311676979	48.22	0	2496.348521	3	832.7874756	-0.259519928	-0.000215951	108.7874244
																			KISSIQSIVPALEIANAHR	2	41399285	2xTMT6plex [N-Term; K1]		0.591		4.026110172	39.28	1	2505.491712	4	627.1280518	-0.532964744	-0.000333835	81.26611353
																			ISSIQSIVPALEIANAHR	6	41399285	1xTMT6plex [N-Term]	3.505			3.784864902	43.24	0	2148.233818	3	716.7489014	-0.775836608	-0.000555559	94.63800722
																			VGGTSDVEVNEK	12	41399285	2xTMT6plex [N-Term; K12]	2.028	1.715	1.739	4.718378067	62.68	0	1691.9216	2	846.4642944	-0.17028733	-0.000144056	37.80843286
																			GVMLAVDAVIAELKK	4	41399285	3xTMT6plex [N-Term; K14; K15]	0.406			5.009722233	49.95	1	2244.396822	3	748.8030396	-1.00547623	-0.000752229	113.0784348
																			GYISPYFINTSK	10	41399285	2xTMT6plex [N-Term; K12]	0.522	0.694	0.609	2.946767092	45.7	0	1848.030757	2	924.5183716	-0.698243756	-0.000645188	62.63089938
																			AAVEEGIVLGGGCALLR	18	41399285	1xTMT6plex [N-Term]; 1xCarbamidomethyl [C13	1.013	0.599	1.064	2.844613314	39.81	0	1914.067998	2	957.5362549	-1.444595687	-0.001382527	77.47528826
																			EIGNIISDAMK	10	41399285	2xTMT6plex [N-Term; K11]	1.379	0.807	1.489	3.190019846	45.37	0	1648.934414	2	824.9717407	1.086201669	0.000895538	78.94988645
																			LSDGVAVLK	12	41399285	2xTMT6plex [N-Term; K9]	1.071	1.125	0.825	3.773132324	54.2	0	1359.861173	2	680.4342041	-0.030162546	-2.05E-05	57.93783433
																			VGLQVVAVK	10	41399285	2xTMT6plex [N-Term; K9]	1.842	1.135	0.918	3.646413803	52.09	0	1370.913543	2	685.9598999	-0.743566447	-0.000509683	54.13428412
																			GVMLAVDAVIAELK	2	41399285	2xTMT6plex [N-Term; K14]				2.436344147	26.33	0	1887.138927	2	944.0718994	-1.274374579	-0.001202461	112.6616524
																			VTDALNATR	6	41399285	1xTMT6plex [N-Term]	1.195	0.767	1.104	3.659813643	52.27	0	1189.673817	2	595.340332	-0.360827896	-0.000214634	34.8957084
																			DPGMGAMGGMGGGMGGGMF	6	41399285	1xTMT6plex [N-Term]			0.504	2.498749018	26.83	0	1903.783067	2	952.3950195	-0.160200763	-0.000152494	81.87922589
																			VGEVIVTK	6	41399285	2xTMT6plex [N-Term; K8]	2.143	1.865	1.919	3.331897497	39.27	0	1302.839709	2	651.923584	0.140031616	9.12E-05	45.23790857
																			IMQSSSEVGYDAMAGDFVNMVEK	4	41399285	2xTMT6plex [N-Term; K23]; 1xOxidation [M2				1.826237917	12.04	0	2982.429902	3	989.4818115	-1.383883249	-0.0013684	97.35254469
																			IPAMTIAK	8	41399285	2xTMT6plex [N-Term; K8]	0.825	0.847	0.68	2.645531893	51.82	0	1302.82195	2	651.9150391	0.653270387	0.000425548	68.82441878
																			IGIEIIK	12	41399285	2xTMT6plex [N-Term; K7]	1.313	100	100	2.524600744	29.45	0	1243.838981	2	622.4230957	-0.052976109	-3.29E-05	68.0688176
																			GANPVEIR	4	41399285	1xTMT6plex [N-Term]		0.656	0.629	2.701686144	35.1	0	1084.631224	2	542.8192139	-0.066921939	-3.63E-05	35.31363276
																			CEFQDAYVLLSEK	4	41399285	2xTMT6plex [N-Term; K13]; 1xCarbamidomethyl [C1				1.771952868	7.76	0	2060.077449	2	1030.538452	-3.79675683	-0.003910807	81.48382813
																			IQEIIEQLDVTTSEYEKEK	2	41399285	3xTMT6plex [N-Term; K17; K19]				1.335618496	1.17	1	2982.649009	3	994.8883057	0.454097074	0.000451471	107.4815112
																			IPAMTIAK	3	41399285	2xTMT6plex [N-Term; K8]; 1xOxidation [M4]	0.933	0.771		1.605599642	25.53	0	1318.816865	2	651.9150391	0.653270387	0.000425548	68.82441878
																			GIIDPTK	16	41399285	2xTMT6plex [N-Term; K7]	0.407	0.751	0.764	3.055323124	34.41	0	1201.755645	2	601.3812866	-0.28987408	-0.000174179	39.31969783
																			DDAMLLK	4	41399285	2xTMT6plex [N-Term; K7]	0.946		1.334	2.892309904	35.57	0	1263.73828	2	632.3728638	0.134979738	8.53E-05	60.43306863
																			KGVITVK	6	41399285	3xTMT6plex [N-Term; K1; K7]	1.102	1.222	1.163	3.392436266	16.37	1	1431.986597	3	477.9994507	-1.95406104	-0.00093273	38.20593545
24430192	KRT16	keratin, type I cytoskeletal 16	3868	NP_005548.2	57.50528541	1	12	24	794	473	51.236	5.05	0.15	0.16	0.15	0.15	Significant	0.00047																
																			ASLENSLEETK	70	24430192	2xTMT6plex [N-Term; K11]	0.749	0.681	0.749	5.134794235	76.83	0	1678.926351	2	839.9658203	-1.183723752	-0.000993693	47.08874863
																			LLEGEDAHLSSQQASGQSYSSR	16	24430192	1xTMT6plex [N-Term]	0.154	0.154	0.164	6.98848629	55.93	0	2579.253503	3	860.4223022	-0.445521046	-0.000383037	42.90414471
																			EVFTSSSSSSSR	8	24430192	1xTMT6plex [N-Term]	0.291	0.156	0.136	4.103929043	49.84	0	1489.733182	2	745.3706055	0.50508877	0.000376224	31.87676555
																			ALEEANADLEVK	217	24430192	2xTMT6plex [N-Term; K12]	0.606	0.627	0.555	5.218453407	90.06	0	1759.984201	2	880.4959717	0.264854295	0.00023307	51.10169088
																			APSTYGGGLSVSSR	33	24430192	1xTMT6plex [N-Term]	0.122	0.17	0.151	5.101079464	73.67	0	1567.827751	2	784.4176025	0.113206393	8.87E-05	39.32741745
																			ISSVLAGGSCR	12	24430192	1xTMT6plex [N-Term]; 1xCarbamidomethyl [C10	0.724	0.759	0.837	4.074535847	52.1	0	1335.725201	2	668.3656616	-0.863948522	-0.000576999	54.83568128
																			TDLEMQIEGLKEELAYLR	4	24430192	2xTMT6plex [N-Term; K11]		0.097		3.244133711	15.08	1	2609.42606	3	870.4790649	-1.310102568	-0.001139539	107.4413753
																			ASLENSLEETKGR	10	24430192	2xTMT6plex [N-Term; K11]	0.24	0.184	0.187	5.08894968	44.04	1	1892.048926	3	631.3532104	-2.033743573	-0.001282647	42.91752663
																			GSCGIGGGIGGGSSR	158	24430192	1xTMT6plex [N-Term]; 1xCarbamidomethyl [C3	0.79	0.779	0.729	5.403667927	114.21	0	1507.748455	2	754.3773193	-0.724980431	-0.000546544	34.52531152
																			EVASNSELVQSSR	14	24430192	1xTMT6plex [N-Term]	0.174	0.191	0.159	4.630272865	67.19	0	1634.854694	2	817.9337158	3.340419577	0.00273055	37.57481686
																			TDLEMQIEGLK	4	24430192	2xTMT6plex [N-Term; K11]	0.859	0.946		3.609122753	47.74	0	1734.971193	2	867.9890747	-0.18463473	-0.000160168	84.89662329
																			KNHEEEMLALR	10	24430192	2xTMT6plex [N-Term; K1]	0.154	0.157	0.14	4.461184502	36.34	1	1828.015124	4	457.755249	-8.72936227	-0.003989352	39.33718614
																			EQSSSSFSQGQSS	16	24430192	1xTMT6plex [N-Term]	0.29	0.174	0.147	3.914475679	91.27	0	1574.713175	2	787.8604126	0.237462491	0.000186968	25.81596347
																			QFTSSSSMK	25	24430192	2xTMT6plex [N-Term; K9]	0.795	0.786	0.734	4.360666752	55.43	0	1460.781936	2	730.8937378	-1.189078274	-0.000868492	32.92057792
																			LEQEIATYR	30	24430192	1xTMT6plex [N-Term]	0.873	0.965	0.895	3.989738941	51.78	0	1351.741896	2	676.3740845	-0.742660038	-0.000501942	59.56020918
																			TKYEHELALR	8	24430192	2xTMT6plex [N-Term; K2]	0.111	0.127		4.536514282	35.06	1	1718.000126	4	430.2549133	-1.339988079	-0.000575525	42.531178
																			VLDELTLAR	31	24430192	1xTMT6plex [N-Term]	0.836	0.802	0.88	3.858995199	41.24	0	1258.756818	2	629.8816528	-0.626805691	-0.000394498	67.98248171
																			DAETWFLSK	6	24430192	2xTMT6plex [N-Term; K9]	0.01	0.071	0.053	3.16270256	39.24	0	1554.856815	2	777.9315186	-0.678480883	-0.00052747	73.64595038
																			VTMQNLNDR	14	24430192	1xTMT6plex [N-Term]	0.936	0.83	0.827	3.519829035	42.79	0	1319.6939	2	660.3503418	-0.373735341	-0.000246608	33.03499222
																			DQYEQMAEK	2	24430192	2xTMT6plex [N-Term; K9]	0.184			3.637370825	19.88	0	1599.808879	2	808.4052734	-0.324077095	-0.000261823	40.96076906
																			TDLEMQIEGLKEELAYLR	3	24430192	2xTMT6plex [N-Term; K11]; 1xOxidation [M5			0.01	1.901996136	8.53	1	2625.420975	3	870.4790649	-1.310102568	-0.001139539	107.4413753
																			GQTGGDVNVEMDAAPGVDLSR	6	24430192	1xTMT6plex [N-Term]				2.080275774	15.83	0	2317.129154	2	1159.067139	-0.929234457	-0.001076578	57.51182137
																			IIAATIENAQPILQIDNAR	3	24430192	1xTMT6plex [N-Term]	0.01			1.652011037	4.43	0	2293.307711	2	1147.155029	-2.149160458	-0.002464343	92.5114333
																			LASYLDK	51	24430192	2xTMT6plex [N-Term; K7]	0.689	0.687	0.676	3.160550594	38.22	0	1267.76621	2	634.3873291	0.924415808	0.000585972	69.69252583
																			QFTSSSSMK	6	24430192	2xTMT6plex [N-Term; K9]; 1xOxidation [M8]		0.776	0.751	2.715188742	36.34	0	1476.776851	2	730.8937378	-1.189078274	-0.000868492	32.92057792
																			DQYEQMAEK	6	24430192	2xTMT6plex [N-Term; K9]; 1xOxidation [M6]	0.288	0.163	0.191	2.740729094	37.52	0	1615.803794	2	808.4052734	-0.324077095	-0.000261823	40.96076906
																			VTMQNLNDR	12	24430192	1xTMT6plex [N-Term]; 1xOxidation [M3]	0.775	0.76	0.858	2.103149652	31.21	0	1335.688815	2	660.3503418	-0.373735341	-0.000246608	33.03499222
																			DYSPYFK	18	24430192	2xTMT6plex [N-Term; K7]	0.722	0.787	0.802	3.307164431	35.13	0	1377.745474	2	689.37677	0.572848235	0.00039462	55.89878755
																			KNHEEEMLALR	1	24430192	2xTMT6plex [N-Term; K1]; 1xOxidation [M7]	0.439			1.488894701	2.49	1	1844.010039	4	457.755249	-8.72936227	-0.003989352	39.33718614
4503529	EIF4A1	eukaryotic initiation factor 4A-I isoform 1	1973	NP_001407.1	54.67980296	1	11	21	254	406	46.125	5.48	1.56	1.56	1.63	1.59	Significant	0.00022																
																			GIYAYGFEKPSAIQQR	15	4503529	2xTMT6plex [N-Term; K9]	0.768	1.343	1.252	4.605382919	27.73	0	2286.264673	3	762.7591553	-0.769923845	-0.00058675	52.2933608
																			LQMEAPHIIVGTPGR	14	4503529	1xTMT6plex [N-Term]; 1xOxidation [M3]	1.318	1.285	1.292	5.121593475	56.45	0	1864.031219	3	622.0153198	0.100487939	6.24E-05	43.82215091
																			LNSNTQVVLLSATMPSDVLEVTK	2	4503529	2xTMT6plex [N-Term; K23]				3.062567472	22.55	0	2917.63203	3	973.2163696	0.865635794	0.000841869	92.46886584
																			MFVLDEADEMLSR	13	4503529	1xTMT6plex [N-Term]	100	1.237	1.319	4.445526123	50.89	0	1784.876023	2	892.9421387	0.547771239	0.000488852	96.36472649
																			GIDVQQVSLVINYDLPTNR	21	4503529	1xTMT6plex [N-Term]	1.745	100		3.956655025	51.42	0	2373.29754	3	791.7715454	1.071555684	0.000847707	88.83460315
																			DFTVSAMHGDMDQK	19	4503529	2xTMT6plex [N-Term; K14]; 1xOxidation [M	1.21	1.173	1.36	4.941544056	57.63	0	2055.987982	3	686.0009155	0.102574545	7.03E-05	40.04894543
																			DFTVSAMHGDMDQK	5	4503529	2xTMT6plex [N-Term; K14]			1.642	4.169080257	36.63	0	2039.993067	3	686.0009155	0.102574545	7.03E-05	40.04894543
																			GVAINMVTEEDKR	10	4503529	2xTMT6plex [N-Term; K12]	1.298	1.265	1.366	5.95613575	53.87	1	1920.062468	3	640.6918335	-0.791860548	-0.000506807	51.8518046
																			GYDVIAQAQSGTGK	19	4503529	2xTMT6plex [N-Term; K14]	1.448	1.49		4.311146736	84.58	0	1853.016898	2	927.0126343	0.59052062	0.000547122	54.90388901
																			LQMEAPHIIVGTPGR	20	4503529	1xTMT6plex [N-Term]	1.282	4.438	100	5.159289837	48.08	0	1848.036304	3	622.0153198	0.100487939	6.24E-05	43.82215091
																			ATQALVLAPTR	8	4503529	1xTMT6plex [N-Term]	2.324	1.731	1.756	4.659785271	56.32	0	1369.836465	2	685.421936	0.095004269	6.51E-05	52.50863259
																			VLITTDLLAR	7	4503529	1xTMT6plex [N-Term]	1.338	100		3.460241795	31.03	0	1343.845967	2	672.4265137	-0.161213603	-0.000108323	86.46727257
																			GFKDQIYDIFQK	10	4503529	3xTMT6plex [N-Term; K3; K12]	0.919	0.947	1.153	3.868040085	20.17	1	2189.257352	3	730.4254761	2.066094538	0.001507738	87.63870692
																			GVAINMVTEEDKR	6	4503529	2xTMT6plex [N-Term; K12]; 1xOxidation [M6	1.503	1.218	1.104	3.625261784	39.03	1	1936.057383	3	640.6918335	-0.791860548	-0.000506807	51.8518046
																			KEELTLEGIR	16	4503529	2xTMT6plex [N-Term; K1]	2.563	2.577	2.434	4.118234634	48.11	1	1645.988892	2	823.498291	0.251077788	0.000206636	49.42540259
																			KGVAINMVTEEDKR	9	4503529	3xTMT6plex [N-Term; K1; K13]	1.739	1.668	1.576	4.687574863	16.78	2	2277.320363	4	570.085144	-0.709749999	-0.000404082	43.78714909
																			DIETFYNTSIEEMPLNVADLI	10	4503529	1xTMT6plex [N-Term]			100	2.447560549	30.68	0	2656.326516	2	1328.665527	-1.030777837	-0.001369041	112.6740116
																			DQIYDIFQK	10	4503529	2xTMT6plex [N-Term; K9]	4.24	100	1.74	3.062594414	26.08	0	1627.909579	2	814.4573975	-1.266002824	-0.001030469	73.11595901
																			KGVAINMVTEEDKR	2	4503529	3xTMT6plex [N-Term; K1; K13]; 1xOxidation [M7	1.715			3.327445984	9.54	2	2293.315278	4	570.085144	-0.709749999	-0.000404082	43.78714909
																			KVDWLTEK	5	4503529	3xTMT6plex [N-Term; K1; K8]	1.637	0.957		2.848537683	19.42	1	1706.045568	3	569.3540039	1.107929815	0.00063006	57.89528673
																			QFYINVER	6	4503529	1xTMT6plex [N-Term]	2.369	1.856		2.9047575	35.2	0	1297.710202	2	649.3590088	0.415137466	0.000269364	63.88641699
																			EELTLEGIR	8	4503529	1xTMT6plex [N-Term]	1.177		2.207	3.307424068	37.61	0	1288.730997	2	644.8657227	-5.298613531	-0.003414244	51.6572586
																			DFTVSAMHGDMDQK	3	4503529	2xTMT6plex [N-Term; K14]; 2xOxidation [M7; M11	1.164			2.071088791	12.06	0	2071.982897	3	686.0009155	0.102574545	7.03E-05	40.04894543
																			VDWLTEK	6	4503529	2xTMT6plex [N-Term; K7]	100	100	100	2.119421959	25.85	0	1348.787673	2	674.8964233	-1.559378362	-0.001051635	60.49206049
																			AIIPCIK	8	4503529	2xTMT6plex [N-Term; K7]; 1xCarbamidomethyl [C5	0.838	0.991		2.736866951	31.64	0	1272.811386	2	636.9108887	2.447074079	0.001557332	64.34632447
																			VFDMLNR	6	4503529	1xTMT6plex [N-Term]	1.629	1.038	100	2.142726898	33.45	0	1123.613131	2	562.3103638	0.284874797	0.000160045	75.27381224
11321601	PFKP	ATP-dependent 6-phosphofructokinase, platelet type isoform 1	5214	NP_002618.1	41.96428571	1	23	26	174	784	85.542	7.33	1.08	1.02	1.16	1.09	Significant	0.0014																
																			AIGVLTSGGDAQGMNAAVR	2	11321601	1xTMT6plex [N-Term]; 1xOxidation [M14]			1	2.686554909	51.99	0	2033.064704	2	1017.03009	-5.803886082	-0.005899838	45.07123468
																			AIGVLTSGGDAQGMNAAVR	6	11321601	1xTMT6plex [N-Term]	1.463	1.433	1.286	3.306869507	50.04	0	2017.069789	2	1017.03009	-5.803886082	-0.005899838	45.07123468
																			EIGWTDVGGWTGQGGSILGTK	11	11321601	2xTMT6plex [N-Term; K21]	0.969	1.595	1.173	3.707212687	54.19	0	2577.371323	3	859.7949219	-0.430731494	-0.000370052	88.6937911
																			NVLGHMQQGGAPSPFDR	8	11321601	1xTMT6plex [N-Term]	1.013	1.03		4.288109779	38.56	0	2040.028258	3	680.6795654	-2.017261732	-0.001371757	47.35193304
																			NVIFQPVAELK	13	11321601	2xTMT6plex [N-Term; K11]	1.124	0.553	100	4.777285099	54.01	0	1716.046013	2	858.5266113	-0.039092046	-3.35E-05	91.28071132
																			DLQSNVEHLTEK	8	11321601	2xTMT6plex [N-Term; K12]	1.379	1.254	1.373	4.229029655	59.56	0	1871.027462	3	624.3468628	-0.762800719	-0.00047574	60.20124853
																			EWSGLLEELAR	7	11321601	1xTMT6plex [N-Term]	2.104	1.05	100	3.500356913	52.61	0	1531.831774	2	766.418396	-1.4743119	-0.001129199	95.78391065
																			IIEVVDAIMTTAQSHQR	4	11321601	1xTMT6plex [N-Term]		100	1.466	3.941409349	53.78	0	2141.158604	3	714.3903809	-0.940776583	-0.000671451	106.7956318
																			GITNLCVIGGDGSLTGANLFR	12	11321601	1xTMT6plex [N-Term]; 1xCarbamidomethyl [C6	1.078	1.273	0.814	4.95896244	75.38	0	2364.254295	3	788.7549438	-1.69900803	-0.001338962	92.75215869
																			EKHEEFCVPMVMVPATVSNNVPGSDFSIGADTALNTITDTCDR	2	11321601	2xTMT6plex [N-Term; K2]; 2xCarbamidomethyl [C7; C41]				3.405872822	4.41	1	5183.483416	4	1296.613403	-9.961000086	-0.01290817	95.32059502
																			KNVLGHMQQGGAPSPFDR	2	11321601	2xTMT6plex [N-Term; K1]			0.922	5.523940086	46.33	1	2397.286153	4	600.0721436	-8.096209634	-0.004852233	45.81367877
																			FTTDDSICVLGISK	6	11321601	2xTMT6plex [N-Term; K14]; 1xCarbamidomethyl [C8			100	2.799215555	29.07	0	2014.093099	2	1007.549744	-0.441223553	-0.000444333	81.96818363
																			SFAGNLNTYK	6	11321601	2xTMT6plex [N-Term; K10]	1.092	1.05		4.635148048	51.42	0	1572.878613	2	786.9418335	-1.413407108	-0.001111559	59.29805916
																			ASYDVSDSGQLEHVQPWSV	4	11321601	1xTMT6plex [N-Term]			1.087	3.771356344	15.09	0	2333.12472	3	778.3790894	-0.859638397	-0.000668548	72.70218791
																			KFLEHLSGAGK	8	11321601	3xTMT6plex [N-Term; K1; K11]	0.82	0.808		4.787765503	27.95	1	1874.146679	4	469.2923279	0.428138014	0.000200598	46.26119735
																			MLAIYDGFDGFAK	6	11321601	2xTMT6plex [N-Term; K13]	0.882	0.99	1.865	3.000424862	36.21	0	1906.018478	2	953.5118408	-1.087428794	-0.00103633	94.31634949
																			FLEHLSGAGK	6	11321601	2xTMT6plex [N-Term; K10]	1.264	1.083	1.235	3.330709934	44.74	0	1516.888784	2	758.9490356	1.32533056	0.00100519	53.8589218
																			YLEEIATQMR	6	11321601	1xTMT6plex [N-Term]	1.551	100	0.899	2.792491674	34.36	0	1482.782381	2	741.8948975	0.092745825	6.88E-05	65.15189267
																			ELVVTQLGYDTR	6	11321601	1xTMT6plex [N-Term]	1.512	1.849	1.504	2.889024734	48.06	0	1622.895102	2	811.9506226	-0.698592787	-0.000566871	61.62804728
																			MGIYVGAK	6	11321601	2xTMT6plex [N-Term; K8]	2.115	1.297	2.159	3.461037636	43.8	0	1296.775	2	648.8919067	1.184960045	0.000768313	54.77149996
																			TFVLEVMGR	6	11321601	1xTMT6plex [N-Term]		100	2.397	3.18615365	32.92	0	1280.723409	2	640.8654175	0.116231917	7.44E-05	80.6583597
																			AMEWITAK	12	11321601	2xTMT6plex [N-Term; K8]	0.97	1.282	1.373	3.664726734	49.02	0	1407.807029	2	704.406311	-1.195568464	-0.000841565	67.89400727
																			KFTTDDSICVLGISK	6	11321601	3xTMT6plex [N-Term; K1; K15]; 1xCarbamidomethyl [C9	1.175		0.906	2.546264887	13.28	1	2371.350994	3	791.1238403	2.519001239	0.001991145	70.95327071
																			VTILGHVQR	15	11321601	1xTMT6plex [N-Term]	1.152	1.148	1.288	3.896427155	46.93	0	1251.773471	3	417.9295654	0.53670179	0.000223943	39.82064318
																			LPDDQIPK	3	11321601	2xTMT6plex [N-Term; K8]			0.982	2.953701973	33.7	0	1383.824787	2	692.4094238	-9.550373626	-0.006608022	47.37100971
																			IKELVVTQLGYDTR	2	11321601	2xTMT6plex [N-Term; K2]		0.662		2.527071476	4.81	1	2093.237061	3	698.4157104	-2.141674456	-0.001494344	73.0394768
																			NESCSENYTTDFIYQLYSEEGK	1	11321601	2xTMT6plex [N-Term; K22]; 1xCarbamidomethyl [C4				1.616515756		0	3135.450497	3	1045.81958	-2.01234739	-0.002103205	88.94174441
372466572	KRT8	keratin, type II cytoskeletal 8 isoform 1	3856	NP_001243211.1	55.57729941	1	22	31	352	511	56.573	5.43	0.99	0.96	0.98	0.98	Significant	0.000082																
																			AEAESMYQIK	14	372466572	2xTMT6plex [N-Term; K10]	0.95	0.86	0.959	4.734941483	53.54	0	1627.876565	2	814.442749	1.017820952	0.000828443	52.272465
																			LESGMQNMSIHTK	6	372466572	2xTMT6plex [N-Term; K13]	1.118	1.082	1.138	4.733989716	50.44	0	1934.023974	3	645.3473511	1.823282805	0.001175424	50.11237886
																			ASLEAAIADAEQR	11	372466572	1xTMT6plex [N-Term]	0.827	0.836	1.383	3.505609751	46.2	0	1573.838316	2	787.4234619	0.846070471	0.000665789	71.71015982
																			LKLEAELGNMQGLVEDFK	6	372466572	3xTMT6plex [N-Term; K2; K18]	0.728	100	100	4.426811695	31.77	1	2721.546399	3	907.8530884	-0.619975938	-0.000562431	96.06526693
																			GGLGGGYGGASGMGGITAVTVNQSLLSPLVLEVDPNIQAVR	5	372466572	1xTMT6plex [N-Term]			1.745	8.51291275	69.6	0	4154.211707	4	1039.308228	-0.150960951	-0.000156781	103.493293
																			GGLGGGYGGASGMGGITAVTVNQSLLSPLVLEVDPNIQAVR	4	372466572	1xTMT6plex [N-Term]; 1xOxidation [M13]			0.483	5.543060303	36.04	0	4170.206622	4	1039.308228	-0.150960951	-0.000156781	103.493293
																			LESGMQNMSIHTK	6	372466572	2xTMT6plex [N-Term; K13]; 1xOxidation [M5	1.137	0.997		3.721143007	48.46	0	1950.018889	3	645.3473511	1.823282805	0.001175424	50.11237886
																			LEAELGNMQGLVEDFK	4	372466572	2xTMT6plex [N-Term; K16]	100	100		2.198619843	14.83	0	2251.20444	2	1126.105469	-0.346219111	-0.000389705	96.76309259
																			QLETLGQEK	8	372466572	2xTMT6plex [N-Term; K9]	1.346	1.159	1.423	4.399294376	47.12	0	1503.878279	2	752.4431763	0.529829491	0.0003984	43.87114589
																			LQAEIEGLK	12	372466572	2xTMT6plex [N-Term; K9]	1.373	0.903	0.585	4.262069702	56.75	0	1458.893201	2	729.9500122	-0.310616251	-0.000226578	69.17444547
																			EYQELMNVK	41	372466572	2xTMT6plex [N-Term; K9]	0.687	0.716	0.615	4.131864071	49.21	0	1611.88165	2	806.4436646	-0.991051941	-0.000798729	52.61948067
																			LEGLTDEINFLR	6	372466572	1xTMT6plex [N-Term]		100	0.541	2.640158892	32.98	0	1648.910752	2	824.9580688	-1.146953942	-0.000945612	91.33029209
																			TEMENEFVLIK	4	372466572	2xTMT6plex [N-Term; K11]	100			2.54099369	17.83	0	1811.002493	2	906.0048828	-0.002344006	-2.12E-06	87.34203738
																			SNMDNMFESYINNLR	10	372466572	1xTMT6plex [N-Term]	0.274	0.466		2.313309431	11.59	0	2076.968026	2	1038.98645	-1.156498003	-0.001201005	93.70660838
																			YEELQSLAGK	8	372466572	2xTMT6plex [N-Term; K10]	1.213	100	0.572	3.640323639	36.1	0	1595.904494	2	798.4541626	-2.158816333	-0.001722632	58.52629961
																			DVDEAYMNKVELESR	2	372466572	2xTMT6plex [N-Term; K9]				2.983228445	15.71	1	2256.158218	3	752.7244263	0.22481517	0.000169073	62.19474375
																			AQYEDIANR	10	372466572	1xTMT6plex [N-Term]	0.937	0.973	0.906	3.406784534	44.59	0	1308.674545	2	654.842041	1.727298249	0.001130236	39.59057787
																			EYQELMNVK	24	372466572	2xTMT6plex [N-Term; K9]; 1xOxidation [M6]	0.61	0.666	0.646	2.821333647	41.8	0	1627.876565	2	806.4436646	-0.991051941	-0.000798729	52.61948067
																			AEAESMYQIK	16	372466572	2xTMT6plex [N-Term; K10]; 1xOxidation [M6	0.753	0.959	0.865	2.679849863	43.51	0	1643.87148	2	814.442749	1.017820952	0.000828443	52.272465
																			QLYEEEIR	6	372466572	1xTMT6plex [N-Term]	1.405	1.125	1.179	3.494765997	54.65	0	1308.699697	2	654.8536987	0.32381832	0.00021189	49.15633951
																			KDVDEAYMNK	6	372466572	3xTMT6plex [N-Term; K1; K10]	1.008	0.842	0.879	3.726412296	10.14	1	1900.04531	3	634.0187988	-1.824659447	-0.001155645	41.30673479
																			LALDIEIATYR	8	372466572	1xTMT6plex [N-Term]	3.505			2.008917332	16.81	0	1506.87291	2	753.9402466	0.203218241	0.000153112	85.16699517
																			NKYEDEINK	12	372466572	3xTMT6plex [N-Term; K2; K9]	0.918	0.83	0.912	3.587960243	28.5	1	1840.041939	2	920.526123	1.646768851	0.001515062	36.94344344
																			WSLLQQQK	4	372466572	2xTMT6plex [N-Term; K8]		0.811	1.615	3.43593502	37.49	0	1488.89387	2	744.9501343	-0.589535178	-0.000438878	73.24977514
																			FASFIDK	41	372466572	2xTMT6plex [N-Term; K7]	0.677	0.84	0.69	3.288649321	38.52	0	1285.755645	2	643.3812866	-0.27093625	-0.000174179	75.39418814
																			LLEGEESR	6	372466572	1xTMT6plex [N-Term]	1.498	1.484	1.512	3.487635136	41	0	1161.631283	2	581.3189697	-0.534039401	-0.000310178	40.15125271
																			KLLEGEESR	14	372466572	2xTMT6plex [N-Term; K1]	0.985	0.987	0.995	3.586319447	34.85	1	1518.889178	2	759.9478149	-0.543085828	-0.000412444	38.7728649
																			TKTEISEMNR	5	372466572	2xTMT6plex [N-Term; K2]; 1xOxidation [M8]	0.76	0.749		1.940837979	15.34	1	1682.914741	3	561.6427612	-0.600653479	-0.00033695	28.47368186
																			SNMDNMFESYINNLRR	2	372466572	1xTMT6plex [N-Term]	0.677			1.820988894	11.08	1	2233.069137	3	745.0274658	-0.578812828	-0.000430843	95.47611688
																			FLEQQNK	19	372466572	2xTMT6plex [N-Term; K7]	0.833	0.763	0.782	2.620012999	32.3	0	1364.793821	2	682.9000854	-0.679261254	-0.000463526	39.10866059
																			YEDEINKR	6	372466572	2xTMT6plex [N-Term; K7]	1.059	1.134	1.172	3.56978035	27.49	1	1524.842228	3	508.9517822	-0.940779502	-0.00047818	33.28155655
																			ISSSSFSR	2	372466572	1xTMT6plex [N-Term]		0.594		2.338689089	16.3	0	1099.594504	2	550.3001709	-1.308330589	-0.000719317	40.51034848
																			YEDEINK	4	372466572	2xTMT6plex [N-Term; K7]	1.202		0.999	3.350680113	32.52	0	1368.741117	2	684.8745117	0.46013632	0.000314904	42.23766616
																			TEISEMNR	2	372466572	1xTMT6plex [N-Term]			0.797	2.316627264	17.93	0	1208.614253	2	604.8109131	0.245431389	0.000148316	35.86927433
																			LSELEAALQR	2	372466572	1xTMT6plex [N-Term]			0.666	2.303794146	24.18	0	1358.784095	2	679.8966675	1.444659931	0.00098149	85.22516519
157419138	LAMC2	laminin subunit gamma-2 isoform a precursor	3918	NP_005553.2	30.25984912	1	28	28	214	1193	130.892	6.19	0.76	0.83	0.8	0.8	Significant	0.00065																
																			ALHEGVGSGSGSPDGAVVQGLVEK	8	157419138	2xTMT6plex [N-Term; K24]	0.766	0.551	0.636	6.634297371	43.72	0	2708.461928	3	903.493103	1.043954874	0.000942504	59.58998133
																			EATQAEIEADR	16	157419138	1xTMT6plex [N-Term]	0.713	0.845	0.733	4.596095085	73.21	0	1461.738267	2	731.3733521	0.793720455	0.000580106	33.66010661
																			LAESHVESASNMEQLTR	10	157419138	1xTMT6plex [N-Term]	0.697	0.756	0.463	5.118054867	54.98	0	2131.065097	2	1066.035889	-0.279886452	-0.000298228	43.76241619
																			LAESHVESASNMEQLTR	9	157419138	1xTMT6plex [N-Term]; 1xOxidation [M12]	0.706	0.794	1.091	4.293376446	48.72	0	2147.060012	2	1066.035889	-0.279886452	-0.000298228	43.76241619
																			ELEFDTNMDAVQMVITEAQK	8	157419138	2xTMT6plex [N-Term; K20]	0.584			3.362851381	38.05	0	2770.404338	3	924.1394043	-0.244996503	-0.000226246	103.4142623
																			AQGGDGVVPDTELEGR	10	157419138	1xTMT6plex [N-Term]	0.939	0.784	0.829	4.59032917	59.67	0	1828.923836	2	914.9645996	-1.046200619	-0.000956711	40.45186837
																			HPSAHDVILEGAGLR	8	157419138	1xTMT6plex [N-Term]	0.743	0.83	0.94	4.479968071	29.01	0	1800.991797	3	601.0018921	-0.37397198	-0.000224507	48.89101581

Bhat et al,2017. Cigarette smoke and chewing tobacco alter expression of different sets of miRNAs in oral keratinocytes

Supplementary Table S2(A). List of all proteins and peptides identified in OKF6/TERT1 cells chronically treated with chewing tobacco using Sequest and Mascot search algorithms

Accession	Gene_Symbol	Description	Gene_ID	Refseq_Accession	Coverage	Proteins	#Unique_Peptides	#Peptides	#PSMS	#AAs	MW [kDa]	calc. pI	OKF6-STE-6M/OKF6P_R1	OKF6-STE-6M/OKF6P_R2	OKF6-STE-6M/OKF6P_R3	Median fold change	Status	P-value (6M)	Sequence	#PSMSs	Protein Accessions	Modifications	OKF6-STE-6M/OKF6P_R1	OKF6-STE-6M/OKF6P_R2	OKF6-STE-6M/OKF6P_R3	Xcorr	Ionscore	# Missed Cleavages	Theo. MH+ [Da]	Charge	m_z	DeltaM [ppm]	Deltam/z [Da]	RT [min]
																			SDGTCVCKPGFGGPNCEHGAFSCPACYNQVK	2	157419138	3xTMT6plex [N-Term; K8; K31]; 5xCarbamidomethyl [C5; C7; C16; C23; C26]		0.979		5.571975708	5.78	0	4148.911242	4	1037.980225	-2.934100488	-0.003043331	57.13337524
																			CIHNTAGIYCDQCK	6	157419138	2xTMT6plex [N-Term; K14]; 3xCarbamidomethyl [C1; C10; C13	0.751	0.76	0.79	5.994043827	49.33	0	2198.055685	3	733.3566284	-0.160763031	-0.000117789	35.64511377
																			ALGSAAADAQR	10	157419138	1xTMT6plex [N-Term]	0.871	0.886	0.686	3.871039152	72.79	0	1259.690529	2	630.3483276	-0.913491477	-0.000575358	32.23893068
																			DAQISEGASR	18	157419138	1xTMT6plex [N-Term]	0.981	0.751	0.944	3.839276791	45.61	0	1262.653809	2	631.8309937	0.713738541	0.000450602	22.05973118
																			LDPVYFVAPAK	10	157419138	2xTMT6plex [N-Term; K11]	1.094	1.401	100	2.578656912	30.67	0	1677.998	2	839.5020752	-0.671377066	-0.000563285	77.88053496
																			GHLHLLETSIDGILADVK	5	157419138	2xTMT6plex [N-Term; K18]	0.01			3.226207256	16.66	0	2389.385516	3	797.133667	0.3898896	0.000310532	100.6188056
																			GQFCQDCASGYK	6	157419138	2xTMT6plex [N-Term; K12]; 2xCarbamidomethyl [C4; C7	0.564	0.955	0.986	4.376398087	46.46	0	1878.887875	2	939.9489136	1.424096921	0.001337859	38.98746869
																			IQMDQFMQQLQR	2	157419138	1xTMT6plex [N-Term]		0.521		3.433360338	29.05	0	1794.919225	2	897.9624634	-0.877556029	-0.000787571	85.57100237
																			SSAEYSVHK	8	157419138	2xTMT6plex [N-Term; K9]	1.049	1.001	0.974	3.513200283	28.77	0	1465.805114	3	489.2721252	-2.245685696	-0.001097246	31.74440629
																			KAEAEEAMK	12	157419138	3xTMT6plex [N-Term; K1; K9]	0.741	0.788	0.767	4.499897003	41.33	1	1693.976168	2	847.4910889	-0.747918211	-0.000633478	29.62787462
																			EFDLQVDNR	4	157419138	1xTMT6plex [N-Term]	0.751	0.658		3.606615543	47.11	0	1364.70076	2	682.8532104	-1.183689209	-0.000807691	47.69659999
																			ITAPLMPLGK	9	157419138	2xTMT6plex [N-Term; K10]	0.977		100	3.593619823	35.15	0	1498.943128	2	749.9754639	0.348928765	0.000261512	71.73079873
																			SLGLQLAK	4	157419138	2xTMT6plex [N-Term; K8]	100	0.549		2.830124855	35.98	0	1287.840043	2	644.4235229	-0.212760555	-0.000137001	53.2980203
																			EVEGELER	14	157419138	1xTMT6plex [N-Term]	0.705	0.839	0.691	3.134763956	43.15	0	1189.626198	2	595.3174438	1.188007893	0.000706643	23.92261424
																			MEALISK	6	157419138	2xTMT6plex [N-Term; K7]	0.99	100	0.832	2.650589943	35.29	0	1249.759016	2	625.3833618	0.345036769	0.000215606	67.13538313
																			TLPCGLTK	2	157419138	2xTMT6plex [N-Term; K8]; 1xCarbamidomethyl [C4		1.031		3.110946655	40	0	1347.807029	2	674.4066772	-0.705603838	-0.000475509	56.10242276
																			KAEAEEAMK	6	157419138	3xTMT6plex [N-Term; K1; K9]; 1xOxidation [M8	0.682	0.84	0.767	2.826029062	24.77	1	1709.971083	2	847.4910889	-0.747918211	-0.000633478	29.62787462
																			LQGVSDQSFQVEEAK	3	157419138	2xTMT6plex [N-Term; K15]				1.529310822		0	2123.138469	2	1062.073242	0.347845895	0.000369262	52.81746708
																			LSYISQK	6	157419138	2xTMT6plex [N-Term; K7]	2.69	0.694	0.682	3.141523123	37.16	0	1296.792759	2	648.8996582	-0.554401422	-0.000359472	47.99941
																			ADSLSSLVTR	2	157419138	1xTMT6plex [N-Term]			100	1.90486443	22.69	0	1277.726246	2	639.3671265	0.571452367	0.00036508	63.56335953
																			LNEHPSNNWSPQLSYFEYR	5	157419138	1xTMT6plex [N-Term]	100		100	2.203936815	5.6	0	2610.257466	3	870.7568359	-0.579003228	-0.000503782	65.70551642
																			ETEDYSK	4	157419138	2xTMT6plex [N-Term; K7]	0.769	0.644		3.063319206	38.89	0	1329.693832	2	665.3502197	-0.503594781	-0.000334813	34.44472195
																			ITSTFHQDVDGWK	1	157419138	2xTMT6plex [N-Term; K13]	100			1.812754512		0	1992.059097	3	664.692627	2.123817201	0.001410256	46.0091387
47519639	MAP4	microtubule-associated protein 4 isoform 1	4134	NP_002366.2	37.84722222	1	33	33	158	1152	120.93	5.43	0.91	0.85	0.96	0.91	Significant	0.0012																
																			GISEDSHLESLQDVGQSAAPTFMISPETVTGTGK	4	47519639	2xTMT6plex [N-Term; K34]				3.598942518	17.55	0	3947.994847	4	987.7543945	0.228266509	0.000225299	75.16441665
																			TTTAAAVASTGPSSR	4	47519639	1xTMT6plex [N-Term]	1.078	1.285		5.274697781	50.98	0	1606.859779	2	803.9335327	0.005905735	4.74E-06	32.91047296
																			DFIATLEAEAFDDVVGETVGK	23	47519639	2xTMT6plex [N-Term; K21]	1.65	0.622	100	4.188511848	49.57	0	2684.407099	3	895.4767456	3.198012109	0.002861589	113.4978464
																			LATNTSAPDLK	2	47519639	2xTMT6plex [N-Term; K11]			1.106	4.34843111	60.77	0	1588.931043	2	794.9697876	0.790245315	0.000627823	50.97264082
																			QPAPTTIGGLNK	2	47519639	2xTMT6plex [N-Term; K12]		0.921		4.119069099	62.19	0	1654.989227	2	827.9979858	-0.321120104	-0.000265725	51.42903101
																			TTTLSGTAPAAGVVPSR	4	47519639	1xTMT6plex [N-Term]		1.332	1.678	5.125453472	73.71	0	1815.017343	2	908.0128174	0.559568	0.000507813	43.08116652
																			WPTETDVSSAK	4	47519639	2xTMT6plex [N-Term; K11]	0.867	0.93		4.107738495	67.88	0	1678.905222	2	839.9552612	-1.17709388	-0.000988115	52.7418409
																			VGSLDNVGHLPAGGAVK	2	47519639	2xTMT6plex [N-Term; K17]				3.057774305	34.32	0	2049.185694	3	683.7340698	0.957420629	0.000653978	49.2824867
																			DMQPSMESDMALVK	6	47519639	2xTMT6plex [N-Term; K14]	0.604	1.812		3.219146729	45.77	0	2040.02159	2	1020.511963	-2.422140419	-0.002470609	72.1252231
																			TAGPIASAQK	2	47519639	2xTMT6plex [N-Term; K10]			0.902	4.211065769	59.62	0	1401.846585	2	701.4273682	0.623868642	0.000437284	37.48095536
																			KPMSLASGLVPAAPPK	10	47519639	3xTMT6plex [N-Term; K1; K16]	100	1.642	0.81	3.085367441	18.55	0	2251.381506	3	751.1304932	-2.034376581	-0.001526719	62.67131873
																			EIEMASEERPPAQALEIMMGLK	4	47519639	2xTMT6plex [N-Term; K22]				2.508090496	8.6	0	2931.539392	3	977.850708	-0.621269688	-0.000607092	89.70724213
																			EAQTLDSQIQETSI	6	47519639	1xTMT6plex [N-Term]	100	100	1.798	2.760778189	47.56	0	1791.917354	2	896.4612427	-1.196996297	-0.001072459	57.67432917
																			DMAQLPETEIAPAK	4	47519639	2xTMT6plex [N-Term; K14]	0.851			2.331669092	30.43	0	1972.082534	2	986.5462646	1.378389001	0.001359148	62.88591619
																			ATPMPSRPSTTPFIDK	6	47519639	2xTMT6plex [N-Term; K16]	0.702	0.762	0.73	4.250996113	18.71	0	2204.214945	3	735.4096069	-0.307551321	-0.00022597	49.07622996
																			KPESNAVTK	6	47519639	3xTMT6plex [N-Term; K1; K9]	0.901	0.859	0.917	3.506406784	27.2	0	1661.020082	3	554.3453979	0.938458044	0.000519599	30.30604971
																			ASPSKPASAPASR	5	47519639	2xTMT6plex [N-Term; K5]	0.835	0.771	0.825	2.456891537	6.59	0	1684.974639	3	562.3302612	0.944460154	0.000530464	25.25482649
																			DMEIAQTQK	4	47519639	2xTMT6plex [N-Term; K9]	1.39	1.249		4.243049145	44.72	0	1521.8347	2	761.4191895	-2.363905719	-0.001798737	48.26017127
																			GISEDSHLESLQDVGQSAAPTFMISPETVTGTGK	2	47519639	2xTMT6plex [N-Term; K34]; 1xOxidation [M23			100	2.277569532	1.86	0	3963.989762	4	987.7543945	0.228266509	0.000225299	75.16441665
																			DVPPLSETEATPVPIKDMEIAQTQK	4	47519639	3xTMT6plex [N-Term; K16; K25]	100			1.814306259	1.67	1	3424.885233	3	1142.298828	-0.964214791	-0.001100775	67.94065067
																			DMTLPPETNVILTK	4	47519639	2xTMT6plex [N-Term; K14]				1.770143151	7.4	0	2030.160785	2	1015.582336	-1.669054226	-0.001694224	78.49640111
																			ETEMALAK	2	47519639	2xTMT6plex [N-Term; K8]	1.178			3.222363949	39.34	0	1350.770309	2	675.8887939	0.001910484	1.29E-06	52.88848342
																			KPTSAKPSSTTPR	2	47519639	3xTMT6plex [N-Term; K1; K6]	1.107	0.838		1.714425206		0	2045.2322	4	512.0630493	-0.895866188	-0.000458064	25.76384591
																			DMELPTEK	2	47519639	2xTMT6plex [N-Term; K8]	1.046			3.858893156	36.43	0	1420.775788	2	710.8915405	0.011602596	8.24E-06	57.2136934
																			TQPTSLPK	4	47519639	2xTMT6plex [N-Term; K8]	1.085	0.865		2.961080313	41.19	0	1329.814222	2	665.4102173	-0.800464959	-0.000532235	42.72321546
																			DMSPLSETEMALGK	8	47519639	2xTMT6plex [N-Term; K14]				1.699096918	6.88	0	1967.022971	2	984.0144043	-0.731367285	-0.000719308	71.73415122
																			SVPADLSRPK	6	47519639	2xTMT6plex [N-Term; K10]	0.716	0.638		4.083506584	30.31	0	1527.925898	3	509.9797668	-0.753147055	-0.000383584	39.30429536
																			TDYIPLLDVDEK	8	47519639	2xTMT6plex [N-Term; K12]		100	100	1.311766148	4.89	0	1879.046466	2	940.0258179	-1.12149851	-0.001053674	83.70119287
																			NVVLPTETEVAPAK	4	47519639	2xTMT6plex [N-Term; K14]				1.63346386	6.86	0	1926.131199	2	963.5687866	-0.468565076	-0.000451259	52.3780378
																			DVTPPPETEVVLIK	3	47519639	2xTMT6plex [N-Term; K14]				1.501614809	0.78	0	1995.177815	2	998.0910034	-1.546094809	-0.001542367	77.05710112
																			DVAPSTVK	2	47519639	2xTMT6plex [N-Term; K8]	0.855			2.972631216	34.68	0	1274.772023	2	637.8905029	1.338254483	0.000852985	35.85578782
																			SPSTLLPK	3	47519639	2xTMT6plex [N-Term; K8]	0.648		0.862	2.807126522	21.74	0	1300.824059	2	650.9159546	0.441035576	0.000286855	54.06533779
																			STQTVAK	2	47519639	2xTMT6plex [N-Term; K7]	100			2.557650089	27.98	0	1192.730158	2	596.8682861	-0.723419601	-0.000431422	31.57579791
																			DMALATK	4	47519639	2xTMT6plex [N-Term; K7]	0.835	0.895		2.335586071	40.51	0	1207.712066	2	604.3599854	0.520374966	0.000314232	48.89401933
4505763	PGK1	phosphoglycerate kinase 1	5230	NP_000282.1	60.91127098	1	19	23	201	417	44.586	8.1	0.79	0.83	0.78	0.8	Significant	0.00036																
																			AHSSMVGVNLPQK	16	4505763	2xTMT6plex [N-Term; K13]	0.847	0.822	0.707	5.146076679	60.29	0	1826.035859	2	913.5223389	0.844471026	0.000771017	44.03779052
																			AHSSMVGVNLPQK	2	4505763	2xTMT6plex [N-Term; K13]; 1xOxidation [M5		0.813		4.562518597	32.95	0	1842.030774	2	913.5223389	0.844471026	0.000771017	44.03779052
																			ALESPERPFLAILGGAK	10	4505763	2xTMT6plex [N-Term; K17]	0.986		0.788	4.077908993	30.89	0	2227.321459	3	743.112854	1.144572872	0.000849777	94.57647299
																			ACANPAAGSVILLENLR	12	4505763	1xTMT6plex [N-Term]; 1xCarbamidomethyl [C2	0.826	0.875		5.128544331	83.83	0	1998.100361	3	666.7039795	-1.489261248	-0.000991898	88.6864657
																			ITLPVDFVTADK	6	4505763	2xTMT6plex [N-Term; K12]	0.861	1.237	100	3.295405626	67.11	0	1777.051158	2	889.027832	-1.559081452	-0.001385284	92.69944246
																			VLNNMEIGTSLFDEEGAK	2	4505763	2xTMT6plex [N-Term; K18]; 1xOxidation [M5	1.154			4.254588604	46	0	2441.263412	3	809.0940552	-0.364789572	-0.000294904	89.59542812
																			VLNNMEIGTSLFDEEGAK	9	4505763	2xTMT6plex [N-Term; K18]		0.91	0.987	3.586721182	51.32	0	2425.268497	3	809.0940552	-0.364789572	-0.000294904	89.59542812
																			GCITIIGGGDTATCCAK	10	4505763	2xTMT6plex [N-Term; K17]; 3xCarbamidomethyl [C2; C14; C15	0.802	0.651	0.697	5.172567368	81.55	0	2213.112866	3	738.375	-1.093071229	-0.000806363	55.77800754
																			SVVLMSHLGRPDGVPMPDK	7	4505763	2xTMT6plex [N-Term; K19]	1.066	0.584	1.133	3.816208124	23.51	0	2493.372191	3	831.7947388	-1.013884495	-0.000842664	62.67240003
																			SVVLMSHLGRPDGVPMPDKYSLEPVAVELK	2	4505763	3xTMT6plex [N-Term; K19; K30]		0.52		4.292109489	16.77	1	3951.205462	5	791.0463257	-0.744102543	-0.00058802	80.02353781
																			YSLEPVAVELK	10	4505763	2xTMT6plex [N-Term; K11]	1.442	1.38	0.904	4.454042435	46.36	0	1706.014044	2	853.5112915	0.739810916	0.000631064	79.73405122
																			LGDVYVNDAFGTAHR	8	4505763	1xTMT6plex [N-Term]	1.085	1.506	1.088	2.689470053	45.39	0	1863.955077	3	621.9897461	-0.210100959	-0.00013054	54.15234591
																			VSHVSTGGGASLELLEGK	2	4505763	2xTMT6plex [N-Term; K18]				2.696338892	18.62	0	2199.238518	3	733.7507935	-0.313958173	-0.000230156	69.09001014
																			IQLINNMLDK	6	4505763	2xTMT6plex [N-Term; K10]	0.738	0.595	1.296	2.849335194	31.33	0	1659.986784	2	830.4962158	-0.981139965	-0.00081434	85.31110815
																			VLPGVDALSNI	12	4505763	1xTMT6plex [N-Term]	0.751	0.769	0.717	2.495146751	52.93	0	1326.783033	2	663.895874	1.084327158	0.000719333	85.20708143
																			DCVGPEVEK	11	4505763	2xTMT6plex [N-Term; K9]; 1xCarbamidomethyl [C2	0.85	0.795	0.853	4.08728075	36.71	0	1490.792501	2	745.8999634	0.100092945	7.46E-05	46.41326157
																			FHVEEEGK	20	4505763	2xTMT6plex [N-Term; K8]	0.81	0.844	0.818	3.318857431	38.03	0	1432.783651	2	716.8949585	-0.705031644	-0.000505079	36.93286401
																			KELNYFAK	9	4505763	3xTMT6plex [N-Term; K1; K8]	0.64	0.692	0.736	4.12903738	31.47	1	1700.035004	3	567.3491821	-1.182618756	-0.000670164	48.17199312
																			VDFNVPMK	8	4505763	2xTMT6plex [N-Term; K8]	0.83	1.012	1.723	2.499948978	22.86	0	1407.807029	2	704.4065552	-0.848730269	-0.000597424	57.96455976
																			ELNYFAK	8	4505763	2xTMT6plex [N-Term; K7]	0.965	0.862	0.821	3.442087173	31.24	0	1342.777109	2	671.8917236	-0.698570425	-0.000469012	54.9566895
																			KYAEAVTR	4	4505763	2xTMT6plex [N-Term; K1]		0.757	0.724	2.918698072	11.99	1	1395.83602	3	465.949585	-1.303052193	-0.000606282	29.13319987
																			ALMDEVVK	4	4505763	2xTMT6plex [N-Term; K8]; 1xOxidation [M3]	0.733		0.68	2.422395468	31.94	0	1378.801609	2	689.9047852	0.496432914	0.000342241	48.19782136
																			LTLDKLDVK	6	4505763	3xTMT6plex [N-Term; K5; K9]	0.44	0.01		2.440541029	13.31	1	1732.118733	2	866.5627441	-0.301092956	-0.000260764	72.59953558
																			QIVWNGPVGVFEWEAFAR	5	4505763	1xTMT6plex [N-Term]	1.241	0.725		1.361490965	2.25	0	2334.223253	2	1167.613647	-1.385714979	-0.001617284	115.2600452
																			VDFNVPMK	8	4505763	2xTMT6plex [N-Term; K8]; 1xOxidation [M7]	0.43		0.291	1.642998815	19.1	0	1423.801944	2	704.4065552	-0.848730269	-0.000597424	57.96455976
																			YAEAVTR	4	4505763	1xTMT6plex [N-Term]	0.849	0.767		2.455535412	26.78	0	1038.578126	2	519.7927856	0.162865998	8.46E-05	29.24112163
167466173	HSPA1B	heat shock 70 kDa protein 1B	3304	NP_005337.2	58.97035881	1	12	27	243	641	70.009	5.66	0.78	0.8	0.78	0.79	Significant	0.000072																
																			HWPFQVINDGDKPK	7	167466173	3xTMT6plex [N-Term; K12; K14]	0.744	0.582		4.894096851	23.71	0	2368.338062	4	592.8397827	-0.321174851	-0.000190163	61.17611875
																			SINPDEAVAYGAAVQAAILMGDK	17	167466173	2xTMT6plex [N-Term; K23]	1.141	100	1.246	4.268444061	46.61	0	2762.479887	3	921.4978638	-0.307166546	-0.000282847	103.8219879
																			KFGDPVVQSDMK	12	167466173	3xTMT6plex [N-Term; K1; K12]	0.862	0.791	0.825	4.354756832	42.15	1	2038.161009	3	680.0578613	-0.970411865	-0.000659285	46.45603367
																			EIAEAYLGYPVTNAVITVPAYFNDSQR	8	167466173	1xTMT6plex [N-Term]		100		2.873129845	22.68	0	3230.65711	3	1077.557251	0.027885871	3.00E-05	101.2171436
																			ATAGDTHLGGEDFDNR	12	167466173	1xTMT6plex [N-Term]	0.833	0.876	0.851	4.630966663	42.42	0	1904.893598	3	635.637146	1.725155822	0.001095413	25.28270071
																			AQIHDLVLVGGSTR	22	167466173	1xTMT6plex [N-Term]	0.669	0.727	0.71	4.355434895	44.35	0	1694.975084	3	565.657959	-9.298176135	-0.005253392	48.28515852
																			DAGVIAGLNVLR	10	167466173	1xTMT6plex [N-Term]	0.861	0.989		4.103200912	66.89	0	1426.857929	2	713.932312	-0.407606497	-0.000290798	81.84942328
																			AAAIGIDLGTTYSCVGVFQHGK	5	167466173	2xTMT6plex [N-Term; K22]; 1xCarbamidomethyl [C14			0.601	2.371686697	21.4	0	2723.459092	3	908.4904175	-0.878577028	-0.00079759	72.23880389
																			ELEQVCNPIISGLYQGAGGPGPGGFGAQGPK	14	167466173	2xTMT6plex [N-Term; K31]; 1xCarbamidomethyl [C6	1.324	1.098	0.947	4.078817844	31.31	0	3513.820059	4	879.2102051	-0.304007457	-0.000267057	87.95289896
																			VEILANDQGNR	48	167466173	1xTMT6plex [N-Term]	1.325	1.311	1.289	4.071812153	54.4	0	1457.790972	2	729.3981323	-1.360662537	-0.000991781	36.49181231
																			TTPSYVAFTDTER	12	167466173	1xTMT6plex [N-Term]	1.291	1.404		4.477664471	71.19	0	1716.864196	2	858.9349365	-0.931671316	-0.000799777	51.60226222
																			CQEVISWLDANTLAEKDEFEHK	2	167466173	3xTMT6plex [N-Term; K16; K22]; 1xCarbamidomethyl [C1	100			1.726743579	8.22	1	3349.734153	4	838.1873779	-1.931822334	-0.001617773	84.99288223
																			GGSGSGPTIEEVD	2	167466173	1xTMT6plex [N-Term]		1.07		2.797777414	51.07	0	1433.695734	2	717.3513184	-0.260621024	-0.000186826	45.44502049
																			SAVEDEGLK	6	167466173	2xTMT6plex [N-Term; K9]	0.86	0.781	0.858	4.60304594	51.89	0	1405.793881	2	703.3964233	-5.911791498	-0.00415538	38.16214034
																			SINPDEAVAYGAAVQAAILMGDK	13	167466173	2xTMT6plex [N-Term; K23]; 1xOxidation [M20			1.826	2.541149616	26.48	0	2778.474802	3	921.4978638	-0.307166546	-0.000282847	103.8219879
																			FEELCSDLFR	8	167466173	1xTMT6plex [N-Term]; 1xCarbamidomethyl [C5	1.094	100	100	2.418096066	26.37	0	1544.761646	2	772.8834839	-1.265228566	-0.000977238	79.20910163
																			LVNHFVEEFK	12	167466173	2xTMT6plex [N-Term; K10]	1.088	1.041	0.841	2.882869244	26.65	0	1719.983413	3	573.9990234	-0.520829151	-0.000298606	63.51970602
																			AFYPEEISSMVLTK	10	167466173	2xTMT6plex [N-Term; K14]	0.866	0.93	0.752	3.058972836	26.75	0	2073.134236	2	1037.070313	-0.427955887	-0.000443605	87.71425392
																			GVPQIEVTFDIDANGILNVTATDK	2	167466173	2xTMT6plex [N-Term; K24]			100	2.650573015	23.83	0	2988.629387	3	996.8739014	-7.440396769	-0.007412196	115.3000616
																			YKAEDEVQR	14	167466173	2xTMT6plex [N-Term; K2]	0.724	0.793	0.743	2.897931337	15.46	1	1595.879342	2	798.4438477	0.674845818	0.000538486	32.73629936
																			NALESYAFNMK	2	167466173	2xTMT6plex [N-Term; K11]		0.01		1.596866608	6.25	0	1745.929663	2	873.4682007	-0.308129717	-0.000268986	63.63010502
																			STLEPVEK	2	167466173	2xTMT6plex [N-Term; K8]	1.034			4.034611225	32.33	0	1360.808803	2	680.9086304	0.868213216	0.000590736	41.45426294
																			LIGDAAK	8	167466173	2xTMT6plex [N-Term; K7]	1.088	1.128	0.991	2.640163898	25.21	0	1145.72943	2	573.368042	-0.543667299	-0.000311448	38.40743522
																			ITITNDK	9	167466173	2xTMT6plex [N-Term; K7]	1.307	1.284	1.082	3.007526398	39.15	0	1262.772023	2	631.8894043	-0.389061716	-0.000245648	34.74030574
																			MVQEAEK	5	167466173	2xTMT6plex [N-Term; K7]	1.286	1.351	1.238	3.254307032	41.37	0	1292.728444	2	646.868042	0.281152919	0.000181727	35.4466632
																			SENVQDLLLLDVAPLSLGLETAGGVMTALIK	1	167466173	2xTMT6plex [N-Term; K31]				1.533399105		0	3639.069456	3	1213.692749	-1.583476149	-0.001920793	114.6571695
																			LVNHFVEEFKR	2	167466173	2xTMT6plex [N-Term; K10]		0.531		1.542152643	6.89	1	1876.084524	4	469.7765198	-0.146378499	-6.87E-05	57.33801059
																			AEDEVQR	2	167466173	1xTMT6plex [N-Term]			0.728	2.401926279	22.94	0	1075.558118	2	538.2827148	0.032334375	1.74E-05	23.02407717
740086846	ACLY	ATP-citrate synthase isoform 3	47	NP_001290203.1	37.66233766	1	37	37	180	1155	126.138	8.18	1.5	1.57	1.6	1.56	Significant	0.00036																
																			TTDGVYEGVAIGGDR	8	740086846	1xTMT6plex [N-Term]	1.827	1.019	2.067	5.557625771	49.69	0	1738.880909	2	869.9442139	0.139442773	0.000121237	51.22465415
																			KPASFMTSICDER	12	740086846	2xTMT6plex [N-Term; K1]; 1xCarbamidomethyl [C10	1.055	1.056	4.624	4.905889988	31.72	0	2000.034539	3	667.3498535	0.234309391	0.000156209	62.35034232
																			DLVSSLTSGLLTIGDR	4	740086846	1xTMT6plex [N-Term]	100	1.122		3.10816431	49.2	0	1876.058873	2	938.5325317	-0.578912236	-0.000543037	105.1914701
																			LLQDHPWLLSQNLVVKPDQLIK	6	740086846	3xTMT6plex [N-Term; K16; K22]	100	100	100	2.590606451	13.23	0	3284.970165	4	821.9969482	-1.279056137	-0.001050415	84.65546434
																			FICTTSAIQNR	4	740086846	1xTMT6plex [N-Term]; 1xCarbamidomethyl [C3	1.959	1.889		4.551499844	68.22	0	1539.815078	2	770.409729	-1.881259788	-0.001448396	50.02925166
																			LYRPGSVAYVSR	4	740086846	1xTMT6plex [N-Term]	1.064	1.198		4.59493494	30.75	0	1596.905942	3	532.9729004	-1.123333615	-0.000597953	41.49580981
																			GVTIIGPATVGGIKPGCFK	10	740086846	3xTMT6plex [N-Term; K14; K19]; 1xCarbamidomethyl [C17	100	100	100	2.220325232	19.87	0	2559.529962	3	853.8484497	0.325904016	0.000278054	64.89890723
																			SMGFIGHYLDQK	10	740086846	2xTMT6plex [N-Term; K12]	1.1	1.037	0.803	2.695451498	24.64	0	1853.998411	3	618.670166	-1.330186213	-0.000822054	69.82359978
																			FGGALDAAAK	2	740086846	2xTMT6plex [N-Term; K10]			1.836	3.883669853	40.23	0	1378.809471	2	689.9077148	-0.956131015	-0.000659161	49.33681882
																			VDATADYICK	6	740086846	2xTMT6plex [N-Term; K10]; 1xCarbamidomethyl [C9	100	2.733	2.3	4.431346416	40.1	0	1613.860915	2	807.4351807	1.344550887	0.001084959	51.71417841
																			DYQGPLKEHEVTIFVR	4	740086846	2xTMT6plex [N-Term; K7]		1.248	0.707	3.974735737	7.52	1	2389.328001	4	598.0874634	0.009352263	5.59E-06	52.70681131
																			LGQEATVGK	2	740086846	2xTMT6plex [N-Term; K9]	2.513			3.371781349	30.16	0	1360.820036	2	680.9140625	0.596941534	0.000406165	46.9877904
																			AIVWGMQTR	4	740086846	1xTMT6plex [N-Term]	1.413	1.365		3.295552731	36.43	0	1290.718993	2	645.862793	-0.529551749	-0.000341751	61.65305699
																			EHEVTIFVR	8	740086846	1xTMT6plex [N-Term]		2.238	1.98	3.80507803	43.41	0	1358.762966	2	679.8842163	-1.332125512	-0.000905021	42.79019484
																			YPGSTFMDHVLR	6	740086846	1xTMT6plex [N-Term]	100	100	100	2.853538036	26.11	0	1651.846378	3	551.2866211	-0.646524256	-0.000355986	58.1587923
																			AFDSGIIPMEFVNK	4	740086846	2xTMT6plex [N-Term; K14]	100			1.90971899	29.85	0	2026.108355	2	1013.556458	-1.340906043	-0.00135841	87.16964943
																			DEPSVAAMVYPFTGDHK	6	740086846	2xTMT6plex [N-Term; K17]				1.865260839	13.26	0	2322.184039	3	774.7332153	0.453687596	0.000351182	66.22056152
																			LLVGVDEK	6	740086846	2xTMT6plex [N-Term; K8]	1.422	2.125	1.556	3.603980303	42.54	0	1330.834624	2	665.9208984	-0.07758665	-5.16E-05	52.43139196
																			EAYPEEAYIADLDAK	4	740086846	2xTMT6plex [N-Term; K15]	100	1.66		3.129839659	33.35	0	2156.116337	2	1078.562134	0.303396488	0.000327079	80.10763394
																			QHFPATPLLDYALEVEK	4	740086846	2xTMT6plex [N-Term; K17]		100	100	1.672551513	5.36	0	2429.348068	3	810.4539185	-0.356428507	-0.00028863	97.17817744
																			SAYDSTMETMNYAQIR	2	740086846	1xTMT6plex [N-Term]				1.638226151	11.85	0	2109.978256	2	1055.490356	-2.284288647	-0.0024099	56.81868021
																			AISEQTGK	6	740086846	2xTMT6plex [N-Term; K8]	1.373	1.666	1.613	3.44276619	48.14	0	1291.762187	2	646.3853149	0.90297024	0.000583211	30.82255
																			TASFSESR	6	740086846	1xTMT6plex [N-Term]	1.479	1.638	2.346	3.825709581	35.84	0	1113.573768	2	557.2896729	-1.525958067	-0.000849633	29.81900035
																			EILIPVFK	8	740086846	2xTMT6plex [N-Term; K8]	1.846	1.628	1.754	3.204838037	30.8	0	1416.923045	2	708.9645996	-0.791864635	-0.000561006	90.91909122
																			YQDTPGVK	2	740086846	2xTMT6plex [N-Term; K8]	1.869			3.591222286	42.72	0	1365.777837	2	683.3922119	-0.504981013	-0.000344846	39.50905684
																			AVQGMLDFDYVCSR	3	740086846	1xTMT6plex [N-Term]; 1xCarbamidomethyl [C12				1.592392206	6.23	0	1889.90872	2	945.4571533	-0.894434401	-0.0008452	74.91244766
																			LTLLNPK	6	740086846	2xTMT6plex [N-Term; K7]	0.823	3.932	100	2.666124344	34.79	0	1256.83423	2	628.9199829	-1.225658604	-0.000770225	60.25765752
																			EAGVFVPR	6	740086846	1xTMT6plex [N-Term]	1.61	0.576	0.698	2.91800189	36.93	0	1103.64106	2	552.3240967	-0.129925054	-7.17E-05	43.93559682
																			TIAIIAEGIPEALTR	8	740086846	1xTMT6plex [N-Term]	100			1.831507802	11.76	0	1797.068315	2	899.0366821	-1.239720365	-0.001113931	92.38499888
																			LIMGIGHR	2	740086846	1xTMT6plex [N-Term]	0.981			3.076108217	26.59	0	1125.6764	2	563.3414307	-0.724099639	-0.000407551	55.81359887
																			EEMGIGGVLGLLWFQK	1	740086846	2xTMT6plex [N-Term; K16]		100		1.478596687		0	2235.261167	2	1118.13269	-1.370386007	-0.001531585	115.4919326
																			VMGEVGK	2	740086846	2xTMT6plex [N-Term; K7]		1.075		2.897451878	34.24	0	1177.701501	2	589.3533936	-1.690131687	-0.000995235	48.5402536
																			YPGSTFMDHVLR	2	740086846	1xTMT6plex [N-Term]; 1xOxidation [M7]		1.242		2.699851274	14.86	0	1667.841293	3	551.2866211	-0.646524256	-0.000355986	58.1587923
																			VTPDTDWAR	2	740086846	1xTMT6plex [N-Term]	1.976			3.460030556	32.24	0	1289.668731	2	645.3374023	-0.932985712	-0.000601621	51.24572343
																			TILSLMTR	5	740086846	1xTMT6plex [N-Term]				1.698583126	16.26	0	1163.701946	2	582.354248	-0.624142851	-0.000363158	67.65645311
																			GQELIYAGMPITEVFK	2	740086846	2xTMT6plex [N-Term; K16]				1.523745656		0	2254.255748	2	1127.631592	0.07068131	7.97E-05	89.80603227
																			LNPEDIKK	2	740086846	3xTMT6plex [N-Term; K7; K8]	1.429	1.046		3.082888603	6.65	1	1644.029918	3	548.6820068	0.942213883	0.000516343	44.82237915
																			AKPAMPQDSVPSPR	1	740086846	2xTMT6plex [N-Term; K2]			0.856	2.769611597		0	1939.083537	3	647.0338135	1.727460227	0.001116563	37.93551138
71037379	PYGL	glycogen phosphorylase, liver form isoform 1	5836	NP_002854.3	44.86422668	1	29	34	167	847	97.087	7.17	1.15	1.31	1.5	1.32	Significant	0.0058																
																			LKQEYFVVAATLQDIIR	7	71037379	2xTMT6plex [N-Term; K2]	1.112	1.252	100	4.556377888	28.94	1	2465.453202	3	822.4914551	2.68113561	0.002203405	111.9239782
																			GIVGVENVAELK	7	71037379	2xTMT6plex [N-Term; K12]	1.271	1.259	100	5.034859657	85.37	0	1686.020192	2	843.5134888	-0.291420553	-0.00024567	74.03750838
																			WIDTQVVLALPYDTPVPGYMNNTVNTMR	4	71037379	1xTMT6plex [N-Term]		100		2.953600168	21.48	0	3437.743498	3	1146.586792	0.676090745	0.000774742	105.3624676
																			GAGTVFDAFPDQVAIQLNDTHPALAIPELMR	6	71037379	1xTMT6plex [N-Term]			0.786	2.938475847	24.28	0	3536.840904	4	884.963562	-2.399345448	-0.002121526	101.2690528
																			SRPEFMLPVHFYGK	2	71037379	2xTMT6plex [N-Term; K14]	0.751			4.156018734	27.34	0	2166.193422	4	542.3036499	-0.301243009	-0.000163138	77.45785341
																			DYYFALAHTVR	2	71037379	1xTMT6plex [N-Term]	1.143			4.367905617	43.57	0	1584.837194	3	528.9499512	-1.194617581	-0.000631091	68.41848798
																			MSLIEEEGSK	4	71037379	2xTMT6plex [N-Term; K10]	1.241		100	4.122885227	59.74	0	1580.86058	2	790.9342651	0.426061252	0.000336772	63.3971662
																			DIINMLFYHDR	6	71037379	1xTMT6plex [N-Term]	1.358	1.625		3.687046766	46.73	0	1665.862028	2	833.4345703	-0.098474542	-8.20E-05	94.04065285
																			DGWQVEEADDWLR	6	71037379	1xTMT6plex [N-Term]	1.044			2.711137772	22.54	0	1847.876158	2	924.440979	-0.798875066	-0.000738111	79.0211578
																			IDDVAALDK	8	71037379	2xTMT6plex [N-Term; K9]	1.549	1.175	100	3.832199097	46.25	0	1417.830266	2	709.4185791	-0.271370194	-0.000192378	60.11531451
																			IDDVAALDKK	6	71037379	3xTMT6plex [N-Term; K9; K10]	1.205	1.152		3.626789331	18.62	1	1775.088161	3	592.3676758	0.176213079	0.000104265	58.83540536
																			VFADYEAYVK	4	71037379	2xTMT6plex [N-Term; K10]	100			2.298087835	29.85	0	1662.91433	2	831.9606323	-0.205808295	-0.000171121	87.11490099
																			AAPGYHMAK	2	71037379	2xTMT6plex [N-Term; K9]; 1xOxidation [M7]	1.237			2.707461119	38.82	0	1419.781877	3	473.9318848	-0.546431207	-0.000258604	30.69714757
																			AAPGYHMAK	2	71037379	2xTMT6plex [N-Term; K9]	1.077			3.208882809	33.2	0	1403.786962	3	473.9318848	-0.546431207	-0.000258604	30.69714757
																			EYYEALPELK	7	71037379	2xTMT6plex [N-Term; K10]	0.76		100	2.547627449	29.73	0	1712.95111	2	856.9790039	-0.22093888	-0.000189229	69.88180404
																			INPSSMFDVQVK	4	71037379	2xTMT6plex [N-Term; K12]	1.3			2.857550859	28.65	0	1823.013727	2	912.0110474	0.598710008	0.000545728	95.67139864
																			LHSFLGDDVFLR	4	71037379	1xTMT6plex [N-Term]	1.034			4.233451366	29.19	0	1647.905607	3	549.9735718	0.336592114	0.000184891	94.64318821
																			GIVGVENVAELKK	2	71037379	3xTMT6plex [N-Term; K12; K13]			0.271	1.858922482	17.19	1	2043.278087	3	681.7631836	-1.512123469	-0.001029896	56.32396169
																			VSQLYMNPK	2	71037379	2xTMT6plex [N-Term; K9]	1.194			3.017971039	34.37	0	1537.881256	2	769.4437256	-0.703258539	-0.000540764	54.56959349
																			DFSELEPDKFQNK	5	71037379	3xTMT6plex [N-Term; K9; K13]	100	100		3.039768696	10.9	1	2284.242824	3	762.085022	-1.011767229	-0.000770374	56.22597505
																			EYAQNIWNVEPSDLK	4	71037379	2xTMT6plex [N-Term; K15]				2.11553359	11.72	0	2264.196318	2	1132.602783	0.870660479	0.000985673	68.30483723
																			MSLIEEEGSKR	8	71037379	2xTMT6plex [N-Term; K10]	0.986	1.406	2.076	4.113374233	19.15	1	1736.961691	3	579.6588135	0.112903865	6.54E-05	47.43658557
																			VIFLENYR	6	71037379	1xTMT6plex [N-Term]	1.631	1.195	5.229	2.893500805	30.16	0	1282.735689	2	641.8728638	2.153365722	0.0013811	71.08699832
																			VLYPNDNFFEGK	7	71037379	2xTMT6plex [N-Term; K12]	1.543	1.582	1.124	2.476181269	19.35	0	1901.02092	2	951.0133057	-0.834173816	-0.000792891	68.80368886
																			ISLSNESNK	2	71037379	2xTMT6plex [N-Term; K9]	0.7			1.940560102	11.51	0	1449.831329	2	725.4199219	0.854023482	0.000619095	31.00028475
																			YEYGIFNQK	4	71037379	2xTMT6plex [N-Term; K9]	1.028		100	1.693986058	8.65	0	1619.883365	2	810.4436646	-2.044615378	-0.001656019	59.87546256
																			AWELTQK	6	71037379	2xTMT6plex [N-Term; K7]	1.163	0.993	0.884	3.214220047	44.79	0	1333.788008	2	667.3967285	-1.369999385	-0.000913644	54.8409935
																			IFVDIEK	8	71037379	2xTMT6plex [N-Term; K7]	1.091	1.515		3.266314507	35.45	0	1321.81316	2	661.4105225	0.460338793	0.000304241	78.53969389
																			APNDFNLR	4	71037379	1xTMT6plex [N-Term]	0.963		0.92	3.115622044	22.94	0	1175.637037	2	588.3222656	0.18483596	0.00010865	46.96636897
																			DLSQLTK	6	71037379	2xTMT6plex [N-Term; K7]	1.118	1.088	1.1	3.322624683	36.49	0	1262.772023	2	631.8892212	-0.679067299	-0.000428754	45.55697356
																			YGNPWEK	8	71037379	2xTMT6plex [N-Term; K7]	1.079	1.426	1.639	3.035600901	28.4	0	1351.741058	2	676.3731079	-1.567104643	-0.00105916	47.43666643
																			IGEDYVK	4	71037379	2xTMT6plex [N-Term; K7]	1.976		1.95	2.890593052	26.65	0	1281.745474	2	641.3760376	-0.527097385	-0.000337802	37.97863564
																			IVALFPK	6	71037379	2xTMT6plex [N-Term; K7]	1.163	0.761		2.511074066	26.84	0	1245.833501	2	623.4195557	-1.337828749	-0.000833356	99.07148492
																			VIPATDLSEQISTAGTEASGTGNMK	2	71037379	2xTMT6plex [N-Term; K25]; 1xOxidation [M24				1.176263809	4.81	0	2952.523602	3	984.8433228	-2.772912053	-0.002729029	64.06597612
																			LITSVADVVNNDPMVGSK	2	71037379	2xTMT6plex [N-Term; K18]				1.602995157		0	2317.283753	2	1159.143799	-1.481110695	-0.001716077	81.38016271
5453603	CCT2	T-complex protein 1 subunit beta isoform 1	10576	NP_006422.1	53.08411215	1	22	22	249	535	57.452	6.46	1.12	1.02	1.13	1.09	Significant	0.001																
																			SLHDALCVLAQTVK	10	5453603	2xTMT6plex [N-Term; K14]; 1xCarbamidomethyl [C7	0.947	0.802	1.194	4.3971591	36.28	0	2013.156703	3	671.7236938	-0.086633609	-5.81E-05	98.24165475
																			TVYGGGCSEMLMAHAVTQLANR	6	5453603	1xTMT6plex [N-Term]; 1xCarbamidomethyl [C7				3.215605259	27.56	0	2595.267913	3	865.7595825	-1.432893069	-0.00123958	70.88796526
																			VQDDEVGDGTTSVTVLAAELLR	10	5453603	1xTMT6plex [N-Term]	1.427	1.376	1.646	5.827342987	88.41	0	2517.324542	3	839.7791748	-0.624129273	-0.000523712	103.2191872
																			EALLSSAVDHGSDEVK	8	5453603	2xTMT6plex [N-Term; K16]	1.2	1.207	0.654	5.177409649	54.35	0	2115.133384	3	705.7158203	-0.225112281	-0.000158714	61.46864486
																			LALVTGGEIASTFDHPELVK	8	5453603	2xTMT6plex [N-Term; K20]	1.013	1.286	100	4.079594135	46.75	0	2555.44851	3	852.4893188	1.914757018	0.001631021	92.63014554
																			MLPTIIADNAGYDSADLVAQLR	2	5453603	1xTMT6plex [N-Term]; 1xOxidation [M1]	0.184			3.022075415	15.82	0	2592.354069	3	859.4572144	-0.801104765	-0.000687978	99.22737736
																			MLPTIIADNAGYDSADLVAQLR	12	5453603	1xTMT6plex [N-Term]	3.303	1.012	1.215	3.498361588	48.18	0	2576.359154	3	859.4572144	-0.801104765	-0.000687978	99.22737736
																			GSGNLEAIHIIK	14	5453603	2xTMT6plex [N-Term; K12]	1.404	1.518	0.431	3.516080379	47.57	0	1710.031426	2	855.519043	-0.360427587	-0.000308171	56.09850389
																			GATQQILDEAER	4	5453603	1xTMT6plex [N-Term]	1.453	1.307		4.894545078	67.34	0	1559.822666	2	780.4154663	0.634961403	0.000495214	53.3174899
																			EAVAMESYAK	4	5453603	2xTMT6plex [N-Term; K10]		1.153	1.163	4.639853001	61.98	0	1556.839451	2	778.9232178	-0.187471563	-0.000145932	59.51418117
																			AAHSEGNTTAGLDMR	48	5453603	1xTMT6plex [N-Term]; 1xOxidation [M14]	1.115	1.004	1.277	4.927075863	55.12	0	1775.854377	3	587.2911987	-0.237955702	-0.00013959	37.54674649
																			AAHSEGNTTAGLDMR	40	5453603	1xTMT6plex [N-Term]	0.991	1.005	0.98	5.843164444	86.93	0	1759.859462	3	587.2911987	-0.237955702	-0.00013959	37.54674649
																			LIEEVMIGEDK	11	5453603	2xTMT6plex [N-Term; K11]	1.192	1.658		2.97158885	31.74	0	1733.975944	2	867.4909058	-0.812731323	-0.000704628	74.44769749
																			ILIANTGMDTDK	6	5453603	2xTMT6plex [N-Term; K12]	1.114	0.949		3.220624685	44.31	0	1749.982092	2	875.4956665	1.122427379	0.000982114	60.15851051
																			DASLMVTNDGATILK	8	5453603	2xTMT6plex [N-Term; K15]	100	100	0.01	2.877383471	28.65	0	2007.119648	2	1004.06012	-3.330888716	-0.003342746	71.03655603
																			VAEIEHAEK	8	5453603	2xTMT6plex [N-Term; K9]	1.316	1.48	1.362	3.399830103	42.16	0	1483.852064	3	495.2895813	1.432923494	0.000708749	38.1673061
																			QDLMNIAGTTLSSK	4	5453603	2xTMT6plex [N-Term; K14]				1.928628802	21.92	0	1937.077783	2	969.0418091	-0.744320104	-0.000720903	75.83924142
																			KIHPQTIIAGWR	4	5453603	2xTMT6plex [N-Term; K1]	0.748	0.381		4.024010658	25.77	1	1878.147793	3	626.7208252	0.068959396	4.32E-05	59.62242685
																			EAVAMESYAK	10	5453603	2xTMT6plex [N-Term; K10]; 1xOxidation [M5	1.22	1.197	1.141	2.909208298	49.33	0	1572.834366	2	778.9232178	-0.187471563	-0.000145932	59.51418117
																			EAESLIAK	8	5453603	2xTMT6plex [N-Term; K8]	1.301	1.091	1.355	3.695840836	47.15	0	1318.798238	2	659.899231	-5.347820261	-0.003526348	44.2908528
																			LAVEAVLR	12	5453603	1xTMT6plex [N-Term]	2.328	2.045	0.204	2.707846403	47.18	0	1099.70366	2	550.355896	0.77748417	0.000427501	54.9471302
																			LKGSGNLEAIHIIK	3	5453603	3xTMT6plex [N-Term; K2; K14]	100			1.762681365	7.45	1	2180.373385	4	545.8491211	0.582443819	0.000317486	58.48756446
																			ILIANTGMDTDKIK	1	5453603	3xTMT6plex [N-Term; K12; K14]				1.33734405		1	2220.324051	3	740.7792358	-0.40389621	-0.000298927	56.47223585
																			KLGGSLADSYLDEGFLLDK	3	5453603	3xTMT6plex [N-Term; K1; K19]				1.80019629	3.66	1	2728.537609	3	910.1835938	-0.505981664	-0.000460197	82.01190642
																			VLVDMSR	5	5453603	1xTMT6plex [N-Term]	1.129	0.599	1.083	2.526189327	30.89	0	1048.602232	2	524.8041992	-1.058535321	-0.000554991	43.16237227
10863945	XRCC5	X-ray repair cross-complementing protein	7520	NP_066964.1	52.04918033	1	27	27	127	732	82.652	5.81	1.35	1.68	1.44	1.49	Significant	0.0043																
																			LGGHGPSFPLK	6	10863945	2xTMT6plex [N-Term; K11]	2.246	2.284	2.136	4.325977325	37.35	0	1567.936069	3	523.3168335	-0.077555279	-4.05E-05	49.89492771
																			YAPTEAQLNAVDALIDSMSLAK	18	10863945	2xTMT6plex [N-Term; K22]	2.256	1.448	1.564	4.401222706	40.87	0	2779.495202	3	927.1705322	0.662400743	0.000613713	107.9648301
																			QVFAENKDEIALVLFGTDGTDNPLSGGDQYQNITVHR	2	10863945	2xTMT6plex [N-Term; K7]				3.238454342	6.25	1	4520.30979	4	1130.83313	0.19892912	0.000224805	97.93981711
																			DQVTAQEIFQDNHEDGPTAK	4	10863945	2xTMT6plex [N-Term; K20]	0.113		2.167	4.023397923	38.74	0	2701.346958	3	901.1210938	0.655180555	0.000589957	57.52015648
																			EEASGSSVTAEEAK	13	10863945	2xTMT6plex [N-Term; K14]	1.777	1.389	1.723	6.26645565	93.83	0	1852.954023	2	926.9799805	-0.722226501	-0.000669126	27.55970312
																			ASFEEASNQLINHIEQFLDTNETPYFMK	8	10863945	2xTMT6plex [N-Term; K28]	1.361			3.437243223	23.52	0	3774.872547	3	1258.958496	-3.076196244	-0.00387075	112.453094

Bhat et al,2017. Cigarette smoke and chewing tobacco alter expression of different sets of miRNAs in oral keratinocytes

Supplementary Table S2(A). List of all proteins and peptides identified in OKF6/TERT1 cells chronically treated with chewing tobacco using Sequest and Mascot search algorithms

Accession	Gene_Symbol	Description	Gene_ID	Refseq_Accession	Coverage	Proteins	#Unique_Peptides	#Peptides	#PSMS	#AAs	MW [kDa]	calc. pI	OKF6-STE-6M/OKF6P_R1	OKF6-STE-6M/OKF6P_R2	OKF6-STE-6M/OKF6P_R3	Median fold change	Status	P-value (6M)	Sequence	#PSMs	Protein Accessions	Modifications	OKF6-STE-6M/OKF6P_R1	OKF6-STE-6M/OKF6P_R2	OKF6-STE-6M/OKF6P_R3	Xcorr	Ionscore	# Missed Cleavages	Theo. MH+ [Da]	Charge	m_z	DeltaM [ppm]	Deltam/z [Da]	RT [min]
																			KKDQVTAQEIFQDNHEDGPTAK	4	10863945	4xTMT6plex [N-Term; K1; K2; K22]	0.969		0.971	7.4809165	29.8	2	3415.862748	5	683.9770508	-1.932353328	-0.001320131	42.83616113
																			ANPQVGVAFPHIK	2	10863945	2xTMT6plex [N-Term; K13]		1.412		4.642048836	37.01	0	1836.089609	3	612.7009888	-0.65148313	-0.000398727	55.65167381
																			IQPGSQQADFLDALIVSMDVIQHETIGK	2	10863945	2xTMT6plex [N-Term; K28]; 1xOxidation [M18			1.733	4.863550186	42.35	0	3527.88199	4	882.7268677	1.034777562	0.000912643	105.0925875
																			HLMLPDFDLLEDIESK	10	10863945	2xTMT6plex [N-Term; K16]	1.815	1.05		3.534158468	37.7	0	2373.277605	3	791.7629395	-1.407370388	-0.00111336	102.4033845
																			IQPGSQQADFLDALIVSMDVIQHETIGK	2	10863945	2xTMT6plex [N-Term; K28]				2.42011404	15.57	0	3511.887075	4	882.7268677	1.034777562	0.000912643	105.0925875
																			KASFEEASNQLINHIEQFLDTNETPYFMK	2	10863945	3xTMT6plex [N-Term; K1; K29]				2.989619017	9.44	1	4132.130442	4	1033.780396	-7.427182946	-0.007672522	109.7632699
																			LTIGSNLSIR	2	10863945	1xTMT6plex [N-Term]	100			3.464417696	43.23	0	1302.794266	2	651.9011841	0.633549045	0.000412692	61.93528093
																			SQLDIIIHSLK	3	10863945	2xTMT6plex [N-Term; K11]	1.639			2.991157532	22.83	0	1725.067477	3	575.6928711	-1.980663827	-0.001138926	91.78783555
																			EEASGSSVTAEEAKK	2	10863945	3xTMT6plex [N-Term; K14; K15]	1.548			3.20622015	8.91	1	2210.211918	3	737.4091797	0.483339399	0.000356094	38.37329551
																			EPLPPIQQHIWNMLNPPAEVTTK	5	10863945	2xTMT6plex [N-Term; K23]				3.457606316	22.08	0	3111.706532	3	1037.908569	1.485470357	0.001540783	92.19637602
																			TDTLEDLFPTTK	4	10863945	2xTMT6plex [N-Term; K12]		100		2.119146824	29.94	0	1839.015166	2	920.0109863	-0.255742181	-0.000235157	79.42000296
																			DKPSGDTAAVFEEGGDVDDLLDMI	6	10863945	2xTMT6plex [N-Term; K2]	0.394		100	2.513324261	15.58	0	2967.454519	3	989.8237305	0.714102351	0.000706355	103.2807471
																			HLMLPDFDLLEDIESK	2	10863945	2xTMT6plex [N-Term; K16]; 1xOxidation [M3			1.177	4.153190136	33.89	0	2389.27252	3	791.7629395	-1.407370388	-0.00111336	102.4033845
																			HNYECLVYVQLPFMEDLR	2	10863945	1xTMT6plex [N-Term]; 1xCarbamidomethyl [C5	100			2.395749569	10.41	0	2555.262417	3	852.4263916	0.862739057	0.000734842	96.89236858
																			MVMISLEGEDGLDEIYSFSESLR	2	10863945	1xTMT6plex [N-Term]			100	2.887473106	12.44	0	2849.378628	3	950.4665527	2.273117746	0.002158991	109.717859
																			TLFPLIEAK	2	10863945	2xTMT6plex [N-Term; K9]		1.088		2.758393526	25.65	0	1489.939423	2	745.4727783	-0.767064323	-0.00057144	83.65748131
																			DKPSGDTAAVFEEGGDVDDLLDMI	1	10863945	2xTMT6plex [N-Term; K2]; 1xOxidation [M23]				1.096273541		0	2983.449434	3	989.8237305	0.714102351	0.000706355	103.2807471
																			EGLEIVK	6	10863945	2xTMT6plex [N-Term; K7]	1.363	1.894	1.668	2.884269953	35.67	0	1245.78186	2	623.3937988	-1.235098856	-0.000769332	48.9338494
																			KTWTVVDAK	2	10863945	3xTMT6plex [N-Term; K1; K9]			1.287	2.829913378	4.01	1	1735.072117	3	579.0286255	-0.457713053	-0.000264722	46.22937082
																			HIEIFTDLSSR	3	10863945	1xTMT6plex [N-Term]		100		1.938925266	11.23	0	1546.842673	3	516.2855225	-0.425813949	-0.000219556	67.78475491
																			EDIIQGFR	4	10863945	1xTMT6plex [N-Term]			1.404	2.213805199	30.43	0	1206.668003	2	603.8321533	-9.093685543	-0.00548653	53.12327212
																			GITEQQK	4	10863945	2xTMT6plex [N-Term; K7]		1.62	1.523	3.350287914	34.03	0	1261.751622	2	631.3803101	1.364228234	0.000860659	29.43998003
																			VDEEQMK	2	10863945	2xTMT6plex [N-Term; K7]	1.341			2.916997194	41.1	0	1336.718273	2	668.862854	0.118407757	7.91E-05	36.67899757
																			CFSVLGFCK	2	10863945	2xTMT6plex [N-Term; K9]; 2xCarbamidomethyl [C1; C8				1.623688459	8.72	0	1575.842763	2	788.4257202	0.889092313	0.000700535	77.37931203
260099723	LDHA	L-lactate dehydrogenase A chain isoform [illegible]	3939	NP_001158886.1	52.35457064	1	18	20	339	361	39.812	8.43	0.8	0.89	0.89	0.86	Significant	0.0012																
																			DLADELALVDVIEDK	23	260099723	2xTMT6plex [N-Term; K15]	2.055	1.273	1.097	4.910812378	93.81	0	2116.178937	2	1058.593018	-0.084351917	-8.93E-05	102.2215605
																			VIGSGCNLDSAR	28	260099723	1xTMT6plex [N-Term]; 1xCarbamidomethyl [C6	0.938	0.934	0.93	4.715711594	77.43	0	1477.763043	2	739.3862915	1.531901764	0.001131894	37.24424387
																			GEMMDLQHGSLFLR	23	260099723	1xTMT6plex [N-Term]; 1xOxidation [M]	0.695	1.153	100	3.871815443	50.83	0	1878.940355	3	626.9840088	-1.533780891	-0.000960628	66.99877173
																			GEMMDLQHGSLFLR	18	260099723	1xTMT6plex [N-Term]	0.98	1.093	0.926	3.878205776	44.78	0	1862.94544	3	626.9840088	-1.533780891	-0.000960628	66.99877173
																			LKGEMMDLQHGSLFLR	6	260099723	2xTMT6plex [N-Term; K2]	0.943	0.783	100	3.227262259	24.76	1	2333.287399	4	584.0762939	-1.737029374	-0.001013247	84.72607724
																			GYTSWAIGLSVADLAESIMK	16	260099723	2xTMT6plex [N-Term; K20]	1.017		100	4.820116043	46.71	0	2570.394032	3	857.46875	-0.908456046	-0.000778363	114.1329037
																			KSADTLWGIQK	6	260099723	3xTMT6plex [N-Term; K1; K11]	0.504	0.501	0.562	2.837706327	11.84	1	1934.167809	3	645.3939209	-0.30975838	-0.000199708	52.28200789
																			VTLTSEEEAR	47	260099723	1xTMT6plex [N-Term]	0.879	0.856	0.813	4.675195694	70.99	0	1363.72664	2	682.3670654	0.157091142	0.000107115	43.88710277
																			LKGYTSWAIGLSVADLAESIMK	2	260099723	3xTMT6plex [N-Term; K2; K22]				3.133616447	13.37	1	3040.735991	3	1014.249207	-0.961724761	-0.000974784	112.5710764
																			GYTSWAIGLSVADLAESIMK	2	260099723	2xTMT6plex [N-Term; K20]; 1xOxidation [M19	100			2.226243258	31.76	0	2586.388947	3	857.46875	-0.908456046	-0.000778363	114.1329037
																			QVVESAYEVIK	11	260099723	2xTMT6plex [N-Term; K11]	1.328	1.244	1.103	3.170071602	42.73	0	1723.004208	2	862.0049438	-0.926727097	-0.000798377	58.60254817
																			SADTLWGIQK	23	260099723	2xTMT6plex [N-Term; K10]	0.726	1.035	0.74	3.27631259	46.44	0	1576.909914	2	788.9578857	-0.899699858	-0.000709373	58.60554888
																			DYNVTANSK	6	260099723	2xTMT6plex [N-Term; K9]	0.91	0.951	1.045	4.375481129	54.75	0	1469.800029	2	735.399231	-6.016822537	-0.004421763	36.15657815
																			LVIITAGAR	48	260099723	1xTMT6plex [N-Term]	0.799	0.877	0.811	3.069191694	45.74	0	1142.74586	2	571.8761597	-0.714773154	-0.000408402	50.88792399
																			TLHPDLGTDKDKEQWK	16	260099723	4xTMT6plex [N-Term; K10; K12; K16]	0.64	0.681	0.708	4.633420467	10.6	2	2827.612394	3	943.2084351	-0.580859039	-0.000547481	54.88376162
																			DQLIYNLLK	16	260099723	2xTMT6plex [N-Term; K9]	1.142	1.296	1.199	3.601190805	40.45	0	1577.9667	2	789.4873657	0.478213712	0.000377303	96.71534819
																			TLHPDLGTDKDK	6	260099723	3xTMT6plex [N-Term; K10; K12]	1.075	1.065		3.895605564	15.94	1	2027.174016	4	507.5483093	-1.28683344	-0.000652159	45.80710201
																			VHPVSTMIK	8	260099723	2xTMT6plex [N-Term; K9]	0.786			3.560096502	43.9	0	1469.891427	2	735.4501953	1.147788857	0.000843563	42.03935445
																			GEMMDLQHGSLFLR	6	260099723	1xTMT6plex [N-Term]; 2xOxidation [M3; M4]	0.802		0.659	2.413901091	20.3	0	1894.93527	3	626.9840088	-1.533780891	-0.000960628	66.99877173
																			LIIVSNPVDILTYVAWK	4	260099723	2xTMT6plex [N-Term; K17]				1.860337019	11.01	0	2402.446326	2	1201.726929	0.106250813	0.000127631	112.444272
																			VHPVSTMIK	2	260099723	2xTMT6plex [N-Term; K9]; 1xOxidation [M7]	0.843			2.266981125	16.34	0	1485.886342	2	735.4501953	1.147788857	0.000843563	42.03935445
																			FIIPNVVK	20	260099723	2xTMT6plex [N-Term; K8]	0.785	0.828	0.846	2.974152088	30.29	0	1387.907729	2	694.4563599	-1.646949138	-0.001142907	68.48009
																			EEQTPQNK	2	260099723	2xTMT6plex [N-Term; K8]	1.823			2.641346931	30.58	0	1431.784379	2	716.3945313	-1.810943071	-0.00129644	27.20612577
																			QQEGESR	2	260099723	1xTMT6plex [N-Term]				1.745908499	15.4	0	1062.537717	2	531.7727051	0.391831973	0.000208168	11.0366653
21361657	PDIA3	protein disulfide-isomerase A3 precursor	2923	NP_005304.3	61.78217822	1	26	26	250	505	56.747	6.35	1.06	0.5	1.02	0.86	Significant	0.041																
																			KTFSHELSDFGLESTAGEIPVVAIR	6	21361657	2xTMT6plex [N-Term; K1]	0.843			6.180625439	43.36	1	3161.724685	4	791.1852417	-1.754692036	-0.001386963	83.09405992
																			FIQENIFGICPHMTEDNKDLIQGK	6	21361657	3xTMT6plex [N-Term; K18; K24]; 1xCarbamidomethyl [C10	1.443			3.686175346	9.24	1	3534.869207	4	884.4682007	-5.15825185	-0.004558436	77.07543337
																			MDATANDVPSPYEVR	6	21361657	1xTMT6plex [N-Term]	1.284	100	1.172	4.634408474	46.33	0	1893.921393	2	947.4660645	1.826409619	0.001729538	54.66612363
																			TFSHELSDFGLESTAGEIPVVAIR	11	21361657	1xTMT6plex [N-Term]	0.962	2.881	100	4.738928795	21.45	0	2804.46679	3	935.4937134	-0.07235717	-6.76E-05	97.5715391
																			DASIVGFFDDSFSEAHSEFLK	12	21361657	2xTMT6plex [N-Term; K21]	100	100	1.493	5.10529232	62.4	0	2806.397597	3	936.137207	-0.188460912	-0.000176299	101.237856
																			DGEEAGAYDGPR	10	21361657	1xTMT6plex [N-Term]	0.776	0.671	0.783	4.234223843	55.9	0	1465.675667	2	733.3416138	0.193691598	0.000141945	29.50408391
																			EATNPPVIQEEKPK	8	21361657	3xTMT6plex [N-Term; K12; K14]	1.037	1.093	0.983	4.414767265	30.39	0	2267.321408	3	756.4453735	0.070080699	5.30E-05	45.63205617
																			SEPIPESNDGPVK	6	21361657	2xTMT6plex [N-Term; K13]	0.638	1.098	1.124	3.757732391	45.53	0	1826.990014	2	913.9987183	0.079734118	7.28E-05	44.82910642
																			VVVAENFDEIVNNENK	12	21361657	2xTMT6plex [N-Term; K16]	2.034	100	100	3.086368322	30.55	0	2291.228347	3	764.4137573	-0.710565294	-0.000542689	75.66832868
																			TADGIVSHLK	13	21361657	2xTMT6plex [N-Term; K10]	0.985	0.226	0.848	4.145504951	52.49	0	1498.899349	2	749.9526367	-0.902083586	-0.000676066	48.22413196
																			VDCTANTNTCNK	6	21361657	2xTMT6plex [N-Term; K12]; 2xCarbamidomethyl [C3; C10	1.531	0.933	1.384	4.017229557	37.23	0	1855.904253	2	928.4549561	-0.871602413	-0.000808805	32.66371436
																			FISDKDASIVGFFDDSFSEAHSEFLK	2	21361657	3xTMT6plex [N-Term; K5; K26]				3.21458602	19.92	1	3625.866941	4	907.2134399	-9.655874015	-0.008752729	100.4824136
																			FLQDYFDGNLK	14	21361657	2xTMT6plex [N-Term; K11]	1.169	1.301	100	3.447132349	46.4	0	1817.983807	2	909.494751	-0.869868515	-0.000790703	93.73855048
																			GFPTIYFSPANK	14	21361657	2xTMT6plex [N-Term; K12]	0.996	0.943	1.322	2.831918955	36.62	0	1800.009628	2	900.5074463	-1.117572842	-0.001005821	79.13559164
																			DLLIAYYDVDYEK	36	21361657	2xTMT6plex [N-Term; K13]	0.797	3.561	0.635	3.136658192	33.88	0	2078.109795	2	1039.558105	-0.414137165	-0.000430311	94.77258688
																			VVVAENFDEIVNNENKDVLIEFYAPWCGHCK	2	21361657	3xTMT6plex [N-Term; K16; K31]; 2xCarbamidomethyl [C27; C3				1.97947979	8.24	1	4396.234925	4	1099.811768	-2.202841219	-0.002421052	105.0211207
																			LSKDPNIVIAK	6	21361657	3xTMT6plex [N-Term; K3; K11]	0.753	0.744	0.695	3.452610731	16.92	1	1885.208945	3	629.0742188	-0.446698496	-0.000280707	46.80907969
																			YGVSGYPTLK	4	21361657	2xTMT6plex [N-Term; K10]	2.301	1.587		3.72298193	36.51	0	1542.893201	2	771.949707	-0.689294307	-0.000531754	55.155576
																			FVMQEEFSR	11	21361657	1xTMT6plex [N-Term]	1.12	0.977	1.104	3.328223467	41.3	0	1401.703402	2	701.3560181	0.968295297	0.000678631	60.54800036
																			FIQENIFGICPHMTEDNK	8	21361657	2xTMT6plex [N-Term; K18]; 1xCarbamidomethyl [C10			100	1.591772199	7.72	0	2651.3362	3	884.4483032	-2.203900823	-0.001947761	72.96059659
																			MDATANDVPSPYEVR	2	21361657	1xTMT6plex [N-Term]; 1xOxidation [M1]	1.622			2.057111263	30.58	0	1909.916308	2	947.4660645	1.826409619	0.001729538	54.66612363
																			TVAYTEQK	12	21361657	2xTMT6plex [N-Term; K8]	1.157	1.009	1.347	3.835304976	44.11	0	1397.804052	2	699.4057007	0.052315764	3.66E-05	34.70168567
																			QAGPASVPLR	2	21361657	1xTMT6plex [N-Term]	1.286			3.649727345	32.79	0	1224.726187	2	612.8665161	-0.351959027	-0.000215527	38.2581739
																			DPNIVIAK	23	21361657	2xTMT6plex [N-Term; K8]	1.008	0.996	1.136	3.017391682	37.37	0	1327.834958	2	664.4210205	-0.145714175	-9.67E-05	58.32668816
																			LNFAVASR	11	21361657	1xTMT6plex [N-Term]	1.243	1.224	1.192	3.046082258	43.65	0	1106.651959	2	553.8291016	-0.933134389	-0.000516327	52.26257919
																			FAHTNVESLVNEYDDNGEGIILFRPSHLTNK	1	21361657	2xTMT6plex [N-Term; K31]				1.898702621		0	3988.060499	5	798.4192505	1.666620004	0.001329316	84.79375636
																			FVMQEEFSR	4	21361657	1xTMT6plex [N-Term]; 1xOxidation [M3]	1.41	1.138		1.868756175	28.02	0	1417.698317	2	701.3560181	0.968295297	0.000678631	60.54800036
																			GIVPLAK	2	21361657	2xTMT6plex [N-Term; K7]	1.058			2.455747366	18.39	0	1155.786551	2	578.3972168	0.524087903	0.000302867	58.27289803
40354195	KRT18	keratin, type I cytoskeletal 18	3875	NP_954657.1	62.09302326	1	23	24	215	430	48.029	5.45	1.4	1.4	1.41	1.4	Significant	0.0000056																
																			AQIFANTVDNAR	14	40354195	1xTMT6plex [N-Term]	1.492	1.582	1.713	4.286262035	63.74	0	1548.833171	2	774.9157104	-5.827910799	-0.004513231	47.47480684
																			GGMGSGGLATGIAGGLAGMGGIQNEK	6	40354195	2xTMT6plex [N-Term; K26]	100		1.674	6.281977654	51.14	0	2719.42714	3	912.4784546	-0.454785566	-0.000414677	75.73719635
																			GGMGSGGLATGIAGGLAGMGGIQNEK	4	40354195	2xTMT6plex [N-Term; K26]; 1xOxidation [M			1.44	5.330696106	53.74	0	2735.422055	3	912.4784546	-0.454785566	-0.000414677	75.73719635
																			YALQMEQLNGILLHLESELAQTR	6	40354195	1xTMT6plex [N-Term]		1.486		4.081028461	38.48	0	2899.554893	3	967.180542	-9.59477384	-0.009273524	114.508705
																			LLEDGEDFNLGDALDSSNSMQTIQK	7	40354195	2xTMT6plex [N-Term; K25]	0.01	100		2.666665077	23.94	0	3198.587658	3	1066.865234	-2.034812141	-0.002169508	105.198978
																			LQLETEIEALKEELLFMK	7	40354195	3xTMT6plex [N-Term; K11; K18]	2.138	0.684		3.010620832	28.72	1	2864.66618	3	955.5585938	-1.728609789	-0.00165063	109.633142
																			EELDKYWSQQIEESTTVVTTQSAEVGAAETTLTELR	8	40354195	2xTMT6plex [N-Term; K5]	100	3.518	1.676	7.383236885	44.17	1	4501.287383	4	1126.078003	0.621759624	0.00069968	102.744003
																			YWSQQIEESTTVVTTQSAEVGAAETTLTELR	6	40354195	1xTMT6plex [N-Term]		100	1.535	5.343587399	33.48	0	3657.833295	4	915.2139282	0.160682726	0.000146938	102.3207434
																			ETMQSLNDR	6	40354195	1xTMT6plex [N-Term]	1.587	1.265	1.31	3.750977039	55.87	0	1322.65718	2	661.8305054	-2.605495864	-0.001723089	38.01241578
																			LEAEIATYR	12	40354195	1xTMT6plex [N-Term]	1.902	2.034	1.886	3.687997103	43.93	0	1294.720433	2	647.8633423	-0.791344341	-0.000512285	50.75152065
																			IVLQIDNAR	27	40354195	1xTMT6plex [N-Term]	0.822	0.805	0.786	3.429313183	48.49	0	1270.768051	2	635.8876343	-0.046826258	-2.98E-05	51.78171565
																			QAQEYEALLNIK	5	40354195	2xTMT6plex [N-Term; K12]	100	100	100	2.583379269	24.81	0	1878.073684	2	939.5394287	-1.120029604	-0.001051749	67.05433563
																			VIDDTNITR	10	40354195	1xTMT6plex [N-Term]	1.521	1.862	1.358	3.516595125	48.85	0	1275.710596	2	638.3590698	0.209246861	0.000133469	39.86486319
																			TVQSLEIDLDSMR	8	40354195	1xTMT6plex [N-Term]	100	100	100	2.614585638	22.54	0	1735.909766	2	868.4579468	-0.661909585	-0.000574508	81.38822799
																			LLEDGEDFNLGDALDSSNSMQTIQK	6	40354195	2xTMT6plex [N-Term; K25]; 1xOxidation [M20			0.01	2.003798962	6.52	0	3214.582573	3	1066.865234	-2.034812141	-0.002169508	105.198978
																			VVSETNDTK	6	40354195	2xTMT6plex [N-Term; K9]	1.527	1.673	1.493	3.988177061	44.48	0	1450.815345	2	725.9110107	-0.413316296	-0.000299823	34.78315156
																			YETELAMR	6	40354195	1xTMT6plex [N-Term]	100	1.721	2.49	3.017670155	44.57	0	1241.639739	2	621.3231201	-0.624815396	-0.000387898	32.9861286
																			VKYETELAMR	12	40354195	2xTMT6plex [N-Term; K2]	1.332	1.471	1.413	3.74993825	23.02	1	1697.966048	2	849.4869995	0.397041766	0.000337082	44.25456141
																			KVIDDTNITR	8	40354195	2xTMT6plex [N-Term; K1]	0.961	0.814	1.24	3.517569542	27.02	1	1632.968491	2	816.9875488	-0.410304151	-0.000335007	41.52959241
																			AQYDELAR	12	40354195	1xTMT6plex [N-Term]	1.089	1.046	0.884	3.235019684	42.55	0	1194.631618	2	597.8192139	-0.390451431	-0.000233223	39.24082734
																			ASLENSLR	8	40354195	1xTMT6plex [N-Term]	1.08	1.13	1.402	2.882064104	39.14	0	1118.636703	2	559.8215942	-0.707203184	-0.000395552	44.15949522
																			STFSTNYR	2	40354195	1xTMT6plex [N-Term]	1.909			3.429884195	41.15	0	1204.615967	2	602.8117676	0.241600857	0.000145518	38.10985493
																			LASYLDR	12	40354195	1xTMT6plex [N-Term]	1.317	1.398	1.717	2.766142845	34.71	0	1066.609426	2	533.8082886	-0.117298385	-6.26E-05	56.24145575
																			ETMQSLNDR	4	40354195	1xTMT6plex [N-Term]; 1xOxidation [M3]		1.785	1.791	1.5877738	23.44	0	1338.652095	2	661.8305054	-2.605495864	-0.001723089	38.01241578
																			KNHEEEVK	2	40354195	3xTMT6plex [N-Term; K1; K8]	0.797			1.830724001	4.94	1	1699.994595	3	567.3364868	0.18346483	0.000103963	30.04469796
																			EELLFMK	2	40354195	2xTMT6plex [N-Term; K7]				1.739658713	17.9	0	1367.800881	2	684.4052124	1.657847074	0.001133802	74.64157416
																			DWSHYFK	8	40354195	2xTMT6plex [N-Term; K7]	1.855	1.519	1.317	2.578378677	20.99	0	1440.767607	3	480.927124	-0.546784703	-0.000262597	61.18841057
																			VKYETELAMR	1	40354195	2xTMT6plex [N-Term; K2]; 1xOxidation [M9]	1.516			1.377302408	8.48	1	1713.960963	2	849.4869995	0.397041766	0.000337082	44.25456141
21614499	EZR	ezrin	7430	NP_003370.2	44.19795222	1	16	30	220	586	69.37	6.27	0.98	0.91	0.97	0.95	Significant	0.00053																
																			SQEQLAAELAEYTAK	6	21614499	2xTMT6plex [N-Term; K15]	1.203	100		3.456320524	39.72	0	2110.14322	2	1055.576538	1.222330243	0.001289646	78.40200017
																			QLLTLSSELSQAR	4	21614499	1xTMT6plex [N-Term]	2.24	1.024		3.765939713	51.38	0	1674.958765	2	837.9821167	-1.0796096	-0.000904151	78.75620908
																			GTDLWLGVDALGLNIYEK	13	21614499	2xTMT6plex [N-Term; K18]	1.284	1.546	1.243	4.758788109	55.47	0	2435.358633	3	812.4584351	0.870202751	0.000706419	103.0292613
																			GTDLWLGVDALGLNIYEKDDK	5	21614499	3xTMT6plex [N-Term; K18; K21]			0.493	3.939820766	38.48	1	3022.670414	3	1008.227417	-0.898478188	-0.000905268	99.16268432
																			KPDTIEVQQMK	9	21614499	3xTMT6plex [N-Term; K1; K11]	0.928	0.765	0.866	4.951767921	43.06	0	2004.176659	3	668.7305908	0.279696371	0.000186854	42.3317132
																			KAPDFVFYAPR	12	21614499	2xTMT6plex [N-Term; K1]	0.737	0.828	0.648	3.476797581	26.85	1	1769.015047	3	590.3424683	-1.24127539	-0.000731945	54.53779583
																			APDFVFYAPR	18	21614499	1xTMT6plex [N-Term]	0.79	0.794	0.433	3.595981359	31.71	0	1411.757152	2	706.3810425	-1.660391136	-0.001172035	65.18701339
																			DNAMLEYLK	6	21614499	2xTMT6plex [N-Term; K9]	100	0.962	0.793	3.017518044	37.04	0	1554.880186	2	777.9331665	-0.726664814	-0.000564931	63.71556138
																			AKEAQDDLVK	8	21614499	3xTMT6plex [N-Term; K2; K10]	0.435	0.457	0.369	3.340918779	30.76	1	1804.078325	2	902.5438843	1.201203217	0.001083532	35.39752778
																			RKPDTIEVQQMK	18	21614499	3xTMT6plex [N-Term; K2; K12]	0.874	0.775	0.851	4.093585968	16.43	1	2160.27777	4	540.8242188	-1.261175779	-0.000681122	39.6647313
																			VTTMDAELEFAIQPNTTGK	2	21614499	2xTMT6plex [N-Term; K19]	100			2.649563313	29.67	0	2524.336911	3	842.1146851	-2.935002133	-0.002469645	79.69920076
																			KPDTIEVQQMK	8	21614499	3xTMT6plex [N-Term; K1; K11]; 1xOxidation [M10			0.855	4.497064114	24.67	0	2020.171574	3	668.7305908	0.279696371	0.000186854	42.3317132
																			RKEDEVEEWQHR	14	21614499	2xTMT6plex [N-Term; K2]	0.915	0.889	0.967	5.985223293	26.16	2	2099.103421	3	700.3725586	-0.142279197	-9.96E-05	32.50398835
																			QLFDQVVK	12	21614499	2xTMT6plex [N-Term; K8]	1.369	1.393	1.305	4.03649807	44.99	0	1434.872072	2	717.9403687	0.968068663	0.000694527	67.82446326
																			IALLEEAR	12	21614499	1xTMT6plex [N-Term]	1.003	1.059	2.142	3.440700293	46.62	0	1143.69349	2	572.3504028	0.034514547	1.97E-05	69.49373373
																			KFVIKPIDK	4	21614499	4xTMT6plex [N-Term; K1; K5; K9]	0.446	0.888		2.754837513	4.15	1	2004.339121	4	501.8404541	0.432055754	0.000216497	57.66246428
																			ELSEQIQR	7	21614499	1xTMT6plex [N-Term]		0.946	1.153	3.255117893	44.6	0	1231.684381	2	616.3450928	-1.195471135	-0.000736222	45.88565953
																			FVIKPIDKK	2	21614499	4xTMT6plex [N-Term; K4; K8; K9]	0.614	0.411		2.204612494		1	2004.339121	4	501.8398743	-0.725101692	-0.000363337	63.42701961
																			KENPLQFK	6	21614499	3xTMT6plex [N-Term; K1; K8]	1.141	0.83	1.245	3.384720564	22.69	1	1691.045903	3	564.354248	1.353145188	0.000762744	50.20749501
																			ALQLEEER	10	21614499	1xTMT6plex [N-Term]	0.905	0.865	0.841	3.430027723	41.19	0	1216.673482	2	608.8400269	-0.579653516	-0.000352625	40.39575263
																			FYPEDVAEELIQDITQK	3	21614499	2xTMT6plex [N-Term; K17]		100		2.541933775	21.53	0	2496.327392	2	1248.668701	1.095082223	0.001366842	113.9676164
																			IGFPWSEIR	4	21614499	1xTMT6plex [N-Term]		0.996	1.093	2.909641981	30.66	0	1333.746588	2	667.3757935	-1.70756271	-0.001138728	82.08487989
																			FVIKPIDK	8	21614499	3xTMT6plex [N-Term; K4; K8]	1.014	0.898	0.786	2.657356024	10.37	0	1647.081226	3	549.6986694	0.139319651	7.65E-05	56.62551159
																			EAQDDLVK	6	21614499	2xTMT6plex [N-Term; K8]	1.198	1.194	100	3.323882818	44.88	0	1375.783316	2	688.3946533	-0.934841526	-0.00064307	43.60825115
																			EGILSDEIYCPPETAVLLGSYAVQAK	2	21614499	2xTMT6plex [N-Term; K26]; 1xCarbamidomethyl [C10				1.356391907	0.94	0	3281.737952	3	1094.581665	-2.288407217	-0.002503318	95.42565125
																			ENPLQFK	10	21614499	2xTMT6plex [N-Term; K7]	1.214	0.567	1.292	2.805651665	23.95	0	1333.788008	2	667.3933105	-6.495204785	-0.004331613	50.09883136
																			EKEELMLR	7	21614499	2xTMT6plex [N-Term; K2]	1.05	0.965	1.085	2.921281338	9.58	1	1505.876171	3	502.6308289	1.170509435	0.000587547	31.90273899
																			LQDYEEK	4	21614499	2xTMT6plex [N-Term; K7]	0.964	1.087		3.019783497	45.65	0	1382.756767	2	691.881958	-0.092333213	-6.38E-05	51.90361745
																			LFFLQVK	2	21614499	2xTMT6plex [N-Term; K7]	0.846			2.326160192	22.22	0	1352.870615	2	676.9376831	-1.866829713	-0.00126279	89.42239087
																			GFPTWLK	2	21614499	2xTMT6plex [N-Term; K7]			1.22	2.277904987	26.91	0	1306.792365	2	653.8956299	-6.413967972	-0.004190862	69.59167059
																			QAVDQIK	2	21614499	2xTMT6plex [N-Term; K7]	0.935			3.25781703	36.44	0	1259.772358	2	630.3895264	-0.461611672	-0.000290763	39.60523974
4507115	FSCN1	fascin	6624	NP_003079.1	49.49290061	1	21	21	172	493	54.496	7.24	1.03	0.72	1.16	0.97	Significant	0.018																
																			KQIWTLEQPPDEAGSAAVCLR	7	4507115	2xTMT6plex [N-Term; K1]; 1xCarbamidomethyl [C19	0.473	100	0.01	4.400589466	30.66	1	2827.517669	3	943.1780396	0.670553668	0.000632001	71.1936381
																			YWTVGSDSAVTSSGDTPVDFFFEFCDYNK	7	4507115	2xTMT6plex [N-Term; K29]; 1xCarbamidomethyl [C25			1.574	4.793609142	34.72	0	3799.751429	4	950.6930542	-0.274355611	-0.000260621	111.0179808
																			QIWTLEQPPDEAGSAAVCLR	6	4507115	1xTMT6plex [N-Term]; 1xCarbamidomethyl [C18			1.798	4.686805725	44.66	0	2470.259774	3	824.0899048	-1.86755036	-0.001537778	77.2774206
																			DVPWGVDSLITLAFQDQR	6	4507115	1xTMT6plex [N-Term]	1.149			5.18033123	55.23	0	2289.207662	3	763.7408447	0.13918776	0.00010621	107.2515342
																			LVARPEPATGYTLEFR	14	4507115	1xTMT6plex [N-Term]	1.139	1.317	1.387	5.045115948	32.35	0	2049.133041	3	683.7166748	1.18597671	0.000810075	61.18899952
																			LSCFAQTVSPAEK	4	4507115	2xTMT6plex [N-Term; K13]; 1xCarbamidomethyl [C3	1.029	100		3.342020273	41.42	0	1896.030105	2	948.5186768	-0.014954602	-1.42E-05	64.53279643
																			DELFALEQSCAQVVLQAANER	2	4507115	1xTMT6plex [N-Term]; 1xCarbamidomethyl [C10			1.904	4.573133945	56.08	0	2620.323831	3	874.1122437	-0.631003294	-0.000551144	100.5911544
																			YLAPSGPSGTLK	8	4507115	2xTMT6plex [N-Term; K12]	1.231	1.173	100	4.150877476	70.09	0	1648.967428	2	824.9860229	-1.612628313	-0.001329586	51.38379396
																			FLIVAHDDGR	26	4507115	1xTMT6plex [N-Term]	0.764	0.409	1.436	3.975438118	66.81	0	1371.758215	3	457.9238586	-0.869200018	-0.000397444	50.7696784
																			VGKDELFALEQSCAQVVLQAANER	6	4507115	2xTMT6plex [N-Term; K3]; 1xCarbamidomethyl [C13		100	100	3.249166489	25.72	1	3133.671604	4	784.1724243	-1.192299273	-0.000934069	94.72510256
																			ASAETVDPASLWEY	6	4507115	1xTMT6plex [N-Term]	1.234	1.371	1.617	2.829653263	64.04	0	1767.863862	2	884.4337769	-2.027599036	-0.00179226	85.6379876
																			LINRPIIVFR	6	4507115	1xTMT6plex [N-Term]	1.402	1.686	4.494	3.97710371	19.47	0	1469.95177	3	490.6520996	-6.819469918	-0.003341431	58.29963918
																			YLTAEAFGFK	4	4507115	2xTMT6plex [N-Term; K10]	1.342	1.996	1.57	3.757421732	36.71	0	1604.908851	2	802.9569092	-1.438879612	-0.001154635	78.62710577
																			GEHGFIGCR	8	4507115	1xTMT6plex [N-Term]; 1xCarbamidomethyl [C8	1.107	1.194	1.139	3.839333057	44.91	0	1261.630906	3	421.2151184	-0.082770159	-3.48E-05	33.39741799
																			VNASASSLK	4	4507115	2xTMT6plex [N-Term; K9]	1.798	0.811		3.719134092	31.29	0	1334.804386	2	667.90625	0.627350351	0.000418695	42.69195561
																			KVTGTLDANR	32	4507115	2xTMT6plex [N-Term; K1]	1.204	0.797	1.202	3.770444393	34.36	1	1532.916062	2	766.961792	0.16031822	0.000122877	34.8286645
																			WSLQSEAHR	4	4507115	1xTMT6plex [N-Term]			0.971	2.966663837	34.19	0	1342.706514	3	448.2404175	0.138072918	6.18E-05	41.34903196
																			VTGTLDANR	10	4507115	1xTMT6plex [N-Term]	2.135	1.789	1.594	3.621121645	36.19	0	1175.658167	2	588.3317261	-1.693622874	-0.000995561	32.66167169
																			GDHAGVLK	2	4507115	2xTMT6plex [N-Term; K8]	1.146			2.867437363	34.27	0	1254.757042	3	418.923645	-0.526110939	-0.000220047	29.65920802
																			YLAADKDGNVTCER	8	4507115	2xTMT6plex [N-Term; K6]; 1xCarbamidomethyl [C12	1.196	1.28	1.162	3.325047731	9.55	1	2070.06901	2	1035.539551	1.359912393	0.001407556	41.48820522
																			YFGGTEDR	2	4507115	1xTMT6plex [N-Term]			1.414	3.214256287	56.14	0	1173.573768	2	587.2901611	-0.615815038	-0.000361352	39.14129679
19913412	MVP	major vault protein isoform 1	9961	NP_005106.2	42.32922732	1	29	29	187	893	99.266	5.48	0.67	0.69	0.66	0.67	Significant	0.00017																
																			IPPYHYIHVLDQNSNVSR	6	19913412	1xTMT6plex [N-Term]	0.553	0.596	0.428	5.561743736	54.53	0	2381.256344	4	596.0697632	0.369147311	0.000219759	53.01664052
																			DITPLQVVLPNTALHLK	8	19913412	2xTMT6plex [N-Term; K17]	1.212	0.842		3.323154688	32.01	0	2330.421173	3	777.4786987	0.158564216	0.000123174	96.0393329
																			VPHNAAVQVYDYR	10	19913412	1xTMT6plex [N-Term]	0.643	0.635	0.62	5.231887341	48.63	0	1760.928134	2	880.9663696	-1.516849062	-0.001335531	42.8264898
																			IEGEGSVLQAK	6	19913412	2xTMT6plex [N-Term; K11]	1.019	0.805	0.758	4.798906326	64.78	0	1588.931043	2	794.9613647	-9.811664189	-0.007795029	48.04734999
																			GAVASVTFDDFHK	12	19913412	2xTMT6plex [N-Term; K13]	0.69	0.691	0.731	5.221153736	75.17	0	1852.000519	3	618.0046997	-0.525663409	-0.00032451	66.75276626
																			ELLELEALSMAVESTGTAK	12	19913412	2xTMT6plex [N-Term; K19]	0.887	0.788	0.936	5.219954967	52.45	0	2450.346413	3	817.4547729	1.368246399	0.001117559	103.4681451
																			KEVEVVEIIQATIIR	12	19913412	2xTMT6plex [N-Term; K1]	0.767	0.682	0.842	6.028686523	45.86	1	2198.352425	3	733.4555054	-0.210301457	-0.000154106	99.87594731
																			AQQLAEVEVK	12	19913412	2xTMT6plex [N-Term; K10]	0.643	0.649	0.699	4.714886665	66.82	0	1572.936128	2	786.9720459	0.436665458	0.000343423	46.54487788
																			ALQPLEEGEDEEK	6	19913412	2xTMT6plex [N-Term; K13]	0.587	0.618	0.468	3.902554512	44.16	0	1945.016623	2	973.0110474	-0.927860161	-0.000902352	52.51202171
																			DAQGLVLFDVTGQVR	6	19913412	1xTMT6plex [N-Term]		0.503		3.262660027	37.35	0	1847.022428	2	924.0144653	-0.418985674	-0.000386938	86.65095432
																			TAVFGFETSEAK	4	19913412	2xTMT6plex [N-Term; K12]	0.802	0.714		4.236855984	32.72	0	1744.952172	2	872.9796143	-0.12628104	-0.000110177	72.93779413
																			AQLELEVSK	4	19913412	2xTMT6plex [N-Term; K9]	0.883	0.749		3.419670105	53.8	0	1474.888116	2	737.947937	0.326705079	0.000240927	50.80320584
																			DLAVAGPEMQVK	6	19913412	2xTMT6plex [N-Term; K12]	0.685	0.703	0.648	3.71510458	43.75	0	1715.976613	2	858.4924927	0.638605185	0.000547916	56.60917983
																			ILDQSEAEK	6	19913412	2xTMT6plex [N-Term; K9]	0.642	0.717		3.963554859	56.17	0	1490.846645	2	745.9262085	-1.008995672	-0.000752129	50.17178727
																			SFFLQPGEQLEQGIQDVYVLSEQQGLLLR	2	19913412	1xTMT6plex [N-Term]				2.559946537	8.6	0	3563.894714	3	1188.633789	-2.216671124	-0.002633327	112.4392349
																			LFSVPDFVGDACK	6	19913412	2xTMT6plex [N-Term; K13]; 1xCarbamidomethyl [C12	1.211	1.277	0.768	2.742534637	36.34	0	1913.024292	2	957.0151978	-0.613025246	-0.000586366	90.45864642
																			ALLDFEDKDGDK	4	19913412	3xTMT6plex [N-Term; K8; K12]	0.756	0.631		3.066931486	14.04	1	2053.142047	3	685.0526733	0.691413209	0.00047319	65.44088208
																			AQALAIETEAELQR	6	19913412	1xTMT6plex [N-Term]	100	0.01	100	2.32401371	28.44	0	1771.975143	2	886.4892578	-2.203397161	-0.001952182	57.93207008
																			LAQDPFPLYPGEVLEK	7	19913412	2xTMT6plex [N-Term; K16]		1.676		2.706487417	18.91	0	2274.278592	2	1137.643433	0.438413473	0.000498537	96.11763304
																			GPLEYVPSAK	7	19913412	2xTMT6plex [N-Term; K10]	0.553	100		2.579087019	23.88	0	1518.893201	2	759.9505005	0.344597344	0.000261703	66.61157499
																			ELELVYAR	4	19913412	1xTMT6plex [N-Term]		0.726	0.542	2.731909513	46.94	0	1221.704054	2	611.3555298	-0.222050247	-0.00013564	66.89102174
																			VVFGPELVSLGPEEQFTVLSLSAGRPK	5	19913412	2xTMT6plex [N-Term; K27]				1.593543649	5.13	0	3314.876434	3	1105.630371	0.037917326	4.19E-05	105.818443
																			QAIPLDENEGIYVQDVK	6	19913412	2xTMT6plex [N-Term; K17]	0.659	1.198	0.385	1.887786746	9.45	0	2389.301512	2	1195.152344	-1.716321686	-0.002050405	61.69694892
																			ALLDFEDK	8	19913412	2xTMT6plex [N-Term; K8]	0.678	0.652		3.423566103	29.69	0	1408.808803	2	704.9088745	1.185223598	0.000834877	86.21087384
																			QMTEAIGPSTIR	2	19913412	1xTMT6plex [N-Term]			0.821	2.288248539	18.65	0	1532.830394	2	766.9211426	3.01075466	0.002307488	50.61067865
																			LLQSLGLK	2	19913412	2xTMT6plex [N-Term; K8]	0.955			2.297981262	45.27	0	1329.886993	2	665.4468384	-0.446144816	-0.000296661	82.57105741
																			ELPPGVEELLNK	8	19913412	2xTMT6plex [N-Term; K12]	0.686	1.044	0.01	1.90166223	12.49	0	1796.056972	2	898.5310059	-1.245250727	-0.001118271	94.13179096
																			VEVVEER	6	19913412	1xTMT6plex [N-Term]	0.691	0.737	0.769	2.825817108	41.71	0	1088.614905	2	544.8110962	0.009978563	5.43E-06	37.71466344
																			HADLEIR	4	19913412	1xTMT6plex [N-Term]		0.818	0.696	3.237542868	40.73	0	1082.615574	2	541.8115234	0.181611095	9.83E-05	31.24323026
17921989	TUBA4A	tubulin alpha-4A chain isoform 1	7277	NP_005991.1	64.73214286	1	5	19	518	448	49.892	5.06	1.28	1.03	0.86	1.06	Significant	0.013																
																			AYHEQLSVAEITNACFEPANQMVK	43	17921989	2xTMT6plex [N-Term; K24]; 1xCarbamidomethyl [C15	1.482	1.325	1.887	4.971693516	40.84	0	3208.617125	3	1070.209961	-0.559651535	-0.000598569	72.51123941
																			NLDIERPTYTNLNR	12	17921989	1xTMT6plex [N-Term]	1.097	1.333	1.458	4.746809006	27.62	0	1948.044954	3	650.0187988	-1.596973221	-0.001036992	44.0613833
																			VGINYQPPTVVPGGDLAK	5	17921989	2xTMT6plex [N-Term; K18]	100	0.709		3.04886651	24.84	0	2283.311289	2	1142.158691	-0.517860839	-0.000591219	80.08766306
																			IHFPLATYAPVISAEK	4	17921989	2xTMT6plex [N-Term; K16]	1.276	1.48		3.60306406	31.69	0	2215.289097	3	739.1016235	0.551009559	0.000406882	81.69282156
																			AFVHWYVGEGMEEGEFSEAR	60	17921989	1xTMT6plex [N-Term]; 1xOxidation [M11]	1.014	1.094	1.093	4.193455219	50.07	0	2575.176104	3	859.0631714	-0.444066657	-0.000381183	77.35264765
																			AFVHWYVGEGMEEGEFSEAR	159	17921989	1xTMT6plex [N-Term]	1.068	1.065	1.124	4.800458431	48.34	0	2559.181189	3	859.0631714	-0.444066657	-0.000381183	77.35264765
																			FDGALNVDLTEFQTNLVPYPR	10	17921989	1xTMT6plex [N-Term]	1.646	1.619	1.724	4.742277622	74.42	0	2638.371433	3	880.1283569	-0.34697132	-0.000305146	104.4415982
																			QLFHPEQLITGK	37	17921989	2xTMT6plex [N-Term; K12]	1.192	1.224	1.303	4.304189682	46.16	0	1869.09984	2	935.0534058	-0.162980359	-0.000152313	66.83108121
																			SIQFVDWCPTGFK	22	17921989	2xTMT6plex [N-Term; K13]; 1xCarbamidomethyl [C8	1.376	1.188	1.163	3.881448746	39.68	0	2043.07739	2	1022.042969	0.622139918	0.00063554	87.09814847
																			AVFVDLEPTVIDEIR	8	17921989	1xTMT6plex [N-Term]	0.804	0.304	0.784	3.076227427	26.34	0	1945.084359	2	973.0457153	-0.105587162	-0.000102688	100.3623402
																			DVNAAIAAIK	6	17921989	2xTMT6plex [N-Term; K10]	0.987	1.359	0.829	3.901872396	51.96	0	1443.893535	2	722.4498901	-0.714503208	-0.000515833	58.44763371
																			LEFSIYPAPQVSTAVVEPYNSILTTHTTLEHSDCAFMVDNEAIYDICR	17	17921989	1xTMT6plex [N-Term]; 2xCarbamidomethyl [C34; C47]				2.73541522	5.3	0	5746.75929	4	1437.44397	-0.912067739	-0.001310358	106.1922846
																			AVCMLSNTTAIAEAWAR	39	17921989	1xTMT6plex [N-Term]; 1xCarbamidomethyl [C3	2.175	1.012	100	2.735594511	25.77	0	2094.067346	2	1047.537109	-0.192968961	-0.000202045	93.62566765
																			EDAANNYAR	14	17921989	1xTMT6plex [N-Term]	1.783	2.081	1.508	3.349599123	46.31	0	1252.611945	2	626.8093872	-0.35677924	-0.000223453	27.54228179
																			AYHEQLSVAEITNACFEPANQMVK	7	17921989	2xTMT6plex [N-Term; K24]; 1xCarbamidomethyl [C15]; 1xOxidation [M22]	0.99		100	2.569916964	7.08	0	3224.61204	3	1070.209961	-0.559651535	-0.000598569	72.51123941
																			EIIDPVLDR	10	17921989	1xTMT6plex [N-Term]	1.34	0.567	0.727	3.260353804	33.62	0	1298.751733	2	649.880127	0.958340396	0.000622323	63.3554707
																			YMACCLLYR	16	17921989	1xTMT6plex [N-Term]; 2xCarbamidomethyl [C4; C5	0.875	0.696	0.789	3.333981514	29.42	0	1478.715564	2	747.8575439	-1.784967331	-0.001334005	48.25769338

Bhat et al,2017. Cigarette smoke and chewing tobacco alter expression of different sets of miRNAs in oral keratinocytes

Supplementary Table S2(A). List of all proteins and peptides identified in OKF6/TERT1 cells chronically treated with chewing tobacco using Sequest and Mascot search algorithms

Accession	Gene_Symbol	Description	Gene_ID	Refseq_Accession	Coverage	Proteins	#Unique_Peptides	#Peptides	#PSMS	#AAs	MW [kDa]	calc. pI	OKF6-STE-6M/OKF6P_R1	OKF6-STE-6M/OKF6P_R2	OKF6-STE-6M/OKF6P_R3	Median fold change	Status	P-value (6M)	Sequence	#PSMs	Protein Accessions	Modifications	OKF6-STE-6M/OKF6P_R1	OKF6-STE-6M/OKF6P_R2	OKF6-STE-6M/OKF6P_R3	Xcorr	Ionscore	# Missed Cleavages	Theo. MH+ [Da]	Charge	m_z	DeltaM [ppm]	Deltam/z [Da]	RT [min]
																			YMACCLLYR	8	17921989	1xTMT6plex [N-Term]; 2xCarbamidomethyl [C4; C5]; 1xOxidation [M2	1.192	1.123	0.892	1.688249826	34.9	0	1494.710479	2	747.8575439	-1.784967331	-0.001334005	48.25769338
																			FDLMYAK	16	17921989	2xTMT6plex [N-Term; K7]	1.655	2.363	1.156	3.340296984	32.15	0	1345.759016	2	673.3817749	-2.037969117	-0.001371308	81.28086024
																			LSVDYGK	14	17921989	2xTMT6plex [N-Term; K7]	1.364	1.45	0.981	3.336693764	38.06	0	1239.73491	2	620.3706055	-0.786621795	-0.000487601	39.27987182
																			LSVDYGKK	2	17921989	3xTMT6plex [N-Term; K7; K8]		0.439		2.610091925	6.99	1	1596.992804	3	533.0023193	-0.250296799	-0.000133241	41.4246211
																			AVCMLSNTTAIAEAWAR	5	17921989	1xTMT6plex [N-Term]; 1xCarbamidomethyl [C3]; 1xOxidation [M4]		100	100	0.970247626	2.39	0	2110.062261	2	1047.537109	-0.192968961	-0.000202045	93.62566765
																			TIGGGDDSFTTFFCETGAGK	2	17921989	2xTMT6plex [N-Term; K20]; 1xCarbamidomethyl [C14				1.398387671		0	2526.222275	2	1263.612915	-1.473303403	-0.001860946	86.59924344
																			FDLMYAK	2	17921989	2xTMT6plex [N-Term; K7]; 1xOxidation [M4]	1.858			2.091995478	14.82	0	1361.753931	2	673.3817749	-2.037969117	-0.001371308	81.28086024
31621305	LRPPRC	leucine-rich PPR motif-containing protein, mitochondrial precurso	10128	NP_573566.2	27.83357245	1	35	35	142	1394	157.805	6.13	1.06	1.05	1.05	1.05	Significant	0.00001																
																			GDVENIEVVQK	6	31621305	2xTMT6plex [N-Term; K11]	1.066	1.099	1.139	4.559975147	68.14	0	1687.963071	2	844.484375	-0.946643933	-0.00079895	47.23638162
																			DLPVTEAVFSALVTGHAR	2	31621305	1xTMT6plex [N-Term]		100		2.935257912	23.94	0	2112.165069	3	704.7250366	-2.136517067	-0.001504226	94.53081426
																			EKDIQEESTFSSR	19	31621305	2xTMT6plex [N-Term; K2]	1.191	1.214	1.082	4.055737019	35.66	1	2014.04932	3	672.0213623	0.106136459	7.13E-05	37.4919189
																			SGGLGGSHALLLLR	2	31621305	1xTMT6plex [N-Term]	1.767			3.672627687	45.15	0	1579.948141	3	527.3210449	0.278860814	0.000146862	61.21549014
																			VIEPQYFGLAYLFR	6	31621305	1xTMT6plex [N-Term]	1.351	1.254		3.394412994	25.58	0	1945.078486	2	973.0428467	-0.035762374	-3.48E-05	109.2020184
																			DIQEESTFSSR	4	31621305	1xTMT6plex [N-Term]	1.454	1.421		4.074452877	43.22	0	1527.748832	2	764.378418	0.476117203	0.000363694	37.62042925
																			YAGEPVPFIEPPESFEFYAQQLR	2	31621305	1xTMT6plex [N-Term]				1.979533672	16.93	0	2943.476626	3	981.8313599	0.985263419	0.0009667	105.1938651
																			DAHLLVESK	6	31621305	2xTMT6plex [N-Term; K9]		1.013	1.06	4.103412151	50.92	0	1469.8728	2	735.4393921	-0.879191936	-0.00064615	41.98508103
																			ALYEHLTAK	8	31621305	2xTMT6plex [N-Term; K9]	0.923	0.884		4.141611576	52.64	0	1503.893535	2	752.449585	-1.091844659	-0.000821009	57.05181469
																			VIEEQLEPAVEK	4	31621305	2xTMT6plex [N-Term; K12]		100		2.291889668	28.86	0	1842.062451	2	921.5355225	0.715177639	0.000658701	53.94809657
																			LVEKGETDLIQK	2	31621305	3xTMT6plex [N-Term; K4; K12]	0.515			3.106016397	9.59	1	2060.257017	3	687.4246216	1.113504808	0.000764702	65.80960879
																			LQDAINILK	8	31621305	2xTMT6plex [N-Term; K9]	100	0.241	100	2.456724167	40.89	0	1485.940485	2	743.4749146	1.391025807	0.001033491	90.2310486
																			TVLDQQQTPSR	2	31621305	1xTMT6plex [N-Term]		0.851		3.354679823	34.69	0	1501.817186	2	751.4133911	1.544326822	0.001159648	34.41267054
																			IQEENVIPR	2	31621305	1xTMT6plex [N-Term]	0.947			3.284152031	32.2	0	1326.757881	2	663.8823242	-0.38350818	-0.000254411	52.15610337
																			VIQALAMK	2	31621305	2xTMT6plex [N-Term; K8]	1.666			3.788589478	46.12	0	1331.848499	2	666.4283447	0.777418331	0.000517702	75.63083168
																			NVQGIIEILK	2	31621305	2xTMT6plex [N-Term; K10]	100			2.287952662	30.32	0	1585.008899	2	793.0083008	0.268498493	0.000212786	96.71154048
																			SVLELIPELNEKEEAYNSLMK	3	31621305	3xTMT6plex [N-Term; K12; K21]				1.702169418	1.26	1	3136.741864	3	1046.249878	-2.162534002	-0.002261104	95.97193802
																			MEEANIQPNR	2	31621305	1xTMT6plex [N-Term]			1.256	3.829446554	34.17	0	1430.725929	2	715.8665161	-0.120871114	-8.65E-05	37.05401388
																			SSLLLGFR	4	31621305	1xTMT6plex [N-Term]	0.708	0.494		2.383630753	28.14	0	1121.68801	2	561.3469849	-1.174304642	-0.000658602	71.31087217
																			KIIETPGIR	6	31621305	2xTMT6plex [N-Term; K1]	0.997	0.79	0.664	3.410514832	19.08	1	1484.95647	3	495.656189	-1.654112776	-0.000818762	49.21042711
																			AFAETHIK	4	31621305	2xTMT6plex [N-Term; K8]	1.205	1.296		3.157403469	27.04	0	1374.814557	3	458.9434814	0.970527939	0.000444765	39.61811614
																			VYLQNEYK	2	31621305	2xTMT6plex [N-Term; K8]		1.366		3.70162034	21.4	0	1514.861901	2	757.9343872	-0.266054574	-0.000201518	46.95268502
																			GETDLIQK	2	31621305	2xTMT6plex [N-Term; K8]			1.239	3.230989695	28.22	0	1361.804052	2	681.4053345	-0.484133298	-0.000329647	48.2365193
																			LDDLFLK	6	31621305	2xTMT6plex [N-Term; K7]	1.252	0.903		2.999599695	37.35	0	1321.81316	2	661.409668	-0.832570392	-0.000550251	91.5645184
																			TVQLTSSELESTLETLK	3	31621305	2xTMT6plex [N-Term; K17]				1.31529808		0	2337.316493	2	1169.160278	-1.374648827	-0.001606495	89.52643247
																			AENQPIRDVLK	2	31621305	2xTMT6plex [N-Term; K11]	0.797			2.016457796	14.25	1	1741.037239	3	581.0170898	-0.300400668	-0.000174336	51.52085351
																			LFECDRDQMYYNLLK	3	31621305	2xTMT6plex [N-Term; K15]; 1xCarbamidomethyl [C4				1.890273809	3.45	1	2466.256159	3	822.7557983	-1.344872655	-0.0011056	75.16022619
																			GMQELGVHPDQETYTDYVIPCFDSVNSAR	2	31621305	1xTMT6plex [N-Term]; 1xCarbamidomethyl [C21				2.153699875	0.65	0	3557.651449	3	1186.551147	-3.530316943	-0.004186546	91.52845613
																			LLAEILR	5	31621305	1xTMT6plex [N-Term]	100	100	100	2.259081364	34.35	0	1056.697847	2	528.8515015	-2.006657172	-0.001060215	76.20457604
																			LIASYCNVGDIEGASK	1	31621305	2xTMT6plex [N-Term; K16]; 1xCarbamidomethyl [C6				1.208467484		0	2155.146926	2	1078.076294	-0.74913193	-0.000807245	63.51258072
																			FSPTDFLAK	6	31621305	2xTMT6plex [N-Term; K9]	100	100	0.76	3.126475096	25.95	0	1483.856087	2	742.4302979	-1.865488775	-0.001384058	77.97121321
																			SCGSLLPELK	2	31621305	2xTMT6plex [N-Term; K10]; 1xCarbamidomethyl [C2				1.774689198	11.64	0	1561.902386	2	781.4544678	-0.465319172	-0.000363392	62.10523484
																			LVELTQK	4	31621305	2xTMT6plex [N-Term; K7]	0.858	1.196		2.819532871	31.55	0	1288.824059	2	644.9145508	-1.733291278	-0.001116954	62.56455216
																			ISIMAER	4	31621305	1xTMT6plex [N-Term]	1.217	1.08		2.467783213	23.77	0	1048.602232	2	524.8048706	0.222001185	0.000116395	44.36199307
																			DILIACR	4	31621305	1xTMT6plex [N-Term]; 1xCarbamidomethyl [C6	1.085	1.186		2.583771706	28.82	0	1089.628781	2	545.3166504	-2.530255072	-0.001378519	56.82561776
148536855	COPA	coatomer subunit alpha isoform 1	1314	NP_001091868.1	30.0081103	1	30	30	133	1233	139.235	7.59	1.22	1.21	1.78	1.4	Significant	0.018																
																			GHYNNVSCAVFHPR	6	148536855	1xTMT6plex [N-Term]; 1xCarbamidomethyl [C8	1.205	1.177		5.749179363	56.63	0	1886.928151	4	472.4875793	0.178455556	8.42E-05	36.10979628
																			YAVTTGDHGIIR	4	148536855	1xTMT6plex [N-Term]	1.306			5.24849844	68.91	0	1531.843007	3	511.2866516	1.563093589	0.000798138	27.65708337
																			KDMSGHYQNALYLGDVSER	2	148536855	2xTMT6plex [N-Term; K1]	1.093			5.235496044	23.22	1	2641.344456	4	661.0914307	-0.213169376	-0.000140763	83.54908179
																			KVQVPNCDEIFYAGTGNLLLR	4	148536855	2xTMT6plex [N-Term; K1]; 1xCarbamidomethyl [C7	1.093	1.04		4.208328247	26.46	1	2865.569705	3	955.8610229	-0.415059645	-0.000396461	88.19888951
																			DMSGHYQNALYLGDVSER	8	148536855	1xTMT6plex [N-Term]	1.255	1.102		5.224385262	44.21	0	2284.086561	3	762.0333252	-0.498463623	-0.000379511	60.42697172
																			LVGQSIIAYLQK	3	148536855	2xTMT6plex [N-Term; K12]	100	100		3.350343704	41.3	0	1791.114427	2	896.0602417	-0.681258296	-0.000610106	98.60459739
																			GVNWAAFHPTMPLIVSGADDR	6	148536855	1xTMT6plex [N-Term]	100	100		3.293040991	33.79	0	2483.270279	3	828.4280396	-0.287482866	-0.000237966	101.0287743
																			GITGVDLFGTTDAVVK	6	148536855	2xTMT6plex [N-Term; K16]			1.003	2.790495634	35.41	0	2051.178878	2	1026.091797	-1.248267534	-0.00128021	85.45880819
																			LLELGPKPEVAQQTR	6	148536855	2xTMT6plex [N-Term; K7]	1.443	1.432		3.746355057	12.55	0	2137.274509	3	713.0958252	-0.742494263	-0.000528971	65.8959354
																			DADSQNPDAPEGK	2	148536855	2xTMT6plex [N-Term; K13]		1.314		3.773777723	48.31	0	1801.896842	2	901.4545288	2.740943362	0.002469449	27.73921022
																			VTTVTEIGK	5	148536855	2xTMT6plex [N-Term; K9]		1.43	1.432	4.290039539	61.63	0	1405.866652	2	703.4321899	-6.791965066	-0.004774299	45.16770875
																			QELILSNSEDK	4	148536855	2xTMT6plex [N-Term; K11]	1.438		1.303	3.979752064	64.37	0	1733.968551	2	867.4824829	-6.263861985	-0.00543067	46.10271871
																			GFFEGTIASK	4	148536855	2xTMT6plex [N-Term; K10]		100	100	2.427668571	39.01	0	1514.861901	2	757.9343262	-0.346636383	-0.000262553	73.48865971
																			GYPEVALHFVK	6	148536855	2xTMT6plex [N-Term; K11]	1.047	1.925	100	2.980628967	31.81	0	1718.004148	3	573.3387451	-1.43552531	-0.000822079	61.32962771
																			DADSITLFDVQQK	4	148536855	2xTMT6plex [N-Term; K13]			100	1.936431646	19.76	0	1938.058428	2	969.5316162	-1.275657168	-0.001236149	69.25628727
																			ERPAYAVHGNMLHYVK	2	148536855	2xTMT6plex [N-Term; K16]			2.616	2.698863268	4.75	0	2343.279611	4	586.5743408	-1.740299677	-0.001019502	40.41916402
																			KYDEVLHMVR	7	148536855	2xTMT6plex [N-Term; K1]	1.234	0.953	100	4.10101366	24.9	1	1747.992932	3	583.3348999	-1.593394869	-0.000928414	47.8500304
																			LLQPPAPIMPLDTNWPLLTVSK	4	148536855	2xTMT6plex [N-Term; K22]			100	2.34529233	19.83	0	2902.688029	3	968.2285156	-5.868799826	-0.005678432	104.5491345
																			MCTLIDKFDEHDGPVR	2	148536855	2xTMT6plex [N-Term; K7]; 1xCarbamidomethyl [C2	100			2.469078302	10.61	1	2391.220107	4	598.5610352	0.921422914	0.000550831	78.44089546
																			ASNLENSTYDLYTIPK	4	148536855	2xTMT6plex [N-Term; K16]			100	1.589723945	12.06	0	2287.222199	2	1144.113403	-1.166816838	-0.001334385	64.30418223
																			SILLSVPLLVVDNK	6	148536855	2xTMT6plex [N-Term; K14]				1.458675742	7.18	0	1968.25092	2	984.626709	-2.428049798	-0.002389506	99.04417492
																			DVAVMQLR	10	148536855	1xTMT6plex [N-Term]	0.863	0.968	0.78	3.018514633	36.31	0	1160.665895	2	580.8370972	0.881456118	0.000511538	48.81666266
																			VLTIDPTEFK	6	148536855	2xTMT6plex [N-Term; K10]	0.946	2.716	100	2.266710997	21.37	0	1620.96128	2	810.9837646	-0.634051619	-0.000513887	71.31499409
																			QLDFNSSK	4	148536855	2xTMT6plex [N-Term; K8]	0.946	0.951		3.591407776	30.24	0	1396.783651	2	698.8950195	-0.635808917	-0.000444044	43.1298918
																			ETIPDIDPNAK	3	148536855	2xTMT6plex [N-Term; K11]	1.128	100		2.408362389	25.54	0	1670.936522	2	835.9707642	-1.358812652	-0.001135245	55.762467
																			VWDISGLR	6	148536855	1xTMT6plex [N-Term]		100		2.97554636	38.24	0	1174.678174	2	587.8425293	-0.333628613	-0.000195953	75.87420896
																			ISPLQFR	2	148536855	1xTMT6plex [N-Term]		1.646		2.163073924	24.22	0	1089.661796	2	545.3339844	-1.012662832	-0.00055173	60.38236145
																			FEEAVEK	4	148536855	2xTMT6plex [N-Term; K7]	1.36	1.703		2.512305975	38.41	0	1309.740389	2	655.3737793	-0.081547649	-5.34E-05	55.22486352
																			ALDDKNCWEK	1	148536855	3xTMT6plex [N-Term; K5; K10]; 1xCarbamidomethyl [C7		0.882		2.287359715		1	1966.067109	3	656.0272217	0.001652569	1.08E-06	54.01189915
																			TLDLPIYVTR	2	148536855	1xTMT6plex [N-Term]		0.01		1.746418238	9.6	0	1419.840882	2	710.4231567	-1.299521278	-0.000922557	65.84191312
66346679	SERBP1	plasminogen activator inhibitor 1 RNA-binding protein isoform 1	26135	NP_001018077.1	46.56862745	1	19	19	128	408	44.938	8.65	1.31	1.28	1.38	1.32	Significant	0.0005																
																			SEEAHAEDSVMDHHFR	4	66346679	1xTMT6plex [N-Term]; 1xOxidation [M11]			1.493	4.352002621	47.78	0	2141.950796	3	709.3233032	-0.246812286	-0.000174904	37.4301169
																			SEEAHAEDSVMDHHFR	8	66346679	1xTMT6plex [N-Term]		1.455	1.575	5.06829977	51.81	0	2125.955881	3	709.3233032	-0.246812286	-0.000174904	37.4301169
																			EAGGGGVGGPGAK	6	66346679	2xTMT6plex [N-Term; K13]	1.656	1.924		4.805525303	68.06	0	1471.826912	2	736.4168701	-0.304835862	-0.000224333	32.59650887
																			SAAQAAAQTNSNAAGK	8	66346679	2xTMT6plex [N-Term; K16]	1.784	1.717		5.277949333	88.23	0	1919.034673	2	960.0214844	0.531089935	0.00050959	32.39673066
																			RPDQQLQGEGK	10	66346679	2xTMT6plex [N-Term; K11]	1.509	1.354	1.438	4.036943436	31.43	0	1713.964803	2	857.4849854	-1.230210137	-0.001054268	33.07602608
																			KNPLPPSVGVVDKK	6	66346679	4xTMT6plex [N-Term; K1; K13; K14]	1.077	1.171	1.32	5.381424427	22.2	2	2394.525418	4	599.3865967	-0.359437091	-0.00021517	44.71572063
																			PGHLQEGFGCVVTNR	7	66346679	1xTMT6plex [N-Term]; 1xCarbamidomethyl [C10	1.35	1.212	1.348		61.81	0	1899.969681	3	633.9940796	-1.050364376	-0.00066522	43.59676412
																			SKSEEAHAEDSVMDHHFR	4	66346679	2xTMT6plex [N-Term; K2]; 1xOxidation [M13]	1.338	1.305		5.657701492	49.19	1	2586.240719	4	647.3167725	1.755646223	0.001135131	30.60546304
																			KEETQPPVALK	6	66346679	3xTMT6plex [N-Term; K1; K11]			1.411	4.564987659	24.29	1	1927.183124	3	643.065979	0.134653379	8.65E-05	45.01974978
																			ENEVEEVKEEGPK	6	66346679	3xTMT6plex [N-Term; K8; K13]	1.087	1.089	1.2	4.218023777	31.73	1	2203.206104	3	735.0732422	-0.422378777	-0.000310196	43.45261972
																			KPNEGADGQWK	7	66346679	3xTMT6plex [N-Term; K1; K11]	1.354	1.389	1.728	4.070802689	22.19	1	1917.079722	3	639.6942139	-6.068338372	-0.003877829	37.76723017
																			EETQPPVALK	4	66346679	2xTMT6plex [N-Term; K10]	2.092			3.848600149	31.45	0	1569.925229	2	785.4660034	-0.317903078	-0.000249542	50.41818241
																			KNPLPPSVGVVDK	3	66346679	3xTMT6plex [N-Term; K1; K13]	0.92		1.653	4.676030636	43.77	1	2037.267523	3	679.7608643	0.25375334	0.000172321	53.94692843
																			SSFSHYSGLK	8	66346679	2xTMT6plex [N-Term; K10]	1.231	1.222	1.088	3.599175453	28.42	0	1570.862963	3	524.2924805	-0.047918725	-2.51E-05	43.95944282
																			ENEVEEVK	4	66346679	2xTMT6plex [N-Term; K8]		1.148	1.502	3.361101151	40.7	0	1433.788795	2	717.3996582	2.262792303	0.001622183	34.59187914
																			SSASAPDVDDPEAFPALA	10	66346679	1xTMT6plex [N-Term]	100			1.705771446	20.77	0	1988.965032	2	994.9855347	-0.623110031	-0.000619672	67.46641552
																			EMTLDEWK	8	66346679	2xTMT6plex [N-Term; K8]	1.389	1.29		3.512017965	37.41	0	1509.802337	2	755.4047852	-0.028710712	-2.17E-05	75.11199099
																			FDQLFDDESDPFEVLK	7	66346679	2xTMT6plex [N-Term; K16]	100			2.191012144	12.77	0	2402.216779	2	1201.61145	-0.48090971	-0.000577625	100.0960454
																			RFEKPLEEK	10	66346679	3xTMT6plex [N-Term; K4; K9]	1.321	1.273	1.262	4.202582836	20.33	1	1863.130695	4	466.5378418	-0.621272841	-0.000289378	44.26072235
																			DELTESPK	2	66346679	2xTMT6plex [N-Term; K8]	1.956			3.27901721	42.76	0	1376.767332	2	688.8867798	-0.761769736	-0.00052439	39.35329712
4757900	CALR	calreticulin precursor	811	NP_004334.1	60.91127098	1	20	20	184	417	48.112	4.44	1.12	1.05	1.08	1.08	Significant	0.00035																
																			KPEDWDEEMDGEWEPPVIQNPEYK	30	4757900	3xTMT6plex [N-Term; K1; K24]	0.636	0.875	0.63	5.122476578	30.42	0	3647.781891	4	912.7020264	1.202119228	0.001096267	80.86743491
																			IDDPTDSKPEDWDKPEHIPDPDAK	6	4757900	4xTMT6plex [N-Term; K8; K14; K24]	1.21		0.939	4.144281387	11.92	0	3676.915237	5	736.1889648	0.130742407	9.61E-05	57.24788415
																			LFPNSLDQTDMHGDSEYNIMFGPDICGPGTK	8	4757900	2xTMT6plex [N-Term; K31]; 1xCarbamidomethyl [C26	1.158	1.204		6.579847336	47.08	0	3914.843966	4	979.4658203	-0.642411299	-0.000628735	94.84380947
																			LFPNSLDQTDMHGDSEYNIMFGPDICGPGTK	12	4757900	2xTMT6plex [N-Term; K31]; 1xCarbamidomethyl [C26]; 1xOxidation [M]		0.974		3.720925331	25.79	0	3930.838881	4	979.4658203	-0.642411299	-0.000628735	94.84380947
																			GTWIHPEIDNPEYSPDPSIYAYDNFGVLGLDLWQVK	2	4757900	2xTMT6plex [N-Term; K36]			0.523	4.162746429	22.11	0	4607.317502	4	1152.584229	-0.524703908	-0.000604369	107.4358178
																			HEQNIDCGGGYVK	6	4757900	2xTMT6plex [N-Term; K13]; 1xCarbamidomethyl [C7	1.461	1.473	1.396	5.274180889	48.37	0	1934.979467	3	645.6655273	1.324118056	0.000854047	41.4048662
																			AKIDDPTDSKPEDWDKPEHIPDPDAK	2	4757900	5xTMT6plex [N-Term; K2; K10; K16; K26]		0.291		2.600269794	5.7	1	4105.210246	6	685.041626	0.761722101	0.000521172	48.78067048
																			GQTLVVQFTVK	15	4757900	2xTMT6plex [N-Term; K11]	0.913			3.296318054	50.63	0	1678.030363	2	839.5169678	-2.207429142	-0.001852067	80.08385284
																			DKGLQTSQDAR	4	4757900	2xTMT6plex [N-Term; K2]		1.078	0.77	4.138816833	23.65	1	1676.933168	3	559.6498413	1.074906153	0.000600849	23.76156114
																			IDNSQVESGSLEDDWDFLPPKK	8	4757900	3xTMT6plex [N-Term; K21; K22]	100			2.948498011	12.94	1	3206.682435	3	1069.563965	-1.588360823	-0.00169779	82.44828252
																			EQFLDGDGWTSR	18	4757900	1xTMT6plex [N-Term]	0.904	1.183	1.083	3.162789583	41.51	0	1639.791365	2	820.3989868	-0.407635509	-0.000334219	58.51075185
																			IDNSQVESGSLEDDWDFLPPK	4	4757900	2xTMT6plex [N-Term; K21]		100		1.814502478	12.56	0	2849.42454	3	950.4782715	-1.501526647	-0.001426162	85.51320897
																			FYALSASFEPFSNK	6	4757900	2xTMT6plex [N-Term; K14]		100	100	2.126210451	12.6	0	2066.099899	2	1033.552368	-1.180635978	-0.001219566	93.16928686
																			LFPNSLDQTDMHGDSEYNIMFGPDICGPGTK	2	4757900	2xTMT6plex [N-Term; K31]; 1xCarbamidomethyl [C26]; 2xOxidation [M11; M20]			1.169	3.238236904	22.44	0	3946.833796	4	979.4658203	-0.642411299	-0.000628735	94.84380947
																			DPDASKPEDWDER	3	4757900	2xTMT6plex [N-Term; K6]	0.936	1.065		3.991962433	4.38	0	2017.98672	3	673.3336792	-0.116607479	-7.84E-05	45.7177389
																			KPEDWDEEMDGEWEPPVIQNPEYK	6	4757900	3xTMT6plex [N-Term; K1; K24]; 1xOxidation [M9	0.599	100		3.400945425	14.23	0	3663.776806	4	912.7020264	1.202119228	0.001096267	80.86743491
																			IKDPDASKPEDWDER	16	4757900	3xTMT6plex [N-Term; K2; K8]	1.083	1.113	1.189	3.881152391	20.73	1	2488.328679	2	1244.665649	-1.871272841	-0.002328171	43.10132291
																			GLQTSQDAR	4	4757900	1xTMT6plex [N-Term]	0.808	100		2.690596819	37.22	0	1204.64833	2	602.8277588	-0.07409787	-4.46E-05	20.50521961
																			VHVIFNYK	5	4757900	2xTMT6plex [N-Term; K8]	1.387	1.347	1.302	2.737730265	22.54	0	1477.893141	3	493.302002	-1.142742041	-0.00056295	49.50189231
																			QIDNPDYK	4	4757900	2xTMT6plex [N-Term; K8]	0.926	1.107		3.241495848	27.98	0	1450.794215	2	725.8997803	-1.331176472	-0.000965632	41.91036193
																			FVLSSGK	15	4757900	2xTMT6plex [N-Term; K7]	1.021	0.795	0.808	2.629771471	32.16	0	1195.74508	2	598.3759155	-0.439797179	-0.000262943	52.80870561
																			GKNVLINK	2	4757900	3xTMT6plex [N-Term; K2; K8]		0.352		2.897108555	13.62	1	1573.040423	3	525.0175781	-1.425440165	-0.000747425	48.10598792
																			LKEEEEDK	6	4757900	3xTMT6plex [N-Term; K2; K8]	0.905	0.757		2.715985775	32.49	1	1706.977942	2	853.9931641	0.649900021	0.000554682	35.98640806
54607053	GCN1	eIF-2-alpha kinase activator GCN1	10985	NP_006827.1	18.00823662	1	39	39	140	2671	292.524	7.43	1.08	1.02	1.07	1.05	Significant	0.00031																
																			LSVADSQAEAK	6	54607053	2xTMT6plex [N-Term; K11]	1.015	1.887	1.253	5.030290127	49.92	0	1576.894657	2	788.9509888	0.02759161	2.18E-05	39.60137988
																			LYRPPPVLDALGR	2	54607053	1xTMT6plex [N-Term]	1.598			4.128397942	28.54	0	1696.010741	3	566.0079346	-0.878950248	-0.000496903	67.07022121
																			AADTQVSETLK	2	54607053	2xTMT6plex [N-Term; K11]		1.4	3.552		47.66	0	1620.920872	2	810.956665	-9.142131568	-0.007409336	41.30510121
																			GDVTDTEQLAQEMLIISHHPSLVAVQSGLWPALLAR	2	54607053	1xTMT6plex [N-Term]				2.656084538	19.03	0	4125.200415	4	1032.053833	-1.675641922	-0.00172809	101.7373711
																			ALADENEFVR	6	54607053	1xTMT6plex [N-Term]	1.152	1.152	1.141	4.476228237	61.65	0	1392.73206	2	696.8686523	-1.458796389	-0.001015856	44.83410907
																			AVMSNLSAHGVK	6	54607053	2xTMT6plex [N-Term; K12]	1.089	0.816	0.806	4.099005222	45.88	0	1671.961632	3	557.9923706	0.55443043	0.000308995	41.5190717
																			GIKEEVQLTSK	5	54607053	3xTMT6plex [N-Term; K3; K11]	0.448	0.988	0.898	4.197563171	37.31	1	1919.178039	3	640.3912964	-9.745291716	-0.006234317	46.3832231
																			GEHVPGFCLPK	4	54607053	2xTMT6plex [N-Term; K11]; 1xCarbamidomethyl [C8	1.06		0.887	3.470909357	35.94	0	1698.940168	3	566.984375	-0.939674057	-0.00053215	60.70746653
																			ALGTLVSHVTLR	4	54607053	1xTMT6plex [N-Term]	1.537	100		3.613199472	36.52	0	1495.915778	3	499.3093262	-1.572912332	-0.000784315	61.20726061
																			EDAGGMIQR	6	54607053	1xTMT6plex [N-Term]	1.268	1.431	1.474	3.493677378	47.26	0	1205.614587	2	603.3109741	0.069949541	4.22E-05	37.84296655
																			EMLQAQLDR	6	54607053	1xTMT6plex [N-Term]	1.003	1.011	1.162	3.36124301	48.5	0	1332.714301	2	666.8591919	-2.396695927	-0.001597055	43.5927802
																			LMDEAVLALR	2	54607053	1xTMT6plex [N-Term]		0.939		2.86056304	24.83	0	1359.786738	2	680.3968506	-0.230475939	-0.000156699	86.45839248
																			LTEVLTDSHVK	2	54607053	2xTMT6plex [N-Term; K11]		0.922		2.152901411	22.99	0	1699.999457	3	567.3376465	-0.629763069	-0.000356866	60.30199289
																			LTSADALRPSVVSITGPLIR	1	54607053	1xTMT6plex [N-Term]	0.01			1.748827457		0	2295.359746	3	765.7908936	-0.705260228	-0.000539609	77.63176893
																			EILSELGK	6	54607053	2xTMT6plex [N-Term; K8]	1.314	0.713	1.194	3.545089722	34.25	0	1346.829538	2	673.9133301	-7.53961319	-0.005077287	62.00275687
																			LMEIYQEK	2	54607053	2xTMT6plex [N-Term; K8]	1.076			3.172611713	32.65	0	1511.854373	2	756.4303589	-0.616108654	-0.000465733	74.67002898
																			AASQSTQVPTITEGVAAALLLLK	2	54607053	2xTMT6plex [N-Term; K23]				2.338968515	14.06	0	2740.622452	3	914.2120361	-0.327141581	-0.000298857	116.0231747
																			LTTPPVNTR	2	54607053	1xTMT6plex [N-Term]		1.541		2.748024702	17.09	0	1227.725852	2	614.3659058	-1.073029911	-0.000658693	41.58595402
																			AYSDQAIVNLLK	4	54607053	2xTMT6plex [N-Term; K12]				1.463501692	20.99	0	1793.057306	2	897.0308228	-1.638052603	-0.001468561	76.50718157
																			DLPEGAVK	4	54607053	2xTMT6plex [N-Term; K8]	1.062	0.902		3.325974464	37.8	0	1286.772023	2	643.8900757	0.661715651	0.000425739	49.48962017
																			DLAPYLPSVTPGLK	5	54607053	2xTMT6plex [N-Term; K14]				1.550332904	10.43	0	1929.146121	2	965.0770264	0.339603293	0.000327572	98.79213637
																			GAAYGLAGLVK	4	54607053	2xTMT6plex [N-Term; K11]	100	100		1.685385942	14.5	0	1477.914271	2	739.4602661	-0.686896025	-0.000507587	68.01270302
																			SLVSGLIR	4	54607053	1xTMT6plex [N-Term]	2.068			2.360854626	32.87	0	1073.68801	2	537.3481445	0.933355398	0.000501066	58.04983269
																			EMDVVSQHK	2	54607053	2xTMT6plex [N-Term; K9]			1.502	3.146991491	26.25	0	1530.835034	3	510.9494934	-0.723236251	-0.000369052	38.64873333
																			LVLPSLLAALEEESWR	2	54607053	1xTMT6plex [N-Term]		1.069		2.465076923	12.07	0	2055.168758	2	1028.086426	-1.548669659	-0.001591389	116.7426
																			LVEVQCLLLLEVLGGSHK	2	54607053	2xTMT6plex [N-Term; K18]; 1xCarbamidomethyl [C6				1.450719595	3.26	0	2465.456573	3	822.4904175	0.051224348	4.21E-05	108.9330683
																			IDPEAFITR	4	54607053	1xTMT6plex [N-Term]	100	100		2.511144876	22.55	0	1290.725518	2	645.8665161	0.184149581	0.000118843	72.98756494
																			KQVFTSEK	2	54607053	3xTMT6plex [N-Term; K1; K8]	1.596			3.341737747	31.17	1	1654.014268	3	552.0090942	-0.930285298	-0.000512902	43.00184986
																			DLILPTIQK	4	54607053	2xTMT6plex [N-Term; K9]	0.9			3.095557451	26.47	0	1498.960887	2	749.9836426	-0.585774958	-0.000439027	73.08746663
																			DAVLYFSESLVPTAR	3	54607053	1xTMT6plex [N-Term]				1.326711059	3.83	0	1897.026845	2	949.015625	-1.513526289	-0.0014356	86.27134087
																			ENVNSLLPVFEEFLK	6	54607053	2xTMT6plex [N-Term; K15]				1.87327373	6.94	0	2236.262941	2	1118.633423	-1.508050244	-0.001686198	109.7860775
																			EEFAIMQTPAGELYDK	2	54607053	2xTMT6plex [N-Term; K16]				1.294657111		0	2300.188456	2	1150.597412	-0.394863842	-0.000454131	82.3344615
																			ALGLVIR	10	54607053	1xTMT6plex [N-Term]	3.102	1.341	1.299	1.663767457	29.44	0	970.6610674	2	485.8333435	-1.707051346	-0.000828484	51.49449934
																			ALGAMVK	2	54607053	2xTMT6plex [N-Term; K7]		0.798		2.928318262	17.96	0	1147.727322	2	574.3674316	0.230752762	0.000132421	47.41886639
																			EDAGGMIQR	4	54607053	1xTMT6plex [N-Term]; 1xOxidation [M6]	0.705	100		1.16017437	4.33	0	1221.609502	2	603.3109741	0.069949541	4.22E-05	37.84296655
																			NPEILAIAPVLLDALTDPSR	2	54607053	1xTMT6plex [N-Term]				1.523529649	1.35	0	2347.343427	2	1174.173462	-1.610429829	-0.001890116	112.0948509
																			TLGDLVR	2	54607053	1xTMT6plex [N-Term]	1.065			2.166297913	12.43	0	1002.614511	2	501.8102417	-1.300860448	-0.000652131	50.66189868
																			GMGFLMR	4	54607053	1xTMT6plex [N-Term]		100	100	1.475701809	21.26	0	1040.558259	2	520.7821045	-1.274523184	-0.000663108	56.76584755
																			ALQAAIQQLAEAQPEATAK	1	54607053	2xTMT6plex [N-Term; K19]				0.876607895		0	2410.370594	2	1205.68811	-0.684632014	-0.000825108	88.20749702
																			NAPNDASYDAVR	1	54607053	1xTMT6plex [N-Term]	1.747			2.632713795	10.5	0	1521.749501	2	761.3778076	-0.76363135	-0.000581028	35.39107499
24307939	CCT5	T-complex protein 1 subunit epsilon isoform a	22948	NP_036205.1	62.66173752	1	27	28	180	541	59.633	5.66	1.09	1.16	0.95	1.07	Significant	0.0033																
																			KQQISLATQMVR	8	24307939	2xTMT6plex [N-Term; K1]	1.152	1.161	0.471	3.957998991	25.59	1	1861.109358	3	621.0413818	0.125681928	7.80E-05	46.13740218
																			QQHVIETLIGK	4	24307939	2xTMT6plex [N-Term; K11]	1.165		1.038	4.299319267	40.76	0	1724.047076	3	575.3540649	0.328212743	0.000188618	52.46829243
																			EMNPALGIDCLHK	2	24307939	2xTMT6plex [N-Term; K13]; 1xCarbamidomethyl [C10]; 1xOxidation [M2]	1.188			4.048911572	41.26	0	1972.039624	3	658.0177612	-0.453352246	-0.00029801	53.26804002
																			EMNPALGIDCLHK	12	24307939	2xTMT6plex [N-Term; K13]; 1xCarbamidomethyl [C10	1.789	1.556	1.412	4.126145363	38.64	0	1956.044709	3	658.0177612	-0.453352246	-0.00029801	53.26804002
																			AFADALEVIPMALSENSGMNPIQTMTEVR	4	24307939	1xTMT6plex [N-Term]	100			3.465659857	44.52	0	3364.678849	3	1122.229492	-1.464031999	-0.001641999	111.5591725
																			MMVDKDGDVTVTNDGATILSMMDVDHQIAK	2	24307939	3xTMT6plex [N-Term; K5; K30]		1.116		6.390100479	24.61	1	3937.998642	4	985.2539063	-1.230686052	-0.00121161	93.87060239
																			VVYGGGAAEISCALAVSQEADK	4	24307939	2xTMT6plex [N-Term; K22]; 1xCarbamidomethyl [C12				3.821726799	51.7	0	2653.390738	3	885.1352539	0.177466795	0.000156963	82.76744887
																			QQISLATQMVR	6	24307939	1xTMT6plex [N-Term]		1.616	2.036	3.16447401	37.85	0	1503.851463	2	752.4296265	0.341070711	0.00025646	57.81038684
																			DGDVTVTNDGATILSMMDVDHQIAK	2	24307939	2xTMT6plex [N-Term; K25]	100			2.660984039	21.02	0	3104.564421	3	1035.525879	-0.430711388	-0.000445724	92.32264319
																			QMAEIAVNAVLTVADMER	4	24307939	1xTMT6plex [N-Term]	100	1.479		2.98622632	27.89	0	2190.14599	2	1095.57666	0.024483528	2.68E-05	115.1534871
																			SIHDALCVIR	44	24307939	1xTMT6plex [N-Term]; 1xCarbamidomethyl [C7	1.004	0.987	1.016	3.972945213	50.61	0	1412.788135	2	706.8984375	1.035647146	0.000731575	52.08500621
																			LDVTSVEDYK	10	24307939	2xTMT6plex [N-Term; K10]	1.358	2.459	2.537	3.766227484	50.23	0	1626.899074	2	813.9538574	0.838499309	0.000682077	72.78451965
																			GVIVDKDFSHPQMPK	5	24307939	3xTMT6plex [N-Term; K6; K15]; 1xOxidation [M13	1.2	1.125	1.131	3.727318764	14.63	1	2401.351663	4	601.0932007	-0.287457116	-0.000172571	50.08764855
																			DTEPLIQTAK	2	24307939	2xTMT6plex [N-Term; K10]			1.697	4.046461582	50.08	0	1573.920144	2	787.4637451	0.044293464	3.49E-05	50.6257895
																			HKLDVTSVEDYK	6	24307939	3xTMT6plex [N-Term; K2; K12]	0.872	0.972	0.949	4.14443922	31.77	1	2121.215881	3	707.7424316	-1.479890922	-0.001046389	50.42586681
																			IAILTCPFEPPKPK	8	24307939	3xTMT6plex [N-Term; K12; K14]; 1xCarbamidomethyl [C6	100	100	100	2.554934025	15.54	0	2298.386258	3	766.7992554	-1.324788951	-0.001014959	74.72519101
																			DVDFELIK	6	24307939	2xTMT6plex [N-Term; K8]	1.135	0.732		3.279173613	44.49	0	1436.840103	2	718.9223022	-1.931250253	-0.001387449	82.9401854
																			LMGLEALK	8	24307939	2xTMT6plex [N-Term; K8]	1.599	1.236	2.463	3.608453512	42.1	0	1332.832515	2	666.9197388	-0.235701736	-0.000157075	67.36721081
																			WVGGPEIELIAIATGGR	9	24307939	1xTMT6plex [N-Term]		1.021	1.493	2.503353596	19.66	0	1968.111577	2	984.5593872	-0.040371663	-3.97E-05	109.5510875
																			ISDSVLVDIKDTEPLIQTAK	4	24307939	3xTMT6plex [N-Term; K10; K20]	100			2.704121351	20.06	1	2872.685001	3	958.2334595	0.286901616	0.000274726	91.87785757
																			SQDDEIGDGTTGVVVLAGALLEEAEQLLDR	2	24307939	1xTMT6plex [N-Term]				1.695706367	3.26	0	3342.711389	3	1114.907715	-0.837065611	-0.00093269	114.3669249
																			ETGANLAICQWGFDDEANHLLLQNNLPAVR	2	24307939	1xTMT6plex [N-Term]; 1xCarbamidomethyl [C9				1.934850693	4.42	0	3608.81173	3	1203.609741	0.814872881	0.000980241	99.19128023
																			MLVIEQCK	8	24307939	2xTMT6plex [N-Term; K8]; 1xCarbamidomethyl [C7	1.448	1.492		3.595707178	37.16	0	1478.847513	2	739.9281616	1.036727704	0.000766581	61.11845576
																			FSELTAEK	4	24307939	2xTMT6plex [N-Term; K8]	1.064		1.112	2.980955362	21.98	0	1382.793153	2	691.9000244	-0.275082267	-0.000190191	63.46092175
																			ISDSVLVDIK	4	24307939	2xTMT6plex [N-Term; K10]				2.304577112	27.63	0	1546.94563	2	773.975769	-0.884920605	-0.000684462	75.0205266
																			VVYGGGAAEISCALAVSQEADKCPTLEQYAMR	3	24307939	2xTMT6plex [N-Term; K22]; 2xCarbamidomethyl [C12; C23				2.657403946	5.24	1	3902.9491	4	976.4935303	0.81754429	0.000797708	90.00512463
																			LMVELSK	13	24307939	2xTMT6plex [N-Term; K7]	1.072	2.391	100	3.146930933	37.43	0	1277.790316	2	639.3983765	-0.657079884	-0.000419805	59.45550323
																			DFSHPQMPK	4	24307939	2xTMT6plex [N-Term; K9]	1.203	1.25		2.553596973	18.81	0	1544.829555	3	515.6138306	-1.69366308	-0.00087214	46.81707946
																			AVTIFIR	11	24307939	1xTMT6plex [N-Term]	1.392	2.918	2.041	1.900840282	25.12	0	1048.671632	2	524.8390503	-0.77055013	-0.000404027	60.27344493
																			KQQISLATQMVR	1	24307939	2xTMT6plex [N-Term; K1]; 1xOxidation [M10]	0.534			1.605457306	2.45	1	1877.104273	3	621.0413818	0.125681928	7.80E-05	46.13740218
157389005	CAPN2	calpain-2 catalytic subunit isoform 1	824	NP_001739.2	51.28571429	1	25	25	196	700	79.959	4.98	0.96	0.96	0.9	0.94	Significant	0.00045																
																			RPTEICADPQFIIGGATR	6	157389005	1xTMT6plex [N-Term]; 1xCarbamidomethyl [C6	100	1.015	0.658	5.073363304	23.13	0	2231.180402	3	744.3986816	0.488361172	0.000363207	58.10650031
																			GSLLGCSIDITSAADSEAITFQK	14	157389005	2xTMT6plex [N-Term; K23]; 1xCarbamidomethyl [C6	1.738	1.175	100	3.773146868	43.35	0	2842.490845	3	948.1687012	0.247964079	0.000234945	91.18767521
																			IMVDMLDSDGSGK	10	157389005	2xTMT6plex [N-Term; K13]	2.996	100	100	2.883499384	57.76	0	1825.943992	2	913.4760132	0.414956423	0.000378844	97.50634617
																			KADYQAVDDEIEANLEEFDISEDDIDDGFR	18	157389005	2xTMT6plex [N-Term; K1]	100	100		3.734357119	28.4	1	3934.839452	4	984.4639893	-1.353201017	-0.001331157	98.92852719
																			LEEEDEDEEDGESGCTFLVGLIQK	8	157389005	2xTMT6plex [N-Term; K24]; 1xCarbamidomethyl [C15	0.88	0.885	1.585	3.459482908	35.61	0	3199.524055	3	1067.178101	-1.346091517	-0.001435617	97.46405948
																			KMGEDMHTIGFGIYEVPEELSGQTNIHLSK	2	157389005	3xTMT6plex [N-Term; K1; K30]	0.623			5.527040482	16.28	1	4048.112684	5	810.4278564	-0.619553761	-0.000501605	82.88157812
																			KADYQAVDDEIEANLEEFDISEDDIDDGFRR	2	157389005	2xTMT6plex [N-Term; K1]		1.025		4.324053288	24.43	2	4090.940563	4	1023.489624	-0.952476867	-0.000974132	94.96801166
																			LFAQLAGEDAEISAFELQTILR	13	157389005	1xTMT6plex [N-Term]			2.479	4.024168968	58.97	0	2664.444598	3	888.819519	-0.222939931	-0.000198004	112.7328598
																			YLNQDYEALR	9	157389005	1xTMT6plex [N-Term]	0.954	1.231	1.348	3.743254662	51.33	0	1513.784824	2	757.3964233	0.493114791	0.000373235	58.09963493
																			ADYQAVDDEIEANLEEFDISEDDIDDGFRR	2	157389005	1xTMT6plex [N-Term]				4.249469757	19.66	1	3733.682668	3	1245.231323	-0.870803367	-0.001083768	104.3279136
																			SDGFSIETCK	6	157389005	2xTMT6plex [N-Term; K10]; 1xCarbamidomethyl [C9	100	100	1.544	3.657151222	58.48	0	1601.824529	2	801.4158325	-0.087931565	-7.04E-05	45.27926879
																			MPCQLHQVIVAR	12	157389005	1xTMT6plex [N-Term]; 1xCarbamidomethyl [C3	1.233	0.912	1.076	4.937322617	45.04	0	1680.923917	3	560.9793091	-0.323116294	-0.000181045	44.76280627
																			EAAEGLGSHER	6	157389005	1xTMT6plex [N-Term]	1.367	1.054	1.131	2.39638567	24.46	0	1384.701822	3	462.2384338	-0.775615595	-0.000357999	30.91837623

Bhat et al,2017. Cigarette smoke and chewing tobacco alter expression of different sets of miRNAs in oral keratinocytes

Supplementary Table S2(A). List of all proteins and peptides identified in OKF6/TERT1 cells chronically treated with chewing tobacco using Sequest and Mascot search algorithms

Accession	Gene_Symbol	Description	Gene_ID	Refseq_Accession	Coverage	Proteins	#Unique_Peptides	#Peptides	#PSMS	#AAs	MW [kDa]	calc. pI	OKF6-STE-6M/OKF6P_R1	OKF6-STE-6M/OKF6P_R2	OKF6-STE-6M/OKF6P_R3	Median fold change	Status	P-value (6M)	Sequence	#PSMSs	Protein Accessions	Modifications	OKF6-STE-6M/OKF6P_R1	OKF6-STE-6M/OKF6P_R2	OKF6-STE-6M/OKF6P_R3	Xcorr	Ionscore	# Missed Cleavages	Theo. MH+ [Da]	Charge	m_z	DeltaM [ppm]	Deltam/z [Da]	RT [min]
																			NECLEAGTLFQDPSFPAIPSALGFK	4	157389005	2xTMT6plex [N-Term; K25]; 1xCarbamidomethyl [C3				2.344319582	22.94	0	3167.648743	3	1056.550049	-4.151270135	-0.004383255	106.2518604
																			KPPPNLFK	17	157389005	3xTMT6plex [N-Term; K1; K8]	0.91	0.888	0.823	4.002175808	43.72	0	1628.05026	3	543.3543091	-1.158338875	-0.000628611	49.28330727
																			DGELLFVHSAEGSEFWSALLEK	2	157389005	2xTMT6plex [N-Term; K22]				3.1699543	18.62	0	2922.528945	3	974.8469238	-0.933091058	-0.000908995	108.4146369
																			FADDQLIIDFDNFVR	6	157389005	1xTMT6plex [N-Term]		100	100	2.490626097	24.37	0	2057.054122	2	1029.030884	0.179410994	0.000184529	103.0689721
																			LEICNLTPDTLTSDTYKK	6	157389005	3xTMT6plex [N-Term; K17; K18]; 1xCarbamidomethyl [C4		0.01	0.01	2.41772747	15.3	1	2799.541708	3	933.8500977	-2.131860095	-0.00198941	70.02259788
																			NPWGEVEWTGR	8	157389005	1xTMT6plex [N-Term]	1.377	100	100	2.540156364	22.1	0	1559.780407	2	780.3915405	-2.950732867	-0.002301248	70.24307313
																			ALEEAGFK	14	157389005	2xTMT6plex [N-Term; K8]	1.015	0.999	1.254	3.862087488	50.85	0	1322.772023	2	661.8904419	1.197408952	0.00079195	47.44200526
																			KALEEAGFK	7	157389005	3xTMT6plex [N-Term; K1; K9]	0.898	1.084		3.915718079	9.32	1	1680.029918	2	840.5184937	-0.123536676	-0.000103773	44.85348735
																			MPCQLHQVIVAR	4	157389005	1xTMT6plex [N-Term]; 1xCarbamidomethyl [C3]; 1xOxidation [M1]	1.137		0.937	3.446757317	24.72	0	1696.918832	3	560.9793091	-0.323116294	-0.000181045	44.76280627
																			SDTFINLR	7	157389005	1xTMT6plex [N-Term]	0.986	1.086	0.877	3.086882353	24.33	0	1194.668003	2	597.8380737	0.726361579	0.00043388	51.67558514
																			EFYILWTK	9	157389005	2xTMT6plex [N-Term; K8]	100	2.636	1.039	2.337621927	19.18	0	1557.908123	2	779.4559937	-2.190177497	-0.001706048	84.39589265
																			RHEDGEFWMSFSDFLR	2	157389005	1xTMT6plex [N-Term]				2.726818085	14.42	1	2288.075602	3	763.3637695	0.503914184	0.000384331	91.80011458
																			NYPNTFWMNPQYLIK	2	157389005	2xTMT6plex [N-Term; K15]	0.01			1.143106341		0	2387.26223	2	1194.132202	-2.137315734	-0.002551167	90.50879852
4502101	ANXA1	annexin A1	301	NP_000691.1	65.31791908	1	20	20	331	346	38.69	7.02	0.79	0.79	0.75	0.78	Significant	0.00029																
																			QAWFIENEEQEYVQTVK	13	4502101	2xTMT6plex [N-Term; K17]	0.607	0.881	0.723	4.036955357	33.18	0	2599.344439	3	867.1173706	-2.647014951	-0.002293501	79.03474273
																			AAYLQETGKPLDETLKK	16	4502101	4xTMT6plex [N-Term; K9; K16; K17]	0.884	0.716	0.362	4.009245396	19.36	1	2821.684497	4	706.1765137	-0.09634759	-6.80E-05	52.35945282
																			GTDVNVFNTILTTR	23	4502101	1xTMT6plex [N-Term]	0.814	0.936	0.963	2.941754818	49.71	0	1779.980229	2	890.493042	-0.798551356	-0.000710703	78.19427516
																			AAYLQETGKPLDETLK	8	4502101	3xTMT6plex [N-Term; K9; K16]	0.821	0.841	1.378	4.435410023	34.34	0	2464.426602	3	822.1463623	-0.839159071	-0.000689349	67.9546298
																			TPAQFDADELR	18	4502101	1xTMT6plex [N-Term]	0.709	0.772	0.717	4.777585506	64.73	0	1491.764088	2	746.3859863	0.407508302	0.000303953	53.83076837
																			GLGTDEDTLIEILASR	45	4502101	1xTMT6plex [N-Term]	0.676	1.192	0.669	5.170063496	73.71	0	1932.048702	3	644.6871338	-0.959609771	-0.000618004	99.81819113
																			GVDEATIIDILTK	50	4502101	2xTMT6plex [N-Term; K13]	0.703	0.705	0.625	4.534866333	65.41	0	1846.093751	2	923.5499878	-0.569881168	-0.000526027	102.0720772
																			ALTGHLEEVVLALLK	22	4502101	2xTMT6plex [N-Term; K15]	0.818	0.777		5.370408535	43.55	0	2064.283283	2	1032.644287	-0.96182596	-0.000992741	111.6557923
																			GGPGSAVSPYPTFNPSSDVAALHK	11	4502101	2xTMT6plex [N-Term; K24]	1.213	1.115	1.028	6.06107235	55.35	0	2814.482664	3	938.8320313	-0.399107095	-0.000374427	70.29793276
																			SEDFGVNEDLADSDAR	26	4502101	1xTMT6plex [N-Term]	0.895	0.941		4.762748718	95.21	0	1968.898409	2	984.9536133	0.782631797	0.000770461	55.97815277
																			DITSDTSGDFR	17	4502101	1xTMT6plex [N-Term]	0.699	0.733	0.786	4.500157833	56.51	0	1442.696068	2	721.8520508	0.524589009	0.000378411	47.54903191
																			MYGISLCQAILDETKGDYEK	4	4502101	3xTMT6plex [N-Term; K15; K20]; 1xCarbamidomethyl [C7			0.412	3.994183779	21.93	1	3021.588006	3	1007.865906	-1.60250664	-0.001614038	92.60890177
																			CATSKPAFFAEK	4	4502101	3xTMT6plex [N-Term; K5; K12]; 1xCarbamidomethyl [C1	0.759		0.942	2.995489359	9.3	0	2044.150444	3	682.0546875	-0.457397841	-0.000311663	53.05873867
																			MYGISLCQAILDETK	6	4502101	2xTMT6plex [N-Term; K15]; 1xCarbamidomethyl [C7				1.759827852	8.87	0	2200.175783	2	1100.5896	-1.754619462	-0.001930236	86.10166459
																			NALLSLAK	4	4502101	2xTMT6plex [N-Term; K8]		0.887	1.001	3.306978464	46.6	0	1287.840043	2	644.4234619	-0.307547414	-0.000198036	74.75961122
																			SEIDMNDIK	8	4502101	2xTMT6plex [N-Term; K9]	1.062	0.97	0.976	3.702710867	51.46	0	1522.818715	2	761.9057007	-9.581299905	-0.007295291	49.25530785
																			KGTDVNVFNTILTTR	5	4502101	2xTMT6plex [N-Term; K1]	100	1.062	1.14	3.421294212	20.38	1	2137.238124	3	713.0844727	0.34670388	0.000246996	78.38579511
																			ALYEAGER	7	4502101	1xTMT6plex [N-Term]	0.693	0.677	0.612	3.156483889	36.35	0	1137.610154	2	569.3086548	-0.106292729	-6.05E-05	37.0480125
																			VLDLELK	36	4502101	2xTMT6plex [N-Term; K7]	1.541	0.793	1.086	3.154746771	46.06	0	1287.82881	2	644.4188843	1.306116679	0.000841027	2.671679101
																			CLTAIVK	3	4502101	2xTMT6plex [N-Term; K7]; 1xCarbamidomethyl [C1		0.776	0.855	3.19401145	37.53	0	1262.790651	2	631.8981934	-1.219918162	-0.000770251	49.14283001
																			SEIDMNDIK	5	4502101	2xTMT6plex [N-Term; K9]; 1xOxidation [M5]	0.933			3.053142309	27.66	0	1538.81363	2	761.9057007	-9.581299905	-0.007295291	49.25530785
100913206	DHX9	ATP-dependent RNA helicase A	1660	NP_001348.2	28.42519685	1	27	27	151	1270	140.869	6.84	1.14	1.47	1.59	1.4	Significant	0.0091																
																			AAMEALVVEVTK	6	100913206	2xTMT6plex [N-Term; K12]	100		0.01	3.468012333	48.08	0	1719.012664	2	860.0106812	0.827006524	0.000710817	88.11952268
																			GISHVIVDEIHER	16	100913206	1xTMT6plex [N-Term]	1.27	1.455	1.654	4.454807281	76.25	0	1732.954348	2	866.9804077	-0.46718502	-0.000404805	45.32398562
																			AENNSEVGASGYGVPGPTWDR	10	100913206	1xTMT6plex [N-Term]		1.104		5.676305771	49.72	0	2392.136682	3	798.050354	-0.072457516	-5.78E-05	60.4269188
																			DKDDDGGEDDDANCLICGDEYGPETR	6	100913206	2xTMT6plex [N-Term; K2]; 2xCarbamidomethyl [C14; C18		0.54		5.698766232	45.66	1	3503.485121	3	1168.497925	-1.683867844	-0.001966469	58.66484924
																			KFESEILEAISQNSVVIIR	6	100913206	2xTMT6plex [N-Term; K1]	2.601	1.633	1.455	4.36283493	38.06	1	2633.527823	3	878.5143433	0.248110216	0.000217802	106.289252
																			KEEQEVQATLESEEVDLNAGLHGNWTLENAK	2	100913206	3xTMT6plex [N-Term; K1; K31]				2.643348217	26.04	1	4169.160556	4	1043.045166	-0.413044179	-0.000430512	96.45233612
																			AAECNIVVTQPR	9	100913206	1xTMT6plex [N-Term]; 1xCarbamidomethyl [C4	0.796	1.546		4.142699242	72.23	0	1586.852192	2	793.9301147	0.479529342	0.000380471	37.10612409
																			LQISHEAAACITGLR	4	100913206	1xTMT6plex [N-Term]; 1xCarbamidomethyl [C10	100		3.558	3.361911058	33.58	0	1869.021382	3	623.6784058	-0.384348114	-0.000239452	52.82112598
																			KDKDDDGGEDDDANCLICGDEYGPETR	2	100913206	3xTMT6plex [N-Term; K1; K3]; 2xCarbamidomethyl [C15; C19			0.856	6.347329617	35.86	2	3860.743016	4	965.9394531	-1.821646759	-0.001758228	43.40972084
																			TPLHEIALSIK	6	100913206	2xTMT6plex [N-Term; K11]	1.361	1.519	0.981	3.786180735	33.62	0	1680.046013	3	560.6876221	1.368915446	0.000766614	63.26610945
																			KVFDPVPVGVTK	2	100913206	3xTMT6plex [N-Term; K1; K12]	1.176			2.633720636	24.43	1	1973.240245	3	658.4178467	-0.6377586	-0.000419484	53.61634255
																			NELMYQLEQDHDLQAILQER	2	100913206	1xTMT6plex [N-Term]	100			2.451462507	13.79	0	2715.360945	3	905.791748	-0.093427496	-8.46E-05	97.64191235
																			QLYHLGVVEAYSGLTK	5	100913206	2xTMT6plex [N-Term; K16]	0.661	0.788	0.01	2.928250074	17.05	0	2236.274175	3	746.0964355	0.258693962	0.000192837	77.7533483
																			YQILPLHSQIPR	6	100913206	1xTMT6plex [N-Term]		1.899	1.264	2.732322693	23.95	0	1693.995091	3	565.3369141	0.64807006	0.000365943	60.71209538
																			ISAVSVAER	8	100913206	1xTMT6plex [N-Term]	1.531	1.626	1.547	3.305867434	38.33	0	1160.683653	2	580.845459	-0.010158883	-5.90E-06	39.89357574
																			ELDALDANDELTPLGR	4	100913206	1xTMT6plex [N-Term]	100	100		2.48371315	18.18	0	1971.023216	2	986.0143433	-0.916141701	-0.000902868	73.84306358
																			LAAQSCALSLVR	6	100913206	1xTMT6plex [N-Term]; 1xCarbamidomethyl [C6	100		100	1.726126432	30.25	0	1517.867114	2	759.4368896	-0.402593296	-0.000305542	54.96544125
																			AIEPPPLDAVIEAEHTLR	10	100913206	1xTMT6plex [N-Term]	1.139	0.952	1.211	2.106303692	19.62	0	2200.217499	3	734.0771484	-0.275730695	-0.000202222	76.93260294
																			EGETVEPYK	4	100913206	2xTMT6plex [N-Term; K9]	1.367	1.292		3.031065702	29.37	0	1509.820096	2	755.4129639	-0.956687243	-0.000722213	45.61666214
																			MGGEEAEIR	4	100913206	1xTMT6plex [N-Term]		2.39	1.293	2.83858633	27.52	0	1220.614253	2	610.8105469	-0.357025161	-0.000217895	34.78237105
																			LETHMTPEMFR	8	100913206	1xTMT6plex [N-Term]	1.748	1.905	1.809	3.053349257	24.14	0	1620.80755	3	540.9440308	6.163208678	0.003329792	55.14707793
																			DFVNYLVR	10	100913206	1xTMT6plex [N-Term]	0.433	0.987	1.423	2.841356516	42.06	0	1254.704389	2	627.8555298	-0.482703092	-0.000302825	77.30810536
																			QPAIISQLDPVNER	5	100913206	1xTMT6plex [N-Term]	100	100	100	1.381933928	3.54	0	1809.006778	2	905.0089111	2.082792431	0.001883893	65.05246659
																			DVVQAYPEVR	2	100913206	1xTMT6plex [N-Term]	1.72			3.478131056	14.89	0	1404.768445	2	702.8874512	-0.583424445	-0.000409788	49.32881917
																			VFDPVPVGVTK	4	100913206	2xTMT6plex [N-Term; K11]		0.01	100	1.601021409	14.53	0	1615.98235	2	808.4940796	-0.908252687	-0.00073386	61.57179456
																			GMTLVTPLQLLLFASK	2	100913206	2xTMT6plex [N-Term; K16]				1.302348137		0	2190.333604	2	1095.668945	-1.3651231	-0.001495038	112.7309173
																			GATGCGK	2	100913206	2xTMT6plex [N-Term; K7]; 1xCarbamidomethyl [C5				1.984964013	19.71	0	1108.6185	2	554.8131104	0.40062756	0.000222072	23.11191176
52632383	HNRNPL	heterogeneous nuclear ribonucleoprotein L isoform a	3191	NP_001524.2	51.44312394	1	15	16	108	589	64.092	8.22	1.31	1.29	1.37	1.32	Significant	0.00033																
																			SKPGAAMVEMADGYAVDR	8	52632383	2xTMT6plex [N-Term; K2]	1.239	1.073	0.976	4.061088085	25.72	0	2326.193558	3	781.4006958	-0.400850601	-0.000312956	45.84306472
																			QPAIMPGQSYGLEDGSCSYK	7	52632383	2xTMT6plex [N-Term; K20]; 1xCarbamidomethyl [C17	1.469	1.296		3.249799967	37.61	0	2646.294394	3	882.7687988	-0.964055863	-0.000850392	60.29756708
																			SKPGAAMVEMADGYAVDR	8	52632383	2xTMT6plex [N-Term; K2]; 1xOxidation [M7]	1.159	1.177	1.185	4.46728754	28.34	0	2342.188473	3	781.4006958	-0.400850601	-0.000312956	45.84306472
																			TDNAGDQHGGGGGGGGGAGAAGGGGGGENYDDPHK	16	52632383	2xTMT6plex [N-Term; K35]	1.511	1.814	2.096	6.204677582	78.5	0	3439.528397	4	860.6365356	-1.187549979	-0.001021153	25.44862381
																			GLIDGVVEADLVEALQEFGPISYVVVMPK	2	52632383	2xTMT6plex [N-Term; K29]; 1xOxidation [M27		0.995		3.434445143	32.8	0	3561.95303	3	1187.989014	-0.152215476	-0.000180728	113.0838349
																			GLIDGVVEADLVEALQEFGPISYVVVMPK	11	52632383	2xTMT6plex [N-Term; K29]	100	1.131	1.093	3.629524231	26.3	0	3545.958115	3	1187.989014	-0.152215476	-0.000180728	113.0838349
																			LKTDNAGDQHGGGGGGGGGAGAAGGGGGGENYDDPHK	2	52632383	3xTMT6plex [N-Term; K2; K37]	1.126			7.498108387	21.02	1	3909.870356	5	782.7792358	-0.839800424	-0.000656702	27.60247807
																			QALVEFEDVLGACNAVNYAADNQIYIAGHPAFVNYSTSQK	2	52632383	2xTMT6plex [N-Term; K40]; 1xCarbamidomethyl [C13			1.489	3.831350327	24.08	0	4846.418689	5	970.0844727	-5.247670575	-0.005086482	104.3608262
																			SDALETLGFLNHYQMK	6	52632383	2xTMT6plex [N-Term; K16]	2.095	100		3.361161947	36.02	0	2325.231324	3	775.7481079	-0.668006454	-0.000517757	84.3455665
																			QPPLLGDHPAEYGGPHGGYHSHYHDEGYGPPPPHYEGR	4	52632383	1xTMT6plex [N-Term]		1.098	1.337	6.10855484	19.85	0	4404.02333	5	881.6114502	1.093242746	0.000962933	37.84411908
																			IQHPSNVLHFFNAPLEVTEENFFEICDELGVK	6	52632383	2xTMT6plex [N-Term; K32]; 1xCarbamidomethyl [C26	100		1.049	3.282319307	14.71	0	4231.157436	4	1058.544434	-0.362069959	-0.000382994	110.0545027
																			AITHLNNNFMFGQK	9	52632383	2xTMT6plex [N-Term; K14]	1.625	1.837	0.719	3.950695038	32.09	0	2093.136636	3	698.3828125	-1.314496127	-0.00091714	55.89589385
																			YYGGGSEGGR	6	52632383	1xTMT6plex [N-Term]	1.327	100	1.532	2.961874723	39.72	0	1231.590481	2	616.2983398	-0.875244261	-0.000538971	31.18365507
																			MAAAGGGGGGGR	4	52632383	1xTMT6plex [N-Term]	100		1.756	2.557200432	33.69	0	1147.583956	2	574.2958374	0.385396367	0.000221137	18.46110295
																			LCFSTAQHAS	6	52632383	1xTMT6plex [N-Term]; 1xCarbamidomethyl [C2	1.525	1.291	1.329	2.879874706	37.27	0	1350.667351	2	675.8366699	-0.95374057	-0.000644093	35.16433389
																			SDALETLGFLNHYQMK	1	52632383	2xTMT6plex [N-Term; K16]; 1xOxidation [M15					16.08	0	2341.226239	3	775.7481079	-0.668006454	-0.000517757	84.3455665
																			AITHLNNNFMFGQK	2	52632383	2xTMT6plex [N-Term; K14]; 1xOxidation [M10				1.997162104	3.12	0	2109.131551	3	698.3828125	-1.314496127	-0.00091714	55.89589385
																			ISRPGDSDDSR	2	52632383	1xTMT6plex [N-Term]	1.31			3.060331821	19.64	0	1433.718201	3	478.5770874	-1.040366906	-0.000497198	27.10441362
																			LNVCVSK	4	52632383	2xTMT6plex [N-Term; K7]; 1xCarbamidomethyl [C4	1.253	1.247	1.309	3.070602655	38.28	0	1277.765164	2	639.3822632	-6.193910301	-0.003957181	44.27993969
																			FSTPEQAAK	2	52632383	2xTMT6plex [N-Term; K9]			1.316	3.543363094	31.19	0	1436.814951	2	718.9099121	-1.672484859	-0.001201526	43.55434003
167860126	SERPINB5	serpin B5	5268	NP_002630.2	66.66666667	1	21	21	201	375	42.073	6.05	0.86	0.85	0.82	0.84	Significant	0.0002																
																			SLNLSTEFISSTK	10	167860126	2xTMT6plex [N-Term; K13]	1.137	100	100	3.244474888	51.42	0	1885.068265	2	943.0371094	-0.701513057	-0.0006612	65.57462887
																			GDTANEIGQVLHFENVK	16	167860126	2xTMT6plex [N-Term; K17]	0.86	1.388		6.14914608	57.5	0	2329.25523	3	777.0913086	1.778371557	0.00138076	85.32283197
																			DVEDESTGLEK	8	167860126	2xTMT6plex [N-Term; K11]	0.781	0.698	0.668	4.530146599	62.51	0	1679.873981	2	840.4328003	-9.320624187	-0.007828737	41.76717269
																			DVPFGFQTVTSDVNK	10	167860126	2xTMT6plex [N-Term; K15]	1.072	0.914		4.672525406	59.53	0	2112.137741	3	704.7171631	-0.381126747	-0.000268331	79.39305927
																			QLCEKEPLGNVLFSPICLSTSLSLAQVGAK	4	167860126	3xTMT6plex [N-Term; K5; K30]; 2xCarbamidomethyl [C3; C17	0.573			4.040549755	18.38	1	3947.195292	4	987.553833	-0.453405169	-0.00044742	105.1933803
																			ILQHKDELNADHPFIYIIR	16	167860126	2xTMT6plex [N-Term; K5]	0.687	0.601	0.474	4.760969162	20.19	1	2793.58159	4	699.1490479	-2.587547031	-0.001807131	61.6154718
																			HIFSEDTSDFSGMSETK	14	167860126	2xTMT6plex [N-Term; K17]	0.764	0.954	1.078	5.750134468	66.34	0	2376.142962	3	798.050354	-0.572031526	-0.000456126	83.15140115
																			HIFSEDTSDFSGMSETK	4	167860126	2xTMT6plex [N-Term; K17]; 1xOxidation [M13	0.918		1.191	5.181191444	76.31	0	2392.137877	3	798.050354	-0.572031526	-0.000456126	83.15140115
																			QLNSESLSQWTNPSTMANAK	2	167860126	2xTMT6plex [N-Term; K20]		100		3.287290096	21.15	0	2665.365585	3	889.1259155	-0.89736957	-0.000797273	65.78502819
																			DLTDGHFENILADNSVNDQTK	11	167860126	2xTMT6plex [N-Term; K21]	1.091	0.921	0.951	6.499063969	44.97	0	2804.410287	3	935.4735718	-1.470725588	-0.001374839	78.65890787
																			ILVVNAAYFVGK	6	167860126	2xTMT6plex [N-Term; K12]	0.599		100	2.56624198	37.18	0	1752.082399	2	876.5454102	0.653538042	0.000572526	91.62032923
																			ACLENLGLK	11	167860126	2xTMT6plex [N-Term; K9]; 1xCarbamidomethyl [C2	0.78	0.726	0.593	4.017983437	47.9	0	1475.865606	2	738.43573	-0.964172521	-0.000711495	113.5716217
																			VCLEITEDGGDSIEVPGAR	11	167860126	1xTMT6plex [N-Term]; 1xCarbamidomethyl [C2				2.717281818	18.86	0	2246.117193	2	1123.562866	0.562411382	0.000631621	73.86812832
																			ELETVDFKDKLEETK	4	167860126	4xTMT6plex [N-Term; K8; K10; K15]	0.237	0.01		2.877350092	8.08	2	2740.579029	4	685.8990479	-1.702929819	-0.001166753	61.63656696
																			GQINNSIK	4	167860126	2xTMT6plex [N-Term; K8]			0.813	3.759927034	52.91	0	1331.80472	2	666.4051514	-1.272142679	-0.000847123	34.45592098
																			ELETVDFK	8	167860126	2xTMT6plex [N-Term; K8]	1.069	1.336	0.872	3.655225277	43.76	0	1438.819367	2	719.9133301	0.011277475	8.11E-06	89.61462374
																			ELETVDFKDK	9	167860126	3xTMT6plex [N-Term; K8; K10]	0.6	0.662	0.772	2.796480059	18.1	1	1911.104205	3	637.7064819	0.359651127	0.00022911	50.52801385
																			LSSFYSLK	6	167860126	2xTMT6plex [N-Term; K8]	1.209	0.925	1.758	3.475162268	44.63	0	1402.834624	2	701.9203491	-0.856756593	-0.000600944	64.7564264
																			EPLGNVLFSPICLSTSLSLAQVGAK	11	167860126	2xTMT6plex [N-Term; K25]; 1xCarbamidomethyl [C12				2.51620841	14.22	0	3059.721514	3	1020.577759	-0.912149292	-0.000930308	102.8126914
																			IIELPFQNK	20	167860126	2xTMT6plex [N-Term; K9]	0.716	1.069	0.89	3.104857922	27.94	0	1559.956136	2	780.4814453	-0.334339529	-0.000260778	80.62829807
																			NIIFFGK	14	167860126	2xTMT6plex [N-Term; K7]	0.946	0.448	0.963	3.104553461	28.05	0	1296.808015	2	648.9077759	0.200652533	0.000130104	80.58193789
																			DELNADHPFIYIIR	2	167860126	1xTMT6plex [N-Term]				1.273792028	0.28	0	1945.038078	3	649.0172729	-0.417669121	-0.000270794	71.57623205
62868215	LAMB3	laminin subunit beta-3 precursor	3914	NP_000219.2	29.35153584	1	26	26	127	1172	129.489	7.21	0.71	0.73	0.72	0.72	Significant	0.000064																
																			ISSADPSGAFR	6	62868215	1xTMT6plex [N-Term]	0.659	0.872	0.783	4.239112377	51.49	0	1336.705845	2	668.855835	-1.086071502	-0.000725879	40.43463794
																			AHAVEGQVEDVVGNLR	12	62868215	1xTMT6plex [N-Term]	0.645	0.725	0.868	4.519369602	50.22	0	1922.029304	3	641.3469238	-1.605550768	-0.001028639	54.50589204
																			MLSTAYEQSAQAAQQVSDSSR	2	62868215	1xTMT6plex [N-Term]	0.464			4.105399609	44.8	0	2487.198296	3	829.7384644	1.022771852	0.000847945	68.96874383
																			AAEESASQIQSSAQR	12	62868215	1xTMT6plex [N-Term]	0.601	0.599	0.671	5.55686903	78.38	0	1791.903435	3	597.9735107	1.419745346	0.000848016	21.23755286
																			CAPKPGASAGPSTAVQVHDVCVCQHNTAGPNCER	2	62868215	2xTMT6plex [N-Term; K4]; 4xCarbamidomethyl [C1; C21; C23; C32]	1.078			6.21248579	20.6	0	4090.946826	5	818.9996948	5.510047441	0.004508262	36.48210364
																			GYHPPSAYYAVSQLR	4	62868215	1xTMT6plex [N-Term]	0.587	0.63		4.295581341	35.78	0	1938.007112	3	646.6729126	-1.510668879	-0.000975896	53.65445742
																			IQEVGEITNLR	4	62868215	1xTMT6plex [N-Term]	1.189	0.382		3.459809303	38.83	0	1500.858323	2	750.932373	-0.568598806	-0.000426693	71.62367436
																			VAEVQQVLRPAEK	6	62868215	2xTMT6plex [N-Term; K13]	0.849	0.672	0.217	2.523581982	31.54	0	1925.158417	3	642.390686	-0.47378674	-0.000304038	62.81785353
																			VENVASSSGPMR	2	62868215	1xTMT6plex [N-Term]	0.937			4.099811554	52.56	0	1462.752143	2	731.8798828	0.236366087	0.000172872	40.04726117
																			MNEIQAIAAR	5	62868215	1xTMT6plex [N-Term]	0.867	0.771	0.659	3.751445532	52.98	0	1345.745936	2	673.3765259	-0.119362937	-8.03E-05	52.4644224
																			TYGDVATGCR	6	62868215	1xTMT6plex [N-Term]; 1xCarbamidomethyl [C9	0.686	0.785		4.207090378	30.95	0	1328.646616	2	664.8270264	0.120547009	8.01E-05	34.49669556
																			DMPCDEESGR	2	62868215	1xTMT6plex [N-Term]; 1xCarbamidomethyl [C4	0.728			3.349295139	42.8	0	1424.598345	2	712.8024292	-0.535780183	-0.000381636	31.45220962
																			SADLTGLEK	6	62868215	2xTMT6plex [N-Term; K9]	0.759	0.677	0.67	3.692842722	57.79	0	1391.814616	2	696.4100342	-1.310907792	-0.00091227	41.35802098
																			YPVCVACHPCFQTYDADLR	8	62868215	1xTMT6plex [N-Term]; 3xCarbamidomethyl [C4; C7; C10	0.924	0.749	0.552	2.891575098	19.69	0	2601.188601	3	867.7324829	-2.193339433	-0.001901763	58.46303577
																			TEAEELFGETMEMMDR	2	62868215	1xTMT6plex [N-Term]				2.510438204	23.47	0	2147.949658	2	1074.478149	-0.296013397	-0.000317911	104.7555923
																			LQAEAEEAR	4	62868215	1xTMT6plex [N-Term]		1.02	0.939	3.599405527	48.16	0	1245.663646	2	623.3363037	1.352605802	0.000842446	35.72338801
																			LETQVSASR	8	62868215	1xTMT6plex [N-Term]	0.775	0.831	0.704	3.426240444	49.45	0	1219.684381	2	610.3459473	0.193936443	0.000118271	40.69922374
																			MKDMELELLR	2	62868215	2xTMT6plex [N-Term; K2]	0.869			2.647371292	12.79	1	1735.985069	3	579.3331909	-0.028580945	-1.65E-05	79.79562775
																			DMELELLR	6	62868215	1xTMT6plex [N-Term]	100	100		3.083552122	42.62	0	1247.68669	2	624.3463135	-1.073391971	-0.000669628	66.98559395
																			VLYYATCK	4	62868215	2xTMT6plex [N-Term; K8]; 1xCarbamidomethyl [C7	0.716	0.681		3.421265841	43.67	0	1475.833244	2	738.4196777	-0.789222803	-0.000582381	65.4476423
																			SQMEEDVR	5	62868215	1xTMT6plex [N-Term]	1.029	0.936		2.792901754	39.02	0	1222.593517	2	611.800415	0.029444067	1.80E-05	30.77267869
																			LPNVDLVLSQTK	4	62868215	2xTMT6plex [N-Term; K12]		100	0.468	2.594455719	26.6	0	1785.088606	2	893.0464478	-1.673441967	-0.001493621	76.71610432
																			GACYPPVGDLLVGR	2	62868215	1xTMT6plex [N-Term]; 1xCarbamidomethyl [C3		100		1.907809258	12.87	0	1702.914792	2	851.9599609	-1.26072955	-0.001073458	70.1189563
																			GSQAIMLR	10	62868215	1xTMT6plex [N-Term]	0.681	0.742	0.765	2.830495119	39.09	0	1104.63968	2	552.8237915	0.567124126	0.000313234	38.23318
																			LEMSSLPDLTPTFNK	2	62868215	2xTMT6plex [N-Term; K15]				1.42847538	4.74	0	2151.177163	2	1076.090942	-1.187640153	-0.001277412	79.76739284
																			QQGAEAVQAQQLAEGASEQALSAQEGFER	1	62868215	1xTMT6plex [N-Term]				0.887231767		0	3260.598091	3	1087.536011	-1.414474679	-0.001537344	70.85588059
544063423	STIP1	stress-induced-phosphoprotein 1 isoform a	10963	NP_001269581.1	45.59322034	1	27	27	157	590	68.037	7.74	1.11	1.16	1.09	1.12	Significant	0.00035																
																			KETKPEPMEEDLPENK	8	544063423	4xTMT6plex [N-Term; K1; K4; K16]	1.137	1.129	1.153	5.008872032	15.99	1	2830.567812	4	708.3963623	-1.481173223	-0.00104814	42.88869547
																			TYEEGLKHEANNPQLK	6	544063423	3xTMT6plex [N-Term; K7; K16]	1.049		0.838	4.475338459	18.32	1	2558.418162	4	640.3538208	-9.657892224	-0.006177232	43.80893837
																			ALSVGNIDDALQCYSEAIK	7	544063423	2xTMT6plex [N-Term; K19]; 1xCarbamidomethyl [C13	1.436	1.367		5.288439751	85.36	0	2525.33216	3	842.447937	-1.149296275	-0.000967452	99.02212448
																			ELDPTNMTYITNQAAVYFEK	9	544063423	2xTMT6plex [N-Term; K20]	1.513	2.837	1.224	3.356682062	28.77	0	2806.437353	3	936.1488037	-1.958068717	-0.001831732	93.41054232
																			ETKPEPMEEDLPENK	10	544063423	3xTMT6plex [N-Term; K3; K15]	1.187	1.08	1.369	4.805395126	28.69	1	2473.309917	3	825.1079102	-0.299081504	-0.000246776	48.87496705
																			DPQALSEHLK	10	544063423	2xTMT6plex [N-Term; K10]	1.189	1.013	1.094	4.012467861	48.51	0	1595.915727	2	798.460144	-1.701702675	-0.001357887	35.08316678
																			LMDVGLIAIR	6	544063423	1xTMT6plex [N-Term]	1.342	100		3.781665564	59.77	0	1329.812559	2	665.4102173	0.450544935	0.00029957	94.82106733
																			LAYINPDLALEEK	7	544063423	2xTMT6plex [N-Term; K13]	1.041	100	1.061	2.517708302	31.85	0	1947.1203	2	974.0663452	2.626288518	0.00255685	70.32865069
																			AAALEFLNR	4	544063423	1xTMT6plex [N-Term]	1.032	1.253		2.988528252	39.84	0	1233.715288	2	617.3599854	-2.102224801	-0.001296773	63.95582285
																			ALDLDSSCK	4	544063423	2xTMT6plex [N-Term; K9]; 1xCarbamidomethyl [C8	1.109	1.108		4.049443722	53.79	0	1466.792501	2	733.9003906	0.684289018	0.000501855	44.029536
																			DCEECIQLEPTFIK	6	544063423	2xTMT6plex [N-Term; K14]; 2xCarbamidomethyl [C2; C5				2.294250965	15.12	0	2240.134312	2	1120.569458	-1.193260761	-0.001336532	67.91473859
																			AAALEAMK	10	544063423	2xTMT6plex [N-Term; K8]	1.421	1.576	1.432	3.839673042	50.7	0	1262.754265	2	631.8804932	-0.439564443	-0.000277531	47.96257397
																			TVDLKPDWGK	8	544063423	3xTMT6plex [N-Term; K5; K10]	1.244	1.402	0.642	3.370268345	13.22	0	1846.104146	3	616.0388794	-1.116294772	-0.000686932	63.6986991
																			TLLSDPTYR	2	544063423	1xTMT6plex [N-Term]			1.172	3.431397676	36.61	0	1294.720433	2	647.8632202	-0.979910628	-0.000634355	51.5362356
																			YKDAIHFYNK	4	544063423	3xTMT6plex [N-Term; K2; K10]	0.793		0.851	3.360005617	15.23	1	1986.141594	4	497.2905579	-0.600301958	-0.000298071	57.0809966
																			LLEFQLALK	6	544063423	2xTMT6plex [N-Term; K9]	1.325	0.942	1.03	2.570446968	19.05	0	1532.981622	2	766.9959717	1.986122554	0.001522345	100.0126209
																			GDYPQAMK	2	544063423	2xTMT6plex [N-Term; K8]	1.124			3.185700178	33.59	0	1367.739343	2	684.3735962	0.418809926	0.000286411	43.09049391
																			ELGNDAYK	4	544063423	2xTMT6plex [N-Term; K8]	1.561	1.233		3.459058523	33.92	0	1367.757101	2	684.3818359	-0.51630878	-0.000353092	46.52141032
																			AMADPEVQQIMSDPAMR	9	544063423	1xTMT6plex [N-Term]	1.264	0.01	100	1.389165759	4.5	0	2119.018347	2	1060.01001	-2.644558863	-0.002801934	62.33841975
																			EAADGYQR	4	544063423	1xTMT6plex [N-Term]		0.762		2.463527441	28.84	0	1138.569017	2	569.788269	0.214432269	0.000122073	23.29743711
																			LILEQMQK	6	544063423	2xTMT6plex [N-Term; K8]	100	100	1.069	1.519644856	15.16	0	1460.891093	2	730.9483643	-1.123023053	-0.000820307	58.82892547
																			GDYPQAMK	2	544063423	2xTMT6plex [N-Term; K8]; 1xOxidation [M7]			1.171	1.854399085	23.06	0	1383.734258	2	684.3735962	0.418809926	0.000286411	43.09049391
																			ELIEQLR	7	544063423	1xTMT6plex [N-Term]	2.095	0.998	1.198	2.510425329	32.1	0	1129.67784	2	565.3371582	-9.560003191	-0.005399862	49.11791584
																			AMDVYQK	6	544063423	2xTMT6plex [N-Term; K7]	0.988	1.09	1.178	3.19994235	37.91	0	1312.733529	2	656.8704224	0.029554027	1.94E-05	46.30702275
																			TYEEGLK	4	544063423	2xTMT6plex [N-Term; K7]	1.798	1.488		2.796376467	39.96	0	1297.740389	2	649.3730469	-1.211066569	-0.000785825	44.9127205
																			CMMAQYNR	2	544063423	1xTMT6plex [N-Term]; 1xCarbamidomethyl [C1	1.123			2.697095871	23.81	0	1302.595449	2	651.8012695	-0.143135384	-9.32E-05	42.08886387
																			EGLQNMEAR	1	544063423	1xTMT6plex [N-Term]; 1xOxidation [M6]			1.098	1.555770993	14.23	0	1292.646616	2	646.8272705	0.501572214	0.000324178	27.44202662
																			GNECFQK	3	544063423	2xTMT6plex [N-Term; K7]; 1xCarbamidomethyl [C4	1.395		1.062	3.057311296	32.27	0	1340.703292	2	670.8490601	-9.285128847	-0.006224301	35.73064409
222136639	MTHFD1	C-1-tetrahydrofolate synthase, cytoplasmic	4522	NP_005947.3	38.60962567	1	32	32	144	935	101.467	7.3	1.39	1.38	1.5	1.42	Significant	0.00073																
																			LVGPEGFVVTEAGFGADIGMEK	2	222136639	2xTMT6plex [N-Term; K22]			3.587	3.954395294	33.88	0	2681.42606	3	894.4820557	2.071118898	0.001851184	101.7689732
																			IYGADDIELLPEAQHK	6	222136639	2xTMT6plex [N-Term; K16]	1.371	100	100	3.637832403	39.52	0	2270.243269	3	757.4144287	-6.402722293	-0.004845246	64.38370834
																			TDPTTLTDEEINR	8	222136639	1xTMT6plex [N-Term]	1.639	2	1.762	3.678113699	54.27	0	1733.875489	2	867.4414063	0.027112674	2.35E-05	41.62396931
																			GALALAQAVQR	5	222136639	1xTMT6plex [N-Term]	0.757	100	1.431	3.980458021	68.88	0	1326.8055	2	663.90625	-0.208154096	-0.00013809	58.20879847
																			KVVGDVAYDEAK	6	222136639	3xTMT6plex [N-Term; K1; K12]		1.414	1.51	4.252351284	37.71	1	1981.157303	3	661.0579224	0.964304975	0.000636813	56.63923084
																			VVGDVAYDEAK	4	222136639	2xTMT6plex [N-Term; K11]	1.52	1.339		4.557683468	61.13	0	1623.899408	2	812.4537964	0.558971469	0.000453857	54.27672321
																			IFHELTQTDK	4	222136639	2xTMT6plex [N-Term; K10]	1.213	1.274		3.83606267	42.07	0	1689.957592	3	563.9910889	0.663526451	0.000373777	49.12124222
																			TDTESELDLISR	6	222136639	1xTMT6plex [N-Term]	100	100	100	3.223750591	52.65	0	1607.832561	2	804.4187622	-1.439015475	-0.001156848	51.49834692
																			YVVVTGITPTPLGEGK	4	222136639	2xTMT6plex [N-Term; K16]		1.097	100	2.783373833	22.71	0	2089.230913	2	1045.11853	-0.540487467	-0.000564602	74.20792302
																			ITIGQAPTEK	6	222136639	2xTMT6plex [N-Term; K10]	2.416	2.233	2.189	4.192670822	35.74	0	1515.914665	2	758.4616699	0.922600581	0.000699292	47.04764204
																			QPSQGPTFGIK	6	222136639	2xTMT6plex [N-Term; K11]	100	1.96	1.697	4.617639542	64.51	0	1617.936463	2	809.4719849	0.14240767	0.000115203	48.66529573
																			EHGAFDAVK	2	222136639	2xTMT6plex [N-Term; K9]			1.362	3.887748718	41.71	0	1431.799635	2	716.4036865	0.3222985	0.000230733	39.2068791
																			KITIGQAPTEK	6	222136639	3xTMT6plex [N-Term; K1; K11]	1.173	0.69	1.332	5.463840961	33.72	1	1873.17256	3	625.0562134	-9.861725331	-0.006157571	43.66500112
																			DDSNLYINVK	4	222136639	2xTMT6plex [N-Term; K10]	100			2.742804766	38.03	0	1638.910307	2	819.9591064	0.383669944	0.0003144	40.57391689
																			GEWIKPGAIVIDCGINYVPDDK	5	222136639	3xTMT6plex [N-Term; K5; K22]; 1xCarbamidomethyl [C13	100		100	2.87183547	6.01	0	3146.716318	3	1049.575806	-1.097791305	-0.001151479	77.842655
																			EQVPGFTPR	2	222136639	1xTMT6plex [N-Term]	1.687			3.138432741	35.97	0	1259.694552	2	630.350769	-0.230773454	-0.000145352	46.71999342
																			ETGVPIAGR	2	222136639	1xTMT6plex [N-Term]	1.556			2.6446805	38.89	0	1128.657438	2	564.8324585	0.17893134	0.000100976	35.95461788
																			TAHLDEEVNK	4	222136639	2xTMT6plex [N-Term; K10]	1.061	1.019		4.13280344	14.01	0	1613.889906	3	538.6351318	0.579889501	0.000311959	34.54144191
																			AAQAPSSFQLLYDLK	4	222136639	2xTMT6plex [N-Term; K15]		100		1.914607644	11.95	0	2110.194862	2	1055.60022	-0.805208517	-0.000849573	84.18198258
																			LAILQVGNR	6	222136639	1xTMT6plex [N-Term]	1.637	100		2.87958622	35.66	0	1212.762572	2	606.8848877	-0.060530706	-3.67E-05	63.22534174
																			AAEEIGIK	6	222136639	2xTMT6plex [N-Term; K8]		1.39	1.243	3.441653252	51.29	0	1288.787673	2	644.8966064	-1.347824324	-0.00086853	42.09130312
																			GVPTGFILPIR	6	222136639	1xTMT6plex [N-Term]	0.815	1.88	1.075	3.030644894	43.5	0	1398.867037	2	699.9364014	-1.080221054	-0.000755543	78.11111368
																			GDLNDCFIPCTPK	4	222136639	2xTMT6plex [N-Term; K13]; 2xCarbamidomethyl [C6; C10				1.953029394	19.04	0	1995.00799	2	998.0065308	-1.105251996	-0.001102493	55.88479186
																			ASFITPVPGGVGPMTVAMLMQSTVESAK	5	222136639	2xTMT6plex [N-Term; K28]				1.795673728	3.53	0	3264.744634	3	1088.913086	-6.104335786	-0.006643033	108.8327766
																			GCLELIK	4	222136639	2xTMT6plex [N-Term; K7]; 1xCarbamidomethyl [C2	1.596	1.409		3.098658085	33.09	0	1290.785565	2	645.895813	-0.941940683	-0.000607922	56.963477
																			LDIDPETITWQR	4	222136639	1xTMT6plex [N-Term]	1.362	100		2.278349876	18.46	0	1715.916566	2	858.4598999	-2.35602674	-0.002021373	70.41814158
																			MVVASSK	4	222136639	2xTMT6plex [N-Term; K7]	1.592	1.576		2.804663897	35.5	0	1179.717151	2	590.3625488	0.56794652	0.000335008	37.20260781
																			VLLSALER	6	222136639	1xTMT6plex [N-Term]	3.183	100	1.341	2.020351648	26.58	0	1129.714225	2	565.3601685	-1.031000506	-0.000582368	61.5186882
																			GDILVVATGQPEMVK	4	222136639	2xTMT6plex [N-Term; K15]				1.280128956	9.32	0	2015.161119	2	1008.079956	-4.209867164	-0.00424178	65.29397599
																			EISAQIR	4	222136639	1xTMT6plex [N-Term]	1.51	1.526	1.494	2.434860468	31.82	0	1045.620325	2	523.3138428	0.080580755	4.21E-05	35.68942116
																			FSDIQIR	4	222136639	1xTMT6plex [N-Term]	1.778	1.816		2.796749115	33.37	0	1107.635975	2	554.3212891	-0.60780348	-0.000336612	60.44474939
																			TPVPSDIDISR	1	222136639	1xTMT6plex [N-Term]			0.856	3.154368639	11.91	0	1428.789575	2	714.8983765	-0.068806712	-4.92E-05	45.18321612
4503481	EEF1G	elongation factor 1-gamma	1937	NP_001395.1	70.70938215	1	23	23	172	437	50.087	6.67	1.25	1.28	1.32	1.28	Significant	0.00025																
																			VLSAPPHFHFGQTNR	10	4503481	1xTMT6plex [N-Term]	1.229	1.361	1.668	3.51669383	26.05	0	1937.03433	3	646.3499146	0.444154412	0.000286781	46.56852349
																			FPEELTQTFMSCNLITGMFQR	7	4503481	1xTMT6plex [N-Term]; 1xCarbamidomethyl [C12	3.149	2.318	100	4.861989498	43.05	0	2779.345495	3	927.1179199	-2.262437105	-0.002096031	114.0666905
																			ALIAAQYSGAQVR	6	4503481	1xTMT6plex [N-Term]	1.211	1.274		4.300621986	61.59	0	1576.900856	2	788.9536133	-0.574822124	-0.000453219	46.36159687
																			GSTPEAAAQVVQWVSFADSDIVPPASTWVFPTLGIMHHNK	2	4503481	2xTMT6plex [N-Term; K40]			100	4.168744087	15.56	0	4749.453952	5	950.6905518	-6.379685169	-0.006060004	107.4559122
																			KLDPGSEETQTLVR	18	4503481	2xTMT6plex [N-Term; K1]	1.648	1.656	1.76	3.922855616	40.82	1	2031.14864	3	677.7208252	-0.35334394	-0.000239231	48.47913252
																			EYFSWEGAFQHVGK	12	4503481	2xTMT6plex [N-Term; K14]	100	2.627	1.366	4.760475159	23.68	0	2143.101296	3	715.0383301	-0.400823623	-0.000286335	82.6514101
																			LDPGSEETQTLVR	2	4503481	1xTMT6plex [N-Term]			2.318	4.231454372	58.04	0	1673.890745	2	837.4490356	0.029631003	2.48E-05	43.97149895
																			GQELAFPLSPDWQVDYESYTWR	14	4503481	1xTMT6plex [N-Term]	100	100	2.614	2.748098135	17.86	0	2916.40419	3	972.803833	-2.483810916	-0.002414599	96.73406386

Bhat et al,2017. Cigarette smoke and chewing tobacco alter expression of different sets of miRNAs in oral keratinocytes

Supplementary Table S2(A). List of all proteins and peptides identified in OKF6/TERT1 cells chronically treated with chewing tobacco using Sequest and Mascot search algorithms

Accession	Gene_Symbol	Description	Gene_ID	Refseq_Accession	Coverage	Proteins	#Unique_Peptides	#Peptides	#PSMS	#AAs	MW [kDa]	calc. pI	OKF6-STE-6M/OKF6P_R1	OKF6-STE-6M/OKF6P_R2	OKF6-STE-6M/OKF6P_R3	Median fold change	Status	P-value (6M)	Sequence	#PSMSs	Protein Accessions	Modifications	OKF6-STE-6M/OKF6P_R1	OKF6-STE-6M/OKF6P_R2	OKF6-STE-6M/OKF6P_R3	Xcorr	Ionscore	# Missed Cleavages	Theo. MH+ [Da]	Charge	m_z	DeltaM [ppm]	Deltam/z [Da]	RT [min]
																			AAAPAPEEEMDECEQALAAEPK	10	4503481	2xTMT6plex [N-Term; K22]; 1xCarbamidomethyl [C13	1.648	1.184	2.233	3.209808111	31.68	0	2815.353031	3	939.1239014	1.463230904	0.001373171	50.91843119
																			QATENAKEEVR	6	4503481	2xTMT6plex [N-Term; K7]	0.893	1.003	0.732	3.679965019	15.44	1	1732.959383	3	578.3242188	-0.738534332	-0.000426617	26.32332744
																			AKDPFAHLPK	13	4503481	3xTMT6plex [N-Term; K2; K10]	1.06	1.035	0.997	3.818313837	25.32	1	1811.114651	3	604.3728027	-5.960940612	-0.003598649	44.36827469
																			ILGLLDAYLK	6	4503481	2xTMT6plex [N-Term; K10]	100	2.398	1.41	3.302571535	43.57	0	1577.007837	2	789.007019	-0.681863487	-0.000537652	103.2768463
																			STFVLDEFK	10	4503481	2xTMT6plex [N-Term; K9]	2.611	100	2.509	3.344503164	40.04	0	1543.877217	2	772.4432373	1.28343715	0.000990735	76.15943638
																			WFLTCINQPQFR	7	4503481	1xTMT6plex [N-Term]; 1xCarbamidomethyl [C5	100	2.008		2.876085281	38.01	0	1838.957326	2	919.9821167	-0.200609094	-0.000184456	93.5748697
																			VPAFEGDDGFCVFESNAIAYYVSNEELR	10	4503481	1xTMT6plex [N-Term]; 1xCarbamidomethyl [C11	0.01			2.337717533	13.54	0	3427.599003	3	1143.204468	-0.044491695	-5.08E-05	118.0258736
																			QVLEPSFR	8	4503481	1xTMT6plex [N-Term]	2.126	1.934	2.054	2.992130995	36.29	0	1204.688739	2	602.848999	1.645974443	0.000991443	45.40318477
																			KFAETQPK	4	4503481	3xTMT6plex [N-Term; K1; K8]			1.372	3.2765553	30.9	1	1636.003704	2	818.5053101	-0.220056233	-0.000180006	38.35088517
																			DGWSLWYSEYR	2	4503481	1xTMT6plex [N-Term]				1.524415731	7.33	0	1690.806287	2	845.9072266	0.525917727	0.000444613	80.1367228
																			AVLGEVK	9	4503481	2xTMT6plex [N-Term; K7]	1.171	1.163	1.249	3.083745956	38.76	0	1173.76073	2	587.3834839	-0.885381948	-0.000519613	42.2559634
																			YSNEDTLSVALPYFWEHFDK	2	4503481	2xTMT6plex [N-Term; K20]				1.618775249	7.65	0	2919.460531	3	973.8242798	-0.769061444	-0.000748415	96.15207737
																			MAQFDAK	6	4503481	2xTMT6plex [N-Term; K7]	1.419	1.632		2.786306381	25.59	0	1268.707315	2	634.8582764	1.54608108	0.000980762	33.84668057
																			DPFAHLPK	2	4503481	2xTMT6plex [N-Term; K8]	1.524			1.669552922	11.49	0	1382.819642	3	461.6112366	-0.351255643	-0.000161908	48.0905969
																			TFLVGER	6	4503481	1xTMT6plex [N-Term]		1.787	1.505	2.827785254	31.62	0	1050.614511	2	525.8110352	0.269035405	0.000141326	44.97606044
5031877	LMNB1	lamin-B1 isoform 1	4001	NP_005564.1	42.15017065	1	23	26	136	586	66.368	5.16	1.43	1.4	1.41	1.41	Significant	0.000039																
																			ALYETELADAR	10	5031877	1xTMT6plex [N-Term]	1.268	1.249	0.573	4.646683216	70.82	0	1480.784489	2	740.8964233	0.72991019	0.00054042	48.21087592
																			KSMYEEEINETR	4	5031877	2xTMT6plex [N-Term; K1]	1.01	1.388		5.375740051	46.64	1	1987.020662	3	663.0116577	-0.122029666	-8.08E-05	39.93168116
																			SMYEEEINETR	6	5031877	1xTMT6plex [N-Term]	2.216	0.906	1.625	5.00522089	62.16	0	1629.762767	2	815.3775024	-9.227813697	-0.007519574	42.81625236
																			IESLSSQLSNLQK	6	5031877	2xTMT6plex [N-Term; K13]	2.009			3.64423418	39.98	0	1905.105713	2	953.0540771	-2.537902801	-0.002417487	73.76430771
																			LSSEMNTSTVNSAR	6	5031877	1xTMT6plex [N-Term]	2.01	2.156	1.793	4.479751587	59.84	0	1725.863878	2	863.4364014	0.954735999	0.000823872	41.59253051
																			AEHDQLLLNYAK	6	5031877	2xTMT6plex [N-Term; K12]	1.569	1.482	1.139	4.771583557	48.06	0	1873.058369	3	625.0246582	0.562048355	0.000350916	48.34425365
																			DQMQQQLNDYEQLLDVK	5	5031877	2xTMT6plex [N-Term; K17]	5.746	1.702		3.112669468	35.51	0	2566.322323	3	856.1115723	-0.841614128	-0.000719951	92.62177452
																			LALDMEISAYR	4	5031877	1xTMT6plex [N-Term]	2.262			4.44628334	34.99	0	1510.813681	2	755.9105835	0.138648591	0.000104736	95.08550704
																			DAALATALGDK	6	5031877	2xTMT6plex [N-Term; K11]	100	100	100	3.318826914	45.41	0	1503.878279	2	752.4424438	-0.4442146	-0.000334022	54.32818975
																			KIGDTSVSYK	2	5031877	3xTMT6plex [N-Term; K1; K10]			1.207	3.736650467	32.4	1	1785.072511	3	595.6955566	-0.22103759	-0.000131523	45.11338888
																			SMYEEEINETR	2	5031877	1xTMT6plex [N-Term]; 1xOxidation [M2]	2.309			2.324163198	33.57	0	1645.757682	2	815.3775024	-9.227813697	-0.007519574	42.81625236
																			KQLADETLLK	4	5031877	3xTMT6plex [N-Term; K1; K10]	1.047	1.182		4.032470226	34.96	1	1846.161661	3	616.0591431	0.658327624	0.000405126	48.23681309
																			IGDTSVSYK	4	5031877	2xTMT6plex [N-Term; K9]	1.547	2.314		3.831271648	39.56	0	1427.814616	2	714.4106445	-0.422910295	-0.000301919	47.2815721
																			QLADETLLK	4	5031877	2xTMT6plex [N-Term; K9]	100		1.664	4.538676262	46.11	0	1488.903766	2	744.9547729	-1.004988769	-0.000748166	55.91513063
																			LYKEELEQTYHAK	4	5031877	3xTMT6plex [N-Term; K3; K13]	2.299		1.179	3.252063274	14.08	1	2339.321408	4	585.5855713	-0.407461645	-0.000238296	49.84594781
																			TTIPEEEEEEEEAAGVVVEEELFHQQGTPR	5	5031877	1xTMT6plex [N-Term]	100			2.315896273	10.18	0	3640.733975	4	910.9373169	-1.795635693	-0.001634358	92.86474908
																			EYEAALNSK	2	5031877	2xTMT6plex [N-Term; K9]	1.452			3.912791252	49.02	0	1482.82043	2	741.9142456	0.529181364	0.00039234	50.0274283
																			DAALATALGDKK	6	5031877	3xTMT6plex [N-Term; K11; K12]	0.882	0.01		2.960970163	17.69	1	1861.136174	3	621.0508423	0.966896186	0.000599842	54.37923266
																			IQELEDLLAK	5	5031877	2xTMT6plex [N-Term; K10]		100	100	2.157687426	23.41	0	1629.982744	2	815.4943848	-0.767633126	-0.000625614	96.96936357
																			LAQALHEMR	4	5031877	1xTMT6plex [N-Term]	1.366			4.065821171	54.79	0	1297.724806	3	433.2469788	1.214879525	0.000525526	47.75440825
																			LLEGEEER	8	5031877	1xTMT6plex [N-Term]	0.891	0.901	0.91	3.30015111	57.52	0	1203.641848	2	602.324585	0.03776196	2.27E-05	47.24056336
																			LVEVDSGR	7	5031877	1xTMT6plex [N-Term]	1.309	1.55	1.759	3.135245085	37.13	0	1103.625804	2	552.3158569	-1.238357066	-0.000683341	44.64090885
																			KLLEGEEER	2	5031877	2xTMT6plex [N-Term; K1]		0.786		2.727909088	12.36	1	1560.899743	3	520.9711914	-0.462452027	-0.000240614	36.54987775
																			LQIELGK	24	5031877	2xTMT6plex [N-Term; K7]	1.154	1.463	100	3.083035707	38.53	0	1258.813494	2	629.9094238	-1.527751139	-0.000961577	71.00877436
																			LAVYIDK	6	5031877	2xTMT6plex [N-Term; K7]	1.44	1.207	2.247	3.429924011	34.64	0	1279.802595	2	640.4004517	-7.007689875	-0.00448423	56.83551256
																			QIEYEYK	4	5031877	2xTMT6plex [N-Term; K7]	1.476	1.333		3.300004482	35.27	0	1430.793153	2	715.9006958	0.672628018	0.000481196	43.01427866
																			SLETENSALQLQVTER	2	5031877	1xTMT6plex [N-Term]				1.299800992	1.3	0	2047.086878	2	1024.046509	-0.555678358	-0.000568761	52.09046072
19923142	KPNB1	importin subunit beta-1 isoform 1	3837	NP_002256.2	40.98173516	1	26	26	131	876	97.108	4.78	1.43	1.38	1.35	1.39	Significant	0.00028																
																			SSAYESLMEIVK	6	19923142	2xTMT6plex [N-Term; K12]	1.575	100		3.417889833	47.34	0	1814.997408	2	908.0037231	1.521665568	0.00138091	86.81337259
																			ALQSNIIPFCDEVMQLLLENLGNENVHR	4	19923142	1xTMT6plex [N-Term]; 1xCarbamidomethyl [C10	100	100		4.221271515	42.3	0	3495.792574	3	1165.933594	-1.81537945	-0.002115397	116.251084
																			GDQENVHPDVMLVQPR	6	19923142	1xTMT6plex [N-Term]	1.351	1.234		4.359088421	54.99	0	2063.054139	3	688.3555908	-0.930391998	-0.000639816	53.6146352
																			VLANPGNSQVAR	4	19923142	1xTMT6plex [N-Term]	1.107		1.374	3.828609943	49.12	0	1454.827691	2	727.9175415	0.078983795	5.75E-05	38.22802929
																			ESTLEAIGYICQDIDPEQLQDK	12	19923142	2xTMT6plex [N-Term; K22]; 1xCarbamidomethyl [C11	1.081			4.372702122	40.48	0	3023.528353	3	1008.514709	0.40420589	0.000407376	93.06461998
																			QDENDDDDDWNPCK	4	19923142	2xTMT6plex [N-Term; K14]; 1xCarbamidomethyl [C13		2.743	1.691	3.485323906	51.24	0	2223.950076	2	1112.478638	-0.034874603	-3.88E-05	44.14345143
																			LLETTDRPDGHQNNLR	4	19923142	1xTMT6plex [N-Term]			1.73	5.751907825	38.61	0	2108.104594	4	527.7821045	0.945796881	0.00049846	38.37309176
																			VAALQNLVK	4	19923142	2xTMT6plex [N-Term; K9]	1.272	1.468		4.111748695	45.75	0	1413.919356	2	707.4631958	-0.170588539	-0.000120599	75.1111971
																			ESCLEAYTGIVQGLK	12	19923142	2xTMT6plex [N-Term; K15]; 1xCarbamidomethyl [C3	100			2.824730873	28.83	0	2126.156762	2	1063.582764	0.700105362	0.000744267	83.02584104
																			LAATNALLNSLEFTK	4	19923142	2xTMT6plex [N-Term; K15]		100		2.133428335	21.53	0	2064.210512	2	1032.607178	-1.66319822	-0.001716596	96.58927617
																			VQHQDALQISDVVMASLLR	5	19923142	1xTMT6plex [N-Term]	100	100	1.204	2.972657919	12.92	0	2352.29068	3	784.7677612	-0.828957327	-0.000649983	90.36297675
																			LQQVLQMESHIQSTSDR	6	19923142	1xTMT6plex [N-Term]	100	3.48	1.041	2.902873278	25.14	0	2229.149495	3	743.7211304	-0.295007903	-0.000219206	66.55493491
																			VAAGLQIK	7	19923142	2xTMT6plex [N-Term; K8]	1.466	1.591	1.103	3.537988663	41.82	0	1257.829479	2	629.4199829	2.552476601	0.00160529	36.99864525
																			DTAAWTVGR	6	19923142	1xTMT6plex [N-Term]	1.651	1.536	0.658	3.285908699	42.63	0	1205.647602	2	603.328186	1.238720427	0.00074673	31.22807189
																			LELEAAQK	6	19923142	2xTMT6plex [N-Term; K8]	1.489	1.195	1.594	3.960560799	51.43	0	1359.824787	2	680.4158325	-0.293170812	-0.00019933	46.38874677
																			WLAIDANAR	10	19923142	1xTMT6plex [N-Term]	1.59	1.281	0.281	3.120041609	32.12	0	1258.710537	2	629.8575439	-2.165175627	-0.001362665	60.47855409
																			TLATWATK	4	19923142	2xTMT6plex [N-Term; K8]	1.007	3.405		3.681094885	38.23	0	1349.819308	2	675.4133301	0.056093619	3.79E-05	51.9774342
																			TTLVIMER	2	19923142	1xTMT6plex [N-Term]		1.339		2.650989294	32.53	0	1191.69686	2	596.3520508	-0.0297454	-1.77E-05	63.47452243
																			TVSPDRLELEAAQK	4	19923142	2xTMT6plex [N-Term; K14]		1.335	1.055	4.297916412	30.3	1	2015.153725	3	672.3858643	-5.302774884	-0.003561969	46.70002381
																			SNEILTAIIQGMR	4	19923142	1xTMT6plex [N-Term]		100		1.973832607	30.75	0	1674.941007	2	837.9729004	-1.482093244	-0.001241209	92.46089262
																			DCYPAVQK	2	19923142	2xTMT6plex [N-Term; K8]; 1xCarbamidomethyl [C2	1.411			3.434192896	32.34	0	1438.776457	2	719.8929443	1.497836488	0.001077526	39.91196178
																			ANFDKESER	3	19923142	2xTMT6plex [N-Term; K5]	1.515	1.152	0.775	2.633758783		1	1553.832392	3	518.6147461	-1.741827989	-0.00090217	27.15615716
																			AAVENLPTFLVELSR	3	19923142	1xTMT6plex [N-Term]				1.254129887	6.85	0	1888.074129	2	944.5408325	0.137329917	0.000129645	95.71611311
																			DPSVVVR	4	19923142	1xTMT6plex [N-Term]		1.491	1.302	2.576472282	19.8	0	1000.598861	2	500.8035278	0.917514599	0.000459032	32.28750303
																			SDIDEVALQGIEFWSNVCDEEMDLAIEASEAAEQGRPPEHTSK	1	19923142	2xTMT6plex [N-Term; K43]; 1xCarbamidomethyl [C18]				2.205701828		0	5261.493112	5	1053.105347	0.858117333	0.000902996	112.4238618
																			SDYDMVDYLNELR	4	19923142	1xTMT6plex [N-Term]				1.817282081	0.41	0	1861.883945	2	931.4454346	-0.189361628	-0.000176285	84.06151397
5454052	SFN	14-3-3 protein sigma	2810	NP_006133.1	71.37096774	1	15	19	455	248	27.757	4.74	0.88	0.88	0.92	0.89	Significant	0.00022																
																			YLAEVATGDDK	24	5454052	2xTMT6plex [N-Term; K11]	0.868	0.76	0.849	5.035099506	53.92	0	1639.894323	2	820.4430542	-9.446499902	-0.007745631	51.46485839
																			VETELQGVCDTVLGLLDSHLIK	31	5454052	2xTMT6plex [N-Term; K22]; 1xCarbamidomethyl [C9	1.106	0.984	1.169	4.642138004	40.57	0	2897.605816	3	966.5388794	-1.287506529	-0.001243562	110.8066498
																			EKVETELQGVCDTVLGLLDSHLIK	40	5454052	3xTMT6plex [N-Term; K2; K24]; 1xCarbamidomethyl [C11	0.95	0.825	0.894	7.857717037	49.05	1	3383.906304	4	846.7327881	0.892126873	0.000754718	110.4484145
																			TTFDEAMADLHTLSEDSYK	10	5454052	2xTMT6plex [N-Term; K19]	0.696	100	1.393	4.56392622	63.68	0	2632.285269	3	878.0992432	-0.795087765	-0.000697633	106.3189487
																			YLAEVATGDDKK	6	5454052	3xTMT6plex [N-Term; K11; K12]	0.711	0.904	100	4.719202995	30.97	1	1997.152218	3	666.3884888	-0.653401075	-0.00043498	59.76812554
																			SNEEGSEEKGPEVR	31	5454052	2xTMT6plex [N-Term; K9]	0.977	0.825	1.03	4.737241745	40.4	1	2005.023833	2	1003.017944	2.383334201	0.002389321	26.42581255
																			SAYQEAMDISK	22	5454052	2xTMT6plex [N-Term; K11]	0.814	0.729	0.764	5.028225899	60.89	0	1700.892943	2	850.9467163	-3.990158723	-0.003393416	56.60653632
																			GAVEKGEELSCEER	16	5454052	2xTMT6plex [N-Term; K5]; 1xCarbamidomethyl [C11	0.552	0.61	0.64	4.712587833	32.18	1	2051.04794	3	684.3536377	-0.770417908	-0.000526721	38.39027954
																			DSTLIMQLLR	37	5454052	1xTMT6plex [N-Term]	1.27	1.271	1.324	4.110405922	66.2	0	1418.823852	2	709.9172974	2.443154983	0.001733203	90.31582375
																			GEELSCEER	20	5454052	1xTMT6plex [N-Term]; 1xCarbamidomethyl [C6	0.906	0.822	0.817	3.566242218	49.34	0	1337.620461	2	669.3145142	0.965132002	0.00064549	26.04781226
																			SNEEGSEEK	18	5454052	2xTMT6plex [N-Term; K9]		1.491	1.93	3.91492033	41.97	0	1466.737488	2	733.8729858	0.822907778	0.000603495	20.66073378
																			YEDMAAFMK	24	5454052	2xTMT6plex [N-Term; K9]	0.895	1.29	1.165	3.847100258	51.19	0	1563.795143	2	782.4014282	0.279208766	0.000218313	71.72698506
																			DNLTLWTADNAGEEGGEAPQEPQS	43	5454052	1xTMT6plex [N-Term]	1.84	1.073	2.959	2.551413536	20.68	0	2758.264127	3	920.0933838	0.533439554	0.000490456	62.0053808
																			SAYQEAMDISK	24	5454052	2xTMT6plex [N-Term; K11]; 1xOxidation [M7	0.884	0.879	0.893	3.198213577	57.57	0	1716.887858	2	850.9467163	-3.990158723	-0.003393416	56.60653632
																			VISSIEQK	56	5454052	2xTMT6plex [N-Term; K8]	1.111	0.959	0.957	3.934776306	49.24	0	1361.840437	2	681.4239502	0.137042946	9.33E-05	59.72555441
																			TTFDEAMADLHTLSEDSYK	7	5454052	2xTMT6plex [N-Term; K19]; 1xOxidation [M7				2.426323891	13.84	0	2648.280184	3	878.0992432	-0.795087765	-0.000697633	106.3189487
																			NLLSVAYK	24	5454052	2xTMT6plex [N-Term; K8]	0.874	0.882	0.72	3.972506762	46.76	0	1365.850608	2	683.4294434	0.733725009	0.000501079	92.81997229
																			YEDMAAFMK	16	5454052	2xTMT6plex [N-Term; K9]; 1xOxidation [M]	0.954	1.086	1.34	2.4012146	32.43	0	1579.790058	2	782.4014282	0.279208766	0.000218313	71.72698506
																			DSTLIMQLLR	14	5454052	1xTMT6plex [N-Term]; 1xOxidation [M6]	1.102	100		1.706976891	33.93	0	1434.818767	2	709.9172974	2.443154983	0.001733203	90.31582375
																			YEDMAAFMK	2	5454052	2xTMT6plex [N-Term; K9]; 2xOxidation [M4; M8]		0.806		1.65663743	20.36	0	1595.784973	2	782.4014282	0.279208766	0.000218313	71.72698506
																			LAEQAER	6	5454052	1xTMT6plex [N-Term]	1.208	0.935	1.021	2.81289196	25.93	0	1045.583939	2	523.2957764	0.322273863	0.000168482	26.08720983
																			EMPPTNPIR	8	5454052	1xTMT6plex [N-Term]	0.843	0.813		3.245959282	30.22	0	1283.697923	2	642.352356	-0.379914876	-0.000243848	36.980202
																			KEMPPTNPIR	2	5454052	2xTMT6plex [N-Term; K1]	0.775			3.839627266	23.6	1	1640.955818	3	547.6575928	1.466901355	0.000802373	37.45698663
																			NVVGGQR	2	5454052	1xTMT6plex [N-Term]			0.814	2.220030785	26.47	0	958.5631442	2	479.7850342	-0.367696825	-0.00017623	23.36867052
4885049	ACTC1	actin, alpha cardiac muscle 1 precursor	70	NP_005150.1	53.58090186	1	5	14	644	377	41.992	5.39	0.94	0.89	0.91	0.91	Significant	0.00025																
																			SYELPDGQVITIGNER	64	4885049	1xTMT6plex [N-Term]	1.248	1.218	1.377	3.803804874	38.48	0	2020.05485	2	1010.528687	-2.35325448	-0.002376852	85.21825869
																			AVFPSIVGRPR	34	4885049	1xTMT6plex [N-Term]	0.352	0.364	0.444	4.536440372	51.94	0	1427.868434	3	476.627655	-0.015640173	-7.44E-06	49.14656194
																			EITALAPSTMK	42	4885049	2xTMT6plex [N-Term; K11]	1.222	1.44	1.159	4.486789703	61.33	0	1619.94425	2	810.4758301	0.082321506	6.67E-05	67.20350059
																			KYSVWIGGSILASLSTFQQMWISKQEYDEAGPSIVHR	1	4885049	3xTMT6plex [N-Term; K1; K24]; 1xOxidation [M20					6.97	2	4915.605986	5	983.927002	-0.016765863	-1.65E-05	112.3189617
																			DSYVGDEAQSK	44	4885049	2xTMT6plex [N-Term; K11]	1.107	1.161	1.095	4.72834444	60.64	0	1656.848101	2	828.9279175	0.275976378	0.000228625	44.20964203
																			HQGVMVGMGQK	24	4885049	2xTMT6plex [N-Term; K11]	1.133	1.188	1.182	5.080441952	68.53	0	1629.896923	3	549.3018799	-0.456592314	-0.000250501	32.73665634
																			HQGVMVGMGQK	52	4885049	2xTMT6plex [N-Term; K11]; 2xOxidation [M5; M8	1.128	1.098	1.01	5.129117489	55.53	0	1661.886753	3	549.3018799	-0.456592314	-0.000250501	32.73665634
																			HQGVMVGMGQK	41	4885049	2xTMT6plex [N-Term; K11]; 1xOxidation [M	1.106	1.108	1.093	5.131017685	75.89	0	1645.891838	3	549.3018799	-0.456592314	-0.000250501	32.73665634
																			LCYVALDFENEMATAASSSSLEK	10	4885049	2xTMT6plex [N-Term; K23]; 1xCarbamidomethyl [C2]; 1xOxidation [M12]	0.489	2.56	100	4.063639164	41.88	0	3010.47896	3	1004.171204	6.675728383	0.006699047	118.7756937
																			GYSFVTTAER	8	4885049	1xTMT6plex [N-Term]	0.495	0.575	0.458	4.249853611	39.54	0	1359.710596	2	680.3585815	-0.521893456	-0.000354812	44.35081332
																			EITALAPSTMK	30	4885049	2xTMT6plex [N-Term; K11]; 1xOxidation [M10	1.059	1.146	1.148	3.327458143	52.93	0	1635.939165	2	810.4758301	0.082321506	6.67E-05	67.20350059
																			IWHHTFYNELR	48	4885049	1xTMT6plex [N-Term]	1.078	1.014	1.089	3.88049674	43.62	0	1744.91209	3	582.3091431	0.450549471	0.000262056	45.4901325
																			AGFAGDDAPR	7	4885049	1xTMT6plex [N-Term]	1.487	1.259		3.371614456	57.95	0	1205.611216	2	603.3093262	0.132093786	7.96E-05	32.14685543
																			YPIEHGIITNWDDMEK	86	4885049	2xTMT6plex [N-Term; K16]	1.026	1.06	1.033	3.380174637	17.68	0	2419.236803	3	812.4142456	-1.451378761	-0.001178148	72.00949459
																			YPIEHGIITNWDDMEK	64	4885049	2xTMT6plex [N-Term; K16]; 1xOxidation [M14	0.972	0.752	0.816	2.273640156	24.06	0	2435.231718	3	812.4142456	-1.451378761	-0.001178148	72.00949459
																			DLTDYLMK	55	4885049	2xTMT6plex [N-Term; K8]	1.087	1.087	1.065	3.984642267	41.49	0	1456.812173	2	736.9068604	-0.437522932	-0.000322193	64.73083726
																			DLTDYLMK	31	4885049	2xTMT6plex [N-Term; K8]; 1xOxidation [M7]	1.04	0.908	0.72	2.499874353	44.45	0	1472.807088	2	736.9068604	-0.437522932	-0.000322193	64.73083726
																			VAPEEHPTLLTEAPLNPK	2	4885049	2xTMT6plex [N-Term; K18]			0.436	2.528061628	15.2	0	2414.369532	3	805.4564209	-6.139182297	-0.004940752	54.34237207
																			IKIIAPPER	1	4885049	2xTMT6plex [N-Term; K2]		1.029		2.575391769	0.47	1	1494.977205	3	498.9970093	-0.488842215	-0.000243603	55.327609
154354962	IMMT	MICOS complex subunit MIC60 isoform [illegible]	10989	NP_001093639.1	31.17569353	1	1	22	126	757	83.497	6.57	0.72	0.81	0.78	0.77	Significant	0.0012																
																			VVSQYHELVVQAR	8	154354962	1xTMT6plex [N-Term]	0.919	0.919		5.091937542	33.81	0	1756.990734	3	586.3344116	-1.168091942	-0.000684109	56.55258675
																			GIEQAVQSHAVAEEEAR	14	154354962	1xTMT6plex [N-Term]	0.502	0.833	0.807	6.597196102	66.54	0	2053.051162	3	685.0217896	-0.168674635	-0.000115433	48.80925986
																			AVDEAADALLK	6	154354962	2xTMT6plex [N-Term; K11]	0.909	0.767	100	3.837901592	81.94	0	1573.920144	2	787.4641113	0.50964228	0.000401068	74.35865486
																			YSTSGSSGLTTGK	4	154354962	2xTMT6plex [N-Term; K13]	1.334	100		4.15993166	39.61	0	1703.9216	2	852.4644775	0.045834342	3.90E-05	44.95796594
																			KAVDEAADALLK	2	154354962	3xTMT6plex [N-Term; K1; K12]		0.714		3.501346827	30.48	1	1931.178039	3	644.397644	0.17606813	0.00011334	72.75315604
																			EVAGAKPHITAAEGK	6	154354962	3xTMT6plex [N-Term; K6; K15]	0.788	0.736		4.000221729	25.81	0	2166.284963	4	542.3267822	0.154908637	8.39E-05	45.08548
																			LLSYASYCIEHGDLELAAK	2	154354962	2xTMT6plex [N-Term; K19]; 1xCarbamidomethyl [C8			0.962	5.753736973	51.15	0	2611.384196	3	871.1323853	-0.610101066	-0.000531069	94.07481678
																			AAMDNSEIAGEK	4	154354962	2xTMT6plex [N-Term; K12]	0.73	0.916		5.522397518	65.68	0	1693.883106	2	847.4446411	-0.649863874	-0.000550397	46.1091918
																			TDHPEIGEGKPTPALSEASSSSIR	8	154354962	2xTMT6plex [N-Term; K10]	0.65	0.741	0.914	6.798127651	37.66	0	2924.53655	4	731.8894653	-0.177201367	-0.000129558	40.96696412
																			GMSVSDLADK	6	154354962	2xTMT6plex [N-Term; K10]	1.159	1.1	0.877	4.159358501	60.96	0	1480.808151	2	740.9080811	0.496228613	0.00036741	54.54089676
																			SEFEQNLSEK	6	154354962	2xTMT6plex [N-Term; K10]	1.045	0.922	0.912	4.45746994	47.04	0	1668.884487	2	834.9447632	-1.340334116	-0.001118431	44.97154383
																			VQAAQSEAK	6	154354962	2xTMT6plex [N-Term; K9]	1.107	1.118	2.575	4.414123058	54.22	0	1389.8102	2	695.4086914	-0.067223208	-4.67E-05	27.80726653
																			ISSVSEVMK	8	154354962	2xTMT6plex [N-Term; K9]	0.824	100	100	4.257594109	48.89	0	1437.838723	2	719.4233398	0.473285	0.000340254	53.86338023
																			KVQAAQSEAK	6	154354962	3xTMT6plex [N-Term; K1; K10]	0.703	0.761		4.688598633	38.17	1	1747.068095	3	583.0283203	1.3239939	0.000771036	28.01759519
																			GVYSEETLR	12	154354962	1xTMT6plex [N-Term]	0.671	0.774	0.829	3.861198664	54.59	0	1282.684047	2	641.8468018	1.77744132	0.001139948	26.57489261
																			LSEQELQFR	4	154354962	1xTMT6plex [N-Term]	0.456	0.504		2.189433098	35.41	0	1378.752795	2	689.8795166	-0.753330748	-0.000519328	61.72301146
																			SEIQAEQDR	2	154354962	1xTMT6plex [N-Term]	0.74			3.317947149	54.64	0	1304.664374	2	652.8357544	-0.108817977	-7.10E-05	28.47773498
																			ELDSITPEVLPGWK	2	154354962	2xTMT6plex [N-Term; K14]		100		2.20420146	11.98	0	2042.157414	2	1021.583374	1.007545677	0.001028783	84.02347209
																			DAMENEMR	4	154354962	1xTMT6plex [N-Term]	0.956	0.867		3.300866127	50.42	0	1224.555023	2	612.7808838	-0.434886032	-0.000266271	35.33874841
																			AFDSAVAK	6	154354962	2xTMT6plex [N-Term; K8]	0.934		0.719	3.159055233	39.33	0	1266.745809	2	633.8768921	0.551815276	0.000349505	46.73832995
																			VAMIDETR	4	154354962	1xTMT6plex [N-Term]	1.844	1.659		3.209153652	41.55	0	1163.629175	2	582.3183594	0.229781107	0.00013369	49.86159853
																			DAMENEMR	2	154354962	1xTMT6plex [N-Term]; 1xOxidation [M3]		0.674		1.794281721	28.13	0	1240.549938	2	612.7808838	-0.434886032	-0.000266271	35.33874841
																			VAQDWLK	2	154354962	2xTMT6plex [N-Term; K7]				1.32699573	10.49	0	1317.793093	2	659.3999023	-0.428771787	-0.000282516	55.07284516
																			GMSVSDLADK	2	154354962	2xTMT6plex [N-Term; K10]; 1xOxidation [M2	0.515			1.927385092	26.39	0	1496.803066	2	740.9080811	0.496228613	0.00036741	54.54089676
156523968	PARP1	poly [ADP-ribose] polymerase 1	142	NP_001609.2	34.51676529	1	29	29	118	1014	113.012	8.88	1.62	1.59	1.6	1.6	Significant	0.00003																
																			KTAEAGGVTGK	4	156523968	3xTMT6plex [N-Term; K1; K11]	1.284	1.4		4.38556385	49.7	1	1706.041545	3	569.3508911	-2.007805827	-0.0011418	26.6096994
																			KFYPLEIDYGQDEEAVK	4	156523968	3xTMT6plex [N-Term; K1; K17]	1.487		0.811	3.842167377	33.55	1	2731.479759	3	911.1651001	0.361585315	0.000329221	84.62060173
																			GFSLLATEDKEALKK	2	156523968	4xTMT6plex [N-Term; K10; K14; K15]	1.829			3.351547003	14	2	2566.562591	4	642.3968506	1.161749868	0.000745426	74.28499701
																			HPDVEVDGFSELR	6	156523968	1xTMT6plex [N-Term]	1.561	1.646		3.707862854	41.73	0	1728.875429	3	576.963623	0.512767207	0.000295504	55.87829833
																			KPPLLNNADSVQAK	2	156523968	3xTMT6plex [N-Term; K1; K14]			1.577	5.614970207	40.31	0	2182.316264	3	728.1101685	-0.142714835	-0.000103816	44.03315581
																			GIYFADMVSK	14	156523968	2xTMT6plex [N-Term; K10]	1.513	100	100	3.201926231	48.08	0	1588.880922	2	794.9434814	-0.777553156	-0.00061772	74.34774226
																			GDEVDGVDEVAK	2	156523968	2xTMT6plex [N-Term; K12]			1.877	4.095065594	49.01	0	1690.889966	2	845.9435425	-6.007208788	-0.005078765	41.51121731
																			TAEAGGVTGK	2	156523968	2xTMT6plex [N-Term; K10]			100	3.310047626	25.51	0	1348.783651	2	674.8947754	-1.020451834	-0.000688184	29.86534573
																			FYTLIPHDFGMK	2	156523968	2xTMT6plex [N-Term; K12]	2.108			3.10687542	39.25	0	1927.055198	3	643.0233765	0.196452355	0.000126192	85.89692697
																			MIFDVESMK	8	156523968	2xTMT6plex [N-Term; K9]	1.854	1.668		3.707496881	42.21	0	1557.842093	2	779.4245605	-0.159776303	-0.000124453	86.76475195
																			GGAAVDPDSGLEHSAHVLEK	2	156523968	2xTMT6plex [N-Term; K20]			100	2.324092388	5.96	0	2447.293072	4	612.5789185	0.315335394	0.00019293	52.90918284
																			LQLLEDDKENR	4	156523968	2xTMT6plex [N-Term; K8]	1.229	1.11		3.026112556	10.25	1	1831.032548	3	611.0160522	0.576580838	0.000351913	58.72611605
																			MVDPEKPQLGMIDR	4	156523968	2xTMT6plex [N-Term; K6]	3.273	2.006		2.749254465	9.1	0	2087.139338	3	696.3849487	0.457655792	0.000318397	61.36209505
																			VADGMVFGALLPCEECSGQLVFK	3	156523968	2xTMT6plex [N-Term; K23]; 2xCarbamidomethyl [C13; C16	100			1.779752374	11.42	0	2985.528828	3	995.8483887	0.597802174	0.000594919	95.23667713
																			VVSEDFLQDVSASTK	6	156523968	2xTMT6plex [N-Term; K15]				1.872984409	24.67	0	2083.132321	2	1042.068237	-1.499300161	-0.00156162	72.65096135
																			QQVPSGESAILDR	2	156523968	1xTMT6plex [N-Term]		1.502		3.015552759	19.62	0	1628.880515	2	814.9434204	-0.583578524	-0.00047529	48.76613898
																			VVDRDSEEAEIIR	6	156523968	1xTMT6plex [N-Term]	2.855	2.01		4.558503151	31.16	1	1759.938758	2	880.4737549	0.83826532	0.000737648	39.8221718
																			MAIMVQSPMFDGK	6	156523968	2xTMT6plex [N-Term; K13]	100		0.542	1.698095441	16.12	0	1913.009904	2	957.0073853	-1.259660069	-0.001204871	74.02386002
																			VFSATLGLVDIVK	2	156523968	2xTMT6plex [N-Term; K13]	100			1.886513591	10.17	0	1820.129743	2	910.5679932	-0.567526507	-0.000516486	103.5999258
																			AMVEYEIDLQK	5	156523968	2xTMT6plex [N-Term; K11]			0.622	1.878846169	12.81	0	1796.986843	2	898.9984131	1.506055476	0.001353181	68.40843009
																			LTVNPGTK	4	156523968	2xTMT6plex [N-Term; K8]	2.042	2.023		3.055418015	44.4	0	1287.803658	2	644.4053955	-0.111324715	-7.17E-05	48.9071676
																			ASLCISTK	4	156523968	2xTMT6plex [N-Term; K8]; 1xCarbamidomethyl [C4	1.774	2.191		3.514478207	42.69	0	1337.786293	2	669.395874	-1.361953799	-0.000911002	47.23115863
																			KLTVNPGTK	4	156523968	3xTMT6plex [N-Term; K1; K9]	1.42	1.203		4.028553486	25.43	1	1645.061553	3	549.0252075	-0.293856121	-0.000161137	46.07253592
																			GFSLLATEDK	4	156523968	2xTMT6plex [N-Term; K10]				1.577295423	16.61	0	1538.88303	2	769.9451294	-0.031179067	-2.40E-05	60.0567764
																			GTNSYYK	4	156523968	2xTMT6plex [N-Term; K7]	1.763	2.593		3.155427217	27.45	0	1290.709423	2	645.8581543	-0.30297776	-0.000195528	36.79682615
																			ELLIFNK	6	156523968	2xTMT6plex [N-Term; K7]	100	1.339	1.264	2.476173639	23.42	0	1334.844794	2	667.9249268	-1.661177677	-0.001108707	70.90640866
																			IAPPEAPVTGYMFGK	3	156523968	2xTMT6plex [N-Term; K15]	2.594	0.588		1.542635322	2.81	0	2036.129091	2	1018.567505	-0.66673296	-0.000678777	80.32403334
																			SANYCHTSQGDPIGLILLGEVALGNMYELK	1	156523968	2xTMT6plex [N-Term; K30]; 1xCarbamidomethyl [C5				1.298578143		0	3721.933374	4	931.2380981	-0.755269263	-0.000702765	111.0798929
																			SDAYYCTGDVTAWTK	2	156523968	2xTMT6plex [N-Term; K15]; 1xCarbamidomethyl [C6				1.333198428		0	2196.068341	2	1098.536865	-0.859372732	-0.000943621	58.84082868
55956788	NCL	nucleolin	4691	NP_005372.2	40.56338028	1	29	29	255	710	76.568	4.7	1.36	1.32	1.34	1.34	Significant	0.000074																
																			EVFEDAAEIR	18	55956788	1xTMT6plex [N-Term]	2.052	2.878	2.763	4.142613888	62.46	0	1407.731725	2	704.3695679	0.09498414	6.69E-05	50.81614751
																			VEGTEPTTAFNLFVGNLNFNK	12	55956788	2xTMT6plex [N-Term; K21]	1.184		100	3.242484331	26.84	0	2770.481601	3	924.166748	1.476173176	0.001363237	104.8481333
																			GLSEDTTEETLK	12	55956788	2xTMT6plex [N-Term; K12]	1.588	1.467	1.658	4.694994926	73.47	0	1780.958045	2	890.9830933	0.48543167	0.000432267	42.8048675
																			KFGYVDFESAEDLEK	8	55956788	3xTMT6plex [N-Term; K1; K15]	1.431	1.343	1.03	4.814443588	34.78	1	2464.321468	3	822.1101685	-2.238274908	-0.00183861	73.67515035
																			EAMEDGEIDGNK	36	55956788	2xTMT6plex [N-Term; K12]	1.537	1.677	1.758	3.902029753	68.51	0	1765.86785	2	883.4378052	0.273820922	0.000241766	29.88821455
																			GLSEDTTEETLKESFDGSVR	7	55956788	2xTMT6plex [N-Term; K12]	0.76	100	0.01	3.232396841	38.34	1	2658.35104	3	886.788147	-0.433634239	-0.000384251	79.78471672
																			TLVLSNLSYSATEETLQEVFEK	12	55956788	2xTMT6plex [N-Term; K22]	1.953		1.674	5.648114204	68.58	0	2959.591605	3	987.2015381	-0.521674066	-0.000514647	105.6224508
																			GYAFIEFASFEDAK	38	55956788	2xTMT6plex [N-Term; K14]	100	100	100	3.017705441	35.53	0	2053.068265	2	1027.03772	-0.049534093	-5.08E-05	96.74684177
																			VTQDELKEVFEDAAEIR	7	55956788	2xTMT6plex [N-Term; K7]		100		3.325352907	24.51	1	2450.31789	3	817.4437866	-0.44213313	-0.000361122	97.82870396
																			SISLYYTGEK	8	55956788	2xTMT6plex [N-Term; K10]	1.754	1.414	1.045	3.151122093	43.48	0	1618.909245	2	809.9562378	-2.499154338	-0.002022952	54.33928175
																			EAMEDGEIDGNK	16	55956788	2xTMT6plex [N-Term; K12]; 1xOxidation [M3	1.777	1.486	1.756	2.839432478	55.54	0	1781.862765	2	883.4378052	0.273820922	0.000241766	29.88821455
																			KVAVATPAK	2	55956788	3xTMT6plex [N-Term; K1; K9]	1.13			5.455944538	30.89	1	1572.045174	3	524.6862793	-0.56600751	-0.000296596	38.95956466
																			ALELTGLK	8	55956788	2xTMT6plex [N-Term; K8]	1.338	1.211	1.493	3.300933361	51.53	0	1302.839709	2	651.9232788	-0.328446247	-0.000213956	71.19403081
																			GIAYIEFK	16	55956788	2xTMT6plex [N-Term; K8]	1.347	1.298	100	3.103596449	48.68	0	1398.839709	2	699.9232178	-0.393170941	-0.000274992	77.21692456
																			VTLDWAKPK	12	55956788	3xTMT6plex [N-Term; K7; K9]	1.354	1.335	1.15	2.720161915	16.59	0	1745.092853	3	582.3694458	0.533674952	0.000310437	68.44494126
																			NDLAVVDVR	2	55956788	1xTMT6plex [N-Term]			100	2.538877487	45.99	0	1229.705117	2	615.3563843	0.305036291	0.000187552	52.06270267
																			QGTEIDGR	2	55956788	1xTMT6plex [N-Term]		1.587		2.94001174	34.64	0	1104.584667	2	552.7959595	-0.022682451	-1.25E-05	22.42403081
																			KAAVTPGK	4	55956788	3xTMT6plex [N-Term; K1; K8]		0.922	1.079	4.029923916	23.87	1	1458.96111	3	486.9925537	1.369044588	0.000665794	30.3616232
																			EALNSCNK	2	55956788	2xTMT6plex [N-Term; K8]; 1xCarbamidomethyl [C6	1.834			3.262621641	32.77	0	1393.750971	2	697.3779297	-1.713186251	-0.001193877	33.22967722
																			KVVVSPTK	6	55956788	3xTMT6plex [N-Term; K1; K8]	1.031	1.049		4.159699917	30.04	1	1545.034275	3	515.6821289	-1.580502963	-0.000813977	40.6358609
																			ESFDGSVR	2	55956788	1xTMT6plex [N-Term]	1.466			2.691430569	26.25	0	1125.573768	2	563.2910767	0.984698066	0.000554175	40.97722793
																			KTVTPAK	4	55956788	3xTMT6plex [N-Term; K1; K7]		1.096	1.199	3.791569948	16.06	1	1431.950211	3	477.9877625	-1.031716377	-0.000492455	28.20708684
																			ALVATPGKK	2	55956788	3xTMT6plex [N-Term; K8; K9]			0.56	3.077111959	19.08	1	1572.045174	3	524.6865234	-0.100103675	-5.25E-05	41.56342265
																			KAAATSAK	2	55956788	3xTMT6plex [N-Term; K1; K8]	0.907			2.285179853	8.81	1	1434.924725	3	478.9788818	-1.834745852	-0.000877574	30.39181306
																			GQNQDYR	2	55956788	1xTMT6plex [N-Term]				1.770909548	28.61	0	1109.553702	2	555.2807007	0.381339981	0.000211559	14.82089475
																			NVAEDEDEEEDDEDEDDDDEDDEDDDDEDDEEEEEEEEEEPVK	1	55956788	2xTMT6plex [N-Term; K44]				1.081236005		0	5737.045134	4	1435.017578	0.583641914	0.000837095	52.71265112
																			VTQDELK	3	55956788	2xTMT6plex [N-Term; K7]	2.384		2.121	3.155457497	41.36	0	1290.766938	2	645.8864746	-0.980247644	-0.000632636	41.0489003
																			GAAIPAK	2	55956788	2xTMT6plex [N-Term; K7]			1.387	2.719047546	22.96	0	1085.708301	2	543.3579712	0.336022864	0.000182411	35.70946179
																			ALVATPGK	8	55956788	2xTMT6plex [N-Term; K8]	1.475	1.48	1.494	3.381684542	39.7	0	1214.787279	2	607.8970337	-0.402298572	-0.000244354	42.0696533
																			FGYVDFESAEDLEK	1	55956788	2xTMT6plex [N-Term; K14]				1.422444344		0	2107.063573	2	1054.033203	-2.108792566	-0.00222168	77.34157924
109148542	AARS	alanine--tRNA ligase, cytoplasmic	16	NP_001596.2	39.04958678	1	28	28	132	968	106.743	5.53	1.13	1.26	1.23	1.21	Significant	0.0011																
																			TITVALADGGRPDNTGR	5	109148542	1xTMT6plex [N-Term]	1.534	1.347	2.32	4.553646564	31.53	0	1943.050768	3	648.3552246	0.181538296	0.000117579	42.8050379
																			VGAEDADGIDMAYR	6	109148542	1xTMT6plex [N-Term]	2.885		1.38	4.290960789	38.34	0	1711.815866	2	856.4112549	-0.36945818	-0.000316222	51.00564802
																			EIADLGEALATAVIPQWQK	5	109148542	2xTMT6plex [N-Term; K19]	1.467	1.079		4.717171669	46.68	0	2511.422296	3	837.81604	4.488037399	0.003757119	111.4481569
																			YNYHLDSSGSYVFENTVATVMALR	6	109148542	1xTMT6plex [N-Term]	1.265	1.455	100	5.554113388	26.31	0	2966.455573	3	989.4887695	-1.286999301	-0.001272609	101.0991139
																			IVAVTGAEAQK	4	109148542	2xTMT6plex [N-Term; K11]	1.334	1.301		4.567789555	68.37	0	1544.941214	2	772.9740601	-0.239642007	-0.000185116	44.30316534
																			AVYTQDCPLAAAK	4	109148542	2xTMT6plex [N-Term; K13]; 1xCarbamidomethyl [C7	1.132	1.274		5.072114468	54.75	0	1866.019541	2	933.5142212	0.870930222	0.000812586	55.92303481
																			SIDTGMGLER	10	109148542	1xTMT6plex [N-Term]	1.272	1.25	1.338	4.089521408	70.43	0	1307.682667	2	654.3440552	-1.401760904	-0.000916529	44.02705513
																			DIINEEVQFLK	4	109148542	2xTMT6plex [N-Term; K12]	100	1.921		3.446774244	41.4	0	1935.083915	2	968.0446167	-1.011745045	-0.000978906	85.47475515
																			VVSIGVPVSELLDDPSGPAGSLTSVEFCGGTHLR	3	109148542	1xTMT6plex [N-Term]; 1xCarbamidomethyl [C28				1.443718076	20.21	0	3681.899542	3	1227.969482	-1.533853466	-0.001882498	100.9534615
																			ITCLCQVPQNAANR	2	109148542	1xTMT6plex [N-Term]; 2xCarbamidomethyl [C3; C5	1.601			3.246526957	17.63	0	1873.957402	2	937.4814453	-0.954261286	-0.000894123	55.71138352
																			MSNYDTDLFVPYFEAIQK	2	109148542	2xTMT6plex [N-Term; K18]	2.609			2.604039907	13.44	0	2639.346747	3	880.4539795	0.241702445	0.000212646	105.5329018
																			GGYVLHIGTIYGDLK	4	109148542	2xTMT6plex [N-Term; K15]	0.766	100		2.838937759	25.42	0	2064.189383	3	688.734436	-0.304170023	-0.000209288	76.1144414
																			[illegible]	[illegible]	109148542	[illegible]	[illegible]	[illegible]	[illegible]	[illegible]	[illegible]	[illegible]	[illegible]	[illegible]	[illegible]	[illegible]	[illegible]	[illegible]

Bhat et al,2017. Cigarette smoke and chewing tobacco alter expression of different sets of miRNAs in oral keratinocytes

Supplementary Table S2(A). List of all proteins and peptides identified in OKF6/TERT1 cells chronically treated with chewing tobacco using Sequest and Mascot search algorithms

Accession	Gene_Symbol	Description	Gene_ID	Refseq_Accession	Coverage	Proteins	#Unique_Peptides	#Peptides	#PSMS	#AAs	MW [kDa]	calc. pI	OKF6-STE-6M/OKF6P_R1	OKF6-STE-6M/OKF6P_R2	OKF6-STE-6M/OKF6P_R3	Median fold change	Status	P-value (6M)	Sequence	#PSMSs	Protein Accessions	Modifications	OKF6-STE-6M/OKF6P_R1	OKF6-STE-6M/OKF6P_R2	OKF6-STE-6M/OKF6P_R3	Xcorr	Ionscore	# Missed Cleavages	Theo. MH+ [Da]	Charge	m_z	DeltaM [ppm]	Deltam/z [Da]	RT [min]
																			LVSVLQNK	4	109148542	2xTMT6plex [N-Term; K8]	1.082	100		3.585589647	41.22	0	1358.877157	2	679.9418945	-0.474353032	-0.000322294	62.86514933
																			KAEEIANEMIEAAK	2	109148542	3xTMT6plex [N-Term; K1; K14]				2.363903761	12.85	1	2234.26693	3	745.4271851	0.032160786	2.40E-05	71.33085045
																			GLVVDMDGFEEER	6	109148542	1xTMT6plex [N-Term]	100	100	100	2.018579006	14.76	0	1724.836267	2	862.920105	-1.932553905	-0.0016667	63.8318051
																			MALELLTQEFGIPIER	3	109148542	1xTMT6plex [N-Term]			0.01	1.78703475	25.69	0	2089.156478	2	1045.075195	-6.397067495	-0.006682237	106.2012125
																			KIQSLGDSK	2	109148542	3xTMT6plex [N-Term; K1; K9]	0.604			2.913430929	8.89	1	1663.035732	3	555.0161133	-1.16970803	-0.000648422	40.73412608
																			QIWQNLGLDDTK	4	109148542	2xTMT6plex [N-Term; K12]	100	100	0.01	1.690187812	9.96	0	1889.053283	2	945.0288086	-1.557733986	-0.001471321	70.89752113
																			ALNEALK	2	109148542	2xTMT6plex [N-Term; K7]	1.01			3.099671364	39.08	0	1216.766544	2	608.8866577	-0.415199049	-0.0002526	45.83558482
																			VMDDLDR	3	109148542	1xTMT6plex [N-Term]	1.063		0.888	2.90295577	34.74	0	1092.555675	2	546.781311	-0.302071276	-0.000165015	43.83222036
																			AQTAPNK	3	109148542	2xTMT6plex [N-Term; K7]	1.147	100		2.680180311	28.86	0	1187.714843	2	594.3613281	0.451985595	0.000268415	21.58248434
154354964	IMMT	MICOS complex subunit MIC60 isoform 1	10989	NP_006830.2	31.26649077	1	1	22	124	758	83.626	6.48	0.01	0.857	0.664	0.51	Non-Significan	0.19																
																			TDHPEIGEGKPTPALSEEASSSIR	6	154354964	2xTMT6plex [N-Term; K10]	0.01	0.857	0.664	6.383597851	42.57	0	3053.579143	4	764.1505737	0.433049813	0.000330588	41.74178793
																			VVSQYHELVVQAR	8	154354964	1xTMT6plex [N-Term]	0.919	0.919		5.091937542	33.81	0	1756.990734	3	586.3344116	-1.168091942	-0.000684109	56.55258675
																			GIEQAVQSHAVAEEEAR	14	154354964	1xTMT6plex [N-Term]	0.502	0.833	0.807	6.597196102	66.54	0	2053.051162	3	685.0217896	-0.168674635	-0.000115433	48.80925986
																			AVDEAADALLK	6	154354964	2xTMT6plex [N-Term; K11]	0.909	0.767	100	3.837901592	81.94	0	1573.920144	2	787.4641113	0.50964228	0.000401068	74.35865486
																			YSTSGSSGLTTGK	4	154354964	2xTMT6plex [N-Term; K13]	1.334	100		4.15993166	39.61	0	1703.9216	2	852.4644775	0.045834342	3.90E-05	44.95796594
																			KAVDEAADALLK	2	154354964	3xTMT6plex [N-Term; K1; K12]		0.714		3.501346827	30.48	1	1931.178039	3	644.397644	0.17606813	0.00011334	72.75315604
																			EVAGAKPHITAAEGK	6	154354964	3xTMT6plex [N-Term; K6; K15]	0.788	0.736		4.000221729	25.81	0	2166.284963	4	542.3267822	0.154908637	8.39E-05	45.08548
																			LLSYASYCIEHGDLELAAK	2	154354964	2xTMT6plex [N-Term; K19]; 1xCarbamidomethyl [C8			0.962	5.753736973	51.15	0	2611.384196	3	871.1323853	-0.610101066	-0.000531069	94.07481678
																			AAMDNSEIAGEK	4	154354964	2xTMT6plex [N-Term; K12]	0.73	0.916		5.522397518	65.68	0	1693.883106	2	847.4446411	-0.649863874	-0.000550397	46.1091918
																			GMSVSDLADK	6	154354964	2xTMT6plex [N-Term; K10]	1.159	1.1	0.877	4.159358501	60.96	0	1480.808151	2	740.9080811	0.496228613	0.00036741	54.54089676
																			SEFEQNLSEK	6	154354964	2xTMT6plex [N-Term; K10]	1.045	0.922	0.912	4.45746994	47.04	0	1668.884487	2	834.9447632	-1.340334116	-0.001118431	44.97154383
																			VQAAQSEAK	6	154354964	2xTMT6plex [N-Term; K9]	1.107	1.118	2.575	4.414123058	54.22	0	1389.8102	2	695.4086914	-0.067223208	-4.67E-05	27.80726653
																			ISSVSEVMK	8	154354964	2xTMT6plex [N-Term; K9]	0.824	100	100	4.257594109	48.89	0	1437.838723	2	719.4233398	0.473285	0.000340254	53.86338023
																			KVQAAQSEAK	6	154354964	3xTMT6plex [N-Term; K1; K10]	0.703	0.761		4.688598633	38.17	1	1747.068095	3	583.0283203	1.3239939	0.000771036	28.01759519
																			GVYSEETLR	12	154354964	1xTMT6plex [N-Term]	0.671	0.774	0.829	3.861198664	54.59	0	1282.684047	2	641.8468018	1.77744132	0.001139948	26.57489261
																			LSEQELQFR	4	154354964	1xTMT6plex [N-Term]	0.456	0.504		2.189433098	35.41	0	1378.752795	2	689.8795166	-0.753330748	-0.000519328	61.72301146
																			SEIQAEQDR	2	154354964	1xTMT6plex [N-Term]	0.74			3.317947149	54.64	0	1304.664374	2	652.8357544	-0.108817977	-7.10E-05	28.47773498
																			ELDSITPEVLPGWK	2	154354964	2xTMT6plex [N-Term; K14]		100		2.20420146	11.98	0	2042.157414	2	1021.583374	1.007545677	0.001028783	84.02347209
																			DAMENEMR	4	154354964	1xTMT6plex [N-Term]	0.956	0.867		3.300866127	50.42	0	1224.555023	2	612.7808838	-0.434886032	-0.000266271	35.33874841
																			AFDSAVAK	6	154354964	2xTMT6plex [N-Term; K8]	0.934		0.719	3.159055233	39.33	0	1266.745809	2	633.8768921	0.551815276	0.000349505	46.73832995
																			VAMIDETR	4	154354964	1xTMT6plex [N-Term]	1.844	1.659		3.209153652	41.55	0	1163.629175	2	582.3183594	0.229781107	0.00013369	49.86159853
																			DAMENEMR	2	154354964	1xTMT6plex [N-Term]; 1xOxidation [M3]		0.674		1.794281721	28.13	0	1240.549938	2	612.7808838	-0.434886032	-0.000266271	35.33874841
																			VAQDWLK	2	154354964	2xTMT6plex [N-Term; K7]				1.32699573	10.49	0	1317.793093	2	659.3999023	-0.428771787	-0.000282516	55.07284516
																			GMSVSDLADK	2	154354964	2xTMT6plex [N-Term; K10]; 1xOxidation [M2	0.515			1.927385092	26.39	0	1496.803066	2	740.9080811	0.496228613	0.00036741	54.54089676
302699237	EIF4G1	eukaryotic translation initiation factor 4 gamma 1 isoform 1	1981	NP_886553.3	19.5625	1	24	29	139	1600	175.48	5.33	1.2	1.23	1.29	1.24	Significant	0.00045																
																			EFLPEGQDIGAFVAEQK	11	302699237	2xTMT6plex [N-Term; K17]	1.373	0.764	0.737	3.782574415	49.93	0	2336.253833	3	779.4232788	0.620607128	0.000483299	85.38174041
																			QVTQLAIDTEER	6	302699237	1xTMT6plex [N-Term]	2.401	2.549	2.249	4.525201797	66.37	0	1631.88018	2	816.4443359	0.744444975	0.000607423	51.70050812
																			EMDEAATAEER	20	302699237	1xTMT6plex [N-Term]	1.405	1.341	1.205	4.604492664	85.39	0	1480.678703	2	740.8425293	-0.622353957	-0.000460753	26.27778541
																			VEYTLGEESEAPGQR	4	302699237	1xTMT6plex [N-Term]	1.217	1.398		4.707348824	66.13	0	1893.939152	2	947.4735718	0.377638683	0.000357612	54.80886724
																			IHNAENIQPGEQK	2	302699237	2xTMT6plex [N-Term; K13]			1.755	4.859632015	50.14	0	1936.065245	3	646.0265503	-0.076036226	-4.91E-05	36.5331309
																			EAVGDLLDAFK	6	302699237	2xTMT6plex [N-Term; K11]	1.201	0.548		2.841021776	37.25	0	1635.935794	2	818.4713745	-0.19655818	-0.000160778	92.20825522
																			EMDEAATAEER	10	302699237	1xTMT6plex [N-Term]; 1xOxidation [M2]	0.98	1.388	1.319	2.854192257	33.76	0	1496.673618	2	740.8425293	-0.622353957	-0.000460753	26.27778541
																			AALSEEELEKK	6	302699237	3xTMT6plex [N-Term; K10; K11]	0.636	0.767	1.045	3.892446756	36.59	1	1934.141319	3	645.3816528	-5.642671401	-0.003637908	43.90453523
																			AIIEEYLHLNDMK	4	302699237	2xTMT6plex [N-Term; K13]	1.314			2.865540266	17.21	0	2047.129819	3	683.0479736	-0.220445991	-0.000150427	96.09053763
																			VPTTEKPTVTVNFR	2	302699237	2xTMT6plex [N-Term; K6]	0.976		1.124	2.470113993		0	2047.195196	3	683.0704956	0.848583667	0.000579072	49.12672338
																			QKEMDEAATAEER	2	302699237	2xTMT6plex [N-Term; K2]		1.367		3.48204875	11.99	1	1965.995176	3	656.0025024	-1.130117616	-0.000740602	32.99732451
																			GEEDADGSK	4	302699237	2xTMT6plex [N-Term; K9]		1.45	100	3.157226324	38.28	0	1365.68981	2	683.3483276	-0.315596235	-0.000215503	21.8036625
																			AALSEEELEK	2	302699237	2xTMT6plex [N-Term; K10]			1.307	1.715862751	9.56	0	1576.883424	2	788.9451904	-0.202786471	-0.000159885	46.84699956
																			NHDEESLECLCR	2	302699237	1xTMT6plex [N-Term]; 2xCarbamidomethyl [C9; C11		0.851		2.23463726	15.25	0	1790.799899	3	597.6047363	-0.135585011	-8.09E-05	41.07925492
																			GPQAGLGPR	4	302699237	1xTMT6plex [N-Term]	0.959	0.897		2.768066168	37.63	0	1081.631558	2	541.3189697	-0.827672666	-0.000447618	32.41999953
																			VFDWIEANLSEQQIVSNTLVR	2	302699237	1xTMT6plex [N-Term]				1.823872685	8.14	0	2690.435096	3	897.4806519	-2.859579703	-0.002564505	108.5711396
																			SDQWKPLNLEEK	2	302699237	3xTMT6plex [N-Term; K5; K12]	100			2.246629	5.99	0	2174.24243	3	725.4194946	0.69022051	0.000500236	61.68188909
																			AASLTEDR	4	302699237	1xTMT6plex [N-Term]		1.302	5.056	3.213756561	43.07	0	1091.589418	2	546.2984619	0.209600901	0.000114399	28.45528671
																			VQQLMAK	6	302699237	2xTMT6plex [N-Term; K7]	1.731	1.248	1.35	3.369562626	40.93	0	1275.785899	2	638.3969727	0.603104722	0.000384716	43.66790438
																			EFEKDKDDDEVFEK	1	302699237	4xTMT6plex [N-Term; K4; K6; K14]	1.174			3.0054636		2	2689.437844	4	673.1149902	0.106872706	7.19E-05	59.47125262
																			SVTAFFK	4	302699237	2xTMT6plex [N-Term; K7]	0.958		100	3.14231801	35.97	0	1257.76073	2	629.3835449	-0.729197714	-0.000458578	66.86419052
																			ITKPGSIDSNNQLFAPGGR	1	302699237	2xTMT6plex [N-Term; K3]				2.334959269		0	2430.350528	3	810.7890015	0.791476172	0.000641188	48.68899201
																			MLTEAIMHDCVVK	2	302699237	2xTMT6plex [N-Term; K13]; 1xCarbamidomethyl [C10			100	1.789652348	3.86	0	2005.068481	3	669.0213013	-9.541230927	-0.00637694	62.20653227
																			GLPLVDDGGWNTVPISK	4	302699237	2xTMT6plex [N-Term; K17]		100		1.666907072	6.48	0	2226.253439	2	1113.630005	-0.317247966	-0.000353137	77.74473138
																			FIGELFK	10	302699237	2xTMT6plex [N-Term; K7]	1.145	1.435	100	2.485556602	33.94	0	1311.807681	2	656.4070435	-0.663409698	-0.000435133	93.9411128
																			LKEELEEAR	8	302699237	2xTMT6plex [N-Term; K2]	0.927	0.949	0.98	3.381020546	19.06	1	1574.915393	2	787.961853	0.658171666	0.000518282	54.58071731
																			YLCDEQK	2	302699237	2xTMT6plex [N-Term; K7]; 1xCarbamidomethyl [C3	1.623			3.056300402	26.97	0	1413.744823	2	707.3765869	0.760178292	0.000537349	43.20410695
																			TIDQIHK	4	302699237	2xTMT6plex [N-Term; K7]	1.173	1.292		2.45512414	32.93	0	1312.798907	3	438.2709351	-0.49878486	-0.000218268	42.44104903
																			IRDPNQGGK	2	302699237	2xTMT6plex [N-Term; K9]	0.852			2.592639923	14.29	1	1442.847982	3	481.6206055	-0.498287941	-0.000239651	30.51727718
																			LLTTIGK	2	302699237	2xTMT6plex [N-Term; K7]	1.206			2.901763916	20.51	0	1203.807681	2	602.4072266	-0.418717215	-0.000252027	69.35251074
21361181	ATP1A1	sodium/potassium-transporting ATPase subunit alpha-1 isoform a	476	NP_000692.2	30.3030303	1	26	26	165	1023	112.824	5.49	1.17	1.21	1.19	1.19	Significant	0.000094																
																			LNIPVSQVNPR	6	21361181	1xTMT6plex [N-Term]	1.065	1.263	1.208	3.612550259	50.23	0	1465.868828	2	733.4379883	-0.087379919	-6.40E-05	54.24106503
																			AVAGDASESALLK	10	21361181	2xTMT6plex [N-Term; K13]	1.152	1.056	1.071	5.113562107	90.24	0	1689.978721	2	845.4932251	0.267598229	0.000226118	59.09822438
																			NPNTSEPQHLLVMK	4	21361181	2xTMT6plex [N-Term; K14]	0.891		1.504	4.223623753	41.18	0	2066.146866	3	689.3866577	-0.69988513	-0.000482022	54.29030049
																			VIMVTGDHPITAK	36	21361181	2xTMT6plex [N-Term; K13]	1.161	1.272	1.274	3.935067177	48.98	0	1840.076661	3	614.0307007	0.482339024	0.000295847	56.05785579
																			KADIGVAMGIAGSDVSK	6	21361181	3xTMT6plex [N-Term; K1; K17]		1.224	100	6.168400288	33.76	1	2306.335678	3	769.4495239	-0.719662221	-0.000553261	68.00947876
																			GVGIISEGNETVEDIAAR	9	21361181	1xTMT6plex [N-Term]	1.252	100	0.811	3.734823465	60.1	0	2059.086878	2	1030.046387	-0.671007384	-0.000690831	79.36053318
																			DKYEPAAVSEQGDK	4	21361181	3xTMT6plex [N-Term; K2; K14]	1.333	1.718		5.485012531	24.9	1	2224.206438	3	742.0718384	-2.462174008	-0.001825461	43.06181156
																			WINDVEDSYGQQWTYEQR	2	21361181	1xTMT6plex [N-Term]	100			2.334921598	16.05	0	2546.178547	3	849.3962402	-1.719995984	-0.001459806	81.5134159
																			TSATWLALSR	6	21361181	1xTMT6plex [N-Term]	1.223	1.384		3.615114927	41.37	0	1334.762966	2	667.8850708	-0.075712647	-5.05E-05	69.38786477
																			SPDFTNENPLETR	6	21361181	1xTMT6plex [N-Term]	0.946	2.115	3.414	3.420320749	28.88	0	1748.865259	2	874.9356689	-0.68462071	-0.000598655	50.05620349
																			YHTEIVFAR	8	21361181	1xTMT6plex [N-Term]	1.628	1.318	1.223	3.621568918	34.86	0	1364.752401	3	455.5885315	-0.996625891	-0.000453383	42.27592545
																			IVEIPFNSTNK	8	21361181	2xTMT6plex [N-Term; K11]	2.041	1.415		2.633775949	29.49	0	1720.004542	2	860.5056763	-0.271092852	-0.00023314	72.02457861
																			EQPLDEELKDAFQNAYLELGGLGER	6	21361181	2xTMT6plex [N-Term; K9]		100	100	2.824881554	24.23	1	3292.710157	4	823.9265747	-7.801431238	-0.006421963	105.1341111
																			DKYEPAAVSEQGDKK	1	21361181	4xTMT6plex [N-Term; K2; K14; K15]		1.214		3.037526608		2	2581.464333	4	646.1217041	0.253071965	0.000163324	37.74061973
																			LIIVEGCQR	7	21361181	1xTMT6plex [N-Term]; 1xCarbamidomethyl [C7	1.187	1.207	100	3.154066324	38.96	0	1316.755773	2	658.882019	0.751054947	0.000494478	48.18220624
																			EVSMDDHK	6	21361181	2xTMT6plex [N-Term; K8]	1.1	1.127	1.107	3.730623007	45.74	0	1418.734986	3	473.5844116	2.605027238	0.001231948	31.51288825
																			GIVVYTGDR	8	21361181	1xTMT6plex [N-Term]	1.109	1.185		3.481893539	52.06	0	1208.683653	2	604.8458252	0.596211112	0.000360315	50.03625322
																			NMVPQQALVIR	2	21361181	1xTMT6plex [N-Term]	1.572			2.521906137	28.95	0	1497.877284	2	749.4431763	1.19613875	0.000895835	58.16016934
																			NIAFFSTNCVEGTAR	2	21361181	1xTMT6plex [N-Term]; 1xCarbamidomethyl [C9				1.853191853	13.34	0	1915.953362	2	958.480835	0.538072531	0.000515461	69.95739123
																			EVSMDDHK	2	21361181	2xTMT6plex [N-Term; K8]; 1xOxidation [M4]	1.101			3.254289865	32.01	0	1434.729901	3	473.5844116	2.605027238	0.001231948	31.51288825
																			LIFDNLK	6	21361181	2xTMT6plex [N-Term; K7]	1.377	2.232	0.543	2.08139348	32.76	0	1320.829144	2	660.9176636	-0.828056806	-0.000546861	66.81112903
																			QAADMILLDDNFASIVTGVEEGR	6	21361181	1xTMT6plex [N-Term]				1.090847373	4.85	0	2693.365361	2	1347.184082	-1.661043676	-0.002236899	105.9021737
																			ADIGVAMGIAGSDVSK	1	21361181	2xTMT6plex [N-Term; K16]				1.2449615		0	1949.077783	2	975.0407715	-1.804443763	-0.001758501	70.38266136
																			DGPNALTPPPTTPEWIK	6	21361181	2xTMT6plex [N-Term; K17]				1.607754469	5.02	0	2292.264004	2	1146.633545	-1.828260725	-0.002095428	69.76824105
																			AAELLAR	10	21361181	1xTMT6plex [N-Term]	1.068	1.064	1.124	2.737405539	34.36	0	972.6039464	2	486.805603	-0.017422624	-8.47E-06	41.72656599
																			RPGGWVEK	2	21361181	2xTMT6plex [N-Term; K8]	1.775			3.419400692	12.72	0	1386.82579	3	462.9468079	0.057789511	2.67E-05	39.75296928
																			DRDMDELKK	2	21361181	3xTMT6plex [N-Term; K8; K9]	1.162			3.299508333	5.27	2	1837.045645	4	460.0167236	-0.315663768	-0.000144972	39.27999787
38455427	CCT4	T-complex protein 1 subunit delta isoform a	10575	NP_006421.2	54.91651206	1	23	24	201	539	57.888	7.83	1.2	1.17	1.22	1.2	Significant	0.00015																
																			AFADAMEVIPSTLAENAGLNPISTVTELR	14	38455427	1xTMT6plex [N-Term]		0.956	1.623	4.201107979	35.3	0	3259.708159	3	1087.234375	-6.008841604	-0.006529023	106.8379998
																			GDVTITNDGATILK	6	38455427	2xTMT6plex [N-Term; K14]	1.147	100	100	3.161209583	35.92	0	1876.079164	2	938.5429688	-0.267941785	-0.00025134	68.53289696
																			DALSDLALHFLNK	6	38455427	2xTMT6plex [N-Term; K13]	2.365	5.026		3.241618395	36.42	0	1915.105319	3	639.0394897	-0.73249325	-0.000467601	91.4774018
																			TDMDNQIVVSDYAQMDR	4	38455427	1xTMT6plex [N-Term]	1.618	100		3.621083021	47.77	0	2230.031748	2	1115.517456	-1.84419824	-0.00205631	65.60440028
																			AQDIEAGDGTTSVVIIAGSLLDSCTK	28	38455427	2xTMT6plex [N-Term; K26]; 1xCarbamidomethyl [C24	1.292	1.159	1.049	6.255850315	66.07	0	3079.623316	3	1027.213379	0.7362812	0.000755823	98.11178509
																			GIEILTDMSRPVELSDR	8	38455427	1xTMT6plex [N-Term]	1.59	0.812	0.749	3.852257967	44.32	0	2160.153184	3	720.7219238	-0.909970478	-0.000655225	78.09590048
																			TLSGMESYCVR	4	38455427	1xTMT6plex [N-Term]; 1xCarbamidomethyl [C9	1.331	1.53		4.382939816	39.16	0	1531.744615	2	774.3739014	0.643360433	0.000497877	37.00682731
																			ALIAGGGAPEIELALR	9	38455427	1xTMT6plex [N-Term]	1.015	0.881	0.991	3.243849516	40.83	0	1780.053	2	890.5305176	0.426238011	0.000379363	70.54979291
																			GIHPTIISESFQK	6	38455427	2xTMT6plex [N-Term; K13]	0.978	0.951	0.977	3.601896286	37.41	0	1915.105319	3	639.0397949	-0.254437377	-0.000162425	51.74028601
																			SIHDALCVIR	44	38455427	1xTMT6plex [N-Term]; 1xCarbamidomethyl [C7	1.004	0.987	1.016	3.972945213	50.61	0	1412.788135	2	706.8984375	1.035647146	0.000731575	52.08500621
																			ETLLNSATTSLNSK	4	38455427	2xTMT6plex [N-Term; K14]	100	100		2.524198055	23.66	0	1937.095542	2	969.0511475	-0.270274808	-0.000261774	55.64571603
																			TLSGMESYCVR	8	38455427	1xTMT6plex [N-Term]; 1xCarbamidomethyl [C9]; 1xOxidation [M5]	1.603	1.342		2.859568119	51.58	0	1547.73953	2	774.3739014	0.643360433	0.000497877	37.00682731
																			VIDPATATSVDLR	6	38455427	1xTMT6plex [N-Term]	3.303	3.202	0.841	2.962494135	34.66	0	1586.895102	2	793.9506836	-0.637516934	-0.000505836	52.47263057
																			AYILNLVK	10	38455427	2xTMT6plex [N-Term; K8]	1.433	1.908	100	3.52191782	43.37	0	1391.902644	2	696.4543457	-0.882772768	-0.000614367	80.90984681
																			KTGCNVLLIQK	2	38455427	3xTMT6plex [N-Term; K1; K11]; 1xCarbamidomethyl [C4		0.901		3.534933567	12.3	1	1961.218464	3	654.4104614	-0.832759168	-0.000544408	59.07212977
																			EDIEFICK	6	38455427	2xTMT6plex [N-Term; K8]; 1xCarbamidomethyl [C7		1.836		3.461624146	40.91	0	1511.817987	2	756.4122314	-0.529917875	-0.00040057	67.83749281
																			FSNISAAK	5	38455427	2xTMT6plex [N-Term; K8]	1.258	0.805		3.62904048	47.64	0	1295.772358	2	648.3891602	-1.014026493	-0.000656974	47.85818533
																			MLVELSK	10	38455427	2xTMT6plex [N-Term; K7]	0.948	0.863	0.843	3.151226997	32.47	0	1277.790316	2	639.3994751	1.062502428	0.000678828	64.19160154
																			LVIEEAER	6	38455427	1xTMT6plex [N-Term]	1.194	1.276		3.451999903	38.78	0	1187.683319	2	594.3459473	1.093844823	0.000649571	52.69460079
																			TAGINVR	4	38455427	1xTMT6plex [N-Term]		1.248	1.102	2.733072758	28.07	0	959.5835453	2	480.2948914	-1.082964752	-0.000519598	30.99740037
																			DIEREDIEFICK	4	38455427	2xTMT6plex [N-Term; K12]; 1xCarbamidomethyl [C11	0.602			2.265526772	9.29	1	2025.072698	3	675.6949463	-1.191385778	-0.000804214	56.35520184
																			TVTIVVR	21	38455427	1xTMT6plex [N-Term]	1.235	1.221	1.252	2.880279303	31.88	0	1016.666547	2	508.836731	-0.355402603	-0.000180663	41.28892408
																			KLGGTIDDCELVEGLVLTQK	2	38455427	3xTMT6plex [N-Term; K1; K20]; 1xCarbamidomethyl [C9			100	1.426592946	7.68	1	2875.641756	3	959.2178955	-0.912156932	-0.000874346	83.54554873
																			VSNSGITR	2	38455427	1xTMT6plex [N-Term]	0.704			2.479415894	10.83	0	1062.610488	2	531.8093262	0.835191973	0.000443742	30.02940395
																			AVADAIR	4	38455427	1xTMT6plex [N-Term]	1.004	1.002		2.288603306	31.01	0	944.5726463	2	472.7896729	-0.611045511	-0.000288588	36.8439861
15149476	RARS	arginine--tRNA ligase, cytoplasmic	5917	NP_002878.2	45.45454545	1	30	30	140	660	75.331	6.68	1.08	1.13	1.01	1.07	Significant	0.001																
																			MLLCEAVAAVMAK	4	15149476	2xTMT6plex [N-Term; K13]; 1xCarbamidomethyl [C4	0.752	1.23		3.818681002	40.85	0	1865.046289	2	933.0263672	-0.445943355	-0.000415852	110.7184572
																			VIVDFSSPNIAK	6	15149476	2xTMT6plex [N-Term; K12]	0.924	1.742	1.754	3.209881067	56.05	0	1748.035842	2	874.5212402	-0.365250663	-0.000319236	71.11983669
																			STIIGESISR	6	15149476	1xTMT6plex [N-Term]	1.273	1.184	1.059	3.883580685	52.29	0	1291.741896	2	646.3751831	0.923854015	0.00059669	39.79459813
																			LFEFAGYDVLR	4	15149476	1xTMT6plex [N-Term]		0.6	0.922	3.746208191	28.88	0	1558.846696	2	779.9276733	0.881715753	0.00068723	100.4385297
																			IYDALDVSLIER	2	15149476	1xTMT6plex [N-Term]			1.14	2.638303041	25.8	0	1635.915503	2	818.4611206	-0.329319614	-0.00026937	87.36296331
																			LMDLLGEGLKR	4	15149476	2xTMT6plex [N-Term; K10]	0.01	0.901		3.661184311	30.95	1	1703.028983	3	568.3471069	-1.300799482	-0.000738433	88.94654397
																			LNDYIFSFDK	4	15149476	2xTMT6plex [N-Term; K10]	1.524	1.544		2.998121023	42.48	0	1719.935794	2	860.470459	-1.251564888	-0.001076306	94.35881439
																			AYQCVVLLQGK	8	15149476	2xTMT6plex [N-Term; K11]; 1xCarbamidomethyl [C4	1.324	100	100	3.772438288	47.73	0	1737.013333	2	869.0176392	8.444725227	0.0073343	65.00706202
																			VEIAGPGFINVHLR	11	15149476	1xTMT6plex [N-Term]	1.306	0.69	1.083	3.453988075	39.02	0	1751.016555	3	584.3429565	-1.278355186	-0.00074614	67.30367697
																			LANIDEEMLQK	6	15149476	2xTMT6plex [N-Term; K11]	100	1.07	1.137	3.111694574	34.24	0	1761.982092	2	881.4937744	-1.032900325	-0.000909976	63.61600876
																			GFDILGIKPVQR	4	15149476	2xTMT6plex [N-Term; K8]	0.987	1.156		2.910323858	18.23	0	1801.11001	3	601.0420532	0.886169062	0.000532029	71.24950012
																			LMDLLGEGLK	2	15149476	2xTMT6plex [N-Term; K10]	100			3.187699556	36.72	0	1546.927872	2	773.9672852	-0.373771467	-0.000289099	100.8590542
																			LQEVFGHAIK	8	15149476	2xTMT6plex [N-Term; K10]	0.725	0.557	0.72	3.099679947	31.56	0	1599.962284	3	533.9916382	-1.201440333	-0.000640753	62.56722948
																			GFVQVDDGR	12	15149476	1xTMT6plex [N-Term]	1.182	1.226	1.146	3.685608864	56.22	0	1221.642517	2	611.3253174	0.688872247	0.000420778	27.98071697
																			GNTAAYLLYAFTR	4	15149476	1xTMT6plex [N-Term]				1.932642579	18.06	0	1689.916172	2	845.4612427	-0.57005102	-0.000481669	95.8442253
																			LLQQEEEIK	6	15149476	2xTMT6plex [N-Term; K9]	1.112		1.021	4.17661047	35.86	0	1587.935794	2	794.4647217	-8.581720166	-0.00681361	51.01783342
																			EIAENITK	10	15149476	2xTMT6plex [N-Term; K8]	1.107	1.042	1.095	3.840601683	44.98	0	1375.819702	2	688.413269	-0.319964936	-0.000220107	36.20930093
																			RFDTEEEFK	2	15149476	2xTMT6plex [N-Term; K9]	1.111			3.878697157	34.16	1	1658.879007	3	553.6314087	0.401158985	0.000221825	50.28905407
																			DFVSEQLTSLLVNGVQLPALGENK	2	15149476	2xTMT6plex [N-Term; K24]				2.042912006	12.56	0	3029.692322	3	1010.562805	-6.092952912	-0.006153258	101.2642871
																			FDTEEEFK	2	15149476	2xTMT6plex [N-Term; K8]			1.798	3.087923765	40.32	0	1502.777896	2	751.8920288	-0.742220667	-0.000557696	51.33699755
																			IVFVPGCSIPLTIVK	6	15149476	2xTMT6plex [N-Term; K15]; 1xCarbamidomethyl [C7	0.456	1.865	0.01	1.880613923	21.74	0	2101.285926	2	1051.14563	-0.924593056	-0.000971417	90.33384214
																			GFVQVDDGRK	8	15149476	2xTMT6plex [N-Term; K10]	0.957	0.729	0.807	4.145258427	25.62	1	1578.900412	3	526.9718018	0.278974807	0.000146824	42.13219422
																			VFHAGFGVVLGEDK	2	15149476	2xTMT6plex [N-Term; K14]	100			2.219213247	5.72	0	1933.094754	3	645.0365601	0.192849202	0.000124265	72.90617445
																			ILLDHEKEWK	2	15149476	3xTMT6plex [N-Term; K7; K10]		0.344		3.055655718	6.49	1	1998.199109	4	500.305603	0.737433707	0.000368385	63.44231632
																			ILLDHEK	4	15149476	2xTMT6plex [N-Term; K7]		1.395	1.342	3.547755003	30.67	0	1325.819308	3	442.6112976	0.023949165	1.06E-05	37.89628792
																			GESFYQDR	2	15149476	1xTMT6plex [N-Term]	1.552			3.062586784	35.93	0	1230.595232	2	615.8021851	1.512647814	0.000930729	35.79094417
																			FPEILQK	2	15149476	2xTMT6plex [N-Term; K7]	1.967			2.873739481	31.49	0	1332.829144	2	666.9179688	-0.362664639	-0.000241685	80.67926804
																			NMINIISR	2	15149476	1xTMT6plex [N-Term]			100	1.700363398	28.86	0	1189.692444	2	595.3499756	0.194018106	0.000115411	60.4326089
																			SLTAEIDR	4	15149476	1xTMT6plex [N-Term]	0.659		0.608	2.790519476	27.97	0	1133.636369	2	567.3218384	0.027828864	1.58E-05	40.75643631
																			AAYPDLENPPLLVTPSQQAK	1	15149476	2xTMT6plex [N-Term; K20]				0.779838502		0	2610.454324	2	1305.729126	-1.282737202	-0.001674263	68.96107904
14165439	HNRNPK	heterogeneous nuclear ribonucleoprotein K isoform a	3190	NP_002131.2	49.35344828	1	18	18	459	464	50.996	5.33	1.25	1.16	1.25	1.22	Significant	0.0006																
																			AQPYDPNFYDETYDYGGFTMMFDDR	19	14165439	1xTMT6plex [N-Term]; 2xOxidation [M20; M21]	1.099	1.687	0.534	2.536980152	21.94	0	3319.370977	3	1096.463623	-1.469604271	-0.001610383	100.6311124
																			AQPYDPNFYDETYDYGGFTMMFDDR	137	14165439	1xTMT6plex [N-Term]	0.895	1.052	1.184	4.573762894	57.36	0	3287.381147	3	1096.463623	-1.469604271	-0.001610383	100.6311124
																			AQPYDPNFYDETYDYGGFTMMFDDR	145	14165439	1xTMT6plex [N-Term]; 1xOxidation [M]	1.224	1.158	1.223	3.763697863	39.55	0	3303.376062	3	1096.463623	-1.469604271	-0.001610383	100.6311124
																			GSYGDLGGPIITTQVTIPK	12	14165439	2xTMT6plex [N-Term; K19]	0.659	1.076		2.572460413	35.26	0	2375.358633	2	1188.183716	0.64089296	0.000761175	66.66081801
																			RPAEDMEEEQAFKR	6	14165439	2xTMT6plex [N-Term; K13]	0.626	0.732	0.769	4.839790344	29.76	1	2194.132672	4	549.2880249	-1.095025226	-0.000600658	37.81046466
																			IIPTLEEGLQLPSPTATSQLPLESDAVECLNYQHYK	6	14165439	2xTMT6plex [N-Term; K36]; 1xCarbamidomethyl [C29	100	1.347		3.66831398	32.47	0	4513.357652	4	1129.093262	-1.425806572	-0.001608794	102.717863
																			LLIHQSLAGGIIGVK	2	14165439	2xTMT6plex [N-Term; K15]	1.138			3.909614325	30.84	0	1977.262488	3	659.7587891	-0.340972685	-0.000224731	73.94591676
																			RPAEDMEEEQAFK	26	14165439	2xTMT6plex [N-Term; K13]	1.216	1.227	1.304	5.549616814	30.18	0	2038.031561	3	680.0159302	0.822301958	0.000558626	39.77958254
																			IDEPLEGSEDR	10	14165439	1xTMT6plex [N-Term]	1.582	1.388	1.301	4.151606083	60.44	0	1488.737933	2	744.8726196	0.019975183	1.49E-05	48.34486567
																			IILDLISESPIK	10	14165439	2xTMT6plex [N-Term; K12]	1.854	1.129	0.883	3.984968901	62.87	0	1799.129408	2	900.0669556	-1.541744121	-0.001386899	111.1250044
																			IITITGTQDQIQNAQYLLQNSVK	3	14165439	2xTMT6plex [N-Term; K23]		0.796		4.269042969	50.97	0	3047.71412	3	1016.574585	-1.613824983	-0.001639492	91.19812253
																			GGDLMAYDR	6	14165439	1xTMT6plex [N-Term]	1.438	0.524	1.335	3.371819735	54.31	0	1226.603688	2	613.8046265	-1.395683324	-0.000855975	40.63766534
																			NTDEMVELR	10	14165439	1xTMT6plex [N-Term]	1.355	1.447	1.595	4.127274036	66.63	0	1335.677581	2	668.3426514	0.33295788	0.000222362	47.96376141
																			ENTQTTIK	10	14165439	2xTMT6plex [N-Term; K8]	1.642	1.829	1.545	3.901585817	41.43	0	1392.809865	2	696.9088745	0.435919829	0.000303577	33.51114001
																			VVLIGGKPDR	7	14165439	2xTMT6plex [N-Term; K7]	1.273	1.123		3.956680059	22.82	0	1511.967369	3	504.6596069	-2.051240842	-0.001033803	47.89348539
																			RPAEDMEEEQAFK	12	14165439	2xTMT6plex [N-Term; K13]; 1xOxidation [M6	1.25	1.241	1.252	4.294660091	23.88	0	2054.026476	3	680.0159302	0.822301958	0.000558626	39.77958254
																			GSDFDCELR	6	14165439	1xTMT6plex [N-Term]; 1xCarbamidomethyl [C6	1.35	1.287	0.3	3.334993362	47.49	0	1327.614981	2	664.3115845	0.686091469	0.000455433	40.1585394
																			GGDLMAYDR	6	14165439	1xTMT6plex [N-Term]; 1xOxidation [M5]	1.533	1.348	1.347	2.378920317	47.33	0	1242.598603	2	613.8046265	-1.395683324	-0.000855975	40.63766534
																			NTDEMVELR	10	14165439	1xTMT6plex [N-Term]; 1xOxidation [M5]	1.471	1.462	1.235	2.960613728	40.7	0	1351.672496	2	668.3426514	0.33295788	0.000222362	47.96376141
																			NAGAVIGK	3	14165439	2xTMT6plex [N-Term; K8]		1.379	0.699	3.364189386	48.49	0	1187.751228	2	594.3803101	1.78082509	0.001057589	35.41546896
																			AQPYDPNFYDETYDYGGFTMMFDDRR	2	14165439	1xTMT6plex [N-Term]		100		1.910180092	1.27	1	3443.482258	3	1148.49707	-1.62635149	-0.001866771	95.41601058
																			DYDDMSPR	3	14165439	1xTMT6plex [N-Term]; 1xOxidation [M5]	0.869		1.266	2.515714645	21.21	0	1243.546233	2	614.2799683	1.092902119	0.000670797	32.01073719
																			DYDDMSPR	6	14165439	1xTMT6plex [N-Term]	1.068	1.059	1.343	3.057422161	37.34	0	1227.551318	2	614.2799683	1.092902119	0.000670797	32.01073719
																			LFQECCPHSTDR	2	14165439	1xTMT6plex [N-Term]; 2xCarbamidomethyl [C5; C6				1.635980248	2.16	0	1778.815155	3	593.609436	-0.786975829	-0.000466628	43.87081801
10835063	NPM1	nucleophosmin isoform 1	4869	NP_002511.1	56.80272109	1	17	17	308	294	32.555	4.78	1.36	1.3	1.41	1.36	Significant	0.00055																
																			LAADEDDDDDDEEDDDEDDDDDDFDDEEAEEKAPVK	4	10835063	3xTMT6plex [N-Term; K32; K36]	3.204			3.709693909	29.18	1	4805.944391	4	1202.241699	0.119875503	0.000144029	60.00820769
																			LAADEDDDDDDEEDDDEDDDDDDFDDEEAEEK	95	10835063	2xTMT6plex [N-Term; K32]	1.893	100	7.825	2.857997656	23.16	0	4181.528204	4	1046.135132	-2.273576976	-0.002376757	56.134625
																			VDNDENEHQLSLR	18	10835063	1xTMT6plex [N-Term]	1.704	2.034	1.849	4.958200455	63.13	0	1797.89287	3	599.9633179	-9.716878579	-0.005823302	36.35024517
																			LAADEDDDDDDEEDDDEDDDDDDFDDEEAEEKAPVKK	6	10835063	4xTMT6plex [N-Term; K32; K36; K37	0.93		0.403	6.283786774	33.42	2	5163.202286	5	1033.446777	0.483104983	0.000498874	65.78289264
																			MTDQEAIQDLWQWR	16	10835063	1xTMT6plex [N-Term]	2.538	2.778	3.306	3.792027712	54.28	0	2049.006126	2	1025.007324	0.608069192	0.000622969	94.8607536
																			TVSLGAGAKDELHIVEAEAMNYEGSPIK	4	10835063	3xTMT6plex [N-Term; K9; K28]; 1xOxidation [M20	1.026			4.665376663	9.25	1	3632.944874	4	904.9955444	2.872299726	0.002597241	92.1350012
																			TVSLGAGAKDELHIVEAEAMNYEGSPIK	8	10835063	3xTMT6plex [N-Term; K9; K28]	1.901	0.905	0.923	5.178278446	14.72	1	3616.949959	4	904.9955444	2.872299726	0.002597241	92.1350012
																			DELHIVEAEAMNYEGSPIK	19	10835063	2xTMT6plex [N-Term; K19]	1.848	1.773	2.632	4.993455887	39.16	0	2603.342724	3	868.4522705	-0.179083821	-0.000155406	80.95717396
																			DELHIVEAEAMNYEGSPIK	8	10835063	2xTMT6plex [N-Term; K19]; 1xOxidation [M11	1.295	100		2.786025047	23.67	0	2619.337639	3	868.4522705	-0.179083821	-0.000155406	80.95717396
																			MSVQPTVSLGGFEITPPVVLR	12	10835063	1xTMT6plex [N-Term]	1.719	1.304		3.368352652	18.47	0	2456.378433	3	819.4641113	-0.265437775	-0.000217339	100.4343175
																			GPSSVEDIK	23	10835063	2xTMT6plex [N-Term; K9]	0.596	1.059	0.96	3.447304964	49.33	0	1389.798966	2	695.4032593	0.198384582	0.000137857	49.65184945
																			ADKDYHFKVDNDENEHQLSLR	4	10835063	3xTMT6plex [N-Term; K3; K8]		0.213	0.274	4.489004135	13.74	2	3260.690314	5	652.9381104	-8.853567621	-0.005773748	43.98520811
																			TVSLGAGAK	26	10835063	2xTMT6plex [N-Term; K9]	1.739	1.69	1.486	4.093010902	50.29	0	1261.788008	2	631.3974609	-0.287247143	-0.000181223	38.92758488
																			SAPGGGSK	14	10835063	2xTMT6plex [N-Term; K8]	1.226	1.369	1.429	2.586324215	38.74	0	1118.656994	2	559.8325195	0.687299596	0.000384426	16.0991507
																			MSVQPTVSLGGFEITPPVVLR	2	10835063	1xTMT6plex [N-Term]; 1xOxidation [M1]				1.09464395	2.28	0	2472.373348	3	819.4641113	-0.265437775	-0.000217339	100.4343175
																			VTLATLK	9	10835063	2xTMT6plex [N-Term; K7]	1.398	1.401	1.007	3.107343674	40.95	0	1203.807681	2	602.4077148	0.392510787	0.000236254	61.34883858
																			MQASIEK	13	10835063	2xTMT6plex [N-Term; K7]	1.423	1.044	1.531	2.91961503	36.18	0	1264.733529	2	632.8704834	0.127194283	8.04E-05	41.56763547
																			LLSISGK	32	10835063	2xTMT6plex [N-Term; K7]	1.541	1.853	1.824	3.144526958	34.2	0	1175.77638	2	588.3917236	-0.178430506	-0.000104897	52.35440633
																			ADKDYHFK	7	10835063	3xTMT6plex [N-Term; K3; K8]	1.41	1.396		3.155201197	19.84	1	1710.978217	3	570.9985962	1.764081032	0.001006101	37.74410464
																			SKGQESFK	4	10835063	3xTMT6plex [N-Term; K2; K8]		1.133	0.985	2.640249014	11.83	1	1597.951668	3	533.3217773	-0.556430315	-0.000296383	32.53636054
74136883	HNRNPU	heterogeneous nuclear ribonucleoprotein U isoform a	3192	NP_114032.2	32.48484848	1	26	26	151	825	90.528	6	1.24	1.06	1.19	1.16	Significant	0.0021																
																			AEGGGGGGRPGAPAAGDGK	5	74136883	2xTMT6plex [N-Term; K19]			1.244	2.036667585	19.81	0	1997.056471	3	666.3569946	-0.020216394	-1.35E-05	22.53922482
																			KDCEVVMMIGLPGAGK	10	74136883	3xTMT6plex [N-Term; K1; K16]; 1xCarbamidomethyl [C3	1.302	1.315	1.439	5.451095581	40.74	1	2392.336941	3	798.1168823	-0.354163048	-0.000282426	80.29862091
																			FDENDVITCFANFESDEVELSYAK	13	74136883	2xTMT6plex [N-Term; K24]; 1xCarbamidomethyl [C9		1.502	1.802	3.506815434	31.2	0	3300.565861	3	1100.859741	-0.360625189	-0.000396756	107.9008785
																			YNILGTNTIMDK	8	74136883	2xTMT6plex [N-Term; K12]	1.409	1.398	1.231	3.821181059	44.81	0	1841.024291	2	921.0148315	-1.034665361	-0.000952422	73.80950568
																			EKPYFPIPEEYTFIQNVPLEDR	14	74136883	2xTMT6plex [N-Term; K2]	1.09	1.192	1.376	5.584051132	41.7	0	3182.681423	3	1061.564819	-0.477038693	-0.000506087	94.80903947
																			TCNCETEDYGEKFDENDVITCFANFESDEVELSYAK	4	74136883	3xTMT6plex [N-Term; K12; K36]; 3xCarbamidomethyl [C2; C4; C21			1.685	4.544220448	33.04	1	5016.262851	5	1004.056396	-1.988519825	-0.001994988	99.69063686
																			KDCEVVMMIGLPGAGK	2	74136883	3xTMT6plex [N-Term; K1; K16]; 1xCarbamidomethyl [C3]; 1xOxidation [M8]		1.856		2.66812849	12.52	1	2408.331856	3	798.1168823	-0.354163048	-0.000282426	80.29862091
																			GYFEYIEENK	9	74136883	2xTMT6plex [N-Term; K10]	0.896	100		3.269897461	36.9	0	1749.909973	2	875.4584961	-0.147168999	-0.000128766	82.77648495
																			GNFTLPEVAECFDEITYVELQK	6	74136883	2xTMT6plex [N-Term; K22]; 1xCarbamidomethyl [C11		0.407	100	3.285927057	20.65	0	3060.56401	3	1020.859863	0.335318506	0.000342088	108.1878643
																			NFILDQTNVSAAAQR	6	74136883	1xTMT6plex [N-Term]	100	1.029	100	2.256869078	27.53	0	1877.00784	2	939.0083618	0.855911615	0.000803276	51.47533573
																			KMCLFAGFQR	2	74136883	2xTMT6plex [N-Term; K1]; 1xCarbamidomethyl [C3		0.315		2.399919987	19.82	1	1715.948959	3	572.6542969	-0.362140725	-0.000207138	72.64199425
																			SSGPTSLFAVTVAPPGAR	8	74136883	1xTMT6plex [N-Term]				2.624147415	35.32	0	1944.075192	2	972.5401611	-1.10391017	-0.001073042	66.08672289
																			VSELKEELK	9	74136883	3xTMT6plex [N-Term; K5; K9]	1.737	100	1.332	1.932652593	14.75	1	1762.092912	2	881.548645	-1.645152147	-0.001449455	52.3599754
																			DLPEHAVLK	2	74136883	2xTMT6plex [N-Term; K9]	1.022			3.020475626	23.31	0	1479.893535	3	493.9695129	0.30424375	0.000150083	48.61143667

Bhat et al,2017. Cigarette smoke and chewing tobacco alter expression of different sets of miRNAs in oral keratinocytes

Supplementary Table S2(A). List of all proteins and peptides identified in OKF6/TERT1 cells chronically treated with chewing tobacco using Sequest and Mascot search algorithms

Accession	Gene_Symbol	Description	Gene_ID	Refseq_Accession	Coverage	Proteins	#Unique_Peptides	#Peptides	#PSMS	#AAs	MW [kDa]	calc. pI	OKF6-STE-6M/OKF6P_R1	OKF6-STE-6M/OKF6P_R2	OKF6-STE-6M/OKF6P_R3	Median fold change	Status	P-value (6M)	Sequence	#PSMSs	Protein Accessions	Modifications	OKF6-STE-6M/OKF6P_R1	OKF6-STE-6M/OKF6P_R2	OKF6-STE-6M/OKF6P_R3	Xcorr	Ionscore	# Missed Cleavages	Theo. MH+ [Da]	Charge	m_z	DeltaM [ppm]	Deltam/z [Da]	RT [min]
																			LLEQYKEESK	7	74136883	3xTMT6plex [N-Term; K6; K10]	0.54		0.776	2.791793823	10.35	1	1954.146404	3	652.0537109	0.089651676	5.84E-05	47.70411859
																			DIDIHEVR	2	74136883	1xTMT6plex [N-Term]	1.156			3.343386173	36.29	0	1225.673817	2	613.3408203	0.446525815	0.000273648	45.05785955
																			KAVVVCPK	2	74136883	3xTMT6plex [N-Term; K1; K8]; 1xCarbamidomethyl [C6		0.887		3.563098669	16.79	1	1588.022331	3	530.0119629	-0.626828795	-0.000331806	41.98619261
																			MMVAGFK	6	74136883	2xTMT6plex [N-Term; K7]	1.138	0.953		2.91076231	29.54	0	1241.715043	2	621.3616943	0.861286075	0.000534736	44.01743947
																			AVVVCPK	4	74136883	2xTMT6plex [N-Term; K7]; 1xCarbamidomethyl [C5	1.468	1.165		2.984856844	44.17	0	1230.764436	2	615.8851318	-1.177177438	-0.000724414	43.1439897
																			KAEGGGGGGRPGAPAAGDGK	1	74136883	3xTMT6plex [N-Term; K1; K20]	1.725			1.715833306		1	2354.314366	4	589.3346558	1.030957	0.000606799	32.2631099
																			FIEIAAR	12	74136883	1xTMT6plex [N-Term]	1.343	1.08	1.358	2.540248394	29.24	0	1048.635246	2	524.8217163	0.867315104	0.000454749	56.93751986
																			DCEVVMMIGLPGAGK	1	74136883	2xTMT6plex [N-Term; K15]; 1xCarbamidomethyl [C2				1.502036691		0	2035.079046	2	1018.042664	-0.48917587	-0.000497756	75.75955713
																			LNTLLQR	3	74136883	1xTMT6plex [N-Term]	1.59			2.647099733	30.35	0	1086.683259	2	543.8450928	-0.322405928	-0.000175177	61.56446945
																			MCLFAGFQR	2	74136883	1xTMT6plex [N-Term]; 1xCarbamidomethyl [C2	0.976			1.635525942	3.1	0	1358.691064	2	679.848877	-0.431697658	-0.000293272	85.67601937
																			ASYGVSK	4	74136883	2xTMT6plex [N-Term; K7]		1.977	1.423	3.046255827	25.96	0	1169.693045	2	585.3469849	-5.43017116	-0.003175817	35.10744939
																			KAEVEGK	8	74136883	3xTMT6plex [N-Term; K1; K7]	0.936	0.994	1.051	3.017005682	13.91	1	1447.908741	3	483.307251	-1.064204027	-0.000513623	34.93276236
																			GKVCFEMK	1	74136883	3xTMT6plex [N-Term; K2; K8]; 1xCarbamidomethyl [C4	1.316			1.358723164	17.11	1	1685.968581	3	562.6627808	3.089236191	0.001736118	34.80651295
94721241	IARS	isoleucine--tRNA ligase, cytoplasmic	3376	NP_002152.2	27.02060222	1	27	27	133	1262	144.406	6.15	1.1	1.26	1.46	1.27	Significant	0.0066																
																			GSELEITLTR	14	94721241	1xTMT6plex [N-Term]	1.56	1.521	1.284	3.998073578	56.1	0	1347.768111	2	674.3876343	-0.088290642	-5.95E-05	50.34714861
																			EEEGTGVVHQAPYFGAEDYR	8	94721241	1xTMT6plex [N-Term]	0.822		1.627	5.140025616	42.94	0	2483.167648	3	828.3948364	0.929533431	0.000769396	45.65277005
																			LFLNETQTQEITEDIPVK	4	94721241	2xTMT6plex [N-Term; K18]		1.513		3.331964254	36.49	0	2576.422355	3	859.4786987	-0.31518846	-0.000270686	92.81907169
																			YAHQSGFHVDR	4	94721241	1xTMT6plex [N-Term]	1.016	1.146		3.834452391	35.99	0	1545.77599	3	515.9296265	-1.07656315	-0.000554708	30.0895737
																			SVVTSIFGVK	4	94721241	2xTMT6plex [N-Term; K10]	2.296			3.995013952	47.93	0	1494.929586	2	747.9677734	-0.88045284	-0.000658107	80.37581967
																			EIVVIHQDPEALK	10	94721241	2xTMT6plex [N-Term; K13]	1.269	1.117	1.502	3.924067259	46.41	0	1949.147184	3	650.3832397	-6.165558876	-0.004005861	49.61708402
																			VMPFSTACNTPLSNFESHQNYK	4	94721241	2xTMT6plex [N-Term; K22]; 1xCarbamidomethyl [C8	100	0.798		3.930550098	19.15	0	3030.485383	3	1010.83313	-0.180344563	-0.000182177	79.98032263
																			LYLINSPVVR	5	94721241	1xTMT6plex [N-Term]	1.341	100	100	3.721344471	48.88	0	1402.861952	2	701.93396	-0.932734774	-0.000654249	72.60497619
																			LQKEEEIEFLYNENTVR	4	94721241	2xTMT6plex [N-Term; K3]	2.662	100		3.305778742	17.23	1	2612.397203	3	871.4696045	-1.126418844	-0.000980884	83.4102002
																			APLKPYPVSPSDK	4	94721241	3xTMT6plex [N-Term; K4; K13]	1.246		1.132	4.599676609	18.35	0	2086.251538	3	696.0882568	-0.633131798	-0.000440291	54.03632896
																			QLSSEELEQFQK	6	94721241	2xTMT6plex [N-Term; K12]	1.866		100	3.331996202	28.85	0	1924.042778	2	962.5238037	-1.271925008	-0.001223619	62.8281325
																			LMAPYTPFLTELMYQNLK	10	94721241	2xTMT6plex [N-Term; K18]	1.056	100	100	2.612827063	19.98	0	2631.43306	3	877.8141479	-1.964462795	-0.001723117	106.8797443
																			AEPDHMVLGK	4	94721241	2xTMT6plex [N-Term; K10]	1.068			3.671135664	53.7	0	1554.87142	2	777.9407959	1.862229146	0.001447763	43.28028608
																			LESDYEILER	2	94721241	1xTMT6plex [N-Term]		100		2.920648813	38.02	0	1495.784155	2	748.3956909	-0.033182637	-2.48E-05	71.52214087
																			YIIEELNVR	4	94721241	1xTMT6plex [N-Term]	1.14	0.91		3.021752834	24.7	0	1377.793932	2	689.4005127	-0.132835086	-9.15E-05	72.80400732
																			FLIQNVLR	4	94721241	1xTMT6plex [N-Term]	0.732	1.182		2.648964167	41.72	0	1231.772409	2	616.3895264	-0.513484164	-0.000316248	86.26317699
																			KTESAVSQMQSVIELGR	4	94721241	2xTMT6plex [N-Term; K1]	0.564			3.035241127	21.47	1	2321.289901	3	774.4328003	-2.607843635	-0.002017854	63.17620355
																			SDTPLIYK	2	94721241	2xTMT6plex [N-Term; K8]			1.86	2.99638176	32.81	0	1394.829538	2	697.9180298	-0.541399265	-0.00037758	55.49367367
																			VLIDPVSVQDKDTLSIHYLMLPR	2	94721241	2xTMT6plex [N-Term; K11]		100		1.833648562	10.83	1	3110.768798	4	778.4471436	-0.66026805	-0.000513485	80.81343542
																			VCMDFNIIR	6	94721241	1xTMT6plex [N-Term]; 1xCarbamidomethyl [C2		100		2.231794357	23.68	0	1396.727843	2	698.8670044	-0.795459862	-0.00055552	68.19877614
																			DSLPVCPVDASGCFTTEVTDFAGQYVK	3	94721241	2xTMT6plex [N-Term; K27]; 2xCarbamidomethyl [C6; C13				1.413109779	7.6	0	3421.669615	3	1141.226685	-1.202351345	-0.00137135	97.4232395
																			AVPSWFVR	2	94721241	1xTMT6plex [N-Term]	0.918			2.767061472	24.28	0	1190.688345	2	595.8478394	0.048216595	2.87E-05	76.01213565
																			KVTLSTDK	7	94721241	3xTMT6plex [N-Term; K1; K8]	1.046	1.333		3.363892317	17.36	1	1579.003369	3	527.0049438	-1.957467027	-0.001030282	38.54182553
																			DVLLPWYNAYR	6	94721241	1xTMT6plex [N-Term]	100	100		1.607641459	12.39	0	1638.884144	2	819.9456787	-0.038390832	-3.15E-05	79.78979367
																			WIDFDNDYK	6	94721241	2xTMT6plex [N-Term; K9]	1.323	100	1.467	2.192079306	14.83	0	1673.857544	2	837.4335327	1.341302727	0.001122575	77.0259686
																			VTLSTDK	2	94721241	2xTMT6plex [N-Term; K7]	1.369			2.708070517	34.07	0	1221.745474	2	611.3762207	-0.253239121	-0.000154697	45.26550276
																			VENMVDQLLR	2	94721241	1xTMT6plex [N-Term]	1.531			1.047065496	11.53	0	1445.798365	2	723.4038696	1.450705619	0.001048714	76.80975295
44889481	MYO1B	unconventional myosin-Ib isoform 2	4430	NP_036355.2	28.66419295	1	27	27	120	1078	124.872	9.2	0.76	0.66	0.96	0.79	Significant	0.012																
																			GDPLGGVISNYLLEK	6	44889481	2xTMT6plex [N-Term; K15]	0.596		100	2.719846964	46.2	0	2033.168313	2	1017.085876	-1.886995921	-0.00191829	92.05239968
																			AAHIFNEALVCHQIR	8	44889481	1xTMT6plex [N-Term]; 1xCarbamidomethyl [C11	0.637	0.59		4.935814381	62.48	0	2008.074815	4	502.7745361	0.746840326	0.000374928	47.621866
																			EQLLQSNPVLEAFGNAK	6	44889481	2xTMT6plex [N-Term; K17]		1.31		3.782495022	44.14	0	2316.296367	3	772.7693481	-1.241471708	-0.000958539	90.65686252
																			EAVTTIAAYWHGTQVR	2	44889481	1xTMT6plex [N-Term]	1.692			3.411062479	26.3	0	2032.08134	3	678.0317993	-0.243607759	-0.00016501	77.76912213
																			DQDKDQCILITGESGAGK	4	44889481	3xTMT6plex [N-Term; K4; K18]; 1xCarbamidomethyl [C7	1.143			4.980975628	26.6	1	2622.401192	3	874.8047485	-0.571775416	-0.000499808	56.82427426
																			LNQVCATHQHFESR	2	44889481	1xTMT6plex [N-Term]; 1xCarbamidomethyl [C5	0.493			5.321419716	28.35	0	1955.970744	3	652.6625977	1.276061392	0.00083198	33.42639044
																			NWPSRPYLFLDSTHK	2	44889481	2xTMT6plex [N-Term; K15]	0.773			3.016181469	11.98	0	2319.265007	4	580.5720215	0.53847555	0.000312217	75.23511195
																			GDFLFSSDHLIEMATK	8	44889481	2xTMT6plex [N-Term; K16]	100			2.758662939	19.19	0	2269.193876	3	757.0683594	-1.476627906	-0.001116918	87.66131569
																			IIIAEVVNK	6	44889481	2xTMT6plex [N-Term; K9]	0.8	0.623	0.761	4.170514584	41.44	0	1456.950322	2	728.9784546	-0.473159793	-0.000344685	78.36757735
																			MPSLSPIDK	4	44889481	2xTMT6plex [N-Term; K9]	1.227	1.155		3.423826218	48.35	0	1445.843808	2	723.4254761	-0.091594653	-6.62E-05	68.45751625
																			SLPIYSPEK	6	44889481	2xTMT6plex [N-Term; K9]	0.87	0.838	0.885	3.357437134	34.79	0	1491.882302	2	746.4441528	-0.853201312	-0.000636438	50.18213732
																			LEASELFK	6	44889481	2xTMT6plex [N-Term; K8]	0.873	1.093	100	3.46869278	43.74	0	1394.829538	2	697.9196167	1.734024389	0.001209334	70.13169906
																			KIYEFTLQR	6	44889481	2xTMT6plex [N-Term; K1]	0.937	0.461	0.599	3.362909555	15.92	1	1655.988498	3	552.6674194	-0.479006478	-0.00026441	51.68548176
																			LEDLATLIQK	6	44889481	2xTMT6plex [N-Term; K10]	100	1.317	100	2.37221694	30.96	0	1601.98783	2	801.4970093	-0.678909847	-0.000543803	86.01092332
																			FLNDTSLPHSCFR	4	44889481	1xTMT6plex [N-Term]; 1xCarbamidomethyl [C11	100	100		2.55019021	20.78	0	1822.910769	3	608.3070068	-2.360023463	-0.001434037	51.47929829
																			VLYQVEGFVDK	3	44889481	2xTMT6plex [N-Term; K11]		0.567		1.860143423	20.28	0	1755.009293	2	878.0077515	-0.607928583	-0.00053346	85.31192698
																			SEVPLVDVTK	6	44889481	2xTMT6plex [N-Term; K10]	0.398	0.908	0.702	2.479323626	23.89	0	1544.92998	2	772.968811	0.236334538	0.00018256	57.29464132
																			YNYLSLDSAK	4	44889481	2xTMT6plex [N-Term; K10]	1.188	0.765		2.353766203	22.04	0	1631.904494	2	816.4556885	-0.24113352	-0.000196753	55.95766263
																			DLSQAMWK	2	44889481	2xTMT6plex [N-Term; K8]		0.802		3.409580708	34.49	0	1436.797192	2	718.9012451	-1.37704586	-0.000989268	60.35084739
																			EICELTGIDQSVLER	6	44889481	1xTMT6plex [N-Term]; 1xCarbamidomethyl [C3	0.645	100	100	2.286679983	17.76	0	1991.031672	2	996.0178223	-1.659571164	-0.001652129	67.53146647
																			VLYQVEGFVDKNNDLLYR	4	44889481	2xTMT6plex [N-Term; K11]			100	1.697138548	2.54	1	2643.454658	3	881.8215332	-1.744637689	-0.00153729	88.82255528
																			RPPTAGSQFK	2	44889481	2xTMT6plex [N-Term; K10]			1.046	2.698130131	11.94	0	1546.910582	3	516.3085327	0.299011809	0.000154182	33.9374694
																			IYEFTLQR	6	44889481	1xTMT6plex [N-Term]	100	100	1.003	2.199339151	26.54	0	1298.730603	2	649.8684692	-0.72491049	-0.000470732	58.44357976
																			LKDAIEEK	2	44889481	3xTMT6plex [N-Term; K2; K8]			0.95	3.213499308	3.94	1	1633.013934	3	545.0090942	-0.737492272	-0.000401445	43.75408946
																			YLGLLENVR	6	44889481	1xTMT6plex [N-Term]	100		100	2.202150583	26.25	0	1305.772802	2	653.3890381	-1.53389481	-0.001001459	76.67320375
																			VSMSSQNDGFFAVHLK	2	44889481	2xTMT6plex [N-Term; K16]			1.164	1.312887311	1.09	0	2225.178894	3	742.3981934	0.508955989	0.000377506	79.96390007
																			LVMSYVAAVCGK	1	44889481	2xTMT6plex [N-Term; K12]; 1xCarbamidomethyl [C10				1.127176881		0	1755.990155	2	878.4988403	0.141922244	0.000124607	83.24866306
71361682	NUMA1	nuclear mitotic apparatus protein 1 isoform	4926	NP_006176.2	13.8534279	1	25	25	110	2115	238.115	5.78	1.21	1.19	1.13	1.17	Significant	0.00042																
																			KLDVEEPDSANSSFYSTR	2	71361682	2xTMT6plex [N-Term; K1]	1.584			5.347237587	32.51	1	2503.271668	3	835.0968018	1.671410055	0.001394664	59.32902168
																			EELEQASQAHGAR	2	71361682	1xTMT6plex [N-Term]		1.565		5.529663563	69.8	0	1654.834627	3	552.2826538	-0.736644133	-0.000406341	27.84450661
																			GEVLGDVLQLETLK	4	71361682	2xTMT6plex [N-Term; K14]	100			2.703212976	43.69	0	1972.173064	2	986.5905762	0.411629063	0.000405902	94.60477638
																			FVLDHEDGLNLNEDLENFLQK	5	71361682	2xTMT6plex [N-Term; K21]	2.319			4.724053383	24.64	0	2960.540572	3	987.5186768	0.305533201	0.000301514	100.5076889
																			AVQAQGGESQQEAQR	22	71361682	1xTMT6plex [N-Term]	1.288	1.368	1.245	5.268461704	78.02	0	1815.914668	3	605.9762573	-0.24759277	-0.000149869	17.87399017
																			KVEELQACVETAR	4	71361682	2xTMT6plex [N-Term; K1]; 1xCarbamidomethyl [C8		1.501	1.266	5.222165585	47.59	1	1991.099582	3	664.3719482	0.858699674	0.000569919	47.90964235
																			SLEAQVAHADQQLR	6	71361682	1xTMT6plex [N-Term]	1.145	1.145	1.21	4.615087032	47.97	0	1794.965976	3	598.9934692	-0.067530615	-4.04E-05	43.43041235
																			EKEHASGSGAQSEAAGR	2	71361682	2xTMT6plex [N-Term; K2]		2.153		4.290086269	35.5	1	2130.093979	4	533.2788086	-0.269530361	-0.000143531	15.92952885
																			AQELGHSQSALASAQR	4	71361682	1xTMT6plex [N-Term]	1.035	1.537		5.077943802	39.66	0	1882.993253	3	628.3357544	-0.288405922	-0.000181022	39.50682823
																			EHASGSGAQSEAAGR	8	71361682	1xTMT6plex [N-Term]	0.637	100		3.290969849	39.27	0	1643.793491	3	548.6027222	0.074585954	4.09E-05	12.31623367
																			QQNELAELHANLAR	2	71361682	1xTMT6plex [N-Term]	0.689			5.240859032	38.2	0	1835.992525	3	612.6680908	-1.528088499	-0.000935186	47.80765344
																			DSAQTSVTQAQR	4	71361682	1xTMT6plex [N-Term]	100			2.517693281	56.97	0	1520.786614	2	760.8973999	0.597562261	0.000454382	19.95274017
																			HLCQQLQAEQAAAEK	2	71361682	2xTMT6plex [N-Term; K15]; 1xCarbamidomethyl [C3			0.883	3.889618635	22.19	0	2183.164307	3	728.3881836	-6.554535865	-0.004769876	41.29690524
																			ASYAEQLSMLK	5	71361682	2xTMT6plex [N-Term; K11]	100			2.669461012	30.97	0	1698.950064	2	849.9772949	-1.618991817	-0.001375293	63.36647603
																			AQADLALEK	6	71361682	2xTMT6plex [N-Term; K9]	1.04	1.17	1.158	4.063237667	49.44	0	1416.846251	2	708.927063	0.422478136	0.000299293	44.25116862
																			IHGTEEGQQILK	2	71361682	2xTMT6plex [N-Term; K12]	1.135			2.550173283	5.17	0	1811.042719	3	604.3513794	-1.730282988	-0.001044539	33.49448213
																			AADALEEQQR	2	71361682	1xTMT6plex [N-Term]	0.942			3.223713398	29.96	0	1359.706573	2	680.3582764	1.987799742	0.001351412	26.54265913
																			GLEAAQIK	4	71361682	2xTMT6plex [N-Term; K8]	1.206	0.294		2.801563501	37.21	0	1287.803658	2	644.4054565	-0.016535178	-1.06E-05	52.53072122
																			ELEVMTAK	4	71361682	2xTMT6plex [N-Term; K8]		1.323	1.234	3.475536346	31.57	0	1378.801609	2	689.9051514	1.027924805	0.000708652	52.0344465
																			ATQEWLEK	6	71361682	2xTMT6plex [N-Term; K8]	1.018	1.08		2.066043615	12.87	0	1462.830601	2	731.9188843	-0.074359473	-5.44E-05	48.17881081
																			LADDLSTLQEK	4	71361682	2xTMT6plex [N-Term; K11]	100		100	1.78769815	9.3	0	1690.962737	2	845.9835205	-1.757882838	-0.001486257	60.74289021
																			ELGELIPLR	2	71361682	1xTMT6plex [N-Term]	1.808			2.41997206	21.25	0	1268.777553	2	634.8928223	0.641886553	0.000407206	95.7113603
																			ATSSTQSLAR	2	71361682	1xTMT6plex [N-Term]				1.498381853	12.57	0	1250.690195	2	625.848938	0.323306734	0.000202178	22.5453086
																			LALLNEK	4	71361682	2xTMT6plex [N-Term; K7]			100	1.589406252	14.99	0	1258.813494	2	629.909668	-1.139861073	-0.000717436	60.08068139
																			LVMAESEK	2	71361682	2xTMT6plex [N-Term; K8]			0.854	1.760888934	10.33	0	1364.785959	2	682.8973999	1.146285744	0.000782217	51.33684706
14043072	HNRNPA2B1	heterogeneous nuclear ribonucleoproteins A2/B1 isoform B1	3181	NP_112533.1	44.19263456	1	13	18	249	353	37.407	8.95	1.19	1.11	1.32	1.21	Significant	0.0026																
																			KIFVGGIKEDTEEHHLR	28	14043072	3xTMT6plex [N-Term; K1; K8]	0.526	0.444		8.3095541	38.13	2	2695.549845	5	539.9158936	0.191487254	0.000103233	44.71434515
																			GFGFVTFDDHDPVDK	18	14043072	2xTMT6plex [N-Term; K15]	1.064	1.147	1.229	3.791395187	42.33	0	2154.090791	3	718.7017212	-0.083792095	-6.02E-05	81.15038562
																			GGGGNFGPGPGSNFR	12	14043072	1xTMT6plex [N-Term]	1.455	1.823	1.198	4.41251421	76.54	0	1606.792369	2	803.9000854	0.327185048	0.000262859	47.5410783
																			NMGGPYGGGNYGPGGSGGSGGYGGR	6	14043072	1xTMT6plex [N-Term]	1.046		1.276	5.689226627	96.09	0	2419.068285	3	807.0284424	1.028874816	0.000829639	46.35303318
																			KLFIGGLSFETTEESLR	12	14043072	2xTMT6plex [N-Term; K1]	0.741	0.963	0.837	4.768558979	33.11	1	2385.342983	3	795.7841797	-2.094792043	-0.001665599	80.90747616
																			LFVGGIKEDTEEHHLR	12	14043072	2xTMT6plex [N-Term; K7]	0.808	0.916		6.502017021	37.12	1	2338.29195	4	585.328186	-0.44300686	-0.00025897	50.517852
																			GGNFGFGDSR	10	14043072	1xTMT6plex [N-Term]	1.352	1.259	1.27	3.403071404	51.65	0	1242.606465	2	621.807312	0.709768911	0.000440982	41.77400868
																			EESGKPGAHVTVK	20	14043072	3xTMT6plex [N-Term; K5; K13]	1.387	1.325	1.398	3.41463232	15.72	0	2026.19	3	676.0682983	0.168434121	0.00011376	27.45752313
																			IDTIEIITDR	27	14043072	1xTMT6plex [N-Term]	1.711	100	100	3.920673609	43.55	0	1417.809976	2	709.4082642	-0.510653543	-0.000362005	73.48551927
																			QEMQEVQSSR	8	14043072	1xTMT6plex [N-Term]	1.413	1.45	0.341	3.776994944	49.06	0	1450.715758	2	725.8624878	1.33811598	0.000970613	31.79752922
																			LFIGGLSFETTEESLR	27	14043072	1xTMT6plex [N-Term]	2.195	100	1.703	2.894027948	21.12	0	2028.085088	2	1014.544922	-1.242807817	-0.00126026	91.59591903
																			DYFEEYGKIDTIEIITDR	4	14043072	2xTMT6plex [N-Term; K8]		0.01	0.01	3.036708832	15.7	1	2678.396534	3	893.4690552	-1.464257665	-0.001307288	91.75993317
																			KLFVGGLK	72	14043072	3xTMT6plex [N-Term; K1; K8]	0.753	0.812	0.878	3.817184925	23.58	1	1549.044446	3	517.0199585	0.565534632	0.000292013	58.62888589
																			NYYEQWGK	12	14043072	2xTMT6plex [N-Term; K8]	1.292	1.817	1.463	3.861892462	39.02	0	1545.8102	2	773.4095459	1.045119818	0.000807778	57.97060766
																			EDTEEHHLR	6	14043072	1xTMT6plex [N-Term]	100		100	2.599963427	22.36	0	1394.686172	3	465.5664368	-1.014670751	-0.000471716	23.73549614
																			DYFEEYGK	10	14043072	2xTMT6plex [N-Term; K8]	1.236	1.351	1.069	3.675152779	39.41	0	1508.767332	2	754.8868408	-0.614216225	-0.000463355	62.76696098
																			LTDCVVMR	12	14043072	1xTMT6plex [N-Term]; 1xCarbamidomethyl [C4	1.418	1.421	1.375	3.471637249	38.89	0	1222.64853	2	611.8278198	-0.136819011	-8.36E-05	51.20105504
																			IFVGGIK	48	14043072	2xTMT6plex [N-Term; K7]	2.036	2.477	1.805	2.747451067	32.13	0	1191.786551	2	596.3962402	-1.130564234	-0.000673696	62.34943694
																			LTDCVVMR	5	14043072	1xTMT6plex [N-Term]; 1xCarbamidomethyl [C4]; 1xOxidation [M7]	1.664	1.584	1.362	2.235037804	29.15	0	1238.643445	2	611.8278198	-0.136819011	-8.36E-05	51.20105504
19920317	CKAP4	cytoskeleton-associated protein 4	10970	NP_006816.2	47.17607973	1	23	23	120	602	65.983	5.92	0.8	0.81	0.71	0.77	Significant	0.0017																
																			VQEQVHTLLSQDQAQAAR	4	19920317	1xTMT6plex [N-Term]		0.674	0.711	3.817556858	24.02	0	2251.199223	3	751.0701294	-1.504934069	-0.001129302	48.46670338
																			LQHVEDGVLSMQVASAR	13	19920317	1xTMT6plex [N-Term]	0.77	0.984	0.532	4.460425377	43.59	0	2069.101089	3	690.3722534	0.540410477	0.000372721	65.47481209
																			VASLEESEGNK	2	19920317	2xTMT6plex [N-Term; K11]	1.465			4.561407089	62.26	0	1620.884487	2	810.9454346	-0.551605856	-0.000447045	48.60886386
																			GAHPSGGADDVAK	6	19920317	2xTMT6plex [N-Term; K13]	0.741			4.850695133	66.28	0	1639.880404	3	547.2982788	-0.073873813	-4.04E-05	30.26600458
																			SSSSSSASAAAAAAAASSSASCSR	2	19920317	1xTMT6plex [N-Term]; 1xCarbamidomethyl [C22			0.987	7.726963997	51.19	0	2319.06801	3	773.6899414	-5.493345255	-0.00424648	37.00934404
																			ASVSQVEADLK	6	19920317	2xTMT6plex [N-Term; K11]	0.769	0.804	100	4.179384232	67.12	0	1604.925958	2	802.9665527	-0.080178932	-6.43E-05	66.47980987
																			SLGETQLVLYGDVEELKR	7	19920317	2xTMT6plex [N-Term; K17]	1.259			4.041855335	10.59	1	2507.412125	3	836.473877	-2.012872386	-0.001682367	78.74543584
																			SINDNIAIFTEVQK	7	19920317	2xTMT6plex [N-Term; K14]	1.163			2.751142263	25.48	0	2050.158476	2	1025.583984	1.080731088	0.001107835	78.55009145
																			VASLEESEGNKQDLK	4	19920317	3xTMT6plex [N-Term; K11; K15]	1.061			3.57953167	10.73	1	2334.311966	3	778.7748413	-0.854733321	-0.000665071	56.03316996
																			TAVDSLVAYSVK	4	19920317	2xTMT6plex [N-Term; K12]	0.925		100	2.519304037	35.29	0	1711.004208	2	856.0046997	-1.218603629	-0.001042518	63.94686948
																			DLSDGIHVVK	12	19920317	2xTMT6plex [N-Term; K10]	0.789	0.795	0.689	4.146291256	49.53	0	1540.909914	2	770.9580078	-0.762280124	-0.000587302	44.74212356
																			QTESLESLLSK	4	19920317	2xTMT6plex [N-Term; K11]	100	0.631		3.361988783	38.94	0	1692.978387	2	846.9925537	-0.328514604	-0.000278084	81.00222931
																			VQSLQATFGTFESILR	5	19920317	1xTMT6plex [N-Term]				1.885569692	27.8	0	2026.117057	2	1013.561157	-0.996327862	-0.001009338	100.6938664
																			SVGELPSTVESLQK	4	19920317	2xTMT6plex [N-Term; K14]	1.649	1.667		2.068180561	22.8	0	1932.105378	2	966.5551758	-1.192138651	-0.001151669	57.00898166
																			STLQTMESDIYTEVR	3	19920317	1xTMT6plex [N-Term]				2.356012821	18.66	0	2002.000037	2	1001.50354	-0.116839096	-0.000116956	92.28818505
																			LALQALTEK	6	19920317	2xTMT6plex [N-Term; K9]	0.962	1.151	1.198	2.814226627	31.84	0	1444.913936	2	722.9603882	-0.302206797	-0.000218331	65.10328491
																			LEGLGSSEADQDGLASTVR	2	19920317	1xTMT6plex [N-Term]				1.447386146	6.49	0	2134.082521	2	1067.544067	-0.779334613	-0.000831582	53.35299789
																			LQHVEDGVLSMQVASAR	2	19920317	1xTMT6plex [N-Term]; 1xOxidation [M11]	100			1.683020711	10.18	0	2085.096004	3	690.3722534	0.540410477	0.000372721	65.47481209
																			DFTSLENTVEER	4	19920317	1xTMT6plex [N-Term]				2.066027641	10.19	0	1668.82781	2	834.916626	-1.099650224	-0.000917543	57.73805038
																			EWDMEALR	8	19920317	1xTMT6plex [N-Term]	1.243	0.972	0.971	2.937753201	30.56	0	1278.634988	2	639.8202515	-1.378087045	-0.000881035	66.18665652
																			ISEVLQK	5	19920317	2xTMT6plex [N-Term; K7]	1.051	0.976	0.967	3.078518152	34.62	0	1274.808409	2	637.9086914	1.331496159	0.000848701	59.92801861
																			LQNEILK	4	19920317	2xTMT6plex [N-Term; K7]	0.848		0.746	3.165356874	32.1	0	1315.834958	2	658.4175415	-5.434946187	-0.003575746	49.47744369
																			GLLDDLR	4	19920317	1xTMT6plex [N-Term]	0.385	2.421		2.179655075	26.97	0	1030.609426	2	515.8083496	-0.002950924	-1.52E-06	53.9727013
																			EIQTSAK	2	19920317	2xTMT6plex [N-Term; K7]	0.846			2.880824566	23.56	0	1234.740723	2	617.8747559	1.224507074	0.000755974	21.86496377
14043070	HNRNPA1	heterogeneous nuclear ribonucleoprotein A1 isoform b	3178	NP_112420.1	47.31182796	1	12	17	216	372	38.723	9.13	1.57	1.31	1.08	1.32	Significant	0.011																
																			KIFVGGIKEDTEEHHLR	28	14043070	3xTMT6plex [N-Term; K1; K8]	0.526	0.444		8.3095541	38.13	2	2695.549845	5	539.9158936	0.191487254	0.000103233	44.71434515
																			SSGPYGGGGQYFAKPR	8	14043070	2xTMT6plex [N-Term; K14]	1.02	0.6	0.549	5.678741932	35.76	0	2087.107444	3	696.3729858	-1.4564274	-0.00101324	42.9936827
																			GFAFVTFDDHDSVDK	26	14043070	2xTMT6plex [N-Term; K15]	1.422	1.101	1.901	5.361237049	56.81	0	2158.085705	3	720.032959	-0.640251159	-0.000460572	72.29045153
																			KLFIGGLSFETTDESLR	12	14043070	2xTMT6plex [N-Term; K1]	1.02	0.962	1.328	6.116363525	47.75	1	2371.327333	3	791.1132813	-0.861142185	-0.000680683	86.70220286
																			SHFEQWGTLTDCVVMR	6	14043070	1xTMT6plex [N-Term]; 1xCarbamidomethyl [C12]; 1xOxidation [M15]	2.171		100	3.044971704	33.86	0	2211.052425	3	737.6890869	0.127858654	9.42E-05	66.31343028
																			GGGFGGNDNFGR	4	14043070	1xTMT6plex [N-Term]		1.539		3.442593336	50.92	0	1383.660292	2	692.3331909	-0.857605056	-0.000593317	43.16198606
																			LFVGGIKEDTEEHHLR	12	14043070	2xTMT6plex [N-Term; K7]	0.808	0.916		6.502017021	37.12	1	2338.29195	4	585.328186	-0.44300686	-0.00025897	50.517852
																			SHFEQWGTLTDCVVMR	11	14043070	1xTMT6plex [N-Term]; 1xCarbamidomethyl [C12	1.047	1.805	5.196	4.257493496	28.34	0	2195.05751	3	737.6890869	0.127858654	9.42E-05	66.31343028
																			LFIGGLSFETTDESLR	22	14043070	1xTMT6plex [N-Term]	1.105	0.892	2.823	2.721563101	33.72	0	2014.069438	2	1007.539124	0.761076199	0.00076643	93.91104209
																			EDSQRPGAHLTVK	9	14043070	2xTMT6plex [N-Term; K13]	1.941	1.779	2.006	3.369465113	25.02	0	1896.07033	4	474.7724915	-1.15728813	-0.000548575	32.68906383
																			QEMASASSSQR	4	14043070	1xTMT6plex [N-Term]	100			2.477632046	41.44	0	1410.684458	2	705.84552	-0.492102209	-0.0003471	26.00403247
																			IEVIEIMTDR	26	14043070	1xTMT6plex [N-Term]	1.478	1.225	1.12	3.778140783	40.51	0	1447.802782	2	724.4043579	-0.927384381	-0.000671335	81.07470721
																			KLFVGGLK	72	14043070	3xTMT6plex [N-Term; K1; K8]	0.753	0.812	0.878	3.817184925	23.58	1	1549.044446	3	517.0199585	0.565534632	0.000292013	58.62888589
																			IEVIEIMTDR	8	14043070	1xTMT6plex [N-Term]; 1xOxidation [M7]	100			1.646099091	21.86	0	1463.797697	2	724.4043579	-0.927384381	-0.000671335	81.07470721
																			DYFEQYGK	10	14043070	2xTMT6plex [N-Term; K8]	0.809	1.176	2.319	3.132143736	31.08	0	1507.783316	2	754.394165	-1.500681066	-0.001131351	55.06784793
																			EDTEEHHLR	6	14043070	1xTMT6plex [N-Term]	100		100	2.599963427	22.36	0	1394.686172	3	465.5664368	-1.014670751	-0.000471716	23.73549614
																			GFGFVTYATVEEVDAAMNARPHK	2	14043070	2xTMT6plex [N-Term; K23]				1.890907764	3	0	2968.539133	4	742.8911133	1.175819096	0.000872616	88.06921434
																			IFVGGIK	48	14043070	2xTMT6plex [N-Term; K7]	2.036	2.477	1.805	2.747451067	32.13	0	1191.786551	2	596.3962402	-1.130564234	-0.000673696	62.34943694
																			NQGGYGGSSSSSSYGSGR	2	14043070	1xTMT6plex [N-Term]				1.246583819		0	1923.863027	2	962.4367065	1.616464319	0.001554928	24.19436236
110556636	TNKS1BP1	182 kDa tankyrase-1-binding protein	85456	NP_203754.2	20.70561018	1	24	24	108	1729	181.685	4.86	0.81	0.9	0.87	0.86	Significant	0.00095																
																			VSAPGVLTAQDR	6	110556636	1xTMT6plex [N-Term]	0.785	0.999	0.963	4.171495438	59.38	0	1442.816458	2	721.9129028	1.435362101	0.001035482	42.75971644
																			DQSSWQNSDASQEVGGHQER	4	110556636	1xTMT6plex [N-Term]	1.016	2.128		5.791824341	53.74	0	2474.112986	3	825.3764038	0.675695003	0.000557249	34.18791213
																			EAAFSPGQQDWSR	7	110556636	1xTMT6plex [N-Term]	0.873	0.862	0.935	4.441935539	62.63	0	1707.828814	2	854.4185791	0.625363102	0.000534007	47.86539408
																			NLEVSSCVGSGGSSEAR	6	110556636	1xTMT6plex [N-Term]; 1xCarbamidomethyl [C7	1.156	1.214	1.09	3.879859686	73.42	0	1924.923184	2	962.96521	-0.021324552	-2.05E-05	36.76932878
																			DLAEVGEGGGHSQAR	6	110556636	1xTMT6plex [N-Term]	0.983	0.985	1.064	5.73878479	60.19	0	1711.856091	3	571.2894897	-1.270493314	-0.000724967	29.68600912
																			DLEVTCDPDSGGSQGLR	4	110556636	1xTMT6plex [N-Term]; 1xCarbamidomethyl [C6		0.951	0.854	3.151914835	34.55	0	2034.959964	2	1017.974304	-9.155930286	-0.009315976	44.84239001
																			VVGKPAQLGTQR	5	110556636	2xTMT6plex [N-Term; K4]		0.733	0.696	2.885991096	8.6	0	1712.058309	3	571.357605	-0.027702674	-1.58E-05	32.78905141
																			EMEEELVPTGSEPGDTR	2	110556636	1xTMT6plex [N-Term]		0.777		3.619161367	38.24	0	2104.990595	2	1052.997803	-1.076466211	-0.001132976	46.89532496
																			SAEEGELAESK	2	110556636	2xTMT6plex [N-Term; K11]	1.007			4.246976852	50.05	0	1607.852852	2	804.4309692	1.125561061	0.000904868	41.79944942
																			DAELQDQEFGK	4	110556636	2xTMT6plex [N-Term; K11]	0.659	0.774		4.01335907	60.33	0	1737.90595	2	869.4564209	-0.221601822	-0.000192562	56.31226939
																			AAEPQEQEFGK	2	110556636	2xTMT6plex [N-Term; K11]	0.729			4.278545856	48.8	0	1691.900471	2	846.4546509	0.918551557	0.000777049	42.08772324
																			EMEEELVPTGSEPGDTR	2	110556636	1xTMT6plex [N-Term]; 1xOxidation [M2]	0.264			2.575302124	34.52	0	2120.98551	2	1052.997803	-1.076466211	-0.001132976	46.89532496
																			KIPSVEDSLGEGSR	4	110556636	2xTMT6plex [N-Term; K1]	0.802	0.899		4.129061222	37.72	1	1932.080226	3	644.6983032	0.067418509	4.34E-05	56.29844431
																			DFCIEASER	6	110556636	1xTMT6plex [N-Term]; 1xCarbamidomethyl [C3	1.139	0.923	1.009	3.347091675	46.46	0	1355.646282	2	678.3269043	0.184704339	0.000125197	42.88820984
																			APVIRPGGTLGLSEAADSDAHLFQDSTEPR	6	110556636	1xTMT6plex [N-Term]	100			3.136233091	11.47	0	3336.702162	4	834.9332275	2.672730683	0.002229527	64.47190699
																			SQEADVQDWEFR	4	110556636	1xTMT6plex [N-Term]				2.217971087	20.69	0	1738.823394	2	869.914917	-0.48103541	-0.000418218	58.59678558
																			GWSQEGPVK	6	110556636	2xTMT6plex [N-Term; K9]	0.886	0.801	0.779	3.333996534	47.19	0	1445.815285	2	723.4104614	-1.133470129	-0.000819394	49.72167106
																			DVSLGDWEFGK	6	110556636	2xTMT6plex [N-Term; K11]			100	2.365257502	24.87	0	1710.910307	2	855.9584351	-0.417305811	-0.000356986	72.97177592
																			GVGQADWTPDLGLR	6	110556636	1xTMT6plex [N-Term]	100	100	100	2.218802929	14.2	0	1713.912149	2	857.4589233	-0.921406802	-0.000789605	66.21436722
																			VNLFPGLSPSALK	8	110556636	2xTMT6plex [N-Term; K13]	0.01	100	100	1.806075335	19.72	0	1801.098777	2	901.0526733	-0.392466155	-0.000353435	87.43838295
																			SYQFGIIGNDR	4	110556636	1xTMT6plex [N-Term]	100			2.42474246	20.36	0	1498.785158	2	749.8959351	-0.376613559	-0.000282231	57.49316916
																			DWTSDVNVK	2	110556636	2xTMT6plex [N-Term; K9]		100		2.405361414	17.63	0	1521.831329	2	761.4192505	-0.068722095	-5.23E-05	49.35898234
																			GCGVGQMDWTQDLAPQNVELFGAPSEAR	1	110556636	1xTMT6plex [N-Term]; 1xCarbamidomethyl [C2				1.601899385		0	3262.545862	3	1088.184448	-2.167075572	-0.002356728	89.1278753
																			ESAASGLGGLLEEEGAGAGAAQEEVLEPGR	2	110556636	1xTMT6plex [N-Term]				2.589512825		0	3083.533031	3	1028.516724	0.838793816	0.000862149	83.49021916
																			KAPAPFRPASER	3	110556636	2xTMT6plex [N-Term; K1]	0.701	1.048		3.092945814	1.45	1	1785.053558	4	447.0185547	-0.655005557	-0.000292305	35.79666389
4503509	EIF3A	eukaryotic translation initiation factor 3 subunit A	8661	NP_003741.1	23.08248915	1	33	33	121	1382	166.468	6.79	1.2	1.21	0.97	1.13	Significant	0.0048																
																			HHNQSTAINLNNPESQSMHLETR	2	4503509	1xTMT6plex [N-Term]	2.054			4.414640903	25.17	0	2887.406666	4	722.6074829	0.497283823	0.000358965	26.38338827
																			DIDIEDLEELDPDFIMAK	2	4503509	2xTMT6plex [N-Term; K18]	1.128			3.025684357	48.26	0	2579.320258	3	860.4434204	-1.763918039	-0.00151657	107.4386455
																			EDAPIGPHLQSMPSEQIR	3	4503509	1xTMT6plex [N-Term]	0.971		1.302	5.600793839	39.18	0	2234.143682	3	745.3865967	0.695988837	0.000518313	53.64871764
																			KPPKPQLMANYYNK	2	4503509	4xTMT6plex [N-Term; K1; K4; K14]	1.21			4.800673008	4.15	0	2608.545501	4	652.8919067	0.113501231	7.40E-05	46.63530448
																			ALEVIKPAHILQEK	6	4503509	3xTMT6plex [N-Term; K6; K14]	1.096	1.267	1.375	3.216628075	17.23	0	2276.430899	4	569.8638916	1.24630897	0.000709284	61.92823581
																			ILQEHEQIK	6	4503509	2xTMT6plex [N-Term; K9]	1.129	1.197	1.203	4.361354351	37.95	0	1595.952113	3	532.6555786	0.043754348	2.33E-05	41.15299255
																			ANEFLEVGK	6	4503509	2xTMT6plex [N-Term; K9]	100	100	0.468	3.034390211	30.09	0	1464.846251	2	732.9260254	-1.008029852	-0.000738304	56.53073761
																			NLTQDEMQR	2	4503509	1xTMT6plex [N-Term]	2.155			3.415832758	36.01	0	1363.683729	2	682.3457642	0.383014259	0.000261155	34.47351162
																			TKNETDEDGWTTVR	2	4503509	2xTMT6plex [N-Term; K2]	1.735			3.746926069	26.28	1	2110.081683	3	704.03125	-1.178091834	-0.000828623	47.22546626
																			NICQQVNIK	2	4503509	2xTMT6plex [N-Term; K9]; 1xCarbamidomethyl [C3	0.845			3.341430187	25.61	0	1574.908868	2	787.9575806	-0.624523239	-0.000491784	52.39785574
																			RLEEIPLIK	4	4503509	2xTMT6plex [N-Term; K9]	1.316		1.217	3.183814526	21.98	1	1569.013985	3	523.6759033	-0.527757605	-0.00027602	54.52226371
																			DLYNWLEVEFNPLK	6	4503509	2xTMT6plex [N-Term; K14]	100			2.183545589	12.47	0	2238.221077	2	1119.614014	-0.145640774	-0.000162988	104.5849574
																			DIDIEDLEELDPDFIMAK	2	4503509	2xTMT6plex [N-Term; K18]; 1xOxidation [M16	100			1.739586473	13.69	0	2595.315173	3	860.4434204	-1.763918039	-0.00151657	107.4386455
																			LTSLVPFVDAFQLER	2	4503509	1xTMT6plex [N-Term]				1.629746675	1.61	0	1964.105429	2	982.5548706	-1.509419514	-0.00148233	108.4038155
																			KQPALDVLYDVMK	4	4503509	3xTMT6plex [N-Term; K1; K13]	100			1.858846903	13.18	1	2207.307673	3	736.4408569	0.156204224	0.00011493	85.55635229
																			LEEIPLIK	2	4503509	2xTMT6plex [N-Term; K8]	2.782			2.194434404	23.21	0	1412.912874	2	706.9602661	0.270219466	0.000190898	86.9022627
																			LESLNIQR	6	4503509	1xTMT6plex [N-Term]	1.243	0.763	1.019	2.573858023	12.39	0	1201.710202	2	601.3590088	0.448301199	0.000269364	54.81573562
																			RAEEQMLK	2	4503509	2xTMT6plex [N-Term; K8]			1.07	4.128154755	12.73	1	1462.845205	3	488.2861938	-0.804348285	-0.000392212	37.26576918
																			ITTMQLER	4	4503509	1xTMT6plex [N-Term]	0.938	1.408		2.559224844	29.87	0	1220.687024	2	610.8466187	-0.871046625	-0.000531638	56.51446031
																			ELEIEER	9	4503509	1xTMT6plex [N-Term]	1.1	1.047	0.468	3.100993633	40	0	1146.620384	2	573.8137207	-0.191322039	-0.000109687	32.58812028
																			EESWGPPR	2	4503509	1xTMT6plex [N-Term]			0.992	2.560510397	25.2	0	1186.605403	2	593.8065186	0.301405484	0.000178825	41.02445025
																			LLLTPWVK	6	4503509	2xTMT6plex [N-Term; K8]	100	100	100	1.996960163	21.9	0	1427.939029	2	714.4728394	-0.439058706	-0.000313475	89.05801056
																			AEEQMLK	2	4503509	2xTMT6plex [N-Term; K7]	0.996			3.134817362	29.25	0	1306.744094	2	653.8754272	-0.394949413	-0.000258049	45.22443665
																			EKEELEQR	2	4503509	2xTMT6plex [N-Term; K2]	0.729			2.188453913	8.97	1	1518.852793	3	506.9558716	0.177019192	8.96E-05	32.63240051
																			EGLYQYK	4	4503509	2xTMT6plex [N-Term; K7]	1.824	1.84		3.302810431	33.32	0	1358.772023	2	679.8890381	-0.900605918	-0.000611859	56.21258015
																			QSVYEEK	2	4503509	2xTMT6plex [N-Term; K7]	1.18			2.809533834	38.73	0	1340.746202	2	670.8762817	-0.682868567	-0.000457777	37.47991198
																			QVEQLEK	6	4503509	2xTMT6plex [N-Term; K7]	1.265	1.385	1.225	3.426035166	37.38	0	1331.793487	2	666.3997803	-0.903318072	-0.000601517	45.90996909
																			LLDMDGIIVEK	6	4503509	2xTMT6plex [N-Term; K11]				1.673314333	8.64	0	1704.001765	2	852.5039063	-0.721325544	-0.00061457	79.62082427
																			VSTVFWK	2	4503509	2xTMT6plex [N-Term; K7]	1.05			2.134961367	22.37	0	1324.80293	2	662.9059448	1.270753861	0.000841749	70.73002582
																			KIDYFER	6	4503509	2xTMT6plex [N-Term; K1]	1.588	1.308	1.28	3.458234787	10.06	1	1428.825121	3	476.9469299	0.780427853	0.000371698	43.97544319
																			YLELCVDLR	2	4503509	1xTMT6plex [N-Term]; 1xCarbamidomethyl [C5				1.504554987	1.75	0	1409.766003	2	705.3859863	-0.926936631	-0.000653382	82.22186907
																			FCLQYTR	1	4503509	1xTMT6plex [N-Term]; 1xCarbamidomethyl [C2		1.257		2.080864906	14.73	0	1216.634595	2	608.8204956	-0.723503233	-0.00044012	59.82001322
																			EEVSSWR	2	4503509	1xTMT6plex [N-Term]	1.237	1.059		2.476496935	27.99	0	1121.578854	2	561.293335	0.481064593	0.000269776	43.8988075
																			SLEDVVR	4	4503509	1xTMT6plex [N-Term]	1.108	1.206		2.383434057	28.81	0	1046.60434	2	523.805603	-0.392512525	-0.000205403	49.86768563
114155142	TPR	nucleoprotein TPR	7175	NP_003283.2	12.314854	1	25	26	106	2363	267.131	5.02	0.99	1.08	1.01	1.03	Significant	0.00071																
																			AIQDHLLEVEQSK	4	114155142	2xTMT6plex [N-Term; K13]	0.987			4.266284466	34.1	0	1968.116612	3	656.7110596	1.023202043	0.00067126	58.86585233
																			QLDTETNLHLNTK	6	114155142	2xTMT6plex [N-Term; K13]	0.876	1.176	1.274	4.753304482	45.96	0	1985.106775	3	662.3737793	0.004730539	3.13E-06	44.90729154

Bhat et al,2017. Cigarette smoke and chewing tobacco alter expression of different sets of miRNAs in oral keratinocytes

Supplementary Table S2(A). List of all proteins and peptides identified in OKF6/TERT1 cells chronically treated with chewing tobacco using Sequest and Mascot search algorithms

Accession	Gene_Symbol	Description	Gene_ID	Refseq_Accession	Coverage	Proteins	#Unique_Peptides	#Peptides	#PSMS	#AAs	MW [kDa]	calc. pI	OKF6-STE-6M/OKF6P_R1	OKF6-STE-6M/OKF6P_R2	OKF6-STE-6M/OKF6P_R3	Median fold change	Status	P-value (6M)	Sequence	#PSMs	Protein Accessions	Modifications	OKF6-STE-6M/OKF6P_R1	OKF6-STE-6M/OKF6P_R2	OKF6-STE-6M/OKF6P_R3	Xcorr	Ionscore	# Missed Cleavages	Theo. MH+ [Da]	Charge	m_z	DeltaM [ppm]	Deltam/z [Da]	RT [min]
																			GQPSNKEDVDDLVSQLR	4	114155142	2xTMT6plex [N-Term; K6]	0.785	1.185		3.822681665	24.89	1	2358.266523	3	786.7602539	-0.133433285	-0.00010489	67.85036166
																			AADSQNSGEGNTGAAESSFSQEVSR	8	114155142	1xTMT6plex [N-Term]	1.586	1.679		6.353588104	94.78	0	2715.229138	3	905.7468262	-1.183301376	-0.001070978	42.48097091
																			QHLSNMEVQVASQSSQR	2	114155142	1xTMT6plex [N-Term]	1.27			6.005144119	45.76	0	2158.08723	3	720.0348511	1.283712085	0.000923454	44.73430828
																			AESQLLECK	2	114155142	2xTMT6plex [N-Term; K9]; 1xCarbamidomethyl [C8			1.482	3.539305925	57.95	0	1535.85035	2	768.428894	0.105046652	8.07E-05	52.01055227
																			LEHEISHLK	4	114155142	2xTMT6plex [N-Term; K9]	0.88	1.025		4.180178642	40.24	0	1563.925898	3	521.9804688	0.610617166	0.00031832	41.7611702
																			TMAQHEELMK	4	114155142	2xTMT6plex [N-Term; K10]	0.877	1.026		3.93915844	31.19	0	1675.891169	3	559.3016968	-0.376920638	-0.000210559	37.79496241
																			ASTALSNEQQAR	2	114155142	1xTMT6plex [N-Term]		0.447		3.077200413	41.11	0	1504.7917	2	752.8999023	0.550406744	0.000414124	25.53200511
																			EQQASMEEK	6	114155142	2xTMT6plex [N-Term; K9]	1.98	1.379	1.237	3.8091681	57.09	0	1537.793229	2	769.4001465	-0.138179338	-0.000106246	29.96895703
																			AVASLSVK	6	114155142	2xTMT6plex [N-Term; K8]	0.985	0.954	0.987	3.986538887	49.85	0	1232.797844	2	616.9019775	-0.945549897	-0.000582836	51.28595298
																			KLELDILPLQEANAELSEK	6	114155142	3xTMT6plex [N-Term; K1; K19]	100	100	100	2.14036417	9.13	1	2840.658786	3	947.5575562	-0.236234979	-0.000223688	91.64035449
																			AIESMEQQLSELK	4	114155142	2xTMT6plex [N-Term; K13]				1.529014349	7.86	0	1964.077449	2	982.5406494	-1.744723395	-0.001713386	86.41851377
																			IAVEAQNK	4	114155142	2xTMT6plex [N-Term; K8]	1.261	1.672		3.575229883	40.35	0	1330.809471	2	665.9082642	-0.165079745	-0.000109845	42.91739687
																			ELENANDLLSATK	4	114155142	2xTMT6plex [N-Term; K13]		100		2.223407269	17.22	0	1876.042778	2	938.5259399	0.972911084	0.000912611	58.82825933
																			ELQELQDSLNAER	6	114155142	1xTMT6plex [N-Term]	100		100	1.759985805	22.18	0	1773.918022	2	887.4611206	-1.723754437	-0.0015289	60.3704135
																			IQNEQLEK	2	114155142	2xTMT6plex [N-Term; K8]	1.272			3.704680204	29.12	0	1459.852064	2	730.4303589	0.943077365	0.000688377	42.55053914
																			LLHDQIEK	2	114155142	2xTMT6plex [N-Term; K8]			0.936	3.569490671	30.19	0	1453.877885	3	485.2975464	0.138010323	6.69E-05	46.56036771
																			VLLMELEEAR	8	114155142	1xTMT6plex [N-Term]	1.07		100	2.328056574	22.46	0	1431.807867	2	716.40448	-4.318965696	-0.003091965	78.87590331
																			QVTEEVR	4	114155142	1xTMT6plex [N-Term]		1.276		2.639690161	36.15	0	1089.610154	2	545.309082	0.673243084	0.000366786	26.83079062
																			LIDIQEK	10	114155142	2xTMT6plex [N-Term; K7]	1.253			3.162941694	42.15	0	1316.818973	2	658.913147	0.033319168	2.19E-05	57.69688291
																			LLEEDVK	2	114155142	2xTMT6plex [N-Term; K7]	1.082			2.781343222	31.06	0	1303.787339	2	652.3967285	-0.888602547	-0.000579274	53.4228006
																			LDELQASDVSVK	2	114155142	2xTMT6plex [N-Term; K12]				1.528346419	7.91	0	1761.999851	2	881.5028687	-0.788862333	-0.000694988	47.56926322
																			SQEQILEILR	3	114155142	1xTMT6plex [N-Term]				1.751785159	7.95	0	1457.852509	2	729.4291382	-1.035415313	-0.000754741	65.68745451
																			EAGEANK	2	114155142	2xTMT6plex [N-Term; K7]				1.780127406	22.38	0	1176.662473	2	588.8349609	0.146520352	8.62E-05	24.86013568
																			TQYEELK	4	114155142	2xTMT6plex [N-Term; K7]	1.132	0.989		2.314740896	10.73	0	1368.777503	2	684.8925781	0.275501314	0.00018855	49.47767387
6274552	STAT1	signal transducer and activator of transcription 1-alpha/beta isoform alpha	6772	NP_009330.1	36.53333333	1	24	24	116	750	87.28	6.05	0.35	0.33	0.4	0.36	Significant	0.0033																
																			TELISVSEVHPSR	4	6274552	1xTMT6plex [N-Term]	0.383	0.392		4.841854572	45.09	0	1682.927465	3	561.6478271	0.869458336	0.000487745	33.84287342
																			ELSAVTFPDIIR	6	6274552	1xTMT6plex [N-Term]	0.306	0.306		4.49499464	55.2	0	1589.910024	2	795.4584961	-0.19403771	-0.000154251	88.59643858
																			FLEQVHQLYDDSFPMEIR	6	6274552	1xTMT6plex [N-Term]	0.01	0.442		4.600038528	38.96	0	2496.243061	3	832.7520142	-0.629842324	-0.00052408	100.7518516
																			WAQLSEVLSWQFSSVTK	2	6274552	2xTMT6plex [N-Term; K17]		0.425		3.761775494	33.74	0	2454.343317	3	818.7866821	0.886655385	0.000725386	107.5714001
																			EAPEPMELDGPK	14	6274552	2xTMT6plex [N-Term; K12]		0.313	0.336	3.860101223	54.96	0	1770.934808	2	885.9723511	1.478269232	0.001308959	51.31768309
																			KELSAVTFPDIIR	4	6274552	2xTMT6plex [N-Term; K1]	100	0.01		3.214428902	28.22	1	1947.167919	3	649.7271118	-0.583838081	-0.000378944	79.58629898
																			VMAAENIPENPLK	4	6274552	2xTMT6plex [N-Term; K13]	0.209	0.234		2.879766941	31.35	0	1884.06649	2	942.5360718	-0.861713385	-0.000811763	63.49841535
																			VMCIEHEIK	8	6274552	2xTMT6plex [N-Term; K9]; 1xCarbamidomethyl [C3	0.363	0.33		4.375463486	44.37	0	1616.890441	3	539.6350098	0.021774435	1.17E-05	55.65157653
																			DQQPGTFLLR	3	6274552	1xTMT6plex [N-Term]	0.432		0.352	3.594106913	38.33	0	1403.78443	2	702.3989258	4.377604118	0.003072606	55.29617456
																			SDQKQEQLLLK	6	6274552	3xTMT6plex [N-Term; K4; K11]	0.205	0.696	0.193	3.597074509	14.2	1	2017.226052	3	673.0795898	-0.909842875	-0.000611786	49.61294652
																			YTYEHDPITK	6	6274552	2xTMT6plex [N-Term; K10]	0.366		0.511	3.124391079	33.46	0	1724.925958	3	575.6468506	0.023756273	1.37E-05	42.76770594
																			FNILGTHTK	6	6274552	2xTMT6plex [N-Term; K9]	0.353	0.257		3.49839139	33.8	0	1488.89387	3	496.9683838	-2.197317673	-0.001090524	53.88650535
																			FHDLLSQLDDQYSR	6	6274552	1xTMT6plex [N-Term]	0.218	0.01		2.423640013	23.94	0	1965.986771	3	656.0005493	0.164837945	0.000108023	85.70502516
																			GLNVDQLNMLGEK	4	6274552	2xTMT6plex [N-Term; K13]				2.362502813	25.16	0	1889.056654	2	945.0299683	-2.114349802	-0.001997063	67.7928199
																			YLYPNIDKDHAFGK	4	6274552	3xTMT6plex [N-Term; K8; K14]	0.306		0.255	3.919054985	15.88	1	2368.326828	4	592.8359375	-2.072395558	-0.001227027	54.46964237
																			SLEDLQDEYDFK	5	6274552	2xTMT6plex [N-Term; K12]				1.636937499	12.63	0	1959.995159	2	980.4993286	-1.92781774	-0.001889257	68.55074046
																			FSLENNFLLQHNIR	2	6274552	1xTMT6plex [N-Term]	100			2.793604374	14.73	0	1974.07586	3	658.6982422	2.184740003	0.001437614	76.12590168
																			QDWEHAANDVSFATIR	4	6274552	1xTMT6plex [N-Term]	0.638			2.257273436	0.87	0	2089.030032	3	697.0152588	0.569995246	0.000396912	67.39507982
																			KLEELEQK	8	6274552	3xTMT6plex [N-Term; K1; K8]	0.35	0.359		3.527119398	27.87	1	1704.051048	3	568.6881104	-0.745133377	-0.000423248	53.64764452
																			LEELEQK	2	6274552	2xTMT6plex [N-Term; K7]				2.738279581	29.07	0	1346.793153	2	673.9000854	-0.191797502	-0.000129156	57.43381277
																			QEQLLLK	2	6274552	2xTMT6plex [N-Term; K7]			0.448	3.018395662	37.87	0	1329.850608	2	665.4301147	1.763304971	0.001172466	56.90597068
																			LLGPNASPDGLIPWTR	5	6274552	1xTMT6plex [N-Term]	0.193	0.269	0.01	2.250935793	25.59	0	1936.085363	2	968.5446167	-1.759091633	-0.001702876	80.89369315
																			MYLMLDNK	4	6274552	2xTMT6plex [N-Term; K8]				1.685634494	11.9	0	1485.820964	2	743.4138794	-0.324326382	-0.000240945	63.62169274
																			IIELLNVTELTQNALINDELVEWK	1	6274552	2xTMT6plex [N-Term; K24]				1.668917775		0	3268.844465	3	1090.286499	0.146333152	0.000159447	117.0376687
4503471	EEF1A1	elongation factor 1-alpha 1	1915	NP_001393.1	65.8008658	1	10	20	269	462	50.109	9.01	1.73	1.69	1.87	1.77	Significant	0.00096																
																			YYVTIIDAPGHR	16	4503471	1xTMT6plex [N-Term]	2.62	2.826	2.728	4.555756092	48.28	0	1633.889957	2	817.444519	-5.016178737	-0.004097942	50.00138699
																			IGGIGTVPVGR	8	4503471	1xTMT6plex [N-Term]	2.066	3.079	2.241	3.727612734	45.11	0	1254.773137	2	627.890625	0.666686252	0.00041827	47.04865289
																			VETGVLKPGMVVTFAPVNVTTEVK	36	4503471	3xTMT6plex [N-Term; K7; K24]	1.843	5.524	1.766	5.689495564	42.64	0	3202.872818	3	1068.296631	0.787061575	0.000840286	77.01031044
																			VETGVLKPGMVVTFAPVNVTTEVK	3	4503471	3xTMT6plex [N-Term; K7; K24]; 1xOxidation [M10			100	2.56763196	15.48	0	3218.867733	3	1068.296631	0.787061575	0.000840286	77.01031044
																			NDPPMEAAGFTAQVIILNHPGQISAGYAPVLDCHTAHIACK	2	4503471	2xTMT6plex [N-Term; K41]; 2xCarbamidomethyl [C33; C4(				2.849413633	10.15	0	4843.452255	5	969.4975586	1.327846009	0.001286272	90.94880222
																			KDGNASGTTLLEALDCILPPTRPTDKPLR	9	4503471	3xTMT6plex [N-Term; K1; K26]; 1xCarbamidomethyl [C16	2.372	1.917	2.001	5.689491272	36.91	1	3837.151119	5	768.2354126	-0.824059731	-0.000632408	78.89629053
																			THINIVVIGHVDSGK	8	4503471	2xTMT6plex [N-Term; K15]	1.895	1.803	2.234	5.019645214	55.13	0	2047.20643	4	512.5568237	-0.471231484	-0.000241177	54.78889256
																			STTTGHLIYK	30	4503471	2xTMT6plex [N-Term; K10]	1.44	1.313	1.47	4.323945522	54.41	0	1578.925564	3	526.9812622	2.324176122	0.001223234	35.32994542
																			DGNASGTTLLEALDCILPPTRPTDKPLR	2	4503471	2xTMT6plex [N-Term; K25]; 1xCarbamidomethyl [C15		100		2.65169549	16.99	0	3479.893224	4	870.7272949	-1.687911704	-0.001468438	88.14988808
																			EHALLAYTLGVK	16	4503471	2xTMT6plex [N-Term; K12]	1.935	2.128	100	4.556538582	46.59	0	1773.067477	2	887.0368652	-0.576944343	-0.000511481	71.88132891
																			SGDAAIVDMVPGKPMCVESFSDYPPLGR	10	4503471	2xTMT6plex [N-Term; K13]; 1xCarbamidomethyl [C16	100			2.16425848	16.79	0	3453.725689	3	1151.911743	-1.451504336	-0.001671033	80.99147224
																			QTVAVGVIK	21	4503471	2xTMT6plex [N-Term; K9]	1.781	1.723	1.642	4.514291286	62.58	0	1372.892807	2	686.9498291	-0.309934521	-0.000212753	45.46677378
																			GNVAGDSK	14	4503471	2xTMT6plex [N-Term; K8]	1.834	1.912	1.865	2.926821232	46.32	0	1205.689022	2	603.3480225	-0.210367782	-0.000126819	24.28233365
																			LPLQDVYK	25	4503471	2xTMT6plex [N-Term; K8]	1.58	1.565	0.969	3.475753546	46.3	0	1433.876823	2	717.4418335	-0.301481833	-0.000216144	68.8466914
																			QLIVGVNK	36	4503471	2xTMT6plex [N-Term; K8]	2.178	2.067	2.057	3.624680966	43.22	0	1328.866592	2	664.9334717	-5.211682395	-0.003462815	47.65431494
																			NMITGTSQADCAVLIVAAGVGEFEAGISK	3	4503471	2xTMT6plex [N-Term; K29]; 1xCarbamidomethyl [C11				2.277580976	6.88	0	3367.764183	3	1123.258667	-0.812281766	-0.000911858	117.7281867
																			IGYNPDTVAFVPISGWNGDNMLEPSANMPWFK	3	4503471	2xTMT6plex [N-Term; K32]				2.05193305	7.91	0	4025.997036	3	1342.670898	0.274658051	0.000368591	110.3575382
																			RYEEIVK	2	4503471	2xTMT6plex [N-Term; K7]		1.859		3.938460827	23.79	1	1394.840772	3	465.618927	1.044023009	0.000485415	35.09308987
																			YAWVLDK	4	4503471	2xTMT6plex [N-Term; K7]	6.123	100		2.795761347	29.13	0	1352.797844	2	676.9017334	-1.222616618	-0.000826977	64.29157323
																			EAAEMGK	6	4503471	2xTMT6plex [N-Term; K7]	1.483	1.847	1.914	3.196498394	31.97	0	1193.66003	2	597.3337402	0.145609927	8.69E-05	38.66839001
																			EVSTYIK	10	4503471	2xTMT6plex [N-Term; K7]	1.89	1.792	100	2.653904915	37.67	0	1297.776774	2	649.392334	0.475466014	0.000308524	51.7386891
																			EAAEMGK	5	4503471	2xTMT6plex [N-Term; K7]; 1xOxidation [M5]	1.787	1.801	1.984	1.968911648	16.57	0	1209.654945	2	597.3337402	0.145609927	8.69E-05	38.66839001
4757810	ATP5A1	ATP synthase subunit alpha, mitochondrial isoform a precursor	498	NP_004037.1	45.38878843	1	21	21	208	553	59.714	9.13	0.71	0.78	0.8	0.76	Significant	0.0013																
																			GIRPAINVGLSVSR	22	4757810	1xTMT6plex [N-Term]	0.684	0.707	1.063	5.697936535	53.8	0	1668.011804	3	556.675354	-0.176862226	-9.83E-05	49.82674987
																			GMSLNLEPDNVGVVVFGNDK	9	4757810	2xTMT6plex [N-Term; K20]	1.504	0.938		3.248134613	36.05	0	2562.363794	3	854.7924805	-0.353643676	-0.000302055	96.6857098
																			ILGADTSVDLEETGR	8	4757810	1xTMT6plex [N-Term]	0.765	0.794	0.878	4.754341602	80.6	0	1804.948988	2	902.9780884	-0.048756052	-4.40E-05	66.59108207
																			FENAFLSHVVSQHQALLGTIR	6	4757810	1xTMT6plex [N-Term]		100		5.290014744	34.66	0	2596.419721	4	649.8594971	-1.371984938	-0.000890562	88.79695463
																			VVDALGNAIDGK	11	4757810	2xTMT6plex [N-Term; K12]	0.829	0.767	100	3.875210047	70.78	0	1629.957592	2	815.4824219	-0.015270336	-1.24E-05	76.06610628
																			EVAAFAQFGSDLDAATQQLLSR	30	4757810	1xTMT6plex [N-Term]	0.671	0.807	0.931	4.23454237	57.03	0	2567.330296	3	856.4454956	-3.25742021	-0.002787625	106.2084613
																			EAYPGDVFYLHSR	23	4757810	1xTMT6plex [N-Term]	0.799	0.802	0.582	4.0788517	31.47	0	1782.90125	3	594.9724731	0.906356195	0.000538648	55.87806374
																			VLSIGDGIAR	10	4757810	1xTMT6plex [N-Term]	0.684	0.71	0.843	3.539447308	57.74	0	1229.741502	2	615.3739624	-0.694589319	-0.000427083	64.54409388
																			TSIAIDTIINQK	8	4757810	2xTMT6plex [N-Term; K12]	0.593	0.652	1.043	3.302469969	36.38	0	1775.067871	2	888.0366211	-1.073256145	-0.000952551	67.40351912
																			HALIIYDDLSK	6	4757810	2xTMT6plex [N-Term; K11]	0.472	0.634	0.717	3.520197153	44.01	0	1746.020192	2	873.5140991	0.417728381	0.000364681	61.69246805
																			KLYCIYVAIGQK	2	4757810	3xTMT6plex [N-Term; K1; K12]; 1xCarbamidomethyl [C4	0.666			2.749668837	14.85	1	2143.291629	3	715.102356	0.413136076	0.000295157	76.94317792
																			AVDSLVPIGR	16	4757810	1xTMT6plex [N-Term]	0.614	0.643	1.124	3.146650791	36.93	0	1255.757152	2	628.3816528	-0.894572597	-0.000561683	64.83319983
																			TGAIVDVPVGEELLGR	10	4757810	1xTMT6plex [N-Term]	100			2.651781082	34.66	0	1854.053394	2	927.5299072	-0.461603144	-0.000427918	84.47463746
																			STVAQLVK	4	4757810	2xTMT6plex [N-Term; K8]	0.657	0.724		3.235249996	46.27	0	1303.834958	2	652.4213867	0.413347945	0.000269469	54.43251281
																			GYLDKLEPSK	8	4757810	3xTMT6plex [N-Term; K5; K10]	0.352	0.387	0.351	2.928498268	18.31	1	1837.103811	3	613.0402832	1.352672266	0.000828333	51.17932496
																			LTDADAMK	6	4757810	2xTMT6plex [N-Term; K8]	0.824	0.988	0.904	3.518983603	51.57	0	1322.739009	2	661.8726807	-0.69844608	-0.000461931	40.92430789
																			EPMQTGIK	3	4757810	2xTMT6plex [N-Term; K8]			0.582	3.700426102	34.26	0	1361.786293	2	681.3966064	-0.262045063	-0.000178425	34.85337135
																			ISEQSDAK	8	4757810	2xTMT6plex [N-Term; K8]	0.834	0.904		2.782817364	41.33	0	1335.752016	2	668.3798218	0.262694485	0.000175447	35.13055543
																			LTDADAMK	2	4757810	2xTMT6plex [N-Term; K8]; 1xOxidation [M7]			1.006	2.204243898	32.53	0	1338.733924	2	661.8726807	-0.69844608	-0.000461931	40.92430789
																			QVAGTMK	4	4757810	2xTMT6plex [N-Term; K7]	0.778		0.903	2.488307953	23.24	0	1192.7124	2	596.8606567	1.372389993	0.000818433	23.13480231
																			ELIIGDR	5	4757810	1xTMT6plex [N-Term]	0.717	0.744	0.666	2.660664558	28.78	0	1044.625076	2	522.8162842	0.206810443	0.00010802	43.16356192
																			QMSLLLR	4	4757810	1xTMT6plex [N-Term]		0.741	0.818	1.994622827	32.43	0	1089.665166	2	545.3364868	0.486941153	0.000265301	62.08608152
																			EPMQTGIK	3	4757810	2xTMT6plex [N-Term; K8]; 1xOxidation [M3]	0.658		0.654	1.726588011	20.84	0	1377.781208	2	681.3966064	-0.262045063	-0.000178425	34.85337135
157649073	CAP1	adenylyl cyclase-associated protein 1	10487	NP_001099000.1	58.31578947	1	20	20	232	475	51.641	8.02	1.02	1.05	1.07	1.05	Significant	0.00019																
																			VENQENVSNLVIEDTELK	8	157649073	2xTMT6plex [N-Term; K18]	2.535			4.641731739	46.7	0	2531.360483	3	844.4567871	-1.846798157	-0.001558304	75.86786105
																			EMNDAAMFYTNR	6	157649073	1xTMT6plex [N-Term]	1.332	0.732		4.20310688	47.5	0	1691.771892	2	846.3891602	-0.501650077	-0.000424339	64.48722508
																			EFHTTGLAWSK	6	157649073	2xTMT6plex [N-Term; K11]	1.26	1.583	1.299	4.635063171	41.63	0	1734.957926	3	578.9905396	-0.496258522	-0.000286996	50.56420494
																			ALLVTASQCQQPAENK	74	157649073	2xTMT6plex [N-Term; K16]; 1xCarbamidomethyl [C9	0.953	1.036	0.998	4.870433807	61.17	0	2216.210923	3	739.4071655	-1.795694592	-0.001326546	39.91605255
																			SALFAQINQGESITHALK	17	157649073	2xTMT6plex [N-Term; K18]	0.916	0.876	0.976	4.331999302	44.67	0	2386.349465	3	796.1207886	-0.69248338	-0.000550836	75.44790477
																			KEPAVLELEGK	10	157649073	3xTMT6plex [N-Term; K1; K11]	1.014	1.061	0.808	5.245721817	52.43	1	1900.172225	3	634.062439	0.283350385	0.000179472	64.44713433
																			NSLDCEIVSAK	49	157649073	2xTMT6plex [N-Term; K11]; 1xCarbamidomethyl [C5		1.311	1.186	3.711802006	49.77	0	1693.919492	2	847.4638062	0.4979308	0.000421727	37.25896342
																			EMNDAAMFYTNR	4	157649073	1xTMT6plex [N-Term]; 1xOxidation [M7]	1.109	1.287		3.141255856	43.33	0	1707.766807	2	846.3891602	-0.501650077	-0.000424339	64.48722508
																			CVNTTLQIK	2	157649073	2xTMT6plex [N-Term; K9]; 1xCarbamidomethyl [C1			1.268	4.018337727	44.75	0	1534.90272	2	767.954895	-0.134641065	-0.00010333	46.95019363
																			LSDLLAPISEQIK	8	157649073	2xTMT6plex [N-Term; K13]	100	0.791	100	2.525902033	30.33	0	1885.141036	2	943.0735474	-0.645820877	-0.000608732	88.2308399
																			AGAAPYVQAFDSLLAGPVAEYLK	5	157649073	2xTMT6plex [N-Term; K23]		1.765	100	2.725824356	17.01	0	2809.554038	3	937.1903687	0.895108974	0.000838286	116.4693024
																			EPAVLELEGK	4	157649073	2xTMT6plex [N-Term; K10]	0.916	0.939		3.527584314	34.63	0	1542.91433	2	771.9596558	-1.487682444	-0.001147683	60.96890763
																			SSEMNVLIPTEGGDFNEFPVPEQFK	6	157649073	2xTMT6plex [N-Term; K25]	0.464	0.621	100	2.545506954	21.22	0	3269.644051	3	1090.551147	-1.578770382	-0.001720672	96.86128573
																			TDGCHAYLSK	4	157649073	2xTMT6plex [N-Term; K10]; 1xCarbamidomethyl [C4	0.957	0.953		3.685715675	28.16	0	1609.840848	3	537.2854614	0.61059287	0.000327652	35.08601107
																			VPTISINK	8	157649073	2xTMT6plex [N-Term; K8]	1.108	0.998	0.888	3.226134777	50.55	0	1329.850608	2	665.4285889	-0.53150754	-0.000353413	54.04089413
																			LEAVSHTSDMHR	2	157649073	1xTMT6plex [N-Term]			1.037	4.106682301	32.1	0	1611.811055	3	537.9418945	0.046701349	2.51E-05	34.31304961
																			ELSGLPSGPSAGSGPPPPPPGPPPPPVSTSSGSDESASR	2	157649073	1xTMT6plex [N-Term]	100			2.858216286	1.18	0	3819.887456	4	955.7278442	0.547406998	0.000522758	58.37024027
																			LEAVSHTSDMHR	4	157649073	1xTMT6plex [N-Term]; 1xOxidation [M10]	1.722	2.021		2.806159258	26.7	0	1627.80597	3	537.9418945	0.046701349	2.51E-05	34.31304961
																			GYADSPSK	6	157649073	2xTMT6plex [N-Term; K8]	0.819	1.017	0.795	3.14135766	39.96	0	1282.704338	2	641.8563843	0.899899263	0.000577152	32.84107345
																			QVAYIYK	2	157649073	2xTMT6plex [N-Term; K7]		0.834		3.1623106	29.95	0	1342.813494	2	671.9095459	-1.250369565	-0.000839507	50.66465366
																			EIGGDVQK	4	157649073	2xTMT6plex [N-Term; K8]	1.023	1.031		3.246576548	38.18	0	1303.762187	2	652.3845215	-0.322521434	-0.000210246	40.09884571
																			LGLVFDDVVGIVEIINSK	1	157649073	2xTMT6plex [N-Term; K18]				1.656604648		0	2388.415419	2	1194.710327	-0.854793981	-0.001020802	113.498065
409971425	BASP1	brain acid soluble protein 1	10409	NP_001258535.1	81.49779736	1	17	17	89	227	22.68	4.63	0.7	0.57	0.69	0.66	Significant	0.0041																
																			AQGPAASAEEPKPVEAPAANSDQTVTVKE	8	409971425	3xTMT6plex [N-Term; K12; K28]	0.69	0.712		6.28610754	46.53	1	3579.910931	4	895.7318726	-1.472222557	-0.001317606	44.07605649
																			AEGAATEEEGTPK	8	409971425	2xTMT6plex [N-Term; K13]	0.728	0.777	0.752	4.156097412	79.49	0	1747.91143	2	874.458313	-1.190108035	-0.001040102	30.0971272
																			KAEGAATEEEGTPK	3	409971425	3xTMT6plex [N-Term; K1; K14]		0.876	1.02	6.152764797	33.99	1	2105.169325	3	702.3944702	-0.221894488	-0.000155708	38.79988406
																			ETPAATEAPSSTPK	6	409971425	2xTMT6plex [N-Term; K14]	0.721			4.037713051	71.65	0	1845.000579	2	923.0050659	1.233780609	0.001138163	34.78701762
																			ESEPQAAAEPAEAK	10	409971425	2xTMT6plex [N-Term; K14]	0.793	0.82	0.85	4.330875397	57.96	0	1885.990742	2	943.4970703	-2.056454951	-0.001939227	29.9012896
																			KTEAPAAPAAQETK	2	409971425	3xTMT6plex [N-Term; K1; K14]		1.039		6.325417519	28.7	1	2100.22678	3	700.7467651	-0.494094187	-0.000345903	31.41758868
																			DKKAEGAATEEEGTPK	2	409971425	4xTMT6plex [N-Term; K2; K3; K16]		0.066		4.597081184	10.27	2	2577.454162	4	645.1201172	1.736768035	0.00111911	31.09893821
																			AQGPAASAEEPKPVEAPAANSDQTVTVK	2	409971425	3xTMT6plex [N-Term; K12; K28]		1.59		4.906199932	53.17	0	3450.868338	3	1150.96106	0.083367695	9.59E-05	42.92916791
																			AAEAAAAPAESAAPAAGEEPSKEEGEPK	16	409971425	3xTMT6plex [N-Term; K22; K28]	0.651	0.634	0.591	5.835977554	65.23	1	3323.721005	3	1108.578369	-0.13559746	-0.000150229	38.65966498
																			AEPPKAPEQEQAAPGPAAGGEAPK	6	409971425	3xTMT6plex [N-Term; K5; K24]	0.426	0.625	0.549	5.834096432	53.39	1	2985.62486	3	995.8799438	0.139995139	0.000139324	37.01723213
																			AEPEKTEGAAEAK	4	409971425	3xTMT6plex [N-Term; K5; K13]	1.403	0.666		3.147557735	15.53	1	2018.137296	3	673.383667	-0.420409176	-0.000282814	31.01388482
																			EGEKDAAAAK	2	409971425	3xTMT6plex [N-Term; K4; K10]	0.473			2.038497925	8.95	1	1676.978611	3	559.6640625	-0.58237475	-0.000325543	32.32419533
																			EKPDQDAEGK	8	409971425	3xTMT6plex [N-Term; K2; K10]	0.76	0.881		2.789931297	31.62	0	1804.005554	2	902.5072021	0.872417977	0.000786923	32.40469849
																			EKPDQDAEGKAEEK	4	409971425	4xTMT6plex [N-Term; K2; K10; K14]	0.392	0.382		3.668694735	7.21	1	2490.385749	4	623.3519287	0.054772137	3.41E-05	37.2511138
																			GYNVNDEK	5	409971425	2xTMT6plex [N-Term; K8]	0.79	0.75		3.506100416	38.63	0	1396.747265	2	698.87677	-0.717088168	-0.000500795	37.00301976
																			KGYNVNDEK	2	409971425	3xTMT6plex [N-Term; K1; K9]	0.751			2.698510408	20.1	1	1754.00516	3	585.3405762	1.1489679	0.000671765	34.5574983
																			AEEKEGEKDAAAAK	1	409971425	4xTMT6plex [N-Term; K4; K8; K14]			0.606	2.820107937		2	2363.358806	4	591.59552	0.611243676	0.000361147	34.97541537
188035924	ALDH7A1	alpha-aminoadipic semialdehyde dehydrogenase isoform 1 precursor	501	NP_001173.2	49.90723562	1	19	19	141	539	58.45	7.99	0.73	0.83	0.79	0.78	Significant	0.0014																
																			GEVITTYCPANNEPIAR	16	188035924	1xTMT6plex [N-Term]; 1xCarbamidomethyl [C8	0.658	0.651	0.659	4.751187325	44.61	0	2134.080019	3	712.0305786	-1.32925201	-0.000945577	49.18520823
																			GSDCGIVNVNIPTSGAEIGGAFGGEK	10	188035924	2xTMT6plex [N-Term; K26]; 1xCarbamidomethyl [C4	0.01	100	0.446	3.75222826	37.4	0	2964.513706	3	988.8417358	-1.029450619	-0.001017273	72.91012738
																			LFIHESIHDEVVNR	20	188035924	1xTMT6plex [N-Term]	0.8	0.831	0.848	5.523492336	55.93	0	1937.044226	4	485.0162048	-0.638356133	-0.000309131	83.72973555
																			GAPTTSLISVAVTK	4	188035924	2xTMT6plex [N-Term; K14]	0.916	1.279		4.415801048	71.65	0	1803.099171	2	902.0540161	0.87894032	0.000792408	74.55855457
																			QASVADYEETVK	4	188035924	2xTMT6plex [N-Term; K12]			0.829	4.955616474	56.69	0	1797.963465	2	899.4859619	0.657448356	0.000591034	43.70769197
																			ILVEGVGEVQEYVDICDYAVGLSR	6	188035924	1xTMT6plex [N-Term]; 1xCarbamidomethyl [C16	1.203			4.085343361	40.99	0	2912.49129	3	971.5	-2.006361365	-0.001947837	118.9351323
																			KEGGTVVYGGK	2	188035924	3xTMT6plex [N-Term; K1; K11]	0.769			3.953663349	26.55	1	1782.072846	3	594.6959229	0.207452063	0.000123232	35.78783275
																			MIGGPILPSER	4	188035924	1xTMT6plex [N-Term]	0.825		0.643	3.369573116	30.46	0	1398.797637	2	699.9017334	-1.034319109	-0.000723402	51.613328
																			EGGTVVYGGK	6	188035924	2xTMT6plex [N-Term; K10]	1.087	1.246	1.154	4.290941715	51.33	0	1424.814951	2	712.9073486	-5.284899889	-0.003765002	37.54602862
																			VMDRPGNYVEPTIVTGLGHDASIAHTETFAPILYVFK	2	188035924	2xTMT6plex [N-Term; K37]; 1xOxidation [M2			100	2.918342113	11.07	0	4533.389227	5	907.4851074	1.58895233	0.001440668	100.8827319
																			QAVSMFLGAVEEAK	4	188035924	2xTMT6plex [N-Term; K14]	0.744			3.169420242	31.85	0	1938.077055	2	969.5418091	-0.368187599	-0.000356788	97.35173327
																			VGNPWDPNVLYGPLHTK	5	188035924	2xTMT6plex [N-Term; K17]	0.715		100	2.846435547	20.2	0	2365.306872	3	789.1065063	-0.805889948	-0.000635392	76.70247289
																			DLPLAQGIK	8	188035924	2xTMT6plex [N-Term; K9]	0.684	0.97	0.624	4.234872341	54.7	0	1412.887722	2	706.9481201	0.878997217	0.000620962	58.80339047
																			STCTINYSK	10	188035924	2xTMT6plex [N-Term; K9]; 1xCarbamidomethyl [C3	0.907	0.848	1.124	4.220322609	59.7	0	1531.81905	2	766.4122925	-1.136993996	-0.000870835	43.61035097
																			IWADIPAPK	10	188035924	2xTMT6plex [N-Term; K9]	0.763	1.254	100	3.377739191	44.69	0	1468.892807	2	734.949646	-0.538989509	-0.000395859	73.85624154
																			VNLLSFTGSTQVGK	6	188035924	2xTMT6plex [N-Term; K14]	2.014	1.807	0.807	2.399260521	34.28	0	1909.115883	2	955.0635986	2.114693854	0.002018598	77.84963665
																			FKNEEEVFAWNNEVK	6	188035924	3xTMT6plex [N-Term; K2; K15]	0.875	1.355	0.398	2.922150135	24.17	1	2570.3858	3	857.4663086	-0.555169092	-0.000475666	62.8695645
																			QVGLMVQER	10	188035924	1xTMT6plex [N-Term]	0.75	0.8	0.65	3.375872612	37.01	0	1288.724472	2	644.866394	0.806523008	0.000519693	42.13212571
																			MIGGPILPSER	2	188035924	1xTMT6plex [N-Term]; 1xOxidation [M1]			0.741	2.660019398	24.17	0	1414.792552	2	699.9017334	-1.034319109	-0.000723402	51.613328
																			QVGLMVQER	4	188035924	1xTMT6plex [N-Term]; 1xOxidation [M5]	0.718	0.847		1.902990818	21.83	0	1304.719387	2	644.866394	0.806523008	0.000519693	42.13212571
																			KAYAQIR	2	188035924	2xTMT6plex [N-Term; K1]	0.493			3.174104691	9.87	1	1307.819977	3	436.6109619	-1.257105597	-0.000548023	36.77395093
77404397	SND1	staphylococcal nuclease domain-containing protein	27044	NP_055205.2	35.49450549	1	24	24	106	910	101.934	7.17	1.25	1.22	0.88	1.12	Significant	0.011																
																			DIQNTQCLLNVEHLSAGCPHVTLQFADSK	2	77404397	2xTMT6plex [N-Term; K29]; 2xCarbamidomethyl [C7; C18	2.256			4.761899948	43.7	0	3753.909285	4	939.2305298	-2.396245805	-0.002248822	81.11718604
																			NDIASHPPVEGSYAPR	4	77404397	1xTMT6plex [N-Term]	1.922	2.1		4.135462284	48.06	0	1938.987105	3	647.0001831	-0.571913857	-0.000369645	43.37234009
																			SSHYDELLAAEAR	14	77404397	1xTMT6plex [N-Term]	0.904	1.495	1.349	5.404278755	67.46	0	1690.859779	2	845.9318848	-1.943631749	-0.001643204	49.51760374
																			DYVAPTANLDQK	2	77404397	2xTMT6plex [N-Term; K12]			1.992	4.414721966	63.03	0	1792.984535	2	896.9958496	-0.062672738	-5.62E-05	53.25829927
																			SASYKPVFVTEITDDLHFYVQDVETGTQLEK	2	77404397	3xTMT6plex [N-Term; K5; K31]			100	4.003824711	19.81	0	4247.236681	4	1062.566284	1.559957508	0.001656377	109.3941929
																			HFVDSHHQKPVNAIIEHVR	4	77404397	2xTMT6plex [N-Term; K9]			0.527	3.237596989	7.34	0	2721.510156	5	545.1033936	-8.19222611	-0.004459045	40.73116773
																			SEAVVEYVFSGSR	6	77404397	1xTMT6plex [N-Term]	1.24		100	3.830422878	48.49	0	1658.858717	2	829.9319458	-1.266978565	-0.001050869	73.82076419
																			VWAHYEEQPVEEVMPVLEEK	3	77404397	2xTMT6plex [N-Term; K20]				3.218107224	20.35	0	2899.495202	3	967.1687012	-1.259537995	-0.001217341	85.0461062
																			VADISGDTQK	7	77404397	2xTMT6plex [N-Term; K10]	1.525	1.306	1.691	3.853584051	52.35	0	1491.841894	2	746.4261475	2.094526162	0.001562351	41.82479585
																			VMQVLNADAIVVK	6	77404397	2xTMT6plex [N-Term; K13]	2.106	100		2.249975204	24.58	0	1858.123611	2	929.5648193	-0.672392363	-0.000624694	81.41074734
																			DTPDEPWAFPAR	8	77404397	1xTMT6plex [N-Term]	2.182	2.081	100	3.310572147	26.95	0	1630.806287	2	815.9060669	-0.876934893	-0.000715055	70.90894224
																			GDVGLGLVK	4	77404397	2xTMT6plex [N-Term; K9]		1.34	1.856	3.423170567	63.87	0	1315.834958	2	658.4216309	0.780659264	0.000513609	57.09390851
																			AAATQPDAK	6	77404397	2xTMT6plex [N-Term; K9]	1.507	1.848	1.735	3.836512327	52.02	0	1330.773086	2	665.8896484	-0.800748836	-0.000532808	28.88645755
																			ADDADEFGYSR	2	77404397	1xTMT6plex [N-Term]	1.287			3.226437569	41.84	0	1474.664768	2	737.8363647	0.46442568	0.000342436	46.47024811
																			TDAVDSVVR	2	77404397	1xTMT6plex [N-Term]	1.919			3.581022978	50.2	0	1190.657832	2	595.8322754	-0.468748229	-0.000279059	38.58012166
																			VITEYLNAQESAK	2	77404397	2xTMT6plex [N-Term; K13]		100		1.597141743	21.26	0	1924.079164	2	962.5413818	-1.910788389	-0.001838254	61.200424
																			VLPAQATEYAFAFIQVPQDDDAR	7	77404397	1xTMT6plex [N-Term]	100		100	2.87271452	16.15	0	2794.424925	3	932.1464233	-0.07452713	-6.94E-05	100.4825949
																			EVCFTIENK	10	77404397	2xTMT6plex [N-Term; K9]; 1xCarbamidomethyl [C3	2.986	100	100	2.395965815	29.96	0	1597.866	2	799.4368896	0.314473726	0.000251243	55.47876464
																			EYGMIYLGK	6	77404397	2xTMT6plex [N-Term; K9]	1.492	3.202	100	2.704520702	30.45	0	1531.859458	2	766.4326172	-0.979375093	-0.000750132	61.30327806
																			LSECEEQAK	2	77404397	2xTMT6plex [N-Term; K9]; 1xCarbamidomethyl [C4		1.72		2.707339048	22.6	0	1551.808879	2	776.4077148	-0.467932946	-0.000363071	33.80400287
																			QINLSNIR	2	77404397	1xTMT6plex [N-Term]			1.791	2.970494986	52.1	0	1186.710537	2	593.8590088	0.172205537	0.000102179	52.63898692
																			QFLPFLQR	2	77404397	1xTMT6plex [N-Term]	0.777			2.189599752	23.26	0	1277.756759	2	639.3820801	0.097816935	6.25E-05	79.03616394
																			GLATVIR	2	77404397	1xTMT6plex [N-Term]		0.635		2.330231905	19.61	0	958.6246818	2	479.8157349	-0.509827722	-0.000244367	41.13838607
																			IHVFYIDYGNR	1	77404397	1xTMT6plex [N-Term]			3.745	1.270418406	2.82	0	1625.863743	3	542.6258545	-0.450470367	-0.000244134	56.77108122
4505753	PGAM1	phosphoglycerate mutase 1 isoform 1	5223	NP_002620.1	60.23622047	1	12	12	155	254	28.786	7.18	0.57	0.69	0.54	0.6	Significant	0.0058																
																			AMEAVAAQGK	8	4505753	2xTMT6plex [N-Term; K10]	0.831	0.93	0.85	4.747850895	76.95	0	1433.818656	2	717.4119873	-1.365479949	-0.000978925	41.93132997
																			YADLTEDQLPSCESLK	12	4505753	2xTMT6plex [N-Term; K16]; 1xCarbamidomethyl [C12	0.983	0.782	1.053	3.809581995	64.73	0	2327.184099	3	776.3991089	-0.569611078	-0.000441863	62.39532086
																			FSGWYDADLSPAGHEEAK	10	4505753	2xTMT6plex [N-Term; K18]	1.384	1.11	1.027	4.187107563	52.44	0	2438.20286	3	813.406311	0.623227662	0.000506518	71.43014741
																			NLKPIKPMQFLGDEETVR	6	4505753	3xTMT6plex [N-Term; K3; K6]	0.667	1.601	0.755	4.563177586	9.26	0	2802.615482	4	701.4072266	-2.999134221	-0.002101355	61.96465169
																			KAMEAVAAQGK	6	4505753	3xTMT6plex [N-Term; K1; K11]		0.643	0.54	5.826007843	46.03	1	1791.076551	3	603.0291748	0.833040691	0.000501788	21.24816771
																			HLEGLSEEAIMELNLPTGIPIVYELDK	7	4505753	2xTMT6plex [N-Term; K27]; 1xOxidation [M11	1.096	100		6.413607121	42.58	0	3497.885344	4	875.2286377	2.108871654	0.001844148	101.7428483
																			HLEGLSEEAIMELNLPTGIPIVYELDK	23	4505753	2xTMT6plex [N-Term; K27]	0.9	0.941	0.471	6.418806553	38.64	0	3481.890429	4	875.2286377	2.108871654	0.001844148	101.7428483
																			FSGWYDADLSPAGHEEAKR	4	4505753	2xTMT6plex [N-Term; K18]	0.471	0.618		5.075926781	43.17	1	2594.303971	4	649.3310547	-0.6099478	-0.000395597	65.92261845
																			VLIAAHGNSLR	16	4505753	1xTMT6plex [N-Term]	0.332	0.622	0.422	4.292860985	59.9	0	1379.832049	3	460.6155701	0.078491494	3.61E-05	37.98443105
																			SYDVPPPPMEPDHPFYSNISK	22	4505753	2xTMT6plex [N-Term; K21]	1.124	0.554	0.784	2.86752224	21.32	0	2875.437687	3	959.1513062	0.583478835	0.000559252	51.93530291
																			ALPFWNEEIVPQIK	19	4505753	2xTMT6plex [N-Term; K14]	0.752	0.703	0.735	3.072958708	28.49	0	2142.236333	2	1071.619751	-1.91742004	-0.002053783	93.43958161
																			AMEAVAAQGK	14	4505753	2xTMT6plex [N-Term; K10]; 1xOxidation [M2	0.924	0.925	0.96	3.114550352	45.18	0	1449.813571	2	717.4119873	-1.365479949	-0.000978925	41.93132997
																			HGESAWNLENR	2	4505753	1xTMT6plex [N-Term]		1.073		4.093710423	36.28	0	1541.765819	2	771.3865967	0.063089591	4.86E-05	36.37188113
																			KAMEAVAAQGK	4	4505753	3xTMT6plex [N-Term; K1; K11]; 1xOxidation [M3	0.629		0.562	3.460566282	46.56	1	1807.071466	3	603.0291748	0.833040691	0.000501788	21.24816771
																			YADLTEDQLPSCESLKDTIAR	2	4505753	2xTMT6plex [N-Term; K16]; 1xCarbamidomethyl [C12				2.043272972	19.33	1	2883.481009	3	961.8305054	-1.403212543	-0.001348712	77.01323238
165905589	LIMA1	LIM domain and actin-binding protein 1 isoform 1	51474	NP_001107018.1	34.07894737	1	22	22	117	760	85.171	6.76	1.04	1.1	1.1	1.08	Significant	0.00034																
																			GNYDEGFGHRPHK	4	165905589	2xTMT6plex [N-Term; K13]	1.217			3.997140884	25.38	0	1972.018963	3	658.0111084	-0.097085622	-6.38E-05	32.72084124
																			GWSMSEQSEESVGGR	6	165905589	1xTMT6plex [N-Term]	1.274	1.483	1.057	5.289067268	59.85	0	1854.848957	2	927.927063	-1.136040447	-0.001053592	44.60840732
																			QSSSTNYTNELK	2	165905589	2xTMT6plex [N-Term; K12]	1.328			3.900020838	58.55	0	1829.964528	2	915.4862061	0.332109921	0.000303875	47.73074175
																			ENVPPGPEVCITHQEGEK	4	165905589	2xTMT6plex [N-Term; K18]; 1xCarbamidomethyl [C10	1.431	1.182		5.235918522	41.34	0	2478.269894	3	826.7610474	-0.526750601	-0.000435143	50.83864541
																			SEISENTDASGK	10	165905589	2xTMT6plex [N-Term; K12]	1.013	1.307	1.033	3.95518899	56.74	0	1695.880129	2	848.4440308	0.386503401	0.000327732	34.23750876
																			ETPHSPGVEDAPIAK	8	165905589	2xTMT6plex [N-Term; K15]	1.072	1.09		5.438776493	55.87	0	2006.095876	3	669.3701782	0.052439153	3.51E-05	46.62036377
																			ISANENSLAVR	6	165905589	1xTMT6plex [N-Term]	1.655	1.247	1.17	4.638221264	57.91	0	1402.785158	2	701.895752	-0.663447103	-0.000465337	43.12697044
																			KGWSMSEQSEESVGGR	2	165905589	2xTMT6plex [N-Term; K1]		1.493		5.642585754	23.65	1	2212.106851	3	738.0406494	0.245689844	0.000181164	40.38327065
																			ADHPPAEVTSHAASGAK	6	165905589	2xTMT6plex [N-Term; K17]	0.389	0.439		5.457952023	39.55	0	2104.118737	4	526.7849121	-0.436440437	-0.000229581	32.44577384
																			GWSMSEQSEESVGGR	6	165905589	1xTMT6plex [N-Term]; 1xOxidation [M4]		1.479	0.884	2.916342258	41.12	0	1870.843872	2	927.927063	-1.136040447	-0.001053592	44.60840732
																			LSLGTYASLHGR	10	165905589	1xTMT6plex [N-Term]	1.255	1.162	1.335	5.040991783	72.17	0	1503.848093	3	501.9516296	-5.158036921	-0.002585635	47.14118394
																			ASSLSESSPPK	2	165905589	2xTMT6plex [N-Term; K11]	1.132			3.725553751	37.77	0	1547.868108	2	774.4364014	-1.668233625	-0.001291103	38.77218752
																			IAWPPPTELGSSGSALEEGIK	10	165905589	2xTMT6plex [N-Term; K21]	1.249	1.043	1.317	2.563341379	25.9	0	2597.422689	3	866.4801636	1.250509849	0.001082701	85.03611731

Bhat et al,2017. Cigarette smoke and chewing tobacco alter expression of different sets of miRNAs in oral keratinocytes

Supplementary Table S2(A). List of all proteins and peptides identified in OKF6/TERT1 cells chronically treated with chewing tobacco using Sequest and Mascot search algorithms

Accession	Gene_Symbol	Description	Gene_ID	Refseq_Accession	Coverage	Proteins	#Unique_Peptides	#Peptides	#PSMS	#AAs	MW [kDa]	calc. pI	OKF6-STE-6M/OKF6P_R1	OKF6-STE-6M/OKF6P_R2	OKF6-STE-6M/OKF6P_R3	Median fold change	Status	P-value (6M)	Sequence	#PSMs	Protein Accessions	Modifications	OKF6-STE-6M/OKF6P_R1	OKF6-STE-6M/OKF6P_R2	OKF6-STE-6M/OKF6P_R3	Xcorr	Ionscore	# Missed Cleavages	Theo. MH+ [Da]	Charge	m_z	DeltaM [ppm]	Deltam/z [Da]	RT [min]
																			LLANQQVFHISCFR	6	165905589	1xTMT6plex [N-Term]; 1xCarbamidomethyl [C12		1.021	0.01	2.665262222	23.51	0	1962.058102	3	654.6900635	-1.256384971	-0.0008217	63.68226121
																			AAEETNMEK	2	165905589	2xTMT6plex [N-Term; K9]			0.807	2.350500345	11.63	0	1480.771765	2	740.8894653	-0.075032453	-5.56E-05	35.58448423
																			ETCVECQK	6	165905589	2xTMT6plex [N-Term; K8]; 2xCarbamidomethyl [C3; C6	1.323	1.097	100	4.023367882	43.42	0	1511.759821	2	756.3837891	0.317910949	0.000240302	32.78431366
																			WPPEDEISKPEVPEDVDLDLK	4	165905589	3xTMT6plex [N-Term; K9; K21]		100		1.580610991	1.81	0	3137.686123	3	1046.565674	-1.164821936	-0.001218282	84.99312455
																			YQAAVSK	5	165905589	2xTMT6plex [N-Term; K7]	1.143	1.14	1.301	2.943887711	36.57	0	1224.735244	2	612.8677368	-5.753796359	-0.003523439	35.61163743
																			SQDVELWEGEVVK	5	165905589	2xTMT6plex [N-Term; K13]	100			1.7989676	4.02	0	1976.074078	2	988.5391235	-1.572668617	-0.001553855	62.89188897
																			EDKPAETK	2	165905589	3xTMT6plex [N-Term; K3; K8]	1.592			1.611209989	7.13	0	1604.946248	3	535.6530151	-1.094117542	-0.000585333	32.06568474
																			LSETSIK	5	165905589	2xTMT6plex [N-Term; K7]	1.726	1.204		3.082800627	27.9	0	1235.761124	2	618.3853149	1.80376512	0.001114511	41.21925901
																			KQVENAK	4	165905589	3xTMT6plex [N-Term; K1; K7]		0.98	0.876	3.017939568	12.09	1	1503.946189	3	501.9872131	0.596752931	0.000299161	26.83254946
																			GEPTQTK	2	165905589	2xTMT6plex [N-Term; K7]	0.925			2.078648567	19.5	0	1218.709423	2	609.8585815	0.380267789	0.000231718	28.46358002
6598323	GDI2	rab GDP dissociation inhibitor beta isoform 1	2665	NP_001485.2	58.42696629	1	16	22	122	445	50.631	6.47	1.37	0.89	1.16	1.14	Significant	0.015																
																			NPYYGGESASITPLEDLYK	4	6598323	2xTMT6plex [N-Term; K19]		1.552	1.511	3.579890013	36.78	0	2575.333206	3	859.114563	-1.58036061	-0.001356652	80.92201473
																			VPSTEAEALASSLMGLFEK	8	6598323	2xTMT6plex [N-Term; K19]		1.93	1.869	4.311440468	32.71	0	2438.325284	3	813.4468384	0.27817729	0.000226096	112.2849694
																			FDLGQDVIDFTGHALALYR	6	6598323	1xTMT6plex [N-Term]	1.789	1.99	1.611	5.401965618	63.9	0	2380.249861	3	794.0871582	-1.235202519	-0.00098003	111.6058456
																			DLGTESQIFISR	6	6598323	1xTMT6plex [N-Term]	1.261	100	0.73	4.131120205	51.27	0	1594.863802	2	797.9356079	0.085951109	6.85E-05	63.35136382
																			TYDATTHFETTCDDIK	10	6598323	2xTMT6plex [N-Term; K16]; 1xCarbamidomethyl [C12	1.375	1.223	100	5.31390667	45.62	0	2376.142962	2	1188.575073	-0.038964669	-4.63E-05	45.24714574
																			KFDLGQDVIDFTGHALALYR	4	6598323	2xTMT6plex [N-Term; K1]	0.808			3.722089767	46.88	1	2737.507756	4	685.1316528	-1.086740985	-0.00074374	105.6699581
																			YIAIVSTTVETK	5	6598323	2xTMT6plex [N-Term; K12]	2.067		100	3.337200165	47.3	0	1783.061723	2	892.0345459	0.051880916	4.63E-05	95.59857806
																			MTGSEFDFEEMK	6	6598323	2xTMT6plex [N-Term; K12]	1.716		100	3.143769979	35.8	0	1908.912358	2	954.9593506	-0.488769493	-0.000466509	73.91258641
																			FVSISDLLVPK	13	6598323	2xTMT6plex [N-Term; K11]	2.072	2.837	100	2.573135614	29.42	0	1676.039865	2	838.5233154	-0.304814125	-0.00025544	102.1579793
																			FKIPGSPPESMGR	6	6598323	2xTMT6plex [N-Term; K2]	0.914	0.195	0.583	4.677865028	19.41	1	1861.04061	3	621.019165	1.252931922	0.000777252	51.6577109
																			DWNVDLIPK	8	6598323	2xTMT6plex [N-Term; K9]	1.759	1.816	1.751	2.552475929	45.72	0	1557.9041	2	779.456543	1.097203287	0.000854669	77.36062454
																			EIRPALELLEPIEQK	4	6598323	2xTMT6plex [N-Term; K15]	100			2.162675858	8.98	0	2236.33169	3	746.1142578	-1.551439134	-0.001156511	73.97561504
																			VTEGSFVYK	8	6598323	2xTMT6plex [N-Term; K9]	100		3.673	3.499011755	26.85	0	1487.851002	2	744.4292603	0.162709715	0.000121044	49.0243421
																			VICILSHPIK	4	6598323	2xTMT6plex [N-Term; K10]; 1xCarbamidomethyl [C3	1.869	1.619		2.927391291	31.62	0	1638.01769	3	546.677063	-0.643799614	-0.000351518	57.65316109
																			QLICDPSYVK	3	6598323	2xTMT6plex [N-Term; K10]; 1xCarbamidomethyl [C4			1.572	2.666646004	30.04	0	1680.939499	2	840.9736938	0.363853257	0.000305808	49.09585783
																			TDDYLDQPCYETINR	4	6598323	1xTMT6plex [N-Term]; 1xCarbamidomethyl [C9		100		1.957126141	25.52	0	2131.980365	2	1066.493896	0.071074177	7.58E-05	49.80331767
																			TFEGIDPK	2	6598323	2xTMT6plex [N-Term; K8]	1.23			2.875322104	36.08	0	1364.782588	2	682.8953247	0.575083584	0.000392432	53.62875956
																			IPGSPPESMGR	5	6598323	1xTMT6plex [N-Term]	1.499	1.562	1.697	2.923561335	19.48	0	1356.714301	2	678.8603516	-0.644774658	-0.000437388	36.746688
																			FLVYVANFDEKDPR	6	6598323	2xTMT6plex [N-Term; K11]	100	100	100	2.145719767	16.81	1	2171.190111	3	724.401062	-0.680808574	-0.000492722	80.06171661
																			LYSESLAR	2	6598323	1xTMT6plex [N-Term]		0.877		3.088897467	36.89	0	1167.657104	2	584.3325806	0.668400689	0.000390231	41.66465892
																			MLLYTEVTR	4	6598323	1xTMT6plex [N-Term]		100		2.687894106	16.05	0	1354.760189	2	677.8829956	-1.088214043	-0.000737135	57.4706702
																			SPYLYPLYGLGELPQGFAR	4	6598323	1xTMT6plex [N-Term]				1.613666534	6.43	0	2370.269534	2	1185.636963	-1.21722391	-0.001442574	98.0294919
4826686	DDX1	ATP-dependent RNA helicase DDX1	1653	NP_004930.1	35.40540541	1	22	22	105	740	82.38	7.23	1.19	1.31	1.11	1.2	Significant	0.0023																
																			GIDIHGVPYVINVTLPDEK	10	4826686	2xTMT6plex [N-Term; K19]	0.817	0.567	0.793	3.682415962	28.2	0	2537.437946	3	846.4883423	4.937277576	0.004176012	83.64179856
																			HYYEVSCHDQGLCR	6	4826686	1xTMT6plex [N-Term]; 2xCarbamidomethyl [C7; C13	1.324	1.542	1.627	4.768105984	33.82	0	2052.921745	4	513.9849854	-1.769884195	-0.000908358	33.36739603
																			GSAFAIGSDGLCCQSR	4	4826686	1xTMT6plex [N-Term]; 2xCarbamidomethyl [C12; C13	100		1.047	3.587023973	49.24	0	1914.899947	2	957.9544678	0.894055524	0.000856013	54.83134907
																			GHQFSCVCLHGDR	12	4826686	1xTMT6plex [N-Term]; 2xCarbamidomethyl [C6; C8	1.297	1.296	1.212	5.717561722	70.55	0	1801.842373	3	601.2838745	-2.942701828	-0.001767428	33.40753912
																			EAQTSFLHLGYLPNQLFR	4	4826686	1xTMT6plex [N-Term]	0.467	0.832		5.045156002	43.74	0	2363.270931	3	788.4279785	-0.655440349	-0.000516328	93.5933627
																			SQHSGNAQVTQTK	13	4826686	2xTMT6plex [N-Term; K13]	1.551	1.971	0.873	5.88020277	54.39	0	1844.002645	3	615.3389893	-0.124770191	-7.67E-05	23.29812772
																			GEDSVPDTVHHVVVPVNPK	6	4826686	2xTMT6plex [N-Term; K19]	1.423	1.713		5.333663464	47.91	0	2483.365843	3	828.4602051	0.08807175	7.29E-05	52.10774211
																			GHVDILAPTVQELAALEK	4	4826686	2xTMT6plex [N-Term; K18]	0.693	1.029		3.599693775	33.13	0	2362.374617	3	788.1288452	-1.115278436	-0.000878235	83.8807821
																			KGHQFSCVCLHGDR	2	4826686	2xTMT6plex [N-Term; K1]; 2xCarbamidomethyl [C7; C9			0.805	4.730222702	28.59	1	2159.100268	4	540.5297852	-1.36945238	-0.000739196	31.88179643
																			IDCDNLEQYFIQQGGGPDK	2	4826686	2xTMT6plex [N-Term; K19]; 1xCarbamidomethyl [C3	100			2.985051394	16.77	0	2655.312487	3	885.7750244	-0.740887493	-0.000655763	82.87582738
																			DNTRPGANSPEMWSEAIK	2	4826686	2xTMT6plex [N-Term; K18]		0.628		2.928865194	7.71	0	2461.254578	3	821.0880127	-2.069446252	-0.001697811	54.85536755
																			VPVDEFDGK	2	4826686	2xTMT6plex [N-Term; K9]			0.987	2.944771051	37.7	0	1463.814616	2	732.411377	0.588193505	0.000430503	59.92425581
																			IMHFPTWVDLK	4	4826686	2xTMT6plex [N-Term; K11]	1.436			2.385943174	30.12	0	1845.049718	3	615.6880493	-0.066995908	-4.12E-05	86.78005215
																			FNFGEEEFK	4	4826686	2xTMT6plex [N-Term; K9]	2.987	100		3.167575121	20.95	0	1604.83608	2	802.9217529	0.093012225	7.46E-05	79.34142581
																			LDDLVSTGK	6	4826686	2xTMT6plex [N-Term; K9]	0.915	0.791	1.447	2.826588869	24.55	0	1405.830266	2	703.4185181	-0.360518051	-0.000253414	59.94247492
																			GKHYYEVSCHDQGLCR	2	4826686	2xTMT6plex [N-Term; K2]; 2xCarbamidomethyl [C9; C15	0.386			1.944075108	14.52	1	2467.201104	4	617.555481	-0.409233715	-0.000252415	40.87555954
																			WQMNPYDR	6	4826686	1xTMT6plex [N-Term]	1.135	1.715	1.185	3.151833057	23.42	0	1338.646222	2	669.8262329	-0.771361149	-0.00051629	52.71263913
																			ALIVEPSR	4	4826686	1xTMT6plex [N-Term]	0.999	0.946		3.023019075	34	0	1113.682925	2	557.3447266	-0.672009046	-0.000374203	50.18982428
																			FGFGFGGTGK	2	4826686	2xTMT6plex [N-Term; K10]	100			1.818091393	10.82	0	1432.798907	2	716.9030762	-0.021640336	-1.55E-05	80.26646679
																			ELLIIGGVAAR	4	4826686	1xTMT6plex [N-Term]	0.01	1.279	100	1.820866942	11.04	0	1340.846302	2	670.9263916	-0.593026116	-0.000397578	79.94595464
																			KYIDNPK	2	4826686	3xTMT6plex [N-Term; K1; K7]			1.073	2.800781012	7.62	1	1564.96659	3	522.3269653	-0.157839731	-8.23E-05	39.23091637
																			FLICTDVAAR	4	4826686	1xTMT6plex [N-Term]; 1xCarbamidomethyl [C4	100	100		1.489454627	12.85	0	1394.766337	2	697.8869019	0.136166849	9.50E-05	56.95140992
46367787	PABPC1	polyadenylate-binding protein 1	26986	NP_002559.2	39.46540881	1	16	23	130	636	70.626	9.5	1.18	1.22	1.19	1.2	Significant	0.0001																
																			GYGFVHFETQEAAER	12	46367787	1xTMT6plex [N-Term]	1.032	1.346	1.432	3.88233757	34.2	0	1969.960556	2	985.484314	0.403764271	0.0003977	60.10458435
																			ALYDTFSAFGNILSCK	6	46367787	2xTMT6plex [N-Term; K16]; 1xCarbamidomethyl [C15			1.48	3.634217501	34.62	0	2265.198961	3	755.7374268	-0.544904259	-0.000411439	105.5330524
																			SKVDEAVAVLQAHQAK	7	46367787	3xTMT6plex [N-Term; K2; K16]	1.138	1.118	0.497	5.281566143	32.06	1	2381.411955	3	794.4745483	-1.202223942	-0.00095433	67.42604239
																			ALDTMNFDVIK	12	46367787	2xTMT6plex [N-Term; K11]	1.131	2.312	1.533	3.615893126	45.3	0	1724.965714	2	862.987793	1.504637153	0.001297724	78.56771478
																			GFGFVSFER	18	46367787	1xTMT6plex [N-Term]	1.105	0.752	100	3.570410728	44.47	0	1274.673088	2	637.8406372	0.713370409	0.000454657	92.73051949
																			QAHLTNQYMQR	4	46367787	1xTMT6plex [N-Term]		1.164	1.173	4.163813591	30.8	0	1618.832125	3	540.2810669	-2.148132813	-0.001159155	31.41164303
																			QAHLTNQYMQR	8	46367787	1xTMT6plex [N-Term]; 1xOxidation [M9]		1.229	1.191	2.696825027	26.44	0	1634.82704	3	540.2810669	-2.148132813	-0.001159155	31.41164303
																			GFGFVCFSSPEEATK	4	46367787	2xTMT6plex [N-Term; K15]; 1xCarbamidomethyl [C6				2.077151775	24.62	0	2121.072698	2	1061.039307	-0.641938747	-0.000680799	80.32784712
																			NLDDGIDDER	4	46367787	1xTMT6plex [N-Term]	1.473	1.695		3.952998877	62.73	0	1390.664768	2	695.8352051	-1.175311108	-0.000817232	43.66517154
																			NFGEDMDDER	2	46367787	1xTMT6plex [N-Term]	1.677			4.400073051	49.69	0	1456.621189	2	728.8132935	-1.289522597	-0.000939173	44.85334427
																			YQGVNLYVK	4	46367787	2xTMT6plex [N-Term; K9]	1.612	1.432		3.390162945	41.18	0	1541.909185	2	771.4580688	-0.210326711	-0.000162152	61.40558547
																			SLGYAYVNFQQPADAER	4	46367787	1xTMT6plex [N-Term]				1.630617619	16.53	0	2158.076648	2	1079.542114	0.140729768	0.000151853	66.79109542
																			KSGVGNIFIK	2	46367787	3xTMT6plex [N-Term; K1; K10]	1.02			3.707781076	15.3	1	1750.119402	3	584.0444336	-0.373916631	-0.000218133	61.57471778
																			ITGMLLEIDNSELLHMLESPESLR	2	46367787	1xTMT6plex [N-Term]				2.538816214	16.52	0	2969.55251	3	990.5228882	0.539246495	0.000533774	107.1042507
																			VDEAVAVLQAHQAK	4	46367787	2xTMT6plex [N-Term; K14]		100		2.365481853	15.74	0	1937.122031	3	646.3781128	-1.159617751	-0.000748774	58.67823186
																			KEFSPFGTITSAK	9	46367787	3xTMT6plex [N-Term; K1; K13]	1.156	0.635	0.502	3.485817909	12.71	1	2100.230803	3	700.7489624	0.729104168	0.000510429	69.39421911
																			EFTNVYIK	6	46367787	2xTMT6plex [N-Term; K8]	1.405	2.472	100	3.533689976	29.39	0	1471.856087	2	736.4310913	-0.802526023	-0.000590601	54.0223207
																			AKEFTNVYIK	3	46367787	3xTMT6plex [N-Term; K2; K10]			0.332	2.459365845	16.05	1	1900.151096	3	634.0551758	-0.064108718	-4.06E-05	49.11956037
																			IVATKPLYVALAQR	2	46367787	2xTMT6plex [N-Term; K5]		1.173		2.393447638	14.06	0	2001.262488	3	667.758606	-0.61136853	-0.000407836	77.31211792
																			SGVGNIFIK	5	46367787	2xTMT6plex [N-Term; K9]	100	100		1.931978106	13.98	0	1392.861507	2	696.934082	-0.444787581	-0.000309764	62.36460739
																			LFPLIQAMHPTLAGK	4	46367787	2xTMT6plex [N-Term; K15]	100	0.435		2.333066463	15.3	0	2095.250209	3	699.0887451	0.703119635	0.000491071	89.08923555
																			EAELGAR	3	46367787	1xTMT6plex [N-Term]	1.54		1.556	2.451565742	37.34	0	974.5468254	2	487.7766113	-0.902330937	-0.000439682	18.29077904
																			VMTDESGK	2	46367787	2xTMT6plex [N-Term; K8]		0.72		2.825691462	15.12	0	1324.718273	2	662.8634644	1.040961664	0.00068949	33.81484989
																			FSPAGPILSIR	3	46367787	1xTMT6plex [N-Term]	0.747			3.254523039	18.91	0	1386.830652	2	693.9199829	1.469191866	0.00101876	80.58045203
63162572	CCT3	T-complex protein 1 subunit gamma isoform a	7203	NP_005989.3	51.00917431	1	26	26	150	545	60.495	6.49	1.1	1.23	1.16	1.17	Significant	0.001																
																			TLIQNCGASTIR	6	63162572	1xTMT6plex [N-Term]; 1xCarbamidomethyl [C6	1.306	1.517	1.435	4.373477459	51.72	0	1562.852192	2	781.9309082	1.50228938	0.001173928	43.27894092
																			AVAQALEVIPR	16	63162572	1xTMT6plex [N-Term]	5.452			3.697134733	48.97	0	1395.852115	2	698.4298096	0.162732587	0.000113575	77.25028164
																			NVLLDPQLVPGGGASEMAVAHALTEK	6	63162572	2xTMT6plex [N-Term; K26]	100	100	100	2.958586216	23.74	0	3075.691276	3	1025.901733	-0.204592929	-0.000209755	85.26274525
																			ISIPVDISDSDMMLNIINSSITTK	16	63162572	2xTMT6plex [N-Term; K24]	100	100	4.461	3.19103837	33.7	0	3065.651445	3	1022.552246	-3.020479959	-0.00308658	106.7030349
																			KGESQTDIEITR	8	63162572	2xTMT6plex [N-Term; K1]	1.725	1.815	1.947	3.992668629	32.96	1	1835.027462	3	612.3464966	-1.376466542	-0.000841951	34.70727596
																			KVQSGNINAAK	6	63162572	3xTMT6plex [N-Term; K1; K11]	0.792	0.958	1.11	4.402420998	25.87	1	1817.121193	3	606.3787231	0.233018907	0.000141141	42.59991358
																			VQSGNINAAK	4	63162572	2xTMT6plex [N-Term; K10]	2.027	1.747		4.01633358	55.55	0	1459.863298	2	730.4351196	-0.229584639	-0.000167581	37.09610428
																			GESQTDIEITR	4	63162572	1xTMT6plex [N-Term]		1.239	1.172	3.943634987	70.12	0	1477.769567	2	739.387146	-1.726938941	-0.001276009	35.12273277
																			GISDLAQHYLMR	8	63162572	1xTMT6plex [N-Term]	1.062	1.271	0.903	3.791966915	33.56	0	1632.872927	3	544.9622192	-0.50378394	-0.000274205	60.59341238
																			IVLLDSSLEYK	9	63162572	2xTMT6plex [N-Term; K11]	1.054	1.344	0.878	3.333396196	38.77	0	1738.040259	2	869.5232544	-0.590786624	-0.000513405	101.9957112
																			NLQDAMQVCR	4	63162572	1xTMT6plex [N-Term]; 1xCarbamidomethyl [C9	1.065	1.191		4.167994976	46.12	0	1463.729634	2	732.3685303	0.102400656	7.49E-05	43.57066882
																			EIQVQHPAAK	4	63162572	2xTMT6plex [N-Term; K10]	1.116	1.101		3.271076918	39.15	0	1578.936797	3	526.984436	1.239939099	0.000652595	32.08052189
																			IDDIVSGHK	5	63162572	2xTMT6plex [N-Term; K9]	1.685	1.591	100	3.628156185	54.51	0	1441.8415	3	481.2854309	0.166269739	7.99E-05	56.35314529
																			ELGIWEPLAVK	4	63162572	2xTMT6plex [N-Term; K11]	100	100		2.469574213	26.45	0	1713.035114	2	857.0201416	-1.230276518	-0.001053753	80.22127487
																			TAVETAVLLLR	6	63162572	1xTMT6plex [N-Term]	0.52	100		1.74547863	25.8	0	1414.883081	2	707.9440308	-1.622901986	-0.001148108	84.86020479
																			NLQDAMQVCR	4	63162572	1xTMT6plex [N-Term]; 1xCarbamidomethyl [C9]; 1xOxidation [M6]	0.905	0.8		2.16311264	22.1	0	1479.724549	2	732.3685303	0.102400656	7.49E-05	43.57066882
																			WSSLACNIALDAVK	4	63162572	2xTMT6plex [N-Term; K14]; 1xCarbamidomethyl [C6				1.755929947	7.88	0	2006.114504	2	1003.561523	0.631422084	0.000633353	92.12742944
																			EILSEVER	4	63162572	1xTMT6plex [N-Term]	0.714	1.087		3.145899057	26.53	0	1203.678233	2	602.3425903	-0.273599645	-0.000164663	46.01634772
																			MLLDPMGGIVMTNDGNAILR	4	63162572	1xTMT6plex [N-Term]	100			1.397638917	4.38	0	2360.233759	2	1180.619263	-1.06363167	-0.00125521	110.1503552
																			KISIPVDISDSDMMLNIINSSITTK	2	63162572	3xTMT6plex [N-Term; K1; K25]				2.434364319	8.67	1	3422.90934	3	1141.639526	-1.552444985	-0.001771293	102.7269835
																			AMTGVEQWPYR	2	63162572	1xTMT6plex [N-Term]				2.264671087	4.79	0	1566.793614	2	783.9008179	0.475456487	0.000372471	61.6351283
																			ALDDMISTLK	2	63162572	2xTMT6plex [N-Term; K10]		100		2.007186651	23.5	0	1564.902051	2	782.9539795	-0.874601464	-0.000684333	75.2644861
																			IVSRPEELREDDVGTGAGLLEIK	4	63162572	2xTMT6plex [N-Term; K23]		100	100	1.856285453	5.7	1	2954.656271	4	739.4195557	0.041248876	3.05E-05	67.08205687
																			TIADIIR	12	63162572	1xTMT6plex [N-Term]	1.062	1.094	1.048	2.371287823	28.86	0	1030.645811	2	515.8256226	-1.787872024	-0.000921331	49.76716347
																			ACTILLR	5	63162572	1xTMT6plex [N-Term]; 1xCarbamidomethyl [C2		0.965	1.029	2.339589119	34.88	0	1075.649517	2	538.3284912	0.175839688	9.46E-05	42.94694992
																			SMIEISR	6	63162572	1xTMT6plex [N-Term]	1.088	1.065	0.99	2.550487518	30.87	0	1064.597146	2	532.802002	-0.393682954	-0.000209557	40.91316717
																			IVSRPEELR	1	63162572	1xTMT6plex [N-Term]	0.601			3.170881987	11.21	0	1327.789515	3	443.2678223	-0.453071152	-0.000200528	38.53140232
388240801	LMNB2	lamin-B2	84823	NP_116126.3	36.12903226	1	20	23	207	620	69.906	5.59	0.99	1.21	0.95	1.05	Significant	0.0059																
																			TVLVNADGEEVAMR	6	388240801	1xTMT6plex [N-Term]	1.323	1.206	1.2	4.883935928	53.42	0	1732.9101	2	866.9581909	-0.574238711	-0.000497552	47.81607318
																			DVMQQQLAEYQELLDVK	10	388240801	2xTMT6plex [N-Term; K17]	1.006	1.836	1.033	3.892138004	50.09	0	2508.341996	3	836.7852783	-0.284869074	-0.000238183	95.50385377
																			ALYESELADAR	8	388240801	1xTMT6plex [N-Term]	1.178	0.882	0.939	4.651884079	64.93	0	1466.768839	2	733.8876343	-0.577613384	-0.000423613	47.64070012
																			GQSSWGTGESFR	2	388240801	1xTMT6plex [N-Term]		1.708		4.727411747	52.41	0	1527.738936	2	764.3729858	-0.157716942	-0.000120475	48.2398047
																			ATSSSGSLSATGR	68	388240801	1xTMT6plex [N-Term]	1.471	1.466	1.783	5.368848324	69.5	0	1497.77063	2	749.3884888	-0.620302548	-0.000464535	22.24938536
																			GLESDVAELR	8	388240801	1xTMT6plex [N-Term]	0.976	1.04	1.124	4.003419876	64.23	0	1317.721161	2	659.3644409	0.33729893	0.000222233	56.34620372
																			QVLEGEEIAYK	4	388240801	2xTMT6plex [N-Term; K11]	1.14	0.838		3.798885345	48.73	0	1736.983729	2	868.994812	-0.647655306	-0.000562483	60.10407552
																			EGELTVAQGR	4	388240801	1xTMT6plex [N-Term]	2.126		1.677	3.484988689	58.09	0	1288.705845	2	644.8565063	-0.084569095	-5.45E-05	40.87790927
																			LALDMEINAYR	4	388240801	1xTMT6plex [N-Term]	1.159			4.371444225	37.26	0	1537.82458	2	769.4157104	-0.283290805	-0.000217826	91.40333902
																			ELEEAMAGER	14	388240801	1xTMT6plex [N-Term]	1.149	1.075	0.853	3.939272165	44.5	0	1363.672496	2	682.3395996	-0.420475775	-0.000286696	41.71241374
																			LELEQTYQAK	10	388240801	2xTMT6plex [N-Term; K10]	0.901	2.165	1.087	3.352884054	35.85	0	1680.957258	2	840.9865112	5.049617353	0.004244095	56.18470573
																			SEVELAAALSDKR	4	388240801	2xTMT6plex [N-Term; K12]			100	2.744043112	25.14	1	1847.063848	3	616.3580322	-2.330423129	-0.001434813	55.55208347
																			MAQALEELR	6	388240801	1xTMT6plex [N-Term]			100	3.020178318	56.01	0	1289.708488	2	645.3572998	-0.903041762	-0.00058233	59.56641095
																			AEDGHAVAK	4	388240801	2xTMT6plex [N-Term; K9]	1.002			3.034524679	37.59	0	1355.768335	3	452.5940552	-0.532954368	-0.000240854	29.22199361
																			ELEEAMAGER	4	388240801	1xTMT6plex [N-Term]; 1xOxidation [M6]		1.098	1.133	2.475153446	43.61	0	1379.667411	2	682.3395996	-0.420475775	-0.000286696	41.71241374
																			LLEGEEER	8	388240801	1xTMT6plex [N-Term]	0.891	0.901	0.91	3.30015111	57.52	0	1203.641848	2	602.324585	0.03776196	2.27E-05	47.24056336
																			AELDEVNK	2	388240801	2xTMT6plex [N-Term; K8]	0.945			3.843690395	48.56	0	1375.783316	2	688.3955078	0.307348545	0.000211423	46.40257871
																			LVEVDSSR	6	388240801	1xTMT6plex [N-Term]	1.224	1.439		3.258022308	36.08	0	1133.636369	2	567.321167	-1.156654516	-0.000655613	41.90901395
																			KLLEGEEER	2	388240801	2xTMT6plex [N-Term; K1]		0.786		2.727909088	12.36	1	1560.899743	3	520.9711914	-0.462452027	-0.000240614	36.54987775
																			LQIELGK	24	388240801	2xTMT6plex [N-Term; K7]	1.154	1.463	100	3.083035707	38.53	0	1258.813494	2	629.9094238	-1.527751139	-0.000961577	71.00877436
																			ISEKEEVTTR	14	388240801	2xTMT6plex [N-Term; K4]	1.195	1.189	0.814	3.115927458	30.98	1	1649.947421	2	825.479126	2.154076596	0.001777057	24.36206006
																			SVFEEEVR	2	388240801	1xTMT6plex [N-Term]	0.532			1.562674403	10.66	0	1223.646933	2	612.3269653	-0.228175244	-0.000139603	50.08366693
																			LESLSYQLSGLQK	2	388240801	2xTMT6plex [N-Term; K13]				1.399608731	4.13	0	1924.115549	2	962.56073	-0.709800959	-0.00068287	71.74505731
																			SEVELAAALSDK	3	388240801	2xTMT6plex [N-Term; K12]				1.231984854	11.27	0	1690.962737	2	845.9837036	-1.541313348	-0.001303152	69.25802569
21361370	PYGB	glycogen phosphorylase, brain form	5834	NP_002853.2	36.77342823	1	21	26	124	843	96.635	6.86	1	1.2	1.07	1.09	Significant	0.0029																
																			GLAGLGDVAEVR	6	21361370	1xTMT6plex [N-Term]	1.098	1.49	1.233	4.487247944	69.83	0	1385.794994	2	693.4017334	0.862888724	0.000597893	58.81860354
																			ARPEYMLPVHFYGR	13	21361370	1xTMT6plex [N-Term]	0.814	1.215	1.907	5.279948235	26.91	0	1965.036638	3	655.6837769	0.070755122	4.63E-05	54.49359385
																			LKQEYFVVAATLQDIIR	7	21361370	2xTMT6plex [N-Term; K2]	1.112	1.252	100	4.556377888	28.94	1	2465.453202	3	822.4914551	2.68113561	0.002203405	111.9239782
																			HLDHVAALFPGDVDR	4	21361370	1xTMT6plex [N-Term]	1.154	1.275		4.488546371	42.22	0	1891.002361	3	631.0062256	0.93194374	0.000587436	57.56803997
																			KTCAYTNHTVLPEALER	2	21361370	2xTMT6plex [N-Term; K1]; 1xCarbamidomethyl [C3			0.586	5.177782059	15.6	1	2461.32735	4	616.0861816	-1.809161021	-0.001113234	42.37037522
																			MSVIEEGDCK	2	21361370	2xTMT6plex [N-Term; K10]; 1xCarbamidomethyl [C9			1.736	3.94049859	49.95	0	1625.8279	2	813.418457	1.068595575	0.000868676	52.90389338
																			LQDFNVGDYIEAVLDR	2	21361370	1xTMT6plex [N-Term]		1.74		3.328777313	31.8	0	2096.08615	2	1048.55127	4.347241405	0.004556096	112.2108817
																			TCAYTNHTVLPEALER	6	21361370	1xTMT6plex [N-Term]; 1xCarbamidomethyl [C2	1.002	0.978	0.828	4.276518822	29.93	0	2104.069455	3	702.0268555	-1.635641705	-0.001147168	48.31273367
																			LLPLVSDEVFIR	4	21361370	1xTMT6plex [N-Term]	1.513	1.275		3.770329952	13.15	0	1629.97771	2	815.4926758	0.224072083	0.000182616	98.31166003
																			AAPGYHMAK	2	21361370	2xTMT6plex [N-Term; K9]; 1xOxidation [M7]	1.237			2.707461119	38.82	0	1419.781877	3	473.9318848	-0.546431207	-0.000258604	30.69714757
																			AAPGYHMAK	2	21361370	2xTMT6plex [N-Term; K9]	1.077			3.208882809	33.2	0	1403.786962	3	473.9318848	-0.546431207	-0.000258604	30.69714757
																			QAVDQISSGFFSPK	6	21361370	2xTMT6plex [N-Term; K14]	100	100	100	1.902932167	16.32	0	1969.079498	2	985.0430298	-0.363103515	-0.00035749	65.49791355
																			VEDVEALDR	6	21361370	1xTMT6plex [N-Term]	1.464	1.487	1.688	3.780227661	56.93	0	1274.678962	2	637.8394165	-5.809472354	-0.003702606	44.46777705
																			DFYELEPEK	4	21361370	2xTMT6plex [N-Term; K9]		1.174	100	2.674793005	33.72	0	1627.86196	2	814.4342041	-0.509095301	-0.000414368	55.79355812
																			EYYDHLPELK	6	21361370	2xTMT6plex [N-Term; K10]	1.271	0.925	0.87	2.640896797	25.76	0	1764.957258	3	588.9902344	-0.62764976	-0.000369258	65.59630758
																			VIPAADLSQQISTAGTEASGTGNMK	2	21361370	2xTMT6plex [N-Term; K25]			100	1.935542226	12.84	0	2905.534107	3	969.1828613	-0.026203659	-2.54E-05	74.00662661
																			WPVSMFEK	6	21361370	2xTMT6plex [N-Term; K8]	100	1.212		3.115939379	36.08	0	1481.822679	2	741.4145508	-0.576113129	-0.000426849	91.27601532
																			LVTSIGDVVNHDPVVGDR	2	21361370	1xTMT6plex [N-Term]	0.733			2.460421324	18.38	0	2121.150147	3	707.7205811	-1.394312393	-0.000985849	64.49020809
																			VAIQLNDTHPALSIPELMR	4	21361370	1xTMT6plex [N-Term]				1.568019867	12.06	0	2347.300517	3	783.1036987	-1.692956895	-0.001324626	79.49454734
																			VIFLENYR	6	21361370	1xTMT6plex [N-Term]	1.631	1.195	5.229	2.893500805	30.16	0	1282.735689	2	641.8728638	2.153365722	0.0013811	71.08699832
																			VLYPNDNFFEGK	7	21361370	2xTMT6plex [N-Term; K12]	1.543	1.582	1.124	2.476181269	19.35	0	1901.02092	2	951.0133057	-0.834173816	-0.000792891	68.80368886
																			ILVDVEK	6	21361370	2xTMT6plex [N-Term; K7]	1.224		1.743	3.156023741	30.99	0	1273.81316	2	637.4091187	-1.726419056	-0.001099568	52.97987958
																			YGNPWEK	8	21361370	2xTMT6plex [N-Term; K7]	1.079	1.426	1.639	3.035600901	28.4	0	1351.741058	2	676.3731079	-1.567104643	-0.00105916	47.43666643
																			INPSSMFDVHVK	2	21361370	2xTMT6plex [N-Term; K12]		1.057		2.188671589	18.24	0	1832.014061	3	611.3427734	-0.160455919	-9.80E-05	76.35838047
																			FSAFLEK	4	21361370	2xTMT6plex [N-Term; K7]	100		100	1.719022155	12.58	0	1299.771295	2	650.3876953	-2.44738056	-0.001590517	68.05565762
																			TITEYAR	4	21361370	1xTMT6plex [N-Term]	0.402	0.839		2.678331137	31.42	0	1082.60434	2	541.8053589	-0.830484905	-0.000449543	39.57279589
																			WLLLCNPGLADTIVEK	1	21361370	2xTMT6plex [N-Term; K16]; 1xCarbamidomethyl [C5				0.876592815		0	2300.308846	2	1150.657104	-0.831960294	-0.000956883	106.6460434
61744477	SLC3A2	4F2 cell-surface antigen heavy chain isoform b	6520	NP_001012680.1	41.2044374	1	20	20	183	631	68.059	5.05	1.24	1.44	1.73	1.47	Significant	0.0092																
																			LKLEPHEGLLLR	6	61744477	2xTMT6plex [N-Term; K2]	1.223	1.548	1.203	4.175922394	33.63	1	1876.178424	3	626.0608521	-5.554534431	-0.003473766	50.37174221
																			DLLLTSSYLSDSGSTGEHTK	13	61744477	2xTMT6plex [N-Term; K20]	1.111	3.086	3.473	2.853666782	25.38	0	2569.339748	3	857.1177368	-0.424362244	-0.000363444	73.10303817
																			GQSEDPGSLLSLFR	18	61744477	1xTMT6plex [N-Term]	1.605		1.209	3.391112089	42.12	0	1734.92238	2	867.9627686	-2.374210324	-0.002059535	84.55929729
																			DDVAQTDLLQIDPNFGSK	10	61744477	2xTMT6plex [N-Term; K18]	2.459	3.2	100	4.312117577	64.33	0	2434.28659	3	812.0993652	-1.251868571	-0.001015802	84.32013627
																			EVELNELEPEKQPMNAASGAAMSLAGAEK	2	61744477	3xTMT6plex [N-Term; K11; K29]	100			2.980067253	17.47	1	3701.933322	3	1234.648926	-0.296706114	-0.000366129	79.52112756
																			IGDLQAFQGHGAGNLAGLK	6	61744477	2xTMT6plex [N-Term; K19]	100	100		3.844726324	29.42	0	2325.307935	3	775.7745972	0.560650005	0.000434561	72.55466695
																			SLLHGDFHAFSAGPGLFSYIR	7	61744477	1xTMT6plex [N-Term]	1.049	1.039		5.28592062	47.47	0	2521.318944	4	631.0847778	-0.659421482	-0.000415653	87.12947243
																			EEGSPLELER	6	61744477	1xTMT6plex [N-Term]	2.034	1.64	2.146	3.88354969	50.36	0	1387.72664	2	694.3666992	-0.373411077	-0.000259096	52.63672812
																			EVELNELEPEK	4	61744477	2xTMT6plex [N-Term; K11]	1.986	1.795		3.483186483	45.8	0	1786.983866	2	893.9941406	-1.601357491	-0.0014308	59.24157342
																			EDFDSLLQSAK	11	61744477	2xTMT6plex [N-Term; K11]	1.546	3.308	1.167	3.315120459	36.73	0	1710.931437	2	855.9684448	-1.065946608	-0.000911881	74.06370275
																			ADLLLSTQPGR	8	61744477	1xTMT6plex [N-Term]	2.024	2.29	1.723	3.760313988	35.58	0	1399.810644	2	700.4088135	-0.210111901	-0.000147058	52.43710896
																			GLVLGPIHK	16	61744477	2xTMT6plex [N-Term; K9]	1.024	1.147	2.031	3.686993837	52.62	0	1391.913877	3	464.6426697	-0.302559522	-0.000140379	63.48095467
																			LEPHEGLLLR	5	61744477	1xTMT6plex [N-Term]	1.864	1.848		3.183146	36.61	0	1405.836465	3	469.2837524	0.169837833	7.96E-05	47.18799657
																			VILDLTPNYR	42	61744477	1xTMT6plex [N-Term]	1.318			4.005477428	36.86	0	1432.836131	2	716.921936	0.324189419	0.000232255	83.49322406
																			WWHTGALYR	5	61744477	1xTMT6plex [N-Term]	1.067	1.041		3.365313292	25.44	0	1418.75307	2	709.8798218	-0.495636152	-0.000351593	55.8540074
																			LDYLSSLK	6	61744477	2xTMT6plex [N-Term; K8]	1.68	2.093	1.145	2.995283842	32.63	0	1396.845188	2	698.9260864	-0.20899842	-0.000145969	67.58025978
																			WCSWSLSQAR	6	61744477	1xTMT6plex [N-Term]; 1xCarbamidomethyl [C2	100	100		2.988624334	31.78	0	1509.746999	2	755.3777466	0.806667649	0.000608932	50.8122444
																			NQKDDVAQTDLLQIDPNFGSK	4	61744477	3xTMT6plex [N-Term; K3; K21]	100			2.477584124	9.84	1	3033.64599	3	1011.885559	-1.274256759	-0.001288548	65.14012682
																			ADLLLSTQPGREEGSPLELER	7	61744477	1xTMT6plex [N-Term]	100		100	1.744074941	14	1	2539.356511	3	847.1221924	-1.767086088	-0.001495754	67.79957615
																			QLPGSHSEAGVQGLSAGDDSETGSDCVTQAGLQLLASSDPPALASK	1	61744477	2xTMT6plex [N-Term; K46]; 1xCarbamidomethyl [C26]				0.858412266	0.31	0	4967.458047	4	1242.61499	-4.00930462	-0.004979013	83.5937929
4506675	RPN1	dolichyl-diphosphooligosaccharide--protein glycosyltransferase subunit 1 precursor	6184	NP_002941.1	54.20098847	1	24	24	150	607	68.527	6.38	1.08	0.94	0.99	1	Significant	0.0017																
																			VIEVSHWGNIAVEENVDLK	28	4506675	2xTMT6plex [N-Term; K19]	0.872	100		3.904468298	19.57	0	2609.433923	3	870.4822998	-0.60419462	-0.000525535	81.16372752
																			VTAEVVLAHLGGGSTSR	6	4506675	1xTMT6plex [N-Term]	1.342	1.195	0.01	4.794380188	50.04	0	1883.054791	3	628.3560791	-0.587654336	-0.000368862	66.40048237
																			DVPAYSQDTFK	6	4506675	2xTMT6plex [N-Term; K11]	1.171	1.166	1.001	3.806073427	70.96	0	1728.920872	2	864.9631958	-1.016326696	-0.000878574	53.75102202
																			FVDHVFDEQVIDSLTVK	10	4506675	2xTMT6plex [N-Term; K17]	1.491	1.102	1.143	4.81222105	40.02	0	2449.337897	3	817.1171265	-0.437287754	-0.000357022	94.56690113
																			TVDLSSHLAK	14	4506675	2xTMT6plex [N-Term; K10]	0.989	0.976	1.106	4.531755924	56.76	0	1528.909914	2	764.9576416	-1.247311472	-0.000953513	41.236291
																			SEDLLDYGPFR	4	4506675	1xTMT6plex [N-Term]	2.229			2.886962891	40.54	0	1540.784489	2	770.8954468	-0.566130642	-0.000436143	96.7527956
																			ISVIVETVYTHVLHPYPTQITQSEK	2	4506675	2xTMT6plex [N-Term; K25]			100	1.94788003	10.93	0	3340.855699	4	835.9693604	-0.026147119	-2.18E-05	97.67909723
																			THYIVGYNLPSYEYLYNLGDQYALK	2	4506675	2xTMT6plex [N-Term; K25]				2.784319878	15.08	0	3455.792764	3	1152.602539	0.086714546	9.99E-05	99.03005211
																			APDELHYTYLDTFGRPVIVAYK	6	4506675	2xTMT6plex [N-Term; K22]	0.802	1.257	0.638	4.04631567	14.27	0	3026.639164	4	757.4169922	2.304367855	0.001743622	76.95374523
																			ALTSEIALLQSR	8	4506675	1xTMT6plex [N-Term]		100	1.03	2.719882488	40.05	0	1530.905273	2	765.9557495	-0.686284502	-0.000525318	70.39755718
																			NIEIDSPYEISR	4	4506675	1xTMT6plex [N-Term]	100	0.794		2.646034718	31.46	0	1664.869281	2	832.9377441	-0.642524168	-0.000534859	53.58718631
																			DISTLNSGK	8	4506675	2xTMT6plex [N-Term; K9]	1.242	1.097	1.256	4.166276932	57.47	0	1392.809865	2	696.9075928	-1.404587355	-0.000978162	33.48009298
																			LAHLGVQVK	6	4506675	2xTMT6plex [N-Term; K9]	0.906	0.758	0.825	3.668379545	46.01	0	1422.919691	3	474.9780273	-0.113687899	-5.39E-05	44.45404258
																			VACITEQVLTLVNK	3	4506675	2xTMT6plex [N-Term; K14]; 1xCarbamidomethyl [C3	100			2.949534416	19.18	0	2046.203319	2	1023.604492	-0.787192057	-0.000805377	106.1998177
																			NLVEQHIQDIVVHYTFNK	2	4506675	2xTMT6plex [N-Term; K18]	100			2.843779325	11.61	0	2655.465892	4	664.6210938	-1.260234601	-0.000836627	88.26228909
																			ATSFLLALEPELEAR	7	4506675	1xTMT6plex [N-Term]				2.356921196	26.83	0	1889.058145	2	945.0315552	-1.22334426	-0.001155484	98.66563911
																			LPVALDPGAK	6	4506675	2xTMT6plex [N-Term; K10]	1.37	1.26	1.613	3.220657349	46.14	0	1438.903372	2	719.954895	-0.596517427	-0.000429165	63.88131963
																			QFVVFEGNHYFYSPYPTK	2	4506675	2xTMT6plex [N-Term; K18]			100	2.547781944	19.13	0	2681.380431	3	894.463623	-1.534597377	-0.001371613	78.77351023
																			LKTEGSDLCDR	2	4506675	2xTMT6plex [N-Term; K2]; 1xCarbamidomethyl [C9	1.152			3.071758509	18.48	1	1751.936205	3	584.6500854	-0.286421584	-0.000167264	42.92361919
																			DTYIENEK	4	4506675	2xTMT6plex [N-Term; K8]	1.17		1.751	4.036101341	42.2	0	1469.788795	2	735.3919678	-8.257304153	-0.006068247	38.34153699
																			IILPEGAK	4	4506675	2xTMT6plex [N-Term; K8]		1.099	3.531	2.744026423	24.84	0	1298.844794	2	649.9259644	-0.10949658	-7.11E-05	64.21126249
																			SLETEHK	4	4506675	2xTMT6plex [N-Term; K7]	1.068	1.085		2.727293968	40.08	0	1301.746537	3	434.5866089	-0.970519074	-0.000421123	29.48157941
																			TILPAAAQDVYYR	6	4506675	1xTMT6plex [N-Term]	2.15	100	1.014	2.781565905	23.2	0	1709.942387	2	855.4758911	1.239115761	0.001059408	64.92224936
																			IDHILDAL	6	4506675	1xTMT6plex [N-Term]	0.947	0.94	0.738	1.765517831	45.35	0	1138.66694	2	569.8372803	0.30162189	0.000171723	93.44773128
386781571	RDX	radixin isoform 1	5962	NP_001247422.1	38.74172185	1	12	28	176	604	71.005	6.71	1.06	1.28	1.1	1.15	Significant	0.0035																
																			AFAAQEDLEK	14	386781571	2xTMT6plex [N-Term; K10]	0.987	1.061	1.131	4.739621162	62.32	0	1579.873194	2	790.4358521	-5.548697498	-0.004383119	47.03481287
																			ILALCMGNHELYMR	8	386781571	1xTMT6plex [N-Term]; 1xCarbamidomethyl [C5	0.981	0.797		4.492577076	47.63	0	1949.996096	3	650.6697998	-0.640778687	-0.000416505	79.80310241
																			KPDTIEVQQMK	9	386781571	3xTMT6plex [N-Term; K1; K11]	0.928	0.765	0.866	4.951767921	43.06	0	2004.176659	3	668.7305908	0.279696371	0.000186854	42.3317132
																			KAPDFVFYAPR	12	386781571	2xTMT6plex [N-Term; K1]	0.737	0.828	0.648	3.476797581	26.85	1	1769.015047	3	590.3424683	-1.24127539	-0.000731945	54.53779583
																			TQNDVLHAENVK	2	386781571	2xTMT6plex [N-Term; K12]	1.222			3.709805012	29.58	0	1826.017232	3	609.3443604	0.709601566	0.000431915	42.89012034
																			APDFVFYAPR	18	386781571	1xTMT6plex [N-Term]	0.79	0.794	0.433	3.595981359	31.71	0	1411.757152	2	706.3810425	-1.660391136	-0.001172035	65.18701339
																			RKPDTIEVQQMK	18	386781571	3xTMT6plex [N-Term; K2; K12]	0.874	0.775	0.851	4.093585968	16.43	1	2160.27777	4	540.8242188	-1.261175779	-0.000681122	39.6647313
																			VTTMDAELEFAIQPNTTGK	2	386781571	2xTMT6plex [N-Term; K19]	100			2.649563313	29.67	0	2524.336911	3	842.1146851	-2.935002133	-0.002469645	79.69920076
																			KPDTIEVQQMK	8	386781571	3xTMT6plex [N-Term; K1; K11]; 1xOxidation [M10			0.855	4.497064114	24.67	0	2020.171574	3	668.7305908	0.279696371	0.000186854	42.3317132
																			QLFDQVVK	12	386781571	2xTMT6plex [N-Term; K8]	1.369	1.393	1.305	4.03649807	44.99	0	1434.872072	2	717.9403687	0.968068663	0.000694527	67.82446326
																			KFVIKPIDK	4	386781571	4xTMT6plex [N-Term; K1; K5; K9]	0.446	0.888		2.754837513	4.15	1	2004.339121	4	501.8404541	0.432055754	0.000216497	57.66246428
																			IALLEEAK	6	386781571	2xTMT6plex [N-Term; K8]	0.968	1.689	1.049	3.40948987	42.25	0	1344.850274	2	672.9276733	-1.638480027	-0.001101755	67.21163618

Bhat et al,2017. Cigarette smoke and chewing tobacco alter expression of different sets of miRNAs in oral keratinocytes

Supplementary Table S2(A). List of all proteins and peptides identified in OKF6/TERT1 cells chronically treated with chewing tobacco using Sequest and Mascot search algorithms

Accession	Gene_Symbol	Description	Gene_ID	Refseq_Accession	Coverage	Proteins	#Unique_Peptides	#Peptides	#PSMS	#AAs	MW [kDa]	calc. pI	OKF6-STE-6M/OKF6P_R1	OKF6-STE-6M/OKF6P_R2	OKF6-STE-6M/OKF6P_R3	Median fold change	Status	P-value (6M)
21361794	CAND1	cullin-associated NEDD8-dissociated protein 1	55832	NP_060918.2	25.85365854	1	25	25	128	1230	136.289	5.78	1.17	1.27	1.21	1.22	Significant	0.00057
20127408	HADHA	trifunctional enzyme subunit alpha, mitochondrial precursor	3030	NP_000173.2	41.80865007	1	24	24	109	763	82.947	9.04	0.59	0.88	0.76	0.74	Significant	0.013
937827788	LDHB	L-lactate dehydrogenase B chain isoform LDHBx	3945	NP_001302466.1	59.82404692	1	17	19	238	341	37.381	6.25	1.27	1.26	1.21	1.25	Significant	0.00022
20127454	ATIC	bifunctional purine biosynthesis protein PURH	471	NP_004035.2	48.47972973	1	22	22	127	592	64.575	6.71	1.15	1.25	1.12	1.18	Significant	0.0011
5803225	YWHAE	14-3-3 protein epsilon	7531	NP_006752.1	69.41176471	1	17	20	296	255	29.155	4.74	1.14	1.24	1.18	1.19	Significant	0.0006
746815975	TUBB6	tubulin beta-6 chain isoform 1	84617	NP_001290453.1	61.65919283	1	9	18	151	446	49.825	4.88	0.97	1.08	1.01	1.02	Significant	0.00099
226529917	TPI1	triosephosphate isomerase isoform 2	7167	NP_001152759.1	67.48251748	1	15	15	214	286	30.772	5.92	0.9	0.94	0.96	0.93	Significant	0.00036
153266822	ALDH1A3	aldehyde dehydrogenase family 1 member A3 isoform 1	220	NP_000684.2	43.1640625	1	18	20	211	512	56.073	7.25	1.25	1.22	1.4	1.29	Significant	0.0019
4503841	XRCC6	X-ray repair cross-complementing protein 6 isoform 1	2547	NP_001460.1	51.39573071	1	24	24	140	609	69.799	6.64	1.37	1.1	1.44	1.3	Significant	0.0063

Sequence	#PSMs	Protein Accessions	Modifications	OKF6-STE-6M/OKF6P_R1	OKF6-STE-6M/OKF6P_R2	OKF6-STE-6M/OKF6P_R3	Xcorr	Ionscore	# Missed Cleavages	Theo. MH+ [Da]	Charge	m_z	DeltaM [ppm]	Deltam/z [Da]	RT [min]
KTQNDVLHAENVK	2	386781571	3xTMT6plex [N-Term; K1; K13]			1.059	3.068309546	8.36	1	2183.275127	4	546.5740356	-0.372981795	-0.00020358	39.39542735
KKEEEATEWQHK	2	386781571	4xTMT6plex [N-Term; K1; K2; K12]	1.372			3.314103842	1.63	2	2459.406425	4	615.6062012	-1.40266467	-0.000862431	38.79572104
EAILNDEIYCPPETAVLLASYAVQAK	4	386781571	2xTMT6plex [N-Term; K26]; 1xCarbamidomethyl [C10				2.07301569	23.58	0	3336.780151	3	1112.93042	-1.0322689	-0.001148151	107.1891541
FVIKPIDKK	2	386781571	4xTMT6plex [N-Term; K4; K8; K9]	0.614	0.411		2.204612494		1	2004.339121	4	501.8398743	-0.725101692	-0.000363337	63.42701961
KENPLQFK	6	386781571	3xTMT6plex [N-Term; K1; K8]	1.141	0.83	1.245	3.384720564	22.69	1	1691.045903	3	564.354248	1.353145188	0.000762744	50.20749501
NQEQLAAELAEFTAK	3	386781571	2xTMT6plex [N-Term; K15]				2.025681973	4.43	0	2121.159205	2	1061.081665	-1.485617802	-0.001575616	87.85498235
IGFPWSEIR	4	386781571	1xTMT6plex [N-Term]		0.996	1.093	2.909641981	30.66	0	1333.746588	2	667.3757935	-1.70756271	-0.001138728	82.08487989
FVIKPIDK	8	386781571	3xTMT6plex [N-Term; K4; K8]	1.014	0.898	0.786	2.657356024	10.37	0	1647.081226	3	549.6986694	0.139319651	7.65E-05	56.62551159
ALELDQER	2	386781571	1xTMT6plex [N-Term]	0.934			3.238109589	38.09	0	1202.657832	2	601.8331909	1.058435661	0.000636468	39.98958084
EDSMMEYLK	2	386781571	2xTMT6plex [N-Term; K9]				1.219772696	11.76	0	1603.811187	2	802.4084473	-0.978424869	-0.000784604	71.51947979
ENPLQFK	10	386781571	2xTMT6plex [N-Term; K7]	1.214	0.567	1.292	2.805651665	23.95	0	1333.788008	2	667.3933105	-6.495204785	-0.004331613	50.09883136
QAADQMK	4	386781571	2xTMT6plex [N-Term; K7]	1.172	1.388		2.957131147	31.84	0	1249.697478	2	625.3516235	-1.206459734	-0.000753855	32.65846343
LFFLQVK	2	386781571	2xTMT6plex [N-Term; K7]	0.846			2.326160192	22.22	0	1352.870615	2	676.9376831	-1.866829713	-0.00126279	89.42239087
VTQQDVK	2	386781571	2xTMT6plex [N-Term; K7]		1.607		2.775376081	29.25	0	1275.767272	2	638.3877563	0.755494622	0.000481918	31.25462266
AQLENEK	2	386781571	2xTMT6plex [N-Term; K7]	1.103			3.151601791	39.15	0	1289.746537	2	645.3763428	-0.874476568	-0.000563927	35.67662432
EKEELMER	4	386781571	2xTMT6plex [N-Term; K2]	0.823	0.839		2.509242773	8.85	1	1521.8347	3	507.949707	-0.086682377	-4.40E-05	35.52617194
ELEEQTR	4	386781571	1xTMT6plex [N-Term]	1.072	0.521		2.411992311	26.64	0	1133.599983	2	567.3035278	-0.17998054	-0.000102013	27.75071337
GYLISGSSYAR	10	21361794	1xTMT6plex [N-Term]	1.248	1.372	1.318	4.344771862	59.77	0	1402.752795	2	701.8803101	0.390843768	0.000274129	44.77371231
SPLMSEFQSQISSNPELAAIFESIQK	6	21361794	2xTMT6plex [N-Term; K26]		1.198		3.482647896	22.85	0	3339.754664	4	835.6934814	-0.768999225	-0.000642067	114.0912876
FTISDHPQPIDPLLK	4	21361794	2xTMT6plex [N-Term; K15]	1.495	0.892		4.091824055	25.61	0	2179.252711	3	727.0894775	0.536044848	0.000389392	84.47443083
ISGSILNELIGLVR	2	21361794	1xTMT6plex [N-Term]	100			2.904150724	47.52	0	1713.047186	2	857.0266724	-0.652681051	-0.000559037	112.9314038
AVAALLTIPEAEK	12	21361794	2xTMT6plex [N-Term; K13]	1.084	1.254	100	3.068420887	36.54	0	1784.093357	2	892.5510254	0.794241649	0.000708501	81.7359782
DLLDTVLPHLYNETK	10	21361794	2xTMT6plex [N-Term; K15]	100	100		2.816686153	25.06	0	2229.253105	3	743.7559204	0.046172626	3.43E-05	87.00967529
FCNVDDDELR	2	21361794	1xTMT6plex [N-Term]; 1xCarbamidomethyl [C2			1.207	4.235578537	53.52	0	1511.699774	2	756.3527832	-0.98156643	-0.000741917	44.19405412
IDLRPVLGEGVPILASFLR	4	21361794	1xTMT6plex [N-Term]	100			2.552932024	8.4	0	2294.379753	3	765.463623	-1.498260857	-0.00114586	112.3652236
ALTLIAGSPLK	5	21361794	2xTMT6plex [N-Term; K11]	3.271		1.421	3.241964817	33.26	0	1542.003086	2	771.5061035	1.196282463	0.000922336	70.93266066
AADIDQEVK	10	21361794	2xTMT6plex [N-Term; K9]	1.057	0.999	1.014	3.973841667	58.32	0	1446.82043	2	723.9151001	1.72354859	0.001246833	42.39584724
TLEDPDLNVR	2	21361794	1xTMT6plex [N-Term]			1.27	4.210573673	47.12	0	1400.758274	2	700.8832397	0.662764022	0.000464186	52.14190071
CLDAVVSTR	4	21361794	1xTMT6plex [N-Term]; 1xCarbamidomethyl [C1	1.215	1.165		3.406436443	42.51	0	1249.677188	2	625.3425903	0.573111256	0.000358102	40.95917111
DSSSTNLESMDTS	4	21361794	1xTMT6plex [N-Term]	1.401	1.49		3.094429493	54.29	0	1602.700227	2	801.8527222	-1.284671973	-0.001029472	44.29772555
TVSPALISR	4	21361794	1xTMT6plex [N-Term]	1.257	1.24		3.178880453	40.02	0	1172.720039	2	586.8635864	-0.121451355	-7.12E-05	49.16628047
MLTGPVYSQSTALTHK	2	21361794	2xTMT6plex [N-Term; K16]	0.863			2.933063984	25.64	0	2192.214945	3	731.4100342	0.275442453	0.000201276	65.56698912
DSSSTNLESMDTS	2	21361794	1xTMT6plex [N-Term]; 1xOxidation [M10]		100		1.484479427	16.1	0	1618.695142	2	801.8527222	-1.284671973	-0.001029472	44.29772555
LTLIDPETLLPR	10	21361794	1xTMT6plex [N-Term]	100			2.680139542	27.86	0	1609.972624	2	805.4898682	-0.102238928	-8.23E-05	95.97800428
DLFTCTIK	4	21361794	2xTMT6plex [N-Term; K8]; 1xCarbamidomethyl [C5	1.228	1.2		3.958589315	41.62	0	1455.828158	2	728.4176636	-0.073965847	-5.38E-05	70.9781517
VYPSSLSK	4	21361794	2xTMT6plex [N-Term; K8]	0.957	1.298		2.516600609	43.74	0	1338.803323	2	669.9050293	-0.404403127	-0.000270708	52.58164258
SSVVTAVK	6	21361794	2xTMT6plex [N-Term; K8]		1.282	1.241	3.550292969	53.38	0	1248.792759	2	624.9006958	1.08605015	0.000678126	36.44694317
LGTLSALDILIK	2	21361794	2xTMT6plex [N-Term; K12]				1.70276022	17	0	1715.108279	2	858.0578613	0.097396912	8.35E-05	106.7390844
VIRPLDQPSSFDATPYIK	6	21361794	2xTMT6plex [N-Term; K18]		100	100	2.095910072	8.51	0	2505.411731	3	835.8084106	-0.419957484	-0.000350722	62.14205544
EYCIQAFESFVR	5	21361794	1xTMT6plex [N-Term]; 1xCarbamidomethyl [C3				1.41115284	8.52	0	1777.878072	2	889.4413452	-1.495305192	-0.001329235	78.91309453
ITSEALLVTQQLVK	4	21361794	2xTMT6plex [N-Term; K14]			100	2.142518759	13	0	2001.235999	2	1001.119019	-2.617402759	-0.00261902	87.30097141
SVILEAFSSPSEEVK	3	21361794	2xTMT6plex [N-Term; K15]				1.180625319	2.71	0	2080.157808	2	1040.580811	-1.664895922	-0.001731623	72.46763238
TYIQCIAAISR	1	21361794	1xTMT6plex [N-Term]; 1xCarbamidomethyl [C5				1.259147882		0	1524.840565	2	762.9240112	0.118813037	9.06E-05	66.90429807
EVEAVIPDHCIFASNTSALPISEIAAVSK	7	20127408	2xTMT6plex [N-Term; K29]; 1xCarbamidomethyl [C10			0.466	3.837153912	19.81	0	3526.886741	4	882.4764404	-0.796552727	-0.000702338	86.84099338
ALMGLYHGQVLCK	12	20127408	2xTMT6plex [N-Term; K13]; 1xCarbamidomethyl [C12	0.714		0.459	4.599730968	39.83	0	1948.091266	3	650.0352173	-0.085819661	-5.57E-05	73.05419785
KTVLGTPEVLLGALPGAGGTQR	4	20127408	2xTMT6plex [N-Term; K1]		1.007		5.120960712	30.36	1	2593.544142	3	865.1854248	-0.933381429	-0.000806922	87.6341326
SEVSSDEDIQFR	10	20127408	1xTMT6plex [N-Term]	0.921	1.016	0.909	4.384746075	67.04	0	1640.79651	2	820.9007568	-1.385478401	-0.001136644	41.00787835
QFTPCQLLADHANSPNKK	4	20127408	3xTMT6plex [N-Term; K17; K18]; 1xCarbamidomethyl [C5	0.609	0.986		4.988275528	31.39	1	2756.51208	4	689.8838501	0.540839504	0.000372708	57.64985587
DSIFSNLTGQLDYQGFEK	4	20127408	2xTMT6plex [N-Term; K18]			0.579	2.944125652	21.95	0	2520.30224	3	840.7709351	-1.582347607	-0.001329331	91.92850698
ELHSEFSEVMNEIWASDQIR	2	20127408	1xTMT6plex [N-Term]	100			4.118554592	21.54	0	2649.281632	3	883.7661743	0.882510636	0.00077934	100.2888573
VIGMHYFSPVDK	6	20127408	2xTMT6plex [N-Term; K12]	0.71	0.587	0.625	2.938730478	35.56	0	1851.023897	3	617.678833	-1.054376751	-0.000650559	70.5599144
ADMVIEAVFEDLSLK	6	20127408	2xTMT6plex [N-Term; K15]				1.793410301	22.11	0	2138.181914	2	1069.595581	0.922040058	0.000985745	109.4453981
MQLLEIITTEK	4	20127408	2xTMT6plex [N-Term; K11]		100		2.323219299	32.11	0	1777.054529	2	889.0314941	0.665613368	0.000591416	90.55761843
GFYIYQEGVK	5	20127408	2xTMT6plex [N-Term; K10]	0.736	1.303		2.714400291	36.89	0	1661.930315	2	831.4699097	1.34061935	0.001114008	66.64592351
FVDLYGAQK	6	20127408	2xTMT6plex [N-Term; K9]	0.644	0.874	0.811	3.805128098	43.49	0	1498.866986	2	749.9370117	-0.159728983	-0.000119706	64.63959633
VIGMHYFSPVDK	2	20127408	2xTMT6plex [N-Term; K12]; 1xOxidation [M4		1.082		3.225206614	12.25	0	1867.018812	3	617.678833	-1.054376751	-0.000650559	70.5599144
DLNSDMDSILASLK	5	20127408	2xTMT6plex [N-Term; K14]				2.761045694	25.17	0	1980.072364	2	990.5393066	-0.518626879	-0.000513459	103.7240786
KLDSLTTSFGFPVGAATLVDEVGVDVAK	3	20127408	3xTMT6plex [N-Term; K1; K28]				2.367375851	6.07	1	3523.986662	3	1175.329956	-3.219962794	-0.003782369	99.6617815
HVAEDLGK	2	20127408	2xTMT6plex [N-Term; K8]			0.723	3.196524858	38.32	0	1326.778171	2	663.8929443	0.332219722	0.000220391	36.87742647
TLQEVTQLSQEAQR	2	20127408	1xTMT6plex [N-Term]				1.692409158	15.08	0	1860.002421	2	930.5048828	0.036728447	3.42E-05	54.67982111
TVLGTPEVLLGALPGAGGTQR	3	20127408	1xTMT6plex [N-Term]				1.797980547	14.65	0	2236.286247	2	1118.638916	-7.016793477	-0.007845779	84.72726635
LTAYAMTIPFVR	2	20127408	1xTMT6plex [N-Term]				1.376690865	6.85	0	1611.913001	2	806.460083	-0.069255831	-5.58E-05	98.9256806
FGELVMTK	2	20127408	2xTMT6plex [N-Term; K8]	1.211			2.556672812	21.32	0	1382.81178	2	691.9107666	1.791258334	0.001238487	70.08774729
GLYPAPLK	2	20127408	2xTMT6plex [N-Term; K8]	0.615			2.909995079	19.56	0	1316.83423	2	658.9198608	-1.355212579	-0.000892295	61.35285384
DATLTALDR	2	20127408	1xTMT6plex [N-Term]	0.803			2.48720932	34.24	0	1204.673482	2	602.840271	-0.180105079	-0.000108484	49.72982282
MVGVPAALDMMLTGR	6	20127408	1xTMT6plex [N-Term]		100		1.3501724	1.95	0	1790.952834	2	895.9790649	-1.105756193	-0.000990179	97.83315599
GQQQVFK	6	20127408	2xTMT6plex [N-Term; K7]	0.677	0.677	0.696	2.879240751	40.18	0	1292.772692	2	646.8898315	-0.236347862	-0.000152772	43.09954799
DKGLVEK	2	20127408	3xTMT6plex [N-Term; K2; K7]	0.272			2.448219299	4.43	1	1475.940041	3	492.6518555	0.658892789	0.000324162	37.32339687
GYTNWAIGLSVADLIESMLK	2	937827788	2xTMT6plex [N-Term; K20]	100			2.810222626	38.97	0	2639.451881	3	880.4902344	1.617314955	0.001422942	109.8330195
VIGSGCNLDSAR	28	937827788	1xTMT6plex [N-Term]; 1xCarbamidomethyl [C6	0.938	0.934	0.93	4.715711594	77.43	0	1477.763043	2	739.3862915	1.531901764	0.001131894	37.24424387
MVVESAYEVIK	12	937827788	2xTMT6plex [N-Term; K11]	1.748	100	1.792	4.832505703	68.16	0	1725.986115	2	863.4960327	-0.768343558	-0.000663075	72.33213179
SLADELALVDVLEDKLK	4	937827788	3xTMT6plex [N-Term; K15; K17]	1.111	0.506		5.331680775	50.64	1	2558.525981	3	853.5131836	-0.384517788	-0.000327933	109.8330889
SLADELALVDVLEDK	16	937827788	2xTMT6plex [N-Term; K15]	2.081	1.48	2.622	4.008817673	56.85	0	2088.184022	2	1044.593872	-1.702397555	-0.00177746	102.5980595
GEMMDLQHGSLFLQTPK	8	937827788	2xTMT6plex [N-Term; K17]	1.825	1.828	3.708	3.616232634	27.96	0	2390.261244	3	802.757019	0.14346723	0.000115073	70.08780419
LKDDEVAQLK	20	937827788	3xTMT6plex [N-Term; K2; K10]	1.185	1.332	1.299	4.558996677	41.97	1	1846.125275	2	923.5656738	-0.652184208	-0.000602007	51.49293506
SADTLWDIQK	12	937827788	2xTMT6plex [N-Term; K10]	1.292	1.33	1.158	4.098417282	61.15	0	1634.915393	2	817.9608154	-0.635280963	-0.000519315	72.94622375
GEMMDLQHGSLFLQTPK	6	937827788	2xTMT6plex [N-Term; K17]; 1xOxidation [M	1.422	1.398		3.723464966	37.15	0	2406.256159	3	802.757019	0.14346723	0.000115073	70.08780419
GLTSVINQK	14	937827788	2xTMT6plex [N-Term; K9]	1.31	1.156	1.216	4.447964191	59.81	0	1417.877885	2	709.4424438	-0.193376799	-0.000137092	52.46419317
YSPDCIIIVVSNPVDILTYVTWK	2	937827788	2xTMT6plex [N-Term; K23]; 1xCarbamidomethyl [C5		2.101		3.992558241	32.64	0	3153.731016	3	1051.918091	2.759623726	0.002901037	0.251553897
IVADKDYSVTANSK	22	937827788	3xTMT6plex [N-Term; K5; K14]	1.17	1.235	1.695	4.356412888	52.93	1	2198.263559	2	1099.636597	1.072432541	0.001178745	44.21712632
LIAPVAEEEATVPNNK	12	937827788	2xTMT6plex [N-Term; K16]	1.542	1.719	1.343	2.276959419	21.14	0	2153.221805	2	1077.112793	-1.623433541	-0.001747806	55.06761483
DYSVTANSK	6	937827788	2xTMT6plex [N-Term; K9]	1.992	1.927	0.964	4.257129669	58.88	0	1442.78913	2	721.8989258	1.001638057	0.000722576	34.35762609
IVVVTAGVR	47	937827788	1xTMT6plex [N-Term]	1.114	1.345	1.072	3.312609196	38.57	0	1142.74586	2	571.8757935	-1.355704704	-0.000774613	51.25098199
GMYGIENEVFLSLPCILNAR	6	937827788	1xTMT6plex [N-Term]; 1xCarbamidomethyl [C15				1.734449148	12.24	0	2525.309367	2	1263.156006	-1.834274767	-0.002316056	107.8690156
LKGEMMDLQHGSLFLQTPK	2	937827788	3xTMT6plex [N-Term; K2; K19]		100		1.887521148	7.96	1	2860.603203	4	719.9039307	-1.468670136	-0.001056193	78.58841109
FIIPQIVK	10	937827788	2xTMT6plex [N-Term; K8]	1.772	1.393	1.657	3.089707851	35.54	0	1415.939029	2	708.4726563	-0.701414383	-0.00049658	85.79408468
LKGEMMDLQHGSLFLQTPK	4	937827788	3xTMT6plex [N-Term; K2; K19]; 1xOxidation [M			1.02	3.168097496	13.83	1	2876.598118	4	719.9039307	-1.468670136	-0.001056193	78.58841109
MVVESAYEVIK	2	937827788	2xTMT6plex [N-Term; K11]; 1xOxidation [M1	2.409			1.786210775	14.42	0	1741.98103	2	863.4960327	-0.768343558	-0.000663075	72.33213179
LKDDEVAQLKK	1	937827788	4xTMT6plex [N-Term; K2; K10; K11]		0.681		2.161568642		2	2203.38317	4	551.6018066	1.010569805	0.000556668	44.81131808
QQEGESR	2	937827788	1xTMT6plex [N-Term]				1.745908499	15.4	0	1062.537717	2	531.7727051	0.391831973	0.000208168	11.0366653
TLHPAVHAGILAR	12	20127454	1xTMT6plex [N-Term]	0.971	0.995	0.925	4.775541306	35.37	0	1584.953561	3	528.9893188	-0.099313339	-5.25E-05	39.43829768
APGQLALFSVSDK	7	20127454	2xTMT6plex [N-Term; K13]	0.602	0.87			41.49	0	1791.041656	2	896.024353	-0.12647127	-0.000113258	80.17057642
EVSDGIIAPGYEEEALTILSK	8	20127454	2xTMT6plex [N-Term; K21]	1.385	2.876		3.928160429	36.85	0	2692.469699	2	1346.737305	-0.878852973	-0.001183142	102.4683512
AEISNAIDQYVTGTIGEDEDLIK	9	20127454	2xTMT6plex [N-Term; K23]	2.782	2.292	3.404	3.634459734	41.38	0	2952.545383	3	984.8522339	-1.095525868	-0.001078197	108.0738898
HVSPAGAAVGIPLSEDEAK	4	20127454	2xTMT6plex [N-Term; K19]	1.179		1.158	6.134141922	64.76	0	2306.275631	3	769.4282227	-2.39200431	-0.001838874	53.53248491
YTQSNSVCYAK	4	20127454	2xTMT6plex [N-Term; K11]; 1xCarbamidomethyl [C8	2.039	1.633		5.276547909	56.29	0	1778.914741	2	889.9611816	0.194197755	0.000172731	46.16253805
VTVVCEPEDYVVVSTEMQSSESK	2	20127454	2xTMT6plex [N-Term; K23]; 1xCarbamidomethyl [C5	0.01			2.789419889	16.97	0	3060.515739	3	1020.842346	-1.063254059	-0.001084702	79.65283233
VVACNLYPFVK	6	20127454	2xTMT6plex [N-Term; K11]; 1xCarbamidomethyl [C4	1.604			2.711179018	35.27	0	1768.02317	2	884.5148926	-0.373860344	-0.000330497	95.92863171
NIPEDNADMAR	4	20127454	1xTMT6plex [N-Term]	1.41			3.469158649	37.33	0	1474.715758	2	737.8609619	-0.753048083	-0.000555266	38.50648582
SLFSNVVTK	8	20127454	2xTMT6plex [N-Term; K9]	1.067	1.137		3.697478056	46.1	0	1452.882636	2	726.944519	-0.60212989	-0.000437412	71.08138254
DVSELTGFPEMLGGR	6	20127454	1xTMT6plex [N-Term]	0.638	1.627	100	2.815735579	40.55	0	1836.936315	2	918.9713135	-0.52517165	-0.000482353	77.56681454
VVIEACDELGIILAHTNLR	9	20127454	1xTMT6plex [N-Term]; 1xCarbamidomethyl [C6	100			2.286973476	12.94	0	2365.311082	3	789.1073608	-1.501925263	-0.001184173	108.5886178
AFTHTAQYDEAISDYFR	8	20127454	1xTMT6plex [N-Term]	100	1.119	4.408	3.13385129	14.7	0	2264.082127	3	755.3638916	-2.211004297	-0.001668632	70.05175439
LTEVSISSDAFFPFR	3	20127454	1xTMT6plex [N-Term]				2.089955807	16.83	0	1945.026845	2	973.0167236	-0.34649104	-0.000336967	98.73914452
TLTPISAAYAR	6	20127454	1xTMT6plex [N-Term]	1.546	1.307	1.392	3.790976524	36.44	0	1392.804831	2	696.9067993	1.070640178	0.000745596	50.15315633
DLIVATIAVK	2	20127454	2xTMT6plex [N-Term; K10]			1.298	3.409823418	35.67	0	1500.976537	2	750.9921265	0.292915763	0.00021983	79.669186
ALFEEVPELLTEAEK	8	20127454	2xTMT6plex [N-Term; K15]				1.645405769	7.04	0	2176.215323	2	1088.610107	-1.095634345	-0.001192168	102.3768554
VCMVYDLYK	4	20127454	2xTMT6plex [N-Term; K9]; 1xCarbamidomethyl [C2			100	1.883647442	19.85	0	1648.884293	2	824.9457397	-0.054562841	-4.50E-05	74.23843228
LDFNLIR	6	20127454	1xTMT6plex [N-Term]	1.205	100		2.566287279	28.12	0	1119.67236	2	560.3394775	-0.608920876	-0.000340896	83.54711412
RAEISNAIDQYVTGTIGEDEDLIK	1	20127454	2xTMT6plex [N-Term; K24]				1.839547634		1	3108.646494	3	1036.88562	-1.346842893	-0.001395619	98.03551138
ANYWWLR	4	20127454	1xTMT6plex [N-Term]	1.398			2.457643509	20.96	0	1237.667944	2	619.3363647	-2.012428152	-0.001245359	81.71178336
DAGLAVR	6	20127454	1xTMT6plex [N-Term]	1.637	1.569	1.38	2.40094924	27.22	0	930.5569962	2	465.7821655	0.062580463	2.91E-05	37.93520288
YLAEFATGNDR	12	5803225	1xTMT6plex [N-Term]	1.356	1.371	1.527	3.690871954	51.3	0	1485.753523	2	743.3803711	-0.038971807	-2.90E-05	55.59741152
EAAENSLVAYK	16	5803225	2xTMT6plex [N-Term; K11]	1.291	1.319	1.335	5.010148525	62.27	0	1652.925958	2	826.9672852	0.80836198	0.000668081	54.37873964
DNLTLWTSDMQGDGEEQNK	17	5803225	2xTMT6plex [N-Term; K19]	1.377	1.299		3.802489519	42.37	0	2639.265931	3	880.4268799	0.059012029	5.19E-05	72.71241656
KEAAENSLVAYK	4	5803225	3xTMT6plex [N-Term; K1; K12]		1.021	0.662	2.872564316	18.89	1	2010.183852	3	670.7332153	0.616986398	0.000413419	43.39510788
AASDIAMTELPPTHPIR	29	5803225	1xTMT6plex [N-Term]	1.171	1.056	1.126	5.494042397	81.55	0	2049.100026	3	683.6984253	-9.420531378	-0.006434537	49.06843246
AASDIAMTELPPTHPIR	2	5803225	1xTMT6plex [N-Term]; 1xOxidation [M7]			1.103	5.422996998	50.31	0	2065.094941	3	683.6984253	-9.420531378	-0.006434537	49.06843246
DSTLIMQLLR	37	5803225	1xTMT6plex [N-Term]	1.27	1.271	1.324	4.110405922	66.2	0	1418.823852	2	709.9172974	2.443154983	0.001733203	90.31582375
VAGMDVELTVEER	28	5803225	1xTMT6plex [N-Term]			1.162	2.624695063	31.02	0	1676.872652	2	838.9393311	-0.75540061	-0.000633355	68.20944527
LICCDILDVLDK	11	5803225	2xTMT6plex [N-Term; K12]; 2xCarbamidomethyl [C3; C4	100	0.995	1.569	3.187869787	37.41	0	1935.069528	2	968.0370483	-1.39915919	-0.001353735	100.7076108
EALQDVEDENQ	18	5803225	1xTMT6plex [N-Term]	1.342	1.393	1.312	3.087606907	82.14	0	1518.712112	2	759.852417	-9.583564594	-0.007277338	44.5966014
DNLTLWTSDMQGDGEEQNK	3	5803225	2xTMT6plex [N-Term; K19]; 1xOxidation [M10			100	2.708369255	15.78	0	2655.260846	3	880.4268799	0.059012029	5.19E-05	72.71241656
EENKGGEDKLK	2	5803225	4xTMT6plex [N-Term; K4; K9; K11]	0.462			3.362110853	4.53	2	2163.279099	3	721.7644653	-0.1183869	-8.54E-05	36.41888436
IISSIEQK	12	5803225	2xTMT6plex [N-Term; K8]	1.52	1.525	1.474	3.677858591	43.66	0	1375.856087	2	688.4310913	-0.858522067	-0.000590601	57.79763906
NLLSVAYK	24	5803225	2xTMT6plex [N-Term; K8]	0.874	0.882	0.72	3.972506762	46.76	0	1365.850608	2	683.4294434	0.733725009	0.000501079	92.81997229
YDEMVESMK	12	5803225	2xTMT6plex [N-Term; K9]	1.249	1.077	2.598	3.540456057	27.88	0	1589.795537	2	803.3931885	-7.069245468	-0.005675863	42.84394887
QMVETELK	8	5803225	2xTMT6plex [N-Term; K8]	1.047	1.103	1.254	3.739144087	36.1	0	1435.823072	2	718.4155273	0.491402815	0.000352784	43.44981515
EDLVYQAK	6	5803225	2xTMT6plex [N-Term; K8]	0.672	0.629	0.716	3.69873929	47.92	0	1423.819702	2	712.4143677	1.234040771	0.000878526	49.02749394
DNLTLWTSDMQGDGEEQNKEALQDVEDENQ	5	5803225	2xTMT6plex [N-Term; K19]		100	1.284	1.302512527	18.15	1	3909.797269	4	978.2025757	-2.249870011	-0.002199134	77.43551109
DSTLIMQLLR	14	5803225	1xTMT6plex [N-Term]; 1xOxidation [M6]	1.102	100		1.706976891	33.93	0	1434.818767	2	709.9172974	2.443154983	0.001733203	90.31582375
YDEMVESMK	9	5803225	2xTMT6plex [N-Term; K9]; 1xOxidation [M]	1.064	1.077	1.105	2.562779665	35.76	0	1605.790452	2	803.3931885	-7.069245468	-0.005675863	42.84394887
EENKGGEDK	6	5803225	3xTMT6plex [N-Term; K4; K9]	1.293	100	2.219	1.574030161	2.37	1	1692.93714	3	564.9835815	-0.560316784	-0.000316194	22.84646291
KVAGMDVELTVEER	2	5803225	2xTMT6plex [N-Term; K1]; 1xOxidation [M5]			0.885	2.805423498	24.17	1	2050.125462	3	684.0455322	-1.667571266	-0.001139577	43.95032072
LICCDILDVLDKHLIPAANTGESK	2	5803225	3xTMT6plex [N-Term; K12; K24]; 2xCarbamidomethyl [C3; C4				1.340711474	4.23	1	3382.868144	4	846.4707642	-2.044877033	-0.001729387	100.0427723
VAGMDVELTVEER	11	5803225	1xTMT6plex [N-Term]; 1xOxidation [M4]			2.571	1.585651636	15.19	0	1692.867567	2	838.9393311	-0.75540061	-0.000633355	68.20944527
LAEQAER	6	5803225	1xTMT6plex [N-Term]	1.208	0.935	1.021	2.81289196	25.93	0	1045.583939	2	523.2957764	0.322273863	0.000168482	26.08720983
EIVHIQAGQCGNQIGTK	18	746815975	2xTMT6plex [N-Term; K17]; 1xCarbamidomethyl [C10	0.94	0.998	0.941	5.235685825	64.68	0	2311.25927	3	771.0914307	0.202756218	0.000156207	45.55101513
AALVDLEPGTMDSVR	8	746815975	1xTMT6plex [N-Term]	0.902	0.626	0.966	3.417700052	42.67	0	1802.951965	2	901.979126	-0.548970186	-0.000494883	72.0401687
EVDEQMLAIQSK	4	746815975	2xTMT6plex [N-Term; K12]	1.057	1.128		4.792182922	69.2	0	1849.014121	2	925.010376	-0.348930204	-0.000322588	66.35070637
VSDTVVEPYNATLSVHQLVENTDETYCIDNEALYDICFR	32	746815975	1xTMT6plex [N-Term]; 2xCarbamidomethyl [C27; C37	3.933	2.117		4.833796501	31.32	0	4822.270146	4	1206.324219	1.015870088	0.0012247	101.9591467
FWEVISDEHGIDPAGGYVGDSALQLER	4	746815975	1xTMT6plex [N-Term]	1.197			5.207048416	38.64	0	3189.569023	4	798.1470337	-0.852144712	-0.000679494	99.74096765
INVYYNESSSQK	8	746815975	2xTMT6plex [N-Term; K12]	1.177	1.187	1.143	3.840741396	45.11	0	1890.000913	2	945.5056763	1.673363775	0.00158133	51.45097032
FPGQLNADLR	24	746815975	1xTMT6plex [N-Term]	1.108	1.147	1.255	3.543502092	43.73	0	1359.758215	2	680.3824463	-0.440557643	-0.000299526	52.32976605
EVDEQMLAIQSK	2	746815975	2xTMT6plex [N-Term; K12]; 1xOxidation [M6		1.049		2.606637001	48.83	0	1865.009036	2	925.010376	-0.348930204	-0.000322588	66.35070637
LAVNMVPFPR	4	746815975	1xTMT6plex [N-Term]	1.652	1.913		3.099212646	32.08	0	1372.797243	2	686.9009399	-1.922976755	-0.001319929	73.50072
LHFFMPGFAPLTSR	4	746815975	1xTMT6plex [N-Term]	1.204		100	3.054291487	30.56	0	1849.998462	3	617.3383789	1.146778656	0.00070718	88.15653368
MASTFIGNSTAIQELFK	1	746815975	2xTMT6plex [N-Term; K17]	1.368			3.492440939		0	2316.267375	3	772.7601318	-0.661686213	-0.000510881	103.8703781
ISEQFSAMFR	2	746815975	1xTMT6plex [N-Term]	1.628			2.453250885	33.08	0	1444.745601	2	722.8766479	0.289240152	0.000208939	77.81599735
ALTVPELTQQMFDAR	14	746815975	1xTMT6plex [N-Term]	1.057	1.176	0.876	2.413100481	19.9	0	1949.036363	2	975.0213013	-0.532304557	-0.00051874	89.43239965
IMNTFSVMPSPK	6	746815975	2xTMT6plex [N-Term; K12]	100			1.970107198	21.19	0	1810.000719	2	905.5042725	0.303382131	0.000274561	75.80620276
LAVNMVPFPR	4	746815975	1xTMT6plex [N-Term]; 1xOxidation [M5]	1.084	100		1.945205092	20.38	0	1388.792158	2	686.9009399	-1.922976755	-0.001319929	73.50072
LTTPTYGDLNHLVSATMSGVTTSLR	5	746815975	1xTMT6plex [N-Term]	100		0.728	1.972118974	16.73	0	2864.502523	3	955.5058594	0.175082757	0.000167175	96.0950149
NMMAACDPR	2	746815975	1xTMT6plex [N-Term]; 1xCarbamidomethyl [C6	1.158			2.256533384	24.59	0	1294.590363	2	647.7979736	-1.307629365	-0.000846422	36.95700301
YLTVATVFR	4	746815975	1xTMT6plex [N-Term]	1.092	100		2.322813988	36.65	0	1298.766989	2	649.8864746	-1.013454501	-0.000658121	75.76769563
NSSYFVEWIPNNVK	3	746815975	2xTMT6plex [N-Term; K14]				2.179470062	13.24	0	2155.158811	2	1078.081421	-1.505992556	-0.001622827	78.89611646
IREEFPDR	2	746815975	1xTMT6plex [N-Term]	0.694			3.936471462	23.41	1	1290.700366	3	430.9062195	2.897184633	0.001246466	36.54519038
IIYGGSVTGATCK	20	226529917	2xTMT6plex [N-Term; K13]; 1xCarbamidomethyl [C12	0.972	1.003	0.995	5.660044193	70.63	0	1784.998077	2	892.9980469	-5.187551807	-0.004629885	48.74974371
HVFGESDELIGQK	20	226529917	2xTMT6plex [N-Term; K13]	0.938	1.058	0.987	4.506455421	61.69	0	1917.048198	2	959.0185547	-9.579860862	-0.009182527	49.05058232
ELASQPDVDGFLVGGASLKPEFVDIINAK	18	226529917	3xTMT6plex [N-Term; K19; K29]	2.343	1.211	100	3.075444221	24.82	0	3717.071789	4	930.0231323	-0.293008364	-0.000272283	101.3289826
DCGATWVVLGHSER	20	226529917	1xTMT6plex [N-Term]; 1xCarbamidomethyl [C2	0.996	0.874	0.979	5.55786562	59.2	0	1815.900933	2	908.4456177	-9.347541008	-0.008487104	47.77456239
VAHALAEGLGVIACIGEK	20	226529917	2xTMT6plex [N-Term; K18]; 1xCarbamidomethyl [C14	0.642	1.161	0.691	7.11563158	65.67	0	2266.299344	3	756.1033325	-1.72076183	-0.00129992	99.99246599
VVLAYEPVWAIGTGK	22	226529917	2xTMT6plex [N-Term; K15]	0.787	0.847	0.847	3.050664902	41.69	0	2061.214869	2	1031.110229	-0.81837447	-0.000843423	86.94345035
VPADTEVVCAPPTAYIDFAR	32	226529917	1xTMT6plex [N-Term]; 1xCarbamidomethyl [C9	0.753	0.838	0.787	5.620622635	62.82	0	2421.232163	3	807.7487183	-0.231788943	-0.000187072	94.35362895
SNVSDAVAQSTR	6	226529917	1xTMT6plex [N-Term]	1.022	1.039	1.328	4.331014633	60.6	0	1463.765151	2	732.385376	-1.144580382	-0.000837698	29.98735259
IAVAAQNCYK	9	226529917	2xTMT6plex [N-Term; K10]; 1xCarbamidomethyl [C8	1.035	0.93		3.84059906	49.61	0	1595.897969	2	798.4525757	-0.059090753	-4.72E-05	50.84222543
QSLGELIGTLNAAK	12	226529917	2xTMT6plex [N-Term; K14]			100	2.668451309	33.46	0	1873.115883	2	937.0606079	-1.03797619	-0.000972125	81.08597443
TATPQQAQEVHEK	6	226529917	2xTMT6plex [N-Term; K13]	0.893	1.036		5.17655611	38.36	0	1925.04926	3	642.3544312	-0.270228393	-0.000173401	32.32291191
KQSLGELIGTLNAAK	4	226529917	3xTMT6plex [N-Term; K1; K15]	100		1.302	2.790657043	15.91	1	2230.373778	3	744.1288452	-0.805253131	-0.000598672	73.96028047
VVFEQTK	10	226529917	2xTMT6plex [N-Term; K7]	0.848	0.829	0.739	2.837207317	36.23	0	1308.792759	2	654.9003296	0.476645125	0.000311915	44.45565329
EAGITEK	6	226529917	2xTMT6plex [N-Term; K7]	1.028	0.981	1.056	3.063572168	41.34	0	1205.714174	2	603.3603516	-0.620010128	-0.000373777	35.79556971
VIADNVK	9	226529917	2xTMT6plex [N-Term; K7]		1.014	0.954	3.28004837	36.01	0	1216.766544	2	608.8864746	-0.716169634	-0.000435706	36.69393317
AVEAAQVAFQR	18	153266822	1xTMT6plex [N-Term]	1.079	1.161	1.353	4.24002409	57.43	0	1418.795329	2	709.9016724	0.521108681	0.000369673	45.15016418
LECGGSAMEDK	8	153266822	2xTMT6plex [N-Term; K11]; 1xCarbamidomethyl [C3	1.715	1.465	1.795	4.6339674	77.18	0	1654.818064	2	827.9129028	0.281229745	0.000232692	48.39542539
EQICEVEEGDKPDVDK	9	153266822	3xTMT6plex [N-Term; K11; K16]; 1xCarbamidomethyl [C4	1.204	1.257		4.885926723	36.78	0	2577.332109	3	859.7825928	0.432967218	0.000371967	53.10351749
LAPALCCGNTMVLKPAEQTPLTALYLGSLIK	4	153266822	3xTMT6plex [N-Term; K14; K31]; 2xCarbamidomethyl [C6; C7	0.683	100		3.783215523	29.27	0	4031.271434	4	1008.571838	-1.466052205	-0.001477514	106.7813513
IFINNEWHESK	8	153266822	2xTMT6plex [N-Term; K11]	1.466	1.269	1.357	4.039561272	38.32	0	1875.016504	3	625.6767578	-0.417938988	-0.000261214	60.39833534
EEIFGPVQPILK	10	153266822	2xTMT6plex [N-Term; K12]	2.886	2.014	2.073	3.393475533	48.64	0	1828.098443	2	914.5512695	-1.739576724	-0.001590059	75.01365101
ELGEYALAEYTEVK	8	153266822	2xTMT6plex [N-Term; K14]	100	2.234		3.448260546	29.36	0	2073.115609	2	1037.060669	-0.746364249	-0.00077365	77.8120103
GLFIKPTVFSEVTDNMR	14	153266822	2xTMT6plex [N-Term; K5]	0.943	100	1.803	4.111605167	21.17	0	2412.336123	3	804.784668	1.379375046	0.001109172	86.8095632
LLHQLADLVER	22	153266822	1xTMT6plex [N-Term]	1.062	1.001	1.076	3.236455202	34.25	0	1535.910693	3	512.6414185	-0.64501726	-0.00033023	91.13558955
TIPTDDNVVCFTR	10	153266822	1xTMT6plex [N-Term]; 1xCarbamidomethyl [C10	1.364	1.457	1.517	4.34239006	60.49	0	1766.894451	2	883.9508057	-0.065573739	-5.79E-05	65.43858567
ILELIESGK	10	153266822	2xTMT6plex [N-Term; K9]	1.813	1.683	2.475	3.973679781	56.37	0	1459.913602	2	730.4593506	-1.491518486	-0.001088744	95.16190303
FATCNPSTR	4	153266822	1xTMT6plex [N-Term]; 1xCarbamidomethyl [C4	1.68	1.708		2.686816692	38.99	0	1282.641137	2	641.8237305	-0.742508932	-0.000476186	35.01538205
YFAGWADK	6	153266822	2xTMT6plex [N-Term; K8]	1.244	1.206	1.313	4.005849361	47.61	0	1415.772358	2	708.3850098	-6.791154452	-0.004807364	62.09491434
KFATCNPSTR	2	153266822	2xTMT6plex [N-Term; K1]; 1xCarbamidomethyl [C5		1.19		3.570556641	23.25	1	1639.899032	3	547.3050537	0.961176745	0.000525411	31.20357807
LECGGSAMEDK	12	153266822	2xTMT6plex [N-Term; K11]; 1xCarbamidomethyl [C3]; 1xOxidation [M8]	1.235	1.393	1.275	2.75581336	39.55	0	1670.812979	2	827.9129028	0.281229745	0.000232692	48.39542539
IAKEEIFGPVQPILK	9	153266822	3xTMT6plex [N-Term; K3; K15]	2.218	1.345	1.926	3.09239459	21.6	1	2369.477515	3	790.4968262	-0.671006314	-0.000529978	70.99694358
VTLELGGK	12	153266822	2xTMT6plex [N-Term; K8]	1.356	1.403	1.57	3.598306894	43.22	0	1274.808409	2	637.9078979	0.086670622	5.52E-05	53.78768884
ANSTDYGLTAAVFTK	4	153266822	2xTMT6plex [N-Term; K15]		100	100	2.897013187	19.43	0	2017.100627	2	1009.051636	-2.296556534	-0.002316193	69.35591415
GLFIKPTVFSEVTDNMR	15	153266822	2xTMT6plex [N-Term; K5]; 1xOxidation [M16]	1.128	0.307	0.701	2.556332111	12.46	0	2428.331038	3	804.784668	1.379375046	0.001109172	86.8095632
VFVEEQVYSEFVR	8	153266822	1xTMT6plex [N-Term]	100			2.096195698	17.34	0	1859.974081	2	930.4889526	-1.85600251	-0.001726058	97.39935158
SIEEVIK	14	153266822	2xTMT6plex [N-Term; K7]	1.209	1.233	1.446	3.06017518	38.37	0	1275.792424	2	638.3998413	-0.01439326	-9.18E-06	48.70245313
SIEEVIKR	4	153266822	2xTMT6plex [N-Term; K7]	0.661	0.179		2.736366987	18.23	1	1431.893535	3	477.9699707	1.273516033	0.000607846	46.19379332
SDSFENPVLQQHFR	4	4503841	1xTMT6plex [N-Term]	1.528		1.167	5.325106144	57.88	0	1932.97654	3	644.9976807	1.007980256	0.000649467	55.75266865
DIISIAEDEDLR	12	4503841	1xTMT6plex [N-Term]	1.438		0.974	4.472200871	64.92	0	1617.853297	2	809.4302979	0.013680551	1.11E-05	72.8211963
NLEALALDLMEPEQAVDLTLPK	4	4503841	2xTMT6plex [N-Term; K22]	1.262	100		3.237418175	30.27	0	2881.599667	3	961.2030029	-1.808638097	-0.001737257	108.4869519

Bhat et al,2017. Cigarette smoke and chewing tobacco alter expression of different sets of miRNAs in oral keratinocytes

Supplementary Table S2(A). List of all proteins and peptides identified in OKF6/TERT1 cells chronically treated with chewing tobacco using Sequest and Mascot search algorithms

Accession	Gene_Symbol	Description	Gene_ID	Refseq_Accession	Coverage	Proteins	#Unique_Peptides	#Peptides	#PSMS	#AAs	MW [kDa]	calc. pI	OKF6-STE-6M/OKF6P_R1	OKF6-STE-6M/OKF6P_R2	OKF6-STE-6M/OKF6P_R3	Median fold change	Status	P-value (6M)	Sequence	#PSMs	Protein Accessions	Modifications	OKF6-STE-6M/OKF6P_R1	OKF6-STE-6M/OKF6P_R2	OKF6-STE-6M/OKF6P_R3	Xcorr	Ionscore	# Missed Cleavages	Theo. MH+ [Da]	Charge	m_z	DeltaM [ppm]	Deltam/z [Da]	RT [min]
																			DTGIFLDLMHLK	6	4503841	2xTMT6plex [N-Term; K12]	1.2	1.683	100	2.742251158	34.47	0	1861.065762	3	621.0265503	-0.357107797	-0.000221534	108.6181748
																			IMLFTNEDNPHGNDSAK	4	4503841	2xTMT6plex [N-Term; K17]	1.41	100		2.811863899	31.16	0	2361.190915	3	787.7348633	-0.37220042	-0.000292945	53.17544313
																			DLLAVVFYGTEK	4	4503841	2xTMT6plex [N-Term; K12]	1.683	1.897		3.734088421	41.22	0	1813.051158	2	907.0286865	-0.585522984	-0.000530792	104.7405463
																			KPGGFDISLFYR	9	4503841	2xTMT6plex [N-Term; K1]	1.462	1.49	1.866	2.834405422	10.67	0	1858.062726	3	620.0250854	-1.088597449	-0.000674227	73.03008648
																			VEYSEEELK	8	4503841	2xTMT6plex [N-Term; K9]	1.299	1.403	1.271	4.194195747	56.87	0	1583.856875	2	792.4274292	-5.86741246	-0.004646571	45.10037214
																			IMATPEQVGK	7	4503841	2xTMT6plex [N-Term; K10]	1.139	1.595	1.419	4.180187702	54.49	0	1531.891821	2	766.4495239	-0.032318495	-2.48E-05	48.56961593
																			NIYVLQELDNPGAK	6	4503841	2xTMT6plex [N-Term; K14]	1.196	100	100	2.762798786	23.49	0	2032.147912	2	1016.575806	-1.760251729	-0.001788546	77.25992518
																			FDDPGLMLMGFKPLVLLK	6	4503841	3xTMT6plex [N-Term; K12; K18]	1.02	1.282	100	2.621001244	22.54	0	2721.605435	3	907.87323	-0.109431217	-9.93E-05	106.1872766
																			DSLIFLVDASK	12	4503841	2xTMT6plex [N-Term; K11]	100	2.488		2.536177158	41.16	0	1665.982744	2	833.4937134	-1.557040249	-0.001297001	81.83450611
																			IQVTPPGFQLVFLPFADDKR	8	4503841	2xTMT6plex [N-Term; K19]	0.99	1.108	2.547	3.106627464	13.06	1	2746.569628	3	916.1921997	-2.760734273	-0.002527516	98.53067152
																			NIPPYFVALVPQEEELDDQK	2	4503841	2xTMT6plex [N-Term; K20]		1.242		3.242182255	24.83	0	2802.496582	3	934.8377686	0.774278933	0.000723305	97.94963847
																			VHFEESSK	4	4503841	2xTMT6plex [N-Term; K8]	1.522	0.719		3.13398385	32.84	0	1420.783651	3	474.2663269	0.546834059	0.000258978	35.72090091
																			ELVYPPDYNPEGK	8	4503841	2xTMT6plex [N-Term; K13]	1.184	1.783	1.491	2.513352156	20.28	0	1979.052614	2	990.0291138	-0.84057944	-0.000831775	71.47248927
																			QELLEALTK	2	4503841	2xTMT6plex [N-Term; K9]		100		2.578855515	31.04	0	1502.919416	2	751.961731	-2.149400629	-0.001615188	74.6182631
																			AMFESQSEDELTPFDMSIQCIQSVYISK	9	4503841	2xTMT6plex [N-Term; K28]; 1xCarbamidomethyl [C20		100		2.264653444	8.8	0	3741.810207	3	1247.940063	-1.221184416	-0.001523147	116.5908196
																			LGSLVDEFK	4	4503841	2xTMT6plex [N-Term; K9]		100	100	2.412231445	27.98	0	1465.866652	2	733.4354858	-2.01710047	-0.0014784	76.66213202
																			IISSDRDLLAVVFYGTEK	2	4503841	2xTMT6plex [N-Term; K18]				1.870256066	5.88	1	2484.411396	3	828.8095703	1.111413152	0.000920402	100.5502664
																			ILELDQFK	8	4503841	2xTMT6plex [N-Term; K8]	1.37	1.397	100	2.696834326	20.55	0	1463.887387	2	732.4459229	-1.925173275	-0.001409118	73.94229071
																			QIILEKEETEELKR	2	4503841	3xTMT6plex [N-Term; K6; K13]		1.299		4.127470016	4.25	2	2445.453151	4	612.1174927	-2.048690597	-0.001252494	53.19417479
																			IMATPEQVGK	4	4503841	2xTMT6plex [N-Term; K10]; 1xOxidation [M2		1.519	1.679	2.86817646	47.98	0	1547.886736	2	766.4495239	-0.032318495	-2.48E-05	48.56961593
																			ALKPPPIK	3	4503841	3xTMT6plex [N-Term; K3; K8]	1.058			2.859332561	20.59	0	1551.060096	3	517.6921997	1.256953937	0.00064987	45.59258036
																			FTVPMLK	2	4503841	2xTMT6plex [N-Term; K7]	1.993			2.306458712	30.63	0	1293.800487	2	647.4031372	-1.150815719	-0.000744463	70.66810526
55770844	CTNNA1	catenin alpha-1 isoform 1	1495	NP_001894.2	35.54083885	1	27	27	96	906	100.009	6.29	0.9	1.03	1.14	1.02	Significant	0.0046																
																			AAAGEFADDPCSSVK	4	55770844	2xTMT6plex [N-Term; K15]; 1xCarbamidomethyl [C11	0.997	1.037		4.248071194	60.19	0	1982.989363	2	991.9981689	-0.15193696	-0.000150645	43.94259693
																			EELVAAVEDVR	2	55770844	1xTMT6plex [N-Term]	1.667			4.280869007	73.51	0	1458.800139	2	729.9041748	0.640052979	0.000466855	74.3279435
																			AHVLAASVEQATENFLEK	4	55770844	2xTMT6plex [N-Term; K18]		0.9		2.981950045	30.5	0	2415.328395	3	805.7815552	0.710929142	0.000572376	92.68551084
																			SDALNSAIDK	2	55770844	2xTMT6plex [N-Term; K10]	1.123			4.623106003	53.6	0	1491.841894	2	746.4249878	0.53984671	0.000402683	53.88282736
																			TIADHCPDSACK	2	55770844	2xTMT6plex [N-Term; K12]; 2xCarbamidomethyl [C6; C11		0.893		3.455406189	34.22	0	1832.903525	3	611.6390381	-0.525866296	-0.000321287	32.37745242
																			VIHVVTSEMDNYEPGVYTEK	6	55770844	2xTMT6plex [N-Term; K20]	0.692			2.970312834	19.48	0	2768.421703	3	923.4776001	-1.248356429	-0.001151992	77.27360551
																			NVPILYTASQACLQHPDVAAYK	3	55770844	2xTMT6plex [N-Term; K22]; 1xCarbamidomethyl [C12		100		1.691159725	12.25	0	2917.56462	3	973.1922607	-0.819383201	-0.000796868	64.51796633
																			IALYCHQLNICSK	5	55770844	2xTMT6plex [N-Term; K13]; 2xCarbamidomethyl [C5; C11	100		100	2.158142805	16.17	0	2078.129108	3	693.3808594	-0.521240473	-0.000361068	54.43848164
																			LAQENMDLFK	6	55770844	2xTMT6plex [N-Term; K10]	1.028	0.753	100	3.859780312	38.65	0	1666.923849	2	833.9650879	-0.569869314	-0.000474964	71.01029867
																			IAEQVASFQEEK	6	55770844	2xTMT6plex [N-Term; K12]	1.692	2.199	100	2.784959078	29.77	0	1837.01075	2	919.0087891	-0.243975165	-0.000224092	48.14586704
																			LLILADMADVYK	4	55770844	2xTMT6plex [N-Term; K12]	1.571	100		2.232734919	24.93	0	1823.075264	2	912.039978	-1.417854415	-0.001292428	104.3074161
																			LLSNTVMPR	2	55770844	1xTMT6plex [N-Term]	0.865			3.190629959	21.59	0	1259.734309	2	630.3711548	0.575073893	0.00036222	55.85336727
																			IVAECNAVR	2	55770844	1xTMT6plex [N-Term]; 1xCarbamidomethyl [C5	0.902			3.171100855	37.42	0	1260.693172	2	630.850769	0.863965921	0.000544598	36.52835573
																			SKLDAEVSK	6	55770844	3xTMT6plex [N-Term; K2; K9]	0.898	0.761		3.253600121	26.98	1	1664.019747	3	555.3450928	0.587517254	0.00032588	38.83932814
																			ESQFLKEELVAAVEDVR	3	55770844	2xTMT6plex [N-Term; K6]				2.337126017	13.1	1	2420.343711	3	807.4535522	0.988557233	0.000797549	97.92508823
																			ASYVASTK	4	55770844	2xTMT6plex [N-Term; K8]	0.861			3.287299633	44.59	0	1284.756373	2	642.8826294	1.252345656	0.00080448	35.50942765
																			ALKPEVDKLNIMAAK	3	55770844	4xTMT6plex [N-Term; K3; K8; K15]	0.01		100	2.001090527	0.46	1	2557.592117	4	640.1530151	-0.737468306	-0.000471536	61.25285181
																			NLMNAVVQTVK	2	55770844	2xTMT6plex [N-Term; K11]				1.890111208	6.78	0	1674.997683	2	838.0019531	-0.628717288	-0.00052655	76.83099741
																			EKQDETQTK	4	55770844	3xTMT6plex [N-Term; K2; K9]	0.811	0.899		2.813276052	17.34	1	1794.021204	3	598.6775513	-1.729818689	-0.001034444	27.38870443
																			QIIVDPLSFSEER	6	55770844	1xTMT6plex [N-Term]	100	0.788		1.992798686	17.44	0	1761.958431	2	881.480957	-2.15287003	-0.001896634	72.7662695
																			FTEQVEAAVEALSSDPAQPMDENEFIDASR	2	55770844	1xTMT6plex [N-Term]				1.106369615	0.03	0	3525.652888	3	1175.886963	-1.85858856	-0.002184246	107.2455361
																			ALLSAVTR	2	55770844	1xTMT6plex [N-Term]		1.416		2.81449914	37.5	0	1059.67236	2	530.3397217	-0.182613638	-9.68E-05	59.72545172
																			LNIMAAK	4	55770844	2xTMT6plex [N-Term; K7]	1.109	1.2		2.778009892	36.73	0	1218.764436	2	609.8855591	-0.487400124	-0.000297013	66.94649732
																			AVMDHVSDSFLETNVPLLVLIEAAK	2	55770844	2xTMT6plex [N-Term; K25]				1.370230794		0	3169.758293	3	1057.254761	-2.70182539	-0.002854711	107.6380063
																			CVIALQEK	2	55770844	2xTMT6plex [N-Term; K8]; 1xCarbamidomethyl [C1			0.955	2.712459087	2.47	0	1418.844143	2	709.9251099	-0.845430024	-0.000599767	47.88079081
																			LDAEVSK	2	55770844	2xTMT6plex [N-Term; K7]		1.479		2.646292448	35.98	0	1219.729824	2	610.3673096	-2.034548411	-0.0012408	41.01168259
																			LVYDGIR	6	55770844	1xTMT6plex [N-Term]	0.832	0.836	1.018	2.376921654	28.19	0	1064.630161	2	532.8185425	-0.331344231	-0.00017638	58.80135144
38202257	GANAB	neutral alpha-glucosidase AB isoform 2 precursoı	23193	NP_938148.1	37.81779661	1	27	27	127	944	106.807	6.14	1.01	0.99	0.94	0.98	Significant	0.00045																
																			AEKDEPGAWEETFK	8	38202257	3xTMT6plex [N-Term; K3; K14]	0.719	0.798	1.384	4.771863937	47.55	1	2324.237738	3	775.4155884	-2.377770221	-0.001842168	64.74072124
																			DPAEGDGAQPEETPR	4	38202257	1xTMT6plex [N-Term]	1.563	1.883		3.578310966	37.34	0	1797.845251	2	899.4263306	0.074068006	6.66E-05	31.29459474
																			VSQGSKDPAEGDGAQPEETPR	4	38202257	2xTMT6plex [N-Term; K6]	1.14	1.247		4.690413952	28.64	1	2613.315658	3	871.7762451	-0.564764702	-0.000491969	38.12865631
																			LYNLDVFQYELYNPMALYGSVPVLLAHNPHR	2	38202257	1xTMT6plex [N-Term]; 1xOxidation [M15]			100	3.393750668	10.04	0	3892.009367	4	973.7583618	0.578232325	0.000562621	106.5429625
																			KLVAIVDPHIK	2	38202257	3xTMT6plex [N-Term; K1; K11]	0.937			4.924341679	21.26	1	1920.261315	3	640.7588501	0.355145919	0.000227324	58.13469245
																			DENSVELTMAEGPYK	4	38202257	2xTMT6plex [N-Term; K15]				3.355568647	23.16	0	2141.083656	2	1071.0448	-0.62280641	-0.00066674	57.52905283
																			LSFQHDPETSVLVLR	5	38202257	1xTMT6plex [N-Term]	1.423	0.01	0.01	3.535628796	25.43	0	1970.090842	3	657.368103	-0.551222282	-0.000361986	76.79848256
																			DEADVLEVDQGFDDHNLPCDVIWLDIEHADGK	2	38202257	2xTMT6plex [N-Term; K32]; 1xCarbamidomethyl [C19		100		1.948976874	15.71	0	4137.975174	4	1035.246216	-2.934067566	-0.003035275	98.13752263
																			FGAVWTGDNTAEWDHLK	2	38202257	2xTMT6plex [N-Term; K17]	100			1.996650696	19.8	0	2405.229016	3	802.4139404	-0.726538274	-0.000582497	90.52579088
																			MMDYLQGSGETPQTDVR	4	38202257	1xTMT6plex [N-Term]	1.222	100		3.126886845	28.12	0	2157.01537	2	1079.009766	-1.444213168	-0.001557595	70.52048522
																			TCEESSFCK	8	38202257	2xTMT6plex [N-Term; K9]; 2xCarbamidomethyl [C2; C8	1.159	1.426	1.382	3.545389652	51.91	0	1605.7653	2	803.3847046	-1.972642149	-0.0015838	37.70915895
																			GLLEFEHQR	14	38202257	1xTMT6plex [N-Term]	0.878	0.884	0.901	3.842657328	35.05	0	1357.742565	3	453.2522888	-0.185414325	-8.39E-05	44.40814208
																			LVAIVDPHIK	6	38202257	2xTMT6plex [N-Term; K10]	1.065	1.634	1.719	2.315209389	27.49	0	1563.00342	3	521.6726074	-0.096669254	-5.04E-05	46.63007004
																			AHAHLDTGR	4	38202257	1xTMT6plex [N-Term]	0.854	1.06		2.972501278	33.85	0	1206.654084	3	402.8890991	-1.110713282	-0.000446749	15.44246593
																			FSFSGNTLVSSSADPEGHFETPIWIER	3	38202257	1xTMT6plex [N-Term]				2.181025743	5.89	0	3239.584673	3	1080.531616	-1.35126494	-0.001459179	91.24267477
																			SIRPGLSPYR	2	38202257	1xTMT6plex [N-Term]			0.998	3.992541313	28.31	0	1374.8055	3	458.9400635	0.100101205	4.59E-05	39.44223717
																			DVHNIYGLYVHMATADGLR	4	38202257	1xTMT6plex [N-Term]				1.651126385	14.72	0	2374.217515	3	792.0765381	-1.03380679	-0.000818161	69.14449392
																			EPWLLPSQHNDIIR	4	38202257	1xTMT6plex [N-Term]	1.056			2.498657465	18.61	0	1947.064961	3	649.6937866	0.947669091	0.000615058	66.30474412
																			DGDKPEETQGK	2	38202257	3xTMT6plex [N-Term; K4; K11]	1.381			2.295668364	8.26	0	1891.017582	3	631.0175171	0.219900194	0.000138613	32.27019495
																			VTEGGEPYR	2	38202257	1xTMT6plex [N-Term]			1.42	2.539736986	21.19	0	1236.642182	2	618.8238525	-1.418164365	-0.000876881	34.24543585
																			NLGLYVK	10	38202257	2xTMT6plex [N-Term; K7]	100	100	0.742	2.822049094	26.73	0	1264.80293	2	632.9049072	-0.309690044	-0.000195848	52.75854522
																			VVIIGAGKPAAVVLQTK	2	38202257	3xTMT6plex [N-Term; K8; K17]				1.109492064	1.1	0	2351.535699	3	784.517395	0.822028173	0.000644343	78.0594409
																			LDLLEDR	5	38202257	1xTMT6plex [N-Term]	1.475	1.151	1.076	2.832198143	29.72	0	1102.630555	2	551.8198242	1.647748189	0.000908429	63.99268764
																			AFFAGSQR	4	38202257	1xTMT6plex [N-Term]	0.827	1.013		2.326170683	19.2	0	1112.605009	2	556.8064575	0.565734522	0.00031472	42.17561708
																			YFTWDPSR	10	38202257	1xTMT6plex [N-Term]	1.28	100		2.758146048	26.85	0	1300.652353	2	650.8300781	0.404881442	0.000263305	70.21380586
																			VPDVLVADPPIAR	6	38202257	1xTMT6plex [N-Term]		0.673	100	1.920802355	11.19	0	1590.941659	2	795.9734497	-1.279598109	-0.001017883	61.58269683
																			IDELEPR	4	38202257	1xTMT6plex [N-Term]	1.486	1.558		2.672881842	37.36	0	1100.614905	2	550.8108521	-0.43377428	-0.000238709	51.76815437
14141152	HNRNPM	heterogeneous nuclear ribonucleoprotein M isoform ε	4670	NP_005959.2	38.21917808	1	20	20	119	730	77.464	8.7	1.23	1.25	1.12	1.2	Significant	0.0011																
																			GDFFPPERPQQLPHGLGGIGMGLGPGGQPIDANHLNK	4	14141152	2xTMT6plex [N-Term; K37]	1.34			6.124032497	13.4	0	4277.244235	5	856.2546387	-0.034529388	-2.95E-05	74.48984879
																			MGAGMGFGLER	2	14141152	1xTMT6plex [N-Term]	1.116			3.356778145	50.11	0	1354.680893	2	677.8436279	-0.674314246	-0.00045674	69.13083512
																			GIGMGNIGPAGMGMEGIGFGINK	9	14141152	2xTMT6plex [N-Term; K23]	0.712	1.078	100	3.861959219	52	0	2636.37629	3	879.4627075	-1.032019373	-0.00090693	86.00730488
																			MVPAGMGAGLER	4	14141152	1xTMT6plex [N-Term]	1.151	1.101		3.997099876	57.65	0	1417.749307	2	709.378479	0.264361133	0.000187399	61.83276148
																			GNFGGSFAGSFGGAGGHAPGVAR	18	14141152	1xTMT6plex [N-Term]	1.023		1.946	7.143595219	87.78	0	2264.115828	3	755.3753052	-1.972236845	-0.001488458	62.1485259
																			MGPLGLDHMASSIER	6	14141152	1xTMT6plex [N-Term]	2.21			3.782664299	26.43	0	1842.940355	3	614.9862061	2.01325781	0.001236771	64.02200406
																			DKFNECGHVLYADIK	8	14141152	3xTMT6plex [N-Term; K2; K15]; 1xCarbamidomethyl [C6	1.052	0.986		4.284220219	23.18	1	2496.352391	3	832.7886963	-0.342765234	-0.000285221	57.79617481
																			MGPAMGPALGAGIER	2	14141152	1xTMT6plex [N-Term]	1.466			3.161349297	33.09	0	1656.876298	2	828.9417114	-0.091538782	-7.58E-05	59.9319035
																			GIGTVTFEQSIEAVQAISMFNGQLLFDRPMHVK	3	14141152	2xTMT6plex [N-Term; K33]		0.314	100	3.680610657	4.06	0	4122.192048	4	1031.302368	-1.068565874	-0.001101208	110.0653259
																			AFITNIPFDVK	6	14141152	2xTMT6plex [N-Term; K11]	1.177	1.227	1.173	3.143502474	26.07	0	1723.019464	2	862.0119629	-1.633683663	-0.001407434	81.96556863
																			FEPYANPTK	4	14141152	2xTMT6plex [N-Term; K9]	1.384		1.335	4.128147602	42.39	0	1524.846251	2	762.9221191	-6.091833026	-0.004644554	45.73830334
																			GCAVVEFK	16	14141152	2xTMT6plex [N-Term; K8]; 1xCarbamidomethyl [C2	1.143	1.294	1.372	3.729801416	49.82	0	1367.775729	2	684.3915405	0.055319513	3.78E-05	58.33341544
																			ADILEDKDGK	9	14141152	3xTMT6plex [N-Term; K7; K10]	1.291	1.386	1.034	2.766956568	26.25	1	1791.04669	2	896.0281372	1.288304808	0.001153707	48.44795957
																			MGANSLER	4	14141152	1xTMT6plex [N-Term]	1.121	0.943		2.309100866	28.83	0	1106.582559	2	553.7944946	-0.764789017	-0.000423151	38.05462226
																			MVPAGMGAGLER	2	14141152	1xTMT6plex [N-Term]; 1xOxidation [M1]		1.267		2.635988235	45.3	0	1433.744222	2	709.378479	0.264361133	0.000187399	61.83276148
																			ADILEDK	8	14141152	2xTMT6plex [N-Term; K7]	1.588	1.569	0.93	2.824042797	32.71	0	1261.740389	2	631.3745117	1.07632086	0.000679019	62.03198637
																			VGSEIER	4	14141152	1xTMT6plex [N-Term]	1.717	2.371		2.447604656	36.98	0	1018.57304	2	509.7897949	-0.713641753	-0.000363448	32.79422983
																			EVFSMAGVVVR	2	14141152	1xTMT6plex [N-Term]				1.694654346	17.54	0	1422.797637	2	711.9020386	-0.58789215	-0.000418226	68.65418237
																			NLPFDFTWK	4	14141152	2xTMT6plex [N-Term; K9]	100		100	1.600377917	12.47	0	1625.909185	2	813.4558716	-2.902275219	-0.002359418	88.4721723
																			ACQIFVR	2	14141152	1xTMT6plex [N-Term]; 1xCarbamidomethyl [C2	1.582			2.495429993	23.75	0	1122.629116	2	561.8182983	0.182152489	0.000102245	43.67158172
																			RGEIIAK	2	14141152	2xTMT6plex [N-Term; K7]		1.27		2.79196167	12.94	1	1244.809078	3	415.6072388	-1.537963886	-0.000638157	36.20533776
109389365	G6PD	glucose-6-phosphate 1-dehydrogenase isoform ε	2539	NP_000393.4	46.23853211	1	21	21	135	545	62.429	8.05	1.93	2.22	1.88	2.01	Significant	0.0028																
																			NIHESCMSQIGWNR	8	109389365	1xTMT6plex [N-Term]; 1xCarbamidomethyl [C6	2.122	2.266	100	5.528884411	49.17	0	1960.931915	3	654.3158569	0.562029091	0.000367367	43.61826568
																			NIHESCMSQIGWNR	8	109389365	1xTMT6plex [N-Term]; 1xCarbamidomethyl [C6]; 1xOxidation [M7]	1.919	2.088		4.313572407	47.8	0	1976.92683	3	654.3158569	0.562029091	0.000367367	43.61826568
																			NSYVAGQYDDAASYQR	10	109389365	1xTMT6plex [N-Term]		2.156	1.598	3.322309971	27.42	0	2036.951113	2	1018.979126	-0.067751687	-6.90E-05	49.04074002
																			LQFHDVAGDIFHQQCK	10	109389365	2xTMT6plex [N-Term; K16]; 1xCarbamidomethyl [C15	1.547	1.846	3.618	4.676468849	29.93	0	2401.248705	3	801.0875244	-0.285463064	-0.000228489	58.12368529
																			LNSHMNALHLGSQANR	4	109389365	1xTMT6plex [N-Term]	2.118	2.899		6.280424118	39.27	0	1992.039492	4	498.7652283	-0.205068255	-0.000102126	40.45967424
																			EMVQNLMVLR	18	109389365	1xTMT6plex [N-Term]	1.941	100		3.484179258	63.06	0	1461.811907	2	731.4095459	-0.062698302	-4.58E-05	88.82990873
																			GGYFDEFGIIR	10	109389365	1xTMT6plex [N-Term]	0.922	100		3.154834986	56.2	0	1502.784095	2	751.8946533	-1.37434205	-0.00103267	73.89948291
																			DVMQNHLLQMLCLVAMEKPASTNSDDVR	4	109389365	2xTMT6plex [N-Term; K18]; 1xCarbamidomethyl [C12	100			2.742806673	9.88	0	3673.857448	4	919.217041	-3.025228294	-0.002778564	92.82184811
																			GPTEADELMK	6	109389365	2xTMT6plex [N-Term; K10]	1.158	1.124	100	3.678104162	48.17	0	1548.834365	2	774.9214478	0.809316891	0.000626749	46.09762963
																			LILDVFCGSQMHFVR	7	109389365	1xTMT6plex [N-Term]; 1xCarbamidomethyl [C7	1.672	100	100	2.300897121	21.58	0	2051.076789	3	689.696106	0.996980942	0.000686944	91.68321045
																			LILDVFCGSQMHFVR	2	109389365	1xTMT6plex [N-Term]; 1xCarbamidomethyl [C7]; 1xOxidation [M11]	1.562			2.979268789	46.85	0	2067.071704	3	689.696106	0.996980942	0.000686944	91.68321045
																			KPGMFFNPEESELDLTYGNR	7	109389365	2xTMT6plex [N-Term; K1]	100	100		1.918277502	4.12	0	2802.417286	3	940.1421509	-0.10714305	-0.000100658	74.42096032
																			DNIACVILTFK	5	109389365	2xTMT6plex [N-Term; K11]; 1xCarbamidomethyl [C5				1.964254498	19.08	0	1752.012999	2	876.5093384	-0.912431694	-0.000799296	92.80616994
																			VGFQYEGTYK	4	109389365	2xTMT6plex [N-Term; K10]		100	100	2.691417217	19.52	0	1649.893929	2	825.447876	-3.305574242	-0.002726923	52.72896332
																			IIVEKPFGR	4	109389365	2xTMT6plex [N-Term; K5]	2.223	1.764		3.160228729	10.38	0	1516.961555	3	506.3249207	-0.887706814	-0.000448872	55.10359911
																			KQSEPFFK	2	109389365	3xTMT6plex [N-Term; K1; K8]			1.791	3.75718832	24.08	1	1698.019354	3	566.6780396	0.124752609	7.06E-05	51.58837515
																			KPGMFFNPEESELDLTYGNR	2	109389365	2xTMT6plex [N-Term; K1]; 1xOxidation [M4]			100	2.111112595	6.98	0	2818.412201	3	940.1421509	-0.10714305	-0.000100658	74.42096032
																			EPFGTEGR	2	109389365	1xTMT6plex [N-Term]	1.827			3.016294718	30.65	0	1121.578854	2	561.293335	0.481064593	0.000269776	35.24658885
																			DGLLPENTFIVGYAR	4	109389365	1xTMT6plex [N-Term]		100		1.330797195	1.53	0	1894.027179	2	947.515686	-1.628012376	-0.00154175	80.07415003
																			LSNHISSLFR	6	109389365	1xTMT6plex [N-Term]	100	100	1.72	2.17204833	15.36	0	1402.800414	3	468.2714539	-0.431877356	-0.000201946	51.77837405
																			LEDFFAR	2	109389365	1xTMT6plex [N-Term]	2.732			2.317293882	23.59	0	1126.609426	2	563.8084106	0.105652465	5.95E-05	83.43701225
																			WDGVPFILR	2	109389365	1xTMT6plex [N-Term]	100			1.907530546	16.2	0	1331.767323	2	666.3868408	-0.689451797	-0.000459095	97.71980994
																			LTVADIR	6	109389365	1xTMT6plex [N-Term]	1.982	1.983		2.804454088	45.32	0	1016.630161	2	508.8184509	-0.527098793	-0.000267932	58.52596511
																			LKLEDFFAR	2	109389365	2xTMT6plex [N-Term; K2]		1.561		2.031149864	12.12	1	1596.951385	3	532.9877319	-1.717008574	-0.000913993	75.61910696
4557305	ALDOA	fructose-bisphosphate aldolase A isoform 1	226	NP_000025.1	68.95604396	1	17	21	162	364	39.395	8.09	0.83	0.832	0.831	0.83	Non-Significant	1																
																			TVPPAVTGITFLSGGQSEEEASINLNAINK	4	4557305	2xTMT6plex [N-Term; K30]	0.01		0.603	3.812372684	25.29	0	3515.899748	4	879.7284546	-2.207079604	-0.001939968	117.757393
																			ALANSLACQGK	6	4557305	2xTMT6plex [N-Term; K11]; 1xCarbamidomethyl [C8	0.679	0.635	0.821	4.792935371	77.89	0	1590.903783	2	795.9555664	0.046208011	3.68E-05	49.23326401
																			ALSDHHIYLEGTLLKPNMVTPGHACTQK	2	4557305	3xTMT6plex [N-Term; K15; K28]; 1xCarbamidomethyl [C25	0.514			3.845576763	11.54	0	3819.065281	5	764.6195068	0.824062815	0.00062943	61.90065514
																			LQSIGTENTEENR	8	4557305	1xTMT6plex [N-Term]	0.855	0.884		4.29502058	48.71	0	1719.871072	2	860.4379883	-1.379328682	-0.001186134	42.5585721
																			GILAADESTGSIAK	14	4557305	2xTMT6plex [N-Term; K14]	0.702	0.713	0.562	5.593325138	89.77	0	1791.0264	2	896.0164795	-0.400544417	-0.000358693	59.94112383
																			FSHEEIAMATVTALR	6	4557305	1xTMT6plex [N-Term]; 1xOxidation [M8]	1.12	0.643		4.346595287	32.86	0	1921.005064	2	953.0090942	0.400615484	0.000381588	67.58931575
																			FSHEEIAMATVTALR	20	4557305	1xTMT6plex [N-Term]	0.758	0.853	0.848	6.291340828	46.18	0	1905.010149	2	953.0090942	0.400615484	0.000381588	67.58931575
																			IGEHTPSALAIMENANVLAR	6	4557305	1xTMT6plex [N-Term]	1.529	2.323	1.144	4.153810501	21.59	0	2336.25938	3	779.4280396	4.359589706	0.003395044	78.46607242
																			LQSIGTENTEENRR	5	4557305	1xTMT6plex [N-Term]		0.331	1.135	3.739622116	27.23	1	1875.972183	3	625.9956665	0.140242868	8.77E-05	36.69668555
																			QLLLTADDR	14	4557305	1xTMT6plex [N-Term]	0.774	0.804		4.037846565	69.24	0	1273.731332	2	637.3696289	0.510031028	0.000324821	54.56934034
																			YTPSGQAGAAASESLFVSNHAY	4	4557305	1xTMT6plex [N-Term]	0.624			3.619152308	28.63	0	2457.188383	2	1229.096802	-0.836893251	-0.001028202	66.55135386
																			VQEIQVVK	2	94557305	2xTMT6plex [N-Term; K8]			1.04	3.326373816	42.9	0	1400.887722	2	700.9463501	-1.64047029	-0.001149057	46.22489505
																			ALQASALK	12	4557305	2xTMT6plex [N-Term; K8]	0.847	0.804	0.845	3.448075533	52.25	0	1259.808743	2	630.407959	-0.080814846	-5.09E-05	48.67353601
																			ADDGRPFPQVIK	6	4557305	2xTMT6plex [N-Term; K12]	1.073	0.969		3.078594446	10.71	0	1801.037239	3	601.0177002	0.726273677	0.000436015	55.12901731
																			PYQYPALTPEQK	1	4557305	2xTMT6plex [N-Term; K12]			1.124		27.88	0	1893.052221	2	947.0297852	0.038605644	3.65E-05	59.79515703
																			AAQEEYVK	6	4557305	2xTMT6plex [N-Term; K8]	0.582	0.625	0.557	3.731850386	44.33	0	1395.788402	2	698.3968506	-1.416409624	-0.000988504	34.02038283
																			VNPCIGGVILFHETLYQK	2	4557305	2xTMT6plex [N-Term; K18]; 1xCarbamidomethyl [C4	0.418			2.933045864	23.98	0	2546.420522	3	849.4788818	0.616751161	0.000523503	94.16407691
																			VLAAVYK	21	4557305	2xTMT6plex [N-Term; K7]	0.938	0.979	0.772	3.51181078	34.75	0	1221.797116	2	611.4023438	0.241431246	0.00014749	49.68648777
																			KDGADFAK	6	4557305	3xTMT6plex [N-Term; K1; K8]	0.882			2.988016844	22.67	1	1538.914554	2	769.961853	1.21855673	0.000937627	37.59183431
																			AAQEEYVKR	4	4557305	2xTMT6plex [N-Term; K8]	0.161	0.188		3.16666913	18.93	1	1551.889513	3	517.9675293	-0.952309661	-0.000492626	31.92093002
																			CQYVTEK	4	4557305	2xTMT6plex [N-Term; K7]; 1xCarbamidomethyl [C1	0.923	1.003		3.47413826	36.42	0	1385.749908	2	693.3788452	0.365072864	0.00025295	33.73137574
																			GGVVGIK	7	4557305	2xTMT6plex [N-Term; K7]	0.894	0.967		2.596080065	39.91	0	1087.723951	2	544.3649902	-1.146569632	-0.000623576	44.00198306
																			LDDSLLYLR	2	94557305	1xTMT6plex [N-Term]				1.540431023	21.35	0	1336.767383	2	668.8876953	0.547069752	0.000365653	98.54194421
																			DGADFAK	4	4557305	2xTMT6plex [N-Term; K7]	1.173	1.189		2.90922761	30.44	0	1181.656659	2	591.3322754	0.520406029	0.000307471	44.98278501
																			DALPEYSTFDVNMKPVVQEPNQK	1	94557305	3xTMT6plex [N-Term; K14; K23]				1.014983773		0	3336.775289	3	1112.930298	0.315015126	0.000350378	65.053069
																			FLSPEFIPR	2	94557305	1xTMT6plex [N-Term]	0.01			2.692970514	23.67	0	1334.766989	2	667.8878174	1.025876154	0.000684653	77.52106661
15991831	HK1	hexokinase-1 isoform HKI-ta/tb	3098	NP_277033.1	29.31596091	1	21	24	147	921	102.672	6.76	0.72	0.73	0.71	0.72	Significant	0.000064																
																			ASGVEGADVVK	8	15991831	2xTMT6plex [N-Term; K11]	0.732	0.704	0.778	4.419379711	69.23	0	1489.862629	2	745.4351807	0.305832307	0.000227824	41.83150236
																			GAAMVTAVAYR	12	15991831	1xTMT6plex [N-Term]	0.518	0.647	0.715	4.195102692	57.33	0	1338.740122	2	669.8765259	4.222625227	0.002826499	34.80266121
																			TTVGVDGSLYK	4	15991831	2xTMT6plex [N-Term; K11]	0.751	0.698		4.351520538	63.11	0	1597.920144	2	799.4637451	0.043628197	3.49E-05	58.25025367
																			GAALITAVGVR	12	15991831	1xTMT6plex [N-Term]	0.957	0.593	1.064	4.157474995	58.51	0	1256.788787	2	628.8984375	0.645677307	0.00040574	60.16330628
																			AAQLCGAGMAAVVDK	6	15991831	2xTMT6plex [N-Term; K15]; 1xCarbamidomethyl [C5	1.024	100	100	3.515939236	51.44	0	1920.044709	2	960.5258179	-0.18246857	-0.000175174	63.1494049
																			ATDCVGHDVVTLLR	10	15991831	1xTMT6plex [N-Term]; 1xCarbamidomethyl [C4	0.805	1.085	0.696	5.152123451	39.26	0	1784.952634	3	595.6551514	-0.971061307	-0.000577766	61.5715757
																			HIDLVEGDEGR	4	15991831	1xTMT6plex [N-Term]	0.847	0.791		3.986247063	50.03	0	1468.759337	2	734.8826294	-0.922500306	-0.000677465	42.90303969
																			FLLSESGSGK	4	15991831	2xTMT6plex [N-Term; K10]	0.683	0.651		3.778872967	50.35	0	1482.856815	2	741.9317017	-0.464461356	-0.000344365	65.75823439
																			GDFIALDLGGSSFR	10	15991831	1xTMT6plex [N-Term]	0.48	0.727		2.601686716	29.93	0	1683.890351	2	842.4481812	-0.751548527	-0.000632763	82.6808917
																			KLPVGFTFSFPCQQSK	4	15991831	3xTMT6plex [N-Term; K1; K16]; 1xCarbamidomethyl [C12	0.362	0.149		4.176822662	25.19	1	2558.440812	3	853.4837646	-1.591471129	-0.001357228	77.7828457
																			MVSGMYLGELVR	4	15991831	1xTMT6plex [N-Term]	0.47	100		3.014086962	36.42	0	1583.848686	2	792.4273682	-0.774380713	-0.000613251	81.75507933
																			AILQQLGLNSTCDDSILVK	3	15991831	2xTMT6plex [N-Term; K19]; 1xCarbamidomethyl [C12				1.944704652	3.21	0	2546.426395	2	1273.717163	0.257098291	0.000327341	93.51011469
																			DFNPTATVK	4	15991831	2xTMT6plex [N-Term; K9]	0.68	0.742		3.426305532	41.37	0	1450.830601	2	725.9185181	-0.579803863	-0.000420599	54.69832405
																			MISGMYLGEIVR	5	15991831	1xTMT6plex [N-Term]				1.596067429	11.32	0	1597.864336	2	799.4351196	-0.859667593	-0.000686816	101.3698113
																			IDEAILITWTK	6	15991831	2xTMT6plex [N-Term; K11]				2.059892893	23.19	0	1761.056243	2	881.0314941	-0.301948761	-0.000265874	102.5432939
																			AEMELGLR	10	15991831	1xTMT6plex [N-Term]	0.55	0.635	0.607	3.266788244	36.86	0	1147.63426	2	574.3153687	-9.410197732	-0.005399733	47.56114506
																			GQISETLK	6	15991831	2xTMT6plex [N-Term; K8]	0.868	0.898	0.832	3.428822041	44.8	0	1333.809137	2	667.4082031	-0.005540523	-3.70E-06	43.43680706
																			NILIDFTK	5	15991831	2xTMT6plex [N-Term; K8]			100	2.300944805	25.4	0	1421.876823	2	711.440979	-1.50594774	-0.001070636	77.93812823
																			TVCGVVSR	10	15991831	1xTMT6plex [N-Term]; 1xCarbamidomethyl [C3	0.761	0.763	0.83	3.046337843	29.99	0	1106.618945	2	553.8128662	-0.441857721	-0.000244484	31.12149101
																			SIPDGSEK	2	15991831	2xTMT6plex [N-Term; K8]			0.652	2.867609262	24.85	0	1290.730552	2	645.8687744	-0.217041329	-0.000140071	37.51176023
																			DMLLEVK	6	15991831	2xTMT6plex [N-Term; K7]	0.754	0.608		3.119456768	43.51	0	1305.785231	2	653.3959351	-0.487846544	-0.000318511	71.57088103
																			LSDETLIDIMTR	4	15991831	1xTMT6plex [N-Term]				1.731353879	22.16	0	1635.882489	2	818.4432983	-1.936918045	-0.001584285	93.59647854
																			SANLVAATLGAILNR	2	15991831	1xTMT6plex [N-Term]				1.062840104	9.28	0	1713.022034	2	857.0131836	-1.718303934	-0.001471746	103.102799
																			FLSQIESDR	3	15991831	1xTMT6plex [N-Term]				1.485193729	18.47	0	1323.710596	2	662.3592529	0.478314049	0.000316575	62.95948961
																			GAAMVTAVAYR	3	15991831	1xTMT6plex [N-Term]; 1xOxidation [M4]		0.786	0.809	2.09624505	25.19	0	1354.735037	2	669.8765259	4.222625227	0.002826499	34.80266121
228008291	SYNCRIP	heterogeneous nuclear ribonucleoprotein Q isoform 1	10492	NP_006363.4	35.79454254	1	14	20	120	623	69.56	8.59	1.57	1.46	2.21	1.75	Significant	0.017																
																			TGYTLDVTTGQR	6	228008291	1xTMT6plex [N-Term]	2.364	2.396	2.876	4.746033669	59.21	0	1540.816852	2	770.9112549	-1.050607911	-0.000809397	41.79359486
																			VTEGLTDVILYHQPDDK	12	228008291	2xTMT6plex [N-Term; K17]	1.397	100	100	3.019670725	30.52	0	2401.301512	3	801.1063232	1.209671734	0.000968262	83.88983101
																			EFNEDGALAVLQQFK	9	228008291	2xTMT6plex [N-Term; K15]	2.068		100	3.273718596	38.56	0	2167.17994	3	723.0661011	1.757963234	0.001269941	99.11163634
																			AIEALKEFNEDGALAVLQQFK	4	228008291	3xTMT6plex [N-Term; K6; K21]	0.01	0.33		4.795571804	24.42	1	3021.722783	3	1007.911621	-0.818555813	-0.000824483	108.8461342
																			DLEGENIEIVFAKPPDQK	4	228008291	3xTMT6plex [N-Term; K13; K18]	100	100		3.912566423	27.21	0	2729.532858	3	910.5167847	1.078277515	0.000981065	85.31037533
																			VWGNVGTVEWADPIEDPDPEVMAK	4	228008291	2xTMT6plex [N-Term; K24]	100	100		3.10584569	22.73	0	3112.570158	3	1038.19458	-0.311972285	-0.000323679	101.5340724
																			GFCFLEYEDHK	12	228008291	2xTMT6plex [N-Term; K11]; 1xCarbamidomethyl [C3	1.31	1.299	1.309	3.861279726	35.59	0	1902.946041	3	634.9869995	0.212284084	0.000134655	93.87060383
																			DLFEDELVPLFEK	6	228008291	2xTMT6plex [N-Term; K13]		100		3.311164618	41.74	0	2052.13053	2	1026.567871	-1.00618965	-0.001032416	102.2178786
																			NLANTVTEEILEK	4	228008291	2xTMT6plex [N-Term; K13]		100	100	2.51018548	27.36	0	1932.105378	2	966.5562134	-0.118079578	-0.000114071	82.18365394
																			SAFLCGVMK	6	228008291	2xTMT6plex [N-Term; K9]; 1xCarbamidomethyl [C5	1.383	1.467		3.858357191	51.79	0	1470.821299	2	735.9140625	-0.306196273	-0.00022518	75.28522302
																			LKDYAFIHFDER	8	228008291	2xTMT6plex [N-Term; K2]	1.346	1.092		3.720880032	24.03	1	2012.100568	4	503.7805481	-0.102011396	-5.13E-05	71.79942757
																			VAEKLDEIYVAGLVAHSDLDER	2	228008291	2xTMT6plex [N-Term; K4]		100		3.043519974	18.12	1	2900.576958	4	725.8989258	-1.063497037	-0.000771189	86.47830096
																			EAAQEAVK	10	228008291	2xTMT6plex [N-Term; K8]	1.542	1.529	1.762	3.661378145	46.85	0	1303.762187	2	652.3850708	0.52014207	0.000339071	20.53000102
																			EQILEEFSK	6	228008291	2xTMT6plex [N-Term; K9]	2.179	1.254	100	3.443170309	30.53	0	1580.893595	2	790.9495239	-1.153511181	-0.000911789	65.0093497
																			DYAFIHFDER	4	228008291	1xTMT6plex [N-Term]	1.51			3.294579983	45.08	0	1541.758609	3	514.5908203	-0.454793958	-0.000233727	61.31875545
																			LFVGSIPK	8	228008291	2xTMT6plex [N-Term; K8]	6.929	100	3.451	2.833257198	36.61	0	1318.84988	2	659.9281616	-0.631677513	-0.000416544	66.64259136
																			LMMDPLTGLNR	6	228008291	1xTMT6plex [N-Term]	1.38	100	100	2.494659662	25.13	0	1489.806821	2	745.4069214	-0.171348767	-0.000127638	78.02408863
																			AGPIWDLR	2	228008291	1xTMT6plex [N-Term]	1.779			2.573340654	22.75	0	1156.667609	2	578.8375854	0.246448016	0.000142529	72.14733946
																			LDEIYVAGLVAHSDLDER	5	228008291	1xTMT6plex [N-Term]		100		1.90702796	6.97	0	2244.170942	3	748.7281494	-0.466728177	-0.000349139	95.38887894
																			AFSQFGK	2	228008291	2xTMT6plex [N-Term; K7]	1.319			3.018163443	27.64	0	1242.724679	2	621.864624	-2.178924399	-0.001353902	44.08806906
41393561	LAP3	cytosol aminopeptidase	51056	NP_056991.2	53.94990366	1	22	22	133	519	56.131	7.93	0.49	0.56	0.63	0.56	Significant	0.0052																
																			GVLFASGQNLAR	7	41393561	1xTMT6plex [N-Term]	0.489	0.638	0.448	4.062058449	56.62	0	1461.837528	2	731.4212646	-1.556419973	-0.001137617	62.04329223
																			LNLPINIIGLAPLCENMPSGK	8	41393561	2xTMT6plex [N-Term; K21]; 1xCarbamidomethyl [C14	100	2.226	0.01	3.055760145	20.92	0	2722.539985	3	908.1821899	-2.926780075	-0.002656092	102.7704646
																			AAGIDEQENWHEGKENIR	6	41393561	2xTMT6plex [N-Term; K14]	0.357	0.335	0.49	5.92693615	61.84	1	2554.305034	4	639.3328857	1.831898966	0.001169807	29.9966508
																			TIQVDNTDAEGR	8	41393561	1xTMT6plex [N-Term]	0.715	0.549	0.6	4.632653713	68.25	0	1547.78628	2	774.3971558	0.487698752	0.000377427	43.22977661
																			TFYGLHQDFPSVVLVGLGK	4	41393561	2xTMT6plex [N-Term; K19]	100	0.8		3.030841589	19.09	0	2535.437552	3	845.8173218	-0.055070561	-4.65E-05	92.77718717
																			GSDEPPVFLEIHYK	8	41393561	2xTMT6plex [N-Term; K14]	0.614	0.498	0.342	4.042841911	30.95	0	2089.137013	3	697.0505981	0.109333946	7.61E-05	79.68326088
																			AAGIDEQENWHEGK	2	41393561	2xTMT6plex [N-Term; K14]		0.707		4.745059967	64.99	0	2042.034339	3	681.3493042	-0.479537648	-0.000326411	38.92139277
																			LILADALCYAHTFNPK	4	41393561	2xTMT6plex [N-Term; K16]; 1xCarbamidomethyl [C8	0.807			2.831600189	26.19	0	2305.27788	3	769.097168	-0.403302247	-0.000309908	101.2643846
																			WAHLDIAGVMTNKDEVPYLR	6	41393561	2xTMT6plex [N-Term; K13]	0.272			4.242599487	11.55	1	2786.506376	4	697.3824463	0.566686753	0.000394769	83.33513091
																			ETLNISGPPLK	8	41393561	2xTMT6plex [N-Term; K11]	0.792			3.461324692	33.03	0	1626.983079	2	813.9953003	0.150865698	0.000122728	60.18379765
																			LYGSGDQEAWQK	4	41393561	2xTMT6plex [N-Term; K12]	0.885	0.895		2.994779587	19.51	0	1839.964134	2	920.4864502	0.80973895	0.000744945	63.15231472
																			GITFDSGGISIK	8	41393561	2xTMT6plex [N-Term; K12]	0.706	0.498		3.201653481	52.24	0	1652.962343	2	826.984375	-0.526128138	-0.000434835	73.06455367
																			ASANMDLMR	8	41393561	1xTMT6plex [N-Term]	0.664	0.681	0.614	3.41339016	43.67	0	1237.623044	2	619.3148193	-0.550618485	-0.000340729	43.82964799
																			GLVLGIYSK	6	41393561	2xTMT6plex [N-Term; K9]	0.431	0.447	0.569	3.199985504	38.61	0	1407.897558	2	704.4523926	-0.035218294	-2.48E-05	75.13700862
																			EKEDDVPQFTSAGENFDK	6	41393561	3xTMT6plex [N-Term; K2; K18]	0.403	0.418	0.393	3.306642294	20.24	1	2743.402966	3	915.1390991	-0.08071243	-7.38E-05	52.59677506

Bhat et al,2017. Cigarette smoke and chewing tobacco alter expression of different sets of miRNAs in oral keratinocytes

Supplementary Table S2(A). List of all proteins and peptides identified in OKF6/TERT1 cells chronically treated with chewing tobacco using Sequest and Mascot search algorithms

Accession	Gene_Symbol	Description	Gene_ID	Refseq_Accession	Coverage	Proteins	#Unique_Peptides	#Peptides	#PSMS	#AAs	MW [kDa]	calc. pI	OKF6-STE-6M/OKF6P_R1	OKF6-STE-6M/OKF6P_R2	OKF6-STE-6M/OKF6P_R3	Median fold change	Status	P-value (6M)	Sequence	#PSMs	Protein Accessions	Modifications / OKF6-STE-6M/OKF6P_R1 / OKF6-STE-6M/OKF6P_R2 / OKF6-STE-6M/OKF6P_R3 / Xcorr / Ionscore / # Missed Cleavages / Theo. MH+ [Da] / Charge / m/z / DeltaM [ppm] / Deltam/z [Da] / RT [min]
																			QVVDCQLADVNNIGK	4	41393561	[illegible]
																			TLIEFLLR	8	41393561	[illegible]
																			ADMGGAATICSAIVSAAK	2	41393561	[illegible]
																			MPLFEHYTR	5	41393561	[illegible]
																			ASANMDLMR	7	41393561	[illegible]
																			LFEASIETGDR	4	41393561	[illegible]
																			ANKPGDVVR	4	41393561	[illegible]
																			FAEIIEK	6	41393561	[illegible]
10864011	SQRDL	sulfide:quinone oxidoreductase, mitochondrial	58472	NP_067022.1	40	1	19	19	122	450	49.929	9.11	0.66	0.82	0.64	0.71	Significant	0.0064				
																			TAAAVAAQSGILDR	4	10864011	[illegible]
																			KYDGYTSCPLVTGYNR	6	10864011	[illegible]
																			RYPNVFGIGDCTNLPTSK	4	10864011	[illegible]
																			KVGAENVAIVEPSER	22	10864011	[illegible]
																			VGAENVAIVEPSER	2	10864011	[illegible]
																			NCIHTDDDEK	4	10864011	[illegible]
																			VTELNPDKNCIHTDDDEK	5	10864011	[illegible]
																			YPNVFGIGDCTNLPTSK	7	10864011	[illegible]
																			GLPEGFAHPK	6	10864011	[illegible]
																			AEPLETFPFDQSK	14	10864011	[illegible]
																			TSPVADAAGWVDVDKETLQHR	4	10864011	[illegible]
																			YADALQEIIQER	7	10864011	[illegible]
																			KYADALQEIIQER	2	10864011	[illegible]
																			GYWGGPAFLR	6	10864011	[illegible]
																			IMYLSEAYFR	5	10864011	[illegible]
																			VTELNPDK	6	10864011	[illegible]
																			TISVIMK	10	10864011	[illegible]
																			LSMYLMK	4	10864011	[illegible]
																			KLFHLGMS	4	10864011	[illegible]
12056468	JUP	junction plakoglobin	3728	NP_068831.1	40.13422819	1	16	21	110	745	81.693	6.14	0.84	0.79	0.82	0.81	Significant	0.00032				
																			MEEIVEGCTGALHILAR	3	12056468	[illegible]
																			MEEIVEGCTGALHILAR	12	12056468	[illegible]
																			AGDKDDITEPAVCALR	2	12056468	[illegible]
																			TMQNTSDLDTAR	6	12056468	[illegible]
																			GIMEEDEACGR	10	12056468	[illegible]
																			NLALCPANHAPLQEAAVIPR	2	12056468	[illegible]
																			HVAAGTQQPYTDGVR	4	12056468	[illegible]
																			VTEWQQTYTYDSGIHSGANTCVPSVSSK	2	12056468	[illegible]
																			EAADAIDAEGASAPLMELLHSR	4	12056468	[illegible]
																			SAIVHLINYQDDAELATR	8	12056468	[illegible]
																			LLNDEDPVVVTK	6	12056468	[illegible]
																			QEGLESVLK	2	12056468	[illegible]
																			GIMEEDEACGR	13	12056468	[illegible]
																			VAAGVLCELAQDK	2	12056468	[illegible]
																			EGLLAIFK	4	12056468	[illegible]
																			LLNQPNQWPLVK	4	12056468	[illegible]
																			NLSDVATK	2	12056468	[illegible]
																			ALMGSPQLVAAVVR	4	12056468	[illegible]
																			LVQNCLWTLR	6	12056468	[illegible]
																			LVQLLVK	4	12056468	[illegible]
																			ALPELTK	12	12056468	[illegible]
																			VSVELTNSLFK	2	12056468	[illegible]
																			EAMCPGVSGEDSSLLLATQVEGQATNLQR	2	12056468	[illegible]
42544159	HSPH1	heat shock protein 105 kDa isoform 1	10808	NP_006635.2	30.3030303	1	19	21	103	858	96.804	5.39	1.47	1.72	1.49	1.56	Significant	0.0026				
																			FICEQDHQNFLR	8	42544159	[illegible]
																			GCALQCAILSPAFK	8	42544159	[illegible]
																			QDLPSLDEKPR	8	42544159	[illegible]
																			DISTTLNADEAVAR	6	42544159	[illegible]
																			EKENLSYDLVPLK	4	42544159	[illegible]
																			LLTETEDWLYEEGEDQAK	9	42544159	[illegible]
																			SVLDAAQIVGLNCLR	6	42544159	[illegible]
																			DLLNMYIETEGK	4	42544159	[illegible]
																			NAVEEYVYEFR	4	42544159	[illegible]
																			LVEHFCAEFK	4	42544159	[illegible]
																			VVNVELPIEANLVWQLGK	3	42544159	[illegible]
																			FVVQNVSAQK	6	42544159	[illegible]
																			LKETAENSLK	6	42544159	[illegible]
																			KVDQPPEAK	4	42544159	[illegible]
																			KPVTDCVISVPSFFTDAER	5	42544159	[illegible]
																			MFEELGQR	6	42544159	[illegible]
																			AGGIETIANEFSDR	2	42544159	[illegible]
																			IEVPLYSLLEQTHLK	2	42544159	[illegible]
																			AFNDPFIQK	4	42544159	[illegible]
																			FQEAEERPK	2	42544159	[illegible]
																			SLDQDPVVR	2	42544159	[illegible]
57863257	TCP1	T-complex protein 1 subunit alpha isoform a	6950	NP_110379.2	48.38129496	1	18	18	142	556	60.306	6.11	1.16	1.19	1.18	1.18	Significant	0.000056				
																			AFHNEAQVNPER	4	57863257	[illegible]
																			ASGATILSTLANLEGEETFEAAMLGQAEEVVQER	5	57863257	[illegible]
																			GANDFMCDEMER	15	57863257	[illegible]
																			EQLAIAEFAR	8	57863257	[illegible]
																			YINENLIVNTDELGR	6	57863257	[illegible]
																			SQNVMAAASIANIVK	4	57863257	[illegible]
																			SQMESMLISGYALNCVVGSQGMPK	2	57863257	[illegible]
																			YPVNSVNILK	3	57863257	[illegible]
																			SSLGPVGLDK	4	57863257	[illegible]
																			GANDFMCDEMER	26	57863257	[illegible]
																			SLHDALCVVK	6	57863257	[illegible]
																			LLEVEHPAAK	8	57863257	[illegible]
																			SLLVIPNTLAVNAAQDSTDLVAK	6	57863257	[illegible]
																			ICDDELILIK	8	57863257	[illegible]
																			GANDFMCDEMER	8	57863257	[illegible]
																			QAGVFEPTIVK	2	57863257	[illegible]
																			IACLDFSLQK	8	57863257	[illegible]
																			DDKHGSYEDAVHSGALND	7	57863257	[illegible]
																			DCLINAAK	4	57863257	[illegible]
																			SVVPGGGAVEAALSIYLENYATSMGSR	8	57863257	[illegible]
212549553	ILF3	interleukin enhancer-binding factor 3 isoform c	3609	NP_060090.2	30.28953229	1	21	21	124	898	95.748	8.81	1.15	1.16	1.01	1.11	Significant	0.0019				
																			GSSEQAESDNMDVPPEDDSKEGAGEQK	2	212549553	[illegible]
																			AYAALAALEK	12	212549553	[illegible]
																			SIGTANRPMGAGEALR	2	212549553	[illegible]
																			YELISETGGSHDK	6	212549553	[illegible]
																			SGGNSYGSGGASYNPGSHGGYGGGSGGGSSYQGK	2	212549553	[illegible]
																			GSSEQAESDNMDVPPEDDSK	2	212549553	[illegible]
																			EDITQSAQHALR	2	212549553	[illegible]
																			LAAFGQLHK	12	212549553	[illegible]
																			EKPTTALLDK	18	212549553	[illegible]
																			AVSDWIDEQEK	10	212549553	[illegible]
																			SIGTANRPMGAGEALR	4	212549553	[illegible]
																			VLGMDPLPSK	8	212549553	[illegible]
																			VLQDMGLPTGAEGR	6	212549553	[illegible]
																			AEPPQAMNALMR	2	212549553	[illegible]
																			GDLDLELVLLCK	6	212549553	[illegible]
																			GWPLELICEK	16	212549553	[illegible]
																			LNQLKPGLQYK	5	212549553	[illegible]
																			EPPLSLTIHLTSPVVR	6	212549553	[illegible]
																			LFPDTPLALDANK	4	212549553	[illegible]
																			VLAGETLSVNDPPDVLDR	3	212549553	[illegible]
																			IFVNDDR	2	212549553	[illegible]
																			SCVIVIR	4	212549553	[illegible]
9951915	AHCY	adenosylhomocysteinase isoform 1	191	NP_000678.1	43.05555556	1	18	19	134	432	47.685	6.34	1.4	1.42	1.25	1.36	Significant	0.0016				
																			ALDIAENEMPGLMR	12	9951915	[illegible]
																			GISEETTTGVHNLYK	15	9951915	[illegible]
																			VPAINVNDSVTK	6	9951915	[illegible]
																			VAVVAGYGDVGK	6	9951915	[illegible]
																			KLDEAVAEAHLGK	14	9951915	[illegible]
																			QAQYLGMSCDGPFKPDHYR	2	9951915	[illegible]
																			KALDIAENEMPGLMR	5	9951915	[illegible]
																			VADIGLAAWGR	8	9951915	[illegible]
																			ATDVMIAGK	6	9951915	[illegible]
																			WLNENAVEK	6	9951915	[illegible]
																			AGIPVYAWK	6	9951915	[illegible]
																			YPVGVHFLPK	2	9951915	[illegible]
																			ESLIDGIK	12	9951915	[illegible]
																			IILLAEGR	12	9951915	[illegible]
																			ATDVMIAGK	4	9951915	[illegible]
																			FDNLYGCR	6	9951915	[illegible]
																			SKFDNLYGCR	4	9951915	[illegible]
																			YPQLLPGIR	6	9951915	[illegible]
																			YSASKPLK	3	9951915	[illegible]
																			VNIKPQVDR	5	9951915	[illegible]
1002095800	DDX5	probable ATP-dependent RNA helicase DDX5 isoform b	1655	NP_001307526.1	42.01954397	1	17	24	124	614	69.044	8.85	1.32	1.42	1.39	1.38	Significant	0.00046				
																			QNFTEPTAIQAQGWPVALSGLDMVGVAQTGSGK	6	1002095800	[illegible]
																			FVINYDYPNSSEDYIHR	5	1002095800	[illegible]
																			TTYLVLDEADR	6	1002095800	[illegible]
																			GDGPICLVLAPTR	6	1002095800	[illegible]
																			GVEICIATPGR	8	1002095800	[illegible]
																			LIDFLECGK	4	1002095800	[illegible]
																			STCIYGGAPK	2	1002095800	[illegible]
																			APILIATDVASR	8	1002095800	[illegible]
																			QVSDLISVLR	4	1002095800	[illegible]
																			EANQAINPK	4	1002095800	[illegible]
																			TIVFVETK	10	1002095800	[illegible]
																			NFYQEHPDLAR	2	1002095800	[illegible]
																			QLAEDFLK	4	1002095800	[illegible]
																			MLDMGFEPQIR	10	1002095800	[illegible]
																			DWVLNEFK	6	1002095800	[illegible]
																			KWNLDELPK	2	1002095800	[illegible]
																			WNLDELPK	6	1002095800	[illegible]
																			LMEEIMSEK	6	1002095800	[illegible]
																			TAQEVETYR	2	1002095800	[illegible]
																			QTLMWSATWPK	6	1002095800	[illegible]
																			GYSSLLK	8	1002095800	[illegible]
																			ELAQQVQQVAAEYCR	3	1002095800	[illegible]
																			IVDQIRPDR	4	1002095800	[illegible]
																			LLQLVEDR	2	1002095800	[illegible]
4885409	HDLBP	vigilin isoform a	3069	NP_005327.1	23.34384858	1	25	25	105	1268	141.352	6.87	1.25	0.96	1.47	1.23	Significant	0.014				
																			AACLESAQEPAGAWGNK	2	4885409	[illegible]
																			ASVITQVFHVPLEER	10	4885409	[illegible]
																			VATLNSEEESDPPTYK	4	4885409	[illegible]
																			GNSLQEILER	4	4885409	[illegible]
																			IEGDPQGVQQAK	2	4885409	[illegible]
																			DAPWTASSSEK	2	4885409	[illegible]
																			VHIEFTEGEDK	4	4885409	[illegible]
																			ELQAEQEDR	8	4885409	[illegible]
																			IVGELEQMVSEDVPLDHR	4	4885409	[illegible]
																			EALEALVPVTIEVEVPFDLHR	6	4885409	[illegible]
																			TEIVFTGEK	4	4885409	[illegible]
																			RCDIIIISGR	6	4885409	[illegible]
																			TTTIAVEVK	2	4885409	[illegible]
																			EIAEEYGGVMVSFPR	2	4885409	[illegible]
																			VIFPAAEDKDQDLITIIGK	4	4885409	[illegible]
																			LSVTVDPK	4	4885409	[illegible]
																			IQDIELK	12	4885409	[illegible]
																			DKFPEVIINFPDPAQK	4	4885409	[illegible]
																			DCVEAAK	6	4885409	[illegible]
																			DFSVQIK	10	4885409	[illegible]
																			LVGEIMQETGTR	4	4885409	[illegible]
																			APDMSSSEEFPSFGAQVAPK	1	4885409	[illegible]
																			VKELQAEQEDR	2	4885409	[illegible]
																			GAVITQIR	2	4885409	[illegible]
																			DLIIEQR	2	4885409	[illegible]
208973244	YWHAZ	14-3-3 protein zeta/delta	7534	NP_001129174.1	68.97959184	1	11	15	252	245	27.728	4.79	1.02	1.06	1	1.03	Significant	0.0003				
																			GIVDQSQQAYQEAFEISK	33	208973244	[illegible]
																			DICNDVLSLLEK	12	208973244	[illegible]
																			SVTEQGAELSNEER	20	208973244	[illegible]
																			YLAEVAAGDDKK	10	208973244	[illegible]
																			TAFDEAIAELDTLSEESYK	18	208973244	[illegible]
																			FLIPNASQAESK	8	208973244	[illegible]
																			DSTLIMQLLR	37	208973244	[illegible]
																			YLAEVAAGDDK	12	208973244	[illegible]
																			YDDMAACMK	8	208973244	[illegible]
																			DNLTLWTSDTQGDEAEAGEGGEN	28	208973244	[illegible]
																			NLLSVAYK	24	208973244	[illegible]
																			DSTLIMQLLR	14	208973244	[illegible]

Bhat et al,2017. Cigarette smoke and chewing tobacco alter expression of different sets of miRNAs in oral keratinocytes

Supplementary Table S2(A). List of all proteins and peptides identified in OKF6/TERT1 cells chronically treated with chewing tobacco using Sequest and Mascot search algorithms

Accession	Gene_Symbol	Description	Gene_ID	Refseq_Accession	Coverage	Proteins	#Unique_Peptides	#Peptides	#PSMS	#AAs	MW [kDa]	calc. pI	OKF6-STE-6M/OKF6P_R1	OKF6-STE-6M/OKF6P_R2	OKF6-STE-6M/OKF6P_R3	Median fold change	Status	P-value (6M)	Sequence	#PSMSs	Protein Accessions	Modifications	OKF6-STE-6M/OKF6P_R1	OKF6-STE-6M/OKF6P_R2	OKF6-STE-6M/OKF6P_R3	Xcorr	Ionscore	# Missed Cleavages	Theo. MH+ [Da]	Charge	m_z	DeltaM [ppm]	Deltam/z [Da]	RT [min]
																			YDDMAACMK	7	208973244	2xTMT6plex [N-Term; K9]; 1xCarbamidomethyl [C7]; 1xOxidation [M]	1.068	1.04	1.046	2.444274902	27.75	0	1578.736643	2	781.8748779	0.480898002	0.00037576	57.47897622
																			VVSSIEQK	8	208973244	2xTMT6plex [N-Term; K8]	0.905	1	1.029	3.883118153	45.63	0	1347.824787	2	674.4160156	-0.02407583	-1.62E-05	52.94310957
																			EMQPTHPIR	4	208973244	1xTMT6plex [N-Term]; 1xOxidation [M2]	0.564		100	2.449209452	23.08	0	1353.714636	3	451.908905	-1.827720587	-0.000824737	18.46995257
																			LAEQAER	6	208973244	1xTMT6plex [N-Term]	1.208	0.935	1.021	2.81289196	25.93	0	1045.583939	2	523.2957764	0.322273863	0.000168482	26.08720983
																			EKIETELR	3	208973244	2xTMT6plex [N-Term; K2]	1.723		0.937	2.341940403	22.27	1	1475.883365	2	738.4459839	0.898874172	0.000663317	41.26144769
50592996	TUBB3	tubulin beta-3 chain isoform 1	10381	NP_006077.2	41.77777778	1	2	14	270	450	50.4	4.93	1	1.07	1.1	1.05	Significant	0.00079																
																			AILVDLEPGTMDSVR	24	50592996	1xTMT6plex [N-Term]	0.923	0.984	1.029	3.743171215	38.6	0	1844.998915	2	923.0027466	-0.378718888	-0.000349368	75.34357767
																			GHYTEGAELVDSVLDVVR	33	50592996	1xTMT6plex [N-Term]	1.17	1.17	1.141	5.213302612	59.43	0	2188.144728	3	730.0530396	-0.074166783	-5.41E-05	91.62180174
																			ISVYYNEASSHK	6	50592996	2xTMT6plex [N-Term; K12]	0.995	1.068	1.101	3.892976522	52.43	0	1855.995434	3	619.3361206	-0.875774565	-0.000541811	56.03445331
																			EVDEQMLAIQSK	4	50592996	2xTMT6plex [N-Term; K12]	1.057	1.128		4.792182922	69.2	0	1849.014121	2	925.010376	-0.348930204	-0.000322588	66.35070637
																			EIVHLQAGQCGNQIGAK	66	50592996	2xTMT6plex [N-Term; K17]; 1xCarbamidomethyl [C10	1.015	1.041	1.03	5.308478355	74.46	0	2281.248705	3	761.0859375	-2.387381081	-0.001815403	47.31921529
																			IMNTFSVVPSPK	54	50592996	2xTMT6plex [N-Term; K12]	1.243	100	3.414	3.53404808	40.35	0	1778.028648	2	889.5175781	-0.432417104	-0.000384425	77.97312852
																			FPGQLNADLR	24	50592996	1xTMT6plex [N-Term]	1.108	1.147	1.255	3.543502092	43.73	0	1359.758215	2	680.3824463	-0.440557643	-0.000299526	52.32976605
																			ISEQFTAMFR	23	50592996	1xTMT6plex [N-Term]	1.376	2.039	100	4.103599548	44.54	0	1458.761251	2	729.8840942	-0.232802617	-0.000169802	80.40800158
																			AILVDLEPGTMDSVR	14	50592996	1xTMT6plex [N-Term]; 1xOxidation [M11]	0.902	1.387	1.301	2.488433123	28.93	0	1860.99383	2	923.0027466	-0.378718888	-0.000349368	75.34357767
																			EVDEQMLAIQSK	2	50592996	2xTMT6plex [N-Term; K12]; 1xOxidation [M6		1.049		2.606637001	48.83	0	1865.009036	2	925.010376	-0.348930204	-0.000322588	66.35070637
																			ALTVPELTQQMFDAK	22	50592996	2xTMT6plex [N-Term; K15]	1.226	1.258	1.163	2.925591469	30.01	0	2150.193147	2	1075.599365	-0.787627499	-0.000846776	86.5537756
																			ALTVPELTQQMFDAK	9	50592996	2xTMT6plex [N-Term; K15]; 1xOxidation [M11	2.039	100	100	2.555007935	25.04	0	2166.188062	2	1075.599365	-0.787627499	-0.000846776	86.5537756
																			LAVNMVPFPR	4	50592996	1xTMT6plex [N-Term]	1.652	1.913		3.099212646	32.08	0	1372.797243	2	686.9009399	-1.922976755	-0.001319929	73.50072
																			LAVNMVPFPR	4	50592996	1xTMT6plex [N-Term]; 1xOxidation [M5]	1.084	100		1.945205092	20.38	0	1388.792158	2	686.9009399	-1.922976755	-0.001319929	73.50072
																			ISEQFTAMFR	2	50592996	1xTMT6plex [N-Term]; 1xOxidation [M8]	100			1.61943233	17.77	0	1474.756166	2	729.8840942	-0.232802617	-0.000169802	80.40800158
																			NMMAACDPR	2	50592996	1xTMT6plex [N-Term]; 1xCarbamidomethyl [C6	1.158			2.256533384	24.59	0	1294.590363	2	647.7979736	-1.307629365	-0.000846422	36.95700301
																			YLTVATVFR	4	50592996	1xTMT6plex [N-Term]	1.092	100		2.322813988	36.65	0	1298.766989	2	649.8864746	-1.013454501	-0.000658121	75.76769563
																			NSSYFVEWIPNNVK	3	50592996	2xTMT6plex [N-Term; K14]				2.179470062	13.24	0	2155.158811	2	1078.081421	-1.505992556	-0.001622827	78.89611646
																			LATPTYGDLNHLVSATMSGVTTSLR	3	50592996	1xTMT6plex [N-Term]				1.399895072	1.99	0	2834.491959	3	945.5006714	-1.586802824	-0.00149926	95.01535195
25777602	PSMD2	26S proteasome non-ATPase regulatory subunit 2 isoform 1	5708	NP_002799.3	34.14096916	1	23	23	98	908	100.136	5.2	1.22	1.2	1.13	1.18	Significant	0.00053																
																			LAQGLTHLGK	6	25777602	2xTMT6plex [N-Term; K10]	1.774	1.636	1.451	3.757145882	40.66	0	1495.936069	3	499.3167419	-0.264890947	-0.000132087	51.32849881
																			TITGFQTHTTPVLLAHGER	8	25777602	1xTMT6plex [N-Term]	0.944	1.139	1.299	6.402181149	56.32	0	2308.261095	4	577.8203125	-0.725537507	-0.000418682	46.81694731
																			VCLYLTSCVNYVPEPENSALLR	2	25777602	1xTMT6plex [N-Term]; 2xCarbamidomethyl [C2; C8	100			3.301769018	23.05	0	2826.436753	3	942.8161011	-1.06236849	-0.001000906	89.33978921
																			APVQPQQSPAAAPGGTDEKPSGK	4	25777602	3xTMT6plex [N-Term; K19; K23]	0.735		1.076	3.864479065	23.51	0	2905.598646	3	969.2047729	0.385461928	0.000373333	32.29492422
																			SSTTSMTSVPKPLK	4	25777602	3xTMT6plex [N-Term; K11; K14]		0.959	1.416	4.363342762	21.72	0	2151.266202	3	717.7595215	-1.018301163	-0.000730212	51.8544044
																			DKAPVQPQQSPAAAPGGTDEKPSGK	2	25777602	4xTMT6plex [N-Term; K2; K21; K25]		1.214		5.82144165	14.15	1	3377.883484	4	845.2255859	-0.879142225	-0.00074241	35.36129413
																			DAGDKDKEQELSEEDK	2	25777602	4xTMT6plex [N-Term; K5; K7; K16]			0.531	3.976830006	10.15	2	2752.465849	4	688.8716431	-0.402110121	-0.000276699	37.77015558
																			LVGSQEELASWGHEYVR	6	25777602	1xTMT6plex [N-Term]	0.494	0.528	100	3.00734067	27.26	0	2189.118847	3	730.3775024	-0.408048098	-0.000297755	68.20791665
																			DTSLYRPALEELR	4	25777602	1xTMT6plex [N-Term]	1.369	0.844		4.079973221	24.53	0	1791.980229	3	597.9971313	-1.890633042	-0.001129326	55.31722516
																			EIVPYNMAHNAEHEACDLLMEIEQVDMLEK	4	25777602	2xTMT6plex [N-Term; K30]; 1xCarbamidomethyl [C16				2.301947117	5.93	0	4029.947051	4	1008.240845	-1.365301747	-0.001375523	103.8430747
																			DIDENAYAK	6	25777602	2xTMT6plex [N-Term; K9]	1.037	1.162		4.27390337	56.95	0	1496.799694	2	748.90448	1.328762255	0.000994445	39.67137247
																			EPLLTLVK	10	25777602	2xTMT6plex [N-Term; K8]	1.084	1.485	1.053	3.266187191	43.33	0	1370.902309	2	685.9541626	-0.919521894	-0.000630287	73.56076774
																			GEAIEAILAALEVVSEPFR	4	25777602	1xTMT6plex [N-Term]	100	100		2.170568228	13.05	0	2243.248464	2	1122.124756	-2.776946624	-0.003114691	113.3905646
																			LNILDTLSK	4	25777602	2xTMT6plex [N-Term; K9]	1.955	100		2.556342363	27.09	0	1474.924501	2	737.9655151	-0.506749032	-0.000373708	85.06520193
																			MLVTFDEELRPLPVSVR	8	25777602	1xTMT6plex [N-Term]	0.88	100		3.450103521	15.14	0	2230.24669	3	744.0858154	-1.702668568	-0.00126579	78.37371366
																			ELDIMEPK	4	25777602	2xTMT6plex [N-Term; K8]		1.346	1.266	2.123667717	27.74	0	1432.812173	2	716.9095459	-0.250062871	-0.000179147	68.10035053
																			EWQELDDAEK	4	25777602	2xTMT6plex [N-Term; K10]		100	0.01	2.253600597	17.2	0	1720.879401	2	860.9439697	0.733126984	0.000630812	60.43642298
																			EDVLTLLLPVMGDSK	3	25777602	2xTMT6plex [N-Term; K15]				1.787335396	3.18	0	2088.202649	2	1044.60376	-1.152449812	-0.001203274	104.979741
																			LLTDDGNK	4	25777602	2xTMT6plex [N-Term; K8]	1.524	1.413		3.489943266	31.98	0	1333.772751	2	667.3898315	-0.27368535	-0.000182517	52.67138597
																			WLPLGLGLNHLGK	2	25777602	2xTMT6plex [N-Term; K13]	1.745			2.379510164	28.33	0	1876.157295	3	626.0567017	-0.929106283	-0.00058105	88.39663406
																			SETELKDTYAR	1	25777602	2xTMT6plex [N-Term; K6]	1.437			1.932247877		1	1770.9638	3	590.9926758	-0.183750933	-0.000108472	41.02974961
																			YLYSSEDYIK	2	25777602	2xTMT6plex [N-Term; K10]		100		1.066169977	3.25	0	1738.930374	2	869.9691162	0.334465303	0.000290806	70.88425831
																			VPDDIYK	4	25777602	2xTMT6plex [N-Term; K7]		1.016	1.03	2.633311987	31.08	0	1307.761124	2	654.3847656	0.864370395	0.000565195	49.65074563
34147630	TUFM	elongation factor Tu, mitochondrial precurso	7284	NP_003312.3	48.57142857	1	18	18	138	455	49.843	7.61	0.85	0.78	0.84	0.82	Significant	0.0007																
																			ADAVQDSEMVELVELEIR	10	34147630	1xTMT6plex [N-Term]	0.894	0.881	0.886	5.291006088	64.07	0	2275.168893	3	759.0611572	0.010974872	8.32E-06	97.91089571
																			TVVTGIEMFHK	7	34147630	2xTMT6plex [N-Term; K11]	0.872	1.001	0.945	3.738265753	46.99	0	1719.986784	3	574.0002441	-0.351472541	-0.000201509	64.41879059
																			QIGVEHVVVYVNK	6	34147630	2xTMT6plex [N-Term; K13]	0.63	0.937	1.096	4.104439735	30.21	0	1942.152603	3	648.0562744	0.858146875	0.000555551	51.39861722
																			DKPHVNVGTIGHVDHGK	2	34147630	3xTMT6plex [N-Term; K2; K17]		0.759		5.472416878	23.84	0	2497.424251	5	500.2906494	-0.044125337	-2.20E-05	46.99597267
																			GITINAAHVEYSTAAR	26	34147630	1xTMT6plex [N-Term]	0.72	0.693	0.662	5.634465218	57.7	0	1903.023491	2	952.0148926	-0.516012417	-0.000490992	46.63686131
																			KYEEIDNAPEER	6	34147630	2xTMT6plex [N-Term; K1]	0.971	0.835	0.947	4.641605377	29.59	1	1951.017292	3	651.0102539	-0.555136674	-0.000361027	36.31253325
																			GTVVTGTLER	8	34147630	1xTMT6plex [N-Term]	0.741	0.798	0.763	4.098875046	62.52	0	1261.731332	2	631.3695679	0.418133539	0.000263786	40.80122373
																			TTLTAAITK	6	34147630	2xTMT6plex [N-Term; K9]	0.839	1.287	0.925	4.159566879	61.94	0	1377.871737	2	689.4355469	-5.748089453	-0.003960065	47.63912332
																			ELAMPGEDLK	8	34147630	2xTMT6plex [N-Term; K10]	0.744	0.747	0.5	3.727340221	41.75	0	1560.870751	2	780.9385986	-0.531923847	-0.000415132	60.28030579
																			DLEKPFLLPVEAVYSVPGR	6	34147630	2xTMT6plex [N-Term; K4]	100		0.82	3.190322161	17.75	0	2587.489981	3	863.1676636	-0.596592588	-0.000514559	85.74870641
																			ELLTEFGYK	7	34147630	2xTMT6plex [N-Term; K9]	0.922	1.037		3.49972558	29.71	0	1557.892867	2	779.4505005	0.550600482	0.000428888	87.04795278
																			LLDAVDTYIPVPAR	6	34147630	1xTMT6plex [N-Term]	1.333	0.583		3.196599483	31.22	0	1772.015552	2	886.5107422	-0.758421673	-0.000671967	85.32099217
																			ILAEGGGAK	6	34147630	2xTMT6plex [N-Term; K9]	0.901	0.849	0.867	3.756865978	47.15	0	1273.788008	2	637.3917236	-9.292797784	-0.005918527	42.23576637
																			VEAQVYILSK	7	34147630	2xTMT6plex [N-Term; K10]		100		2.488309622	30.45	0	1607.977265	2	804.4917603	-0.634954368	-0.000510496	65.46261333
																			KGDECELLGHSK	6	34147630	3xTMT6plex [N-Term; K1; K12]; 1xCarbamidomethyl [C5	1.097		1.078	3.806375265	14.06	1	2060.141336	4	515.7904053	-0.749864207	-0.000386207	38.29341168
																			GEETPVIVGSALCALEGR	4	34147630	1xTMT6plex [N-Term]; 1xCarbamidomethyl [C13			100	1.448176622	10.63	0	2087.10042	2	1044.052856	-0.9506679	-0.00099207	86.58001136
																			QPMILEK	2	34147630	2xTMT6plex [N-Term; K7]	0.769			3.1994555	26.32	0	1316.801215	2	658.9042358	-0.015105023	-9.95E-06	57.76083929
																			IILPPEK	15	34147630	2xTMT6plex [N-Term; K7]	0.842	0.989	0.887	2.874926805	27.73	0	1267.838981	2	634.4234009	0.429437666	0.000272229	59.19973237
148613856	DDX17	probable ATP-dependent RNA helicase DDX17 isoform 3	10521	NP_001091974.1	33.92612859	1	16	23	121	731	80.407	8.37	0.92	1.17	1.11	1.06	Significant	0.005																
																			DMVGIAQTGSGK	2	148613856	2xTMT6plex [N-Term; K12]			1.153	4.407876015	65.7	0	1621.898363	2	811.4539185	1.355013429	0.001098847	50.01879748
																			FVINYDYPNSSEDYVHR	6	148613856	1xTMT6plex [N-Term]	1.218	1.185	0.602	4.216942787	44.03	0	2347.119241	3	783.0458374	1.583965981	0.001239252	68.56658054
																			LTPYEVDELRR	5	148613856	1xTMT6plex [N-Term]	0.877	1.002	1.009	4.622374535	38.9	1	1619.895437	3	540.635376	-2.38406148	-0.00128731	45.6355171
																			GDGPICLVLAPTR	6	148613856	1xTMT6plex [N-Term]; 1xCarbamidomethyl [C6	1.438	1.725	2.022	3.996110439	63.02	0	1597.893328	2	799.4514771	1.470061562	0.001174501	70.17936501
																			SSQSSSQQFSGIGR	6	148613856	1xTMT6plex [N-Term]	1.258	1.032	1.404	4.215635777	28.91	0	1684.845192	2	842.9260864	-0.175463264	-0.000147814	32.24599158
																			GVEICIATPGR	8	148613856	1xTMT6plex [N-Term]; 1xCarbamidomethyl [C5	1.119	1.245		4.67056179	50.39	0	1401.772151	2	701.3901978	0.690617096	0.000484044	48.22089863
																			VLEEANQAINPK	5	148613856	2xTMT6plex [N-Term; K12]	0.78	1.179		3.211826324	43.41	0	1784.03182	2	892.5189819	-0.63467075	-0.000566136	65.89309132
																			STCIYGGAPK	2	148613856	2xTMT6plex [N-Term; K10]; 1xCarbamidomethyl [C3	1.33			4.202385902	37.05	0	1511.829221	2	756.4179688	-0.370365907	-0.000279965	41.29195412
																			NFYVEHPEVAR	2	148613856	1xTMT6plex [N-Term]	0.839			3.675665617	34.37	0	1589.827357	3	530.6137085	-0.493683616	-0.000261624	42.67943213
																			LTPYEVDELR	4	148613856	1xTMT6plex [N-Term]	1.479	1.762		3.731387615	44	0	1463.794326	2	732.401123	0.439832112	0.000321912	62.98366754
																			APILIATDVASR	8	148613856	1xTMT6plex [N-Term]	0.977	0.977	0.792	3.555317879	42.19	0	1455.873245	2	728.4396973	-0.773953882	-0.000563389	56.98387905
																			DGWPAMCIHGDK	2	148613856	2xTMT6plex [N-Term; K12]; 1xCarbamidomethyl [C7	1.246			2.879039288	25.9	0	1844.918781	3	615.6447754	0.537780788	0.000330721	57.68360574
																			ELAQQVQQVADDYGK	9	148613856	2xTMT6plex [N-Term; K15]	100	0.258	100	2.615769386	22.59	0	2150.149368	2	1075.579956	1.519536002	0.001633615	75.35469553
																			MLDMGFEPQIR	10	148613856	1xTMT6plex [N-Term]	1.302	0.01	1.498	3.182462692	33.69	0	1565.801736	2	783.40448	-0.033649894	-2.63E-05	68.74450247
																			LIDFLESGK	6	148613856	2xTMT6plex [N-Term; K9]	0.869		100	2.095726967	27.09	0	1479.882302	2	740.4441528	-0.860119711	-0.000636438	90.85186876
																			QTLMWSATWPK	6	148613856	2xTMT6plex [N-Term; K11]		1.816		2.017383814	28.13	0	1806.997683	2	904.0021973	-0.312573035	-0.000282409	85.54065511
																			TIIFVETK	10	148613856	2xTMT6plex [N-Term; K8]	0.946	100	100	3.776169062	34.56	0	1408.881574	2	704.9437866	-0.906440851	-0.000638534	65.10968759
																			DWVLNEFR	4	148613856	1xTMT6plex [N-Term]	100	0.01		2.716156244	29.71	0	1307.694552	2	654.350647	-0.408998177	-0.000267422	96.22689876
																			WDLSELPK	4	148613856	2xTMT6plex [N-Term; K8]	0.942	0.862		2.641740322	28.86	0	1445.840437	2	723.4239502	0.129081066	9.33E-05	84.73911697
																			APLPDLYPFGTMR	4	148613856	1xTMT6plex [N-Term]	0.705	100		2.032912493	12.04	0	1706.913729	2	853.9588013	-1.993856443	-0.00170167	83.81086708
																			QLAEDFLR	6	148613856	1xTMT6plex [N-Term]	0.402	100	100	2.376462221	32.17	0	1220.683653	2	610.8449707	-0.809672307	-0.000494177	62.64772871
																			IVDQIRPDR	4	148613856	1xTMT6plex [N-Term]		1.715		3.394581556	15.04	0	1340.784764	3	447.6002502	1.069062284	0.000477794	34.9336631
																			GTAYTFFTPGNLK	2	148613856	2xTMT6plex [N-Term; K13]				0.921481371	0.31	0	1875.041656	2	938.0244751	0.009399958	8.81E-06	68.0002282
109637759	CAST	calpastatin isoform f	831	NP_001035905.1	41.6	1	22	22	99	750	80.216	5.15	0.59	0.86	0.97	0.81	Significant	0.019																
																			TYAGGTASATK	10	109637759	2xTMT6plex [N-Term; K11]	0.766	0.855	0.823	5.089231014	62.81	0	1485.831329	2	743.4194946	0.258237799	0.000191849	30.13249835
																			SLTPAVPVESKPDKPSGK	4	109637759	4xTMT6plex [N-Term; K11; K14; K18]	0.886	0.773		5.000781536	15.27	0	2753.658282	4	689.1705933	0.821168295	0.000565304	51.33931221
																			VSASSGATSK	5	109637759	2xTMT6plex [N-Term; K10]	0.869	1.039		4.500371933	61.18	0	1352.778565	2	676.8925781	-0.506734818	-0.00034275	34.25582769
																			CGEDDETIPSEYR	2	109637759	1xTMT6plex [N-Term]; 1xCarbamidomethyl [C1			1.344	3.969571352	36.49	0	1799.795524	2	900.3945923	-7.565498138	-0.006808175	40.12202713
																			DTMSDQALEALSASLGTR	6	109637759	1xTMT6plex [N-Term]		100	100	3.263333559	35.7	0	2095.053864	2	1048.029053	-1.44861729	-0.001517466	86.5315283
																			EQLPPMSEDFLLDALSEDFSGPQNASSLK	4	109637759	2xTMT6plex [N-Term; K29]			1.67	2.364447594	14.66	0	3623.819115	4	906.7084961	-1.920690515	-0.001740059	107.4925199
																			GTVPDDAVEALADSLGK	10	109637759	2xTMT6plex [N-Term; K17]	100	0.921	100	3.05919981	49.72	0	2116.153785	2	1058.579834	-0.658539687	-0.000696786	88.04142033
																			KPADDQDPIDALSGDLDSCPSTTETSQNTAK	6	109637759	3xTMT6plex [N-Term; K1; K31]; 1xCarbamidomethyl [C19		0.77	0.01	2.83497858	3.06	0	3964.95366	4	991.9916992	-2.192420327	-0.002173211	59.98151861
																			QPDPDENKPMEDK	2	109637759	3xTMT6plex [N-Term; K8; K13]	0.703			3.337271929	18.22	0	2230.162859	3	744.0610352	2.552866813	0.00189777	37.6544837
																			DDTIPPEYR	16	109637759	1xTMT6plex [N-Term]	0.896	0.737	0.972	3.644010544	37.28	0	1334.678962	2	667.8430176	-0.152144265	-0.000101532	33.48185769
																			DTSQSDKDLDDALDK	4	109637759	3xTMT6plex [N-Term; K7; K15]		0.851	1.025	4.227209091	5.7	1	2353.233775	3	785.0778809	-6.240668492	-0.004895251	46.19706832
																			TSESPSKPGEK	4	109637759	3xTMT6plex [N-Term; K7; K11]	0.984	0.825		1.962145448	8.67	0	1834.052504	3	612.0230103	1.076033382	0.000657834	28.29210898
																			EESTEVLK	2	109637759	2xTMT6plex [N-Term; K8]			1.36	3.231644392	44.07	0	1392.798632	2	696.9017944	-1.665425826	-0.001159801	42.24472013
																			KTEKEESTEVLK	3	109637759	4xTMT6plex [N-Term; K1; K4; K12]	0.184		0.849	3.670865059		2	2337.404693	4	585.1068726	0.413777031	0.000241791	40.93173841
																			EADPEDGKPVMDK	4	109637759	3xTMT6plex [N-Term; K8; K13]	0.808	1.142		3.260854483	6.15	0	2118.135582	3	706.7174072	0.985224792	0.000695613	46.34129664
																			QAEPELDLR	2	109637759	1xTMT6plex [N-Term]	0.925			3.470261097	41.55	0	1299.710596	2	650.3594971	0.862831025	0.000560715	48.25161985
																			SAAPPQEK	4	109637759	2xTMT6plex [N-Term; K8]	0.829	0.776		3.141639709	38.45	0	1285.751622	2	643.378418	-1.60440202	-0.001031431	29.31285099
																			TKPQDMISAGGESVAGITAISGKPGDK	1	109637759	4xTMT6plex [N-Term; K2; K23; K27]				1.52540338		0	3531.986234	4	883.7518311	-0.209403775	-0.000184903	60.05911423
																			SESELIDELSEDFDR	5	109637759	1xTMT6plex [N-Term]				1.762259007	2.67	0	2012.949776	2	1006.97699	-1.526609282	-0.001536494	92.39110604
																			LAAAISEVVSQTPASTTQAGAPPR	1	109637759	1xTMT6plex [N-Term]				0.828492463		0	2552.388146	2	1276.696533	-0.923003719	-0.001177932	77.20001421
																			EETIPPDYR	2	109637759	1xTMT6plex [N-Term]	0.597			2.894533873	16.28	0	1348.694612	2	674.8518066	1.279015453	0.000862501	38.36829187
																			TEKEESTEVLK	1	109637759	3xTMT6plex [N-Term; K3; K11]		1.138		1.629998922		1	1980.146798	3	660.7200928	-0.541959661	-0.00035772	44.11362431
																			EADPEDGKPVMDK	1	109637759	3xTMT6plex [N-Term; K8; K13]; 1xOxidation [M11	0.673			1.510474086		0	2134.130497	3	706.7174072	0.985224792	0.000695613	46.34129664
28178825	IDH1	isocitrate dehydrogenase [NADP] cytoplasmic	3417	NP_005887.2	53.38164251	1	19	20	143	414	46.63	7.01	1.05	1.24	1.25	1.18	Significant	0.003																
																			VEITYTPSDGTQK	12	28178825	2xTMT6plex [N-Term; K13]	1.1	1.281	1.189	4.231511593	58.47	0	1897.031879	2	949.0110474	-8.993472185	-0.008530452	47.00125782
																			ISGGSVVEMQGDEMTR	6	28178825	1xTMT6plex [N-Term]	1.387	2.686		3.7389853	38.76	0	1924.930578	2	962.9682617	-0.691371686	-0.000665421	48.18305711
																			KISGGSVVEMQGDEMTR	4	28178825	2xTMT6plex [N-Term; K1]	0.918	1.072		4.719310284	31.08	1	2282.188473	3	761.4001465	-1.133305556	-0.000862139	52.40927803
																			LIFPYVELDLHSYDLGIENR	7	28178825	1xTMT6plex [N-Term]	1.244	0.786	1.368	4.069072247	42.35	0	2635.396919	3	879.135437	-1.958602442	-0.001720565	105.0417856
																			TVEAEAAHGTVTR	31	28178825	1xTMT6plex [N-Term]	0.985	1.379	1.366	5.789176464	77.9	0	1570.83865	3	524.284729	0.626265287	0.000327921	26.09688496
																			SIEDFAHSSFQMALSK	10	28178825	2xTMT6plex [N-Term; K16]	1.209	100		2.334496975	17.89	0	2256.173475	3	752.7290039	-0.450351563	-0.00033869	101.8806197
																			LVSGWVKPIIIGR	2	28178825	2xTMT6plex [N-Term; K7]	0.849			2.562268734	7.36	0	1896.219895	3	632.7449951	0.28318528	0.000178994	79.58134663
																			ATDFVVPGPGK	10	28178825	2xTMT6plex [N-Term; K11]	1.03	0.965	0.928	3.430617094	47.23	0	1545.9041	2	773.4503174	-6.948577454	-0.005370917	51.19250054
																			LIDDMVAQAMK	6	28178825	2xTMT6plex [N-Term; K11]	100	100	0.817	2.98871088	43.03	0	1692.94287	2	846.9733887	-1.990171263	-0.001684623	90.0714877
																			SDYLNTFEFMDK	4	28178825	2xTMT6plex [N-Term; K12]	0.815	100		2.37699151	35.53	0	1967.982486	2	984.4940186	-0.87680182	-0.000862765	87.26841259
																			DATNDQVTK	2	28178825	2xTMT6plex [N-Term; K9]	1.296			3.727936268	46.13	0	1449.794943	2	725.4014282	0.438962293	0.000318203	32.06947331
																			GWPLYLSTK	6	28178825	2xTMT6plex [N-Term; K9]	2.149	1.648	1.245	3.327253819	39.48	0	1522.903372	2	761.9550781	-0.323145913	-0.00024606	68.90681279
																			SEGGFIWACK	10	28178825	2xTMT6plex [N-Term; K10]; 1xCarbamidomethyl [C9		100		2.290626049	23.59	0	1612.85577	2	806.9309082	-0.762692966	-0.000615057	59.40719502
																			GQETSTNPIASIFAWTR	4	28178825	1xTMT6plex [N-Term]				1.59864819	15.11	0	2108.097384	2	1054.552612	0.267700809	0.00028217	100.8024557
																			KISGGSVVEMQGDEMTR	2	28178825	2xTMT6plex [N-Term; K1]; 1xOxidation [M15]	1.474			2.218570471	10.46	1	2298.183388	3	761.4001465	-1.133305556	-0.000862139	52.40927803
																			CATITPDEK	2	28178825	2xTMT6plex [N-Term; K9]; 1xCarbamidomethyl [C1	1.114			3.58439064	41.24	0	1492.808151	2	746.9073486	-0.489235254	-0.000365167	43.08396636
																			NILGGTVFR	5	28178825	1xTMT6plex [N-Term]	100			1.799440622	26.43	0	1205.720373	2	603.3630981	-1.205388057	-0.00072668	55.90408907
																			FKDIFQEIYDK	2	28178825	3xTMT6plex [N-Term; K2; K11]	1.088			2.844625711	8.44	1	2133.219904	3	711.7449951	0.247729529	0.000176154	98.72347375
																			DLAACIK	12	28178825	2xTMT6plex [N-Term; K7]; 1xCarbamidomethyl [C5	1.006	1.107	1.158	3.135351181	38.25	0	1248.738615	2	624.8720093	-1.499981894	-0.000936543	44.57473427
																			SQFEAQK	2	28178825	2xTMT6plex [N-Term; K7]	1.331			3.177722692	34.84	0	1295.735972	2	648.371521	-0.15956014	-0.000103374	37.46988983
																			IIWELIK	4	28178825	2xTMT6plex [N-Term; K7]	100	1.338		2.572081327	24.44	0	1372.89683	2	686.9516602	-0.572655922	-0.000393099	99.95809296
5453832	HYOU1	hypoxia up-regulated protein precursor	10525	NP_006380.1	33.73373373	1	24	24	109	999	111.266	5.22	1.19	1.01	1.7	1.3	Significant	0.024																
																			VAIVKPGVPMEIVLNK	6	5453832	3xTMT6plex [N-Term; K5; K16]	1.177	0.763	1.17	3.992257357	37.77	0	2394.512521	3	798.8426514	0.367649597	0.000293447	81.07370994
																			LSAASTWLEDEGVGATTVMLK	6	5453832	2xTMT6plex [N-Term; K21]	100	100	100	3.165606499	18.58	0	2637.420975	3	879.8110352	-0.918537948	-0.000807524	98.38806288
																			SLAEDFAEQPIKDAVITVPVFFNQAER	2	5453832	2xTMT6plex [N-Term; K12]	0.505			3.497503281	17.99	1	3492.877891	4	873.9754639	0.611183333	0.000533697	107.4230018
																			TVLSANADHMAQIEGLMDDVDFK	2	5453832	2xTMT6plex [N-Term; K23]				3.340159416	16.53	0	2978.500364	3	993.5023804	-2.610735527	-0.002592026	100.4735351
																			VESVFETLVEDSAEEESTLTK	6	5453832	2xTMT6plex [N-Term; K21]	100	4.714	100	3.330256224	26.86	0	2800.439187	3	934.149231	-2.159310916	-0.002015673	110.6659179
																			VEFEELCADLFER	11	5453832	1xTMT6plex [N-Term]; 1xCarbamidomethyl [C7	1.788	1.222		3.680718184	34.04	0	1885.920331	2	943.4608154	-3.16910557	-0.00298834	96.4420071
																			EVEEEPGIHSLK	4	5453832	2xTMT6plex [N-Term; K12]	1.297			3.380131245	32.96	0	1825.01075	2	913.010376	1.493494285	0.001362822	44.61685318
																			FPEHELTFDPQR	4	5453832	1xTMT6plex [N-Term]	1.117	0.871		3.266399145	33.29	0	1744.8856	3	582.2995605	-0.843487604	-0.000490596	57.72804607
																			KTPVIVTLK	6	5453832	3xTMT6plex [N-Term; K1; K9]	1.087	0.994	1.45	4.936151028	31.07	1	1686.149639	3	562.7221069	1.262106714	0.000709367	59.01740798
																			LPATEKPVLLSK	6	5453832	3xTMT6plex [N-Term; K6; K12]	1.281	1.378	1.256	2.647680759	13.39	0	1983.28211	3	661.7655029	-0.07789156	-5.15E-05	57.21480293
																			DAVITVPVFFNQAER	6	5453832	1xTMT6plex [N-Term]		100		1.33118093	14.56	0	1935.053728	2	968.0287476	-1.813666857	-0.001754771	81.38722412
																			VPGPVQQALQSAEMSLDEIEQVILVGGATR	2	5453832	1xTMT6plex [N-Term]				2.232683659	9.9	0	3364.798371	3	1122.27124	0.236775889	0.000265568	113.2266447
																			LYQPEYQEVSTEEQREEISGK	2	5453832	2xTMT6plex [N-Term; K21]	100			1.368059278	6.48	1	3000.520231	3	1000.842407	-2.520379711	-0.002520817	61.21179068
																			AHFNLDESGVLSLDR	2	5453832	1xTMT6plex [N-Term]	1.424			2.533597946	8.88	0	1901.991856	3	634.6688232	0.031780663	2.01E-05	75.34384519
																			EVQYLLNK	4	5453832	2xTMT6plex [N-Term; K8]	100	100		2.327759743	22.33	0	1464.882636	2	732.9447021	-0.34720401	-0.000254307	55.80344319
																			VQEVLLK	6	5453832	2xTMT6plex [N-Term; K7]	1.311	100	0.919	2.840893269	37.98	0	1286.844794	2	643.9261475	0.174062852	0.000111996	53.66749153
																			DAVVYPILVEFTR	8	5453832	1xTMT6plex [N-Term]	0.01	100		1.711877823	10.96	0	1750.994088	2	875.9997559	-1.058199604	-0.000926451	92.4794762
																			AVGKEELGK	6	5453832	3xTMT6plex [N-Term; K4; K9]	0.881	0.465		2.495739698	25.3	1	1618.014268	2	809.5108032	0.038105543	3.08E-05	43.43001844
																			VLQLINDNTATALSYGVFR	3	5453832	1xTMT6plex [N-Term]			100	0.828865826	2.05	0	2324.281162	2	1162.642822	-1.201945271	-0.001396829	108.740885
																			LQDLTLR	6	5453832	1xTMT6plex [N-Term]	1.454	1.333	100	2.769068241	40.74	0	1087.667275	2	544.3366089	-1.226199035	-0.000666848	51.52379149
																			VKPFVVR	4	5453832	2xTMT6plex [N-Term; K2]	1.149	1.412		2.994520664	2.71	0	1302.866199	3	434.9602356	-0.034518426	-1.50E-05	48.55879805
																			GDATPEGEK	2	5453832	2xTMT6plex [N-Term; K9]				1.853187561	6.07	0	1361.731281	2	681.368042	-1.816228841	-0.001236608	25.52375084
																			GVGDSFK	4	5453832	2xTMT6plex [N-Term; K7]	1.318	1.541		2.696947336	23.61	0	1167.677395	2	584.3426514	0.540760982	0.000315717	48.20853286
																			LIPEMDQIFTEVEMTTLEK	1	5453832	2xTMT6plex [N-Term; K19]				0.662638247		0	2725.444412	2	1363.226807	0.706061456	0.000962166	117.2520076
41349456	PREP	prolyl endopeptidase	5550	NP_002717.3	40.28169014	1	20	20	90	710	80.648	5.86	1.06	1.21	1.18	1.15	Significant	0.0016																
																			YTIGHAWTTDYGCSDSK	14	41349456	2xTMT6plex [N-Term; K17]; 1xCarbamidomethyl [C13	1.88	1.407	100	4.959465027	55.29	0	2420.159281	3	807.3900757	-1.490583387	-0.001202483	53.89534197
																			LWYCDLQQESSGIAGILK	2	41349456	2xTMT6plex [N-Term; K18]; 1xCarbamidomethyl [C4	0.875			2.969810486	42.44	0	2539.363066	3	847.1253052	-0.671082713	-0.000568041	104.188505
																			LIDNFEGEYDYVTNEGTVFTFK	6	41349456	2xTMT6plex [N-Term; K22]	0.649	1.479	0.606	4.313333988	35.39	0	3059.529005	3	1020.513062	-1.429432334	-0.001457797	101.5602901
																			LYYHVLGTDQSEDILCAEFPDEPK	4	41349456	2xTMT6plex [N-Term; K24]; 1xCarbamidomethyl [C16			1	5.49134922	31.66	0	3297.638966	4	825.164978	-0.26801922	-0.000220958	88.09043419
																			ICDPYAWLEDPDSEQTK	7	41349456	2xTMT6plex [N-Term; K17]; 1xCarbamidomethyl [C2	0.739	100		2.800822973	20.05	0	2525.227026	3	842.4133301	-0.629407023	-0.000529799	82.94014786
																			DETAVQDYHGHK	2	41349456	2xTMT6plex [N-Term; K12]		0.63		3.972779036	20.04	0	1857.949546	3	619.9874268	-0.979249714	-0.000606466	29.50441015
																			VLVPEHEKDVLEWIACVR	4	41349456	2xTMT6plex [N-Term; K8]; 1xCarbamidomethyl [C16		100	1.344	4.928992748	10.42	1	2650.479099	4	663.3753052	0.110030343	7.29E-05	93.44832057
																			GGGEYGETWHK	4	41349456	2xTMT6plex [N-Term; K11]	1.207	1.023		3.609341145	24.34	0	1678.858941	3	560.2910767	-0.157189818	-8.80E-05	40.27381146
																			SDGTETSTNLHQK	6	41349456	2xTMT6plex [N-Term; K13]	0.504	0.994	100	3.603876591	15.29	0	1875.98124	3	625.9994507	1.363766719	0.0008528	25.1158189
																			WMGGAELSDDGR	6	41349456	1xTMT6plex [N-Term]	100	100		2.647388697	38.58	0	1522.715758	2	761.8617554	0.31285037	0.000238191	49.21561915
																			DVLEWIACVR	2	41349456	1xTMT6plex [N-Term]; 1xCarbamidomethyl [C8				2.046536446	23.36	0	1489.803451	2	745.4052734	-0.121267674	-9.03E-05	95.64171064
																			VIEEVSDMFAFIAR	5	41349456	1xTMT6plex [N-Term]		100	0.463	1.970497727	24.74	0	1855.982537	2	928.4954224	0.55556372	0.000515558	112.3586305
																			VFLDPNILSDDGTVALR	2	41349456	1xTMT6plex [N-Term]				1.695630193	10.95	0	2074.138186	2	1037.572144	-0.566664571	-0.00058767	92.44685121
																			LPEADDIQYPSMLLLTADHDDR	8	41349456	1xTMT6plex [N-Term]		100	100	2.054544687	10.7	0	2757.360276	3	919.78479	-7.419767736	-0.006819658	83.35242397
																			ELPDVLER	2	41349456	1xTMT6plex [N-Term]	1.065			3.213054895	29.42	0	1199.683319	2	600.34552	0.370638697	0.000222325	64.11534877
																			GYAFSEDGEYFAYGLSASGSDWVTIK	2	41349456	2xTMT6plex [N-Term; K26]				2.117664814	4.09	0	3278.593396	3	1093.536255	0.248770841	0.000271873	108.2423237
																			SNFLVLCYLHDVK	3	41349456	2xTMT6plex [N-Term; K13]; 1xCarbamidomethyl [C7		100		1.956637859	11.5	0	2066.150889	3	689.3873291	-1.672198933	-0.001151672	79.04942824
																			IPMFIVHK	3	41349456	2xTMT6plex [N-Term; K8]	0.792	0.969		1.628957748	15.97	0	1442.895784	3	481.6367188	-0.125476838	-6.04E-05	77.59069492
																			VINIDFR	4	41349456	1xTMT6plex [N-Term]	100			1.89783752	20.04	0	1105.65671	2	553.331604	-0.704379741	-0.000389401	61.39593702
																			WMGGAELSDDGR	2	41349456	1xTMT6plex [N-Term]; 1xOxidation [M2]		2.04		1.952461839	28.31	0	1538.710673	2	761.8617554	0.31285037	0.000238191	49.21561915
																			GGILANK	2	41349456	2xTMT6plex [N-Term; K7]			1.198	2.595683575	24.91	0	1130.729765	2	565.8685913	0.125022965	7.07E-05	44.82529855
22027538	PDCD6IP	programmed cell death 6-interacting protein isoform	10015	NP_037506.2	29.26267281	1	24	24	102	868	95.963	6.52	1.08	1.01	1.04	1.04	Significant	0.00038																
																			TSEVDLAKPLVK	2	22027538	3xTMT6plex [N-Term; K8; K12]	1.281			3.745419264	31.93	0	1987.240639	3	663.085083	0.028518659	1.89E-05	63.30165004
																			STPVNVPISQK	4	22027538	2xTMT6plex [N-Term; K11]	1.251	1.101		3.996111631	54.04	0	1627.978327	2	814.4919434	-1.054916531	-0.000858691	51.27252918
																			EPSAPSIPTPAYQSSPAGGHAPTPPTPAPR	4	22027538	1xTMT6plex [N-Term]	1.094	0.846		5.25528574	26.52	0	3165.616642	3	1055.87561	-1.378554578	-0.001454658	45.96265096
																			LLDEEEATDNDLR	6	22027538	1xTMT6plex [N-Term]	1.161	1.328	100	3.3630867	46.54	0	1761.870403	2	881.4391479	0.349519713	0.000307904	50.45208361
																			EVFPVLAAK	6	22027538	2xTMT6plex [N-Term; K9]	1.42	1.503	1.404	3.391156435	54.6	0	1431.897558	2	716.4516602	-1.057636764	-0.000757214	77.26084375
																			AVQADGQVK	6	22027538	2xTMT6plex [N-Term; K9]	1.026	1.025	1.004	3.799787283	65.58	0	1373.815285	2	687.4115601	0.406515485	0.000279239	31.58249415
																			SVNFDMTSK	4	22027538	2xTMT6plex [N-Term; K9]	1.044	100		4.439923763	57.89	0	1486.797586	2	743.9020386	-0.528304303	-0.000392741	60.43446749
																			LALASLGYEK	4	22027538	2xTMT6plex [N-Term; K10]	1.492	1.348		3.260977745	44.6	0	1522.924501	2	761.9656982	-0.250311571	-0.000190603	72.96901713
																			LQHAAELIK	10	22027538	2xTMT6plex [N-Term; K9]	1.061	0.889	0.953	3.725725651	50.16	0	1480.92517	3	494.3127747	-0.944737397	-0.000466362	45.27914442
																			SVIEQGGIQTVDQLIK	3	22027538	2xTMT6plex [N-Term; K16]		100		1.890503168	11.63	0	2186.279654	2	1093.642212	-1.146665693	-0.001253466	80.5085498
																			DTIVLLCKPEPELNAAIPSANPAK	6	22027538	3xTMT6plex [N-Term; K8; K24]; 1xCarbamidomethyl [C7				2.147508383	4.7	0	3248.853146	3	1083.619751	-2.599760572	-0.002815413	75.1190881
																			LANQAADYFGDAFK	6	22027538	2xTMT6plex [N-Term; K14]	100	100	100	2.375815868	26.42	0	1989.048198	2	995.0211182	-6.655495774	-0.006619051	78.58788252
																			EILDESLR	6	22027538	1xTMT6plex [N-Term]	0.974	1.064	1.041	3.490617752	45.69	0	1203.678233	2	602.3395996	-5.242905517	-0.003155386	48.51224452
																			YDEYVNVK	6	22027538	2xTMT6plex [N-Term; K8]	100	100	2.528	2.908241987	41.25	0	1487.814616	2	744.411438	0.660752053	0.000491538	46.26224229
																			ELPELLQR	2	22027538	1xTMT6plex [N-Term]	0.984			3.018044233	30.22	0	1226.730603	2	613.8689575	0.028611875	1.75E-05	68.83465499
																			SLLSNLDEVK	2	22027538	2xTMT6plex [N-Term; K10]				1.485105991	5.94	0	1575.935794	2	788.4702759	-1.598302543	-0.001259411	63.40396718
																			FYNELTEILVR	4	22027538	1xTMT6plex [N-Term]				1.535554767	10.54	0	1625.910024	2	813.4575806	-1.315913646	-0.001069779	92.47178079
																			DLQQSIAR	2	22027538	1xTMT6plex [N-Term]	1.161			2.724594355	21.13	0	1159.663252	2	580.3361206	1.476757097	0.00085627	43.44121233
																			MKDAIIAK	2	22027538	3xTMT6plex [N-Term; K2; K8]		1.176	1.136	2.922065496		1	1577.006346	3	526.34021	-0.170872608	-8.98E-05	43.7555208
																			FTDLFEK	4	22027538	2xTMT6plex [N-Term; K7]	100	100		2.260696888	27.33	0	1357.776774	2	679.3917236	-0.444590294	-0.000301827	99.98149935
																			YYDQICSIEPK	5	22027538	2xTMT6plex [N-Term; K11]; 1xCarbamidomethyl [C6				1.24136436	5.53	0	1873.977007	2	937.4907837	-1.449488002	-0.001358154	57.80575769
																			SLLSNLDEVKK	2	22027538	3xTMT6plex [N-Term; K10; K11]				1.217936277	3.81	1	1933.193689	3	645.0687866	-0.973677252	-0.000627436	71.82513234
																			KTSEVDLAKPLVK	2	22027538	4xTMT6plex [N-Term; K1; K9; K13]	0.842		0.97	2.819816828	1.68	1	2344.498534	4	586.8792725	-1.396501726	-0.000818524	46.10529384
																			KQEGLLK	4	22027538	3xTMT6plex [N-Term; K1; K7]	1.224	1.026		2.808373213	12.18	1	1502.987325	3	501.6663208	-1.940181136	-0.000972023	47.15602837
108773810	LARS	leucine--tRNA ligase, cytoplasmic isoform	51520	NP_064502.9	27.38095238	1	27	27	111	1176	134.379	7.3	1.52	0.9	1.36	1.26	Significant	0.021																
																			LALADAGDTVEDANFVEAMADAGILR	6	108773810	1xTMT6plex [N-Term]		0.912	1.167	3.916736841	44.66	0	2877.450153	3	959.8183594	-3.346200451	-0.003209508	103.0516519
																			TPISEHAVFNVDLMSK	3	108773810	2xTMT6plex [N-Term; K16]	0.979	0.01		4.329220772	41.22	0	2246.22551	3	749.4138184	0.619576315	0.000463903	82.54937816
																			FSEAEHWLDYFPPLAIQDLK	7	108773810	2xTMT6plex [N-Term; K20]	100	100	100	2.820941448	9.21	0	2877.522738	3	959.8441162	-1.717570159	-0.001647449	102.174646

Bhat et al,2017. Cigarette smoke and chewing tobacco alter expression of different sets of miRNAs in oral keratinocytes

Supplementary Table S2(A). List of all proteins and peptides identified in OKF6/TERT1 cells chronically treated with chewing tobacco using Sequest and Mascot search algorithms

Accession	Gene_Symbol	Description	Gene_ID	Refseq_Accession	Coverage	Proteins	#Unique_Peptides	#Peptides	#PSMS	#AAs	MW [kDa]	calc. pI	OKF6-STE-6M/OKF6P_R1	OKF6-STE-6M/OKF6P_R2	OKF6-STE-6M/OKF6P_R3	Median fold change	Status	P-value (6M)	Sequence	#PSMs	Protein Accessions	Modifications	OKF6-STE-6M/OKF6P_R1	OKF6-STE-6M/OKF6P_R2	OKF6-STE-6M/OKF6P_R3	Xcorr	Ionscore	# Missed Cleavages	Theo. MH+ [Da]	Charge	m_z	DeltaM [ppm]	Deltam/z [Da]	RT [min]
																			VFASELNAGIIK	6	108773810	2xTMT6plex [N-Term; K12]	1.915	100	0.01	2.649376869	41.1	0	1720.040928	2	860.5239258	-0.205098317	-0.000176389	77.09662475
																			SLGLSDEEIVK	6	108773810	2xTMT6plex [N-Term; K11]		0.786	100	3.011645079	47.27	0	1647.956923	2	824.4822998	0.242548436	0.000199855	52.62307863
																			VIASELGSMPELK	6	108773810	2xTMT6plex [N-Term; K13]	2.905	1.956	100	3.002017498	45.07	0	1832.060342	2	916.5359497	2.336349935	0.002140167	68.11574767
																			NLETFCEETR	2	108773810	1xTMT6plex [N-Term]; 1xCarbamidomethyl [C6	1.354			4.084541321	47.54	0	1527.731074	2	764.3688965	-0.364849062	-0.000278696	54.30950021
																			GTGVVTSVPSDSPDDIAALR	4	108773810	1xTMT6plex [N-Term]			100	2.468725681	27	0	2186.150207	2	1093.577515	-1.122646155	-0.001227137	55.69297782
																			GFYEGIMLVDGFK	3	108773810	2xTMT6plex [N-Term; K13]				1.745624304	21.76	0	1934.049778	2	967.5267944	-1.791863298	-0.001732776	100.8952872
																			VDIGDTIIYLVH	4	108773810	1xTMT6plex [N-Term]				2.116371393	30.88	0	1586.899125	2	793.9533081	0.135201371	0.000107275	104.9149883
																			QEFEFWYPVDLR	6	108773810	1xTMT6plex [N-Term]	100		100	2.201314688	21.09	0	1857.937301	2	929.4713135	-1.050119869	-0.000975528	89.84520057
																			VFEVNASNLEK	6	108773810	2xTMT6plex [N-Term; K11]	1.358	100	100	3.120482206	53.16	0	1707.968157	2	854.494812	8.308540989	0.007095362	64.99648864
																			EVWDYVFFK	10	108773810	2xTMT6plex [N-Term; K9]	1.41	1.166	100	3.351023912	23.04	0	1690.924501	2	845.9655151	-0.442016519	-0.000373708	103.2551667
																			FDDPLLGPR	3	108773810	1xTMT6plex [N-Term]	1.727			3.69751358	26.6	0	1258.699303	2	629.8527222	-0.902109074	-0.000567742	73.21275045
																			ILDLQLEFDEK	2	108773810	2xTMT6plex [N-Term; K11]	100			1.667221427	2.83	0	1821.040987	2	911.0241089	-0.025291338	-2.30E-05	92.65304893
																			FASEAEDK	2	108773810	2xTMT6plex [N-Term; K8]			1.345	2.84630537	31.53	0	1354.725467	2	677.8665771	0.303180892	0.000205363	42.41587451
																			SFITTDVNPYYDSFVR	5	108773810	1xTMT6plex [N-Term]		100	100	1.856857181	11.34	0	2153.075251	2	1077.042236	0.903273701	0.000972408	83.9481763
																			VLEPYPSK	2	108773810	2xTMT6plex [N-Term; K8]	1.516			2.867700815	29.67	0	1390.834624	2	695.9213257	0.540134086	0.000375619	50.72664534
																			ELAVEGMHR	2	108773810	1xTMT6plex [N-Term]			1.161	2.867044926	17.41	0	1270.677522	2	635.84198	-0.659930665	-0.00041928	35.75545187
																			EMVANWDSLR	7	108773810	1xTMT6plex [N-Term]	100			2.143066406	18.39	0	1449.735765	2	725.3701172	-1.93636321	-0.001403607	56.38005218
																			LYTWVEWVK	4	108773810	2xTMT6plex [N-Term; K9]			100	2.288598537	15.04	0	1681.971786	2	841.4904175	1.053954027	0.00088636	101.2450095
																			ELMGEEILGASLSAPLTSYK	2	108773810	2xTMT6plex [N-Term; K20]				1.326728106	2.36	0	2567.404262	2	1284.197998	-6.053840635	-0.007771328	94.75769855
																			TEDIIIK	2	108773810	2xTMT6plex [N-Term; K7]	1.701			2.678140163	27.94	0	1289.808074	2	645.4081421	0.723471737	0.00046657	58.51353176
																			YTIYSPK	2	108773810	2xTMT6plex [N-Term; K7]			1.103	2.756255627	28.34	0	1329.78186	2	665.3949585	0.587067858	0.000390336	52.89242264
																			YQWGIMK	4	108773810	2xTMT6plex [N-Term; K7]	0.655		100	2.195808649	9.56	0	1383.785899	2	692.395752	-1.208260433	-0.000835987	59.0581166
																			EKLDQLK	2	108773810	3xTMT6plex [N-Term; K2; K7]	1.542			2.10986805	9.42	1	1560.992804	3	521.0024414	-0.021467908	-1.12E-05	49.57236701
																			VMPFVAMIK	3	108773810	2xTMT6plex [N-Term; K9]	0.46	100		2.515784264	14.65	0	1493.89882	2	747.4529419	-0.142801463	-0.000106665	91.34420051
40316915	RNPEP	aminopeptidase B isoform a	6051	NP_064601.3	39.84615385	1	20	20	90	650	72.549	5.74	1.06	1.22	1.07	1.12	Significant	0.0021															
																			QHMDITGEENPLNK	2	40316915	2xTMT6plex [N-Term; K14]			1.005	4.82578373	39.8	0	2084.08466	3	695.3662109	-0.278308032	-0.000193339	52.58588478
																			GLSGTAVLDLR	5	40316915	1xTMT6plex [N-Term]	0.891			3.503222704	67.86	0	1330.789181	2	665.8986206	0.588997077	0.000391915	91.49111836
																			IEPGVDPDDTYNETPYEK	6	40316915	2xTMT6plex [N-Term; K18]	2.006	1.557		4.294690132	46.65	0	2540.24445	3	847.4195557	-0.132450959	-0.000112153	62.23867014
																			YSALIEVPDGFTAVMSASTWEK	4	40316915	2xTMT6plex [N-Term; K22]	1.015			3.561282158	24.9	0	2860.484303	3	954.1677246	1.509296212	0.001439106	104.1388981
																			AIEAVAISPWK	13	40316915	2xTMT6plex [N-Term; K11]	1.056	1.26		4.136819839	52.14	0	1642.993249	2	822.0008545	0.720060399	0.000591527	90.47524646
																			RPLHSAQAVDVASASNFR	6	40316915	1xTMT6plex [N-Term]	1.179	1.135		4.123723507	15.7	0	2155.156964	3	719.0575562	0.534071091	0.000383669	35.73666114
																			VDIIPGFEFDR	5	40316915	1xTMT6plex [N-Term]	1.055	1.468		3.322439432	33.23	0	1536.82596	2	768.9160767	-0.704985285	-0.00054172	91.4719284
																			ETFASTASQLHSNVVNYVQQIVAPK	4	40316915	2xTMT6plex [N-Term; K25]			100	3.38635993	29.64	0	3189.730833	3	1063.914429	-0.658327394	-0.000699962	95.12186924
																			LDSHPCLEVTAAALR	4	40316915	1xTMT6plex [N-Term]; 1xCarbamidomethyl [C6	1.15	100		4.360663414	24.57	0	1882.005398	3	628.0066528	0.003871452	2.43E-06	64.15703378
																			RVDIIPGFEFDR	2	40316915	1xTMT6plex [N-Term]	1.252			4.072318077	24.92	1	1692.927071	3	564.9798584	-1.210421123	-0.000683052	71.28325093
																			KKPFVYTQGQAVLNR	4	40316915	3xTMT6plex [N-Term; K1; K2]	0.671		0.904	4.074715137	14.82	1	2436.46941	3	812.8280029	0.018645448	1.51E-05	45.10658342
																			GFCFVSYLAHLVGDQDQFDSFLK	2	40316915	2xTMT6plex [N-Term; K23]; 1xCarbamidomethyl [C3				3.900723696	28.79	0	3151.596314	3	1051.211548	7.544349695	0.007925582	116.5528488
																			VWAEPCLIDAAK	6	40316915	2xTMT6plex [N-Term; K12]; 1xCarbamidomethyl [C6	100	1.225	100	2.53765583	21.56	0	1831.018812	2	916.0141602	1.218628932	0.001115666	69.27011417
																			TYQLVYFLDK	4	40316915	2xTMT6plex [N-Term; K10]	100			2.168055534	23.96	0	1748.00348	2	874.5058594	0.550645357	0.000481265	98.84965755
																			NDHQEDFWK	2	40316915	2xTMT6plex [N-Term; K9]	0.772			2.717985392	24.92	0	1676.843291	3	559.6185303	-1.343595851	-0.000751	53.18799443
																			AFFPCFDTPAVK	4	40316915	2xTMT6plex [N-Term; K12]; 1xCarbamidomethyl [C5	0.983	1.277		1.715423465	9.48	0	1857.997349	2	929.5006714	-1.766696042	-0.001641258	76.35545271
																			SPLPPGNVK	9	40316915	2xTMT6plex [N-Term; K9]		1.031	1.229	3.450391769	42.1	0	1366.845857	2	683.9268188	0.368853086	0.000252083	38.29160496
																			WGQIVLK	2	40316915	2xTMT6plex [N-Term; K7]		0.567		2.368926048	27.7	0	1301.834564	2	651.4206543	-0.408689602	-0.000266023	77.57740697
																			AYVHEFK	4	40316915	2xTMT6plex [N-Term; K7]	1.024			3.353961229	27.63	0	1351.777443	2	676.3911133	-1.844310624	-0.001246549	40.73009171
																			LFGPYVWGR	2	40316915	1xTMT6plex [N-Term]	0.571			2.011839151	13.89	0	1323.741108	2	662.3736572	-0.808811382	-0.000535328	88.94336164
4502107	ANXA5	annexin A5	308	NP_001145.1	65.625	1	19	20	179	320	35.914	5.05	1.16	1.29	1.36	1.27	Significant	0.0021															
																			QVYEEEYGSSLEDDVVGDTSGYYQR	14	4502107	1xTMT6plex [N-Term]	100	2.345	0.01	3.591131449	27.52	0	3117.401014	3	1039.803345	-1.774892062	-0.00184435	73.29850361
																			GTVTDFPGFDER	16	4502107	1xTMT6plex [N-Term]	1.146	1.311	1.481	4.145593166	64.24	0	1569.774653	2	785.3907471	-0.277281439	-0.000217635	68.89270441
																			GLGTDEESILTLLTSR	14	4502107	1xTMT6plex [N-Term]	1.329	2.28	1.311	3.688187838	59.47	0	1934.064352	2	967.5349731	-0.869940514	-0.00084126	107.0032096
																			DPDAGIDEAQVEQDAQALFQAGELK	15	4502107	2xTMT6plex [N-Term; K25]	2.039	1.874	5.915	4.620543957	36.84	0	3116.578808	3	1039.528076	-2.930474388	-0.003044351	96.1352418
																			GAGTDDHTLIR	8	4502107	1xTMT6plex [N-Term]	1.404	1.31		3.911023378	50.99	0	1384.738208	2	692.8726807	-0.088877359	-6.15E-05	33.04500518
																			ETSGNLEQLLLAVVK	7	4502107	2xTMT6plex [N-Term; K15]				3.015590429	24.25	0	2072.236727	2	1036.621704	-0.287214712	-0.000297588	101.0121543
																			NFATSLYSMIK	4	4502107	2xTMT6plex [N-Term; K11]		100	0.779	3.08520484	38.67	0	1732.970799	2	866.987915	-1.295931747	-0.001122906	90.49536987
																			DLLDDLKSELTGK	9	4502107	3xTMT6plex [N-Term; K7; K13]	0.822		100	3.167560816	24.01	1	2134.257411	3	712.0899658	-0.968505041	-0.000689013	96.1671055
																			VLTEIIASR	22	4502107	1xTMT6plex [N-Term]	0.623	3.162	0.804	3.063454151	39.34	0	1230.761903	2	615.8846436	0.086978135	5.35E-05	77.82761274
																			LYDAYELK	13	4502107	2xTMT6plex [N-Term; K8]	0.94	1.073	0.983	3.355526924	31.33	0	1472.840103	2	736.9232788	-0.557951139	-0.000410886	65.921543
																			FITIFGTR	10	4502107	1xTMT6plex [N-Term]	1.342	1.947		2.934346914	33.41	0	1183.70366	2	592.3551636	-0.515197834	-0.000304921	87.92997298
																			QEISAAFK	4	4502107	2xTMT6plex [N-Term; K8]	1.368		1.312	3.732514381	53.14	0	1351.798572	2	676.3989258	-5.916131045	-0.003998709	45.92606468
																			GDTSGDYK	4	4502107	2xTMT6plex [N-Term; K8]	1.395	1.434		2.668554306	19.12	0	1300.678517	2	650.8429565	0.092025767	5.98E-05	27.65138583
																			DLIDDLK	36	4502107	2xTMT6plex [N-Term; K7]	1.329	2.178	1.952	2.906009436	38.83	0	1289.771689	2	645.3887939	-1.068118789	-0.000688815	67.18816778
																			SIPAYLAETLYYAMK	6	4502107	2xTMT6plex [N-Term; K15]	100	0.734	100	2.293182135	15.66	0	2192.207735	2	1096.606934	-0.521981782	-0.000572146	108.8152072
																			MLVVLLQANR	3	4502107	1xTMT6plex [N-Term]	100			1.330350518	20.96	0	1385.850007	2	693.4281006	-0.78102112	-0.000541189	81.92733457
																			ADAETLR	6	4502107	1xTMT6plex [N-Term]	1.245		1.558	2.826098204	33.01	0	1004.55739	2	502.7822876	-0.091069647	-4.57E-05	31.86049247
																			KNFATSLYSMIK	2	4502107	3xTMT6plex [N-Term; K1; K12]	100			2.324007034	4.85	1	2090.228694	3	697.4145508	0.193693518	0.000134955	100.2685703
																			GDTSGDYKK	2	4502107	3xTMT6plex [N-Term; K8; K9]	1.146	1.084		2.091435432	12.88	1	1657.936412	3	553.3166504	-0.611487942	-0.000337936	28.96570481
																			GAGTNEK	2	4502107	2xTMT6plex [N-Term; K7]				1.762646794	22.23	0	1134.651908	2	567.8291626	-0.757602117	-0.000429807	22.09904969
24234747	ILF2	interleukin enhancer-binding factor 2 isoform 1	3608	NP_004506.2	56.41025641	1	13	13	68	390	43.035	5.26	1.17	1.64	1.25	1.35	Significant	0.011															
																			AQDPSEVLTMLTNETGFEISSSDATVK	4	24234747	2xTMT6plex [N-Term; K27]; 1xOxidation [M10			1.303	4.234993935	52.98	0	3344.681953	3	1110.2323	-1.409521497	-0.001563952	101.0282091
																			AQDPSEVLTMLTNETGFEISSSDATVK	5	24234747	2xTMT6plex [N-Term; K27]			1.213	5.851546288	71.91	0	3328.687038	3	1110.2323	-1.409521497	-0.001563952	101.0282091
																			ILGQEGDASYLASEISTWDGVIVTPSEK	4	24234747	2xTMT6plex [N-Term; K28]			1.772	5.423271656	58.1	0	3423.793552	3	1141.93457	-1.283460125	-0.001464767	102.6439545
																			WFEENASQSTVK	2	24234747	2xTMT6plex [N-Term; K12]	1.489			3.050445795	52.61	0	1883.990349	2	942.4990234	0.223809533	0.000210827	67.69604033
																			KEGEEEENTEEPPQGEEEESMETQE	3	24234747	2xTMT6plex [N-Term; K1]		0.01		2.892566919	11.59	1	3510.511377	4	882.3799438	-2.366900463	-0.002086722	43.58421413
																			KEGEEEENTEEPPQGEEEESMETQE	2	24234747	2xTMT6plex [N-Term; K1]; 1xOxidation [M22]		100		2.578496933	28.65	1	3526.506292	4	882.3799438	-2.366900463	-0.002086722	43.58421413
																			KLDPELHLDIK	8	24234747	3xTMT6plex [N-Term; K1; K11]	1.106	1.077	0.906	3.615728855	15.93	1	2008.240973	4	502.8153076	-0.783213404	-0.00039322	67.91296446
																			VKPAPDETSFSEALLK	4	24234747	3xTMT6plex [N-Term; K2; K16]	1.153	1.896		4.498105049	32.05	0	2419.405138	3	807.1394653	-0.535368751	-0.000431758	60.50934148
																			NQDLAPNSAEQASILSLVTK	5	24234747	2xTMT6plex [N-Term; K20]	5.076			2.643875122	20.57	0	2557.423752	3	853.145752	-0.410319418	-0.000349787	86.15027636
																			QPLALNVAYR	7	24234747	1xTMT6plex [N-Term]	1.228	1.915	1.302	3.549619198	30.8	0	1373.810251	2	687.4077759	-1.437936707	-0.000987726	60.65827742
																			VLQSALAAIR	4	24234747	1xTMT6plex [N-Term]	100	1.011		3.21716857	42.82	0	1270.804437	2	635.9049683	-1.398371386	-0.000888528	76.3164527
																			ILITTVPPNLR	9	24234747	1xTMT6plex [N-Term]	1.031			3.138412237	23.34	0	1465.930366	2	733.4677124	-1.512681205	-0.001108743	70.28190478
																			GGPGGGFRPFVPHIPFDFYLCEMAFPR	3	24234747	1xTMT6plex [N-Term]; 1xCarbamidomethyl [C21	0.01		100	1.56694603	2.33	0	3297.632767	4	825.1623535	-1.571620573	-0.001295657	96.58374775
																			AYEKPPEK	5	24234747	3xTMT6plex [N-Term; K4; K8]	1.142	1.838		1.527038693	6.5	0	1648.987719	3	550.3342285	0.250588207	0.000137739	39.4510755
																			ILPTLEAVAALGNK	3	24234747	2xTMT6plex [N-Term; K14]			0.01	1.811119437	7.74	0	1868.162105	2	934.5836182	-1.148557649	-0.001072846	100.9807757
19743813	ITGB1	integrin beta-1 isoform 1A precursor	3688	NP_002202.2	25.43859649	1	16	16	121	798	88.357	5.39	1.15	1.19	1.13	1.16	Significant	0.00023															
																			SAVTTVVNPK	12	19743813	2xTMT6plex [N-Term; K10]	1.148	1.122	1.073	4.334511757	56.06	0	1473.9041	2	737.4554443	-0.331044689	-0.000243964	36.79431468
																			HCECSTDEVNSEDMDAYCR	14	19743813	1xTMT6plex [N-Term]; 3xCarbamidomethyl [C2; C4; C18	1.37	1.298	1.264	6.469047546	72.93	0	2607.005352	3	869.6743164	1.167438627	0.001014506	24.75923626
																			HCECSTDEVNSEDMDAYCR	13	19743813	1xTMT6plex [N-Term]; 3xCarbamidomethyl [C2; C4; C18]; 1xOxidation [M14	1.443	1.587	1.446	6.056850433	56.48	0	2623.000267	3	869.6743164	1.167438627	0.001014506	24.75923626
																			DKLPQPVQPDPVSHCK	10	19743813	3xTMT6plex [N-Term; K2; K16]; 1xCarbamidomethyl [C15	1.1	1.079	1.024	5.866259575	25.07	1	2532.42114	3	844.8106689	-1.455498063	-0.001228645	49.1329118
																			LLVFSTDAGFHFAGDGK	12	19743813	2xTMT6plex [N-Term; K17]	1.323	1.365	2.056	4.713869095	54.32	0	2240.211575	3	747.4086304	-0.105693909	-7.89E-05	91.12765431
																			RAEDYPIDLYYLMDLSYSMK	2	19743813	2xTMT6plex [N-Term; K20]			100	3.851682186	32.69	1	2944.487674	3	982.1656494	-1.792871425	-0.001759696	104.6550184
																			LSENNIQTIFAVTEEFQPVYK	2	19743813	2xTMT6plex [N-Term; K21]	1.311			2.993871689	11.24	0	2928.575895	3	976.8614502	-2.082262601	-0.002032688	108.4936868
																			TVMPYISTTPAK	4	19743813	2xTMT6plex [N-Term; K12]	0.441			3.356223822	45.3	0	1767.012664	2	884.0098267	-0.162618918	-0.000143675	60.2020602
																			SLGTDLMNEMR	4	19743813	1xTMT6plex [N-Term]	1.634	100		3.639528036	51.82	0	1495.744615	2	748.3753052	-0.856642521	-0.000640659	61.27739792
																			FCECDNFNCDR	10	19743813	1xTMT6plex [N-Term]; 3xCarbamidomethyl [C2; C4; C9	1.721	1.351	1.2	4.361308098	53.22	0	1765.692991	2	883.348999	-1.285383776	-0.001134797	43.63178914
																			LSEGVTISYK	8	19743813	2xTMT6plex [N-Term; K10]	1.343			4.047717571	49.48	0	1554.91433	2	777.9605713	-0.298609297	-0.000232156	65.76985943
																			GEVFNELVGK	6	19743813	2xTMT6plex [N-Term; K10]	1.016		1.175	4.010594368	53.12	0	1549.899015	2	775.4456177	-9.714083496	-0.007527924	64.27948707
																			WDTGENPIYK	6	19743813	2xTMT6plex [N-Term; K10]	1.245	2.516	1.345	3.330152035	40.24	0	1680.899743	2	840.9535522	0.050605152	4.25E-05	63.21566207
																			GICECGVCK	4	19743813	2xTMT6plex [N-Term; K9]; 3xCarbamidomethyl [C3; C5; C8	1.058	0.917		4.171538353	37.65	0	1540.768612	2	770.8880005	0.073052217	5.63E-05	37.66850987
																			GCPPDDIENPR	8	19743813	1xTMT6plex [N-Term]; 1xCarbamidomethyl [C2	1.165	1.1	1.18	4.131980896	59.14	0	1498.715758	2	749.8613281	-0.252496177	-0.00018921	36.6385216
																			IGFGSFVEK	4	19743813	2xTMT6plex [N-Term; K9]			0.01	1.950415611	17.63	0	1441.845523	2	721.425415	-1.365667711	-0.000984541	67.32775769
																			DDLENVK	2	19743813	2xTMT6plex [N-Term; K7]	1.283			2.979654789	39.67	0	1290.730552	2	645.8696289	1.107002927	0.000714421	41.64482302
91208426	PRPF8	pre-mRNA-processing-splicing factor 8	10594	NP_006436.3	13.10492505	1	24	24	90	2335	273.427	8.84	1.42	1.45	1.04	1.3	Significant	0.01															
																			EQSQLTATQTR	6	91208426	1xTMT6plex [N-Term]	1.327	1.81	1.548	4.482305527	51.02	0	1491.796451	2	746.4016113	-0.338393183	-0.000252407	25.50518053
																			TNHIYVSSDDIK	6	91208426	2xTMT6plex [N-Term; K12]	1.434	1.394	1.247	4.110084534	42.68	0	1850.005999	3	617.3404541	0.438049762	0.000270132	40.0013721
																			EVGIEFMDLYSHLVPVYDVEPLEK	4	91208426	2xTMT6plex [N-Term; K24]		1.059		5.389636656	64.61	0	3279.726324	3	1093.911499	-1.945415275	-0.00212681	110.3198933
																			EMEPLGWIHTQPNESPQLSPQDVTTHAK	4	91208426	2xTMT6plex [N-Term; K28]				2.602050781	7.58	0	3628.847001	4	907.9673462	0.152551382	0.000138396	64.97869413
																			EELGLIEQAYDNPHEALSR	2	91208426	1xTMT6plex [N-Term]				1.799647927	22.94	0	2413.219683	3	805.0767822	-1.61195035	-0.001296663	77.40193723
																			GMLDPLEVHLLDFPNIVIK	4	91208426	2xTMT6plex [N-Term; K19]	100	100		2.723665953	30.2	0	2621.514087	3	874.508667	-1.00693849	-0.000879901	107.052616
																			LVVDSHVQYR	6	91208426	1xTMT6plex [N-Term]	1.482	1.542		3.841049433	49.16	0	1444.810979	3	482.2753601	0.37946297	0.000182751	47.52914705
																			SQEPLPDDDEEFELPEFVEPFLK	5	91208426	2xTMT6plex [N-Term; K23]		0.01		2.171666145	6.84	0	3207.602563	3	1069.87085	-1.424082873	-0.001522631	102.5833811
																			ALNMAIPGGPK	2	91208426	2xTMT6plex [N-Term; K11]	1.199			3.04120636	38.86	0	1526.912891	2	763.9597168	-0.480444074	-0.000366798	69.00522683
																			ASGFEESMK	4	91208426	2xTMT6plex [N-Term; K9]	1.156		0.668	4.172992229	45.99	0	1443.755387	2	722.3828125	2.05126161	0.00148076	45.90021998
																			DLILADYGK	6	91208426	2xTMT6plex [N-Term; K9]	1.376		1.988	3.50688076	53.75	0	1465.866652	2	733.4362183	-1.017798284	-0.000745978	66.92061025
																			YELQLANPK	2	91208426	2xTMT6plex [N-Term; K9]	1.62			3.471758842	40.82	0	1533.9041	2	767.454834	-1.113910087	-0.000854316	57.79998484
																			GPGNPVPGPLAPLPDYMSEEK	2	91208426	2xTMT6plex [N-Term; K21]			0.778	2.396386385	28.03	0	2623.384195	3	875.1296997	-3.678278966	-0.003216513	75.9049454
																			EAVVNTQELLDLLVK	4	91208426	2xTMT6plex [N-Term; K15]		100		2.492056608	17.5	0	2142.278592	2	1071.644287	1.26316846	0.001353029	105.6633488
																			QQIAEIEK	2	91208426	2xTMT6plex [N-Term; K8]	1.474			3.242057085	37.62	0	1416.846251	2	708.9260864	-0.956023589	-0.000677269	45.28628703
																			ALDIPLVK	8	91208426	2xTMT6plex [N-Term; K8]	1.074	1.269		2.438839197	33.11	0	1326.876094	2	663.9414063	-0.420951136	-0.000279275	79.44433884
																			VDDESMQR	2	91208426	1xTMT6plex [N-Term]		1.428		3.162197113	24.9	0	1208.577867	2	604.7935791	1.666572903	0.001007092	26.80555034
																			VWAEYALK	2	91208426	2xTMT6plex [N-Term; K8]	1.26			2.403808594	22.31	0	1437.850608	2	719.4296265	0.951677233	0.000684185	73.30472713
																			FICISDLR	2	91208426	1xTMT6plex [N-Term]; 1xCarbamidomethyl [C3	1.306			2.663687229	33.53	0	1252.69211	2	626.8497314	0.061148805	3.83E-05	71.50903718
																			NNVVINYK	2	91208426	2xTMT6plex [N-Term; K8]			1.329	2.944319963	22.82	0	1421.851671	2	711.4275513	-2.703953596	-0.00192231	42.08606998
																			VQMLLSDR	4	91208426	1xTMT6plex [N-Term]	0.97	0.757		2.540677786	19.29	0	1190.676459	2	595.8418579	-0.016880898	-1.00E-05	57.9065759
																			TAEEVAALIR	6	91208426	1xTMT6plex [N-Term]	100	100		1.975855827	29.43	0	1301.762632	2	651.3860474	1.679596963	0.001093218	50.71698135
																			GSELQLPFQACLK	3	91208426	2xTMT6plex [N-Term; K13]; 1xCarbamidomethyl [C11				1.306593299	0.38	0	1949.09304	2	975.0487671	-1.427484607	-0.00139115	74.47237884
																			VILKPDK	2	91208426	3xTMT6plex [N-Term; K4; K7]			1.021	1.942539334	4.08	0	1500.012812	3	500.6759338	0.291033296	0.000145518	48.76618259
33188445	MACF1	microtubule-actin cross-linking factor 1	23499	NP_036222.3	5.064456722	1	26	26	75	5430	620.034	5.38	0.99	0.96	0.95	0.97	Significant	0.00015															
																			LDIEASEAECR	4	33188445	1xTMT6plex [N-Term]; 1xCarbamidomethyl [C10	1.255	0.736		3.639593363	36.41	0	1521.741638	2	761.3729858	-1.934191875	-0.00147167	54.04946068
																			VPEGGEGISATEVDSR	4	33188445	1xTMT6plex [N-Term]	0.948	0.869		4.180765152	39.34	0	1831.923502	2	916.465271	-0.128977991	-0.000118139	41.38939662
																			LLPQAEMFEHLSGK	2	33188445	2xTMT6plex [N-Term; K14]		0.741		3.092561245	25.07	0	2058.145803	3	686.7198486	-0.393903853	-0.000270237	86.1862815
																			ESIAEHKPHIDK	4	33188445	3xTMT6plex [N-Term; K7; K12]		0.924	0.881	4.085086346	26.24	0	2091.21655	4	523.559021	-1.09767476	-0.000573869	36.66688116
																			EKDASSCQEQLDEFR	2	33188445	2xTMT6plex [N-Term; K2]; 1xCarbamidomethyl [C7			0.927	4.023146629	18.62	1	2300.122896	3	767.3739624	-6.76562674	-0.005187258	40.80955144
																			YADITVTSSK	2	33188445	2xTMT6plex [N-Term; K10]		1.198		3.662642717	55.2	0	1542.877945	2	771.9415283	-1.403046419	-0.001082365	48.67820989
																			DIMEIEGEPAPDHR	2	33188445	1xTMT6plex [N-Term]	0.874			4.217032909	36.48	0	1837.895178	3	613.3009644	-3.720926892	-0.002279558	54.1673553
																			SSAPGECHQIMK	2	33188445	2xTMT6plex [N-Term; K12]; 1xCarbamidomethyl [C7	1.035			3.550710917	31.04	0	1802.929345	3	601.6488647	1.495104779	0.000898523	33.88258277
																			ATSFATVVK	2	33188445	2xTMT6plex [N-Term; K9]	1.004			3.915170431	52.74	0	1381.845523	2	691.4267578	0.518484149	0.000358233	60.86451046
																			DQEPIPQNIDR	4	33188445	1xTMT6plex [N-Term]	1.551	1.308		3.069263935	41.65	0	1553.812101	2	777.4101563	0.601726554	0.000467485	42.45601064
																			GALPDDTEALQSLIDTHK	6	33188445	2xTMT6plex [N-Term; K18]	1.383	100		3.353830814	34.32	0	2382.291675	3	794.7683105	-0.544378922	-0.00043229	79.89124896
																			LVLDTVNEVSR	2	33188445	1xTMT6plex [N-Term]	0.969			3.521763086	23.23	0	1473.847424	2	737.4272461	-0.141305332	-0.000104131	66.97852958
																			SATVELEK	6	33188445	2xTMT6plex [N-Term; K8]	0.748	1.017	0.944	3.624870539	37.89	0	1334.793153	2	667.8999634	-0.376427004	-0.000251226	48.64035279
																			QHADHLALNEEIVNR	1	33188445	1xTMT6plex [N-Term]		1.165		2.765231133		0	1988.051102	3	663.3551636	-0.082863737	-5.49E-05	43.3415276
																			ATEVTVAR	4	33188445	1xTMT6plex [N-Term]	0.982	0.749		2.699058533	26.66	0	1075.630889	2	538.3199463	1.605223634	0.000863314	19.748349
																			ELEALNK	6	33188445	2xTMT6plex [N-Term; K7]	1.118	1.136	1.07	3.261033297	35.85	0	1274.772023	2	637.8898315	0.284910502	0.000181598	43.83551101
																			ALLELVPWR	2	33188445	1xTMT6plex [N-Term]	100			2.377923489	30.21	0	1325.814273	2	663.4104004	-0.565108375	-0.000374614	102.0657443
																			EFESWLER	4	33188445	1xTMT6plex [N-Term]				2.050263166	26.93	0	1324.673482	2	662.8407593	0.573420317	0.000379797	77.60407671
																			SLLDDWASK	2	33188445	2xTMT6plex [N-Term; K9]				2.474729776	25.29	0	1492.841165	2	746.9246826	0.618447827	0.000461622	71.07413245
																			MYISELK	2	33188445	2xTMT6plex [N-Term; K7]	1.433			2.42193079	22.1	0	1341.785231	2	671.3956299	-0.929637878	-0.000623687	71.05794317
																			LQDELVTLR	2	33188445	1xTMT6plex [N-Term]	0.987			2.290450811	26.17	0	1315.778282	2	658.3937378	1.457111707	0.000958618	63.96957048
																			ESTDIEK	2	33188445	2xTMT6plex [N-Term; K7]		1.651		2.856302023	15.4	0	1279.714568	2	640.3598022	-1.750427688	-0.001120024	35.61903479
																			GTLVEEINCK	2	33188445	2xTMT6plex [N-Term; K10]; 1xCarbamidomethyl [C9				1.320676327	14.48	0	1620.903114	2	810.9537964	-1.726066499	-0.001398893	52.83577465
																			QLEDILVLAK	2	33188445	2xTMT6plex [N-Term; K10]				1.756892443	9.92	0	1600.008565	2	800.5073853	-0.669441533	-0.000535556	89.44590285
																			LIIEMLER	2	33188445	1xTMT6plex [N-Term]	100			1.773748398	11.57	0	1245.743811	2	623.3759155	0.597124931	0.000371932	92.46801421
																			ELESFLR	2	33188445	1xTMT6plex [N-Term]				1.200008869	16.75	0	1122.63564	2	561.8207397	-1.280458023	-0.000718744	56.61241571
21361547	RNH1	ribonuclease inhibitor	6050	NP_002930.2	58.35140998	1	18	18	97	461	49.941	4.82	1.19	1.08	0.79	1.02	Significant	0.013															
																			ELSLAGNELGDEGAR	6	21361547	1xTMT6plex [N-Term]	1.093	0.839	1.353	4.969423771	66.7	0	1759.902372	2	880.456665	2.091666096	0.001840564	56.91058403
																			ELTVSNNDINEAGVR	8	21361547	1xTMT6plex [N-Term]	1.188	0.863	1.427	5.068113327	71.32	0	1859.966035	2	930.4870605	0.435117489	0.000404652	82.12603503
																			LESCGVTSDNCR	6	21361547	1xTMT6plex [N-Term]; 2xCarbamidomethyl [C4; C11	1.566	2.125		4.22692728	46.98	0	1626.741321	2	813.8729858	-1.614294958	-0.00131302	32.21458231
																			ELDLSNNCLGDAGILQLVESVR	11	21361547	1xTMT6plex [N-Term]; 1xCarbamidomethyl [C8	1.206	1.196	0.978	3.899491787	61.63	0	2644.381346	3	882.1310425	-1.048180361	-0.00092393	100.1854319
																			VNPALAELNLR	6	21361547	1xTMT6plex [N-Term]	1.07	1.132	100	3.576479912	50.77	0	1438.857929	2	719.9326172	0.0199846	1.44E-05	71.69792638
																			SNELGDVGVHCVLQGLQTPSCK	6	21361547	2xTMT6plex [N-Term; K22]; 2xCarbamidomethyl [C11; C21	100	100		4.165878296	13.58	0	2856.474819	3	952.829895	0.109655647	0.00010441	70.81624176
																			LLCETLLEPGCQLESLWVK	8	21361547	2xTMT6plex [N-Term; K19]; 2xCarbamidomethyl [C3; C11	1.35	0.695		3.207767487	31.76	0	2746.492366	3	916.1689453	-0.03042153	-2.79E-05	108.9768553
																			TLPTLQELHLSDNLLGDAGLQLLCEGLLDPQCR	3	21361547	1xTMT6plex [N-Term]; 2xCarbamidomethyl [C24; C32				4.280835629	28.8	0	3932.045888	4	983.7672729	0.349318628	0.000343384	111.8220862
																			DSPCQLEALK	6	21361547	2xTMT6plex [N-Term; K10]; 1xCarbamidomethyl [C4	1.153	1.168	1.316	4.424354553	52.15	0	1618.887464	2	809.9474487	0.096956052	7.85E-05	60.74882424
																			VLWLADCDVSDSSCSSLAATLLANHSLR	3	21361547	1xTMT6plex [N-Term]; 2xCarbamidomethyl [C7; C14				1.719448924	6.22	0	3290.634677	3	1097.54834	-1.279585601	-0.00140355	97.07333825
																			LDDCGLTEAR	6	21361547	1xTMT6plex [N-Term]; 1xCarbamidomethyl [C4	1.13	1.229	1.593	4.224281311	55.01	0	1378.683395	2	689.8448486	-0.706967516	-0.000487342	48.89308434
																			ELCQGLGQPGSVLR	6	21361547	1xTMT6plex [N-Term]; 1xCarbamidomethyl [C3	1.092	0.01	100	2.519287109	29.47	0	1742.942069	2	871.9750977	0.487223594	0.000424601	39.95491502
																			DLCGIVASK	6	21361547	2xTMT6plex [N-Term; K9]; 1xCarbamidomethyl [C3	0.746	1.576		3.009689093	29.9	0	1420.823407	2	710.9138794	-2.058673107	-0.001462505	47.991553
																			ELALGSNK	4	21361547	2xTMT6plex [N-Term; K8]			0.697	3.086421728	39.8	0	1289.782922	2	645.3953247	0.349278979	0.000225247	46.78767018
																			WAELLPLLQQCQVVR	4	21361547	1xTMT6plex [N-Term]; 1xCarbamidomethyl [C11	100	1.068		1.57066977	19.23	0	2082.173132	2	1041.589111	-1.049828046	-0.001092962	105.6378803
																			TLWIWECGITAK	4	21361547	2xTMT6plex [N-Term; K12]; 1xCarbamidomethyl [C7	100			1.998378277	10.31	0	1936.076662	2	968.5410767	-0.921900317	-0.000892435	95.21572381
																			VLCQGLK	2	21361547	2xTMT6plex [N-Term; K7]; 1xCarbamidomethyl [C3	1.168			3.226940632	34.13	0	1275.7859	2	638.3963013	-0.449645146	-0.000286825	53.17496385
																			DISSALR	2	21361547	1xTMT6plex [N-Term]			0.709	2.569905043	23.89	0	990.5781255	2	495.7926941	-0.014089152	-6.98E-06	37.03428815
32454741	SERPINH1	serpin H1 precursor	871	NP_001226.2	47.84688995	1	16	16	100	418	46.411	8.69	1.13	1.28	1.18	1.2	Significant	0.0014															
																			AVLSAEQLRDEEVHAGLGELLR	9	32454741	1xTMT6plex [N-Term]	1.306	1.037		4.351075172	27.21	1	2634.441244	4	659.3662109	0.67188441	0.00044251	76.68589414
																			GVVEVTHDLQK	12	32454741	2xTMT6plex [N-Term; K11]	1.188	1.237	1.163	4.799574852	55.74	0	1682.984141	2	841.9945068	-1.42844966	-0.001202029	38.54720625
																			DTQSGSLLFIGR	6	32454741	1xTMT6plex [N-Term]	1.163	1.348		4.157497883	62.93	0	1522.842673	2	761.9245605	-0.543934226	-0.000414163	70.09837987
																			KDLYLASVFHATAFELDTDGNPFDQDIYGR	6	32454741	2xTMT6plex [N-Term; K1]	2.04	2.385	100	6.783832073	32.67	1	3876.948489	4	969.9944458	1.925215964	0.001865991	99.96750522
																			DEEVHAGLGELLR	12	32454741	1xTMT6plex [N-Term]		1.199	1.022	3.663055658	30.82	0	1666.896165	3	556.3034668	-0.19059338	-0.0001059	64.03664477
																			DQAVENILVSPVVVASSLGLVSLGGK	6	32454741	2xTMT6plex [N-Term; K26]			1.487	4.35198307	38.71	0	3009.760008	3	1003.923096	-1.752242907	-0.001757944	112.94063
																			KPAAAAAPGTAEK	2	32454741	3xTMT6plex [N-Term; K1; K13]		1.444		4.939554214	23.79	0	1870.136508	3	624.0491943	-1.860127414	-0.001159564	30.54701101
																			LQIVEMPLAHK	13	32454741	2xTMT6plex [N-Term; K11]	1.938	1.926	2.551	2.98043561	34.37	0	1737.049718	3	579.6878052	-0.492808379	-0.000285344	59.13196713
																			ATTASQAK	4	32454741	2xTMT6plex [N-Term; K8]	1.305	1.324		3.584518671	49.13	0	1235.735972	2	618.3713379	-0.463657903	-0.000286479	20.55526678
																			AVLSAEQLR	4	32454741	1xTMT6plex [N-Term]	0.998	1.194		3.501303911	51.59	0	1215.725852	2	608.3655396	-1.686077855	-0.001024904	41.53911332
																			KAVAISLPK	8	32454741	3xTMT6plex [N-Term; K1; K9]	0.998	1.618	0.976	4.627212048	23.35	1	1614.092125	3	538.7022705	0.08281029	4.46E-05	58.03359204
																			AVAISLPK	8	32454741	2xTMT6plex [N-Term; K8]	1.226	1.3	1.514	3.341817141	48.15	0	1256.83423	2	628.9213867	1.008221665	0.000633584	56.9814165
																			LYGPSSVSFADDFVR	7	32454741	1xTMT6plex [N-Term]	1.876			2.669587374	29.36	0	1888.964244	2	944.9857788	0.019405972	1.83E-05	81.83808224
																			TGLYNYYDDEKEK	1	32454741	3xTMT6plex [N-Term; K11; K13]			1.224	3.014928102		1	2325.221754	3	775.7449341	-0.647234572	-0.000501655	47.78708854
																			SALQSINEWAAQTTDGK	1	32454741	2xTMT6plex [N-Term; K17]				1.067743301		0	2278.207946	2	1139.6073	-0.273333532	-0.000311355	72.65320167
																			HLAGLGLTEAIDK	1	32454741	2xTMT6plex [N-Term; K13]				0.900096476		0	1796.068205	2	898.5378418	0.112431003	0.000100967	60.64702152
29725609	EGFR	epidermal growth factor receptor isoform a precursor	1956	NP_005219.2	24.79338843	1	21	21	81	1210	134.19	6.68	1.41	1.71	1.65	1.59	Significant	0.0033															
																			ACGADSYEMEEDGVR	6	29725609	1xTMT6plex [N-Term]; 1xCarbamidomethyl [C2	100	1.613	100	3.969882011	55.79	0	1917.815608	2	959.4116821	0.25021061	0.000239929	38.67436017
																			GSTAENAEYLR	6	29725609	1xTMT6plex [N-Term]	1.593	2.064	2.139	4.942775249	76.97	0	1439.732788	2	720.3700562	0.033113567	2.38E-05	36.8896751
																			ATGQVCHALCSPEGCWGPEPR	6	29725609	1xTMT6plex [N-Term]; 3xCarbamidomethyl [C6; C10; C15	1.56	1.837	1.279	6.034397602	55.12	0	2598.184912	3	866.7313232	-2.115270322	-0.001831954	58.31176268
																			EILDEAYVMASVDNPHVCR	3	29725609	1xTMT6plex [N-Term]; 1xCarbamidomethyl [C18	100			4.257400513	48.2	0	2447.189646	3	816.4017334	0.409022371	0.000333652	83.49402952
																			RPAGSVQNPVYHNQPLNPAPSR	4	29725609	1xTMT6plex [N-Term]	1.713	1.483		5.547427177	43.6	0	2628.395631	4	657.8526611	-2.593399057	-0.00170412	39.96453671
																			GPDNCIQCAHYIDGPHCVK	4	29725609	2xTMT6plex [N-Term; K19]; 3xCarbamidomethyl [C5; C8; C17	1.131	1.568		6.057684898	50.11	0	2699.289267	4	675.5780029	0.338955428	0.000228735	47.95428846
																			ALMDEEDMDDVVDADEYLIPQQGFFSSPSTSR	4	29725609	1xTMT6plex [N-Term]	0.604			2.932057858	19.05	0	3836.735631	4	959.9382935	-1.117466067	-0.001071855	105.2932863
																			EISDGDVIISGNK	4	29725609	2xTMT6plex [N-Term; K13]		100	3.3	3.020915747	44.78	0	1805.005664	2	902.9978638	-9.536463668	-0.008606685	50.90864948
																			GDSFTHTPPLDPQELDILK	8	29725609	2xTMT6plex [N-Term; K19]	0.989		100	4.122005463	28.37	0	2581.391389	3	861.1348267	-0.566562489	-0.000487507	79.70157802
																			GSHQISLDNPDYQQDFFPK	4	29725609	2xTMT6plex [N-Term; K19]		1.22	100	2.60106349	14.53	0	2694.356401	3	898.7896729	-0.718314515	-0.000645132	58.47703666
																			SPSDCCHNQCAAGCTGPR	4	29725609	1xTMT6plex [N-Term]; 4xCarbamidomethyl [C5; C6; C10; C14]		100		3.012136459	27.4	0	2263.926255	3	755.3131104	-0.652603109	-0.000492482	18.4321857
																			ITDFGLAK	2	29725609	2xTMT6plex [N-Term; K8]		100		2.520804882	34.07	0	1322.808409	2	661.9074097	-0.654723735	-0.000433037	74.49795595
																			WMALESILHR	2	29725609	1xTMT6plex [N-Term]	100			2.137167931	25.09	0	1484.82452	3	495.6129456	-0.159628343	-7.90E-05	93.23155531
																			NLQEILHGAVR	4	29725609	1xTMT6plex [N-Term]	1.791	1.817		2.844203234	18.83	0	1478.864077	3	493.6252136	-2.02140339	-0.00099646	58.58818449
																			IICAQQCSGR	2	29725609	1xTMT6plex [N-Term]; 2xCarbamidomethyl [C3; C7			1.265	2.9510324	22.98	0	1421.71907	2	711.3621826	-1.393436768	-0.000990538	33.85893336
																			ELIIEFSK	2	29725609	2xTMT6plex [N-Term; K8]	0.581			2.368821621	24.89	0	1436.876488	2	718.9419556	0.101764357	7.31E-05	87.09995972
																			ESDCLVCR	4	29725609	1xTMT6plex [N-Term]; 2xCarbamidomethyl [C4; C7	1.375			2.872550964	21.01	0	1267.597223	2	634.3016357	-0.969081978	-0.000614203	34.13045296
																			GKSPSDCCHNQCAAGCTGPR	4	29725609	2xTMT6plex [N-Term; K2]; 4xCarbamidomethyl [C7; C8; C12; C16]	100			2.545233965	3.88	1	2678.205614	4	670.3058472	-1.514113817	-0.001013777	20.32666212
																			GLWIPEGEK	3	29725609	2xTMT6plex [N-Term; K9]	100		1.24	1.73360157	13.55	0	1486.866986	2	743.9366455	-0.653612182	-0.000485917	62.43597773
																			ELVEPLTPSGEAPNQALLR	3	29725609	1xTMT6plex [N-Term]	100			1.188245177	3.37	0	2263.249527	2	1132.127686	-0.632986435	-0.000716303	64.67994251
																			LLGAEEK	2	29725609	2xTMT6plex [N-Term; K7]	1.406			3.020066261	26.7	0	1217.75056	2	609.3778076	-1.823826405	-0.001110483	46.76085845

Bhat et al,2017. Cigarette smoke and chewing tobacco alter expression of different sets of miRNAs in oral keratinocytes

Supplementary Table S2(A). List of all proteins and peptides identified in OKF6/TERT1 cells chronically treated with chewing tobacco using Sequest and Mascot search algorithms

Accession	Gene_Symbol	Description	Gene_ID	Refseq_Accession	Coverage	Proteins	#Unique_Peptides	#Peptides	#PSMS	#AAs	MW [kDa]	calc. pI	OKF6-STE-6M/OKF6P_R1	OKF6-STE-6M/OKF6P_R2	OKF6-STE-6M/OKF6P_R3	Median fold change	Status	P-value (6M)	Sequence	#PSMSs	Protein Accessions	Modifications	OKF6-STE-6M/OKF6P_R1	OKF6-STE-6M/OKF6P_R2	OKF6-STE-6M/OKF6P_R3	Xcorr	Ionscore	# Missed Cleavages	Theo. MH+ [Da]	Charge	m_z	DeltaM [ppm]	Deltam/z [Da]	RT [min]
5454158	VARS	valine--tRNA ligase	7407	NP_006286.1	19.0664557	1	20	20	82	1264	140.387	7.59	1.21	1.37	1.25	1.28	Significant	0.0014																
																			GFVPSPTSQPGGHESLVDR	12	5454158	1xTMT6plex [N-Term]	1.292	1.294	1.26	5.033790588	44.21	0	2196.124661	3	732.7132568	0.253304297	0.000185429	43.83782607
																			ITPAHDQNDYEVGQR	6	5454158	1xTMT6plex [N-Term]	1.149			4.831240654	38.25	0	1971.972183	3	657.9954834	-0.145146412	-9.54E-05	37.25278219
																			VQGSDSDEEVVVATTR	4	5454158	1xTMT6plex [N-Term]	1.446	1.678		4.261178493	56.29	0	1920.97118	2	960.9891357	-0.09640729	-9.26E-05	40.26723012
																			GIEDNPMVVPLCNR	2	5454158	1xTMT6plex [N-Term]; 1xCarbamidomethyl [C12	0.636			2.569102049	32.31	0	1842.940355	2	921.9725342	-1.390804981	-0.001281585	62.22402026
																			DVSGPMPDSYSPR	8	5454158	1xTMT6plex [N-Term]	0.85	1.251	1.047	4.167327404	51.68	0	1636.783837	2	818.8943481	-1.477025177	-0.001208785	40.046808
																			VPLEVQEADEAK	6	5454158	2xTMT6plex [N-Term; K12]	1.309	1.168	1.558	3.326541185	47.36	0	1785.999851	2	893.503418	-0.163125714	-0.000145671	62.24259928
																			KAVLVALK	2	5454158	3xTMT6plex [N-Term; K1; K8]	0.774			4.356149673	33.03	1	1529.075746	3	510.3622131	-2.393711176	-0.001220055	56.25943769
																			SLGNVIDPLDVIYGISLQGLHNQLLNSNLDPSEVEK	2	5454158	2xTMT6plex [N-Term; K36]				2.236250877	11.23	0	4362.359701	4	1091.344482	-0.825471247	-0.000900251	110.0241087
																			LSAAVTEAFVR	6	5454158	1xTMT6plex [N-Term]				2.590526342	38.15	0	1392.804831	2	696.9052734	-1.120447722	-0.000780282	63.67901457
																			LHEEGIIYR	8	5454158	1xTMT6plex [N-Term]	1.49	1.514		3.336547136	39.78	0	1358.762966	3	453.5925293	0.050487559	2.29E-05	47.76878997
																			AASGYPVK	2	5454158	2xTMT6plex [N-Term; K8]			1.288	3.538049936	42.44	0	1250.750894	2	625.8789063	-0.286284025	-0.000179035	38.50079158
																			TWHAWMDNIR	4	5454158	1xTMT6plex [N-Term]	0.905	0.967		3.275886059	26.17	0	1558.778633	3	520.2648926	0.956918669	0.000497208	63.42570272
																			AVLVALK	4	5454158	2xTMT6plex [N-Term; K7]	0.521	1.589		2.975559711	35.76	0	1171.817851	2	586.4122925	-0.463398865	-0.00027151	55.17784205
																			DINLDVNR	2	5454158	1xTMT6plex [N-Term]			1.246	3.506086588	37.04	0	1187.658167	2	594.3322754	-0.751469383	-0.000446244	43.40574057
																			TLLSVPGYK	4	5454158	2xTMT6plex [N-Term; K9]	1.827	1.803		3.340154409	25.95	0	1435.892473	2	718.4494629	-0.573551826	-0.000411779	72.07103933
																			GALINVPPPFLGLPR	2	5454158	1xTMT6plex [N-Term]				1.318543077	7	0	1790.088991	2	895.5473022	-0.92933805	-0.000831799	106.2286452
																			ACFTMDPK	2	5454158	2xTMT6plex [N-Term; K8]; 1xCarbamidomethyl [C2	1.275			2.585333824	27.91	0	1427.742714	2	714.3753662	0.519513682	0.000370866	56.9476148
																			KVDEAIALFQK	2	5454158	3xTMT6plex [N-Term; K1; K11]			0.821	2.565452099	9.43	1	1949.20386	3	650.406189	0.078967898	5.13E-05	69.13104428
																			EAFLQEVWK	2	5454158	2xTMT6plex [N-Term; K9]				1.440790772	1.19	0	1607.91975	2	804.4624023	-1.38189266	-0.001110986	75.64097074
																			ALSPLEEWLR	2	5454158	1xTMT6plex [N-Term]				1.192895651	10.11	0	1442.820481	2	721.9137573	-0.168317241	-0.000121426	83.96818208
40217847	SNRNP200	U5 small nuclear ribonucleoprotein 200 kDa helicase	23020	NP_054733.2	16.15168539	1	27	27	83	2136	244.353	6.06	1.14	1.06	1.13	1.11	Significant	0.00051																
																			VHGSSEAFWILVEDVDSEVILHHEYFLLK	2	40217847	2xTMT6plex [N-Term; K29]				5.39627409	28.49	0	3870.051832	5	774.8193359	4.067358315	0.003148178	112.2256129
																			SPTLYGISHDDLK	4	40217847	2xTMT6plex [N-Term; K13]	1.472	1.243		4.584192276	41.88	0	1904.052949	3	635.3563843	0.866962252	0.000550247	57.87428688
																			NIEMTQEDVR	4	40217847	1xTMT6plex [N-Term]	1.451	1.116		4.12777853	68.47	0	1463.736159	2	732.3712158	-0.685785733	-0.000501905	46.73029137
																			EIDLLLGQTDDTR	4	40217847	1xTMT6plex [N-Term]	0.681	0.681		3.242949963	39.71	0	1717.91696	2	859.4622803	0.188680176	0.000162068	78.52484343
																			GNIIISTPEK	6	40217847	2xTMT6plex [N-Term; K10]	100	100	1.171	3.487053633	47.07	0	1529.930315	2	765.4692383	0.578616223	0.000442621	45.72350585
																			YAQDEHLITFFVPVFEPLPPQYFIR	2	40217847	1xTMT6plex [N-Term]		1.546		2.610819817	13.49	0	3295.739323	3	1099.25	-1.176200428	-0.00129215	111.9384269
																			GVESVFDIMEMEDEER	3	40217847	1xTMT6plex [N-Term]			0.57	2.764642477	14.29	0	2143.972502	2	1072.488647	-1.158423498	-0.001241814	94.32195677
																			HINMDGTINVDDFK	2	40217847	2xTMT6plex [N-Term; K14]	100			2.713947296	14.87	0	2077.078846	3	693.0313721	0.345201598	0.000239004	70.28843594
																			KPVIVFVPSR	6	40217847	2xTMT6plex [N-Term; K1]	0.812	1.156	1.016	3.678232431	19.77	0	1600.035055	3	534.0166626	0.237459153	0.000126648	50.68338402
																			GTQVYSPEK	2	40217847	2xTMT6plex [N-Term; K9]	1.779			3.740256786	42.39	0	1466.825515	2	733.916687	0.396838909	0.000291047	39.94459446
																			FYDDAIVSQK	4	40217847	2xTMT6plex [N-Term; K10]	1.128	100		2.889283657	19.41	0	1643.904494	2	822.4561157	0.280420982	0.000230493	63.27175686
																			KLPEEVVK	2	40217847	3xTMT6plex [N-Term; K1; K8]	0.818			3.38757515	19.69	1	1629.055405	3	543.6900635	0.142695999	7.75E-05	50.89093221
																			ITDYGGDK	4	40217847	2xTMT6plex [N-Term; K8]		0.65	0.737	2.677313089	19	0	1326.730552	2	663.8691406	0.340898157	0.00022614	39.05444578
																			DTLGLFLR	2	40217847	1xTMT6plex [N-Term]	100			1.961014748	31.65	0	1163.698575	2	582.3528442	-0.140168116	-8.16E-05	98.43500053
																			EGSASTEVLR	2	40217847	1xTMT6plex [N-Term]	1.802			2.438947201	13.88	0	1277.689861	2	639.3495483	1.533572229	0.000979715	34.41352519
																			GDPLLDQR	2	40217847	1xTMT6plex [N-Term]			1.299	2.483609915	15.1	0	1142.636703	2	571.8218994	-0.158188403	-9.04E-05	42.38349698
																			DIDAFWLQR	4	40217847	1xTMT6plex [N-Term]	100			2.566806078	18.42	0	1392.747316	2	696.8769531	-0.49280296	-0.000343175	79.33918759
																			SLQYEYK	4	40217847	2xTMT6plex [N-Term; K7]	1.063	0.97		3.476491213	34.91	0	1388.782588	2	694.8951416	0.301453322	0.000209327	53.4481039
																			EEEKLELQK	5	40217847	3xTMT6plex [N-Term; K4; K9]	1.227	1.333		2.473259687	10.36	1	1833.093641	3	611.7022095	-0.853961847	-0.000521797	51.25630454
																			GLIEIISNAAEYENIPIR	2	40217847	1xTMT6plex [N-Term]				1.122435689		0	2244.243713	2	1122.624512	-0.876300773	-0.000983316	105.695
																			EEGWWVVIGDAK	2	40217847	2xTMT6plex [N-Term; K12]				1.7523278	0.03	0	1847.010356	2	924.008606	-0.227684667	-0.000210268	89.80679699
																			DILCGAADEVLAVLK	2	40217847	2xTMT6plex [N-Term; K15]; 1xCarbamidomethyl [C4				1.098290086		0	2045.171684	2	1023.089233	-0.241467809	-0.000246922	105.1015274
																			IIYIAPMR	2	40217847	1xTMT6plex [N-Term]		0.01		1.735251069	7.54	0	1205.727767	2	603.3668823	-1.060456264	-0.000639311	75.51707744
																			LIGLSATLPNYEDVATFLR	2	40217847	1xTMT6plex [N-Term]				1.184901834		0	2322.290664	2	1161.647339	-1.40486963	-0.001631258	101.9497388
																			QVLDLEDLVFTQGSHFMANK	1	40217847	2xTMT6plex [N-Term; K20]				1.556336164		0	2750.458757	3	917.4906006	-0.548552922	-0.000502924	96.98527006
																			DLIPYLEK	7	40217847	2xTMT6plex [N-Term; K8]	1.261	1.038	100	1.990857363	14.67	0	1448.876488	2	724.9404297	-2.005371074	-0.001452767	72.02229541
																			LATYGITVAELTGDHQLCK	1	40217847	2xTMT6plex [N-Term; K19]; 1xCarbamidomethyl [C18				1.113180876		0	2548.38453	3	850.1313477	-1.977869191	-0.001680124	75.66737818
4827050	USP14	ubiquitin carboxyl-terminal hydrolase 14 isoform	9097	NP_005142.1	54.048583	1	18	18	87	494	56.033	5.3	0.91	0.86	1.13	0.97	Significant	0.0073																
																			AQLFALTGVQPAR	8	4827050	1xTMT6plex [N-Term]	1.223	0.952		3.650990963	59.4	0	1600.937242	2	800.9721069	-0.190296536	-0.000152326	72.55506807
																			FPLMLDMYELCTPELQEK	7	4827050	2xTMT6plex [N-Term; K18]; 1xCarbamidomethyl [C11	2.111	1.469		3.242800713	26.93	0	2715.384789	3	905.7991943	-0.648012095	-0.000586534	108.2560716
																			YEPFSFADDIGSNNCGYYDLQAVLTHQGR	4	4827050	1xTMT6plex [N-Term]; 1xCarbamidomethyl [C15			1.241	6.496020794	49.22	0	3566.648412	4	892.4171143	-0.500517165	-0.000446292	95.84245778
																			SSSSGHYVSWVK	4	4827050	2xTMT6plex [N-Term; K12]	0.806	0.726		3.394787073	23.53	0	1781.958655	3	594.6573486	-0.65207549	-0.000387324	43.1135046
																			ETDSSSASAATPSK	4	4827050	2xTMT6plex [N-Term; K14]			1.676	4.465116501	76.39	0	1796.927808	2	898.9672852	-0.286131417	-0.000257079	31.27732109
																			GEQGQYLQQDANECWIQMMR	5	4827050	1xTMT6plex [N-Term]; 1xCarbamidomethyl [C14	100	2.802		3.58460021	25.63	0	2714.232256	3	905.4140625	-1.702695114	-0.001540503	91.01836467
																			CTESEEEEVTK	6	4827050	2xTMT6plex [N-Term; K11]; 1xCarbamidomethyl [C1	1.203	2.095	2.071	3.942151546	44.97	0	1798.878081	2	899.942627	-0.057543505	-5.18E-05	34.21331443
																			TVFVEDMTEEQLASAMELPCGLTNLGNTCYMNATVQCIR	2	4827050	1xTMT6plex [N-Term]; 3xCarbamidomethyl [C20; C29; C37				2.536380053	9.14	0	4696.173292	4	1174.804199	4.615507915	0.005418806	105.7122996
																			RVEIMEEESEQ	10	4827050	1xTMT6plex [N-Term]	1.093	1.117	0.869	3.209332466	68.07	1	1607.778417	2	804.3933105	0.576574321	0.000463502	46.7880501
																			EKFEGVELNTDEPPMVFK	4	4827050	3xTMT6plex [N-Term; K2; K18]				1.979072452	13.28	1	2796.509679	3	932.8426514	1.330788014	0.001240521	73.67500284
																			LEAIEDDSVK	2	4827050	2xTMT6plex [N-Term; K10]			100	1.832576156	20.17	0	1576.883424	2	788.9448853	-0.589848414	-0.000465061	52.5929664
																			GKENQLQLSCFINQEVK	3	4827050	3xTMT6plex [N-Term; K2; K17]; 1xCarbamidomethyl [C10			100	2.613465786	5.72	1	2722.516496	3	908.1775513	0.589241092	0.00053474	75.85943198
																			GGTLKDDDWGNIK	6	4827050	3xTMT6plex [N-Term; K5; K13]		1.018	100	3.332097292	17.75	1	2106.17983	3	702.7322998	1.194804939	0.000838825	58.78930649
																			DLFDSMDK	6	4827050	2xTMT6plex [N-Term; K8]	100	100	1.648	2.509329557	30.71	0	1428.744488	2	714.8756714	-0.29513784	-0.000210838	64.60274882
																			VSIVTPEDILR	4	4827050	1xTMT6plex [N-Term]				1.617118359	14.27	0	1470.87291	2	735.9401855	0.125200314	9.21E-05	96.96305744
																			LQEEITK	6	4827050	2xTMT6plex [N-Term; K7]	0.705	1.054	0.769	2.978837013	35.42	0	1318.798238	2	659.9031982	0.668695445	0.000440937	42.57169689
																			DDDWGNIK	2	4827050	2xTMT6plex [N-Term; K8]	1.064			2.921358585	17.64	0	1420.747265	2	710.8771362	-0.189455978	-0.000134585	49.89285325
																			RVEIMEEESEQ	2	4827050	1xTMT6plex [N-Term]; 1xOxidation [M5]		1.2	1.118	1.428978324	31.08	1	1623.773332	2	804.3933105	0.576574321	0.000463502	46.7880501
																			SLIDQFFGVEFETTMK	2	4827050	2xTMT6plex [N-Term; K16]				1.183938861		0	2350.240491	2	1175.623047	-0.712399436	-0.000837155	105.2736742
29029559	CSE1L	exportin-2 isoform 1	1434	NP_001307.2	23.99588054	1	22	22	103	971	110.346	5.77	0.94	1.45	0.8	1.06	Significant	0.033																
																			GSNTIASAAADK	6	29029559	2xTMT6plex [N-Term; K12]	3.823	2.548	0.727	4.376844406	74.82	0	1563.874256	2	782.4417725	1.28653686	0.001005991	39.58128694
																			ATIELCSTHANDASALR	7	29029559	1xTMT6plex [N-Term]; 1xCarbamidomethyl [C6	1.831	1.636		5.099937916	54.39	0	2059.043968	3	687.0186768	-1.209855934	-0.000830382	45.77747261
																			SQICDNAALYAQK	4	29029559	2xTMT6plex [N-Term; K13]; 1xCarbamidomethyl [C4		2.062	2.27	4.654443741	65.38	0	1940.031168	2	970.5181885	-1.065713226	-0.001033758	45.01114539
																			IHLAQSLHK	4	29029559	2xTMT6plex [N-Term; K9]	1.486	1.482		4.48773241	44.1	0	1504.936403	3	502.3173523	0.731210145	0.000366808	40.23478524
																			DLEGSDIDTR	2	29029559	1xTMT6plex [N-Term]			1.836	3.927676439	60.33	0	1349.674604	2	675.3403931	-0.811245306	-0.000547459	38.62339846
																			SANVNEFPVLK	4	29029559	2xTMT6plex [N-Term; K11]	1.367		100	3.229620218	34.85	0	1675.978327	2	838.4915771	-1.461715277	-0.001224902	63.06268989
																			KICAVGITK	6	29029559	3xTMT6plex [N-Term; K1; K9]; 1xCarbamidomethyl [C3	0.239	1.113	0.684	4.55601263	24.79	1	1677.070009	3	559.694397	-0.81784423	-0.000457194	51.11174204
																			AADEEAFEDNSEEYIR	10	29029559	1xTMT6plex [N-Term]		1.833	100	2.604017735	26.9	0	2116.950838	2	1058.977539	-1.43458929	-0.001518478	53.21009363
																			IVEDEPNKICEADR	2	29029559	2xTMT6plex [N-Term; K8]; 1xCarbamidomethyl [C10	1.561			3.883162022	18.3	1	2146.121439	3	716.045105	-0.315722392	-0.00022586	50.99259528
																			ALTLPGSSENEYIMK	6	29029559	2xTMT6plex [N-Term; K15]	2.928	100	100	2.269901514	16.62	0	2111.145863	2	1056.068481	-7.662464096	-0.00808829	66.46012236
																			QLSDAISIIGR	5	29029559	1xTMT6plex [N-Term]	100	100	0.91	2.52964139	31.33	0	1401.826295	2	701.4169312	0.207710962	0.000145587	62.68814523
																			DAAIYLVTSLASK	2	29029559	2xTMT6plex [N-Term; K13]				1.623963952	17.65	0	1810.072622	2	905.5389404	-1.11457441	-0.00100873	95.05936819
																			MFGMVLEK	6	29029559	2xTMT6plex [N-Term; K8]	100	100		3.289606571	32.9	0	1412.804586	2	706.9049683	-1.363151409	-0.000962933	87.12239843
																			LLQAFLER	4	29029559	1xTMT6plex [N-Term]	2.003	2.172		2.851941586	46.36	0	1218.740774	2	609.8740845	0.096989709	5.91E-05	95.17852187
																			ICAVGITK	6	29029559	2xTMT6plex [N-Term; K8]; 1xCarbamidomethyl [C2	2.225	1.812	1.957	3.612859488	44.01	0	1319.812114	2	660.4094849	-0.319123785	-0.000210592	48.45921424
																			VCASVTFK	2	29029559	2xTMT6plex [N-Term; K8]; 1xCarbamidomethyl [C2			1.353	3.272954464	24	0	1369.791379	2	685.3986206	-1.032448506	-0.00070712	46.31372885
																			TLDPDPAIR	2	29029559	1xTMT6plex [N-Term]	1.621			2.711512566	36	0	1226.694218	2	613.8508911	0.234619646	0.000143903	46.70892058
																			LLQTDDEEEAGLLELLK	6	29029559	2xTMT6plex [N-Term; K17]				1.587610245	9.27	0	2387.332143	2	1194.167847	-1.560876494	-0.001863165	98.21702097
																			SNELWTEIK	2	29029559	2xTMT6plex [N-Term; K9]			100	1.597897291	17.88	0	1577.893929	2	789.4493408	-1.599701557	-0.00126208	60.04461755
																			VSGNVEK	6	29029559	2xTMT6plex [N-Term; K7]	1.8	1.794	1.999	2.869485617	42.55	0	1190.714508	2	595.8604126	-0.806116563	-0.000479927	30.09898237
																			IIIPEIQK	7	29029559	2xTMT6plex [N-Term; K8]	1.398	2.421		2.992304087	30.07	0	1411.928858	2	706.4689941	1.31268742	0.000926711	81.05837763
																			VIVPNMEFR	4	29029559	1xTMT6plex [N-Term]	1.642	0.776	100	2.357890606	19.18	0	1333.749959	2	667.3781738	-0.665442382	-0.000443767	62.17712825
11056044	PPA1	inorganic pyrophosphatase	5464	NP_066952.1	67.47404844	1	16	17	92	289	32.639	5.86	1.16	1.13	1.26	1.18	Significant	0.0011																
																			HTGCCGDNDPIDVCEIGSK	6	11056044	2xTMT6plex [N-Term; K19]; 3xCarbamidomethyl [C4; C5; C14	1.944	1.574	1.626	5.691674709	51.27	0	2592.189278	3	864.7349854	0.433909166	0.000374925	44.90290098
																			VIAINVDDPDAANYNDINDVK	8	11056044	2xTMT6plex [N-Term; K21]	1.846	100	0.875	3.67118597	44.7	0	2746.429959	3	916.147522	-0.708844595	-0.000648931	74.8208965
																			GQYISPFHDIPIYADKDVFHMVVEVPR	2	11056044	2xTMT6plex [N-Term; K16]; 1xOxidation [M21			1.124	3.824549675	9.46	1	3646.913231	5	726.9887695	-0.984526072	-0.000714947	93.97016013
																			GQYISPFHDIPIYADKDVFHMVVEVPR	7	11056044	2xTMT6plex [N-Term; K16]			0.9	5.969074726	27.94	1	3630.918316	5	726.9887695	-0.984526072	-0.000714947	93.97016013
																			GYIWNYGAIPQTWEDPGHNDK	4	11056044	2xTMT6plex [N-Term; K21]	0.898	1.358		3.872020245	36.44	0	2919.446613	3	973.8200684	-0.329104108	-0.000320267	91.814781
																			DKDFAIDIIK	8	11056044	3xTMT6plex [N-Term; K2; K10]	1.2	100	0.653	4.395748615	15.68	1	1865.135111	3	622.383728	0.814606954	0.000506451	70.23106595
																			YKVPDGKPENEFAFNAEFK	4	11056044	4xTMT6plex [N-Term; K2; K7; K19]	100		1.124	3.502107382	12.58	1	3146.733238	4	787.4417114	3.742974773	0.002944536	72.53353629
																			DVFHMVVEVPR	2	11056044	1xTMT6plex [N-Term]	1.641			3.461932898	43.46	0	1556.84565	3	519.6199341	-0.257420449	-0.000133588	66.94480623
																			AAPFSLEYR	8	11056044	1xTMT6plex [N-Term]	0.801	0.904	0.797	3.081692457	49.2	0	1282.699303	2	641.8533325	0.066437288	4.26E-05	66.16316441
																			GISCMNTTLSESPFK	7	11056044	2xTMT6plex [N-Term; K15]; 1xCarbamidomethyl [C4	100		2.528	1.82984519	17.19	0	2130.097533	2	1065.549927	-2.32659505	-0.002477937	64.04745736
																			GQYISPFHDIPIYADK	6	11056044	2xTMT6plex [N-Term; K16]	100	1.454	100	3.169476986	21.26	0	2322.253439	3	774.7548828	-1.440080761	-0.001114744	70.47445716
																			YVANIFPYK	24	11056044	2xTMT6plex [N-Term; K9]	0.901	0.794		3.80501771	41.2	0	1572.919022	2	786.963501	0.44727231	0.000351762	81.33913098
																			VIAINVDDPDAANYNDINDVKR	3	11056044	2xTMT6plex [N-Term; K21]				1.704854846	4.03	1	2902.53107	3	968.1810303	-0.872634823	-0.000844283	60.64927514
																			VLGILAMIDEGETDWK	3	11056044	2xTMT6plex [N-Term; K16]			100	1.813022017	2.65	0	2248.229927	2	1124.611572	-6.253305582	-0.007029434	109.8188386
																			DFAIDIIK	2	11056044	2xTMT6plex [N-Term; K8]	100			2.353013277	28.72	0	1392.850274	2	696.9296875	1.310126461	0.000912405	78.09007595
																			LKPGYLEATVDWFR	3	11056044	2xTMT6plex [N-Term; K2]	100	100		1.570586324	1.5	0	2153.215932	3	718.4093018	-1.198108617	-0.000859929	87.94655273
																			GEIIGVK	3	11056044	2xTMT6plex [N-Term; K7]		1.041	1.353	2.86479187	31.69	0	1173.76073	2	587.3840942	0.154611206	9.07E-05	45.31113847
																			DPLNPIK	4	11056044	2xTMT6plex [N-Term; K7]	1.155	1.126		3.048789263	36.32	0	1254.782194	2	627.8947754	0.063828807	4.00E-05	58.50799056
51873036	OGDH	2-oxoglutarate dehydrogenase mitochondrial isoform 1 precursor	4967	NP_002532.2	32.74682307	1	25	25	87	1023	115.861	6.86	0.73	0.75	0.76	0.75	Significant	0.00014																
																			LEAADEGSGDVK	10	51873036	2xTMT6plex [N-Term; K12]	0.769	0.818	0.776	4.335032463	67.17	0	1648.879401	2	824.9431152	-0.271312292	-0.000223681	37.96076721
																			DPAAAPATGNK	2	51873036	2xTMT6plex [N-Term; K11]	0.638			3.814595699	60.44	0	1470.831663	2	735.9192505	-0.298438937	-0.000219477	32.84359498
																			DMVGQVAITR	6	51873036	1xTMT6plex [N-Term]	0.745	0.711		3.940532684	60.52	0	1318.735037	2	659.8711548	-0.002873729	-1.89E-06	51.32930767
																			YPNAELAWCQEEHK	4	51873036	2xTMT6plex [N-Term; K14]; 1xCarbamidomethyl [C9		1.032	0.731	3.813945532	36.49	0	2233.111209	3	745.0371094	-6.463674958	-0.004811368	50.16984762
																			VIPEDGPAAQNPENVK	2	51873036	2xTMT6plex [N-Term; K16]	0.876			3.959543467	44.32	0	2136.170104	2	1068.588135	-0.520000139	-0.000555404	57.03596742
																			GSLAAVAHAQSLVEAQPNVDK	2	51873036	2xTMT6plex [N-Term; K21]	0.201			3.103956699	4.96	0	2563.424421	3	855.1462402	-0.098796311	-8.44E-05	88.82856227
																			HWLDSPWPGFFTLDGQPR	2	51873036	1xTMT6plex [N-Term]	100			2.839716434	19.39	0	2385.197766	3	795.737793	0.44410416	0.000353092	101.593228
																			GHHVAQLDPLGILDADLDSSVPADIISSTDK	2	51873036	2xTMT6plex [N-Term; K31]				3.585083008	17.34	0	3657.93759	4	915.2397461	-0.119147741	-0.000108959	103.1534212
																			NQGYYDYVKPR	4	51873036	2xTMT6plex [N-Term; K9]	0.874	0.81		3.094927549	17.25	0	1861.000854	3	621.0045166	-0.997965857	-0.000619072	50.56664216
																			AEQFYCGDTEGK	4	51873036	2xTMT6plex [N-Term; K12]; 1xCarbamidomethyl [C6	1.1	0.791		3.327903032	54.42	0	1862.899485	2	931.9526978	-0.733325764	-0.000683056	46.06436136
																			LLDTAFDLDVFK	8	51873036	2xTMT6plex [N-Term; K12]	0.84	0.596	0.863	2.516779184	28.98	0	1855.061723	2	928.0343628	-0.147544451	-0.000136852	99.23222892
																			VVNAPIFHVNSDDPEAVMYVCK	2	51873036	2xTMT6plex [N-Term; K22]; 1xCarbamidomethyl [C21			100	2.105727434	10.78	0	2962.520706	3	988.1705933	-7.925451727	-0.007826438	73.95327942
																			VFHLPTTTFIGGQESALPLR	4	51873036	1xTMT6plex [N-Term]		100		1.61543107	11.62	0	2413.344096	3	805.1188354	-0.888013585	-0.000714361	80.90139855
																			QKFETPGIMQFTNEEK	2	51873036	3xTMT6plex [N-Term; K2; K16]				2.056055784	8.56	1	2614.415385	3	872.1420288	-1.473329082	-0.001283965	65.34665749
																			DVVVDLVCYR	4	51873036	1xTMT6plex [N-Term]; 1xCarbamidomethyl [C8	0.01	100		1.923693061	25.7	0	1466.787466	2	733.8972168	-0.211016427	-0.000154758	63.09547063
																			FLQMCNDDPDVLPDLK	3	51873036	2xTMT6plex [N-Term; K16]; 1xCarbamidomethyl [C5				1.787392139	5.74	0	2378.213625	2	1189.607666	-2.341942158	-0.002784819	94.00686286
																			ICEEAFAR	4	51873036	1xTMT6plex [N-Term]; 1xCarbamidomethyl [C2	0.777	0.773		2.967821836	33.9	0	1224.624424	2	612.8147583	-1.783443474	-0.001092024	47.10786028
																			LNVLANVIR	2	51873036	1xTMT6plex [N-Term]	100			2.427522659	36.17	0	1240.793872	2	620.9012451	1.081013055	0.000670657	77.56923092
																			IEQLSPFPFDLLLK	2	51873036	2xTMT6plex [N-Term; K14]			0.458	1.534028888	4.75	0	2118.261485	2	1059.630005	-4.131630791	-0.004375937	114.3936662
																			SWDIFFR	4	51873036	1xTMT6plex [N-Term]	0.807	0.419		3.125195265	25.55	0	1199.64106	2	600.324707	0.898029032	0.000538656	92.10221009
																			SSFDEMLPGTHFQR	2	51873036	1xTMT6plex [N-Term]				1.917850852	6.26	0	1880.916248	3	627.6432495	-0.559852064	-0.000351012	54.26642765
																			FEEFLQR	2	51873036	1xTMT6plex [N-Term]				1.338778496	18.02	0	1197.646539	2	599.3266602	-0.413892984	-0.000247849	76.32700955
																			SSPYPTDVAR	2	51873036	1xTMT6plex [N-Term]	0.747			2.431423187	21.55	0	1321.694946	2	661.3515625	0.682721836	0.000451175	35.29762226
																			QILLPFR	4	51873036	1xTMT6plex [N-Term]	0.849	100		1.786944747	19.7	0	1115.713831	2	558.359436	-2.003846887	-0.00111786	69.5706083
																			VYYDLTR	4	51873036	1xTMT6plex [N-Term]	0.741	0.741		2.796873093	26.94	0	1158.63564	2	579.8213501	-0.187103417	-0.000108392	59.50940344
156416003	SDHA	succinate dehydrogenase [ubiquinone] flavoprotein subunit, mitochondrial isoform 1	6389	NP_004159.2	37.5	1	18	18	116	664	72.645	7.39	0.68	0.52	0.71	0.63	Significant	0.0085																
																			IDEYDYSKPIQGQQK	12	156416003	3xTMT6plex [N-Term; K8; K15]	0.771	0.828	0.885	4.192679882	24.16	0	2499.369815	3	833.7949829	0.232210722	0.00019346	46.41271325
																			GVIALCIEDGSIHR	6	156416003	1xTMT6plex [N-Term]; 1xCarbamidomethyl [C6	0.858	0.833	0.721	3.398481131	63.06	0	1768.95772	3	590.3236694	-0.714827271	-0.0004215	65.39400735
																			ACALSIEESCRPGDK	10	156416003	2xTMT6plex [N-Term; K15]; 2xCarbamidomethyl [C2; C10	0.929	0.971	0.908	4.21962595	30.52	0	2151.093845	3	717.7027588	-0.056498145	-4.05E-05	44.81669798
																			GCGPEKDHVYLQLHHLPPEQLATR	6	156416003	2xTMT6plex [N-Term; K6]; 1xCarbamidomethyl [C2	0.6	0.511		7.779659748	30.22	1	3253.730456	5	651.5521851	0.418843229	0.000272561	45.08837816
																			TYFSCTSAHTSTGDGTAMITR	8	156416003	1xTMT6plex [N-Term]; 1xCarbamidomethyl [C5	0.7	0.81	0.766	6.973955154	43.54	0	2494.153989	3	837.3882446	0.50870955	0.000425646	40.20696973
																			TYFSCTSAHTSTGDGTAMITR	2	156416003	1xTMT6plex [N-Term]; 1xCarbamidomethyl [C5]; 1xOxidation [M18]			0.969	3.397200108	52.42	0	2510.148904	3	837.3882446	0.50870955	0.000425646	40.20696973
																			NTVVATGGYGR	2	156416003	1xTMT6plex [N-Term]			0.885	4.266275406	49.29	0	1323.721829	2	662.3644409	-0.169426882	-0.000112137	35.68416634
																			VGSVLQEGCGK	2	156416003	2xTMT6plex [N-Term; K11]; 1xCarbamidomethyl [C9	1.295			3.698340654	47.69	0	1591.887798	2	796.4473877	-0.188128444	-0.00014974	50.04178818
																			SMQNHAAVFR	16	156416003	1xTMT6plex [N-Term]	0.562	0.557	0.551	4.807981491	43.46	0	1389.725869	2	695.3666992	0.181882993	0.000126384	39.14424852
																			GSDWLGDQDAIHYMTEQAPAAVVELENYGMPFSR	3	156416003	1xTMT6plex [N-Term]				2.083018541	5.13	0	4026.883967	4	1007.477173	0.718703661	0.000723534	101.9687081
																			VTLEYRPVIDK	6	156416003	2xTMT6plex [N-Term; K11]	0.523	0.504	1.124	2.897606373	14.09	0	1791.078041	3	597.6973877	-0.240991209	-0.000143878	51.2320116
																			DHVYLQLHHLPPEQLATR	1	156416003	1xTMT6plex [N-Term]		0.252		2.073214769		0	2396.303628	4	599.8314209	0.094038062	5.63E-05	44.72166877
																			KHTLSYVDVGTGK	2	156416003	3xTMT6plex [N-Term; K1; K13]		0.01		2.893755198	20.7	1	2092.236951	2	1046.620361	-1.675065412	-0.001752317	40.14313021
																			SMQNHAAVFR	6	156416003	1xTMT6plex [N-Term]; 1xOxidation [M2]	0.564	0.643	1.049	3.034631968	22.11	0	1405.720784	2	695.3666992	0.181882993	0.000126384	39.14424852
																			EPIPVLPTVHYNMGGIPTNYK	4	156416003	2xTMT6plex [N-Term; K21]	100			2.509480953	11.58	0	2798.531528	3	933.5089111	-6.913687184	-0.006449391	78.6638083
																			WHFYDTVK	10	156416003	2xTMT6plex [N-Term; K8]	0.685	0.606	0.513	3.337821245	22.47	0	1553.851671	3	518.6230469	1.877164375	0.000972278	60.34973668
																			AFGGQSLK	4	156416003	2xTMT6plex [N-Term; K8]	0.847		0.723	3.783985138	38.04	0	1265.761793	2	633.3840942	-0.696121057	-0.000440562	45.83456043
																			KPFEEHWR	6	156416003	2xTMT6plex [N-Term; K1]	0.653	0.719	0.627	3.106527328	14.47	0	1586.884368	3	529.6329956	0.041550858	2.20E-05	45.90256128
																			VPPIKPNAGEESVMNLDK	4	156416003	3xTMT6plex [N-Term; K5; K18]	0.64	0.946		1.567338347	3.48	0	2625.488884	3	875.8328857	-1.820751733	-0.001593454	57.46232395
																			SMTLEIR	6	156416003	1xTMT6plex [N-Term]	0.666	0.676	0.68	2.723493099	44.67	0	1078.612796	2	539.8099976	-0.07228063	-3.90E-05	45.36574172
84043963	EIF5B	eukaryotic translation initiation factor 5B	9669	NP_056988.3	20.08196721	1	21	21	83	1220	138.742	5.49	1.48	1.34	1.25	1.36	Significant	0.0024																
																			APIICVLGHVDTGK	6	84043963	2xTMT6plex [N-Term; K14]; 1xCarbamidomethyl [C5	0.784	1.135	0.972	3.426182032	56.78	0	1938.124674	3	646.7124634	-0.948062478	-0.000612488	68.53859254
																			ETVAVKPTENNEEEFTSK	5	84043963	3xTMT6plex [N-Term; K6; K18]	1.77	2.699		4.751430988	22.57	0	2739.465566	3	913.8266602	-0.050579665	-4.62E-05	55.54083239
																			AETPTAAEDDNEGDK	4	84043963	2xTMT6plex [N-Term; K15]	1.126	1.361		3.775798798	47.06	0	2020.971129	3	674.3253174	-4.814611858	-0.003243397	33.31760113
																			TSEVPYAGINIGPVHIK	3	84043963	2xTMT6plex [N-Term; K16]	1.266			2.903973818	30.28	0	2140.21666	3	714.0769653	-0.148253171	-0.000105765	61.49853399
																			SPDSDVAATLK	2	84043963	2xTMT6plex [N-Term; K11]			1.191	4.46873951	48.83	0	1561.883758	2	781.4458618	0.440898888	0.000344316	51.02269463
																			DAQEMADSLGVR	6	84043963	1xTMT6plex [N-Term]	100	0.37	1.834	3.810830593	49.3	0	1520.757623	2	760.881897	-0.726739535	-0.000552597	50.34981167
																			AIIVEFAQQGLNAALFYENK	2	84043963	2xTMT6plex [N-Term; K20]			0.525	4.250235081	30.9	0	2697.501608	3	899.8359985	-3.027265862	-0.002722018	111.5865355
																			AETPTAAEDDNEGDKK	4	84043963	3xTMT6plex [N-Term; K15; K16]	1.17		1.13	4.755679131	24.94	1	2378.229024	3	793.4138794	-0.815361509	-0.000646372	36.78584796
																			KSPDSDVAATLK	4	84043963	3xTMT6plex [N-Term; K1; K12]	1.562		0.82	3.740358353	29.47	1	1919.141653	3	640.3857422	0.531473275	0.000339991	44.40817321
																			DAQEMADSLGVR	2	84043963	1xTMT6plex [N-Term]; 1xOxidation [M5]	1.902			2.517567873	39.61	0	1536.752538	2	760.881897	-0.726739535	-0.000552597	50.34981167
																			EVEAAQGVK	6	84043963	2xTMT6plex [N-Term; K9]	1.886	1.918	1.815	4.262821674	58.82	0	1388.814951	2	694.9117432	0.906570112	0.000629529	41.55706839
																			AIIVEFAQQGLNAALFYENKDPR	2	84043963	2xTMT6plex [N-Term; K20]			2.908	3.025282145	26.42	1	3065.682426	3	1022.564148	-1.479452766	-0.001511844	107.2380203
																			IEPIPGESPK	2	84043963	2xTMT6plex [N-Term; K10]	1.348			3.550065756	34.25	0	1524.903766	2	762.9535522	-2.58228611	-0.001968869	55.18896832
																			IPGMLIIDTPGHESFSNLR	6	84043963	1xTMT6plex [N-Term]	1.316	100		3.21595192	20.78	0	2326.242668	3	776.0852051	-0.690222756	-0.000535209	80.2086649
																			GLLLPPPMK	12	84043963	2xTMT6plex [N-Term; K9]	1.796	1.241		2.244058132	20.43	0	1423.9111	2	712.4580688	-1.57219414	-0.001119332	70.19848493
																			DELIHELK	6	84043963	2xTMT6plex [N-Term; K8]		1.21	1.364	2.707799435	24.58	0	1454.861901	3	485.625946	0.951330683	0.000461352	56.3568412
																			EMSSDSEYDSDDDR	2	84043963	1xTMT6plex [N-Term]				2.251902819	17.45	0	1879.733711	2	940.3705444	0.053612481	5.04E-05	29.4466072
																			AMQEALAK	2	84043963	2xTMT6plex [N-Term; K8]			1.261	3.28217721	34.56	0	1319.775728	2	660.3920288	0.797512157	0.000526269	48.453916
																			KIPQQLESK	2	84043963	3xTMT6plex [N-Term; K1; K9]		1.323		2.725039482	19.3	1	1758.109231	3	586.7077637	-0.28062214	-0.000164455	48.8435517
																			AQVMEVK	2	84043963	2xTMT6plex [N-Term; K7]	1.164			2.579003096	25.32	0	1262.754265	2	631.8805542	-0.342894556	-0.000216496	49.78153873
																			ASVMLEHDPQYAVILAFDVR	1	84043963	1xTMT6plex [N-Term]				1.319045901		0	2503.321646	3	835.1123047	0.285489682	0.000238224	93.50011261
																			LKEGDTIIVPGVEGPIVTQIR	2	84043963	2xTMT6plex [N-Term; K2]		1.892		1.408719778	0.25	1	2692.601322	3	898.2047729	-0.578177813	-0.000518934	70.11174784
39777597	TGM2	protein-glutamine gamma-glutamyltransferase 2 isoform a	7052	NP_004604.2	41.48471616	1	20	20	102	687	77.28	5.22	1.07	1.46	1.21	1.24	Significant	0.0083																
																			VVTNYNSAHDQNSNLLIEYFR	6	39777597	1xTMT6plex [N-Term]	0.508	0.875	100	3.581448078	31.33	0	2726.373558	3	909.4620972	-0.667529982	-0.000606645	76.5579332
																			KLVAEVSLQNPLPVALEGCTFTVEGAGLTEEQK	2	39777597	3xTMT6plex [N-Term; K1; K33]; 1xCarbamidomethyl [C19	0.702			4.936905861	30.15	1	4215.318971	4	1054.586792	1.510345669	0.001591647	94.4602965
																			VGQSMNMGSDFDVFAHITNNTAEEYVCR	4	39777597	1xTMT6plex [N-Term]; 1xCarbamidomethyl [C27		100		2.95531559	12.34	0	3421.544875	4	856.1419678	0.340753605	0.000291476	92.46736167
																			DCLTESNLIK	7	39777597	2xTMT6plex [N-Term; K10]; 1xCarbamidomethyl [C2	0.985		100	4.062173843	56.34	0	1650.913679	2	825.9608765	0.483192851	0.000398855	62.92370198
																			SEGTYCCGPVPVR	2	39777597	1xTMT6plex [N-Term]; 2xCarbamidomethyl [C6; C7	1.155			3.628825903	24.62	0	1710.814092	2	855.911377	0.809641595	0.000692573	39.37247953
																			GQPFWLTLHFEGR	2	39777597	1xTMT6plex [N-Term]	0.738			2.932102203	29.17	0	1816.969605	3	606.3279419	-0.182824416	-0.000110729	92.94555331
																			SVPLCILYEK	8	39777597	2xTMT6plex [N-Term; K10]; 1xCarbamidomethyl [C5	0.889	0.894	1.156	3.249229431	36.05	0	1679.980636	2	840.4934082	-0.652521658	-0.000548112	77.47118146
																			YPEGSSEER	2	39777597	1xTMT6plex [N-Term]	1.204			2.75749445	25.81	0	1282.611276	2	641.8081055	-1.8256837	-0.001170821	29.52525576
																			LVVNFESDK	6	39777597	2xTMT6plex [N-Term; K9]	0.857	1.372	1.006	3.074627638	36.35	0	1508.872465	2	754.9403076	0.578660155	0.000436562	68.85112278
																			VVSGMVNCNDDQGVLLGR	4	39777597	1xTMT6plex [N-Term]; 1xCarbamidomethyl [C8		100		2.070319176	12.28	0	2162.089538	2	1081.547363	-0.965823784	-0.001044099	57.70085185
																			LAEKEETGMAMR	16	39777597	2xTMT6plex [N-Term; K4]	1.108	1.574	1.19	3.573568821	24.19	1	1823.975961	3	608.6633911	-0.186861101	-0.00011361	40.92768939
																			DLYLENPEIK	9	39777597	2xTMT6plex [N-Term; K10]	1.269	1.986	1.134	3.380897045	33.84	0	1691.962009	2	846.4841309	-0.604967041	-0.000511791	64.35824123
																			TVEIPDPVEAGEEVK	9	39777597	2xTMT6plex [N-Term; K15]	0.595	0.662	1.914	2.36033535	18.45	0	2070.137072	2	1035.574097	1.857113438	0.00192224	69.24869162
																			ALLVEPVINSYLLAER	3	39777597	1xTMT6plex [N-Term]	0.709	0.01		1.542607307	9.73	0	2029.189493	2	1015.097656	-0.718168512	-0.00072865	102.9068563
																			YLLNLNLEPFSEK	4	39777597	2xTMT6plex [N-Term; K13]	100	100		1.821759343	12.16	0	2038.162499	2	1019.583557	-1.305893023	-0.001330811	90.72888167
																			SLIVGLK	8	39777597	2xTMT6plex [N-Term; K7]	1.303	1.032	1.287	3.052648544	42.58	0	1187.812766	2	594.4104004	0.638317142	0.000379101	46.12344983
																			SSPVYVGR	3	39777597	1xTMT6plex [N-Term]	1.085	1.167		2.534351349	26.97	0	1093.620325	2	547.3132324	-1.039159774	-0.000568223	36.83491089
																			YDAPFVFAEVNADVVDWIQQDDGSVHK	2	39777597	2xTMT6plex [N-Term; K27]				2.262477875	6.89	0	3522.75817	4	881.4501953	5.899295098	0.005195447	111.6379872
																			LVVNFESDKLK	3	39777597	3xTMT6plex [N-Term; K9; K11]	0.839			1.696676254	6.36	1	1979.214424	3	660.4093628	-0.449294974	-0.000296417	76.21843457
																			DEREDITHTYK	2	39777597	2xTMT6plex [N-Term; K11]			1.098	4.00483799	10.43	1	1864.980512	3	622.331665	-0.037696272	-2.34E-05	35.51976746
4506723	RPS3A	40S ribosomal protein S3a isoform 1	6189	NP_000997.1	64.77272727	1	17	17	140	264	29.926	9.73	1.18	1.17	1.31	1.22	Significant	0.0014																
																			VFEVSLADLQNDEVAFR	6	4506723	1xTMT6plex [N-Term]			1.573	4.74709177	58.77	0	2181.138914	3	727.7171631	-0.906889596	-0.000659351	96.99966387
																			ACQSIYPLHDVFVR	19	4506723	1xTMT6plex [N-Term]; 1xCarbamidomethyl [C2	1.089	2.031	1.702	4.761203766	38.68	0	1934.015569	3	645.3447876	2.192734659	0.001413594	64.04893645
																			LMELHGEGSSSGK	16	4506723	2xTMT6plex [N-Term; K13]	1.146	1.15	100	4.089144707	54.76	0	1789.951855	3	597.3217773	-0.601054574	-0.00035862	50.7213212

Bhat et al,2017. Cigarette smoke and chewing tobacco alter expression of different sets of miRNAs in oral keratinocytes

Supplementary Table S2(A). List of all proteins and peptides identified in OKF6/TERT1 cells chronically treated with chewing tobacco using Sequest and Mascot search algorithms

Accession	Gene_Symbol	Description	Gene_ID	Refseq_Accession	Coverage	Proteins	#Unique_Peptides	#Peptides	#PSMS	#AAs	MW [kDa]	calc. pI	OKF6-STE-6M/OKF6P_R1	OKF6-STE-6M/OKF6P_R2	OKF6-STE-6M/OKF6P_R3	Median fold change	Status	P-value (6M)	Sequence	#PSMs	Protein Accessions	Modifications	OKF6-STE-6M/OKF6P_R1	OKF6-STE-6M/OKF6P_R2	OKF6-STE-6M/OKF6P_R3	Xcorr	Ionscore	# Missed Cleavages	Theo. MH+ [Da]	Charge	m_z	DeltaM [ppm]	Deltam/z [Da]	RT [min]
																			KTSYAQHQQVR	8	4506723	2xTMT6plex [N-Term; K1]	1.11	1.187		5.189045906	29.46	1	1804.022986	3	602.0126343	0.201428715	0.000121127	21.3770744
																			NCLTNFHGMDLTR	4	4506723	1xTMT6plex [N-Term]; 1xCarbamidomethyl [C2		1.363	1.091	4.766293049	40.31	0	1807.878089	3	603.2966309	-1.520960894	-0.000916571	61.6058981
																			ATGDETGAK	13	4506723	2xTMT6plex [N-Term; K9]	1.298	1.535		3.994974852	54.08	0	1307.720716	2	654.3649292	1.426801927	0.000932929	22.19339575
																			LMELHGEGSSSGK	6	4506723	2xTMT6plex [N-Term; K13]; 1xOxidation [M2		0.957		3.358424902	28.56	0	1805.94677	3	597.3217773	-0.601054574	-0.00035862	50.7213212
																			ADGYEPPVQESV	14	4506723	1xTMT6plex [N-Term]	1.236	1.3	1.308	2.937297344	52.52	0	1519.747769	2	760.3766479	-1.1515408	-0.000875026	48.11848646
																			KWQTMIEAHVDVK	2	4506723	3xTMT6plex [N-Term; K1; K13]	1.484			2.954111814	18.17	1	2272.30907	4	568.8320923	-1.11361144	-0.000632617	64.77969304
																			LIPDSIGK	4	4506723	2xTMT6plex [N-Term; K8]	1.25	1.122		3.188129425	45.08	0	1300.824059	2	650.9155884	-0.122008958	-7.94E-05	64.05649285
																			LFCVGFTK	8	4506723	2xTMT6plex [N-Term; K8]; 1xCarbamidomethyl [C3	2.025	100	1.08	3.79857707	27.77	0	1429.827764	2	715.4169312	-0.824340765	-0.000589333	70.83493892
																			EVQTNDLK	2	4506723	2xTMT6plex [N-Term; K8]	1.36			3.198972464	43	0	1404.809865	2	702.9084473	-0.176065641	-0.000123669	41.47568887
																			APAMFNIR	12	4506723	1xTMT6plex [N-Term]	1.06	1.324	0.839	2.815441847	30.4	0	1148.644765	2	574.8258057	-0.3748869	-0.000215306	66.50750159
																			KVVDPFSK	5	4506723	3xTMT6plex [N-Term; K1; K8]	0.814	0.931	1.21	2.852342606	27.72	1	1607.01354	3	536.3430786	0.711039338	0.000380883	48.13167286
																			TTDGYLLR	2	4506723	1xTMT6plex [N-Term]	1.905			3.2638309	50.67	0	1167.657104	2	584.3331299	1.609287194	0.000939548	44.69025133
																			MMEIMTR	5	4506723	1xTMT6plex [N-Term]	1.421	1.426	1.395	2.987140417	34.9	0	1140.577673	2	570.7918701	-1.060607842	-0.000604853	46.60951427
																			KDWYDVK	8	4506723	3xTMT6plex [N-Term; K1; K7]	1.115	1.218	1.198	2.983943701	10.14	1	1640.961504	3	547.6586914	0.010121353	5.54E-06	46.98522047
																			IASDGLK	6	4506723	2xTMT6plex [N-Term; K7]	1.281	1.26		3.143663406	27.14	0	1161.724345	2	581.3659668	0.268664207	0.000156057	42.42339918
469608410	TAGLN2	transgelin-2 isoform a	8407	NP_001264153.1	76.81818182	1	14	14	126	220	24.438	8.25	1.06	0.84	0.8	0.9	Significant	0.008																
																			NVIGLQMGTNR	6	469608410	1xTMT6plex [N-Term]	0.958	0.95	0.98	3.83445859	56.69	0	1431.793949	2	716.4003906	-0.310044613	-0.00022196	49.24932707
																			GASQAGMTGYGMPR	9	469608410	1xTMT6plex [N-Term]	1.133	2.234	1.003	4.89972353	96.58	0	1612.777312	2	806.892395	0.124802762	0.00010064	43.30727954
																			GASQAGMTGYGMPR	16	469608410	1xTMT6plex [N-Term]; 1xOxidation [M	0.971	0.759	1.076	3.117140293	64.58	0	1628.772227	2	806.892395	0.124802762	0.00010064	43.30727954
																			TLMNLGGLAVAR	4	469608410	1xTMT6plex [N-Term]	1.226	1.118		4.122649193	70.98	0	1444.850735	2	722.9284668	-0.746226737	-0.000539093	70.73420627
																			YGINTTDIFQTVDLWEGK	8	469608410	2xTMT6plex [N-Term; K18]	1.277	1.54	100	3.671316624	35.14	0	2558.354275	3	853.4567871	0.599048435	0.000510859	107.2627802
																			QYDADLEQILIQWITTQCR	12	469608410	1xTMT6plex [N-Term]; 1xCarbamidomethyl [C18	1.314		1.22	5.10498333	48.73	0	2623.338753	3	875.1176758	-0.106305848	-9.30E-05	113.1431067
																			QMEQISQFLQAAER	11	469608410	1xTMT6plex [N-Term]	1.323	100		2.916625977	32.65	0	1907.984662	2	954.4951782	-0.829338264	-0.000791182	86.23325824
																			KIQASTMAFK	7	469608410	3xTMT6plex [N-Term; K1; K10]	0.673	0.679	0.609	5.253075123	43.76	1	1812.102037	3	604.7058716	0.565203323	0.000341402	46.49137965
																			IQASTMAFK	12	469608410	2xTMT6plex [N-Term; K9]	1.197	0.768	2.194	3.793005705	42.39	0	1454.844142	2	727.925354	-0.488672406	-0.000355471	64.00129525
																			NFSDNQLQEGK	2	469608410	2xTMT6plex [N-Term; K11]	1.211			3.414488792	49.71	0	1737.917184	2	869.4606323	-1.838793927	-0.001597836	45.80388633
																			DDGLFSGDPNWFPK	13	469608410	2xTMT6plex [N-Term; K14]	1.964	100	100	2.638920546	21.23	0	2053.043112	2	1027.024292	-0.87920493	-0.000902523	82.51285976
																			GASQAGMTGYGMPR	2	469608410	1xTMT6plex [N-Term]; 2xOxidation [M7; M12]	0.554			1.69308269	19.9	0	1644.767142	2	806.892395	0.124802762	0.00010064	43.30727954
																			ENFQNWLK	14	469608410	2xTMT6plex [N-Term; K8]	1.112	1.196	1.434	3.273169518	33.5	0	1536.857484	2	768.9318237	-0.724419196	-0.000556665	62.76184493
																			NVIGLQMGTNR	3	469608410	1xTMT6plex [N-Term]; 1xOxidation [M7]		1.121	0.887	2.357671261	25.04	0	1447.788864	2	716.4003906	-0.310044613	-0.00022196	49.24932707
																			GPAYGLSR	4	469608410	1xTMT6plex [N-Term]			0.861	2.541151762	29.13	0	1049.59411	2	525.2962646	-8.438760318	-0.004428637	33.21757655
																			DVGRPQPGR	2	469608410	1xTMT6plex [N-Term]			100	2.84564209	13.4	0	1210.685384	3	404.2335205	0.515239917	0.000207931	17.32400613
																			DGTVLCELINALYPEGQAPVK	1	469608410	2xTMT6plex [N-Term; K21]; 1xCarbamidomethyl [C6				1.582317829		0	2745.489723	3	915.8320923	-2.913773256	-0.002666578	107.3266932
224809329	FKBP5	peptidyl-prolyl cis-trans isomerase FKBP5 isoform 1	2289	NP_001139248.1	37.19912473	1	18	18	102	457	51.18	5.9	1.13	1.2	1.16	1.16	Significant	0.0003																
																			KGEGYSNPNEGATVEIHLEGR	4	224809329	2xTMT6plex [N-Term; K1]	0.827	1.639		6.391740322	47.22	1	2715.410227	4	679.6082153	0.29621238	0.000201085	56.12003615
																			GTDSQAMEEEKPEGHV	14	224809329	2xTMT6plex [N-Term; K11]	1.169	1.14	0.902	5.76959753	63.3	0	2202.074883	2	1101.541504	0.385342255	0.000424276	31.59728431
																			GTDSQAMEEEKPEGHV	10	224809329	2xTMT6plex [N-Term; K11]; 1xOxidation [M7	1.149	0.994	1.148	3.763847828	30.35	0	2218.069798	2	1101.541504	0.385342255	0.000424276	31.59728431
																			EEQCILYLGPR	4	224809329	1xTMT6plex [N-Term]; 1xCarbamidomethyl [C4	0.978	1.277		3.764195681	34.15	0	1606.846044	2	803.9277344	1.336904683	0.0010741	64.029563
																			AWDIGVATMK	4	224809329	2xTMT6plex [N-Term; K10]	0.784	1.02		2.618383169	48.61	0	1549.881256	2	775.4442139	-0.067724946	-5.25E-05	77.51100427
																			FAEQDAKEEANK	6	224809329	3xTMT6plex [N-Term; K7; K12]	0.9	0.71	0.77	3.367360353	15.19	1	2067.132545	3	689.7154541	-0.356090041	-0.000245362	29.75986842
																			EKLEQAAIVK	14	224809329	3xTMT6plex [N-Term; K2; K10]	1.342	1.272	1.279	4.822957039	25.24	1	1816.151096	3	606.0543823	-1.377741835	-0.000834062	44.91154506
																			GEAQLLMNEFESAK	6	224809329	2xTMT6plex [N-Term; K14]	1.289			2.71860528	27.32	0	2025.072698	3	675.6943359	-2.095424774	-0.001414463	87.79094514
																			AKESWEMDTK	4	224809329	3xTMT6plex [N-Term; K2; K10]	0.724	0.591		2.524297714	19.9	1	1912.04531	3	638.0202026	0.38936847	0.000248163	50.62910209
																			YGFGEAGKPK	4	224809329	3xTMT6plex [N-Term; K8; K10]	1.311	1.179	1.081	2.42957592	9.9	0	1741.025167	3	581.012207	-1.780190378	-0.001033119	51.26707798
																			VLEVNPQNK	4	224809329	2xTMT6plex [N-Term; K9]	0.911			4.044384003	33.85	0	1498.899349	2	749.953186	-0.169123889	-0.00012675	47.84245765
																			LQISMCQK	6	224809329	2xTMT6plex [N-Term; K8]; 1xCarbamidomethyl [C6	1.02	1.121	1.183	3.682898998	44.71	0	1465.827112	2	733.4180908	1.222961842	0.000896325	60.02410201
																			GEDLFEDGGIIR	6	224809329	1xTMT6plex [N-Term]	100	100	100	2.776982069	29.48	0	1549.805953	2	775.4064941	-0.155663843	-0.000120624	61.26079897
																			KTSEGVTNEK	2	224809329	3xTMT6plex [N-Term; K1; K10]			0.97	2.924523592	5.53	1	1780.041939	3	594.019165	0.563198633	0.000334172	31.52210658
																			ESWEMDTK	2	224809329	2xTMT6plex [N-Term; K8]	1.546			3.185751915	36.07	0	1483.750301	2	742.3793945	0.816163271	0.000605491	55.60411221
																			NEPFVFSLGK	4	224809329	2xTMT6plex [N-Term; K10]	100	100		2.188954592	17.92	0	1595.91975	2	798.4637451	0.29047474	0.000231787	80.50120485
																			IYANMFK	4	224809329	2xTMT6plex [N-Term; K7]	1.159			2.913974762	31.49	0	1344.775	2	672.8916016	0.688795523	0.000463138	67.28598625
																			LEQAAIVK	2	224809329	2xTMT6plex [N-Term; K8]	1.551			2.949755669	9.76	0	1329.850608	2	665.4302979	2.038682472	0.001355572	50.21142181
																			FAEQDAK	2	224809329	2xTMT6plex [N-Term; K7]	1.348			2.425316572	26.39	0	1266.709423	2	633.8582764	-0.115982105	-7.35E-05	39.69033671
13259510	DCTN1	dynactin subunit 1 isoform 1	1639	NP_004073.2	22.0657277	1	22	22	95	1278	141.607	5.81	1.15	1.03	0.78	0.99	Significant	0.012																
																			LVLTQEQLHQLHSR	6	13259510	1xTMT6plex [N-Term]	1.081	0.949	0.997	5.172646523	55.96	0	1931.10241	3	644.3724365	0.179565988	0.000115587	43.66649886
																			AFLQGQEATDIALLLR	5	13259510	1xTMT6plex [N-Term]	1.318			3.774219513	48.73	0	2045.159256	2	1023.084229	0.941125365	0.000962376	98.06004744
																			AEITDAEGLGLK	6	13259510	2xTMT6plex [N-Term; K12]	1.524	1.257	1.001	4.713686466	94.03	0	1674.967822	2	837.9867554	-0.948190044	-0.000794094	63.38754399
																			QLTAHLQDVNR	6	13259510	1xTMT6plex [N-Term]	1.061	0.572	0.991	4.465610981	63.39	0	1523.849155	3	508.6209412	-0.580722619	-0.000294978	36.5783779
																			LSHEGPGSELPAGALYR	4	13259510	1xTMT6plex [N-Term]		0.536	0.53	4.272895813	25.39	0	1983.049705	3	661.6845703	-5.318854324	-0.003515851	46.87020025
																			ELTNQQEASVER	16	13259510	1xTMT6plex [N-Term]	1.285	1.301	1.333	4.963910103	52.27	0	1632.839044	2	816.9232788	0.145229984	0.000118569	31.35276081
																			MQEQQADLQR	4	13259510	1xTMT6plex [N-Term]	1.679	1.26		3.910594463	46.86	0	1475.747392	2	738.3765869	-1.013060828	-0.000747511	32.85700494
																			GSDGAASSYQLK	2	13259510	2xTMT6plex [N-Term; K12]	0.508			3.224681854	34.28	0	1641.884821	2	821.4454956	-0.673853031	-0.000553195	44.6821254
																			IKGEELSEANVR	6	13259510	2xTMT6plex [N-Term; K2]	1.205	1.265	1	4.044868469	33.25	1	1803.037633	3	601.6836548	-0.677769842	-0.000407348	40.10745084
																			AKEEQQDDTVYMGK	6	13259510	3xTMT6plex [N-Term; K2; K14]	0.914	1.169	1.028	4.975129604	30.34	1	2329.231273	3	777.0825195	0.743835003	0.000577521	39.49430046
																			FELSENCSERPGLR	2	13259510	1xTMT6plex [N-Term]; 1xCarbamidomethyl [C7			0.99	3.175625801	8.73	0	1922.959176	3	641.6583862	0.743349846	0.000476477	50.92666889
																			RVEAAQETVADYQQTIK	2	13259510	2xTMT6plex [N-Term; K17]		0.751		4.895591736	7.24	1	2408.318559	3	803.4404297	-4.909195861	-0.003940969	50.05070755
																			SLDFLIELLHK	2	13259510	2xTMT6plex [N-Term; K11]	2.707			2.135416031	17.47	0	1786.087878	3	596.0337524	-0.657204942	-0.000391275	108.1406494
																			EQLDMAGAR	2	13259510	1xTMT6plex [N-Term]			1	2.910925388	53.79	0	1219.630237	2	610.3189697	0.348862395	0.000212742	38.57414957
																			IQLEQVQEWK	5	13259510	2xTMT6plex [N-Term; K10]	100	100		2.049690247	14.72	0	1759.015441	2	880.0112305	-0.146054716	-0.000128456	63.48446626
																			AESLQQEVEALK	2	13259510	2xTMT6plex [N-Term; K12]			100	1.754936814	12.47	0	1803.0264	2	902.0160522	-0.871799666	-0.000785939	76.62816526
																			SLSDTVEK	4	13259510	2xTMT6plex [N-Term; K8]	1.072	1.25		3.605438471	41.17	0	1336.772417	2	668.8909302	1.620770697	0.001083301	27.94344031
																			VDELTTDLEILK	4	13259510	2xTMT6plex [N-Term; K12]				1.480634093	7.91	0	1847.077767	2	924.0413818	-1.234132188	-0.001139769	88.48914011
																			AIEMELR	4	13259510	1xTMT6plex [N-Term]	1.106	1.11		2.391891718	29.48	0	1090.612796	2	545.8100586	0.040442859	2.21E-05	59.31461459
																			EFEETMDALQADIDQLEAEK	3	13259510	2xTMT6plex [N-Term; K20]				1.32279706	0.2	0	2783.369727	3	928.4599609	-1.579817857	-0.001465789	107.2319756
																			DSPLLLQQISAMR	2	13259510	1xTMT6plex [N-Term]				1.692663312	13.62	0	1700.956657	2	850.9814453	-0.612969764	-0.000521317	90.63801238
																			ASEQIYGTPSSSPYECLR	2	13259510	1xTMT6plex [N-Term]; 1xCarbamidomethyl [C16				0.833113015	5.49	0	2274.090978	2	1137.547974	-1.01455676	-0.001153597	49.56412094
117190174	HNRNPC	heterogeneous nuclear ribonucleoproteins C1/C2 isoform b	3183	NP_004491.2	43.3447099	1	16	16	167	293	32.318	5.08	1.18	1.05	1.25	1.16	Significant	0.0025																
																			SAAEMYGSSFDLDYDFQR	17	117190174	1xTMT6plex [N-Term]; 1xOxidation [M5]	1.053	0.742	0.382	4.061746597	39.9	0	2347.038608	3	777.6868896	1.039326427	0.000807572	80.71834646
																			SAAEMYGSSFDLDYDFQR	33	117190174	1xTMT6plex [N-Term]	1.079	1.24	1.382	4.444452763	60.54	0	2331.043693	3	777.6868896	1.039326427	0.000807572	80.71834646
																			MIAGQVLDINLAAEPK	12	117190174	2xTMT6plex [N-Term; K16]	100	100		2.740797281	33.76	0	2141.240432	2	1071.124146	0.272013183	0.000291223	86.37850355
																			SEEEQSSSSVK	14	117190174	2xTMT6plex [N-Term; K11]	1.361	1.664	1.328	4.18208313	52.81	0	1654.85358	2	827.9312134	0.948596197	0.000784894	30.58088052
																			GFAFVQYVNER	10	117190174	1xTMT6plex [N-Term]	5.64	1.313	0.657	3.519525528	49.88	0	1558.821543	2	779.9141846	-0.289295062	-0.00022548	77.55869987
																			VFIGNLNTLVVK	14	117190174	2xTMT6plex [N-Term; K12]	1.342	1.674	100	3.668082476	51.03	0	1775.119512	2	888.0630493	-0.388918708	-0.000345189	93.35387893
																			KSDVEAIFSK	8	117190174	3xTMT6plex [N-Term; K1; K10]	1.504	1.578	1.442	4.19664669	33.17	1	1811.088161	3	604.3665771	-1.64713389	-0.000994368	68.16888674
																			SDVEAIFSK	8	117190174	2xTMT6plex [N-Term; K9]	2.289	2.123	100	3.958196878	49.08	0	1453.830266	2	727.4185181	-0.348615103	-0.000253414	72.89174204
																			VDSLLENLEK	10	117190174	2xTMT6plex [N-Term; K10]	1.707	100	100	2.883940697	32.26	0	1617.946359	2	809.4766235	-0.239915054	-0.000194085	83.17034919
																			QKVDSLLENLEK	4	117190174	3xTMT6plex [N-Term; K2; K12]	0.786	0.567		2.789429903	18.41	1	2103.262831	3	701.7590942	-0.048284577	-3.39E-05	68.08978603
																			LKGDDLQAIK	4	117190174	3xTMT6plex [N-Term; K2; K10]	0.964	1.145		3.714975834	13.98	1	1788.119796	3	596.710083	-2.292920515	-0.001366672	51.45370713
																			AAVAGEDGR	8	117190174	1xTMT6plex [N-Term]	1.253	0.872	0.952	3.541684628	36.05	0	1074.574103	2	537.7905884	-0.188523236	-0.000101291	18.97332099
																			LKGDDLQAIKK	3	117190174	4xTMT6plex [N-Term; K2; K10; K11]	0.511	0.454		2.501829624	3.55	2	2145.377691	4	537.0985107	-2.553238655	-0.001369415	45.96120194
																			IVGCSVHK	10	117190174	2xTMT6plex [N-Term; K8]; 1xCarbamidomethyl [C4	1.503	1.629	0.957	3.227006912	35.74	0	1357.802612	3	453.2721863	-0.446782252	-0.000202214	41.68239439
																			NDKSEEEQSSSSVK	2	117190174	3xTMT6plex [N-Term; K3; K14]			100	2.616660118	17.21	1	2241.181346	3	747.7321777	0.283026238	0.000211438	27.66442174
																			GDDLQAIK	6	117190174	2xTMT6plex [N-Term; K8]	1.327	0.497	1.583	3.083981276	48.67	0	1317.777837	2	659.3930664	0.773493431	0.000509646	48.91800769
																			KELTQIK	4	117190174	3xTMT6plex [N-Term; K1; K7]	1.004	0.87		3.251970291	21.54	1	1547.01354	3	516.3431396	0.856977188	0.000441918	48.02411524
21626466	MATR3	matrin-3 isoform a	9782	NP_061322.2	30.93270366	1	19	19	83	847	94.565	6.25	1.16	1.23	1.04	1.14	Significant	0.0023																
																			GPSLNPVLDYDHGSR	14	21626466	1xTMT6plex [N-Term]	1.002	0.948	1.04	4.116985321	47.89	0	1855.949991	3	619.3219604	0.720303706	0.000445616	51.63696746
																			EDAMAMVDHCLK	2	21626466	2xTMT6plex [N-Term; K12]; 1xCarbamidomethyl [C10	1.659			4.226758003	50.13	0	1877.932382	3	626.6491699	0.305999103	0.000191549	68.28657873
																			SQAFIEMETR	4	21626466	1xTMT6plex [N-Term]	1.559	1.283		4.5235219	74.3	0	1440.735431	2	720.8712769	-0.106549099	-7.68E-05	60.12026783
																			VIHLSNLPHSGYSDSAVLK	2	21626466	2xTMT6plex [N-Term; K19]	0.823			4.798052788	42.52	0	2495.402229	3	832.4727783	0.622312876	0.00051764	65.78815494
																			GYPHLCSICDLPVHSNK	12	21626466	2xTMT6plex [N-Term; K17]; 2xCarbamidomethyl [C6; C9	0.994	1.176	1	6.069530964	41.95	0	2455.262641	4	614.5671387	-6.482582447	-0.003979111	48.80288859
																			GDADQASNILASFGLSAR	6	21626466	1xTMT6plex [N-Term]			0.576	3.726988792	37.91	0	2022.045348	2	1011.525391	-0.911670948	-0.00092172	91.37681447
																			DLSAAGIGLLAAATQSLSMPASLGR	4	21626466	1xTMT6plex [N-Term]	0.967	2.141		4.623944759	50.79	0	2600.427902	3	867.4789429	-2.163731098	-0.001875542	111.5014489
																			INEAFIEMATTEDAQAAVDYYTTTPALVFGKPVR	2	21626466	2xTMT6plex [N-Term; K31]			100	3.86925149	12.6	0	4191.172417	4	1048.543335	-4.988464842	-0.005226879	107.6254986
																			KDGSASAAAK	2	21626466	3xTMT6plex [N-Term; K1; K10]		2.015		4.06033802	37.56	1	1592.957482	3	531.6571655	-0.337879683	-0.000179409	23.57153369
																			YQLLQLVEPFGVISNHLILNK	2	21626466	2xTMT6plex [N-Term; K21]			100	2.219516277	18.92	0	2896.706456	3	966.2380981	-2.31814183	-0.002238325	107.4672686
																			GAPPSSNIEDFHGLLPK	6	21626466	2xTMT6plex [N-Term; K17]	1.466	1.115	1.44	3.4374578	35.51	0	2237.233038	3	746.4153442	-0.696782646	-0.000519622	63.48369966
																			SQESGYYDR	4	21626466	1xTMT6plex [N-Term]		1.458	1.208	3.838218927	66.39	0	1333.622175	2	667.3156738	1.421726697	0.000948023	23.41888216
																			DGSASAAAK	2	21626466	2xTMT6plex [N-Term; K9]	100			2.857575893	49.19	0	1235.699587	2	618.3531494	-0.456738742	-0.000282196	22.93858391
																			ITPENLPQILLQLK	4	21626466	2xTMT6plex [N-Term; K14]	100	100		2.545922518	16.17	0	2078.298933	2	1039.651367	-1.672225754	-0.001737692	109.0531311
																			LAEPYGK	6	21626466	2xTMT6plex [N-Term; K7]	1.423	1.397		3.089419842	28.38	0	1235.739995	2	618.3734131	-0.360406013	-0.000222684	44.03657456
																			FDSEYER	2	21626466	1xTMT6plex [N-Term]	1.988			2.992874861	45.93	0	1174.557784	2	587.7823486	-0.309286082	-0.000181637	39.2053161
																			LCSLFYTNEEVAK	1	21626466	2xTMT6plex [N-Term; K13]; 1xCarbamidomethyl [C2				1.187163472		0	2032.082535	2	1016.544128	-0.764966007	-0.000777237	86.01616302
																			MDYEDDR	2	21626466	1xTMT6plex [N-Term]	1.458			2.826426744	35.9	0	1172.509119	2	586.758667	0.800168084	0.000469102	33.62575473
																			MDYEDDR	2	21626466	1xTMT6plex [N-Term]; 1xOxidation [M1]	1.272			1.925581574	17.3	0	1188.504034	2	586.758667	0.800168084	0.000469102	33.62575473
																			ALWFQGR	2	21626466	1xTMT6plex [N-Term]		1.419	1.396	2.63304472	23.23	0	1106.63083	2	553.8184814	-1.033379284	-0.000571785	68.35595013
386642862	TARS	threonine--tRNA ligase, cytoplasmic isoform [illegible]	6897	NP_001245367.1	31.21693122	1	22	22	104	756	86.806	6.77	0.8	1.07	0.89	0.92	Significant	0.0074																
																			NELSGALTGLTR	4	386642862	1xTMT6plex [N-Term]	1.287	1.189		4.082652569	81.1	0	1460.827023	2	730.9168701	-0.382745949	-0.000279563	69.80984275
																			FMADIDLDPGCTLNK	8	386642862	2xTMT6plex [N-Term; K15]; 1xCarbamidomethyl [C11	1.211	100	100	3.543428421	47.98	0	2168.113183	3	723.376709	1.102743687	0.000796958	84.01191021
																			FQQDDAHIFCAMEQIEDEIK	2	386642862	2xTMT6plex [N-Term; K20]; 1xCarbamidomethyl [C10	100			3.30985713	21.6	0	2925.4163	3	975.8094482	-0.857602194	-0.000836281	104.503929
																			FNLTYVSHDGDDK	6	386642862	2xTMT6plex [N-Term; K13]	0.048	1.313	3.055	3.182437658	27.26	0	1969.006727	3	657.0058594	-1.880143399	-0.001234005	49.86110843
																			GFQEVVTPNIFNSR	6	386642862	1xTMT6plex [N-Term]	100	100	1.645	2.82410264	35.25	0	1836.980563	2	918.9933472	-0.6235363	-0.000572712	74.11726002
																			MIAILTENYGGK	6	386642862	2xTMT6plex [N-Term; K12]	1.006	1.77	1.277	2.981831551	38.82	0	1768.007913	2	884.5072632	-0.375152627	-0.000331636	73.06770811
																			AEHDSILAEK	6	386642862	2xTMT6plex [N-Term; K10]	0.974	0.97	0.865	4.107958317	37.57	0	1570.884093	3	524.2990112	-1.026370183	-0.000537436	38.13844985
																			QVMVVPVGPTCDEYAQK	4	386642862	2xTMT6plex [N-Term; K17]; 1xCarbamidomethyl [C11	100			1.699069619	15.07	0	2379.24526	2	1190.123535	-2.297303095	-0.002732924	61.01270597
																			GAYIYNALIEFIR	2	386642862	1xTMT6plex [N-Term]	1.053			2.116819859	28.28	0	1771.994422	2	886.5006104	-0.269914436	-0.000239143	111.0832428
																			WELNSGDGAFYGPK	2	386642862	2xTMT6plex [N-Term; K14]		100		1.973355532	13.73	0	1999.032548	2	1000.018799	-1.113895695	-0.001113357	71.75412908
																			QLENSLNEFGEK	4	386642862	2xTMT6plex [N-Term; K12]		100	100	2.331588268	29.45	0	1866.000913	2	933.5053711	1.367795418	0.001276154	64.38445743
																			ETLLAMFK	4	386642862	2xTMT6plex [N-Term; K8]	0.564	0.236		2.469508648	27.35	0	1410.84308	2	705.9248657	-0.442929973	-0.000312452	107.3866246
																			LEMYNILK	12	386642862	2xTMT6plex [N-Term; K8]	4.472	0.929	100	2.315150261	27.24	0	1481.880193	2	741.4431763	-0.754150668	-0.00055878	71.35069118
																			IYGISFPDPK	10	386642862	2xTMT6plex [N-Term; K10]	0.954	2.589	100	2.254098415	17.45	0	1594.924501	2	797.9660034	0.143671965	0.000114573	87.53044549
																			AILGSVER	4	386642862	1xTMT6plex [N-Term]	1.043	1.139		2.915538073	51.9	0	1073.651625	2	537.329895	0.827669834	0.000444315	41.72200668
																			TISETIER	6	386642862	1xTMT6plex [N-Term]	1.276	1.187	1.107	3.371968985	53.85	0	1177.662583	2	589.335144	0.363564185	0.000214078	35.36037332
																			KETLLAMFK	4	386642862	3xTMT6plex [N-Term; K1; K9]	2.385	1.024		2.796672583	13.29	1	1768.100975	3	590.0388184	0.524369444	0.000309046	87.88872307
																			QVDAESWK	2	386642862	2xTMT6plex [N-Term; K8]	1.127			3.614289522	21.33	0	1420.783651	2	710.8944092	-1.484244715	-0.001054395	53.73997766
																			VNTPTTTVYR	2	386642862	1xTMT6plex [N-Term]	1.653			3.621124506	37.22	0	1380.768445	2	690.8886719	1.174585069	0.000810915	37.16391963
																			ISGTVNIR	2	386642862	1xTMT6plex [N-Term]		1.418		2.655060291	31.77	0	1088.662524	2	544.8342285	-1.233999261	-0.000671704	53.82123249
																			ADMETLQR	2	386642862	1xTMT6plex [N-Term]	1.136			2.655190706	36.82	0	1192.619338	2	596.8138428	0.897693707	0.000535303	36.23564528
																			GCLDFLR	6	386642862	1xTMT6plex [N-Term]; 1xCarbamidomethyl [C2	1.206	100	0.941	2.269373894	28.16	0	1109.597481	2	555.3016357	-1.339418708	-0.000743108	54.69837241
312433975	EIF3C	eukaryotic translation initiation factor 3 subunit C isoform a	8663	NP_001186071.1	23.32968237	1	18	18	92	913	105.278	5.68	1.06	1.08	1.06	1.07	Significant	0.000039																
																			RLDEEEEDNEGGEWER	10	312433975	1xTMT6plex [N-Term]	1.517	1.376	1.413	7.292843819	43.53	1	2220.984264	3	741.0004272	1.10973694	0.000821569	26.7634422
																			GTEITHAVVIK	8	312433975	2xTMT6plex [N-Term; K11]	1.107	1.129	1.007	4.474756718	61.16	0	1625.999063	3	542.6711426	-0.115901435	-6.28E-05	48.82599286
																			SEQDQAENEGEDSAVLMER	6	312433975	1xTMT6plex [N-Term]		100		3.043958902	31.72	0	2366.061528	3	794.6903687	0.046168944	3.67E-05	49.03513138
																			SEQDQAENEGEDSAVLMER	2	312433975	1xTMT6plex [N-Term]; 1xOxidation [M17]		5.249		4.119888306	41.53	0	2382.056443	3	794.6903687	0.046168944	3.67E-05	49.03513138
																			DAHNALLDIQSSGR	4	312433975	1xTMT6plex [N-Term]	1.198	0.936		4.428974152	41.08	0	1725.908126	3	575.9746094	0.665391734	0.000382802	50.15893801
																			MIINEELMASLDQPTQTVVMHR	4	312433975	1xTMT6plex [N-Term]				2.294092894	20.2	0	2785.424807	3	929.1474609	1.085063396	0.001007454	103.0078975
																			TCHSFIINEK	8	312433975	2xTMT6plex [N-Term; K10]; 1xCarbamidomethyl [C2	0.922	1.092	0.962	4.34997654	43.65	0	1706.929997	3	569.6477051	-0.84093409	-0.000478472	49.25480601
																			DLMLMSHLQDNIQHADPPVQILYNR	4	312433975	1xTMT6plex [N-Term]		100		2.547785044	17.13	0	3190.633889	4	798.4128418	-1.363767405	-0.001087821	77.6919349
																			GCILTLVER	6	312433975	1xTMT6plex [N-Term]; 1xCarbamidomethyl [C2	1.873	100	100	3.095861912	42.02	0	1289.744873	2	645.3761597	0.1312166	8.46E-05	75.23130602
																			DFESHITSYK	12	312433975	2xTMT6plex [N-Term; K10]	1.146	1.063	1.129	3.753613234	41.43	0	1684.894657	3	562.3027954	-0.489306277	-0.00027481	45.10522487
																			KLNEILQAR	4	312433975	2xTMT6plex [N-Term; K1]	1.195	1.152		3.092442513	16.46	1	1542.973183	3	514.9954834	-0.833225556	-0.000428548	50.00923653
																			ELLGQGLLLR	10	312433975	1xTMT6plex [N-Term]	3.737	100	100	2.425333738	41.58	0	1340.846302	2	670.9263306	-0.684065867	-0.000458614	75.61198541
																			SIVDKEGVPR	3	312433975	2xTMT6plex [N-Term; K5]	0.605			3.40504837	11.22	1	1557.936463	3	519.9832153	-0.879338753	-0.000456651	40.73282827
																			MDEEFTK	2	312433975	2xTMT6plex [N-Term; K7]	1.446			3.047262192	33.72	0	1357.707374	2	679.3572998	-0.037630071	-2.55E-05	54.59574045
																			AKELLGQGLLLR	2	312433975	2xTMT6plex [N-Term; K2]				1.727305412	5.64	1	1769.14131	3	590.3847656	-0.88564152	-0.000522275	70.28571026
																			FEELTNLIR	2	312433975	1xTMT6plex [N-Term]		0.921		1.865262389	2.88	0	1363.778282	2	682.3920898	-1.010913957	-0.000689331	84.03815223
																			DEAQVCAIIER	2	312433975	1xTMT6plex [N-Term]; 1xCarbamidomethyl [C6	0.94			2.011315823	6.16	0	1532.794008	2	766.9005127	-0.169350463	-0.00012979	51.72579737
																			VWDLFPEADK	2	312433975	2xTMT6plex [N-Term; K10]				1.591597199	12.52	0	1677.925229	2	839.4647217	-1.825206852	-0.00153128	86.78800047
																			APTTDEDKK	1	312433975	3xTMT6plex [N-Term; K8; K9]	0.81			2.161192417	5.66	1	1691.978277	3	564.6647949	0.919045857	0.000518335	32.24756194
13994151	PDLIM1	PDZ and LIM domain protein 1	9124	NP_066272.1	55.01519757	1	12	12	123	329	36.049	7.02	1.1	1.11	0.95	1.06	Significant	0.0024																
																			HPECYVCTDCGTNLK	20	13994151	2xTMT6plex [N-Term; K15]; 3xCarbamidomethyl [C4; C7; C10	1.219	1.265	1.15	4.754857063	52.48	0	2312.087379	3	771.3651733	-2.773502648	-0.002137527	52.59705348
																			GHFFVEDQIYCEK	17	13994151	2xTMT6plex [N-Term; K13]; 1xCarbamidomethyl [C11	1.375	1.099	0.782	3.822696686	37.03	0	2130.073033	3	710.6956787	-0.258088422	-0.000183249	54.48908334
																			MNLASEPQEVLHIGSAHNR	2	13994151	1xTMT6plex [N-Term]	0.852			4.687756062	27.15	0	2332.202928	4	583.8063354	0.250358521	0.000145972	56.97063988
																			GCTDNLTLTVAR	10	13994151	1xTMT6plex [N-Term]; 1xCarbamidomethyl [C2	1.085	1.114	0.9	4.105473995	73.04	0	1549.820557	2	775.4141846	0.345253276	0.00026754	59.23704397
																			VITNQYNNPAGLYSSENISNFNNALESK	11	13994151	2xTMT6plex [N-Term; K28]	1.394			4.532420635	53.05	0	3559.806911	3	1187.27417	0.293815269	0.000348642	92.79649373
																			AALANLCIGDVITAIDGENTSNMTHLEAQNR	22	13994151	1xTMT6plex [N-Term]; 1xCarbamidomethyl [C7	1.029		1.865	3.93714118	45.16	0	3541.757645	4	886.1952515	0.43222872	0.000382712	100.8688784
																			VAASIGNAQK	8	13994151	2xTMT6plex [N-Term; K10]	1.084	1.05	0.97	4.655112267	68.34	0	1416.857484	2	708.9329834	0.851184321	0.000603003	35.63032765
																			SAMPFTASPASSTTAR	2	13994151	1xTMT6plex [N-Term]			0.672	4.866990566	64.92	0	1811.915914	2	906.4611816	-0.456582308	-0.000413644	50.00804558
																			DFEQPLAISR	10	13994151	1xTMT6plex [N-Term]	0.933	0.783	100	4.012063026	54.02	0	1404.768445	2	702.8873901	-0.670321551	-0.000470823	61.30719243
																			VWSPLVTEEGKR	5	13994151	2xTMT6plex [N-Term; K11]	0.985	0.957		3.821924448	30.53	1	1859.079104	3	620.3644409	-0.179963345	-0.000111522	67.49871614
																			AALANLCIGDVITAIDGENTSNMTHLEAQNR	2	13994151	1xTMT6plex [N-Term]; 1xCarbamidomethyl [C7]; 1xOxidation [M23]				1.104881763	1.37	0	3557.75256	4	886.1952515	0.43222872	0.000382712	100.8688784
																			VWSPLVTEEGK	6	13994151	2xTMT6plex [N-Term; K11]	0.831	1.728	0.826	3.268056393	35.68	0	1702.977993	2	851.9923706	-0.310349907	-0.00026426	61.4604488
																			VTPPEGYEVVTVFPK	8	13994151	2xTMT6plex [N-Term; K15]	0.876	0.467	2.551	2.318262815	11.11	0	2120.204364	2	1060.606689	0.819848444	0.000869123	67.39336405
4501867	ACO2	aconitate hydratase, mitochondrial precurso	50	NP_001089.1	31.53846154	1	18	18	83	780	85.372	7.61	0.65	0.71	0.64	0.67	Significant	0.0011																
																			VAMSHFEPNEYIHYDLLEK	4	4501867	2xTMT6plex [N-Term; K19]	0.714		100	2.518389225	13.96	0	2793.432208	4	699.1123657	-1.637834404	-0.001143795	70.66269525
																			DSSGQHVDVSPTSQR	6	4501867	1xTMT6plex [N-Term]	0.69	0.767	0.4	4.172008038	34.51	0	1828.898684	3	610.3048096	0.651518287	0.000397187	28.84907203
																			IVYGHLDDPASQEIER	8	4501867	1xTMT6plex [N-Term]	0.644	0.664	1.086	4.265097141	47.77	0	2071.065749	3	691.0269165	0.215880022	0.000149034	59.49870352
																			DINQEVYNFLATAGAK	4	4501867	2xTMT6plex [N-Term; K16]		0.677		3.460494757	34.46	0	2212.201404	2	1106.603149	-1.076589081	-0.001190816	102.0143694
																			GEFDPGQDTYQHPPK	4	4501867	2xTMT6plex [N-Term; K15]	0.572	0.598		3.369999886	35.08	0	2174.091853	3	725.3687744	-0.038373637	-2.78E-05	40.5861753
																			VAMQDATAQMAMLQFISSGLSK	2	4501867	2xTMT6plex [N-Term; K22]		1.054		4.915107727	49.58	0	2786.465499	3	929.4920654	-1.383789227	-0.001285294	110.5274891
																			VAVPSTIHCDHLIEAQVGGEK	4	4501867	2xTMT6plex [N-Term; K21]; 1xCarbamidomethyl [C9		0.749	0.616	5.84920454	38.17	0	2718.464906	4	680.3725586	1.28708853	0.000874726	53.73478097
																			DLGGIVLANACGPCIGQWDR	7	4501867	1xTMT6plex [N-Term]; 2xCarbamidomethyl [C11; C14	0.01	1.929	1.34	2.844485998	30.95	0	2401.1954	3	801.0686646	-1.649194253	-0.001320013	83.27765648
																			SQFTITPGSEQIR	6	4501867	1xTMT6plex [N-Term]	0.01	0.699	0.713	4.391590595	38.29	0	1692.911815	2	846.960083	0.634702656	0.000537248	50.01235951
																			LEAPDADELPK	6	4501867	2xTMT6plex [N-Term; K11]	0.765	0.567	0.803	2.487000465	43.04	0	1655.925623	2	828.4663696	-0.096938042	-8.03E-05	57.74906046
																			DLEDLQILIK	6	4501867	2xTMT6plex [N-Term; K10]	0.661		100	2.749699831	31.59	0	1658.014044	2	829.5102539	-0.490386377	-0.000406534	91.13215799
																			FNPETDYLTGTDGK	6	4501867	2xTMT6plex [N-Term; K14]	0.468	0.733	0.73	2.441862822	24.1	0	2016.032607	2	1008.519348	-0.589063358	-0.000593785	57.30060789
																			LQLLEPFDK	6	4501867	2xTMT6plex [N-Term; K9]	0.952	100	100	3.087852001	31.09	0	1560.940151	2	780.9733276	-0.494879039	-0.000386238	84.97509203
																			LNRPLTLSEK	4	4501867	2xTMT6plex [N-Term; K10]	0.833	0.875		2.043349266	22.47	0	1629.009962	3	543.6748657	0.050305382	2.73E-05	52.95549995
																			VAGILTVK	2	4501867	2xTMT6plex [N-Term; K8]			100	2.377280235	13.68	0	1258.84988	2	629.9285278	-0.079966594	-5.03E-05	64.52093895
																			LTIQGLK	4	4501867	2xTMT6plex [N-Term; K7]	0.692	0.844		2.833087444	38.42	0	1230.81858	2	615.9117432	-1.925451821	-0.001184941	59.37688585
																			DFTPGKPLK	2	4501867	3xTMT6plex [N-Term; K6; K9]			0.618	2.92441678	2.19	0	1690.050654	3	564.0223389	1.070773564	0.000603221	51.13378566
																			EDIANLADEFK	2	4501867	2xTMT6plex [N-Term; K11]				1.463280559	5.52	0	1722.931437	2	861.9689331	-0.491719545	-0.0004236	80.729264
118498356	KTN1	kinectin isoform a	3895	NP_001072989.1	19.52837141	1	22	22	84	1357	156.179	5.64	1.04	1	1.24	1.09	Significant	0.0046																
																			ECMAGTSGSEEVK	6	118498356	2xTMT6plex [N-Term; K13]; 1xCarbamidomethyl [C2	1.389	1.372	1.374	4.483464241	66.64	0	1842.89777	2	921.9512329	-1.400630968	-0.00129061	34.90919009
																			SVLAETEGILQK	3	118498356	2xTMT6plex [N-Term; K12]	0.828			3.804648638	46.12	0	1746.041322	2	873.5234985	-0.91700561	-0.000800565	92.07040249
																			EVIDLLKPDQVEGIQK	6	118498356	3xTMT6plex [N-Term; K7; K16]	1.634		100	3.030159235	18.33	0	2511.500101	3	837.8381958	-0.026608662	-2.23E-05	76.27044126
																			AQQSLELIQSK	6	118498356	2xTMT6plex [N-Term; K11]	0.863	100	100	3.743688345	58.61	0	1703.010356	2	852.0083008	-0.60533249	-0.000515444	59.14070825
																			TAEHEAAQQDLQSK	2	118498356	2xTMT6plex [N-Term; K14]			1.256	5.39715147	43.34	0	2014.060553	3	672.0248413	-0.289278402	-0.000194208	33.68379108
																			AHVQEVAQHNLK	4	118498356	2xTMT6plex [N-Term; K12]	0.927	0.759		4.494565964	39.21	0	1832.054286	4	458.7692261	0.430171137	0.000197024	31.88983585
																			GELTTLIHQLQEK	4	118498356	2xTMT6plex [N-Term; K13]	0.758	1.964		3.771281719	26.36	0	1968.152997	3	656.7220459	-0.71780735	-0.000470918	82.41590619
																			LLEEQLQHEISNK	6	118498356	2xTMT6plex [N-Term; K13]	0.903	0.275	1.451	2.681203604	21.16	0	2039.153725	3	680.3886108	-1.19959591	-0.000815387	60.9771619
																			AQQLSITSK	6	118498356	2xTMT6plex [N-Term; K9]	1.327	1.144	1.183	4.395081997	65.37	0	1433.8728	2	717.4403687	0.460867022	0.000330412	41.25345525
																			AQLNETLTK	8	118498356	2xTMT6plex [N-Term; K9]	1.021	0.931		4.033975124	53.48	0	1475.883365	2	738.4454346	0.154484176	0.000114	53.76969872
																			VQELQNLLK	4	118498356	2xTMT6plex [N-Term; K9]	1.359			2.688797951	38.74	0	1542.961949	2	771.986084	1.906825215	0.001471079	78.48096402
																			LVNELTEK	4	118498356	2xTMT6plex [N-Term; K8]	1.34	1.147		3.813205242	48.91	0	1403.851002	2	702.4291382	-0.001462272	-1.03E-06	65.65663741
																			MQQSVYVK	2	118498356	2xTMT6plex [N-Term; K8]		1.057		3.211750746	22.37	0	1440.828492	2	720.9174805	-0.560755499	-0.000403976	42.10728521
																			SVEQEENK	2	118498356	2xTMT6plex [N-Term; K8]		1.195		3.62608099	36.73	0	1420.768394	2	710.8873291	-0.712816304	-0.000506373	29.27760109
																			VQLQEAER	4	118498356	1xTMT6plex [N-Term]	1.189	0.655		3.042603016	26.47	0	1201.673817	2	601.3408813	0.557027459	0.000334683	41.11156385
																			LTDTLVSK	4	118498356	2xTMT6plex [N-Term; K8]	100	100		2.460785389	28	0	1334.829538	2	667.920166	2.635019042	0.001758651	48.70404973
																			LQALANEQAAAAHELEK	2	118498356	2xTMT6plex [N-Term; K17]		0.01		1.614341855		0	2265.260316	3	755.7565918	-2.248531588	-0.001697836	60.55438667
																			EISGLWNELDSLK	1	118498356	2xTMT6plex [N-Term; K13]	100			1.422783375		0	1962.094814	2	981.5489502	-2.135395979	-0.002094925	100.9829282
																			RLEAMLK	2	118498356	2xTMT6plex [N-Term; K7]	0.768			2.936047554	13.17	1	1318.828098	3	440.2806091	-0.624999894	-0.000274756	48.28923102
																			LSDALAVEDDQVAPVPLNVVETSSSVR	2	118498356	1xTMT6plex [N-Term]				1.120064735	2.54	0	3039.60474	3	1013.872559	-0.53201284	-0.000539036	80.74968901
																			SVEELLEAELLK	3	118498356	2xTMT6plex [N-Term; K12]				1.713206053	13.16	0	1831.082852	2	916.0441895	-0.955556843	-0.000874852	99.07210351
																			VSVPSNLSYGEWLHGFEK	3	118498356	2xTMT6plex [N-Term; K18]				1.584433675		0	2507.333481	3	836.4465942	-3.290780911	-0.002750362	79.40192469
4503131	CTNNB1	catenin beta-1	1499	NP_001895.1	29.57746479	1	11	16	82	781	85.442	5.86	0.86	0.82		0.84	Non-Significan	#N/A																
																			MEEIVEGCTGALHILAR	3	4503131	1xTMT6plex [N-Term]; 1xCarbamidomethyl [C8]; 1xOxidation [M1]	2.578			3.878248692	35.02	0	2144.104126	3	710.0407715	-0.681260561	-0.000483266	81.67058513
																			MEEIVEGCTGALHILAR	12	4503131	1xTMT6plex [N-Term]; 1xCarbamidomethyl [C8	1.261	1.094	100	4.9167943	50.73	0	2128.109211	3	710.0407715	-0.681260561	-0.000483266	81.67058513
																			LLNDEDQVVVNK	4	4503131	2xTMT6plex [N-Term; K12]	1.039	100		3.562664986	57.85	0	1844.052949	2	922.5302734	0.17429814	0.000160707	69.04740313

Bhat et al,2017. Cigarette smoke and chewing tobacco alter expression of different sets of miRNAs in oral keratinocytes

Supplementary Table S2(A). List of all proteins and peptides identified in OKF6/TERT1 cells chronically treated with chewing tobacco using Sequest and Mascot search algorithms

Accession	Gene_Symbol	Description	Gene_ID	Refseq_Accession	Coverage	Proteins	#Unique_Peptides	#Peptides	#PSMS	#AAs	MW [kDa]	calc. pI	OKF6-STE-6M/OKF6P_R1	OKF6-STE-6M/OKF6P_R2	OKF6-STE-6M/OKF6P_R3	Median fold change	Status	P-value (6M)	Sequence	#PSMSs	Protein Accessions	Modifications	OKF6-STE-6M/OKF6P_R1	OKF6-STE-6M/OKF6P_R2	OKF6-STE-6M/OKF6P_R3	Xcorr	Ionscore	# Missed Cleavages	Theo. MH+ [Da]	Charge	m_z	DeltaM [ppm]	Deltam/z [Da]	RT [min]
																			HQEAEMAQNAVR	2	4503131	1xTMT6plex [N-Term]; 1xOxidation [M6]	1.016			4.250850677	38.84	0	1628.801219	3	543.6052246	-0.060450516	-3.28E-05	27.03765017
																			HAVVNLINYQDDAELATR	2	4503131	1xTMT6plex [N-Term]	1.143			4.503149033	37.96	0	2271.193075	3	757.7364502	0.758410175	0.000574165	66.22786908
																			TMQNTNDVETAR	10	4503131	1xTMT6plex [N-Term]	1.119	1.046		4.321681976	48.35	0	1608.7849	2	804.8953247	-0.949192112	-0.000763523	31.74871489
																			AAVMVHQLSK	8	4503131	2xTMT6plex [N-Term; K10]	0.813	0.809		3.85801053	51.01	0	1541.92379	2	771.4658203	0.372524897	0.000287203	42.16418356
																			TSMGGTQQQFVEGVR	2	4503131	1xTMT6plex [N-Term]	0.715			2.584107161	25.4	0	1853.937712	2	927.4719849	-0.549588819	-0.000509452	51.26946429
																			AAVSHWQQQSYLDSGIHSGATTTAPSLSGK	2	4503131	2xTMT6plex [N-Term; K30]				2.014776468	5.23	0	3543.823229	4	886.7115479	0.319467472	0.000283034	60.21298962
																			LAEPSQMLK	2	4503131	2xTMT6plex [N-Term; K9]	0.921			3.669286489	48.77	0	1474.870357	2	737.9385986	-0.29589338	-0.000218202	63.79291736
																			VAAGVLCELAQDKEAAEAIEAEGATAPLTELLHSR	4	4503131	2xTMT6plex [N-Term; K13]; 1xCarbamidomethyl [C7				2.43358469	8.9	1	4092.168729	4	1023.79718	-0.449281786	-0.000459634	102.9509681
																			HQEAEMAQNAVR	2	4503131	1xTMT6plex [N-Term]	0.835			2.99062562	14.67	0	1612.806304	3	543.6052246	-0.060450516	-3.28E-05	27.03765017
																			TMQNTNDVETAR	2	4503131	1xTMT6plex [N-Term]; 1xOxidation [M2]		1.505		2.128145218	31.8	0	1624.779815	2	804.8953247	-0.949192112	-0.000763523	31.74871489
																			VAAGVLCELAQDK	2	4503131	2xTMT6plex [N-Term; K13]; 1xCarbamidomethyl [C7	100			1.762995839	11.23	0	1832.035191	2	916.522522	1.406455141	0.001288338	71.21631703
																			AAMFPETLDEGMQIPSTQFDAAHPTNVQR	5	4503131	1xTMT6plex [N-Term]				1.762367368	6.71	0	3431.65614	3	1144.557861	0.84237297	0.000963578	79.70992446
																			EGLLAIFK	4	4503131	2xTMT6plex [N-Term; K8]	1.431	1.097		2.944336414	21.76	0	1348.860444	2	674.9327393	-1.662495467	-0.001121237	90.96548859
																			LVQNCLWTLR	6	4503131	1xTMT6plex [N-Term]; 1xCarbamidomethyl [C5		0.698	100	2.025776148	20.98	0	1531.861634	2	766.4334717	-1.284555067	-0.00098388	65.39937786
																			ALPELTK	12	4503131	2xTMT6plex [N-Term; K7]	0.61	0.728	0.718	2.832141638	35.55	0	1229.786945	2	615.3967285	-0.621805877	-0.000382344	50.45003139
																			MVALLNK	4	4503131	2xTMT6plex [N-Term; K7]	100	1.833		1.832590222	9.35	0	1246.795736	2	623.9015503	0.070802245	4.41E-05	63.42811285
164698494	Sep-09	septin-9 isoform a	10801	NP_001106963.1	38.05460751	1	18	18	90	586	65.361	8.97	0.86	0.98	1.01	0.95	Non-Significant	#N/A																
																			VPEVPTAPATDAAPK	4	164698494	2xTMT6plex [N-Term; K15]	0.962		0.932	3.49313879	58.56	0	1922.099899	2	961.5446777	-9.271199306	-0.008910086	47.79768689
																			SQEATEAAPSCVGDMADTPR	4	164698494	1xTMT6plex [N-Term]; 1xCarbamidomethyl [C11		100		3.363917351	44.66	0	2322.05394	2	1161.530273	-0.288574261	-0.000335043	46.20810842
																			LEPKPQPPVAEATPR	10	164698494	2xTMT6plex [N-Term; K4]	0.826	0.93	0.9	5.407478809	29.37	0	2088.221745	3	696.7455444	0.160237188	0.000111537	48.12630512
																			SFEVEEVETPNSTPPR	2	164698494	1xTMT6plex [N-Term]		1.142		3.491613388	37.3	0	2047.01813	2	1024.01123	-1.43912868	-0.001472961	51.89202629
																			LGDSSGPALK	2	164698494	2xTMT6plex [N-Term; K10]	1.244			3.656789303	43.55	0	1402.830601	2	701.9178467	-1.556831327	-0.001091985	46.61739301
																			APVDFGYVGIDSILEQMR	8	164698494	1xTMT6plex [N-Term]		0.775	0.649	3.602310896	49.59	0	2239.16302	3	747.0591431	-0.064484265	-4.81E-05	104.3901623
																			QVENAGAIGPSR	6	164698494	1xTMT6plex [N-Term]	0.879	1.316	1.392	4.33972168	58.54	0	1427.780407	2	714.3950806	1.735268813	0.001238791	33.42243607
																			SITHDIEEK	4	164698494	2xTMT6plex [N-Term; K9]	0.758			4.139952183	52.23	0	1529.857544	2	765.4320068	-0.527243944	-0.000403304	43.55497096
																			LTVIDTPGFGDHINNENCWQPIMK	4	164698494	2xTMT6plex [N-Term; K24]; 1xCarbamidomethyl [C18	100	100		2.338895559	16.15	0	3257.64876	3	1086.556641	2.028722397	0.002202955	93.77767842
																			ADTLTLEER	8	164698494	1xTMT6plex [N-Term]	1.345	0.773	1.129	3.294257641	42.86	0	1276.694612	2	638.8502197	-1.134826498	-0.000724413	34.06164433
																			ATVASSTQK	6	164698494	2xTMT6plex [N-Term; K9]	0.821	0.996	1.29	3.752277851	56.6	0	1350.799301	2	675.9031982	-0.133791619	-9.04E-05	34.97482217
																			DITSSIHFEAYR	8	164698494	1xTMT6plex [N-Term]	100	100	100	3.464738607	24.06	0	1667.859051	3	556.6243286	-0.370834787	-0.000206167	51.43184981
																			EFDEDSEDR	4	164698494	1xTMT6plex [N-Term]		1.921	1.22	4.014975071	46.83	0	1370.590934	2	685.7988892	-0.315673828	-0.00021633	34.19074091
																			FINDQYEK	6	164698494	2xTMT6plex [N-Term; K8]	1.072	1.362	1.335	3.681815863	40.5	0	1514.825515	2	757.9173584	1.270685538	0.000962433	59.47338122
																			VVNIVPVIAK	6	164698494	2xTMT6plex [N-Term; K10]	1.001	100	100	2.757309437	29.95	0	1510.013257	2	755.5115356	1.680865417	0.001269065	74.2892098
																			YLQEEVNINR	2	164698494	1xTMT6plex [N-Term]				1.408120394	12.17	0	1506.811373	2	753.9088135	-0.678483647	-0.000511173	53.88769429
																			STLINTLFK	4	164698494	2xTMT6plex [N-Term; K9]				1.719846845	14.72	0	1494.929586	2	747.9682617	-0.227203009	-0.000169826	75.8354406
																			RVELSGPK	2	164698494	2xTMT6plex [N-Term; K8]		0.864		4.011602879	25.36	1	1343.841106	3	448.6187134	0.357934221	0.000160336	35.40738141
156151394	HNRNPR	heterogeneous nuclear ribonucleoprotein R isoform 1	10236	NP_001095868.1	32.2327044	1	12	18	103	636	71.17	8.13	1.28	1.45	1.38	1.37	Significant	0.0013																
																			TGYTLDVTTGQR	6	156151394	1xTMT6plex [N-Term]	2.364	2.396	2.876	4.746033669	59.21	0	1540.816852	2	770.9112549	-1.050607911	-0.000809397	41.79359486
																			STAYEDYYYHPPPR	7	156151394	1xTMT6plex [N-Term]	0.836	1.302	1.302	4.009177685	43.3	0	1987.938758	3	663.3173218	-0.676966513	-0.000448589	43.09920792
																			EEEEPMDTSSVTHTEHYK	3	156151394	2xTMT6plex [N-Term; K18]	0.31	1.214		5.448690891	28.52	0	2607.228483	4	652.5631714	0.910214387	0.000593284	37.03158113
																			LCDSYEIRPGK	8	156151394	2xTMT6plex [N-Term; K11]; 1xCarbamidomethyl [C2	1.645	1.458		3.470659733	20.2	0	1795.977676	3	599.3307495	0.010843763	6.49E-06	53.92224675
																			GFCFLEYEDHK	12	156151394	2xTMT6plex [N-Term; K11]; 1xCarbamidomethyl [C3	1.31	1.299	1.309	3.861279726	35.59	0	1902.946041	3	634.9869995	0.212284084	0.000134655	93.87060383
																			EFNEEGALSVLQQFK	5	156151394	2xTMT6plex [N-Term; K15]	1.6			3.417670727	36.46	0	2197.190505	3	733.0689697	0.842493941	0.00061704	98.42653767
																			DLYEDELVPLFEK	6	156151394	2xTMT6plex [N-Term; K13]		100	0.913	2.935118437	29.45	0	2068.125445	2	1034.564819	-1.490696869	-0.001541474	95.06060005
																			EIEGEEIEIVLAKPPDKK	2	156151394	4xTMT6plex [N-Term; K13; K17; K18]			0.814	3.042105675	6.87	1	2953.763141	4	739.1956787	-0.763726183	-0.000563967	76.3436714
																			SAFLCGVMK	6	156151394	2xTMT6plex [N-Term; K9]; 1xCarbamidomethyl [C5	1.383	1.467		3.858357191	51.79	0	1470.821299	2	735.9140625	-0.306196273	-0.00022518	75.28522302
																			DYAFVHFEDR	4	156151394	1xTMT6plex [N-Term]	2.166	1.814		3.515748501	44.96	0	1527.742959	3	509.9185486	-1.22161129	-0.000622103	54.54929034
																			EEEEPMDTSSVTHTEHYK	2	156151394	2xTMT6plex [N-Term; K18]; 1xOxidation [M6	0.887			3.798317432	18.04	0	2623.223398	4	652.5631714	0.910214387	0.000593284	37.03158113
																			GYAFITFCGK	2	156151394	2xTMT6plex [N-Term; K10]; 1xCarbamidomethyl [C8	0.861			2.780025005	32.99	0	1621.881256	2	811.444519	0.311413635	0.000252538	81.75433402
																			EAAQEAVK	10	156151394	2xTMT6plex [N-Term; K8]	1.542	1.529	1.762	3.661378145	46.85	0	1303.762187	2	652.3850708	0.52014207	0.000339071	20.53000102
																			ENILEEFSK	6	156151394	2xTMT6plex [N-Term; K9]	100	1.662	1.592	2.738269091	27.46	0	1566.877945	2	783.9408569	-2.238529698	-0.001753751	71.80395615
																			LFVGSIPK	8	156151394	2xTMT6plex [N-Term; K8]	6.929	100	3.451	2.833257198	36.61	0	1318.84988	2	659.9281616	-0.631677513	-0.000416544	66.64259136
																			NLATTVTEEILEK	2	156151394	2xTMT6plex [N-Term; K13]			0.01	1.422051907	9.32	0	1919.110129	2	960.0588379	0.140612696	0.000134926	94.05996299
																			AGPIWDLR	2	156151394	1xTMT6plex [N-Term]	1.779			2.573340654	22.75	0	1156.667609	2	578.8375854	0.246448016	0.000142529	72.14733946
																			SFSEFGK	6	156151394	2xTMT6plex [N-Term; K7]	1.796	1.359	1.514	3.064883947	32.74	0	1259.703609	2	630.3551025	-0.540557215	-0.000340471	49.50663844
																			LMMDPLSGQNR	6	156151394	1xTMT6plex [N-Term]		100		1.72441721	9.92	0	1490.765685	2	745.8859253	-0.745196964	-0.000555457	61.94696332
7661920	EIF4A3	eukaryotic initiation factor 4A-III	9775	NP_055555.1	48.90510949	1	14	17	96	411	46.841	6.73	1.25	1.01	1.05	1.1	Significant	0.0045																
																			KLDYGQHVVAGTPGR	12	7661920	2xTMT6plex [N-Term; K1]	1.243	1.287	1.239	5.819386005	35.58	1	2056.170379	3	686.0612183	-0.621089019	-0.000425688	37.012257
																			GIYAYGFEKPSAIQQR	15	7661920	2xTMT6plex [N-Term; K9]	0.768	1.343	1.252	4.605382919	27.73	0	2286.264673	3	762.7591553	-0.769923845	-0.00058675	52.2933608
																			EANFTVSSMHGDMPQK	2	7661920	2xTMT6plex [N-Term; K16]; 1xOxidation [M9		1.222		5.337425709	51.07	0	2253.104409	3	751.7064209	0.133307321	0.000100118	40.32630519
																			EANFTVSSMHGDMPQK	4	7661920	2xTMT6plex [N-Term; K16]		0.921	0.743	4.127759933	36.63	0	2237.109494	3	751.7064209	0.133307321	0.000100118	40.32630519
																			DVIAQSQSGTGK	4	7661920	2xTMT6plex [N-Term; K12]	1.801	2.028		4.345722675	47.65	0	1648.92702	2	824.9674683	0.387993786	0.000319887	42.42621865
																			VEFETSEEVDVTPTFDTMGLR	2	7661920	1xTMT6plex [N-Term]			1.144	3.650702	24.69	0	2631.26973	3	877.7602539	-1.338164313	-0.00117369	86.40098898
																			RDELTLEGIK	6	7661920	2xTMT6plex [N-Term; K10]	0.779	0.946	1.587	4.958242416	27.87	1	1631.973242	3	544.6596069	-5.499188949	-0.00299151	48.49727575
																			ETQALILAPTR	2	7661920	1xTMT6plex [N-Term]	1.056			3.422571898	31.68	0	1441.857595	2	721.4325562	0.167183423	0.000120527	57.26011962
																			ELAVQIQK	6	7661920	2xTMT6plex [N-Term; K8]	1.544	1.404	1.121	3.746592522	41.38	0	1386.872072	2	693.9390869	-0.846813415	-0.000587211	53.64009061
																			MLVLDEADEMLNK	6	7661920	2xTMT6plex [N-Term; K13]	100	2.043	100	2.104495764	12.85	0	1979.059356	2	990.0325317	-0.79292894	-0.000784627	88.68907321
																			KGVAINFVK	2	7661920	3xTMT6plex [N-Term; K1; K9]	1.495			3.76457572	18.93	1	1663.087374	3	555.0333252	-1.173266115	-0.000650415	60.6611403
																			VEFETSEEVDVTPTFDTMGLR	4	7661920	1xTMT6plex [N-Term]; 1xOxidation [M18]				1.970533371	7.46	0	2647.264645	3	877.7602539	-1.338164313	-0.00117369	86.40098898
																			LDYGQHVVAGTPGR	5	7661920	1xTMT6plex [N-Term]	1.396	0.542	0.505	2.785997629	23.16	0	1698.912484	3	566.9758301	0.266843866	0.000151115	46.14176185
																			KVDWLTEK	5	7661920	3xTMT6plex [N-Term; K1; K8]	1.637	0.957		2.848537683	19.42	1	1706.045568	3	569.3540039	1.107929815	0.00063006	57.89528673
																			EQIYDVYR	6	7661920	1xTMT6plex [N-Term]	1.079	1.087	1.419	3.409768581	35.03	0	1314.689132	2	657.8484497	0.373011422	0.000245197	43.77821944
																			VDWLTEK	6	7661920	2xTMT6plex [N-Term; K7]	100	100	100	2.119421959	25.85	0	1348.787673	2	674.8964233	-1.559378362	-0.001051635	60.49206049
																			QFFVAVER	6	7661920	1xTMT6plex [N-Term]	100	100	100	1.947969913	30.66	0	1224.693824	2	612.8503418	-0.340465708	-0.000208483	53.5863637
																			YLPPATQVVLISATLPHEILEMTNK	1	7661920	2xTMT6plex [N-Term; K25]				1.125712872		0	3236.836878	3	1079.61499	-1.995876567	-0.002153442	108.468292
																			DIEQYYSTQIDEMPMNVADLI	2	7661920	1xTMT6plex [N-Term]				0.917041779		0	2717.28875	2	1359.147827	-0.137174647	-0.000186372	106.1065719
4758648	KIF5B	kinesin-1 heavy chain	3799	NP_004512.1	19.31464174	1	21	21	75	963	109.617	6.51	0.9	1.13	0.98	1	Significant	0.0045																
																			SAEIDSDDTGGSAAQK	10	4758648	2xTMT6plex [N-Term; K16]	2.858	1.322	1.272	4.937544346	68.82	0	2010.002764	2	1005.504944	-0.075872875	-7.63E-05	32.905346
																			QISSLRDEVEAK	2	4758648	2xTMT6plex [N-Term; K12]			1.339	2.949337244	26.77	1	1833.048198	3	611.6860352	-2.534353028	-0.00154853	44.44226212
																			LITDLQDQNQK	4	4758648	2xTMT6plex [N-Term; K11]	1.125	0.977		3.65412426	47.44	0	1774.011084	2	887.508606	-0.647552852	-0.000574383	62.59577393
																			AAEMMASLLK	4	4758648	2xTMT6plex [N-Term; K10]	100		100	2.223248959	38.15	0	1522.873728	2	761.9397583	-0.977026795	-0.000743944	79.68587802
																			LYLVDLAGSEK	6	4758648	2xTMT6plex [N-Term; K11]	0.856	100	1.144	2.359849215	19.79	0	1665.982744	2	833.4943237	-0.824317699	-0.00068665	81.53576327
																			LQAENDASK	2	4758648	2xTMT6plex [N-Term; K9]			1.044	3.706438065	33.45	0	1433.800029	2	717.4029541	-0.974499126	-0.000698618	32.4671924
																			ILQDSLGGNCR	2	4758648	1xTMT6plex [N-Term]; 1xCarbamidomethyl [C10			1.205	4.303618431	23.52	0	1461.768128	2	731.3894043	2.328668744	0.001701987	53.27323789
																			LFVQDLATR	2	4758648	1xTMT6plex [N-Term]	1.448			2.668570518	28.65	0	1291.757152	2	646.3821411	-0.113646313	-7.34E-05	65.52697714
																			RDQDNMQAELNR	3	4758648	1xTMT6plex [N-Term]		0.612		2.998163462	16.83	1	1718.844146	3	573.6191406	-0.743170928	-0.000425798	39.40660749
																			GLEETVAK	10	4758648	2xTMT6plex [N-Term; K8]	0.785	0.875	0.749	3.602226734	50.03	0	1304.782588	2	652.8951416	0.320860448	0.000209327	47.99080947
																			ELQTLHNLR	2	4758648	1xTMT6plex [N-Term]	0.955			3.868814945	48.26	0	1352.784764	3	451.599823	0.112097759	5.05E-05	43.02763206
																			DQDNMQAELNR	2	4758648	1xTMT6plex [N-Term]		1.52		2.599779606	11.71	0	1562.743035	2	789.8728027	0.239931364	0.000189394	25.06738588
																			HVAVTNMNEHSSR	4	4758648	1xTMT6plex [N-Term]	100	1.218		1.638943553	2.84	0	1710.854317	3	570.9563599	0.122465041	6.98E-05	25.29688736
																			VQTANEVK	2	4758648	2xTMT6plex [N-Term; K8]	1.139			3.215534449	40.01	0	1346.804386	2	673.9055176	-0.465883358	-0.000313727	35.77333127
																			KLFVQDLATR	4	4758648	2xTMT6plex [N-Term; K1]		0.965	0.81	1.956878304	21.93	1	1649.015047	3	550.3430176	-0.332250228	-0.000182629	60.50469754
																			SLTEYLQNVEQK	3	4758648	2xTMT6plex [N-Term; K12]				1.881856441		0	1910.063513	2	955.534729	-0.697417745	-0.000666056	68.85342012
																			DQDNMQAELNR	2	4758648	1xTMT6plex [N-Term]; 1xOxidation [M5]		100		1.452404618	30.4	0	1578.73795	2	789.8728027	0.239931364	0.000189394	25.06738588
																			EYELLSDELNQK	4	4758648	2xTMT6plex [N-Term; K12]				1.598521352	3.66	0	1939.042444	2	970.0238037	-1.089645114	-0.001056434	63.5976914
																			TNLSVHEDK	2	4758648	2xTMT6plex [N-Term; K9]	1.091			2.035524368	2.7	0	1500.842228	3	500.9527283	0.935204532	0.000467865	33.72228675
																			TNLSVHEDKNR	2	4758648	2xTMT6plex [N-Term; K9]		1.093		3.963492632	10.48	1	1770.986266	4	443.5021362	0.253379647	0.000112183	26.87003684
																			ALESALK	2	4758648	2xTMT6plex [N-Term; K7]	0.575			2.720191956	33.79	0	1189.755645	2	595.3811035	-0.600601269	-0.000357284	48.66521645
																			LQAENDASKEEVK	1	4758648	3xTMT6plex [N-Term; K9; K13]			0.63	2.408231974		1	2148.211524	3	716.7411499	-1.222976853	-0.000875738	37.53437416
66392203	NME1-NME2	NME1-NME2 protein	654364	NP_001018146.1	77.90262172	1	6	14	158	267	30.118	8.92	1.22	1.23	1.2	1.22	Significant	0.000053																
																			EISLWFKPEELVDYK	16	66392203	3xTMT6plex [N-Term; K7; K15]	1.192	0.917	1.482	4.80484724	30.47	0	2583.467738	3	861.8283081	1.019097846	0.000877602	97.08717996
																			VMLGETNPADSKPGTIR	12	66392203	2xTMT6plex [N-Term; K12]; 1xOxidation [M2	1.171	1.485	1.552	4.420407295	27.08	0	2260.237138	3	748.7453613	-9.220058965	-0.006897349	46.94808783
																			VMLGETNPADSKPGTIR	14	66392203	2xTMT6plex [N-Term; K12]	1.458	1.394	1.401	6.463523865	41.68	0	2244.242223	3	748.7453613	-9.220058965	-0.006897349	46.94808783
																			NIIHGSDSVK	8	66392203	2xTMT6plex [N-Term; K10]		1.39	1.08	4.27665329	40.94	0	1527.889513	3	509.967865	-0.308137004	-0.000156933	37.36394306
																			DRPFFPGLVK	9	66392203	2xTMT6plex [N-Term; K10]	1.284	1.262	100	4.000727177	27.01	0	1633.983019	3	545.3327026	0.32781256	0.000178547	72.67506377
																			TFIAIKPDGVQR	14	66392203	2xTMT6plex [N-Term; K6]	0.706	1.686	1.255	3.967687607	38.06	0	1803.089275	2	902.0479126	-0.402805728	-0.000363147	48.0233598
																			GDFCIQVGR	31	66392203	1xTMT6plex [N-Term]; 1xCarbamidomethyl [C4	1.071	1.227	1.183	3.838706732	54.64	0	1280.661872	2	640.833374	-1.874595611	-0.001200362	47.03312522
																			YMNSGPVVAMVWEGLNVVK	11	66392203	2xTMT6plex [N-Term; K19]	100	100	100	2.678786278	24.93	0	2551.381693	3	851.1292114	-3.375458356	-0.002870694	105.3609273
																			GLVGEIIK	12	66392203	2xTMT6plex [N-Term; K8]	0.611	1.285	1.86	3.442770004	49.79	0	1286.844794	2	643.9254761	-0.869398988	-0.000559391	57.74624954
																			YMHSGPVVAMVWEGLNVVK	4	66392203	2xTMT6plex [N-Term; K19]				2.457158089	4.69	0	2574.397678	3	858.8040161	-0.070871007	-6.08E-05	92.98478275
																			DRPFFAGLVK	4	66392203	2xTMT6plex [N-Term; K10]	1.265	100		3.377313375	15.9	0	1607.967369	3	536.6607666	0.234827332	0.000125865	74.3320684
																			FMQASEDLLK	5	66392203	2xTMT6plex [N-Term; K10]	1.752			2.490768909	35.51	0	1639.91295	2	820.4603882	0.335192906	0.000274844	86.79043032
																			QHYIDLK	10	66392203	2xTMT6plex [N-Term; K7]	1.256	1.097	1.273	3.188317537	37.32	0	1374.814557	2	687.9100342	-1.283846331	-0.000882525	44.45700147
																			SCAHDWVYE	6	66392203	1xTMT6plex [N-Term]; 1xCarbamidomethyl [C2	0.986	0.941	0.966	2.263282537	34.85	0	1395.620067	2	698.3095703	-5.877570261	-0.004101427	42.88067174
																			EHYVDLK	2	66392203	2xTMT6plex [N-Term; K7]	1.837			2.726912737	26.86	0	1361.782922	3	454.598938	-0.485822773	-0.000220528	40.76722783
40068518	PGD	6-phosphogluconate dehydrogenase, decarboxylating isoform	5226	NP_002622.2	45.13457557	1	17	17	102	483	53.106	7.23	1.28	1.49	1.67	1.48	Significant	0.0057																
																			DVLGMAQDEMAQAFEDWNK	12	40068518	2xTMT6plex [N-Term; K19]	2.238	100	100	4.037967205	53.44	0	2656.278744	3	886.0964966	-1.433412783	-0.001269181	104.2821496
																			AVSTGVQAGIPMPCFTTALSFYDGYR	6	40068518	1xTMT6plex [N-Term]; 1xCarbamidomethyl [C14		100		2.644498825	37.69	0	3038.49533	3	1013.502014	-1.263977434	-0.001280197	100.1532228
																			LVPLLDTGDIIIDGGNSEYR	18	40068518	1xTMT6plex [N-Term]	1.345	1.819	1.31	4.075098038	52.22	0	2389.281221	3	797.0981445	-0.561137901	-0.000446905	99.70788026
																			SAVENCQDSWR	4	40068518	1xTMT6plex [N-Term]; 1xCarbamidomethyl [C6	1.434	1.261		4.683910847	66.74	0	1580.732471	2	790.8690796	-1.004692661	-0.000794075	38.54530863
																			GILFVGSGVSGGEEGAR	9	40068518	1xTMT6plex [N-Term]	1.321	1.537		3.834242105	64.82	0	1820.970392	2	910.9871826	-1.814266525	-0.001651863	61.76385624
																			AGQAVDDFIEK	4	40068518	2xTMT6plex [N-Term; K11]	1.741	100		3.919059277	63	0	1650.910307	2	825.9592285	0.528763583	0.000436471	72.15114979
																			VDDFLANEAK	6	40068518	2xTMT6plex [N-Term; K10]	1.491	1.883	100	4.212030411	55.87	0	1579.873194	2	790.4399414	-0.371882694	-0.000293764	72.20221191
																			TIFQGIAAK	8	40068518	2xTMT6plex [N-Term; K9]	1.246	1.22		3.872226954	62.29	0	1406.877157	2	703.9423828	0.235965875	0.000165987	72.69833054
																			MVHNGIEYGDMQLICEAYHLMK	2	40068518	2xTMT6plex [N-Term; K22]; 1xCarbamidomethyl [C15	100			2.856475353	19.52	0	3110.533595	4	778.390625	2.274410413	0.001768658	98.10687571
																			WTAISALEYGVPVTLIGEAVFAR	7	40068518	1xTMT6plex [N-Term]		100	100	2.288342476	7.1	0	2692.491154	2	1346.747314	-1.412046886	-0.001900962	112.6764096
																			DAFDRNPELQNLLLDDFFK	2	40068518	2xTMT6plex [N-Term; K19]	2.126			3.596055269	10.02	1	2768.465951	3	923.4907837	-2.945059078	-0.002717765	97.19048882
																			VVGAQSLK	6	40068518	2xTMT6plex [N-Term; K8]	1.374	1.453		3.423499346	44.79	0	1259.808743	2	630.4077148	-0.46839848	-0.000295046	51.33397314
																			FQFDGDKK	4	40068518	3xTMT6plex [N-Term; K7; K8]	1.576	1.517		2.574456692	9.43	1	1671.967318	3	557.9938965	-0.108726333	-6.06E-05	57.28118452
																			SFLEDIR	4	40068518	1xTMT6plex [N-Term]			0.835	2.217827082	37.54	0	1108.61999	2	554.8137207	0.157390496	8.72E-05	60.46441118
																			DVLGMAQDEMAQAFEDWNK	3	40068518	2xTMT6plex [N-Term; K19]; 1xOxidation [M				1.759232402	3.69	0	2672.273659	3	886.0964966	-1.433412783	-0.001269181	104.2821496
																			EAWPHIK	1	40068518	2xTMT6plex [N-Term; K7]	1.113			2.652229071	21.08	0	1338.793427	3	446.9367371	1.666023746	0.000743487	47.02272796
																			SFLEDIRK	2	40068518	2xTMT6plex [N-Term; K8]		100		1.978206158	10.5	1	1465.877885	3	489.297699	0.44916008	0.000219471	51.90735997
																			FQFDGDK	4	40068518	2xTMT6plex [N-Term; K7]	1.478		1.693	2.55877471	34.89	0	1314.709423	2	657.859436	1.652395788	0.00108621	54.86000191
4507947	YARS	tyrosine--tRNA ligase, cytoplasmic	8565	NP_003671.1	44.6969697	1	21	21	82	528	59.106	7.05	1.04	0.98	1.12	1.05	Significant	0.0015																
																			VHLMNPMVPGLTGSK	2	4507947	2xTMT6plex [N-Term; K15]	1.142			3.923497438	42.38	0	2039.154594	3	680.3893433	-0.547979465	-0.000372472	66.7926975
																			VDAQFGGIDQR	4	4507947	1xTMT6plex [N-Term]	1.093	1.077		4.012211323	73.97	0	1434.753858	2	717.8804321	-0.18832651	-0.000135101	52.09196314
																			AGCEVTILFADLHAYLDNMK	4	4507947	2xTMT6plex [N-Term; K20]; 1xCarbamidomethyl [C3			0.774	4.218592644	34.96	0	2739.425015	3	913.8053589	-8.575248352	-0.007830417	114.4376699
																			AMLESIGVPLEK	6	4507947	2xTMT6plex [N-Term; K12]	1.803	100		3.917393446	30.52	0	1745.028314	2	873.0169067	-1.01846682	-0.000888627	78.9882375
																			DFAAEVVHPGDLK	6	4507947	2xTMT6plex [N-Term; K13]	0.938	0.01	0.945	3.962206364	46.21	0	1856.03182	3	619.3447876	-6.470781861	-0.004003326	54.45691641
																			LASAAYPDPSK	4	4507947	2xTMT6plex [N-Term; K11]		0.882	1.178	3.230490208	36.71	0	1577.893929	2	789.449646	-1.212887493	-0.000956904	43.15555638
																			MSSSEEESK	4	4507947	2xTMT6plex [N-Term; K9]	1.091	1.391		4.134226799	47.08	0	1471.735045	2	736.3711548	-0.008364065	-6.15E-06	27.96678538
																			GVESQGMLLCASIEGINR	2	4507947	1xTMT6plex [N-Term]; 1xCarbamidomethyl [C10			100	2.502170801	23.83	0	2163.109939	2	1082.058105	-0.464568389	-0.000502456	77.33002625
																			QVEHPLLSGLLYPGLQALDEEYLK	3	4507947	2xTMT6plex [N-Term; K24]				2.545350313	11.17	0	3183.770572	3	1061.928467	0.086309807	9.16E-05	97.63428048
																			VSYYENVIK	5	4507947	2xTMT6plex [N-Term; K9]	2.518	100	0.691	2.885923147	35.67	0	1572.903766	2	786.9559937	0.600847114	0.000472537	61.99142646
																			ISEECIAQWK	3	4507947	2xTMT6plex [N-Term; K10]; 1xCarbamidomethyl [C5				2.312931061	28.54	0	1721.929663	2	861.4682617	-0.241712835	-0.000208106	70.3321427
																			GQPDEELKPK	4	4507947	3xTMT6plex [N-Term; K8; K10]	0.831	0.779		2.628508091	20.21	0	1828.078325	3	610.0307617	-0.324364521	-0.000197655	39.25392789
																			IDVGEAEPR	4	4507947	1xTMT6plex [N-Term]	0.881	1.093		3.50361571	57.91	0	1214.657832	2	607.8323364	-0.358988701	-0.000218024	41.26732288
																			KAGAEVVK	7	4507947	3xTMT6plex [N-Term; K1; K8]	1.054	0.913	1.029	3.894208193	19.1	1	1488.971675	3	496.9954529	0.087464779	4.34E-05	40.10543072
																			NLQEVLGEEK	6	4507947	2xTMT6plex [N-Term; K10]				2.1985147	22.9	0	1616.925958	2	808.9664307	-0.230574488	-0.000186411	59.22142823
																			LVVVLCNLKPQK	3	4507947	3xTMT6plex [N-Term; K9; K12]; 1xCarbamidomethyl [C6	100			1.565444946	1	0	2098.338914	3	700.117981	0.226808496	0.00015864	66.94672782
																			EYTLDVYR	6	4507947	1xTMT6plex [N-Term]	1.39	1.21	1.262	3.559251308	38.06	0	1287.678233	2	644.3427124	-0.066154192	-4.26E-05	49.86507016
																			KIFTFAEK	2	4507947	3xTMT6plex [N-Term; K1; K8]	0.929			2.720786333	17.71	1	1671.04484	3	557.6860962	-0.66110481	-0.000368245	69.54068763
																			IFTFAEK	4	4507947	2xTMT6plex [N-Term; K7]	100	1.221		2.338728905	30.7	0	1313.786945	2	657.3966064	-0.767878977	-0.000504415	74.80701285
																			SEFVILR	2	4507947	1xTMT6plex [N-Term]	0.899			2.427414894	21.13	0	1092.661461	2	546.8345947	0.413314774	0.000225807	58.72683652
																			TVVSGLVQFVPK	1	4507947	2xTMT6plex [N-Term; K12]		100		1.251099586		0	1732.077313	2	866.541748	-0.631476575	-0.000546883	75.66381227
5453607	CCT7	T-complex protein 1 subunit eta isoform a	10574	NP_006420.1	34.06998158	1	17	17	104	543	59.329	7.65	1.22	1.07	1.37	1.22	Significant	0.005																
																			ATISNDGATILK	12	5453607	2xTMT6plex [N-Term; K12]	1.18	1.205	1.355	4.598573685	62.41	0	1661.983807	2	831.4950562	-0.584274834	-0.000485528	60.53042097
																			GGAEQFMEETER	4	5453607	1xTMT6plex [N-Term]	2.011	1.487		4.676044941	68.39	0	1612.747452	2	806.8776245	0.322854913	0.000260342	45.69231598
																			STVDAPTAAGR	4	5453607	1xTMT6plex [N-Term]	2.206	2.131		3.972993612	67.33	0	1274.690195	2	637.8485107	-0.353133355	-0.000225068	29.50448841
																			SQDAEVGDGTTSVTLLAAEFLK	7	5453607	2xTMT6plex [N-Term; K22]	100	1.519	0.982	3.899437904	27.81	0	2710.455112	3	904.1569214	0.40558877	0.000366443	97.59054663
																			VHTVEDYQAIVDAEWNILYDK	5	5453607	2xTMT6plex [N-Term; K21]		100	2.632	6.389057636	49.34	0	2979.550409	3	993.8544922	-0.498597874	-0.000495199	99.85513272
																			TATQLAVNK	4	5453607	2xTMT6plex [N-Term; K9]	1.569	1.702		4.62624836	50.15	0	1403.862235	2	702.4355469	1.126841338	0.000790965	27.62820658
																			SLHDAIMIVR	8	5453607	1xTMT6plex [N-Term]	0.915	0.884		3.968673468	47.04	0	1383.797971	2	692.4031372	0.741758612	0.000513222	48.97436422
																			GGAEQFMEETER	6	5453607	1xTMT6plex [N-Term]; 1xOxidation [M7]	1.165	1.327		2.572519779	31.26	0	1628.742367	2	806.8776245	0.322854913	0.000260342	45.69231598
																			QQLLIGAYAK	8	5453607	2xTMT6plex [N-Term; K10]	100	100		3.287840366	33	0	1562.967035	2	781.9869385	-0.277841353	-0.000217128	55.03092859
																			IKEIAVTVK	6	5453607	3xTMT6plex [N-Term; K2; K9]	0.83	0.742	0.748	4.485010147	24.86	1	1688.128904	3	563.3804932	-1.171562081	-0.000659249	49.68659114
																			SLHDAIMIVR	2	5453607	1xTMT6plex [N-Term]; 1xOxidation [M7]		0.611		3.02064395	35.2	0	1399.792886	2	692.4031372	0.741758612	0.000513222	48.97436422
																			YNFFTGCPK	4	5453607	2xTMT6plex [N-Term; K9]; 1xCarbamidomethyl [C7	1.066			3.411191702	35.83	0	1591.834306	2	796.4212646	0.594576252	0.000473233	73.87197635
																			LLDVVHPAAK	6	5453607	2xTMT6plex [N-Term; K10]		1.22	1.507	2.838333607	29.05	0	1520.95647	3	507.6566467	-0.71204829	-0.000360998	62.04205503
																			QVKPYVEEGLHPQIIIR	2	5453607	2xTMT6plex [N-Term; K3]				2.150694609	12.75	0	2477.464435	4	620.121521	-0.073085055	-4.53E-05	52.27869746
																			VHTVEDYQAIVDAEWNILYDKLEK	2	5453607	3xTMT6plex [N-Term; K21; K24]				2.66498065	2.43	1	3578.934961	4	895.4869385	-2.524992448	-0.002259196	102.6801094
																			EIAVTVK	9	5453607	2xTMT6plex [N-Term; K7]	1.552	1.482	1.593	3.198658705	39.55	0	1217.786945	2	609.3970947	-0.026496322	-1.61E-05	59.85322822
																			QLCDNAGFDATNILNK	3	5453607	2xTMT6plex [N-Term; K16]; 1xCarbamidomethyl [C3				1.828421712	8.5	0	2252.174537	2	1126.588745	-1.919849263	-0.002161918	79.21233806
																			TCTFILR	4	5453607	1xTMT6plex [N-Term]; 1xCarbamidomethyl [C2	1.652	0.922	1.111	2.351641655	23	0	1139.644431	2	570.3260498	0.343729469	0.000195865	42.12234495
																			ALEIIPR	8	5453607	1xTMT6plex [N-Term]	1.015	1.045	1.12	2.571485281	33.79	0	1040.666547	2	520.8378906	1.881496053	0.000979005	55.589383
72534658	IFIT3	interferon-induced protein with tetratricopeptide repeats 3 isoform a	3437	NP_001026853.1	49.3877551	1	14	15	85	490	55.95	5.2	0.28	0.27	0.28	0.28	Significant	0.00015																
																			HLDGNNEAALECLR	6	72534658	1xTMT6plex [N-Term]; 1xCarbamidomethyl [C12	0.271	0.371	0.287	5.749380112	52.4	0	1840.917311	3	614.3112183	0.9725071	0.000596768	45.66971942
																			QAEELIQQEHADQAEIR	6	72534658	1xTMT6plex [N-Term]	0.3	0.244	0.275	5.859850883	79.92	0	2237.135954	3	746.3832397	-0.352178739	-0.000262624	46.54491025
																			GLNPLNAYSDLAEFLETECYQTPFNK	6	72534658	2xTMT6plex [N-Term; K26]; 1xCarbamidomethyl [C19				2.964078903	25.13	0	3492.739743	3	1164.915039	-2.627940579	-0.003059571	112.0950365
																			SEDTAVQHGLEGLSISK	7	72534658	2xTMT6plex [N-Term; K17]	0.281			5.197986603	51.36	0	2229.212697	3	743.7410889	-1.786899224	-0.001327793	60.09058118
																			QMQNTGESEASGNK	12	72534658	2xTMT6plex [N-Term; K14]	0.332	0.238		4.49881506	50.88	0	1938.959125	3	646.9911499	-0.117729645	-7.61E-05	26.85252395
																			ALEEKPNNPEFSSGLAIAMYHLDNHPEK	2	72534658	3xTMT6plex [N-Term; K5; K28]		0.173		4.579681873	27.45	0	3839.00412	5	768.6054077	-1.611773676	-0.001237521	70.0850134
																			DAPSGIGSIFLSASELEDGSEEMGQGAVSSSPR	2	72534658	1xTMT6plex [N-Term]				3.026914597	32.37	0	3496.658702	3	1166.222656	-1.511811833	-0.001762097	96.29355596
																			FSNPYSIEYSELDCEEGWTQLK	2	72534658	2xTMT6plex [N-Term; K22]; 1xCarbamidomethyl [C14			0.292	4.078022957	35.11	0	3153.512703	3	1051.843994	1.498707099	0.001575397	92.50011716
																			QMQNTGESEASGNK	2	72534658	2xTMT6plex [N-Term; K14]; 1xOxidation [M2	0.288			3.236747026	41.79	0	1954.95404	3	646.9911499	-0.117729645	-7.61E-05	26.85252395
																			EAEGEQFVEEALEK	10	72534658	2xTMT6plex [N-Term; K14]	0.01	0.01		2.967592716	29.98	0	2066.069387	2	1033.539917	1.53467468	0.001585372	78.4702677
																			VCNQIEFLNTEFK	6	72534658	2xTMT6plex [N-Term; K13]; 1xCarbamidomethyl [C2				2.658873558	29.53	0	2100.119983	2	1050.563843	0.202901205	0.000213058	80.57027827
																			SPCQTDVLR	2	72534658	1xTMT6plex [N-Term]; 1xCarbamidomethyl [C3	0.251			3.268499613	40.38	0	1304.683001	2	652.8450928	-0.070931502	-4.63E-05	37.2028204
																			QAIELSPDNQYVK	4	72534658	2xTMT6plex [N-Term; K13]				2.21317625	19.39	0	1963.090063	2	982.0474854	-1.206519721	-0.001184253	49.90657028
																			QFSTDVLK	4	72534658	2xTMT6plex [N-Term; K8]	0.267		0.183	3.253578901	37.94	0	1395.824787	2	698.4171143	1.550922182	0.001082408	56.78637146
																			EMIEALK	16	72534658	2xTMT6plex [N-Term; K7]	0.308	0.298	0.378	2.974083424	33.97	0	1291.76958	2	646.3884277	-0.00124732	-8.06E-07	67.48390612
																			AIELFQR	6	72534658	1xTMT6plex [N-Term]	0.01	0.204	0.185	2.616368055	28.64	0	1105.65671	2	553.3312378	-1.366811279	-0.000755612	50.78768346
659667952	SSB	lupus La protein	6741	NP_001281074.1	54.16666667	1	22	22	111	408	46.808	7.12	1.13	1.23	1.17	1.18	Significant	0.00061																
																			EVTWEVLEGEVEK	8	659667952	2xTMT6plex [N-Term; K13]	1.578	100	0.77	3.510581017	35.36	0	2005.089394	2	1003.046997	-1.334768107	-0.001338165	85.77864604
																			IIEDQQESLNK	8	659667952	2xTMT6plex [N-Term; K11]	1.024	1.245		4.662191868	67.63	0	1774.9951	2	888.0004272	-0.857330712	-0.000760879	54.39013314
																			EDLHILFSNHGEIK	4	659667952	2xTMT6plex [N-Term; K14]	1.49	1.308		2.94641161	31.43	0	2110.16971	4	528.2988281	1.787894112	0.00094319	63.33190931
																			FSGDLDDQTCR	4	659667952	1xTMT6plex [N-Term]; 1xCarbamidomethyl [C10	1.554	1.374		3.708505869	53.39	0	1542.705587	2	771.8562622	-0.220039352	-0.000169728	40.51031153
																			GSIFVVFDSIESAK	14	659667952	2xTMT6plex [N-Term; K14]	1.974			2.967194319	33.2	0	1957.10465	2	979.0551147	-0.867188074	-0.000848589	104.4967659
																			GQVLNIQMR	6	659667952	1xTMT6plex [N-Term]	1.146	1.429	1.263	3.347550154	49.17	0	1287.740457	2	644.3682251	-8.761808042	-0.005641467	47.73445387
																			KIIEDQQESLNK	2	659667952	3xTMT6plex [N-Term; K1; K12]	0.355			3.606676102	21.8	1	2132.252995	3	711.4230347	0.729821419	0.000518721	51.95638949
																			LDEGWVPLEIMIK	4	659667952	2xTMT6plex [N-Term; K13]	100	100		2.545888186	23.97	0	2001.149492	2	1001.077881	-0.503056495	-0.000503346	100.4233828
																			QKLEEDAEMK	8	659667952	3xTMT6plex [N-Term; K2; K10]	1.03	1.251	0.912	3.803610563	24.93	1	1908.071525	2	954.5388184	-0.61045471	-0.000582396	41.22366954
																			LTTDFNVIVEALSK	2	659667952	2xTMT6plex [N-Term; K14]	100			1.942431331	32.96	0	2008.173064	2	1004.589355	-0.81148509	-0.000814801	110.820605
																			AELMEISEDK	6	659667952	2xTMT6plex [N-Term; K10]	1.801	2.724	100	3.539364815	35.94	0	1622.871145	2	811.9395142	0.373985522	0.000303465	70.0842259
																			EETDKEEPASK	4	659667952	3xTMT6plex [N-Term; K5; K11]	1.634	1.383		2.469741344	12.06	1	1950.063463	3	650.6925049	-0.256982181	-0.000167044	30.2031847
																			ICHQIEYYFGDFNLPR	6	659667952	1xTMT6plex [N-Term]; 1xCarbamidomethyl [C2	0.921	2.999	0.737	2.603478193	11.99	0	2301.132389	3	767.7151489	-0.65000287	-0.000498581	81.81982916
																			LEEDAEMK	6	659667952	2xTMT6plex [N-Term; K8]	1.313	1.478	0.995	3.520579338	37.83	0	1422.755052	2	711.8811646	-5.93E-06	-4.22E-09	50.47147037
																			ETDLLILFK	6	659667952	2xTMT6plex [N-Term; K9]		100	100	1.907794952	23.84	0	1549.960552	2	775.482666	-1.610885352	-0.001248404	91.13324945
																			KFVETPGQK	2	659667952	3xTMT6plex [N-Term; K1; K9]	0.816			4.152006626	17.07	1	1721.056467	3	574.3581543	2.000120676	0.00114744	40.34051358
																			EGIILFK	4	659667952	2xTMT6plex [N-Term; K7]	1.32			2.693392515	33.62	0	1277.823331	2	639.4177856	3.88476947	0.002482025	94.53260475
																			AKQEQEAK	5	659667952	3xTMT6plex [N-Term; K2; K8]	0.467	0.665	0.801	2.462993145	15.01	1	1618.973132	3	540.3295288	0.556868896	0.000300519	22.40170677
																			YKETDLLILFKDDYFAK	2	659667952	4xTMT6plex [N-Term; K2; K11; K17]	0.01			2.062034369	6.34	2	3038.762413	4	760.444519	-2.029216927	-0.001541577	99.35027732

Bhat et al,2017. Cigarette smoke and chewing tobacco alter expression of different sets of miRNAs in oral keratinocytes

Supplementary Table S2(A). List of all proteins and peptides identified in OKF6/TERT1 cells chronically treated with chewing tobacco using Sequest and Mascot search algorithms

Accession	Gene_Symbol	Description	Gene_ID	Refseq_Accession	Coverage	Proteins	#Unique_Peptides	#Peptides	#PSMS	#AAs	MW [kDa]	calc. pI	OKF6-STE-6M/OKF6P_R1	OKF6-STE-6M/OKF6P_R2	OKF6-STE-6M/OKF6P_R3	Median fold change	Status	P-value (6M)	Sequence	#PSMs	Protein Accessions	Modifications	OKF6-STE-6M/OKF6P_R1	OKF6-STE-6M/OKF6P_R2	OKF6-STE-6M/OKF6P_R3	Xcorr	Ionscore	# Missed Cleavages	Theo. MH+ [Da]	Charge	m_z	DeltaM [ppm]	Deltam/z [Da]	RT [min]
																			GFPTDATLDDIKEWLEDK	2	659667952	3xTMT6plex [N-Term; K12; K18]		100		1.521993995	6.84	1	2780.496138	3	927.5021362	-1.540095142	-0.00142741	103.0879053
																			MAALEAK	4	659667952	2xTMT6plex [N-Term; K7]		1.022	1.212	3.115846872	36.63	0	1191.717151	2	596.3619995	-0.359662997	-0.000214308	42.85410693
																			AAQPGSGK	4	659667952	2xTMT6plex [N-Term; K8]	0.988	1.069		2.585691929	32.96	0	1173.699193	2	587.3527832	-0.769322971	-0.000451477	19.90250779
5031635	CFL1	cofilin-1	1072	NP_005498.1	73.4939759	1	10	15	183	166	18.491	8.09	1.25	1.31	1.3	1.29	Significant	0.00021																
																			HELQANCYEEVK	26	5031635	2xTMT6plex [N-Term; K12]; 1xCarbamidomethyl [C7	1.488	1.416	1.421	4.96638155	43.64	0	1978.010432	3	660.008728	0.605882563	0.000399481	38.45031922
																			EILVGDVGQTVDDPYATFVK	31	5031635	2xTMT6plex [N-Term; K20]	1.67	2.052	0.01	4.651925564	65.07	0	2624.422355	3	875.4783325	-0.72804265	-0.000636897	96.94599504
																			HELQANCYEEVKDR	19	5031635	2xTMT6plex [N-Term; K12]; 1xCarbamidomethyl [C7	1.203	1.181	1.095	4.980019093	27.62	1	2249.138486	3	750.3850708	0.966082061	0.000724284	25.74949069
																			KEDLVFIFWAPESAPLK	13	5031635	3xTMT6plex [N-Term; K1; K17]	1.371	1.632	1.506	4.592571259	52.08	1	2677.557222	3	893.1902466	-0.386719618	-0.000345155	100.808738
																			YALYDATYETK	14	5031635	2xTMT6plex [N-Term; K11]	1.274	1.213	1.172	4.850142479	68.77	0	1795.951838	2	898.4779663	-1.771697183	-0.001590941	59.09262505
																			KSSTPEEVK	10	5031635	3xTMT6plex [N-Term; K1; K9]	1.106	1.172	1.028	3.459278822	36.61	1	1692.014662	3	564.6760254	-0.673225919	-0.000379703	32.42733332
																			AVLFCLSEDKK	18	5031635	3xTMT6plex [N-Term; K10; K11]; 1xCarbamidomethyl [C5	0.988	1.192	0.871	3.553387165	26.92	1	1997.170845	3	666.3954468	0.47154305	0.000313917	71.78089801
																			LGGSAVISLEGKPL	5	5031635	2xTMT6plex [N-Term; K12]		100		1.9768399	5.38	0	1799.104256	2	900.0554199	-0.385172926	-0.000346483	83.5043388
																			KNIILEEGK	4	5031635	3xTMT6plex [N-Term; K1; K9]	0.949	1.035		4.308897495	32.18	1	1731.098332	3	577.7044067	0.193440683	0.000111622	47.52279514
																			AVLFCLSEDK	15	5031635	2xTMT6plex [N-Term; K10]; 1xCarbamidomethyl [C5	0.895	0.999	1.165	3.013589859	23.68	0	1639.91295	2	820.4547729	-6.513206424	-0.005340546	68.5555125
																			NIILEEGK	12	5031635	2xTMT6plex [N-Term; K8]	1.523	1.584	1.597	3.502896786	48.18	0	1373.840437	2	687.4240112	0.224699266	0.00015435	47.3917925
																			ESKKEDLVFIFWAPESAPLK	2	5031635	4xTMT6plex [N-Term; K3; K4; K20]			0.449	3.336945534	16.55	2	3250.889738	4	813.4727173	-6.367185922	-0.005174755	88.41964668
																			MIYASSK	10	5031635	2xTMT6plex [N-Term; K7]	1.178	1.229	1.346	2.857272387	29.23	0	1257.727716	2	629.3674927	-0.005524596	-3.47E-06	46.82987487
																			EDLVFIFWAPESAPLK	2	5031635	2xTMT6plex [N-Term; K16]				0.773588717	0.6	0	2320.299327	2	1160.652588	-0.615368342	-0.000713919	110.0920705
																			MLPDKDCR	2	5031635	2xTMT6plex [N-Term; K5]; 1xCarbamidomethyl [C7	1.117			2.633641243	17.91	1	1492.801626	2	746.9043579	-0.125053245	-9.33E-05	39.71362938
4506649	RPL3	60S ribosomal protein L3 isoform a	6122	NP_000958.1	35.73200993	1	14	14	92	403	46.08	10.18	1.48	1.52	1.55	1.52	Significant	0.00018																
																			TVFAEHISDECKR	11	4506649	2xTMT6plex [N-Term; K12]; 1xCarbamidomethyl [C11	1.42	1.393	1.417	5.689491272	26.88	1	2050.079181	3	684.03125	0.007863111	5.37E-06	40.42166157
																			TVFAEHISDECK	10	4506649	2xTMT6plex [N-Term; K12]; 1xCarbamidomethyl [C11	1.616	1.677	1.635	4.448369026	54.01	0	1893.97807	3	631.99646	-1.712288669	-0.001081012	44.7448102
																			SINPLGGFVHYGEVTNDFVMLK	4	4506649	2xTMT6plex [N-Term; K22]	2.41	1.588		5.440297604	43.55	0	2895.547907	3	965.8530273	-1.166559442	-0.001125943	99.92919923
																			SINPLGGFVHYGEVTNDFVMLK	2	4506649	2xTMT6plex [N-Term; K22]; 1xOxidation [M20			0.324	3.092238188	24.92	0	2911.542822	3	965.8530273	-1.166559442	-0.001125943	99.92919923
																			LEQQVPVNQVFGQDEMIDVIGVTK	2	4506649	2xTMT6plex [N-Term; K24]; 1xOxidation [M16			1.568	3.44659996	42.05	0	3160.696421	3	1048.904419	-0.891313232	-0.000934305	94.62372783
																			LEQQVPVNQVFGQDEMIDVIGVTK	9	4506649	2xTMT6plex [N-Term; K24]			1.223	4.363668919	51.7	0	3144.701506	3	1048.904419	-0.891313232	-0.000934305	94.62372783
																			NNASTDYDLSDK	2	4506649	2xTMT6plex [N-Term; K12]	2.454			3.357854366	36.44	0	1800.901593	2	900.9534302	-1.115773591	-0.001004699	42.90921976
																			DDPSKPVHLTAFLGYK	8	4506649	3xTMT6plex [N-Term; K5; K16]		1.27	1.521	3.353965044	18.66	0	2475.421457	4	619.6113281	0.818365695	0.00050645	73.1177877
																			KVACIGAWHPAR	2	4506649	2xTMT6plex [N-Term; K1]; 1xCarbamidomethyl [C4	0.725			4.083055496	10.66	1	1824.046699	3	608.6872559	0.282557528	0.000171799	48.89507164
																			IGQGYLIK	6	4506649	2xTMT6plex [N-Term; K8]		3.688	1.869	3.496705532	32.17	0	1349.855693	2	675.4309692	-0.764143488	-0.000515742	56.58198684
																			EVDRPGSK	6	4506649	2xTMT6plex [N-Term; K8]	0.99	1.171	1.073	2.554744005	9.39	0	1345.783985	3	449.2664795	0.668997828	0.000300109	24.69812299
																			SLLVQTK	6	4506649	2xTMT6plex [N-Term; K7]	1.628	1.824	1.734	3.506144524	40.25	0	1246.813494	2	623.9101563	-0.36758505	-0.000229155	44.85104006
																			AFMGPLK	8	4506649	2xTMT6plex [N-Term; K7]	1.617	1.459	2.341	2.994957209	32.66	0	1221.742972	2	611.3744507	-1.102631931	-0.000673566	60.06987025
																			GCVVGTK	6	4506649	2xTMT6plex [N-Term; K7]; 1xCarbamidomethyl [C2	2.088	1.884	1.33	2.83089304	33.57	0	1178.69675	2	589.8527832	1.30614278	0.000769773	22.71763743
																			AFMGPLKK	4	4506649	3xTMT6plex [N-Term; K7; K8]	0.735	0.763		2.652757168	11.37	1	1579.000867	3	527.0048218	-0.60465956	-0.000318253	61.48347298
																			FIDTTSK	6	4506649	2xTMT6plex [N-Term; K7]	0.721	1.408	1.296	2.511869669	24.97	0	1269.745474	2	635.3762207	-0.24366596	-0.000154697	52.51224428
16579885	RPL4	60S ribosomal protein L4	6124	NP_000959.2	40.04683841	1	15	15	111	427	47.667	11.06	1.16	1.07	1.2	1.14	Significant	0.0011																
																			AAAAAAALQAK	14	16579885	2xTMT6plex [N-Term; K11]	1.08	0.836	1.185	5.319098473	89.63	0	1414.87822	2	707.9422607	-0.688939593	-0.000487383	49.99742473
																			SGQGAFGNMCR	2	16579885	1xTMT6plex [N-Term]; 1xCarbamidomethyl [C10	1.582			4.713152885	47.57	0	1413.656469	2	707.3323975	0.742161832	0.000524581	36.09865625
																			APIRPDIVNFVHTNLR	16	16579885	1xTMT6plex [N-Term]	1.081	0.919	100	4.961306572	35.95	0	2091.202458	4	523.5565796	0.971000855	0.00050764	72.79841134
																			QPYAVSELAGHQTSAESWGTGR	8	16579885	1xTMT6plex [N-Term]	1.151	1.346	100	6.67190361	79.67	0	2561.258194	3	854.423584	-0.779084623	-0.000665146	61.15564369
																			YAICSALAASALPALVMSK	8	16579885	2xTMT6plex [N-Term; K19]; 1xCarbamidomethyl [C4	1.639			4.148874283	39.42	0	2395.349331	3	799.1209717	-0.404496716	-0.00032297	106.6242039
																			NIPGITLLNVSK	9	16579885	2xTMT6plex [N-Term; K12]	3.203	1.555	0.824	3.455828905	43.13	0	1727.083127	2	864.0444946	-0.818855888	-0.000707116	86.65476631
																			IEEVPELPLVVEDK	7	16579885	2xTMT6plex [N-Term; K14]	3.384	1.543	100	2.774027109	33.18	0	2067.198944	2	1034.102051	-1.025217	-0.001059664	92.27467733
																			KLDELYGTWR	11	16579885	2xTMT6plex [N-Term; K1]	1.939	0.931	1.273	3.680495024	14.82	1	1738.989227	3	580.3339844	-1.050400412	-0.000608878	60.23826995
																			SNYNLPMHK	4	16579885	2xTMT6plex [N-Term; K9]	1.06			3.390643358	47.97	0	1561.856104	3	521.289978	-0.462992696	-0.000241043	46.58283207
																			SGQGAFGNMCR	2	16579885	1xTMT6plex [N-Term]; 1xCarbamidomethyl [C10]; 1xOxidation [M9]	1.372			2.103777885	24.23	0	1429.651384	2	707.3323975	0.742161832	0.000524581	36.09865625
																			LDELYGTWR	2	16579885	1xTMT6plex [N-Term]	1.448			3.568165779	29.07	0	1381.731332	2	691.3707886	2.148737869	0.001484489	74.02399319
																			FCIWTESAFR	6	16579885	1xTMT6plex [N-Term]; 1xCarbamidomethyl [C2			1.445	2.882575512	11.04	0	1545.772151	2	773.3890991	-0.795186931	-0.000614589	76.30892192
																			EAVLLLK	11	16579885	2xTMT6plex [N-Term; K7]	1.212	1.317	1.034	3.299221039	36.3	0	1243.838981	2	622.4221802	-1.525075566	-0.000948474	64.8333846
																			NVTLPAVFK	7	16579885	2xTMT6plex [N-Term; K9]	100	100		1.881342411	14.47	0	1446.908457	2	723.9580078	0.194798087	0.000140928	63.91358984
																			GPCIIYNEDNGIIK	2	16579885	2xTMT6plex [N-Term; K14]; 1xCarbamidomethyl [C3				0.998672962		0	2064.119983	2	1032.563477	-0.148394959	-0.000153153	58.73229781
																			KVYASQR	2	16579885	2xTMT6plex [N-Term; K1]			0.935	2.572337389	9.05	1	1309.799241	3	437.2706604	-1.38429522	-0.000604383	28.68775164
6996014	HARS	histidine--tRNA ligase, cytoplasmic isoform	3035	NP_002100.2	43.02554028	1	15	19	79	509	57.374	5.88	1.37	1.39	1.29	1.35	Significant	0.00051																
																			TTETQVLVASAQK	6	6996014	2xTMT6plex [N-Term; K13]	1.76	1.86	1.685	4.734714985	81.15	0	1834.068599	2	917.5368042	-1.236116012	-0.001133561	46.07065525
																			ASAELIEEEVAK	9	6996014	2xTMT6plex [N-Term; K12]	1.099	1.272	1.083	3.724197865	54.94	0	1746.988952	2	873.9971924	-1.05523528	-0.000921742	73.15064592
																			GLDYYTGVIYEAVLLQTPAQAGEEPLGVGSVAAGGR	4	6996014	1xTMT6plex [N-Term]			1.555	6.152519226	34.82	0	3851.006449	4	963.5065308	-0.559818103	-0.000538966	105.1824047
																			REDLVEEIK	10	6996014	2xTMT6plex [N-Term; K9]	1.389	1.399	1.265	5.250209808	42.05	1	1588.931043	3	530.3153076	0.205592033	0.000108891	44.48500671
																			QALEGLGDLK	4	6996014	2xTMT6plex [N-Term; K10]	0.997	1.277		3.510604143	49.87	0	1501.899015	2	751.4516602	-1.978087389	-0.001485444	61.80592719
																			AALEELVK	6	6996014	2xTMT6plex [N-Term; K8]	0.93	1.096		3.390109777	49.68	0	1330.834624	2	665.9196777	-1.912079236	-0.001272331	63.0930931
																			AQLGPDESK	4	6996014	2xTMT6plex [N-Term; K9]	1.103	1.17		3.497354984	39.14	0	1402.794215	2	701.8995361	-1.724803502	-0.001209772	37.89361693
																			ISFDLSLAR	2	6996014	1xTMT6plex [N-Term]	1.142			3.39715004	22.61	0	1250.730603	2	625.8692627	0.516058873	0.000322725	95.65694043
																			LVSELWDAGIK	4	6996014	2xTMT6plex [N-Term; K11]	100	100		2.58518672	23.97	0	1688.998729	2	845.0014648	-1.820902792	-0.001537751	89.68185917
																			REDLVEEIKR	2	6996014	2xTMT6plex [N-Term; K9]	1.099			4.464654446	16.12	2	1745.032154	4	437.0134583	-0.086395077	-3.77E-05	49.9763245
																			YDGLVGMFDPK	5	6996014	2xTMT6plex [N-Term; K11]	100			1.595305204	16.73	0	1699.91295	2	850.4599609	-0.17930624	-0.000152403	77.22057849
																			ILDGMFAICGVSDSK	3	6996014	2xTMT6plex [N-Term; K15]; 1xCarbamidomethyl [C9	100	100		1.461582422	7.28	0	2071.096805	2	1036.049438	-2.512778188	-0.002602103	93.5492097
																			EFYQCDFDIAGNFDPMIPDAECLK	3	6996014	2xTMT6plex [N-Term; K24]; 2xCarbamidomethyl [C5; C22				1.923189044		0	3353.556893	3	1118.521851	-1.75769858	-0.001964847	99.21075841
																			VSWEEVK	6	6996014	2xTMT6plex [N-Term; K7]	1.561	1.775		3.192161322	34.13	0	1334.772023	2	667.8903809	1.095189834	0.000730914	58.90339445
																			KVPCVGLSIGVER	2	6996014	2xTMT6plex [N-Term; K1]; 1xCarbamidomethyl [C4		100		2.124874115	10.39	1	1872.11411	3	624.7098999	0.553794785	0.000345589	48.53112904
																			LEALEEK	2	6996014	2xTMT6plex [N-Term; K7]	1.416			2.745572329	33.88	0	1289.771689	2	645.3893433	-0.21631469	-0.000139498	61.29809927
																			VFDVIIR	3	6996014	1xTMT6plex [N-Term]	1.981	100		2.50461483	24.68	0	1090.682197	2	545.8450317	0.541107726	0.000295088	95.33085941
																			IFSIVEQR	2	6996014	1xTMT6plex [N-Term]	100			2.501001358	26.88	0	1220.720039	2	610.8646851	1.683299301	0.001027419	68.83048748
																			YDLTVPFAR	2	6996014	1xTMT6plex [N-Term]				1.616174102	21.82	0	1310.730603	2	655.8704224	2.26193434	0.001482393	53.75289978
155722983	TRAP1	heat shock protein 75 kDa, mitochondrial isoform 1 precursor	10131	NP_057376.2	33.66477273	1	16	17	128	704	80.06	8.21	1.01	1.11	1.24	1.12	Significant	0.0035																
																			ELGSSVALYSR	6	155722983	1xTMT6plex [N-Term]	100	1.116	1.218	3.773846388	57.67	0	1410.77901	2	705.8934326	0.410166561	0.000289327	49.69702844
																			ELISNASDALEK	6	155722983	2xTMT6plex [N-Term; K12]	0.662	0.581	1.205	3.047614098	52.19	0	1747.984201	2	874.4955444	-0.222171809	-0.000194176	59.10745176
																			GTITIQDTGIGMTQEELVSNLGTI AR	9	155722983	1xTMT6plex [N-Term]		1.24	0.672	4.41961956	43.81	0	2947.560766	3	983.1915894	-0.187139007	-0.000183868	94.74233944
																			SAAPGSLGYQWLSDGSGVFEIAE ASGVR	7	155722983	1xTMT6plex [N-Term]	1.162	1.584	1.486	5.769528866	61.58	0	3040.521344	3	1014.178223	-0.404447498	-0.00040991	100.6913085
																			GVVDSEDIPLNLSR	72	155722983	1xTMT6plex [N-Term]	1.313	1.533	1.788	4.148833275	65.84	0	1742.948594	2	871.9779053	-0.034627025	-3.02E-05	73.03318811
																			LVSDGQALPEMEIHLQTNAEK	2	155722983	2xTMT6plex [N-Term; K21]		100		2.499607801	9.88	0	2781.4857	3	927.8327026	-0.771375472	-0.00071519	64.83069113
																			YVAQAHDKPR	6	155722983	2xTMT6plex [N-Term; K8]	0.917	1.072		4.088224411	28.87	0	1642.942945	3	548.3187866	-0.692937464	-0.000379486	29.19392119
																			FFEDYGLFMR	4	155722983	1xTMT6plex [N-Term]		2.554		3.565431356	33.98	0	1553.766002	2	777.3861694	-0.605137975	-0.000470121	100.5059367
																			ELLQESALIR	6	155722983	1xTMT6plex [N-Term]	0.725	1.329	1.573	3.003807068	41.33	0	1400.831046	2	700.9179688	-1.702318069	-0.00119233	57.13226866
																			SIFYVPDMKPSMFDVSR	3	155722983	2xTMT6plex [N-Term; K9]	0.786	0.631		2.555282354	10.95	0	2477.297295	3	826.4355469	-2.102063007	-0.001735812	86.87367692
																			EWQHEEFYR	4	155722983	1xTMT6plex [N-Term]	0.862			3.618659496	35.83	0	1552.738208	2	776.8723755	-0.472341979	-0.000366712	49.6950288
																			AQLLQPTLEINPR	6	155722983	1xTMT6plex [N-Term]		1.115		2.049636841	13.22	0	1722.011135	2	861.5085449	-0.767594485	-0.000660903	59.67531626
																			AFLDALQNQAEASSK	4	155722983	2xTMT6plex [N-Term; K15]		100		1.490417957	9.85	0	2051.11734	2	1026.060425	-1.836521271	-0.00188346	77.85143466
																			YESSALPSGQLTSLSEYASR	4	155722983	1xTMT6plex [N-Term]				1.421728253	9.73	0	2375.1928	2	1188.097412	-2.211341012	-0.002626181	70.72033136
																			ATDILPK	4	155722983	2xTMT6plex [N-Term; K7]	1.087	1.061		2.808956861	30.69	0	1215.771295	2	608.3889771	-0.507956093	-0.000308779	49.61132013
																			FFIDQSK	6	155722983	2xTMT6plex [N-Term; K7]	1.354	1.986	100	2.058720827	22.8	0	1342.777109	2	671.8920288	-0.244026213	-0.000163836	61.72760373
																			LLDIVAR	15	155722983	1xTMT6plex [N-Term]	1.143			2.387044668	25.22	0	1028.666547	2	514.8363647	-1.063267602	-0.000546874	83.45666042
5730023	RUVBL2	ruvB-like 2	10856	NP_006657.1	43.62850972	1	19	19	85	463	51.125	5.64	1.18	1.17	1.13	1.16	Significant	0.00017																
																			KEVVHTVSLHEIDVINSR	4	5730023	2xTMT6plex [N-Term; K1]	0.876		0.795	5.317939758	14.66	1	2533.450242	4	634.1193848	2.158168497	0.001366903	54.20607483
																			GTSYQSPHGIPIDLLDR	5	5730023	1xTMT6plex [N-Term]	0.998	1.319	1.335	3.980071306	45.41	0	2098.113034	3	700.0425415	0.017897853	1.25E-05	64.31475372
																			GLGLDDALEPR	4	5730023	1xTMT6plex [N-Term]	1.458	100		3.80257988	60.06	0	1384.76336	2	692.8858032	0.700426759	0.000484963	58.02051973
																			FVQCPDGELQK	4	5730023	2xTMT6plex [N-Term; K11]; 1xCarbamidomethyl [C4	2.309	1.186		3.74566555	41.2	0	1778.951127	2	889.9785156	-0.771291566	-0.000686045	52.70661112
																			GTEVQVDDIK	4	5730023	2xTMT6plex [N-Term; K10]	0.859	1.263		3.880930901	67.71	0	1561.883758	2	781.4469604	1.847703724	0.001442949	43.97038073
																			AVLIAGQPGTGK	6	5730023	2xTMT6plex [N-Term; K12]	1.094	1.152	1.169	4.00621891	54.09	0	1569.972848	2	785.4898071	-0.325217209	-0.000255291	53.42964826
																			EVVHTVSLHEIDVINSR	7	5730023	1xTMT6plex [N-Term]	1.243	0.219	1.698	4.23119688	15.04	0	2176.192347	4	544.803772	0.418805179	0.00022785	58.5282624
																			ALESDMAPVLIMATNR	7	5730023	1xTMT6plex [N-Term]	100	0.715		2.568665981	41.26	0	1961.039734	2	981.0231934	-0.318260379	-0.000312061	92.01749395
																			DYDAMGSQTK	2	5730023	2xTMT6plex [N-Term; K10]	0.98			3.541898012	28.38	0	1573.793229	2	787.3990479	-1.531177559	-0.001204878	42.26501183
																			LLIVSTTPYSEK	4	5730023	2xTMT6plex [N-Term; K12]	1.478	1.104		2.149621964	18.7	0	1809.077373	2	905.0406494	-1.852061125	-0.001675261	69.23270513
																			AAGVVLEMIR	9	5730023	1xTMT6plex [N-Term]	100	1.135		2.362144709	40.8	0	1287.765609	2	644.3862305	-0.329495132	-0.000212156	68.36722016
																			EYQDAFLFNELK	6	5730023	2xTMT6plex [N-Term; K12]	100	100	100	2.109246969	13.33	0	1975.0577	2	988.0323486	-0.1413753	-0.000139612	82.62385527
																			GTEVQVDDIKR	3	5730023	2xTMT6plex [N-Term; K10]	0.843		1.055	3.647237539	16.19	1	1717.984869	3	573.3283081	-8.439115997	-0.004832758	39.36554277
																			QASQGMVGQLAAR	2	5730023	1xTMT6plex [N-Term]	1.133			2.607473373	7.56	0	1545.836876	2	773.4215088	-0.734211929	-0.000567486	42.73377981
																			IGLETSLR	4	5730023	1xTMT6plex [N-Term]	1.447	1.118		3.23659873	31.74	0	1117.67784	2	559.3425903	0.057739413	3.23E-05	59.54290249
																			VYSLFLDESR	6	5730023	1xTMT6plex [N-Term]	100	100	100	1.90160799	12.3	0	1457.783761	2	729.3950195	-0.684976419	-0.000499274	73.05834067
																			VQAGDVITIDK	2	5730023	2xTMT6plex [N-Term; K11]		100		1.971761942	19.02	0	1616.962343	2	808.9849243	0.141600584	0.000114481	52.03275589
																			MIESLTK	4	5730023	2xTMT6plex [N-Term; K7]		0.891	1.261	3.04198432	28.22	0	1279.76958	2	640.3890991	1.04797161	0.000670581	50.27978872
																			TTEMETIYDLGTK	2	5730023	2xTMT6plex [N-Term; K13]				1.9183532	7.7	0	1960.034915	2	980.5227051	1.64184639	0.001609038	74.27441408
24119203	TPM3	tropomyosin alpha-3 chain isoform Tpm3.1cy	7170	NP_705935.1	48.79032258	1	11	15	179	248	29.015	4.78	1	1.03	0.94	0.99	Significant	0.00071																
																			KIQVLQQQADDAEER	26	24119203	2xTMT6plex [N-Term; K1]	0.806	0.811	0.849	6.570205688	70.08	1	2229.22393	3	743.7470093	1.141407105	0.000848151	43.48423777
																			EQAEAEVASLNR	20	24119203	1xTMT6plex [N-Term]	1.02	1.265	0.996	4.707933426	69.04	0	1545.807016	2	773.4071045	-0.053787843	-4.16E-05	48.96826091
																			LVIIEGDLER	22	24119203	1xTMT6plex [N-Term]	1.786	1.968	1.545	3.56127429	38.31	0	1385.820147	2	693.4136353	-0.11013131	-7.63E-05	75.90399976
																			IQVLQQQADDAEER	10	24119203	1xTMT6plex [N-Term]	0.846	1.655	2.461	3.235919476	36.59	0	1871.966035	2	936.4865112	-0.154558927	-0.000144665	47.36009996
																			MLDQTLLDLNEM	8	24119203	1xTMT6plex [N-Term]	1.856	1.011	1.016	2.669377565	63.22	0	1664.84366	2	832.9259033	0.522403782	0.00043486	101.0289858
																			[illegible]															
21464101	YWHAG	14-3-3 protein gamma	7532	NP_036611.2	64.77732794	1	8	13	194	247	28.285	4.89	1.1	0.96	1.04	1.03	Significant	0.0015																
																			[illegible]															
24638454	ATP2A2	sarcoplasmic/endoplasmic reticulum calcium ATPase 2 isoform b	488	NP_733765.1	19.96161228	1	14	20	88	1042	114.683	5.34	0.72	0.6	0.96	0.76	Significant	0.019																
																			[illegible]															
83700235	EIF4A2	eukaryotic initiation factor 4A-II	1974	NP_001958.2	45.94594595	1	6	16	126	407	46.373	5.48	0.78	0.72	0.78	0.76	Significant	0.00069																
																			[illegible]															
106049292	PC	pyruvate carboxylase, mitochondrial precursor	5091	NP_071504.2	24.10865874	1	19	19	99	1178	129.551	6.84	0.86	0.9	0.88	0.88	Significant	0.00017																
																			[illegible]															
5803187	TALDO1	transaldolase	6888	NP_006746.1	42.72997033	1	17	17	112	337	37.516	6.81	1	0.95	0.81	0.92	Significant	0.0038																
																			[illegible]															

Bhat et al,2017. Cigarette smoke and chewing tobacco alter expression of different sets of miRNAs in oral keratinocytes

Supplementary Table S2(A). List of all proteins and peptides identified in OKF6/TERT1 cells chronically treated with chewing tobacco using Sequest and Mascot search algorithms

Accession	Gene_Symbol	Description	Gene_ID	Refseq_Accession	Coverage	Proteins	#Unique_Peptides	#Peptides	#PSMS	#AAs	MW [kDa]	calc. pI	OKF6-STE-6M/OKF6P_R1	OKF6-STE-6M/OKF6P_R2	OKF6-STE-6M/OKF6P_R3	Median fold change	Status	P-value (6M)	Sequence	#PSMs	Protein Accessions	Modifications	OKF6-STE-6M/OKF6P_R1	OKF6-STE-6M/OKF6P_R2	OKF6-STE-6M/OKF6P_R3	Xcorr	Ionscore	# Missed Cleavages	Theo. MH+ [Da]	Charge	m_z	DeltaM [ppm]	Deltam/z [Da]	RT [min]
																			ILDWHVANTDK	4	5803187	2xTMT6plex [N-Term; K11]	1.025		1.599	2.403164148	17.35	0	1769.99504	3	590.6693726	-0.833739183	-0.000491905	58.17577097
																			KFAADAVK	4	5803187	3xTMT6plex [N-Term; K1; K8]	1.435	1.071		3.61553669	21.74	1	1536.971675	3	512.9953613	-0.093967656	-4.81E-05	39.88645683
																			VSTEVDAR	10	5803187	1xTMT6plex [N-Term]		1.146	0.745	3.478466272	40.95	0	1105.605068	2	553.3068848	1.288381621	0.000712221	38.20564864
																			LVPVLSAK	5	5803187	2xTMT6plex [N-Term; K8]	1.125		0.953	2.859747648	31.82	0	1284.86553	2	642.9362793	-0.19285773	-0.000123898	72.81939061
																			LSFDKDAMVAR	3	5803187	2xTMT6plex [N-Term; K5]	1.01	100		2.636316776		1	1710.961297	3	570.9918213	-0.225950647	-0.000128864	59.09848555
																			ILDWHVANTDKK	3	5803187	3xTMT6plex [N-Term; K11; K12]	0.928	0.846		3.142978907	9.35	1	2127.252935	4	532.5683594	-0.624034866	-0.00033187	50.40096735
																			FAADAVK	4	5803187	2xTMT6plex [N-Term; K7]	1.153	0.441		2.625311613	30.69	0	1179.71378	2	590.3599243	-1.024135861	-0.000604094	49.85875913
																			TIVMGASFR	1	5803187	1xTMT6plex [N-Term]	0.87			2.009752035		0	1210.681545	2	605.8445435	0.21937566	0.000132797	60.15010926
34740329	HNRNPA3	heterogeneous nuclear ribonucleoprotein A	220988	NP_919223.1	29.89417989	1	10	12	121	378	39.571	9.01	1.13	1.22	0.71	1.02	Significant	0.023																
																			SSGSPYGGGYGSGGGSGGYGSR	16	34740329	1xTMT6plex [N-Term]	1.111	0.986	1.008	6.01610899	80.71	0	2139.952904	3	713.9852905	-5.414062126	-0.003861946	34.02580738
																			GFAFVTFDDHDTVDK	15	34740329	2xTMT6plex [N-Term; K15]	0.951	1.03	0.873	5.22302866	51.83	0	2172.101355	3	724.7058105	0.701123187	0.000507637	96.70017143
																			KLFIGGLSFETTDDSLR	6	34740329	2xTMT6plex [N-Term; K1]	0.858	0.61	0.775	6.39465189	43.23	1	2357.311682	3	786.4416504	-0.544929266	-0.000428189	87.69279084
																			LFIGGLSFETTDDSLREHFEK	6	34740329	2xTMT6plex [N-Term; K21]	0.242	0.443	100	4.287283897	26.64	1	2899.524194	4	725.637146	0.882873949	0.000639979	94.5713364
																			KLFIGGLSFETTDDSLREHFEK	2	34740329	3xTMT6plex [N-Term; K1; K22]			0.283	5.759974957	29.68	2	3256.782089	5	652.1617432	-0.761438628	-0.000495968	85.31910368
																			IETIEVMEDR	12	34740329	1xTMT6plex [N-Term]	1.141	100	0.996	3.696579695	43.82	0	1463.761311	2	732.3851929	1.228459978	0.000899086	63.58610511
																			GEEGHDPKEPEQLR	4	34740329	2xTMT6plex [N-Term; K8]	0.92			3.977035284	17.6	1	2079.087102	3	693.7003784	-0.250295475	-0.000173462	32.67596306
																			EDSVKPGAHLTVK	11	34740329	3xTMT6plex [N-Term; K5; K13]	1.242	1.288		3.93898344	25.96	0	2068.236951	3	690.0823975	-2.084643728	-0.001437179	42.2487925
																			EDTEEYNLR	6	34740329	1xTMT6plex [N-Term]	1.692	1.837		3.810845137	37.65	0	1397.674604	2	699.3417358	1.138054367	0.000795315	39.0350549
																			KLFVGGLK	72	34740329	3xTMT6plex [N-Term; K1; K8]	0.753	0.812	0.878	3.817184925	23.58	1	1549.044446	3	517.0199585	0.565534632	0.000292013	58.62888589
																			IFVGGIK	48	34740329	2xTMT6plex [N-Term; K7]	2.036	2.477	1.805	2.747451067	32.13	0	1191.786551	2	596.3962402	-1.130564234	-0.000673696	62.34943694
																			LFIGGLSFETTDDSLR	3	34740329	1xTMT6plex [N-Term]	100			1.183593631	7.12	0	2000.053788	2	1000.530518	-0.014496485	-1.45E-05	93.9785264
4505029	LTA4H	leukotriene A-4 hydrolase isoform 1	4048	NP_000886.1	36.82487725	1	18	18	94	611	69.241	6.18	0.84	0.92	0.82	0.86	Significant	0.0013																
																			LVVDLTDIDPDVAYSSVPYEK	8	4505029	2xTMT6plex [N-Term; K21]	100	1.419	0.987	2.961252213	32.53	0	2796.495914	3	932.8369751	0.163877575	0.000152761	92.16884082
																			SLSNVIAHEISHSWTGNLVTNK	2	4505029	2xTMT6plex [N-Term; K22]	0.94			4.586482525	22.75	0	2865.562311	4	717.1460571	0.0305649	2.19E-05	80.60335908
																			GSPMEISLPIALSK	6	4505029	2xTMT6plex [N-Term; K14]	0.965	0.696	0.234	3.931825161	46.14	0	1901.118192	2	951.0623779	-0.374742942	-0.000356215	85.79310968
																			EHPYLFSQCQAIHCR	4	4505029	1xTMT6plex [N-Term]; 2xCarbamidomethyl [C9; C14		0.824	0.786	4.677517891	44.52	0	2175.042529	4	544.515625	-0.854539612	-0.000464665	40.60448612
																			HFNALGGWGELQNSVK	4	4505029	2xTMT6plex [N-Term; K16]		1.315		3.975749254	27.74	0	2215.202407	3	739.0718994	-0.569680589	-0.000420653	76.28722204
																			DLSSHQLNEFLAQTLQR	9	4505029	1xTMT6plex [N-Term]	1.755	1.37	100	3.404286861	23.19	0	2229.18251	3	743.7323608	0.008563467	6.36E-06	92.58827107
																			LTYTAEVSVPK	7	4505029	2xTMT6plex [N-Term; K11]	1.074	100	100	3.465219259	39.47	0	1665.982744	2	833.494812	-0.238139659	-0.000198368	67.68661045
																			AILPCQDTPSVK	4	4505029	2xTMT6plex [N-Term; K12]; 1xCarbamidomethyl [C5	0.744		0.611	3.29648757	36.42	0	1787.013727	2	894.0102539	-0.277427919	-0.000247884	47.70793045
																			ASMHPVTAMLVGK	2	4505029	2xTMT6plex [N-Term; K13]		1.274		2.666208506	29.99	0	1800.027603	3	600.6806641	-0.090944439	-5.46E-05	66.30126294
																			SAYEFSETESMLK	6	4505029	2xTMT6plex [N-Term; K13]	0.558			2.846406221	34.1	0	1980.003615	2	990.5062866	0.849130868	0.000840641	94.21589914
																			TFGETHPFTK	6	4505029	2xTMT6plex [N-Term; K10]	0.809	0.873	0.838	3.045464754	35.9	0	1622.894264	3	541.6361084	-0.302918495	-0.000163868	36.39887938
																			APLPLGHIK	3	4505029	2xTMT6plex [N-Term; K9]	0.748			3.302439928	28.62	0	1403.913877	3	468.6428223	0.026089118	1.22E-05	46.49252243
																			EDDLNSFNATDLK	5	4505029	2xTMT6plex [N-Term; K13]	0.984			3.169612169	20.93	0	1940.001307	2	970.5025024	-1.844770503	-0.001789429	73.26880202
																			WEDAIPLALK	6	4505029	2xTMT6plex [N-Term; K10]	1.346	0.684	0.899	2.622884274	25.92	0	1613.9667	2	807.4868164	-0.213156504	-0.000172014	85.32193763
																			DFLYSYFK	4	4505029	2xTMT6plex [N-Term; K8]	100	100		2.067552567	20.12	0	1540.845188	2	770.9249878	-1.615479661	-0.001244602	80.80395389
																			SLVLDTK	6	4505029	2xTMT6plex [N-Term; K7]	1.029	0.978	0.863	3.123354912	40	0	1233.78186	2	617.3945313	-0.059832295	-3.69E-05	45.69631391
																			DLAAFDK	10	4505029	2xTMT6plex [N-Term; K7]	0.861	1.225		2.882898092	40.19	0	1237.719259	2	619.3624268	-1.359407121	-0.000841282	54.9295124
																			TLVWSEK	2	4505029	2xTMT6plex [N-Term; K7]		0.647		1.923213124	15.94	0	1320.792759	2	660.9000854	0.102626575	6.78E-05	66.24973026
574280091	PRKCSH	glucosidase 2 subunit beta isoform 3 precursor	5589	NP_001276032.1	41.4953271	1	15	15	105	535	60.154	4.41	0.96	0.99	1.26	1.07	Significant	0.0079																
																			ETMVTSTTEPSR	6	574280091	1xTMT6plex [N-Term]	1.27	1.246	1.191	3.464782238	58.95	0	1567.783503	2	784.3948975	-0.628000054	-0.000492284	35.04034064
																			AQQEQELAADAFK	4	574280091	2xTMT6plex [N-Term; K13]	100	1.254		3.272204161	43.92	0	1907.027462	2	954.0180664	0.73089797	0.000696921	53.34757898
																			YEQGTGCWQGPNR	10	574280091	1xTMT6plex [N-Term]; 1xCarbamidomethyl [C7	1.368	1.242	1.55	4.143444538	45.66	0	1781.822683	2	891.414917	-0.070307571	-6.26E-05	36.1000286
																			GVSLTNHHFYDESKPFTCLDGSATIPFDQVNDDYCDCK	2	574280091	3xTMT6plex [N-Term; K14; K38]; 3xCarbamidomethyl [C18; C35; C3[illegible]			100	3.204852581	5.14	0	5140.400622	5	1028.887817	1.820518429	0.001871639	80.32188566
																			ESLQQMAEVTR	4	574280091	1xTMT6plex [N-Term]	1.142	1.215		4.560894012	55.77	0	1520.794008	2	760.8990479	-2.096902577	-0.001594478	58.36806187
																			LWEEQLAAAK	12	574280091	2xTMT6plex [N-Term; K10]	1.369	0.586	1.094	3.594459295	40.52	0	1616.941214	2	808.9740601	-0.228971102	-0.000185116	69.76393062
																			SLEDQVEMLR	6	574280091	1xTMT6plex [N-Term]	1.508	1.095	1.014	3.596256971	41.1	0	1448.761645	2	724.8842773	-0.25349408	-0.000183626	60.81678414
																			VNDGVCDCCDGTDEYNSGVICENTCK	3	574280091	2xTMT6plex [N-Term; K26]; 5xCarbamidomethyl [C6; C8; C9; C21; C25]				1.907888293	1.23	0	3499.458061	3	1167.156128	-1.208810297	-0.00141006	54.27248962
																			ELDDDMDGTVSVTELQTHPELDTDGDGALSEAEAQALLSGDTQTDATSFYDR	2	574280091	1xTMT6plex [N-Term]; 1xOxidation [M6]				1.851285696	3.27	0	5790.593326	4	1448.405029	0.856828864	0.001240387	112.1400286
																			ESLQQMAEVTR	4	574280091	1xTMT6plex [N-Term]; 1xOxidation [M6]		1.129	1.27	2.900243521	49.61	0	1536.788923	2	760.8990479	-2.096902577	-0.001594478	58.36806187
																			KLIELQAGK	4	574280091	3xTMT6plex [N-Term; K1; K9]	0.863	0.86		4.807606697	31.39	1	1687.108503	3	563.0405884	-0.765222438	-0.000430338	51.41856244
																			ETMVTSTTEPSR	12	574280091	1xTMT6plex [N-Term]; 1xOxidation [M3]	1.303	1.77	2.939	2.096617222	42.53	0	1583.778418	2	784.3948975	-0.628000054	-0.000492284	35.04034064
																			SLEDQVEMLR	10	574280091	1xTMT6plex [N-Term]; 1xOxidation [M8]	0.602	100	100	2.343542099	30.19	0	1464.75656	2	724.8842773	-0.25349408	-0.000183626	60.81678414
																			LIELQAGK	4	574280091	2xTMT6plex [N-Term; K8]	1.259	1.196		3.715609074	45.19	0	1329.850608	2	665.4282837	-0.990470042	-0.000658589	57.98735022
																			SLKDMEESIR	4	574280091	2xTMT6plex [N-Term; K3]	0.514	0.558		3.394569159	17.6	1	1665.924577	3	555.9801025	0.706542903	0.000392349	56.1790239
																			ILIEDWK	10	574280091	2xTMT6plex [N-Term; K7]	1.062	1.11	0.946	2.895323515	30.22	0	1374.839709	2	687.9229736	-0.7551894	-0.000519132	86.41657716
																			EEAEKPER	4	574280091	2xTMT6plex [N-Term; K5]	1.503			1.938392997	8.63	0	1445.800029	3	482.6048584	-0.004754936	-2.29E-06	28.02205529
																			TVKEEAEKPER	4	574280091	3xTMT6plex [N-Term; K3; K8]	0.928	1.062	1.218	2.537180424		1	2003.174016	3	668.3959351	-0.381461726	-0.000254711	26.33267202
93141018	H2AFY	core histone macro-H2A.1 isoform 3	9555	NP_613258.2	45.69892473	1	12	14	78	372	39.592	9.79	1.01	0.99	0.94	0.98	Significant	0.00045																
																			AASADSTTEGTPADGFTVLSTK	12	93141018	2xTMT6plex [N-Term; K22]	1.249	1.502	1.505	4.895804882	75.25	0	2585.334662	3	862.4517212	2.300744389	0.001982731	64.64832667
																			FVIHCNSPVWGADKCEELLEK	8	93141018	3xTMT6plex [N-Term; K14; K21]; 2xCarbamidomethyl [C5; C15	0.771	1.083	1.109	3.358222961	13.28	1	3218.694537	4	805.4294434	0.436937238	0.000351592	67.09122363
																			GVTIASGGVLPNIHPELLAK	8	93141018	2xTMT6plex [N-Term; K20]	1.001	0.89		5.747594357	55.15	0	2444.464101	3	815.4926758	-0.256377768	-0.000208902	83.28706332
																			EFVEAVLELR	10	93141018	1xTMT6plex [N-Term]	2.34	1.236	0.764	3.854055643	53.1	0	1433.820147	2	717.4138184	0.148964813	0.000106794	99.54060881
																			LNLIHSEISNLAGFEVEAIINPTNADIDLK	2	93141018	2xTMT6plex [N-Term; K30]			2	6.908458233	58.38	0	3722.041661	4	931.2663574	0.520767277	0.000484579	108.4689055
																			HILLAVANDEELNQLLK	6	93141018	2xTMT6plex [N-Term; K17]		1.314	0.929	3.327798605	32.63	0	2391.401166	3	797.8042603	-1.228852073	-0.000979559	84.99265188
																			FVIHCNSPVWGADK	7	93141018	2xTMT6plex [N-Term; K14]; 1xCarbamidomethyl [C5	1.008	1.114	1.245	4.489873409	45.21	0	2088.110087	3	696.7080688	-0.207650465	-0.000144532	70.79652096
																			NCLALADDK	6	93141018	2xTMT6plex [N-Term; K9]; 1xCarbamidomethyl [C2	0.844	0.868	0.842	4.2096591	56.58	0	1477.808485	2	739.4076538	-0.307450402	-0.000227176	42.71302711
																			SIAFPSIGSGR	9	93141018	1xTMT6plex [N-Term]	0.83	1.011	0.01	3.307305574	44.21	0	1320.747316	2	660.8766479	-0.981793827	-0.000648351	67.93110818
																			NCLALADDKK	2	93141018	3xTMT6plex [N-Term; K9; K10]; 1xCarbamidomethyl [C2	1.162			3.266752243	20.29	1	1835.06638	3	612.3608398	0.864258245	0.000528657	44.68896846
																			AGVIFPVGR	2	93141018	1xTMT6plex [N-Term]	1.234			2.805185556	31.72	0	1144.703995	2	572.8556519	0.0282614	1.62E-05	62.65482291
																			LEAIITPPPAK	2	93141018	2xTMT6plex [N-Term; K11]	1.075			1.935679317	30.15	0	1608.01365	2	804.5114746	1.257575612	0.001011099	66.38053657
																			GKLEAIITPPPAK	2	93141018	3xTMT6plex [N-Term; K2; K13]	0.456			2.662684202	14.14	1	2022.293009	3	674.7697144	0.781670312	0.000526922	56.60802587
																			SLFLGQK	2	93141018	2xTMT6plex [N-Term; K7]	0.869			3.321951866	41.2	0	1250.787279	2	625.8982544	1.561175985	0.00097635	58.14202278
10716563	CANX	calnexin precursor	821	NP_001737.1	31.25	1	20	20	125	592	67.526	4.6	0.97	0.95	0.47	0.79	Significant	0.04																
																			APVPTGEVYFADSFDR	17	10716563	1xTMT6plex [N-Term]	0.862	0.884	0.883	3.259080887	45.9	0	1999.996273	2	1000.500549	-1.225340877	-0.001225339	72.48095559
																			QKSDAEEDGGTVSQEEEDRKPK	2	10716563	4xTMT6plex [N-Term; K2; K20; K22]	0.329			5.366436958	9.88	2	3378.779472	5	676.5620117	0.438100137	0.000296049	39.640281
																			QKSDAEEDGGTVSQEEEDR	4	10716563	2xTMT6plex [N-Term; K2]	1.047	0.662		3.839488745	36.23	1	2567.210918	3	856.4080811	-0.478416451	-0.000409399	35.13155695
																			GTLSGWILSK	10	10716563	2xTMT6plex [N-Term; K10]	1.489	0.685	1.151	3.600299358	51.57	0	1519.924835	2	760.4664917	0.573277321	0.000435669	79.57753511
																			TPELNLDQFHDK	15	10716563	2xTMT6plex [N-Term; K12]	1.167	1.849	1.412	3.26944375	35.37	0	1915.032548	3	639.0165405	1.316208455	0.000840194	59.18535006
																			IVDDWANDGWGLK	14	10716563	2xTMT6plex [N-Term; K13]	1.991	1.65	100	2.903563261	39.21	0	1947.037633	2	974.0220337	-0.432650696	-0.000421194	88.15576861
																			KDDTDDEIAK	6	10716563	3xTMT6plex [N-Term; K1; K10]	1.289	1.249	1.181	4.125054836	22.32	1	1837.015784	3	613.0093994	-1.164458047	-0.000713043	34.81120592
																			DDTDDEIAK	4	10716563	2xTMT6plex [N-Term; K9]	1.543	1.391		3.505695105	47.01	0	1479.757889	2	740.3826294	0.062854243	4.65E-05	40.95875431
																			IPDPEAVKPDDWDEDAPAK	5	10716563	3xTMT6plex [N-Term; K8; K19]	1.168		1.124	2.485512495	9.79	0	2795.470651	3	932.4962158	1.231646248	0.001147677	76.1702455
																			RPDADLK	2	10716563	2xTMT6plex [N-Term; K7]		1.161		3.714274168	17.55	0	1272.767607	3	424.927887	1.179342296	0.000500343	29.97989919
																			TPYTIMFGPDK	5	10716563	2xTMT6plex [N-Term; K11]	1.024			3.279426098	31.41	0	1727.94425	2	864.4731445	-3.031195885	-0.002618869	85.40832902
																			YDGKWEVEEMK	6	10716563	3xTMT6plex [N-Term; K4; K11]	100	100	100	2.278395653	6.66	1	2101.124289	3	701.0462036	-0.109993567	-7.70E-05	58.91979517
																			KPEDWDERPK	10	10716563	3xTMT6plex [N-Term; K1; K10]	0.888	0.966	0.898	3.407395124	6.25	0	1987.121587	3	663.045166	-0.322991371	-0.000213941	38.92195022
																			AEEDEILNR	2	10716563	1xTMT6plex [N-Term]				1.81625855	16.17	0	1317.684775	2	659.3461304	0.158529712	0.000104446	44.27015384
																			WEVEEMK	8	10716563	2xTMT6plex [N-Term; K7]	1.442	1.286	1.469	3.282027483	33.19	0	1408.754659	2	704.8805542	-0.586937944	-0.000413426	67.49682985
																			DKGDEEEEGEEKLEEK	1	10716563	4xTMT6plex [N-Term; K2; K12; K16]	0.805			2.570734024		2	2809.476079	4	703.1243286	-0.211746553	-0.000148724	53.3348354
																			GLVLMSR	7	10716563	1xTMT6plex [N-Term]	0.954	0.739	0.329	2.455106497	40.22	0	1004.612403	2	502.8096619	-0.353857398	-0.000177745	45.809876
																			EIEDPEDR	2	10716563	1xTMT6plex [N-Term]		1.074		3.289701939	20.08	0	1231.600377	2	616.3032227	-0.981030473	-0.000604119	41.24186719
																			IVDDWANDGWGLKK	2	10716563	3xTMT6plex [N-Term; K13; K14]	0.01			1.544084072	3.23	1	2304.295528	3	768.7644043	-7.320445303	-0.005622823	77.77785099
																			WEVEEMKESK	3	10716563	3xTMT6plex [N-Term; K7; K10]	0.354	0.01	0.01	1.756344199		1	1982.087175	3	661.3684692	1.856383962	0.001226505	50.60352585
15718687	RPS3	40S ribosomal protein S3 isoform 1	6188	NP_000996.2	71.19341564	1	16	16	161	243	26.671	9.66	1.09	1.25	1.07	1.13	Significant	0.0025																
																			ELAEDGYSGVEVR	26	15718687	1xTMT6plex [N-Term]	1.56	1.339	1.069	5.273017406	86.33	0	1652.832896	2	826.9207153	0.761228837	0.000629092	34.34643466
																			FVDGLMIHSGDPVNYYVDTAVR	10	15718687	1xTMT6plex [N-Term]	1.943	1.04		3.089323521	22.56	0	2697.354403	3	899.7871094	-2.827898697	-0.002542615	79.06808605
																			GLCAIAQAESLR	12	15718687	1xTMT6plex [N-Term]; 1xCarbamidomethyl [C3	1.541	1.429	1.824	4.625356674	76.74	0	1517.830728	2	759.4179077	-1.442473309	-0.001094715	60.55181014
																			DEILPTTPISEQK	10	15718687	2xTMT6plex [N-Term; K13]	1.297	1.368	1.383	3.495108843	37.34	0	1929.094479	2	965.0508423	-0.036960184	-3.56E-05	65.09828489
																			GCEVVVSGK	8	15718687	2xTMT6plex [N-Term; K9]; 1xCarbamidomethyl [C2	1.36	1.438		3.581981659	59.23	0	1392.792107	2	696.8997192	0.039342959	2.74E-05	36.88567407
																			AELNEFLTR	12	15718687	1xTMT6plex [N-Term]	100	100	1.185	3.194977999	40.33	0	1321.731332	2	661.3692627	-0.062629502	-4.14E-05	69.10835488
																			IMLPWDPTGK	10	15718687	2xTMT6plex [N-Term; K10]	1.086	1.715	0.562	2.66786027	28.19	0	1615.928206	2	808.4671631	-0.715816532	-0.000578354	93.64255132
																			KFVADGIFK	10	15718687	3xTMT6plex [N-Term; K1; K9]	0.982	1.118	0.925	4.817445755	29.35	1	1712.071389	3	571.3616333	-0.608910156	-0.000347499	70.18545713
																			KPLPDHVSIVEPK	14	15718687	3xTMT6plex [N-Term; K1; K13]	0.941	1.08	0.974	4.688374996	26.79	0	2146.320286	3	716.1113281	-0.398470352	-0.000285082	45.35646007
																			ELTAVVQK	12	15718687	2xTMT6plex [N-Term; K8]	1.389	1.483	1.384	3.842382908	51.18	0	1345.845523	2	673.4215088	-7.267982626	-0.004890791	45.25201032
																			FIMESGAK	8	15718687	2xTMT6plex [N-Term; K8]	1.534	1.498	1.618	3.315437555	33.97	0	1340.764829	2	670.8857422	-0.463671918	-0.000310837	58.41809684
																			FVADGIFK	8	15718687	2xTMT6plex [N-Term; K8]	1.29	1.452	100	3.185340643	34.3	0	1354.813494	2	677.9110107	0.923133982	0.000625337	59.55419146
																			LLGGLAVR	4	15718687	1xTMT6plex [N-Term]	1.687	1.567		2.524348259	33.88	0	1027.682531	2	514.3452759	0.72404443	0.000372044	64.28163996
																			GGKPEPPAMPQPVPTA	6	15718687	2xTMT6plex [N-Term; K3]	0.948	1.132	1.253	2.509678602	20.16	0	2032.130153	2	1016.568298	-0.410032945	-0.00041662	47.13706633
																			FGFPEGSVELYAEK	4	15718687	2xTMT6plex [N-Term; K14]	100	100		1.179135323	6.76	0	2031.083915	2	1016.043701	-1.865440528	-0.001894433	82.48235208
																			TEIIILATR	3	15718687	1xTMT6plex [N-Term]	3.297	100		2.283342838	27.83	0	1258.793204	2	629.9000854	-0.24569688	-0.000154641	69.7325397
																			FIMESGAK	2	15718687	2xTMT6plex [N-Term; K8]; 1xOxidation [M3]	1.359			1.877414227	29.01	0	1356.759744	2	670.8857422	-0.463671918	-0.000310837	58.41809684
																			IMLPWDPTGK	2	15718687	2xTMT6plex [N-Term; K10]; 1xOxidation [M2		1.082		1.513095617	8.55	0	1631.923121	2	808.4671631	-0.715816532	-0.000578354	93.64255132
5032179	TRIM28	transcription intermediary factor 1-beta	10155	NP_005753.1	39.04191617	1	21	21	90	835	88.493	5.77	1.4	1.26	1.75	1.47	Significant	0.0097																
																			HEPLVLFCESCDTLTCR	4	5032179	1xTMT6plex [N-Term]; 3xCarbamidomethyl [C8; C11; C16	1.686		0.82	3.581120491	33.35	0	2366.114039	3	789.3778076	2.041618233	0.001610234	67.77411134
																			IVAERPGTNSTGPAPMAPPR	5	5032179	1xTMT6plex [N-Term]	0.513	1.636	1.822	6.029350758	42.78	0	2248.206951	3	750.0743408	0.675374185	0.000506127	39.23113385
																			LDLDLTADSQPPVFK	4	5032179	2xTMT6plex [N-Term; K15]	100	1.736		3.216038942	38.8	0	2117.189442	2	1059.097168	-1.125498008	-0.001191446	88.14147417
																			ADVQSIIGLQR	6	5032179	1xTMT6plex [N-Term]	1.626	1.121	1.193	3.620681286	48.25	0	1428.837194	2	714.9223633	0.179448366	0.000128201	55.93964461
																			MIVDPVEPHGEMK	10	5032179	2xTMT6plex [N-Term; K13]	1.915	2.175	1.939	4.428121567	32.55	0	1940.038561	3	647.3502197	-1.26555748	-0.00081841	49.65414445
																			DHQYQFLEDAVR	7	5032179	1xTMT6plex [N-Term]	2.061		1.021	3.219930649	26.5	0	1749.875764	3	583.9634399	0.001613954	9.41E-07	39.35441472
																			DIVENYFMR	4	5032179	1xTMT6plex [N-Term]	3.666	1.245		3.435796976	42.19	0	1415.719052	2	708.3630981	-0.093691568	-6.63E-05	73.19249302
																			FSAVLVEPPPMSLPGAGLSSQELSGGPGDGP	2	5032179	1xTMT6plex [N-Term]				1.655513883	6.9	0	3179.613196	3	1060.543213	0.594274133	0.000629854	105.1546383
																			VLVNDAQK	2	5032179	2xTMT6plex [N-Term; K8]	1.305			3.290547609	40.82	0	1344.825121	2	672.9163818	0.271858303	0.000182801	45.10890821
																			VFPGSTTEDYNLIVIER	2	5032179	1xTMT6plex [N-Term]	0.769			1.677636147	10.39	0	2182.159315	2	1091.580078	-2.949152225	-0.00321776	84.02781866
																			MAILQIMK	9	5032179	2xTMT6plex [N-Term; K8]	0.705	100	100	2.678108692	25.51	0	1405.86752	2	703.4369507	-0.637067718	-0.000447816	82.58477892
																			DCQLNAHK	2	5032179	2xTMT6plex [N-Term; K8]; 1xCarbamidomethyl [C2			1.57	2.991499186	29.47	0	1443.777854	3	481.9305725	-0.47769863	-0.000229897	31.73691948
																			EEETEAAIGAPPTATEGPETKPVLMALAEGPGAEGPR	2	5032179	2xTMT6plex [N-Term; K21]				1.430515766	4.11	0	4132.116025	4	1033.7854	0.906787824	0.000936738	77.32079285
																			LLASLVK	6	5032179	2xTMT6plex [N-Term; K7]	2.265	1.681		2.949412823	39.54	0	1201.828416	2	601.4173584	-0.811965429	-0.000487922	84.30624685
																			DIVENYFMR	2	5032179	1xTMT6plex [N-Term]; 1xOxidation [M8]				1.589085698	22.5	0	1431.713967	2	708.3630981	-0.093691568	-6.63E-05	73.19249302
																			VTEGQQER	2	5032179	1xTMT6plex [N-Term]				2.492647409	27	0	1175.621781	2	588.3144531	-0.128867976	-7.58E-05	17.572501
																			KLLASLVK	6	5032179	3xTMT6plex [N-Term; K1; K8]				1.714865446	4.1	1	1559.086311	3	520.3662109	-1.431234104	-0.000743806	56.90020042
																			GAAAAATGQPGTAPAGTPGAPPLAGMAIVK	3	5032179	2xTMT6plex [N-Term; K30]				1.481533527	2.78	0	3030.681046	3	1010.897583	-0.940431051	-0.000950049	61.49548213
																			TVYCNVHK	2	5032179	2xTMT6plex [N-Term; K8]; 1xCarbamidomethyl [C4	1.291			2.805318356	31.25	0	1478.818991	3	493.6108093	-0.754515253	-0.00037193	35.75970812
																			LSPPYSSPQEFAQDVGR	3	5032179	1xTMT6plex [N-Term]	0.458			1.426695704	5.16	0	2107.065749	2	1054.036255	-0.24489714	-0.000258007	61.76039535
																			SAEAFGK	4	5032179	2xTMT6plex [N-Term; K7]	0.934	0.793		2.87377286	32.36	0	1167.677395	2	584.3423462	0.01805534	1.05E-05	45.71781139
																			LLPCLHSACSACLGPAAPAAANSSGDGGAAGDGTVVDCPVCK	3	5032179	2xTMT6plex [N-Term; K42]; 5xCarbamidomethyl [C4; C9; C12; C38; C41]				1.557794571	4.32	0	4570.165729	4	1143.294312	-2.25650597	-0.002578152	63.59265108
5803227	YWHAQ	14-3-3 protein theta	10971	NP_006817.1	65.71428571	1	9	14	191	245	27.747	4.78	1.23	1.19	1.12	1.18	Significant	0.00074																
																			YLAEVACGDDR	12	5803227	1xTMT6plex [N-Term]; 1xCarbamidomethyl [C7	1.235	1.22	1.168	4.456773758	60.04	0	1497.720509	2	749.3640747	0.242845084	0.000181857	54.88552577
																			AVTEQGAELSNEER	24	5803227	1xTMT6plex [N-Term]	1.08	1.04	1.022	4.848586082	71.21	0	1761.881637	2	881.4456787	1.387114676	0.001221966	36.44740389
																			YLIANATNPESK	10	5803227	2xTMT6plex [N-Term; K12]	1.514	1.577	1.39	4.224280357	52.47	0	1779.00527	2	890.0054932	-0.87730031	-0.000780361	61.6516983
																			TAFDEAIAELDTLNEDSYK	10	5803227	2xTMT6plex [N-Term; K19]	1.283	1.764	1.182	4.120842934	37.44	0	2603.312864	3	868.4401855	-2.635364911	-0.002286893	99.15466571
																			KQTIDNSQGAYQEAFDISK	4	5803227	3xTMT6plex [N-Term; K1; K19]		1.562		6.237860203	51.72	1	2830.518998	3	944.1773682	-0.511244692	-0.000482363	64.07737446
																			SICTTVLELLDK	4	5803227	2xTMT6plex [N-Term; K12]; 1xCarbamidomethyl [C3	2.699	100		3.386046171	37.01	0	1850.070907	2	925.5385132	-0.625750509	-0.000578841	115.1607764
																			DSTLIMQLLR	37	5803227	1xTMT6plex [N-Term]	1.27	1.271	1.324	4.110405922	66.2	0	1418.823852	2	709.9172974	2.443154983	0.001733203	90.31582375
																			YDDMATCMK	6	5803227	2xTMT6plex [N-Term; K9]; 1xCarbamidomethyl [C7	1.269	1.349	1.442	3.793501139	43.43	0	1592.752292	2	796.8810425	1.579631026	0.00125798	50.24331165
																			VISSIEQK	56	5803227	2xTMT6plex [N-Term; K8]	1.111	0.959	0.957	3.934776306	49.24	0	1361.840437	2	681.4239502	0.137042946	9.33E-05	59.72555441
																			NLLSVAYK	24	5803227	2xTMT6plex [N-Term; K8]	0.874	0.882	0.72	3.972506762	46.76	0	1365.850608	2	683.4294434	0.733725009	0.000501079	92.81997229
																			DSTLIMQLLR	14	5803227	1xTMT6plex [N-Term]; 1xOxidation [M6]	1.102	100		1.706976891	33.93	0	1434.818767	2	709.9172974	2.443154983	0.001733203	90.31582375
																			QTIDNSQGAYQEAFDISK	3	5803227	2xTMT6plex [N-Term; K18]				1.523873091	3.65	0	2473.261103	2	1237.131836	-1.903606131	-0.002354057	62.80629891
																			DNLTLWTSDSAGEECDAAEGAEN	3	5803227	1xTMT6plex [N-Term]; 1xCarbamidomethyl [C15				1.502789497	3.14	0	2684.146714	3	895.3815918	-6.144194462	-0.005497306	119.9681034
																			YDDMATCMK	2	5803227	2xTMT6plex [N-Term; K9]; 1xCarbamidomethyl [C7]; 1xOxidation [M8]	1.768			2.068782806	13.24	0	1608.747207	2	796.8810425	1.579631026	0.00125798	50.24331165
																			EMQPTHPIR	4	5803227	1xTMT6plex [N-Term]; 1xOxidation [M2]	0.564		100	2.449209452	23.08	0	1353.714636	3	451.908905	-1.827720587	-0.000824737	18.46995257
																			LAEQAER	6	5803227	1xTMT6plex [N-Term]	1.208	0.935	1.021	2.81289196	25.93	0	1045.583939	2	523.2957764	0.322273863	0.000168482	26.08720983
194272210	KARS	lysine--tRNA ligase isoform 1	3735	NP_001123561.1	33.76	1	18	18	81	625	71.451	6.81	1.26	1.33	1.45	1.35	Significant	0.0017																
																			GDIIGVQGNPGK	4	194272210	2xTMT6plex [N-Term; K12]	1.121	1.513		3.547950029	42.02	0	1612.942276	2	806.9732666	-1.872197734	-0.001509873	51.85075548
																			VTYHPDGPEGQAYDVDFTPPFR	6	194272210	1xTMT6plex [N-Term]	0.776	1.537	1.599	4.991240025	26.39	0	2737.309561	3	913.1077881	-0.273981503	-0.000249991	74.5036745
																			LVGEFLEVTCINPTFICDHPQIMSPLAK	9	194272210	2xTMT6plex [N-Term; K28]; 2xCarbamidomethyl [C10; C17		100		3.972594976	33.15	0	3687.935288	4	922.7400513	0.836990859	0.000771692	107.5222694
																			YSHLQPGDHLTDITLK	2	194272210	2xTMT6plex [N-Term; K16]		1.319		4.765349388	46.26	0	2296.270152	4	574.8222656	-1.271392217	-0.000729865	54.00491311
																			EICNAYTELNDPMR	4	194272210	1xTMT6plex [N-Term]; 1xCarbamidomethyl [C3	1.598	0.841		2.587096214	31.46	0	1954.920013	2	977.9638062	0.164904285	0.000161187	70.27422729
																			LPETNLFETEETR	6	194272210	1xTMT6plex [N-Term]	1.381	1.35	0.753	3.007220507	24.83	0	1807.927524	2	904.4664917	-1.005389618	-0.000908836	67.86544623
																			KILDDICVAK	4	194272210	3xTMT6plex [N-Term; K1; K10]; 1xCarbamidomethyl [C7	0.981	0.954		3.394852638	33.86	1	1862.138817	3	621.3850098	0.890946937	0.000553022	69.51770839
																			MLVVGGIDR	6	194272210	1xTMT6plex [N-Term]	1.417	1.402		3.441804886	40.57	0	1188.697195	2	594.852478	0.407736041	0.000242337	60.61328886
																			SEEEFIHINNK	6	194272210	2xTMT6plex [N-Term; K11]	1.538	1.789	1.606	3.746199369	24.51	0	1817.979784	3	606.6651001	0.5297655	0.000321034	47.86802379
																			EVLLFPAMKPEDK	5	194272210	3xTMT6plex [N-Term; K9; K13]	1.4	1.111		3.286514759	11.65	0	2204.296774	3	735.4362793	-1.129198847	-0.000829696	81.8496056
																			ILDDICVAK	6	194272210	2xTMT6plex [N-Term; K9]; 1xCarbamidomethyl [C6	1.048	3.202	100	3.280600071	50.62	0	1504.880922	2	752.9437256	-0.496695861	-0.000373734	71.21751261
																			INMVEELEK	4	194272210	2xTMT6plex [N-Term; K9]				2.004959583	13.17	0	1562.886401	2	781.9462891	-0.703483631	-0.000549732	64.41929789
																			YLDLILNDFVR	4	194272210	1xTMT6plex [N-Term]				1.880879879	16.14	0	1609.915109	2	805.4593506	-2.288889708	-0.001842459	103.4217207
																			VAMFLTDSNNIK	3	194272210	2xTMT6plex [N-Term; K12]			100	1.168354154	0.23	0	1811.013727	2	906.0095825	-1.015028716	-0.000919115	68.08115322
																			AVECPPPR	2	194272210	1xTMT6plex [N-Term]; 1xCarbamidomethyl [C4	1.192			2.506724596	17.18	0	1154.618945	2	577.8120728	-1.797893753	-0.001037941	28.57288806
																			FELFVMK	5	194272210	2xTMT6plex [N-Term; K7]	2.765	100		2.407127857	23.9	0	1371.811051	2	686.4090576	-0.155098346	-0.000106383	89.0758019
																			LQVMANSR	4	194272210	1xTMT6plex [N-Term]		0.525	1.077	2.617452621	18.79	0	1147.645494	2	574.3261108	-0.477926603	-0.000274245	38.33974063
																			SQAIHQLK	1	194272210	2xTMT6plex [N-Term; K8]	0.999			2.940362215	24.05	0	1382.852005	3	461.6221619	-0.052474376	-2.42E-05	35.82183469
91823262	ANXA8	annexin A8 isoform 2	653145	NP_001035173.1	57.79816514	1	2	15	116	327	36.858	5.78	1.03	0.99	100	34.01	Non-Significant	#N/A																
																			GIGTNEQAIIDVLTK	12	91823262	2xTMT6plex [N-Term; K15]	100	0.321	0.826	3.91865921	64.57	0	2030.189777	2	1015.599731	1.186928756	0.001204845	92.36049684
																			SSSHFNPDPDAETLYK	8	91823262	2xTMT6plex [N-Term; K16]	0.887	0.851	0.854	5.702804089	54.2	0	2266.139197	3	756.0505371	-0.944064635	-0.000713127	46.11302173
																			AYEEDYGSSLEEDIQADTSGYLER	27	91823262	1xTMT6plex [N-Term]	1.289	0.811	100	5.074197292	47.36	0	2969.337351	3	990.449585	-1.060676782	-0.001049836	91.23192367
																			SETHGSLEEAMLTVVK	5	91823262	2xTMT6plex [N-Term; K16]	1.213		100	5.191122532	36.43	0	2189.18879	3	730.3997803	-1.828375746	-0.00133422	82.12384011
																			SWIEQEGVTVK	6	91823262	2xTMT6plex [N-Term; K11]	0.889	0.993		4.310604572	61.9	0	1733.983807	2	867.4944458	-1.264001676	-0.001095879	59.5973183
																			DDVSSFVDPGLALQDAQDLYAAGEK	11	91823262	2xTMT6plex [N-Term; K25]	1.373	0.965	100	4.721148491	46.45	0	3082.562096	3	1028.192383	0.162049453	0.000166509	97.72697534
																			SEIDLNLIK	10	91823262	2xTMT6plex [N-Term; K9]	0.929	1.07	1.078	3.64244175	53.38	0	1502.919416	2	751.9629517	-0.524958078	-0.000394485	77.59300504
																			CTQNLHSYFAER	2	91823262	1xTMT6plex [N-Term]; 1xCarbamidomethyl [C1			1.265	4.489290237	29.52	0	1754.848169	3	585.6152344	-9.698420239	-0.005673085	42.67728741
																			ILVCLLQGSR	5	91823262	1xTMT6plex [N-Term]; 1xCarbamidomethyl [C4	1.934	100	0.722	2.45731926	29.72	0	1387.829272	2	694.4176636	-0.879972477	-0.000610626	82.56305909
																			LIVALMYPPYR	6	91823262	1xTMT6plex [N-Term]				1.703364611	17.54	0	1564.912273	2	782.9595337	-0.308028249	-0.000241019	87.95973983
																			FITILCTR	8	91823262	1xTMT6plex [N-Term]; 1xCarbamidomethyl [C6		100	1.196	2.754993677	28.53	0	1252.728495	2	626.8672485	-1.017570601	-0.00063737	80.84486513
																			EGVIIEILASR	2	91823262	1xTMT6plex [N-Term]	1.116			1.379170179	12.24	0	1428.862346	2	714.9346313	-0.251658033	-0.000179792	99.26305716
																			VFEEYEK	4	91823262	2xTMT6plex [N-Term; K7]	1.031		0.807	3.336134195	39.13	0	1401.766603	2	701.3873291	0.555073236	0.000389042	60.17754059
																			DLTETLK	6	91823262	2xTMT6plex [N-Term; K7]	0.932	0.988	0.957	3.22570467	34.69	0	1277.771689	2	639.3892822	-0.31387992	-0.000200533	44.97509592
																			SIEDSIK	3	91823262	2xTMT6plex [N-Term; K7]	0.995		0.998	3.039762259	34.9	0	1249.740389	2	625.3739014	0.109890323	6.87E-05	44.99581467
																			SETHGSLEEAMLTVVK	1	91823262	2xTMT6plex [N-Term; K16]; 1xOxidation [M11				1.449336171		0	2205.183705	3	730.3997803	-1.828375746	-0.00133422	82.12384011
4758032	COPB2	coatomer subunit beta'	9276	NP_004757.1	25.93818985	1	18	18	80	906	102.422	5.27	1.07	1.28	0.99	1.11	Significant	0.006																
																			QQALTVSTDPEHR	6	4758032	1xTMT6plex [N-Term]	1.028	1.402	1.69	4.377293587	43.24	0	1710.897227	3	570.9699707	-1.092164155	-0.00062286	32.08586552
																			DNNQFASASLDR	6	4758032	1xTMT6plex [N-Term]	7.413	3.81	0.99	5.026600361	77.2	0	1566.770964	2	783.888855	-0.338925774	-0.00026551	43.58885633
																			KNWVVTGADDMQIR	4	4758032	2xTMT6plex [N-Term; K1]	1.316	1.402		5.080654144	50.6	1	2091.142115	3	697.7192383	0.500522534	0.000348888	67.70580971
																			AAESLADPTEYENLFPGLK	2	4758032	2xTMT6plex [N-Term; K19]		100		2.480829716	23.07	0	2523.338291	3	841.7841797	-0.120973673	-0.000101753	87.96268412
																			SFGSAQEFAWAHDSSEYAIR	3	4758032	1xTMT6plex [N-Term]	1.231			4.125521183	25.08	0	2488.173068	3	830.0627441	0.245787515	0.000203854	79.97182123
																			VHMFEAHSDYIR	2	4758032	1xTMT6plex [N-Term]	1.052			3.824009418	24.86	0	1733.863091	3	578.6258545	-0.046383961	-2.68E-05	53.35071611
																			GSNNVALGYDEGSIIVK	3	4758032	2xTMT6plex [N-Term; K17]		100	100	2.410783768	15.61	0	2194.211968	2	1097.608276	-1.227039896	-0.001346193	58.8214393
																			VWQLGSSSPNFTLEGHEK	4	4758032	2xTMT6plex [N-Term; K18]			100	2.190507412	9.35	0	2474.307994	3	825.4407959	-0.064508819	-5.32E-05	62.58992649
																			GVNCIDYYSGGDKPYLISGADDR	2	4758032	2xTMT6plex [N-Term; K13]; 1xCarbamidomethyl [C4				1.907710195	3.59	0	2993.471507	3	998.4954834	0.130285293	0.000130002	58.74297793
																			QLAELAISK	6	4758032	2xTMT6plex [N-Term; K9]		2.635	100	2.414920807	32.85	0	1430.898286	2	715.9523315	-0.6288945	-0.000449942	58.7013487
																			EAFVVEEWVK	10	4758032	2xTMT6plex [N-Term; K10]	0.884	100	0.769	2.593101978	24.48	0	1693.956529	2	847.4802856	-1.909583204	-0.001617375	76.78009471
																			FELALQLGELK	2	4758032	2xTMT6plex [N-Term; K11]	100			2.157214165	23.07	0	1719.045679	2	860.0250244	-1.690788041	-0.001453271	95.05097952
																			SFKPDFGAESIYGGFLLGVR	3	4758032	2xTMT6plex [N-Term; K3]	100		0.761	1.783233047		0	2618.43828	3	873.4854126	1.300241825	0.001134868	100.4917378
																			AMGDAEIKDGER	5	4758032	2xTMT6plex [N-Term; K8]	0.606	0.702	0.616	3.438351393	5.98	1	1749.920555	3	583.9789429	0.983383661	0.000573614	42.72561847
																			LESTLNYGMER	4	4758032	1xTMT6plex [N-Term]				1.529076695	24.09	0	1541.783109	2	771.3955688	0.487789306	0.000376033	55.66975166
																			AMGDAEIK	8	4758032	2xTMT6plex [N-Term; K8]	0.964	1.148	1.001	3.211769104	41.09	0	1292.844443	2	646.8669434	-1.418558753	-0.000916906	38.60976998
																			VAHFLEK	6	4758032	2xTMT6plex [N-Term; K7]	0.993			3.051260471	26.4	0	1301.798178	2	651.4038696	1.754602096	0.001142069	49.93645446
																			IWDYQNK	4	4758032	2xTMT6plex [N-Term; K7]	1.459	1.324		3.144350767	33.79	0	1424.793821	2	712.8999634	-0.822008188	-0.000585596	66.01105612
146231940	CTNND1	catenin delta-1 isoform 1ABC	1500	NP_001078927.1	25.82644628	1	17	17	59	968	108.103	6.23	0.83	0.84	0.75	0.8	Significant	0.0012																

Bhat et al,2017. Cigarette smoke and chewing tobacco alter expression of different sets of miRNAs in oral keratinocytes

Supplementary Table S2(A). List of all proteins and peptides identified in OKF6/TERT1 cells chronically treated with chewing tobacco using Sequest and Mascot search algorithms

Accession	Gene_Symbol	Description	Gene_ID	Refseq_Accession	Coverage	Proteins	#Unique_Peptides	#Peptides	#PSMS	#AAs	MW [kDa]	calc. pI	OKF6-STE-6M/OKF6P_R1	OKF6-STE-6M/OKF6P_R2	OKF6-STE-6M/OKF6P_R3	Median fold change	Status	P-value (6M)	Sequence	#PSMSs	Protein Accessions	Modifications	OKF6-STE-6M/OKF6P_R1	OKF6-STE-6M/OKF6P_R2	OKF6-STE-6M/OKF6P_R3	Xcorr	Ionscore	# Missed Cleavages	Theo. MH+ [Da]	Charge	m_z	DeltaM [ppm]	Deltam/z [Da]	RT [min]
																			HYEDGYPGGSDNYGSLSR	6	146231940	1xTMT6plex [N-Term]	0.795	0.551	0.683	4.92966032	36.74	0	2202.988955	3	735.0010376	-0.179654569	-0.000131926	38.88331068
																			TVQPVAMGPDGLPVDASSVSNNYIQTLGR	4	146231940	1xTMT6plex [N-Term]			100	2.275061131	22.73	0	3215.656792	3	1072.553467	-3.403609304	-0.00364828	80.68949071
																			SNAAAYLQHLCYR	2	146231940	1xTMT6plex [N-Term]; 1xCarbamidomethyl [C11	0.905			4.401621342	40.5	0	1795.911104	3	599.3083496	-0.338636959	-0.000202721	63.30338934
																			DMDLTEVITGTLWNLSSHDSIK	3	146231940	2xTMT6plex [N-Term; K22]		1.173		6.488376141	70.87	0	2933.533044	3	978.5164185	0.565138033	0.000552617	117.3613433
																			SYEDMIGEEVPSDQYYWAPLAQHER	4	146231940	1xTMT6plex [N-Term]			1.565	4.56301403	37.16	0	3242.493809	3	1081.5	-2.578959435	-0.00278742	84.09190666
																			ALSAIADLLTNEHER	10	146231940	1xTMT6plex [N-Term]	4.252	1.44	0.746	3.364902496	45.99	0	1882.023156	3	628.0117798	-1.259306786	-0.000790015	97.56481372
																			SQSSHSYDDSTLPLIDR	2	146231940	1xTMT6plex [N-Term]			0.634	5.448515415	41.08	0	2150.056307	3	717.3580322	1.505467405	0.001078947	51.66176124
																			EVHLGACGALK	2	146231940	2xTMT6plex [N-Term; K11]; 1xCarbamidomethyl [C7		0.837		4.208488464	38.15	0	1612.924518	3	538.3131714	0.274513873	0.00014759	49.99024017
																			GKKPIEDPANDTVDFPK	2	146231940	4xTMT6plex [N-Term; K2; K3; K17]	0.726			4.568998814	8.26	1	2787.606246	4	697.6566772	-0.490480184	-0.000341816	58.90398629
																			GIPVLVGLLDHPK	5	146231940	2xTMT6plex [N-Term; K13]	1.162	0.763		2.111810923	35.68	0	1816.146061	3	606.0531616	-0.622123266	-0.000376622	97.89880519
																			SLDNNYSTPNER	2	146231940	1xTMT6plex [N-Term]	0.905			4.512010574	46.74	0	1638.792094	2	819.9007568	1.307897374	0.001071686	35.02362169
																			GYELLFQPEVVR	6	146231940	1xTMT6plex [N-Term]	0.684	100	0.479	3.363567114	25.51	0	1678.936573	2	839.9716797	-0.29209263	-0.000245202	79.57752941
																			QDVYGPQPQVR	2	146231940	1xTMT6plex [N-Term]	0.809			3.402349949	39.63	0	1515.811707	2	758.4094238	-0.089729977	-6.80E-05	36.16437229
																			LVENCVCLLR	2	146231940	1xTMT6plex [N-Term]; 2xCarbamidomethyl [C5; C7		100		2.222748995	24.08	0	1504.817721	2	752.9122314	-0.355398111	-0.000267405	71.8585006
																			YRPSMEGYR	2	146231940	1xTMT6plex [N-Term]			0.866	3.691568613	28.68	0	1387.698986	3	463.2377625	-0.18122553	-8.38E-05	36.50940318
																			EPNEDCKPR	1	146231940	2xTMT6plex [N-Term; K7]; 1xCarbamidomethyl [C6			0.676	2.200709581		0	1602.831012	3	534.947937	-1.094223178	-0.000584618	27.91962781
																			NLAVDAR	4	146231940	1xTMT6plex [N-Term]	0.988	0.842		2.656433821	42.34	0	987.5784599	2	494.2915039	-2.763018444	-0.001364349	34.51463841
150418007	RANBP2	E3 SUMO-protein ligase RanBP2	5903	NP_006258.3	7.94044665	1	21	22	62	3224	357.974	6.2	0.99	0.8	1.13	0.97	Significant	0.0095																
																			KAEDIENDALSPEEQEECK	2	150418007	3xTMT6plex [N-Term; K1; K19]; 1xCarbamidomethyl [C18			0.953	2.849993944	23.88	1	2921.465308	3	974.4866943	-6.76996305	-0.006592737	47.19047243
																			EIDTDSTSQGESK	4	150418007	2xTMT6plex [N-Term; K13]			1.331	3.739021301	43.22	0	1854.933287	2	927.9703369	0.059354224	5.50E-05	32.41316129
																			APGTNVAMASNQAVR	3	150418007	1xTMT6plex [N-Term]	0.627	0.567		3.14530468	18.09	0	1715.906018	2	858.4557495	-1.046459738	-0.000897813	39.38846884
																			SWWDAVCTLIHR	2	150418007	1xTMT6plex [N-Term]; 1xCarbamidomethyl [C7	1.354			2.612270355	16.72	0	1772.910376	3	591.6414795	-0.27654158	-0.000163428	100.3996673
																			ELVGPPLAETVFTPK	5	150418007	2xTMT6plex [N-Term; K15]		100		1.642799258	10.69	0	2056.209449	2	1028.608887	0.509372963	0.000523689	73.63705307
																			DTSFLGSDDIGNIDVR	4	150418007	1xTMT6plex [N-Term]				1.867869496	10.27	0	1952.976265	2	976.9905396	-1.261140999	-0.001231489	64.77899173
																			EIVETFANK	4	150418007	2xTMT6plex [N-Term; K9]	1.109		100	2.852278233	25.86	0	1508.872465	2	754.940918	1.387676923	0.001046914	61.25513265
																			LQDVADSFK	2	150418007	2xTMT6plex [N-Term; K9]	1.901			2.574217796	18.63	0	1480.841165	2	740.9254761	1.695089586	0.001255079	67.70108082
																			IAVAVLEETTR	2	150418007	1xTMT6plex [N-Term]	2.143			2.829273462	34.21	0	1430.84161	2	715.9233398	-1.542541456	-0.001103566	82.74581899
																			KIESFGSPK	2	150418007	3xTMT6plex [N-Term; K1; K9]	1.074			3.890218258	17.15	1	1680.029918	3	560.6821289	1.140002764	0.000638413	44.55670692
																			ILDDSDSNLSVVK	6	150418007	2xTMT6plex [N-Term; K13]			100	1.351307154	10.58	0	1863.047529	2	932.026123	-1.37387598	-0.001279798	57.63888312
																			EYLESLQCLESDK	4	150418007	2xTMT6plex [N-Term; K13]; 1xCarbamidomethyl [C8				1.518584967	12.73	0	2072.062193	2	1036.528687	-5.837982646	-0.006048332	72.34932912
																			LLVQHEINTLR	2	150418007	1xTMT6plex [N-Term]			1.232	2.907692432	14.94	0	1564.937242	3	522.3178711	1.161791797	0.000606044	49.6178863
																			KLPVPLESVK	6	150418007	3xTMT6plex [N-Term; K1; K10]	0.774	100	100	2.951070547	8.75	1	1797.181668	3	599.7320557	-0.030079214	-1.80E-05	53.94405158
																			EPTSHDNKDICK	1	150418007	3xTMT6plex [N-Term; K8; K12]; 1xCarbamidomethyl [C11	1.059			2.280421257		1	2131.142064	4	533.5405884	-0.722914911	-0.000385159	34.13070472
																			LFPGSPAIYK	2	150418007	2xTMT6plex [N-Term; K10]	1.229			3.023577452	36.48	0	1550.934672	2	775.9713135	0.437454354	0.000339232	71.34971609
																			EVADCFK	2	150418007	2xTMT6plex [N-Term; K7]; 1xCarbamidomethyl [C5	1.632			3.012645483	26.64	0	1326.712794	2	663.859436	-0.903518601	-0.000599355	46.87048467
																			GFSGAGEK	2	150418007	2xTMT6plex [N-Term; K8]		1.004		2.563373566	19.1	0	1210.683208	2	605.8447876	-0.75142257	-0.000454867	38.10101428
																			GEAGQNLLEMMACDR	2	150418007	1xTMT6plex [N-Term]; 1xCarbamidomethyl [C13				1.127237558	6.73	0	1923.892418	2	962.4459839	-4.016454604	-0.003863613	70.73515987
																			ALCTGEK	8	150418007	2xTMT6plex [N-Term; K7]; 1xCarbamidomethyl [C3	0.788	0.798		2.812403202	43.72	0	1236.702229	2	618.8547363	-0.027058858	-1.67E-05	39.22677832
																			SEEPDSITK	2	150418007	2xTMT6plex [N-Term; K9]		1.337		3.260961771	27.6	0	1463.79936	2	732.4033813	0.086074168	6.30E-05	39.18809221
																			KEGHWDCSICLVR	2	150418007	2xTMT6plex [N-Term; K1]; 2xCarbamidomethyl [C7; C10		0.562	0.928	2.696742535		1	2118.098871	4	530.2798462	-0.62115815	-0.000328919	52.5874847
15431301	RPL7	60S ribosomal protein L7	6129	NP_000962.2	41.93548387	1	13	13	139	248	29.207	10.65	1.1	1.11	0.94	1.05	Significant	0.0027																
																			IALTDNALIAR	6	15431301	1xTMT6plex [N-Term]	0.965	1.306	1.59	3.976727247	47.96	0	1399.84703	2	700.427063	-0.129023696	-9.03E-05	62.60010228
																			YGIICMEDLIHEIYTVGK	8	15431301	2xTMT6plex [N-Term; K18]; 1xCarbamidomethyl [C5	2.339	1.948	100	5.126121521	61.51	0	2612.386838	3	871.4666138	-0.593423372	-0.00051675	112.0195214
																			TTHFVEGGDAGNREDQINR	16	15431301	1xTMT6plex [N-Term]	0.914	1.136	1.116	5.35637331	25.22	1	2345.143164	4	587.0419922	1.268327685	0.000743603	30.30884052
																			TTHFVEGGDAGNR	28	15431301	1xTMT6plex [N-Term]	1.164	1.27	0.498	3.938206911	45.53	0	1589.786949	2	795.3963623	-0.944026258	-0.0007504	26.55785547
																			KTTHFVEGGDAGNREDQINR	16	15431301	2xTMT6plex [N-Term; K1]	0.922	0.899	0.841	5.272515774	22.02	2	2702.401059	5	541.2856445	-0.719091544	-0.000388655	30.41872886
																			IVEPYIAWGYPNLK	6	15431301	2xTMT6plex [N-Term; K14]	1.365	1.066		3.487057686	34.07	0	2121.214869	2	1061.110474	-0.565036759	-0.000599282	99.4978002
																			EVPAVPETLK	12	15431301	2xTMT6plex [N-Term; K10]	1.167	1.118	1.222	3.371179819	45.52	0	1540.935066	2	770.9710083	-0.211396602	-0.000162874	63.34030091
																			AGNFYVPAEPK	6	15431301	2xTMT6plex [N-Term; K11]	2.236	100	2.787	2.966368675	36.82	0	1650.925564	2	825.9642334	-2.649115879	-0.002186747	49.85053756
																			EANNFLWPFK	14	15431301	2xTMT6plex [N-Term; K10]	1.286	100	1.201	2.86511898	29.54	0	1723.957198	2	862.4818726	-0.423248798	-0.000364831	86.13540333
																			KAGNFYVPAEPK	3	15431301	3xTMT6plex [N-Term; K1; K12]		1.384	1.076	3.838106394	31.42	1	2008.183459	3	670.0662231	0.327437013	0.000219185	53.00797213
																			SVNELIYK	12	15431301	2xTMT6plex [N-Term; K8]	1.24	1.177	1.292	4.084879398	42.85	0	1423.856087	2	712.430481	-1.68690218	-0.001200953	50.37579257
																			EVPAVPETLKK	10	15431301	3xTMT6plex [N-Term; K10; K11]	0.886	0.77	0.698	3.496253252	17.85	1	1898.192961	3	633.402771	0.420952083	0.000266349	51.30367972
																			KTTHFVEGGDAGNR	2	15431301	2xTMT6plex [N-Term; K1]	1.357			2.00158906	3.52	1	1947.044844	3	649.6868286	0.559226896	0.000362947	32.41720178
19913414	AP2A1	AP-2 complex subunit alpha-1 isoform 1	160	NP_055018.2	23.64380757	1	12	22	88	977	107.478	7.03	0.92	1.46	1.24	1.21	Significant	0.016																
																			EMGEAFAADIPR	4	19913414	1xTMT6plex [N-Term]	1.357			3.796006203	47.07	0	1535.772544	2	768.390625	0.930495864	0.000714515	74.27061351
																			AADLLYAMCDR	8	19913414	1xTMT6plex [N-Term]; 1xCarbamidomethyl [C9	0.935	100		4.087017536	49.63	0	1527.749701	2	764.3778687	-0.811700576	-0.000620038	89.54701523
																			YLALESMCTLASSEFSHEAVK	8	19913414	2xTMT6plex [N-Term; K21]; 1xCarbamidomethyl [C8			100	2.854915142	15.34	0	2831.435973	3	944.4821777	-1.41031862	-0.001331076	91.8656623
																			ACNQLGQFLQHR	8	19913414	1xTMT6plex [N-Term]; 1xCarbamidomethyl [C2	1.118	0.567	1.177	4.495413303	38.16	0	1700.885223	3	567.630127	-5.524053296	-0.003131927	48.47381364
																			LEPNAQAQMYR	2	19913414	1xTMT6plex [N-Term]			1.179	3.566688538	35.88	0	1549.799428	2	775.4031982	-0.198700309	-0.000153973	46.09301448
																			DDVQGYAAK	4	19913414	2xTMT6plex [N-Term; K9]	1.08		1.395	3.887354136	63.32	0	1424.778565	2	712.8924561	-0.652480777	-0.00046482	36.02957986
																			ANHPMDAEVTK	2	19913414	2xTMT6plex [N-Term; K11]	0.843			3.695846081	41.14	0	1670.893612	3	557.635437	-1.109457138	-0.000617928	35.65980326
																			TCVSLAVSR	4	19913414	1xTMT6plex [N-Term]; 1xCarbamidomethyl [C2		1.763	1.329	3.411153793	45.84	0	1221.682273	2	611.3397217	-8.272609701	-0.00505325	43.9384206
																			NADVELQQR	10	19913414	1xTMT6plex [N-Term]	1.148	1.004	0.712	3.68661499	51.74	0	1301.701094	2	651.3540039	-0.278741027	-0.000181419	30.04954233
																			GLAVFISDIR	2	19913414	1xTMT6plex [N-Term]	0.858			3.168102026	51.33	0	1319.788453	2	660.3973999	-0.704162331	-0.000464673	96.10163646
																			ILVAGDSMDSVK	6	19913414	2xTMT6plex [N-Term; K12]	1.632	1.546	1.866	2.214015961	27.67	0	1692.960628	2	846.9824829	-1.736171319	-0.001469635	55.96323134
																			ATIQGVLR	4	19913414	1xTMT6plex [N-Term]	0.584		1.004	3.051699638	40.74	0	1086.683259	2	543.8449097	-0.659404713	-0.000358282	46.89384734
																			HLCELLAQQF	4	19913414	1xTMT6plex [N-Term]; 1xCarbamidomethyl [C3				1.573087573	24.63	0	1487.787801	2	744.3967285	-1.089166579	-0.000810224	68.28084542
																			VAILAEK	4	19913414	2xTMT6plex [N-Term; K7]		1.393	2.203	2.56475234	34.5	0	1201.79203	2	601.3995972	-0.093846572	-5.64E-05	59.93929369
																			VLQIVTNR	2	19913414	1xTMT6plex [N-Term]	1.291			2.618297815	21.42	0	1171.736023	2	586.3707886	-1.470093544	-0.000861281	60.70603338
																			FINLFPETK	2	19913414	2xTMT6plex [N-Term; K9]				2.095532417	12.13	0	1566.929586	2	783.968689	0.328565937	0.00025742	85.21771061
																			LINNAIK	4	19913414	2xTMT6plex [N-Term; K7]		1.624	1.558	3.213801384	36.23	0	1243.813829	2	622.4102783	-0.441014051	-0.00027427	46.11044332
																			QSAALCLLR	2	19913414	1xTMT6plex [N-Term]; 1xCarbamidomethyl [C6			0.71	2.52968049	15.34	0	1260.729558	2	630.8650513	-5.339662972	-0.003365935	49.63532898
																			ALQVGCLLR	2	19913414	1xTMT6plex [N-Term]; 1xCarbamidomethyl [C6				1.302313209	13.78	0	1258.750293	2	629.8789063	0.192754672	0.000121315	72.96437539
																			ALLLSTYIK	3	19913414	2xTMT6plex [N-Term; K9]		100		1.354979038	1.5	0	1479.955073	2	740.480896	-0.376759959	-0.000278794	81.92103943
																			IAGDYVSEEVWYR	1	19913414	1xTMT6plex [N-Term]	1.293			1.235443473		0	1815.911481	2	908.4581909	-1.308056085	-0.001187657	72.44662385
																			FFQPTEMAAQDFFQR	2	19913414	1xTMT6plex [N-Term]			100	1.118708611	0.26	0	2092.015962	2	1046.509277	-2.239104569	-0.002342121	88.48619651
4502443	DST	dystonin isoform 1e precursor	667	NP_001714.1	9.701774254	1	22	22	66	2649	306.564	6.32	0.9	1.1	0.94	0.98	Significant	0.0039																
																			SVQEIAEQLLLENHPAR	6	4502443	1xTMT6plex [N-Term]	0.846	100	0.562	4.035295963	64.35	0	2176.192347	3	726.0684814	-0.668839804	-0.000485175	84.34548194
																			EAMFFESYGHSSHMLTDTK	2	4502443	2xTMT6plex [N-Term; K19]	100			3.490668535	38.83	0	2676.283829	4	669.8272705	1.278904432	0.000855678	69.47705754
																			GLVDEGFAQQLR	4	4502443	1xTMT6plex [N-Term]	0.877			3.438821077	37.12	0	1561.853572	2	781.4297485	-0.865240962	-0.00067569	70.40718176
																			YAEQYSATVK	2	4502443	2xTMT6plex [N-Term; K10]	0.974			3.473919153	49.31	0	1617.888844	2	809.4477539	-0.378633861	-0.000306294	34.53632792
																			YYQADQLVQR	4	4502443	1xTMT6plex [N-Term]	1.498		1.11	3.633483887	35	0	1512.800808	2	756.902832	-1.600063595	-0.001210289	42.9803696
																			TMLPGEHQQVLSNLQSR	4	4502443	1xTMT6plex [N-Term]	1	0.986		4.435422897	19.66	0	2167.149102	3	723.0542603	-0.403344782	-0.000291369	57.97624771
																			AIEEFESSLK	4	4502443	2xTMT6plex [N-Term; K10]	0.698	0.763		2.659338236	36.77	0	1610.904159	2	805.9568481	1.403062404	0.0011301	73.36769126
																			SSDSVFSNTTSTR	2	4502443	1xTMT6plex [N-Term]	100			2.853281736	21.38	0	1617.791759	2	809.4000244	0.626099199	0.000506449	38.3030859
																			KIQENQPENSK	2	4502443	3xTMT6plex [N-Term; K1; K11]			0.968	4.349477768	11.1	1	2002.153615	3	668.0559692	-0.130112472	-8.68E-05	33.64984951
																			QYDMHTEVTTLK	6	4502443	2xTMT6plex [N-Term; K12]	0.777	0.804	0.452	3.634178162	21.68	0	1924.02502	3	642.0152588	3.224270539	0.002067859	34.50546134
																			QQVDELTAANR	2	4502443	1xTMT6plex [N-Term]		1.259		3.951401949	32.45	0	1473.785886	2	737.3977661	1.607707459	0.001184708	40.7025058
																			ILTTEQTK	4	4502443	2xTMT6plex [N-Term; K8]		1.132	1.015	3.594571352	38.42	0	1391.851002	2	696.4302368	1.577189519	0.001097606	40.34182648
																			ADFDFHTGLK	2	4502443	2xTMT6plex [N-Term; K10]	0.871			2.640938759	14.61	0	1608.878613	3	536.9645996	0.392874963	0.000210696	58.85045633
																			ASNVASIK	2	4502443	2xTMT6plex [N-Term; K8]	0.454			2.600841761	39.48	0	1247.772358	2	624.3913574	2.468866802	0.001540292	37.74715903
																			WTQEPQPLEEK	2	4502443	2xTMT6plex [N-Term; K11]				2.156211138	18.55	0	1843.000185	2	921.9975586	-6.698025643	-0.006172231	51.48238556
																			EQMQQGQHMEANHYQK	1	4502443	2xTMT6plex [N-Term; K16]	1.292			1.784680069		0	2445.180368	4	612.0509644	0.678891381	0.000415003	36.95848988
																			INEEIEK	8	4502443	2xTMT6plex [N-Term; K7]	0.631	1.015		2.979470015	36.31	0	1332.777503	2	666.8921509	-0.358193462	-0.000238696	53.06544247
																			ELTYELNALQLEK	1	4502443	2xTMT6plex [N-Term; K13]				1.400922298		0	2022.152328	2	1011.579163	-0.632931886	-0.000639942	76.88166767
																			LTIEAYR	6	4502443	1xTMT6plex [N-Term]	0.894	1.181	1.432	2.520578861	23.16	0	1094.640726	2	547.8240967	0.174467632	9.55E-05	53.97489739
																			VVEQIPK	2	4502443	2xTMT6plex [N-Term; K7]	1.04			2.776000261	31.02	0	1270.813494	2	635.9098511	-0.840927144	-0.000534331	54.22218798
																			LLEVSEPLMTGISSLYYSS	2	4502443	1xTMT6plex [N-Term]				0.918774307	2.14	0	2318.203882	2	1159.60437	-1.043219556	-0.001209198	110.5132347
																			MLVSEIEMK	2	4502443	2xTMT6plex [N-Term; K9]				1.127480865	0.26	0	1537.873393	2	769.4398804	-0.591321637	-0.000454689	65.69928022
4503607	ETFA	electron transfer flavoprotein subunit alpha, mitochondrial isoform a	2108	NP_000117.1	48.64864865	1	12	12	76	333	35.058	8.38	0.76	0.79	0.75	0.77	Significant	0.00025																
																			AAVDAGFVPNDMQVGQTGK	12	4503607	2xTMT6plex [N-Term; K19]	0.685	0.78	0.7	3.528484821	35.32	0	2363.242951	3	788.4187012	-0.592721275	-0.000466915	62.07328485
																			ASSTSPVEISEWLDQK	12	4503607	2xTMT6plex [N-Term; K16]	1.093			3.101750851	43.96	0	2235.190899	3	745.7349854	-0.221844726	-0.000165288	94.11531919
																			GLLPEELTPLILATQK	11	4503607	2xTMT6plex [N-Term; K16]	0.651	0.01	2.939	3.760590792	39.48	0	2194.346277	3	732.1206055	0.449325733	0.000328659	104.8804219
																			LLYDLADQLHAAVGASR	6	4503607	1xTMT6plex [N-Term]	0.69	0.951	0.644	4.098881245	49.86	0	2042.123205	3	681.3786621	-0.867446132	-0.000590477	101.7572958
																			TIYAGNALCTVK	2	4503607	2xTMT6plex [N-Term; K12]; 1xCarbamidomethyl [C9		0.914		3.514418602	63.9	0	1769.003162	2	885.0048828	-0.380607008	-0.000336648	63.17323755
																			GTSFDAAATSGGSASSEK	8	4503607	2xTMT6plex [N-Term; K18]	0.808	0.741	0.903	4.966082096	77.82	0	2089.044963	2	1045.026123	0.00322815	3.37E-06	45.78988645
																			VLVAQHDVYK	8	4503607	2xTMT6plex [N-Term; K10]	0.765	0.828	0.728	4.49180603	59.84	0	1629.972848	3	543.9954224	-0.695950339	-0.000378127	42.63490077
																			DPEAPIFQVADYGIVADLFK	2	4503607	2xTMT6plex [N-Term; K20]		0.429		2.627604723	14.85	0	2666.448176	3	889.4837036	-4.357208312	-0.003872757	109.9507939
																			TIVAINKDPEAPIFQVADYGIVADLFK	4	4503607	3xTMT6plex [N-Term; K7; K27]				3.280320406	2.96	1	3635.070332	4	909.5233154	0.302538507	0.000274937	104.86581
																			SDRPELTGAK	4	4503607	2xTMT6plex [N-Term; K10]	0.732	0.69		2.82839942	23.18	0	1531.884427	3	511.2993164	-0.673126008	-0.000343717	33.71099018
																			VVVSGGR	4	4503607	1xTMT6plex [N-Term]		0.813	0.762	2.550564289	25.19	0	902.5620816	2	451.7847595	0.178184938	8.04E-05	30.07515199
																			VVPEMTEILK	3	4503607	2xTMT6plex [N-Term; K10]	0.646		0.785	2.947626114	26.96	0	1616.969737	2	808.989624	1.38206475	0.001117378	79.62631746
119395758	DIAPH1	protein diaphanous homolog 1 isoform 1	1729	NP_005210.3	17.45283019	1	20	20	71	1272	141.258	5.41	0.89	1.2	1.41	1.17	Significant	0.016																
																			EQLIDMNAEGDETGVMDSLLEALQSGAAFR	3	119395758	1xTMT6plex [N-Term]			0.694	6.054899216	68.81	0	3439.655865	3	1147.222656	-0.712170082	-0.00081654	112.1383859
																			KLSVEEFFMDLHNFR	2	119395758	2xTMT6plex [N-Term; K1]	1.075			3.50835371	25.55	1	2370.268044	4	593.3225098	0.069841257	4.14E-05	101.1691838
																			DVQNFPAATDEKDK	2	119395758	3xTMT6plex [N-Term; K12; K14]			1.894	4.246397972	14.78	1	2265.232987	3	755.744873	-5.704256228	-0.004307156	41.8746574
																			MMHSNMETLYK	4	119395758	2xTMT6plex [N-Term; K11]	1.068	1.062		4.654926777	43.86	0	1842.931653	3	614.9822998	0.375941268	0.000230945	46.94150195
																			EMVSQYLYTSK	4	119395758	2xTMT6plex [N-Term; K11]	1.779	100		3.251138926	25.23	0	1806.971193	2	903.9896851	0.498274234	0.000450184	66.36139721
																			ELGEYFLFDPK	4	119395758	2xTMT6plex [N-Term; K11]	1.479	100		2.004081011	21.04	0	1815.993309	2	908.5001221	-0.187929864	-0.00017064	92.44933971
																			LVAEDLSQDCFWTK	4	119395758	2xTMT6plex [N-Term; K14]; 1xCarbamidomethyl [C10				1.823877811	9.54	0	2170.125462	2	1085.565063	-1.203495798	-0.001305868	102.0012231
																			DAQEQYNK	5	119395758	2xTMT6plex [N-Term; K8]	1.763	1.313		3.591803551	34.61	0	1453.768729	2	727.3885498	0.7527259	0.000547145	28.00519985
																			KLDSELTAR	4	119395758	2xTMT6plex [N-Term; K1]		1.394	1.27	2.688643456	16.88	1	1490.894264	3	497.6364136	0.284341195	0.000141308	40.90413934
																			EETAGSYDSR	2	119395758	1xTMT6plex [N-Term]				1.99330759	36.66	0	1343.627654	2	672.3175049	0.05871837	3.94E-05	22.76672903
																			AEMDEVER	4	119395758	1xTMT6plex [N-Term]	1.174	1.214		3.408712626	40	0	1207.582618	2	604.2937622	-1.963125257	-0.001185318	32.25899002
																			SAMMYIQELR	4	119395758	1xTMT6plex [N-Term]	0.79			2.080068588	19.55	0	1470.764622	2	735.8862305	0.382139665	0.000281019	82.28849977
																			VLEAMTER	8	119395758	1xTMT6plex [N-Term]	0.709	1.368	1.279	2.987036943	40.4	0	1177.644825	2	589.3266602	1.035017073	0.000609441	50.00351008
																			LNAILFK	4	119395758	2xTMT6plex [N-Term; K7]	1.638	1.241		2.842590332	37.28	0	1276.839315	2	638.9228516	-0.695894142	-0.000444272	80.84903438
																			LIEECISQIVLHK	2	119395758	2xTMT6plex [N-Term; K13]; 1xCarbamidomethyl [C5		100		1.906762242	15.44	0	2040.192754	3	680.7344971	-1.870357129	-0.001271963	91.39743212
																			EIENEDMR	2	119395758	1xTMT6plex [N-Term]	1.156			2.484454155	25.61	0	1264.604082	2	640.8033447	0.324623966	0.000207857	28.72413353
																			EIENEDMR	2	119395758	1xTMT6plex [N-Term]; 1xOxidation [M7]	1.475			1.933045149	29.6	0	1280.598997	2	640.8033447	0.324623966	0.000207857	28.72413353
																			FPDELAHVEK	4	119395758	2xTMT6plex [N-Term; K10]	1.247	2.532		2.641855001	21.9	0	1642.920478	3	548.3118286	0.276531853	0.00015144	52.16808268
																			QMPEPEQLK	2	119395758	2xTMT6plex [N-Term; K9]	0.846			3.199948788	17.14	0	1557.871085	2	779.4387817	-0.512509312	-0.000399212	50.34623668
																			LYKPEVQLR	3	119395758	2xTMT6plex [N-Term; K3]		1.933	1.868	2.164093018	5.43	0	1603.993584	3	535.3364258	0.710983985	0.000380138	50.41927418
																			EKDIIIK	4	119395758	3xTMT6plex [N-Term; K2; K7]	0.712			2.431831598	9.52	1	1546.018291	3	516.0111084	0.311105836	0.000160325	46.63788445
156071459	SLC25A5	ADP/ATP translocase 2	292	NP_001143.2	45.30201342	1	6	16	122	298	32.831	9.69	0.99	0.99	1	1	Significant	0.000011																
																			DFLAGGVAAAISK	19	156071459	2xTMT6plex [N-Term; K13]	1.243	0.804	100	4.466392994	66.78	0	1677.993978	2	839.4998779	-0.892911801	-0.00074915	90.72780073
																			LLLQVQHASK	24	156071459	2xTMT6plex [N-Term; K10]	1.093	1.118	1.078	4.600649834	62.09	0	1595.004483	2	798.0054321	-0.561172208	-0.000447536	56.80001662
																			AAYFGIYDTAK	4	156071459	2xTMT6plex [N-Term; K11]		1.593	100	4.610656738	66.95	0	1677.925229	2	839.4656372	-0.733945659	-0.000615753	72.95003215
																			GMGGAFVLVLYDEIK	4	156071459	2xTMT6plex [N-Term; K15]		1.309		3.785112858	44.77	0	2070.170955	2	1035.588257	-0.83008996	-0.000859214	116.5761027
																			GMGGAFVLVLYDEIKK	4	156071459	3xTMT6plex [N-Term; K15; K16]			0.738	5.55839777	41.86	1	2427.42885	3	809.8138428	-0.772574523	-0.000625123	103.8980395
																			QIFLGGVDK	6	156071459	2xTMT6plex [N-Term; K9]	1.34	2.343	1.377	3.379742146	46.05	0	1434.872072	2	717.9408569	1.648660696	0.001182809	45.95248601
																			GTDIMYTGTLDCWR	6	156071459	1xTMT6plex [N-Term]; 1xCarbamidomethyl [C12	1.088	0.911	100	2.08856988	25.38	0	1917.903635	2	959.4553223	-0.139271205	-0.000133554	75.11820304
																			EQGVLSFWR	11	156071459	1xTMT6plex [N-Term]	1.235	1.527	0.958	3.309967995	39.53	0	1350.736751	2	675.8718262	-0.278067691	-0.000187798	67.36787463
																			GLGDCLVK	6	156071459	2xTMT6plex [N-Term; K8]; 1xCarbamidomethyl [C5	1.054	0.666	0.794	3.284594774	43.17	0	1319.775729	2	660.3927612	1.90719596	0.001258535	52.41706808
																			GAWSNVLR	6	156071459	1xTMT6plex [N-Term]	1.193	1.038	1.233	3.101995945	41.19	0	1131.647208	2	566.3270264	-0.381758221	-0.000216008	48.52633178
																			GMGGAFVLVLYDEIKK	4	156071459	3xTMT6plex [N-Term; K15; K16]; 1xOxidation [M2			0.797	3.442583799	11.02	1	2443.423765	3	809.8138428	-0.772574523	-0.000625123	103.8980395
																			QIFLGGVDKR	6	156071459	2xTMT6plex [N-Term; K9]	0.783	0.696	0.903	4.609538078	28.24	1	1590.973183	3	530.9959717	0.112634873	5.97E-05	46.96725051
																			GNLANVIR	4	156071459	1xTMT6plex [N-Term]	1.148	1.201		2.589091778	58.69	0	1085.662858	2	543.3349609	-0.196133632	-0.000106467	52.83431799
																			YFPTQALNFAFK	4	156071459	2xTMT6plex [N-Term; K12]	1.745	1.049		2.621410847	14.25	0	1905.067477	2	953.0360107	-1.434041397	-0.001365973	101.2922915
																			GTDIMYTGTLDCWR	6	156071459	1xTMT6plex [N-Term]; 1xCarbamidomethyl [C12]; 1xOxidation [M5]		100	100	1.573530197	12.03	0	1933.89855	2	959.4553223	-0.139271205	-0.000133554	75.11820304
																			GIIDCVVR	4	156071459	1xTMT6plex [N-Term]; 1xCarbamidomethyl [C5	1.092	0.512		2.453022718	32.5	0	1160.665895	2	580.8362427	-0.591228225	-0.000343109	55.60796221
																			KGTDIMYTGTLDCWR	3	156071459	2xTMT6plex [N-Term; K1]; 1xCarbamidomethyl [C13		1.702		2.324502707	5.63	1	2275.16153	3	759.057373	-1.742333159	-0.001321363	57.67905106
																			LAADVGK	7	156071459	2xTMT6plex [N-Term; K7]	0.899	0.956	0.902	2.867013931	36.39	0	1131.71378	2	566.3599243	-1.067573099	-0.000604094	39.55778558
124256496	HSPA1L	heat shock 70 kDa protein 1-like	3305	NP_005518.3	28.23712949	1	2	15	170	641	70.331	6.02	0.51		0.01	0.26	Non-Significan	#N/A																
																			SINPDEAVAYGAAVQAAILMGDK	17	124256496	2xTMT6plex [N-Term; K23]	1.141	100	1.246	4.268444061	46.61	0	2762.479887	3	921.4978638	-0.307166546	-0.000282847	103.8219879
																			ATAGDTHLGGEDFDNR	12	124256496	1xTMT6plex [N-Term]	0.833	0.876	0.851	4.630966663	42.42	0	1904.893598	3	635.637146	1.725155822	0.001095413	25.28270071
																			IINEPTAAAIAYGLDK	31	124256496	2xTMT6plex [N-Term; K16]	1.572	1.737	2.328	4.13507843	46.05	0	2118.221077	2	1059.61499	0.768167576	0.000813574	75.35766658
																			DAGVIAGLNVLR	10	124256496	1xTMT6plex [N-Term]	0.861	0.989		4.103200912	66.89	0	1426.857929	2	713.932312	-0.407606497	-0.000290798	81.84942328
																			VEILANDQGNR	48	124256496	1xTMT6plex [N-Term]	1.325	1.311	1.289	4.071812153	54.4	0	1457.790972	2	729.3981323	-1.360662537	-0.000991781	36.49181231
																			TTPSYVAFTDTER	12	124256496	1xTMT6plex [N-Term]	1.291	1.404		4.477664471	71.19	0	1716.864196	2	858.9349365	-0.931671316	-0.000799777	51.60226222
																			SINPDEAVAYGAAVQAAILMGDK	13	124256496	2xTMT6plex [N-Term; K23]; 1xOxidation [M20			1.826	2.541149616	26.48	0	2778.474802	3	921.4978638	-0.307166546	-0.000282847	103.8219879
																			FEELCADLFR	2	124256496	1xTMT6plex [N-Term]; 1xCarbamidomethyl [C5			0.01	3.005153179	26.92	0	1528.766731	2	764.8865967	-0.532645438	-0.000407145	95.573143
																			AFYPEEISSMVLTK	10	124256496	2xTMT6plex [N-Term; K14]	0.866	0.93	0.752	3.058972836	26.75	0	2073.134236	2	1037.070313	-0.427955887	-0.000443605	87.71425392
																			GVPQIEVTFDIDANGILNVTATDK	2	124256496	2xTMT6plex [N-Term; K24]			100	2.650573015	23.83	0	2988.629387	3	996.8739014	-7.440396769	-0.007412196	115.3000616
																			YKAEDEVQR	14	124256496	2xTMT6plex [N-Term; K2]	0.724	0.793	0.743	2.897931337	15.46	1	1595.879342	2	798.4438477	0.674845818	0.000538486	32.73629936
																			NALESYAFNMK	2	124256496	2xTMT6plex [N-Term; K11]		0.01		1.596866608	6.25	0	1745.929663	2	873.4682007	-0.308129717	-0.000268986	63.63010502
																			GTLEPVEK	2	124256496	2xTMT6plex [N-Term; K8]	0.511			3.294013977	23.46	0	1330.798238	2	665.9041138	2.038572779	0.001356465	41.85083847
																			LIGDAAK	8	124256496	2xTMT6plex [N-Term; K7]	1.088	1.128	0.991	2.640163898	25.21	0	1145.72943	2	573.368042	-0.543667299	-0.000311448	38.40743522
																			ITITNDK	9	124256496	2xTMT6plex [N-Term; K7]	1.307	1.284	1.082	3.007526398	39.15	0	1262.772023	2	631.8894043	-0.389061716	-0.000245648	34.74030574
																			AEDEVQR	2	124256496	1xTMT6plex [N-Term]			0.728	2.401926279	22.94	0	1075.558118	2	538.2827148	0.032334375	1.74E-05	23.02407717
217272892	EFTUD2	116 kDa U5 small nuclear ribonucleoprotein component isoform a	9343	NP_004238.3	27.36625514	1	18	19	60	972	109.366	5	0.88	1.11	1.2	1.06	Significant	0.0079																
																			GHVTQDAPIPGSPLYTIK	2	217272892	2xTMT6plex [N-Term; K18]	1.17			5.453670979	72.22	0	2352.332752	3	784.7818604	-0.733108575	-0.000574838	59.70423179
																			GLSEDVSISK	4	217272892	2xTMT6plex [N-Term; K10]		1.177	0.985	4.389397144	59.44	0	1492.862295	2	746.9343262	-0.615573354	-0.000459483	46.82694891
																			LMEPYYFVEVQAPADCVSAVYTVLAR	2	217272892	1xTMT6plex [N-Term]; 1xCarbamidomethyl [C16			0.963	3.387854338	13.18	0	3220.62601	3	1074.212158	-1.269380739	-0.001362734	106.3744639
																			VLSGTIHAGQPVK	4	217272892	2xTMT6plex [N-Term; K13]		1.464	1.368	3.625695467	23.36	0	1765.073625	3	589.0292358	-0.266584046	-0.000156847	49.88697004
																			ILDAVVAQEPLHR	2	217272892	1xTMT6plex [N-Term]		1.966		2.749413252	39.39	0	1689.98492	3	563.9995117	-0.555273446	-0.000312801	73.2020674
																			SFVEFILEPLYK	4	217272892	2xTMT6plex [N-Term; K12]	2.045	1.006		3.497104645	31.79	0	1943.129408	2	972.0673828	-0.987739155	-0.000959652	113.2913093
																			RYDQDLCYTDILFTEQER	2	217272892	1xTMT6plex [N-Term]; 1xCarbamidomethyl [C7	1.481			4.084206581	16.19	1	2594.239433	3	865.4195557	1.804344706	0.001560301	77.4207877
																			GNEEAQIFRPLK	4	217272892	2xTMT6plex [N-Term; K12]	1.074		0.526	2.279075623	14.82	0	1860.074353	3	620.6962891	-0.021022009	-1.30E-05	55.66524789
																			YDWDLLAAR	4	217272892	1xTMT6plex [N-Term]	1.22	2.16		2.9641366	32.65	0	1351.720767	2	676.3637085	-0.463496476	-0.000313259	83.03485293
																			NVTLCGHLHHGK	2	217272892	2xTMT6plex [N-Term; K12]; 1xCarbamidomethyl [C5		0.551		2.353522778	2.66	0	1831.016127	4	458.5099182	0.93714716	0.000428983	31.34147212
																			GLAEDIENEVVQITWNR	3	217272892	1xTMT6plex [N-Term]				1.566300035	12.09	0	2215.155627	2	1108.078979	-2.232057904	-0.002472178	105.443779
																			DSIVQGFQWGTR	4	217272892	1xTMT6plex [N-Term]	100	100		2.686424971	33.66	0	1622.848821	2	811.9283447	0.364811015	0.000296017	78.58037913
																			SYPSLTTK	6	217272892	2xTMT6plex [N-Term; K8]	1.065	1.185	1.192	3.479939699	36.35	0	1354.798238	2	677.9028931	0.200415682	0.000135761	44.17142123
																			TLDELGIHLTK	4	217272892	2xTMT6plex [N-Term; K11]		0.825	100	2.259111404	22.69	0	1698.020192	3	566.6773682	-1.55535124	-0.000880339	62.24213513
																			GGGQIIPTAR	2	217272892	1xTMT6plex [N-Term]	2.065			2.908091545	32.06	0	1198.710537	2	599.8590088	0.170481629	0.000102179	39.39309849
																			TCFVDCLIEQTHPEIR	2	217272892	1xTMT6plex [N-Term]; 2xCarbamidomethyl [C2; C6	100			2.317787647	17.57	0	2247.10994	3	749.708252	0.117070096	8.77E-05	78.63917988
																			AFIPAIDSFGFETDLR	3	217272892	1xTMT6plex [N-Term]	100	100	1.207	1.187977552		0	2028.063958	2	1014.534363	-1.237319983	-0.001254682	94.53024059
																			SIWAFGPDATGPNILVDDTLPSEVDK	2	217272892	2xTMT6plex [N-Term; K26]				1.324017644		0	3215.687631	3	1072.563843	-3.313580457	-0.003551813	99.56843472
																			ALLGSVK	4	217272892	2xTMT6plex [N-Term; K7]	0.77		0.833	2.689048767	27.14	0	1145.765816	2	573.3857422	-1.40344997	-0.000804012	51.75132916
4502643	CCT6A	T-complex protein 1 subunit zeta isoform a	908	NP_001753.1	40.48964218	1	17	17	92	531	57.988	6.68	1.23	1.28	1.19	1.23	Significant	0.00045																
																			DGNVLLHEMQIQHPTASLIAK	4	4502643	2xTMT6plex [N-Term; K21]	1.211	100		4.057944775	18.02	0	2773.54349	4	694.1426392	1.888129984	0.001309203	69.93987688
																			AQLGVQAFADALLIIPK	4	4502643	2xTMT6plex [N-Term; K17]		1.55		3.463075638	46.06	0	2226.362596	2	1113.681396	-3.179909356	-0.003539816	116.8190801
																			QADLYISEGLHPR	8	4502643	1xTMT6plex [N-Term]	1.259	1.341		4.517929554	65.09	0	1727.927799	3	576.6466675	-1.360090737	-0.00078338	50.19036629
																			IITEGFEAAK	12	4502643	2xTMT6plex [N-Term; K10]	1.226	1.385	1.31	4.662745476	66.9	0	1536.903766	2	768.956665	1.488608575	0.001143924	64.37581776
																			MLVSGAGDIK	4	4502643	2xTMT6plex [N-Term; K10]	1.131	1.239		3.324973106	58.07	0	1448.854707	2	724.9315796	0.811378589	0.000587785	58.88598053
																			VHAELADVLTEAVVDSILAIK	2	4502643	2xTMT6plex [N-Term; K21]			100	2.583815575	16.15	0	2664.558789	3	888.8563843	-1.572229514	-0.001396433	113.8823897
																			KQDEPIDLFMIEIMEMK	4	4502643	3xTMT6plex [N-Term; K1; K17]		1.801	1.039	2.669579268	25.73	1	2797.515693	3	933.1738281	-3.131912726	-0.002920525	106.1831752
																			GIDPFSLDALSK	10	4502643	2xTMT6plex [N-Term; K12]	1.746	1.367	0.634	2.736771345	39.58	0	1720.988558	2	860.9978638	-0.062086954	-5.34E-05	79.29535001
																			ALQFLEEVK	8	4502643	2xTMT6plex [N-Term; K9]	2.651	1.36	1.061	3.588950157	47.78	0	1534.924501	2	767.9663696	0.626459355	0.000480784	69.69088362
																			GFVVINQK	8	4502643	2xTMT6plex [N-Term; K8]	1.182	1.102	1.466	3.803432703	48.62	0	1362.850942	2	681.9309692	2.729239455	0.001859773	54.51331597
																			VATAQDDITGDGTTSNVLIIGELLK	4	4502643	2xTMT6plex [N-Term; K25]				2.186394691	17.47	0	3002.666167	3	1001.558594	-1.644811553	-0.001646273	100.7701392
																			VLAQNSGFDLQETLVK	6	4502643	2xTMT6plex [N-Term; K16]	100	100	100	2.148735523	10.39	0	2220.264004	2	1110.634521	-1.007867193	-0.001118866	78.71231278
																			TEVNSGFFYK	6	4502643	2xTMT6plex [N-Term; K10]				1.806488514	18.18	0	1649.893929	2	825.4492188	-1.677865438	-0.00138415	58.83581696
																			ETLIDVAR	2	4502643	1xTMT6plex [N-Term]	1.297			2.966023207	31.48	0	1145.672754	2	573.3400269	0.020058902	1.15E-05	54.47966944
																			SVTLLIK	6	4502643	2xTMT6plex [N-Term; K7]	1.318	1.157	0.772	2.79991293	39.98	0	1231.838981	2	616.4224243	-1.14354815	-0.000704334	72.07302782
																			EMDRETLIDVAR	2	4502643	1xTMT6plex [N-Term]			1.151	2.50767374	2.58	1	1676.883886	3	559.6325684	-0.437580611	-0.000244591	52.56771706
																			GLQDVLR	2	4502643	1xTMT6plex [N-Term]	1.013			2.519158125	27.22	0	1029.62541	2	515.315979	-0.707715816	-0.000364341	54.69752353
4507949	YWHAB	14-3-3 protein beta/alpha	7529	NP_003395.1	55.28455285	1	5	11	211	246	28.065	4.83	1.26	1.18	1.21	1.22	Significant	0.00037																
																			QTTVSNSQQAYQEAFEISK	7	4507949	2xTMT6plex [N-Term; K19]	1.515		100	4.854146004	55.06	0	2617.350981	3	873.1229248	1.237982252	0.001080078	90.47265715
																			AVTEQGHELSNEER	30	4507949	1xTMT6plex [N-Term]	1.081	1.054	1.094	5.924292088	62.96	0	1827.903435	2	914.4563599	1.098622895	0.001004088	31.83391547
																			TAFDEAIAELDTLNEESYK	14	4507949	2xTMT6plex [N-Term; K19]	1.812	2.717	6.439	4.686876297	47.4	0	2617.328514	3	873.1152344	1.007036368	0.000878582	102.8490966
																			YLSEVASGDNK	4	4507949	2xTMT6plex [N-Term; K11]	1.634	1.42		5.041928291	62.12	0	1640.889572	2	820.9488525	0.521941354	0.000428224	45.82320102
																			DSTLIMQLLR	37	4507949	1xTMT6plex [N-Term]	1.27	1.271	1.324	4.110405922	66.2	0	1418.823852	2	709.9172974	2.443154983	0.001733203	90.31582375
																			YDDMAAAMK	12	4507949	2xTMT6plex [N-Term; K9]	1.054	1.309	1.151	4.237743855	54.62	0	1473.748193	2	737.3778076	0.098791894	7.28E-05	52.30776316
																			VISSIEQK	56	4507949	2xTMT6plex [N-Term; K8]	1.111	0.959	0.957	3.934776306	49.24	0	1361.840437	2	681.4239502	0.137042946	9.33E-05	59.72555441
																			NLLSVAYK	24	4507949	2xTMT6plex [N-Term; K8]	0.874	0.882	0.72	3.972506762	46.76	0	1365.850608	2	683.4294434	0.733725009	0.000501079	92.81997229

Bhat et al,2017. Cigarette smoke and chewing tobacco alter expression of different sets of miRNAs in oral keratinocytes

Supplementary Table S2(A). List of all proteins and peptides identified in OKF6/TERT1 cells chronically treated with chewing tobacco using Sequest and Mascot search algorithms

Accession	Gene_Symbol	Description	Gene_ID	Refseq_Accession	Coverage	Proteins	#Unique_Peptides	#Peptides	#PSMS	#AAs	MW [kDa]	calc. pI	OKF6-STE-6M/OKF6P_R1	OKF6-STE-6M/OKF6P_R2	OKF6-STE-6M/OKF6P_R3	Median fold change	Status	P-value (6M)	Sequence	#PSMs	Protein Accessions	Modifications	OKF6-STE-6M/OKF6P_R1	OKF6-STE-6M/OKF6P_R2	OKF6-STE-6M/OKF6P_R3	Xcorr	Ionscore	# Missed Cleavages	Theo. MH+ [Da]	Charge	m_z	DeltaM [ppm]	Deltam/z [Da]	RT [min]
																			YDDMAAAMK	25	4507949	2xTMT6plex [N-Term; K9]; 1xOxidation [M]	1.215	0.88	1.298	2.709822893	42.15	0	1489.743108	2	737.3778076	0.098791894	7.28E-05	52.30776316
																			DNLTLWTSENQGDEGDAGEGEN	6	4507949	1xTMT6plex [N-Term]	1.41			2.251163483	19.97	0	2580.117128	3	860.7113037	0.864175036	0.000743224	70.91676967
																			DSTLIMQLLR	14	4507949	1xTMT6plex [N-Term]; 1xOxidation [M6]	1.102	100		1.706976891	33.93	0	1434.818767	2	709.9172974	2.443154983	0.001733203	90.31582375
																			EMQPTHPIR	4	4507949	1xTMT6plex [N-Term]; 1xOxidation [M2]	0.564		100	2.449209452	23.08	0	1353.714636	3	451.908905	-1.827720587	-0.000824737	18.46995257
																			LAEQAER	6	4507949	1xTMT6plex [N-Term]	1.208	0.935	1.021	2.81289196	25.93	0	1045.583939	2	523.2957764	0.322273863	0.000168482	26.08720983
4507651	TPM4	tropomyosin alpha-4 chain isoform Tpm4.2cy	7171	NP_003281.1	49.59677419	1	4	14	122	248	28.504	4.69	0.93	1.04	0.98	0.98	Significant	0.001																
																			IQALQQQADEAEDR	4	4507651	1xTMT6plex [N-Term]	0.996	1.696		4.554738522	74.45	0	1843.934735	2	922.4708862	-0.12972751	-0.000119605	56.4008338
																			KIQALQQQADEAEDR	7	4507651	2xTMT6plex [N-Term; K1]	0.334	1.073	0.989	7.641087055	46.97	1	2201.19263	3	734.4031982	1.095527275	0.000803822	50.15262824
																			EKAEGDVAALNR	14	4507651	2xTMT6plex [N-Term; K2]	0.993	1	0.971	3.843165398	46.39	1	1730.980118	2	865.9940186	0.370991768	0.00032109	38.28920085
																			IQLVEEELDR	18	4507651	1xTMT6plex [N-Term]	1.297	2.38	1.828	3.785353422	49.84	0	1472.815789	2	736.911499	-0.046111079	-3.40E-05	68.55083794
																			AEGDVAALNR	6	4507651	1xTMT6plex [N-Term]	0.948	1.026	0.982	4.27511692	56.26	0	1244.67963	2	622.8432007	-0.406203171	-0.000252796	35.2050086
																			KLVILEGELER	15	4507651	2xTMT6plex [N-Term; K1]	0.853	0.956	0.684	4.428694725	31.82	1	1757.093692	3	586.3692017	-0.364123743	-0.000213267	59.71334727
																			EENVGLHQTLDQTLNELNCI	15	4507651	1xTMT6plex [N-Term]; 1xCarbamidomethyl [C19	100	1.513	0.707	3.414426565	22.44	0	2569.276547	3	857.0984497	1.653882335	0.001416427	85.66828029
																			MEIQEMQLK	20	4507651	2xTMT6plex [N-Term; K9]	1.324	0.962		3.55449605	46.32	0	1607.890106	2	804.4488525	0.200447856	0.000161149	72.61134951
																			SLEAASEK	6	4507651	2xTMT6plex [N-Term; K8]	1.099	0.859	1.023	3.799231529	49.52	0	1292.746202	2	646.8760376	-1.085932169	-0.000701917	34.71213245
																			YSEKEDKYEEEIK	4	4507651	4xTMT6plex [N-Term; K4; K7; K13]	1.247	1.244		4.074170113	13.75	2	2606.437115	2	1303.714355	-6.016290363	-0.007840541	43.42246357
																			CGDLEEELK	2	4507651	2xTMT6plex [N-Term; K9]; 1xCarbamidomethyl [C1	1.337			2.410672665	15.73	0	1550.81363	2	775.9111938	0.954876385	0.000740418	56.19358147
																			TIDDLEEK	4	4507651	2xTMT6plex [N-Term; K8]	0.951		1.374	3.846661568	22.09	0	1420.793546	2	710.8937988	-9.308469751	-0.006612707	46.8645142
																			EDKYEEEIK	13	4507651	3xTMT6plex [N-Term; K3; K9]	1.54	1.287	1.151	2.958414555	24.3	1	1870.041271	2	935.5215454	-2.917801748	-0.002728205	47.94373045
																			HIAEEADR	4	4507651	1xTMT6plex [N-Term]	100	1.038		2.72195363	28.11	0	1169.611216	2	585.3092651	0.03179128	1.86E-05	20.53571929
35493916	RPN2	dolichyl-diphosphooligosaccharide--protein glycosyltransferase subunit 2 isoform 1 precurso	6185	NP_002942.2	36.76703645	1	15	15	78	631	69.241	5.69	1.59	1.41	1.67	1.55	Significant	0.0024																
																			LQVTNVLSQPLTQATVK	6	35493916	2xTMT6plex [N-Term; K17]			100	2.894384861	39.74	0	2298.379702	2	1149.692383	-0.96306759	-0.001106747	71.16746753
																			SNLDPSNVDSLFYAAQASQALSGCEISISNETK	11	35493916	2xTMT6plex [N-Term; K33]; 1xCarbamidomethyl [C24	1.841	2.316	1.983	4.99356842	45.45	0	3974.969361	4	994.4976807	-0.117760342	-0.000117023	95.27936893
																			SIVEEIEDLVAR	8	35493916	1xTMT6plex [N-Term]	1.635	1.707		4.325984001	62.33	0	1601.894768	2	801.4501953	-1.032442975	-0.000826933	109.8841686
																			ASLDRPFTNLESAFYSIVGLSSLGAQVPDAK	10	35493916	2xTMT6plex [N-Term; K31]	100	100		3.310055494	16.26	0	3711.999797	3	1238.003418	-1.103462816	-0.001365351	113.8766822
																			FSSGYYDFLVEVEGDNR	2	35493916	1xTMT6plex [N-Term]			100	3.180002451	21.28	0	2226.055244	2	1113.530884	-0.338280857	-0.000376516	95.06716155
																			YIANTVELR	8	35493916	1xTMT6plex [N-Term]	1.881	1.913	1.954	3.778615713	61.62	0	1307.752067	2	654.380127	0.696061794	0.000455138	46.77468293
																			EETVLATVQALQTASHLSQQADLR	4	35493916	1xTMT6plex [N-Term]				1.993892789	5.76	0	2838.515865	3	946.8424072	-1.126208365	-0.001065587	88.6854477
																			YHVPVVVVPEGSASDTHEQAILR	4	35493916	1xTMT6plex [N-Term]	1.44			2.089990377	9.26	0	2732.456894	4	683.8721313	3.587108967	0.002450405	41.63133594
																			LMDHVGTEPSIK	2	35493916	2xTMT6plex [N-Term; K12]		1.152		2.657940149	10.1	0	1784.998077	3	595.670166	-1.19428315	-0.000710598	50.25869453
																			LSKEETVLATVQALQTASHLSQQADLR	4	35493916	2xTMT6plex [N-Term; K3]		100		2.360471964	8.6	1	3395.889852	4	849.7296143	1.994976735	0.00169368	90.13327483
																			MLAQQAVK	2	35493916	2xTMT6plex [N-Term; K8]	1.321			3.52139926	43.92	0	1346.823013	2	673.914856	-0.428947183	-0.000288858	44.03921026
																			FELDTSER	6	35493916	1xTMT6plex [N-Term]	1.225	1.354	1.177	3.497914314	33.18	0	1225.626198	2	613.3165894	-0.24126366	-0.00014785	52.76112279
																			QEIQHLFR	2	35493916	1xTMT6plex [N-Term]			1.508	2.495213032	27.11	0	1299.737086	2	650.3724365	0.392953991	0.000255368	50.42477376
																			LMDHVGTEPSIKEDQVIQLMNAIFSK	6	35493916	3xTMT6plex [N-Term; K12; K26]				1.678729773	2.97	1	3630.984236	4	908.500061	-1.603341958	-0.001455427	101.8386589
																			NPILWNVADVVIK	3	35493916	2xTMT6plex [N-Term; K13]				1.374583006	2.9	0	1939.17809	2	970.0917969	-0.91415018	-0.00088635	95.14192366
116534937	IFIT1	interferon-induced protein with tetratricopeptide repeats 1 isoform 1	3434	NP_001539.3	33.89121339	1	15	15	87	478	55.325	7.2	0.24	0.22	0.31	0.26	Significant	0.011																
																			ALQETPTSVLLHHQIGLCYK	9	116534937	2xTMT6plex [N-Term; K20]; 1xCarbamidomethyl [C18	0.303	0.01		3.01209116	23.95	0	2766.537677	4	692.3897705	-0.153433208	-0.00010612	59.60433842
																			EAENLMQEEHDNQANVR	7	116534937	1xTMT6plex [N-Term]	0.254	0.252	0.228	6.781844616	70.28	0	2256.051238	3	752.6889038	0.407892235	0.000306742	40.47858685
																			EAENLMQEEHDNQANVR	14	116534937	1xTMT6plex [N-Term]; 1xOxidation [M6]	0.201	0.318	0.222	5.002901554	50.42	0	2272.046153	3	752.6889038	0.407892235	0.000306742	40.47858685
																			KPTFEVAHLDLAR	6	116534937	2xTMT6plex [N-Term; K1]	0.246	0.202		4.053602695	37.34	0	1955.147852	4	489.5424805	0.122591752	5.99E-05	58.35271255
																			LAADFENSVR	11	116534937	1xTMT6plex [N-Term]	0.223		0.01	3.784256697	41.05	0	1350.721495	2	675.8640747	-0.46073594	-0.000311163	58.79490845
																			VLDQIEFLDTK	4	116534937	2xTMT6plex [N-Term; K11]	0.26	0.01		2.792890787	43.8	0	1779.030423	2	890.0180664	-0.880455713	-0.000783179	88.24848651
																			LAEAQTYLDK	4	116534937	2xTMT6plex [N-Term; K10]	0.536	1.248		2.380441427	31.86	0	1609.920144	2	805.4630127	-0.866582967	-0.000697565	60.2674629
																			ALDLESLSLLGFVYK	6	116534937	2xTMT6plex [N-Term; K15]	0.01	0.01		2.295535803	11.98	0	2126.251314	2	1063.627686	-1.514283015	-0.001609873	106.0058992
																			KALDLESLSLLGFVYK	2	116534937	3xTMT6plex [N-Term; K1; K16]				2.732547283	11.11	1	2483.509209	3	828.5083618	0.532721688	0.000441006	106.7209059
																			SAIFHFESAVEK	2	116534937	2xTMT6plex [N-Term; K12]			0.195	3.368394852	22.6	0	1823.010356	3	608.34198	0.565472052	0.00034362	69.84760434
																			KSDVNAIIHYLK	2	116534937	3xTMT6plex [N-Term; K1; K12]			0.148	2.210488796	9.59	1	2088.278422	4	522.8244019	-1.266137742	-0.000661012	61.90070144
																			MECPEIDCEEGWALLK	6	116534937	2xTMT6plex [N-Term; K16]; 2xCarbamidomethyl [C3; C8	0.01			2.024500847	8.86	0	2438.180611	2	1219.59314	-0.659501235	-0.000803992	103.3343861
																			MYIEAGNHR	8	116534937	1xTMT6plex [N-Term]	0.196	0.201	0.264	3.424735308	38.57	0	1319.672771	2	660.3394775	-0.827789964	-0.000546206	32.81595576
																			KAEENFQK	2	116534937	3xTMT6plex [N-Term; K1; K8]			0.222	3.606357574	15.02	1	1680.988782	3	561.0009766	-0.241073887	-0.000135081	37.98658505
																			SDVNAIIHYLK	2	116534937	2xTMT6plex [N-Term; K11]	0.386			2.495286226	21.07	0	1731.020527	3	577.6778564	-0.872632091	-0.000503515	77.04048794
																			IEQASLTR	2	116534937	1xTMT6plex [N-Term]			0.637	2.743138075	26.2	0	1146.668003	2	573.8375244	-0.201341517	-0.000115436	39.17661777
9257257	WDR1	WD repeat-containing protein 1 isoform 1	9948	NP_059830.1	36.13861386	1	19	19	80	606	66.152	6.65	1.14	1.06	0.83	1.01	Significant	0.0084																
																			FGAVFLWDSGSSVGEITGHNK	2	9257257	2xTMT6plex [N-Term; K21]			1.624	4.504034996	31.04	0	2666.397872	3	889.4695435	-1.423080534	-0.001264833	93.34231052
																			GPVTDVAYSHDGAFLAVCDASK	6	9257257	2xTMT6plex [N-Term; K22]; 1xCarbamidomethyl [C18	0.665		100	4.002793312	40.66	0	2738.385987	3	913.4660034	-0.923736137	-0.000843182	60.21223638
																			SYIYSGSHDGHINYWDSETGENDSFAGK	2	9257257	2xTMT6plex [N-Term; K28]	1.158			5.409776688	31.89	0	3594.64499	4	899.4160767	-0.699223256	-0.000628365	62.10348223
																			NIDNPALADIYTEHAHQVVVAK	2	9257257	2xTMT6plex [N-Term; K22]	1.446			4.950475216	24.47	0	2876.567062	4	719.8956299	-2.21528948	-0.001593107	72.46970408
																			DYSGQGVVK	10	9257257	2xTMT6plex [N-Term; K9]	0.981	1.096	1.087	4.095932484	57.63	0	1410.799301	2	705.9039307	0.910206097	0.000642059	35.07572959
																			DIAWTEDSK	8	9257257	2xTMT6plex [N-Term; K9]	1.828	1.997	2.739	3.676328897	53.98	0	1522.815345	2	761.9113159	0.007030358	5.35E-06	61.98699558
																			YAPSGFYIASGDVSGK	4	9257257	2xTMT6plex [N-Term; K16]	0.671	1.024		3.18822813	45.8	0	2077.100627	2	1039.054199	0.238104738	0.000247284	76.37890302
																			LYSILGTTLK	4	9257257	2xTMT6plex [N-Term; K10]	100	100		3.070545197	25.62	0	1566.987101	2	783.9969482	-0.307242877	-0.000240723	93.78261851
																			SIQCLTVHK	4	9257257	2xTMT6plex [N-Term; K9]; 1xCarbamidomethyl [C4	1.132	1.044		4.160962105	42.12	0	1543.903054	3	515.3056641	-0.398673024	-0.000205171	52.3123327
																			VINSVDIK	6	9257257	2xTMT6plex [N-Term; K8]	1.135	0.939	0.715	3.419437408	42.75	0	1345.845523	2	673.4256592	-1.100275329	-0.0007404	49.07239315
																			YEYQPFAGK	8	9257257	2xTMT6plex [N-Term; K9]	0.854	1.997	0.354	3.215491533	27.56	0	1560.846251	2	780.9255981	-1.493485304	-0.00116555	57.06627563
																			KVFASLPQVER	5	9257257	2xTMT6plex [N-Term; K1]	1.193	1.184		2.973386526	14.58	1	1732.052161	3	578.0224609	0.385925156	0.000222814	62.18837051
																			VCALGGSK	2	9257257	2xTMT6plex [N-Term; K8]; 1xCarbamidomethyl [C2			1.148	3.415038586	43.19	0	1249.733864	2	625.3704224	-0.236757158	-0.000147942	42.11698922
																			IKDIAWTEDSK	2	9257257	3xTMT6plex [N-Term; K2; K11]			1.077	2.725482941	8.72	1	1993.157303	3	665.0574951	0.315429977	0.000209567	53.16971231
																			IAVVGEGR	6	9257257	1xTMT6plex [N-Term]	1.047	1.007	1.03	3.170686483	38.77	0	1029.62541	2	515.3164063	0.122190069	6.29E-05	39.73435264
																			DIAWTEDSKR	4	9257257	2xTMT6plex [N-Term; K9]	1.431		0.896	3.536279202	17.83	1	1678.916455	3	560.305542	-8.566682035	-0.004794248	43.47696379
																			IKDIAWTEDSKR	1	9257257	3xTMT6plex [N-Term; K2; K11]	0.461			2.734117031	1.4	2	2149.258414	4	538.0701904	0.240770838	0.00012937	60.12924763
																			MTVDESGQLISCSMDDTVR	2	9257257	1xTMT6plex [N-Term]; 1xCarbamidomethyl [C12				1.44871676		0	2373.093362	2	1187.04834	-1.668388848	-0.001979621	62.61532438
																			IIGGDPK	2	9257257	2xTMT6plex [N-Term; K7]		0.98	0.71	2.024790049	13.6	0	1157.72943	2	579.3684692	0.200043772	0.000115798	40.08228229
5174447	GNB2L1	guanine nucleotide-binding protein subunit beta-2-like	10399	NP_006089.1	62.46056782	1	16	16	106	317	35.055	7.69	1.55	1.68	1.46	1.57	Significant	0.0017																
																			AEPPQCTSLAWSADGQTLFAGYTDNLVR	7	5174447	1xTMT6plex [N-Term]; 1xCarbamidomethyl [C6	100		1.068	4.842096329	30.61	0	3297.604757	3	1099.87146	-1.495047937	-0.001643359	93.27689189
																			IIVDELKQEVISTSSK	7	5174447	3xTMT6plex [N-Term; K7; K16]	1.214	1.189	1.212	4.793292999	35.5	1	2476.484117	3	826.1651001	-1.360601107	-0.001123169	96.4134678
																			HLYTLDGGDIINALCFSPNR	12	5174447	1xTMT6plex [N-Term]; 1xCarbamidomethyl [C15	1.721	4.378	1.498	6.342540741	55.85	0	2505.275759	3	835.7635498	0.134633507	0.000112431	89.01762938
																			YTVQDESHSEWVSCVR	12	5174447	1xTMT6plex [N-Term]; 1xCarbamidomethyl [C14	1.677	1.499	1.898	6.025183201	47.23	0	2211.033797	2	1106.020264	-0.247235592	-0.000273323	45.46384311
																			TNHIGHTGYLNTVTVSPDGSLCASGGK	4	5174447	2xTMT6plex [N-Term; K27]; 1xCarbamidomethyl [C22	1.248	1.512		7.855666637	59.2	0	3201.636281	3	1067.881592	-1.892344677	-0.002019533	53.82090212
																			DGQAMLWDLNEGK	4	5174447	2xTMT6plex [N-Term; K13]				2.499963522	26.51	0	1935.004618	2	968.0057983	-0.154206512	-0.000149195	72.070642
																			DVLSVAFSSDNR	13	5174447	1xTMT6plex [N-Term]	1.669	1.226	100	2.84654665	30.86	0	1538.801202	2	769.9039307	-0.401073169	-0.000308586	50.8560636
																			LWDLTTGTTTR	8	5174447	1xTMT6plex [N-Term]	3.067	1.775	1.356	2.894065142	33.94	0	1493.816124	2	747.4112549	-0.596167333	-0.000445282	68.34858418
																			QEVISTSSK	2	5174447	2xTMT6plex [N-Term; K9]		2.141		3.61153698	47.2	0	1436.83608	2	718.9225464	1.208337862	0.000868092	33.45996681
																			DETNYGIPQR	3	5174447	1xTMT6plex [N-Term]		1.498	0.96	3.351094484	30.04	0	1421.722223	2	711.3644409	-0.434778364	-0.000309067	34.58909143
																			VWNLANCK	10	5174447	2xTMT6plex [N-Term; K8]; 1xCarbamidomethyl [C7	1.627	1.852	1.424	3.751808882	47.34	0	1462.824076	2	731.9152832	-0.537408266	-0.000393067	67.20386676
																			IIVDELK	11	5174447	2xTMT6plex [N-Term; K7]	1.838	4.041	3	3.280488253	37.99	0	1287.82881	2	644.4187012	1.021753621	0.000657922	50.52520494
																			YWLCAATGPSIK	2	5174447	2xTMT6plex [N-Term; K12]; 1xCarbamidomethyl [C4				2.006804943	10.35	0	1825.008248	2	913.0070801	-0.747483608	-0.000682082	72.00159567
																			IWDLEGK	4	5174447	2xTMT6plex [N-Term; K7]	0.812	100		2.699821949	33.33	0	1318.777109	2	659.8918457	-0.526156957	-0.000346942	67.57718514
																			LWNTLGVCK	6	5174447	2xTMT6plex [N-Term; K9]; 1xCarbamidomethyl [C8	100		100	2.326492548	16.94	0	1548.897241	2	774.9506836	-2.033867978	-0.001575126	71.85824746
																			VWQVTIGTR	1	5174447	1xTMT6plex [N-Term]	0.535			2.40032649		0	1288.757487	2	644.8821411	-0.373362283	-0.000240587	69.53687813
66933016	IMPDH2	inosine-5'-monophosphate dehydrogenase 2	3615	NP_000875.2	41.43968872	1	14	15	88	514	55.77	6.9	1.82	1.87	1.87	1.85	Significant	0.000081																
																			QLLCGAAIGTHEDDKYR	4	66933016	2xTMT6plex [N-Term; K15]; 1xCarbamidomethyl [C4	1.779	2.146		5.275309563	23.78	1	2405.264749	4	602.0725708	1.539931572	0.000925986	48.39741989
																			GKLPIVNEDDELVAIIAR	3	66933016	2xTMT6plex [N-Term; K2]	2.605			4.727107525	23.29	1	2423.427381	3	808.4805298	-0.142279704	-0.000114935	87.83996754
																			REDLVVAPAGITLK	6	66933016	2xTMT6plex [N-Term; K14]	1.337	100	1.713	5.774170399	44.26	1	1940.194468	3	647.4018555	-1.780730647	-0.001151655	62.94741813
																			ALALGASTVMMGSLLAATTEAPGEYFFSDGIR	18	66933016	1xTMT6plex [N-Term]		1.694		4.142122269	28.96	0	3476.764293	4	869.9467773	0.283609965	0.000246511	112.858374
																			VAQGVSGAVQDK	4	66933016	2xTMT6plex [N-Term; K12]	2.165	1.652		5.179255486	78.13	0	1616.937191	2	808.9725342	0.371572488	0.000300405	40.0085154
																			DIDFLKEEEHDCFLEEIMTK	4	66933016	3xTMT6plex [N-Term; K6; K20]; 1xCarbamidomethyl [C12		100	100	3.454585791	12.19	1	3228.641164	4	807.9098511	-7.306294484	-0.005897351	89.16377308
																			TSSAQVEGGVHSLHSYEK	2	66933016	2xTMT6plex [N-Term; K18]		1.115		2.731547832	4.22	0	2374.240308	4	594.3154907	-0.073880211	-4.39E-05	43.33175899
																			AMMYSGELK	12	66933016	2xTMT6plex [N-Term; K9]	1.637	1.722	1.397	4.172319412	59.35	0	1487.800229	2	744.4042358	0.649589689	0.00048323	52.26133826
																			KYEQGFITDPVVLSPK	6	66933016	3xTMT6plex [N-Term; K1; K16]	100	100	1.214	2.885914803	23.07	1	2508.468073	3	836.8257446	-2.149516408	-0.001797331	61.65315166
																			LVGIISSR	6	66933016	1xTMT6plex [N-Term]	1.571	1.781	2.14	2.808647394	41.22	0	1073.68801	2	537.3479614	0.592277791	0.000317961	53.52200093
																			NLIDAGVDALR	6	66933016	1xTMT6plex [N-Term]	100	100	100	2.489905596	24.96	0	1385.794994	2	693.4005737	-0.81076138	-0.000561775	63.51328654
																			DKYPNLQVIGGNVVTAAQAK	5	66933016	3xTMT6plex [N-Term; K2; K20]				2.233109474	14.06	1	2773.617925	3	925.2105713	-0.275446306	-0.000254661	76.20555999
																			EANEILQR	6	66933016	1xTMT6plex [N-Term]	1.989	2.13	2.02	3.162423611	37.43	0	1201.673817	2	601.34021	-0.560391776	-0.000336704	40.99646277
																			EDLVVAPAGITLK	3	66933016	2xTMT6plex [N-Term; K13]	0.868	100		1.822210908	9.6	0	1784.093357	2	892.5494995	-0.916295415	-0.000817378	72.66806453
																			GMGSLDAMDK	2	66933016	2xTMT6plex [N-Term; K10]; 1xOxidation [M8	1.583			2.023098469	21.65	0	1498.764572	2	749.8857422	-0.242836672	-0.000181978	46.74533275
																			GMGSLDAMDK	1	66933016	2xTMT6plex [N-Term; K10]			1.486	1.731451631		0	1482.769657	2	749.8857422	-0.242836672	-0.000181978	46.74533275
11559929	COPG1	coatomer subunit gamma-1	22820	NP_057212.1	30.66361556	1	17	19	65	874	97.655	5.47	1.23	1.22	1.14	1.2	Significant	0.00057																
																			HPSAVTACNLDLENLVTDSNR	6	11559929	1xTMT6plex [N-Term]; 1xCarbamidomethyl [C8	1.137	1.07	100	6.386172771	69.08	0	2555.27213	3	852.4290161	0.142904483	0.00012172	70.55108979
																			TLEEAVGNIVK	5	11559929	2xTMT6plex [N-Term; K11]	1.052		100	3.669621944	58.5	0	1630.977993	2	815.9929199	0.349553306	0.000285057	71.74064703
																			KDEESGGGSNPFQHLEK	2	11559929	3xTMT6plex [N-Term; K1; K17]		1.109		5.04157114	43.36	1	2546.345391	4	637.3411865	-0.971979787	-0.000618749	42.85079603
																			ALQQYTLEPSEKPFDLK	4	11559929	3xTMT6plex [N-Term; K12; K17]	1.564			3.284570456	22.19	0	2694.532129	3	898.8482056	-0.76669369	-0.000688627	83.875064
																			QLEEEDGSR	4	11559929	1xTMT6plex [N-Term]	1.464	1.353		3.919180632	43.69	0	1291.63274	2	646.3204346	0.660304279	0.000426435	23.59349339
																			LLLLDTVTMQVTAR	2	11559929	1xTMT6plex [N-Term]			0.957	2.713156939	13.29	0	1803.061122	2	902.0324707	-1.91722494	-0.001728437	99.02263534
																			CVMDDDNEVR	2	11559929	1xTMT6plex [N-Term]; 1xCarbamidomethyl [C1	1.265			3.836587191	41.16	0	1481.656194	2	741.3327637	1.387949349	0.001028232	35.32136929
																			VVLEHEEVR	10	11559929	1xTMT6plex [N-Term]	1.191	1.314	1.167	3.913345098	58.31	0	1338.757881	3	446.9236755	-1.051182363	-0.000469093	34.41373919
																			ILHLLGQEGPK	2	11559929	2xTMT6plex [N-Term; K11]		1.204		2.140841007	28.12	0	1663.030697	3	555.0146484	-0.784932895	-0.000435123	71.95094795
																			ATFYLNVLEQK	4	11559929	2xTMT6plex [N-Term; K11]	100	100		2.204829931	20.59	0	1784.035842	2	892.5198975	-1.863201426	-0.001662009	78.71769623
																			EEGGFEYK	4	11559929	2xTMT6plex [N-Term; K8]	2.08		0.853	3.538096428	33.03	0	1416.741117	2	708.8741455	-0.07242987	-5.13E-05	49.5882652
																			ILYLINQGEHLGTTEATEAFFAMTK	3	11559929	2xTMT6plex [N-Term; K25]			100	1.396723151	0.13	0	3256.732807	3	1086.245239	-3.574777931	-0.003880699	111.5837474
																			ETGLSHLCEFIEDCEFTVLATR	4	11559929	1xTMT6plex [N-Term]; 2xCarbamidomethyl [C8; C14				1.502598643	0.15	0	2856.374546	3	952.7960815	-0.299306369	-0.000284977	112.8347893
																			GLGPLFK	2	11559929	2xTMT6plex [N-Term; K7]	0.794			2.813351631	22.89	0	1189.770901	2	595.3882446	-1.419216242	-0.000844271	66.71854971
																			SSPEPVALTESETEYVIR	4	11559929	1xTMT6plex [N-Term]			100	1.655612469	1.48	0	2236.154624	2	1118.5802	-0.670722569	-0.00074992	64.41225704
																			SLPYNQPGTCYTLVALPK	2	11559929	2xTMT6plex [N-Term; K18]; 1xCarbamidomethyl [C10				1.089506149		0	2480.362338	2	1240.683594	-0.97860299	-0.001213645	78.55690433
																			SIATLAITTLLK	2	11559929	2xTMT6plex [N-Term; K12]				1.120382786		0	1703.108279	2	852.0571289	-0.762017023	-0.000648899	102.283894
																			VVVVQAISALCQK	1	11559929	2xTMT6plex [N-Term; K13]; 1xCarbamidomethyl [C11				0.856646776		0	1873.134511	2	937.0712891	0.422139999	0.000395363	78.32344838
																			QEIFQEQLAAVPEFR	2	11559929	1xTMT6plex [N-Term]				1.085221291		0	2034.085756	2	1017.54657	0.052425733	5.33E-05	80.94421749
524552373	ANXA8L1	annexin A8-like protein 1 isoform 1	728113	NP_001092315.2	57.79816514	1	2	15	112	327	36.856	5.78	1.01	0.9	0.94	0.95	Significant	0.0011																
																			GIGTNEQAIIDVLTK	12	524552373	2xTMT6plex [N-Term; K15]	100	0.321	0.826	3.91865921	64.57	0	2030.189777	2	1015.599731	1.186928756	0.001204845	92.36049684
																			SSSHFNPDPDAETLYK	8	524552373	2xTMT6plex [N-Term; K16]	0.887	0.851	0.854	5.702804089	54.2	0	2266.139197	3	756.0505371	-0.944064635	-0.000713127	46.11302173
																			AYEEDYGSSLEEDIQADTSGYLER	27	524552373	1xTMT6plex [N-Term]	1.289	0.811	100	5.074197292	47.36	0	2969.337351	3	990.449585	-1.060676782	-0.001049836	91.23192367
																			SETHGSLEEAMLTVVK	5	524552373	2xTMT6plex [N-Term; K16]	1.213		100	5.191122532	36.43	0	2189.18879	3	730.3997803	-1.828375746	-0.00133422	82.12384011
																			DDVSSFVDPALALQDAQDLYAAGEK	6	524552373	2xTMT6plex [N-Term; K25]	100			3.241720438	35.47	0	3096.577746	3	1032.862671	-1.384197343	-0.001428758	105.8116104
																			SEIDLNLIK	10	524552373	2xTMT6plex [N-Term; K9]	0.929	1.07	1.078	3.64244175	53.38	0	1502.919416	2	751.9629517	-0.524958078	-0.000394485	77.59300504
																			AWIEQEGVTVK	7	524552373	2xTMT6plex [N-Term; K11]	1.034	1.007	100	3.764716625	30.5	0	1717.988892	2	859.4979248	-0.1857699	-0.000159575	68.79747333
																			CTQNLHSYFAER	2	524552373	1xTMT6plex [N-Term]; 1xCarbamidomethyl [C1			1.265	4.489290237	29.52	0	1754.848169	3	585.6152344	-9.698420239	-0.005673085	42.67728741
																			ILVCLLQGSR	5	524552373	1xTMT6plex [N-Term]; 1xCarbamidomethyl [C4	1.934	100	0.722	2.45731926	29.72	0	1387.829272	2	694.4176636	-0.879972477	-0.000610626	82.56305909
																			LIVALMYPPYR	6	524552373	1xTMT6plex [N-Term]				1.703364611	17.54	0	1564.912273	2	782.9595337	-0.308028249	-0.000241019	87.95973983
																			FITILCTR	8	524552373	1xTMT6plex [N-Term]; 1xCarbamidomethyl [C6		100	1.196	2.754993677	28.53	0	1252.728495	2	626.8672485	-1.017570601	-0.00063737	80.84486513
																			EGVIIEILASR	2	524552373	1xTMT6plex [N-Term]	1.116			1.379170179	12.24	0	1428.862346	2	714.9346313	-0.251658033	-0.000179792	99.26305716
																			VFEEYEK	4	524552373	2xTMT6plex [N-Term; K7]	1.031		0.807	3.336134195	39.13	0	1401.766603	2	701.3873291	0.555073236	0.000389042	60.17754059
																			DLTETLK	6	524552373	2xTMT6plex [N-Term; K7]	0.932	0.988	0.957	3.22570467	34.69	0	1277.771689	2	639.3892822	-0.31387992	-0.000200533	44.97509592
																			SIEDSIK	3	524552373	2xTMT6plex [N-Term; K7]	0.995		0.998	3.039762259	34.9	0	1249.740389	2	625.3739014	0.109890323	6.87E-05	44.99581467
																			SETHGSLEEAMLTVVK	1	524552373	2xTMT6plex [N-Term; K16]; 1xOxidation [M11				1.449336171		0	2205.183705	3	730.3997803	-1.828375746	-0.00133422	82.12384011
33413400	ESD	S-formylglutathione hydrolase	2098	NP_001975.1	78.36879433	1	14	14	125	282	31.442	7.02	1	0.95	1.32	1.09	Significant	0.011																
																			GEDESWDFGTGAGFYVDATEDPWK	57	33413400	2xTMT6plex [N-Term; K24]	1.161	1.364	1.856	3.599422932	35.81	0	3137.441646	3	1046.484741	-0.62975743	-0.000658609	99.09724578
																			SGYHQSASEHGLVVIAPDTSPR	2	33413400	1xTMT6plex [N-Term]				4.070784092	14.77	0	2537.29458	4	635.0795898	0.768470092	0.000487459	45.73452027
																			SYPGSQLDILIDQGKDDQFLLDGQLLPDNFIAACTEK	8	33413400	3xTMT6plex [N-Term; K15; K37]; 1xCarbamidomethyl [C34	1.156	1.317		6.218817234	30.42	1	4825.521312	5	965.9080811	-2.075140884	-0.002002727	104.2043204
																			LQEGYDHSYYFIATFITDHIR	8	33413400	1xTMT6plex [N-Term]	100	1.538	1.523	4.695633888	26.13	0	2818.403796	4	705.3557739	-0.897631091	-0.000632472	103.6342584
																			KAFSGYLGTDQSK	4	33413400	3xTMT6plex [N-Term; K1; K13]	0.637	0.604		5.723998547	32.91	1	2089.189666	3	697.0680542	-0.027174337	-1.89E-05	46.75328656
																			SVSAFAPICNPVLCPWGK	2	33413400	2xTMT6plex [N-Term; K18]; 2xCarbamidomethyl [C9; C14		1.051		4.628552914	60.45	0	2461.313614	3	821.1087036	-0.835586389	-0.000685547	95.17613036
																			VFEHDSVELNCK	6	33413400	2xTMT6plex [N-Term; K12]; 1xCarbamidomethyl [C11		1.308	1.255	4.613533974	35.38	0	1935.004619	3	645.6734619	0.627250279	0.000404577	44.6435316
																			AFSGYLGTDQSK	6	33413400	2xTMT6plex [N-Term; K12]	100	100	0.843	2.821320772	40.32	0	1731.931771	2	866.4692383	-0.329815244	-0.000285609	51.50005149
																			AYDATHLVK	8	33413400	2xTMT6plex [N-Term; K9]	0.96	0.941		3.543121815	45.53	0	1475.862235	2	738.4337158	-1.409467175	-0.00104009	47.91340101
																			MYSYVTEELPQLINANFPVDPQR	4	33413400	1xTMT6plex [N-Term]				1.575776815	16.38	0	2953.49671	3	985.1682129	-2.242866262	-0.002208099	103.481621
																			CPALYWLSGLTCTEQNFISK	4	33413400	2xTMT6plex [N-Term; K20]; 2xCarbamidomethyl [C1; C12			100	1.562003613	8.51	0	2846.462129	3	949.4865112	-6.024418319	-0.005716093	106.0265375
																			FAVYLPPK	8	33413400	2xTMT6plex [N-Term; K8]	2.089	100	100	2.238295555	16.13	0	1392.86553	2	696.9373779	1.399610611	0.000974735	71.06875551
																			CFGGLQK	2	33413400	2xTMT6plex [N-Term; K7]; 1xCarbamidomethyl [C1	1.057			2.637855053	21.64	0	1267.723299	2	634.3640137	-2.010380539	-0.001274303	48.35821557
																			KIPVVFR	6	33413400	2xTMT6plex [N-Term; K1]	1.122	1.027	0.9	3.152001858	6.37	1	1316.881849	3	439.631897	-0.539837368	-0.000236967	57.72138566
158937236	NPEPPS	puromycin-sensitive aminopeptidase	9520	NP_006301.3	25.46245919	1	19	19	89	919	103.211	5.72	0.69	0.66	0.68	0.67	Significant	0.00017																
																			DAESIHQYLLQR	8	158937236	1xTMT6plex [N-Term]	0.637	0.614	0.603	4.618801594	46.29	0	1701.912149	3	567.9755249	-0.075099628	-4.26E-05	46.2942733
																			KPYPDDENLVEVK	8	158937236	3xTMT6plex [N-Term; K1; K13]	0.56	0.566	0.659	5.093026638	30.25	0	2233.26831	3	745.0946045	0.425361591	0.000316649	48.28230193
																			YAAVTQFEATDAR	2	158937236	1xTMT6plex [N-Term]	0.688			3.514238834	42.68	0	1671.853966	2	836.4312744	0.781478617	0.000653259	85.98404363
																			DVFSPIGER	6	158937236	1xTMT6plex [N-Term]	0.826	1.167		3.303447485	35.94	0	1248.678568	2	624.8435669	1.032634896	0.000644715	51.88652315
																			AQELDALDNSHPIEVSVGHPSEVDEIFDAISYSK	3	158937236	2xTMT6plex [N-Term; K34]				2.086399317	0.73	0	4170.091918	4	1043.276611	-1.751284538	-0.001825754	88.54478395
																			LSVEGFAVDK	6	158937236	2xTMT6plex [N-Term; K10]	0.518		100	2.52990818	27.78	0	1522.888116	2	761.9478149	0.156093419	0.000118856	78.49363131
																			DNWEELYNR	5	158937236	1xTMT6plex [N-Term]	0.839			3.147309542	40.9	0	1467.706573	2	734.3563843	-0.736765326	-0.000540678	63.92120942
																			VALSNMNVIDR	5	158937236	1xTMT6plex [N-Term]	100	0.01	0.637	2.082684517	28.46	0	1460.809264	2	730.9083862	0.1585429	0.0001158	52.66980854
																			MLHDYIGDKDFK	6	158937236	3xTMT6plex [N-Term; K9; K12]	0.39	0.567	0.327	2.698833466	9.53	1	2169.198122	4	543.0550537	0.121069508	6.57E-05	53.77447065
																			GMNMYLTK	2	158937236	2xTMT6plex [N-Term; K8]	0.75			3.672804117	44.88	0	1415.779099	2	708.3931885	0.000743848	5.27E-07	63.87900802
																			ETALLIDPK	4	158937236	2xTMT6plex [N-Term; K9]		100		1.809243798	28	0	1457.897952	2	729.4522095	-0.555357586	-0.000404827	53.75593133
																			QADMQEEK	4	158937236	2xTMT6plex [N-Term; K8]	0.662	0.893		3.410315275	43.76	0	1436.74555	2	718.8760376	-0.523303996	-0.000375927	33.73991507
																			LPADVSPINYSLCLKPDLLDFTFEGK	2	158937236	3xTMT6plex [N-Term; K15; K26]; 1xCarbamidomethyl [C13				1.404449701	5.86	0	3639.995119	3	1214.000122	-2.55648394	-0.003101863	102.3955531
																			NVKPDQWVK	2	158937236	3xTMT6plex [N-Term; K3; K9]			0.651	3.076158047	7.48	0	1801.093915	3	601.036438	0.46931192	0.000281758	51.94535711
																			ILMDKPEMNVVLK	4	158937236	3xTMT6plex [N-Term; K5; K13]	0.01			1.998477101	5.05	0	2217.331779	3	739.7808228	-1.742647771	-0.001288009	68.48389842
																			LEAAAQVR	11	158937236	1xTMT6plex [N-Term]	0.818	0.803	0.802	2.592961311	45.48	0	1086.646874	2	543.8273926	0.584160563	0.000317388	36.06915997
																			FALEVAAK	4	158937236	2xTMT6plex [N-Term; K8]		0.01		2.15982151	24.13	0	1306.813494	2	653.909729	-1.004582669	-0.000656401	72.0278101
																			ATFDISLVVPK	2	158937236	2xTMT6plex [N-Term; K11]	100			1.814689636	21.56	0	1648.008565	2	824.5077515	-0.205514898	-0.000169345	84.24908642
																			AFPCWDEPAIK	5	158937236	2xTMT6plex [N-Term; K11]; 1xCarbamidomethyl [C4	0.961	100		1.961381078	11.2	0	1791.950398	2	896.4803467	1.684337567	0.001509125	73.46820778
82659109	UBR4	E3 ubiquitin-protein ligase UBR4	23352	NP_065816.2	5.151456685	1	20	20	58	5183	573.476	6.04	0.98	1.21	1.15	1.11	Significant	0.0038																
																			GITQNALDYMK	2	82659109	2xTMT6plex [N-Term; K11]	0.869			2.618328094	47.78	0	1711.945313	2	856.4764404	0.1702504	0.00014573	74.61576836
																			GREEWESAALQNANTK	2	82659109	2xTMT6plex [N-Term; K16]	1.054			5.435747623	32.84	1	2262.187879	3	754.7348633	0.953773013	0.000719205	51.60144736
																			TSPADHGGSVGSESGGSAVDSVAGEHSVSGR	2	82659109	1xTMT6plex [N-Term]		1.049		6.201497555	42.1	0	3069.430691	4	768.1138916	0.992088291	0.000761287	32.84568725
																			AQQALSELHTVEK	2	82659109	2xTMT6plex [N-Term; K13]	1.14			4.222455025	18.31	0	1912.090397	3	638.0361328	1.803381004	0.001149409	60.91595232
																			DSDQLDVIQENR	2	82659109	1xTMT6plex [N-Term]			1.398	3.452839613	36.27	0	1660.833958	2	830.9207764	0.19126197	0.000158827	48.42976098
																			ACAEIAAQR	12	82659109	1xTMT6plex [N-Term]; 1xCarbamidomethyl [C2	1.175	1.423	1.373	3.509832621	43.88	0	1218.646222	2	609.8265991	-0.246558687	-0.000150234	29.16201458
																			LEQVSSDEGIGTLAENLLEALR	2	82659109	1xTMT6plex [N-Term]		100		1.869107962	11.69	0	2586.382392	2	1293.694824	-0.007613917	-9.85E-06	116.5226592
																			ATTPLYHGFK	2	82659109	2xTMT6plex [N-Term; K10]			1.264	2.773740053	33.97	0	1592.920084	3	531.6450806	0.379202463	0.000201346	50.99865105
																			KVQLTPGQTEVK	2	82659109	3xTMT6plex [N-Term; K1; K12]			0.867	3.501964331	14.56	1	2015.246787	3	672.4217529	1.94439663	0.001306146	47.3138364
																			EKDPLAALR	2	82659109	2xTMT6plex [N-Term; K2]	1.366			2.758005619	16.52	1	1470.904434	3	490.9726868	-0.630426589	-0.000309099	51.13730897
																			ESAFQSEPR	4	82659109	1xTMT6plex [N-Term]	0.407	1.21		3.333455086	30.81	0	1279.647996	2	640.3279419	0.477724393	0.00030566	34.30431138
																			IQIGTQAIER	2	82659109	1xTMT6plex [N-Term]	0.151			3.13424468	25.76	0	1357.80008	2	679.4040527	0.551670869	0.000374529	54.3854397
																			GLLGDATEEFIESLDSTTDEEEDEEVYK	3	82659109	2xTMT6plex [N-Term; K29]				1.420502782	0.91	0	3750.753322	3	1250.91626	-5.091268736	-0.006365364	107.5613147
																			AVSNINTLLDK	5	82659109	2xTMT6plex [N-Term; K11]				1.059599757	9.86	0	1645.988892	2	823.4977417	-0.41638286	-0.000342681	61.57609234
																			LIKPPAPTSK	2	82659109	3xTMT6plex [N-Term; K3; K10]			1.07	2.814168692	4.65	0	1739.139803	3	580.3853149	0.913413756	0.000529518	42.06518476
																			ALATNPALR	2	82659109	1xTMT6plex [N-Term]			1.152	2.854509592	27.5	0	1155.704723	2	578.3565674	0.982236728	0.000567588	40.81799245
																			ASYDAWK	4	82659109	2xTMT6plex [N-Term; K7]	1.115	0.901		3.202145815	29.44	0	1298.714508	2	649.8596191	-1.960991991	-0.001273384	50.37767988
																			GVLPYVGNLITK	2	82659109	2xTMT6plex [N-Term; K12]				1.376820803	0.31	0	1732.077313	2	866.5413208	-1.12481032	-0.000974129	84.27517138
																			EPTYQLNIHDIK	2	82659109	2xTMT6plex [N-Term; K12]			100	1.402523756	4.96	0	1929.084583	3	643.699585	-0.197864412	-0.000127232	53.8777466
																			ILGPAESDEFLAR	2	82659109	1xTMT6plex [N-Term]	0.618			2.155759573	20.34	0	1646.895102	2	823.9505615	-0.762533766	-0.000627907	80.49862189
124494254	PA2G4	proliferation-associated protein 2G4	5036	NP_006182.2	44.92385787	1	15	15	155	394	43.759	6.55	1.11	0.98	0.89	1	Significant	0.0041																
																			SEMEVQDAELK	5	124494254	2xTMT6plex [N-Term; K11]	1.343	1.34		4.49162817	67.06	0	1736.914072	2	868.9599609	-0.821511566	-0.000713448	54.68266499
																			TAENATSGETLEENEAGD	13	124494254	1xTMT6plex [N-Term]	1.426	1.473	1.416	3.994978428	80.71	0	2066.919932	2	1033.96228	-1.281265464	-0.001324137	35.6674313
																			LVKPGNQNTQVTEAWNK	6	124494254	3xTMT6plex [N-Term; K3; K17]	3.303	0.998		4.062438965	27.05	0	2614.491996	3	872.1679688	-1.010861767	-0.000880963	59.4902799

Bhat et al,2017. Cigarette smoke and chewing tobacco alter expression of different sets of miRNAs in oral keratinocytes

Supplementary Table S2(A). List of all proteins and peptides identified in OKF6/TERT1 cells chronically treated with chewing tobacco using Sequest and Mascot search algorithms

Accession	Gene_Symbol	Description	Gene_ID	Refseq_Accession	Coverage	Proteins	#Unique_Peptides	#Peptides	#PSMS	#AAs	MW [kDa]	calc. pI	OKF6-STE-6M/OKF6P_R1	OKF6-STE-6M/OKF6P_R2	OKF6-STE-6M/OKF6P_R3	Median fold change	Status	P-value (6M)	Sequence	#PSMSs	Protein Accessions	Modifications	OKF6-STE-6M/OKF6P_R1	OKF6-STE-6M/OKF6P_R2	OKF6-STE-6M/OKF6P_R3	Xcorr	Ionscore	# Missed Cleavages	Theo. MH+ [Da]	Charge	m_z	DeltaM [ppm]	Deltam/z [Da]	RT [min]
																			AAHLCAEAALR	63	124494254	1xTMT6plex [N-Term]; 1xCarbamidomethyl [C5	1.055	0.937	0.878	4.57962656	63.13	0	1411.767734	2	706.3878174	0.442003036	0.000312003	36.50349916
																			HELLQPFNVLYEK	8	124494254	2xTMT6plex [N-Term; K13]	1.474		1.043	3.543326855	28.42	0	2088.189383	3	696.7353516	1.01461942	0.000706239	78.41745861
																			AEFEVHEVYAVDVLVSSGEGK	11	124494254	2xTMT6plex [N-Term; K21]			1.036	3.284703255	14.3	0	2722.433982	3	908.1484985	-1.116612763	-0.001013302	94.66349838
																			ITSGPFEPDLYK	11	124494254	2xTMT6plex [N-Term; K12]	1.43	1.3	0.01	2.713265896	25.08	0	1825.014772	2	913.0105591	-0.510103234	-0.000465473	71.39703046
																			TIIQNPTDQQK	4	124494254	2xTMT6plex [N-Term; K11]	1.365	1.545		3.943825245	35.71	0	1744.000519	2	872.5037842	-0.13053931	-0.00011383	49.01823401
																			GIAFPTSISVNNCVCHFSPLK	8	124494254	2xTMT6plex [N-Term; K21]; 2xCarbamidomethyl [C13; C15	100	100	0.778	2.779683352	21.77	0	2806.478448	3	936.1642456	-0.094051531	-8.80E-05	75.31982277
																			AFFSEVER	8	124494254	1xTMT6plex [N-Term]	1.192	1.209		3.013914347	38.11	0	1213.641454	2	607.3241577	-0.342094703	-0.00020759	64.5036852
																			MGVVECAK	2	124494254	2xTMT6plex [N-Term; K8]; 1xCarbamidomethyl [C6	1.76			3.580584288	36.47	0	1351.747799	2	676.3778076	0.398849826	0.000269572	44.30540443
																			RFDAMPFTLR	2	124494254	1xTMT6plex [N-Term]	1.157			3.169412136	12.41	1	1482.80887	3	494.9415588	0.844939423	0.000417628	64.42641479
																			SDQDYILK	8	124494254	2xTMT6plex [N-Term; K8]		1.335	1.442	3.654722929	40	0	1439.814616	2	720.4112549	0.428434062	0.000308433	53.32021134
																			QHVIDGEK	2	124494254	2xTMT6plex [N-Term; K8]		1.231		2.147395611	19.34	0	1383.799635	3	461.9375916	-1.021500339	-0.000471184	30.90470624
																			FDAMPFTLR	4	124494254	1xTMT6plex [N-Term]	2.397			1.937032461	28.67	0	1326.707759	2	663.8587036	1.787271195	0.001185593	79.82756448
4758516	HDGF	hepatoma-derived growth factor isoform a	3068	NP_004485.1	58.33333333	1	13	13	72	240	26.772	4.73	0.96	0.93	0.85	0.91	Significant	0.0013																
																			GPPQEEEEEEDEEEEATKEDAEAPGIR	4	4758516	2xTMT6plex [N-Term; K18]	0.691	0.697		5.293784142	33.13	1	3500.607661	4	875.9071045	-0.306515712	-0.000268248	58.71340359
																			GPPQEEEEEEDEEEEATK	16	4758516	2xTMT6plex [N-Term; K18]	0.81	0.866	0.813	4.443730831	52.4	0	2562.161902	2	1281.571899	-9.905701842	-0.012690006	39.84803189
																			SCVEEPEPEPEAAEGDGDKK	4	4758516	3xTMT6plex [N-Term; K19; K20]; 1xCarbamidomethyl [C2		1.09	1.404	4.537666321	32.26	1	2860.412544	3	954.1412964	-1.121603893	-0.001069417	38.06774628
																			KSCVEEPEPEPEAAEGDGDK	4	4758516	3xTMT6plex [N-Term; K1; K20]; 1xCarbamidomethyl [C3	0.832		0.661	6.36900425	46.34	1	2860.412544	3	954.140564	-1.889767782	-0.001801838	37.75639028
																			IDEMPEAAVK	8	4758516	2xTMT6plex [N-Term; K10]	1.158	1.061		3.860754251	52.03	0	1560.870751	2	780.9386597	-0.4537173	-0.000354097	58.57132864
																			EAENPEGEEKEAATLEVERPLPMEVEK	2	4758516	3xTMT6plex [N-Term; K10; K27]		0.232		3.763928175	13.37	1	3740.950747	4	935.9921875	-1.022865646	-0.000956623	69.99052048
																			EAENPEGEEK	4	4758516	2xTMT6plex [N-Term; K10]	0.926	0.647		3.896517515	41.55	0	1589.805902	2	795.4060669	-0.657168863	-0.000522385	32.19543
																			EAATLEVERPLPMEVEK	4	4758516	2xTMT6plex [N-Term; K17]			0.919	2.459201097	28.74	0	2399.325618	3	800.4452515	-1.840861213	-0.001472275	56.06838277
																			ASGYQSSQK	4	4758516	2xTMT6plex [N-Term; K9]		0.699	0.979	3.978276968	55.37	0	1413.773814	2	707.3912964	1.062442537	0.000751027	22.82307996
																			DLFPYEESK	12	4758516	2xTMT6plex [N-Term; K9]	0.893	0.888	0.924	3.068046808	37.28	0	1585.851396	2	793.4292603	-0.095703915	-7.59E-05	70.85069515
																			LVIDEPAK	4	4758516	2xTMT6plex [N-Term; K8]	1.179	1.093		3.318502665	30.94	0	1342.834624	2	671.920166	-1.167752695	-0.000784049	52.7101685
																			GNAEGSSDEEGK	4	4758516	2xTMT6plex [N-Term; K12]				2.029903173	37.35	0	1637.801879	2	819.4046021	0.029516124	2.42E-05	14.25426251
																			YQVFFFGTHETAFLGPK	2	4758516	2xTMT6plex [N-Term; K17]				1.823402047	3.5	0	2447.316374	3	816.4436646	0.027120459	2.21E-05	90.43901668
7305503	STOML2	stomatin-like protein 2, mitochondrial isoform	30968	NP_038470.1	50.28089888	1	12	12	85	356	38.51	7.39	0.92	0.99	0.95	0.95	Significant	0.00045																
																			AEQINQAAGEASAVLAK	6	7305503	2xTMT6plex [N-Term; K17]	0.01	0.548		2.529112577	35.9	0	2129.196653	2	1065.101196	-0.721798934	-0.000768426	63.6054252
																			ESLNASIVDAINQAADCWGIR	8	7305503	1xTMT6plex [N-Term]; 1xCarbamidomethyl [C17	100	1.135	0.848	3.224778891	27.7	0	2532.271402	3	844.7614746	-0.60464367	-0.000510374	107.4634608
																			QAQILASEAEK	6	7305503	2xTMT6plex [N-Term; K11]	0.918	1.029	0.875	4.74007082	67.53	0	1645.952507	2	823.4801025	0.256288151	0.000210919	51.21261797
																			DVQGTDASLDEELDR	6	7305503	1xTMT6plex [N-Term]	0.914	0.926		4.559836864	80.03	0	1891.908245	2	946.4580688	0.325399138	0.000307813	46.60859024
																			VKESMQMQVEAER	4	7305503	2xTMT6plex [N-Term; K2]	0.6	0.354		4.802890778	28.23	1	2023.071652	3	675.0288696	0.199462396	0.000134509	49.75534108
																			ASYGVEDPEYAVTQLAQTTMR	13	7305503	1xTMT6plex [N-Term]	0.738	0.896		5.025392056	51.7	0	2559.259834	3	853.7581787	0.058322649	4.98E-05	91.55276758
																			ESMQMQVEAER	8	7305503	1xTMT6plex [N-Term]	1.103	1.351	1.144	4.541250229	65.03	0	1566.745343	2	783.8760986	-0.269753075	-0.000211317	38.04178195
																			ESAINVAEGK	6	7305503	2xTMT6plex [N-Term; K10]	0.91			4.39110899	53.94	0	1475.846979	2	738.427063	-0.087843414	-6.48E-05	49.78774192
																			ATVLESEGTR	10	7305503	1xTMT6plex [N-Term]	0.955	0.829	0.768	4.238215446	62.98	0	1291.705511	2	646.3500366	-9.842853272	-0.006357034	35.98655812
																			ESMQMQVEAER	10	7305503	1xTMT6plex [N-Term]; 1xOxidation [M	1.144	0.871	1.084	3.139336586	40.15	0	1582.740258	2	783.8760986	-0.269753075	-0.000211317	38.04178195
																			APVPGTPDSLSSGSSR	4	7305503	1xTMT6plex [N-Term]	0.643	0.991		3.940192699	30.66	0	1743.907458	2	872.4558716	-1.715220566	-0.001495593	43.84172372
																			DSNTILLPSNPGDVTSMVAQAMGVYGALTK	2	7305503	2xTMT6plex [N-Term; K30]				1.339798093	8.16	0	3508.843162	3	1170.283081	-2.414358678	-0.002823869	110.9192081
																			ILEPGLNILIPVLDR	2	7305503	1xTMT6plex [N-Term]	0.909			2.171940804	20.3	0	1904.1782	2	952.59198	-0.796642385	-0.000758475	109.4024971
46593007	UQCRC1	cytochrome b-c1 complex subunit 1, mitochondrial precursor	7384	NP_003356.2	31.66666667	1	10	10	92	480	52.612	6.37	0.91	0.96	1.15	1	Significant	0.0052																
																			LCTSATESEVAR	6	46593007	1xTMT6plex [N-Term]; 1xCarbamidomethyl [C2	1.401	1.391	1.713	4.609684944	66.42	0	1552.783838	2	776.8953857	-0.220691133	-0.000171343	40.6081091
																			AVELLGDIVQNCSLEDSQIEK	15	46593007	2xTMT6plex [N-Term; K21]; 1xCarbamidomethyl [C12	1.598	2.918	1.572	4.372302532	39.61	0	2818.490845	3	940.1674805	-1.049240147	-0.000985758	95.64683237
																			MVLAAAGGVEHQQLLDLAQK	6	46593007	2xTMT6plex [N-Term; K20]	1.555	1.059	100	5.485462666	52.4	0	2550.447799	3	850.819458	-1.559720832	-0.001325996	78.63054208
																			EVESMGAHLNAYSTR	12	46593007	1xTMT6plex [N-Term]	0.804	0.653	1.031	6.038747311	52.35	0	1893.932627	3	631.9763184	-9.622719213	-0.006074927	43.20682228
																			EVESMGAHLNAYSTR	4	46593007	1xTMT6plex [N-Term]; 1xOxidation [M5]	1.61	0.848		4.888905525	50.63	0	1909.927542	3	631.9763184	-9.622719213	-0.006074927	43.20682228
																			NALVSHLDGTTPVCEDIGR	4	46593007	1xTMT6plex [N-Term]; 1xCarbamidomethyl [C14		1.09		4.506610394	54.91	0	2283.16006	3	761.7261353	1.660882995	0.001264021	58.86861931
																			ADLTEYLSTHYK	6	46593007	2xTMT6plex [N-Term; K12]	1.222	1.716		2.981290102	34.01	0	1899.0264	3	633.680542	0.354379783	0.000224326	75.8857429
																			YIYDQCPAVAGYGPIEQLPDYNR	4	46593007	1xTMT6plex [N-Term]; 1xCarbamidomethyl [C6	0.638		100	2.962527275	19.03	0	2931.41846	3	977.812439	1.468202029	0.001434638	79.02680766
																			EMQENDASMR	8	46593007	1xTMT6plex [N-Term]	1.357	1.089		3.719898462	56.72	0	1439.645629	2	720.326355	-0.136109233	-9.80E-05	32.44183902
																			IAEVDASVVR	13	46593007	1xTMT6plex [N-Term]	0.813	1.082	1.074	3.510903597	63.07	0	1287.746982	2	644.3771973	0.105844744	6.82E-05	48.01016289
																			RIPLAEWESR	12	46593007	1xTMT6plex [N-Term]	0.901	0.721	1.024	4.83365345	52.82	1	1485.837528	3	495.9503174	-0.759848598	-0.000376337	52.46842177
																			EMQENDASMR	2	46593007	1xTMT6plex [N-Term]; 1xOxidation [M2]		1.113		2.098727465	25.74	0	1455.640544	2	720.326355	-0.136109233	-9.80E-05	32.44183902
34098946	YBX1	nuclease-sensitive element-binding protein	4904	NP_004550.2	45.37037037	1	6	8	77	324	35.903	9.88	1.72	1.52	1.37	1.54	Significant	0.0043																
																			NYQQNYQNSESGEKNEGSESAPEGQAQQR	4	34098946	2xTMT6plex [N-Term; K14]	1.915	100		4.376273632	19.75	1	3715.722071	4	929.6860962	0.13018617	0.000120934	33.13446634
																			EDVFVHQTAIK	6	34098946	2xTMT6plex [N-Term; K11]	2.465	2.45	2.802	4.298875332	63.87	0	1744.999791	2	873.0026245	-1.042273226	-0.000909383	44.98116996
																			AADPPAENSSAPEAEQGGAE	28	34098946	1xTMT6plex [N-Term]	1.656	1.653	1.515	3.387462139	58.16	0	2126.967551	3	709.6595459	-1.629867219	-0.001155558	27.76550235
																			GAEAANVTGPGGVPVQGSK	8	34098946	2xTMT6plex [N-Term; K19]	1.379	1.624	1.768	5.034312725	70.64	0	2154.191902	2	1077.598511	-1.001264383	-0.001078458	44.20876423
																			EDGNEEDKENQGDETQGQQPPQR	12	34098946	2xTMT6plex [N-Term; K8]	1.767	2.472	1.536	5.774454594	50.8	1	3086.429912	4	772.3618164	-1.450360166	-0.001119109	28.79873689
																			RPQYSNPPVQGEVMEGADNQGAGEQGRPVR	2	34098946	1xTMT6plex [N-Term]			0.841	6.263278484	25.71	0	3452.692677	4	863.928894	0.309735611	0.000267355	37.33451081
																			RPQYSNPPVQGEVMEGADNQGAGEQGRPVR	6	34098946	1xTMT6plex [N-Term]; 1xOxidation [M14]	0.66	0.774		5.014139175	22.9	0	3468.687592	4	863.928894	0.309735611	0.000267355	37.33451081
																			NDTKEDVFVHQTAIK	8	34098946	3xTMT6plex [N-Term; K4; K15]	1.572	1.176	1.099	4.045953751	21.93	1	2432.375235	4	608.848877	-0.640089357	-0.000389234	44.51677574
																			RPENPKPQDGK	3	34098946	3xTMT6plex [N-Term; K6; K11]	1.857	2.216		2.802950382	7.38	0	1953.14847	4	489.0424194	-0.318575692	-0.000155556	31.46732542
21361144	PSMC3	26S protease regulatory subunit 6A	5702	NP_002795.2	43.96355353	1	17	17	102	439	49.172	5.24	1.059	1.058	1.061	1.06	Non-Significant	1																
																			AMEVDERPTEQYSDIGGLDK	6	21361144	2xTMT6plex [N-Term; K20]	0.82	0.733	0.756	3.960199594	28.73	0	2711.359831	3	904.4591675	1.150043373	0.001039394	59.14244369
																			AVCVEAGMIALR	2	21361144	1xTMT6plex [N-Term]; 1xCarbamidomethyl [C3	0.956			3.725450754	46.42	0	1518.833371	2	759.9203491	0.03344158	2.54E-05	94.03556247
																			GATELTHEDYMEGILEVQAK	17	21361144	2xTMT6plex [N-Term; K20]	0.955	100	100	4.945150852	49.27	0	2692.390403	3	898.1353149	0.367210572	0.000329558	76.75904405
																			KMNVSPDVNYEELAR	2	21361144	2xTMT6plex [N-Term; K1]		0.687		5.281615734	25.91	1	2223.184374	3	741.7333984	0.570574584	0.000422831	61.05728425
																			LKPGDLVGVNK	13	21361144	3xTMT6plex [N-Term; K2; K11]	1.04	1.173	1.126	3.878442526	22.92	0	1827.16708	3	609.7280884	1.440233215	0.000877182	49.60350755
																			GATELTHEDYMEGILEVQAK	6	21361144	2xTMT6plex [N-Term; K20]; 1xOxidation [M11	100			3.370659828	19.82	0	2708.385318	3	898.1353149	0.367210572	0.000329558	76.75904405
																			APSIIFIDELDAIGTK	4	21361144	2xTMT6plex [N-Term; K16]				2.415952444	29.52	0	2161.252042	2	1081.129028	-0.58411946	-0.000631215	98.77792087
																			MSTEEIIQR	4	21361144	1xTMT6plex [N-Term]		0.546	1.071	3.055693865	31.99	0	1335.713967	2	668.3602295	-0.587360501	-0.000392273	42.63618406
																			KIEFPMPNEEAR	6	21361144	2xTMT6plex [N-Term; K1]	0.393	1.905	0.901	2.949488163	11.95	1	1919.046089	3	640.3532104	-0.526966157	-0.000337091	54.53206248
																			EKAPSIIFIDELDAIGTK	8	21361144	3xTMT6plex [N-Term; K2; K18]	100	0.769		2.315398455	19.48	1	2647.55253	3	883.1881714	-0.970522705	-0.000856503	109.9266186
																			QTYFLPVIGLVDAEK	3	21361144	2xTMT6plex [N-Term; K15]				1.874113441	10.98	0	2151.246563	2	1076.125977	-0.877019414	-0.000943342	104.4292147
																			DSYLILETLPTEYDSR	4	21361144	1xTMT6plex [N-Term]				1.581421018	19.31	0	2144.096046	2	1072.547485	-3.895388404	-0.004176043	84.40391261
																			VDILDPALLR	6	21361144	1xTMT6plex [N-Term]	1.167		100	2.639873743	23.27	0	1353.830317	2	677.4177856	-1.494013623	-0.00101132	82.3691453
																			VTHELQAMK	4	21361144	2xTMT6plex [N-Term; K9]	1.145		0.747	2.8253901	10.6	0	1514.876505	2	757.9411621	-0.962092451	-0.000728726	44.41121103
																			DAFALAK	6	21361144	2xTMT6plex [N-Term; K7]	1.077	1.14	0.978	2.989836454	42.91	0	1193.72943	2	597.3686523	0.500789781	0.000298904	53.07031867
																			MNVSPDVNYEELAR	6	21361144	1xTMT6plex [N-Term]		100	100	2.140633583	10.8	0	1865.926479	2	933.4658813	-1.067852732	-0.000996267	55.84444543
																			LLDSEIK	4	21361144	2xTMT6plex [N-Term; K7]	1.079	1.045		2.656933069	35.58	0	1275.792424	2	638.399353	-0.779848895	-0.000497463	63.99840479
																			EKFENLGIQPPK	1	21361144	3xTMT6plex [N-Term; K2; K12]		1.204		2.138989449		1	2087.246787	3	696.4221802	2.491405126	0.001733392	51.86010448
7657015	RTCB	tRNA-splicing ligase RtcB homolog	51493	NP_055121.1	40.59405941	1	15	15	92	505	55.175	7.23	1.07	1.07	1.09	1.08	Significant	0.000038																
																			NVTDVVNTCHDAGISK	6	7657015	2xTMT6plex [N-Term; K16]; 1xCarbamidomethyl [C9	1.261	1.434	1.151	4.894494057	64.99	0	2188.143237	3	730.0533447	1.025344068	0.000747867	45.48601631
																			GLPQLGTLGAGNHYAEIQVVDEIFNEYAAK	6	7657015	2xTMT6plex [N-Term; K30]	100	100		3.252794981	13.53	0	3675.942282	4	919.7409058	-0.132946354	-0.000122176	109.8961992
																			GMAAAGNYAWVNR	4	7657015	1xTMT6plex [N-Term]	1.551		100	3.997891903	56.89	0	1609.810661	2	805.4080811	-1.103061787	-0.00088786	48.53994531
																			EQLAQAMFDHIPVGVGSK	4	7657015	2xTMT6plex [N-Term; K18]	1.571	100		3.329129696	30	0	2385.300072	3	795.7697754	-2.221551991	-0.001766356	79.83838436
																			GQVCVMIHSGSR	24	7657015	1xTMT6plex [N-Term]; 1xCarbamidomethyl [C4]; 1xOxidation [M6]	1.35	1.233	1.176	4.095812798	52.87	0	1575.793297	3	520.6051025	1.520765257	0.000790696	25.72596583
																			EGYAWAEDKEHCEEYGR	4	7657015	2xTMT6plex [N-Term; K9]; 1xCarbamidomethyl [C12		0.63	0.734	5.413977623	19.35	1	2587.192372	4	647.5527344	-1.261823448	-0.000816145	42.8943538
																			LVMEEAPESYK	6	7657015	2xTMT6plex [N-Term; K11]	0.793	1.181	1.594	3.371895313	35.19	0	1753.944644	2	877.4754639	-0.566110013	-0.000496463	55.53769811
																			GQVCVMIHSGSR	14	7657015	1xTMT6plex [N-Term]; 1xCarbamidomethyl [C4	1.031	1.012	1.043	4.923627377	70.05	0	1559.798382	3	520.6051025	1.520765257	0.000790696	25.72596583
																			SYNDELQFLEK	2	7657015	2xTMT6plex [N-Term; K11]		100		2.115571737	25.43	0	1843.984201	2	922.4955444	-0.210605282	-0.000194176	67.9259428
																			LRPIAVIK	2	7657015	2xTMT6plex [N-Term; K8]	0.989			3.275375366	8.88	0	1367.950262	3	456.6557007	1.671377128	0.00076212	51.42193272
																			VFNTTPDDLDLHVIYDVSHNIAK	2	7657015	2xTMT6plex [N-Term; K23]		0.903		3.657694578		0	3084.640621	4	771.9156494	0.047634804	3.67E-05	95.32986128
																			GVIPMNAK	2	7657015	2xTMT6plex [N-Term; K8]	0.908			3.000530243	30.96	0	1287.785899	2	644.3960571	-0.824377863	-0.000530811	47.66992318
																			LMFEELR	6	7657015	1xTMT6plex [N-Term]				2.201488733	30.57	0	1166.644097	2	583.8249512	-1.260758319	-0.000735428	64.7376829
																			GGGVGGFLPAMK	6	7657015	2xTMT6plex [N-Term; K12]	100			2.06391573	28.59	0	1548.897241	2	774.9523926	0.173043274	0.000134013	78.10850106
																			NLDFQDVLDK	2	7657015	2xTMT6plex [N-Term; K10]				1.707062483	1	0	1664.925958	2	832.9649048	-2.056896087	-0.00171229	73.16833965
																			LADMGIAIR	2	7657015	1xTMT6plex [N-Term]	0.521			1.588074803	26.43	0	1188.697195	2	594.8518066	-0.721881698	-0.000429049	57.61543956
271398239	CNDP2	cytosolic non-specific dipeptidase isoform	55748	NP_060705.2	52.21052632	1	16	16	70	475	52.845	5.97	1.06	1.01	1.31	1.12	Significant	0.0067																
																			VYMGHGGKPWVSDFSHPHYLAGR	4	271398239	2xTMT6plex [N-Term; K8]			100	2.254868269	7.47	0	3056.57177	5	612.119873	-0.494559179	-0.000302331	56.35397729
																			YPSLSLHGIEGAFSGSGAK	6	271398239	2xTMT6plex [N-Term; K19]	1.077	0.791	0.01	5.449739456	36.87	0	2336.265067	3	779.4262695	-0.347279194	-0.000270445	83.02425684
																			GNILIPGINEAVAAVTEEEHK	6	271398239	2xTMT6plex [N-Term; K21]	0.814	0.89		4.319870472	37.9	0	2662.481601	3	888.1644287	-1.077302163	-0.000956099	99.73668751
																			DVGAQILLHSHK	4	271398239	2xTMT6plex [N-Term; K12]	1.033	0.98		4.538289547	44.81	0	1776.053224	3	592.6893921	0.22482408	0.0001331	44.50582763
																			GICYFFIEVECSNK	2	271398239	2xTMT6plex [N-Term; K14]; 2xCarbamidomethyl [C3; C11			1.389	3.379096508	43.46	0	2224.118269	3	742.0429077	-1.84281813	-0.001366215	101.6524421
																			QKLPDGSEIPLPPILLGR	4	271398239	2xTMT6plex [N-Term; K2]	1.278			3.465253353	16.24	1	2401.458287	3	801.1570435	-0.712087699	-0.000570016	88.67831208
																			MMEVAAADVK	6	271398239	2xTMT6plex [N-Term; K10]	1.154	1.144	0.95	3.614066601	55.59	0	1522.837342	2	761.9229126	0.792090711	0.000603113	51.94958261
																			DVDYVCISDNYWLGK	4	271398239	2xTMT6plex [N-Term; K15]; 1xCarbamidomethyl [C6	1.26			3.302561283	35.2	0	2305.15749	3	769.0568237	-0.682156686	-0.00052416	87.46448401
																			LVPNMTPEVVGEQVTSYLTK	4	271398239	2xTMT6plex [N-Term; K20]	0.7	1.427		2.857488632	23.84	0	2663.47301	3	888.4953003	-0.624275556	-0.000554247	101.3522034
																			EGGSIPVTLTFQEATGK	5	271398239	2xTMT6plex [N-Term; K17]	100			1.661104798	6.95	0	2193.21672	2	1097.110596	-1.278826541	-0.001402372	67.08011501
																			YNYIEGTK	4	271398239	2xTMT6plex [N-Term; K8]	0.953	1.003		3.21534729	40.31	0	1445.804052	2	723.4055176	-0.202713327	-0.000146542	53.09581437
																			WVAIQSVSAWPEK	6	271398239	2xTMT6plex [N-Term; K13]		100		1.759693861	5.4	0	1959.110404	2	980.0588379	-0.002566854	-2.51E-06	76.98670874
																			KKPCITYGLR	4	271398239	3xTMT6plex [N-Term; K1; K2]; 1xCarbamidomethyl [C4	1.257	1.45		3.067461252	2.67	1	1923.181685	4	481.5509033	0.051233459	2.46E-05	40.68775255
																			QLGGSVELVDIGK	4	271398239	2xTMT6plex [N-Term; K13]	0.01			2.19384551	20.22	0	1773.052221	2	887.0282593	-1.679970436	-0.001489338	67.46371123
																			LYDDIDFDIEEFAK	4	271398239	2xTMT6plex [N-Term; K14]				1.69606781	9.95	0	2191.121088	2	1096.062256	-1.758337899	-0.001926366	101.8953695
																			GSTDDKGPVAGWINALEAYQK	3	271398239	3xTMT6plex [N-Term; K6; K21]			100	1.641956449	0.15	1	2907.581933	3	969.8643188	-1.213945854	-0.001176549	88.24825398
23110935	PSMA1	proteasome subunit alpha type-1 isoform 1	5682	NP_683877.1	66.91449814	1	16	16	80	269	30.22	6.99	0.86	0.98	1.11	0.98	Significant	0.0053																
																			ETLPAEQDLTTK	4	23110935	2xTMT6plex [N-Term; K12]	0.917		0.975	3.959629297	53	0	1804.010415	2	902.5093994	0.613570806	0.000553444	59.34283422
																			AQPAQPADEPAEK	4	23110935	2xTMT6plex [N-Term; K13]	0.886	1.024		3.351178885	28.63	0	1809.974699	2	905.4907227	-0.292737517	-0.000264924	34.40276945
																			AQSELAAHQK	4	23110935	2xTMT6plex [N-Term; K10]		1.208	1.192	3.417333126	43.31	0	1540.884761	3	514.2996216	-0.292036054	-0.000149998	32.48854397
																			DLEFTIYDDDDVSPFLEGLEERPQR	8	23110935	1xTMT6plex [N-Term]	100	100		2.191075563	18.66	0	3227.558183	3	1076.524414	0.156656355	0.000168539	98.42272771
																			KAQPAQPADEPAEK	2	23110935	3xTMT6plex [N-Term; K1; K14]		0.896		4.337202072	12.92	1	2167.232593	3	723.0812378	-1.584219019	-0.001144457	28.7041254
																			FVFDRPLPVSR	5	23110935	1xTMT6plex [N-Term]	1.032	100		3.566433668	21.56	0	1561.905214	3	521.3065796	-0.017946332	-9.34E-06	68.84463954
																			HMSEFMECNLNELVK	7	23110935	2xTMT6plex [N-Term; K15]; 1xCarbamidomethyl [C8	0.873		100	2.497969627	11.21	0	2339.159815	3	780.3925171	1.360582338	0.001060873	79.80681566
																			THAVLVALK	6	23110935	2xTMT6plex [N-Term; K9]	1.035	1.144	0.894	4.564816952	61.88	0	1409.924442	2	705.4662476	0.551034626	0.000388459	47.63993474
																			NVSIGIVGK	6	23110935	2xTMT6plex [N-Term; K9]	0.788	0.905	1.536	3.955301762	42.37	0	1344.861507	2	672.9342651	-0.188358847	-0.000126658	56.56288584
																			AQPAQPADEPAEKADEPMEH	6	23110935	2xTMT6plex [N-Term; K13]	0.664	0.744	0.673	3.932196379	10.65	1	2619.276101	3	873.7630005	-0.631180178	-0.000551078	29.40611186
																			QGSATVGLK	2	23110935	2xTMT6plex [N-Term; K9]			1.369	3.624016523	39.12	0	1318.809471	2	659.9087524	0.573906109	0.000378436	39.29864071
																			LVSLIGSK	6	23110935	2xTMT6plex [N-Term; K8]	0.962	1.033	1.32	3.516243935	36.73	0	1274.844794	2	637.9257202	-0.494570253	-0.00031525	66.90739917
																			IHQIEYAMEAVK	6	23110935	2xTMT6plex [N-Term; K12]	1.957	100		2.312099218	28.15	0	1890.055926	2	945.5336914	2.211782436	0.002090196	70.01769407
																			RPYGVGLLIAGYDDMGPHIFQTCPSANYFDCR	4	23110935	1xTMT6plex [N-Term]; 2xCarbamidomethyl [C23; C31				2.105539083	14.41	0	3919.855585	4	980.7164917	-2.920628803	-0.002862111	89.78895575
																			AQPAQPADEPAEKADEPMEH	2	23110935	2xTMT6plex [N-Term; K13]; 1xOxidation [M18	0.761			3.913912058	4.39	1	2635.271016	3	873.7630005	-0.631180178	-0.000551078	29.40611186
																			LLCNFMR	4	23110935	1xTMT6plex [N-Term]; 1xCarbamidomethyl [C3	1.174	100	100	2.372479916	31.01	0	1182.632486	2	591.8196411	-0.40653664	-0.000240392	70.6320662
																			ADEPMEH	4	23110935	1xTMT6plex [N-Term]		0.918	0.641	2.260002613	23.88	0	1057.482176	2	529.2445679	-0.299851685	-0.000158544	22.95589017
5729991	PSMC4	26S protease regulatory subunit 6B isoform	5704	NP_006494.1	48.80382775	1	14	15	74	418	47.337	5.21	1.13	0.97	1.04	1.05	Significant	0.002																
																			ADTLDPALLRPGR	6	5729991	1xTMT6plex [N-Term]	1.356	1.296	1.101	3.648705721	30.5	0	1623.93797	3	541.9839478	-0.418808042	-0.000226706	49.39408222
																			GVLMYGPPGCGK	6	5729991	2xTMT6plex [N-Term; K12]; 1xCarbamidomethyl [C10	1.326	0.958	0.584	3.446594238	56.15	0	1693.91699	2	847.4609375	-1.411846043	-0.001195775	49.58853924
																			EAVELPLTHFELYK	6	5729991	2xTMT6plex [N-Term; K14]	0.9	1.205		3.917927027	38.02	0	2147.215263	3	716.4095459	-0.54890849	-0.000392875	88.93006896
																			ISGADINSICQESGMLAVR	3	5729991	1xTMT6plex [N-Term]; 1xCarbamidomethyl [C10			1.337	5.455245972	90.89	0	2250.141968	3	750.7178345	-1.340987402	-0.001005804	76.85094523
																			ELLKPNASVALHK	10	5729991	3xTMT6plex [N-Term; K4; K13]	1.082	1.088	1.05	4.570241451	22.11	0	2107.320621	4	527.5844116	-2.279736447	-0.001201034	47.7379679
																			EFLHAQEEVK	8	5729991	2xTMT6plex [N-Term; K10]	1.045	0.782	1.043	3.551412106	44.16	0	1687.941942	3	563.3186035	-0.405620068	-0.000228221	49.80160942
																			RFDAQTGADR	6	5729991	1xTMT6plex [N-Term]	0.848	1.286	0.852	5.076553345	34.8	1	1365.707242	3	455.9072876	0.049434437	2.25E-05	25.58586906
																			AQDEIPALSVSRPQTGLSFLGPEPEDLEDLYSR	2	5729991	1xTMT6plex [N-Term]				1.863760233	8.85	0	3858.959893	4	965.4911499	-4.437200982	-0.004280745	95.31185747
																			VVGSEFVQK	6	5729991	2xTMT6plex [N-Term; K9]	1.22	100	1.247	4.017798901	37.24	0	1450.866986	2	725.937439	0.423939405	0.00030754	59.81624679
																			KDEQEHEFYK	2	5729991	3xTMT6plex [N-Term; K1; K10]	1.223			3.953388214	18.11	1	2040.100517	4	510.7798462	-1.451842373	-0.000740476	38.80002297
																			LIFSTITSK	7	5729991	2xTMT6plex [N-Term; K9]	1.755	100	0.642	2.909932852	40.15	0	1467.918687	2	734.4619751	-1.371918421	-0.001006932	76.47732976
																			MNLSEEVDLEDYVARPDK	3	5729991	2xTMT6plex [N-Term; K18]		0.01		1.817997217	3.76	0	2581.321989	3	861.1105347	-1.913080241	-0.001646092	73.13382394
																			GVLMYGPPGCGK	2	5729991	2xTMT6plex [N-Term; K12]; 1xCarbamidomethyl [C10]; 1xOxidation [M4]			0.824	3.499701023	46.94	0	1709.911905	2	847.4609375	-1.411846043	-0.001195775	49.58853924
																			KIEFPLPDR	2	5729991	2xTMT6plex [N-Term; K1]	1.379			3.714431524	17.49	1	1572.951385	3	524.9887085	0.119335081	6.26E-05	62.23920247
																			VIMATNR	4	5729991	1xTMT6plex [N-Term]		1.144	1.096	2.463630438	30.76	0	1033.602566	2	517.3053589	0.846537602	0.000437492	35.76522847
																			ENAPAIIFIDEIDAIATK	1	5729991	2xTMT6plex [N-Term; K18]				1.010596991		0	2402.358298	2	1201.679443	-2.78401488	-0.003344101	111.4822762
728798446	AIM1	absent in melanoma 1 protein	202	NP_001615.2	13.00058038	1	16	16	58	1723	188.558	5.86	1.5	1.73	1.71	1.64	Significant	0.002																
																			AAGAPGASDADGLKPR	4	728798446	2xTMT6plex [N-Term; K14]	1.38		1.761	5.276669979	43.94	0	1912.065245	3	638.0228882	-5.822782067	-0.00371118	36.38782499
																			SPEAVGSECPSR	2	728798446	1xTMT6plex [N-Term]; 1xCarbamidomethyl [C9	1.219			3.833735704	42.89	0	1504.726323	2	752.866394	-0.539130638	-0.000405622	31.41911378
																			SLVLENVTDTAQDIPTTVDTK	2	728798446	2xTMT6plex [N-Term; K21]	100			3.191358089	27.77	0	2718.481326	3	906.8334961	1.695333054	0.001536244	69.99311694
																			DDAVFDDEVAPNAASDNASAEK	4	728798446	2xTMT6plex [N-Term; K22]	1.619	2.248		4.179301739	69.95	0	2709.289168	3	903.7678833	-0.026485787	-2.39E-05	61.26260152
																			ALEAVPAPPASGPR	6	728798446	1xTMT6plex [N-Term]	1.486	1.562	1.459	4.190296173	60.03	0	1561.889957	2	781.4488525	0.301626964	0.000235554	51.23170042
																			GIWVAYEKPGFTGHQYLLEEGEYR	2	728798446	2xTMT6plex [N-Term; K8]		100		4.436841011	25.92	0	3300.709369	4	825.9333496	0.666365091	0.000549869	80.0894778
																			LLEKEDSEAADSK	2	728798446	3xTMT6plex [N-Term; K4; K13]		1.663		3.577588558	28.67	1	2122.18464	3	708.065979	-0.592054177	-0.000418816	41.91017161
																			ASAEQSVLFK	4	728798446	2xTMT6plex [N-Term; K10]	1.864		100	3.7758286	57.51	0	1537.899015	2	769.4515991	-2.011157939	-0.001546479	55.96789474
																			SFVLPVESTQDVSSQVIPESSEVR	6	728798446	1xTMT6plex [N-Term]		100		2.522008181	19.48	0	2848.477748	3	950.1619263	-2.289914819	-0.002174257	76.99031682
																			GFYTSFEDWGGK	4	728798446	2xTMT6plex [N-Term; K12]	100		100	1.979757667	15.49	0	1851.931771	2	926.4680786	-1.560831496	-0.001445277	79.01063806
																			GTAAESGEEAAR	6	728798446	1xTMT6plex [N-Term]				2.485099316	36.75	0	1377.680752	2	689.3441162	0.147619014	0.000101686	24.30452227
																			LKPNLVLDIK	5	728798446	3xTMT6plex [N-Term; K2; K10]	1.726		100	2.894692898	15.71	0	1840.223867	3	614.0805054	1.682356879	0.001031971	71.00615598
																			VVIYSEPDVSEK	4	728798446	2xTMT6plex [N-Term; K12]		100		1.806007624	16.34	0	1823.020252	2	912.0136108	-0.168231983	-0.000153345	51.88235157
																			EDSEAADSK	2	728798446	2xTMT6plex [N-Term; K9]	100			3.335515738	22.4	0	1409.716024	2	705.3623657	1.014704584	0.000715223	27.7053647
																			GSSIDVLGIVANLK	3	728798446	2xTMT6plex [N-Term; K14]				2.158167362	12.99	0	1844.12572	2	922.5656738	-0.894105934	-0.000824422	95.15668265
																			VFTFGLGK	2	728798446	2xTMT6plex [N-Term; K8]	100			2.374224901	28.38	0	1326.81858	2	663.9127197	-0.314102381	-0.000208378	80.86758563
189458819	TFRC	transferrin receptor protein 1 isoform 1	7037	NP_001121620.1	27.23684211	1	18	18	61	760	84.818	6.61	1.37	1.33	2.22	1.64	Significant	0.03																
																			GFVEPDHYVVVGAQR	6	189458819	1xTMT6plex [N-Term]	1.366	1.886	1.957	5.526858807	32.89	0	1902.007112	3	634.6680908	-9.144560476	-0.005797673	48.79379629
																			VEYHFLSPYVSPK	4	189458819	2xTMT6plex [N-Term; K13]	2.009	2.179		3.998048782	26.88	0	2024.12572	3	675.3799438	-0.21816186	-0.000147196	72.85439786
																			HPVTGQFLYQDSNWASK	4	189458819	2xTMT6plex [N-Term; K17]			4.176	3.29852128	41.66	0	2436.271215	3	812.762085	0.199876274	0.000162318	66.31785358
																			LLNENSYVPR	4	189458819	1xTMT6plex [N-Term]	0.127	0.042		3.609407187	45.57	0	1433.794994	2	717.4017334	0.83400129	0.000597893	58.68072622
																			LTTDFGNAEK	4	189458819	2xTMT6plex [N-Term; K10]	1.304	2.232		4.418911457	52.02	0	1553.857544	2	777.4312744	-1.461814749	-0.001135726	58.08602941
																			LAVDEEENADNNTK	6	189458819	2xTMT6plex [N-Term; K14]	2.471	2.198	1.525	3.47134757	40.63	0	2020.023499	2	1010.515564	0.174388906	0.000176135	39.61668503
																			LVHANFGTK	4	189458819	2xTMT6plex [N-Term; K9]	1.368	1.392		3.285803795	42.53	0	1444.867655	3	482.2939453	-0.257658547	-0.000124094	42.04178098
																			AVLGTSNFK	6	189458819	2xTMT6plex [N-Term; K9]	1.924	2.689	2.339	3.927750349	54.11	0	1394.840772	2	697.9237061	-0.45598081	-0.00031801	48.50835324
																			EAGSQKDENLALYVENQFR	2	189458819	2xTMT6plex [N-Term; K6]				2.482338905	15.44	1	2669.393514	3	890.4685669	-0.886724416	-0.000789005	82.50500078
																			AAAEVAGQFVIK	2	189458819	2xTMT6plex [N-Term; K12]			100	1.887890339	34.41	0	1661.999063	2	831.5026245	-0.656159553	-0.000545268	71.11310796
																			SAFSNLFGGEPLSYTR	5	189458819	1xTMT6plex [N-Term]	100			1.757475853	14.61	0	1975.012257	2	988.0093994	-0.372104969	-0.000367456	84.85249372
																			LTHDVELNLDYER	2	189458819	1xTMT6plex [N-Term]	100			3.313688278	17.52	0	1845.954408	3	615.9897461	0.150123562	9.24E-05	54.83372593
																			DENLALYVENQFR	2	189458819	1xTMT6plex [N-Term]				1.693062305	11.34	0	1839.943843	2	920.4737549	-1.96207313	-0.001805052	90.65630889
																			LAQMFSDMVLK	2	189458819	2xTMT6plex [N-Term; K11]	100			1.911487937	14.6	0	1740.979255	2	870.9925537	-0.818325733	-0.000712344	104.5973255
																			EEPGEDFPAAR	2	189458819	1xTMT6plex [N-Term]	1.79			3.115823746	12.76	0	1446.706239	2	723.8569336	0.24306766	0.000175824	42.96039598
																			VSASPLLYTLIEK	1	189458819	2xTMT6plex [N-Term; K13]				1.216319084		0	1892.150872	2	946.5787964	-0.293764398	-0.000277923	89.55640887
																			MVTSESK	4	189458819	2xTMT6plex [N-Term; K7]	2.082	2.162		2.854114771	33.35	0	1239.701895	2	620.354187	-0.643232511	-0.000398708	38.33621095
																			LYWDDLKR	1	189458819	2xTMT6plex [N-Term; K7]	1.163			3.166884184	2.11	1	1566.904434	3	522.9726563	-0.650231104	-0.000339617	69.79206745
4507943	XPO1	exportin-1	7514	NP_003391.1	19.98132586	1	18	18	78	1071	123.306	6.06	1.25	1.51	1.28	1.35	Significant	0.0037																
																			LDINLLDNVVNCLYHGEGAQQR	6	4507943	1xTMT6plex [N-Term]; 1xCarbamidomethyl [C12			100	2.773532391	33.45	0	2770.414377	3	924.1417847	-1.291029285	-0.001192229	103.2537714
																			ETLVYLTHLDYVDTER	6	4507943	1xTMT6plex [N-Term]			2.579	4.125074387	32.32	0	2196.13858	3	732.7171021	-0.831818025	-0.000608929	82.68089943
																			ICLEYWNHLAAELYR	2	4507943	1xTMT6plex [N-Term]; 1xCarbamidomethyl [C2			1.636	3.535352707	15.26	0	2180.116011	3	727.3759155	-1.29243029	-0.000939216	98.58882914
																			SAFPHLQDAQVK	4	4507943	2xTMT6plex [N-Term; K12]	1.427		1.271	3.792428732	41.01	0	1799.021589	3	600.3461914	1.351700705	0.00081058	49.69836948
																			DTDSINLYK	2	4507943	2xTMT6plex [N-Term; K9]	1.318			3.95993042	55.32	0	1526.846645	2	763.9257813	-1.54485063	-0.001179375	58.74958442
																			DLLGLCEQK	5	4507943	2xTMT6plex [N-Term; K9]; 1xCarbamidomethyl [C6	1.404			3.225066185	26.36	0	1533.871086	2	767.4370728	-2.749059049	-0.002108351	70.1804018
																			YMLLPNQVWDSIIQQATK	8	4507943	2xTMT6plex [N-Term; K18]	100			2.146065474	12.58	0	2606.441651	3	869.4852905	-0.127540921	-0.000110809	110.7023977
																			AVGHPFVIQLGR	8	4507943	1xTMT6plex [N-Term]	1.048	1.175	1.041	3.690191507	31.49	0	1522.905548	3	508.3074341	1.445313596	0.000733692	56.2358588
																			QLLDFSQK	4	4507943	2xTMT6plex [N-Term; K8]	1.224			3.307764769	41.08	0	1436.851336	2	718.9283447	-1.338577504	-0.000961668	64.36204946
																			YYGLQILENVIK	5	4507943	2xTMT6plex [N-Term; K12]				1.40592134	9.87	0	1911.135556	2	956.0696411	-1.857902453	-0.001775352	97.75073953
																			IYLDMLNVYK	5	4507943	2xTMT6plex [N-Term; K10]	100			1.889304638	11.2	0	1729.996286	2	865.5003662	-1.635817457	-0.001414979	88.02287011
																			QLYLPMLFK	4	4507943	2xTMT6plex [N-Term; K9]	1.154	1.775		2.495880842	20.97	0	1610.974428	2	805.989563	-1.600803459	-0.001289427	103.3183843
																			QMSVPGIFNPHEIPEEMCD	2	4507943	1xTMT6plex [N-Term]; 1xCarbamidomethyl [C18				1.158766508	4.4	0	2459.124269	2	1230.064331	-1.172401355	-0.00144154	78.5880079
																			QLGSILK	4	4507943	2xTMT6plex [N-Term; K7]			0.709	2.334218264	33.43	0	1216.80293	2	608.9053345	0.380337112	0.000231398	56.0294389
																			DFLVQIK	4	4507943	2xTMT6plex [N-Term; K7]	1.168			2.897847891	37.69	0	1320.829144	2	660.9189453	1.112751794	0.000734783	70.40658615
																			NQCEGIK	4	4507943	2xTMT6plex [N-Term; K7]; 1xCarbamidomethyl [C3	2.575	1.738		2.971687555	29.92	0	1306.718942	2	653.8627319	-0.577716285	-0.000377456	31.5850962
																			EFAGEDTSDLFLEER	2	4507943	1xTMT6plex [N-Term]			100	1.295653939	4.78	0	1986.949382	2	993.9819336	3.627957292	0.003604284	74.44092674
																			FLVTVIK	3	4507943	2xTMT6plex [N-Term; K7]	100			2.071843624	20.98	0	1277.859716	2	639.4338989	0.630031257	0.000402546	99.96299481
21735621	MDH2	malate dehydrogenase, mitochondrial isoform 1 precursor	4191	NP_005909.2	62.13017751	1	14	14	109	338	35.481	8.68	0.75	0.7	0.66	0.7	Significant	0.0014																

Bhat et al,2017. Cigarette smoke and chewing tobacco alter expression of different sets of miRNAs in oral keratinocytes

Supplementary Table S2(A). List of all proteins and peptides identified in OKF6/TERT1 cells chronically treated with chewing tobacco using Sequest and Mascot search algorithms

Accession	Gene_Symbol	Description	Gene_ID	Refseq_Accession	Coverage	Proteins	#Unique_Peptides	#Peptides	#PSMS	#AAs	MW [kDa]	calc. pI	OKF6-STE-6M/OKF6P_R1	OKF6-STE-6M/OKF6P_R2	OKF6-STE-6M/OKF6P_R3	Median fold change	Status	P-value (6M)	Sequence	#PSMs	Protein Accessions	Modifications	OKF6-STE-6M/OKF6P_R1	OKF6-STE-6M/OKF6P_R2	OKF6-STE-6M/OKF6P_R3	Xcorr	Ionscore	# Missed Cleavages	Theo. MH+ [Da]	Charge	m_z	DeltaM [ppm]	Deltam/z [Da]	RT [min]
																			AGAGSATLSMAYAGAR	8	21735621	1xTMT6plex [N-Term]	1.072	1.134	0.786	5.312167168	82.68	0	1683.86857	2	842.4377441	-0.212753392	-0.000179124	45.14703919
																			GCDVVVIPAGVPR	19	21735621	1xTMT6plex [N-Term]; 1xCarbamidomethyl [C2	0.825	0.576	0.72	3.800485849	44.84	0	1567.882764	2	784.4471436	2.708537573	0.002123335	58.77303811
																			VAVLGASGGIGQPLSLLLK	8	21735621	2xTMT6plex [N-Term; K19]	0.445	0.977		3.323228598	46.73	0	2251.41536	2	1126.208496	-2.506966329	-0.002822111	106.0533058
																			VNVPVIGGHAGK	4	21735621	2xTMT6plex [N-Term; K12]		0.808	0.702	4.783116817	68.62	0	1605.984082	3	536.0001831	1.192070599	0.000638149	50.9554058
																			DDLFNTNATIVATLTAACAQHCPEAMICVIANPVNSTIPITAEVFK	2	21735621	2xTMT6plex [N-Term; K46]; 3xCarbamidomethyl [C18; C22; C28]		100		3.80601573	26.89	0	5460.7716	4	1365.946899	-1.067992617	-0.001458016	117.5367082
																			LTLYDIAHTPGVAADLSHIETK	8	21735621	2xTMT6plex [N-Term; K22]	0.776	0.757		5.557741165	55.54	0	2823.565665	4	706.6473389	0.658911097	0.00046512	93.81051112
																			IQEAGTEVVK	8	21735621	2xTMT6plex [N-Term; K10]	0.7	0.627	0.305	4.467795372	52.1	0	1531.909579	2	766.458252	-0.229748384	-0.000175977	46.0953277
																			EGVVECSFVK	2	21735621	2xTMT6plex [N-Term; K10]; 1xCarbamidomethyl [C6	1.269			3.728576183	41.35	0	1611.88165	2	806.4442749	-0.233928659	-0.000188533	60.76088006
																			TIIPLISQCTPK	11	21735621	2xTMT6plex [N-Term; K12]; 1xCarbamidomethyl [C9	0.66	100	0.663	3.826887608	44.83	0	1829.097063	2	915.0513916	-0.850734992	-0.000778038	72.77819296
																			ANTFVAELK	6	21735621	2xTMT6plex [N-Term; K9]	0.953	1.475	0.582	4.230772495	46.77	0	1450.866986	2	725.9369507	-0.249149519	-0.000180741	71.67796932
																			IFGVTTLDIVR	8	21735621	1xTMT6plex [N-Term]	0.697	0.827	1.188	3.089595318	34.53	0	1462.883081	2	731.9450073	-0.234531089	-0.000171546	79.7396683
																			MISDAIPELK	16	21735621	2xTMT6plex [N-Term; K10]	1.051	0.381	1.066	3.057816982	28.4	0	1574.922786	2	787.9666138	2.009259812	0.001582215	74.05021072
																			GYLGPEQLPDCLK	7	21735621	2xTMT6plex [N-Term; K13]; 1xCarbamidomethyl [C11	0.615	0.894	0.635	2.842473745	22.97	0	1948.061405	2	974.5343628	0.022379139	2.18E-05	61.85846368
																			VSSFEEK	2	21735621	2xTMT6plex [N-Term; K7]			0.796	2.95439291	24.95	0	1283.724739	2	642.3667603	1.172500433	0.000752584	48.89951783
4504327	HADHB	trifunctional enzyme subunit beta, mitochondrial isoform 1 precursor	3032	NP_000174.1	39.02953586	1	18	18	97	474	51.262	9.41	0.67	0.69	0.59	0.65	Significant	0.0022																
																			EAALGAGFSDK	6	4504327	2xTMT6plex [N-Term; K11]	0.717	0.855	0.758	4.485950947	59.14	0	1523.846979	2	762.4277344	0.796096994	0.000606565	49.99989025
																			EVVDYIIFGTVIQEVK	17	4504327	2xTMT6plex [N-Term; K16]	0.75	0.676	0.608	3.99967289	49.1	0	2310.336106	3	770.7821045	-1.881177992	-0.001448718	106.3206817
																			TPFLLSGTSYK	4	4504327	2xTMT6plex [N-Term; K11]	0.572		0.689	3.785498857	44.75	0	1671.972179	2	836.489624	-0.124435758	-0.000104027	70.34824901
																			DFMYVSQDPK	4	4504327	2xTMT6plex [N-Term; K10]	0.756	0.739		2.872452497	40.84	0	1687.876565	2	844.4390869	-3.357669627	-0.002833666	57.01081822
																			ALAMGYKPK	6	4504327	3xTMT6plex [N-Term; K7; K9]	0.604	0.617	0.597	3.159867525	21.36	0	1666.032895	3	556.015686	-0.23428981	-0.000130112	37.94683182
																			DQLLLGPTYATPK	6	4504327	2xTMT6plex [N-Term; K13]		1.844	0.707	2.302338839	18.68	0	1875.099171	2	938.0523682	-0.912528735	-0.000855541	59.41050502
																			LAAAFAVSR	6	4504327	1xTMT6plex [N-Term]	0.568	0.538	0.648	3.388242245	46.23	0	1134.683259	2	567.8453979	0.229137458	0.000129999	53.63948081
																			AAPAVQTK	6	4504327	2xTMT6plex [N-Term; K8]	0.589	1.223	0.79	3.423434496	45.27	0	1243.777443	2	622.3921509	-0.335994345	-0.000208951	33.8109478
																			LEQDEYALR	8	4504327	1xTMT6plex [N-Term]	0.878	0.784	0.814	3.52643466	43.71	0	1365.721161	2	683.364502	0.41482571	0.000283268	51.37337116
																			KAQDEGLLSDVVPFK	2	4504327	3xTMT6plex [N-Term; K1; K15]		0.999		2.745966911	23.42	1	2333.368359	3	778.4613037	0.428240491	0.000333081	70.35704472
																			AMDSDWFAENYMGR	4	4504327	1xTMT6plex [N-Term]	0.01			1.674209595	13.32	0	1921.841034	2	961.4240723	-0.086666247	-8.33E-05	90.19509763
																			FNFLAPELPAVSEFSTSETMGHSADR	4	4504327	1xTMT6plex [N-Term]				1.429671645	4.09	0	3069.482516	3	1023.829163	-3.121561039	-0.003193859	89.78092145
																			LMLDLNK	4	4504327	2xTMT6plex [N-Term; K7]	0.731		0.84	3.310153484	25.47	0	1304.801215	2	652.9036865	-0.857236422	-0.000559262	62.54694312
																			LVMAAANR	6	4504327	1xTMT6plex [N-Term]	0.992	0.737	0.849	2.915930748	55.55	0	1074.629115	2	537.8187256	0.985727876	0.000529646	38.85228986
																			AQDEGLLSDVVPFK	4	4504327	2xTMT6plex [N-Term; K14]				1.768768549	5.52	0	1976.110464	2	988.5563354	-2.565343211	-0.002534701	82.73568747
																			AALTGLLHR	3	4504327	1xTMT6plex [N-Term]	0.596		0.84	2.628758669	17.51	0	1180.736357	2	590.8713989	-0.708226212	-0.000418114	46.90356861
																			DLMPHDLAR	2	4504327	1xTMT6plex [N-Term]			0.46	2.585304737	30.92	0	1296.693172	3	432.9025879	0.029661508	1.28E-05	45.9789989
																			KLMLDLNK	5	4504327	3xTMT6plex [N-Term; K1; K8]	0.96	0.717	0.01	2.152802944	9.17	1	1662.05911	3	554.6912842	0.113942435	6.31E-05	51.21470017
19923315	SHMT2	serine hydroxymethyltransferase, mitochondrial isoform 1 precursor	6472	NP_005403.2	42.85714286	1	16	16	70	504	55.958	8.53	1.1	1.25	1.41	1.25	Significant	0.0051																
																			GLELIASENFCSR	4	19923315	1xTMT6plex [N-Term]; 1xCarbamidomethyl [C11	1.054	0.881		3.564384937	58.74	0	1724.883886	2	862.9448853	-0.807023707	-0.000696011	76.02421276
																			GWTGQESLSDSDPEMWELLQR	11	19923315	1xTMT6plex [N-Term]	0.85	100		4.453126907	33.28	0	2693.271461	3	898.4282837	-0.431837562	-0.000387685	98.7272833
																			HADIVTTTTHK	4	19923315	2xTMT6plex [N-Term; K11]	1.219	1.235		4.537620544	50.5	0	1681.96374	3	561.3254395	-1.17416956	-0.000658304	28.88008395
																			LIIAGTSAYAR	6	19923315	1xTMT6plex [N-Term]	0.978		2.06	3.405258894	40.92	0	1364.809916	2	682.9086304	0.049752121	3.40E-05	64.86501399
																			VLELVSITANK	6	19923315	2xTMT6plex [N-Term; K11]		1.116		3.214426041	49.78	0	1645.030029	2	823.0185547	-0.119107248	-9.80E-05	90.57903105
																			ALEAFDLDPAQWGVNVQPYSGSPANLAVYTALLQPHDR	2	19923315	1xTMT6plex [N-Term]				2.111810446	7.74	0	4353.21415	4	1089.057739	-1.15387357	-0.001255765	102.7824811
																			AALEALGSCLNNK	4	19923315	2xTMT6plex [N-Term; K13]; 1xCarbamidomethyl [C9	0.615	0.763		2.568591356	38.9	0	1819.01479	2	910.0105591	-0.52117	-0.000474008	62.53121607
																			IMGLDLPDGGHLTHGYMSDVK	4	19923315	2xTMT6plex [N-Term; K21]			100	2.170976162	9.64	0	2714.404613	4	679.3499146	-9.867702385	-0.006696234	67.08604781
																			AMADALLER	4	19923315	1xTMT6plex [N-Term]	1.07	100		3.393517017	50.96	0	1218.671374	2	609.8391724	-0.250923624	-0.000152897	65.03813218
																			AHLLADMAHISGLVAAK	3	19923315	2xTMT6plex [N-Term; K17]	0.01			3.104415655	20.48	0	2176.26765	3	726.0938721	-0.269644711	-0.000195606	96.67316955
																			LGAPALTSR	4	19923315	1xTMT6plex [N-Term]		1.33	1.396	3.018536568	39.89	0	1114.678174	2	557.842041	-1.227680583	-0.000684234	37.25421116
																			EYSLQVLK	4	19923315	2xTMT6plex [N-Term; K8]	0.712	100		2.179045439	25.89	0	1437.871737	2	719.4403687	1.198594174	0.000861712	53.16768539
																			YSEGYPGK	2	19923315	2xTMT6plex [N-Term; K8]		1.186		3.215510368	35.11	0	1358.735638	2	679.8723755	1.35170265	0.000918303	34.86811556
																			GWTGQESLSDSDPEMWELLQR	2	19923315	1xTMT6plex [N-Term]; 1xOxidation [M15]				1.528285623	3.4	0	2709.266376	3	898.4282837	-0.431837562	-0.000387685	98.7272833
																			AFPMPGFDEH	4	19923315	1xTMT6plex [N-Term]	1.974	0.62		1.65491128	7.47	0	1376.650638	2	688.8286133	-0.500125072	-0.000344249	71.92692393
																			EIPYTFEDR	2	19923315	1xTMT6plex [N-Term]		1.892		2.852333069	28.66	0	1398.710262	2	699.8583984	-0.530106213	-0.000370732	50.52920473
																			EVCDEVK	4	19923315	2xTMT6plex [N-Term; K7]; 1xCarbamidomethyl [C3	1.177	1.128		3.123735666	33.16	0	1336.718273	2	668.8630981	0.483459436	0.000323125	38.34260844
15149465	CALD1	caldesmon isoform 5	800	NP_149131.1	33.64661654	1	15	15	70	532	61.176	6.71	0.87	0.96	0.97	0.93	Significant	0.0012																
																			SAKPTKPAASDLPVPAEGVR	7	15149465	3xTMT6plex [N-Term; K3; K6]	1.033		0.977	5.804100037	33.61	0	2678.580811	4	670.4002686	-0.584828818	-0.000391628	43.24817571
																			GNVFSSPTAAGTPNK	6	15149465	2xTMT6plex [N-Term; K15]	1.254	1.155	1.369	4.773547649	64.34	0	1906.043447	2	953.524353	-1.058394191	-0.001008673	42.37493119
																			STHQAAIVSK	10	15149465	2xTMT6plex [N-Term; K10]	0.789	0.912	0.859	4.689799309	56.86	0	1499.894598	2	750.4508057	-0.175486914	-0.000131606	32.54657704
																			YEIEETETVTK	8	15149465	2xTMT6plex [N-Term; K11]	1.081	1.04	1.09	3.841596127	49.16	0	1799.967882	2	900.4885254	1.051330565	0.000946181	53.36885115
																			GETESEEFEK	8	15149465	2xTMT6plex [N-Term; K10]	0.86	0.956	0.981	3.948645353	51.36	0	1642.821218	2	821.9093628	-5.946273373	-0.004884332	38.50926348
																			LEQYTSAIEGTK	6	15149465	2xTMT6plex [N-Term; K12]		100		2.697098255	28.21	0	1797.999851	2	899.5020752	-1.655667198	-0.001488445	62.06876043
																			MQNDTAENETTEKEEK	4	15149465	3xTMT6plex [N-Term; K13; K16]	1.454	1.612		3.679894447	25.7	1	2584.301537	3	862.105896	0.618259231	0.000532589	39.4665036
																			GSIGENQIKDEK	2	15149465	3xTMT6plex [N-Term; K9; K12]			0.975	2.684537649	20.1	1	2005.153281	3	669.0552979	-0.967654359	-0.000646765	34.83002343
																			SLAALSQIAYQR	2	15149465	1xTMT6plex [N-Term]	0.244			1.978955746	27.58	0	1549.889957	2	775.4486694	0.06768041	5.24E-05	79.06098938
																			QQEAALELEELKK	2	15149465	3xTMT6plex [N-Term; K12; K13]				1.945114851	12.49	1	2216.310509	3	739.4411011	-0.793867708	-0.000586486	64.49832613
																			KVLEEEEQR	2	15149465	2xTMT6plex [N-Term; K1]	1.002			3.188716173	27.01	1	1617.921206	3	539.9785156	-0.131573156	-7.10E-05	34.8988667
																			MPEDGLSDDKKPFK	3	15149465	4xTMT6plex [N-Term; K10; K11; K14]	0.974	1.228	1.29	2.783302069		1	2523.429862	4	631.6132813	0.567897694	0.000358263	54.28171974
																			EFDPTITDASLSLPSR	6	15149465	1xTMT6plex [N-Term]	2.037	1.409	100	2.102810621	19.91	0	1978.033052	2	989.519165	-1.01040368	-0.000999306	65.76535549
																			INEWLTK	2	15149465	2xTMT6plex [N-Term; K7]		100		1.768449903	13.88	0	1361.819308	2	681.4123535	-1.378603416	-0.000938704	63.01333022
																			QKEFDPTITDASLSLPSR	2	15149465	2xTMT6plex [N-Term; K2]	100			1.611050487	1.67	1	2463.349524	3	821.7877808	-0.298550748	-0.000245145	57.53392612
5453543	AKR1C1	aldo-keto reductase family 1 member C1	1645	NP_001344.2	58.82352941	1	1	13	82	323	36.765	7.88	1.45	1.46	1.61	1.5	Significant	0.0012																
																			HIDSAHLYNNEEQVGLAIR	8	5453543	1xTMT6plex [N-Term]	1.427	1.437	1.453	6.493859768	52.99	0	2408.251987	3	803.4220581	-0.151804544	-0.000121861	46.86134649
																			LNDGHFMPVLGFGTYAPAEVPK	4	5453543	2xTMT6plex [N-Term; K22]	3.475	2.789		3.789035797	28.41	0	2818.500228	3	940.1703491	-1.324848826	-0.001244696	91.85483379
																			NLQLDYVDLYLIHFPVSVKPGEEVIPK	4	5453543	3xTMT6plex [N-Term; K19; K27]	100		100	2.714096069	9.98	0	3813.180945	4	954.0484009	-2.405203033	-0.002292869	104.4364345
																			EEPWVDPNSPVLLEDPVLCALAK	10	5453543	2xTMT6plex [N-Term; K23]; 1xCarbamidomethyl [C19		1.786	5.079	3.841878414	44.05	0	3049.63203	3	1017.212952	-2.534276088	-0.002576203	100.4847844
																			ILFDTVDLCATWEAVEK	2	5453543	2xTMT6plex [N-Term; K17]; 1xCarbamidomethyl [C9			1.745	3.949516296	52.81	0	2468.314719	3	823.4428101	-0.341254789	-0.000280775	104.6700335
																			YKPVCNQVECHPYFNQR	22	5453543	2xTMT6plex [N-Term; K2]; 2xCarbamidomethyl [C5; C10	1.723	1.672	1.669	5.729713917	42.99	0	2697.343017	4	675.0913696	0.234262243	0.000157971	39.59018973
																			DIVLVAYSALGSHR	4	5453543	1xTMT6plex [N-Term]	2.392	100		3.547852278	37.74	0	1729.979835	3	577.3311157	-0.601736512	-0.000346997	80.78086362
																			QLEMILNKPGLK	2	5453543	3xTMT6plex [N-Term; K8; K12]		100		2.294942617	8.25	0	2071.291629	3	691.1014404	-0.898377086	-0.000620267	77.55351676
																			SIGVSNFNR	4	5453543	1xTMT6plex [N-Term]		1.377		3.32353282	53.11	0	1222.674151	2	611.8401489	-0.924079761	-0.000564924	47.14993944
																			EDIFYTSK	6	5453543	2xTMT6plex [N-Term; K8]	1.723	100	2.939	3.184840202	29.5	0	1460.803717	2	730.9067383	1.699538734	0.001241346	51.61610646
																			LAIEAGFR	8	5453543	1xTMT6plex [N-Term]		1.732	1.498	2.801836014	33.94	0	1105.65671	2	553.3306274	-2.470863843	-0.001365964	52.49389932
																			GVVVLAK	6	5453543	2xTMT6plex [N-Term; K7]	1.359	1.369	1.478	3.208216906	43.61	0	1143.786551	2	572.3959961	-1.604908274	-0.000917836	55.74388556
																			KLLDFCK	4	5453543	3xTMT6plex [N-Term; K1; K7]; 1xCarbamidomethyl [C6	1.17	100		3.126327753	16.63	1	1610.990696	3	537.6692505	1.552997698	0.000833955	60.24168333
32455266	PRDX1	peroxiredoxin-1	5052	NP_859048.1	76.88442211	1	12	15	162	199	22.096	8.13	1.09	1.18	1.11	1.13	Significant	0.00059																
																			HGEVCPAGWKPGSDTIKPDVQK	8	32455266	4xTMT6plex [N-Term; K10; K17; K22]; 1xCarbamidomethyl [C5	1.351	1.656	1.6	5.340525627	21.54	0	3322.838782	4	831.4644165	-0.886661848	-0.000736559	44.78111339
																			KQGGLGPMNIPLVSDPK	12	32455266	3xTMT6plex [N-Term; K1; K17]	0.963	1.268	0.615	4.505271435	35.14	1	2438.440812	3	813.4833984	-2.120210371	-0.001723336	60.91060462
																			QITVNDLPVGR	13	32455266	1xTMT6plex [N-Term]	1.096	1.304	1.277	3.191392183	49.7	0	1440.837194	2	720.9219971	-0.330376934	-0.00023801	50.46492346
																			TIAQDYGVLK	16	32455266	2xTMT6plex [N-Term; K10]	1.08	1.413	1.161	4.114012241	59.74	0	1565.930315	2	783.470459	2.124391308	0.001663324	61.24453231
																			KLNCQVIGASVDSHFCHLAWVNTPK	2	32455266	3xTMT6plex [N-Term; K1; K25]; 2xCarbamidomethyl [C4; C16			1.097	3.952134848	9.42	1	3568.912409	5	714.5872192	-1.518526647	-0.001083898	76.35809451
																			ATAVMPDGQFK	34	32455266	2xTMT6plex [N-Term; K11]	0.96	1.226	1.452	4.052209377	57.67	0	1622.897634	2	811.9523315	-0.152753974	-0.000123952	53.33486435
																			LNCQVIGASVDSHFCHLAWVNTPK	2	32455266	2xTMT6plex [N-Term; K24]; 2xCarbamidomethyl [C3; C15			100	2.34747839	4.16	0	3211.654514	4	803.6692505	0.204836206	0.000164466	83.39502684
																			ATAVMPDGQFK	2	32455266	2xTMT6plex [N-Term; K11]; 1xOxidation [M5			1.191	2.737766981	52.36	0	1638.892549	2	811.9523315	-0.152753974	-0.000123952	53.33486435
																			LVQAFQFTDK	10	32455266	2xTMT6plex [N-Term; K10]	1.246	0.898	0.543	2.308000326	37.91	0	1654.956864	2	827.9821777	0.129948251	0.000107529	67.77411515
																			GLFIIDDK	25	32455266	2xTMT6plex [N-Term; K8]	0.918	0.972	0.829	3.583465338	50.12	0	1378.834624	2	689.9197388	-1.75698441	-0.001211295	73.26184271
																			DISLSDYK	12	32455266	2xTMT6plex [N-Term; K8]	1.288	1.278	1.331	3.457821369	46.22	0	1398.788067	2	699.8981323	0.658311619	0.000460419	52.24986883
																			QGGLGPMNIPLVSDPK	4	32455266	2xTMT6plex [N-Term; K16]	100	100		1.810313702	11.75	0	2081.182917	2	1041.094482	-0.590474888	-0.000614443	68.5625444
																			IGHPAPNFK	4	32455266	2xTMT6plex [N-Term; K9]	1.228	1.329		2.806742907	26.18	0	1438.85709	3	480.2901917	-0.742678919	-0.000356203	45.12514155
																			ADEGISFR	6	32455266	1xTMT6plex [N-Term]	1.084	1.103	1.044	3.125806093	36.22	0	1123.594504	2	562.3015137	1.109754227	0.000623457	42.28683237
																			SKEYFSK	5	32455266	3xTMT6plex [N-Term; K2; K7]	0.547	0.63	0.681	2.481574297	15.86	1	1575.934955	3	525.9829102	-0.493707718	-0.00025935	41.76937599
																			SVDETLR	7	32455266	1xTMT6plex [N-Term]	1.093	1.154	100	3.049638271	31.39	0	1048.583605	2	524.7949829	-0.8731585	-0.00045779	36.4327287
92859701	MYO6	unconventional myosin-VI isoform 1	4646	NP_004990.3	17.9766537	1	19	19	64	1285	148.62	8.53	1.03	0.99	0.57	0.86	Significant	0.028																
																			QKEEESQQQAVLEQER	6	92859701	2xTMT6plex [N-Term; K2]	1.032	1.164	0.761	5.062793732	29.57	1	2417.267251	3	806.4230957	-5.178372651	-0.004172504	34.79962839
																			DPLLDDHGDFIR	4	92859701	1xTMT6plex [N-Term]	1.031	1.088		4.386748314	46.46	0	1641.843401	3	547.9523315	-0.584496118	-0.000319884	64.52074292
																			VVAGVLHLGNIDFEEAGSTSGGCNLK	2	92859701	2xTMT6plex [N-Term; K26]; 1xCarbamidomethyl [C23	0.439			3.30172658	33.13	0	3102.629405	3	1034.881592	0.263528291	0.000272544	86.94199279
																			VMLTTAGGTK	2	92859701	2xTMT6plex [N-Term; K10]		0.882		4.286092758	52.1	0	1436.854707	2	718.930542	-0.626107581	-0.000449813	43.47344896
																			LCAGASEDIR	10	92859701	1xTMT6plex [N-Term]; 1xCarbamidomethyl [C2	1.363	1.225	0.496	4.046586514	68.95	0	1320.677916	2	660.8435669	1.469774756	0.00097055	45.24758886
																			YLTESYGTGQDIDDR	2	92859701	1xTMT6plex [N-Term]		1.302		3.119810343	23.32	0	1961.928981	2	981.4685669	0.446631388	0.00043813	47.15806056
																			ASFHELYNMYK	2	92859701	2xTMT6plex [N-Term; K11]	1.057			3.601126194	34.17	0	1860.971862	3	620.9942627	-1.948999948	-0.001209011	65.06323843
																			SDPDHLAELVK	4	92859701	2xTMT6plex [N-Term; K11]	1.045	0.872		3.292781353	19.54	0	1681.952507	3	561.3225708	0.387961218	0.000217511	57.61194433
																			LPQPSDQHFTSAVHQK	2	92859701	2xTMT6plex [N-Term; K16]		1.102		4.69255209	15.03	0	2278.234435	4	570.3143311	0.464850646	0.00026476	40.47447615
																			DVEDNCSLMYLNEATLLHNIK	3	92859701	2xTMT6plex [N-Term; K21]; 1xCarbamidomethyl [C6				1.631177306	13.05	0	2950.50545	3	984.1734619	0.129754781	0.000127614	88.19469183
																			LSFISVGNK	2	92859701	2xTMT6plex [N-Term; K9]	100			1.993529797	27.6	0	1422.872072	2	711.9394531	-0.310639311	-0.000221	78.06325074
																			IVEANPLLEAFGNAK	2	92859701	2xTMT6plex [N-Term; K15]				1.242929459	2.32	0	2044.184297	2	1022.595093	-0.679191757	-0.000694197	95.77558913
																			GPAVLATK	2	92859701	2xTMT6plex [N-Term; K8]		0.802		2.696841002	30.13	0	1214.787279	2	607.8971558	-0.201324599	-0.000122283	49.01707557
																			FVEIHFNEK	6	92859701	2xTMT6plex [N-Term; K9]	2.126	0.01	0.755	2.083387852	19.76	0	1620.914999	3	540.9759521	-1.046208276	-0.000565272	55.0876971
																			EEQELYQK	2	92859701	2xTMT6plex [N-Term; K8]	1.199			2.585951805	19.75	0	1524.830995	2	762.9185791	-0.72990835	-0.000556493	42.44656257
																			EYDALVK	4	92859701	2xTMT6plex [N-Term; K7]	0.893	0.999		2.922291756	37.2	0	1295.761124	2	648.3847656	0.8723753	0.000565195	53.93406479
																			QFEEIWER	3	92859701	1xTMT6plex [N-Term]	100			2.010184288	24.54	0	1365.700031	2	683.354248	0.869915591	0.000594022	66.66413875
																			DDEGFIIR	2	92859701	1xTMT6plex [N-Term]		0.972		2.380997896	23.5	0	1193.636369	2	597.3212891	-0.89397829	-0.000533542	58.2986543
																			SSEELLSALQK	4	92859701	2xTMT6plex [N-Term; K11]				1.588097334	10.32	0	1662.967822	2	831.9873047	-0.294386333	-0.000244777	65.97790968
528281407	PHB	prohibitin isoform 1	5245	NP_001268644.1	69.85294118	1	16	16	113	272	29.786	5.76	0.78	0.77	0.85	0.8	Significant	0.00099																
																			GVQDIVVGEGTHFLIPWVQKPIIFDCR	8	528281407	2xTMT6plex [N-Term; K20]; 1xCarbamidomethyl [C26			0.999	5.755400658	23.7	0	3581.970686	4	896.2467651	-1.523036787	-0.001363868	100.9199117
																			KLEAAEDIAYQLSR	8	528281407	2xTMT6plex [N-Term; K1]	1.189	100	0.744	3.46934557	26.16	1	2065.169375	3	689.0595703	-2.526573637	-0.001739267	71.3805098
																			AATFGLILDDVSLTHLTFGK	2	528281407	2xTMT6plex [N-Term; K20]			1.128	3.944225788	19.78	0	2577.469246	3	859.8278198	-0.131705681	-0.000113156	104.7157133
																			AAIISAEGDSK	16	528281407	2xTMT6plex [N-Term; K11]	0.844	0.79	0.81	4.351126671	71.02	0	1519.873194	2	760.4401245	-0.14561515	-0.000110658	40.20616668
																			KAAIISAEGDSK	8	528281407	3xTMT6plex [N-Term; K1; K12]	0.487	0.534		5.884687424	45.77	1	1877.131089	3	626.381958	0.123642637	7.74E-05	40.529095
																			DLQNVNITLR	10	528281407	1xTMT6plex [N-Term]	0.808	0.927	0.859	3.678748846	53.69	0	1414.821543	2	707.9152832	1.234294359	0.000873153	51.38869068
																			AAELIANSLATAGDGLIELR	6	528281407	1xTMT6plex [N-Term]			0.772	2.665698767	16.02	0	2227.249527	2	1114.128906	0.45293533	0.0005044	105.4727812
																			QVSDDLTER	13	528281407	1xTMT6plex [N-Term]	1.001	1.024	0.895	3.608385801	48.28	0	1291.669125	2	646.3340454	-6.434286866	-0.004155485	34.75834279
																			QVAQQEAER	4	528281407	1xTMT6plex [N-Term]	0.626	1.172		3.444565296	42.35	0	1287.685444	2	644.3463135	-0.072717196	-4.68E-05	18.5789922
																			FDAGELITQR	10	528281407	1xTMT6plex [N-Term]	100	0.01	100	2.809149981	35.08	0	1378.752795	2	689.8795166	-0.753330748	-0.000519328	56.11224749
																			NITYLPAGQSVLLQLPQ	4	528281407	1xTMT6plex [N-Term]				1.92034173	9.13	0	2084.195307	2	1042.599121	-2.082934591	-0.002170621	95.4705351
																			VLPSITTEILK	10	528281407	2xTMT6plex [N-Term; K11]	0.997	0.535	1.097	2.420459747	31.45	0	1672.06608	2	836.5385132	2.194833824	0.001834954	85.52141928
																			ILFRPVASQLPR	5	528281407	1xTMT6plex [N-Term]	100		100	2.883161783	13.35	0	1626.005262	3	542.6731567	-0.212142706	-0.000114982	55.01022432
																			IFTSIGEDYDER	2	528281407	1xTMT6plex [N-Term]				1.493907928	0.13	0	1673.821997	2	837.4145508	-0.102691624	-8.59E-05	66.09270692
																			LEAAEDIAYQLSR	3	528281407	1xTMT6plex [N-Term]		100		1.422316194	11.86	0	1707.911481	2	854.4597778	0.467538319	0.000399257	87.55242903
																			VFESIGK	4	528281407	2xTMT6plex [N-Term; K7]	1.034	1.332		2.88350749	34.49	0	1237.755645	2	619.3818359	0.606157607	0.000375138	61.08592733
14141157	HNRNPH3	heterogeneous nuclear ribonucleoprotein H3 isoform a	3189	NP_036339.1	43.6416185	1	8	9	48	346	36.903	6.87	0.98	0.98	0.99	0.98	Significant	0.000012																
																			DGMDNQGGYGSVGR	2	14141157	1xTMT6plex [N-Term]	0.809			3.256570816	40.4	0	1641.748849	2	821.3770752	-1.202936355	-0.00098746	36.82895985
																			GGGGSGGYYGQGGMSGGGWR	4	14141157	1xTMT6plex [N-Term]	100	100		4.842745781	82.7	0	2033.908537	2	1017.458618	0.699450393	0.000711309	49.69017423
																			GMGGHGYGGAGDASSGFHGGHFVHMR	10	14141157	1xTMT6plex [N-Term]	0.797	0.899		7.491443634	55.62	0	2772.246935	5	555.2553101	0.183610256	0.000101803	37.07960368
																			GMGGHGYGGAGDASSGFHGGHFVHMR	8	14141157	1xTMT6plex [N-Term]; 1xOxidation [M]	0.736	0.851		6.782629013	44.96	0	2788.24185	5	555.2553101	0.183610256	0.000101803	37.07960368
																			ATGEADVEFVTHEDAVAAMSK	1	14141157	2xTMT6plex [N-Term; K21]			0.01	3.635248184		0	2636.327803	3	879.4485474	1.246498952	0.001095393	71.61190944
																			STGEAFVQFASK	5	14141157	2xTMT6plex [N-Term; K12]	1.593			3.414910555	41.95	0	1729.952507	2	865.4788208	-1.237975279	-0.001070819	58.04495965
																			VHIDIGADGR	12	14141157	1xTMT6plex [N-Term]	1.11	1.009	1.003	4.049481392	55.59	0	1281.711265	2	641.3599243	1.019873078	0.000653591	39.49564768
																			EIAENALGK	2	14141157	2xTMT6plex [N-Term; K9]	1.301			4.181018829	60.13	0	1402.830601	2	701.9188843	-0.077539877	-5.44E-05	54.13753632
																			GGDGYDGGYGGFDDYGGYNNYGYGNDGFDDR	2	14141157	1xTMT6plex [N-Term]				2.151492119	4.97	0	3508.37363	3	1170.130493	0.939567029	0.001098784	72.11530261
																			GLPFGCSK	2	14141157	2xTMT6plex [N-Term; K8]; 1xCarbamidomethyl [C6	1.125			2.666947842	38.46	0	1323.749514	2	662.3783569	-0.058019143	-3.84E-05	58.63522587
4504505	HSD17B4	peroxisomal multifunctional enzyme type 2 isoform 1	3295	NP_000405.1	28.94021739	1	16	16	79	736	79.636	8.84	1.02	1.02	1.16	1.06	Significant	0.0019																
																			AVANYDSVEEGEK	6	4504505	2xTMT6plex [N-Term; K13]	1.031	0.92	0.912	4.094250202	86.39	0	1868.964193	2	934.9804688	-5.635469121	-0.005266245	41.13080822
																			FAKPVYPGQTLQTEMWK	4	4504505	3xTMT6plex [N-Term; K3; K17]	1.056	0.778		4.663641453	16.62	0	2711.519791	3	904.5113525	-0.105561764	-9.54E-05	80.05999925
																			IDSEGGVSANHTSR	4	4504505	1xTMT6plex [N-Term]		1.379	1.733	5.691594601	70.71	0	1658.829542	3	553.6151123	0.748638743	0.000413955	29.05521652
																			VLHGEQYLELYKPLPR	8	4504505	2xTMT6plex [N-Term; K12]	0.979	0.854	0.562	4.265491486	18.13	0	2413.400772	4	604.1061401	0.811442881	0.000489584	67.09605169
																			KICDFENASKPQSIQESTGSIIEVLSK	2	4504505	4xTMT6plex [N-Term; K1; K10; K27]; 1xCarbamidomethyl [C3		0.987		2.867710829	24.64	1	3925.17622	4	982.0477295	-1.816881804	-0.001782895	90.77866756
																			ATSTATSGFAGAIGQK	2	4504505	2xTMT6plex [N-Term; K16]			0.609	2.330167294	39.99	0	1926.069661	2	963.538147	-0.334450358	-0.000322087	47.47707473
																			LKDIGPEVVK	4	4504505	3xTMT6plex [N-Term; K2; K10]	0.694	0.931		3.422696114	15.44	1	1785.145282	3	595.7193604	-0.982819342	-0.000584825	54.68497796
																			VLQQFADNDVSR	6	4504505	1xTMT6plex [N-Term]	2.192	0.567	100	2.414423704	32.54	0	1620.8543	2	810.9314575	0.825712134	0.00066918	51.58174007
																			AYALAFAER	12	4504505	1xTMT6plex [N-Term]	0.981	1.897	1.629	3.434743404	42.57	0	1240.688739	2	620.8477173	-0.46795757	-0.000290295	65.29063342
																			GALVVVNDLGGDFK	9	4504505	2xTMT6plex [N-Term; K14]	100			2.517541647	22.77	0	1862.07877	2	931.5427856	-0.255107864	-0.000237515	94.06683833
																			VAVAIPNRPPDAVLTDTTSLNQAALYR	3	4504505	1xTMT6plex [N-Term]			100	0.907551527	3.35	0	3095.705063	3	1032.572876	-0.319216557	-0.0003294	68.6768688
																			GNIMLSQK	4	4504505	2xTMT6plex [N-Term; K8]	1.509	1.075		3.285281658	49.62	0	1348.802278	2	674.9042969	-0.712053958	-0.00048021	51.81782924
																			DIGPEVVK	2	4504505	2xTMT6plex [N-Term; K8]			1.265	3.322415829	38.36	0	1314.803323	2	657.9049072	-0.597471014	-0.000392778	51.7414875
																			ISDEDWDIIHR	6	4504505	1xTMT6plex [N-Term]	100	100	100	3.052083731	31.09	0	1627.827751	3	543.2803345	-0.799102995	-0.000433601	57.63226127
																			GSLAADK	6	4504505	2xTMT6plex [N-Term; K7]	1.04	1.006	0.995	2.548777103	43.3	0	1119.677395	2	560.3421021	-0.41726165	-0.000233599	35.42048092
																			LQSTFVFEEIGR	1	4504505	1xTMT6plex [N-Term]				1.498716354	4.79	0	1654.900188	2	827.9530029	-0.881261985	-0.0007292	81.45500536
577019448	GPI	glucose-6-phosphate isomerase isoform 3	2821	NP_001276718.1	34.50586265	1	15	15	87	597	67.243	8.97	1	0.92	0.93	0.95	Significant	0.0007																
																			TFTTQETITNAETAK	2	577019448	2xTMT6plex [N-Term; K15]	1.934			4.511572838	61.45	0	2114.138135	2	1057.571045	-1.571153841	-0.001660818	60.82462518
																			TITDVINIGIGGSDLGPLMVTEALKPYSSGGPR	2	577019448	2xTMT6plex [N-Term; K25]; 1xOxidation [M19			1.057	5.207026482	39.07	0	3803.066496	4	951.5223389	0.270620761	0.000257297	99.16648961
																			TITDVINIGIGGSDLGPLMVTEALKPYSSGGPR	2	577019448	2xTMT6plex [N-Term; K25]			1.48	4.682319164	24.97	0	3787.071581	4	951.5223389	0.270620761	0.000257297	99.16648961
																			TLAQLNPESSLFIIASK	6	577019448	2xTMT6plex [N-Term; K17]	1.812	1.004		2.840723515	31.45	0	2290.342254	2	1145.671753	-2.630672605	-0.00301257	91.85036624
																			VFEGNRPTNSIVFTK	5	577019448	2xTMT6plex [N-Term; K15]	1.573	1.521		3.492882252	19.29	0	2167.227559	3	723.0805054	-0.275101115	-0.000198736	65.03808958
																			VWYVSNIDGTHIAK	10	577019448	2xTMT6plex [N-Term; K14]	1.544	0.946	0.562	3.169744253	34.29	0	2061.153332	3	687.7233276	1.017920464	0.000699363	66.07540276
																			MIPCDFLIPVQTQHPIR	11	577019448	1xTMT6plex [N-Term]; 1xCarbamidomethyl [C4	0.905	1.069	0.991	3.538398504	34.12	0	2294.23508	3	765.4177856	1.622965156	0.001241155	79.69523074
																			HFVALSTNTTK	2	577019448	2xTMT6plex [N-Term; K11]		1.147		4.256455898	54.6	0	1676.973576	2	838.9909668	0.644329622	0.000540262	39.87406408
																			NLVTEDVMR	2	577019448	1xTMT6plex [N-Term]	1.255			3.663993597	47.91	0	1305.703402	2	653.3555298	0.291567221	0.00019035	58.95041758
																			EWFLQAAK	8	577019448	2xTMT6plex [N-Term; K8]	1.258	0.934	1.08	3.365110874	29.15	0	1450.845857	2	725.9274292	1.188870912	0.000862434	82.07173842
																			DVMPEVNK	8	577019448	2xTMT6plex [N-Term; K8]	0.991		1.046	3.309013128	33.42	0	1389.781208	2	695.3952637	1.470018258	0.001021502	48.30080841
																			AVLHVALR	14	577019448	1xTMT6plex [N-Term]	0.864	0.847	0.898	3.381983519	43.58	0	1107.719979	2	554.3640747	0.806723792	0.000446812	41.76140405
																			MLVDLAK	8	577019448	2xTMT6plex [N-Term; K7]	1.015	0.98	0.999	3.320102215	33.97	0	1247.779751	2	624.3934326	-0.130348024	-8.13E-05	75.20717309
																			VDHQTGPIVWGEPGTNGQHAFYQLIHQGTK	1	577019448	2xTMT6plex [N-Term; K30]			0.696	1.656840324		0	3773.955247	5	755.5936279	-4.296148339	-0.003242694	76.84313285
																			SPEDLER	4	577019448	1xTMT6plex [N-Term]		0.934	0.864	2.262965202	30.06	0	1074.562869	2	537.7855225	0.8366024	0.000449491	30.06220688
																			INYTEGR	2	577019448	1xTMT6plex [N-Term]			1.247	2.95556283	15.23	0	1081.583939	2	541.2952271	-0.704215743	-0.000380834	35.78699604
308818195	DPYSL2	dihydropyrimidinase-related protein 2 isoform	1808	NP_001184222.1	33.67799114	1	14	15	87	677	73.457	6.35	0.9	0.9	1.36	1.05	Significant	0.021																
																			ILDLGITGPEGHVLSRPEEVEAEAVNR	6	308818195	1xTMT6plex [N-Term]	100	100	0.974	3.026478767	20.19	0	3129.674157	4	783.173645	-0.449382844	-0.000351605	68.22107756
																			QIGENLIVPGGVK	6	308818195	2xTMT6plex [N-Term; K13]		100	100	2.991291761	47.21	0	1782.08894	2	891.546875	-1.384397795	-0.00123356	63.72323219
																			AALAGGTTMIIDHVVPEPGTSLLAAFDQWR	4	308818195	1xTMT6plex [N-Term]	100	0.611		5.136677265	44.19	0	3366.771762	3	1122.926514	-2.012136507	-0.002258135	109.4699822
																			IVLEDGTLHVTEGSGR	13	308818195	1xTMT6plex [N-Term]	0.88	0.894	0.603	5.006536007	45.55	0	1912.033721	3	638.0164795	0.608993738	0.000388139	60.4666234
																			GTVVYGEPITASLGTDGSHYWSK	8	308818195	2xTMT6plex [N-Term; K23]	1.763	100		3.678530216	37.5	0	2883.492894	3	961.8340454	-1.841894441	-0.001770363	72.86626039
																			MVIPGGIDVHTR	4	308818195	1xTMT6plex [N-Term]	0.843	0.881		3.409754276	47.52	0	1523.856549	3	508.6237488	0.094692569	4.81E-05	48.19804271
																			GIQEEMEALVK	8	308818195	2xTMT6plex [N-Term; K11]	0.721	1.015	100	3.38953352	38.88	0	1704.960628	2	852.9842529	0.352365541	0.000300385	79.2166926
																			GSPLVVISQGK	6	308818195	2xTMT6plex [N-Term; K11]	0.893		1.194	3.631722927	46.03	0	1542.961949	2	771.9859619	1.748596669	0.001349009	59.54980715
																			SSAEVIAQAR	2	308818195	1xTMT6plex [N-Term]	1.238			3.741255283	41.3	0	1260.71093	2	630.8591919	0.140166202	8.84E-05	36.93998949
																			AITIANQTNCPLYITK	6	308818195	2xTMT6plex [N-Term; K16]; 1xCarbamidomethyl [C10	100	100		2.430480003	23.87	0	2279.28336	2	1140.143799	-1.33313997	-0.001519302	68.31659342
																			FQMPDQGMTSADDFFQGTK	6	308818195	2xTMT6plex [N-Term; K19]	1.052		0.976	2.646060467	30.24	0	2609.24163	3	870.4186401	-0.100845334	-8.77E-05	85.61557471
																			GLYDGPVCEVSVTPK	4	308818195	2xTMT6plex [N-Term; K15]; 1xCarbamidomethyl [C8	0.898	100		1.96782732	18.57	0	2079.119648	2	1040.062622	-0.808476499	-0.00084046	55.15929506
																			KPFPDFVYK	4	308818195	3xTMT6plex [N-Term; K1; K9]	0.979	0.789		3.094186306	11.12	0	1828.097604	3	610.0360718	-2.156224022	-0.001313929	63.76338127
																			TVTPASSAK	4	308818195	2xTMT6plex [N-Term; K9]	0.8	0.888		3.397539854	43.82	0	1319.793487	2	660.3997803	-0.911531339	-0.000601517	33.25307969
																			MSVIWDK	6	308818195	2xTMT6plex [N-Term; K7]	1.2	0.908	1.016	2.513372183	26.13	0	1336.769915	2	668.8878784	-1.073194457	-0.000717307	63.25670181
209869993	GART	trifunctional purine biosynthetic protein adenosine-3 isoform 1	2618	NP_001129477.1	21.18811881	1	18	18	83	1010	107.699	6.7	1.14	1.22	1.53	1.3	Significant	0.0083																
																			VDLGGFAGLFDLK	6	209869993	2xTMT6plex [N-Term; K13]	1.579	1.145	1.483	3.586750269	42.03	0	1810.051492	2	905.5284424	-1.040983852	-0.000942117	100.7113077
																			DIQQHKEEAWVIGSVVAR	4	209869993	2xTMT6plex [N-Term; K6]	0.886			5.619861126	17.12	1	2523.408377	4	631.6078491	0.471510829	0.000297454	89.64757062
																			GDTVATLSER	8	209869993	1xTMT6plex [N-Term]	1.432	1.239	1.582	3.732913256	64.04	0	1277.689861	2	639.3469849	-2.47910196	-0.001583762	35.47163008
																			LDLSVTEAVVAGIAK	3	209869993	2xTMT6plex [N-Term; K15]		100		3.175051212	31.45	0	1944.178149	2	972.592041	-0.691247739	-0.000671954	101.7359273
																			AFAHITGGGLLENIPR	2	209869993	1xTMT6plex [N-Term]	1.523			2.680408001	11.28	0	1895.070047	3	632.3613892	-0.228281552	-0.000144203	71.44980137
																			ENLISALEEAK	5	209869993	2xTMT6plex [N-Term; K11]	100		1.328	2.541206121	34.72	0	1674.967822	2	837.9868774	-0.802431646	-0.000672024	80.04257796
																			AVQEIMQEK	6	209869993	2xTMT6plex [N-Term; K9]	1.302	2.093	1.411	2.918816328	35.42	0	1533.871085	2	767.4371948	-2.58969062	-0.001986126	47.66625115
																			VLAVTAIR	10	209869993	1xTMT6plex [N-Term]	1.056	1.355	1.508	3.235881805	35.54	0	1071.708746	2	536.357605	-0.758068893	-0.000406215	50.82083607
																			GVEITGFPEAQALGLEVFHAGTALK	6	209869993	2xTMT6plex [N-Term; K25]		100		2.257134438	10	0	3013.676278	3	1005.230286	0.00847921	8.52E-06	101.558476
																			VLIIGSGGR	4	209869993	1xTMT6plex [N-Term]	1.289	0.835		2.835269451	39.97	0	1100.698909	2	550.8536987	1.100665157	0.00060575	55.88032079
																			ASGLAAGK	2	209869993	2xTMT6plex [N-Term; K8]	0.964			3.057544947	41.61	0	1132.709029	2	566.8581543	0.002475261	1.40E-06	35.88507967
																			AAVAGLDK	6	209869993	2xTMT6plex [N-Term; K8]	1.169	1.497	1.831	3.058051348	31.78	0	1202.750894	2	601.8789063	-0.297709195	-0.000179035	43.08210935
																			TVDGMQQEGTPYTGILYAGIMLTK	3	209869993	2xTMT6plex [N-Term; K24]				1.692229152	1.01	0	3045.604101	3	1015.870117	-2.726033071	-0.002767472	104.2042269
																			IAQLCNK	2	209869993	2xTMT6plex [N-Term; K7]; 1xCarbamidomethyl [C5	1.438			3.251126051	33.56	0	1304.776063	2	652.8914185	-0.38538869	-0.000251423	45.83750419
																			AIAFLQQPR	3	209869993	1xTMT6plex [N-Term]				1.3154006	17.88	0	1272.762572	2	636.8850098	0.134142262	8.54E-05	59.38224296

Bhat et al,2017. Cigarette smoke and chewing tobacco alter expression of different sets of miRNAs in oral keratinocytes

Supplementary Table S2(A). List of all proteins and peptides identified in OKF6/TERT1 cells chronically treated with chewing tobacco using Sequest and Mascot search algorithms

Accession	Gene_Symbol	Description	Gene_ID	Refseq_Accession	Coverage	Proteins	#Unique_Peptides	#Peptides	#PSMS	#AAs	MW [kDa]	calc. pI	OKF6-STE-6M/OKF6P_R1	OKF6-STE-6M/OKF6P_R2	OKF6-STE-6M/OKF6P_R3	Median fold change	Status	P-value (6M)	Sequence	#PSMs	Protein Accessions	Modifications	OKF6-STE-6M/OKF6P_R1	OKF6-STE-6M/OKF6P_R2	OKF6-STE-6M/OKF6P_R3	Xcorr	Ionscore	# Missed Cleavages	Theo. MH+ [Da]	Charge	m_z	DeltaM [ppm]	Deltam/z [Da]	RT [min]
																			ILSGPFVQK	2	209869993	2xTMT6plex [N-Term; K9]	1.145			2.768102407	24.95	0	1446.908457	2	723.9584961	0.869728485	0.000629209	68.78618554
																			IKDTVLQR	7	209869993	2xTMT6plex [N-Term; K2]	1.265		1.306	2.981771469	21.38	1	1430.90952	2	715.958313	-0.119080511	-8.52E-05	44.25927721
																			VLEFNCR	4	209869993	1xTMT6plex [N-Term]; 1xCarbamidomethyl [C6	0.969	1.111		2.4053092	21.85	0	1166.618945	2	583.8125	-1.046948539	-0.000610695	50.6124679
18105007	CAD	CAD protein isoform 1	790	NP_004332.2	10.29213483	1	16	18	69	2225	242.829	6.46	1.52	1.42	1.39	1.44	Significant	0.00074																
																			VIMGEEVEPVGLMTGSGVVGVK	3	18105007	2xTMT6plex [N-Term; K22]	1.679			3.327940226	59.12	0	2645.465817	3	882.4970703	4.098014585	0.003613719	89.11863467
																			ASDPGLPAEEPK	2	18105007	2xTMT6plex [N-Term; K12]			1.526	3.47202754	53.58	0	1668.920872	2	834.9639282	-0.175145924	-0.000146152	44.6171057
																			SVGEVMGIGR	4	18105007	1xTMT6plex [N-Term]		1.643	1.704	3.860193491	52.71	0	1233.682273	2	617.3445435	-0.375004122	-0.000231318	61.76541337
																			TLHEWLQQHGIPGLQGVDTR	2	18105007	1xTMT6plex [N-Term]				2.678955793	13.99	0	2514.34147	3	838.784668	-0.803269474	-0.000673231	75.47734076
																			IIAHAQLLEQHR	6	18105007	1xTMT6plex [N-Term]	0.804	1.037	1.279	4.093884468	34.8	0	1657.969939	3	553.328064	-0.181152542	-0.000100115	40.72371356
																			VTAVDWHFEEAVDGECPPQR	4	18105007	1xTMT6plex [N-Term]; 1xCarbamidomethyl [C16	100			3.273915052	12.16	0	2571.213552	3	857.7420654	-0.742626351	-0.000636484	70.38184806
																			VLGTPVETIELTEDR	8	18105007	1xTMT6plex [N-Term]	1.851			3.295811653	27.43	0	1901.042888	2	951.0236816	-1.473842998	-0.001400919	73.0103121
																			LAADFSVPLIIDIK	6	18105007	2xTMT6plex [N-Term; K14]				1.834302068	8.45	0	1973.208721	2	987.1088867	0.899857922	0.000887804	108.1845445
																			KEEILLIK	4	18105007	3xTMT6plex [N-Term; K1; K8]	2.634	2.227		3.689779758	21.46	1	1673.118005	3	558.3771362	-0.687051714	-0.000383173	70.2286445
																			SILEQLAEK	4	18105007	2xTMT6plex [N-Term; K9]	2.095	100		2.710275173	31.8	0	1488.903766	2	744.9545898	-1.250948881	-0.000931271	74.13979926
																			MAEIGEHVAPSEAANSLEQAQAAER	2	18105007	1xTMT6plex [N-Term]				1.984964252	14.13	0	2909.426064	3	970.4732666	-7.155131238	-0.006939108	59.34262283
																			AMLSTGFK	4	18105007	2xTMT6plex [N-Term; K8]	1.876	1.776		3.319519758	39.89	0	1312.769915	2	656.8887329	0.209001067	0.000137185	65.29172863
																			ILALDCGLK	6	18105007	2xTMT6plex [N-Term; K9]; 1xCarbamidomethyl [C6	1.692	100	3.25	2.638997555	30.94	0	1460.891093	2	730.9472046	-2.710852528	-0.00198013	70.4123738
																			SFEEAFQK	6	18105007	2xTMT6plex [N-Term; K8]	1.534		100	2.791423082	30.96	0	1443.788402	2	722.3961792	-2.299354642	-0.001659891	59.37357591
																			TLGVDLVALATR	2	18105007	1xTMT6plex [N-Term]	100			1.997839451	13.75	0	1457.888895	2	729.4488525	1.052006179	0.000766854	85.14685541
																			VPQFSFSR	2	18105007	1xTMT6plex [N-Term]	1.093			2.349300623	17.26	0	1196.662524	2	598.8341675	-1.224638554	-0.00073274	60.48568143
																			VFNTGGAPR	2	18105007	1xTMT6plex [N-Term]	1.3			2.036062956	1.28	0	1147.642123	2	574.3250732	0.651016863	0.000373567	40.22740233
																			SFPFVSK	2	18105007	2xTMT6plex [N-Term; K7]	1.403	1.423		2.742493391	31.63	0	1269.76073	2	635.383606	-0.62616989	-0.000397543	73.04944243
4826643	ANXA3	annexin A3	306	NP_005130.1	60.0619195	1	18	18	119	323	36.353	5.92	0.9	0.92	0.7	0.84	Significant	0.0069																
																			GIGTDEFTLNR	11	4826643	1xTMT6plex [N-Term]	0.732	0.937	0.705	4.569674969	71.8	0	1451.769174	2	726.3885498	0.447357188	0.00032473	63.25224251
																			GAGTNEDALIEILTTR	6	4826643	1xTMT6plex [N-Term]		100	0.01	3.787669897	55.36	0	1903.033386	2	952.0193481	-1.033492608	-0.000983385	90.81858402
																			GELSGHFEDLLLAIVNCVR	12	4826643	1xTMT6plex [N-Term]; 1xCarbamidomethyl [C17	0.862	100	0.751	4.565666676	25.76	0	2371.264132	3	791.0911255	-2.238635117	-0.001769465	110.6970023
																			SDTSGDYEITLLK	11	4826643	2xTMT6plex [N-Term; K13]	1.055		100	2.850600243	41.69	0	1900.031545	2	950.5188599	-0.579744815	-0.000550767	69.18471053
																			WGTDEDKFTEILCLR	2	4826643	2xTMT6plex [N-Term; K7]; 1xCarbamidomethyl [C13			1.142	4.663181782	21.79	1	2341.226239	3	781.0787354	-1.958740781	-0.001528618	92.62327423
																			DISQAYYTVYK	6	4826643	2xTMT6plex [N-Term; K11]	1.054	0.705		3.99266839	30.96	0	1808.983472	2	904.9945679	-0.891797983	-0.000806624	57.13363255
																			ALLTLADGR	17	4826643	1xTMT6plex [N-Term]	0.753	0.591	0.811	3.503188133	41.83	0	1158.704389	2	579.8549194	-1.57620255	-0.000913176	53.65530372
																			MLISILTER	6	4826643	1xTMT6plex [N-Term]	1.128	1.006		3.087094545	28.71	0	1304.780924	2	652.8939209	-0.275251667	-0.000179572	92.94402249
																			SEIDLLDIR	10	4826643	1xTMT6plex [N-Term]	1.331	0.781		2.935110092	59.4	0	1302.746647	2	651.8764038	-0.856837985	-0.000558121	67.49865393
																			EYQAAYGK	7	4826643	2xTMT6plex [N-Term; K8]	0.907	1.136	0.921	3.827005863	46.02	0	1387.762187	2	694.3849487	0.312734373	0.000217	36.8273323
																			RDESLKVDEHLAK	3	4826643	3xTMT6plex [N-Term; K6; K13]			0.656	3.892651558	6.86	2	2227.301342	4	557.5800781	-1.283670039	-0.00071478	36.97377589
																			DYPDFSPSVDAEAIQK	4	4826643	2xTMT6plex [N-Term; K16]			100	1.644872427	9.69	0	2240.1487	2	1120.574951	-2.711336194	-0.003036898	63.74241233
																			DIVDSIK	3	4826643	2xTMT6plex [N-Term; K7]			0.434	2.825705051	13.46	0	1247.761124	2	624.3841553	-0.07238014	-4.52E-05	49.7353827
																			LTFDEYR	12	4826643	1xTMT6plex [N-Term]	0.948	0.899	0.816	2.448415279	28.75	0	1172.614905	2	586.8110352	-0.094837188	-5.56E-05	51.4667727
																			GDLSGHFEHLMVALVTPPAVFDAK	1	4826643	2xTMT6plex [N-Term; K24]				1.238667965		0	3009.62722	3	1003.88031	-0.279962984	-0.000280861	96.40421895
																			DESLKVDEHLAK	2	4826643	3xTMT6plex [N-Term; K5; K12]	0.692			2.401104212	0.13	1	2071.200231	4	518.5552368	-0.53755999	-0.000278349	51.25238031
																			GIGTDEK	2	4826643	2xTMT6plex [N-Term; K7]		0.858		2.53646183	30.5	0	1177.682874	2	589.3455811	0.858931889	0.000505775	31.58343477
																			VDEHLAK	4	4826643	2xTMT6plex [N-Term; K7]	1.095			3.197467804	26.13	0	1269.756708	3	423.9241943	1.041284369	0.000440726	35.16935534
223555975	TPM4	tropomyosin alpha-4 chain isoform Tpm4.1cy	7171	NP_001138632.1	41.90140845	1	2	14	125	284	32.703	4.73	1.02	1.07	1.05	1.05	Significant	0.00019																
																			ASDAEGDVAALNR	6	223555975	1xTMT6plex [N-Term]	0.933	0.969	0.819	4.668531418	65.27	0	1517.775715	2	759.3908691	-0.826030313	-0.000626864	32.67116669
																			KASDAEGDVAALNR	12	223555975	2xTMT6plex [N-Term; K1]	0.851	0.807	1.095	4.551401138	65	1	1875.0361	3	625.6827393	0.028891982	1.81E-05	46.35743744
																			IQLVEEELDR	18	223555975	1xTMT6plex [N-Term]	1.297	2.38	1.828	3.785353422	49.84	0	1472.815789	2	736.911499	-0.046111079	-3.40E-05	68.55083794
																			KLVILEGELER	15	223555975	2xTMT6plex [N-Term; K1]	0.853	0.956	0.684	4.428694725	31.82	1	1757.093692	3	586.3692017	-0.364123743	-0.000213267	59.71334727
																			EENVGLHQTLDQTLNELNCI	15	223555975	1xTMT6plex [N-Term]; 1xCarbamidomethyl [C19	100	1.513	0.707	3.414426565	22.44	0	2569.276547	3	857.0984497	1.653882335	0.001416427	85.66828029
																			MEIQEMQLK	20	223555975	2xTMT6plex [N-Term; K9]	1.324	0.962		3.55449605	46.32	0	1607.890106	2	804.4488525	0.200447856	0.000161149	72.61134951
																			AEQAEADK	6	223555975	2xTMT6plex [N-Term; K8]	1.855	1.353	2.024	3.345167875	46.74	0	1319.720716	2	660.3638306	-0.251119183	-0.000165704	26.71381538
																			SLEAASEK	6	223555975	2xTMT6plex [N-Term; K8]	1.099	0.859	1.023	3.799231529	49.52	0	1292.746202	2	646.8760376	-1.085932169	-0.000701917	34.71213245
																			AEQAEADKK	10	223555975	3xTMT6plex [N-Term; K8; K9]	1.354	1.532	1.56	2.406741858	16.76	1	1676.978611	3	559.6641235	-0.473187032	-0.000264508	34.59182199
																			YSEKEDKYEEEIK	4	223555975	4xTMT6plex [N-Term; K4; K7; K13]	1.247	1.244		4.074170113	13.75	2	2606.437115	2	1303.714355	-6.016290363	-0.007840541	43.42246357
																			CGDLEEELK	2	223555975	2xTMT6plex [N-Term; K9]; 1xCarbamidomethyl [C1	1.337			2.410672665	15.73	0	1550.81363	2	775.9111938	0.954876385	0.000740418	56.19358147
																			TIDDLEEK	4	223555975	2xTMT6plex [N-Term; K8]	0.951		1.374	3.846661568	22.09	0	1420.793546	2	710.8937988	-9.308469751	-0.006612707	46.8645142
																			EDKYEEEIK	13	223555975	3xTMT6plex [N-Term; K3; K9]	1.54	1.287	1.151	2.958414555	24.3	1	1870.041271	2	935.5215454	-2.917801748	-0.002728205	47.94373045
																			HIAEEADR	4	223555975	1xTMT6plex [N-Term]	100	1.038		2.72195363	28.11	0	1169.611216	2	585.3092651	0.03179128	1.86E-05	20.53571929
212276104	LRRFIP1	leucine-rich repeat flightless-interacting protein 1 isoform 5	9208	NP_001131025.1	18.48404255	1	12	13	54	752	82.639	4.61	1.36	1.04	1.29	1.23	Significant	0.0062																
																			AMVSNAQLDNEK	4	212276104	2xTMT6plex [N-Term; K12]	1.245	1.111		4.702671528	74.84	0	1777.951855	2	889.4789429	-0.700535174	-0.000622759	34.62448937
																			IAAESSENVDCPENPK	10	212276104	2xTMT6plex [N-Term; K16]; 1xCarbamidomethyl [C11	1.535	1.465	1.435	4.360197067	64.37	0	2218.106183	2	1109.557251	0.469888742	0.000521132	39.66576811
																			DHNEEEGEETGLRDEKPIK	3	212276104	3xTMT6plex [N-Term; K16; K19]			1.013	5.85721159	6	1	2912.520455	4	728.8840942	-2.028386398	-0.001476929	35.31051134
																			EFTNQEAAEPK	2	212276104	2xTMT6plex [N-Term; K11]	1.385			3.646540403	47.69	0	1721.911036	2	861.4605713	1.643672679	0.001415129	43.15020092
																			EIKDSLAEVEEK	6	212276104	3xTMT6plex [N-Term; K3; K12]	1.083	100		3.186485529	14.06	1	2077.199562	3	693.0707397	-0.912792277	-0.000632017	61.4406356
																			SPVPVETLK	6	212276104	2xTMT6plex [N-Term; K9]	1.367	1.347	1.116	3.519575357	56.04	0	1427.887387	2	714.4474487	0.163542965	0.00011676	55.01204998
																			DSLAEVEEK	4	212276104	2xTMT6plex [N-Term; K9]	1.89	1.833		4.268862724	55.26	0	1477.81501	2	739.411377	0.31610604	0.000233573	54.87713505
																			KSPVPVETLK	2	212276104	3xTMT6plex [N-Term; K1; K10]	1.237			3.451766014	24.34	1	1785.145282	3	595.7197876	-0.264817119	-0.000157579	55.22977148
																			EIDCLSPEAQK	4	212276104	2xTMT6plex [N-Term; K11]; 1xCarbamidomethyl [C4	1.311	1.464		4.246141911	51.58	0	1747.930057	2	874.4678955	-0.882469163	-0.000771247	51.53163813
																			SAVEAQNEVTENPK	2	212276104	2xTMT6plex [N-Term; K14]				1.633540988	5.43	0	1974.054405	2	987.5296631	-1.193355218	-0.001177874	40.25363787
																			DMLLELEEQLAESR	6	212276104	1xTMT6plex [N-Term]	1.018		100	2.289969683	18.97	0	1904.983659	2	952.9935303	-2.034218565	-0.001937577	95.91328153
																			EVEERPEKDFTEK	1	212276104	3xTMT6plex [N-Term; K8; K13]		0.605		3.225899935		1	2323.274852	4	581.5740967	-0.127105603	-7.38E-05	38.96673411
																			AMVSNAQLDNEK	2	212276104	2xTMT6plex [N-Term; K12]; 1xOxidation [M2		1.505		1.918420196	24.7	0	1793.94677	2	889.4789429	-0.700535174	-0.000622759	34.62448937
																			ASEVEVK	2	212276104	2xTMT6plex [N-Term; K7]	0.775			2.464611292	15.66	0	1219.729824	2	610.368103	-0.733511057	-0.000447343	37.64353352
255958282	PLIN3	perilipin-3 isoform 1	10226	NP_005808.3	52.07373272	1	13	13	79	434	47.046	5.44	1.03	1.02	1.02	1.02	Significant	0.000011																
																			SVVTGGVQSVMGSR	6	255958282	1xTMT6plex [N-Term]	0.757	1.018	1.007	4.680616379	57.09	0	1592.862756	2	796.9346924	-0.40689907	-0.000324067	48.37311955
																			VASMPLISSTCDMVSAAYASTK	8	255958282	2xTMT6plex [N-Term; K22]; 1xCarbamidomethyl [C11	100	100	100	3.285878897	39.66	0	2748.40223	3	916.8057861	0.20917551	0.000191633	96.41685046
																			EALDHMVEYVAQNTPVTWLVGPFAPGITEK	11	255958282	2xTMT6plex [N-Term; K30]		100	100	4.018789768	35.61	0	3770.986789	4	943.5031738	1.080935237	0.001019048	112.3145327
																			SEEWADNHLPLTDAELAR	10	255958282	1xTMT6plex [N-Term]	0.944	0.888		4.13211298	33.62	0	2296.140705	3	766.0522461	0.644621319	0.00049338	65.19531867
																			QVEDLQATFSSIHSFQDLSSSILAQSR	6	255958282	1xTMT6plex [N-Term]	100		100	2.704003096	24.39	0	3223.64325	3	1075.216064	-2.981176848	-0.003203417	96.39452707
																			TLTAAAVSGAQPILSK	6	255958282	2xTMT6plex [N-Term; K16]				2.953276157	39.3	0	1986.199947	2	993.6050415	1.439436053	0.001429504	66.93405595
																			GAVQSGVDK	8	255958282	2xTMT6plex [N-Term; K9]	0.833	0.946	0.945	3.445109606	63.65	0	1318.773086	2	659.8905029	0.487854493	0.000321685	30.06207556
																			DTVATQLSEAVDATR	6	255958282	1xTMT6plex [N-Term]		0.726	100	3.278048992	33.03	0	1805.944237	2	903.4747314	-1.135605038	-0.00102542	58.89642959
																			LEPQIASASEYAHR	9	255958282	1xTMT6plex [N-Term]	1.061	1.07		4.073052406	40.57	0	1800.944178	3	600.9855347	-1.180972803	-0.000708955	47.79049446
																			GLDKLEENLPILQQPTEK	2	255958282	3xTMT6plex [N-Term; K4; K18]			0.597	2.800685406	21.21	1	2752.606357	3	918.2063599	-0.665002517	-0.000610163	78.31061385
																			QLQGPEKEPPKPEQVESR	1	255958282	3xTMT6plex [N-Term; K7; K11]			1.04	4.644860744		1	2763.560804	4	691.638916	-9.75894486	-0.006742359	38.47994287
																			DIAQQLQATCTSLGSSIQGLPTNVK	2	255958282	2xTMT6plex [N-Term; K25]; 1xCarbamidomethyl [C10				1.738710999	5.54	0	3088.671269	3	1030.228271	-0.326382055	-0.000336029	86.74062271
																			EPPKPEQVESR	4	255958282	2xTMT6plex [N-Term; K4]	0.882	0.934		3.036494732	4.05	0	1753.984869	3	585.3327637	-0.64514489	-0.000377191	30.21123726
112789562	IFI16	gamma-interferon-inducible protein 16 isoform 2	3428	NP_005522.2	31.96159122	1	18	18	69	729	82.044	9.32	0.76	0.91	0.98	0.89	Significant	0.0054																
																			GEFTYYEIQDNTGK	2	112789562	2xTMT6plex [N-Term; K14]		0.685		3.54390049	44.96	0	2123.069721	2	1062.038696	0.186036342	0.000197484	66.50527713
																			EVDATSPAPSTSSTVK	2	112789562	2xTMT6plex [N-Term; K16]		0.876		4.07612133	41.38	0	2035.095936	3	679.0362549	-0.847287606	-0.000574771	36.35332119
																			MDVVGTGQCHNIPCEEGDK	2	112789562	2xTMT6plex [N-Term; K19]; 2xCarbamidomethyl [C9; C14			1.522	4.507897854	31.98	0	2604.225663	3	868.7387695	-9.18042418	-0.007969299	44.70212641
																			MFHATVATENEVFR	6	112789562	1xTMT6plex [N-Term]	0.783		0.01	4.011412621	37.08	0	1880.952634	3	627.6557617	0.05213648	3.27E-05	87.7028351
																			VSEEQTQPPSPAGAGMSTAMGR	2	112789562	1xTMT6plex [N-Term]			100	3.070674896	29.9	0	2418.159074	2	1209.573486	-8.013432185	-0.009688877	43.17419141
																			TEGAEATPGAQK	2	112789562	2xTMT6plex [N-Term; K12]	0.875			4.270382881	67.96	0	1617.884821	2	809.4470825	1.277865418	0.00103372	32.12655091
																			LTTINCEEGDKLK	4	112789562	3xTMT6plex [N-Term; K11; K13]; 1xCarbamidomethyl [C6	0.81		0.879	3.951553106	11.92	1	2208.25128	3	736.7507324	-6.175159728	-0.004545435	48.17406623
																			GLEVINDYHFR	6	112789562	1xTMT6plex [N-Term]	0.659	0.536	0.432	3.693189859	49.95	0	1591.843007	3	531.2860107	0.296390134	0.000157269	59.25267648
																			LISEMHSFIQIK	8	112789562	2xTMT6plex [N-Term; K12]	0.556	100	0.01	3.047489405	17.51	0	1904.107961	3	635.3729858	-1.868124361	-0.001185703	75.84818656
																			LTCFELAPK	4	112789562	2xTMT6plex [N-Term; K9]; 1xCarbamidomethyl [C3		100	0.01	2.274644375	33.93	0	1536.886007	2	768.9465942	-0.062179913	-4.78E-05	71.9203786
																			IQIADLMEEK	4	112789562	2xTMT6plex [N-Term; K10]		0.461	1.404	2.657946587	26.81	0	1647.939165	2	824.4736938	0.574229518	0.000473148	81.53195383
																			VLNTSLK	6	112789562	2xTMT6plex [N-Term; K7]	0.87	1.132	1.117	3.257479906	39.29	0	1232.797844	2	616.902832	0.440714999	0.000271656	48.09318679
																			MEVVVHGR	6	112789562	1xTMT6plex [N-Term]	0.877	0.779	0.807	2.952040672	22.33	0	1155.650579	2	578.328064	-1.494950415	-0.00086382	32.80778689
																			VFNIDLK	4	112789562	2xTMT6plex [N-Term; K7]	0.59		100	3.006968021	32.45	0	1306.813494	2	653.9099731	-0.630940024	-0.00041226	83.27609823
																			DILNPDSSMETSPDFFF	4	112789562	1xTMT6plex [N-Term]				1.667945862	5.4	0	2191.010268	2	1096.008301	-0.430266126	-0.000471359	113.1948196
																			KSEDTISK	4	112789562	3xTMT6plex [N-Term; K1; K8]	0.82	1.01		2.836615324	10.77	1	1594.961898	3	532.3250122	-0.887073796	-0.000471616	37.01859056
																			IFEDIPTLEDLAETLKK	2	112789562	3xTMT6plex [N-Term; K16; K17]	100			1.292683959	0.25	1	2662.552196	3	888.1877441	-1.320867298	-0.001172293	111.5855203
																			VLSTTKPFEYETPEMEK	1	112789562	3xTMT6plex [N-Term; K6; K17]				1.655273795		0	2716.472231	3	906.1618042	-0.505001424	-0.000457274	76.80229308
586798211	USO1	general vesicular transport factor p115 isoform 1	8615	NP_001276978.1	18.80781089	1	15	15	64	973	109.127	4.89	0.79	0.8	0.85	0.81	Significant	0.00052																
																			SSQTSGTNEQSSAIVSAR	6	586798211	1xTMT6plex [N-Term]	0.854	1.38	1.103	4.386626244	43.8	0	2039.020255	2	1020.013794	0.027381104	2.79E-05	28.68592448
																			GVMGGQSAGPQHTEAETIQK	6	586798211	2xTMT6plex [N-Term; K20]	0.918	0.704		6.887491226	70.63	0	2484.291692	3	828.7679443	-0.97100602	-0.000804087	41.36318496
																			GVMGGQSAGPQHTEAETIQK	2	586798211	2xTMT6plex [N-Term; K20]; 1xOxidation [M3		100		4.363914013	34.22	0	2500.286607	3	828.7679443	-0.97100602	-0.000804087	41.36318496
																			SQLNSQSVEITK	2	586798211	2xTMT6plex [N-Term; K12]		1.029		4.758662701	61.53	0	1792.021649	2	896.5161133	1.842177801	0.001650611	44.51499034
																			VASSTLLDDR	4	586798211	1xTMT6plex [N-Term]	1.109	1.159		4.142559528	64.71	0	1305.721161	2	653.3642578	0.059932398	3.91E-05	53.5678713
																			TLEQHDNIVTHYK	10	586798211	2xTMT6plex [N-Term; K13]	0.515	0.577	0.85	5.544497967	41.92	0	2056.12276	4	514.781189	-9.646165596	-0.004958425	40.3537422
																			DSEQVAELK	2	586798211	2xTMT6plex [N-Term; K9]	1.194			3.742637157	39.75	0	1476.830995	2	738.918335	-1.08425956	-0.000800634	43.68671744
																			ELEGVITK	4	586798211	2xTMT6plex [N-Term; K8]	0.908	0.848		3.362944603	46.74	0	1346.829538	2	673.9193726	1.433282485	0.000965194	48.44921223
																			MKPWFEVGDENSGWSAQK	3	586798211	3xTMT6plex [N-Term; K2; K18]	0.637			2.587759495	7.95	0	2783.442997	3	928.4865723	0.778455631	0.000722262	75.60165213
																			EQDLQLEELR	4	586798211	1xTMT6plex [N-Term]			0.01	2.353167295	28.36	0	1501.805953	2	751.4066162	0.001925598	1.45E-06	48.34643931
																			LSALLQETK	3	586798211	2xTMT6plex [N-Term; K9]	0.969			2.265823841	6.26	0	1460.908851	2	730.9591675	1.51092995	0.001103665	81.86766835
																			LMDLLADSR	6	586798211	1xTMT6plex [N-Term]	100	1.371		2.209684372	44.7	0	1262.697589	2	631.8522949	-0.218101509	-0.000137698	71.47630086
																			DQYNLLK	4	586798211	2xTMT6plex [N-Term; K7]	1.042	1.256		3.199888468	35.18	0	1351.798572	2	676.4030762	0.224414906	0.000151682	61.61169371
																			ASQKPQPNFPSPEYMIFDHEFTK	2	586798211	3xTMT6plex [N-Term; K4; K23]				1.248864651	0.17	0	3425.780709	4	857.1947021	-6.926954253	-0.005932557	72.42766034
																			QELATLK	4	586798211	2xTMT6plex [N-Term; K7]	1.264	1.108		2.460280657	35.52	0	1260.792759	2	630.8996582	-0.570231503	-0.000359472	52.49744366
																			IVAFENAFER	2	586798211	1xTMT6plex [N-Term]				1.114117265	5.78	0	1424.773531	2	712.8898926	-0.717421912	-0.000511082	73.5197628
23238211	ARPC2	actin-related protein 2/3 complex subunit 2	10109	NP_690601.1	45.66666667	1	11	11	69	300	34.311	7.36	1.13	1.09	1.04	1.09	Significant	0.00057																
																			DDETMYVESK	6	23238211	2xTMT6plex [N-Term; K10]	1.4	1.424	1.24	4.637595654	77.21	0	1674.829674	2	837.9187012	0.269802809	0.000225937	45.14550327
																			VYGSFLVNPESGYNVSLLYDLENLPASK	4	23238211	2xTMT6plex [N-Term; K28]	1.241			3.772232056	47.93	0	3546.877222	4	887.4749756	0.239761259	0.000212601	105.8429224
																			ASHTAPQVLFSHR	4	23238211	1xTMT6plex [N-Term]	1.097	0.997		5.186507225	56.39	0	1679.917903	3	560.6437988	-0.631087759	-0.000353392	41.7282623
																			ELQAHGADELLK	12	23238211	2xTMT6plex [N-Term; K12]	1.437	1.442	1.296	5.035421371	48.39	0	1782.016169	2	891.5102539	-1.648844466	-0.001469134	49.58471369
																			DTDAAVGDNIGYITFVLFPR	2	23238211	1xTMT6plex [N-Term]			1.098	5.134865284	63.13	0	2413.260092	3	805.0904541	-1.360270003	-0.001094228	105.0392673
																			DSIVHQAGMLK	16	23238211	2xTMT6plex [N-Term; K11]	0.944	0.899	0.934	4.876075268	58.34	0	1656.950733	2	828.9804688	1.767300586	0.001464165	48.46241235
																			DNTINLIHTFR	4	23238211	1xTMT6plex [N-Term]	1.121	1.185		3.692628384	56.89	0	1572.869556	3	524.9612427	-0.242539284	-0.000127161	63.82252405
																			IIEETLALK	6	23238211	2xTMT6plex [N-Term; K9]	100	2.695	1.217	2.437057018	30.14	0	1487.944902	2	744.4755249	-0.758748063	-0.000564488	77.30271332
																			DDETMYVESK	5	23238211	2xTMT6plex [N-Term; K10]; 1xOxidation [M5		1.64	1.099	2.432089806	41.97	0	1690.824589	2	837.9187012	0.269802809	0.000225937	45.14550327
																			YFQFQEEGK	3	23238211	2xTMT6plex [N-Term; K9]	1.239			3.171218157	36.19	0	1633.862629	2	817.4351807	0.278877867	0.000227824	61.13735987
																			VFMQEFK	5	23238211	2xTMT6plex [N-Term; K7]	2.377	100		2.39089179	20.78	0	1386.785565	2	693.8963623	-0.084296107	-5.85E-05	69.85266618
																			EPPLELK	2	23238211	2xTMT6plex [N-Term; K7]	0.982			2.380907059	23.44	0	1283.79751	2	642.4033203	1.444343821	0.000927123	53.13549092
4503729	FKBP4	peptidyl-prolyl cis-trans isomerase FKBP4	2288	NP_002005.1	38.99782135	1	16	16	63	459	51.772	5.43	1.26	1.22	1.39	1.29	Significant	0.0016																
																			GEDLTEEEDGGIIR	10	4503729	1xTMT6plex [N-Term]	0.656	2.053	1.628	4.376208305	75.12	0	1761.870403	2	881.4393311	0.557373217	0.00049101	44.53354981
																			GEGYAKPNEGAIVEVALEGYYK	3	4503729	3xTMT6plex [N-Term; K6; K22]			1.293	5.802571297	36.82	0	3044.654763	3	1015.555847	-0.583059897	-0.000591739	87.75440042
																			SNTAGSQSQVETEA	14	4503729	1xTMT6plex [N-Term]	1.734	1.646	1.864	3.719779253	87.01	0	1637.781589	2	819.3946533	0.269578452	0.000220755	27.41803652
																			RGEAHLAVNDFELAR	2	4503729	1xTMT6plex [N-Term]		1.568		6.077880859	32.35	1	1927.034724	3	643.0157471	-1.056524326	-0.000678653	44.49603391
																			GEAHLAVNDFELAR	2	4503729	1xTMT6plex [N-Term]	1.039			4.343502998	32.16	0	1770.933613	3	590.9829712	0.422017072	0.000249121	61.10757483
																			VFVHYTGWLLDGTK	2	4503729	2xTMT6plex [N-Term; K14]			0.677	3.612560511	15.05	0	2094.178818	3	698.7304688	-0.938343938	-0.00065502	90.55888565
																			AWDIAIATMK	2	4503729	2xTMT6plex [N-Term; K10]	1.128			3.386593819	39.85	0	1577.912556	2	789.4599609	0.056438489	4.45E-05	93.71436661
																			ALELDSNNEK	2	4503729	2xTMT6plex [N-Term; K10]		1.213		3.960134983	45.08	0	1590.873922	2	795.9401855	-0.520139427	-0.000413738	50.68460905
																			IVSWLEYESSFSNEEAQK	2	4503729	2xTMT6plex [N-Term; K18]	100			2.722323656	17.23	0	2604.323369	3	868.7774658	-2.121483502	-0.001841676	98.95553027
																			KLYANMFER	4	4503729	2xTMT6plex [N-Term; K1]	0.974	0.78		3.968015909	26.25	1	1629.918704	3	543.9772339	-0.954519903	-0.000518597	54.65780943
																			AKESWEMNSEEK	5	4503729	3xTMT6plex [N-Term; K2; K12]	100	0.952	1.268	4.566130638	15.78	1	2155.130831	3	719.0487061	0.34074686	0.000244785	41.92878084
																			DKFSFDLGK	2	4503729	3xTMT6plex [N-Term; K2; K9]	1.596			2.90148592	22.23	1	1744.024833	3	582.0130005	-0.220533073	-0.000128205	64.21240409
																			FEIGEGENLDLPYGLER	3	4503729	1xTMT6plex [N-Term]	100			1.704530358	2.64	0	2180.10728	2	1090.55542	-1.704662098	-0.001858173	82.06064685
																			LEQSTIVK	6	4503729	2xTMT6plex [N-Term; K8]	1.586	1.027	1.665	3.376317501	41.24	0	1375.856087	2	688.4317017	0.028709625	1.98E-05	49.70082011
																			QALLQYK	2	4503729	2xTMT6plex [N-Term; K7]	1.318			3.105844498	25.37	0	1321.824393	2	661.4155273	-0.46538141	-0.000307576	58.53751548
																			FSFDLGK	2	4503729	2xTMT6plex [N-Term; K7]				2.110158443	14.77	0	1271.739995	2	636.3737183	0.129730478	8.25E-05	65.12002806
5453555	RAN	GTP-binding nuclear protein Ran isoform 1	5901	NP_006316.1	63.42592593	1	11	11	92	216	24.408	7.49	1.14	1.6	1.43	1.39	Significant	0.0092																
																			LIGDPNLEFVAMPALAPPEVVMDPALAAQYEHDLEVAQTTALPDEDDDL	4	5453555	1xTMT6plex [N-Term]		100		1.91749239	5.14	0	5488.690525	5	1098.547852	3.575800532	0.003925292	111.4247346
																			KLIGDPNLEFVAMPALAPPEVVMDPALAAQYEHDLEVAQTTALPDEDDDL	13	5453555	2xTMT6plex [N-Term; K1]	1.692	100	0.521	2.772305965	9.91	1	5845.94842	5	1169.992065	-2.942065227	-0.003439832	105.8958772
																			YVATLGVEVHPLVFHTNR	9	5453555	1xTMT6plex [N-Term]	1.393	1.834	2.03	4.874919415	40.93	0	2281.265452	4	571.0720825	0.45947223	0.000262045	72.42526014
																			LVLVGDGGTGK	24	5453555	2xTMT6plex [N-Term; K11]	1.215	1.536	1.245	4.134624958	54.31	0	1473.9041	2	737.4550171	-0.910792169	-0.00067121	58.76428813
																			SNYNFEKPFLWLAR	7	5453555	2xTMT6plex [N-Term; K7]	100	0.911	1.103	3.782208443	20.85	0	2243.23773	3	748.4188843	1.947948705	0.001456571	94.41151461
																			KYVATLGVEVHPLVFHTNR	2	5453555	2xTMT6plex [N-Term; K1]	0.443			1.922952175	14.35	1	2638.523347	5	528.5106812	0.360910856	0.000190454	89.01294623
																			VCENIPIVLCGNK	9	5453555	2xTMT6plex [N-Term; K13]; 2xCarbamidomethyl [C2; C10	100	1.986	1.141	3.507136822	48.29	0	1974.09166	2	987.5474243	-2.070799077	-0.002043974	66.53035085
																			KNLQYYDISAK	6	5453555	3xTMT6plex [N-Term; K1; K11]	1.202	1.05	1.1	4.75934124	23.23	1	2030.188938	3	677.3967896	-6.463678041	-0.004374163	47.18104449
																			NLQYYDISAK	4	5453555	2xTMT6plex [N-Term; K10]	1.362	1.71		3.318092346	30.48	0	1672.931043	2	836.9695435	0.458694377	0.000383682	42.34826895
																			FNVWDTAGQEK	6	5453555	2xTMT6plex [N-Term; K11]	2.074	2.046		2.42821908	29.81	0	1752.932106	2	876.9691162	-0.655888566	-0.000574864	59.33873138
																			HLTGEFEK	8	5453555	2xTMT6plex [N-Term; K8]	1.639	1.549	1.535	3.596393585	37.15	0	1418.804386	3	473.6062927	-0.043040586	-2.04E-05	39.36200261
153946395	TNC	tenascin precursor	3371	NP_002151.2	10.44979555	1	15	15	58	2201	240.7	4.89	0.82	0.98	1.15	0.98	Significant	0.0093																
																			YAPISGGDHAEVDVPK	5	153946395	2xTMT6plex [N-Term; K16]	1.074	1.068		4.558451653	37.35	0	2113.13299	3	705.0477295	-2.060799513	-0.001451581	55.59904164
																			GYDCSDMSCPNDCHQHGR	2	153946395	1xTMT6plex [N-Term]; 3xCarbamidomethyl [C4; C9; C13	1.07			5.246084213	66.36	0	2424.937548	3	808.9844971	0.573264635	0.000463377	30.64262921
																			TPVLSAEASTAK	4	153946395	2xTMT6plex [N-Term; K12]	1.835	1.615		4.491948128	67.96	0	1632.957258	2	816.9829712	0.862308432	0.000704056	50.68268116
																			DLTATEVQSETALLTWRPPR	2	153946395	1xTMT6plex [N-Term]			0.843	4.351345062	28.29	0	2513.356117	3	838.4550781	-2.162914743	-0.001812058	85.2495264
																			EQCTAGAGCCLQPATGR	6	153946395	1xTMT6plex [N-Term]; 3xCarbamidomethyl [C3; C9; C10			100	2.666308165	29.2	0	2065.941494	2	1033.474365	-0.019618779	-2.03E-05	31.61457785
																			ITAQGQYELR	6	153946395	1xTMT6plex [N-Term]	1.774	1.503	1.537	4.160611153	54.33	0	1407.779344	2	704.3934937	0.260235874	0.000183177	41.89927628
																			EVIVGPDTTSYSLADLSPSTHYTAK	6	153946395	2xTMT6plex [N-Term; K25]			100	2.621251106	21.38	0	3110.629781	3	1037.547485	-0.603895495	-0.000626165	71.91735385
																			DHGETAFAVYDK	2	153946395	2xTMT6plex [N-Term; K12]		1.059		2.42027998	25.67	0	1810.937585	2	905.9716187	-0.896831192	-0.000812053	43.46222261
																			ETFTTGLDAPR	6	153946395	1xTMT6plex [N-Term]	0.558	0.73	0.746	4.069524765	56.67	0	1436.758274	2	718.8831787	0.561195219	0.000403151	45.31500183
																			GLEPGQEYNVLLTAEK	5	153946395	2xTMT6plex [N-Term; K16]				1.990429759	28.5	0	2219.23237	2	1110.118042	-1.605161169	-0.001781113	72.42756344
																			VPGDQTSTIIQELEPGVEYFIR	4	153946395	1xTMT6plex [N-Term]			100	2.376901388	13.6	0	2720.434427	3	907.4815063	-1.639920811	-0.001487099	100.8095399
																			ACGCAAAPDVK	2	153946395	2xTMT6plex [N-Term; K11]; 2xCarbamidomethyl [C2; C4			1.666	3.115476608	37.61	0	1577.818004	2	789.4119873	-0.828036327	-0.000653245	36.92410708
																			ASTEQAPELENLTVTEVGWDGLR	2	153946395	1xTMT6plex [N-Term]				1.584557652	13.53	0	2744.394019	3	915.4694824	-0.045457895	-4.16E-05	80.34496212
																			VFAILENK	4	153946395	2xTMT6plex [N-Term; K8]	0.765	100		2.602037668	29.68	0	1391.866258	2	696.4368286	0.08808789	6.13E-05	82.9778995
																			FTTDLDSPR	2	153946395	1xTMT6plex [N-Term]	0.739			2.700298548	11.99	0	1280.668397	2	640.8384399	0.941947826	0.000603161	47.81807295
45007007	OAS3	2'-5'-oligoadenylate synthase 3	4940	NP_006178.2	20.33118675	1	17	17	75	1087	121.093	8.4	0.36	0.28	0.56	0.4	Significant	0.041																
																			FIQDHLKPSPQFQEQVK	3	45007007	3xTMT6plex [N-Term; K7; K17]	0.4	0.271		5.114658356	21.95	0	2756.570246	4	689.8978271	-0.278417084	-0.000191869	63.61638192
																			LLTSGCQEGEHK	8	45007007	2xTMT6plex [N-Term; K12]; 1xCarbamidomethyl [C6	0.296	0.276	0.358	4.776339531	57.81	0	1816.962754	3	606.3252563	-0.84657048	-0.000512729	41.9815736
																			AQLESWWQDQVPSLSLQFPEQNVPEALQFQLVSTALK	2	45007007	2xTMT6plex [N-Term; K37]				1.650963902	13.55	0	4713.496863	4	1179.129639	-0.029301494	-3.45E-05	109.2099474
																			AQLEACQQER	6	45007007	1xTMT6plex [N-Term]; 1xCarbamidomethyl [C6	0.303	0.3	0.574	3.58369112	42.96	0	1461.731742	2	731.3685913	-1.256374723	-0.000918241	29.89958964
																			ASLESWWQNPVPGLR	5	45007007	1xTMT6plex [N-Term]				2.505597591	24.49	0	1969.049311	2	985.0280151	-0.28324662	-0.000278863	90.69980529
																			GMGDPVQSWK	4	45007007	2xTMT6plex [N-Term; K10]	0.171			2.732551098	24.56	0	1562.84012	2	781.9240112	0.400751766	0.000313155	57.83944049
																			QDCFNMAQGFR	2	45007007	1xTMT6plex [N-Term]; 1xCarbamidomethyl [C3	0.928			2.612925768	4.14	0	1602.735448	2	801.8712769	-0.106429956	-8.53E-05	62.63099563
																			DSQFNMAEGFR	2	45007007	1xTMT6plex [N-Term]	0.655			3.747541428	30.95	0	1530.720843	2	765.8646851	0.816842074	0.000625179	63.06992403
																			GGCDSELVIFLDCFK	2	45007007	2xTMT6plex [N-Term; K15]; 2xCarbamidomethyl [C3; C13				2.112952232	21.72	0	2218.128833	2	1109.567139	-0.826136076	-0.000916238	107.0750176
																			ALGALAAALR	8	45007007	1xTMT6plex [N-Term]	0.129	0.01		2.974721909	44.9	0	1155.741108	2	578.3735352	-1.137622855	-0.000657399	74.58562243
																			AEIISEIR	8	45007007	1xTMT6plex [N-Term]		0.318	0.479	3.085564852	35.71	0	1159.688404	2	580.3480835	0.419252435	0.000243101	56.18271204
																			DAFSLAEGLR	5	45007007	1xTMT6plex [N-Term]				2.54204464	48.34	0	1307.715681	2	654.3614502	-0.044137557	-2.89E-05	65.35211467
																			AEILDEMR	6	45007007	1xTMT6plex [N-Term]	0.01	0.159	0.63	2.696259737	26.83	0	1205.639739	2	603.324585	1.78651367	0.001076946	56.53038306
																			SDADLVVFLSCFSQFTEQGNK	2	45007007	2xTMT6plex [N-Term; K21]; 1xCarbamidomethyl [C11				1.710897207	6.02	0	2850.438416	3	950.8186035	0.996796443	0.000947102	112.50487
																			AVDTICSFLK	2	45007007	2xTMT6plex [N-Term; K10]; 1xCarbamidomethyl [C6	0.01			1.820323944	13.77	0	1611.918036	2	806.4622192	-0.542157491	-0.000436957	99.38959162
																			DFIISRPTK	2	45007007	2xTMT6plex [N-Term; K9]			0.655	2.266727686	5.02	0	1534.935734	3	512.3167725	0.019325117	9.89E-06	51.8324277
																			AIDIILR	8	45007007	1xTMT6plex [N-Term]	0.38	0.206	0.298	2.250755787	28.26	0	1042.682197	2	521.8441162	-1.190082778	-0.000620439	59.66060816
4885379	HIST1H1E	histone H1.4	3008	NP_005312.1	42.00913242	1	4	14	127	219	21.852	11.03	1.87	1.74	1.82	1.81	Significant	0.00044																
																			ASGPPVSELITK	11	4885379	2xTMT6plex [N-Term; K12]	2.468	2.746	2.739	4.860414505	78.21	0	1656.993643	2	829.0006104	0.181601858	0.000150457	66.19361867
																			KASGPPVSELITK	16	4885379	3xTMT6plex [N-Term; K1; K13]	1.721	1.428	1.877	5.893885612	42.56	1	2014.251538	3	672.0892334	0.798716189	0.000536272	58.09582745
																			KALAAAGYDVEK	12	4885379	3xTMT6plex [N-Term; K1; K12]	1.152	1.152	1.007	6.476112366	60.9	1	1923.151824	3	641.7223511	0.351419294	0.000225278	52.13334856
																			ALAAAGYDVEK	5	4885379	2xTMT6plex [N-Term; K11]	2.065	2.053		4.63003397	59.06	0	1565.893929	2	783.4494629	-1.456049294	-0.001140009	51.35155329
																			KPAAAAGAK	6	4885379	3xTMT6plex [N-Term; K1; K9]	2.273	2.067	1.976	4.782018185	28.63	0	1471.956359	3	491.3237305	0.189330511	9.29E-05	27.31521347
																			KATGAATPK	10	4885379	3xTMT6plex [N-Term; K1; K9]	1.709	1.552	1.624	4.393535137	31	1	1531.977489	3	511.3306885	0.015267644	7.80E-06	25.59137409
																			SGVSLAALK	10	4885379	2xTMT6plex [N-Term; K9]	2.54	100	100	3.625626802	59.93	0	1303.834958	2	652.4209595	-0.242020423	-0.000157777	61.01925825
																			GTGASGSFK	20	4885379	2xTMT6plex [N-Term; K9]	1.768	1.906	1.992	3.84473753	55.74	0	1269.720322	2	635.364624	1.299000139	0.000824683	32.06205911
																			AASGEAKPK	15	4885379	3xTMT6plex [N-Term; K7; K9]	1.259	1.961	2.248	2.505070686	12.35	0	1545.956753	2	773.4815674	-0.579048782	-0.000447592	34.75106002
																			ATGAATPK	6	4885379	2xTMT6plex [N-Term; K8]	100	1.401	1.924	2.701102734	40.43	0	1174.719594	2	587.8638306	0.673082169	0.000395341	26.03022919
																			KSAGAAK	3	4885379	3xTMT6plex [N-Term; K1; K7]		100	1.261	2.535447359	10.59	1	1319.861397	3	440.6254272	0.251525109	0.000110659	22.07052733
																			KPAGAAK	6	4885379	3xTMT6plex [N-Term; K1; K7]	24.863	5.708	6.759	2.264737606	16.75	0	1329.882132	3	443.9654846	-0.173972243	-7.71E-05	24.93449607
																			KAASGEAKPK	2	4885379	4xTMT6plex [N-Term; K1; K8; K10]	1.906	2.287		1.973932862		1	1903.214648	4	476.559845	1.524637704	0.000725428	26.06840674
																			GTLVQTK	5	4885379	2xTMT6plex [N-Term; K7]	2.149	1.798	2.043	3.357786417	40.19	0	1204.766544	2	602.8865967	-0.520657407	-0.000313635	34.71154277
4885281	GLUD1	glutamate dehydrogenase 1, mitochondrial isoform a precursor	2746	NP_005262.1	27.59856631	1	14	14	78	558	61.359	7.8	0.83	0.74	0.76	0.78	Significant	0.0012																
																			AKPYEGSILEADCDILIPAASEK	5	4885281	3xTMT6plex [N-Term; K2; K23]; 1xCarbamidomethyl [C13			0.583	3.198639631	17.73	0	3177.732028	3	1059.915527	0.000330818	3.50E-07	72.35830722
																			LQHGSILGFPK	2	4885281	2xTMT6plex [N-Term; K11]		0.712		4.224870682	50.81	0	1655.004483	3	552.3397217	0.07799318	4.30E-05	68.8076516
																			DIVHSGLAYTMER	12	4885281	1xTMT6plex [N-Term]	0.832	0.814	0.798	3.810566425	34.85	0	1720.888971	3	574.3009644	-0.366777639	-0.000210395	49.27020379
																			TAAYVNAIEK	4	4885281	2xTMT6plex [N-Term; K10]	0.841	0.784		4.948320389	58.05	0	1537.899015	2	769.4535522	0.528833285	0.000406646	59.74060867
																			DSNYHLLMSVQESLER	2	4885281	1xTMT6plex [N-Term]	0.912			2.634652615	15.75	0	2150.074934	3	717.3632202	0.080827198	5.79E-05	74.43312035

Bhat et al,2017. Cigarette smoke and chewing tobacco alter expression of different sets of miRNAs in oral keratinocytes

Supplementary Table S2(A). List of all proteins and peptides identified in OKF6/TERT1 cells chronically treated with chewing tobacco using Sequest and Mascot search algorithms

Accession	Gene_Symbol	Description	Gene_ID	Refseq_Accession	Coverage	Proteins	#Unique_Peptides	#Peptides	#PSMS	#AAs	MW [kDa]	calc. pI	OKF6-STE-6M/OKF6P_R1	OKF6-STE-6M/OKF6P_R2	OKF6-STE-6M/OKF6P_R3	Median fold change	Status	P-value (6M)
20070205	LAD1	ladinin-1	3898	NP_005549.2	34.04255319	1	16	16	69	517	57.097	9.67	0.97	1.05	0.99	1	Significant	0.00057
47519616	TPM2	tropomyosin beta chain isoform Tpm2.1sm/cy	7169	NP_998839.1	46.83098592	1	2	14	114	284	32.97	4.67	1.01	2.53		1.77	Non-Significant	#N/A
5729953	NUDC	nuclear migration protein nudC	10726	NP_006591.1	45.31722054	1	14	14	61	331	38.219	5.38	0.9	1.58	1.4	1.29	Significant	0.024
5453990	PSME1	proteasome activator complex subunit 1 isoform	5720	NP_006254.1	60.24096386	1	15	15	99	249	28.705	6.02	0.72	0.74	0.71	0.72	Significant	0.00015
48762920	PFKL	ATP-dependent 6-phosphofructokinase, liver type isoform b	5211	NP_002617.3	19.74358974	1	9	13	58	780	84.964	7.5	0.94	0.72	0.95	0.87	Significant	0.0074
42734430	PTRF	polymerase I and transcript release factor	284119	NP_036364.2	31.02564103	1	10	10	117	390	43.45	5.6	1.19	1.28	1.4	1.29	Significant	0.0022
14591909	RPL5	60S ribosomal protein L5	6125	NP_000960.2	37.37373737	1	10	10	95	297	34.341	9.72	1.3	1.26	1.07	1.21	Significant	0.0034
544186032	PDIA6	protein disulfide-isomerase A6 isoform a	10130	NP_001269633.1	41.66666667	1	14	14	98	492	53.867	5.33	0.96	0.96	0.93	0.95	Significant	0.00011
4503609	ETFB	electron transfer flavoprotein subunit beta isoform	2109	NP_001976.1	58.82352941	1	15	15	76	255	27.826	8.1	0.72	0.74	0.76	0.74	Significant	0.00024
24797086	IPO5	importin-5	3843	NP_002262.3	17.66816143	1	15	15	54	1115	125.464	4.92	1.02	1.32	1.54	1.3	Significant	0.013
7549809	PLS3	plastin-3 isoform 1	5358	NP_005023.2	32.53968254	1	18	18	79	630	70.766	5.6	1.01	1.29	1.22	1.17	Significant	0.0051
5031977	NAMPT	nicotinamide phosphoribosyltransferase precursor	10135	NP_005737.1	43.3808554	1	15	15	62	491	55.487	7.15	0.68	0.98	0.74	0.8	Significant	0.013
11863154	ARCN1	coatomer subunit delta isoform 1	372	NP_001646.2	30.52837573	1	15	15	70	511	57.174	6.21	1.13	1.12	1.12	1.13	Significant	0.0000088
50592988	UQCRC2	cytochrome b-c1 complex subunit 2, mitochondrial precursor	7385	NP_003357.2	19.73509934	1	5	5	19	453	48.413	8.63	1.28	0.72	1.21	1.07	Significant	0.026

Sequence	#PSMs	Protein Accessions	Modifications	OKF6-STE-6M/OKF6P_R1	OKF6-STE-6M/OKF6P_R2	OKF6-STE-6M/OKF6P_R3	Xcorr	Ionscore	# Missed Cleavages	Theo. MH+ [Da]	Charge	m_z	DeltaM [ppm]	Deltam/z [Da]	RT [min]
NYTDNELEK	2	4885281	2xTMT6plex [N-Term; K9]	0.793			3.704126835	54.38	0	1583.831723	2	792.4187622	-0.931289553	-0.000737503	46.49055083
DDGSWEVIEGYR	6	4885281	1xTMT6plex [N-Term]	0.583			3.181992531	26.79	0	1654.791031	2	827.8981323	-1.234628134	-0.001021526	62.86570242
ALASLMTYK	10	4885281	2xTMT6plex [N-Term; K9]	0.75	0.965	0.9	4.062063694	43.1	0	1455.864543	2	728.4359131	0.004211844	3.07E-06	70.6349318
RDDGSWEVIEGYR	2	4885281	1xTMT6plex [N-Term]	1.221			3.343880653	17.27	1	1810.892142	3	604.3015747	-1.088507074	-0.000657056	56.39873963
DIVHSGLAYTMER	4	4885281	1xTMT6plex [N-Term]; 1xOxidation [M11]	0.776		0.744	3.233889818	28.2	0	1736.883886	3	574.3009644	-0.366777639	-0.000210395	49.27020379
GASIVEDK	6	4885281	2xTMT6plex [N-Term; K8]		0.714	0.761	3.345249891	56.21	0	1276.751288	2	638.8789063	-0.588940076	-0.000375965	46.79882012
MVEGFFDR	9	4885281	1xTMT6plex [N-Term]	0.859	0.803		3.175993919	39.6	0	1229.61861	2	615.3127441	-0.324026285	-0.000199214	75.82298847
GFIGPGIDVPAPDMSTGER	6	4885281	1xTMT6plex [N-Term]	0.982		100	2.042868853	18.03	0	2145.08477	2	1073.044922	-1.026957084	-0.001101455	66.07205107
YNLGLDLR	4	4885281	1xTMT6plex [N-Term]	0.879	100		3.130409956	43.57	0	1192.688739	2	596.8486328	1.048442028	0.000625233	62.7767177
FTMELAK	4	4885281	2xTMT6plex [N-Term; K7]	0.685	100		3.085471392	37.98	0	1297.759016	2	649.3825073	-0.984598486	-0.000638886	64.56765305
GLPCTELFVAPVGVASK	4	20070205	2xTMT6plex [N-Term; K17]; 1xCarbamidomethyl [C4	0.956	0.914		3.721407413	46.25	0	2203.256082	2	1102.129761	-1.7417202	-0.001918728	88.56393705
IPSKEEEADMSSPTQR	8	20070205	2xTMT6plex [N-Term; K4]	0.899	1.131		4.522712708	20.14	1	2263.164032	3	760.392334	1.536414619	0.001167244	33.5267847
TQESGDQDPQEAQK	2	20070205	2xTMT6plex [N-Term; K14]	1.391			3.404587507	38.95	0	2019.003098	2	1010.005615	0.423921465	0.000427949	30.79792875
NLSSTTDDEAPR	2	20070205	1xTMT6plex [N-Term]	1.001			4.266755581	56.11	0	1534.754646	2	767.8807983	-0.212086221	-0.00016275	37.12122019
TLEDEEEQER	4	20070205	1xTMT6plex [N-Term]	1.217	1.334		4.3690238	41.12	0	1506.712112	2	753.8596802	-0.018788219	-1.42E-05	29.72788134
[truncated: 11,438,888 more chars]
